# A revision of the Morelloid Clade of *Solanum* L. (Solanaceae) in North and Central America and the Caribbean

**DOI:** 10.3897/phytokeys.123.31738

**Published:** 2019-05-30

**Authors:** Sandra Knapp, Gloria E. Barboza, Lynn Bohs, Tiina Särkinen

**Affiliations:** 1 Department of Life Sciences, Natural History Museum, Cromwell Road, London SW7 5BD, UK Natural History Museum London United Kingdom; 2 Instituto Multidisciplinario de Biología Vegetal (CONICET-Universidad Nacional de Córdoba), Casilla de Correo 495, 5000 Córdoba, Argentina Instituto Multidisciplinario de Biología Vegetal Córdoba Argentina; 3 Department of Biology, 1400 South, University of Utah, Salt Lake City, Utah, USA University of Utah Salt Lake City United States of America; 4 Royal Botanic Garden Edinburgh, 20A Inverleith Row, Edinburgh EH3 5LR, UK Royal Botanic Garden Edinburgh Edinburgh United Kingdom

**Keywords:** black nightshades, Caribbean, Antilles, Mexico, Central America, North America, *
Solanum
*, vegetables, weeds

## Abstract

The Morelloid Clade, also known as the black nightshades or “Maurella” (Morella), is one of the 10 major clades within the mega-diverse genus *Solanum* L. The clade is most species rich in the central to southern Andes, but species occur around the tropics and subtropics, some extending well into the temperate zone. Plants of the group are herbaceous or short-lived perennials, with small white or purplish white flowers, and small juicy berries. Due to the complex morphological variation and weedy nature of these plants, coupled with the large number of published synonyms (especially for European taxa), our understanding of species limits and diversity in the Morelloid Clade has lagged behind that of other major groups in *Solanum*. Here we provide the second in a three-part series of revisions of the morelloid solanums treating the species occurring in North and Central America and the Caribbean (for the Old World see “PhytoKeys 106”, the third part will treat species of South America). Synonymy, morphological descriptions, distribution maps, and common names and uses are provided for all 18 species occurring in this region. We treat 10 of these species as native, and eight as putatively naturalised, introduced and/or invasive in the region. We provide complete descriptions with nomenclatural details, including lecto- and neotypifications, for all species. Keys to all species occurring in the whole region and for each area within it (i.e., North America, Central America and Mexico, and the islands of the Caribbean), illustrations to aid identification both in herbaria and in the field, and distribution maps are provided. Preliminary conservation assessments are provided for all species. Details of all specimens examined are provided in three Supplementary materials sections.

## Introduction

*Solanum* L., with approximately 1,400 species, is one of the largest genera of flowering plants (Frodin 2004). The genus poses a taxonomic challenge not only due to its large size, but also due to the 6,931 published names (see solanaceaesource.org), many of which are associated with the cultivated and widespread species of the genus, including the potato (*S.tuberosum* L.), tomato (*S.lycopersicum* L.), and eggplant (*S.melongena* L.). Recent taxonomic and molecular systematic efforts (http://www.solanaceaesource.org) have helped to identify major clades within *Solanum* (e.g., Weese and Bohs 2007), clarify relationships and the monophyly of previously recognised morphological sections (e.g., Stern et al. 2011), and to provide taxonomic revisions for major clades with keys for species identification (e.g., Knapp 2013).

The Morelloid Clade of *Solanum*, also known as the Black nightshades or “Maurella” (Morella), is amongst the 10 robustly supported major clades within *Solanum* (Bohs 2005; Weese and Bohs 2007). This group, which includes the type species of the genus, *S.nigrum* L., has traditionally been considered difficult, due in part to the weedy nature of its species and its worldwide distribution. The clade comprises 74 currently recognised non-spiny herbaceous and suffrutescent species with simple or branched hairs with or without glandular tips and inflorescences usually arising from the internodes (Särkinen et al. 2015b). Ploidy level varies from diploid to hexaploid within the group (e.g., Edmonds and Chweya 1997; Moyetta et al. 2013; Särkinen et al. 2018; Chiarini et al. 2018), in part contributing to the difficulties in its taxonomy. While taxonomic revisions of the smaller New World sections within the morelloids have recently been published (Del Vitto and Petenatti 1999; Barboza 2003; Barboza and Hunziker 2005), the group in its entirety has not been revised since the 19^th^ century (Dunal 1852).

General overviews of black nightshade taxonomy have been published (Edmonds 1977, 1978, 1979a), including geographically focused taxonomic treatments for South America (Edmonds 1972), North America (Schilling 1981) and Africa (Edmonds and Chweya 1997; Olet 2004; Manoko 2007; Edmonds 2012) and detailed cytological and morphological studies (Venkateswarlu and Roo 1972; Edmonds 1982, 1983, 1984). Genomic work is being developed with the use of the European *S.nigrum* as a model system for studying plant/insect interactions (e.g., Schmidt et al. 2004). These studies have greatly enhanced our understanding of the complex morphological and ploidy level variation present in the group, but much taxonomic work remains. A taxonomic treatment of morelloids in the Old World (19 species) has recently been published by Särkinen et al. (2018), including a revision of the nomenclature and typification of the ca. 371 names associated with those species. South America, especially the southern Andes, where more than half of the known species are found (Barboza et al. 2013), is the centre of diversity for morelloids in the Americas. North and Central America and the Caribbean have a distinctly different complement of species to those from continental South America, although some species with wide distributions span the Americas (e.g., *S.americanum*, *S.nigrescens*). In addition, several Old World species (e.g., *S.nigrum*, *S.retroflexum*) have been introduced or are sporadically cultivated in North America.

Here we provide a taxonomic revision of all 18 native and naturalised (or semi-naturalised) species of the Morelloid Clade occurring in North and Central America and the Caribbean based on a detailed morphological study, including both native and introduced taxa. Some of these taxa have been treated in Särkinen et al. (2018), but we include full descriptions here to aid identification and distributional understanding in the region. One morelloid species from Argentina, *S.pilcomayense* Morong, has been collected twice in port areas of Texas and New Jersey in the late 19^th^ and early 20^th^ centuries probably with ballast or wool waste. It has not been collected again since, so we have not included this taxon in the treatment; a description can be found in Barboza et al. (2013). The work presented here is part of our molecular systematic and taxonomic work focusing on producing a monographic treatment of the Morelloid Clade (e.g., Barboza et al. 2013; Särkinen et al. 2013, 2015a, b, 2018) across its entire range in a series of geographically focussed works, the first of which treated taxa occurring in the Old World (Särkinen et al. 2018) and the last of which will treat the South American species (in preparation).

## Taxonomy and relationships

Knowledge of the European species of black nightshades stretches back to the Greeks and Romans (see summary in Särkinen et al. 2018), and perceptions of the toxicity of these plants among European immigrants to the New World is likely in part to have derived from confusion over the identity of *S.nigrum* and *Atropabella-donna* L., both of which have been referred to as “deadly nightshade”.

*Solanumnigrum* was the only species of this group treated by Linnaeus (1753). His circumscription was extremely broad and comprised six infraspecific taxa, many of which were based on the plates in Dillenius’s *Hortus Elthamensis* (Dillenius 1732). He recognised the European *S.nigrum* (as var. vulgare), *S.villosum* (as var. villosum), and *S.americanum* (as var. patulum), included the African cultivated species *S.scabrum* (as var. guineense), and recognised the native North American *S.emulans* (as var. virginicum), but he had not seen material of other species treated here (see individual species treatments for details). He clearly recognised all these taxa as very similar and as variants of a worldwide complex; his diagnosis reads “Habitat in Orbis totius, cultis” [Habitat in all the world, cultivated]. He also noted many of these looked like mixtures (“Tot varietates β, γ, δ, ε, ζ videntur esse hybridae proles” [All of the varieties β, γ, δ, ε, ζ seem to be hybrid offspring]). In *Species plantarum*Linnaeus (1753) did not cite many of the works based on non-European plants (e.g., Piso 1648; Rheede von Draakestein 1689) he had previously cited in *Hortuscliffortianus* (Linnaeus 1737), and in the Clifford herbarium (BM) specimens of *S.nigrum*, *S.scabrum* and *S.villosum* are preserved, all grown from cultivation.

Miller (1768), in the sixth edition of his *Gardener’s dictionary* and the first to use Linnaean binomials (see Stearn 1974), described seven members of the Morelloid Clade, five of these as new names (*S.villosum* Mill., *S.luteum* Mill., *S.rubrum* Mill., *S.americanum* Mill., and *S.scabrum* Mill.). He did not recognise infraspecific taxa, but also did not indicate he was raising Linnaeus’s varieties to species level. He did not include any new American taxa.

Lamarck (1794) recognised seven taxa, including some not known to either Miller or Linnaeus, such as *S.radicans* L.f. and *S.corymbosum* Jacq., both members of the clade belonging to the Radicans group (sensu Särkinen et al. 2015b). He additionally described *S.chenopodioides* Lam., from material said to be from “île de France” (Mauritius, see species description) and *S.triangulare* Lam. (= *S.americanum*) based on an illustration from Rumphius (1750). Some of these early authors re-used epithets (e.g., *villosum* used by both Miller and Lamarck), but it is not clear whether they were referring to earlier names or not; the principle of priority had not yet become established for botanical naming (see Knapp et al. 2004).

The name for the Morelloid Clade is derived from Dunal’s (1813: 119) un-ranked group “Maurella” that included herbaceous or subherbaceous species with entire leaves. He included 15 species in this group, all of which are still considered members of the Morelloid clade. In his *Solanorumsynopsis* (Dunal 1816) he maintained Maurella, adding to it taxa described by himself and others, most of which are still considered related (except for *S.quadrangulare* Thunb. = *S.africanum* Mill., a member of the African Non-Spiny Clade, see Knapp and Vorontsova 2016). Dumortier (1827) used this group, with a changed spelling to “Morella” for the Belgian species. His concept of Morella was narrow and included only those species later recognised as members of SolanumsectionSolanum: it did not include species now recognised as part of the more broadly defined group (Bohs 2005; Weese and Bohs 2007; Särkinen et al. 2013, 2015b). In the *Prodromus*Dunal (1852) paid little attention to earlier names and erected an entirely new framework for *Solanum* mostly composed of *gradi ambigui* (names of ambiguous rank). Morella, however, was one of the names he continued to use. Within it Dunal (1852) recognised two groups based on inflorescence position, “Morellae spuriae” (6 spp.) and “Morellae verae” (54 spp.). Circumscription of Morella remained obscure and loose during most 19^th^ and 20^th^ centuries, with many herbaceous non-spiny taxa treated as members of the group, resulting in the large number of names associated with the Morelloid Clade. Many of these names do not belong to the clade as now recognised based on phylogenetic data (Bohs 2005; Weese and Bohs 2007).

Following the rules on the use of autonyms, Seithe (1962) was the first to name as sectionSolanum the group containing the type species of the genus (*S.nigrum*). She also recognised other groups of the Morelloid clade at the sectional level, as sections *Campanulisolanum* Bitter, *Chamaesarachidium* Bitter and *Episarcophyllum* Bitter (all groups confined to South America, see Del Vitto and Petenatti 1999; Barboza 2003; Barboza and Hunziker 2005), now considered part of the larger Morelloid Clade (Särkinen et al. 2015b). Her sectional names were followed by Danert (1967, 1970) with little change. D’Arcy (1972) lectotypified the infrageneric groupings in *Solanum* and provided an overview of the history of these infrageneric names.

**Figure 1.**
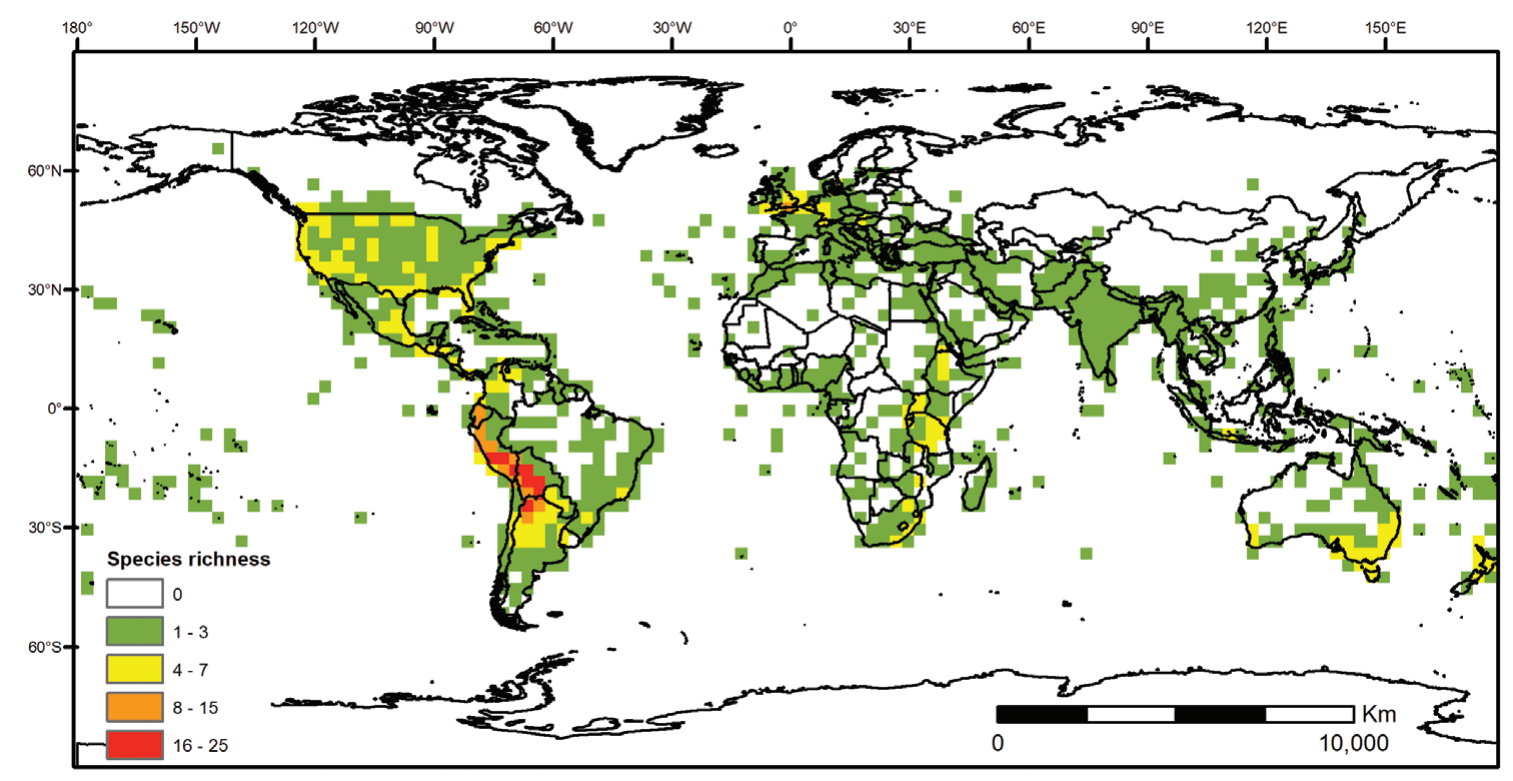
Global distribution of the Morelloid Clade of *Solanum*, showing number of species per 3 degree grid cells (ca. 300 km^2^) based on native and non-native records.

Within the morelloids, four well-supported clades have been recognised based on a detailed molecular phylogenetic study (Särkinen et al. 2015b). These clades loosely correspond to the previously recognised morphological sections: 1) the Radicans clade which comprises four species of SolanumsectionParasolanum A.Child (but not including the type species, *S.triflorum* Nutt.; Bohs 2005), 2) the Episarcophyllum clade that includes most species of SolanumsectionEpisarcophyllum Bitter; 3) the Chamaesarachidium clade that includes two species of SolanumsectionChamaesarachidium Bitter; and finally the largest group, 4) the Black nightshade clade, that includes all species of the traditional SolanumsectionSolanum plus *S.triflorum* (the first branching species in the black nightshade clade in Särkinen et al. 2015b). The first three clades are restricted to the New World, while most species of the Black nightshade clade occur in the New World but with a secondary centre of diversity in the Old World treated in Särkinen et al. (2018; see Figure 1).

The Black nightshade clade within the morelloids, also known as the *S.nigrum* complex and traditionally recognised as SolanumsectionSolanum, contains many widespread and morphologically variable species; this group has always been considered difficult. Although Edmonds (1972) suggested estimates of species richness had been exaggerated in the group and provided relatively low estimates of species numbers, our ongoing work in the group shows the Black nightshade clade s.s. includes ca. 62 species that are mostly restricted to South America (Särkinen et al. 2015b). Eighteen morelloid species are found in North and Central America and the Caribbean, of which seventeen are black nightshades (incl. *S.triflorum*) and one (*S.corymbosum*) is a member of the Radicans group. Eleven of the black nightshades are native (three are endemic to North America north of Mexico, one endemic to Mexico, the rest widespread) and eight are introduced from the Old World or South America (Table 3; Särkinen et al. 2015b). Most of the widespread species in Central America and the Caribbean are shared with northern South America (Colombia, Venezuela and the Guianas). *Solanumcorymbosum* occurs in Mexico widely disjunct from the main species range in Peru and is treated here (Table 3); it is possibly an introduction brought by European explorers from its native range (see Mitchell 2014 and species discussion).

Numerical taxonomic studies have been undertaken in order to resolve species relationships, parental origin of polyploids, and species delimitation in the morelloids (Soria and Heiser 1961; Heiser et al. 1965; Edmonds 1978), but the power of these methods has remained limited due to the complex and often overlapping morphological variation between the closely related species. Species of morelloids show large amounts of morphological variation, especially in growth form, pubescence and leaf morphology. Ongoing phylogenetic work to resolve both relationships and species identity (e.g., Särkinen et al. 2015b) has more promise for untangling these problems.

Floristic treatments of morelloids in North and Central America and the Caribbean in the 19^th^and early 20^th^century (e.g., Gray 1878, Schulz 1909) often treated the native species as *S.nigrum* or as infraspecific taxa of *S.nigrum*. Later floristic works (Table 1) treated these taxa in variable ways, but in general citations of *S.nigrum* as a general term for all morelloids became less common and authors recognised similar numbers of taxa, albeit with variable species epithets (Table 1, see Costa Rica, Guatemala or the state of Illinois as examples; for current distribution of the species treated here see Tables 4 and 5).

**Table 1.** Species epithets (the many misspellings e.g., *S.sarrachoides* as “*S.sarachoides*” and *S.ptychanthum* as “*S.ptycanthum*” have been corrected here) used in representative 20^th^ and early 21^st^ century regional floras in North and Central America and the Caribbean. For distribution of species we recognise in this treatment occurring in each country and/or state see Tables 4 and 5, the searchable files in the Supplementary Material or the dataset on the NHM Data Portal.

Floristic area	Reference	Epithets used
**North America (Canada)**		
Alberta	Moss (1983)	nigrum, sarrachoides, triflorum
British Colombia	Klinkenberg (2017)	americanum, nigrum, physalifolium, triflorum
Eastern Canada (St. Lawrence area)	Marie Antonin (1995)	nigrum
**North America (United States)**		
Regional floras		
Carolinas	Radford et al. (1964)	americanum, gracile, nigrum, sarrachoides
Central and Northeastern United States and adjacent Canada	Fernald (1970)	americanum, nigrum, sarrachoides, triflorum, villosum (mention of interius as outside of range)
Intermountain West	Cronquist et al. (1984)	nigrum (incl. americanum and interius in synonymy), sarrachoides, triflorum
Northeastern United States and adjacent Canada	Gleason and Cronquist (1963)	nigrum, sarrachoides, triflorum
Pacific Northwest	Hitchcock and Cronquist (1976)	nigrum (“diploid and hexaploid”), sarrachoides, triflorum
Sonoran Desert	Shreve and Wiggins (1964)	douglasii, furcatum, sarrachoides (as sarachoides), triflorum
Southeastern United States	Small (1913)	nigrum, gracile, triflorum
**State floras**		
Arizona	Kearney and Peebles (1951)	americanum, douglasii, nodiflorum, sarrachoides, triflorum
Arkansas	Smith (1994)	ptychanthum, sarrachoides
California	Munz (1968)	douglasii, furcatum, nodiflorum, sarrachoides, triflorum
	Nee (2012)	americanum, douglasii, furcatum, nigrum, physalifolium var. nitidibaccatum, triflorum
Colorado	Harrington (1954)	interius, sarrachoides, triflorum
Illinois	Jones and Fuller (1955)	nigrum, triflorum
	Mohlenbrock (2014)	physalifolium, ptychanthum, triflorum
Kentucky	Jones (2005)	physalifolium, ptychanthum (incl. americanum and nigrum in synonymy)
Maine	Haines and Vining (1994)	americanum, nigrum, villosum
Maryland	Brown and Brown (1984)	americanum, nigrum, sarrachoides
Massachusetts	Ahmadjian (1979)	nigrum
Michigan	Voss (1996)	physalifolium, ptychanthum, triflorum
Mississippi	Timme (1989)	americanum, pseudogracile
Missouri	Steyermark (1977)	americanum, sarrachoides, triflorum, villosum
	Yatskevich (2013)	nigrum, ptychanthum, sarrachoides
Nebraska	Kaul et al. (2006)	interius, nigrum, physalifolium var. nitidibaccatum, ptychanthum
New Mexico	Martin and Hutchins (1980)	americanum, douglasii, nigrum (incl. interius), sarrachoides, triflorum
Ohio	Cooperrider (1995)	nigrum (americanum and ptychanthum in synonymy), physalifolium, triflorum
Oregon	Peck (1941)	douglasii, nigrum, triflorum
Pennsylvania	Rhoads and Block (2007)	americanum, nigrum
Texas	Correll and Johnston (1970)	americanum, douglasii, nodiflorum, villosum
Utah	Albee et al. (1988)	nigrum, sarrachoides, triflorum
Utah	Welsh et al. (1993)	nigrum, sarrachoides, triflorum
West Virginia	Strausbaugh and Core (1979)	americanum
**Central America (incl. Mexico)**		
Belize	Balick et al. (2000)	americanum
Costa Rica	Standley and Morton (1938)	nigrum
	Bohs (2015)	americanum, macrotonum, nigrescens
El Salvador	Standley and Calderón (1941)	nigrum
Guatemala	Gentry and D’Arcy (1974)	americanum, nigrescens (suggestion that macrotonum might occur)
	Knapp et al. (2006)	americanum, nigrescens
Honduras	Nelson (1986)	americanum
Mesoamerica (southern Mexico and Central America)	Knapp et al. (2005)	americanum, macrotonum, nigrescens
Mexico (northern)	Felger (2000)	americanum
Mexico (Baja California)	Wiggins (1980)	douglasii, furcatum, nodiflorum, sarrachoides
Mexico (Chiapas)	Breedlove (1986)	americanum, nigrescens
Mexico (Coahuila)	Villarreal Quintanilla (2001)	douglasii, nigrescens (incl. americanum), nigrum
Mexico (Durango)	González Elizondo et al. (1991)	americanum, aff. douglasii, nigrescens (incl. americanum)
Mexico (Valle de México)	Sánchez Sánchez (1968)	nigrum
Mexico (Oaxaca/Puebla)	Davila Aranda et al. (1993)	americanum, nigrum
Mexico (Quintana Roo)	Sousa and Cabrera (1983)	americanum, nigrescens
Mexico (Veracruz)	Nee (1993)	americanum, douglasii, nigrescens
Mexico (Yucatán)	Standley (1930)	nigrum
Nicaragua	D’Arcy (2001)	americanum, nigrescens
Panama	D’Arcy (1974)	americanum, macrotonum, nigrescens
	Correa et al. (2004)	americanum, macrotonum, nigrescens, physalifolium var. nitidibaccatum
**Caribbean**		
Jamaica	Adams (1972)	americanum, antillarum (specimen cited is macrotonum)
West Indies (Bahamas, Greater and Lesser Antilles)	Knapp (2012)	americanum, nigrescens
Bahamas	Correll and Correll (1982)	americanum
French Antilles (Guadeloupe, Martinique, St. Martin, St. Barthelemy)	Sastré and Breuil (2007)	americanum

## Morphology

**Figure 2.**
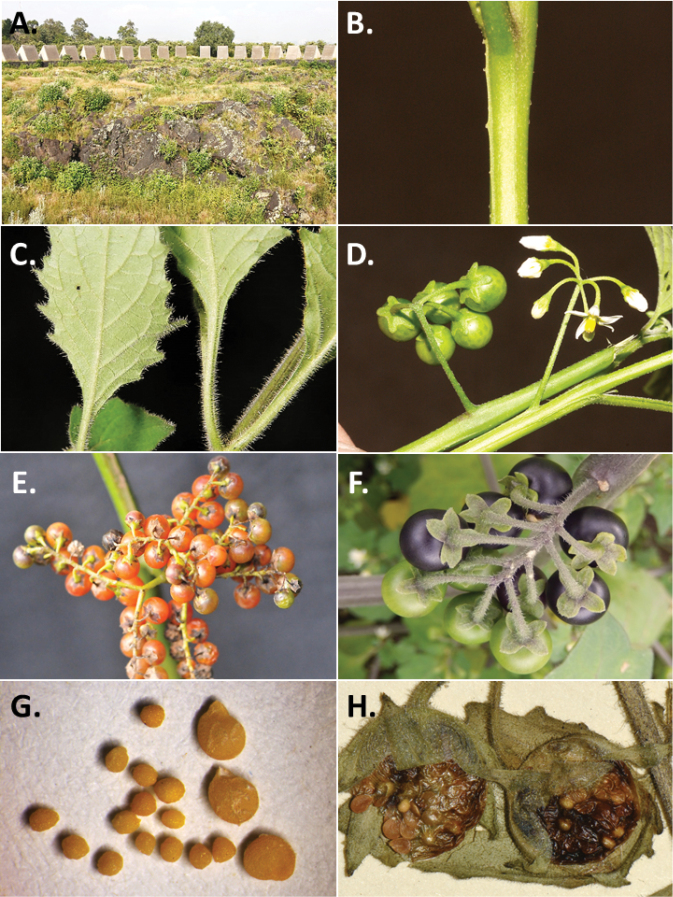
Morphology of the Morelloid Clade of *Solanum*. **A** Most species are found in disturbed habitats in the wild (*S.douglasii*, *Ochoterena et al. 979*) **B** small “spinose” processes are common on herbaceous stems in many species of black nightshades (*S.emulans*, *Nee 61357*) **C** glandular hairs are present in some species of morelloids (*S.pruinosum*, *Amith 30248*) **D** small stellate flowers in lateral inflorescences that are developmentally terminal characterise the clade (*S.emulans*, *Nee 61357*) **E** orange-red fleshy berries of *S.corymbosum* in highly branched inflorescences (*Särkinen et al. 4604B*) **F** black fleshy berries of *S.nigrum* (Nijmegen accession A44750150) **G** stone cells (also known as sclerotic granules or brachysclerids, L side of photo) are found in the fruits of most species of the Morelloid Clade and are round in shape as compared to the tear-drop shaped or ellipsoid seeds (R side of photo) (the African species *S.umalilaense* Manoko, Nijmegen accession A24750133) **H** stone cells are easy to see in herbarium specimens of some taxa (e.g., *S.sarrachoides*, *Blom 2s.n.* BM001207745). Photos by S. Knapp, T. Särkinen, and G. van der Weerden (previously published in “PhytoKeys 106”).

*Habit and stems*. Members of the Morelloid Clade are either herbs or shrubs; species range from annuals (e.g., *S.triflorum*) to short-lived perennials (e.g., *S.douglasii*), although some species can develop woody bases and appear to be somewhat shrubby (e.g., *S.nigrescens*). Stems are usually weak, and occasionally somewhat scrambling, but can reach 2 m in height (Fig. 2A). Plants of all species usually have herbaceous upper stems, even if the base is woody. The stems can be hollow (drying flattened, e.g., *S.scabrum*) or solid (e.g., *S.americanum*, *S.villosum*); this can be a useful character for identification of herbarium specimens.

Sympodial growth is characteristic of Solanaceae giving the stems a typical “zig-zag” appearance; details of sympodial structure have proved useful for infrageneric classification within *Solanum* (Child and Lester 1991; Knapp 2002a). Vegetative growth is initially monopodial, but with the onset of flowering, becomes sympodial. The inflorescence is developmentally terminal, and stem continuation is initiated in the axil of the leaf below each inflorescence. Each lateral shoot with alternate leaves arranged in a 1/3 phyllotaxic spiral and a terminal inflorescence is termed a sympodial unit. In some cases, when the axes of sympodial units are fused, the inflorescences appear to originate laterally from the middle of an internode; and when growth of the axes is suppressed, the leaves appear paired (geminate) at a node (Danert 1958). All the members of the Morelloid Clade have difoliate sympodial units with leaves not strongly paired (geminate) at the nodes (Fig. 2C, D), and the inflorescences often arise internodally through axis fusion (Danert 1958, 1967). Occasionally inflorescences appear to be opposite the geminate leaves (e.g., *S.sarrachoides*) especially on very young shoots (e.g., *S.americanum*).

“Spinose” processes are common on herbaceous stems in many species of black nightshades (Fig. 2B). They usually occur along the angles of upper parts of larger stems and are often decurrent from leaf bases. These are not true prickles, like those found in the “spiny” solanums (Leptostemonum Clade, see Vorontsova and Knapp 2016) but are similar in that they are outgrowths of the epidermis and are usually associated with trichomes as the enlarged basal portions of stem trichomes that have fallen off. They have been used to differentiate species in this group, but these structures are variable within species where they do occur, and even within stems on a single plant. In addition, they often change markedly in appearance when plants are pressed and dried. Their absence, however, can be diagnostic when combined with other characters.

*Leaves*. Species of the Morelloid Clade have simple leaves that are generally elliptic or ovate in outline (see Fig. 2 and species descriptions and illustrations). As with other vegetative characters in this group, leaf morphology can be extremely variable within a species or even in a single plant. Many of the infraspecific taxa described for the Eurasian species *S.nigrum* are based on leaf morphology, particularly in pubescence density and leaf margins.

Leaf margins vary from entire to quite deeply sinuate and lobed. Most populations of *S.triflorum* have deeply pinnatifid leaves, but a wing of leaf blade is always present along the midrib; some individuals, however, can have almost simple leaves. Other species have variously entire or toothed margins, often the teeth occur only in the basal half to third of the leaf blade. The leaf blade in members of the Morelloid Clade is usually somewhat decurrent on to the petiole and the leaf base is cuneate to attenuate. Leaf apices are acute to attenuate but vary considerably within species.

Petiole length to some extent is related to leaf size, and on individual plants larger leaves always have longer petioles. *Solanumscabrum* tends to have long petioles with little decurrent leaf blade tissue; this character can be helpful in distinguishing it from the otherwise very similar *S.nigrum*.

*Pubescence*. Trichomes in species of the Morelloid Clade are simple or branched (e.g., *S.pallidum* Rusby of the Andes), but never stellate (Seithe 1962; Roe 1971). The species treated here have only simple trichomes; these are usually 1–6-celled and uniseriate. Occasionally the trichome base is enlarged with the lowermost cell much larger than more distal cells and these enlarged bases persist as “pseudospines” on stems (see above, Fig. 2B). Much importance has been placed on differences in density of pubescence as a taxonomic character (e.g., the densely pubescent plants of *S.nigrum* sometimes recognised infraspecifically as var. or subsp. schultesii in European floras), but pubescence within taxa is continuously variable and apparently also related to environment, with plants growing in sunny sites often recorded on labels as more densely pubescent.

The presence or absence of glandular trichomes has also been previously treated as taxonomically significant (see Edmonds 1972, 1979b, 1982), with glandular and eglandular morphotypes being treated as separate subspecies or varieties (see Edmonds 2012). Manoko (2007; Manoko et al. 2008) showed that this character did not correspond to coherent groups in AFLP analyses of *S.nigrum* or *S.villosum*. Seithe (1962, 1979) showed that in most *Solanum* species glandular trichomes are found on cotyledons and hypocotyls of seedlings and are lost as plants mature; she suggested that species with glandular trichomes were more “primitive”. It is equally probable that the retention of glandular tips on trichomes is a simple paedomorphic character and that it has little taxonomic significance if not correlated with other characteristics. Some species treated here are only occasionally glandular (e.g., *S.triflorum*), with the glandular trichomes very small and sparse; in these plants eglandular trichomes dominate. *Solanumpruinosum* consistently possesses glandular trichomes (Fig. 2C) that are spreading and translucent, unlike the more appressed, white-coloured trichomes found in other taxa such as *S.nigrescens*.

Modern developmental work has not been undertaken with morelloid trichomes, but work has been done with the glandular trichomes of tomatoes and their relatives (e.g., Bergau et al. 2015). These studies suggest that these trichomes play a role in pest defence through release of metabolites in response to insect contact. Local ecological and herbivore pressures may also play a role in the presence or absence of glandular trichomes in the morelloids; this may help explain the highly heterogeneous distributions of glandular and eglandular individuals in some morelloid species.

*Inflorescences*. The inflorescence of members of the Morelloid Clade is developmentally terminal and later overtopped by the leading axillary shoot so that it appears lateral (Fig. 2D); this is common to many clades of the genus (an exception is the Pteroidea clade with axillary inflorescences, Knapp and Helgason 1997; Tepe and Bohs 2010). The basic structure is a scorpioid cyme that is unbranched or variously branched. Most members of the black nightshades have unbranched (simple) or merely forked (once-branched or furcate) inflorescences, but *S.corymbosum* has inflorescences that consistently branch more than once (Fig. 2E). The degree of branching in some species of the group may also depend upon plant or inflorescence age (e.g., *S.pseudogracile*). In all *Solanum* species the inflorescence expands from the tip producing flowers in a proliferating manner (Lippmann et al. 2008).

All members of the group have distinct peduncles, usually somewhat longer than the distal flower-bearing portion, but inflorescence length and flower number vary both between and within species. Many species in the group have what are termed “sub-umbellate” inflorescences, where the flower-bearing rhachis is very short and the pedicels are all very closely spaced and congested at the very tip of the inflorescence. We use the term sub-umbelliform in the species descriptions for this flower clustering (see Fig. 2D). This inflorescence is not a true umbel, but is described as such in much previous literature, usually as an umbellate or subumbellate cyme (e.g., Edmonds and Chewya 1997; Edmonds 2012). Both peduncles and pedicels usually have pubescence that is similar to that of the stems and leaves, or somewhat reduced distally. The peduncles of *S.chenopodioides* are unique in being strongly deflexed, creating a right or acutely downward facing angle with the stem (Fig. 7D).

Pedicels in flower are usually deflexed or spreading, but this can be very difficult to see in herbarium specimens. In fruit, pedicels are usually somewhat pendent from the weight of the berry, but are strongly (e.g., *S.chenopodioides*, *S.macrotonum*) or weakly (e.g., *S.nigrescens*) deflexed in some species. Other species have markedly spreading pedicels in fruit (e.g., *S.americanum*). The abscission zone at the pedicel base in members of the Morelloid Clade is at the very base, and if and when pedicels fall, the scars are generally flush with the rhachis. In *S.interius* the basal flower in the inflorescence has the pedicel articulation markedly above the base; this can be a useful identification character. Pedicel persistence with fruit ripening is an important species character in this group. Ripe berries either fall or are taken from the plant with the pedicel still attached (e.g., *S.emulans*, *S.nigrescens*) or the berry falls alone, and the pedicel is left behind (e.g., *S.americanum*, *S.villosum*). The presence of old pedicels can be useful for identification of non-flowering herbarium specimens.

*Calyces*. The calyx in all members of the Morelloid Clade is 5-merous and synsepalous. The calyx tube is generally conical or occasionally somewhat elongate (e.g., *S.corymbosum*), and the lobes are extremely variable in size and shape ranging from deltate and rounded (e.g., *S.americanum*) to long-triangular (e.g., *S.emulans*). The position of the calyx lobes in fruit is an important identification character; they can be strongly reflexed (e.g., *S.americanum*), spreading (e.g., *S.emulans*, *S.nigrescens*) or appressed to the berry (e.g., *S.corymbosum*, *S.douglasii*). The calyces of the weedy introduced species *S.nitidibaccatum* and *S.sarrachoides* are accrescent in fruit with the calyx lobes expanding to envelope almost the entire berry (several South American members of the group also have accrescent calyces; see Särkinen and Knapp 2016).

*Corollas*. In common with most species of *Solanum*, members of the Morelloid Clade have 5-merous sympetalous corollas that are variously stellate. Fasciated floral mutants are often observed, where 4–6-merous corollas can occur on individual plants that are otherwise 5-merous (e.g., *S.scabrum*). Colour is generally white or pale violet-tinged in the species treated here, but anthocyanin pigmentation can vary depending on environmental growth conditions. In most species at least some individuals (collections) with purple and violet flowers have been recorded (a single South American species of the group has pale yellow flowers *S.huayavillense* Del Vitto & Peten.; all of the species treated here have white or pale purple flowers). At the base of the corolla tube there is usually a ring or irregular area of differently coloured tissue usually referred to as the “eye”; in the species of the Morelloid clade this is usually yellow or greenish yellow. This eye is usually similar in texture to the rest of the corolla and not shiny as occurs in the Dulcamaroid Clade (see Knapp 2013); in some species the eye has darker brown or blackish purple margins (e.g., *S.nitidibaccatum*). The colours of the eye usually disappear in herbarium specimens and are rarely noted on labels.

Corollas in the Morelloid Clade are stellate, deeply stellate or rotate-stellate, and corolla lobes are deltate to long-triangular. *Solanumtriflorum* has deeply stellate corollas, with narrow, reflexed corolla lobes, whereas *S.corymbosum* has corollas with the lobes approximately the same length as the tubular portion, and the lobes are not strongly reflexed at anthesis. These characters, particularly those of the degree to which corolla lobes are reflexed, can be very difficult to see in herbarium specimens. As is seen in many other groups of solanums (e.g., Dulcamaroid Clade, African Non-Spiny Clade, see Knapp 2013; Knapp and Vorontsova 2016) where flowers last more than one day, the corolla lobes can be more or less reflexed through the life of the flower. Lobes are often spreading on day one, become reflexed to strongly reflexed on subsequent days, and as the flower ages, become spreading again.

Corollas of members of the Morelloid Clade are usually very small, as compared to other groups of *Solanum* species; these species have among the tiniest flowers of any *Solanum*. Corolla diameter varies from 4–20 mm; *S.nitidibaccatum* has the smallest corollas and *S.douglasii* the largest. Adaxial lobe surfaces are usually glabrous, while abaxial corolla lobe surfaces are variously papillate, with longer simple uniseriate trichomes on the margins and tips.

*Androecium*. The stamens of members of the Morelloid Clade are ellipsoid-anthered and equal to very slightly unequal in size and length. The filament tube and filaments are variously pubescent adaxially. The trichomes on filaments are simple and uniseriate; they are usually weak-walled and tangled. The filament tube is generally very short to almost absent and the free portion of the filaments is distinct. Filament length in comparison to anther length is a useful character for distinguishing species. In most species of morelloids the free portion of the filament is more or less equal to the anther length, but in some species pairs with otherwise similar anther length (e.g., *S.americanum*, *S.emulans*) differences in free filament length can be diagnostic (*S.emulans* has much longer filaments than does *S.americanum*). *Solanumdouglasii* and *S.nigrescens* are similar in overall stamen length, but *S.douglasii* has very short filaments and longer anthers, while the filaments and anthers of *S.nigrescens* are more equal in size. The length of filaments can affect the biophysical properties of anther vibration and thus vibratile pollination (e.g., Timerman et al. 2014; Switzer and Combes 2016), and may be an important characteristic involved in speciation in this group.

Anthers of members of the Morelloid Clade conform to the poricidal morphology of all other species of *Solanum* (see Knapp 2001). In common with other “non-spiny” solanums, the anther is ellipsoid and the terminal pore usually “unzips” during anthesis to become an elongate slit. The anthers are loosely connivent, and not connected by either “glue” (as in *S.dulcamara*, see Glover et al. 2004) or elongate papillae (as in the tomatoes, see Peralta et al. 2008). Anther size is an important identification feature in the Morelloid Clade, varying from less than 1 mm (*S.americanum*, *S.emulans*) to ca. 4 mm long (*S.douglasii*); in such small flowers, small differences can be very important. Pollen is not useful in distinguishing members of this group (Edmonds 1984).

*Gynoecium*. The gynoecium in members of the Morelloid Clade is bicarpellate; the carpels are fused in a superior ovary with axile placentation. The ovary is glabrous, and usually conical to globose. The flowers lack nectaries, as do all species of *Solanum*. The style is straight or slightly curved and usually sparsely to densely pubescent in the lower half to third where it is enclosed in the anther cone. It is usually exserted from the anther cone, but in some species (e.g., most populations of *S.americanum*, *S.corymbosum*) only barely exceeds the length of the stamens. This may be related to self-fertilisation and thus self-compatibility, as has been observed in the tomatoes (Rick et al. 1977, 1978, 1979; Rick and Tanksley 1981; Peralta et al. 2008), but all species of the Morelloid Clade tested have been self-compatible (Edmonds 1979a; Schilling and Heiser 1979; Eijlander and Stiekema 1994; Olet 2004). None of the species of the Morelloid Clade treated here has heterostylous flowers. The stigma is either very minutely capitate (e.g., *S.nigrescens*) or larger and more obviously globose-capitate (e.g., *S.douglasii*, *S.emulans*). The ovules are anatropous and non-arillate.

*Fruits*. As with all species of *Solanum*, the fruit is a bicarpellate berry. Fruits of members of the Morelloid Clade are usually brightly coloured and juicy (Fig. 2E, F). Most species have globose berries, but those of the introduced species *S.villosum* are usually somewhat longer than wide. Berry colour is usually green, yellowish green (e.g. *S.triflorum*) or varying shades of purple and purple-black (many species); immature berries are usually described as green on herbarium labels. *Solanumvillosum* has bright orange or yellow translucent berries and *S.corymbosum* has orange to red berries that are opaque (Fig. 2E). Colour polymorphisms are common in species of this group in the Old World; both *S.nigrum* and *S.tarderemotum* Bitter, for example, have individuals and populations with green or purple berries. Manoko (2007) showed that berry colour did not differentiate groups within European populations of *S.nigrum*. Despite this variation, berry colour is an important identification aid in this group, but is often not recorded on herbarium labels, especially of older specimens.

The pericarp (epicarp) of the berries is thin and either matte (e.g., *S.chenopodioides*, *S.emulans*) or shiny (e.g., *S.americanum*, *S.pseudogracile*). Surface characteristics are useful for species identification, especially when combined with other characters (see discussion of *S.americanum*). The mesocarp is always juicy and very liquid; these fruits are eaten by both birds and mammals (including people). In general, the mesocarp of fresh fruits is green or greenish yellow, but in species with purple berries it is sometimes purplish. Berries of some species are markedly translucent (e.g., *S.villosum*, Fig. 55E), while most species are opaque (e.g., *S.nigrum*, Fig. 2F, *S.interius*, Fig. 22D). Neither of these characters is usually mentioned on herbarium labels.

Like some other groups of non-spiny solanums such as the Pachystemonum (Cyphomandra) Clade (Bohs 1998) and the Archaesolanum Clade (Symon 1994) berries of members of the Morelloid Clade contain small, hard inclusions commonly referred to as sclerotic granules, stone cells or brachysclereids (Bitter 1911, 1914; in species descriptions here referred to as stone cells). These concretions are composed of modified sclerenchyma cells with massively enlarged cell walls (Fig. 2G, H); the stone cells of pears and quinces (Rosaceae) are classic examples of this cell type. Neither their function nor their origin in Solanaceae is known. Bitter (1914) suggested that they existed in an evolutionary series in the family, with more “advanced” taxa lacking them altogether (e.g., the spiny solanums). Some members of the Archaesolanum Clade have more stone cells than seeds in each berry (e.g., *S.aviculare* G.Forst. with an average of 12–55 seeds and 491–607 stone cells, Symon 1994). In the Morelloid Clade these stone cells are usually quite small and are always round in shape, ca. 0.5 mm in diameter, and brown to white in colour (Fig. 2G, L hand side of photograph). Stone cells can usually be easily seen in dried specimens without dissecting the berry (see fig. 1 in Bitter 1914; Fig. 2H); they appear globose and are often larger than the seeds. Sometimes stone cells of different sizes are found in the same berry, but this character is not consistent within species. The number of these is usually relatively consistent within a species, and varies from absent (e.g., *S.chenopodioides*, *S.retroflexum*, *S.scabrum*, *S.villosum*) to (1)2–4(–6) (e.g., *S.interius, S.macrotonum, S.nigrescens*) to more than 10 (e.g., *S.furcatum*, *S.triflorum*). *Solanumamericanum* varies from 0 to 4 stone cells per berry. Bitter (1914) reported that in crosses involving morelloid species with and without stone cells hybrids had stone cells present in the fruit, indicating that this was an inherited character. Cultivated species (e.g., *S.scabrum*, *S.villosum*) tend to lack stone cells; this may be related to human-mediated selection.

*Seeds*. Members of the Morelloid Clade have flattened seeds, like many other solanums. Unlike other groups, however, they are usually tear-drop rather than kidney shaped, with the hilum and micropyle at one of the short ends of the seed (see Fig. 2G, R hand side of photograph). Seed size varies from 1–3 mm long, and in general polyploid species have larger seeds than diploids (e.g., *S.americanum* seed size is 1–1.5 mm, while that of *S.scabrum* is 2–2.8 mm). *Solanuminterius*, however, has very large seeds (1.8–2 mm) and is diploid. Seed size is a good feature for distinguishing *S.nigrum* (hexaploid) from *S.americanum* (diploid). Seed number per berry in the Morelloid Clade is generally quite high (Särkinen et al. 2015b), with usually 30–50 seeds in each berry.

Seed coat morphology has been suggested as a useful character for species-level taxonomy in *Solanum* (Souèges 1907; Lester and Durrands 1984) and has been useful in delimiting groups in some clades (e.g., Geminata Clade, Knapp 2002a). All of the species treated here have sinuate-walled (digitate) testal cells. The lateral walls of these cells of the outer epidermal layer develop lignified radial thickenings that form as hair-like structures (Souèges 1907; Lester and Durrands 1984; Axelius 1992; Peralta et al. 2008). When the outer wall of the epidermis is removed, either naturally (e.g., by passage through frugivore guts; see Barnea et al. 1990) or by enzymatic digestion (Lester and Durrands 1984; Knapp 2002a) the seeds appear pubescent; seed measurements here include these projections. Edmonds (1983) examined seed coat patterns in some members of the Morelloid Clade (species previously included in SolanumsectionSolanum) and found no useful variation for delimiting either species or species groups.

*Chromosomes*. Chromosome numbers in the Morelloid Clade are variations on the base number of 12 (Table 2). The chromosomes are very small; an unvouchered diploid accession grown in India (Bhiravamurty 1975) had median, submedian and subterminal centromeres indicating variation in chromosome morphology. The Morelloid Clade, along with the potatoes, is one of the few lineages in *Solanum* where polyploidy is common (see discussion of polyploidy and hybridisation in Särkinen et al. 2018; Chiarini et al. 2018). Polyploidy is common in the Old World members of the group (Särkinen et al. 2015b, 2018), but less so for New World taxa (although many South American species have not had chromosome counts). Variation in ploidy level within a species is not common in *Solanum*, but *S.macrotonum* appears to have diploid (Central and South America) and hexaploid (South America) populations and similar variation occurs elsewhere in both morelloids (e.g., *S.interandinum* Bitter of the northern Andes) and in the potatoes (see Spooner et al. 2014). DNA amounts in unreplicated gametic nuclei (C-values) vary between 1.03 pg in *S.americanum* (as *S.nodiflorum*) and 3.10 pg in *S.nigrum* (Bennett and Leitch 2012).

**Table 2.** Chromosome counts for species of the Morelloid Clade of *Solanum*. For references see individual species treatments.

Species	Haploid chromosome number
*Solanumamericanum* Mill.	12 (2n=2×=24)
*Solanumchenopodioides* Lam.	12 (2n=2×=24)
*Solanumcorymbosum* Jacq.	–
*Solanumdouglasii* Dunal	12 (2n=2×=24)
*Solanumemulans* Raf.	12 (2n=2×=24)
*Solanumfurcatum* Dunal	36 (2n=6×=72)
*Solanuminterius* Rydb.	12 (2n=2×=24)
*Solanummacrotonum* Bitter	12 (2n=2×=24); 36 (2n=6×=72)
*Solanumnigrescens* M.Martens & Galeotti	12 (2n=2×=24)
*Solanumnigrum* L.	36 (2n=6×=72)
*Solanumnitidibaccatum* Bitter	12 (2n=2×=24)
*Solanumpruinosum* Bitter	–
*Solanumpseudogracile* Heiser	12 (2n=2×=24)
*Solanumretroflexum* Dunal	24 (2n=4×=48)
*Solanumsarrachoides* Sendtn.	12 (2n=2×=24)
*Solanumscabrum* Mill.	36 (2n=6×=72)
*Solanumtriflorum* Nutt.	12 (2n=2×=24)
*Solanumvillosum* Mill.	24 (2n=4×=48)

Many chromosome counts have been reported for members of this group, often as unvouchered counts of “Solanumnigrum”. In the species treatments we only report counts that are based on identifiable material or those that are vouchered and for which we have verified the specimen in question. Chromosome counts recorded in floras (e.g., Radford et al. 1964) without vouchers are not listed.

## Biology and natural history

*Habitats and distribution*. Members of the Morelloid Clade are plants of disturbed habitats and occur in landslides, along roads and streams, and at the edges of cultivated fields (Fig. 2A). Many of the species have broad elevational ranges (e.g., *S.americanum*) and extremely broad distributions (see Tables 3, 4 and 5). Species diversity in the region treated here is highest in North America (Canada and the United States; Table 3), with 7 native and 8 adventive species. This is largely due to the high numbers of adventive species occurring in the temperate parts of the continent as weeds of agriculture or escapes from cultivation, but *S.emulans* and *S.pseudogracile* are endemic to North America. The southeastern states of the United States have the highest species diversity on the North American continent (see Table 5). Central America and the Caribbean share three extremely common and widespread species (*S.americanum*, *S.macrotonum* and *S.nigrescens*); these also occur in Mexico and south to northern South America.

**Table 3.** Status and general distribution of the Morelloid Clade in the Caribbean, North and Central America; regionally endemic taxa are in boldface type.

Species	Status in the region	Distribution
* Solanum americanum *	native	Southeastern and west coast United States of America, Mexico, Central America and Caribbean islands
* Solanum chenopodioides *	introduced	Sporadically adventive in United States of America
* Solanum corymbosum *	introduced	Central Mexico
*** Solanum douglasii ***	**native**	**Southwestern United States of America to Nicaragua**
*** Solanum emulans ***	**native**	**Canada, United States of America**
* Solanum furcatum *	introduced	West coast of United States of America (California and Oregon)
*** Solanum interius ***	**native**	**United States of America**
* Solanum macrotonum *	native	Southern Mexico, Central America and Caribbean islands
* Solanum nigrescens *	native	Southeastern United States of America, Mexico, Central America and Caribbean islands
* Solanum nigrum *	introduced	Sporadically adventive in North America (spreading?)
* Solanum nitidibaccatum *	native	Common weed of agriculture; United States of America, Mexico (Baja California)
*** Solanum pruinosum ***	**native**	**Mexico**
*** Solanum pseudogracile ***	**native**	**United States of America**
* Solanum retroflexum *	introduced	Sporadic escape from cultivation in western United States of America
* Solanum sarrachoides *	introduced	Sporadic weed of agriculture; United States of America
* Solanum scabrum *	introduced	Only known from cultivation; Canada and United States of America
* Solanum triflorum *	native	Canada and United States of America
* Solanum villosum *	introduced	Sporadic escape from cultivation or on ship’s ballast, Canada, United States of America

**Table 4.** Morelloid species distribution by country for the Caribbean, North and Central America; introduced/adventive species are in brackets ().

Country	Species (introduced/adventive)
Anguilla	[no records]
Antigua and Barbuda	* americanum *
Bahamas	*americanum*, *nigrescens*
Barbados	* americanum *
Belize	*americanum*, *nigrescens*
Bermuda	* americanum *
British Virgin Islands	* americanum *
Canada (see Table 5 for province distribution)	*americanum*, *emulans*, (*nigrum*), *nitidibaccatum*, (*scabrum*), *triflorum*
Cayman Islands	*americanum*, *nigrescens*
Costa Rica	*americanum*, *macrotonum*, *nigrescens*
Cuba	*americanum*, *nigrescens*
Dominica	*americanum*, *nigrescens*
Dominican Republic	*americanum*, *macrotonum*, *nigrescens*
El Salvador	*americanum*, *douglasii*, *macrotonum*, *nigrescens*
Grenada	*americanum*, *nigrescens*
Guadeloupe	*americanum*, *nigrescens*
Guatemala	*americanum*, *douglasii*, *macrotonum*, *nigrescens*
Haiti	*americanum*, *macrotonum*, *nigrescens*
Honduras	*americanum*, *douglasii*, *nigrescens*
Jamaica	*americanum*, *macrotonum*, *nigrescens*
Martinique	* americanum *
Mexico	*americanum*, (*corymbosum*), *douglasii*, *macrotonum*, *nigrescens*, (*nitidibaccatum*), *pruinosum*
Montserrat	* americanum *
Netherlands Antilles (incl. Aruba)	* americanum *
Nicaragua	*americanum*, *douglasii*, *nigrescens*
Panama	*americanum*, *macrotonum*, *nigrescens*, (*nitidibaccatum*)
Puerto Rico	* americanum *
Saint Kitts and Nevis	*americanum*, *nigrescens*
Saint Lucia	* americanum *
Saint Vincent and the Grenadines	*americanum*, *nigrescens*
Trinidad and Tobago	*americanum*, *nigrescens*
United States of America (see Table 5 for state distribution)	*americanum*, (*chenopodioides*), *douglasii*, *emulans*, *interius*, (*furcatum*), *nigrescens*, (*nigrum*), *nitidibaccatum*, *pseudogracile*, (*retroflexum*), (*sarrachoides*), (*scabrum*), *triflorum*, (*villosum*)

**Table 5.** Distribution of morelloid species by political division in continental North America (Canada and the United States [excluding Hawaii*]) based on specimens seen and verified for this treatment. Many of these species are adventive and are to be expected to occur widely in disturbed habitats (e.g., *S.nigrum*, *S.nitidibaccatum*, *S.sarrachoides*); we have not listed records for which we have been unable to examine vouchers for verification. Species known only in cultivation are in parentheses (). Adventives are distinguished in Table 4 but not here. *Solanumpilcomayense* is known from two old collections (*Reverchon 3918* from Sabine Pass, Texas, coll. 1905; *Parker s.n.* from Camden, New Jersey, coll. 1874) and is listed here, but not treated (see pg. 4, 17).

**Canada**	
**Province/Territory**	**Species**
Alberta	* triflorum *
British Columbia	*americanum*, *emulans*, *nigrum*, *nitidibaccatum*, *triflorum*
Manitoba	*emulans, nitidibaccatum*, *triflorum*
New Brunswick	*emulans*, *nigrum*, *nitidibaccatum*
Newfoundland and Labrador	–
Nova Scotia	* nigrum *
Ontario	*emulans*, *nigrum*, *nitidibaccatum*
Prince Edward Island	–
Quebec	*emulans*, *nigrum*, *nitidibaccatum*
Saskatchewan	*emulans*, *triflorum*
**United States of America (excluding Hawaii – see Särkinen et al. 2018)**	
**State**	**Species**
Alabama	*americanum*, *emulans*, *nigrescens*, *pseudogracile*
Alaska	*nigrum*, *nitidibaccatum*
Arizona	*americanum*, *douglasii*, *nitidibaccatum*, *triflorum*
Arkansas	*emulans*, *nitidibaccatum*, *sarrachoides*
California	*americanum*, *chenopodioides*, *douglasii*, *furcatum*, *nigrum*, *nitidibaccatum*, *triflorum*
Colorado	*emulans*, *interius*, *nitidibaccatum*, *triflorum*
Connecticut	*emulans*, *sarrachoides*
Delaware	* emulans *
District of Columbia	*emulans*, *nigrum*, *triflorum*
Florida	*americanum*, *emulans*, *chenopodioides*, *nigrum*, *nigrescens*, *pseudogracile*, *sarrachoides*, *villosum*
Georgia	*americanum*, *chenopodioides*, *emulans*, *nigrum*, *pseudogracile*
Idaho	*interius*, *nigrum*, *nitidibaccatum*, *triflorum*
Illinois	(*americanum*), *emulans*, (*retroflexum*), *sarrachoides*, (*scabrum*), (*villosum*)
Indiana	* emulans *
Iowa	*emulans*, *interius*, *nigrum, triflorum*
Kansas	*emulans*, *interius*, *sarrachoides*, *triflorum*
Kentucky	* emulans *
Louisiana	*americanum*, *emulans*, *nigrescens*, *pseudogracile*
Maine	*emulans*, *nigrum*
Maryland	*chenopodioides, emulans*, *nigrum*, *sarrachoides*, *villosum*
Massachusetts	*emulans*, *nigrum*, *nitidibaccatum*, *triflorum*
Michigan	*emulans*, *triflorum*
Minnesota	*emulans*, *nitidibaccatum*, *triflorum*
Mississippi	*americanum*, *emulans*, *nigrescens*, *pseudogracile*
Missouri	*americanum*, *chenopodioides*, *emulans*, *nigrum*, *nitidibaccatum*, *sarrachoides*, *triflorum*, (*villosum*)
Montana	*interius*, *nigrum*, *nitidibaccatum*, *triflorum*
Nebraska	*emulans*, *interius*, *triflorum*
Nevada	*interius*, *nigrum*, *nitidibaccatum*, *triflorum*
New Hampshire	* emulans *
New Jersey	*emulans*, *nigrum*, *pilcomayense*, *villosum*
New Mexico	*douglasii*, *emulans*, *interius*, *nitidibaccatum*, *triflorum*
New York	(*americanum*), *emulans*, *nigrum*, *nitidibaccatum*
North Carolina	*chenopodioides*, *emulans*, *nigrescens*, *nigrum*, *nitidibaccatum*, *pseudogracile*, *sarrachoides*
North Dakota	*emulans*, *interius*, *nitidibaccatum*, *triflorum*
Ohio	* emulans *
Oklahoma	*emulans*, *interius*, *nigrum*, *sarrachoides*, *triflorum*
Oregon	*americanum*, *furcatum*, *nigrum*, *nitidibaccatum*, *triflorum*
Pennsylvania	*emulans*, *nigrum*, *nitidibaccatum*, *villosum*
Rhode Island	*emulans*, *sarrachoides*
South Carolina	*americanum*, *emulans*, *pseudogracile*, *sarrachoides*, (*villosum*)
South Dakota	*emulans*, *interius*, *triflorum*
Tennessee	* emulans *
Texas	*americanum*, *emulans*, *interius*, *nigrescens*, (*nigrum*), *nitidibaccatum*, *pilcomayense*, *pseudogracile*, *triflorum*
Utah	*americanum*, *interius*, (*nigrescens*), *nigrum*, *nitidibaccatum*, *triflorum*
Vermont	* emulans *
Virginia	*emulans*, *nigrum*, *sarrachoides*
Washington	*americanum*, *furcatum*, *nigrum*, *nitidibaccatum*, *sarrachoides*, *triflorum*
West Virginia	* emulans *
Wisconsin	*chenopodioides*, *emulans*, (*nigrum*), *nitidibaccatum*, (*scabrum*)
Wyoming	*emulans*, *interius*, *nitidibaccatum*, *triflorum*

*Although politically part of the United States of America, the islands of the state of Hawaii are biogeographically part of the Pacific and were treated in the monograph of the morelloids from the Old World that included the Pacific region (see Särkinen et al. 2018).

Adventive species from Europe such as *S.nigrum* and *S.villosum* are often recorded as growing on ballast near ports. *Solanumpilcomayense* (not treated here, see Introduction) of the Río Paraná drainage (Argentina, Paraguay, Brazil) has been recorded only twice, once in New Jersey and once in Texas, both times in the 19^th^ century; unlike *S.nigrum* and *S.villosum* however, it has not spread further, nor has it been collected recently. It must not be assumed, however, that only adventive non-natives occur on ballast, many collections of *S.emulans* have also been made on such disturbed sites along the eastern seaboard of the United States.

Several members of the group (e.g., *S.nigrum*, *S.nigrescens*, *S.nitidibaccatum*) are registered as noxious weeds of agriculture (see below) in both Europe and North America (Arnold 1985; Burgert et al. 1973; Ogg et al. 1981; Rogers and Ogg 1981; Defelice 2003; Orgeron et al. 2018). *Solanumtriflorum* is listed as a declared weed in Tasmania (Weed Management Act 1999 2000). Confusion over the identification of individual species (Ogg et al. 1981) and the common use of “S.nigrum agg.” in describing these species makes assessment of their invasive status very difficult in the absence of vouchers. Most morelloid species are weedy in nature.

We list the status and general distribution of the species in the group in Table 3, and in Table 4 document country distribution from herbarium specimens (see Materials and methods).

*Pollination and dispersal*. Like all solanums, flowers of members of the Morelloid Clade are buzz-pollinated by bees (Buchmann et al. 1977; De Luca and Vallejo-Marín 2013). Females of solitary bees and bumblebees vibrate the anthers with their indirect flight muscles causing pollen to “squirt” out of the terminal pores; they curl their bodies over the anther cone and rotate around the flower (Buchmann et al. 1977). The pollen is then groomed from the body and packed into the corbiculae, but the area of the venter that contacts the stigma of the next flower cannot be reached. Smaller bees visit and buzz individual anthers (Symon 1979), but do not usually contact the stigma and thus in solanums with large flowers are more properly seen as pollen thieves. Some bees also exhibit “milking” behaviour, where insects grasp the lower part of the anthers and try to force pollen out of the apical pores using upwards pressure (Buchmann et al. 1977). “Gleaning” of loose pollen grains is also done by various small bees and flies (Symon 1979; Knapp 1986). Buchmann et al. (1977) studied *S.douglasii* where flowers were visited and buzzed by a wide range of bees in various families, but no more recent pollination studies have been carried out. Few pollination studies have been carried out on the morelloid species.

Members of the Morelloid Clade have juicy berries with thin pericarp (skins) that are typical for bird-dispersed fruits (Knapp 2002b). Studies of dispersal of morelloid species have mostly been done on the species occurring in the USA with native bird and mammal frugivores (quail, American robins and deer mice; Tamboia et al. 1996). Green fruits are expected to be more attractive to mammals but Tamboia et al. (1996) found that both birds and mammals preferred the purple berries of *S.americanum* to the green berries of *S.sarrachoides* (probably = *S.nitidibaccatum*, no vouchers cited). The suite of characters expected to be attractive to mammals such as green colour, odour, and abscission shortly after ripening are all found in some of the morelloids, suggesting that mammals may be important fruit dispersers for these plants as well as birds.

Glycoalkaloid concentrations are very low in ripe berries of *S.americanum* and other members of the Morelloid Clade that have been tested, and alkaloid levels are similar across the clade (Cipollini et al. 2002). Higher concentrations in unripe fruit (Cipollini et al. 2002) of these species make them unattractive to frugivores (Cipollini and Levey 1997a). This loss of secondary metabolites in ripe berries is common across *Solanum* species with brightly coloured, fleshy fruits (e.g., Bradley et al. 1979) and is most likely related to fruit persistence (Cipollini and Levey 1997b), where risk of fungal infection is balanced by probability of animal ingestion and thus dispersal. Glycoalkaloids are known to have a constipative effect (see above, e.g., Gerard 1597) and to inhibit seed germination after ingestion (Cipollini and Levey 1997b), but Wahaj et al. (1998) found that ripe berries of *S.americanum* had a laxative effect on birds thus speeding seed passage through the gut. They suggested this was due to some other chemical compound (perhaps calystegines (?), see Dräger et al. 1994). Bravo et al. (2014), however, found that ingestion of seeds of *S.nigrum* by great bustards (*Otistarda*) reduced seedling emergence, but suggested that because seed numbers were so high, the bustrads still functioned as efficient seed dispersers.

*Conservation status*. Most morelloid species are weedy and widely distributed; in the Old World many species are also cultivated (e.g., *S.scabrum*, *S.tarderemotum*, *S.villosum*) and are distributed widely via human migration. Many introductions of species from Europe particularly to North America may have resulted from transport of soil or seed with introduced crops, but even casual visitors to far-flung places have been implicated in the introduction of alien species (Chown et al. 2012). It is likely that the early explorations of the southern hemisphere inadvertently carried seeds of nightshades with them, accounting for the widespread nature of many of these taxa. The genetic structure of populations of extremely widespread species such as *S.americanum* will need to be investigated to determine if structure exists in the distribution that can be related to natural or human-mediated causes.

Preliminary conservation assessments for the Caribbean and North and Central American members of the Morelloid Clade (including introduced taxa) are presented in Table 6. All of these species can be assigned the status of Least Concern; Table 6 records only the Extent of Occurrence (EOO), because Area of Occurrence (AOO) is highly influenced by collection or georeferencing deficit.

**Table 6.** Preliminary conservation assessments for morelloid species from the Caribbean and North and Central America. For details see Materials and Methods and individual species treatments. Preliminary assessments are based on EOO only (see Materials and Methods) and have been calculated for worldwide ranges for each species. The EOO and conservation status of species known to be solely cultivated, introduced or adventive in the region has been assessed in Särkinen et al. (2018).

Species	Preliminary conservation assessment (IUCN 2017)	EOO (km^2^) [worldwide range]
*Solanumamericanum* Mill.	LC	444,094,992
*Solanumchenopodioides* Lam.	LC	77,207,558
*Solanumcorymbosum* Jacq.	LC	1,621,244 (all); 148,300 (Mexico and Central America only)
*Solanumdouglasii* Dunal	LC	6,419,607
*Solanumemulans* Raf.	LC	5,394,300
*Solanumfurcatum* Dunal	LC	209,035,647 (North America only 4,169 – EN)
*Solanuminterius* Rydb.	LC	4,506,327
*Solanummacrotonum* Bitter	LC	3,804,650
*Solanumnigrescens* M.Martens & Galeotti	LC	15,340,166
*Solanumnigrum* L.	LC	78,076,619
*Solanumnitidibaccatum* Bitter	LC	See Särkinen et al. 2018
*Solanumpruinosum* Bitter	LC	294,305
*Solanumpseudogracile* Heiser	LC	1,048,309
*Solanumretroflexum* Dunal	LC	See Särkinen et al. 2018
*Solanumsarrachoides* Sendtn.	LC	100,440,077
*Solanumscabrum* Mill.	LC	See Särkinen et al. 2018
*Solanumtriflorum* Nutt.	LC	91,711,478
*Solanumvillosum* Mill.	LC	See Särkinen et al. 2018

## Uses

Black nightshades are used as potherbs (often referred to on English language labels as “spinach”) worldwide, especially in Africa. In the Americas, these plants are used in similar ways, especially among communities of African origin, but also more widely. It is not clear whether the use of leaves of morelloid solanums was brought to the Americas by enslaved peoples from Africa; it is more likely their use as potherbs developed in parallel indigenously on both continents.

Moerman (1998) records medicinal, culinary and other uses for several morelloid species amongst native peoples of North America. Some of these are identifiable to species (i.e., *S.douglasii*, S. *nitidibaccatum*, *S.triflorum*) but a great many uses are attributed to “*Solanumnigrum*”. Use of these plants to relieve loneliness due to death of family members among people of the Cherokee Nation living in the Smoky Mountains in North Carolina (Banks 2004) is likely to refer to *S.emulans*, as is the use of “*S.nigrum*” as “the best relished” potherb by the Cherokee in northwestern Georgia (Witthoft 1947). Ceremonial use in medicine ceremonies by the Objiwa (Chippewa)in Minnesota (Reagan 1928, quoted in Moerman 1998) certainly refers to *S.emulans*. The use of ripe berries as food by several peoples of coastal and central California (Tübatulabal, Ohlone, Mendocino) is likely to refer to *S.douglasii*, and the smoking of leaves to relieve toothache and as a remedy for scarlet fever among the Ohlone (as Costanoan) recorded by Harrington (Bocke 1984) also probably is attributable to *S.douglasii* (with the recorded common name of chichiquelite, taken from the Spanish). The Houma people of Louisiana’s use of a morelloid to treat worms in children could apply to *S.americanum*, *S.nigrescens* or *S.pseudogracile*. Where uses of these species are clearly of a single taxon (e.g., *S.douglasii*, *S.nitidibaccatum*, *S.triflorum* from Moerman 1998) or are vouchered they are recorded in the species treatments.

Many common names for these taxa in Mexico (see species treatments) include the suffix “-quelite” which is a generic term for potherbs. In the United States of America, the berries are eaten in pies and jams, but leaves appear not to be used as widely as they are further south (but see above and species treatments). Many manuals or floras list black nightshade berries as toxic (e.g., Kingsbury 1964), but see Särkinen et al. (2018) for a discussion of this in relation to plant chemistry.

## Species concepts

Our goal for the treatment of the Morelloid Clade has been to provide circumscriptions for the members of this morphologically variable group of species, while clearly highlighting areas, taxa and populations where further in-depth research would be useful. Delimitation of species here basically follows what is known as the “morphological cluster” species concept (Mallet 1995): i.e., “assemblages of individuals with morphological features in common and separate from other such assemblages by correlated morphological discontinuities in a number of features” (Davis and Heywood 1963). Biological (Mayr 1982), phylogenetic (Cracraft 1989) and the host of other finely defined species concepts (see Mallet 1995) are almost impossible to apply in practice and are therefore of little utility in a practical sense (see Knapp 2008a). It is important, however, to clearly state the criteria for the delimitation of species, rather than dogmatically follow particular ideological lines (see Luckow 1995; Davis 1997). Our decisions relied on clear morphological discontinuities to define the easily distinguished species. Specific characters used for recognition are detailed with each species description and in the key. Some potential reasons for variability and intergradation are recent divergence, hybridization and environmental influence on morphology. In this revision we have tried to emphasise similarities between populations instead of differences, which so often reflect incomplete collecting or local variation. We have not recognised subspecies or varieties, but have rather described and documented variation where present, rather than formalised such variability with a name which then encumbers the literature. We have been conservative in our approach, recognising as distinct entities those population systems (sets of specimens) that differ in several morphological characteristics. Many of the species in the group (and of morelloids in general) are extremely widespread and variable; variation exists in certain characters, but the pattern of variation is such that no reliable units can be consistently extracted, nor is geography a completely reliable predictor of character states. Here variability within and between populations seems more important than the variations of the extremes other taxonomists have recognised as distinct. We describe this variation realising that others may wish to interpret it differently.

Although infraspecific taxa have been recognised by others within the group, we do not recognise any here due to the complex morphological variation observed within each species, where the inspection of large number of specimens quickly reveals no apparent natural breaks in variation but rather a mixing between highly morphologically variable populations of widespread species.

## Materials and methods

Our taxonomic treatment is based on results from recent molecular systematic studies considering the taxonomy of the section and the molecular phylogenetic study of the entire Morelloid Clade by Särkinen et al. (2015b). Descriptions are based on field work and examination (physical and virtual) of 20,370 [=16,004 collections] herbarium specimens (of which 13,111 were from the New World) from 228 herbaria (A, AD, AK, ALCB, ANG, APSC, ARIZ, ASE, ASU, AZU, B, B-W, BA, BAH, BBLM, BH, BHCB, BHSC, BLMV, BM, BOIS, BP, BR, BRI, BRU, BRY, BSD, BSHC, BUT, C, CAL, CANB, CAS, CEN, CEPEC, CESJ, CGE, CHR, CICY, CLEMS, CM, CNS, COI, COL, COLO, CONN, CORD, CPUN, CR, CRMO, DBG, DD, DES, DNA, DS, DSC, DSM, DUKE, DVPR, E, EA, EAC, ECON, EIU, EKY, EMS, ESA, EWU, F, FCQ, FSU, FT, FUEL, FURB, G, G-DC, GA, GAS, GB, GH, GMUF, GOET, H, HAJB, HAMAB, HAO, HAS, HBG, HO, HOXA, HST, HUCS, HUEFS, HUEM, HUSA, HUT, IAC, ICN, ID, IDS, IFP, ILLS, INB, IND, INPA, IPA, JE, JEPS, JOI, JPB, K, KFTA, KHD, KIRI, KUFS, L, LAGU, LE, LEA, LIL, LL, LOJA, LP, LPB, LSU, LU, M, MA, MAC, MARY, MBM, MBML, MEL, MERL, MEXU, MHES, MHU, MICH, MIN, MISS, MISSA, MO, MOL, MONT, MONTU, MOR, MPU, MPUC, MT, MU, MY, NCU, NE, NEBC, NSW, NY, OBI, OS, OSC, OTA, OUPR, OXF, P, P-LA, PACA, PAL, PBL, PERTH, PH, PNNL, POM, PSM, Q, QAP, QCNE, RAB, RENO, RM, RSA, S, SASK, SD, SGO, SI, SJRP, SMDB, SOC, SRSC, TAN, TEX, TO, U, UB, UBC, UC, UCR, UDBC, UEC, UFP, UFRN, UMO, UNCC, UOS, UPCB, UPS, URV, US, USF, USM, USMS, UT, UTC, V, VEN, VIES, VPI, VT, W, WAG, WCW, WELT, WIS, WOLL, WTU, WU, WWB, YU, Z). Some of these specimens (especially for North America) were examined digitally through the portals of SEINet (http://swbiodiversity.org/seinet/index.php), the Southeast Regional Network of Expertise and Collections (SERNEC, http://sernecportal.org/portal/index.php), the Consortium of Northeast Herbaria (www.cnh.org), the Consortium of Midwest Herbaria (www.midwestherbaria.org), and the Consortium of Pacific Northwest Herbaria (http://www.pnwherbaria.org/); we include only those specimens we have been able to unequivocally identify from these images or that are duplicates of collections we have personally examined. We have compared introduced and adventive species across their entire ranges, not only collections from North and Central America and the Caribbean.

Measurements were made from dried herbarium material supplemented by measurements and observations from living material. Colours of corollas, fruits, etc., are described from living material or from herbarium label data. Specimens with latitude and longitude data on the labels were mapped directly. Some species had few or no georeferenced collections; in these cases, we retrospectively georeferenced the collections using available locality data. Maps were constructed with the points in the centres of degree squares in a 1° square grid. Conservation threat status was assessed following the IUCN Red List Categories and Criteria (IUCN 2017) using the GIS-based method of Moat (2007) as implemented in the online assessment tools in GeoCat (http://geocat.kew.org). The Extent of Occurrence (EOO) measures the range of the species, and the Area of Occupancy (AOO) represents the number of occupied points within that range based on the default grid size of 2 km^2^. We present only the EOO in the threat assessments for widespread species; AOO is very sensitive to georeferencing bias and collecting effort.

Type specimens for many morelloids have proved difficult to trace; most of the names for the introduced European species (e.g., *S.nigrum*, *S.villosum*) and for North and Central American species introduced elsewhere (e.g., *S.americanum*, *S.triflorum*) have been treated in Särkinen et al. (2018). Decisions on choices of lectotypes and synonymy can be found there.

Where specific herbaria have not been cited in protologues we have followed McNeill (2014) and designated lectotypes rather than assuming holotypes exist. We cite page numbers for all previous lectotypifications. In general, we have lectotypified names with the best preserved, or in some cases with the only, herbarium sheet we have seen; in these cases, we have not outlined our reasoning for the lectotypifications. Where there has been difficulty or where the choice may not be obvious, we detail our reasoning at the end of the species discussions (e.g., see Smith and Figueiredo 2011).

Georg Bitter described many taxa of *Solanum* in the course of his monumental work on the genus *Solanum* and worked widely in Germany in the period between the two World Wars (Weber 1928), including, but not exclusively at Berlin. His protologues frequently include specific herbarium citations, but often do not. We have cited specimens as holotypes only when a single specimen with a single herbarium citation is indicated in the protologue; we have not assumed his types are all in B. The collections of Edward Palmer made in the southwestern United States and Mexico were numbered for each collecting trip (McVaugh 1956), so care must be taken in assuming collections with the same number are duplicates, localities and years must also be taken into account.

D’Arcy (1974a) cited “type”, “syntype” or “lectotype” for many of the names treated here in his treatment of *Solanum* for Flora of Panama. He explicitly cited some of these as “lectotype” and we treat these as validly published lectotypifications because his intention was clear. We also treat his citations as “type” coupled with the citation of a single herbarium as unintentional (“inadvertent”) lectotypifications (e.g., Prado et al. 2015), following the stipulations of Art. 7.11 of the *Code* (Turland et al. 2018).

Type specimens with sheet numbers are cited with the herbarium acronym followed by the sheet number (e.g. SD [acc. # 6543]); barcodes are written as a continuous string in the way they are read by barcode readers (e.g., G00104280, MO-1781232), with the exception of those herbaria (e.g., A, GH, NY, US) where the barcode consists of only a number; here we have added the acronym to the string. For widely distributed and adventive species we have cited only types based on material from the Americas; the synonymy for *S.americanum*, *S.nigrum*, and *S.villosum* in particular is extensive and includes many names based on Old World collections. Details of names based on types from outside the Americas can be found in Särkinen et al. (2018) or on Solanaceae Source (www.solanaceasoursce.org).

Identities of all collections seen for this study are in Supplementary materials sections (Index to Numbered Collections; Suppl. material 1) and full searchable specimen details are available on the Solanaceae Source website (www.solanaceaesource.org), in Suppl. material 3 and in the dataset for this study deposited in the Natural History Museum Data Portal (https://doi.org/10.5519/0079673). We have not included traditional specimen citations in the main body of this paper but provide these as Suppl. material 2 (also see Tables 3 and 4). For all taxa we cite only specimens from the Caribbean, North and Central America, but Suppl. material 3 and the data set on the NHM Data Portal (https://doi.org/10.5519/0079673) include all material seen for all species treated here.

Citation of literature follows BPH-2 (Bridson 2004) with alterations implemented in IPNI (International Plant Names Index, http://www.ipni.org) and Harvard University Index of Botanical Publications (http://kiki.huh.harvard.edu/databases/publication_index.html). Following Knapp (2013) we have used the square bracket convention for publications in which a species is described by one author in a publication edited or compiled by another, the traditional “in” attributions such as Dunal in DC. for those taxa described by Dunal in Candolle’s *Prodromus Systematis Naturalis Regni Vegetabilis*. This work is cited here as Prodr. [A.P. de Candolle] and the names are thus attributed only to Dunal. For “ex” attributions we cite only the publishing author, as suggested in the *Code* (Turland et al. 2018). Standard forms of author names are according to IPNI (International Plant Names Index, http://www.ipni.org).

## Taxonomic treatment

### The Morelloid Clade

#### The Morelloid Clade, sensu Bohs (2005) and Särkinen et al. (2013, 2015b)

Solanum grad. ambig. Maurella Dunal, Hist. Solanum 119, 151. 1813. Lectotype. S.nigrum L. (designated by D’Arcy 1972).

SolanumsectionMorella Dumort., Fl. Belg. 39. 1827. Lectotype. S.nigrum L. (designated by D’Arcy 1972).

SolanumsectionInermis G.Don, Gen. Syst. 4: 400. 1838. Lectotype. S.nigrum L. (designated by D’Arcy 1972).

Solanum grad ambig. Morella G.Don, Gen. Syst. 4: 411. 1838. Lectotype. S.nigrum L. (designated by D’Arcy 1972).

SolanumsectionPachystemonum Dunal, Prodr. [A. P. de Candolle] 13(1): 28, 31. 1852. Lectotype. S.nigrum L. (designated by D’Arcy 1972).

SolanumsubsectionMorella Dunal, Prodr. [A. P. de Candolle] 13(1): 28, 44. 1852. Lectotype. S.nigrum L. (designated by D’Arcy 1972).

SolanumsectionCampanulisolanum Bitter, Repert. Spec. Nov. Regni Veg. 11: 234. 1912. Lectotype. S.fiebrigii Bitter (designated by Seithe 1962).

SolanumsectionEpisarcophyllum Bitter, Repert. Spec. Nov. Regni Veg. 11: 241. 1912. Lectotype. S.sinuatirecurvum Bitter (designated by Seithe 1962).

SolanumsectionMorella (Dunal) Bitter, Bot. Jahrb. 54: 416, 493. 1917. Lectotype. S.nigrum L. (designated by D’Arcy 1972).

SolanumsectionChamaesarachidium Bitter, Repert. Spec. Nov. Regni Veg. 15: 93. 1919. Type. S.chamaesarachidium Bitter (= S.weddellii Phil.).

Solanum series Transcaucasica Pojark., Bot. Mater.Gerb.Inst. Komorova Akad. Nauk S.S.S.R. 17: 332. 1955. Lectotype. S.transcauscasica Pojark. (= S.villosum Mill.) (designated by D’Arcy 1972 [as type]).

Solanum series Alata Pojark., Bot. Mater.Gerb.Inst. Komorova Akad. Nauk S.S.S.R. 17: 336.1955. Type. S.alatum Moench [nom. et typ. cons. prop.] (= S.villosum Mill.) (designated by D’Arcy 1972 [as type]).

Solanum series Pseudoflava Pojark., Bot. Mater.Gerb.Inst. Komorova Akad. Nauk S.S.S.R. 17: 338. 1955. Type. S.pseudoflavum Pojark. (= S.villosum Mill.) (designated by D’Arcy 1972 [as type]).

SolanumsectionParasolanum Child, Feddes Repert. 95: 142. 1984. Type. S.triflorum Nutt.

SolanumsectionDulcamara (Moench) Dumort. subsect. 2 “*herbaceous plants confined to the central Andes*” of Nee (1999: 295) [includes the species of Child’s section Parasolanum excluding the type]

SolanumsectionSolanum subsects. 1 “Solanum”, 2 “Glandular pubescent group”, 3 “Campanulisolanum”, 4 “Chamaesarachidium” and 6 “Episarcophyllum” of Nee (1999: 306–308), excluding his subsect. 5 “Gonatotrichum” [now recognised as being part of the Brevantherum clade, see Stern et al. 2013].

Solanum series Lutea Pojark. ex Ivanina, Bot. Zhurn. (Moscow & Leningrad) 85(6): 144. 2000. Type. S.villosum Mill.

##### Description.

Herbs, occasionally woody at the base; unarmed. Stems terete or angled, sometimes hollow, lacking true prickles but sometimes with prickle-like processes along the angles, glabrous or pubescent with simple or branched (only in South America) uniseriate trichomes, these eglandular or glandular. Sympodial units difoliate or trifoliate, the leaves usually not geminate. Leaves simple with entire or variously dentate or lobed margins or occasionally deeply pinnatifid, concolorous, glabrous to densely pubescent with eglandular and/or glandular simple or branched (only in South America) uniseriate trichomes; petioles generally well developed, the leaves never sessile. Inflorescences opposite the leaves or arising internodally, unbranched or many times branched, not bracteate (except in *S.triflorum* where a single bracteole sometimes present), with few to many (up to 100) flowers, these clustered at the tip (umbelliform or sub-umbelliform) or spaced along the rhachis; peduncle various, usually not longer than the inflorescence branches; pedicels articulated at the base (in *S.interius* the basal flower with the articulation slightly above the base). Flowers 5-merous (occasionally fasciate and 6–7-merous in *S.scabrum*), actinomorphic to very slightly zygomorphic in anther length and calyx lobe length, all perfect. Calyx with the lobes deltate to spathulate or long-triangular. Corolla stellate or rotate-stellate, white or purplish-tinged to lavender or purple, rarely pale yellow (South America only), usually with an “eye” at the base of the lobes of a contrasting colour (yellow, green or dark purple-black), the lobes spreading or reflexed at anthesis. Stamens equal or very slightly unequal, the filaments equal to very slightly unequal, glabrous or more usually densely pubescent with tangled uniseriate weak-walled simple uniseriate trichomes, the anthers ellipsoid (sometimes slightly tapering in *S.scabrum*) and connivent, with distal pores that elongate to slits with drying and/or age. Ovary conical, glabrous or occasionally very minutely puberulent; style straight or curved and bent, usually pubescent with simple uniseriate trichomes in the lower half, exserted from the anther cone, sometimes only very slightly so; stigma minutely capitate to capitate or clavate. Fruit a globose or somewhat elongate juicy berry with thin pericarp, green, black, yellow or red-orange at maturity, occasionally marbled with white (e.g., *S.nitidibaccatum*), opaque or translucent; fruiting pedicels spreading or deflexed; fruiting calyx lobes reflexed, appressed or accrescent at fruit maturity. Seeds flattened and tear-drop shaped, yellow or tan-brown. Stone cells absent or present, if present few to numerous. Chromosome number: n=12, 24, 36 (see section on Chromosomes, and individual species treatments).

##### Distribution.

A worldwide species group occurring in on all continents except Antarctica, but with highest species diversity in the central and southern Andes and Africa.

##### Discussion.

In the synonymy of the group presented here we have included all groups that are members of the clade as we define it, not only those containing species from North and Central America; for more detailed discussion of morphology and group definition see Särkinen et al. (2015b). *Solanumnigrum* is the lectotype species of *Solanum* (Hitchcock and Green 1929), and thus if this group were to be formally recognised at the infrageneric level it would necessarily be called [rank] *Solanum* (as recognised by Seithe 1962).

Members of the Morelloid Clade are among the most widely collected of solanums, in part because are they are herbaceous and widespread. They are also among the most difficult to identify, due to their extreme vegetative plasticity (see Morphology above) and their lack of striking distinguishing characters. Combinations of characters are most useful for identification and we have included these in the species treatments as well as in the keys. Geography is very helpful in assisting with species identification in this group, but the large number of potentially invasive and introduced species means one must exercise caution if a species is not readily identifiable (taking into account variation of course). We have limited our treatment of non-native introduced species to those that have become naturalised and persistent in the region (mostly in North America).

The Morelloid clade suffers from two extreme sorts of taxonomic recognition issues. Firstly, in many parts of the world (in more recent floras) all taxa are treated as a single highly variable species (usually *S.nigrum*, e.g., Standley and Morton 1938) and local endemic taxa are overlooked. Secondly, and especially in Europe in the late 19^th^ and early 20^th^ century, many minor variants were described and were then transferred and recombined at different taxonomic levels, creating a confusing morass of names, many of which lack types. The latter is unfortunate because of the nomenclatural work entailed in sorting out the identities and types for these names is time-consuming and often quite difficult (see Särkinen et al. 2018), but the former is more serious, because endemic taxa have been overlooked (e.g., *S.emulans*, *S.pseudogracile*) and thus have possibly been placed at risk due to their being equated with widespread invasive weeds.

We provide here a key to the group throughout the range treated in this monograph, and then separate keys for the Caribbean, North America (excluding Mexico) and Central America (including Mexico). We hope this will facilitate identification for those working on local floras, but it must be kept in mind that these species are sometimes adventive and may occur outside the ranges where we have encountered them. These plants are all remarkably similar and distinguishing features are usually minute differences in anther length; geography is often a good indicator of what species one has, but not always. Combinations of characters are useful in identifying these taxa and to this end we provide a synoptic character list after the geographical keys.

##### Artificial key to the species of the Morelloid Clade occurring in North and Central America and the Caribbean

**Table:** 

1	Foliage with glandular trichomes, the plants sticky to the touch	**2**
–	Foliage lacking glandular trichomes (sometimes with a few scattered trichomes with glandular tips, plants not markedly glandular); plants not sticky to the touch	**7**
2	Mature berry orange, red or dark yellow; rare weed of disturbed places; North America	*** S. villosum ***
–	Mature berry green, purple or black; adventive, native or cultivated plants; North America, Mexico, and Central America	**3**
3	Calyx lobes enlarged in fruit and enclosing the berry; weeds of agricultural land; North America	**4**
–	Calyx lobes not enlarged and enclosing the berry; plants of open places or cultivated; Mexico and North America	**5**
4	Leaf bases attenuate to cuneate; inflorescences mostly internodal, with 4–8(–10) flowers; corolla with a central greenish yellow star with black or purple margins; berries dark green to greenish brown, marbled with white, becoming translucent and shiny; stone cells 1–3, ca. 0.5 mm in diameter	*** S. nitidibaccatum ***
–	Leaf bases cordate or truncate; inflorescences mostly leaf-opposed, with 2–5(–7) flowers; corolla with a central greenish yellow star, no black or purple margins; berries pale green, not marbled with white, becoming matte, opaque, not marbled with white; stone cells 4–6, 0.8–1 mm in diameter	*** S. sarrachoides ***
5	Anthers to 2 mm long, usually less; fruit surface matte, with a glaucous cast; fruiting calyx lobes strongly reflexed; stone cells absent	*** S. retroflexum ***
–	Anthers more than 2 mm long; fruit surface more or less shiny, not glaucous or markedly matte; fruiting calyx lobes not strongly reflexed; stone cells present (2–6) or absent	**6**
6	Stone cells 4–6 in each berry; seeds ca. 1.5 mm long; inflorescence with flowers clustered near tip (sub-umbellate); glandular trichomes 0.5–2 mm long; Mexico, Central Volcanic Belt	*** S. pruinosum ***
–	Stone cells absent (rarely 2); seeds 1.8–2 mm long; inflorescence with flowers spaced along the rhachis; glandular trichomes to 0.5 mm long; North America, adventive	*** S. nigrum ***
7	Leaves deeply pinnatifid; prostrate herbs with fleshy leaves	*** S. triflorum ***
–	Leaves with entire or various toothed margins, but not deeply pinnatifid; plants erect or ascending (rarely prostrate with fleshy leaves [simple-leaved plants of *S.triflorum*])	**8**
8	Mature berries red or orange (green when immature)	**9**
–	Mature berries green, purple or black (green when immature)	**10**
9	Inflorescences 4–7 times branched; leaf margins entire, the leaves completely glabrous (occasionally sparsely ciliate); style ca. 2 mm long; anthers 0.8–1.5 (–1.8) mm long; stone cells 2, apical; central Mexico	*** S. corymbosum ***
–	Inflorescences unbranched (if branched merely forked); leaf margins toothed, the leaves pubescent, sometimes sparsely so; style > 2 mm long; anthers 1.8–2.2 mm long; stone cells absent; adventive in North America	*** S. villosum ***
10	Anthers less than or equal to 2 mm long	**11**
–	Anthers more than 2 mm long	**13**
11	Mature berries dropping with the pedicel; calyx lobes appressed to spreading in fruit; stone cells more than 4, usually 8 per berry; eastern and central North America	*** S. emulans ***
–	Mature berries dropping without the pedicel; calyx lobes strongly reflexed in fruit; stone cells absent or at most 2(4); widespread and subtropical or cultivated	**12**
12	Mature berry shiny black; corolla 3–6 mm in diameter, the lobes 2–3 mm long; leaves usually elliptic to ovate in outline; widespread, subtropical	*** S. americanum ***
–	Mature berry matte black with a glaucous cast; corolla 11–16 mm in diameter, the lobes 5–6 mm long; leaves rhomboid in outline; cultivated, rarely escaped	*** S. retroflexum ***
13	Buds narrowly ellipsoid to narrowly ovoid; corolla deeply stellate with narrowly triangular lanceolate lobes; berries shiny, with more than 10 stone cells	*** S. triflorum ***
–	Buds elliptic to ovoid to obovoid or elongate or globose (not narrow); corolla stellate with triangular to deltate lobes; berries matte or somewhat shiny, usually with fewer than 10 stone cells (but *S.furcatum* and *S.nigrescens* sometimes with more)	**14**
14	Stone cells in mature berries absent (occasionally 2); corolla less than 15 mm in diameter	**15**
–	Stone cells present in mature berries, always more than 2; corolla (10–)15–20 mm in diameter	**18**
15	Berries 10–20 mm in diameter, shiny, slightly flattened; fruiting pedicels strongly spreading; anthers somewhat tapering, often drying brownish orange; cultivated	*** S. scabrum ***
–	Berries less than 15 mm in diameter, somewhat shiny, matte or slightly glaucous, globose; fruiting pedicels weakly spreading or more usually deflexed; anthers ellipsoid, not drying brownish orange; native, adventive, or naturalised	**16**
16	Inflorescences with the flowers spaced along the rhachis; anthers 1.8–2.5 mm long; fruiting pedicels spreading; berry surface slightly shiny; seeds 1.8–2 mm long; adventive in North America	*** S. nigrum ***
–	Inflorescences with the flowers clustered at the tips (sub-umbelliform), only a few spaced along the rhachis; anthers more than 2 mm long; fruiting pedicels deflexed, usually strongly so; berry surface matte or glaucous; seeds 1–1.5 mm long; native or adventive	**17**
17	Peduncle in fruit strongly deflexed downwards; berries 4–9 mm in diameter; calyx lobes appressed to surface of berry in fruit; styles exserted to 1.5 mm from the anther cone at anthesis; adventive in North America	*** S. chenopodioides ***
–	Peduncle in fruit straight, not deflexed downward; berries 8–14 mm in diameter; calyx lobes reflexed in fruit; style exserted 2–2.5 mm from the anther cone at anthesis; coastal habitats, southeastern United States	*** S. pseudogracile ***
18	Buds globose or subglobose; style long-exserted from anther cone, even in bud; inflorescences usually forked; western North America	*** S. furcatum ***
–	Buds ellipsoid or ovoid; style exserted from the anther cone, but not in bud; inflorescences usually unbranched; widespread	**19**
19	Anthers (2.7)3–4.5 mm long; corolla (10–)15–20 mm in diameter	**20**
–	Anthers less than 3 mm long; corolla less than 15 mm in diameter	**21**
20	Fruiting pedicels 15–17 mm long, strongly deflexed; anthers ellipsoid with straight sides; buds ellipsoid or subglobose; free portion of the filaments half the length of the anthers; cloud forests, Mexico to Panama, Caribbean	*** S. macrotonum ***
–	Fruiting pedicels 10–12 mm long, deflexed but not strongly so; anthers slightly tapering to the tip; buds ovoid, tapering to the apex; free portion of the filaments minute, ca. 1/4 the length of the anther; montane and dry forests, southwestern United States to Nicaragua	*** S. douglasii ***
21	Basal flower in the inflorescence with the articulation above the rhachis; calyx lobes unequal, lanceolate, the longest one 1.7–4.5 mm long; seeds 1.8–2 mm long; stone cells 2–4; prairies and open woodlands, midwestern United States	*** S. interius ***
–	All flowers with the articulation at the inflorescence rhachis; calyx lobes equal, deltate, 0.5–1 mm long; seeds 1.2–1.5 mm long; stone cells usually more than 5; forests and coastal areas, southeastern United States, Caribbean, Mexico and Central America	*** S. nigrescens ***

##### Artificial key to the species of the Morelloid Clade occurring in the Caribbean

[we include *S.pseudogracile* here because although not yet recorded from the Bahamas, we suspect that it might occur there, considering its habitat and distribution in nearby Florida]

**Table:** 

1	Corolla to 20 mm in diameter; anthers more than 3 mm long (rarely slightly less); fruiting pedicels 15–17 mm long, strongly deflexed; cloud forests	*** S. macrotonum ***
–	Corolla always less than 20 mm in diameter; anthers 1.5–2.7 mm long; fruiting pedicels usually less than 15 mm, deflexed (not strongly so) or spreading; many habitat types	**2**
2	Anthers 1.2–1.5 mm long, almost globose; berry very shiny; widespread	*** S. americanum ***
–	Anthers more than 2 mm long, ellipsoid; berry matte or somewhat shiny, not very shiny; southeastern United States of America and the Caribbean	**3**
3	Stone cells absent in mature berries; calyx lobes obovate; styles exserted to 2.5 mm beyond the anther cone at anthesis; coastal dunes in southeastern United States of America	*** S. pseudogracile ***
–	Stone cells (2)6–8 (or more) in each mature berry; calyx lobes deltate to broadly deltate; style exserted ca. 1 mm beyond the anther cone at anthesis; many forest and open habitats throughout the Caribbean	*** S. nigrescens ***

##### Artificial key to the species of the Morelloid Clade occurring in North America (Canada and the United States [except Hawaii])

**Table:** 

1	Foliage with glandular trichomes, the plants sticky to the touch	**2**
–	Foliage lacking glandular trichomes (sometimes with a few scattered trichomes with glandular tips, not markedly glandular); plants not sticky to the touch	**6**
2	Mature berry orange, red or dark yellow, usually somewhat ellipsoid; calyx lobes with translucent sinuses; rare weed of disturbed places	*** S. villosum ***
–	Mature berry green, purple or black, usually globose or subglobose; widespread weedy species	**3**
3	Calyx lobes enlarged in fruit and enclosing the berry	**4**
–	Calyx lobes not enlarged and enclosing the berry	**5**
4	Leaf bases attenuate to cuneate; inflorescences mostly internodal, with 4–8 (–10) flowers; corolla with a central greenish yellow star with black or purple margins; berries dark green to greenish brown, marbled with white, becoming translucent and shiny; stone cells 1–3, ca. 0.5 mm in diameter	*** S. nitidibaccatum ***
–	Leaf bases cordate or truncate; inflorescences mostly leaf-opposed, with 2–5 (–7) flowers; corolla with a central greenish yellow star without black or purple margins; berries pale green, not marbled with white, becoming matte, opaque; stone cells 4–6, 0.8–1 mm in diameter	*** S. sarrachoides ***
5	Inflorescences with the flowers clustered near the tips; anthers 1.1.3–1.8 mm long; mature berry matte with a glaucous cast; seeds 1.3–1.5 mm long; cultivated	*** S. retroflexum ***
–	Inflorescences with flowers spaced along the rhachis; anthers 1.8–2.5 mm long; mature berry matte, but not glaucous; seeds 1.8–2 mm long; adventive	*** S. nigrum ***
6	Leaves deeply pinnatifid; prostrate herbs with fleshy leaves	*** S. triflorum ***
–	Leaves with entire or various toothed margins, not pinnatifid; plants erect or scrambling (rarely prostrate, if so the leaves fleshy)	**7**
7	Mature berry orange, red or dark yellow (shiny and translucent at maturity); calyx lobes with translucent sinuses; rare weed of disturbed places	*** S. villosum ***
–	Mature berries green, purple or black; calyx lobes without translucent sinuses; various habitats	**8**
8	Anthers less than or equal to 2 mm long	**9**
–	Anthers more than 2 mm long	**10**
9	Mature berries dropping with the pedicel; calyx lobes appressed to spreading in fruit; stone cells more than 4, usually 8 per berry; eastern and central North America	*** S. emulans ***
–	Mature berries dropping without the pedicel; calyx lobes strongly reflexed in fruit; stone cells absent or at most 2(4); widespread and subtropical or cultivated	**11**
10	Mature berry shiny; corolla 3–6 mm in diameter, the lobes 2–3 mm long; widespread, subtropical	*** S. americanum ***
–	Mature berry matte with a glaucous cast; corolla 11–16 mm in diameter, the lobes 5–6 mm long; cultivated, rarely escaped	*** S. retroflexum ***
11	Buds narrowly ellipsoid to narrowly ovoid; corolla deeply stellate with narrowly lanceolate strap-like lobes; berries shiny, with more than 10 stone cells; prostrate herbs	*** S. triflorum ***
–	Buds ellipsoid to ovoid to obovoid; corolla stellate with triangular to deltate lobes, not markedly lanceolate and strap-like; berries matte or somewhat shiny, usually with fewer than 10 stone cells; erect or straggling herbs	**12**
12	Stone cells in mature berries absent (occasionally 2); corolla less than 15 mm in diameter	**13**
–	Stone cells present in mature berries, always more than 2; corolla (10-)15–20 mm in diameter	**16**
13	Berries 10–20 mm in diameter, shiny, slightly flattened; fruiting pedicels strongly spreading; anthers somewhat tapering, often drying brownish orange; cultivated	*** S. scabrum ***
–	Berries less than 15 mm in diameter, somewhat shiny, matte or slightly glaucous, globose; fruiting pedicels weakly spreading or more usually deflexed; anthers ellipsoid, not drying brownish orange; native, adventive, or naturalised	**14**
14	Inflorescences with the flowers spaced along the rhachis; anthers 1.8–2.5 mm long; fruiting pedicels spreading; berry surface slightly shiny; seeds 1.8–2 mm long; sporadically adventive, most commonly along east and west coasts	*** S. nigrum ***
–	Inflorescences with the flowers clustered at the tips (sub-umbelliform), only a few spaced along the rhachis; anthers more than 2 mm long; fruiting pedicels deflexed, usually strongly so; berry surface matte or glaucous; seeds 1–1.5 mm long; native or adventive	**15**
15	Peduncle in fruit at right angles or more usually strongly deflexed downwards; berries 4–9 mm in diameter; calyx lobes appressed to surface of berry in fruit; styles exserted to 1.5 mm from the anther cone at anthesis; adventive in North America	*** S. chenopodioides ***
–	Peduncle in fruit slightly ascending, not deflexed downward; berries 8–14 mm in diameter; calyx lobes reflexed in fruit; style exserted 2–2.5 mm from the anther cone at anthesis; coastal habitats, southeastern United States	*** S. pseudogracile ***
16	Buds globose or subglobose; style long-exserted from anther cone, even in bud; inflorescences usually forked; western North America	*** S. furcatum ***
–	Buds ellipsoid or ovoid; style exserted from the anther cone, but not in bud; inflorescences usually unbranched; widespread	**17**
17	Anthers (2.7)3–4.5 mm long, slightly tapering to the tip; buds ovoid, tapering to the apex; free portion of the filaments minute, ca. 1/4 the length of the anther; corolla to 20 mm in diameter; montane and dry forests, southwestern United States	*** S. douglasii ***
–	Anthers less than 3 mm long, ellipsoid with straight sides; buds ellipsoid; free portion of the filaments half the length of the anthers; corolla less than 15 mm in diameter; east of the Rocky Mountains	**18**
18	Basal flower in the inflorescence with the articulation above the rhachis; calyx lobes unequal, lanceolate, the longest one 1.7–4.5 mm long; seeds 1.8–2 mm long; stone cells 2–4; prairies and open woodlands, midwestern United States	*** S. interius ***
–	All flowers with the articulation at the inflorescence rhachis; calyx lobes equal, deltate, 0.5–1 mm long; seeds 1.2–1.5 mm long; stone cells usually more than 5; forests and coastal areas, southeastern United States	*** S. nigrescens ***

##### Artificial key to the species of the Morelloid Clade occurring in the Central America (including Mexico)

**Table:** 

1	Foliage with glandular trichomes, the plants sticky to the touch	*** S. pruinosum ***
–	Foliage lacking glandular trichomes, the plants not sticky to the touch	**2**
2	Inflorescences many (4–7) times branched; mature berries red	*** S. corymbosum ***
–	Inflorescences unbranched or at most forked; mature berries green, purple or black	**3**
3	Anthers 1.2–1.5 mm long, almost globose; berries very shiny; pedicels not dropping with the berry, remaining on the rhachis; calyx lobes strongly reflexed in fruit; stone cells 0(4)	*** S. americanum ***
–	Anthers more than 2 mm long, ellipsoid or slightly tapering; berry matte or somewhat shiny, not very shiny; pedicels dropping with the berries, not remaining on the plant; calyx lobes appressed to somewhat reflexed in fruit, at least the bases appressed to the berry; stone cells 2–6(–13)	**4**
4	Free portion of the filaments ca. 1/4 the length of the anthers; buds ovoid, tapering at the apex; plants usually white-pubescent	*** S. douglasii ***
–	Free portion of the filaments from half as long as the anthers to almost equal their length; buds ellipsoid; plants glabrous to white pubescent	**5**
5	Anthers less than 3 mm long; corolla 8–10 mm in diameter; fruiting pedicels 10–12 mm long, somewhat deflexed; wide variety of forest types and open areas	*** S. nigrescens ***
–	Anthers more than 3 mm long; corolla to 20 mm in diameter; fruiting pedicels 15–17 mm long, strongly deflexed; cloud forests	*** S. macrotonum ***

##### Synoptical character list for the morelloids of North and Central America and the Caribbean

Taxa in parentheses indicate that these species are polymorphic for this character

Cultivated plants – retroflexum, scabrum

Leaves pinnatifid – triflorum

Leaves glandular – (nigrum), nitidibaccatum, pruinosum, (retroflexum), sarrachoides, (triflorum – very sparsely), (villosum)

Inflorescences branched more than once (i.e., highly branched, not merely forked) – (americanum), corymbosum

Basal flower in inflorescence with the pedicel articulation significantly above the rhachis – interius

Corolla eye with purple or darker coloration – chenopodioides, nitidibaccatum

Anthers 1.5 mm long or less, never longer – americanum, emulans, retroflexum

Anthers tapering – douglasii, scabrum

Anthers drying brownish tan – scabrum [cultivated]

Free portion of the filaments always much shorter than the anthers – americanum, douglasii

Style greatly exceeding the anther cone (equal to or longer than the anther cone) – douglasii, furcatum, nigrescens, pseudogracile

Berries red or orange at maturity – corymbosum, pruinosum?, villosum

Berries green at maturity – (nigrum), pruinosum?, triflorum

Berries very shiny – americanum, pseudogracile, (scabrum)

Berry pericarp translucent – americanum, villosum

Berries glaucous (i.e., whitish like blueberries) – chenopodioides, retroflexum

Berries more than 1 cm in diameter – scabrum, triflorum

Calyx in fruit strongly accrescent (at least partially covering the berry) – nitidibaccatum, sarrachoides

Pedicels remaining on plant after berries drop (deciduous berries) – americanum, nigrum, villosum

Stone cells absent – (americanum), chenopodioides, (nigrum), pseudogracile, retroflexum, scabrum, villosum

More than 6 stones cells per berry – emulans, (douglasii), furcatum, nigrescens, triflorum

##### Species descriptions

###### 1. Solanum americanum

Taxon classificationPlantaeSolanalesSolanaceae

Mill., Gard. Dict. ed. 8, no. 5. 1768

Figures 3 , 4

Solanum oleraceum Dunal, Encycl. [J. Lamarck & al.] Suppl. 3: 750. 1814. Type. “Antilles” *Herb. Richard s.n.* (lectotype, designated by D’Arcy 1974a, pg. 735: P [P00319557]; isolectotypes: G-DC [G00144258], MPU [n.v.]).
Solanum erythrocarpon G.Mey., Prim. Fl. Esseq. 109. 1818. Type. Suriname. Saramacca: Hamburg (Essequibo), *E.K. Rodschied 31* (lectotype, designated by Särkinen et al. 2018, pg. 52: GOET [GOET003505]).
Solanum nigrum Vell., Fl. Flumin. 85. 1829 [1825], nom. illeg., not Solanumnigrum L. (1753) Type. Brazil. [Rio de Janeiro]: “undequaeque nascitur” (lectotype, designated by Knapp et al. 2015, pg. 832: [illustration] Original parchment plate of Flora Fluminensis in the Manuscript Section of the Biblioteca Nacional, Rio de Janeiro [cat. no.: mss1198651_112] and later published in Vellozo, Fl. Flumin. Icon. 2: tab. 109. 1831).
Solanum tenuiflorum Steud., Nomencl. ed. 2, 2: 606. 1841. Type. Based on (replacement name for) Solanumnigrum Vell.
Solanum indecorum A.Rich., Hist. Fls. Cuba, Fanerogamia 11: 121. 1841. Type. Cuba. Sin loc., 1836, *R. de la Sagra s.n.* (lectotype, designated by Särkinen et al. 2018, pg. 52: P [P00370899]).
Solanum nigrum L. var. angulosum Sendtn., Fl. Bras. (Martius) 10: 16. 1846, as SolanumnigrumL.subsp.nodiflorum(Jacq.) Sendtn.var.angulosum Sendtn. Type. Based on Solanumtenuiflorum Steud. (= Solanumnigrum Vell.)
Solanum nigrum L. subsp. aguaraquiya Sendtn., Fl. Bras. (Martius) 10: 17. 1846. Type. Brazil. Rio Grande do Sul: “Pat. Joan a St. Barbara”, *C.F.P. Martius s.n.* (lectotype, designated by Särkinen et al. 2018, pg. 52: M [M-0171809]; isolectotype: M [M-0171810]).
Solanum nigrum L. var. minus Hook.f., Trans. Linn. Soc. London 20(2): 201. 1847, as “*minor*” Type. Ecuador. Galápagos Islands: James Island [Santiago], *C. Darwin s.n.* (lectotype, designated by Särkinen et al. 2018, pg. 52: CGE [CGE00297]; isolectotype: K [K000922162]).
Solanum amarantoides Dunal, Prodr. [A. P. de Candolle] 13(1): 55. 1852. Type. Brazil. Rio de Janeiro, *C. Gaudichaud 522* (lectotype, designated by D’Arcy 1974a, pg. 735 [as holotype]; second step designated by Särkinen et al. 2018, pg. 52: P [P00319574]; isolectotypes: P [P00319575], MPU [n.v.]).
Solanum pterocaulum Dunal var. aguaraquiya (Sendtn.) Dunal, Prodr. [A. P. de Candolle] 13(1): 52. 1852, as ‘*pterocaulon*’. Type. Based on SolanumnigrumL.subsp.aguaraquiya Sendtn.
Solanum ptychanthum Dunal, Prodr. [A. P. de Candolle] 13(1): 54. 1852. Type. United States of America. Georgia: Chatham Co., Savannah, *Anon. s.n.* (holotype: G-DC [G00144485]).
Solanum nodiflorum Jacq. var. macrophyllum Dunal, Prodr. [A. P. de Candolle] 13(1): 46. 1852. Type. Brazil. Rio de Janeiro: Rio de Janeiro, *C. Gaudichaud 521* (lectotype, designated by D’Arcy 1974a, pg. 735: P [P00319582]; isolectotypes: P [P00319583, P00319585], G-DC [G00144100], G [G00343373]).
Solanum nodiflorum Jacq. var. acuminatum Dunal, Prodr. [A. P. de Candolle] 13(1): 46. 1852. Type. Brazil. Minas Gerais: Sin loc., *M. Vauthier 537* (lectotype, designated by D’Arcy 1974a, pg. 735 [as type ex Herb. Drake]: P [P00319615]; isolectotypes: P [P00319614], G-DC [G00343360]).
Solanum nodiflorum Jacq. var. petiolastrum Dunal, Prodr. [A. P. de Candolle] 13(1): 46. 1852. Type. Brazil. Rio de Janeiro: Novo Friburgo, 1842, *P. Claussen 180* (holotype: P [P00319584]).
Solanum inops Dunal, Prodr. [A. P. de Candolle] 13(1): 55. 1852. Type. Mexico. “sin. loc.” [Tamaulipas: Tampico, 4 Feb 1827], *J.L. Berlandier 46* (holotype: G-DC [G00144469]; isotypes: BM [BM000775579], F [F0073104F], LE, P [P00336046, P00336047, P00336048], W [acc. # 1889-0291394, acc. # 1889-0144848]).
Solanum nigrum L. var. oleraceum (Dunal) Hitchc., Rep. Missouri Bot. Gard 4: 111. 1893. Type. Based on Solanumoleraceum Dunal
Solanum nigrum L. var. americanum (Mill.) O.E.Schulz, Symb. Antill. (Urban) 6: 160. 1909. Type. Based on Solanumamericanum Mill.
Solanum nigrum L. forma grandifolium O.E.Schulz, Symb. Antill. (Urban) 6: 160. 1909, as forma ‘*grandifolia*’ Type. Puerto Rico. “Prope Cayey in sylvis ad rivulum superiorem m. Sept. fl. et. fr.”, *P.E.E. Sintenis 2429* (no herbarium cited; no duplicates found).
Solanum nigrum L. forma parvifolium O.E.Schulz, Symb. Antill. (Urban) 6: 160. 1909, as SolanumnigrumL.var.americanum(Mill.) O.E.Schulzformaparvifolia O.E.Schulz. Type. Cuba. La Habana: Santiago de las Vegas, “Baker Herb. Cub. 3377” (no herbarium cited; no duplicates found).
Solanum minutibaccatum Bitter, Repert. Spec. Nov. Regni Veg. 10: 549. 1912. Type. Bolivia. La Paz: “San Carlos, bei Mapiri”, 750 m, Aug 1907, *O. Buchtien 1443* (lectotype, designated by Särkinen et al. 2018, pg. 54: US [US00027684, acc. # 1175843]; isotypes: GOET [GOET003478], NY [NY00172089]).
Solanum inconspicuum Bitter, Repert. Spec. Nov. Regni Veg. 11: 204. 1912. Type. Peru. Lima: Lima, 12 Jul 1910, *C. Seler 222* (holotype: B, destroyed; no duplicates found).
Solanum tenellum Bitter, Repert. Spec. Nov. Regni Veg. 11: 219. 1912. Type. Brasil. Minas Gerais: “Prope urbem Caldas florens fructibusque instructum”, 4 Oct 1869, *A.F. Regnell III 970* (holotype: UPS; isotype: US [US00027821, acc. # 201069]).
Solanum minutibaccatum Bitter subsp. curtipedunculatum Bitter, Repert. Spec. Nov. Regni Veg. 11: 205. 1912. Type. Bolivia. La Paz: Guanai-Tipuani, Apr-Jun 1892, *M. Bang 1462* (holotype: W; isotypes: BM [BM000617672], E [E00106087], M [M-0171808], MO [MO-503647], NDG [NDG42278], NY [NY00172090, NY00172091, NY00172092], PH [PH00030453], US [US00027685, acc. # 1324656; US02835359], WIS [0256198WIS]).
Solanum sciaphilum Bitter, Repert. Spec. Nov. Regni Veg. 11: 220. 1912. Type. Brazil. Santa Catarina: Pedras Grandes, Aug 1890, *E. Ule 1678* (holotype: B, destroyed, F neg. 2851; lectotype, designated by Särkinen et al. 2018, pg. 54: HBG [HBG511539]; isolectotype: HBG [HBG511540]).
Solanum curtipes Bitter, Repert. Spec. Nov. Regni Veg. 11: 228. 1912. Type. Paraguay. Cordillera: San Bernardino, Aug 1898–1899, *É. Hassler 3104* (holotype: B, destroyed; lectotype, designated by Morton 1976, pg. 149: G [G00306710]; isolectotypes: G [G00306711, G00306712, G00306713, G00306714], K [K000532497], P [P00325762], NY [NY00139112], UC [UC950837]).
Solanum calvum Bitter, Repert. Spec. Nov. Regni Veg. 12: 81. 1913. Type. Mexico. Baja California: Guadalupe Island, 1875, *E. Palmer 60* [pro parte] (holotype: UPS; isotypes: BM [BM001017192], MO [MO-159620, acc. # 5257812; MO-568722, acc. # 1713454], NY [NY00138967, NY00759880], YU [YU065319]).
Solanum nodiflorum Jacq. var. sapucayense Chodat, Bull. Soc. Bot. Genève, sér. 2, 8: 150. 1916. Type. Paraguay. Paraguarí: Sapucaí [“Sapucay”], 1914, *R. Chodat & W. Vischer 46* (holotype: G [G00306708]).

####### Type.^1^

Cultivated at the Chelsea Physic Garden [in protologue said to “grow naturally in Virginia”], *Herb. Miller s.n.* (lectotype, designated by Edmonds 1972, pg. 103 [as type]: BM [BM000617683]).

####### Description.

Annual to short-lived perennial herbs up to 1.5 m tall, subwoody at base. Stems terete or somewhat angled with ridges, older stems often appearing spinescent, not markedly hollow; new growth pubescent with simple, spreading, uniseriate 2–8-celled eglandular trichomes 0.2–0.8 mm long, often clustered along the stem angles; older stems glabrescent, with only the trichome bases persisting as pseudo-spines. Sympodial units difoliate, the leaves not geminate. Leaves simple, 3.5–10.5 cm long, 1.0–4.5 cm wide, ovate to elliptic; adaxial surface sparsely pubescent with simple, uniseriate trichomes like those on stem, these evenly spread along the lamina and the veins; abaxial surface similar but more densely pubescent; major veins 3–6 pairs; base attenuate, decurrent on the petiole; margins entire or occasionally sinuate-dentate; apex acute; petioles (0.3–)2.0–3.8(-4.0) cm long, sparsely pubescent with simple uniseriate trichomes like those on stems. Inflorescences 0.6–2.5 cm long, lateral and internodal, unbranched or rarely forked, with (3–)4–6(8) flowers (very rarely with many flowers in unusual many-branched inflorescences) clustered near the tips (umbelliform to sub-umbelliform), sparsely pubescent with simple uniseriate trichomes like those on stems; peduncle (0.5–)1.0–1.8 cm long, delicate; pedicels 3–9 mm long, 0.2–0.3 mm in diameter at the base and 0.4–0.5 mm at the apex, stout, straight and spreading, articulated at the base; pedicel scars spaced 0–0.5 mm apart, clustered at the tip of the inflorescence. Buds broadly ellipsoid, the corolla exserted 1/3 beyond the calyx lobe tips before anthesis. Flowers 5-merous, all perfect. Calyx tube 0.8–1.3 mm long, the lobes 0.3–0.5 mm long, 0.5–0.6 mm wide, broadly triangular with obtuse apices, sparsely pubescent with simple uniseriate trichomes like those of the stem. Corolla 3–6 mm in diameter, stellate, white with a yellow-green central portion near the base, lobed 1/2–2/3 of the way to the base, the lobes 2.0–3.2 mm long, 1.0–2.5 mm wide, strongly reflexed at anthesis, later spreading, densely papillate abaxially with 1–4-celled simple uniseriate trichomes, these denser on the tips and margins. Stamens equal; filament tube minute; free portion of the filaments 0.5–0.8 mm long, adaxially pubescent with tangled uniseriate trichomes; anthers 0.7–1.5 mm long, 0.5–0.6 mm wide, ellipsoid to almost globose and very plump-looking, yellow, poricidal at the tips, the pores lengthening to slits with age and drying. Ovary globose, glabrous; style 2.2–2.6 mm long, densely pubescent with 2–3-celled simple uniseriate trichomes 2/3 from the base where included in the anther cone, almost included to exserted 0.5(–1.0) mm beyond the anther cone; stigma minutely capitate, the surface minutely papillate, green in live plants. Fruit a globose berry, 4–9(–12) mm in diameter, purplish-black at maturity, opaque, the surface of the pericarp markedly shiny; fruiting pedicels 13–18 mm long, ca. 0.7–1.0 mm in diameter at the base and 0.8–1.0 mm in diameter at the apex, stout, straight and spreading, spaced ca. 1(-3) mm apart or tightly clustered, not falling with the fruit, remaining on the plant and persistent on older inflorescences; fruiting calyx lobes not accrescent, the tube less than 1 mm long, the lobes 1(–2) mm long, strongly reflexed at fruit maturity. Seeds 30–50 per berry, 1.0–1.5 mm long, 0.8–1.3 mm wide, flattened and tear-drop shaped with a subapical hilum, pale yellow, the surfaces minutely pitted, the testal cells pentagonal in outline. Stone cells mostly absent (Australia, South Pacific, and South America), but if present (North America, Mexico, Caribbean, Eurasia and Africa) 2–4(6) per berry, 2–4 larger ones >0.5 mm, and two smaller ones <0.5 mm in diameter. Chromosome number: *2n*=2×=24 (see Särkinen et al. 2018 for vouchers).

**Figure 3.**
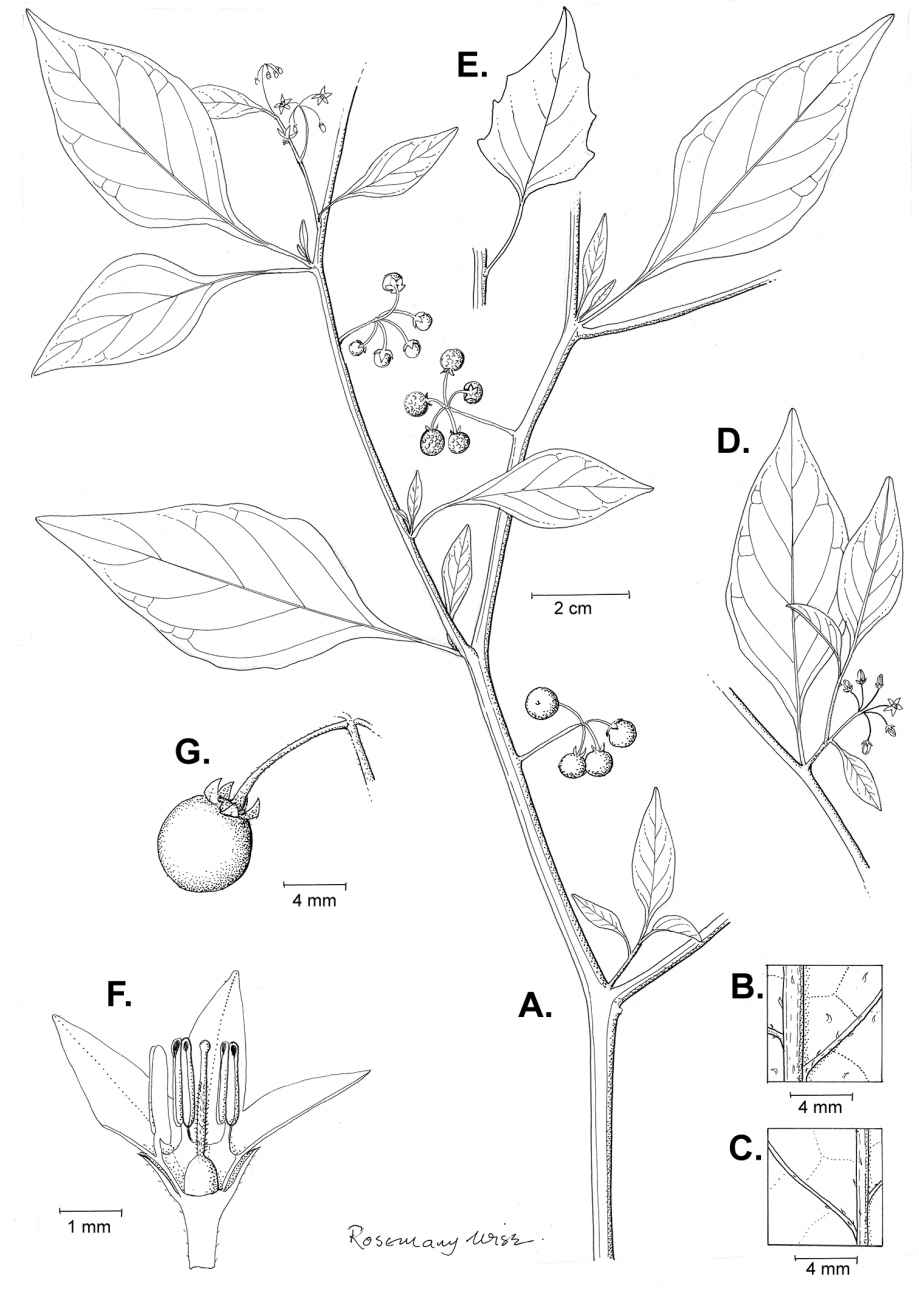
*Solanumamericanum* Mill. **A** Habit **B** detail of abaxial leaf surface **C** detail of adaxial leaf surface **D** branch with inflorescence **E** leaf **F** dissected flower **G** fruit (**A–D, F–G***Cremers 8084***E***Farrugia 2773*). Drawing by R. Wise (previously published in “PhytoKeys 106”).

**Figure 4.**
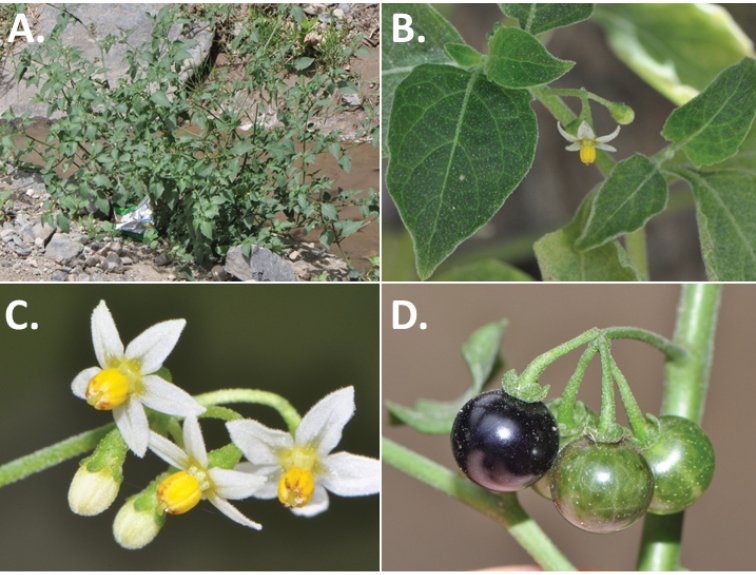
*Solanumamericanum* Mill. **A** Habit **B** leaves and young inflorescence **C** buds and flowers **D** mature, shiny black fruits with reflexed calyx lobes (**A, D***Knapp et al. 10210*; B *Knapp et al. 10205***C***Knapp et al. 10360*). Photos by S. Knapp.

####### Distribution.

**Figure 5.**
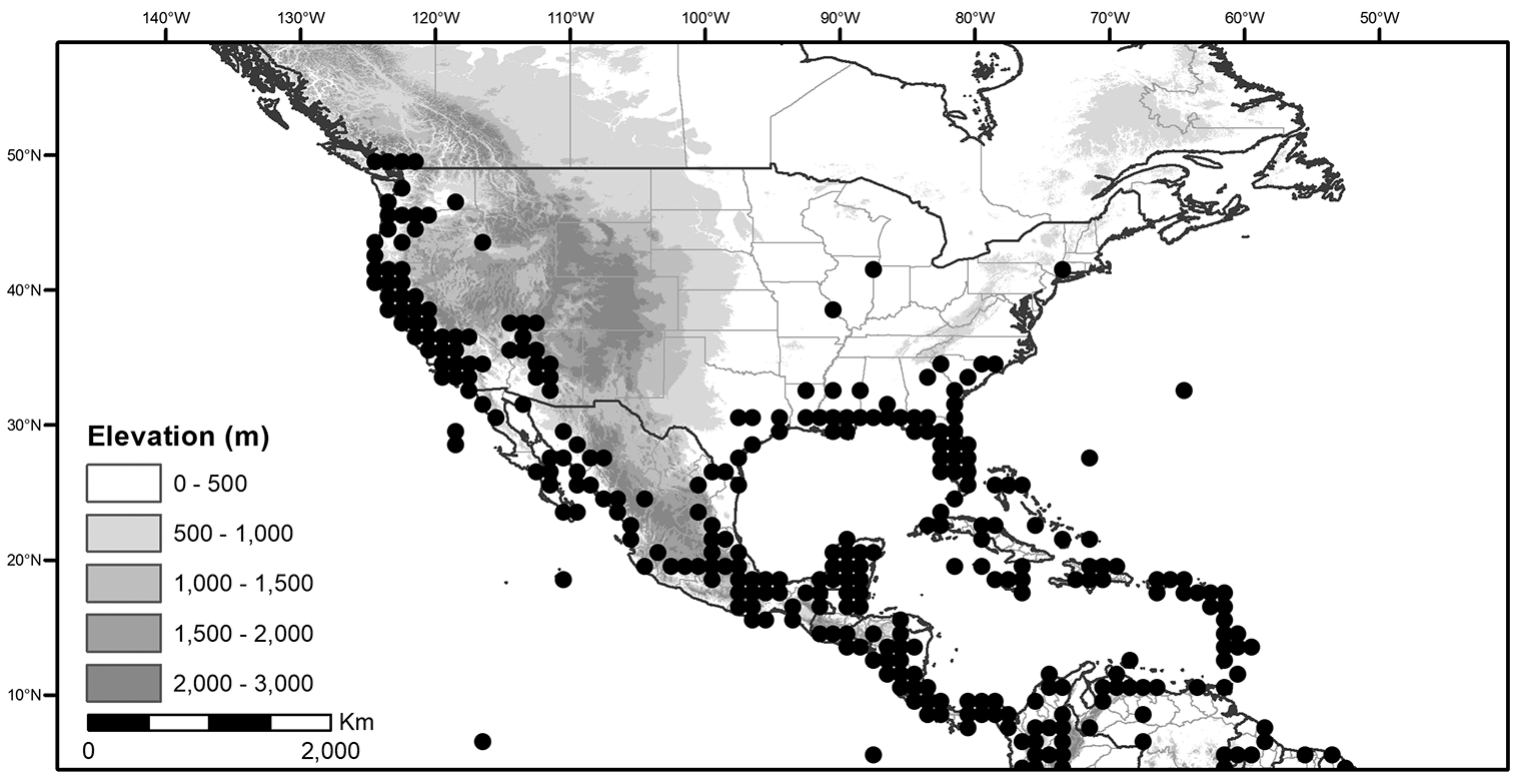
Distribution of *Solanumamericanum* Mill. (points in northern South America included to show continuous distribution).

(Figure 5) *Solanumamericanum* is a globally distributed weed found throughout the tropics and subtropics; it is not clear where it is native, or if this circumtropical distribution is its native range. In the region treated in this monograph it is commonest in Central America and the Caribbean, but is found around the coasts in North America, especially around the Gulf of Mexico and the Pacific.

####### Ecology.

*Solanumamericanum* is a weedy species that colonises disturbed soil and it is found in open areas, along roads, treefall gaps and at the back of beaches from sea level to 2,000 m elevation.

####### Common names.

United States of America. American nightshade (many sources), American black nightshade (Brown and Brown 1984; NatureServe 2017), Hierba mora negra (Correll and Johnston 1970). Mexico and Central America. Hierba (yerba) mora (many sources). Mexico [Guerrero] Saltonchis (*Ignacio 4*), [Oaxaca] Moo-jo-chi (*Hernández Ortega 62*), [Puebla] Pchfux-yáas (Zapotec, *Hunn OAX-205*), [Quintana Roo] Ik kootz (Maya, *Ucán Ek 4390*), [Sonora] Chichiquelite (*Gentry 1269*), [Veracruz] Tomatequelite (*Balvanera L. 259*), Wal ts’ok (Huastec, *Alcorn 2347*). Guatemala. [Alta Verapaz] macúy, [Santa Rosa] quilete (Gentry and Standley 1974). Belize. Bocano (Gentry and Standley 1974). Bahamas. Gooma bush, Ink berry (Correll and Correll 1982); gumma bush (*Richey 99–712*). French Antilles. Agouman, Herbe amère (Sastré and Breuil 2007). British West Indies [St. Lucia] Agouma (*Proctor 17826*). Trinidad and Tobago [Trinidad]. Agouma (*Broadway s.n.*).

####### Uses.

The leaves are widely used as a potherb in Mexico (“quelite”) and the countries around the Caribbean. Gentry and Standley (1974) state that in Guatemala the “foliage is used as one of the common pot herbs and is consumed in large quantities. It is found in most of the markets.”

####### Preliminary conservation status (IUCN 2017).

LC (Least Concern). *Solanumamericanum* is a cosmopolitan weed of the tropics and subtropics (see Särkinen et al. 2018). For EOO see Table 6.

####### Discussion.

*Solanumamericanum* is the most widespread and common species of the morelloid solanums (see Särkinen et al. 2018), and quite possibly the most widely distributed species in *Solanum*. It has been implicated as the diploid parent in the polyploid events that gave rise to the species of the Old World (e.g., Edmonds 1977; Poczai and Hyvönen 2011), although this has been disputed (Ma 1995). The name *S.americanum* has been in common use in North America (e.g., Stebbins and Paddock 1949) for what is now known as *S.emulans*, but more recently (Schilling 1981) the two taxa have been distinguished and the name *S.ptychanthum* has been used for the taxon for which the oldest name is *S.emulans*. The type specimen of *S.ptychanthum*, however, falls within the variation of *S.americanum*, so is treated as a synonym here. The application of the name *S.americanum* to any morelloid species with small anthers from northeastern North America should be viewed with caution.

*Solanumamericanum* can be easily recognised in fruit by its shiny black berries with small, strongly reflexed calyx lobes that are held on erect or spreading pedicels. In flower, the species has tiny almost globose anthers 0.8–1.5 mm long and short filaments usually less than 1 mm long. It has been often confused with *S.emulans* and *S.nigrescens*. *Solanumemulans* has equally short plump anthers but longer filaments (0.6–1 mm versus ca. 0.5 mm long), matte black or green fruits on deflexed pedicels and calyx lobes that are not markedly reflexed in fruit. Ripe berries of *S.americanum* are shiny black (but that can be difficult to see in herbarium specimens) and in North and Central America and the Caribbean usually have four stone cells in each. Berries of *S.emulans* have more than five stone cells. When berries ripen in *S.americanum* they fall from the plant leaving the stout, spreading pedicels behind, while berries drop off with the pedicels in *S.emulans* leaving only the peduncles behind in herbarium specimens. This can, however, be difficult to see in specimens with only very old inflorescences.

*Solanumnigrescens* differs from *S.americanum* in having larger anthers always more than 2 mm long, matte black or green fruits that are held on spreading or deflexed pedicels that drop with the berry, and calyx lobes appressed to the berry base in fruit. Berries of *S.nigrescens* have more than 5 (usually 5–6 large and several smaller) stone cells, while plants of *S.americanum* from this region have 2(-4). Inflorescences of *S.americanum* tend to be more sub-umbelliform in appearance than those of S. *nigrescens*, and calyx lobes of *S.americanum* are strongly reflexed and smaller relative to berry size in fruit. D’Arcy (1974a, b) suggests that *S.americanum* hybridizes with other diploid species (e.g., *S.nigrescens*) and that intermediates are common, but did not cite vouchers. Our observations (but see below) are that the two species are usually distinguishable using the characters above.

Some geographical trends in the morphological variation within the species can be observed, where populations along the coast of the Gulf of Mexico appear more hairy with duller grey-green leaf coloration, with more narrow, lanceolate rather than ovate leaves, with racemose inflorescences rather than strict umbels, with more rounded calyx lobes that do not always strongly reflex in fruit, and with generally larger fruits. The small anthers combined with stout and spreading pedicels in fruit that remain on the plant after fruits drop off are strong indications that these populations belong to *S.americanum* and do not represent a distinct species. Variation appears continuous and could be caused by local introgression from the sympatric diploid species *S.pseudogracile* or *S.nigrescens*. Collections with forked inflorescences (*Nee & McClelland 60259* from Florida; *Dancer s.n.* from Jamaica) are likely to be isolated aberrant individuals; in other parts of the world populations of *S.americanum* with highly branched inflorescences occur (e.g., China, type of *S.merrillianum* T.N.Liou) indicating that plasticity in this character is not unusual. It may also be that plants from cultivated populations in Asia have been brought with rice cultivation to North America. Plants collected as weeds of rice fields in Louisiana (Ma 1995; E. Shilling, pers. comm.) and identified as *S.merrillianum* are somewhat intermediate between *S.americanum* and *S.nigrescens* and could represent recent homoploid hybrids; preliminary data indicate they are members of the clade containing both those taxa. Further studies using molecular markers and carefully comparing Asian and American populations will be necessary to unravel this enigma.

Manoko et al. (2007) distinguished *S.americanum* and *S.nodiflorum* using AFLP markers; we re-examined the material they used and consider the plants they called *S.nodiflorum* to be *S.americanum* as defined here, and plants they called *S.americanum* represent specimens of *S.nigrescens* (see Särkinen et al. 2018: 61).

Typification details for the many synonyms of *S.americanum* can be found in Särkinen et al. (2018).

####### Specimens examined.

See Suppl. materials 1 and 3.

###### 2. Solanum chenopodioides

Taxon classificationPlantaeSolanalesSolanaceae

Lam ., Tabl. Encycl. 2: 18. 1794

Figures 6 , 7

Solanum sublobatum Willd. ex Roem. & Schult., Syst. Veg., ed. 15 bis [Roemer & Schultes] 4: 664. 1819. Type. Argentina. Buenos Aires, *Anon. s.n.* [probably *P. Commerson*] (*Herb. Willdenow 4336*) (lectotype, designated by Edmonds 1972, pg. 105 [as type ex photo]: B [B-W04336-01-0]).
Solanum besseri Weinm., Syst. Veg., ed. 15 bis [Roemer & Schultes] 4: 593. 1819. Type. “In America” [cultivated in Europe?], *Anon. s.n.* (no specimens cited; no original material located; neotype, designated by Särkinen et al. 2018, pg. 65: G-DC [G00144198]).
Solanum subspatulatum Sendtn., Fl. Bras. (Martius) 10: 45, tab. 4, fig. 16–18. 1846. Type. Brazil. Sin. loc., *F. Sellow s.n.* (holotype: B, destroyed, F neg. 3183; lectotype, designated by D’Arcy 1974a, pg. 735 [as type]: P [P00384051]; isolectotype: F [v0361921F, acc. # 621700, fragment]).
Witheringia chenopodioides (Lam.) J.Rémy, Fl. Chil. [Gay] 5: 69. 1849. Type. Based on Solanumchenopodioides Lam.
Solanum isabellei Dunal, Prodr. [A. P. de Candolle] 13(1): 153. 1852. Type. Uruguay. Montevideo, Lat. aust. 34°45'08", 1838, *A. Isabelle s.n.* (lectotype, designated by Särkinen et al. 2018, pg. 65: G-DC (G00145645); isolectotypes: F [v0073298F, acc. # 680251; v0073299F, acc. # 680253], K [K000585686], P [P00384071], W [acc. # 1889-115034]).
Solanum chenopodiifolium Dunal, Prodr. [A. P. de Candolle] 13(1): 44. 1852. Type. Argentina/Uruguay. “Buenos Aires et Montevideo”, *P. Commerson s.n.* (lectotype, designated Edmonds 1972, pg. 108 [as holotype], second step designated by Särkinen et al. 2018, pg. 65: P [P00384081]).
Solanum crenatodentatum Dunal var. ramosissimum Dunal, Prodr. [A. P. de Candolle] 13(1): 54. 1852. Type. United States of America. Louisiana: “Basse Louisiane”, 1839, *G.D. Barbe 82* (holotype: P [P00362535]).
Solanum gracile Dunal, Prodr. [A.P. de Candolle] 13(1): 54. 1852, nom. illeg., not Solanumgracile Sendtn. (1846). Type. Brazil. Rio de Janeiro: “Rio de Janeiro”, 1831–1833, *C. Gaudichaud 520* (lectotype, designated by Henderson 1974, pg. 46: G-DC [G00144391]; isolectotypes: G [G00343457], P [P00384052, P00384053]).
Solanum gracile Dunal var. microphyllum Dunal, Prodr. [A. P. de Candolle] 13(1): 54. 1852. Type. Argentina/Uruguay. “Circa Buenos Ayres et Montevideo”, *P. Commerson s.n.* (lectotype, designated by Morton 1976, pg. 151: P [P00384061, Morton neg. 8207]; possible isolectotype: F [v0073283F, acc. # 976485, fragment only]).
Solanum nodiflorum Jacq. var. microphyllum Hassl., Repert. Spec. Nov. Regni Veg. 9: 118. 1911. Type. Paraguay. Estrella: Mar, *É. Hassler 10271* (holotype: G?, Morton neg. 8612).
Solanum vile Bitter, Repert. Spec. Nov. Regni Veg. 11: 221. 1912. Type. Brazil. Rio de Janeiro: Restinga do Harpoador, *E. Ule 4310* (lectotype, designated by Särkinen et al. 2018, pg. 66: CORD [CORD00004277]; isolectotype: HBG [HBG-511507]).
Solanum gracilius Herter, Rev. Sudamer. Bot. 7: 266. 1943. Type. Based on (replacement name for) S.gracile Dunal
Solanum ottonis Hyl., Uppsala Univ. Årsskr. 7: 279. 1945. Type. Based on (replacement name for) Solanumgracile Dunal

####### Type.^2^

Mauritius. “Ex ins. Mauritiana”, *Herb. Lamarck s.n.* (lectotype, designated by Barboza et al. 2013, pg. 242: P [P00357629]).

####### Description.

Annual herbs to short-lived perennial shrubs up to 1.0 m tall, subwoody and branching at base. Stems terete, green-grey to straw colour, sprawling, somewhat weak and decumbent, not markedly hollow; new growth pubescent with simple, uniseriate appressed 1–6-celled eglandular trichomes, these 0.1–0.6 mm long; older stems more sparsely pubescent, glabrescent. Sympodial units difoliate, the leaves not geminate. Leaves simple, 1.5–5.5(–7.0) cm long, 0.5–3.0(–3.5) cm wide, lanceolate to narrowly ovate, rarely ovate, discolorous; adaxial surface green, sparsely pubescent with appressed 1–4-celled translucent, simple, uniseriate trichomes like those on stem, these denser along the veins; abaxial surface pale grey, more densely pubescent with trichomes like those of the upper surface evenly distributed across lamina and veins; major veins 3–6 pairs, not clearly evident abaxially; base attenuate, decurrent on the petiole; margins entire or sinuate; apex acute to obtuse; petioles (0.5-)1.0–1.5(–3.5) cm long, sparsely pubescent with simple uniseriate trichomes like those of the stems and leaves. Inflorescences 1.0–2.5(–4.0) cm long, lateral, generally internodal but appearing leaf-opposed on young shoots, unbranched or rarely forked, with 3–7(–10) flowers clustered near the tips (sub-umbelliform), sparsely pubescent with appressed 1–2-celled simple uniseriate trichomes; peduncle 1.0–2.3(–4.0) cm long, strongly deflexed downwards in fruit; pedicels 5–10 mm long, ca. 0.5 mm in diameter at the base and 1 mm in diameter at the apex, straight and spreading, articulated at the base; pedicel scars spaced ca. 0–1 mm apart. Buds elongate-oblong, the corolla only slightly exserted from the calyx tube before anthesis. Flowers 5-merous, all perfect. Calyx tube 2–3 mm long, conical, the lobes 0.6–1.2 mm long, less than 1 mm wide, broadly deltate to triangular with acute to obtuse apices, sparsely pubescent with 1–4-celled appressed hairs like those on stem but shorter. Corolla 6–12 mm in diameter, white with a black and yellow-green central portion near the base, the black colour usually distal to the yellow green, deeply stellate, lobed 4/5 of the way to the base, the lobes 3.5–4.0 mm long, 1.5–1.9 mm wide, strongly reflexed at anthesis, later spreading, densely puberulent-papillate abaxially with 1–4-celled simple uniseriate trichomes, these denser on the tips and margins. Stamens equal; filament tube minute; free portion of the filaments 0.6–1.0 mm long, adaxially pubescent with simple tangled uniseriate 4–6-celled simple trichomes; anthers (2.0–)2.3–2.8 mm long, 0.5–0.8 mm wide, narrowly ellipsoid, yellow, poricidal at the tips, the pores lengthening to slits with age and drying, the connective becoming darker brown with age in dry plants. Ovary globose, glabrous; style 3.7–4.5 mm long, densely pubescent with 2–3-celled simple uniseriate trichomes in the lower half where included in the anther cone, exserted up to 1.5 mm beyond the anther cone; stigma capitate, minutely papillate, green in live plants. Fruit a globose berry, 4–9 mm in diameter, dull purplish-black at maturity, opaque, the surface of the pericarp matte and somewhat glaucous; fruiting pedicels 6–13 mm long, 1.2–1.4 mm in diameter at the base, reflexed and slightly curving, dropping with mature fruits, not persistent; fruiting calyx not accrescent, the tube less than 1 mm long, the lobes 1–1.5 mm long, appressed against the berry. Seeds (13–)20–35(–50) per berry, 1.2–1.4 mm long, 1.0–1.2 mm wide, flattened and tear-drop shaped with a subapical hilum, pale yellow, the surfaces minutely pitted, the testal cells pentagonal in outline. Stone cells absent. Chromosome number: *2n*=2×=24 (see Särkinen et al. 2018).

**Figure 6.**
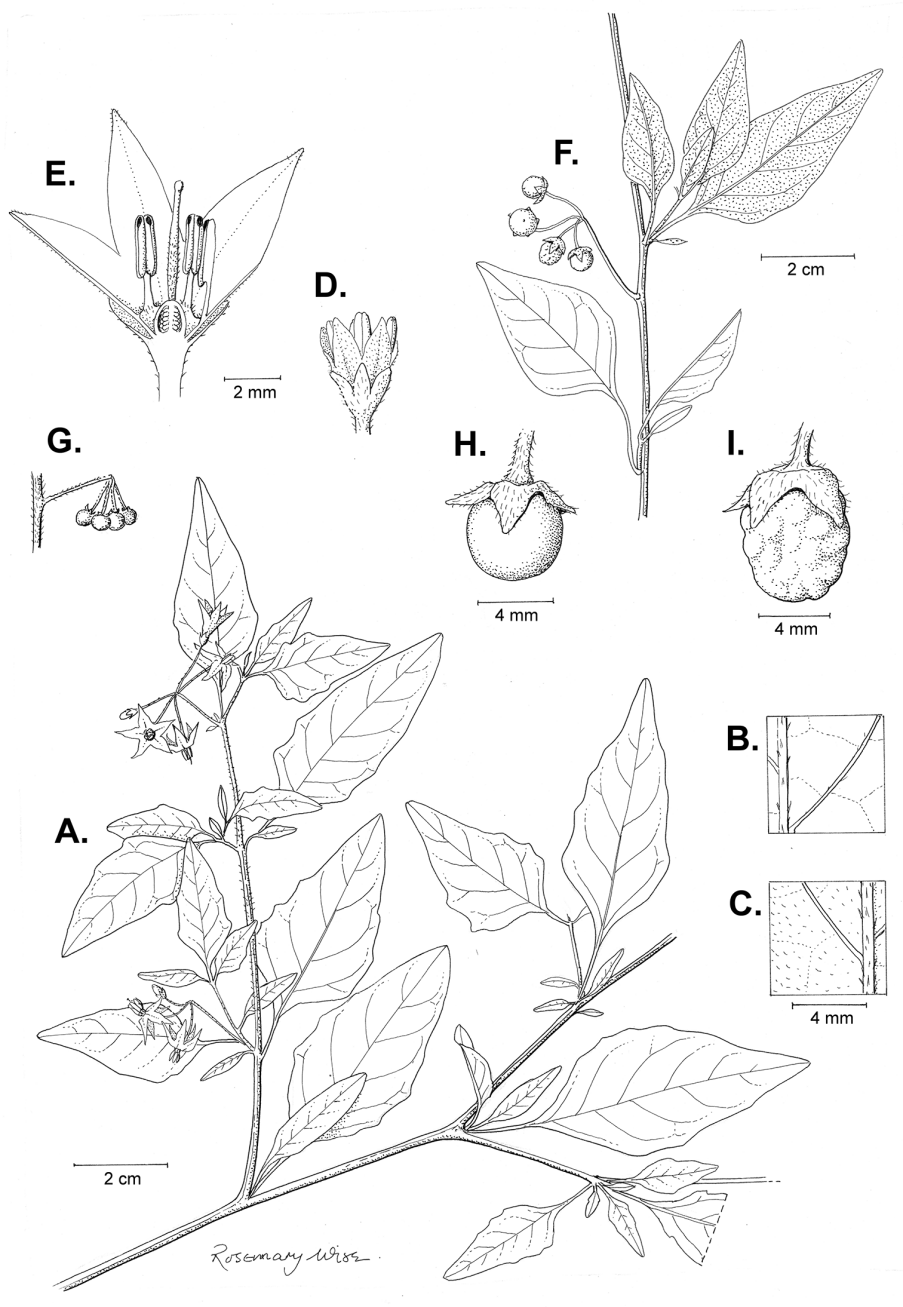
*Solanumchenopodioides* Lam. **A** Habit **B** detail of adaxial leaf surface **C** detail of abaxial leaf surface **D** opening bud **E** dissected flower **F** fruiting branch **G** detail of infructescence **H** maturing fruit **I** fully mature fruit (**A–E***Fox s.n.***F–I***Hieronymus s.n.*). Drawing by R. Wise (previously published in “PhytoKeys 106”).

**Figure 7.**
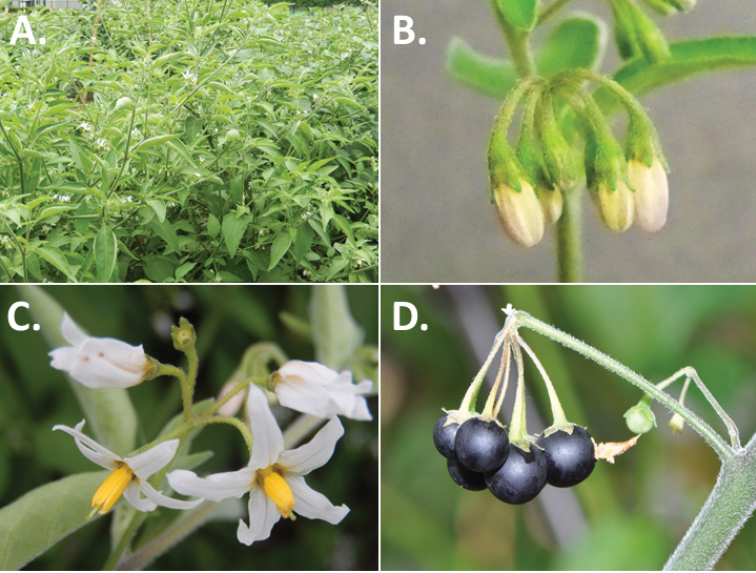
*Solanumchenopodioides* Lam. **A** Habit **B** buds **C** flowers at full anthesis **D** fully mature matte black fruits with appressed calyx lobes (**A–D** Nijmegen accession A14750051). Photos by S. Knapp and G. van der Weerden (previously published in “PhytoKeys 106”).

####### Distribution.

**Figure 8.**
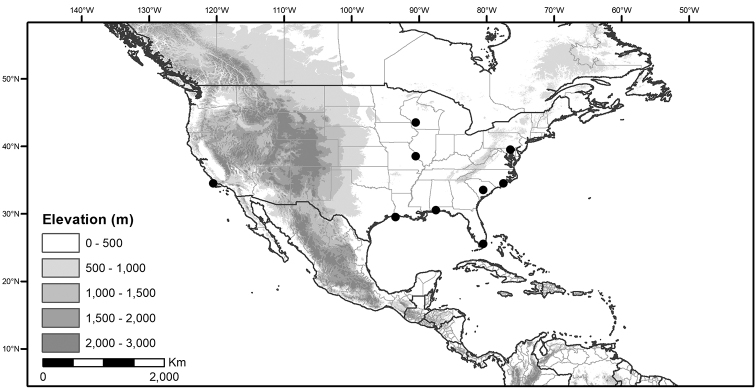
Distribution of *Solanumchenopodioides* Lam.

(Figure 8) *Solanumchenopodioides* is native to southern South America, and has been introduced globally, largely with the wool trade. The species is relatively uncommon in North America, where it is most likely introduced.

####### Ecology.

*Solanumchenopodioides* is an adventive species in North America and occurs only in sporadic populations close to urban areas and human disturbance between 0 and 2,000 m elevation.

####### Common names.

None recorded.

####### Uses.

None recorded.

####### Preliminary conservation status (IUCN 2017).

LC (Least Concern). *Solanumchenopodioides* is a widespread weed of disturbed areas (see Barboza et al. 2013; Särkinen et al. 2018). For EOO see Table 6.

####### Discussion.

*Solanumchenopodioides* is a weedy, ruderal species occurring mainly in coastal parts of North America. The species has distinct grey-green appearance due to the pubescence of appressed, eglandular white trichomes. It is morphologically similar to *S.pseudogracile* and some populations of *S.americanum* around the coast of the Gulf of Mexico. *Solanumchenopodioides* can be distinguished from *S.pseudogracile* only with difficulty, but the short-triangular calyx lobes with acute apices that remain appressed to the berry at fruit maturity, as opposed to the longer, rectangular calyx lobes with rounded to acute apices that are reflexed in fruit of *S.pseudogracile*, are characters that distinguish the taxa. In flower, the extension of style beyond the anther cone is a good character to separate *S.chenopodioides* from *S.pseudogracile*; the style remains almost completely inside the anther cone in *S.chenopodioides* (exserted to 1–1.5 mm) and is clearly exserted in *S.pseudogracile* (exserted to (1)2.0–2.5 mm). Many specimens annotated as *S.chenopodioides* from around the Gulf of Mexico (e.g., Florida) are actually plants of *S.pseudogracile*.

*Solanumchenopodioides* can be distinguished from *S.nigrescens* by the lack of stone cells in fruit, while *S.nigrescens* has always 4–13 stone cells per fruit. Anthers in *S.chenopodioides* are always much longer (2.0–2.8 mm) than in *S.americanum* (0.8–1.5 mm). The strongly deflexed peduncle and pedicels in fruit are distinctive in *S.chenopodioides* but are not always obvious in herbarium specimens.

Typification details for the synonyms of *S.chenopodioides* can be found in Särkinen et al. (2018).

####### Specimens examined.

See Suppl. materials 1 and 3.

###### 3. Solanum corymbosum

Taxon classificationPlantaeSolanalesSolanaceae

Jacq., Collectanea [Jacquin] 1: 78. 1787

Figures 9 , 10

Solanum corymbiferum J.F.Gmel., Syst. Nat., ed. 13[bis] 2(1): 384. 1791, nom. superfl. illeg. Type. Based on Solanumcorymbosum Jacq. (cited in synonymy)
Solanum parviflorum Nocca, Ann. Bot. (Usteri) 6: 61.1793, nom. superfl. illeg. Type. Based on Solanumcorymbosum Jacq. (cited in synonymy)
Solanum parviflorum Salisb., Prodr. Stirp. Chap. Allerton 134. 1796, nom. superfl. illeg. Type. Based on Solanumcorymbosum Jacq. (cited in synonymy)
Solanum cymosum Ruiz & Pav., Fl. Peruv. [Ruiz & Pavon] 2: 31, t. 160. 1799. Type. Peru. “Habitat in Peruviae cultis, versuris et subhumidis locis per Limae et Chancay Provincias”, *H. Ruiz & J.A. Pavón s.n.* (lectotype, designated by Knapp 2008b, pg. 312: MA [MA-747100]).
Solanum corymbosum Jacq. var. cymosum (Ruiz & Pav.) Pers., Syn. Pl. (Persoon) 1: 223. 1805. Type. Based on Solanumcymosum Ruiz & Pav.
Solanum leptanthum Dunal var. parvifolium Dunal, Solan. Syn. 9. 1816. Type. Peru. Cajamarca: sin. loc., *F.W.H.A. von Humboldt & A. Bonpland s.n.* (lectotype, designated here: P [P00670610]; isolectotypes: P [P00136337, P00136338]).
Solanum azureum Van Geert, Cat. Gén. 1879–1880 [Van Geert], Solanumazureum. 1879. Type. Cultivated in the nursery of Auguste Van Geert in Gand, Belgium, from seeds sent by Mr. Roezl from Peru (no specimens cited; no original material found).

####### Type.

Cultivated in Vienna [“Hort. Bot. Vindob.”] seeds said to be from Peru, *N. von Jacquin s.n.* (lectotype, designated by D’Arcy 1970, pg. 559: W [acc. # 0022473]).

####### Description.

Annual to short lived perennial herbs to 30–50 cm tall, subwoody and branching at base. Stems terete, green to straw colour, sprawling, somewhat weak and decumbent, not markedly hollow; new growth nearly glabrous to sparsely pubescent with weak simple, uniseriate appressed 1–8-celled eglandular trichomes, these ca. 0.3 mm long; older stems glabrescent. Sympodial units difoliate or occasionally trifoliate, the leaves not geminate. Leaves simple, 4.5–8 cm long, 1.5–4 cm wide, ovate-lanceolate, chartaceous to subcoriaceous; both surfaces glabrous or sometimes sparsely ciliate near the base of the winged petiole; major veins 7–9 pairs, not clearly evident abaxially in live plants, paler in herbarium specimens; base long-attenuate, decurrent on the petiole; margins entire (in Peru rarely slightly 3-lobed, *Croat 58409*); apex acute; petioles 0.5–1 cm, glabrous to sparsely puberulent, winged to the base. Inflorescences 2–3 cm long, lateral, internodal or opposite the leaves, 4–7 times branched, with 20–50(–60) flowers spaced along the rhachis, nearly glabrous to sparsely pubescent; peduncle 0.1–2 cm, straight in fruit; pedicels 2–2.5 mm long, less than 0.5 mm in diameter at the base, ca. 0.5 mm in diameter at the apex, spreading, articulated at the base; pedicel scars spaced 1–3 mm apart. Buds globose, the corolla about halfway exserted from the calyx tube before anthesis, the tips of the corolla lobes often much more pubescent than the calyx. Flowers 5-merous, all perfect. Calyx tube 0.5–1 mm long, conical or broadly conical, the lobes 0.5–0.6 mm long, ca. 0.5 mm wide, broadly triangular, glabrous to very sparsely puberulent with simple, uniseriate trichomes. Corolla 5–10 mm in diameter, white or purple, the abaxial surface usually purple, rotate-stellate, the lobes 1–2.5 mm long, 1–1.5 mm wide, broadly triangular, reflexed at anthesis, later spreading, glabrous adaxially, minutely white-puberulent abaxially on the tips. Stamens equal; filament tube minute; free portion of the filaments ca. 0.2 mm long, adaxially pubescent with simple tangled white trichomes; anthers 0.8–1.5(-1.8) mm long, ca. 0.5 mm wide, ellipsoid, yellow, somewhat connivent, poricidal at the tips, the pores lengthening to slits with age. Ovary globose, glabrous; style ca. 2 mm long, hardly exserted from the anther cone, pubescent in the lower 2/3 with tangled, white uniseriate simple weak-walled trichomes; stigma globose-capitate, minutely papillate, pale green in live plants. Fruit a globose berry, 4–6 mm in diameter, orange to red when ripe, opaque, the surface of the pericarp shiny or matte; fruiting pedicels 2–3 mm long, ca. 0.5 mm in diameter at base, strongly recurved at the very base, dropping with mature fruits, not persistent; fruiting calyx scarcely accrescent, the tube ca. 1 mm long, the lobes 1–1.3 mm long, appressed to the berry. Seeds 20–30 per berry, 1.5–1.8 mm long, 1.2–1.4 mm wide, flattened reniform with a central hilum, light yellow-tan or reddish brown in herbarium material, the surfaces minutely pitted, the testal cells with sinuate margins. Stone cells 2, ca. 1.5 mm in diameter, globose, prominent near the apex of the berry. Chromosome number not known.

**Figure 9.**
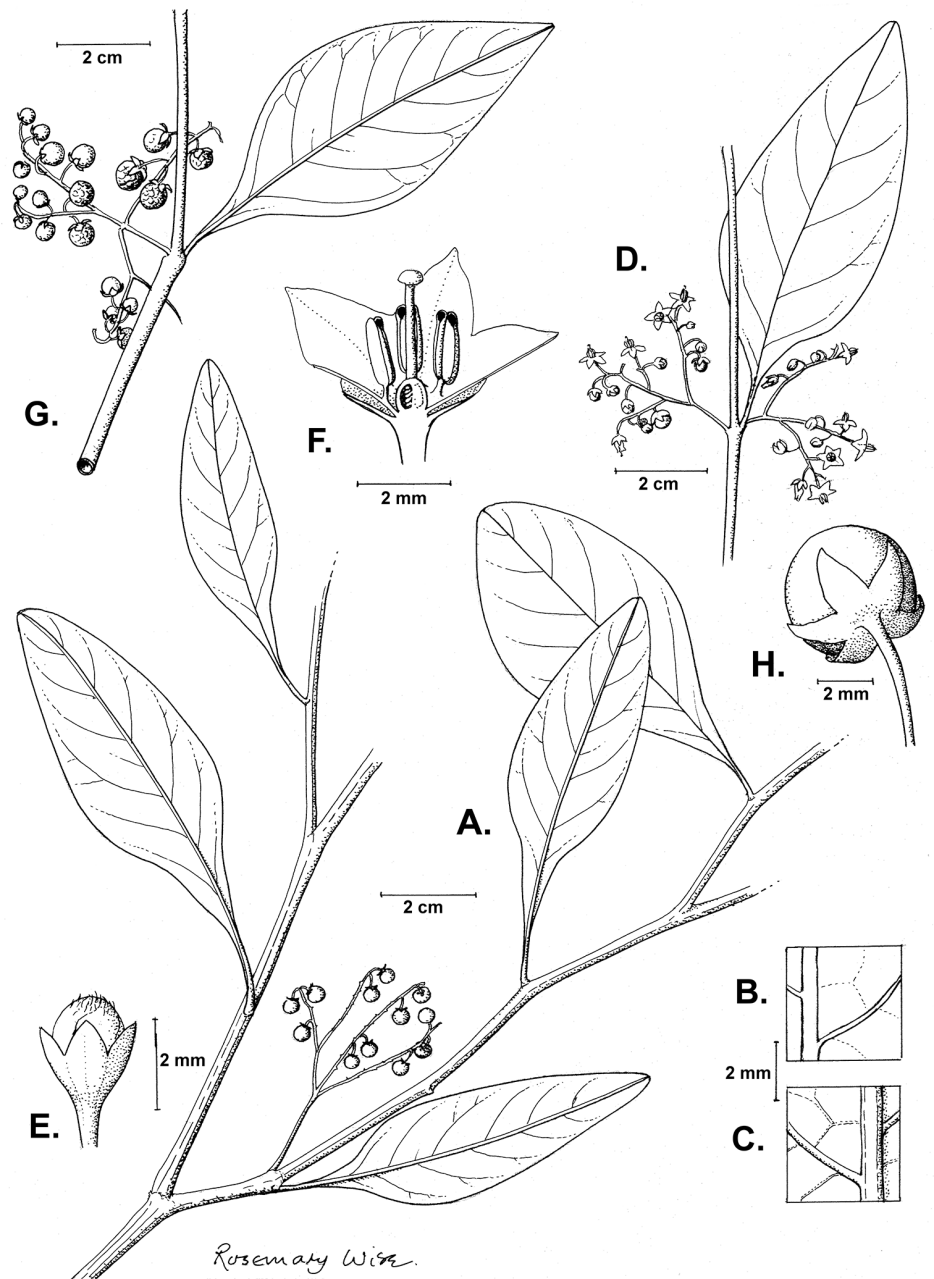
*Solanumcorymbosum* Jacq. **A** Habit **B** detail of adaxial leaf surface **C** detail of abaxial leaf surface **D** flowering branch **E** floral bud **F** dissected flower **G** fruiting branch **H** maturing fruit (**A–F***van der Werff 14657***G–H***Ochoa 14625*). Drawing by R. Wise.

**Figure 10.**
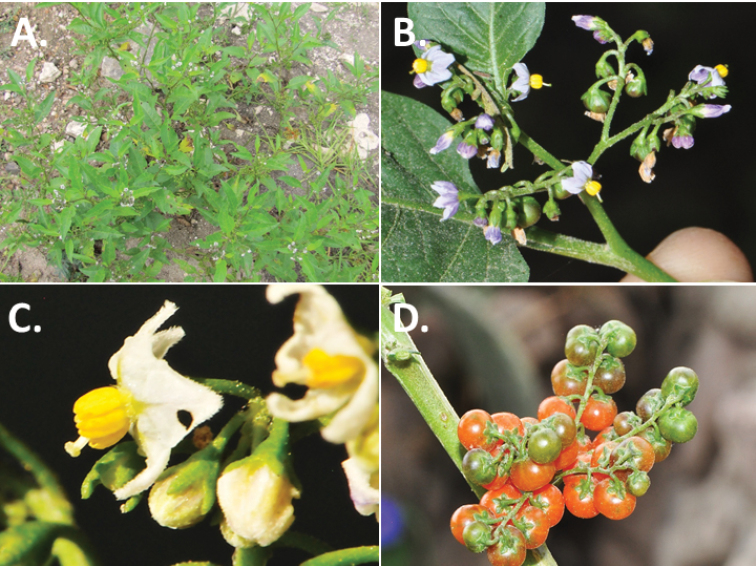
*Solanumcorymbosum* Jacq. **A** Habit **B** inflorescence **C** flowers at full anthesis and buds **D** fully mature red-orange fruits with appressed calyx lobes (**A***Särkinen et al. 4604B***B, D***Särkinen et al. 4078*; **D***Särkinen et al. 4509*). Photos by T. Särkinen.

####### Distribution.

**Figure 11.**
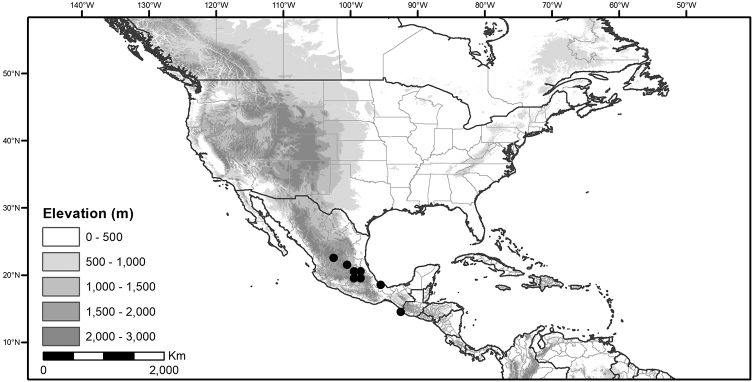
Distribution of *Solanumcorymbosum* Jacq.

(Figure 11) *Solanumcorymbosum* is native to the western slopes of the Andes in Peru, and naturalised in central and southern Mexico, possibly through introduction in colonial times.

####### Ecology.

*Solanumcorymbosum* grows in open, disturbed areas in landslides and along roads from 150 to 2,600 m elevation in Mexico (in its presumed native range in Peru from sea level [in coastal lomas vegetation] to 2,900 m elevation).

####### Common names.

None recorded for the region.

####### Uses.

None recorded.

####### Preliminary conservation status (IUCN 2017).

LC (Least Concern). *Solanumcorymbosum* has a disjunct distribution in Peru and Mexico; in its native range in Peru the species is quite widely distributed, but the AOO for the Mexican plants (76 km^2^, classing it as EN) combined with potential morphological differences from Peruvian populations (see below) suggests it is of some conservation concern. For EOO see Table 6.

####### Discussion.

*Solanumcorymbosum* is a member of the Radicans group and is related to species of southern South America (see Särkinen et al. 2015b). The distribution of this species in Mexico is highly disjunct from what are presumed native populations in Peru and Mexican populations are thought to represent an introduction of this species in post-Columbian times. It is tempting to speculate on an inadvertent introduction between mining areas, perhaps even in Spanish colonial times. Populations in Mexico show nearly identical haplotypes to those from the coastal regions in Peru (Mitchell 2014), supporting this hypothesis.

Mexican populations of *S.corymbosum* differ from Andean populations in having larger leaves (20 cm^2^ Mexico, ca. 9 cm^2^ Andes) and larger and fewer berries; an average of ca. 30 berries of 5.5 cm diameter per inflorescence in Mexican specimens versus an average of ca. 50 berries of 3.5 mm in diameter per inflorescence in Andean specimens (Mitchell 2014). This may be due to founder effects in the establishment of the Mexican populations, and Mitchell (2014) has speculated that these populations may be polyploid.

*Solanumcorymbosum* can be distinguished from other morelloids occurring in Mexico in its orange to red fruits with two large apical stone cells, its highly branched inflorescences and diminutive flowers with rotate-stellate corollas that are usually white adaxially and purple abaxially. The leaves are thicker than other morelloids from the area, and the petioles are strongly winged.

Three collections of *Solanumcorymbosum* in BM [all mounted on a single sheet] 1. “Hort. Paris. L’Heritier 1783 (E Peru Dombey)”, 2. “Hort. Kew. 1785”, 3. “Peru, Dombey 63,” P-Lam [Morton neg. 8364] are possible isotype material of various of the synonyms. Collections attributed to Dombey from Paris are probably isolectotype material of *S.cymosum* (see Knapp 2008b), while those from Kew and the Lamarck herbarium are not type material. It is possible that much of the botanical garden material being described in the late 18^th^ century came from a few collections and is all genetically the same.

*Solanumleptanthum* is a synonym of *S.pubigerum* Dunal (a member of the Dulcamaroid clade, Knapp 2013), but variety *parviflorum* corresponds to *S.corymbosum*. We have selected the best preserved of the three sheets in the Humboldt and Bonpland herbarium at P (P00670610) as the lectotype for var. parvifolium.

####### Specimens examined.

See Suppl. materials 1 and 3.

###### 4. Solanum douglasii

Taxon classificationPlantaeSolanalesSolanaceae

Dunal, Prodr. [A. P. de Candolle] 13(1): 48. 1852

Figure 12 , 13

Solanum umbelliferum Eschsch. var. trachycladum Torr., Pacific Railr. Rep. Parke, Bot. 7(3) [preprint]: 17. 1856. Type. United States of America. California: Ventura County, San Buenaventura Ranch, 16 Feb 1855, *T. Antisell s.n.* (lectotype, designated here: NY [NY00821411]).
Solanum arizonicum Parish, Proc. Calif. Acad. Sci., ser. 3, 2: 165. 1901. Type. United States of America. Arizona: Copper Basin, *J.W. Toumey 397* (holotype: US [acc. # 211749, US00027460; isotype: UC n.v.).
Solanum extusviolascens Bitter, Repert. Spec. Nov. Regni Veg. 11: 7. 1912. Type. Mexico. Sin. loc., *J.G. Schaffner 654* (holotype: B, destroyed; no duplicates found).
Solanum profundeincisum Bitter, Repert. Spec. Nov. Regni Veg. 12: 80. 1913. Type. Mexico. Baja California: Guadalupe Island, cañon near beach, 1875, *E. Palmer 61* (lectotype, designated here: UPS [UPS-V-851402]; isolectotypes: BM [BM001007201], MO [MO-568699, acc. # 5510874], NY [NY00139024, NY00828776], YU [YU065318]).

####### Type.

United States of America. California: “Nova California”, *D. Douglas s.n.* (holotype: G-DC [G00144189]; isotypes: BM [BM000838093], K [K001159712]).

####### Description.

Perennial, subwoody herbs or shrubs, erect to ascending, up to 2 m tall. Stems terete, green or purple-tinged, moderately to densely pubescent with simple, uniseriate 4–10-celled spreading eglandular trichomes, 0.5–1 mm long; new growth more densely pubescent. Sympodial units difoliate, not geminate. Leaves simple, 3–10(–17) cm long, 1.3–5(–7.5) cm wide, (broadly) ovate to lanceolate, green or marked with purple, green above, paler greyish-green below; adaxial surface moderately to densely pubescent with simple, uniseriate trichomes like those on stem, these evenly spread along the lamina and veins; abaxial surface more densely pubescent than the abaxial surface; primary veins 4–6 pairs, clearly evident abaxially; base abruptly contracted to attenuate, at times asymmetric, decurrent on the petiole; margins sinuate-dentate to toothed, rarely entire; apex acute; petiole 1–4(–7) cm long, moderately to densely pubescent with simple, uniseriate like those on stem. Inflorescences 1.5–4.5 cm long, lateral, internodal, unbranched to occasionally forked, with (3–)6–14 flowers spaced along the rhachis, moderately to densely pubescent with simple, uniseriate trichomes like those on stems; peduncle 1.5–4 cm long; pedicels 10–41 mm long, 0.3–0.4 mm in diameter at the base and 0.4–0.6 mm in diameter at the apex, straight and spreading, articulated at the base, spaced ca. 0.5–1 mm apart. Buds ovoid and narrower at the tips, the corolla exserted 1/5 of its length beyond the calyx tube. Flowers 5-merous, all perfect. Calyx tube 1–2 mm long, the lobes (1-)1.5–2.9 mm long, 0.7–1.5 mm wide, lanceolate to broadly triangular with obtuse to acute apices, moderately to densely pubescent with simple, uniseriate trichomes like those on stem. Corolla 13–15(–20) mm in diameter, stellate, white to lilac with a yellow-green central eye with black coloration at the base, lobed 1/3 to the base, the lobes 4.5–7 mm long, 2–4 mm wide, strongly reflexed at anthesis, sparsely pubescent abaxially with 1–4-celled simple uniseriate trichomes like those on stems and leaves but shorter. Stamens equal; filament tube 0.3–1 mm long; free portion of the filaments 0.1–0.5(1) mm long, sparsely pubescent with spreading uniseriate 4–6-celled simple trichomes adaxially; anthers (2.5-)3–4.5 mm long, 0.9–1.2 mm wide, ellipsoid and slightly tapered towards the tips, yellow, poricidal at the tips, the pores lengthening to slits with age. Ovary globose, glabrous; style 6.5–7.5 mm long, exserted 1.7–2.3 mm beyond the anther cone, densely pubescent with 2–3-celled simple uniseriate trichomes to 1/2–2/3 from the base; stigma capitate, minutely papillate, green in live plants. Fruit a globose berry, 6–14 mm in diameter, black at maturity, opaque, the surface of the pericarp matte; fruiting pedicels 8–11 mm long, 0.4–0.5 mm in diameter at the base, 0.5–0.6 mm in diameter at the apex, spaced 1–3 mm apart, spreading to reflexed, dropping with mature fruits, very occasionally remaining on the inflorescence rhachis; fruiting calyx not accrescent, the tube less than 1 mm long, the lobes 1.2–3 mm long, appressed against the berry. Seeds usually >50 per berry, 1.5–1.9 mm long, 1.2–1.5 mm wide, flattened and tear-drop shaped with a subapical hilum, brown, the surfaces minutely pitted, the testal cells pentagonal in outline. Stone cells (2-)6–8 per berry, rather large, 0.5–0.7 mm in diameter. Chromosome number: 2*n*=2×=24 (Henderson 1974; Heiser et al. 1965 as *S.amethystinum*; Edmonds 1982, 1983; Stebbins and Paddock 1949; Heiser 1955 (as *S.amethystinum*); Soria and Heiser 1961 (as *S.amethystinum* and *S.douglasii*).

**Figure 12.**
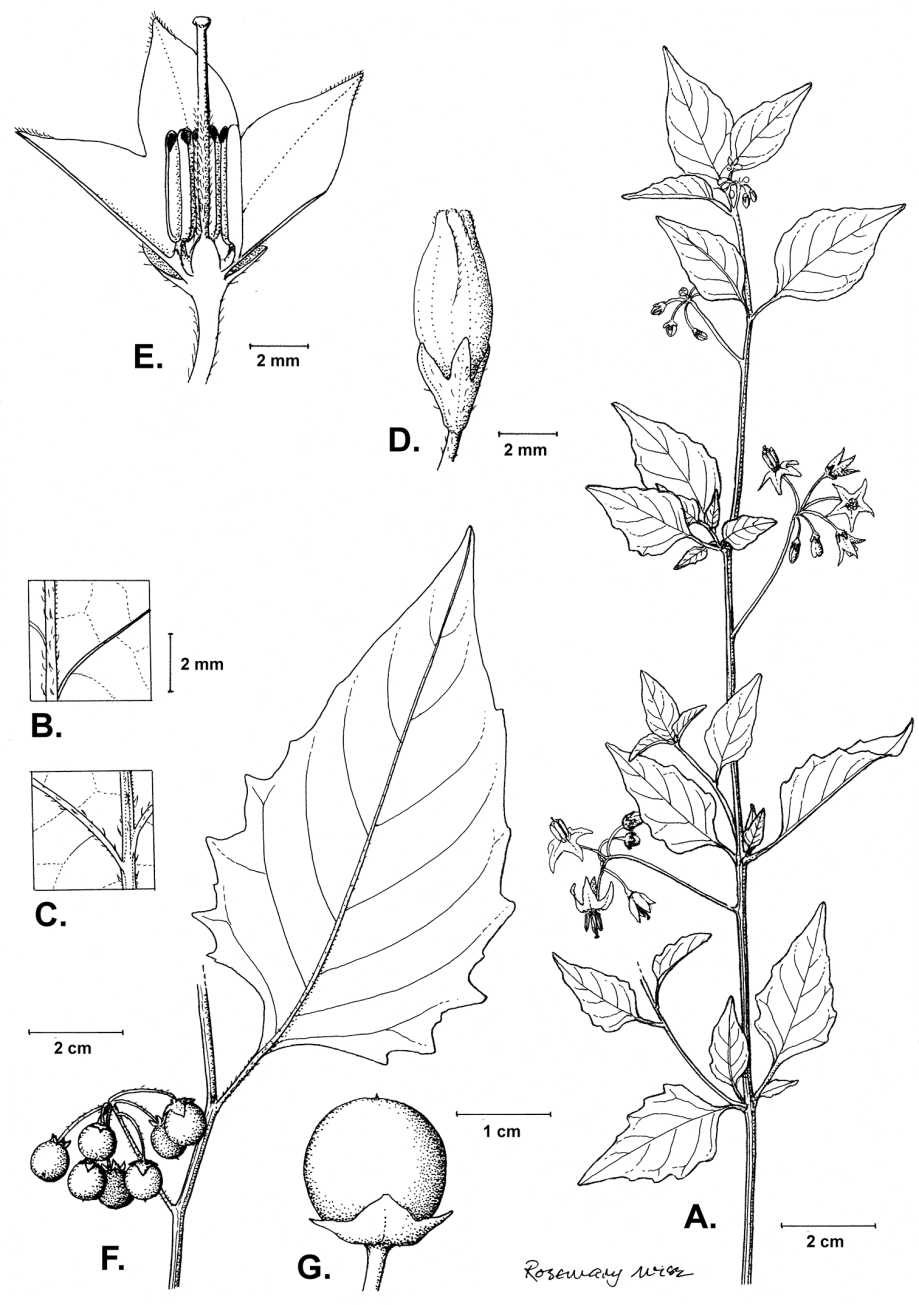
*Solanumdouglasii* Dunal **A** Habit **B** detail of adaxial leaf surface **C** detail of abaxial leaf surface **D** floral bud **E** dissected flower **F** fruiting branch **G** maturing fruit (**A–G***Carter et al. 2149*). Drawing by R. Wise.

**Figure 13.**
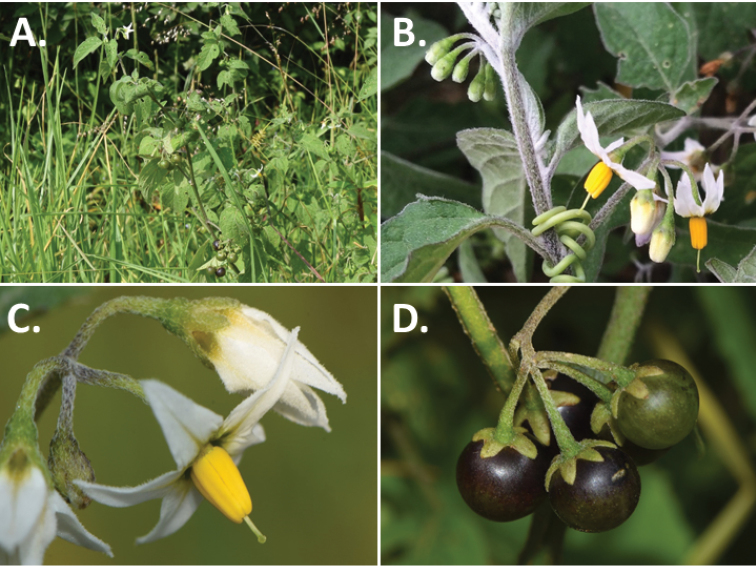
*Solanumdouglasii* Dunal **A** Habit **B** young and maturing inflorescences with buds and flowers **C** flowers at full anthesis **D** fully mature matte black fruits with appressed calyx lobes (**A–D***Ochoterena et al. 979*). Photos by S. Knapp.

####### Distribution.

**Figure 14.**
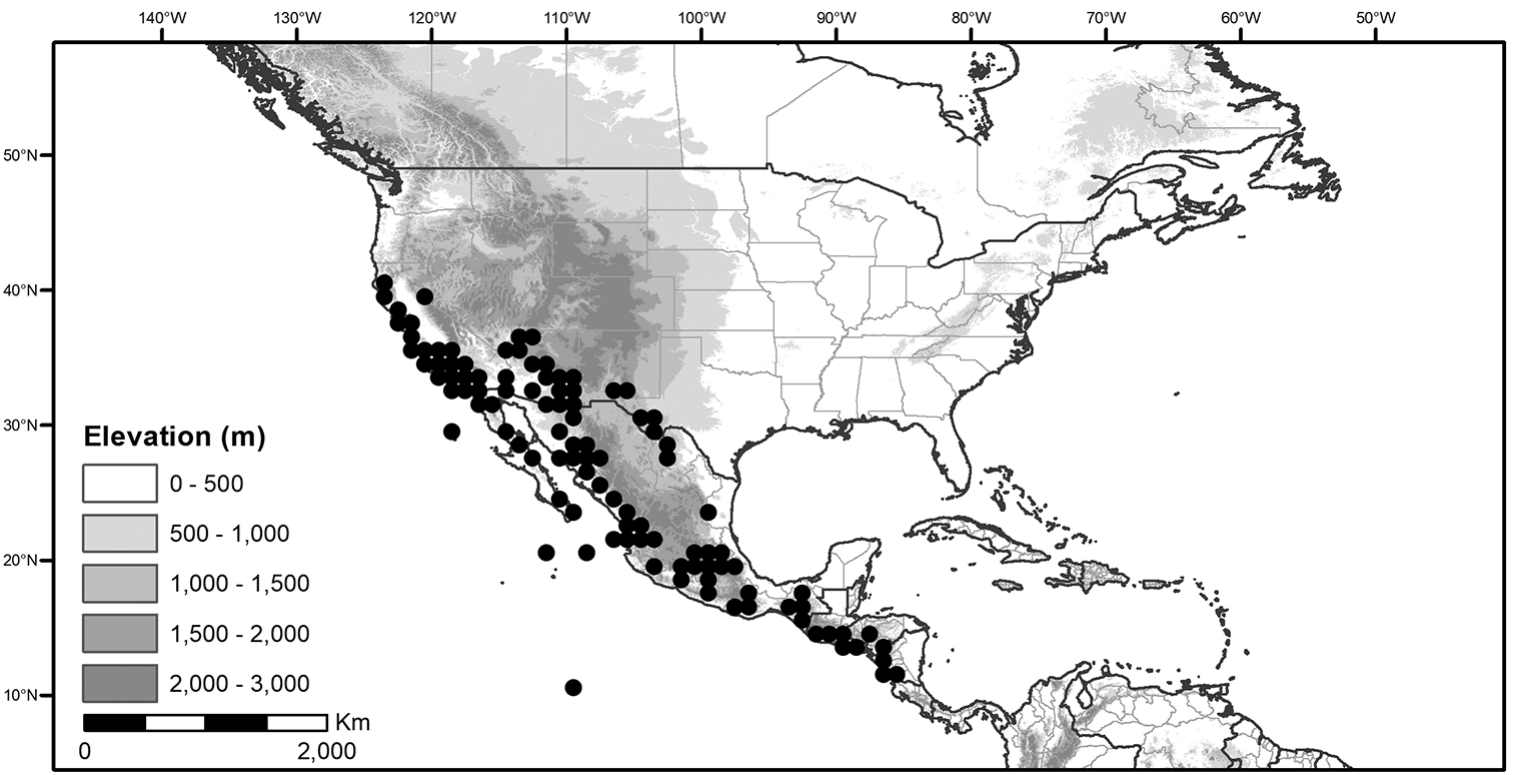
Distribution of *Solanumdouglasii* Dunal

(Figure 14) *Solanumdouglasii* occurs in North America from California east to Arizona and south to Nicaragua; it is the most common black nightshade in the southwestern United States of America and northern Mexico.

####### Ecology.

Open areas and disturbed habitats in a wide variety of vegetation types, from xerophytic to mesophytic forests and oak-pine woodlands between (sea level-) 600 and 3,400 m elevation.

####### Common names.

United States of America. Arizona nightshade (Martin and Hutchins 1980), Douglas’ horse-nettle (NatureServe 2017), Douglas’s nightshade (Peck 1941; Martin and Hutchins 1980; Nee 2012), Greenspot nightshade (USDA Plants 2017). Mexico. Hierba (yerba) mora (many sources, [Chihuahua] Chichequelite (*Pennington 42*), [Chiapas] Moen (Tzeltal, *Shilom Ton 9185*), Mora wamul (Tzeltal, *Gómez López 426*), [Guerrero] Moradito (*Kruse 1656*), [Hidalgo] Tomaquilit (*Villa Kumel 53*), [México] Tomatillo (*la Cruz Bolaños Adec-12*), [Oaxaca] Pchfux-yaas (Zapotec, *Hunn OAX-1547*), Skelemal ch’aben (Tzeltal, *López Pérez 326*), [Puebla] Teconchichi (*Tlapa & Ubierna 105*), [Sonora] Chichicalite (*Guizar N. et al. 4260*).

####### Uses.

United States of America. [California] Leaves used as a potherb (Luiseño people of Orange County, Sparkman 1908); juice of berries used as wash for inflamed eyes and in tattooing or for dye (Luiseño people, Sparkman 1908; Cahuilla people of the Sonoran Desert, Bean and Saubel 1972). Mexico. Leaves used as a potherb (“quelite”). See also section on Uses.

####### Preliminary conservation status (IUCN 2017).

Least Concern (LC). *Solanumdouglasii* is widespread and weedy in the southwestern United States of America and throughout Mexico. For EOO see Table 6.

####### Discussion.

*Solanumdouglasii* is most common west of the Rocky Mountains, along the western coast and southwesternmost United States of America along the Mexican border. *Solanumdouglasii* can be distinguished from the morphologically similar and sympatric *S.nigrescens* by its longer, slightly tapering anthers (greater than 3 mm long and in North America usually 4–4.5 mm long) and the minute free portion of the filaments. Both species are morphologically highly variable and sympatric through much of Mexico and Central America, often growing in the same areas; detailed studies are needed to establish whether interbreeding occurs between particular areas/populations in areas of sympatry. The two species have been put in synonymy by other authors (e.g., Edmonds 1972; D’Arcy 1974a, b), but characterised as “ill-defined” by others (e.g. Nee 1999).

The description of S.umbelliferumvar.trachycladum cites “Santa Inez and San Buenaventura Ranch” and “Flowers apparently white, about as large as in *S.nigrum*” (Torrey 1856) with no collector or date. The plants collected in the several expeditions ordered by the United States Government to plan a railway leading across the Rocky Mountains to the Pacific were variously described by Asa Gray (Harvard) and John Torrey (New York). Thomas Antisell collected between the Rio Grande River and southern California; his collections are described in Volume VII of the Reports (Brendel 1880) by Torrey. We have only found a single specimen collected by Antisell and annotated by Torrey with this name; it has the locality “San Buenaventura Ranch/Feb 16/Dr Antisell”. We select this sheet (NY00821411) as the lectotype following McNeill (2014).

In describing *S.profundeincisum*Bitter (1913) cited two collections of Edward Palmer’s from Guadelupe Island, *Palmer 60* pro parte and *Palmer 61*, both from UPS. *Palmer 60* is a mixed collection, some parts of which were used to describe *S.calvum* (a synonym of *S.americanum*) and some as part of the protologue of *S.profundeincisum*. The collection *Palmer 61* is represented by many duplicates and is not mixed; we select the UPS sheet of *Palmer 61* (UPS-V-851402) cited by Bitter (1913) as the lectotype of *S.profundeincisum*.

####### Specimens examined.

See Suppl. materials 1 and 3.

###### 5. Solanum emulans

Taxon classificationPlantaeSolanalesSolanaceae

Raf., Autik. Bot. 107. 1840

Figure 15 , 16

Solanum nigrum L. var. virginicum L., Sp. Pl. 186. 1753. Type. “Solanumnigrum vulgari simile, caulibus exasperates”, cultivated in England, at James Sherard’s garden in Eltham (Hortus Elthamensis), said to be from Virginia (lectotype, designated by Edmonds in Jarvis 2007, pg. 861, Dillenius, Hortus Elthamensis 2: 368, t. 275, f. 356. 1732).
Solanum pterocaulum Dunal var. heterogonum Dunal, Prodr. [A. P. de Candolle] 13(1): 52. 1852. Type. Cultivated in France at Montpellier “Solanumheterogonum. In hortis bot. cultum” (no specimens cited, described from living plants “v.v. hort. Monsp.”; neotype, designated here: MPU [MPU31070707]).
Solanum adventitium Polg., Magyar. Bot. Lapok 24: 18, pl. 1. 1926. Type. Hungary. Györ, Güterbahnhof, 20 Sep 1918, *S. Polgár 2698* (lectotype, designated here: BP [BP-352743]; isolectotypes: B [B100278541], W [acc. # 1935-0007031]).
Solanum dillenianum Polg., Acta Horti Gothob. 13: 281. 1939. Type. Based on Solanumnigrumvar.virginicum L.

####### Type.

United States of America. “Amer. bor.”, *C.S. Rafinesque s.n.* [ex Herb. Rafinesque] (neotype, designated here: W [acc. # 0009388]).

####### Description.

Annual herbs to subwoody perennial shrubs up to 1.0 m tall, branching at base. Stems terete to ridged, green colour, pubescent with simple, appressed, uniseriate eglandular 1–5-celled trichomes, these ca. 0.2 mm long, new growth more densely pubescent. Sympodial units difoliate, not geminate. Leaves simple, 4.5–10.5(-17.5) cm long, 2.0–6.3(-8.3) cm wide, ovate, thin membranous, slightly discolorous, green above and purplish tinged underneath, especially so in younger growth; adaxial surface glabrous to sparsely pubescent with appressed translucent, simple, uniseriate trichomes like those on stem scattered mainly along veins; abaxial surface glabrous to sparsely pubescent with trichomes like those of the upper surface on both lamina and veins; primary veins 4–6 pairs; base attenuate to acute; margins sinuate dentate, rarely entire; apex acute to acuminate; petiole 1.0–5.0 cm long, pubescent with simple uniseriate trichomes like those of the stems. Inflorescences 1.0–2.5 cm long, lateral, internodal, unbranched or occasionally forked, with (2)3–6 flowers clustered near the tips (sub-umbelliform), sparsely pubescent with appressed simple uniseriate trichomes like those on stem; peduncle 1.0–1.7 cm long, straight; pedicels 8–10 mm long, 0.4–0.5 mm in diameter at the base and 0.5–0.6 mm in diameter at the apex, straight and spreading, articulated at the base; pedicel scars spaced ca. 0–0.5 mm apart. Buds subglobose, corolla exserted from the calyx to 1/3 of its length. Flowers 5-merous, all perfect. Calyx tube 0.7–0.9 mm long, the lobes 0.8–2.2 mm long, 0.7–1.3 mm wide, ovate to elongate with obtuse apices, sparsely pubescent with appressed hairs like those on stem but shorter. Corolla 8–10 mm in diameter, stellate, white with a yellow-green central portion near the base, lobed 1/3 to the base, the lobes 3.0–4.0 mm long, 1.0–1.2 mm wide, strongly reflexed at anthesis, later spreading, densely pubescent abaxially along margins and apex with simple uniseriate trichomes like those on stem and leaves but shorter. Stamens equal; filament tube minute, pubescent with spreading uniseriate simple trichomes adaxially; free portion of the filaments 0.6–1.0 mm long, pubescent like the tube; anthers (1–)1.5–1.7 mm long, 0.4–0.5 mm wide, ellipsoid, yellow, poricidal at the tips, the pores lengthening to slits with age. Ovary globose, glabrous; style 3.5–4.5 mm long, not exceeding anthers, densely pubescent with 2–3-celled simple uniseriate trichomes along 1/3 to 1/2 from the base; stigma capitate, minutely papillate, green in live plants. Fruit a globose berry, 6–8 mm in diameter, dull purplish-black at maturity, opaque, the surface of the pericarp matte to slightly shiny; fruiting pedicels 8–10 mm long, 0.4–0.6 mm in diameter at the base, 0.7–1.0 mm in diameter at the apex, recurved to reflexed, pedicels spaced 0.5–2.5 mm apart, dropping with mature fruits; fruiting calyx somewhat accrescent, the tube less than 1 mm long, the lobes 1.0–2.2 mm long, appressed to the surface of the berry or slightly spreading in mature fruit. Seeds 20–50(–60) per berry, 1.6–1.8 mm long, 1.0–1.2 mm wide, flattened and tear-drop shaped with a subapical hilum, brown, the surfaces minutely pitted, the testal cells pentagonal in outline. Stone cells 6–9(10) per berry, ca. 0.3 mm in diameter. Chromosome number: *2n*=2×=24 (Stebbins and Paddock 1949, as *S.americanum*; Mulligan 1961, as *S.americanum*; Soria and Heiser 1961, as *S.americanum*; Heiser et al. 1965, as *S.americanum*; Edmonds 1983, as *S.americanum*; Crompton and Bassett 1976, as *S.americanum*).

**Figure 15.**
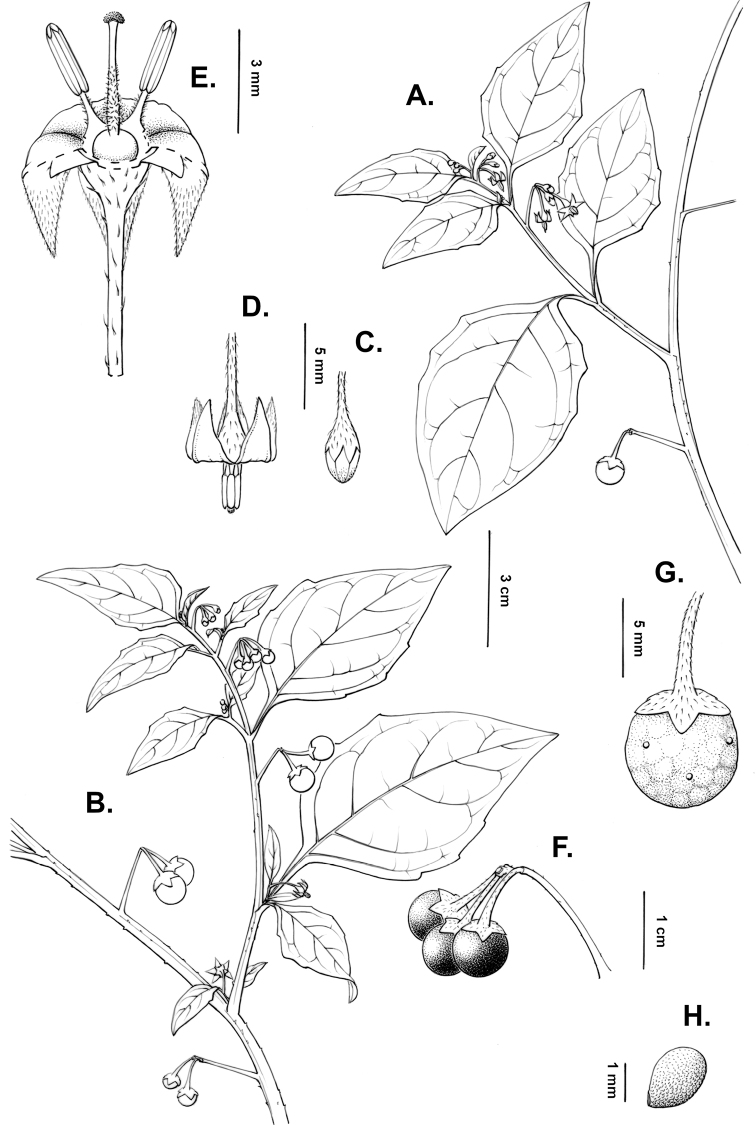
*Solanumemulans* Raf. **A** Habit **B** fruiting habit **C** Bud **D** flower **E** dissected flower **F** mature fruits **G** dried berry **H** seed (**A–H***Ruth 687*). Drawing by C. Banks.

**Figure 16.**
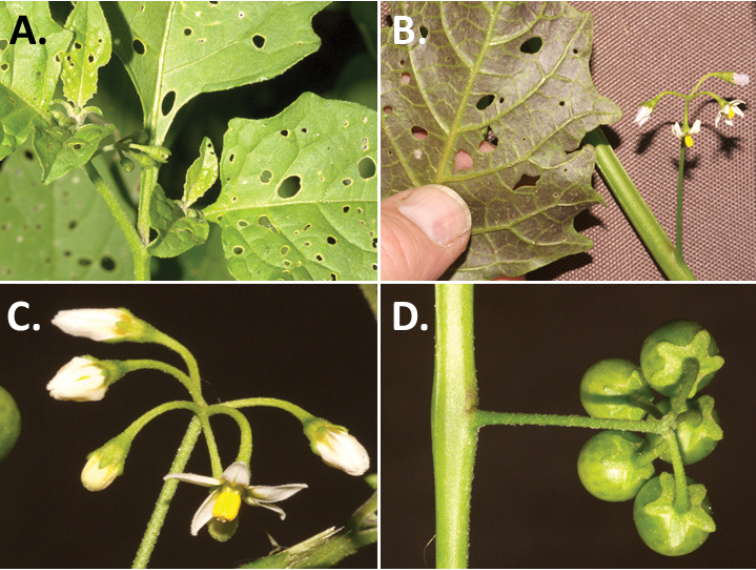
*Solanumemulans* Raf. **A** Leaves and buds **B** leaf abaxial surface and inflorescence **C** buds and flower at full anthesis **D** developing fruits with appressed calyx lobes (**A, C, D***Nee 61357*; **B***Nee 61306*). Photos by M. Nee.

####### Distribution.

**Figure 17.**
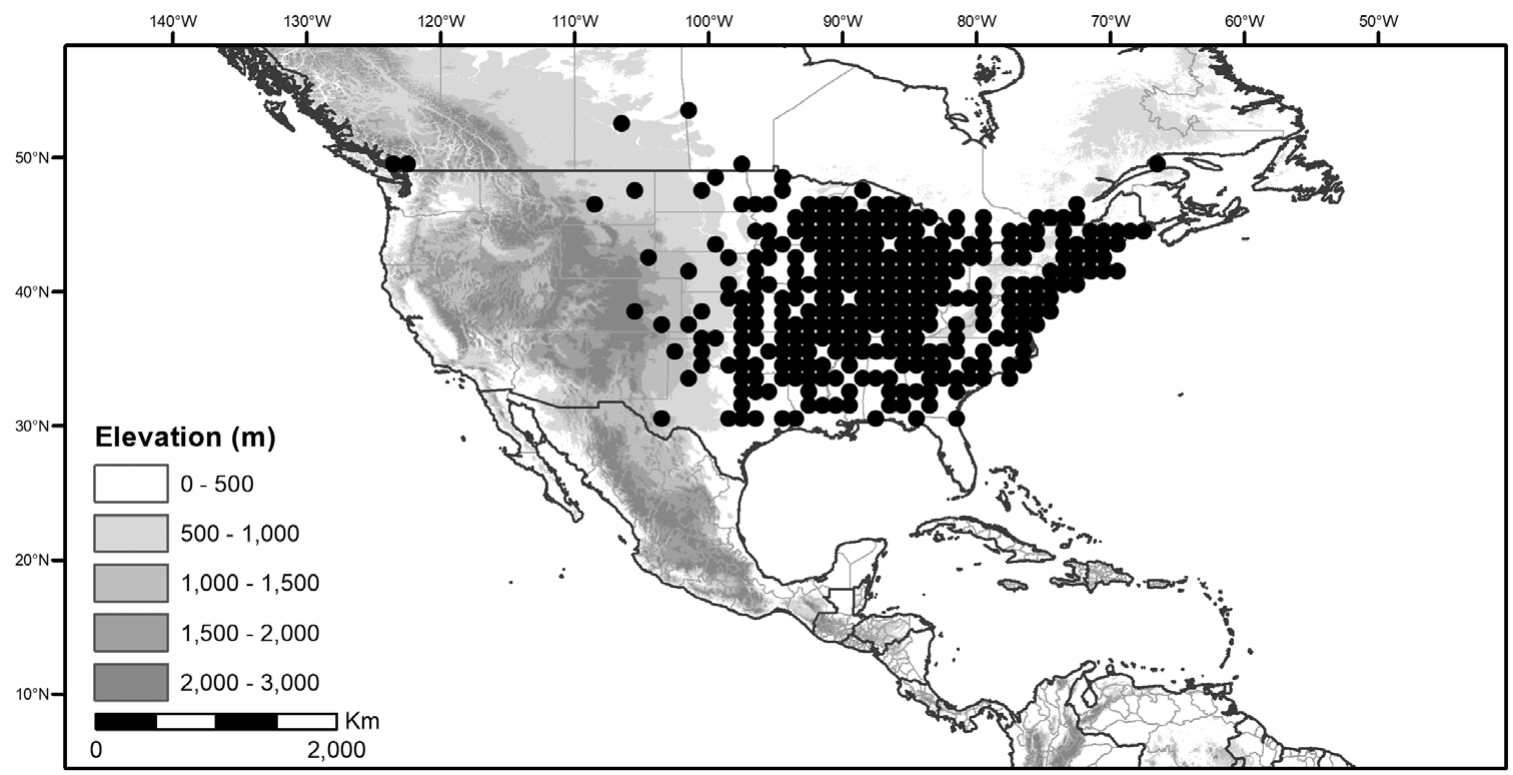
Distribution of *Solanumemulans* Raf.

(Figure 17) *Solanumemulans* is endemic to North America and is the most common species of black nightshade in eastern North America east of the Rocky Mountains from Maine to North Carolina and into Canada. Plants collected near Vancouver (British Columbia) may have been introduced along the railways.

####### Ecology.

Common in disturbed habitats such as riverbanks, gardens, rocky outcrops between sea level and 1,120 m elevation.

####### Common names.

Canada. Eastern black nightshade, morelle noire de l’est (Bassett and Munro 1985, as *S.ptychanthum*); crêve-chien; tue-chien (Québec, *Marie-Victorin et al. 3942*). United States of America. American black nightshade (USDA Plants 2017, as *S.ptychanthum*), Eastern black nightshade (Ogg et al. 1981; Uva et al. 1997, both as *S.ptychanthum*). The common name of “West Indian nightshade” recorded in USDA Plants (2017) for this plant certainly refers to *S.americanum*.

####### Uses.

Strausbaugh and Core (1979, as *S.americanum*) record the use of “ripe berries cooked and eaten in pies” in West Virginia. See also introductory section on Uses.

####### Preliminary conservation status (IUCN 2017).

Least Concern (LC). *Solanumemulans* is common and weedy in the eastern United States and Canada. For EOO see Table 6.

####### Discussion.

*Solanumemulans* can be distinguished from other morelloids in North America by the small anthers 1.0–1.5 mm long, relatively long filaments 0.6–1.0 mm compared to *S.americanum*, calyx lobes longer than *S.americanum* and these appressed in fruit rather than strongly reflexed like in *S.americanum*, pedicel thickened at the apex in fruit (unlike in *S.americanum*), and pedicels that drop off with mature fruits (pedicels remain on the inflorescence in *S.americanum*). *Solanumemulans* has always 4–9(10) stone cells in fruits, while *S.americanum* either lacks or has maximum of 4 stone cells.

*Solanumemulans* can be distinguished from *S.interius* and *S.nigrescens* by its shorter anthers, usually shorter calyx lobes, and usually unbranched inflorescences. When sympatric with the occasionally introduced *S.nigrum*, *S.emulans* can be easily distinguished based on anther length and the numerous stone cells in the berries, but *S.emulans* also generally has thinner leaves that are often purplish tinged beneath. In the Great Plains, the morphologically similar *S.interius* becomes more common than *S.emulans*, while along the southern East and Gulf coasts in the United States of America *S.americanum* becomes more common. *Solanumemulans* is not known from the Caribbean.

Although *S.emulans* appears to have been in cultivation in European botanical gardens since the 18^th^ century, it has not escaped and naturalised beyond where it has initially been introduced. The few European specimens are from areas near oil and clothing factories and have apparently not persisted (Polgár 1926).

Constantine Rafinesque cited no specific specimens in his many descriptions of new taxa, and any herbarium he kept in North America was widely dispersed after his death and is thought to have been destroyed (Pennell 1944; Warren 2004). A specimen in the Vienna herbarium (W acc. #0009388) corresponding to the description of *S.emulans* and labelled “Solanum Virginicum/Amer. Bor. Rafinesque” and the date 1828 may be original material for this name. We have here selected this as a neotype, since there is no evidence in the protologue that this (or any other) specimen was used by Rafinesque to describe *S.emulans*.

The name *S.ptychanthum* has been used for this species in North America (e.g., Schilling 1981; Voss et al. 1993; Jones 2005), but the type of that name corresponds to a plant of *S.americanum* (see *S.americanum* description). *Solanumemulans* was long ignored, but in the protologue Rafinesque clearly refers to a taxon from “New England and Kentucky” that people were calling “S.virginicum” – probably the Linnaean S.nigrumvar.virginicum, not *S.virginicum* L. (an illegitimate name and orthographic variant of the spiny solanum from India *S.virginianum* L., see Jarvis 2007) – and his description matches this widespread small-flowered morelloid from eastern North America. The protologue states “NE states, usually mistaken for S. Virg.[virginianum] but smooth smaller, fl. white small, berries pisiform”. Specimens corresponding to this taxon are in the Dillenian herbarium in OXF under the polynomial (*Solanumnigrum vulgari simile, caulibus exasperatis* Dill. elth. 368, t. 275, f. 256) and correspond to the plate that was the only element cited for Solanumnigrumvar.virginicum (Linnaeus 1753).

D’Arcy (1974a) cited “Hungary, *Polgar s.n.* (MPU)” as the type of *Solanumadventitium*, but without specifying a locality or number. We do not consider this specific enough to constitute the citation of a single unambiguous specimen and it is likely to be in conflict with the protologue; we therefore lectotypify *S.adventitium* here. In the protologue of *S.adventitium*Polgár (1926) cited several of his own collections made at “Meller’schen Ölfabrik” and “Güterbahnhof” (both in Györ, Hungary) between 1915 and 1919, but cited neither numbers nor herbaria. He noted that the plants had disappeared from both localities by October 1919, but again cited no herbarium. His herbarium is kept at BP, and we have selected one of his many collections labelled as *S.adventitium* in that herbarium collected between 1916 and 1919 from the freight depot in Györ as the lectotype (BP-352743).

####### Specimens examined.

See Suppl. materials 1 and 3.

###### 6. Solanum furcatum

Taxon classificationPlantaeSolanalesSolanaceae

Dunal, Encycl. [J. Lamarck & al.] Suppl. 3: 750. 1814

Figures 18 , 19

Solanum deltoideum Colla, Herb. Pedem. 4: 273. 1835. Type. Cultivated in Italy at “h. Ripul:” [Hortus Ripulensis], the seeds originally sent by C. Bertero from Chile [“Chili Quillota”] (no specimens cited; lectotype, designated by Särkinen et al. 2018, pg. 73: TO [herb. Colla]).
Solanum furcatum Dunal var. glabrum G.Don, Gen. Hist. 4: 412. 1837. Type. “In Peruvia” (no specimens cited; no original material located).
Solanum furcatum Dunal var. pilosum G.Don, Gen. Hist. 4: 412. 1837. Type. “In Peruvia” (no specimens cited; no original material located).
Solanum furcatum Dunal var. acutidentatum Nees, Nov. Act. Acad. Caes. Leop. 19, suppl. 1: 386. 1843, as “*acutedentatum*”. Type. “Chile ad Valparaiso, Februario; Peruvia in planitie circa Tacoram, alt. 14,000–17,000’, Aprili” both syntypes collected by *F.J.F. Meyen s.n.* (no specimens cited; no original material located).
Solanum furcatum Dunal var. obtusidentatum Nees, Nov. Act. Acad. Caes. Leop. 19, suppl. 1: 386. 1843, as “*obtusedentatum*”. Type. “Chile. Prov. de San Fernando in Llano del Rio Tinguiririca, 3,000’ alt., martio”; Peruvia ad Arequipam, Aprili” both syntypes collected by *F.J.F. Meyen s.n.* (no specimens cited; no original material located).
Solanum furcatum Dunal var. subintegerrimum Nees, Nov. Act. Acad. Caes. Leop. 19, suppl. 1: 386. 1843. Type. “Chile: Copiapó, Aprili; Peruvia: circa Tacoram, Aprili” both syntypes collected by *F.J.F. Meyen s.n.* (no specimens cited; no original material located).
Witheringia furcata (Dunal) J.Rémy, Fl. Chil. [Gay] 5: 67. 1849. Type. Based on Solanumfurcatum Dunal
Solanum pterocaulum Dunal var. dichotimiflorum Dunal, Prodr. [A. P. de Candolle] 13(1): 52. 1852, as ‘*pterocaulon*’. Type. Cultivated in France at Montpellier “Solanum speciosum hort. botan” (no specimens cited, described from living plants “v.v. hort. Monsp.”; neotype, designated by Särkinen et al. 2018, pg. 73: MPU [MPU310703]).
Solanum crenatodentatum Dunal, Prodr. [A. P. de Candolle] 13(1): 54. 1852. Type. Chile. Région VI (O’Higgins): Colchagua, San Fernando, “in selibus chilensibus San Fernando”, Mar 1831, *C. Gay 2* (lectotype, designated by D’Arcy 1974a, pg. 738: P [P00337274]).
Solanum rancaguense Dunal, Prodr. [A. P. de Candolle] 13(1): 150. 1852. Type. Chile. Región VI (O’Higgins): Rancagua, May-Oct 1828, *C. Bertero 633* (lectotype, designated by Edmonds 1972, pg. 107 [as holotype], second step designated by Särkinen et al. 2018, pg. 73: P [P00384088]; isolectotypes: BM [BM000617677], G [G00144259], M [M-0171928], MO [MO-503700], NY [NY00743695], P [P00384089], P [P00384090], P [P00384091], P [P00384092], P [P00482266], W [acc. # 1889-0283789]).
Solanum bridgesii Phil., Linnaea 33: 203. 1864. Type. Chile. Región V (Valparaíso): Panquegue, *R.A. Philippi s.n.* (lectotype, designated by Särkinen et al. 2018, pg. 74: SGO [SGO000004549]).
Solanum coxii Phil., Linnaea 33: 200. 1864. Type. Chile. Región X (Los Lagos): Todos los Santos, 1862, *G. Cox 38* (lectotype, designated by Särkinen et al. 2018, pg. 74: SGO [SGO000004555]; isolectotype: W [acc. # 1903-0010246]).
Solanum rancaguinum Phil., Anales Univ. Chile 43: 523. 1873. Type. Chile. Región VI (O’Higgins): Rancagua, Mar 1828, *C. Bertero s.n.* (lectotype, designated by Särkinen et al. 2018, pg. 74: SGO [SGO000004594]).
Solanum caudiculatum Phil., Anales Univ. Chile 91: 12. 1895. Type. Chile. Región VIII (Bío-Bío): prov. Ñuble, Coigüeco, *F. Puga s.n.* (no original material located, not at SGO).
Solanum subandinum Phil., Anales Univ. Chile 91: 13. 1895, nom. illeg., not Solanumsubandinum F.Meigen (1893). Type. Chile. Región XIII (Metropolitana): Santiago, Las Condes, *R.A. Philippi s.n.* (lectotype, designated by Särkinen et al. 2018, pg. 74: SGO [SGO000004600, F neg. 2745]).
Solanum ocellatum Phil., Anales Univ. Chile 91: 14. 1895. Type. Chile. Región XIII (Metropolitana): Prope Colina, *F. Philippi s.n.* (lectotype, designated by Särkinen et al. 2018, pg. 74: SGO [SGO000004582]; isotypes: SGO [SGO000004581], W [acc. # 1903-0010230]).
Solanum nigrum L. var. crentatodentatum (Dunal) O.E.Schulz, Symb. Antill. (Urban) 6: 160. 1909. Type. Based on Solanumcrenatodentatum Dunal
Solanum bridgesii Phil. var. ocellatum (Phil.) Witasek ex Reiche, Anales Univ. Chile 124: 460. 1909. Type. Based on Solanumocellatum Phil.
Solanum andinum Reiche, Fl. Chile 5: 346. 1910. Type. Based on (replacement name for) Solanumsubandinum Phil.
Solanum tredecimgranum Bitter, Repert. Spec. Nov. Regni Veg. 11: 6. 1912. Type. Chile. Región V (Valparaíso): Valparaíso, 17 Aug 1895, *O. Buchtien s.n.* (lectotype, designated by Barboza et al. 2013, pg. 246: US [US00432692, acc. # 139293]; isolectotypes: HBG [HBG511497], US [US00681745, acc. # 139294]).
Solanum robinsonianum Bitter, Repert. Spec. Nov. Regni Veg. 11: 7. 1912. Type. Chile. Región V (Valparaíso): Juan Fernández Island, *R.A. Philippi 742* (holotype: B, destroyed, F neg. 2743; lectotype, designated by Särkinen et al. 2018, pg. 74: W [acc. # 0001347]).
Solanum masafueranum Bitter & Skottsb., Nat. Hist. Juan Fernandez & Easter Island 2: 167, pl. 14. 1922. Type. Chile. Región V (Valparaíso): Juan Fernández Islands, Masafuera [Isla Alejandro Selkirk], Las Chozas, 715 m, 3 Mar 1917 [20 Feb 1917 on label], *C. Skottsberg & I. Skottsberg 363* (lectotype, designated by Särkinen et al. 2018, pg. 74: S [acc. # 04-2947]; isolectotypes: BM [BM000617676], LD [1643307], K [K000585692], NY [00172084], GOET [GOET003548], GB [GB0048742], P [P00337092], UPS [acc. # 104031]).
Solanum spretum C.V.Morton & L.B.Sm., Revis. Argentine Sp. Solanum 132. 1976. Type. Argentina. Río Negro: Bariloche, 19 Mar 1939, *A.L. Cabrera 5024* (holotype: GH [GH00077764]; isotypes F [v0073411F, acc. # 1007493], LP [LP006791]).

####### Type.

Peru? [more likely Chile]. “Cette plante croît au Perou”, *J. Dombey [343*] (lectotype, first step designated by Edmonds 1972, pg. 107 [as holotype], second step designated by Barboza et al. 2013, pg. 246: P [P00335357]; isolectotypes: CORD [CORD00006928], F [v0043232F, acc. # 976864], G [G00359946], G-DC [G00144483], P [P00335358]).

####### Description.

Annual or perennial herbs to 1.0 m tall, erect to lax, subwoody at base, sprawling to ca. 2 m across. Stems terete or ridged, green to purple tinged, not markedly hollow sparsely pubescent with simple, uniseriate 1–5-celled eglandular trichomes 0.1–0.5 mm long; new growth sparsely to densely pubescent with similar simple, uniseriate 1–5-celled eglandular trichomes; older stems sparsely pubescent to glabrescent, pale yellowish brown. Sympodial units difoliate, the leaves not geminate. Leaves simple, (1.5–)4.0–8.0(–2.0) cm long, (0.6–2.2–4.6(–6.5) cm wide, ovate to rhomboidal, green above, slightly paler beneath; adaxial surface sparsely pubescent with simple, uniseriate trichomes like those on stem, these evenly spread along lamina and veins; abaxial surface more densely pubescent; major veins 4–6 pairs; base cuneate to acute, the two sides slightly unequal, decurrent on the petiole; margins sinuate-dentate or entire; apex acute; petioles 1.0–3.5 cm long, sparsely pubescent with simple uniseriate trichomes like those on stem. Inflorescences (1.0–)1.5–3.0(–4.0) cm long, lateral, internodal, forked or more rarely unbranched, with 6–14 flowers clustered at the tips (sub-umbelliform) or evenly spaced along the rhachis, sparsely pubescent with simple uniseriate trichomes like those on stem; peduncle (1.0–)1.5–2.0 cm long; pedicels 4.0–7.5 mm long, 0.2–0.3 mm in diameter at the base and 0.3–0.4 mm in diameter at the apex, straight and spreading, articulated at the base; pedicel scars spaced ca. 0.2–2.5 mm apart. Buds subglobose, the corolla exserted 1/3–1/2 from the calyx tube before anthesis. Flowers 5-merous, all perfect. Calyx tube 2–3 mm long, conical, the lobes 0.8–1.5 mm long, 0.6–1.0 mm wide, rectangular to narrowly obovate with obtuse to shortly acute apices, pubescent with simple uniseriate trichomes like those on stem but shorter. Corolla 12–20 mm in diameter, white to lilac with a green or yellow-green central portion near the base, this sometimes purplish near the lobe midvein, stellate, lobed 1/3–1/2 of the way to the base, the lobes 5.5–7.0 mm long, 2.8–5.5 mm wide, strongly reflexed at anthesis, later spreading, densely pubescent abaxially with 1–4-celled simple uniseriate trichomes, especially along the margins and apex, these shorter than the trichomes of the stems and leaves. Stamens equal; filament tube minute; free portion of the filaments 0.9–1.6 (2) mm long, adaxially pubescent with tangled uniseriate 4–6-celled simple trichomes; anthers 2.3–3.3(-3.6) mm long, 0.8–1.0 mm wide, ellipsoid, yellow, poricidal at the tips, the pores lengthening to slits with age. Ovary globose, glabrous; style 6.0–6.5 mm long, densely pubescent with 2–3-celled simple uniseriate trichomes in the lower 1/2–2/3, exserted 2–3 mm beyond the anther cone and somewhat curved; stigma capitate, minutely papillate, yellow or green in live plants. Fruit a globose berry, 6–9 mm in diameter, dull green to purple at maturity, opaque, the surface of the pericarp matte; fruiting pedicels 7–12 mm long, 0.2–0.4 mm in diameter at the base, 0.5–1.0 mm in diameter at the apex, strongly reflexed, dropping with mature fruits, not persistent; fruiting calyx not accrescent, the tube 1.0–2.0 mm long, the lobes 1.5–2.5 mm long, appressed against the berry. Seeds 30–40 per berry, 1.8–2.0 mm long, 1.4–1.5 mm wide, flattened and tear-drop shaped with a subapical hilum, yellow-brown, the surface minutely pitted, the testal cells pentagonal in outline. Stone cells 6–14 per berry, 0.8–1.0 mm in diameter. Chromosome number: *2n*=6×=72 (Stebbins and Paddock 1949; Edmonds 1982, 1983; Chiarini et al. 2017).

**Figure 18.**
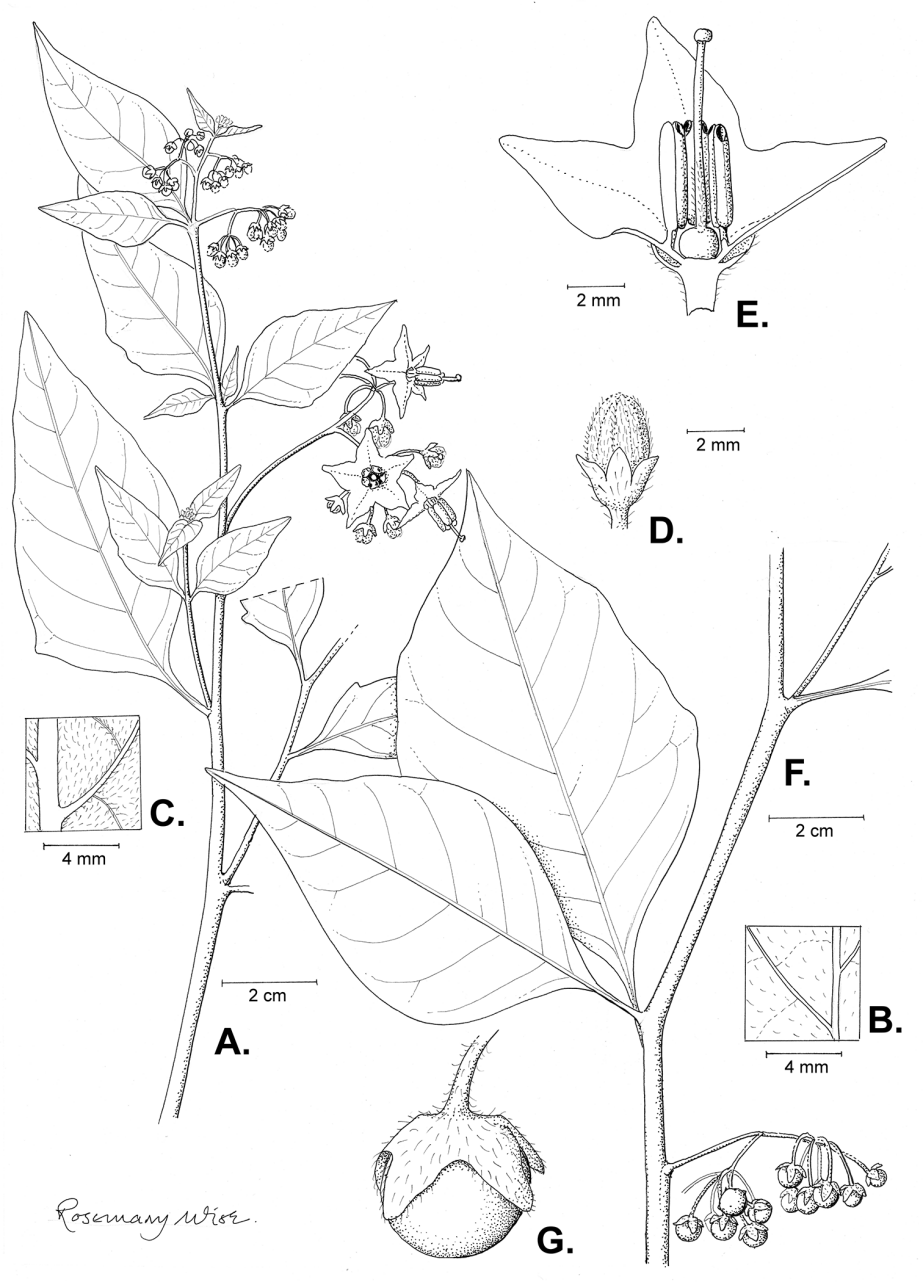
*Solanumfurcatum* Dunal **A** Habit **B** detail of adaxial leaf surface **C** detail of abaxial leaf surface **D** Bud **E** dissected flower **F** detail of infructescence **G** fully mature fruit (**A–G***Särkinen et al. 4095*). Drawing by R. Wise (previously published in “PhytoKeys 106”).

**Figure 19.**
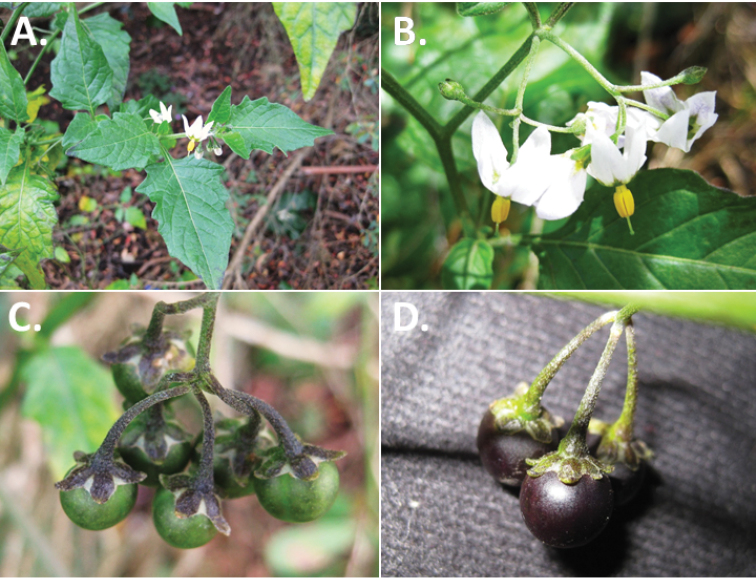
*Solanumfurcatum* Dunal **A** Flowering branch **B** inflorescence with flowers at full anthesis **C** developing fruits **D** mature fruits (**A–D***Knapp s.n.* Golden Gate Park). Photos by S. Knapp.

####### Distribution.

**Figure 20.**
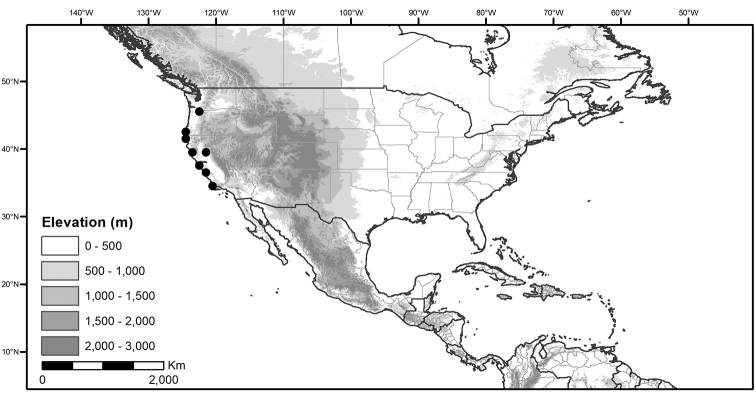
Distribution of *Solanumfurcatum* Dunal.

(Figure 20) *Solanumfurcatum* is native to Chile (incl. the Juan Fernández Islands) and adjacent Andean Argentina. It is probably locally introduced and naturalised along the west coast of the United States of America, Australia and New Zealand. Only a few specimens have been seen from California and Oregon, but in those areas the species is clearly naturalised. Wiggins (1980) recorded *S.furcatum* from Baja California (Mexico), with no specimen citations; all specimens we have seen identified as *S.furcatum* from Baja California are plants of *S.douglasii*.

####### Ecology.

In western North America *S.furcatum* is a plant of disturbed areas in winter-wet areas along sea cliffs and bluffs between sea level and 100 m elevation.

####### Common names.

United States of America. Forked nightshade (USDA Plants 2017).

####### Uses.

None recorded in the region.

####### Preliminary conservation status (IUCN 2017).

Least Concern (LC). *Solanumfurcatum* is introduced into the western United States, where it is not common, but its worldwide range is very large. For EOO see Table 6.

####### Discussion.

*Solanumfurcatum* can be distinguished from the similar and sympatric *S.douglasii* in its usually forked inflorescences, globose buds from which the style is often exserted, ellipsoid anthers on distinct filaments, and berries with usually more than 10 stone cells. *Solanumfurcatum* is not sympatric with S. *nigrescens* but differs from it in the same set of characters and in its style that is exserted for as long as the anther cone (e.g., exserted portion of the style equal to the length of the anther cone).

*Solanumfurcatum* was considered to be an introduction from Chile by Stebbins and Paddock (1949) and is well-established but not common in coastal areas from Oregon to central California. Other Chilean species have similarly become established along the west coast of the United States [such as *Nicotianaacuminata* (Graham) Hook., see Goodspeed 1954; Knapp in press].

Details of typification for the synonyms of *S.furcatum* can be found in Särkinen et al. (2018).

####### Specimens examined.

See Suppl. materials 1 and 3.

###### 7. Solanum interius

Taxon classificationPlantaeSolanalesSolanaceae

Rydb., Bull. Torrey Bot. Club 31: 641. 1905 [“1904”]

Figures 21 , 22

Solanum nigrum L. var. interius (Rydb.) F.C.Gates, Trans. Kansas Acad. Sci. 42: 137. 1940. Type. Based on Solanuminterius Rydb.

####### Type.

United States of America. Nebraska: Hooker County, on Middle Loup River, near Mullen, 20 Jul 1893, *P.A. Rydberg 1385* (lectotype, designated here: NY [NY00138953] isotypes: GH [GH00077424], H [acc. # 1087075], NDG [NDG45091], NEB [NEB-V-0000607], NY [NY00138952], US [US00027625, acc. # 210385; US02828882, acc. # 210353]).

####### Description.

Annual herbs to subwoody perennial shrubs up to 1.0 m tall, branching at base. Stems terete to ridged, pale straw colour, sparsely pubescent with simple, appressed, uniseriate (2)4–8-celled trichomes, these ca. 0.6 mm long, the new growth more densely pubescent. Sympodial units difoliate, the leaves not geminate. Leaves simple, 4.5–11.2 cm long, 2.3–6.8 cm wide, ovate to broadly ovate, membranous, green on both sides; adaxial surface sparsely pubescent with appressed translucent, simple, uniseriate trichomes like those on stem scattered along veins and lamina; abaxial surface more densely pubescent with trichomes like those of the upper surface across both lamina and veins; primary veins 4–6 pairs; base attenuate; margins sinuate dentate, especially so up to 2/3 from the base, to occasionally entire; apex acute to acuminate; petiole 0.5–3.5 cm long, pubescent with simple uniseriate trichomes like those of the stems. Inflorescences 2.4–3.5 cm long, lateral, internodal, unbranched or rarely forked, with (2)3–8 flowers clustered near the tips (sub-umbelliform) or less commonly the distal flowers spaced along the rhachis, the lowermost flower distant from the rest, sparsely pubescent with appressed simple uniseriate trichomes like those on stem, rhachis 2–10 mm long when present; peduncle 1.0–2.0 cm long, straight; pedicels 5–8 mm long, 0.3–0.4 mm in diameter at the base and 0.4–0.5 mm in diameter at the apex, spreading, the terminal pedicels articulated at the base, but the lowermost flower with the pedicel articulated in the basal 1/4 to 1/3; pedicels spaced 0–1.0 mm apart. Buds globose, corolla exserted from the calyx 1/5 to 1/3. Flowers 5-merous, all perfect. Calyx tube 1.0–1.5 mm long, lobes irregularly unequal, the longest 1.7–4.5 mm long, 0.6–0.7 mm wide, lanceolate with acute to acuminate apices, sparsely pubescent with appressed hairs like those on stem but shorter. Corolla 6–12 mm in diameter, stellate, white with a yellow-green central portion near the base, lobed 1/2 to 2/3 to the base, the lobes 4.0–5.0 mm long, 2.0–3.0 mm wide, strongly reflexed at anthesis, later spreading, densely pubescent abaxially with simple uniseriate trichomes like those on stem and leaves but shorter. Stamens equal; filament tube minute; free portion of the filaments 0.7–1.0 mm long, pubescent with tangled uniseriate simple trichomes; anthers 1.8–2.5 mm long, 0.6–0.9 mm wide, ellipsoid, yellow, poricidal at the tips, the pores lengthening to slits with age. Ovary globose, glabrous; style 3.5–4.5 mm long, exserted 0–1 mm beyond anther cone, densely pubescent with 2–3-celled simple uniseriate trichomes along 2/3 from the base; stigma capitate, minutely papillate, green in live plants. Fruit a globose berry, 10–14 mm in diameter, purple-black at maturity, opaque, the surface of the pericarp shiny; fruiting pedicels 6–10 mm long, 0.4–0.6 mm in diameter at the base, 0.6–1.0 mm in diameter at the apex, recurved to reflexed, pedicels spaced 0.5–2.5 mm apart, dropping with mature fruits, occasionally not dropping; fruiting calyx not accrescent, the tube 1.5–2.0 mm long, the lobes (2.0–)3.0–4.0 mm long with the apices spreading to strongly reflexed in fruit. Seeds 20–40 per berry, 1.8–2.0 mm long, 1.5–1.6 mm wide, flattened and tear-drop shaped with a subapical hilum, yellow, the surfaces minutely pitted, the testal cells pentagonal in outline. Stone cells 2–4, 0.8–1.0 mm in diameter, white or cream coloured. Chromosome number: *2n*=2×=24 (Heiser et al. 1965).

**Figure 21.**
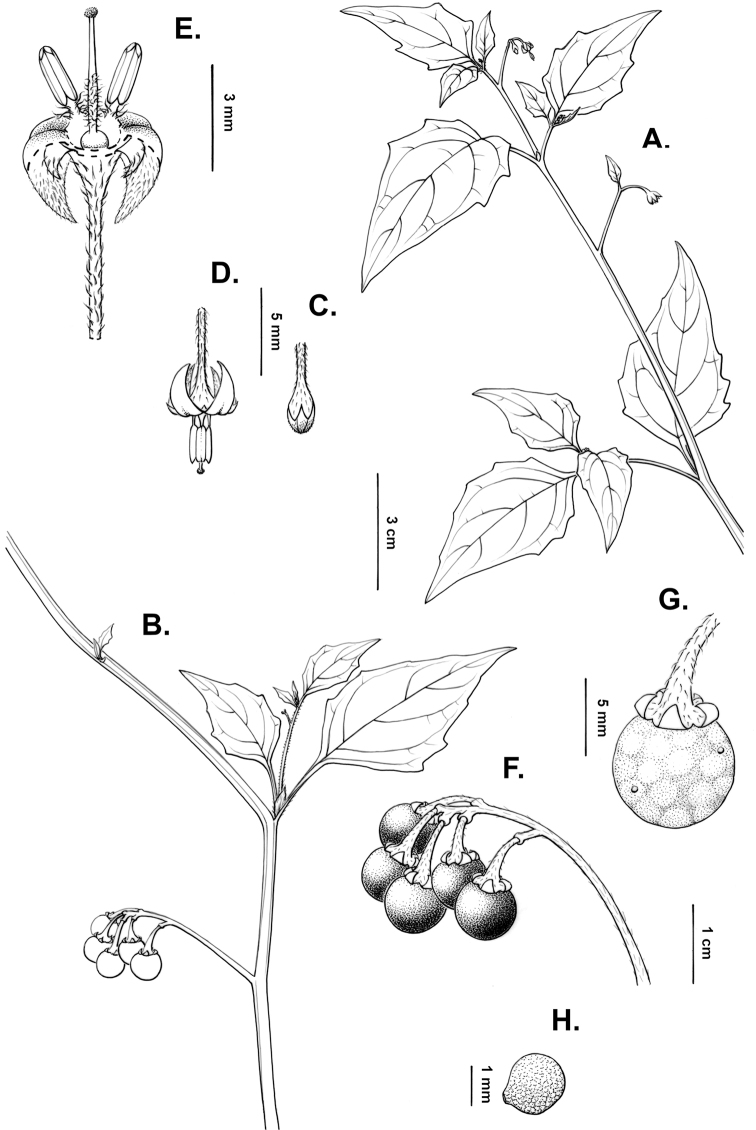
*Solanuminterius* Rydb. **A** Habit **B** fruiting habit **C** bud **D** flower **E** dissected flower **F** mature fruits **G** dried berry **H** seed (**A–H***Wooton 50*). Drawing by C. Banks.

**Figure 22.**
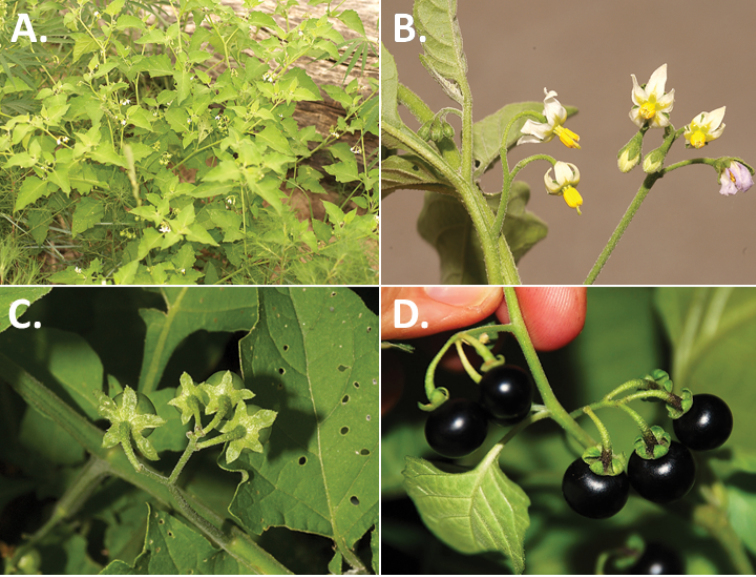
*Solanuminterius* Rydb. **A** Habit **B** flowering branch **C** developing fruits **D** mature fruits (**A–C***Nee 61350*; **D***RBGE cultivated*). Photos by M. Nee and T. Särkinen.

####### Distribution.

**Figure 23.**
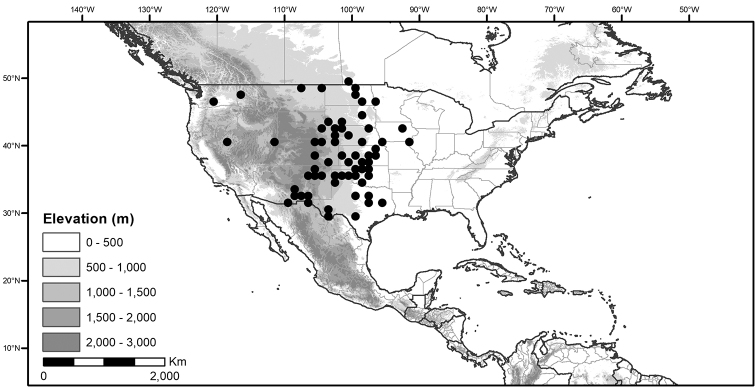
Distribution of *Solanuminterius* Rydb.

(Figure 23) *Solanuminterius* is endemic to North America, and the most common species of morelloid in the Great Plains. It is less common west of the Rocky Mountains, and although it does extend to Arizona and western New Mexico, *S.interius* does not occur on the Gulf Coast where it is replaced by *S.nigrescens*. Records of *S.interius* for Canada (Saskatchewan, Harms 2006) have not been verified with voucher specimens, although it is to be expected there. Specimens annotated by Harms as *S.interius* in SASK are of *S.emulans* (e.g., *Child s.n.*, collected 29 July 1941)

####### Ecology.

*Solanuminterius* is found in open habitats in sand hills and low forest, often in shade under trees between (100-)500 to 2,500 m elevation.

####### Common names.

United States of America. Deadly nightshade (USDA Plants 2017), Inland nightshade, Plains black nightshade, morelle de l’interieur (NatureServe 2017), Yerba mora negra (New Mexico, *Hill 14851*).

####### Uses.

None recorded on herbarium labels; Heiser (1969; quoting Charles Bessey) quotes “a young man from Fort Dodge, Iowa spoke up and said that the people in his neighbourhood made them [berries of “*S.nigrum*”] into pies, preserves, etc. and ate freely of them.” Bessey (quoted in Heiser 1969) went on to say that later he found that in central and western Iowa nightshades were indeed eaten; it is possible these were the berries of *S.interius*.

####### Preliminary conservation status (IUCN 2017).

Least Concern (LC). *Solanuminterius* is widespread through the Great Plains region in the United States of America. For EOO see Table 6.

####### Discussion.

*Solanuminterius* can be distinguished from other North American morelloids by its inflorescence with apparently uneven branches, one with several flowers and the other apparently with a single flower that is actually the basal flower with the articulation ca. 1/4 to 1/3 of the way up the pedicel, very like the pedicel articulation in wild potatoes. Other distinguishing features to be used in combination with this are the medium-sized anthers 1.8–2.5 mm long and relatively long rectangular calyx lobes with rounded apices. *Solanumnigrescens* has more regularly spaced flowers, occasionally branched inflorescences with more than one flower per branch and is more common along the Gulf Coast but distinguishing the two species without locality information can be difficult. The seeds of *S.interius* are much larger than those of *S.nigrescens* (1.8–2 mm versus 1.2–1.5 mm long) or any other of the diploid morelloids occurring in the area. Stone cell number can also be used as a distinguishing character; *S.interius* has 2–4 stone cells in each berry while *S.nigrescens* has more than 6 and often as many as 12.

*Solanuminterius* is sympatric with *S.emulans* and can be distinguished from that species in its longer anthers (1.8–2.5 mm long versus 1.5–1.8 mm long), its rounded calyx lobe apices, and its larger berries (10–14 mm in diameter versus 6–8 mm in diameter) with larger seeds (1.8–2.0 mm long versus 1.6–1.8 mm long). The flowers of *S.emulans* are usually smaller than those of *S.interius*. Nee (on label of *Nee 61337*) says that living plants of the two species are quite distinct, and that *S.interius* is a perennial growing in the shade of single trees.

In describing *S.interius*Rydberg (1905) cited a collection but no herbarium. We have selected the sheet in the herbarium (NY) where he worked (NY00138953) that is annotated “Type” as the lectotype of this species following best practice as described in McNeill (2014).

####### Specimens examined.

See Suppl. materials 1 and 3.

###### 8. Solanum macrotonum

Taxon classificationPlantaeSolanalesSolanaceae

Bitter, Repert. Spec. Nov. Regni Veg. 11: 222. 1912

Figures 24 , 25

Solanum megalophyllum Bitter, Repert. Spec. Nov. Regni Veg. 11: 202. 1912. Type. Cultivated in England (?) ex Herb. A.B. Lambert “Villa Caracas cultum in hort. Boyton, Ph. Woodford”, *Anon. s.n.* (lectotype, designated here: W [acc. # 1889-0291427]; isolectotype: W [acc. # 1889-0291426, F neg. 33091]).
Solanum diodontum Bitter, Repert. Spec. Nov. Regni Veg. 12: 552. 1913. Type. Panama. Chiriqui: around El Potrero Camp, 2800–3000 m, 10–13 Mar 1911, *H. Pittier 3104* (holotype: US [US00027551, acc. # 677494]; isotype: GH [GH00077485], NY [NY00138980], US [US00027550, acc. # 1405957]).
Solanum leonii Heiser, Ceiba 4: 298. 1955. Type. Costa Rica. Cartago: near Robert, Irazú [protologue -wooded ravine 1/2 mile below Finca Robert], 8,500 ft., 4 Oct 1953, *C.B. Heiser 3597* (holotype [two sheets]: IND [sheet 1, IND-0136009, acc. # 95138; sheet 2, IND-00136010, acc. # 95137]; isotype: F [v0073111F, acc. # 143245, F neg. 49431]).
Solanum paredesii Heiser, Ci. & Naturaleza [Quito] 6: 55. 1963. Type. Ecuador. Pichincha: [Cantón Quito] laderas al norte de los terrenos de la Universidad Central, Ciudad Universitaria Quito, 24 May 1962, *C.B. Heiser 5001* (holotype: IND [IND-0136006, acc. # 106787]; isotype: Q [n.v.]).

####### Type.

Venezuela. Aragua: Colonia Tovar, Sep 1847, *J.W.K. Moritz 1643* (holotype: B, destroyed; lectotype, designated by D’Arcy 1974a, pg. 737: P [P00336967]; isolectotypes: B [destroyed, F. neg. 2669], BM [BM000617678], F [v0073325F, acc. # 612111], HBG [HBG-511459], K [K000585559]).

####### Description.

Perennial herb, woody at the base, 0.7–2 m tall, perhaps occasionally annual or only persisting for a few years. Stems terete or angled with spinescent processes, often described as “viney”, arching and scrambling over other vegetation, often drying blackish grey; young stems densely pubescent with somewhat antrorse, simple uniseriate eglandular trichomes 0.5–1 mm long, the trichomes drying white, soon glabrescent; new growth densely white pubescent like the young stems, glabrescent; bark of older stems green to greenish brown. Sympodial units difoliate or unifoliate, the leaves not geminate. Leaves simple, occasionally with a few dentate teeth near the base, (2)4–10(12) cm long, (0.8)1.8–4.5(5.5) cm wide, elliptic to narrowly obovate, sometimes thick (described as succulent), but more often membranous; adaxial surfaces sparsely pubescent with simple 3–4-celled uniseriate trichomes or almost glabrous, the trichomes denser on veins and midrib; abaxial surfaces sparsely pubescent to glabrous like the adaxial surfaces, but the trichomes denser along the veins; principal veins 5–7 pairs, drying paler abaxially; base abruptly attenuate along the petiole; margins entire to sparsely toothed near the base; apex acute to narrowly acute; petiole 0.5–2.5 cm, sparsely pubescent with antrorse simple uniseriate trichomes like those of the stems and leaves. Inflorescences internodal or very occasionally leaf-opposed, 0.7–4 cm long, unbranched, with 2–3(7) flowers clustered in the distal part of the rhachis (sub-umbelliform), sparsely pubescent with simple uniseriate trichomes like those of the stems and leaves; peduncle 0.5–4 cm long; pedicels 1–1.3 cm long, ca. 0.5 mm in diameter at the base, ca. 1 mm in diameter at the apex, tapering gradually and appearing relatively stout, often described as reddish purple or purple, spreading at anthesis, sparsely pubescent or glabrous, articulated at the base; pedicel scars tightly packed in the distal portion of the inflorescence, less than 0.5 mm apart or occasionally the lowermost scar to 2 mm apart. Buds broadly ellipsoid to subglobose, the corolla long-exserted from the calyx tube before anthesis. Flowers 5-merous, perfect. Calyx tube 1–1.5 mm long, conical, the lobes 0.5–0.8(1) mm long, 0.5–1 mm wide, broadly deltate with acute apices, sparsely pubescent with simple uniseriate trichomes like those of the pedicel or almost glabrous. Corolla 10–20 mm in diameter, white to lilac or tinged with lilac, the central portion yellowish green, stellate, lobed 1/2 to 2/3 of the way to the base, the lobes 4–6 mm long, 1.5–3 mm wide, triangular, reflexed or spreading at anthesis. Stamens equal; filament tube minute and barely visible, the free portion of the filaments 1–2 mm long, pubescent with tangled simple uniseriate trichomes adaxially; anthers (2.7)3–4 mm long, 1–1.5 mm wide, ellipsoid, bright yellow, the surfaces smooth, poricidal at the tips, the pores elongating to slits with age. Ovary glabrous; style 5–6 mm long, densely pubescent with tangled simple uniseriate trichomes in the basal half where included in the anther cone, exserted from the anther cone; stigma capitate or minutely capitate, bright green, the surface densely papillate. Fruit a globose berry, 0.8–1 cm in diameter, green turning to black when ripe, or occasionally green when ripe (*Nee & Whalen 16839*), opaque, the pericarp thin, more or less shiny but not brilliantly so; fruiting pedicels 15–17 mm long, tapering from a base 0.7–1 mm in diameter to an apex 1.5–2 cm in diameter, somewhat woody, strongly deflexed(very occasionally appearing spreading due to herbarium specimen preparation), dropping with mature fruits or occasionally remaining on the inflorescence rhachis; fruiting calyx not accrescent, the tube 1–1.5(2) cm long, appressed to the berry, the lobes 0.5–1 mm long, appressed or spreading at the tips. Seeds (10)30–50 per berry, 1.2–1.5 mm long, 0.8–1 mm wide, tear-drop shaped, tan to reddish brown, the surfaces minutely pitted, the testal cells pentagonal, more elongate and rectangular near the hilum. Stone cells (2)4–5(6) per berry, 0.5–0.7 mm in diameter. Chromosome number: *2n* =2×=24 (Heiser 1955, as *S.leonii*), *2n*=6×=72 (Heiser 1963, as. *S.paredesii*).

**Figure 24.**
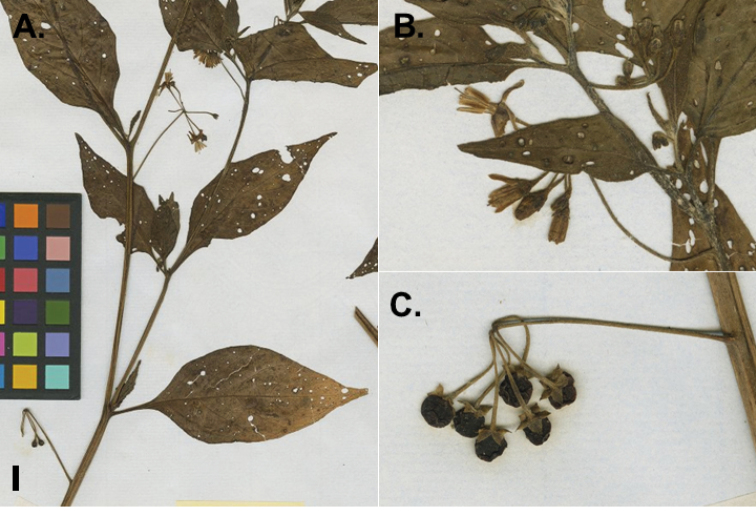
*Solanummacrotonum* Bitter. **A** Reproductive stem **B** detail of inflorescence **C** detail of infructescence (**A–C***Moritz 1643*, K000585559). Scale bar in **A**: 1 cm.

**Figure 25.**
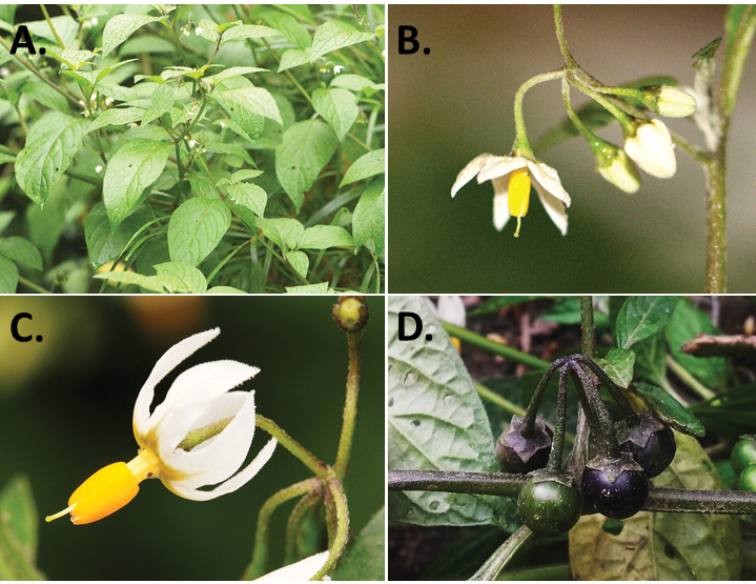
*Solanummacrotonum* Bitter. **A** Habit **B** flowering branch **C** inflorescence with flowers at full anthesis **D** mature fruits (all based on *Ezedin & Särkinen 48*). Photos by T. Särkinen.

####### Distribution.

**Figure 26.**
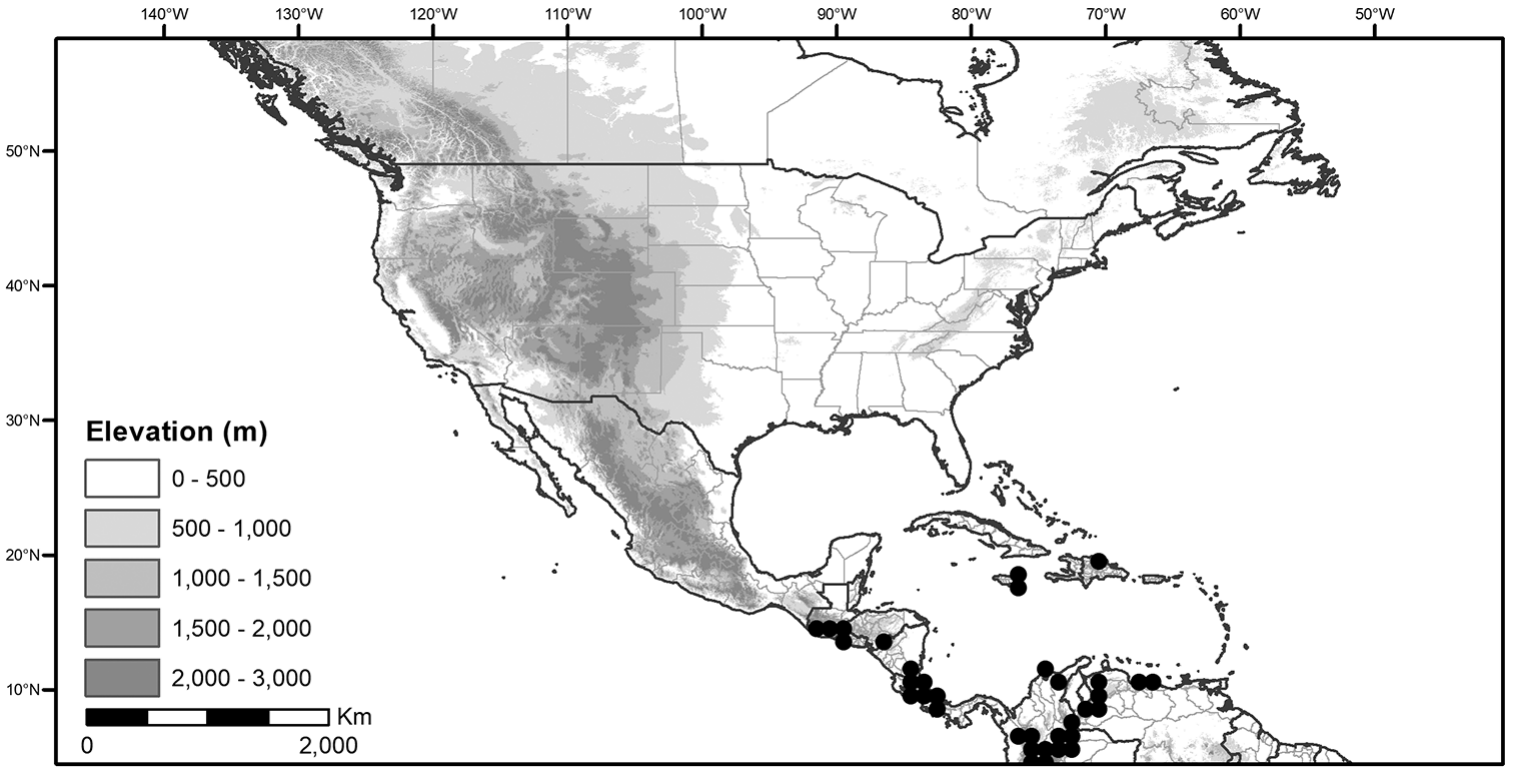
Distribution of *Solanummacrotonum* Bitter (points in northern South America included to show continuous distribution).

(Figure 26) *Solanummacrotonum* occurs from Guatemala to northern South America and in the Antilles on the islands of Hispaniola and Jamaica.

####### Ecology.

*Solanummacrotonum* is a plant of open areas in cloud forests and premontane and montane forests, occurring in treefall gaps and along roads and other disturbances, from (200-)1,000 to 3,400 m elevation.

####### Common names.

Costa Rica. Hierba (yerba) buena (Bohs 2015).

####### Uses.

None recorded.

####### Preliminary conservation status (IUCN 2017).

Least Concern (LC). *Solanummacrotonum* is widespread and weedy in Mexico, Central America and in the Caribbean; it also occurs in northern South America. For EOO see Table 6.

####### Discussion.

*Solanummacrotonum* is broadly sympatric with *S.nigrescens* across its entire range, and in Mexico and northern Central America with *S.douglasii*. It is similar to them in having usually 4 to 5 stone cells per berry and black fruits that are more or less shiny. It can be distinguished from *S.nigrescens* in having longer anthers (to 4 mm rather than to 2.5 mm) and in having more robust, longer fruiting pedicels that are strongly deflexed. It differs from *S.douglasii* in having strictly ellipsoid anthers (versus the slightly tapering anthers of *S.douglasii*) on longer filaments, and similarly in the strongly deflexed fruiting pedicels. Many annotations in herbaria have been done based on elevation (see comments in Bohs 2015) so care must be taken with these determinations. Measurement of anthers is the best way to determine identifications unambiguously. In general, *S.macrotonum* does occupy slightly higher elevations than does *S.nigrescens*, and appears to be confined to cloud forests, but *S.nigrescens* has a wide elevation range and ecological tolerance.

*Solanummacrotonum* is one of few morelloids with differing chromosome counts across its range. D’Arcy (1974a) reported a chromosome number of “n=36” for *S.macrotonum* as a personal communication from J.M. Edmonds; the chromosome count in Edmonds (1972) is not new, and we presume it is a reference to the count (“número de cromosomas – 36”; Heiser 1963) given in the protologue of *S.paredesii*, which Edmonds (1972) placed in tentative synonymy with *S.macrotonum*. Some other chromosome vouchers of *S.macrotonum* at IND, however (e.g., *Heiser 4854*) are noted as having “n=24” on the label; Heiser (1963) did not cite these in the description of *S.paredesii*. Chromosome counts for *S.leonii*, here treated in synonymy with *S.macrotonum*, indicate it is diploid, with n=12 (Heiser 1955). Chromosome number variation within a species is known in *Solanum* (e.g., in the potatoes, see Spooner et al. 2014), and sometimes occurs sporadically at the edges of species ranges. It will be important to assess this across the range of *S.macrotonum*, because we cannot find any morphological characteristic that distinguishes vouchers with different chromosome counts.

Bitter (1912b) cited a single specimen from B in the protologue of *S.macrotonum* that is no longer extant. The sheet at P (P00336967) that has a label with all of the details cited in the protologue was chosen by D’Arcy (1974a) as the lectotype of *S.macrotonum*.

*Solanummegalophyllum* was described from cultivated specimens from the herbarium of Aylmer B. Lambert seen by Bitter (1913) in the herbarium in Vienna. Of the two specimens that are clearly duplicates of this collection, we have selected that with the more complete annotation in Bitter’s hand as the lectotype (W-1889-0291427).

Heiser (1955, 1963) described both *S.leonii* and *S.paredesii* citing “IND” as type. *Heiser 5001* (type of *S.paredesii*) is only represented by a single sheet, but there are two sheets of *Heiser 3597*, the type of *S.leonii*, labelled “sheet 1” and “sheet 2”. We interpret this as a two-sheet holotype (see Turland et al. 2018, Art. 8, Ex. 7). Sheet 2 (IND-0136010) is better material with more leaves and flowers.

It is possible that *S.frutescens* A.Braun & C.D.Bouché is an older name for this taxon; that name has never been used, however, and it has been proposed for suppression (see Knapp et al. 2018; Doubtful Species).

####### Specimens examined.

See Suppl. materials 1 and 3.

###### 9. Solanum nigrescens

Taxon classificationPlantaeSolanalesSolanaceae

M.Martens & Galeotti, Bull. Acad. Roy. Sci. Bruxelles 12(1): 140. 1845

Figures 27 , 28

Solanum nodiflorum Jacq. var. puberulum Dunal, Prodr. [A. P. de Candolle] 13(1): 46. 1852. Type. United States of America. Texas: [Bexar County] “Mexico, Bejar”, Oct 1828, *J.L. Berlandier 1904* (lectotype, designated by Edmonds 1972, pg. 103 [as holotype]: G-DC [G00144231]; isotypes: MO [acc. # 5481286], NY [NY00743232], P [P00319514], W [acc. # 0022313]).
Solanum caribaeum Dunal, Prodr. [A. P. de Candolle] 13(1): 48. 1852. Type. Jamaica. Sin.loc., [protologue – “In insulis Caribaeis, Jamaica, Guadalupâ”], no date, *Anon. s.n.* (lectotype, designated by D’Arcy 1974a, pg. 735: G-DC [G0000144199]).
Solanum crenatodentatum Dunal var. ramosissimum Dunal, Prodr. [A. P. de Candolle] 13(1): 54. 1852. Type. United States of America. Louisiana: “Basse Louisiane”, 1839, *G.D. Barbe 82* (holotype: P [P00362535]).
Solanum nigrum L. var. nigrescens (M.Martens & Galeotti) Kuntze, Revis. Gen. Pl. 2: 455. 1891. Type. Based on Solanumnigrescens M.Martens & Galeotti
Solanum nigrum L. var. amethystinum Kuntze, Revis. Gen. Pl. 2: 455. 1891. Type. Costa Rica. San José/Cartago: “Irazu”, 24 Jun 1874, *O.E. Kuntze s.n.* (neotype, designated here: NY [NY00688134]).
Solanum prionopterum Bitter, Repert. Spec. Nov. Regni Veg. 11: 5. 1912. Type. Venezuela. Distrito Federal: “Caracas, in arena ad rivulum in valle loci dicti Valle”, 25 Mar 1854, *J. Gollmer s.n.* (holotype: B, destroyed [F neg. 2699], possibly the same original material as the type of S.gollmeri; no duplicates found).
Solanum gollmeri Bitter, Repert. Spec. Nov. Regni Veg. 11: 202. 1912. Type. Cultivated in Berlin (“horto bot. Berol.”) from seeds sent from Caracas, Venezuela by J. Gollmer, 1859, *Without collector s.n.* (holotype: B, destroyed [F neg. 2689]; lectotype, designated here: F [V0361922F, acc. # 621268], mounted on sheet with F neg. 2689).
Solanum pruinosum Dunal var. phyllolophum Bitter, Repert. Spec. Nov. Regni Veg. 12: 77. 1913. Type. Cultivated in Europe, seeds from Mexico from David Fairchild as USDA-32065 [protologue “sub. no. 32065, Mexico, S.nigrum”] (no specimens cited, probably described from living plants; original material at B?).
Solanum subelineatum Bitter, Repert. Spec. Nov. Regni Veg. 12: 79. 1913. Type. Cultivated at Bremen from seeds from Mexico sent by U. S. Dept. Agriculture, Bureau of Plant Industry, no. 32067 (original material in Bremen?, destroyed; possibly described from living material).
Solanum oligospermum Bitter, Repert. Spec. Nov. Regni Veg. 12: 80. 1913. Type. Mexico. Oaxaca: Sierra de San Felipe, 7500 ft., Oct 1894, *C.G. Pringle 4948* (lectotype, designated by Edmonds 1972, pg. 108 [as holotype]: Z [Z000033841]; isolectotypes: BM [BM001017184], BR [BR0000005537983], E [E00570141], GOET [GOET003559], HBG [HBG511469], KFTA [KFTA0000498], NDG [NDG45082], NY [NY00139012], PH [PH00030459], S [acc. # S-G5704], US [US00027711, acc. # 251984; US01014256, acc. # 1418095], W [acc. # 1895-0004424]).
Solanum durangoense Bitter, Repert. Spec. Nov. Regni Veg. 12: 82. 1913. Type. Mexico. Durango: “prope urbem Durango”, Apr 1896, *E. Palmer 101* (holotype: B, destroyed; lectotype, designated by D’Arcy 1974a, pg. 738: US [US00027556, acc. # 304231]; isolectotypes: BM [BM001034665], F [V0073093F, acc. # 51213, F. neg. 052464], K [K000063870], MO [MO-568723, acc. # 1718478], NY [NY00138982]).
Solanum purpuratum Bitter, Repert. Spec. Nov. Regni Veg. 13: 85. 1913. Type. Bahamas. Andros Island: Coppice, near Fresh Creek, Northern Section, 28–13 Jan 1910, *J.K. Small & J.J. Carter 8805* (holotype: P [P00369223]; isotypes: F [acc. # 283797], K [K001161011], NY [NY00111385], US [US00027765, acc. # 758168]).
Solanum approximatum Bitter, Repert. Spec. Nov. Regni Veg. 13: 86. 1913. Type. Jamaica. Saint Andrew: Hardwar Gap, 4000 ft., 17 Jun 1903, *G.E. Nichols 89* (holotype: B, destroyed; lectotype, designated here: NY [NY00111374]; isolectotypes: F [F0073167F, acc. # 147000], GH [GH00077545], MO [MO-503650, acc. # 1815480], US [US00027456, acc. # 429037], YU [YU065289]).
Solanum amethystinum (Kuntze) Heiser, Ceiba 4: 296. 1955. Type. Based on SolanumnigrumL.var.amethystinum Kuntze
Solanum costaricense Heiser, Ceiba 4: 297. 1955. Type. Costa Rica. Heredia: La Paz, by waterfall, on road to Vara Blanca, about 29 mi. from Heredia, 1400 m, 13 Sep 1953, *C.B. Heiser 3536* (holotype [two sheet holotype]: IND [IND1000067, acc. # 95105; IND1000068, acc. # 95106]; isotypes: CORD [CORD00004189], US [cited in protologue, n.v.]).

####### Type.

Mexico. Oaxaca: “Cordillera” [“aux bords des ruiseaux de la cordillera de Yavezia”], Nov-Apr 1848, *H. Galeotti 1238* (lectotype, designated by D’Arcy 1974a, pg. 737: P [P00337261]; isolectotypes: BR [BR000000825045, BR0000008250483], W [acc. # 0022312, acc. # 1889-0291397]).

####### Description.

Perennial herbs to 3 m tall, sometimes epiphytic. Stems terete or more usually angled to ridged, green or sometimes tinged purplish green, usually lax and somewhat scrambling, glabrescent to sparsely pubescent with antrorse simple eglandular uniseriate trichomes to 1 mm long, these white when dry and usually somewhat curved, occasionally on older stems the trichome bases enlarged and forming spinescent processes; new growth more densely pubescent. Sympodial units difoliate, geminate or not, the leaves if paired of similar size and shape. Leaves simple, (1.5)4–10.5(15) cm long, (0.5)2–5(7.5) cm wide, elliptic to elliptic ovate, membranous; surfaces sparsely to moderately pubescent with simple eglandular uniseriate trichomes to 1 mm long, these denser on the veins and abaxially; principal veins 5–6 pairs; base abruptly attenuate, usually decurrent on the petiole; margins entire to sinuate or dentate, the teeth irregular and unevenly spaced, often larger in the basal half of the lamina; apex acute or occasionally acuminate; petiole 0.5–2 cm long, sparsely pubescent like the stems and leaves. Inflorescence 1–3.5 cm long, lateral and internodal, unbranched to occasionally forked, with (2)5–10 flowers clustered at the tip (sub-umbelliform) or spaced along the rhachis (depending on inflorescence age), sparsely pubescent with antrorse simple eglandular trichomes like the stems; rhachis 0.3–1 cm long; peduncle 1–2.5 cm long, slender, spreading; pedicels 0.4–0.7 cm long, slender and threadlike, spreading at anthesis, ca. 1 mm in diameter at the base, ca. 0.5 mm in diameter at the apex, sparsely pubescent like the inflorescence axis. Buds ellipsoid with blunt tips, the corolla strongly exserted from the calyx tube long before anthesis. Flowers 5-merous, all perfect. Calyx tube 1–1.2 mm, conical, the lobes 0.5–0.8(1) mm long, 0.5–1 mm wide, broadly deltate to deltate, the apices acute or occasionally somewhat rounded. Corolla 8–10 mm in diameter, white or less often pale purple, with a green or yellow-green (very occasionally dark purple) central portion near the base of the lobes, stellate, lobed ca. 3/4 of the way to the base, the lobes 3–4 mm long, 1.5–2 mm wide, narrowly triangular, reflexed or spreading, densely papillate abaxially, the papillae ca. 0.1 mm long, denser at the tips and margins. Stamens equal; filament tube minute; free portion of the filaments 0.5–2 mm long, densely pubescent adaxially with tangled simple trichomes; anthers 2–2.8(3) mm long, 1–1.1 mm wide, yellow, ellipsoid or narrowly ellipsoid, sagittate at the base, poricidal at the tips, the pores lengthening to slits with age. Ovary globose, glabrous; style 3.5–5 mm long, usually somewhat curved, often exserted from the bud before anthesis, densely pubescent in the basal 2/3 (the portion inside the anther cone), exserted from the anther cone; stigma minutely capitate, the surface papillose. Fruit a globose berry, 6–8 mm in diameter, dull green to purplish black at maturity, opaque, the pericarp thin and usually matte but sometimes slightly shiny; fruiting pedicels 10–12 mm long, ca. 0.5 mm in diameter at the base, ca. 1 mm in diameter at the apex, not markedly woody, spreading, dropping with mature fruits or occasionally remaining on the inflorescence rhachis; fruiting calyx not accrescent, tube ca. 1 mm long, the lobes 0.5–1.1 mm long, spreading and appressed to the berry, very occasionally somewhat reflexed. Seeds (5)10–50 per berry, 1.2–1.5 mm long, 1–1.1 mm wide, tear-drop shaped, pale brown to yellow, the surfaces minutely pitted, the testal cells square or pentagonal in shape, becoming elongate and rectangular near the subapical hilum. Stone cells 4–13, mostly commonly 5 or 6, rather large ca. 0.5 mm in diameter. Chromosome number: *2n*=2×=24 (Heiser 1955 as *S.costaricense*; Heiser et al. 1965 as *S.amethystinum*).

**Figure 27.**
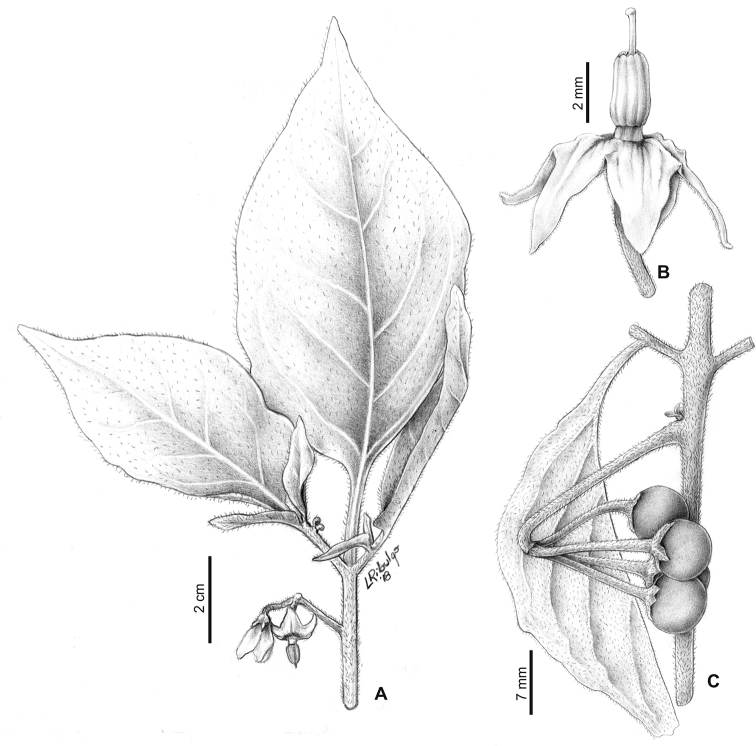
*Solanumnigrescens* M.Martens & Galeotti. **A** New shoot **B** flower **C** inflorescence with mature fruit (**A–C***Ventura A. 672*). Drawing by L. Ribulgo.

**Figure 28.**
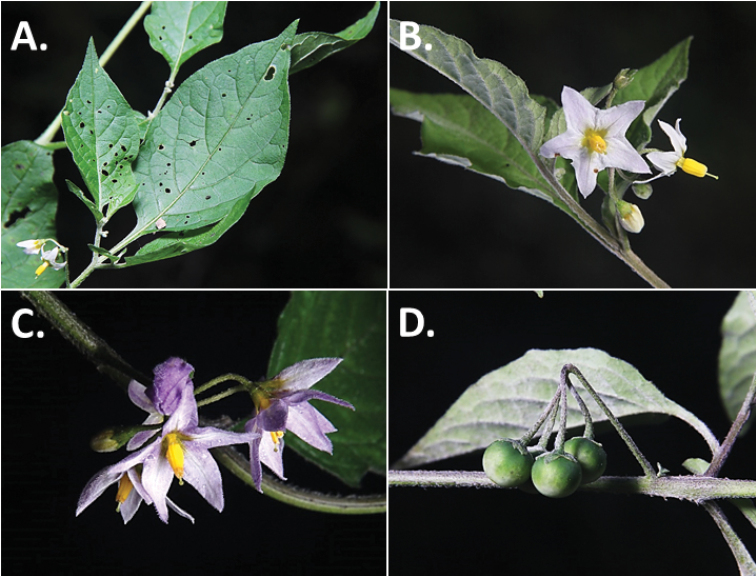
*Solanumnigrescens* M.Martens & Galeotti. **A** Leaves **B** flowering branch **C** inflorescence with flowers at full anthesis **D** developing fruits (**A–D***Amith F0055*). Photos by M. Gorostiza Salazar.

####### Distribution.

**Figure 29.**
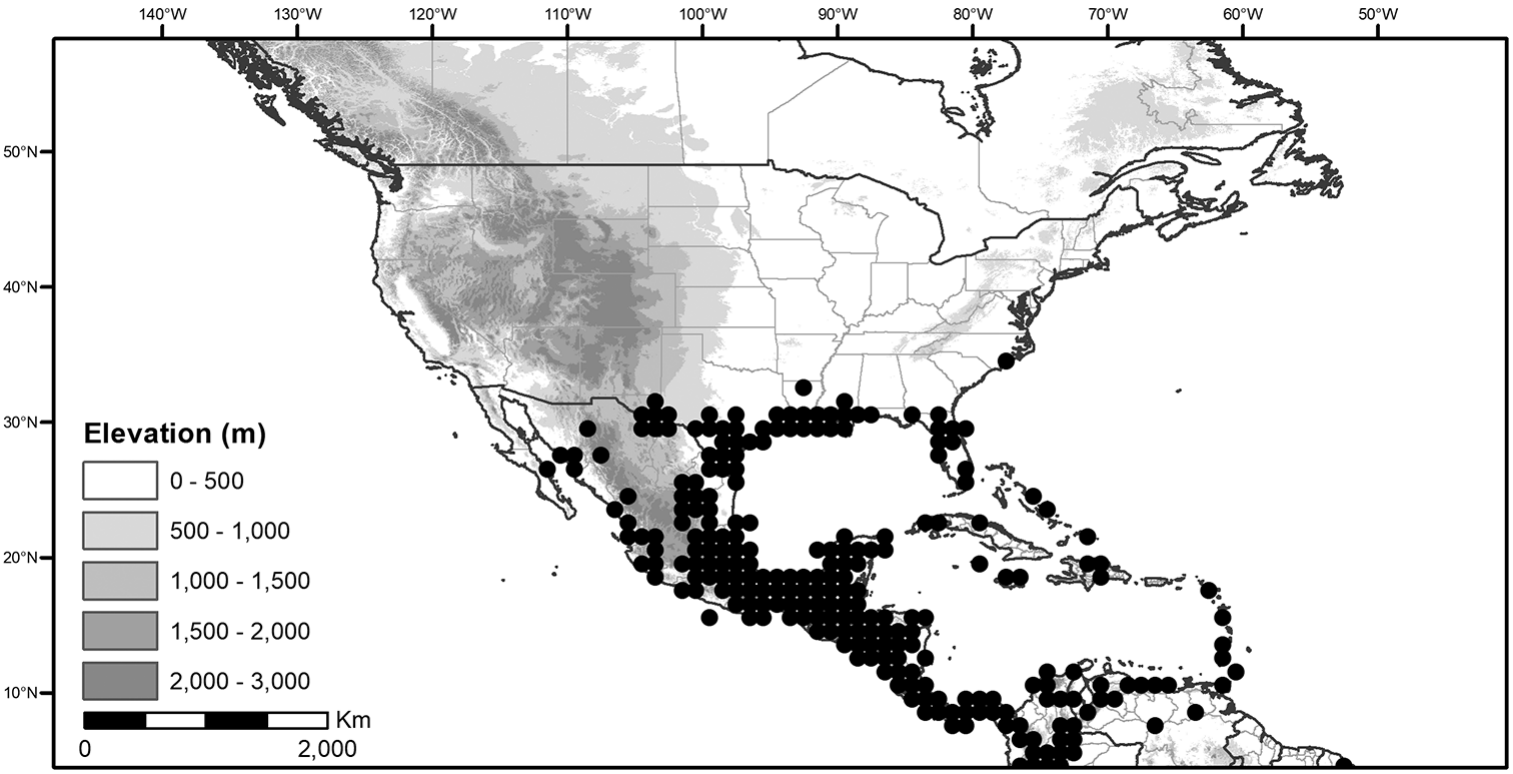
Distribution of *Solanumnigrescens* M.Martens & Galeotti (points in northern South America included to show continuous distribution).

(Figure 29) *Solanumnigrescens* is a widespread species ranging from the southeastern United States of America through Central America, northern South America, and the Caribbean; in the southeastern United States of America it is found along the Gulf Coast and slightly inland but does not extend to the Great Plains.

####### Ecology.

*Solanumnigrescens* is most commonly collected from open areas in cloud forests, deciduous forests and pine forests between sea level and 3,000 m elevation in the region, but most common at lower elevations (ca. 1500 m) in Central America.

####### Common names.

United States of America. Divine nightshade (NatureServe 2017, although the record of this species from Hawaii is certainly in error). Mexico and Central America. Hierba (yerba) mora (many sources). Mexico [Campeche] Chilillo (*Chan 6138*), [Chiapas] Cha’uku (Lacandon, *Lévy & Durán 253*), Ch’il wamal (Tzeltal, *López Pérez 332*), Moen (moem, mu’em) (Tzeltal, *Isidro V. 764*, *Mendez Ton 4546*, *6419*, *Reyes-García et al. 7431*, *Shilom Ton 7625*), Moral wama (Tzeltal, *Gómez López 309*), Mu Itaj (Tzotzil, *Pérez Gómez 159*), Tukumal ejal (Tzotzil, *Pérez Gómez 76*), [Durango] Capulín (*González 1410*), [Guerrero] Yao narambo (*Wagenbreth 120*), yuwa tii (*Avila 52*), [Hidalgo] Tomaquilit (*Villa 48*), [Michoacán] Hierba de golpe (*Hinton 5709*), [Oaxaca] Bishate (bishte, bisnate) (*Elorsa C. 27, 4724*), Bzat (Zapotec, *Hunn OAX-838*), Mahuán (Chinantec, *Martínez Calderón 778*), Pchfuzch-yaas (Zapotec, *Hunn OAX-1056*), Pipitzco (*Macias Acevedo 13*), Quizh-jpchuux-las (Zapotec, *Hunn 1849*), Tomatal montes (*Zarate Marcos 546*), Tonchichi (*Camacho 20*), [Puebla] Barbechos (*Solís M. 4904*), Quelite de jitomate (*Jiménez Chimil JDA-30419*), Teconchichi (*Tlapa & Ubierna 162*, *232*), Tomalkilit (Nahuat, *Ledesma et al. JDA-20153*), [Sonora] Chichiquelite (*Felger et al. 1361*), Mamya (Yaqui, *Felger et al. 1361*), Mombia (mambia) (Mayo, *Van Devender et al. 93-1012*), [Yucatán] Berenjena xiu (*Enríquez 315*), Chilillo (*Góngora 137*), Ik koox (*Ucán Ek 4360*), [Veracruz] Mustulúk (Totonaco, *Cortés 156*), Tomatito de sabana (*Murrieta 56*). Guatemala. Ix ch’yauk’ (Mopan Maya, *Ventur 27*), Macuy (*Ramírez & García 397*). Panama. Kabur gi (Kuna Yala, *de Nevers et al. 7484*).

####### Uses.

Leaves widely used a potherb (“quelite”) in Mexico and Central America.

####### Preliminary conservation status (IUCN 2017).

Least Concern (LC). *Solanumnigrescens* is widespread and weedy in the southern United States, throughout Mexico and Central America and in the Caribbean; it also occurs in northern South America. It has been registered as a noxious weed of agriculture in Louisiana (Orgeron et al. 2018). For EOO see Table 6.

####### Discussion.

*Solanumnigrescens* is one of the commonest and most widely distributed of all morelloid species in Central America of America and the Caribbean. It is very variable morphologically, perhaps due to its wide ecological tolerance and occurrence in many different habitats. It is sympatric or occurs parapatrically with *S.americanum*, *S.douglasii* (in Mexico), *S.interius* and *S.pseudogracile*. It may hybridize with *S.americanum* in the southeastern United States (see discussion under *S.americanum*). Distinguishing features of each of those taxa can be found in the discussions of those species. *Solanumnigrescens* is a perennial and has been reported to be epiphytic (D’Arcy 1974a, b). Where it and *S.americanum* occur in sympatry, the matte berries with appressed to spreading calyx lobes of *S.nigrescens* are distinct from the shiny berries with strongly reflexed tiny calyx lobes of *S.americanum*; anther length also differs (0.7–1.5 mm in *S.americanum* versus 2–2.8(3) mm in *S.nigrescens*). In central Mexico, were *S.nigrescens* and *S.douglasii* co-occur, anther length (3–4 mm in *S.douglasii* versus 1.8–2.5 mm in *S.nigrescens*) is a good distinguishing feature; in fruit, these two taxa can be almost impossible to tell apart. Nee (1993) considered *S.nigrescens* to occupy wetter forest types than does *S.douglasii* but did not have not enough material from Veracruz to make the distinction. We find that the two species occur in very similar habitats, but that *S.nigrescens* is a more Caribbean species on the eastern side of the Sierra Madre and around the Gulf of Mexico, while *S.douglasii*occurs along the Pacific coast and into central Mexico, but also does occur in the Chihuahuan Desert biome. Specimens from Quintana Roo identified as *S.nigrum* in Sousa and Cabrera (1983) are *S.nigrescens* (*Cabrera 875*, *1151*). Like most of these morelloid species, it is very weedy and occupies a wide range of disturbed and undisturbed habitats.

In the southeastern United States (e.g., Texas) the distributions of *S.nigrescens* and *S.interius* are very close if not interdigitating. *Solanumnigrescens* can be distinguished from *S.interius* in its smaller seeds, more numerous stone cells in the berry and usually acute calyx lobe apices. The unusual pedicel articulation of the basal flower (in the lower third of the pedicel) in the inflorescences of *S.interius* has not been seen in *S.nigrescens*. *Solanumnigrescens* also appears to occur in more mesic and coastal habitats than *S.interius*, which is a species of the Great Plains.

Material identified as *S.americanum* by Manoko et al. (2007) represents specimens of *S.nigrescens* (see Särkinen et al. 2018: 61).

Bitter (1914) reported large numbers of stone cells in the berries of many of the names we consider synonyms of *S.nigrescens*. In general, *S.nigrescens* has more stone cells in its berries than does the similar *S.douglasii*, but these can be difficult to see as some of them are very tiny.

Dunal (1852) cited several specimens in describing S.nodiflorumvar.puberulum, all from the Candolle herbarium at G: “Carolina meridionali, *Fraser*”, “Florida, *Mich.f.*”, “Mexico circa Bejar, *Berlandier 1904*” and “China, *Staunton*”. Of these, the Fraser collection is a mixed collection composed of two tiny fragments of *S.nigrescens* (G00144217) and one larger fragment of *Capsicumannuum* L. (G00144268), mounted on the same sheet is a tiny fragment of *S.americanum* attributed to Michaux filius (G00144264), and the Staunton sheet (G00144265) is of a plant of *S.americanum*. Edmonds inadvertently (see Prado et al. 2015) selected as the lectotype for this name the sheet of *Berlandier 1904* held in G-DC (G00144231) by citing it as “holotype”; this is fortunate because it is unambiguous, was cited by Dunal (1852), and has duplicated in several other herbaria. When Berlandier was collecting, southern Texas was part of Mexico, and Bejar was the name for what today is San Antonio, the capital of Bexar County.

D’Arcy (1974a) lectotypified *Solanumcaribaeum* citing a specimen from Jamaica in “G-DC ex Kew”. In the protologue of *S.caribaeum*Dunal (1852) cited “In insulis Caribaeis, Jamaica, Guadalupâ (ex h. DC)” suggesting that more than one specimen was consulted. In G-DC there is a single specimen with the label “S. caribearum Nob. 1835” (G00144199) that is the only element of unambiguous material we have seen, and we designate this sheet in a second stage lectotypification. It is clear that more than this single sheet was used in the preparing the description, G00144199 has only fruits and the description has details of both flowers and fruits.

Kuntze (1891) equated his S.nigrumvar.amethystinum with “S.nigrum subsp. genuinum Sendtn.” a name not validly published (see Särkinen et al. 2018) and distinguished it by its violet flowers. He did not explicitly cite specimens but did cite the locality “Costa Rica. Irazu”. We have selected the specimen in Kuntze’s herbarium held at NY (NY00688134) as the neotype for this name.

Both *S.prionopterum* (Bitter 1912a) and *S.gollmeri* (Bitter 1912b) were described from material collected by J. Gollmer around Caracas, in two separate publications. It is likely that the seed collection grown in Berlin in 1859 that was used to describe *S.gollmeri* was derived from the same material collected in 1854 in Venezuela. The holotypes in B for both these names were destroyed; the photograph of the holotype of *S.gollmeri* (F neg. 2689) has a tiny leaf fragment attached, but no such material is associated with the photograph taken of the holotype of *S.prionopterum* (F neg. 2699). We designate this fragment (F-621268) as the lectotype of *S.gollmeri*; it is not, however, original material for *S.prionopterum*, and we hope a duplicate of the Gollmer collection from 1854 used to describe *S.prionopterum* will eventually be found.

Bitter (1913) described S.pruinosumvar.phyllolophum from living material originally sent by David Fairchild of the USDA as No. 32065; he cited no specimens. In the Germplasm Resources Information Network of the USDA (USDA 2017) the germplasm accession PI-32065 is recorded as “S.nigrum” collected by C.A. Purpus in Puebla, Mexico. Fairchild (1912) recorded the exact locality as “Esperanza, Puebla, 2,700 m [9,850 feet]”. We have not yet seen a collection made by Carl Purpus with this exact information, hence do not designate a neotype until we have searched more exhaustively for a collection with the correct locality. It may be Purpus only collected seeds, and not herbarium specimens.

Bitter (1913) described *S.subelineatum* from living material original sent from the USDA as No. 32067 grown in the Bremen botanical garden; he cited no specimens. In the Germplasm Resources Information Network of the USDA (USDA 2017) the germplasm accession PI-32067 is recorded as “S.nigrum” collected by C.A. Purpus in San Luis Potosí, Mexico. Fairchild (1913) recorded the exact locality as “Rascon, San Luis Potosí, 400 to 500 m [1,300 to 1,650 feet]”. As with S.pruinosumvar.phyllolophum we refrain from lectotypifying this anticipating encountering a duplicate.

In describing *S.oligospermum*Bitter (1913) cited two specimens of *Pringle 4948* at “herb. Haussknecht.!, Turic.!” (today JE and Z). Edmonds (1972) inadvertently lectotypified this name with the Z sheet by stating “(Z holotype!)” (see Prado et al. 2015). We here specify the individual sheet (Z000033841) in that herbarium that is the lectotype. There are many well-preserved duplicates of this collection (see synonymy), all with flowers and fruit.

*Solanumapproximatum*, *S.durangoense*, and *S.purpuratum* were described (Bitter 1913) from B sheets that are now destroyed. We have selected lectotypes for these names from the large number of extant duplicates, using the best-preserved sheets with both flowers and fruits (if possible).

Many specimens of *S.nigrescens* from Venezuela in US were annotated as “*S.jahnii* Bitter” by C.V. Morton, a designation not validly published (nomen nudum) based on *Jahn 588*. That collection corresponds to *S.interandinum* Bitter, a taxon not known from Central America or Mexico.

Heiser (1955) cited only IND as the type in the protologue of *S.costaricense*, two sheets in IND are labelled “Type”. The sheets are clearly labelled as “sheet 1” and “sheet 2” and we interpret them as a two-sheet holotype (see Turland et al. 2018, Art. 8, Ex. 7). IND-1000068 is the better material, with flowers and complete vegetative material.

####### Specimens examined.

See Suppl. materials 1 and 3.

###### 10. Solanum nigrum

Taxon classificationPlantaeSolanalesSolanaceae

L., Sp. Pl. 1: 186. 1753

Figures 30 , 31

Solanum peregrinum E.P.Bicknell, Bull. Torrey Bot. Club 42: 332. 1915. Type. United States of America. Massachusetts: Nantucket County, Nantucket street, *E.P. Bricknell 7719* (holotype: NY [NY00138955]; isotype: NY [NY00073847]).

####### Type.^3^

“Habitat in Orbis totius cultis” [sheet marked with Θ, meaning central part of Asia = Middle East], *Without collector s.n.* (lectotype, designated by Henderson 1974, pg. 19: LINN [LINN 248-18]).

####### Description.

Annual or short-lived perennial herbs to 1.0 m tall, branching 10–30 cm from the base. Stems terete to sharply angled and ridged, green, the ridges often spinescent, not markedly hollow; new growth sparsely to densely pubescent with simple, spreading, uniseriate 1–6-celled trichomes 0.5–0.6 mm long, these eglandular and/or glandular; older stems glabrescent, the trichome bases persisting as pseudospines. Sympodial units difoliate, the leaves not geminate. Leaves simple, 3.8–7.2(–14.5) cm long, 2.5–5.0(–9.5) cm wide, broadly ovate, green; adaxial surface sparsely pubescent with spreading, simple, uniseriate trichomes like those on stem evenly scattered along veins and lamina; abaxial surface more densely pubescent along veins and sparsely along lamina with eglandular and/or glandular trichomes like those of the stems; major veins 5–7 pairs; base obtuse to truncate, somewhat attenuate; margins sinuate-dentate, especially in the lower 2/3, to occasionally entire or deeply toothed; apex acute; petioles 0.5–3.0 cm long, pubescent with simple uniseriate glandular and eglandular trichomes like those of the stems. Inflorescences 0.8–2.0 cm long, lateral, internodal, unbranched (occasionally forked), with (3-)4–10 flowers spaced along the rhachis, pubescent with spreading simple uniseriate trichomes like those on stem; peduncle 0.5–1.5 cm long, straight; pedicels 3–5 mm long, 0.2–0.3 mm in diameter at the base and 0.2–0.3 mm at the apex, spreading, articulated at the base; pedicel scars spaced 0.3–0.7 mm apart. Buds subglobose, the corolla approximately halfway exserted from the calyx before anthesis. Flowers 5-merous, all perfect. Calyx tube 0.8–1.0 mm long, the lobes 0.5–0.8 mm long, 0.6–0.8 mm wide, triangular with acute or somewhat rounded apices, pubescent with spreading simple uniseriate eglandular and glandular trichomes like those of the pedicels. Corolla 10–12 mm in diameter, white with a yellow-green central portion near the base, stellate, lobed 1/2 to 2/3 of the way to the base, the lobes 4.0–5.0 mm long, 2.0–2.5 mm wide, strongly reflexed at anthesis, later spreading, densely papillate-pubescent abaxially with simple uniseriate eglandular trichomes. Stamens equal; filament tube very short to minute; free portion of the filaments 0.5–0.7 mm long, adaxially pubescent with spreading uniseriate simple trichomes; anthers 1.8–2.5 mm long, 0.8–1.0 mm wide, ellipsoid, very slightly wider at base, yellow, poricidal at the tips, the pores lengthening to slits with age and drying. Ovary globose, glabrous; style 2.5–3.5 mm long, densely pubescent with tangled 2–3-celled simple uniseriate trichomes in the lower half where included in the anther cone, exserted 0–1 mm beyond anther cone; stigma capitate, minutely papillate, green in live plants. Fruit a globose berry, 6–10 mm in diameter, purple-black or green to yellowish green at maturity, opaque, the surface of the pericarp matte or slightly shiny; fruiting pedicels 10–12 mm long, 0.4–0.5 mm in diameter at the base, 1.0–1.1 mm in diameter at the apex, generally spreading to occasionally recurved, spaced 1.0–2.0 mm apart, dropping with mature fruits, usually not persistent but occasionally remaining on the rhachis; fruiting calyx not accrescent, tube 0.7–1.5 mm long, the lobes 1.0–2.0 mm long, spreading to reflexed in fruit. Seeds (15-)20–40 per berry, 1.8–2.0 mm long, 1.5–1.6 mm wide, flattened and tear-drop shaped with a subapical hilum, yellow, the surfaces minutely pitted, the testal cells pentagonal in outline. Stone cells absent (North America and Europe) but usually 2(-8) per berry in other areas (Asia), ca. 0.5 mm in diameter, brown. Chromosome number: *2n*=6×=72 (for vouchers and references see Särkinen et al. 2018).

**Figure 30.**
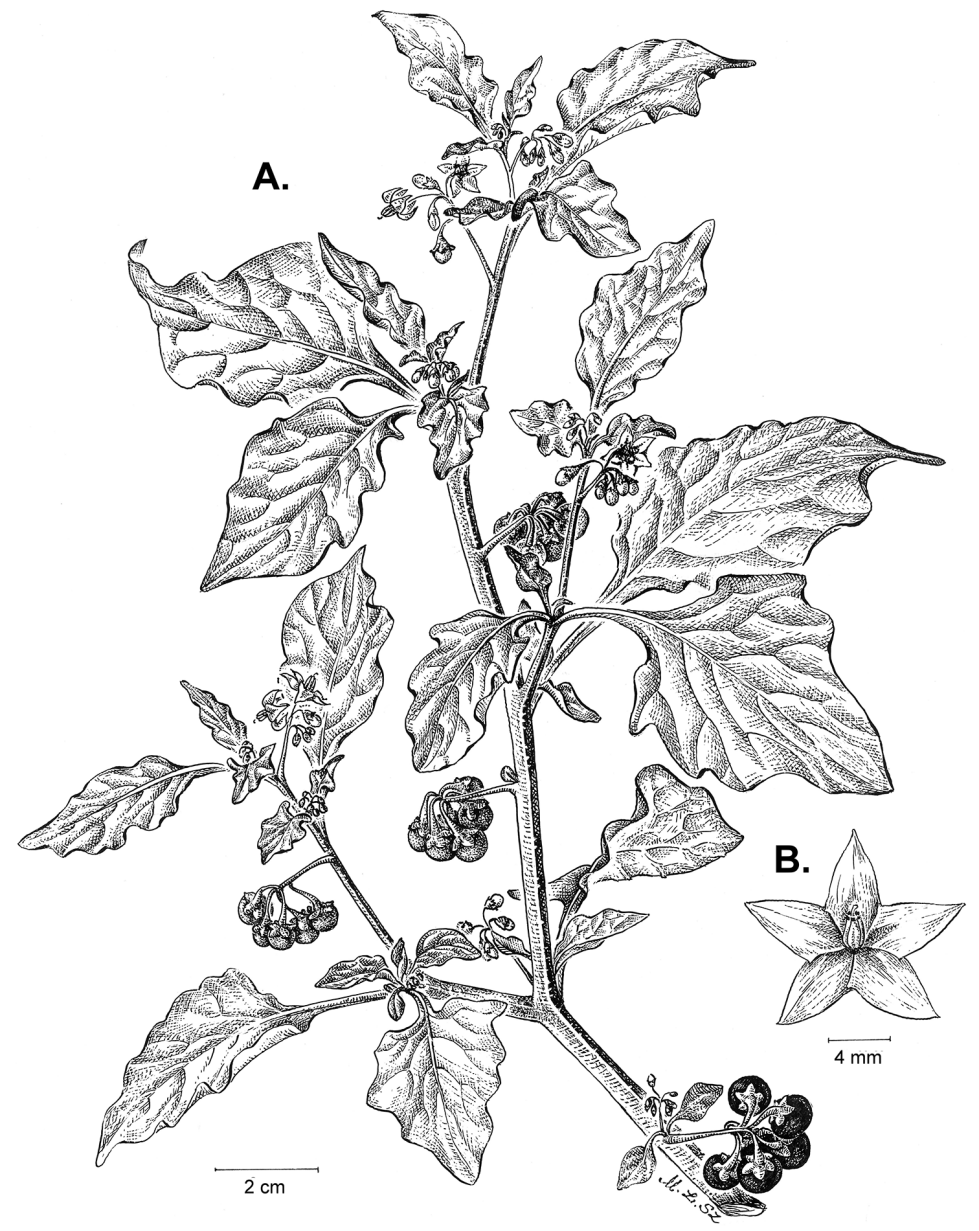
*Solanumnigrum* L. **A** Habit **B** flower (**A ,B***Symon 5449* [ADW 35964]). Drawing by M.L. Szent-Ivany, first published in Symon (1981), courtesy of the Board of the Botanic Gardens and State Herbarium (Adelaide, South Australia), reproduced with permission (previously also published in “PhytoKeys 106”).

**Figure 31.**
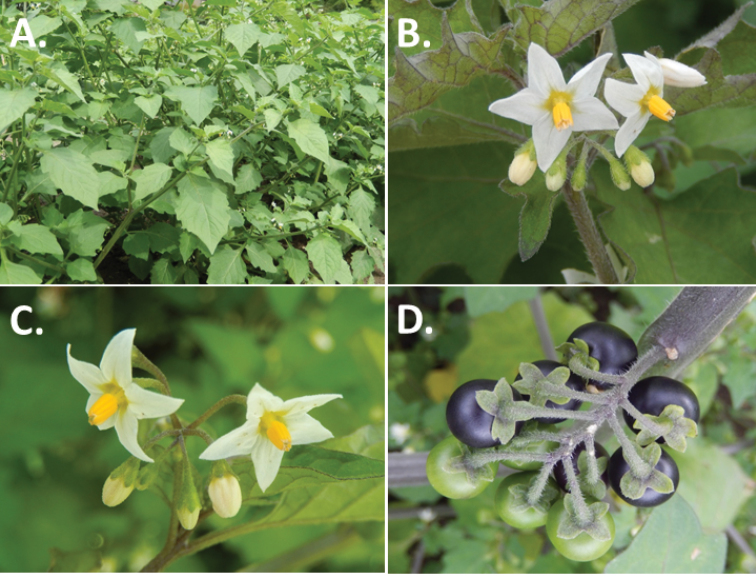
*Solanumnigrum* L. **A** Habit **B** inflorescence (dense indumentum) **C** inflorescence (sparse indumentum) **D** fully mature full-black berries, calyx lobes remaining appressed or slightly spreading (**A** Nijmegen acc. 824750016 **B** Nijmegen acc. A34750479 **C** Nijmegen accession 824750029A **D** Nijmegen accession A44750150). Photos by S. Knapp (previously published in “PhytoKeys 106”).

####### Distribution.

**Figure 32.**
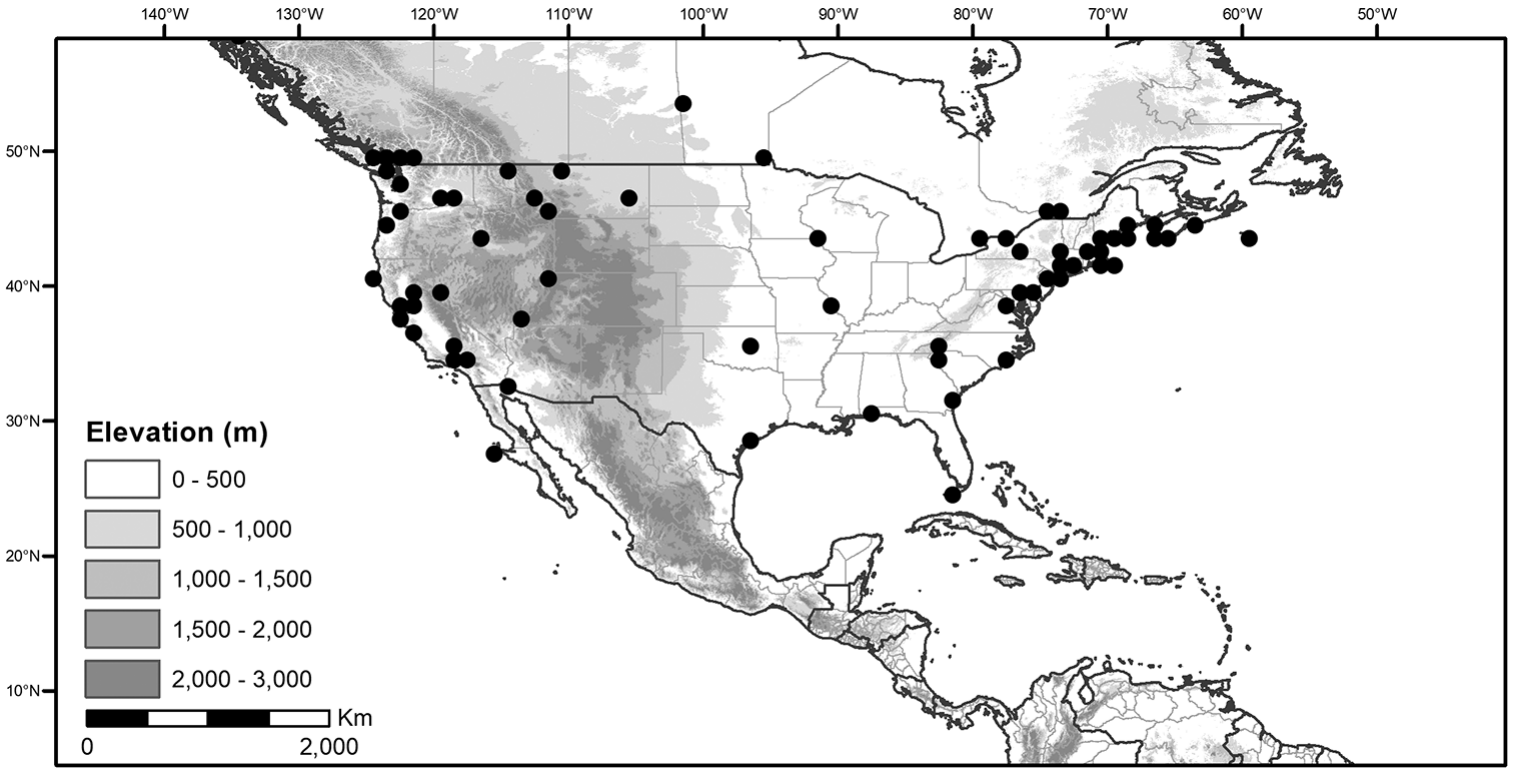
Distribution of *Solanumnigrum* L.

(Figure 32) *Solanumnigrum* is native to Eurasia and has been sporadically introduced and locally naturalised in temperate North America. It is possible that populations from eastern and western areas have different origins (see below).

####### Ecology.

The species is found in disturbed areas between 0–2,200 m elevation in its native range, but around cities and cultivated fields from sea level to 700 m in North America.

####### Common names.

Canada. Black nightshade, morelle noire (Alex et al. 1980; Bassett and Munro 1985). United States of America. Black nightshade (many sources).

####### Uses.

None recorded for the region (see Särkinen et al. 2018 for uses in its native range).

####### Preliminary conservation status (IUCN 2017).

Least Concern (LC). See Särkinen et al. 2018.

####### Discussion.

*Solanumnigrum* is probably introduced in temperate North America; populations on the eastern seaboard best match European populations in overall morphology. Populations from the western coast (e.g., British Colombia) are morphologically more similar to Eurasian plants and it is possible that they are the result either of introductions from that region over the Pacific or are relictual native plants that came across the Bering Straits during warm periods in the Quaternary (e.g., DeChaine 2008; Ickert-Bond et al. 2009). Molecular population genetic analysis of these populations has not been done but should shed some light on the status of *S.nigrum* in North America.

*Solanumnigrum* can be distinguished from other North American species (e.g., *S.americanum, S.douglasii*, *S.emulans*, *S.interius*, *S.nigrescens*) in the character combination of thicker peduncles and pedicels, larger seeds and fruits lacking stone cells. It has longer anthers (2.5–3 mm) than *S.emulans* and *S.americanum*, both of which have tiny anthers ca. 1.5 mm long. Like *S.nigrescens*, it has inflorescences with the flowers spaced along the rhachis, but *S.nigrescens* has prominent stone cells in the berries and smaller seeds. *Solanuminterius* has similarly large seeds but has fewer flowers per inflorescence and distinctive basal flower pedicel position (articulation above the join with the rhachis).

Michael Nee (pers. comm.) has observed its spread and increase in the New York City area over the last decade; it is possible that more collections will be made throughout North America in the coming years.

For typification details of the many synonyms of *S.nigrum* see Särkinen et al. (2018).

####### Specimens examined.

See Suppl. materials 1 and 3.

###### 11. Solanum nitidibaccatum

Taxon classificationPlantaeSolanalesSolanaceae

Bitter, Repert. Spec. Nov. Regni Veg. 11: 208. 1912

Figures 33 , 34

Solanum styleanum Dunal, Prodr. [A. P. de Candolle] 13(1): 44. 1852. Type. Chile. Sin. loc., *J. Styles s.n.* (holotype: G-DC [G00144016]).
Bosleria nevadensis A.Nelson, Proc. Biol. Soc. Washington 18(30): 175. 1905. Type. United States of America. Nevada: Washoe County, Pyramid Lake, 9 Jun 1903, *G.H. True s.n.* (holotype: RM [RM0004387]).
Solanum patagonicum C.V.Morton, Revis. Argentine Sp. Solanum 146. 1976. Type. Chile. Región XII (Magallanes): Río Paine, 100m, 15 Jan 1931, *A. Donat 415* (holotype: BM [BM000617673]; isotypes: BA, BAF, GH [GH00077732], K, SI [SI003331, SI003332], US [US00027733, acc. # 2639758]).
Solanum physalifolium Rusby var. nitidibaccatum (Bitter) Edmonds, Bot. J. Linn. Soc. 92: 27. 1986. Type. Based on Solanumnitidibaccatum Bitter

####### Type.^4^

Chile. Sin. loc., 1829, *E.F. Poeppig s.n.* (lectotype, designated by Edmonds 1986, pg. 27: W [acc. # 0004151]; isolectotype: F [v0073346F, acc. # 875221]).

####### Description.

Annual herbs to 20 cm tall, prostrate and spreading to 30 cm in diameter or more. Stems terete, green, not markedly hollow; new growth densely viscid-pubescent with translucent simple, uniseriate 2–8(10)-celled spreading trichomes 1.5–2.0 mm long with a glandular apical cell; older stems glabrescent. Sympodial units difoliate, the leaves not geminate. Leaves simple, 2.0–5.5(–9.5) cm long, 1.5–5.0 (–6.5) cm wide, ovate to broadly ovate, rarely elliptic; adaxial surface sparsely pubescent with spreading 2–4-celled translucent, simple, uniseriate gland-tipped trichomes like those of the stem, these denser along the veins; abaxial surface more evenly densely pubescent on the lamina and veins; major veins 3–6 pairs, not clearly evident abaxially; base attenuate to cuneate, at times asymmetric, decurrent on the petiole; margins entire or sinuate-dentate; apex acute to obtuse; petioles 0.5–2.7(–4.5) cm long, sparsely pubescent with simple uniseriate glandular trichomes like those of the stems and leaves. Inflorescences 1.0–2.0 cm long, lateral, generally internodal but in new growth appearing leaf-opposed, unbranched, with 4–8(-10) flowers clustered at the tip (sub-umbelliform) or spread along a short rhachis, sparsely pubescent with spreading trichomes like those on stems and leaves; peduncle 0.6–1.3 cm long; pedicels 4–12 mm long, 0.1–0.2 mm in diameter at the base and 0.2–0.4 mm in diameter at the apex, straight and spreading, articulated at the base; pedicel scars spaced 0.3–1 mm apart. Buds subglobose, the corolla only slightly exserted from the calyx tube before anthesis. Flowers 5-merous, all perfect. Calyx tube 1–2 mm long, conical, the lobes 1.7–2.5 mm long, less than 1 mm wide, triangular with acute to obtuse apices, sparsely pubescent with 1–4-celled glandular trichomes like those of the pedicels. Corolla 4–6 mm in diameter, white with a yellow-green central eye with black “V” or “U” shaped edges in the lobe sinuses, rotate-stellate, lobed 1/3 of the way to the base, the lobes 2.3–3.2 mm long, 2.5–3.7 mm wide, spreading at anthesis, sparsely papillate-pubescent abaxially with 1–4-celled simple uniseriate trichomes, especially along tips and midvein. Stamens equal; filament tube minute; free portion of the filaments 1.5–2.0 mm long, adaxially sparsely pubescent with tangled uniseriate 4–6-celled simple trichomes; anthers 1.0–1.4 mm long, 0.5–0.8 mm wide, ellipsoid, yellow, poricidal at the tips, the pores lengthening to slits with age and drying. Ovary globose, glabrous; style 2.5–3.0 mm long, densely pubescent with 2–3-celled simple uniseriate trichomes in the lower half where included in the anther cone, exserted 0.2–1.0 mm beyond the anther cone; stigma capitate, minutely papillate, green in live plants. Fruit a globose berry, 4–13 mm in diameter, brownish green and marbled with white (this not easily visible in herbarium specimens) at maturity, translucent, the surface of the pericarp usually shiny; fruiting pedicels 4–13 mm long, ca. 0.2 mm in diameter at the base, spaced 1–3 mm apart, reflexed and slightly curving, dropping with mature fruits, not persistent; fruiting calyx accrescent, becoming papery in mature fruit, the tube ca. 3mm long, the lobes 2.5–3.5(–4.0) mm long and 3–4 mm wide, appressed against the berry, but the berry clearly visible. Seeds 13–24 per berry, 2.0–2.2 mm long, 1.2–1.4 mm wide, flattened and tear-drop shaped with a subapical hilum, brown, the surfaces minutely pitted, the testal cells pentagonal in outline. Stone cells usually (1-)2–3 per berry, occasionally absent, ca. 0.5 mm in diameter. Chromosome number: 2*n*=2×=24 (see Särkinen et al. 2018).

**Figure 33.**
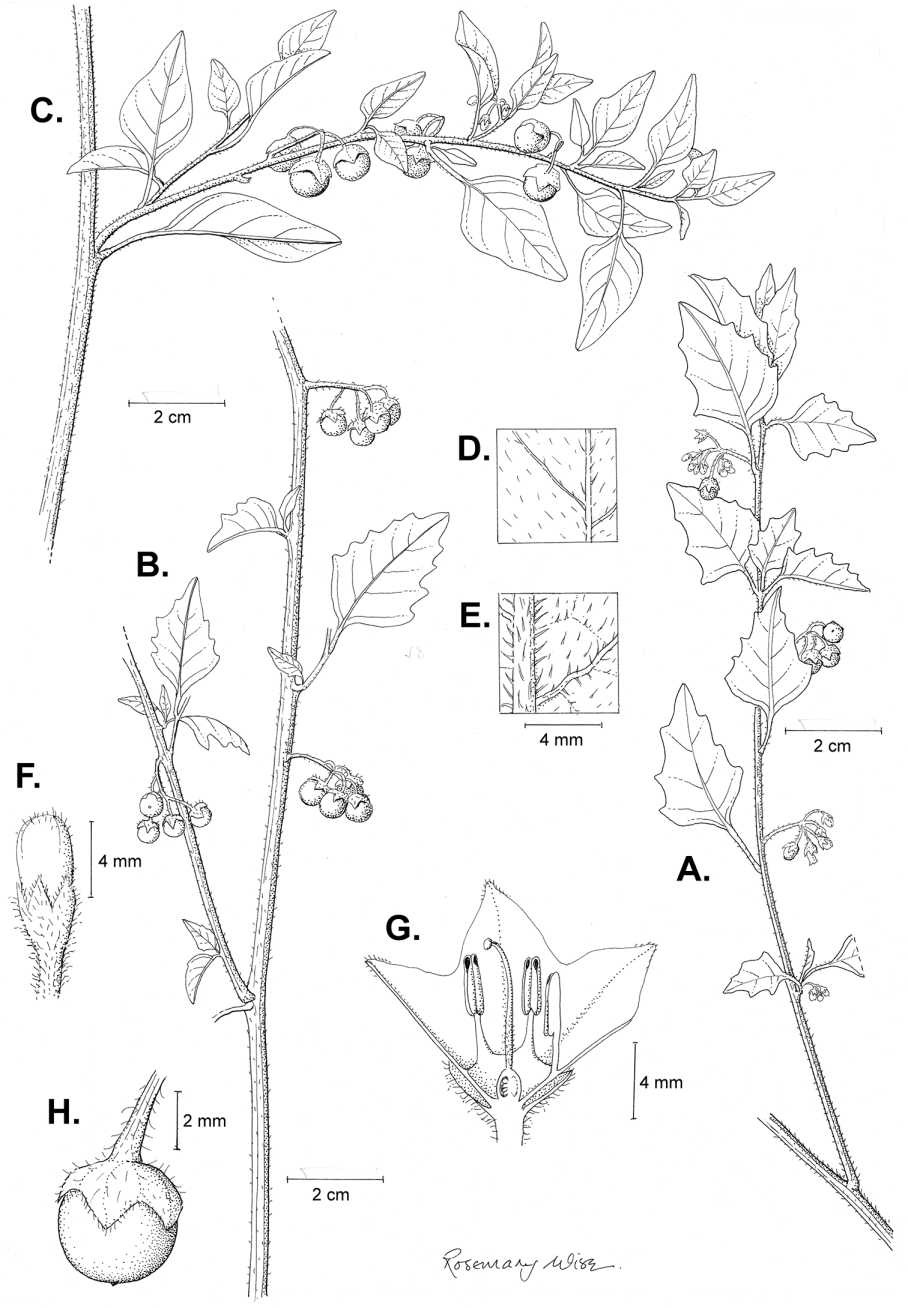
*Solanumnitidibaccatum* Bitter. **A** Habit **B** fruiting habit **C** fruiting habit showing leaf variation **D** detail of adaxial leaf surface **E** detail of abaxial leaf surface **F** bud **G** dissected flower **H** fruit (**A, C, F***Henning 14*; **B, D–E, H***Blake 186*; **C***Arnow 740*). Drawing by R. Wise (previously published in “PhytoKeys 106”).

**Figure 34.**
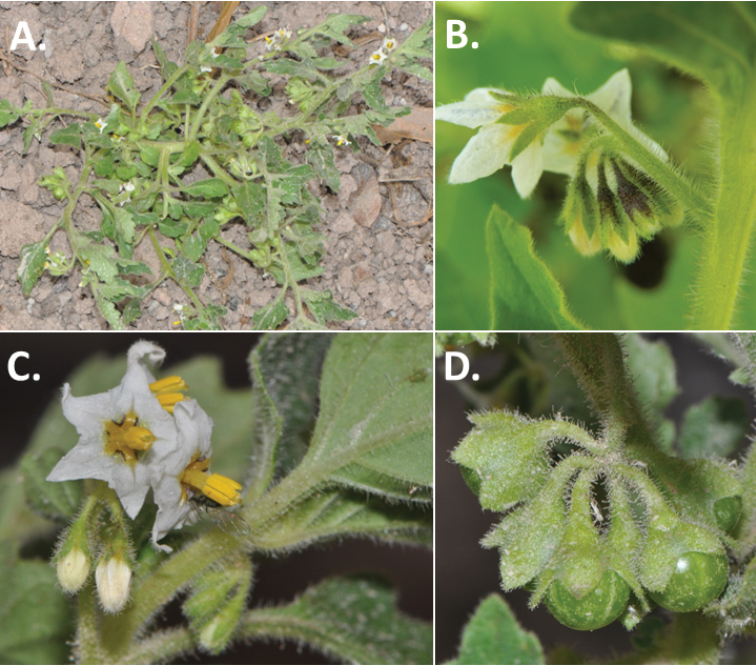
*Solanumnitidibaccatum* Bitter. **A** Habit **B** young inflorescence with flower buds **C** flowers at anthesis **D** maturing fruits (**A–D***Särkinen et al. 4076*). Photos by T. Särkinen (previously published in “PhytoKeys 106”).

####### Distribution.

**Figure 35.**
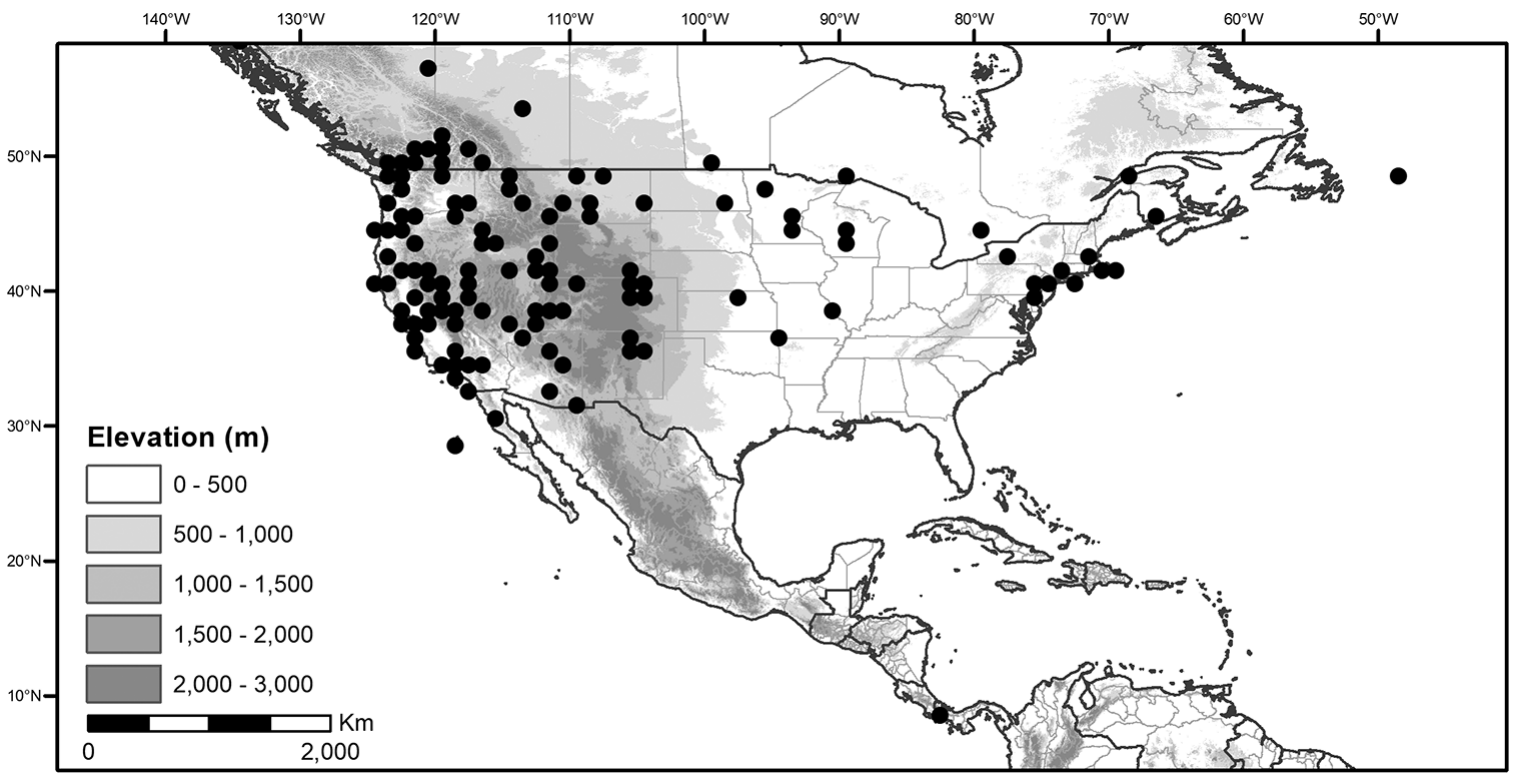
Distribution of *Solanumnitidibaccatum* Bitter.

(Figure 35) *Solanumnitidibaccatum* has an amphitropical distribution in temperate South America and temperate western North America, including northern Baja California. A single collection (*Hammel et al. 6964*) is known from the high elevation regions of Chiriquí, Panama (D’Arcy 1987). *Solanumnitidibaccatum* has often been recorded as adventive in North America, but the large number of early herbarium collections far from ports of entry suggest it is native (see also *S.triflorum*) at least from the Rocky Mountains westwards.

####### Ecology.

*Solanumnitidibaccatum* is a disturbance loving species, usually found growing along roadsides in the shade of trees and shrubs, and in rocky and sandy soil between (0-)1,200 and 2,500 m elevation. It is a common weed of agriculture and is often found growing in sandy soil in seasonal washes (arroyos).

####### Common names.

Canada. Hairy nightshade, morelle poilu (Alex et al. 1980, as *S.sarrachoides*). United States of America. Ground-cherry nightshade (NatureServe 2017, as *S.physalifolium*), Hairy nightshade (Mohlenbrock 2014), Hoe nightshade (USDA Plants 2017, as *S.physalifolium*), “ah-dye-ee na-tizuah” (Paiute, Train et al. 1941, as *S.villosum*).

####### Uses.

The fruit were used either ripe or in a decoction as a cure for diarrhoea by the Paiute people of Nevada (Train et al. 1941, as *S.villosum*); leaves and berries were soaked in water and applied to watermelon seeds to ensure a good crop by the Navajo (Moerman 1998, as *S.physalifolium*). Train et al. (1941) state that the Paiute people of Nevada “used a tea made from the berries when traveling in areas where the water was not potable”.

####### Preliminary conservation status (IUCN 2017).

Least Concern (LC). *Solanumnitidibaccatum* is widespread and weedy especially in the southwestern United States of America and the Great Plains; it also occurs in southern South America. For EOO see Table 6.

####### Discussion.

*Solanumnitidibaccatum* is morphologically similar to and has been treated as *S.sarrachoides* in most previous treatments of North American morelloids (e.g., Schilling 1981; Schilling and Heiser 1979); it is also often identified as *S.physalifolium* Rusby. Edmonds (1986) showed that *S.nitidibaccatum* and *S.sarrachoides* were distinct morphologically, and phylogenetic results (Särkinen et al. 2015b) confirm this; molecular sequence data also show these two taxa are not closely related despite their overall similarity. *Solanumnitidibaccatum* has also sometimes been treated at subspecific rank within *S.physalifolium*, an Andean endemic (see Särkinen and Knapp 2016), but the species are distinct although preliminary data suggest they are closely related (see Särkinen and Knapp 2016). *Solanumnitidibaccatum* is usually thought to be native to the southeastern parts of South America, from which it has been introduced extensively to other parts of the world where it has become a prolific and successful weed of disturbed sites. The species is locally abundant throughout North America (Ogg et al.1981) and is perhaps native there west of the Rockies (see Distribution above).

*Solanumnitidibaccatum* can be distinguished from *S.sarrachoides* in its shorter, plumper anthers, the blackish purple markings in the centre of the corolla on the margins of the central star, and in its fruits that are shiny at maturity, marbled with white (not usually visible on herbarium sheets) and not completely enclosed in the accrescent calyx. In addition, the mature inflorescences of *S.nitidibaccatum* are always internodal while those of *S.sarrachoides* are usually leaf-opposed.

Details of typification of the synonyms of *S.nitidibaccatum* can be found in Barboza et al. (2013) and Särkinen et al. (2018).

####### Specimens examined.

See Suppl. materials 1 and 3.

###### 12. Solanum pruinosum

Taxon classificationPlantaeSolanalesSolanaceae

Dunal, Prodr. [A. P. de Candolle] 13(1): 58. 1852

Figures 36 , 37

Solanum dasyadenium Bitter, Repert. Spec. Nov. Regni Veg. 11: 8. 1912. Type. Mexico. Sin.loc., *J. Schaffner 655* (syntype, B destroyed); *C.A. Uhde 80* (syntype, B destroyed; dups maybe at Halle?).
Solanum dasyadenium subsp. uberius Bitter, Repert. Spec. Nov. Regni Veg. 11: 9. 1912. Type. Mexico. Sin.loc., *A. Aschenborn 412* (syntype B, destroyed); *A. Aschenborn 413* (syntype B, destroyed).
Solanum dasyadenium subsp. potosanum Bitter, Repert. Spec. Nov. Regni Veg. 11: 9. 1912. Type. Mexico. San Luis Potosí: San Luis Potosí, *J. Schaffner 408* (holotype: B, destroyed; lectotype, designated here: GOET [GOET003496]; isolectotypes: BM [BM000579277], M [M-0183327], NY [NY00751028], P [P00366754], US [US00027536, acc. # 939130]).

####### Type.

Mexico. “Circa Mexico”, *J. Berlandier 751* (holotype: G [G00418346]).

####### Description.

Perennial herb, 0.7–1 m tall, perhaps occasionally annual or only persisting for a few years. Stems angled to winged, lacking spinescent processes, usually erect, but occasionally lax and somewhat scrambling; young stems densely to sparsely pubescent with glandular, simple uniseriate trichomes 0.5–2 mm long, the trichomes (2–)4–15 celled, the basal cells larger, the trichomes drying translucent; new growth densely glandular pubescent and sticky; bark of older stems greenish brown. Sympodial units difoliate, the leaves not geminate. Leaves simple, occasionally shallowly toothed, 2.5–6.5 cm long, 1.2–2.8 cm wide, elliptic to ovate, widest in the lower half, membranous; adaxial and abaxial surfaces evenly and densely glandular-pubescent with simple uniseriate trichomes to 2 mm long, these denser abaxially and along the veins; principal veins 4–6 pairs, drying paler than the lamina; base attenuate onto the petiole; margins entire to shallowly and irregularly toothed, the teeth mostly in the basal third of the blade, usually with minute glandular papillae with 2-celled glandular tips that dry dark brown; apex acute to acuminate; petiole 0.5–2 cm, narrowly winged from the attenuate leaf base. Inflorescences 0.8–2.5 cm long, unbranched, internodal, with 3–6 flowers (usually ca. 4) clustered in the distal third or quarter (sub-umbelliform), densely glandular-pubescent like the stems and leaves; peduncle 0.8–2.5 cm long; pedicels 0.7–0.9 cm long at anthesis, ca. 0.5 mm in diameter at the base, ca. 1 mm in diameter at the apex, slender and tapering, densely glandular-pubescent with short uniseriate trichomes and glandular papillae, with only a few trichomes to 2 mm long present, spreading at anthesis, articulated at the base; pedicels scars closely packed in the distal part of the inflorescence, with the lowermost ca. 1 mm distant from the rest. Buds globose to broadly ellipsoid, the corolla strongly exserted from the calyx tube before anthesis. Flowers 5-merous, all perfect. Calyx tube 1.5–2 mm long, conical to cylindrical, the lobes 0.5–1 mm long, 0.8–1 mm wide, deltate to triangular, the tips obtuse or rounded, densely glandular-pubescent like the pedicels with uniseriate trichomes and papillae. Corolla 10–15 mm in diameter, white or pale purple with a darker brownish purple central star, stellate, lobed 1/2 to 2/3 of the way to the base, the lobes 3.5–5 mm long, 2–3 mm wide, triangular, reflexed to spreading at anthesis, the abaxial surfaces densely papillate, the trichomes not glandular. Stamens equal; filament tube to 0.5 mm; free portion of the filaments 0.5–1 mm long, glabrous or with a few weak tangled simple uniseriate trichomes adaxially; anthers 2.5–3.5 mm long, 0.5–1 mm wide, ellipsoid, bright yellow, poricidal at the tips, the pores elongating to slits with age. Ovary conical, glabrous; style 4.5–6 mm long, sparsely pubescent with weak tangled trichomes to densely papillate in the lower part where included in the anther cone, only slightly (ca. 0.5 mm) exserted from the anther cone; stigma capitate, densely papillate. Fruit a globose berry, 0.5–1 cm in diameter, green to deep purple (red when ripe? *Martínez 1211*); opaque (mature fruits not seen on live plants but not markedly translucent when dry), the pericarp thin, matte; fruiting pedicels 6–9 mm long, enlarging from a base 0.6–1 mm in diameter to an apex 1–1.5 mm in diameter, not distinctly woody, spreading; fruiting calyx not accrescent, the tube less than 1 mm long, the lobes 1.5–2 mm long, appressed to the berry, venation very apparent and thickened. Seeds 10–30 per berry, 1–1.5 mm long, 1–1.2 mm wide, tear-drop shaped, reddish gold, the surfaces minutely putted, the testal cells pentagonal. Stone cells 2–4 (–6) per berry, 0.5–0.7 mm in diameter, pale cream. Chromosome number: not known.

**Figure 36.**
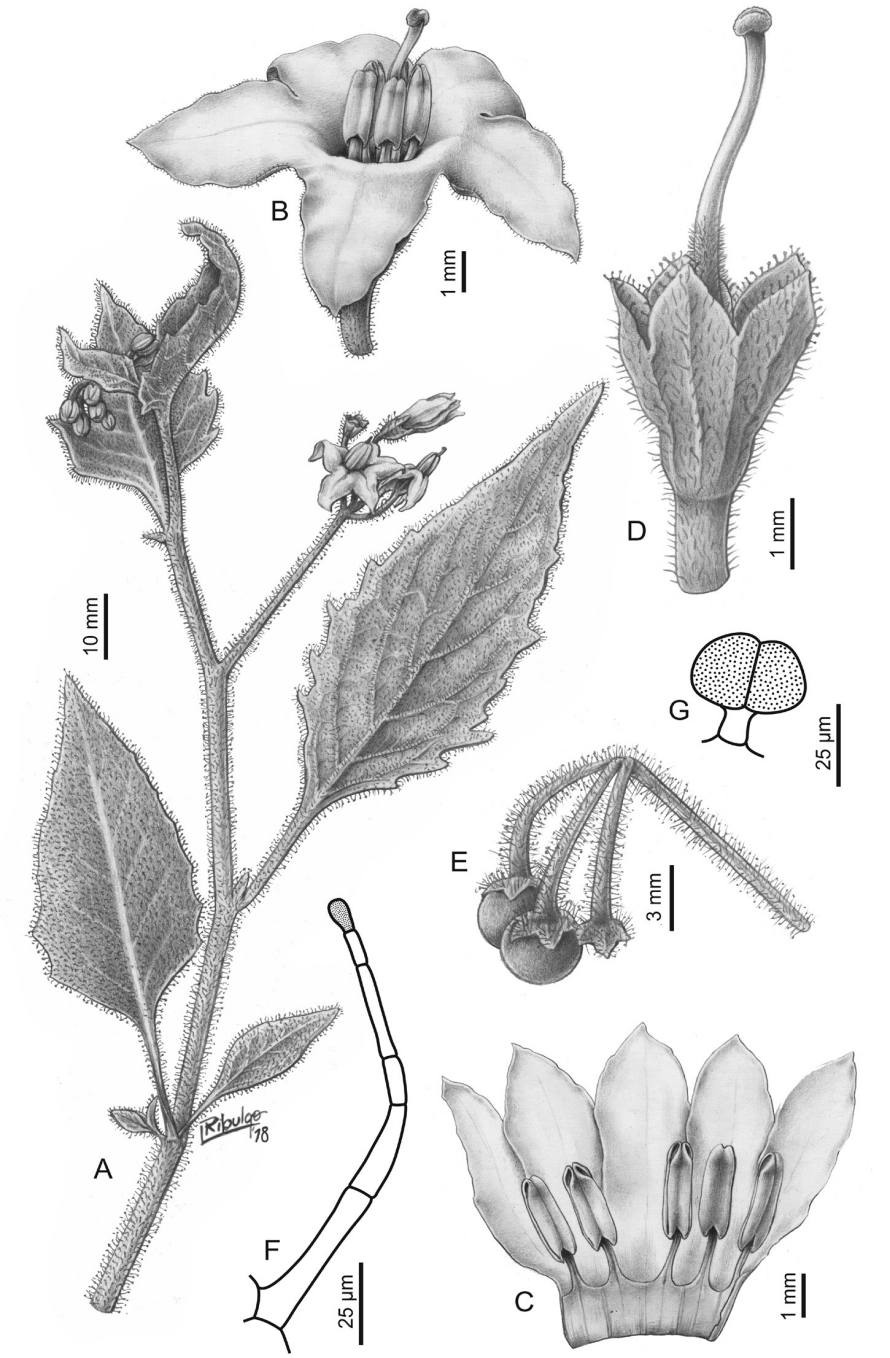
*Solanumpruinosum* Dunal. **A** Habit **B** flower **C** dissected flower **D** calyx **E** infructescence **F** elongate glandular trichome **G** sessile glandular trichome (**A–E***Amith JDA-30248*; **F–G***Ventura A. 2588*). Drawing by L. Ribulgo.

**Figure 37.**
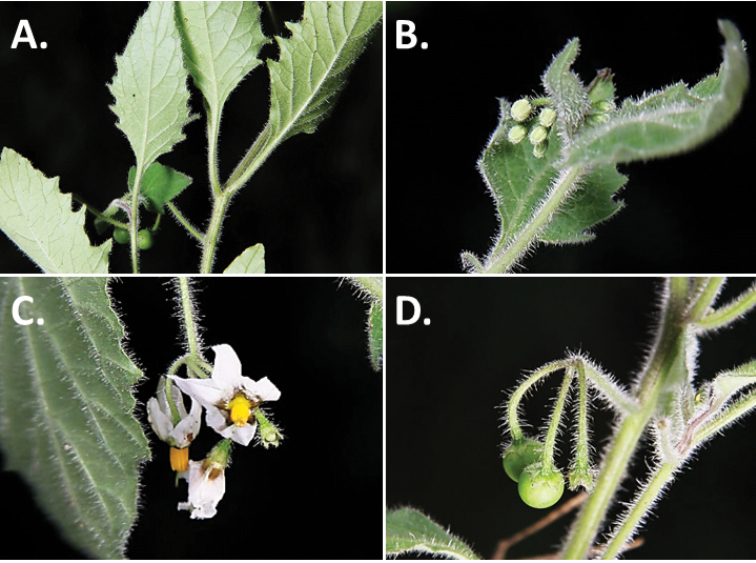
*Solanumpruinosum* Dunal. **A** Leaves **B** young branch with flowering buds **C** inflorescence with flowers at full anthesis **D** developing fruits (**A–D***Amith JDA-30248*). Photos by M. Gorostiza Salazar.

####### Distribution.

**Figure 38.**
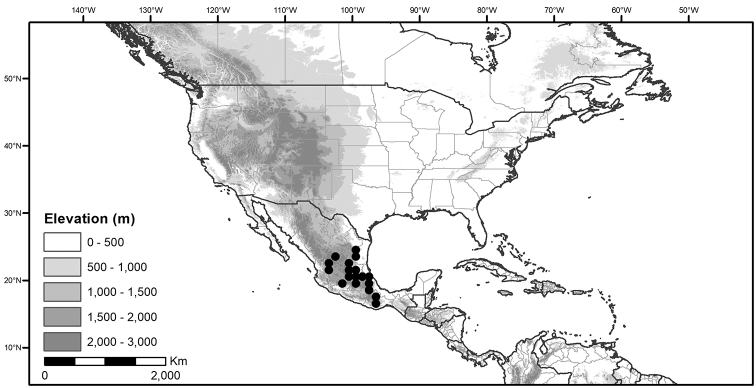
Distribution of *Solanumpruinosum* Dunal.

(Figure 38) *Solanumpruinosum* occurs from the state of Nuevo León in Mexico south to the state of Oaxaca, across the central Volcanic Belt (Rzedowski 1978).

####### Ecology.

*Solanumpruinosum* occurs in pine-oak forests, mesophyll forests and open areas from 1,000 to 2,500 m elevation.

####### Common names.

Mexico [Puebla]. Tomakilit (Nahuat, *Amith JDA-2084*), Tomakilit (silvestre) (Nahuat, *Jiménez Chimil JDA-30248*).

####### Uses.

None recorded.

####### Preliminary conservation status (IUCN 2017).

Least Concern (LC). *Solanumpruinosum* is widespread in Mexico but is not as common as *S.nigrescens*. For EOO see Table 6.

####### Discussion.

*Solanumpruinosum* differs from *S.nigrescens* and *S.douglasii*, with which it is sympatric, in its glandular pubescence. It is possible that these specimens represent isolated glandular populations of those two taxa, but in the absence of data showing this we elect to recognise these populations at the species level until further work across the range in Mexico is done. The three taxa share numerous stone cells (ca. 5–6, more in *S.nigrescens*) in the ripe berries, and subumbellate to somewhat “racemose” inflorescences. The flowers of *S.pruinosum* are intermediate in size between *S.douglasii* (15–20 mm in diameter) and *S.nigrescens* (8–10 mm).

Label data from *Martínez 1211* (MO) note the berries as “rojo” (red), but no other specimens have this data, so we suspect it is either a mistake, or an interpretation of purplish red.

The locality on the type specimen of *S.pruinosum* is only given as “circa Mexico”, but is likely to have been collected in central Mexico; Berlandier was in Mexico City and vicinity from the time of his arrival in Mexico in 1826 until his journey north to the Tamaulipas-Texas borderlands in late 1827 (Lawson 2012).

We have not been able to trace any duplicates of the type specimens of S.dasyadenium and var. uberius, both described from material held in Berlin (Bitter 1912a) and subsequently destroyed. Until we better understand the range of variation in these taxa, and their relationship to *S.douglasii* and *S.nigrescens*, we prefer not to neotypify these names at present.

####### Specimens examined.

See Suppl. materials 1 and 3.

###### 13. Solanum pseudogracile

Taxon classificationPlantaeSolanalesSolanaceae

Heiser, Bot. J. Linn. Soc. 76: 294. 1978

Figures 39 , 40

####### Type.

United States of America. North Carolina: Onslow County, N of Surf City, North Carolina Hwy. 210, 16 Jul 1960, *C.R. Bell 17061* (holotype: IND [IND-0136007, acc. # 145606]; isotype: IND [IND-0136008, acc. # 145605], NCU [NCU00062742]).

####### Description.

Subwoody annual herb to perennial shrub up to 1.0 m tall, branching at base. Stems terete or with minute spinescent processes, green-grey to straw colour, sparsely to moderately pubescent with simple, appressed, uniseriate eglandular 4–9-celled trichomes, these ca. 0.8 mm long; new growth more densely pubescent. Sympodial units difoliate, not geminate. Leaves simple, (1.3)1.8–8.3(–10.5) cm long, (0.6–)1.1–3.7 cm wide, ovate-lanceolate to narrowly ovate, slightly discolorous, green above and pale grey underneath; adaxial surface sparsely pubescent with appressed translucent, simple, uniseriate trichomes like those on stem, these denser along the veins; abaxial surface more densely pubescent like those of the upper surface evenly across lamina and veins; primary veins 3–5(6) pairs; base attenuate to acute, slightly unequal; margins entire to occasionally shallowly sinuate dentate; apex acuminate to acute; petiole (0.7–)1.0–2.4 cm long, pubescent with simple uniseriate trichomes like those of the stems and leaves. Inflorescences 1.2–2.0 cm long, lateral, internodal, unbranched or rarely forked, then with rhachis 0.4–0.5 mm long, with 3–8 flowers spaced along the rhachis, sparsely pubescent with appressed simple uniseriate trichomes like those on stem; peduncle 1.2–1.8 cm long, straight; pedicels 5–8 mm long, 0.2–0.3 mm in diameter at the base and 0.5–0.6 mm in diameter at the apex, straight and spreading, articulated at the base; pedicel scars spaced ca. 0–1 mm apart. Buds ellipsoid, corolla exserted from the calyx to 2/3 of its length. Flowers 5-merous, all perfect. Calyx tube 1.0–1.5 mm long, the lobes 0.5–1.0 mm long, ca. 1 mm wide, broadly ovate to obovate with obtuse to shortly acute apices, sparsely pubescent with appressed hairs like those on stem but shorter. Corolla 10–12 mm in diameter, deeply stellate, white with a yellow-green central portion near the base, some with darker blackish-purple colouration around the central star, lobed 2/3 to 4/5 to the base, the lobes 4.0–5.0 mm long, 1.6–3.0 mm wide, strongly reflexed at anthesis, later spreading, densely pubescent abaxially with simple uniseriate trichomes like those on stem and leaves but shorter. Stamens equal; filament tube minute; free portion of the filaments 0.6–1.0 mm long, adaxially pubescent with tangled uniseriate 4–6-celled simple trichomes; anthers 2.2–2.6 mm long, 0.5–0.7 mm wide, ellipsoid, yellow, poricidal at the tips, the pores lengthening to slits with age. Ovary globose, glabrous; style 3.5–4.0 mm long, exserted up to (1.0-)2.5 mm beyond the anther cone, densely pubescent with 2–3-celled simple uniseriate trichomes at the base; stigma capitate, minutely papillate, green in live plants. Fruit a globose berry, (4-)8–14 mm in diameter, dull purplish-black at maturity, opaque, the surface of the pericarp matte and somewhat glaucous; fruiting pedicels 7–10 mm long, 0.3–0.4 mm in diameter at the base, (0.6-)0.9–1.0 mm in diameter at the apex, deflexed, becoming woody, pedicels spaced (0)0.5–3.0 mm apart, dropping with mature fruits; fruiting calyx not accrescent, the tube 1.0–1.5 mm long, the lobes 2.5–3.0 mm long, lobes reflexed in fruit. Seeds 20–50(-60) per berry, 1.1–1.3 mm long, 0.8–0.9 mm wide, flattened and tear-drop shaped with a subapical hilum, pale yellow, the surfaces minutely pitted, the testal cells pentagonal in outline. Stone cells absent (very rarely 2). Chromosome number: *2n*=2×=24 (Heiser et al. 1965; Heiser et al. 1979).

**Figure 39.**
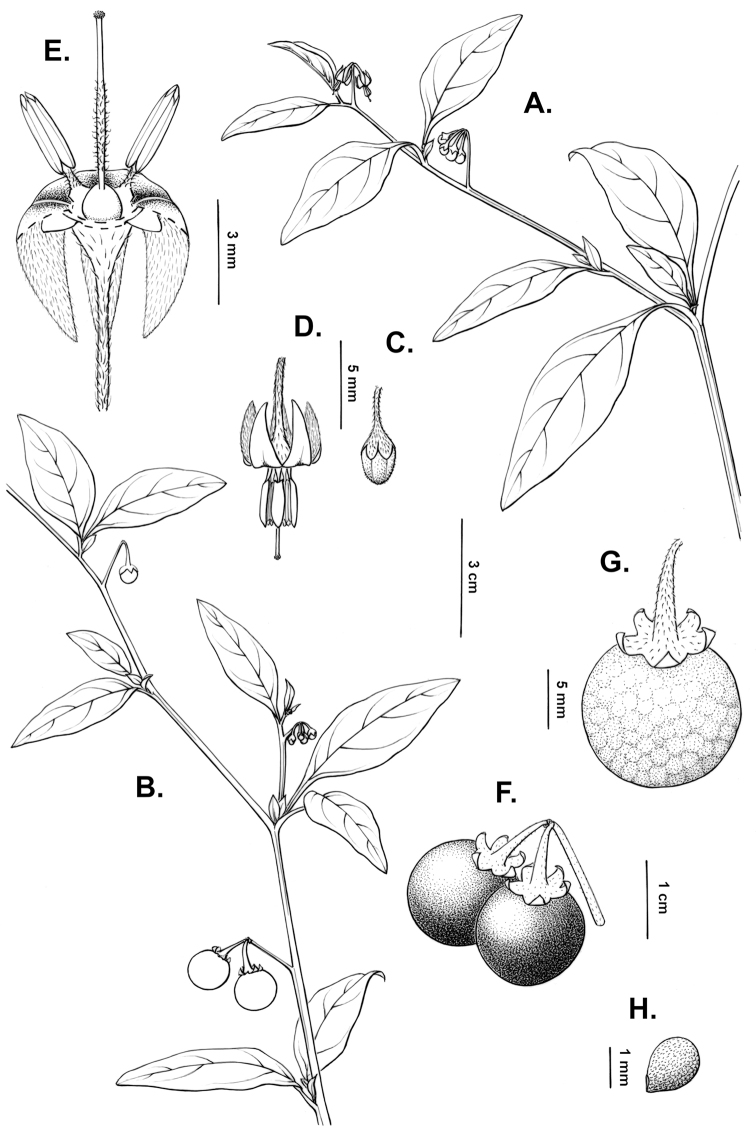
*Solanumpseudogracile* Heiser. **A** Habit **B** fruiting habit **C** bud **D** flower **E** dissected flower **F** mature fruits **G** dried berry **H** seed (**A–H***Kearney 1250*). Drawing by C. Banks.

**Figure 40.**
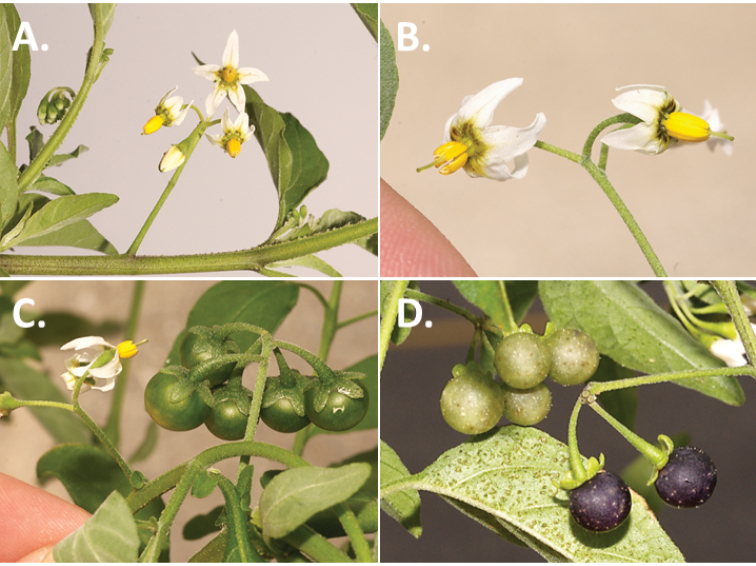
*Solanumpseudogracile* Heiser. **A** Flowering branches **B** flowers at full anthesis **C** developing fruits **D** mature fruits (**A, B, D***Nee & McClelland 60216***C***Nee & McClelland 60224*). Photos by M. Nee.

####### Distribution.

**Figure 41.**
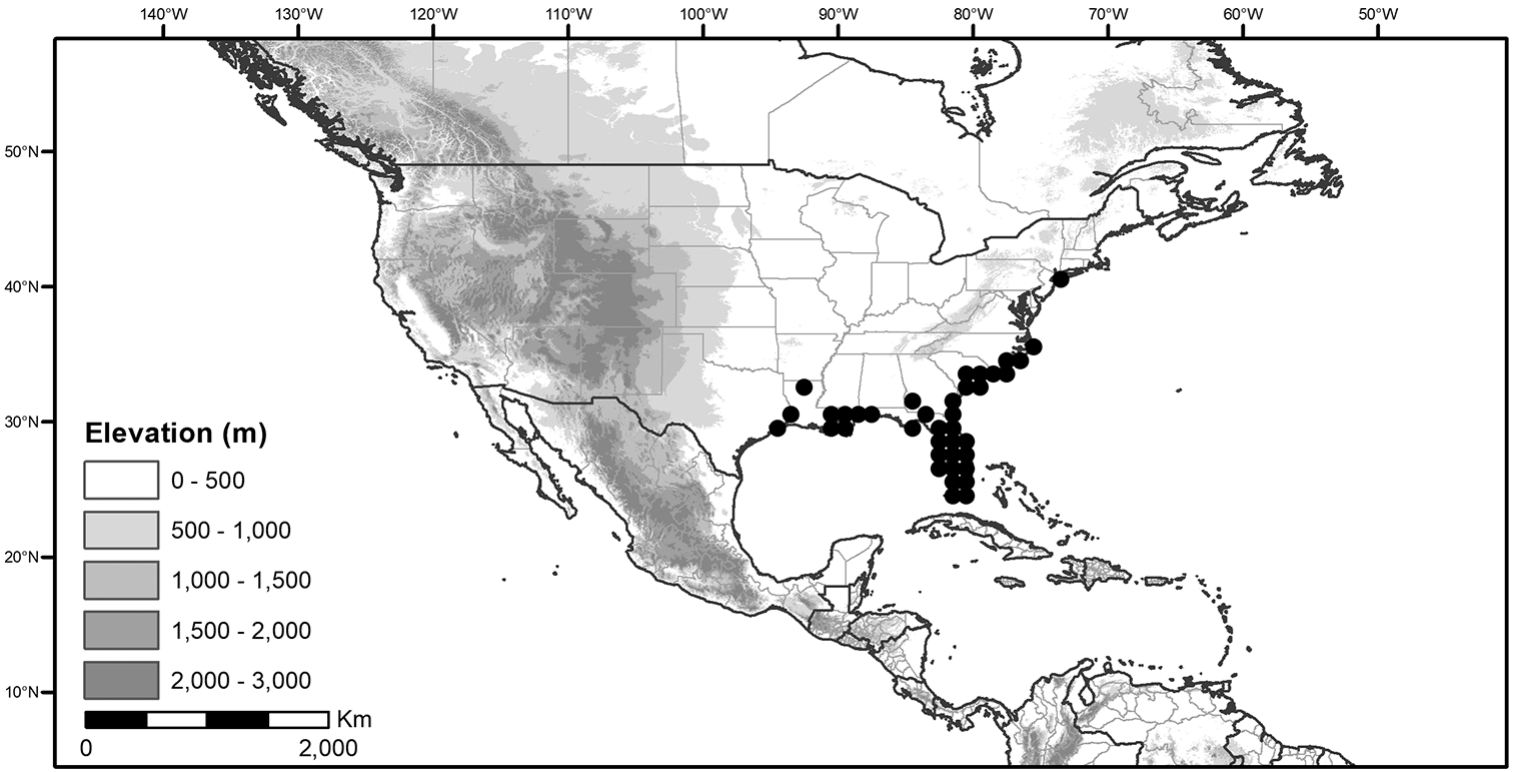
Distribution of *Solanumpseudogracile* Heiser.

(Figure 41) *Solanumpseudogracile* is endemic to the southeastern United States of America from the Atlantic coast of the Carolinas to the Gulf Coast in Florida and Alabama. Although we have seen no collections yet, we expect this species to also occur in the Bahamas.

####### Ecology.

Occurring on sand dunes, sandy moist banks and disturbed areas between 0–400 m elevation. *Solanumpseudogracile* is ecologically distinct from *S.americanum* in growing mostly on hummocks in salt marches or on sand dunes, as an epiphyte on palm trees, on walls, and among dense hedgerows.

####### Common names.

United States of America. Glowing nightshade (USDA Plants 2017). The common name of Coastal-dune nightshade (NatureServe 2017) attributed to *S.gracilius* Herter likely refers to *S.pseudogracile*.

####### Uses.

None recorded.

####### Preliminary conservation status (IUCN 2017).

Least Concern (LC). *Solanumpseudogracile* is common and weedy in coastal habitats in the southeastern United States. For EOO see Table 6. [NB: the Midwest Plants consortium is not showing locality data in North Carolina because of conservation threat – see http://midwestherbaria.org/portal/collections/individual/index.php?occid=15549149&clid=0]

####### Discussion.

*Solanumpseudogracile* is a species of the eastern North American coastal plain, and usually occurs in coastal areas not far from the sea. It is very similar to the adventive *S.chenopodioides* and can be very difficult to distinguish from it. *Solanumpseudogracile* has longer rectangular to obovate calyx lobes that are rounded to acute at the apex and reflexed in fruit, while *S.chenopodioides* has short triangular calyx lobes that are acute at the apex and always appressed in fruit. In addition, *S.pseudogracile* has a longer style that extends (1)2.0–2.5 mm beyond the anther cone, compared to *S.chenopodioides* where the style is exserted only to 1.5 mm beyond the anther cone. The species differs from *S.nigrescens* in lacking stone cells or rarely having 2, while *S.nigrescens* always has 4–13 stone cells per fruit. In the absence of fruit these two species can be very difficult to distinguish; they are widely sympatric along the Gulf Coast of the southern United States of America.

*Solanumpseudogracile* may be merely a form of *S.chenopodioides*, with which it shares many characteristics such as appressed white pubescence and absence of stone cells, but further population level work using molecular and other field markers will need to be undertaken. Distinguishing features of these morelloid species often disappear in herbarium specimens (see D’Arcy 1974b), making analysis using herbarium specimens difficult. Nee (pers. comm.) has seen plants he has identified as the two taxa growing together. The Florida plants identified as S.americanumvar.baylisii by D’Arcy (1974b) fit our concept here of *S.pseudogracile*, while the type of D’Arcy’s variety is a plant of *S.chenopodioides* collected from New Zealand. Many of the plants identified as *S.chenopodioides* in the Florida Plant Atlas (http://florida.plantatlas.usf.edu/) are most likely *S.pseudogracile*.

In describing *S.pseudogracile*Heiser (1978) cited only IND. There are two sheets of *Bell 17061* in IND, one annotated type. We select this sheet (IND-0136007) as the lectotype for *S.pseudogracile*; the sheets are not numbered sheet 1 and sheet 2 as was done by Heiser for other species in this group (e.g., *S.costaricense*, *S.leonii*).

####### Specimens examined.

See Suppl. materials 1 and 3.

###### 14. Solanum retroflexum

Taxon classificationPlantaeSolanalesSolanaceae

Dunal, Prodr. [A. P. de Candolle] 13(1): 50. 1852

Figures 42 , 43

####### Type.^5^

South Africa. Eastern Cape: Graaff Reinet (“Graafeynet”), 3000–4000 ft, 1838, *J.F. Drège 7864b* (lectotype designated by Särkinen et al. 2018, pg. 134: G-DC [G00144331]; isolectotypes: K [K000414172], S [acc. # S-G-5707]).

####### Description.

Annual to perennial herbs to 0.6 m tall, often woody at the base. Stems terete or ridged, 0.3–0.6 cm in diameter, green to yellowish-brown, prostrate or erect, the lowermost lateral branches usually spreading, if stems ridged the ridges sometimes spinescent, not markedly hollow; new growth sparsely to densely pubescent with glandular and/or eglandular simple spreading uniseriate 1–5(–8)-celled trichomes 0.1–0.8 mm long; older stems glabrescent, straw coloured. Sympodial units difoliate, the leaves not geminate. Leaves simple, (0.5–) 1.5–7.5 cm long, 1.5–5.5 cm wide, rhomboidal to lanceolate, slightly discolorous; adaxial surface green sparsely to densely pubescent with simple uniseriate trichomes like those on stem evenly spread along lamina and veins; abaxial surface slightly paler, more densely pubescent along veins and lamina; major veins 3–7 pairs, pairs not strictly opposite, not prominent; base truncate then abruptly attenuate along the petiole; margins shallowly toothed, the teeth rounded; apex acute, the tip sometimes rounded; petioles (0.5-) 1.5–3.5 cm long, sparsely to densely pubescent with simple uniseriate trichomes like those of the stems. Inflorescences 1.8–3.0 cm long, internodal, unbranched, with 3–7 flowers clustered towards the tip of the rhachis (sub-umbelliform), sparsely to densely pubescent with glandular and /or eglandular simple uniseriate trichomes like those on stems; peduncle 1.5–3.5 cm long, erect, green; pedicels 1.0–1.5 cm long, 0.3–0.6 mm in diameter at the base, 0.4–0.6 mm in diameter at the apex, recurving but not fully reflexed, pubescent like the peduncle, becoming woody, green or yellow-brown, articulated at the base; pedicel scars spaced 0–0.5 mm apart. Buds globose, the corolla 1/3 exserted from the calyx before anthesis. Flowers 5-merous, all perfect. Calyx tube 1.0–1.7 mm long, campanulate, the lobes equal, 1.0–1.5 mm long, less than 1 mm wide, oblong with rounded tips, green, sparsely pubescent with simple uniseriate trichomes like of the inflorescence. Corolla 11–16 mm in diameter, white, with a yellow basal star, stellate, lobed to 1/2–2/3 towards the base, the lobes 5.0–6.0 mm long, 2.5–2.7 mm wide, spreading to reflexed, densely papillate-pubescent abaxially with simple uniseriate trichomes, these denser on tips and margins. Stamens equal; filament tube minute; free portion of the filaments 1.2–1.5 mm long, glabrous or adaxially pubescent with tangled 6–8-celled simple uniseriate trichomes; anthers 1.3–1.8(-2.0) mm long, 1.0–1.5 mm wide, ellipsoid, yellow, poricidal at the tips, the pores lengthening to slits with age and drying, the connective becoming brownish in dry material. Ovary rounded, glabrous; style 1.9–2.2 mm long, slightly curved, pubescent with simple uniseriate trichomes 0.2–0.5 mm long in the basal 1/3 where included in the anther cone, exserted 0.5–1.5 mm beyond anther cone; stigma capitate, the surface minutely papillate. Fruit a globose to ellipsoid berry, 6–10 mm in diameter, purple-black at maturity, opaque, the pericarp thin, matte with a glaucous cast; fruiting pedicels 10–15 mm long, 0.4–0.6 mm in diameter at the base, 1.0–1.2 mm in diameter at the apex, becoming woody, recurving to deflexed, pale green to yellow-brown, persistent, spaced 0–0.5 mm apart, not falling with the fruit, remaining on the plant and persistent on older inflorescences; fruiting calyx not accrescent, the tube 1.0–1.5 mm long, the lobes 1.5–2.0 mm long, strongly reflexed. Seeds (5–)12–35 per berry, 1.3–1.5 mm long, 1.6–1.8 mm wide, flattened and tear-drop shaped with a subapical hilum, yellow to brown, the surfaces minutely pitted, the testal cells rectangular to pentagonal in outline. Stone cells absent. Chromosome number: *2n*=4×=48 (see Särkinen et al. 2018).

**Figure 42.**
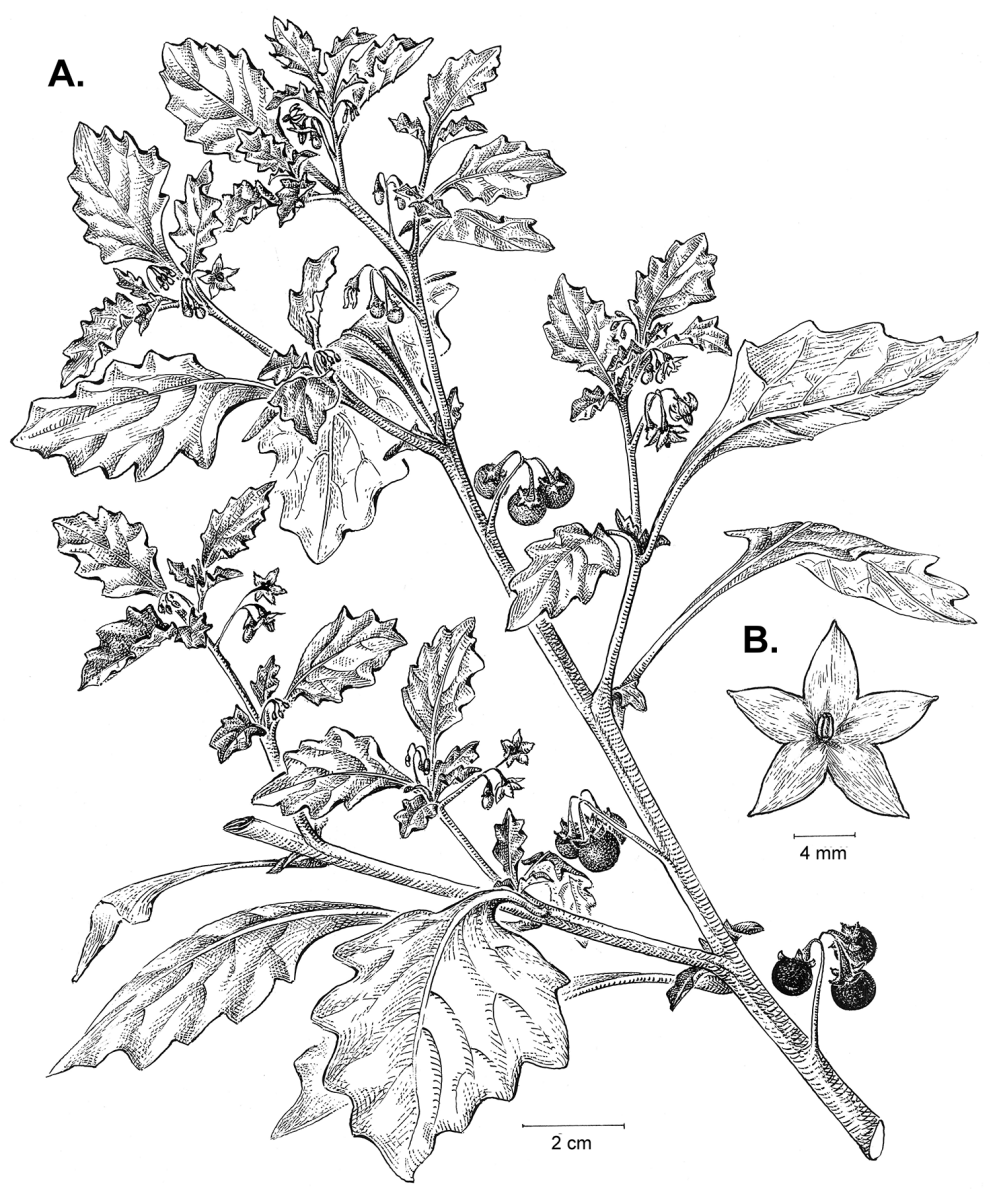
*Solanumretroflexum* Dunal. **A** Habit **B** flower. Drawing by M.L. Szent-Ivany, first published in Symon (1981), courtesy of the Board of the Botanic Gardens and State Herbarium (Adelaide, South Australia), reproduced with permission (previously also published in “PhytoKeys 106”).

**Figure 43.**
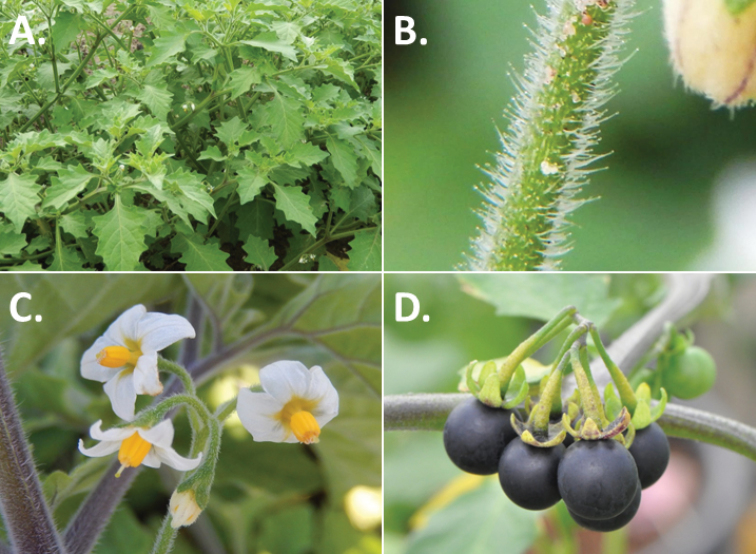
*Solanumretroflexum* Dunal. **A** Habit **B** glandular indumentum present in some individuals **C** flowers **D** mature, slightly ellipsoid matte black-purple fruits with reflexed calyx lobes (**A, C** Nijmegen accession A1450022 **B** Nijmegen accession 944750163 **C** Nijmegen accession A14750023 **D** Nijmegen accession A14750025). Photos by S. Knapp and G. van der Weerden (previously published in “PhytoKeys 106”).

####### Distribution.

**Figure 44.**
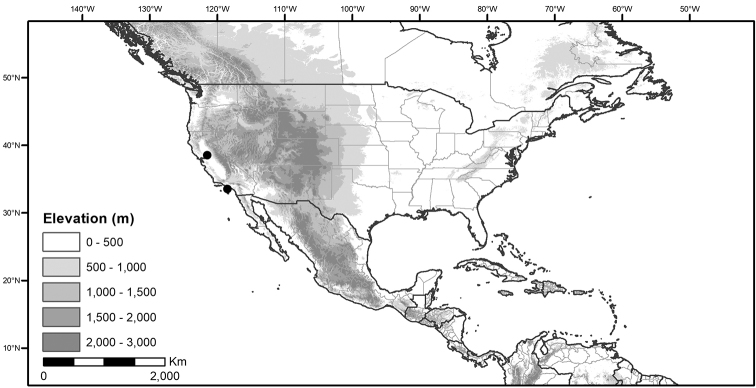
Distribution of *Solanumretroflexum* Dunal (specimens mapped are both cultivated and adventive).

(Figure 44) *Solanumretroflexum* is native to southern Africa but introduced to North America as a garden plant. In North America it is mostly cultivated.

####### Ecology.

A cultivated plant or a rare adventive in flower beds and other cultivated areas.

####### Common names.

United States of America. Sunberry (Schilling 1981), Wonderberry (see Heiser 1969).

####### Uses.

In its native South Africa, the berries are used for jam or as a fruit (Heiser 1969, Särkinen et al. 2018); its cultivation by Luther Burbank was also as a jam fruit (Heiser 1969).

####### Preliminary conservation status (IUCN 2017).

Least Concern (LC). *Solanumretroflexum* is a rare adventive species in North America; for conservation status in its native range see Särkinen et al. (2018).

####### Discussion.

In its native range *S.retroflexum* is a species that shows great variation in its indumentum, the trichomes varying from eglandular to glandular and the leaves from nearly glabrous to densely pubescent. In the geographic region treated here, it has only been collected sporadically from cultivation and appears not to escape or naturalise. The species can be distinguished from other morelloids in North America based on a character combination of inflorescences with 1–4 flowers, filaments 1.2–1.5 mm long, strongly reflexed calyx lobes in fruit, and matte purple berries that lack stone cells and drop without the pedicels. *Solanumamericanum* has similar small anthers and persistent pedicels, but the berries are very shiny and contain stone cells.

*Solanumretroflexum* is a tetraploid of uncertain parentage (see discussion in Särkinen et al. 2018). The berries are used in a local jam industry in South Africa (Viljoen 2011) and this is the species introduced as the “wonderberry” by Luther Burbank in the 1930s (Heiser 1969). The story of the mystery surrounding the identity of Burbank’s “wonderberry” is told in detail in Heiser (1969); it was variously considered a fraud or a case of mistaken identity. Burbank contended he had created the “wonderberry” through hybridisation of “S.guineense” (= *S.scabrum*) and “S.villosum” (probably = *S.nitidibaccatum*). Various correspondents (see Heiser 1969) suggested it was actually the “garden huckleberry” (*S.scabrum*) or one of the native black nightshades from North America. Stebbins and Paddock (1949) suggested that the true identity of the “wonderberry” was *S.nigrum*, a species occasionally found in agricultural fields in the western United States. Examination of specimens grown from the original seeds sold by John Childs, the sole distributor of Burbank’s “wonderberry”, have shown that these were plants of *S.retroflexum*, but how Burbank came to grow them is still not known. Typification details for the synonyms of *S.retroflexum* can be found in Särkinen et al. (2018).

####### Specimens examined.

See Suppl. materials 1 and 3.

###### 15. Solanum sarrachoides

Taxon classificationPlantaeSolanalesSolanaceae

Sendtn., Fl. Bras. (Martius) 10: 18, tab. 1, fig. 1–8. 1846

Figures 45 , 46

Solanum sarachidium Bitter, Repert. Spec. Nov. Regni Veg. 11: 211. 1912. Type. Paraguay. Gran Chaco: Loma Clavel, Nov 1903, *T. Rojas 2493* (lectotype, designated by Edmonds 1986, pg. 17: BM [BM000087577]; isolectotype: G [G00306752]).
Solanum sarrachoides Sendtn. var. sarachidium (Bitter) C.V.Morton, Revis. Argentine Sp. Solanum 122. 1976. Type. Based on Solanumsarachidium Bitter

####### Type.^6^

Brazil. “Brasilia australis”, *F. Sellow s.n.* (lectotype, designated by Edmonds 1986, pg. 16: P [P00371162]).

####### Description.

Annual herbs to 70 cm tall, usually smaller (but very rarely to 1 m), spreading and decumbent with age. Stems terete, green, generally erect, branching and later spreading, not markedly hollow; new growth densely viscid-pubescent with simple, uniseriate, spreading trichomes with a glandular apical cell, the trichomes of two lengths, 1–4-celled trichomes to 0.5 mm long and 5–14-celled trichomes to 2.0 mm long; older stems glabrescent. Sympodial units difoliate, the leaves not geminate. Leaves simple, 3.0–7.5 cm long, 3.0–6.0 cm wide, broadly ovate; adaxial and abaxial surfaces sparsely to densely pubescent with spreading, simple, uniseriate glandular trichomes like those of the stem, evenly distributed on lamina and veins; major veins 3–4 pairs; base truncate to cordate, sometimes asymmetric; margins entire or regularly sinuate-dentate; apex acute; petioles 0.5–3.2 cm long, sparsely pubescent with trichomes like those of the stem and leaves. Inflorescences 0.7–1.7 cm long, lateral, usually leaf-opposed but occasionally internodal (always very near the node), unbranched, with 2–5(6–7) flowers clustered at the tip (sub-umbelliform), sparsely pubescent with spreading trichomes like those of the stems; peduncle 0.7–1.0 cm long; pedicels 5–7 mm long, 0.1–0.2 mm in diameter at the base, 0.3–0.4 mm in diameter at the apex, straight and spreading, articulated at the base; pedicel scars spaced ca. 0(-1) mm apart. Buds globose, the corolla only slightly exserted from the calyx tube before anthesis, almost completely included within the calyx lobes and only the tip of the corolla showing. Flowers 5-merous, all perfect. Calyx tube 0.5–1.0 mm long, the lobes 1.5–2.0 mm long, 1.3–1.5 mm wide, lanceolate to narrowly ovate with acute apices, sparsely pubescent with 1–4-celled spreading glandular trichomes like those on the pedicels but shorter. Corolla 5–8 mm in diameter, white with a yellow-green central eye, pentagonal-stellate, lobed 1/2–1/3 of the way to the base, the lobes 3.0–4.5 mm long, 5.0–7.0 mm wide, spreading at anthesis, sparsely papillate-pubescent abaxially with glandular 1–4-celled simple uniseriate trichomes and eglandular papillae, these denser along margins, tips and midvein. Stamens equal; filament tube minute; free portion of the filaments 1.0–1.5 mm long, adaxially sparsely pubescent with tangled uniseriate 4–6-celled simple trichomes; anthers 1.2–2.0 mm long, 0.4–0.8 mm wide, ellipsoid, yellow, poricidal at the tips, the pores lengthening to slits with age and drying. Ovary globose, glabrous; style 3.0–3.5 mm long, densely pubescent with 2–3-celled simple uniseriate trichomes in the lower 1/2–2/3 where included in the anther cone, not usually exserted beyond the anther cone; stigma capitate, minutely papillate, green in live plants. Fruit a globose berry, 6–9 mm in diameter, green-brownish grey at maturity, opaque, the surface of the pericarp usually matte; fruiting pedicels 5–9 mm long, 0.2–0.3 mm in diameter at the base, spaced 0–1 mm apart, reflexed, dropping with mature fruits, not persistent; fruiting calyx accrescent, becoming papery in mature fruit, the tube 3–4 mm long, the lobes 5.5–8.0 mm long and 3.5–4.0 mm wide, the tips slightly reflexed or spreading. Seeds (23-)59–69(-93) per berry, 1.3–1.7 mm long, 1.0–1.5 mm wide, flattened and tear-drop shaped with a subapical hilum, pale yellow, the surfaces minutely pitted, the testal cells pentagonal in outline. Stone cells 4–6 per berry, (0.5) 0.8–1 mm in diameter. Chromosome number: 2*n*=2×=24 (see Särkinen et al. 2018).

**Figure 45.**
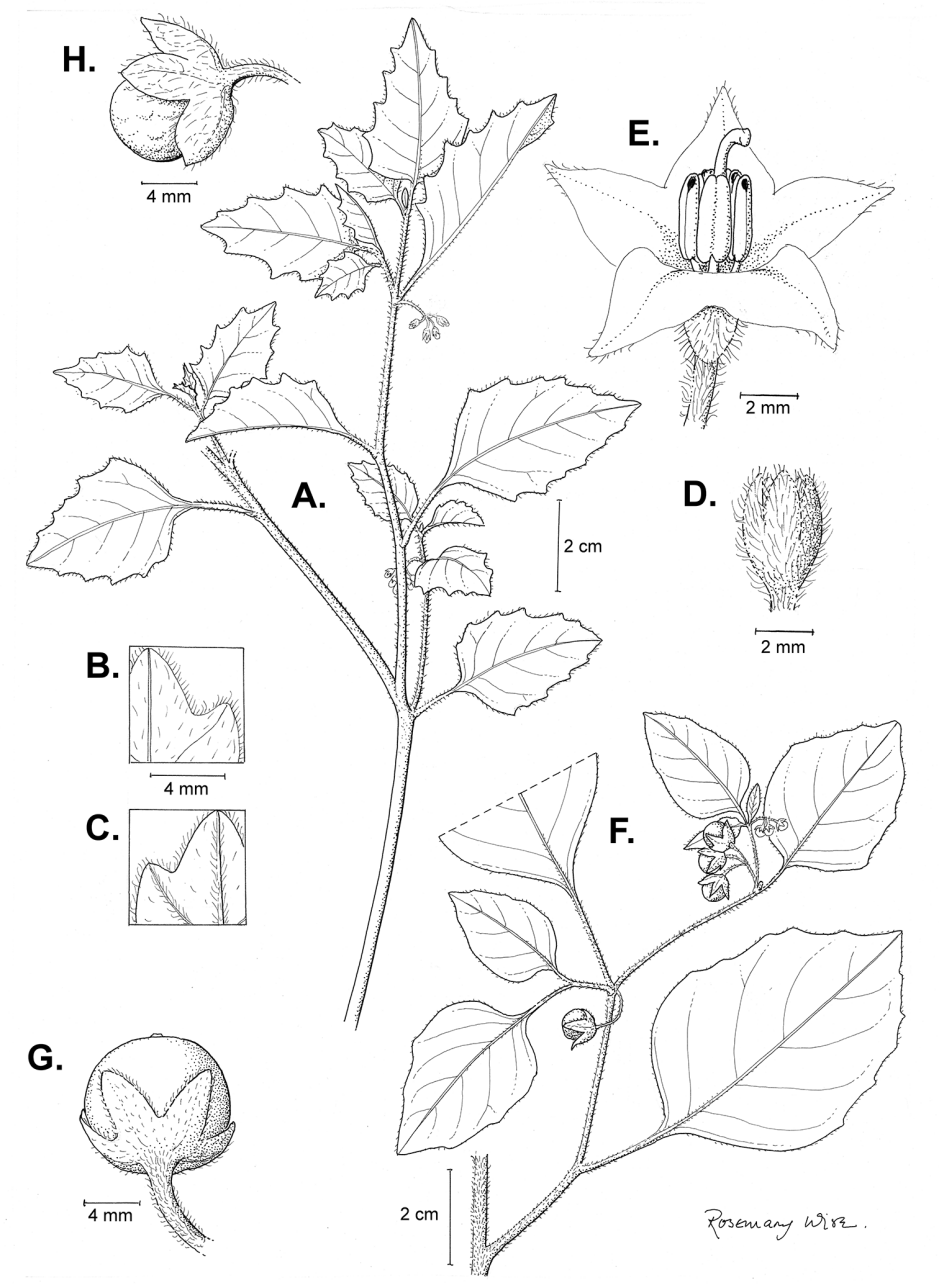
*Solanumsarrachoides* Sendtn. **A** Habit **B** detail of adaxial leaf surface **C** detail of abaxial leaf surface **D** bud **E** flower **F** fruiting habit **G** maturing fruit (**A–E***Macoun s.n.*; **F, G***Ahles 55038*). Drawing by R. Wise (previously published in “PhytoKeys 106”).

**Figure 46.**
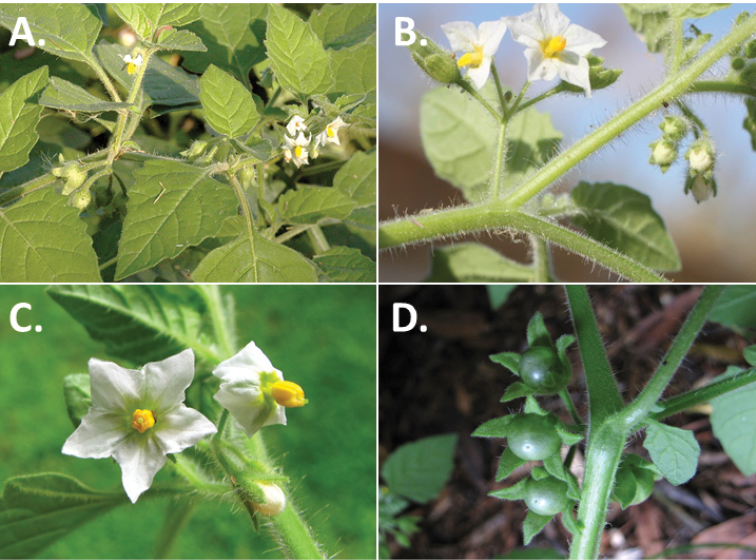
*Solanumsarrachoides* Sendtn. **A** Habit **B** inflorescence **C** flowers at full anthesis **D** developing fruits (unvouchered photos). Photos by D.G. Smith, S. Martín de la Vega, and B.W. Wells Association (previously published in “PhytoKeys 106”).

####### Distribution.

**Figure 47.**
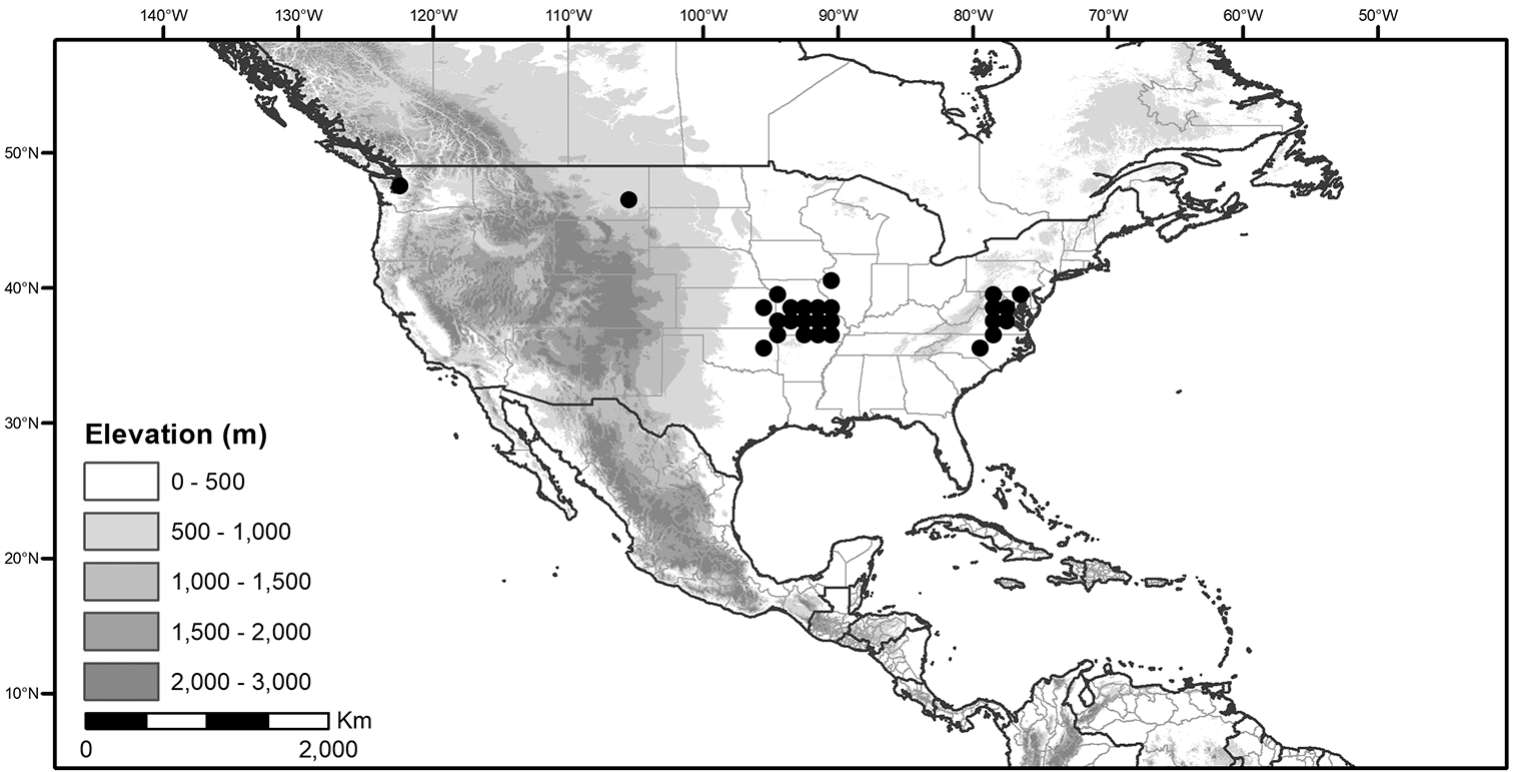
Distribution of *Solanumsarrachoides* Sendtn.

(Figure 47) *Solanumsarrachoides* is native to southern South America, and is sporadically introduced to North America, where it is much less common than the morphologically similar *S.nitidibaccatum*.

####### Ecology.

Sporadically occurs as a weed of cultivation 0–500 m elevation in urban areas, along riversides, and other disturbed areas (agriculture). *Solanumsarrachoides* is less common than *S.nitidibaccatum* as a weed of agriculture.

####### Common names.

Canada and United States of America. Hairy nightshade (many sources, but unclear if individual accounts are referring to *S.sarrachoides* or *S.nitidibaccatum*).

####### Uses.

None recorded.

####### Preliminary conservation status (IUCN 2017).

Least Concern (LC). *Solanumsarrachoides* is introduced and weedy in the United States; it also occurs in southern South America. For EOO see Table 6.

####### Discussion.

Many accounts of North American black nightshades have treated as *Solanumsarrachoides* the species whose correct name is *S.nitidibaccatum* (e.g., Stebbins and Paddock 1949; Schilling and Heiser 1979; Schilling 1981). Records of *S.sarrachoides* in the North American literature should therefore be dealt with care due to common misidentification of voucher material. The two taxa can be distinguished based using the following suite of characters: *S.sarrachoides* has generally truncate leaf bases, leaf-opposed mature inflorescences that are umbellate to sub-umbellate with fewer flowers (2–5), shorter calyx lobes 1.0–1.4 mm long, and a corolla with yellow-green central eye. *Solanumnitidibaccatum* has cuneate leaf bases, usually internodal mature inflorescences that are racemose with more flowers (4–8), longer calyx lobes 1.8–2.5 mm long, and corolla with black-purple edged central eye. The accrescent calyx almost completely encloses the matte-surfaced mature berry in *S.sarrachoides*, while the shiny, marbled berry of *S.nitidibaccatum* is always ca. halfway exserted from the calyx lobes. *Solanumsarrachoides* usually has more stone cells in each berry (4–6) than does *S.nitidibaccatum* (1–2, or absent). Though morphologically very similar, preliminary data from both nuclear and plastid DNA sequences suggests the two species are not closely related (T. Särkinen, prelim. data).

Typification details of the synonyms of *S.sarrachoides* can be found in Barboza et al. (2013) and Särkinen et al. (2018).

####### Specimens examined.

See Suppl. materials 1 and 3.

###### 16. Solanum scabrum

Taxon classificationPlantaeSolanalesSolanaceae

Mill., Gard. Dict. ed. 8, no. 6. 1768

Figures 48 , 49

Solanum fistulosum Dunal, Encycl. [J. Lamarck & al.] Suppl. 3: 749. 1814. Type. “Originaire de l’Isle de France [Mauritius], est cultivée en Amerique [Brazil]”, *Herb. Richard s.n.* (lectotype, designated by D’Arcy 1974a, pg. 735: P [P00335259]).
Solanum oleraceum Dunal var. macrocarpum Dunal, Prodr. [A. P. de Candolle] 13(1): 50. 1852. Type. Brazil. Bahia: Ilheus, 1841, *C.F.P. Martius 1255* (lectotype, designated by Edmonds 1972, pg. 108 [as holotype]: G-DC [G00144295]; isolectotype: P [P00366815]).

####### Type.^7^

Cultivated in Chelsea Physic Garden, said in protologue to “grow naturally in North America”, *Herb. Miller s.n.* (lectotype, designated by Henderson 1974, pg. 61 [as type]: BM [BM000847083]).

####### Description.

Annual or short-lived perennial herbs to 1.5 m tall, often woody at the base. Stems terete, ridged, or winged, green to purple, erect or ascending, if ridged or winged the stems later spinescent, usually somewhat hollow; new growth puberulent with simple spreading uniseriate 2–8-celled eglandular trichomes 0.3–0.8 mm long; older stems glabrescent, with or without prominent pseudospines. Sympodial units difoliate, the leaves usually not geminate, but if leaves paired then one is usually smaller. Leaves simple, 4–15(20) cm long, 3–10(16) cm wide, broadly ovate to elliptic, very variable in size depending on cultivars and growth conditions, green to dark green above to somewhat purple coloured, slightly paler; adaxial and abaxial surfaces glabrous or sparsely pubescent with simple uniseriate trichomes like those on the stem mainly along veins and scattered along lamina; major veins 3–6(–8) pairs, paler green or often purple tinged; base abruptly acute or truncate, narrowly winged onto the petiole; margins entire or rarely shallowly sinuate; apex rounded to acute; petioles 1–5(8) cm long, glabrous or sparsely pubescent with simple uniseriate trichomes like those of the stem. Inflorescences 1–2 (–4) cm long, internodal, unbranched, forked or many times branched (in cultivars), with 4–10(30+) flowers clustered towards the tips (sub-umbelliform) or spread along the rhachis, glabrous or sparsely pubescent with simple uniseriate trichomes like those on the stem; peduncle 1–5(-8) cm long, erect and thick, much thickened at the apex, subwoody, green or purple-tinged; pedicels 0.4–1 cm long, 0.3–0.5 mm in diameter at the base, 0.75–0.9 mm in diameter at the apex and abruptly expanding to the calyx tube, stout, erect and/or spreading, green or purple-tinged, glabrous or minutely pubescent like the peduncle, articulated at the base; pedicel scars tightly clustered near the tip of the rhachis, spaced 0–2 mm apart, sometimes with short stumps ca. 0.5–1.0 mm long. Buds globose to subglobose, the corolla exserted 1/2–1/3 from the calyx tube before anthesis. Flowers 5-merous or occasionally fasciate and 6–7-merous in cultivars, all perfect. Calyx tube 0.9–1.1 mm long, abruptly cup-shaped with a broad base, the lobes slightly unequal, 0.9–1.5 mm long, 0.5–1.5 mm wide, broadly deltate with a rounded tip, green or purple-tinged, glabrous or sparsely pubescent with simple uniseriate trichomes like those of the pedicels, the margins often drying scarious and white. Corolla 7–12 mm in diameter, white, purple-tinged or occasionally lilac to dark purple, with a yellow basal star, stellate, lobed ca. 1/2 of the way to the base, the lobes 2.5–4 mm long, 1.5–3 mm wide, spreading or reflexed, densely papillate on tips and margins. Stamens equal; filament tube very short, to 0.1 mm long; free portion of the filaments 0.5–0.8 mm long, glabrous or pubescent with tangled uniseriate simple trichomes; anthers 2–3 mm long, ellipsoid or slightly tapering towards the tips, yellow, orange or brown, poricidal at the tips, the pores lengthening to slits with age and drying, the connective often becoming brownish black in dry specimens. Ovary rounded, glabrous; style 2.5–5 mm long, densely pubescent with simple uniseriate trichomes 0.2–0.5 mm long in the basal 1/2 where included in the anther cone, exserted 0–1.5 mm beyond the anther cone; stigma capitate, the surface minutely papillate. Fruit a globose to slightly flattened berry, 10–20 mm in diameter, purplish black at maturity, opaque, the pericarp thick, shiny; fruiting pedicels 7–15(20) mm long, 0.5–1 mm in diameter at the base, 1.1–1.5 mm in diameter at the apex, stout, erect and spreading, purple or brown, usually not falling with the fruit, remaining on the plant and often persistent on older inflorescences; fruiting calyx not accrescent, the tube 1.5–2 mm long, usually tearing unevenly, the lobes 2–3 mm long, usually with thicker white margins in dry material, appressed or spreading to slightly reflexed. Seeds (20–)100–150 per berry, 2–2.8 mm long, 1.5–1.8 mm wide, flattened and tear-drop shaped with a subapical hilum, yellow-brown or purple, the surfaces minutely pitted, thin and the embryo clearly visible, the testal cells rectangular to pentagonal in outline. Stone cells absent. Chromosome number: *2n*=6×=72 (see Särkinen et al. 2018).

**Figure 48.**
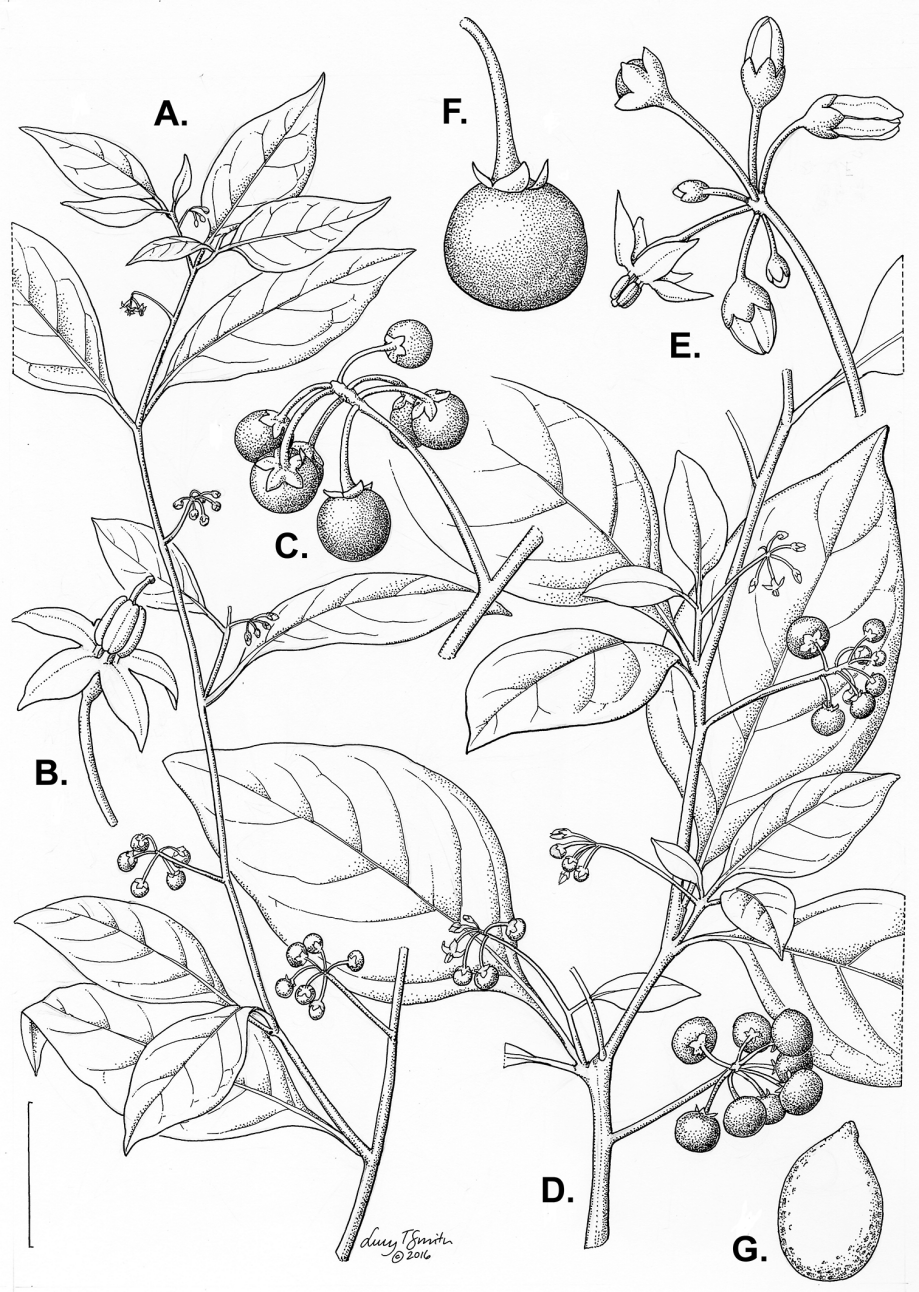
*Solanumscabrum* Mill. **A** Habit of wild form **B** flower of wild form **C** infructescence of wild form **D** habit of cultivated form **E** inflorescence of cultivated form **F** fruit of cultivated form **G** seed (**A–C***Pilz 2108*; D-G *Nee 16088*). Scale bars: 4 cm (**A, D**); 3.3 mm (**B**); 1.5 cm (**C, F**); 7 mm (**E**); 2 mm (**G**). Drawing by L. Smith (previously published in “PhytoKeys 106”).

**Figure 49.**
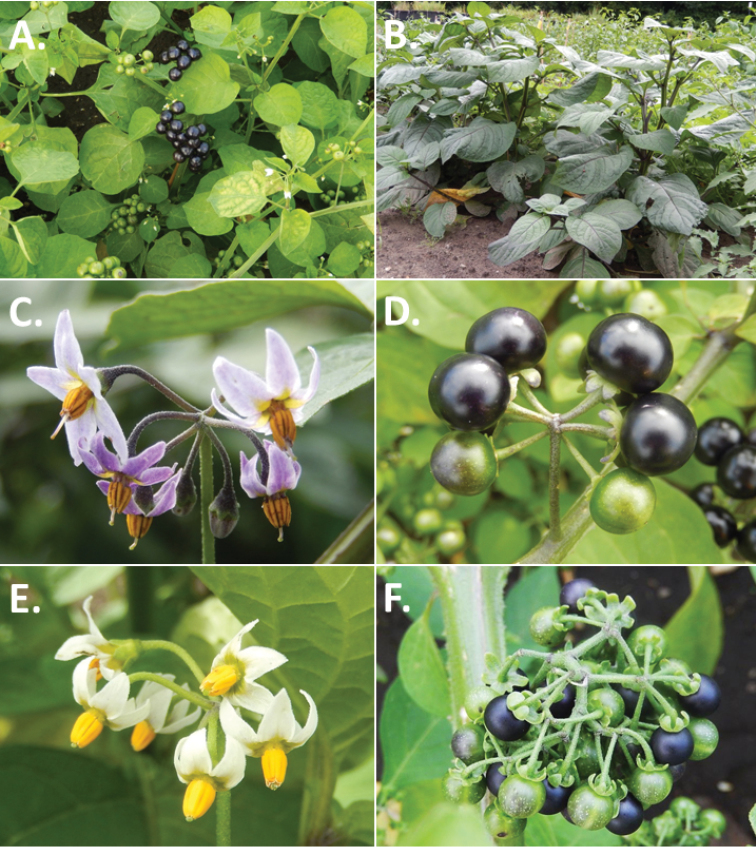
*Solanumscabrum* Mill. **A** Common habit **B** habit in taller varieties **C** flowers of the larger berried variety at full anthesis **D** fruits of a larger berried variety **E** flowers of the smaller berried variety at full anthesis **F** Fruits of a smaller berried variety (**A** Nijmegen accession BG13 **B** Nijmegen accession A34750072 **C** Nijmegen accession GB22 **D** Nijmegen accession H065 **E** Nijmegen accession A34750067 **F** Nijmegen accession 2010/3). Photos by S. Knapp (previously published in “PhytoKeys 106”).

####### Distribution.

**Figure 50.**
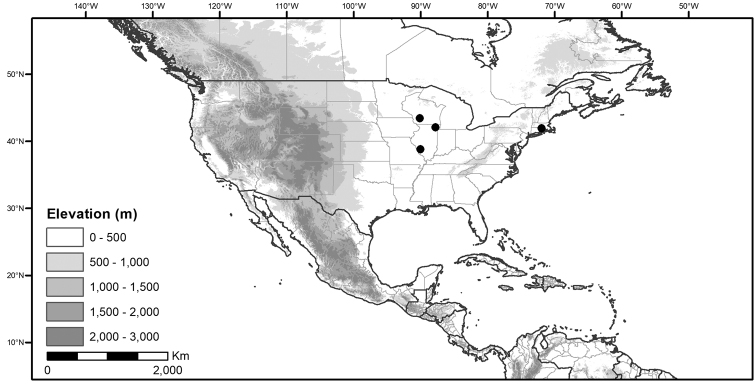
Distribution of *Solanumscabrum* Mill. (specimens mapped are all cultivated).

(Figure 50) *Solanumscabrum* is native to tropical Africa; introduced worldwide as a cultivated plant.

####### Ecology.

In the Americas only known from cultivation, although plants could persist in subtropical areas.

####### Common names.

United States of America. Garden huckleberry (Heiser 1969).

####### Uses.

Berries used for jam (in Africa leaves also consumed as spinach).

####### Preliminary conservation status (IUCN 2017).

Least Concern (LC). *Solanumscabrum* is only known from cultivation in North America; for conservation status in its native range see Särkinen et al. (2018).

####### Discussion.

*Solanumscabrum* is a species known only from cultivation in North and Central America and the Caribbean. It is the mostly commonly cultivated morelloid species in Africa, and there is used from both its leaves (eaten as spinach) and its fruits. Specimens of *S.scabrum* occasionally have been collected from areas where enslaved people were brought from western Africa (e.g., Bahia, Brazil), so it is possible it could occur in especially the Caribbean.

*Solanumscabrum* can be distinguished from the somewhat similar *S.americanum* by the larger anthers (2.5–3.0 mm long versus 0.8–1.5 mm long) that usually dry a dirty brownish tan. In both these species, as well as *S.retroflexum*, the berries drop off without the pedicels at maturity, and lack stone cells except in some populations of *S.americanum* where up to 4 stone cells have been observed (other populations lacking stone cells completely). Both *S.scabrum* and *S.americanum* have purple-black, shiny berries, while *S.retroflexum* has matte, waxy looking purple berries (with a bloom like blueberries, Heiser 1969).

Material seen from North America represents only a fraction of the diversity of *S.scabrum* across its native range in Africa and is largely composed of specimens of large berried cultivars with simple inflorescences. The cultivated plants are sold in the garden trade in United States of America under the names of ‘garden huckleberry’. The origin and identity of this garden plant gained huge interest in the 1960’s (Soria and Heiser 1959, 1961).

Typification details for the synonyms of *S.scabrum*, and a complete discussion of its morphological variability in its native range can be found in Särkinen et al. (2018).

####### Specimens examined.

See Suppl. materials 1 and 3.

###### 17. Solanum triflorum

Taxon classificationPlantaeSolanalesSolanaceae

Nutt., Gen. N. Amer. Pl. 1: 128. 1818

Figure 51 , 52

Solanum triflorum Nutt. var. majus Hook., Fl. Bor.-Amer. 2: 90. 1837, as “*major*”. Type. Canada. Saskatchewan: “Carleton House Fort, Saskatchewan River”, *J. Richardson s.n.* (lectotype, designated by Särkinen et al. 2018, pg. 167: BM [BM000934745]; isolectotype: K [K001159656, large plants]).
Solanum triflorum Nutt. var. minus Hook., Fl. Bor.-Amer. 2: 90. 1837, as “*minor*”. Type. Canada. Saskatchewan: “In the Garden (a weed) of Carleton House Fort, entrance of Badger’s Hole, and Saskatchewan River to Edmonton House [protologue]”, *T. Drummond s.n.* (lectotype, designated by Särkinen et al. 2018, pg. 167: E [E00526685]; isolectotypes: BM [BM000934744], K [K001159656]).
Solanum mendocinum Phil., Anales Univ. Chile 21(2): 403. 1862. Type. Argentina. Mendoza: Mendoza, 1860–1861, *W. Díaz s.n.* (lectotype, designated by Barboza et al. 2013, pg. 260: SGO [SGO000004580]).
Solanum calophyllum Phil., Anales Univ. Chile 21(2): 403. 1862. Type. Argentina. Mendoza: Mendoza, 1860–1861, *R. Philippi s.n.* (lectotype, designated by Särkinen et al. 2018, pg. 167 [cited as holotype in Barboza et al. 2013]: SGO [SGO000004552]; isolectotype: G [G00343450]).
Solanum pyrethrifolium Griseb., Abh. Königl. Ges. Wiss. Göttingen 24: 250. 1879. Type. Argentina. Tucumán: Lules, Dec 1873, *P. G. Lorentz & G. Hieronymus* 1132 (lectotype, designated by Morton 1976, pg. 102: CORD [CORD00006111]; isolectotype: GOET [GOET003594]).
Solanum gaudichaudii Dunal var. pyrethrifolium (Griseb.) Kuntze, Revis. Gen. Pl. 3(3): 226. 1898. Type. Based on Solanumpyrethrifolium Griseb.
Solanum triflorum Nutt. var. calophyllum (Phil.) Bitter, Abh. Naturwiss. Vereine Bremen 23: 144. 1914. Type. Based on Solanumcalophyllum Phil.
Solanum triflorum Nutt. var. pyrethrifolium (Griseb.) Bitter ex Probst, Mitteil. Naturfor. Gesellsch. Solothurn 9: 41. 1932. Type. Based on Solanumpyrethrifolium Griseb.

####### Type.^8^

United States of America. North Dakota: nr. Fort Mandan, *Anon. [Lewis & Clark] s.n.* (lectotype, designated by Barboza et al. 2013, pg. 260: PH [PH00030496]).

####### Description.

Annual herbs to 40 cm tall, much branched at the base, to 70 cm in diameter. Stems terete, green, decumbent and prostrate, forming adventitious roots at the nodes, not markedly hollow; new growth glabrous to sparsely pubescent with eglandular simple, uniseriate (3-)4–10-celled spreading trichomes 0.5–2.0 mm long, occasionally with a few glandular trichomes with a 1-many-celled apical gland; older stems glabrescent. Sympodial units difoliate or trifoliate, the leaves not geminate. Leaves simple and shallowly lobed to deeply pinnatifid, (0.5-)2.0–4.0(-5.0) cm long, 0.2–2.9 cm wide, narrowly elliptic to oblong or ovate-elliptic, fleshy in texture, green to dark green; adaxial surface glabrous to sparsely pubescent with simple, uniseriate trichomes like those on stem, scattered along lamina and more densely along the veins; abaxial surface more densely pubescent on veins and lamina; major veins 3–6 pairs, not clearly evident abaxially; base cuneate, decurrent on the petiole; sinuate-lobate to deeply pinnatifid to near-pinnate, with 3–6 linear to triangular pairs of lobes; apex acute; petioles (0.5-)1.0–2.0(-2.4) cm long, pubescent with simple uniseriate trichomes like those of the stems. Inflorescences 1.0–2.0 cm long, internodal, unbranched, with 1–5(–6) flowers clustered near the tips (sub-umbelliform), glabrous to sparsely pubescent with spreading trichomes like those of the stems; peduncle 0.8–3.5 cm long, often with apical leafy “bracteoles” (small, leaf-like structures amongst the pedicels); pedicels 3–12 mm long, 0.4–0.5 mm in diameter at the base and 0.4–0.5 mm in diameter at the apex, straight and spreading, articulated at the base; pedicel scars spaced 0(-0.5) mm apart. Buds narrowly ellipsoid or occasionally narrowly ovoid, the corolla exserted 1/5–2/5 from the calyx tube before anthesis. Flowers 5-merous, all perfect. Calyx tube 1.0–1.5 mm long, conical, the lobes 2.5–3.5(–7.0) mm long, 0.8–1.0(–4.0) mm wide, triangular-oblong with acute apices, densely pubescent with simple, uniseriate eglandular trichomes like those of the stem. Corolla 10–14 mm in diameter, white to lilac with a yellow-green central eye with black-purple coloration at the base, deeply stellate, lobed 1/2–3/4 of the way to the base, the lobes 4.0–5.0 mm long, 1.8–2.2 mm wide, reflexed at anthesis, densely pubescent abaxially with short simple uniseriate eglandular trichomes like those on stems and leaves. Stamens equal; filament tube minute; free portion of the filaments 0.6–1.0 mm long, adaxially sparsely pubescent with tangled simple, uniseriate trichomes; anthers 2.8–3.1(–4) mm long, 0.4–0.5 mm wide, narrowly ellipsoid, pale yellow, poricidal at the tips, the pores lengthening to slits with age and drying. Ovary globose, glabrous; style 2.5–3.5 mm long, densely pubescent with 2–3-celled simple uniseriate trichomes to 1/2 from the base, not exserted beyond the anther cone; stigma capitate, minutely papillate, green in live plants. Fruit a globose berry, 8–10(-20) mm in diameter, dark green at maturity, opaque, the surface of the pericarp usually shiny; fruiting pedicels 12–17 mm long, 0.5–1.0 mm in diameter at the base, 1.0–1.5 mm in diameter at the apex, spaced 0–0.5(–1.0) mm apart, reflexed and becoming woody, dropping with mature fruits, not persistent; fruiting calyx elongating in fruit, but not becoming papery nor covering the entire fruit, the tube 2.5–3.0 mm long, the lobes (4.0-)4.5–5.5(–8.0) mm long and 2.2–3.5 mm wide, strongly reflexed to spreading. Seeds 40–60 per berry, 2.0–2.5 mm long, 1.7–2.0 mm wide, subglobose, yellow, the surfaces minutely pitted, the testal cells pentagonal in outline. Stone cells 13–30, 1.0–1.5 mm in diameter. Chromosome number: 2*n*=2×=24 (South American populations only, see Särkinen et al. 2018).

**Figure 51.**
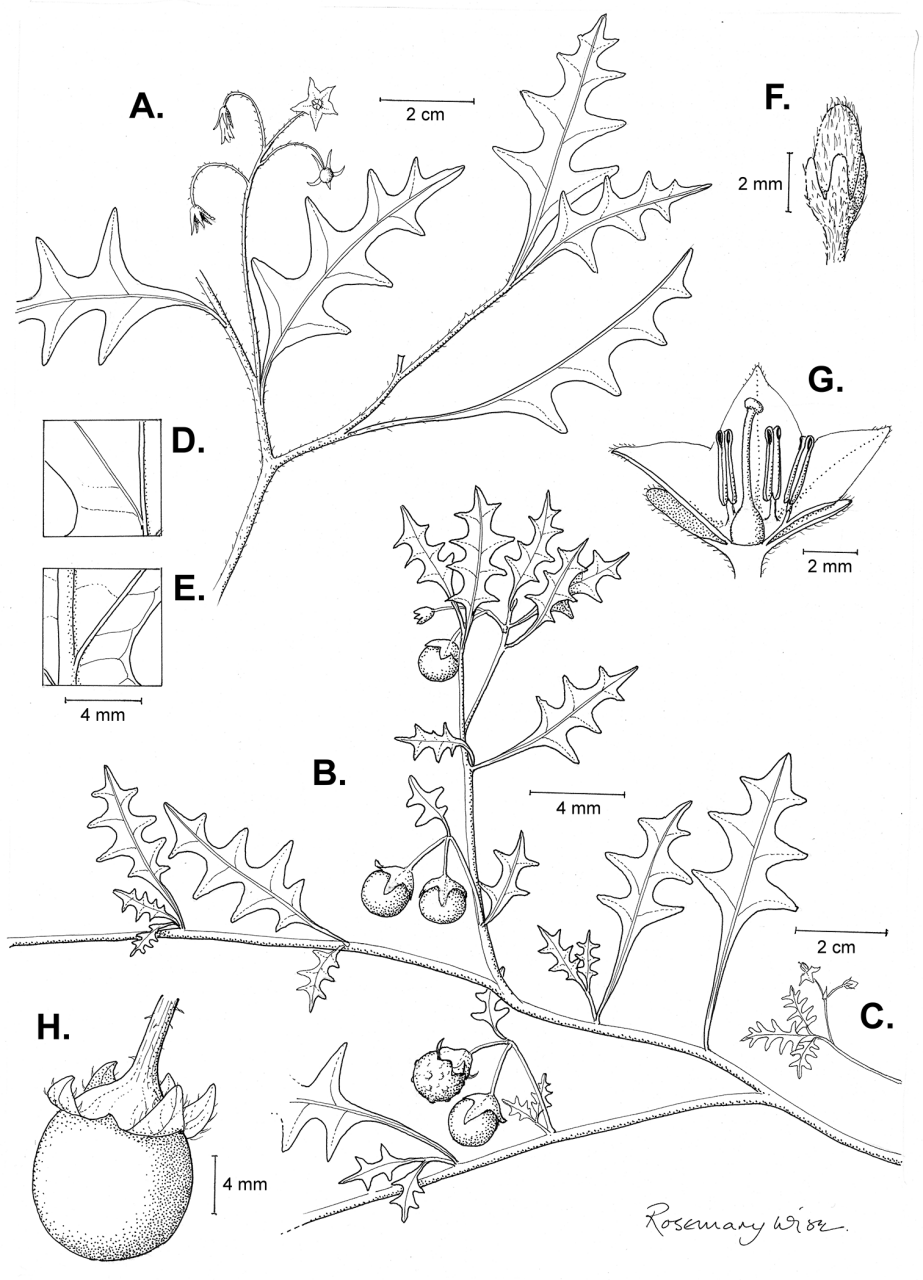
*Solanumtriflorum* Nutt. **A** Flowering habit **B** fruiting habit **C** flowering branch **D** detail of adaxial leaf surface **E** detail of adaxial leaf surface **F** bud **G** dissected flower **H** fruit (**A, C, F ,G***Donat 55*; **B, D, E, H***Baker 577*). Drawing by R. Wise (previously published in “PhytoKeys 106”).

**Figure 52.**
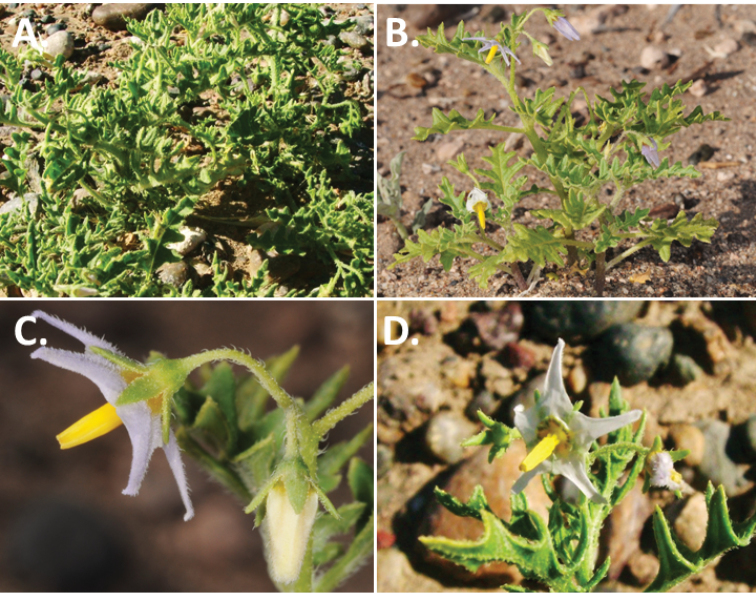
*Solanumtriflorum* Nutt. **A** Habit **B** flowering habit **C** flower and flower bud **D** flower. (**A, D***Barboza et al. 2345***B, C***Sérsic 5040*). Photos by G. Barboza and A. Sérsic (previously published in “PhytoKeys 106”).

####### Distribution.

**Figure 53.**
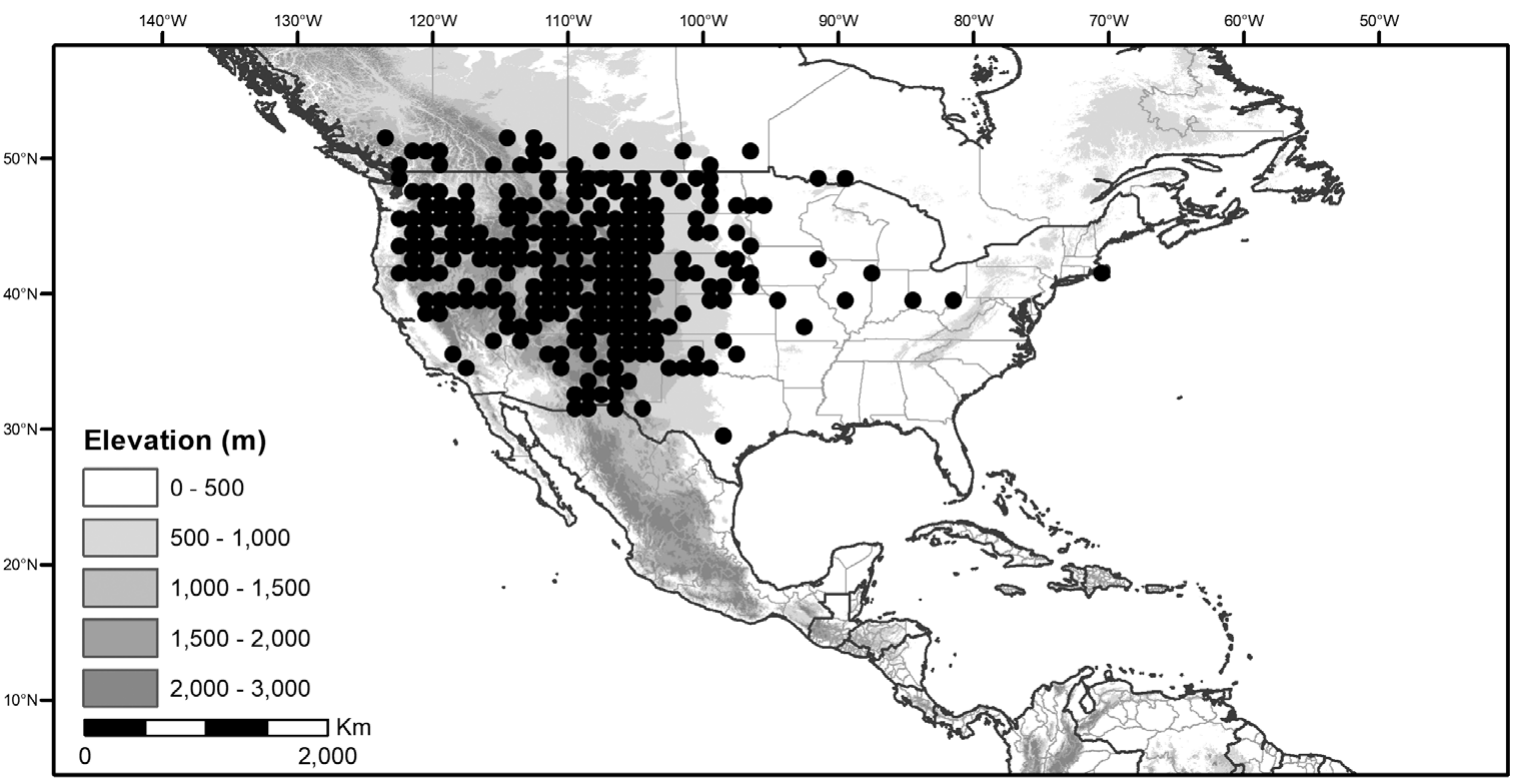
Distribution of *Solanumtriflorum* Nutt.

(Figure 53) *Solanumtriflorum* is native to the Americas with a disjunct (amphitropical) distribution between temperate South and North America. In North America it occurs in the United States of America from New Mexico and California north to Canada. The species has been introduced outside its native range in temperate areas of Europe, South Africa and Australia (see Särkinen et al. 2018).

####### Ecology.

In temperate and boreal regions *S.triflorum* shows broad ecological lability, growing along road sides, sandy soils, in cultivation, and in salt plains between (0-)700 and 2,900 m elevation.

####### Common names.

Canada. Wild tomato (Moss 1983). United States of America. Cut-leaf nightshade (many sources; USDA Plants 2017), Husk tomato (*Coombs & Bundy 2456*), Three-flowered nightshade (Peck 1941).

####### Uses.

Berries eaten in times of food shortages and famine (Acoma, Keres, Laguna peoples); fruit boiled and ground for use in a condiment (Zuni people); decoction of the berries taken for diarrhoea (Blackfoot people), stomach aches (Lakota people), used as lotion for sores on horses (Navajo people); planted with watermelons to make them more prolific and ripen earlier (Keres and Navajo peoples)(Moerman 1998 and references therein).

####### Preliminary conservation status (IUCN 2017).

Least Concern (LC); *Solanumtriflorum* is weedy and common where it occurs (see Särkinen et al. 2018). For EOO see Table 6.

####### Discussion.

*Solanumtriflorum* is a distinctive species with a prostrate habit, fleshy, usually pinnatifid, leaves, and deeply stellate flowers with long, thin anthers. The inflorescences usually have a small bracetole at the apex, and berry size varies from small (ca. 10 mm) to very large (ca. 20 mm), but usually a given plant has either small or large berries. Numerous stone cells are found in the berries, sometimes almost outnumbering seeds, and large berries can have as many as 30 stone cells. Pubescence of *S.triflorum* is quite variable (e.g., Subils 1983), and some plants have a few glandular trichomes, but for the most part plants from North America are either glabrous or very sparsely pubescent with spreading and often somewhat tangled simple trichomes.

*Solanumtriflorum* has a classic American Amphitropical Distribution (Gray and Hooker 1880; Raven 1963; AAD sensu Simpson et al. 2017), with populations occuring in North and South America, but not between (see also *S.nitidibaccatum*). Because of its weedy nature, it is often assumed to be introduced to North America (e.g., https://plants.usda.gov/core/profile?symbol=SOTR), but the amphitropical distribution pattern is found in other Solanaceae native to both regions such as *Lycium* L. (Levin et al. 2007), and groups of solanums such as the Carolinense (subsection Lathyrocarpum G.Don, Wahlert et al. 2015, as “section”) and Elaeagnifolium (Knapp et al. 2017) clades. *Solanumelaeagnifolium* Cav. (Elaeagnifolium clade, Knapp et al. 2017) has an almost identical amphitropical distribution (sensu Simpson et al. 2017) AAD, and is similarly weedy; it has also been assumed to be introduced. Distribution of these disjunct groups is more likely to be the result of long distance dispersal than of vicariance (Guilliams et al 2017), with dispersal after being eaten and passed through an animal’s gut (endozoochory) being less common than disperal via attachment to an animal’s fur or feathers (epizoochory) (Schenk and Saunders 2017); soft juicy berries make endozoochory more likely as a distribution mechanism, although there is no information on frugivores or fruit dispersal for *S.triflorum*. The distribution of *S.triflorum* in temperate areas, but also at higher elevations in deserts and into the more boreal regions of North America places it in the temperate AAD category of Simpson et al. (2017); annuals like *S.triflorum* predominate in this category. Amongst temperate AAD species the most common direction for distribution is from North to South America, but we suspect that like Verbenaceae (Frost et al. 2017) and *Lycium* (Levin et al. 2007), most *Solanum* disjunctions will have a South America to North America directionality. To date, only North American populations of *S.triflorum* have been included in molecular phylogenetic studies (Särkinen et al. 2015b).

Typification details for the synonyms of *S.triflorum* can be found in Särkinen et al. (2018).

####### Specimens examined.

See Suppl. materials 1 and 3.

###### 18. Solanum villosum

Taxon classificationPlantaeSolanalesSolanaceae

Mill., Gard. Dict. ed. 8, no. 2. 1768

Figures 54 , 55

####### Type.^9^

Cultivated in Chelsea Physic Garden from “Barbados where it is supposed to grow naturally”, *Herb. Miller s.n.* (lectotype, designated by Henderson 1974, pg. 54: BM [BM000942575]).

####### Description.

Annual to short lived perennial herbs, much branched at base and usually bushy in form, up to 0.5 m tall. Stems terete to ridged, green to purple, ascending, not hollow; new growth densely pubescent with eglandular and/or glandular, simple, spreading, uniseriate 3–10-celled trichomes 0.2–2.0 mm long; older stems glabrescent. Sympodial units difoliate, the leaves not geminate. Leaves simple, 1.5–5.0(–10.0) cm long, 0.7–2.5(–6.5) cm wide, broadly to narrowly ovate to elliptic, green; adaxial surfaces sparsely to densely pubescent with spreading, simple, uniseriate eglandular and/or glandular trichomes like those on stem evenly along veins and lamina; abaxial surfaces more densely pubescent on veins and lamina; major veins 4–6 pairs; base acute to truncate, short-attenuate, often asymmetric; margins sinuate-dentate to rarely entire; apex acute; petioles 0.5–3.0(–4.5) cm long, pubescent with simple uniseriate glandular and/or eglandular trichomes like those on stems. Inflorescences 0.4–2.0 cm long, lateral, internodal, unbranched, with (2–)3–5(–8) flowers clustered at the tips (sub-umbelliform, young inflorescences only) or more commonly spaced along the rhachis, pubescent with spreading simple glandular and/or eglandular uniseriate trichomes like those of the stems; peduncle 0.4–1.5 cm long, straight; pedicels 4–7 mm long, 0.2–0.3 mm in diameter at the base and 0.4–0.5 mm in diameter at the apex, spreading, articulated at the base; pedicel scars spaced 0–1.0 mm apart. Buds globose, the corolla exserted ca. 1/5 from the calyx before anthesis. Flowers 5-merous, all perfect. Calyx tube 1.2–1.5 mm long, conical, the lobes 0.8–1.5 mm long, 0.5–0.8 mm wide, elliptic to triangular with obtuse thickened apices and paler (almost scarious) sinuses, pubescent with spreading simple uniseriate eglandular and/or glandular trichomes like those on stem. Corolla 8–15(-20) mm in diameter, white with a yellow-green central portion near the base and occasionally with purple stripes along lobe midveins abaxially, stellate, lobed 1/2 way to the base, the lobes 2.5–4.5 mm long, 2.0–3.5 mm wide, strongly reflexed at anthesis, later spreading, densely papillate-pubescent abaxially with simple uniseriate eglandular trichomes. Stamens equal; filament tube minute, pubescent with spreading uniseriate simple eglandular trichomes adaxially; free portion of the filaments 1.0–1.3 mm long, pubescent like the tube; anthers 1.8–2.2(–2.4) mm long, 0.5–0.7 mm wide, ellipsoid, yellow, poricidal at the tips, the pores lengthening to slits with age and drying. Ovary globose, glabrous; style 2.8–3.5 (–4.0) mm long, densely pubescent with 2–3-celled simple uniseriate trichomes in the lower half, exserted 0–1 mm beyond anther cone; stigma capitate, the surface minutely papillate, green in live plants. Fruit an ellipsoid berry, usually somewhat longer than broad, 8.5–10 mm long, 8.0–9.5 mm wide, (red-)orange to yellow at maturity, translucent, the pericarp shiny; fruiting pedicels 8–14 mm long, 0.4–0.5 mm in diameter at the base, 0.7–1.5 mm in diameter at the apex, strongly reflexed, becoming woody, spaced 1.0–2.0 mm apart not falling with the fruit, remaining on the plant and always persistent on older inflorescences; fruiting calyx not accrescent, the tube 1.0–1.5 mm long, the lobes 2.0–3.0 mm long and 1.0–2.0 mm wide, strongly reflexed in fruit. Seeds 20–40 per berry, 1.8–2.2 mm long, 1.5–1.7 mm wide, flattened and tear-drop shaped with a subapical hilum, brown, the surfaces minutely pitted, the testal cells pentagonal in outline. Stone cells absent, but occasionally 1–2 found in North African and Arabian material. Chromosome number: *2n*=4×=48 (see Särkinen et al. 2018).

**Figure 54.**
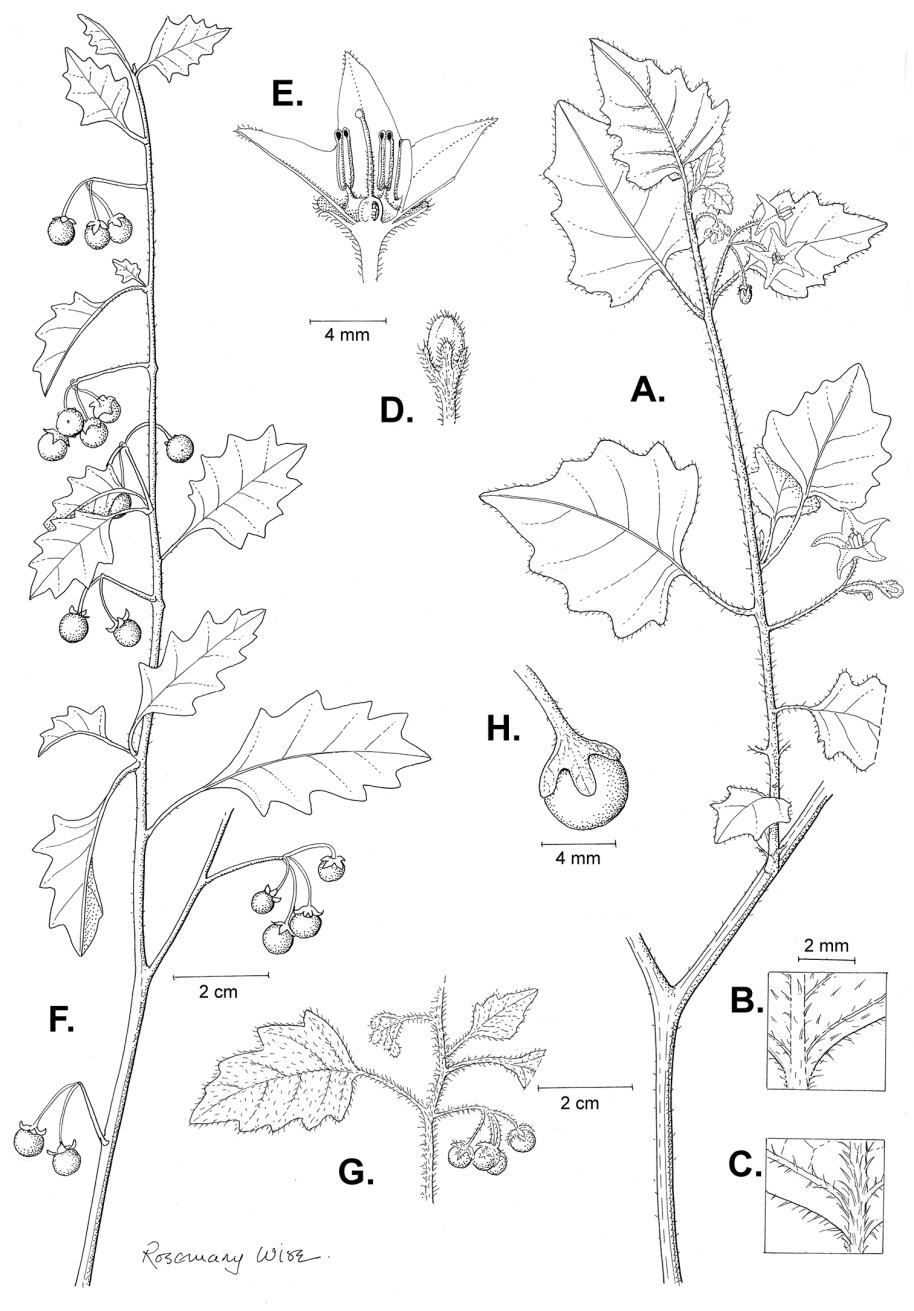
*Solanumvillosum* Mill. **A** Flowering habit **B** detail of adaxial leaf surface **C** detail of abaxial leaf surface **D** bud **E** dissected flower **F** fruiting habit **G** fruiting habit with dense indumentum **H** fruit (**A–E***Wood 2193***F***Popov GP/72/31***G***Wood Y/74/265*). Drawing by R. Wise (previously published in “PhytoKeys 106”).

**Figure 55.**
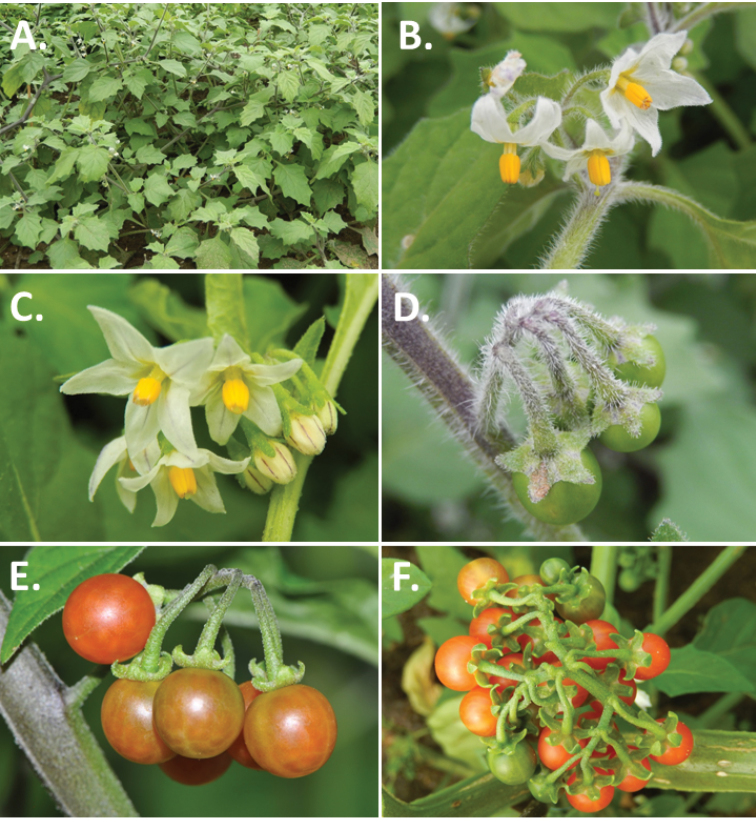
*Solanumvillosum* Mill. **A** Habit **B** inflorescence in densely pubescent plant **C** flowers and buds **D** immature infructescence **E** fully mature fruits **F** fully mature fruits showing reflexed calyx lobes (**A** Nijmegen accession 884750135 **B** Nijmegen accession 914750047 **C** Nijmegen accession A34750061 **D** Nijmegen accession A34750043 **E** Nijmegen accession A14750048 **F** Nijmegen accession A34750038). Photos by T. Särkinen and G. van der Weerden (previously published in “PhytoKeys 106”).

####### Distribution.

**Figure 56.**
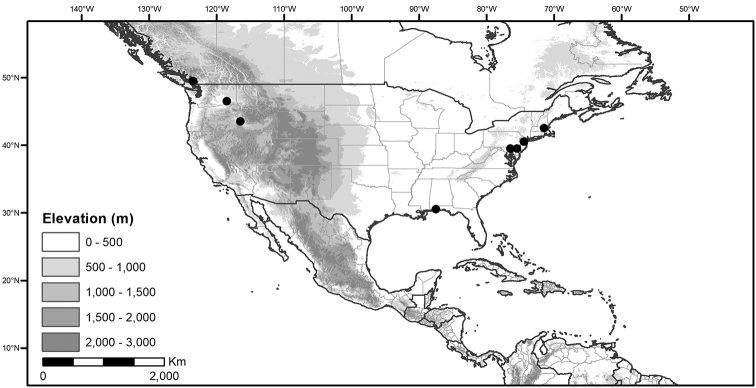
Distribution of *Solanumvillosum* Mill.

(Figure 56) *Solanumvillosum* is native to Europe where it is very common around the Mediterranean basin, the Arabian Peninsula to dry sub-tropical Asia, and eastern Africa (where it is cultivated for its fruit); the species is locally introduced but not persistent and not yet naturalised in North America.

####### Ecology.

In North America, the few collections occur at around sea level near ports and at higher inland elevations where details of cultivated versus adventive status have not been recorded on labels.

####### Common names.

United States of America. Hairy nightshade (USDA Plants 2017; Correll and Johnston 1970, but unclear if this really refers to *S.villosum* or to a pubescent form of *S.nigrum* or to *S.nitidibaccatum*).

####### Uses.

None recorded. In Africa the leaves and berries are eaten, the latter often by children (see Särkinen et al. 2018).

####### Preliminary conservation status (IUCN 2017).

Least Concern (LC). *Solanumvillosum* is a rare adventive species in North America; for conservation status in its native range see Särkinen et al. (2018).

####### Discussion.

*Solanumvillosum* is an occasional non-native species to North America; the first known specimen was collected in 1899 by Curtiss from Pensacola, thought to have arrived in ships’ ballast from Europe (D’Arcy 1974b). The species has clearly not persisted or spread in North America despite several introductions (as seen in the few specimen records from widely distant sites). The many synonyms for *S.villosum* (see Särkinen et al. 2018 for complete synonymy) reflect the broad morphological variation observed within the species, especially in its native range, where indumentum can range from nearly absent (the plants glabrous) to densely pubescent with eglandular and/or eglandular trichomes, and where cultivation and incipient domestication have resulted in a great range of inflorescence morphologies.

*Solanumvillosum* can be easily distinguished from all other morelloid species in North America by its orange-red berries that are often slightly elongated in shape and that lack stone cells. We have been unable to verify with specimens the citation of *S.villosum* from Texas (Correll and Johnston 1970); this is likely to be a misidentification. The Mexican species *S.corymbosum*, which also has orange-red berries, has branched inflorescences, minute flowers and berries with two large apical stone cells. All other species of the group in North America have rounded black or green berries. In bud and flower the calyx sinuses in *S.villosum* are much thinner than the lobes, and in dry specimens are white or transparent below the actual sinus, so that there appears to be a “window” in the calyx tube. In general aspect, plants are similar to those of *S.retroflexum*, but that species has matte purple berries with a distinct white glaucous bloom (also without stone cells, so herbarium specimens can be difficult to distinguish in the absence of good label information), and the leaves of *S.retroflexum* are more rhomboid in shape than those of *S.villosum*. Both of these introduced species are tetraploid and have glandular and eglandular forms in their native ranges.

The typification details for the 45 heterotypic Old World synonyms of *S.villosum* can be found in Särkinen et al. (2018).

####### Specimens examined.

See Suppl. materials 1 and 3.

##### Doubtful species

*Solanumfrutescens* A.Braun & C.D.Bouché, Ind. Sem. Hort. Berol. App. 9. 1853, nom. utique rej. prop.

Original material: Cultivated at Berlin (“hort. Berol.”) from seed sent from Caracas, Venezuela by J. Moritz (original material possibly once at B, now destroyed).

*Solanumfrutescens* was described from material sent as seeds from “Caracas” by J.W.K. Moritz and cultivated in Berlin (Braun et al. 1853). The specimens prepared from this cultivated material, if indeed there were any, have been destroyed, and we have seen no duplicates. The description could either be *S.macrotonum* or *S.nigrescens*, but the species in this group are all very similar, and in cultivation sometimes appear quite different from their state in nature. The only herbarium material from Venezuela collected by Moritz we have seen is the type of *S.macrotonum* (*Moritz 1643*, see *S.macrotonum*). We cannot be certain this is the same collection that was used to describe *S.frutescens* from cultivation in Berlin, and *S.macrotonum* is a name that has been in common use since the 1970s. Knapp et al. (2018) have proposed the name *S.frutescens* for suppression (nom. utique rej. under Art. 46 of the *ICN*, Turland et al. 2018).

##### “Names” (designations) not validly published

Here we only list designations that can be referred to species native to the Americas. For the many designations associated with the adventive Old World species (*S.nigrum*, *S.retroflexum*, *S.scabrum* and *S.villosum*) please see Särkinen et al. (2018). Article numbers refer to the Shenzhen Code (Turland et al. 2018).

*Solanumamaranthifolium* Gillies ex Rusby, Bull. Torrey Bot. Club 26: 152. 1899, nomen nudum; based on a Gillies manuscript name at Kew; two specimens by Gillies (K001166701, K001166704) are annotated “S. amaranthifolium Gill.” in Gillies’ hand = *S.chenopodioides* Lam.

*Solanumasperum* Hornem. ex Walp., Repert. Bot. Syst. (Walpers) 3: 49. 1844, pro syn. *Solanumrumphii* Dunal = *S.americanum* Mill.

Solanumchousboevar.merrillianum (T.N.Liou) C.Y.Wu & S.C.Huang. This citation from Flora of China (Zhang et al. 1994) that also appears on Tropicos (accessed 12 August 2017) is a misprint and confounding of the attribution to Schousboe (Schousboe ex Willd.) with a specific epithet.

*Solanumdecurrens* Wall. ex Dunal, Prodr. [A. P. de Candolle] 13(1): 50. 1852, pro syn. *Solanumrhinozerothis* Blume = *S.americanum* Mill.

*Solanumheterogonum* Dunal, Prodr. [A. P. de Candolle] 13(1): 52. 1852, pro syn. S.pterocaulumvar.heterogonum Dunal = *S.emulans* Raf.

*Solanumjahnii* Bitter ex Pittier, Cat. Fl. Venez. 2: 380. 1947, not validly published; no diagnosis or description in Latin (Art. 39.1) = *S.nigrescens* M.Martens & Galeotti

*Solanummuricatum* Bertero ex Dunal, Prodr. [A. P. de Candolle] 13(1): 150. 1852, pro syn. *Solanumrancaguense* Dunal = *S.furcatum* Dunal

SolanumnigrumL.subsp.chinense Filov, Kult. Fl. SSSR (Zhukovskii) 10: 382. 1958, not validly published; no diagnosis or description in Latin (Art. 39.1) = *S.americanum* Mill.

SolanumnigrumL.var.frutescens Macloskie, Rep. Princeton Univ. Exped. Patagonia 8: 707. 1905, nomen nudum = *S.nitidibaccatum*, *S.triflorum*, *S.chenopodioides*, or *S.pygmaeum* that all occur in Northern Patagonia

SolanumnigrumL.var.merrillianum (Liou) Filov, Kult. Fl. SSSR (Zhukovskii) 10: 383. 1958, as “merrilianum”, not validly published; no direct citation of basionym (Art. 38.1) = *S.americanum* Mill.

SolanumnigrumL.var.violaceum Chen ex Wessely, Feddes Repert. Spec. Nov. Regni Veg. 63(3): 293. 1960, nomen nudum; not intended as a new name, listed as one of the taxa accepted by Filov (1958, Kult. Fl. SSSR 20: 382). = *S.americanum* Mill.

*Solanumnodiflorum* Desv. ex Dunal, Prodr. [A. P. de Candolle] 13(1): 46. 1852, pro syn. *Solanumdesvauxii* Ham. = *S.americanum* Mill.

SolanumnodiflorumJacq.var.acuminatum Chodat, Bull. Herb. Boissier, sér. 2, 2: 811. 1902, not intended as a new name, as “*acuminatum* (?)”, with no specimen cited. In the rest of this work the new taxa are clearly indicated with “nob.” and a specimen (or several) cited = *S.americanum* Mill.

SolanumphoteinocarpumNakam. & Odash.var.violaceum C.Y.Wu & S.C.Huang, Acta Phytotax. Sin. 16(2): 72. 1978, nomen nudum; incorrectly cited in this publication as *violaceum* Chen ex Wessely but Wessely (1960) did not formally publish this name, she cited Filov’s (1958) list of accepted taxa. Filov did not provide Latin descriptions or diagnoses (Art. 38.1) and so all names in that work are not validly published. The varietal epithet should be attributed only to Wu and Huang (1978), but they do not provide a Latin diagnosis either (Art. 39.1) = *S.americanum* Mill.

*Solanumvirgatum* Endl. ex Sendtn., Fl. Bras. (Martius) 10: 13. 1846, pro syn. *Solanumgracile* Sendtn. = *S.chenopodioides* Lam.

## Supplementary Material

XML Treatment for Solanum americanum

XML Treatment for Solanum chenopodioides

XML Treatment for Solanum corymbosum

XML Treatment for Solanum douglasii

XML Treatment for Solanum emulans

XML Treatment for Solanum furcatum

XML Treatment for Solanum interius

XML Treatment for Solanum macrotonum

XML Treatment for Solanum nigrescens

XML Treatment for Solanum nigrum

XML Treatment for Solanum nitidibaccatum

XML Treatment for Solanum pruinosum

XML Treatment for Solanum pseudogracile

XML Treatment for Solanum retroflexum

XML Treatment for Solanum sarrachoides

XML Treatment for Solanum scabrum

XML Treatment for Solanum triflorum

XML Treatment for Solanum villosum

## Footnotes

Types based on American specimens only, for full synonymy see Särkinen et al. (2018: 51–56)

Types based on American specimens only, for full synonymy see Särkinen et al. (2018: 65–66)

Types based on American specimens only, for full synonymy see Särkinen et al. (2018: 92–96)

Types based on American specimens only, for full synonymy see Särkinen et al. (2018: 105–106)

Types based on American specimens only, for full synonymy see Särkinen et al. (2018: 133–134)

Types based on American specimens only, for full synonymy see Särkinen et al. (2018: 141)

Types based on American specimens only, for full synonymy see Särkinen et al. (2018: 146–147)

Types based on American specimens only, for full synonymy see Särkinen et al. (2018: 167–168)

Types based on American specimens only, for full synonymy see Särkinen et al. (2018: 176–183)

## Supplementary materials

Supplementary material 1 Specimens cited in pdf format (traditional format; only specimens from the region cited) Data type: PDF file This dataset is made available under the Open Database License (http://opendatacommons.org/licenses/odbl/1.0/). The Open Database License (ODbL) is a license agreement intended to allow users to freely share, modify, and use this Dataset while maintaining this same freedom for others, provided that the original source and author(s) are credited.Sandra Knapp, Gloria E. Barboza, Lynn Bohs, Tiina Särkinen

Supplementary material 2 Index to numbered collections (exsiccatae; all specimens examined for this treatment, including Old World and South America) Data type: PDF file This dataset is made available under the Open Database License (http://opendatacommons.org/licenses/odbl/1.0/). The Open Database License (ODbL) is a license agreement intended to allow users to freely share, modify, and use this Dataset while maintaining this same freedom for others, provided that the original source and author(s) are credited.Sandra Knapp, Gloria E. Barboza, Lynn Bohs, Tiina Särkinen

Supplementary material 3 Searchable CSV file of all specimens examined for this treatment, including Old World and South America Data type: CSV file This dataset is made available under the Open Database License (http://opendatacommons.org/licenses/odbl/1.0/). The Open Database License (ODbL) is a license agreement intended to allow users to freely share, modify, and use this Dataset while maintaining this same freedom for others, provided that the original source and author(s) are credited.Sandra Knapp, Gloria E. Barboza, Lynn Bohs, Tiina Särkinen
