# Supplementary material for: A revision of the Morelloid Clade of Solanum L. (Solanaceae) in North and Central America and the Caribbean
Source: PhytoKeys. 2019 May 30;123:1–144. doi: 10.3897/phytokeys.123.31738 (PMC6554266; doi:10.3897/phytokeys.123.31738)
Supplement: Supplementary material 1 [file phytokeys-123-001-s001.pdf]

Appendix 1. Specimens examined

We list here in traditional format all specimens examined for this treatment from North and Central America and the Caribbean. Countries, major divisions within them (when known), and collectors (by surname) are listed in alphabetic order.

1. *Solanum americanum* Mill.
ANTIGUA AND BARBUDA. **Antigua**: SW, Blubber Valley, Blubber Valley, 26 Sep 1937, *Box, H.E. 1107* (BM, MO); sin. loc. [ex Herb. Hooker], *Nicholson, D. s.n.* (K); **Barbuda**: S.E. side of The Lagoon, 16 May 1937, *Box, H.E. 649* (BM).

BAHAMAS. Man O'War Cay, Abaco region, 8 Dec 1904, *Brace, L.J.K. 1580* (F); Great Ragged Island, 24 Dec 1907, *Wilson, P. 7832* (K). **Andros Island**: Conch Sound, 8 May 1890, *Northrop, J.I. & Northrop, A.R. 557* (K). **Eleuthera**: North Eleuthera Airport, Low coppice and disturbed area around terminal and landing strip, 15 Dec 1979, *Wunderlin, R.P. et al. 8418* (MO). **Inagua**: Great Inagua, 12 Mar 1890, *Hitchcock, A.S. s.n.* (MO); sin. loc, 3 Dec 1890, *Hitchcock, A.S. s.n.* (F). **New Providence**: sin. loc, 18 Mar 1878, *Brace, L.J.K. 518* (K); Nassau, Union St, 20 Feb 1905, *Wight, A.E. 111* (K); Grantstown, 28 May 1909, *Wilson, P. 8213* (K).

BARBADOS. Moucrieffe (?), St John, Near boiling house, Apr 1940, *Goodwing, H.B. 197* (BM).

BELIZE. carretera a Belmopan, 1 May 1982, *Ramamoorthy, T.P. et al. 3593* (MEXU). **Belize**: Belize Municipal Airstrip near St. Johns College, Belize City, 21 Feb 1970, *Dieckman, L. 177* (MO); Campus of St. John's College, in Belize City, 10 Apr 1969, *Lazor, R.L. & Tyson, E.L. 2136* (MO). **Cayo**: On and around quarry hill 2.5 miles S of junction of Western & Hummingbird Hwys, 25 Jul 1970, *Spellman, D.L. & Newey, W.W. 1861* (MO). **Orange Walk**: Indian Church, 2 Jun 1977, *Arnason, J.T. & Lambert, J. 17626* (MO); As weed on experimental raised field 100 m south of San Antonio village, 15 Mar 1975, *Clevidence, J.P. 7526* (MO); 100 m south of San Antonio village. Square 28Q (field notes 76-2-64), 19 Sep 1976, *Puleston de*, *7631* (MO). **Toledo**: along the Southern Highway 4 miels W of the Bladden Branch, 15 Mar 1987, *Brant, A. & Davidse, G. 1044* (MEXU); 1.5 mi S of Mayan village of San Jose, ca. 5 mi W of Columbia Forest Station, 12 Jun 1973, *Croat, T.B. 24287* (MO); Southern Maya Mountains, Bladen Nature Reserve, dry (during the dry season) river bed of the upper Bladen Branch, SE of 'AC Camp' and SW of the Southern end of the Ek Xux valley, 17 May 1997, *Davidse, G. & Holland, D. 36560* (BM, MEXU); Trail to Esperanza, beginning 1 mile north of Columbia Forest Station, 12 Jun 1973, *Dwyer, J.D. 11145* (MO); In wamil, on hillside near San Antonio, 18 Oct 1952, *Gentle, P.H. 7799* (MO).

BERMUDA. Serpentine marsh, 31 Aug 1905, *Brown, S. & Britton, N.L. 198* (K); Middle Road, Bermuda Islands, 29 Jul 1913, *Collins, F.S. 281* (K, W); Warwick, 1843, *Hunter, R. 20* (BM); Clearing in rocky jungle. Near Castle Harbour Hotel, 16 Mar 1933, *Rendle, A.B. 292* (BM); By side of the path to Hungy Bay, 27 Mar 1933, *Rendle, A.B. 396* (BM); near Clarence Peniston's [Clarence Cove?], 27 Oct 1910, *Smith 69* (K).

BRITISH VIRGIN ISLANDS. **Anegada**: Plentiful along main road between The Settlement and Nutmeg Point, 5 Feb 1971, *D'Arcy, W.G. 5122* (MO). **Tortola**: Experiment Station, Apr 1913, *Fishlock, W.C. 8* (K).

CANADA. **British Columbia**: Burnaby, Barnet Road, above Burrard Inlet, 3 Oct 1965, *Bayly, I. 94* (UBC); Huntingdon, 30 Oct 1957, *Beamish, K.I. s.n.* (UBC); Sechelt, 31 Aug 1969, *D'Arcy, W.G. 3692* (MO); West Vancouver, 18 Aug 1936, *Eastham, J.W. s.n.* (UBC); Vancouver, ex hort UBC, 28 Sep 1949, *Elvidge, D. 87* (UBC); New Westminster, Oct 1897, *Hill, A.J. s.n.* (UBC); New Westminster, 10 Aug 1912, *Hill, A.J. s.n.* (UBC); New Westminster, 10 Aug 1912, *Hill, A.J. s.n.* (UBC); Greater Vancouver, Surrey, 300 m W of Scott Rd, under Skytrain, 6 Sep 2003, *Lomer, F. 5043* (UBC); Greater Vancouver, Surrey, 80 m E of Scott Road, opposite 12344 Lynn Rd, 21 Oct 2004, *Lomer, F. 5549* (UBC); Fraser Valley, Abbotsford, Hwy 1 and Whatcom Road, NE side, 29

Aug 2006, *Lomer, F. 6126* (UBC); Fraser Valley, 5 km E of Agassiz, Herrling Island, 14 Oct 2006, *Lomer, F. 6185* (UBC); Fraser Valley, Hope, Kawkawa Lake Indian Reserve 16, E end Ogilvie Drive near Kawkawa Lake, 22 Aug 2007, *Lomer, F. 6375* (UBC); Fraser Valley, Abbotsford, Eagle Mtn, SW of Sumas Mtn, N of eagle Mtn Drive, cleared moutnian slope for new subdivision, 16 Sep 2007, *Lomer, F. 6425* (UBC); Vancouver, Smithe Street and Pacific Blvd, SW corner, 13 Oct 2009, *Lomer, F. 7212* (UBC); Metro Vancouver, Burnaby, 220 m W of Kensington Avenue, 300 m N of Joe Sakic Way, soil dump in large landfill clearing 1.2 km due NW of Burnaby Lake, 2 Sep 2013, *Lomer, F. 8576* (UBC); banks of Fraser River near Chilukwayak [Chillwack] "Cascade Mountains", Oct 1859, *Lyall, D. s.n.* (K); "Oregon Boundary Commission, Cascade Mountains, 49° N latitude" [exact border of USA and Canada], 1859, *Lyall, D. s.n.* (GH, W); Agassiz, 30 Jul 1889, *Macoun, J. s.n.* (BM); Vancouver Island, Cameron Lake, 25 Sep 1917, *McBey, T.P. s.n.* (UBC); N base of Vedder Mountain, 8 miles E of Huntingdon, 25 Sep 1936, *McCabe, T.T. 3799* (UC); Yale Road [Vancouver], 29 Aug 1913, *Taylor, W. s.n.* (UBC); Yale Rd, 29 Aug 1923, *Taylor, W. s.n.* (UBC); Popcum [Popkum] District, 2 miles from Agassiz on S side of Fraser, 2 Sep 1912, *Taylor, W. s.n.* (UBC).

CAYMAN ISLANDS. **Cayman Brac**: East end, Spot Bay. On the bluff, 24 May 1938, *Kings, W. C.B. 65* (BM). **Grand Cayman**: Governor's Creek Lagoon, W. Bay, 29 May 1967, *Brunt, M. 2056* (BM); 1890, *Hitchcock, A.S. s.n.* (MO); Along Crew Road, 1.5 miles E.S.E. of Georgetown, 28 Apr 1956, *Proctor, G.R. 15277* (BM).

COSTA RICA. **Alajuela**: Road heading NW from San Ramon between Los Angeles and the río Catarata. Fencerows and remnant, 16 Jun 1983, *Barringer, K.A. 3215* (CR); Poás, Dist. San Rafael, Potreros y camino a San Pedro de Poás, 5 Nov 1933, *Brenes, A.M. s.n.* (CR); San Ramón, San Miguel de San Ramón, 9 Mar 1936, *Brenes, A.M. s.n.* (CR); Alajuela, Dist. Sarapiquí, Falls of the Río La Paz about 7 km below Vara Blanca de Sarapiquí, 11 Aug 1971, *Burger, W.C. & Burger, M. 7992* (CR); Rio Hondo, Plains of Santa Clara, 5 May 1903, *Cook, O.F. & Doyle, C.B. 533* (US); Cantón de San Ramón. Cordillera de Tilarán. 2 Km east of Santa Rita, 40 Km north of San Ramón. Primary forest fragment along Quebrada Grande, 1 Dec 1993, *Haber, W.A. et al. 11714* (MO); San Carlos, Distr. Florencia, 2 Km east of Santa Rita. 40 Km north of San Ramon. Primary forest fragment along Quebrada Grande, 1 Dec 1993, *Haber, W.A. et al. 11714* (CR); San Ramón, Entre 2 y 4 km por la carretera que va a San Miguel al oeste de la ciudad, 6 May 1987, *Hernández, R. 870506 SR.15* (CR); Alajuela, Dist. San José, Barrio San José. EStación Experimental Fabio Baudrit, 11 Mar 1993, *Jiménez M, Q. et al. 1188* (CR); Alajuela, Barrio San José. Estación Experimental Fabio Baudrit, 11 Mar 1993, *Jiménez, Q. et al. 1188* (MO); San Carlos, Distr. Fortuna, R. B. Arenal Mundo Aventura, 30 Apr 2004, *Rodríguez G, A. 8780* (CR). **Cartago**: Paraíso, Distr. Orori, 2 km después de Orosí hacia Tapantí, 4 Dec 2000, *Alfaro, E. 3517* (CR); Turrialba, 1 Feb 1981, *Artavia, M. 7261* (CR); Turrialba, Finca del Instituto, Mar 1949, *Buchanan, R.E. 470* (US); On grounds of Inter-American Institute of Agricultural Sciences, Turrialba, 13 Oct 1953, *Heiser, C.B. 3627* (US); Paraíso, Dist. Llanos de Santa Lucía, Finca "El Radio" de Radiográfica Costarricense. Entre Cartago y Paraíso, 23 Mar 1984, *Hernández, R. et al. 840323 -23* (CR); Cartago, Dist. San Nicolás, Sobre terreno arcilloso muy alterado junto a carretera. El Alto de Ochomogo, 10 Apr 1987, *Hernández, R. 870410 -14* (CR); Turrialba, Río Turrialba NE of town, 13 Feb 1984, *Khan, R. et al. 1000* (BM, CR, MO); Turrialba, Weed in cacao orchard, Instituto Interamericana de Ciencias Agrícolas, Turrialba, 12 May 1965, *Lent, R.W. 582* (MO); Turrialba, Florencia Norte, CATIE, Suelo: Serie Colorado, 30 Nov 1981, *Poveda Á, L.J. 3097* (CR); Jiménez, Distr. Pejibaye, Fila Alto Velo de Novia, 22 Apr 2006, *Santamaría, D. & Morales Q, J.F. 4273* (CR); Vicinity of Pejivalle, 7 Feb 1926, *Standley, P.C. & Valerio, J. 46920* (US); Turrialba, Cultures et lieux incultes à Turrialba, 1 Nov 1893, *Tonduz, A. s.n.* (CR); Turrialba, 1 Nov 1893, *Tonduz, A. 8358* (CR); Turrialba, Dist. Tayutic, Jicotea de Chirripó, orilla de sendero hacia las cabañas, 29 Nov 1994, *Umaña, G. et al. 624* (CR). **Guanacaste**: Tilarán, 12 Apr 1938, *Acosta, M.A. s.n.* (CR); Bagaces, Distr. Bagaces, Estacion Palo Verde, camino hacia el Rio Tempisque, 17 Sep 2000, *Chavarría, U. 2080* (CR); Liberia, Distr. Mayorga, Estacion Cacao, 25 Nov 1990, *Espinoza, R. 85* (CR); Abangares, Dist. Sierra, R.B. Monteverde. Cerro Amigos in area of TV towers, cloud forest, 10 Oct 1985, *Haber, W.A. & Bello, E. 3438* (CR); Tilarán, Zona Monteverde, 2 km N Las Nubes, Atlantic slope, Quebrada Azul (tributary of Río Chiquito), 10 Nov 1990, *Haber, W.A. & Ivey, C. 10149* (INB); V. biological station on the W flank of Volcan Cacao, area de Consevacion

Guanacaste, 24 Jul 1994, *Kress, W.J. & Sawyer, N.W. 94-4995* (CR, US); Liberia, Dist. Nacascolo, P.N. Santa Rosa. Rocky, dry river between Manilkara forest and fork in road to Naranjo Beach. Río Poza Salada 5 to 20 m alt, 22 Jan 1978, *Liesner, R.L. 4397* (CR); Santa Cruz, 29 Nov 1980, *Ocampo, R.Á. 3574* (CR); Bagaces, Distr. Bagaces, Finca Monteverde, 9 Jul 1996, *Ronchi, P.S. & Frankie, G. 461* (CR); Bagaces, Distr. Bagaces, Finca Monteverde, 9 Jul 1996, *Ronchi, P.S. & Frankie, G. 710* (CR). **Heredia**: Heredia, La Selva Biological Station; seed #92-4, LB DNA #70, 30 Mar 1992, *Bohs, L. 2400* (UT); Sarapiquí, Dist. Las Horquetas, Z.P. La Selva. Original forest with areas over 30 m. tall near the Río Puerto Viejo about 2 km. upstream from the confluence with the Río Sarapiquí on the O. T. S. La Selva research area, 26 Apr 1973, *Gentry, J.L. & Burger, W.C. 3018* (CR); Santo Domingo, Distr. Tures, Charrales y potreros por el Río Tures, 8 Sep 2003, *Hammel, B. & Pérez, I. 22881* (CR); Barva, Dist. Barva, Potrero en Barva, 28 Sep 1940, *León, J. s.n.* (CR); Sarapiquí, Dist. Puerto Viejo, Z.P. La Selva. Secondary Cut II. Secondary Pioneer, 24 Jun 1972, *Opler, P.A. 906* (CR); Sarapiquí, Dist. Puerto Viejo, Z.P. La Selva. Secondary pioneer. 3 km SE of Puerto Viejo, 8 Dec 1972, *Opler, P.A. 1605* (CR); Sarapiquí, Distr. Horquetas (Buenos Aires), Finca CECANFOR, Sarapiquí, 18 Oct 2006, *Soto, A. et al. 1362* (CR); Santo Domingo, Dist. Santo Tomás, Pont du rio Virilla, près San Juan, 1 Jun 1896, *Tonduz, A. s.n.* (CR); Sarapiquí, Dist. Puerto Viejo, Z.P. La Selva. Finca La Selva. The OTS Field Station on the río Puerto Viejo just E of its junction with the río Sarapiquí. Between Sendero Tres Rios Trail and the 200 m marker of the sendero Las Vegas, 7 Jun 1995, *Whitson, M.K. 2* (CR); Sarapiquí, Dist. Puerto Viejo, Z.P. La Selva. The OTS Field Station on the río Puerto Viejo just E of its junction with the río Sarapiqui. Weedy edge of recently established plantation along Sendero Tres Ríos at about the 2000 m point, 4 Jan 1995, *Wilbur, R.L. 63114* (CR). **Limón**: Port Limon [Puerto Limón], Zent banana farm, 2 May 1903, *Cook, O.F. & Doyle, C.B. 460* (US); Talamanca, Distr. Bratsi, P. I. La Amistad. Quebrada La Catarata. Camino a Río Coen. A orilla de la quebrada, 21 Feb 2007, *Santamaría, D. & Monro, A.K. 5783* (CR); Sables du rio à Sipurio, (Talamanca), Apr 1894, *Tonduz, A. 8711* (US); Zent y la Estrella, *United Fruit Company 228* (US). **Puntarenas**: Golfito, R.F. Golfo Dulce, Penínusal de Osa. La Palma, trocha La Tarde, 2 Km arriba unión Quebrada La Tarde con Río Rincón, 23 Apr 1993, *Aguilar, R. 1744* (INB); Monteverde. In village along road from Pension Manakin heading towards the dump, 5 Aug 1994, *Alverson, W.S. & Tuxill, J.L. 3856* (US); Puntarenas, Distr. Monteverde, Cerro Plano. Finca de Marcos Vargas, 21 Aug 1991, *Bello, E. 2986* (CR); Puntarenas, R.B. Monterverde, Cordillera de Tilarán. Monteverde. Bosque Sapo Dorado, 23 Mar 1993, *Bello, E. 4945* (INB); Aguirre, Dist. Savegre, Dos Bocas, propiedad de Oldemar Chinchilla, 31 Aug 2012, *Chinchilla A, I.F. 90* (CR); Aguirre, Dist. Savegre, Dos Bocas, propiedad de Oldemar Chinchilla, 14 Nov 2012, *Chinchilla A, I.F. 217* (CR); R.B. Monteverde. Cordillera de Tilarán. Provincias de Alajuela, Puntarenas y Guanacaste. Comunidad, 24 Apr 1977, *Dryer, V.J. 1312* (CR); Puntarenas, Monteverde, San Luis Valley, 1-2 km downstream from village of San Luis along Río Guacimal. José Rojas' farm, 30 May 1992, *Haber, W.A. & Joyce, F. 11198* (MO); Puntarenas, Distr. Monteverde, Valle de Rio San Luis, Guacimal. Bosque muy humedo premontano, 11 Feb 1995, *Haber, W.A. & Zuchowski, W. 11891* (CR, MO); Santa Rosa National Park, 30 km Northwest of Liberia, 1976, *Hackforth-Jones, J. 396* (MO); Coto Brus, Distr. Sabalito, Finca Cafrosa. Sabalito, 21 Oct 1990, *Mora, G. 124* (CR); Osa, Distr. Sierpe, Rancho Quemado. Alrededores de la toma de agua. Bosque primario. Rincon, 15 Feb 1991, *Quesada, J. 457* (CR); Osa, Dist. Sierpe, P.N. Corcovado. Rincón. Por las filas de la cuenca superior de la Quebrada Vaquedano, 15 Feb 1991, *Quesada, J.F. 457* (CR); Between Golfo Dulce and Río Térraba, Dec 1947, *Skutch, A.F. 5331* (CR, F, US); Graviers du Río Ceibo à Buenos Aires, 1 Jan 1892, *Tonduz, A. s.n.* (CR); Río Ceibo en Buenos Aires, 1 Jan 1892, *Tonduz, A. 6605* (CR); Puntarenas, Distr. Isla del Coco, Isla del Coco, 7 Nov 2001, *Trusty, J. & Kesler, H. 456* (CR). **San José**: kurz ostlich der Ciudad Universitaria, in San Pedro, 22 Jul 1988, *Döbbeler, P. 1072* (BM, W); 24 Mar 1947, *Echeverría, J.A. 1015* (CR); Montes de Oca, Dist. Mercedes, Campus of Universidad de Costa Rica, 23 Aug 1964, *Eickwort, G. 7* (CR); Weedy vacant lot, near calle 30 Av. 3, 16 Sep 2001, *Gargiullo, M.B. 902* (CR); North of Calle 3, 9 Jan 1984, *Khan, R. et al. 23* (BM, CR); Coto Brus. Cerca de la puente del Rio Terraba en via a la Jardin Biologica Wilson, 10 Jul 1994, *Kress, W.J. & Runk, J.L. 94-4666* (US); Mora, Distr. Colón, Canon Rio Quebrada Honda, 13 Aug 2002, *Kriebel, R. 195* (CR); Montes de Oca, Dist. Sabanilla, Ca. 5 km östl. San Jose, aufgelassenes Finca Gelände (Kaffee), 17 Feb 1971, *Kuhbier, H. 51* (CR); Acosta, Distr. Guaitil, Cangrejal, ribera del Rio Candelaria, 28 Jul 2002, *Morales Q, J.F. 8597* (CR); Acosta, Distr. Sabanillas, Aguabuena, Quebrada Laja, 24 Sep 2003, *Morales Q, J.F. et al. 9942* (CR);

Desamparados, Distr. Desamparados, San Jose, Desamparados, 2 Jan 2007, *Morales Q, J.F. 14736* (CR); Desamparados, Distr. Desamparados, San Jose, Desamparados, 2 Jan 2007, *Morales Q, J.F. 14748* (CR); Dota, junction of road to Savegre and Panamerican Highway at bus stop, 28 Jun 2008, *Moran, R.C. & Stern, S. 7781 [b]* (BM); Mora, Distr. Tabarcia, San Ignacio, 12 Dec 2001, *Murillo R, F. 276* (CR); San José, between Calle 11 and 13, near Avenida 24, 20 Feb 1971, *Nee, M. & Mori, S. 3494* (MEXU); Tibás, 1 Dec 1975, *Ocampo, R.Á. 1145* (CR); Tibás, 1 Oct 1976, *Ocampo, R.Á. 1595* (CR); Bord des chemins á San José, May 1890, *Pittier, H.F. 2543* (US); Mora, Dist. Colón, El Rodeo de Pacaca, 2 Jan 1981, *Pittier, H.F. 3271* (CR); Dota, Distr. Copey, A orilla de la carretera y margenes del rio, 17 Apr 1998, *Rodríguez G, A. et al. 3297* (CR); P.N. Chirripó, Sendero a Base Crestones. El Jardín, Km 13, 18 Jul 2017, *Sánchez G, J. 2784* (field observation only); Santa Ana, Distr. Salitral, Santa Ana, Salitral. La Cuesta Blanca. Camino que lleva a Pabellón, a orillas del camino, 23 Apr 2005, *Santamaría, D. 1844* (CR); Escazú, Distr. San Rafael, Guachipelín, Escazú, 30 Jan 2005, *Solano, D. 1780* (CR); San José, Dist. Hatillo, 4 Jan 1935, *Solís, F. 33* (CR); Montes de Oca, Dist. San Pedro, 31 Dec 1973, *Solomon, J.C. 692* (CR); Turrubares, Distr. San Luis, San Luís, Finca de Melvin Chavarría, 5 Oct 2004, *Soto, A. 158* (CR); Turrubares, Dist. San Juan de Mata, No protegida. Cuenca del Tárcoles. San Luis de Turrubares, finca de Melvin Chavarría, 5 Oct 2004, *Soto, A.D. 158* (CR); Vicinity of Escasú, 29 Jan 1924, *Standley, P.C. 32374* (US); Cerro de Piedra Blanca, above Escasú, 31 Jan 1924, *Standley, P.C. 32644* (US); along the Río María Aguilar, near San José, 25 Mar 1924, *Standley, P.C. 39022* (US); P.N. Braulio Carrillo. La Montura. Lower montane rain forest, 25 Jul 1982, *Todzia, C.A. et al. 1988* (CR); San José, Dist. Mata Redonda, Au bord de la ruote entre San José et Escazú, 21 Sep 1889, *Tonduz, A. s.n.* (CR); San José, Dist. Pavas, Bords des chemins à San José, 1 Oct 1890, *Tonduz, A. s.n.* (CR); San José, Bords des chemins, cultures, lius in cultes, 25 Mar 1890, *Tonduz, A. s.n.* (CR); Jardin Chase á S. José, 16 Sep 1889, *Tonduz, A. 148 b* (US); Lieux incultes etc. à San José, Nov 1892, *Tonduz, A. 1537* (US); sin. loc., 1 Oct 1890, *Tonduz, A. 3041* (CR); defrichements du Roble, massif del Irazu, Jul 1891, *Tonduz, A. 4237* (BM); Vázquez de Coronado, Distr. Cascajal, P. N. Braulio Carrillo. Estación Zurquí, 16 Nov 2005, *Vargas, L.D. & Villalobos, G. 1056* (CR).

CUBA. In Insula Cuba, 1840, *Poeppig, E.F. s.n.* (BM). **Ciudad de la Havana**: Isla de los Pinos, Cayo Cantiles, Mar 1967, *Bisse, J. & Rojas s.n.* (HAJB); orillas del rio Guanabo, cerca de la via Blanca, 4 Jun 1970, *Bisse, J. & Lippold, H. s.n.* (HAJB); Alquizar, sabana humeda cerca de la playa Guanimar, May 1971, *Bisse, J. & Lippold, H. s.n.* (HAJB); Jibacoa, Sierra de Camarones, 2 Oct 1971, *Lippold, H. s.n.* (HAJB). **Granma**: a lo largo del camino de Minas del Frio a Mompie, 23 Mar 1978, *Bisse, J. s.n.* (HAJB); Sierra Maestra, Pico Bayamesa, falda norte, 19 Mar 1970, *Lippold, H. s.n.* (HAJB). **Guantánamo**: Palenque, cerca de Cayo Fortuna, Apr 1972, *Álvarez, A. s.n.* (HAJB). **La Habana**: Santiago de las Vegas, 18 Jan 1905, *Hermann, H.A. van*, *527* (F); San Antonio, 25 Feb 1905, *Hermann, H.A. van*, *633* (BM, F, K); Morro Hill, Santiago, 3 Feb 1899, *Millspaugh, C.F. 1082* (F); In Cuba Orientali, 1856, *Wright, C. 383* (K, MO). **Holguín**: Camino desde La Moa hacia La Melba, *Lippold, H. s.n.* (HAJB). **Matanzas**: San Miguel de los Banos, Lomas al sur de la Loma Jacan, principalmente cerca de arroyos, 25 Oct 1979, *Bisse, J. s.n.* (HAJB). **Pinar del Río**: Santa Cruz de los Pinos, barranca del rio Taco-Taco, cerca de El Retiro, Dec 1974, *Bisse, J. & Klotz s.n.* (HAJB); La Palma, altiplano de Cajalbana, entre El Tecnologico y la torre de TV, 20 Jan 1981, *Bisse, J. & Beurton s.n.* (HAJB); La Palma, Cajalbana, entre el Technológico y la torre de televisión (coordenadas de la cuadricula Cuba 1:250000 [33/24]), 20 Jan 1981, *HFC (Herbario de Flora de Cuba)*, *43449* (B). **Sancti Spíritus**: Trinidad Mountains. San Blas-Buenos Aires. Las Villas province, *Gonzales, A. 583* (BM); Trinidad Mountains. San Blas-Buenos Aires. Santa Clara Province, Aug 1941, *Howard, R.A. 6558* (BM). **Santiago de Cuba**: Sierra Maestra, El Uvero, Loma Liberia, *Bisse, J. & Lippold, H. s.n.* (HAJB); Sierra Maestra, Manguito, pinares de la loma La Botella, 22 Mar 1970, *Lippold, H. s.n.* (HAJB); Vicinity of Santiago City, 15 Feb 1902, *Palmer, E. 363* (MO). **Villa Clara**: Caibarién, Cayo Santa Maria (parte oriental), sendero ecológico El Bago, 20 Feb 2005, *Greuter, W. 26375* (HAJB).

DOMINICA. Stream-cut, wooded gorge lying between the estate house and orange plantations. Sylvania Estate, 11 Aug 1938, *Hodge, W.H. 799* (BM); Castle Bruce, From the village of Petite Soufrière. Growing with grasses on a southeast-facing slope near Martina Warrington's garden in

Lawivier Loui, 5 Aug 1994, *Quinlan, M.B. 51* (UMO); Castle Bruce, Growing as a weed in Catherine Durand's flower garden on sunny E-facing slope of Mt. Piton, Petite Soufriere, 28 May 1998, *Quinlan, M.B. 182* (UMO); Trafalgar, St. George. On cleared ground under citrus, 3 Apr 1965, *Shillingford, C.A. 209* (MO); Windward Is: Dominica; South-east coast. Delices, 19 Sep 1983, *Whitefoord, C. 3678* (BM). **Saint David**: "Windward Coast", 1905, *Bryant, E.M. s.n.* (K). **Saint George**: Botanic Gardens, *Jones, J. 1* (K). **Saint Paul**: Brantridge Estate near Pont Casse, 17 Jan 1969, *Filipps, R. De*, *164* (BM).

DOMINICAN REPUBLIC. **Azua**: Sierra de Ocoa, San José de Ocoa, 25 Mar 1929, *Ekman, E.L. H-12038* (K); Between Azara and Barahona, Feb 1971, *Votava, F. & Liogier, A.H. 36* (MO). **Barahona**: Montiada Nueva, forested hillslopes S.E. of Polo, 21 Aug 1946, *Howard, R.A. & Howard, E.S. 8506* (BM); Rep. Dominicana: Sierra de Baoruco: Prov. Barahona: 8.5 km. de La Cienaga: en el camino a Aguita Blanca y El Platón: límite de zona cafetalera, 22 May 1984, *Zanoni, T.A. et al. 30178* (MO). **Distrito Nacional**: Distrito Nacional; S side of highway (Santo Domingo to San Pedro Macoris) between towns of Guayacanes and Juan Dolio; shallow soil over marine coral limestone, used to farm, 4 Mar 1981, *Zanoni, T.A. et al. 11466* (MO). **Espaillat**: Rocky slopes of serpentine limestone hill, coast road ca 35 E of junction with Puerto Plata--Santiago road, 25 Apr 1970, *Burch, D. 2401* (MO). **La Vega**: Stream banks and hillsides between Constanza and Valle Nuevo, 30 Apr 1970, *Burch, D. 2535* (MO); Along road between Constanza and Valle Nuevo, 19.3 km S of Constanza, 3.8 km S of El Convento, 3 Jul 2006, *Croat, T.B. 97618* (MO); Steep wet ravine with mature secondary growth, 2.5 km w of Duarte Highway on road to El Rio, 29 Jun 1968, *D'Arcy, W.G. & Hoffman, C. 2597* (MO); 5.1 km S of Jarabacoa on road to El Rio, 30 Jun 1968, *D'Arcy, W.G. & Hoffman, C. 2619 A* (MO); A few plants in shade of pines, above Hotel Nueva Suiza, Constanza, 1 Jul 1968, *D'Arcy, W.G. & Hoffman, C. 2624* (MO); Beside abandoned vegetable patch above Hotel Nueva Suiza, Constanza, 1 Jul 1968, *D'Arcy, W.G. 2625* (MO); On shale landslide 37 km NW of Ocoa on road to Constanza (at Nuez), 1 Jul 1968, *D'Arcy, W.G. & Hoffman, C. 2638* (MO); Station 9024-Jarabacoa, 5 Oct 1977, *Liogier, A.H. 9024-14* (MO). **María Trinidad Sánchez**: María Trinidad Sánchez, Moist hillside and stream bank ca 2 km SE Abreu, 25 Apr 1970, *Burch, D. 2379* (MO). **Monte Cristi**: Puerto Libertador, near Puerto-Libertador, Manzanilla Bay, 25 Oct 1946, *Howard, R.A. & Howard, E.S. 9640* (BM). **San Juan de La Maguana**: Cañafistol, Jul 2005, *Schäffler, Y. 55* (W). **Santiago**: Scrub area above roadside cut, Santiago-Puerto Plata road ca 3 km N of Benito Martinez, 24 Apr 1970, *Burch, D. 2336* (MO); Loma Bajita, 12 Jun 1933, *Valeur, E.J. 1021* (K). **Santiago Rodríguez**: Moncion, 25 May 1930, *Valeur, E.J. 138* (F, MO).

EL SALVADOR. Cerro el Candelero. Dulce Nombre de María. Chalatenango, 25 Aug 2006, *Menjívar, J. et al. 415* (N/A); **Cuscatlán**: Cojutepeque, CA-1, ca. 3 mi W of Cojutepeque, 23 Sep 1978, *D'Arcy, W.G. 12125* (MO); **La Libertad**: Cantón Primavera, Finca Colombia, Quetzaltepeque, 2 Feb 1998, *Monro, A.K. et al. 2916* (BM).

GRENADA. **Grenada**: Saint David, Along road between Perdmontemps and Windsor Forest, 10 Nov 1957, *Proctor, G.R. 16962* (BM).

GUADELOUPE. sin. loc, 1849, *Duchassaing, E.P. s.n.* (GOET); sin. loc, *Duchassaing, E.P. s.n.* (W); sin. loc., *Hahn, L. 325* (BM); sin. loc., *Shakespear, R. s.n.* (BM). **Basse-Terre**: Mts. Carbea, Morne Grande Veute, 25 Apr 1974, *Sastre, C. et al. 2708* (MO). **Marie-Galante**: Ravine of Riviere de St. Louis between Grand Bassin and Les Balisiers, 4 Jun 1960, *Proctor, G.R. 21179* (BM).

GUATEMALA. **Alta Verapaz**: vicinity of Secanquim, 8 Jan 1905, *Maxon, W.R. & Hay, R. 3230* (NY). **Petén**: Westufer des Lago Peten Itza, umbegung der Villa Maingua (S Aserradero Maingua S.A.), ca. 1 km W Zeentrum von San Andrés, 1 Apr 1991, *Frisch, R.O. 133* (W); Parque Nacional, Tikal, Santa Elena, en orillando el camino para San Andrés, Km 15, 28 May 1970, *Ortíz, R.T. 1168* (MO); Tikal, Parque Nacional. En calzada entre Templo Nº 4 y grupo 'H' en Ramonal/Zapotal, 9 Jan 1969, *Tun Ortíz, R. 34* (BM); Santa Elena, en orillando el camino para San Andrées, a km 15, 28 May 1970, *Tun Ortíz, R. 1168* (NY, US); San Luis, en orillando el camino para Poctun, en el km. 119, aprox. 200 m. del camino, lado saliente, 7 Dec 1970, *Tun Ortíz, R. 1490* (US). **Retalhuleu**:

Retalhuleu, Oct 1877, *Bernoulli, K.G. & Cario, R. 2346* (GOET); Retalhuleu, Mar 1877, *Bernoulli, K.G. & Cario, R. 2379* (GOET); Retalhuleu, Oct 1874, *Bernoulli, K.G. & Cario, R. 2386* (GOET). **Sacatepéquez**: Santa Lucia Milpas Altas, Cuesta "Las Cañas", 3 Dec 1992, *Castillo, J.J. & Luarca, R. 1653* (MO); San Miguel Dueñas, 8 Aug 1992, *Véliz, M. 92-2183* (MEXU). **Suchitepéquez**: Chojija, prope Mazatenango, 1860, *Bernoulli, K.G. & Cario, R. 2406* (GOET).

HAITI. Bords de la route arête derriére de Port-au-Prince, 27 May 1979, *D'Arcy, W.G. 13386* (MO); Near Petite Source, Mornes des Commissares, 17 Apr 1942, *Holdridge, L.R. 1139* (BM).

HONDURAS. **Comayagua**: Comayagua, ribero del Río Tujaca, 4 Mar 1971, *Hernández R, J. & Hernández M, M. 5334* (MEXU, MO); Siguatepeque, 5 Jul 1936, *Yuncker, T.G. et al. 5670* (K, MO). **Francisco Morazán**: Montaña La Tigra, 20 km de Tegucigalpa, 1 Sep 1981, *Belibasis, L. de, 58* (MEXU); Distrito Central, Tegucigalpa, El Hatillo, 3 Sep 1978, *Díaz Z, A.L. 193* (MO); El Zamorano, Horticulture Dept. EAP, 5 Jan 2001, *Molina R, A. & Molina, A.R. 35125* (MEXU); Colonia el Hogar, SO de Tegucigalpa, 29 May 1982, *Rodríguez, R. 185* (MEXU); Tegucigalpa, alrededores Ciudad Universitaris, 26 May 1978, *Romero, E. 70* (BM, MEXU); Santa Lucía, 12km NE de Tegucigalpa, 1 Jun 1988, *Vásquez, E. 192* (BM). **La Paz**: San Pedro de Tutule, Alrededores de Tutule [San Pedro de Tutule], 18 Apr 1981, *Medina C, M.A. 228* (MO). **Olancho**: Along Río Olancho, on road between San Estéban and Bonito Oriental, 14.8 mi NE of San Estéban, 7 Feb 1987, *Croat, T.B. & Hannon, D.P. 64402* (MO); Catacamas, Alrededores de Catacamas, 7 Apr 1971, *Hernández M, M. & Rendón, G. 5473* (MO).

JAMAICA. sin. loc, *Dancer*, *s.n.* (K); "Moneague", *Prior, A. s.n.* (K). **Hanover**: Lucea, 1 Mar 1891, *Hitchcock, A.S. s.n.* (MO). **Manchester**: Banana Ground, 20 Oct 1962, *Adams, C.D. 11774* (BM); Marshalls Pen, 2.25 miles due NE of Mandeville, 31 Mar 1964, *Proctor, G.R. 24759* (BM); Retford Pen, near Mandeville, 25 Feb 1956, *Stearn, W.T. 365* (BM). **Portland**: Mouth of the Rio Grande, by roadside on E. bank, 18 Mar 1956, *Stearn, W.T. 542* (BM). **Saint Andrew**: 19 Begonia Drive, Mona Heights, 16 Jan 1960, *Adams, C.D. 6145* (MO); Gordon Town, Gordontown, *Ball, J. s.n.* (E); Hope Gardens (?), St Andrew, 8 Mar 1915, *Harris, W. 11898* (BM, F, MO); Hamstead, St Andrew, Jan 1927, *Maxwell, I. s.n.* (BM); Warekia, summit of Long Mountain, 3 Jan 1956, *Proctor, G.R. & Stearn, W.T. 11443* (BM); Gorge east of the Hog Hole River, 1.25 miles due east of Gordon Town, 17 Nov 1958, *Proctor, G.R. 18369* (BM); Along a road between St. Peters and Content Gap, Port Royal Mts, 17 Jun 1963, *Proctor, G.R. 23681* (BM). **Saint Ann**: 2 mi. S of Alderton, vicinity of York Castle, 7 Jul 1966, *Crosby, M.R. 2905* (MO); 2.2. miles west by road of Albion, 9 Aug 1965, *Hespenheide, H.A. 932* (MO); sin. loc, *MacFadyen, J. s.n.* (K); Queen's Highway, about 1 mile W. of Dry Harbour, in disturbed soil by roadside, 16 Feb 1956, *Stearn, W.T. 271* (BM); Winefield, between Claremont and St. Ann's Bay, 21 Feb 1956, *Stearn, W.T. 330* (BM); Averham Park, N.W. of Claremont, by path through pasture, 27 Mar 1956, *Stearn, W.T. 584* (BM). **Saint Elizabeth**: Giddy Hall, St. Elizabeth, Mar 1926, *Maxwell, I. s.n.* (BM); Balaclava, Mar 1928, *Orcutt, C.R. 594* (BM); Giddy Hall -Parish of St. Elizabeth, Feb 1936, *Sangster, I. s.n.* (BM); N. of Accompong, in cultivated ground, 29 May 1956, *Stearn, W.T. 1000* (BM); Roadside weed, about 2 miles east of White House, 22 Jan 1958, *Yuncker, T.G. 18015* (BM). **Saint James**: Orange Road, Montego Bay. St James, 1924, *Norman, C. 119* (BM); **Saint Thomas**: Yallah's Hill, NW slope, 7 Apr 1956, *Stearn, W.T. s.n.* (BM); Garden of Whitfield Hall, 8 Jan 1956, *Stearn, W.T. 113* (BM); Gorge of Plantain Garden River N.N.W. of Whitehall, along side of path, 15 Mar 1956, *Stearn, W.T. 483* (BM). **Trelawny**: Troy, 8 May 1903, *Harris, W. 8538* (BM); Dornoch pumping station, Rio Bueno, by roadside, 1 Feb 1956, *Stearn, W.T. 181* (BM, K). **Westmoreland**: 2.5 miles W.N.W. of Hopewell, Cairn Curran district, at roadside, 9 Mar 1956, *Stearn, W.T. 432* (BM).

MARTINIQUE. Pointe de Jaham, parking de la DEAL, Commune de Schoelcher, 7 Feb 2014, *Delnatte, C. 3039* (BM); Barrage de La Manzo, Commune du François, 3 Nov 2015, *Delnatte, C. 3484* (BM); vallée de St. Pierre, Jun 1870, *Hahn, L. 325 [a]* (K); sin. loc, *Sieber, F.W. s.n.* (W).

MEXICO. sin. loc, *Berlandier, J.L. 504* (BM, W); sin. loc, *Berlandier, J.L. 586* (BM, W); sin. loc., *Kruse, H. 1028* (MEXU); Gomez Farias, 11 Jun 1977, *Neck, J. 72 B* (DNA); sin. loc., *Schiede, C.J.W.*

46 (MO); sin. loc, *Schmitz, A. 61* (W); sin. loc., *Torres C, R. 15288* (MEXU); Lobos, *Wawra, H. 719* (W); sin. loc., *Without Collector s.n.* (MO). **Baja California**: Ensenada, Isla Guadelupe, 1893, *Franceschi, F. s.n.* (US); Isla Guadalupe, 1892, *Franceschi, F. 16* (IND, K); Primiera Agua, near Loreto, Flora of Lower California, 19 Oct 1930, *Jones, M.E. 27342* (NY); Ensenada, Isla Guadelupe, canyon above lobster camp, 16 Dec 1957, *Moran, R. 6448* (K, MEXU); Isla Guadalupe, 1875, *Palmer, E. 60 [b]* (BM, MO, NY, NY, UPS, YU); Isla Guadalupe, 1889, *Palmer, E. 860* (K, US); Ensenada, 5 miles N of San Quintin, 24 Mar 1974, *Taylor, J. & Taylor, C. 15637* (US); Ensenada, Isla Guadelupe, Mesa and slope 1 mile inland from Lobster Camp, 26 Apr 1958, *Wiggins, I.L. & Ernst, W.R. 126* (US); Rancho San Jacinto, 45 miles S of Ensenada, 7 Sep 1930, *Wiggins, I.L. & Demaree, D. 4731* (US); Arroyo Mulege, just below dam west of village, 16 Apr 1963, *Wiggins, I.L. & Wiggins, D.B. 18265* (MEXU). **Baja California Sur**: La Paz, Sierra de La Victoria, arroyo cerca del Rancho La Palmilla, km 8 ca. a San Antonio de la Sierra, 16 km al SE del poblado de San Atonio, 10 Dec 2003, *Domínguez Cadena, R. 2936* (MEXU); Santiago, 8 Mar 1989, *Encarnación B, R. 71* (MEXU); Comondú, Las Cuevitas, below Comondú, 17 Feb 1939, *Gentry, H.S. 4232* (K, MO); Todos Santos, Todos Santos, Lower California, 19 Feb 1923, *Jones, M.E. 24119* (MO); Rancho San Gregorio, Cerro San Francisco, ca. 30 miles N of San Ignacio, 23 Dec 1987, *Mason, C.T. 3875* (MEXU); Arroyo San Juan, 14 Feb 1973, *Moran, R. & Reveal, J.L. 20118* (US); east side of San José del Cabo, irrigated area, 7 Jan 1959, *Moran, R.V. 6914* (K, MEXU); Santo Domingo, 26 Sep 1905, *Nelson, E.W. & Goldman, E.A. 7176* (US); Collected in area of stabilized dunes about 5 miles south of San Quintin Mex, 24 Mar 1974, *Taylor, R.J. & Taylor, C.E.S. 15637* (MO); Rancho Viejo, 0.5 mi W on road to San Javier, 28 mi E of main road, 17 Dec 1959, *Wiggins, I.L. & Ernst, W.R. 547* (MEXU, US); Los Cabos, San Juan del Cabo, patio of Aduana, 7 May 1931, *Wiggins, I.L. 5678* (US). **Campeche**: Calakmul, Ejido Narciso Mendoza, a 1 km del poblado, carretera Xpujil-Narciso, 25 Aug 1997, *Álvarez M, D. 303* (MEXU, MO); Calakmul, a 5.5 km al SE del ejido La Moza camino a San Antonio Soda, 19 Feb 2003, *Álvarez, D. & Ramírez, A. 4038* (MEXU); Hopelchén, 3.86 km al E de X-Canha, 2 Apr 2004, *Álvarez, D. & Ramírez, A. 8726* (MEXU); Calkiní, 4 km al oeste de Tunkasche, sobre el camino Calkinia-Punta Arenas, 25 Mar 1988, *Cabrera, E. & Cabrera, H. de 15867* (MEXU); Champotón, Yohaltun, 31 Jan 1981, *Chan, C. et al. 177* (CICY); Champotón, San Antonio-Pustunich, 10 Apr 1981, *Chan, C. & Burgos, H. 352* (CICY); Carmen, Punta Zacatal a Puerto Rico, 23 Mar 1985, *Chan, C. 4924* (CICY); Calkiní, 5.5 km al oeste del Peten El Remate, 27 Nov 1985, *Durán, R. & Chan, C. 1425* (CICY); candelaca, aprox. 67km al s-sw de Escárcega, Mpio, de Escárcega, 30 May 1988, *Flores C, A. & Ramírez D, R. 718* (MO); Champotón, Tuxpeña, 14 Jan 1932, *Lundell, C.L. 1185* (US); Calakmul, a 2 km al SE de Dos Naciones, 27 Oct 1997, *Martínez S, E. et al. 29419* (MEXU); Calakmul, a 2 km al SE de Dos Naciones, 27 Oct 1997, *Martínez S, E. et al. 29430* (MEXU). **Chiapas**: Ocosingo, a 0.57 km al SO del crucero de Bonampak, 15 Oct 2003, *Águilar M, G. et al. 8184* (MEXU); Amatenango de la Frontera, 22 km S of Frontera Comalapa, 21 Nov 1980, *Breedlove, D.E. & Almeda, F. 47646* (MEXU); Ocozocoautla de Espinosa, Reserva del Ocote, La Cueva, al NW del rancho Corocito, 29 Apr 1983, *Calzada, J.I. et al. 9745* (MEXU); Ocosingo, 500 m al sur de Lacanjá-Chansayab, 9 Oct 1990, *González-Espinosa, M. et al. 1185* (MEXU); Ocosingo, Marqués de Comilla, a 6 km al SE de Benemerito de la Americas, con rumbo a Flor de Cacao, 8 Oct 1984, *Martínez S, E. 8044* (MEXU); Villa Corzo, El Jardín, 6 May 2000, *Negrón Bonilla, L.A. 2* (MEXU); Pueblo Nuevo Solistahuacán, Clinica Yerba Buena, 2 km NW of Pueblo Nuevo Solistahuacán, 23 Jan 1965, *Raven, P.H. & Breedlove, D.E. 19912* (US); Mpio. Villa Flores, Loc. En el predio nicaragua. 16o17'45N 93o 32'37W. Veg. Selva mediana perennifolia, a la orilla del arroyo Nicaragua, con Ficus spp. Inga, Enterolobium cyclocarpum, 3 Jun 2002, *Reyes García, A. & Gómez, D. 4947* (MEXU, MO); Ocosingo, En las orillas de la Colonia Benito Juárez Miramar, 20 Aug 1993, *Reyes-García, A. & Sousa S, M. 2064* (MO); San Juan Cancuc, Chiapas. San Juan Cancuc. Tz'utoj Te'el, 12 km al noreste del poblado. Arroyo caliente, 22 Mar 1988, *Sántiz C, E. 523* (CR); Villa Corzo, Arroyo La Vainilla, 6 Aug 2000, *Vázquez Hernández, J.P. 351* (MEXU); Tuxtla Chico, Talisman, 19 Aug 1985, *Ventura, E. & López, E. 2273* (MEXU); Unión Juárez, Ejido 11 de Abril, 16 Apr 1986, *Ventura, E. & López, E. 3468* (MEXU). **Chihuahua**: Batopilas, Sierra Madre Occidental. Along Arroyo Samachique, between Rio Batopilas and Wimivo, on N side of Barranca de Batopilas between La Bufa and Batopilas, 29 Dec 1973, *Bye, R.A. 6067* (MO); Batopilas, Arroyo San Fernando, W of La Bufa, on S side of Barranca de Batopilas, 26 Dec 1976, *Bye, R.A. 7330* (MEXU); Batopilas, La Bufa region, 26 Dec 1976, *Bye, R.A. 7334* (MEXU); Batopilas, near junction of Arroyo Wimiyo

and Río Batopilas, on N side of Barranca de Batopilas, W of La Bufa, 28 Dec 1976, *Bye, R.A. 7346* (MEXU); La Bufa. In garden of Jeff Chandler, 1 Jul 1977, *Davis, T. 714* (MO). **Colima**: Islas Revillagigedo, Socorro Island, north of Huerta Grande, north of Laguna Escondida, 6 Apr 1981, *Moran, R.V. 29534* (MEXU); Cuauhtémoc, Predio San Juan, a 2 km al SE del Población de Cuauhtemóc, 10 Jan 1990, *Román, M.L. & Amezcua, V. 1267* (MEXU); Islas Revillagigedo, Isla Socorro, lado SE, 10 Aug 1987, *Toriz A, G. & Campos V, A. 716* (MEXU, MO). **Distrito Federal**: Milpa Alta, El Mirador al S de Milpa Alta, Mex 142 (Cd. de Mexico-Oaxtepec) km 24, 25 Aug 1991, *Bye, R.A. & Linares, E. 18188* (MEXU); Xochimilco, Parque Ecológico Xochimilco, cerca de las 7 Fuentes, 20 Apr 1992, *Bye, R.A. 18421* (MEXU); Coyoacán, Cuidad Universitaria, Universidad Nacional Autonoma de Mexico, Jardin Botanico Exterior, 16 May 1992, *Bye, R.A. 18436* (MEXU); Cuemanco, 24 Sep 2004, *Carranza Batista, H. (1) 3* (MEXU); Milpa Alta, Tecoxpa, Calle España, 22 Apr 1977, *Espinosa Garcia, F.J. 197* (MEXU); Xochimilco, Xochimilco, 30 Aug 1977, *Espinosa Garcia, F.J. 243* (MEXU); Xochimilco, Paraje El Acuario, San Gergorio Atlapulco, Area Natural Protegida Ejidos de Xochimilco, 13 May 2005, *Espinosa H, A. et al. 47* (MEXU); Xochimilco, 25 Apr 1965, *Hernández, H. s.n.* (US); Xochimilco, Cienaga Grande, 14 Sep 2007, *Martínez S, E.M. 39832* (MEXU); San Luis Tlaxialtemalco-Cerranía, 17 Nov 1992, *Nava Rodríguez, V. 80* (MEXU); Ciudad Universitaria, jardinera junto al estacionamiento del Instituto de Biología, cerca de la entrada principal, 15 Oct 2015, *Ochoterena, H. et al. 931* (BM, MEXU); Pedregal de San Angel, Ciudad Universitaria. Camellón cerca del Instituto de Biología, en el Circuito Escolar CU2 (o Circuito Deportivo), 16 Oct 2015, *Ochoterena, H. et al. 933* (BM, MEXU); Coyoacán, Cuidad Universitaria, estacionamento, Instituto de Biologia, 15 Jun 2007, *Ramírez H, A. & Ramirez Roo, A. 2* (MEXU); Xochimilco, ejidos de Xochimilco y San Gregorio Atlapulco, Area Natural Protegida Ejidos de Xochimilco, 19 May 2004, *Rivera H, J. et al. 3708* (MEXU); Xochimilco, Xochimilco, 25 Apr 1965, *Vázquez H, J. s.n.* (MEXU); Delegación G.A.Madero, Colonia Nuevo Atzacoalco, Calle 329 de la casa #10 hasta el entronque con Calle 314, lado del canal (muestra 597), 16 Aug 1993, *Vibrans, H. 4449* (MEXU); Del. Coyoacán, Colonia Culhuacán, Calle la Cerrada de Santa Ana (Muestra #701), 13 Sep 1993, *Vibrans, H. 4544* (MEXU); Xochimilco, ceraca la Periferico Sur, cerca del campo Militar Batalion, poco al E de Cuemanco, 14 Nov 2000, *Vibrans, H. 6933* (MEXU). **Durango**: Mezquital, ca. 2 km de Troncon, 23 Apr 1985, *González, S. 3112* (MEXU); city of Durango and vicinity, Apr 1896, *Palmer, E. 959* (K, US); Durango, Sep 1912, *Patoni, C. s.n.* (MEXU); Durango, Jul 1912, *Patoni, C. s.n.* (MEXU); Durango, Aug 1912, *Patoni, C. 527* (MEXU); Mapimí, Puente de Hojuelas, 8 km al SE del municipio de Mapimí, 8 Sep 1983, *Torrecillas, E. 200* (MEXU). **Guanajuato**: León, 1837, *K.T. Hartweg 202* (BM, E, K, W); Celaya, Celaya, 1 Jun 1896, *Nelson, E.W. 3868* (US); Salvatierra, 21 Jun 1986, *Rzedowski, J. 39828* (MEXU); Santiago Maravatio, cerca de Salvatierra, 14 Oct 1989, *Rzedowski, J. 49107* (MEXU); La Loma, cerca de Tócuaro, 4 km al S de Acámbaro, 11 Jul 1986, *Santos Martínez, J. 1510 [a]* (MEXU). **Guerrero**: Tixtal de Guerrero, La Estacada, Cerro de Temalacatlalco, 5 Aug 1997, *Ignacio, D. 4* (MEXU); Acapulco and vicnity, Oct 1894, *Palmer, E.J. 554* (MO);Leonardo Bravo, 18 km al S de Filo de caballo, rumbo a la brecha de Asolcaderos, 9 May 1982, *Rodríguez B, D. & Martínez S, E.M. 101* (MEXU); Leonardo Bravo, a 46 km al SW de Filo de Caballo, 19 Oct 1983, *Soto N, J.C. et al. 5802* (MEXU); Ayutla de los Libres, Ayutla, en la huerta de Don Facundo Perez, 29 Mar 1986, *Soto N, J.C. 12894* (MEXU); Alcozauca, Ixcuinatoyac, 13 Sep 1983, *Viveros, J.L. & Casas, A. 215* (MEXU). **Hidalgo**: Ajacuba, Emiliano Zapata, 19 kmm después de Ajacuba a San Agustín Tlaxiaca, Sierra de Chicavasco (de Wa E), 8 Aug 1988, *Díaz Vilchis, I. 381* (MEXU); Alfajayucan, El Peñón, 19 Nov 1981, *Hernández Magaña, R. 6717* (MEXU, MO). **Jalisco**: Hwy 15 between Guadalajara and Tequila, Km 22, 12 Jul 1972, *Andreasen, M.L. et al. 623* (BH, MO); La Huerta, Arroyo Chamela, cerca del puente en el lecho del arroyo, 9 Dec 1985, *Ayala, M.G. 449* (MEXU); Tequila, 14-18 km southwest on Volcaán de, 7 Nov 1974, *Breedlove, D.E. 39222* (MO); Tlajomulco de Zúñiga, Cajititlán, Puente de Fierro, 27 Sep 1997, *Cortés R, C. & Ortiz C, E. 55* (MEXU); Tlajomulco de Zúñiga, Cajtitlán, de la orilla de la laguna a 50 m por a Huerta de los Aguacates, 5 Oct 1997, *Cortés R, C. & Placeres B, G. 137* (MEXU); 11 miles S of Guadalajara on Route 15, then 2 miles N on side road into the mountains, 6 Oct 1967, *Harker, M. & Mellowes, H. 134* (BM); La Huerta, Rancho Cuixmala, 11 May 1991, *Lott, E.J. et al. 3397* (MEXU); weedy rubble piles in weedy maize field and roadside, with Amaranthus, Ricinus communis, Physalis philadelphica, Lycopersicon esculentum and Solanum cf. nodiflorum. along ave. vallarta in Ciudad Granja, on the western outskirts of Guadalajara, 30 Dec 1978, *Nee, M. & Iltis, H.*

16672 (MEXU, MO); weedy disturbed area in lawn of Instituto Botánico, Universidad de Gudalajara, near Zapopan, 15km w. Guadalajara, 30 Dec 1978, *Nee, M. & Iltis, H. 16674* (BH, K, MEXU, MO, US); 1886, *Palmer, E. 11* (BM, K, NY, US); Acátic, barranca del Río Verde, al NW de Atátic, camino al rancho La Sosa, 13 Apr 1992, *Ramírez-Delgadillo, R. & Orozco de la Rosa, G. 2668* (MEXU); San Martín de Bolaños, 5 km salida al oeste a la Zuluaga, 17 Feb 1992, *Reynoso D, J.J. et al. 635* (MEXU); Zapopan, Barranca de los Camachos, 28 Feb 1992, *Rodríguez C, A. 2133* (MEXU); Antonio Escobedo, La Joya, 31 May 1988, *Román, M.L. & Joya, S.P. 978* (MEXU); Guadalajara, near Guadalajara, 23 Feb 1907, *Safford, W.E. 1396* (US); Tequila, Volcan, along road to microwave station, 25 Oct 1970, *Webster, G.L. et al. 15941* (MEXU, MO); El Tigre, on the Pacific slope of the mountains SW of Autlán about 17 miles on Route 80, 12 Aug 1984, *Wilbur, R.L. 36689* (BM). **México**: Texcoco, Lago de Texcoco, 10 km al S de Texcoco. Suelos salinos con lagunas hasta de 8 m. de prof, 6 Mar 1985, *Rodríguez H, C. s.n.* (MO); Texcoco, Lago de Texcoco, 6 Mar 1985, *Rodríguez H, C. & Espinosa P, J.A. s.n.* (MEXU); Zumpahuacán, carretera de tenancingo a Zumpahuacán, ca. 2 km al N de Tlapizalco, 16 Oct 1994, *Vibrans, H. 4968* (MEXU); Ixtapán de la Sal, carretera que desvia de la carretera Ixtapán de la Sal-Almoloya de Alquisiras, hacia Malinaltenango, ca. 2.5 km al N de la iglesia de Malinaltenango, cerca de una pequeña capilla, 20 Oct 1996, *Vibrans, H. 6260* (MEXU); Tezoyuca, via del tren, 500 m al W de la carretera Texcoco-Lecheria, cerca de la desviación a Teotihuacán, 28 Jun 2001, *Vibrans, H. 7315* (MEXU). **Michoacán**: vicinity of Morelia; jardin du Sacré Coeur, 4 May 1909, *Arsène, G. 3334* (MEXU, MO, US); Morelia, Lado O de cerro El Águila, aproximadamente 2.5 km al SE de Iratzio, 4 Oct 2010, *Cornejo Tenorio, G. et al. 3628* (MEXU, MO); Morelia, al suroeste de la cuidad de Morelia, Campus de la UNAM, 4 Jun 2012, *Flores Tolentino, M. & Cornejo Tenorio, G. 251* (MEXU); Janamuato, alrededores, ca. 5 km al W de Puruándiro, 7 Nov 1985, *Martínez L, A. 124* (MEXU); Zinapécuaro, Coro, borde sureste del Lago de Cuitzeo, 13 Apr 1986, *Novelo R, A. & Rojas M, J. 752* (MEXU); Jacona, alrededores del Preso Sixto Verduzco, 16 Jul 2007, *Reyes, P. 14270* (MEXU); Jacona, alrededores del Preso Sixto Verduzco, 16 Jul 2007, *Reyes, P. 14356* (MEXU); Morelia, alrededores de la Preza Coiotzio, 29 Aug 1986, *Rzedowski, J. 40130* (MEXU); Balneario Huapamacátaro, 10 al SW de Maravatío, 31 Mar 1988, *Rzedowski, J. 46293* (MEXU); Morelia, La Huerta, alrededores de la estación de ferrocarril, 21 Jul 1992, *Rzedowski, J. 51543* (MEXU); Zitácuaro, 6 km al SW de Zitácuaro, 18 Jun 1983, *Soto N, J.C. 5275* (MEXU); Zitácuaro, La Garita, 4 km al SO de Zitácuaro, 6 Mar 1985, *Soto N, J.C. & Aureoles C, S. 7252* (MEXU); Zitácuaro, La Garita, 4 km al SO de Zitácuaro, 6 Mar 1985, *Soto N, J.C. & Aureoles C, S. 7253* (MEXU); Zitácuaro, Coatepec de Morelos, 4 km al SO de Zitácuaro, 6 Mar 1985, *Soto N, J.C. & Aureoles C, S. 7319* (MEXU); Hidalgo, Cruz de Caminos, 12 km al O de Ciudad Hidalgo, 7 Mar 1985, *Soto N, J.C. & Cornejo, S.A. 7346* (MEXU); Parácuaro, Parácuaro, 6 Apr 1985, *Soto N, J.C. et al. 7998* (MEXU); Múgica, Río El Marqués, ca. 200 m al sureste del puente sobre la autopista Morelia-Lázaro Cárdenas, 1 Feb 2004, *Steinmann, V.W. & Porter, J.M. 4052* (MEXU). **Morelos**: Tetela del Volcán, ca. 0.5 km W of T-junction Hueyapan (San Andres Huayapan) and Tlacotepec, E of arroyo-barranca (to S of road) with sand quarry (to N of road) and school; ca. 4 km E of San Antonio Alponoca, 6 Dec 1994, *Bye, R.A. & Linares, E. 19414* (MEXU); Miacatlan, Laguna de Costetelco, 6 Apr 1984, *Fuchs Q, F.M. 211* (MEXU). **Nayarit**: Tepic, Ejido 5 de Mayo, 2.5 km del entronque rumbo a San Blas, carretera Tepic-Mazatlan, en el camino de terraceria rumbo al Rancho el Verdireño, 12 Mar 1991, *Ramírez R, R. & Flores-Franco, G. 836* (MEXU); Tepic, Acaponeta, 11 Apr 1910, *Rose, J.N. et al. 14363* (US). **Nuevo León**: Beside parking lot, top of Chipinque, 7 Sep 1978, *D'Arcy, W.G. 11676 B* (MO); Valle Alto, 14 Jul 1971, *Medler, B. 105* (EIU); Monterrey, Monterrey, Rio Santa Catarina, Sierra Madre Oriental, 17 Jun 1934, *Pennell, F.W. 16799* (US); Montemorelos, Ojo de Agua, 2 Mar 1988, *Tirado, N. 18* (MEXU); Montemorelos, Ojo de Agua, 9 Mar 1988, *Tirado, N. 76* (MEXU); 21 miles N of Matehuala (San Luis Potosí), 21 Aug 1959, *Waterfall, U.T. 15748* (US). **Oaxaca**: Along Hwy 175 between Tuxtepec and Oaxaca, 24 km above (S of) bridge over Río San Juan Bautista at Valle Nacional, 3 Mar 2008, *Croat, T.B. & Díaz-Jiménez, P. 100194* (MO); Vivero Rancho Teja, ca. 3 km southeast of Ixtlan de Juarez, 26 Jul 1966, *Cruden, R.W. 1100* (MEXU); Weeds around town ca 3 km NW of Oaxaca, 17 Sep 1978, *D'Arcy, W.G. 11986* (MO); Santiago Astata, Barra de la Cruz, Dist. Tehuantepec, 31 Mar 1998, *Elorsa C, M. 101* (MEXU); San Pedro Huamelula, Zimatán, 0.5 km N del puente, Dist. Tehuantepec, 8 Jan 1999, *Elorsa C, M. 1444* (MEXU); San Lucas Ojitlán, Presa Cerro de Oro, 25 Oct 1988, *Hernández Ortega, R. 62* (MEXU); San Juan Mixtepec, San Juan Mixtepec, near town, 25 Sep 1996, *Hunn, E. OAX-205* (MEXU); San

Juan Mixtepec, Miahuatlán, near town, 26 Jan 1997, *Hunn, E. OAX-793* (MEXU); Puente Las Catarinas, 1 mile S of Las Margaritas along Hwy 190, 27 Dec 1977, *Jansen, R.K. & Harriman, N.A. 499* (MEXU); Ixtlan, Santiago Comaltepec, 11 Dec 1987, *López L, L.M. 18* (MO); Tlacolula, Mitla, Juana Arena, 27 Apr 2009, *Lopez, I. & Hernández O, H. 32* (MEXU); Oaxaca. Between Km. 38 and 41, Tuxtepec Rd. Hwy. 175 which leaves Hwy. 190, 2 mi. east of Oaxaca, 17 Jan 1965, *Margery, C. 4071* (NY); Mercado de Benito Juarez, procedente de Ojitlán, 12 Aug 1987, *Martin, G.J. M-242* (MEXU); San Pablo Villa de Mitla, vicinity of Mitla, valley of Río Grande de Mitla and nearly foothills, 7 Aug 1971, *Messer, E. 212 a* (MEXU); Zunatlán, Paraje El Perico, 13 km al sur de La Cofradia, comunidad de San Pedro El Alto, 29 Oct 1998, *Miranda, A.G. & Hernández, O.L. 562* (MEXU); Cerro Concordia, 8 Apr 1933, *Morton, C.V. & Makrinius, E. 2658* (US); Huautla de Jimenez, alrededores del Puente de Fierro en dirección a Santa Maria Chilchotla (7 km de Huautla de Jimenez por la carretera a Teotitlán-Mex 182), 9 Mar 2001, *Munn-Estrada, X. et al. 841* (MEXU); San Juan Mixtepec, Río Mixteco 2 km al SE de San Juan Mixtepec, 8 May 1988, *Reyes S, J. 192* (MEXU); San Miguel del Puerto, Xadan, 1.5 km W brecha hacia San Miguel, . Dist. Pochutla, 25 Jan 2000, *Rivera H, J. et al. 2164* (MEXU); Totontepec, Tontontepec, Dist. Mixe, 15 Aug 1986, *Rivera Reyes, J. & Martin, G. 415* (MEXU); Totontepec, Mixe, Villa de Morelos, Mesa Pa'tkum, na payi, 22 Feb 1991, *Rivera Reyes, J. 2545* (MEXU); San Miguel del Puerto, 1 km al W de la finca El Mamey, arroyo Arean, Dist. Pochutla, 28 Jun 2001, *Saynes V, A. et al. 2245* (MEXU); W-facing slope of El Cumbre Mtns, 21 Jul 1966, *Schoenwetter, J. JSOX-58* (US); km 13 del camino Miahuatlán-San Pablo Coatlán, 27 Dec 1978, *Solano, C. & Vara, A. 506* (MEXU); Santiago Textitlán, Colonia Nueva Providencia, Dist. Sola de Vega, 21 Jun 2006, *Trujillo Vasquez, R. 43* (MEXU); Cerro San Felipe, upper slopes, above and west of Tejalapan (San Felipe), about 13 km NW of Oaxaca (air distance), 3 Oct 1962, *Ugent, D. & Ugent, V. 2717* (MEXU); Jct. of Rd to Mitlo & Hwy. 190 to Tehuautepec, CA-1 24 mi. E. of Oaxaca, 8 Mar 1975, *Wallace, K.L. et al. 346* (MO); abut 13.5 miles S of Miahuatlán on Route 175, 7 Aug 1984, *Wilbur, R.L. 36451* (BM); 20 miles north of Oaxaca, 1/2 mile past town of Telixotahuaca on dirt road, 20 Jun 1966, *Windler, D.R. & Snider, J. 979* (MO). **Puebla**: San Antonio Texcala, 1km al S de San Antonio, cerca de la orilla de la carretera 125, 20 Oct 1979, *Bretting, P.K. 151* (NY); Tehuacán, environs of Monumento a la Identidad, junction towards Avenue A. Lopez mateos, in Tehuacán town centre, 4 Mar 2005, *Calzada, J.I. JIC-24498* (K); Xochitlán de Vicente Suarez, Pocapan, 27 Mar 2002, *Cruz Rivas, A. 244 ac* (MEXU); Xochitlán de Vicente Suarez, Tecorralican, 27 Mar 2002, *Cruz Rivas, A. 268 ac* (MEXU); Hueytamalco, Campo Experimental "Las Margaritas", Instituto Nacional de Investigaciones Forestales, Agrícolas y Pecuarias (INIFAP), 23 Oct 2007, *Gómez Chagala, B. 306* (MO); Tlatlauquitepec, Haguataminilol, carretera Teteles-Mazatepec, 24 Mar 1976, *Márquez Ramírez, W. & colaboradores 677* (K); Cholula, Cañada Grande, por el lado noreste de San Pedro, 8 Sep 1987, *Tlapa A, M. & Ubierna, G. 438* (MEXU). **Querétaro**: Queretáro, 5 Jun 1993, *Argüelles, E. s.n.* (MEXU); La Venta, 27 Nov 1976, *Argüelles, E. 667* (MEXU); Jurica, 29 Mar 1978, *Argüelles, E. 996* (MEXU); La Venta, 22 Jan 1987, *Argüelles, E. 2876* (MEXU); Querétaro, 28 Jun 1993, *Argüelles, E. 3441* (MEXU); Landa, ca. 2 km al noroeste de Acatitlán de Zaragoza, 8 Aug 1989, *González E. 822* (MEXU); Vizarrón, Vizarrón, 23 Dec 1982, *Hernández Magaña, R. 9218* (MEXU); Jalpan, El Sabinito, al SE de Tachanaquito, 8 Jan 1992, *López Ch, L. 234* (MEXU). **Quintana Roo**: José María Morelos, Xnoh Cruz, a 2.5 km al SO, 12 Jun 2005, *Álvarez, D. et al. 11053* (MEXU); Cozumel, Isla de Cuzumel, por el camino a Cedral, 6-19 km al S de la zona urbana de Cozumel, 4 Jun 1986, *Cabrera, E. & Cabrera, H. de 11424* (MEXU); 7 km al S de Puerto Morelos, sobre la carretera Cancun-Tulum, 15 Jan 1989, *Cabrera, E. & Colli, W. 16124* (MEXU); Solidaridad, Cobá, 28 Apr 1994, *Sears, R.R. et al. 141* (CICY); Solidaridad, a 14 km al NE-E de Cobá, 3 Feb 1980, *Téllez V, O. & Cabrera, E. 1414* (CICY); Felipe Carrillo Puerto, X-komhá en un huerto al costado oriente de la plaza, 22 Feb 1984, *Ucán Ek, E. & Caballero, J. 3247* (CICY). **San Luis Potosí**: Matehuala, ca. 5 km N de Matehuala, Union de Ejidos, Productores de Tunas y Vainas de Mezquite -Emiliano Zapata, Mex 57 km 7+ (Matehuala-Saltillo), 13 Aug 1991, *Bye, R.A. & Linares, E. 18175* (MEXU); Cárdenas, 10 Jan 1984, *Chemin-Bassler, H. 90* (MEXU); Tamasopo, Beside waterfall, 12 Sep 1978, *D'Arcy, W.G. 11892* (MO); Tamasopo, On road from to Agua Buena, 14 Jun 1981, *Hahn, W.J. 531* (MO); S slope of Sierra Madre Oriental, 39 km (by road) NE of Cuidad Maiz on highway 80 at km 223, 1 Oct 1965, *Roe, K.E. & Roe, E. 2320* (MEXU); San Luis Potosí, in convalli, 1877, *Schaffner, J.G. 692 [a]* (MEXU). **Sinaloa**: Culiacán, La Divisa, por el km 6 al N de la carretera Culiacán-Sanalona, 13 Sep 1984, *Antío, J.A. & Félix, A.L. 128* (MEXU); Culiacán, La

Divisa, km 6 al N de la Carretera Culiacán-Sanalona, 25 Aug 1984, *Félix, A.L. & Antío, J.A. 55* (MEXU); Culiacán, Costa Rica, al SE del dique los Cascabeles, 31 Mar 1984, *Figueroa Rosas, J. & Meza Sánchez, G. 34* (MEXU); Culiacán, El Dorado, ejido San Diego, 12 Feb 1984, *García E, J.D. & Carra M, R. 16* (MEXU); sin. loc, 1921, *Ortega, J.G. 4224* (US); Ahome, Topolobampo, 15 Sep 1897, *Palmer, E. 196 [a]* (US); Mazatlan, Mazatlán, vicinity, 31 Mar 1910, *Rose, J.N. et al. 13811* (US); Mazatlan, vicinity of Mazatlán, 6 Apr 1910, *Rose, J.N. et al. 14134* (US); Cosalá, vicinity of Guadelupe, 18 Apr 1910, *Rose, J.N. et al. 14697* (US); Culiacán, vicinity of Culiacán, 21 Apr 1910, *Rose, J.N. et al. 14877* (NY, US); Culiacán, El Dorado-A. Ponce, 28 Feb 1992, *Vega A, R. & Hernández A, F. 4278* (MEXU); Mazatlán, and vicinity, Jan 1889, *Wright, W.G. 1256* (F, US). **Sonora**: Alamos, Río Mayo watershed, San Bernardo and vicinity, Chorijoa, Sep 1961, *Argüelles, J. 122* (US); 2.5 km al S de Hermosillo, ara de exhibición del CES y zona circundante, 22 Mar 1992, *Búrquez, A. 92-287* (MEXU); "Sonora Alta", *Coulter, T. 1231* (K); Guaymas, Rancho Bacatetito, ca. 1 km NE of ruin of Cuartel at Bacateve, Sierra Bacatete, 13 Mar 1989, *Felger, R.S. et al. 89-170* (MEXU); Guaymas, Las Guásimas. Plants cultivated in yard of home of Alfonso Flores and family, 16 Dec 1988, *Felger, R.S. et al. 88-614* (MO); Guaymas, El Baviso, vicinity of Bahia San Carlos, ca. 2 km inland, 13 Mar 1985, *Felger, R.S. & Russell, J. 85-667* (MEXU); Salitral, Río Mayo, 4 Feb 1935, *Gentry, H.S. 1269* (K, MEXU); Río Yaqui, 2-3 km N of Onavas, on edge of ejido fields, 4 Apr 1991, *Joyal, E. & Estrella, M. 1588* (MEXU, MO); Arroyo La Quema, near Tepoca; 28, 31 May 1997, *Reina G, A.L. & Van Devender, T.R. 97-446* (MO); Hermosillo, Hermosillo, bed of Río de Sonora, 5 Mar 1910, *Rose, J.N. et al. 12422* (US); Camahurios between Agiabampo and Las Bocas on the Gulf of California, 15 Mar 1993, *Van Devender, T.R. et al. 93-300* (MO); Rancho La Junta, 1 km upstream from Mesa Colorado, 3.0 km (by air) north-northeast of Burapaco; junction of arroyo Guajaray and Rio Mayo, 17 Mar 1993, *Van Devender, T.R. et al. 93-378* (MO); Alamos, Arroyo Las rastras, Rancho Penas Coloradas, southwest edge of the sierra de Alamos, 22 Sep 1993, *Van Devender, T.R. et al. 93-1012* (MO). **Tabasco**: Tuota, a 2 km del Inst. Cult. Nacajlca, 6 Oct 1978, *Calzada, J.I. 4914* (MEXU); Cárdenas, at Km 175 between Coatzacoalcos and Cárdenas, 11 Aug 1974, *Conrad, J. et al. 2852* (MO); Balancán, Palo Verde, 5 km al S de Balancán, 4 Mar 1983, *Fernández N, R. & Guadarrama-Zamudio 1352* (MO); a 13 kms de Villahermosa por la carretera a Escarega, Cam. y a unos 800 m al SE, 10 Dec 1965, *González L, L.A. & Pérez J, L.A. 4152* (MEXU); Tenosique, a 2.5 km arriba del campamento de la escollera y 5 km de Tenosique hacia E. Mapata, 27 Feb 1982, *Magaña, M.A. & Zamudio, S. 876* (MEXU); Balancán, Las Estacas, primeros 200 m de la parte final de la carretera W-O, 28 Oct 1975, *Menendez, F. 216* (K, MEXU); Balancán, Ejido El Arenal, 20 Dec 1996, *Ramírez B, A. 10* (MEXU). **Tamaulipas**: Tampico, 4 Feb 1827, *Berlandier, J.L. 46* (BM, G-DC, LE, W); alogn Hwy 85 between Ciudad Monte and Ciudad Valles, 12.3 miles S of Cd. Monte, 12 Jan 1987, *Croat, T.B. & Hannon, D.P. 62947* (MEXU); Tampico, 3 km al W del Pueblo de Tancol, 6 Feb 1968, *García Saucedo, D. s.n.* (MEXU); Mina de Asbesto, a 25 km al Oeste de La Libertad y a 35 km de Cuidad Victoria, 9 Dec 1987, *González Medrano, F. 16964* (MEXU); Soto de la Marina, Soto la Marina, 2 Mar 1903, *Nelson, E.W. 6640* (US). **Veracruz**: Tantoyuca, Potrero Primero, 23 Dec 1978, *Alcorn, J.B. 2347* (MEXU); Yecuatla, El Mirador, carretera Naolinco Misantla, 4 Feb 1976, *Avendaño Reyes, S. 126* (F); Hueyapan de Ocampo, Santa Rosa, Loma Larga, 2 Dec 1996, *Balvanera L, P. 204* (MEXU); San Andrés Tuxtla, Soyata, 9 Dec 1986, *Balvanera L, P. 259* (MEXU); Orizaba, 30 Jun 1865, *Bourgeau, E. 2525* (LE); Hidalgotitlán, Cedillo, Campamento Hermanos, 7 Apr 1974, *Brigada Vásquez*, *392* (MEXU); Catemaco, Playa Escondida, 5 km de la desviación de la carretera La Palma-Balzapote, 11 Aug 1981, *Calzada, J.I. 7765* (MEXU); Tempoal, a 10 km de Tempoal, hacia Panuco, 19 Mar 1971, *Chiang, F. 408* (F, MEXU); Papantla, brecha La Laguna González Ortega, 1.7 km antes de González Ortega, 10 Nov 1981, *Cortés, M.E. & Vázquez, ? 13* (MEXU); alogn Hwy 147 between Palomares (at junction with Hwy 185 in Oaxaca) & Junction with Hwy 175 ust S of Tuxtepec, 5 miles SE of border with Oaxaca to the N, 23.8 miles NW of border with Oaxaca to the S, 20 Feb 1987, *Croat, T.B. & Hannon, D.P. 65478* (MEXU); Hidalgotitlán, camino Cedillo-Agustin Melgar, 21 Jan 1975, *Dorantes, J. 4009* (MEXU); La Laguna, near veracruz, 23 Jan 1906, *Greenman, J.M. 50* (F); 2 km W of Acultzingo along exican Hwy 150, 6 Jun 1973, *Hansen, B. et al. 1721* (MEXU, US); San Andrés Tuxtla, Estación Biológica Tropical Las Tuxtlas, Lote 67, 16 Sep 1987, *Ibarra Manríquez, G. & Sinaca Colín, S. 3189* (MEXU); San Rafael, 1 km del pueblo en carretera San Rafael-Nautla, 22 Apr 1970, *Jarquin L, E. & Lot, A. 22* (MEXU, US); Mirador, Feb 1839, *Linden, J.J. 250* (K); Otatitlán, Playa de Vaca, *Martínez Calderón, G. 1342* (BM,

MEXU, MO); San Andrés Tuxtla, Estación Biológica Las Tuxtlas, 16 Jan 1969, *Martínez-Calderón, G. 1830* (US); Tempoal, 1.5 km NE of Tempoal, along hwy. Mex 105, 4 Jan 1981, *Nee, M. et al. 19522* (F, MEXU); Orizaba, Orizaba, 10 Mar 1904, *Nelson, E.W. 191* (US); Chalma, 500 m al NW de Chalma, terraceria a Chiconamel, 5 Oct 2000, *Rincón G, A. & Durán E, C. 1998* (MEXU); Cazones de Herrera, La Encantada, 20 Mar 2012, *Romo Díza, G. 1263* (MEXU); Barranca de San Miguel, km 17 carretera Orizaba-Cordoba, 14 Apr 1968, *Rosas R, M. 1167* (MEXU); Tantoyuca, NW of Tuxpan, alogn the road leading from hwy 127 to Chicontepec, about 2 miles beyond the road junction at San Sebastian, 12 Jul 1983, *Taylor, C.M. 2045* (DUKE); 3 km al E de Orizaba, 10 Mar 1983, *Torres C, R. et al. 2336* (MEXU); Catemaco, Playa Escondida on gulf of Mexico, 10km N of Sontecomapan, 18 Sep 1982, *Without Collector s.n.* (BM). **Yucatán**: Tahdziú, Tahdziú, 22 Aug 1990, *Cemé, J. 75* (CICY); José María Morelos, Chichankanab, *Gaumer, G.F. 1533* (US); Yaxcabá, a 6 kms al S de Yaxcalá, 2 Mar 1982, *Illsey G, C. 1297* (CICY); Dzemul, Playa San Benito, 2 Dec 1980, *Ordóñez, M.J. & Rangel, R. 68* (CICY); Uayma, en el ejido Mucel de Pixoy, 10 Mar 1983, *Ucán Ek, E. 2270* (CICY); Dzitás, Dzitás, 13 Sep 1984, *Ucán Ek, E. 3523* (CICY); Valladolid, Pixoy, 27 Jan 1987, *Ucán Ek, E. 4390* (CICY, MEXU); Yaxcabá, Tixcacaltuyub, 30 Oct 1980, *Vargas R, C. 214* (CICY).

MONTSERRAT. Centre Hills, Katy Hill, alongside trail, 2 Jun 2008, *Greenaway, J. et al. JG-50* (K); Chance's Mountain, Summit area of Chance's Mt. Soufriere Hills, in clearing, 9 Feb 1959, *Proctor, G.R. 19115* (BM); Plymouth, 19 Jan 1907, *Schafer, J.A. 3* (F). **Saint Anthony**: Plymouth, in a weedy yard, 5 Feb 1959, *Proctor, G.R. 19020* (BM).

NETHERLANDS ANTILLES. **Curaçao**: 17 Oct 1951, *Arnoldo, M. 1719* (US); sin. loc., 19 Feb 1917, *Curran, H.M. & Haman, M. 64* (K).

NICARAGUA. "Guatemala, San Juan de Nicaragua", 1841, *Friedrichsthal, E. von*, *558* (W); sin. loc, *Seemann, B.C. 81* (BM); Chontales, 1867, *Tate, R. 258 (219)* (BM). **Atlántico Norte**: Municipio de Siuna, Calera, 12 Mar 1984, *Ortíz, F. 1798* (MO). **Boaco**: Quebrada Río Grande, al NE de Cerro Mombachito, 10 May 1980, *Moreno, P.P. 347* (MO); Río Fonseca, "Los García", 3.5 km de Santa Lucía, carretera a Bo, 30 Jul 1981, *Moreno, P.P. 10181* (MEXU, MO); Cerro Mombachito, al W de Boaco, 29 Aug 1981, *Moreno, P.P. 10561* (MO); Upper SW slope of Cerro Mombachito, S of road between Boaco and Camoapa, 3 Oct 1979, *Stevens, W.D. et al. 14522* (MO). **Chinandega**: Faldas del Volcán Casita, Montañas El Uval, Hacienda Bellavista; ca 30 km al N de Posoltega, 16 Oct 1982, *Grijalva, A. & Grijalva, M.V. de 1506* (MO). **Estelí**: Mesas Moropotente, ca. 16.0 km (by road) NE of Hwy 1 at Estelí, 11 Jun 1981, *Henrich, J.E. & Stevens, W.D. 450* (MO); Salto de Estanzuela, 13 Apr 1981, *Moreno, P.P. 8142* (MO); Llano del Pozo, 18 km al NE de Estelí, camino a la Laguna de Miraflores, 13 Sep 1981, *Moreno, P.P. 10998* (MO); Municipio Condega, Communidad Venecia, en la parcela 4, ubicada a 3.5 km al este, 29 Jan 2008, *Olivas Matey, C.M. 138* (MO); Loma Ocotecalzado (Mesas Moropotente), ca. 11.0 km NE of Hwy. 1 at Estelí, 15 Nov 1979, *Stevens, W.D. & Grijalva, A. 15590* (MO); Along road from Condega to Yalí, ca. 18.9 km NE of Hwy. 1 and ca. 1 km SW of Darailí, 19 Nov 1979, *Stevens, W.D. 15848* (MEXU, MO). **Granada**: Granada, 1903, *Baker, C.F. 677* (US); Volcán Mombacho, hacienda San Joaquín, 13 Sep 1983, *Grijalva, A. 2976* (MEXU); NE del Volcán Mombacho, en el empalme de los caminos a Santa Isabel y Cutirre, 16 Sep 1980, *Moreno, P.P. 2628* (MO); 3 km al norte de INTECNA, La Playuela, 24 Jun 1981, *Sandino, J.C. 677* (MO); Suroeste de Laguna Juan Tallo, al sureste de Volcán Mombacho, 30 Jun 1981, *Sandino, J.C. & Guzmán, M. 777* (MO). **Jinotega**: El Cedro, 18-20 km al N del Valle del Cuá, 19 Jun 1980, *Moreno, P.P. 820* (MO); alogn highway 3 between Matagalpa and Jinotega, ca. 1.6 km SE of Jinotega (at intersection of bypass), 25 Aug 1978, *Stevens, W.D. 10088* (MEXU, MO); ca. 4.7 km S of Jinotega (square) on old road to Hwy 3 near Matagalpa, then along road to city dump and abandoned mine, 25 Aug 1978, *Stevens, W.D. 10163* (MEXU, MO); Caño Litutus, Río Bocay, 9 Mar 1980, *Stevens, W.D. et al. 16638* (MEXU, MO); Santa Lastenia between Matagalpa and Jinotega, 31 Oct 1982, *Stevens, W.D. 21894* (MO). **Madriz**: Valle el Sapote, 4 km noreste de Somoto, 23 Sep 1980, *Moreno, P.P. 2752* (MEXU, MO); Volcán Somoto, (Tepesomoto) Finca San Martin lado Oeste, 25 Sep 1980, *Moreno, P.P. 2990* (MEXU, MO). **Managua**: Las Maderas, Comarca La Reforma, ca 2km al NE del poblado Las Maderas, 31 Oct 1984, *Grijalva, A. & Aranda, M. 4098* (MEXU, MO); Comarca San Isidro de la Cruz Verde, 2 km S del Colegio Centro America, sobre el camino a San Isidro de la Cruz

Verde, 9 May 1981, *Guzmán-Teare, M. 1805* (MO); Santo Domingo de Guzmán, 1 km al E, 18 Jul 1981, *Moreno, P.P. 9975* (MEXU, MO); Ca. 2.3 km from Hwy. 12 on road along ridge of Sierra de Managua from Hwy. 12, (Carretera vieja a León) at Km 17 to Hwy. 2 (Carretera Sur), 29 Jul 1977, *Stevens, W.D. 2931* (MO); Along Río Montelimar at Hwy 10 bridge, 28 Jul 1978, *Stevens, W.D. 9524* (MEXU, MO). **Matagalpa**: Entre Matagalpa y Jinotega, Km 139, 29 Aug 1979, *Araquistain, M. 174* (MO); Falda norte del Cerro Musún, frente a trocha a Wanawás, 16 May 1980, *Araquistain, M. & Moreno, P.P. 2716* (MO); Hillside above Santa María de Ostuma, 22 Dec 1975, *D'Arcy, W.G. 10474* (MO); Cerro Apante, 2 km al S de la ciudad de Matagalpa, 31 Oct 1980, *Guzmán, M. & Castro, D. 1397* (MEXU, MO); Carretera al Tuma, Hda. La Bonanza, 25 km NE de la ciudad de Matagalpa, 8 Sep 1980, *Guzmán-Teare, M. et al. 756* (MO); Carretera Panamerican, Km 94, a orilla del la carretera, 10 Sep 1980, *Guzmán-Teare, M. et al. 979* (MO); Yasica Norte, 'El Hular' a 24 km de Matagalpa, 12 Feb 1981, *Moreno, P.P. 6691* (BM, MEXU, MO); Fuente Pura, Km 144 carretera Matagalpa-Jinotega, 20 Feb 1981, *Moreno, P.P. 7070* (MEXU, MO); Salto Santa Emilia, NE de Ciudad de Matagalpa, 6 May 1982, *Sandino, J.C. 2731* (MO); Macizos del Peñas Blancas, SE side, drainage of Quebrada el Quebradon, slopes N of Hda. San Martín, 24 Nov 1981, *Stevens, W.D. & Riviere, R. 20895* (MO); Macizos de Peñas Blancas, SE side, drainage of Quebrada El Quebradon, slopes N and W of Hda. San Sebastian; collection locality straddles border with Departmento de Jinotega, 20 Jan 1982, *Stevens, W.D. et al. 21299* (MO); Macizos de Peñas Blancas, SE side, drainage of Quebrada El Quebradon, slopes N and W of Hda. San Sebastian; collection locality straddles border with Departmento de Jinotega, 20 Jan 1982, *Stevens, W.D. et al. 21300* (MO). **Rivas**: Isla Ometepe, Volcán Concepción, cementerio de Los Hatillos, 10 Aug 1984, *Robleto, W. 1039* (MEXU, MO); Isla Omotepe, Volcán Concepción, Cerro El Mogote de las Pilas, 27 Oct 1984, *Robleto, W. 1412* (MEXU). **Zelaya**: Cano Costa Riquita, ca. 1.8 km SW of Colonia Naciones Unidas, above (S of) road between Colonia Nueva Léon and Colonia Naciones Unidas, 6 Nov 1977, *Stevens, W.D. 5009* (BM, MO, QCA); vicinity of Wani including Río Ulí, 15 Mar 1978, *Stevens, W.D. 7310* (MEXU, MO).

PANAMA. sin. loc., *Duke, J.A. 15437* (MO); Cerro Jefe, 21 Dec 1972, *Gentry, A.H. 6758* (MO); tributary of Rio Chagres, 5 miles SW of Cerro Brewster, 14 Dec 1967, *Lewis, W.H. et al. 3398* (MO); sin. loc, *Without Collector s.n.* (GOET). **Bocas del Toro**: Area along trail to Riscó Abajo, 2 km SW of town of Almirante, 3 Jan 1975, *Nee, M. & Hansen, B.F. 14121* (MO); Swan Key, 2 km N of Isla Colón, 10 Oct 1970, *Tyson, E.L. & Loftin, H. 6303* (MO). **Canal Area**: Fort San Lorenzo, 17 Dec 1966, *Burch, D.G. et al. 1033* (K, MO); Farfan Beach, 3 Aug 1965, *Dwyer, J.D. 6787* (MO); Cerro Galera, ca. 2 km from Pacific Ocean near west boundary of Canal Zone, 17 Dec 1972, *Gentry, A.H. 6621* (MO); Pan American Hwy 2 mi E of Arraijan, 5 Apr 1969, *Lewis, W.H. et al. 5183* (MO); 1 km NW of Gamboa. long fence around E side of Ammunition depot, 4 Feb 1974, *Nee, M. 9544* (MO); Sosa Hill, Balboa, 27 Nov 1923, *Standley, P.C. 25290* (MO). **Chiriquí**: David, David, 20 Oct 1975, *Delgado B, A.A. 11* (MO); Road from Gualaca to Chiriqui Grande, Roadside near Fortuna Lake, 18 Mar 1985, *Hampshire, R.J. & Whitefoord, C. 730* (BM); Along river bank of Río Caldero, 10 miles N of David, 24 Apr 1969, *Lazor, R.L. & Correa A, M.D. 2795* (MO); vicinity of Boquete, from Boquete to 3 miles N, 12 Dec 1966, *Lewis, W.H. et al. 388* (K); Vicinity of San Bartolomé, Peninsula de Burica, 28 Jul 1940, *Woodson, R.E. et al. 942* (MO). **Coclé**: La Mesa region N of Cerro Gaital vicinity of (El Valle), 2 Jul 1978, *Hammel, B. 3827* (MO); Boca del Toabré at confluence of Río Toabré at confluence of Río Coclé del Norte, 11 Apr 1969, *Lewis, W.H. et al. 554* (MO); Along road, halfway between El Valle and La Mesa, 3 Jan 1974, *Nee, M. & Dwyer, J. 9182* (MO). **Colón**: Vicinity Sardinilla, 7-8 mi E of cement plant, 12 Sep 1965, *Blum, K.E. & Tyson, E.L. 477* (MO); Sta Rita Ridge, 28 Jul 1972, *D'Arcy, W.G. & D'Arcy, J.J. 6171* (MO); Chagres, Chagres, Isthmus of Panama, Jan 1850, *Fendler, A. 249* (K); En la carretera del río Gatún, 10 Apr 1971, *Flores, G. 27* (MO); Ca. 21 kms from Transisthmica Hwy. on Santa Rita Ridge. [Coordinates on original label: 09.26N 079.38W], 23 Oct 1981, *Knapp, S. & Schmolzel, R. 1808* (MO); along Quebrada Sardinilla at bridge, 7 km NE of Buena Vista, 28 Oct 1973, *Nee, M. 7651* (CORD, MO); Along dirt trail, 4 km NW of Salamanca, 13 km NE of Buenos Aires, 30 Dec 1973, *Nee, M. 9081* (MO). **Darién**: Vicinity of Boca de Cupe, 5 Oct 1938, *Allen, P.H. 876* (MO); Near junction of Río Peresenico and Río Pirre, 20 May 1971, *D'Arcy, W.G. 5533* (MO); Río Pirre near crossing of trail from El Real to Tucutí, 20 miles W of El Real, 14 Jul 1962, *Duke, J.A. 5199* (MO); Village of Manene, 30 Apr 1968, *Kirkbride Jr, J.H. & Bristan, N. 1597* (MO); Upper Río Tuquesa, Jan 1973, *Le Clezio*, *259* (MO); Pacific coast, Guayabo, 12 Jan

1983, *Martin, J.H. 3592* (BM). **Guna Yala (San Blas)**: Puerto Obaldia, Forests around Puerto Obaldía, Aug 1911, *Pittier, H.F. 4378* (MO). **Los Santos**: Isla de Cañas, 21 Sep 1986, *Carrasquilla, L. 3024* (MO); Tonosi, Along upper Rio Pedregal, 29 Apr 1976, *Croat, T.B. 34504* (MO). **Panamá**: Chepo, A lado de la Represa Bayano, Bayano Dam, 13 Feb 1977, *Correa, A. et al. 2991* (MO); Chepo, Río Maje, ca. 30 minutes by speedboat (cayuca with 25 hp engine) from confluence with Río Bayano, 19 Apr 1976, *Croat, T.B. 34391* (MO); Panama, Cerro Jefe, 8 Apr 1970, *D'Arcy, W.G. et al. 3966* (MO); Panama, Roadside ca 13 miles W of Chepo, 25 Jul 1972, *D'Arcy, W.G. & D'Arcy, J.J. 6035* (MO); Chepo, 4 mi. S of Cañita, 25 Jul 1972, *D'Arcy, W.G. & D'Arcy, J.J. 6052* (CORD, MO); Chepo, 5-6 miles E of Chepo on Pan Am Highway, 28 Sep 1961, *Duke, J.A. 4044* (MO); Panamá, Tocumen, near Airport, 6 Jul 1962, *Dwyer, J.D. 1855* (MO); Panama, Cerro Jefe, 29 Jul 1967, *Dwyer, J.D. & Gauger, G.W. 7325* (MO); Chepo, Area around Torti Arriba, 31 Aug 1977, *Folsom, J.P. 5161* (MO); Panamá, Colectado en Tocumén, 4 Nov 1973, *González R, E. & González R, E. 42* (MO); 3.4 miles E of Cañazas checkpoint on Pan American Highway, in selectively cut forest about 4-5 km from road. Panamá-Darien border, foothills of Serrania de Cañazas, 12 Sep 1981, *Knapp, S. 1181* (CR, MEXU, MO); Chiman, Chimán, 12 Dec 1967, *Lewis, W.H. et al. 3299* (MO); Cerro Jefe, 20 Dec 2012, *Martínez, L. & Ortiz, O. 1202* (MO); Altos de Campana, a unos 80 m del Motel Sulin, 26 Dec 1977, *Méndez, R. 187* (QCA); Panamá, Along Río Pacora, 1 km upstream from Pacora, 14 Mar 1971, *Nee, M. & Mori, S.A. 3621* (MO); Panama, Vicinity of television tower on ridge, (around Cerro Penon), 3 km S of Alcalde Diaz, 1 Dec 1973, *Nee, M. 8586* (CORD, MO); Panamá, 1 mile N of Goofy Lake, 4 Dec 1967, *Oliver, R.L. et al. 2677* (MO); Panama, Cerro Azul, 24 Mar 1969, *Porter, D.M. et al. 4067* (MO); Chepo, 3 mi east of El Llano at Finca Risso, 15 Oct 1965, *Tyson, E.L. 1732* (MO); Panamá, Cerro Azul, 3 Nov 1965, *Tyson, E.L. 2136* (MO); Balboa, Saboga Island, near village. Perlas Islands, 19 Dec 1968, *Tyson, E.L. & Loftin, H. 5103* (MO). **Veraguas**: Montijo, Mountain of southern Azuero Peninsula, near proposed route of road from El Cortezo (Los Santos Prov.) to Arenas (Prov. Veraguas) Azuero (Veraguas Province), "El Pavo" ridge above river Los Changuales, 10 km SW of El Cortez (Los Santos Pro, 28 Oct 1978, *Hammel, B. 5404* (MO).

PUERTO RICO. Weed on Km 5 on road to Atalayas, 30 Mar 1964, *Duke, J.A. 7336* (MO); Corozal, Agric. Exp. Sub-Station, 16 Jul 1979, *García, J. & Llano, M. del s.n.* (MO); Fajardo, Puerto Chico, 25 Jul 1979, *Llano, M. del*, *et al. s.n.* (MO); District Bayamon. Santurce, 20 Oct 1937, *Otero, J.I. 165* (MO); Municipio de Salinas, 18 Jun 1992, *Ross, R. et al. SAN 128* (MO); Municipio de Salinas, 13 Mar 1993, *Ross, R. & Salguero, J. SAN 585* (MO); Maricao, 19 Sep 1884, *Sintenis, P. 251* (CORD, K); Guanica, 20 Jan 1886, *Sintenis, P. 3513* (K); Municipio Río Grande: El Verde Research Station, rte 186 at the Río Sonadora. Wet montane forest, 31 May 1994, *Taylor, C.M. 11804* (MO); Maricao Jamela Forest. Point hill top, 13 Dec 1938, *Véliz, I. 1357* (MO). **Adjuntas**: W of Adjuntas along rte 131 ca. 1-2 km N of its intersection with rte 518, 12 Jul 1986, *Taylor, C.M. 7161* (DUKE). **Caguas**: 8 Mar 1874, *Kuntze, C.E.O. 234* (NY). **Cayey**: Municipio of Cayey: Along route 7741, ca. 2 km SE of the intersection with route 741, 15 Jun 1991, *Miller, J.S. & Sherman, C.D. 6349* (MO). **Guanica**: Guanica, ad lagunas, 20 Jan 1886, *Sintenis, P. 3573* (BM). **Ponce**: NE of Ponce, along rte. 511 at km 9, N of rte 14, 1 Feb 1986, *Taylor, C.M. 6684* (DUKE); NE of Ponce along rte 511 ca. 9 miles from rte 14, 1 Feb 1986, *Taylor, C.M. 6706* (DUKE). **Rio Grande**: Municipio de Río Grande: Playa del Coco, immediately west of Punta Picúa, 6 Jun 1993, *Taylor, C.M. 11096* (MO).

SAINT KITTS AND NEVIS. **Saint Kitts**: Old Road river valley, 28 Jul 1937, *Sandwith, N.Y. 972* (K).

SAINT LUCIA. **Savanne Edmund**: Piton Troumassee, .5 mile south of Piton Troumasse, 4 Apr 1958, *Proctor, G.R. 17630* (BM); Savanne Edmund district, southeast of Piton Troumassee, 4 May 1958, *Proctor, G.R. 17826* (BM).

SAINT VINCENT AND THE GRENADINES. **Saint Vincent**: sin. loc. [ex Herb. Hooker], *[LG]*, *s.n.* (K); Hang, 7 Feb 1980, *Gilli, A. s.n.* (W); Road to Mt. St. Andrew, 11 Apr 1947, *Morton, C.V. 4923* (BM); May 1890, *Smith, H.H. & Smith, G.W. 450* (BM); side Below 500 ft, *Smith, H.H. & Smith, G.W. 639* (E).

TRINIDAD AND TOBAGO. **Tobago**: Highmoor, 27 Oct 1909, *Broadway, W.E. 3126* (MO); Mt. Grace, Mount Grace, 27 Apr 1910, *Broadway, W.E. 3620* (BM, E); Crown Point, 2 Apr 1993, *Clement, E.J. & Ryves, T.B. TB93/ 723* (BM); sin. loc., *Rand, R.F. s.n.* (BM); Bacolet Point, 10 Oct 1937, *Sandwith, N.Y. 1703* (K). **Trinidad**: Woodbrook, open lands, 22 May 1929, *Broadway, W.E. s.n.* (BM); St. Annes, 25 May 1907, *Broadway, W.E. s.n.* (E); roadside between Cap de Ville and Erin, 26 Mar 1908, *Broadway, W.E. 2817* (F); 1877, *Fendler, A. 606* (BM); Maracas Valley, Oct 1982, *Seaforth, C.E. s.n.* (BM); Roadside between Cap-de-Villo and ?, 26 Mar 1908, *Without Collector 2817* (E).

TURKS AND CAICOS ISLANDS. **East Caicos**: Scrub around airport runway, 23 Apr 1971, *Burch, D. 4307* (MO); **Middle Caicos**: Big Pond Field Road, Big Well, alongside road, 26 Jan 2008, *Corcoran, M.R. et al. MARC-42* (K).

UNITED STATES OF AMERICA. near Indian Village at the confluence of the Columbia, 1825, *Douglas, D. 23* (BM); Novo Eboraco, 1839, *Gray, A. s.n.* (G-DC); sin. loc., *Herb. Richardson*, *s.n.* (BM); sin. loc, 1838, *Wilkes, C. & US Exploring Expedition under the command of Capt Wilkes s.n.* (US). **Alabama**: Choctaw County, In opening F5, floodplain of Tombigbee R in James River Co. Naheola Reserve, E of Pennington, 21 Jun 1994, *Crouch, V.E. 1029* (UT); Mobile County, Mobile, Jun 1905, *Dukes, W.C. s.n.* (ECON); Baldwin County, Romar Beach, 22 Oct 1969, *Kral, R. 38204* (MO); Mobile County, area by Mobile decks, just N of North boundary of old AFB, 14 Aug 1973, *Kral, R. 51349* (MO); Gevington County, Conechu National Forest; Blue Springs Game Mgmt Area, 26 Jun 1974, *Kral, R. 53519* (MO); Baldwin County, Mobile Causeway, vicinity Blakely River, 3 May 1967, *McDaniel, S. 8866* (FSU, MO); Baldwin County, along Hwy 98 through marshes and edge of Mobile Bay, just W of I-10 overpass, 22 Jul 2013, *Nee, M. & McClelland, D. 60237* (MO, NY); Baldwin County, edge of Mobile Bay at USS Alabama Park, S of Battleship Parkway Hwy 98, 22 Jul 2013, *Nee, M. & McClelland, D. 60242* (MO, NY). **Arizona**: Mohave County, Tassi Spring, Grand Canyon Parashat National Mounment, 29 Mar 2001, *Atwood, N.D. & Madsen, M. 26560* (NY); Mohave County, Tassi Spring, Grand canyon-Parashant National Monument, historic home site, 25 Oct 2002, *Atwood, N.D. et al. 29082* (MEXU); Yavapai County, ca. 2.5 km downstream from Verde confuence with Sycamore canyon, 9 km N of Clarkdale, 15 Sep 2012, *Coburn, F.S. 1263* (NY); Mohave County, Oatman Spring, at head of Silver Creek, approx. 6 miles N of Oatman, 29 Jun 1967, *Crutchfield, J.R. & Mitchell, R.S. 3199* (NY); Pima County, Organ Pipe Cactus National Monument, Quitobaquito, 26 Apr 1990, *Felger, R.S. et al. 90-73* (ASU, NY); Maricopa County, Phoenix, Tres Rios project area, near 91st Ave, Salt River, 5 May 2007, *Jenke, D. & Landrum, L.R. 17* (NY); Pinal County, Sacaton, 26 Sep 1929, *Kearney, T.H. 6249* (GH); Pinal County, Tres Rios project along Salinas River, 10 Nov 2009, *Landrum, L.R. & Grass Class 11349* (GH); Pima County, Santa Crux River just W of Sanders Road crossing, 23 Jun 2015, *Makings, E. & Doan, S. 4677* (NY); Yavapai County, Coconino National Forest, Verde River, ca. 0.5 miles SE of Bridge on road to Tuzigoot Natiobal Monument, 19 Sep 1976, *McGill, L. & Lehto, E. 20785* (NY); Mohave County, Grand Canyon National Park, on the banks of Separation Canyon about 100 yards upstream from Colorado River, Grand Canyon Mile 139.5 R, 7 Apr 1993, *Phillips, A.M. & Phillips, B.G. 9372* (MEXU, NY). **California**: Tehama County, Elder Creek, Lowrey's, 22 Sep 1895, *[LFW]*, *83* (US); Los Angeles County, 29 Jun 1902, *Abrams, L. 2520* (BM, K); Los Angeles County, Soldiers Home, 20 Jun 1902, *Abrams, L. 2570* (GH); Sutter County, On the south side of South Butte, Sutter Buttes, west of Marysville, 22 Oct 1984, *Ahart, L. 4830* (MO); Butte County, about 1/10 of a mile S of the clay burrow for the Oroville Dam, 1/4 mile E of Larkin Road, about 6 miles SW of Oroville, 9 Nov 1986, *Ahart, L. 5440* (NY); Yuba County, on the disturbed area on the south side of Woodruff Lane, just west of Kimball Lane, about 5 miles (air) northeast of Marysville, 5 Sep 1999, *Ahart, L. 8200* (JEPS); Butte County, about 2 miles north and 1/4 mile east of Honcut, Peter Ahart Ranch, Oroville, Peter Ahart Ranch, about 2 miles north and 1/4 mile east of Honcut, about 12 miles south of Oroville, 18 Sep 1999, *Ahart, L. 8261* (JEPS); Butte County, about 2 miles north and 1/4 mile east of Honcut, Peter Ahart Ranch, Oroville, Peter Ahart Ranch, about 2 miles north and 1/4 mile east of Honcut, about 12 miles south of Oroville, 18 Sep 1999, *Ahart, L. 8261* (JEPS); Butte County, about 1 mile southeast of Ord Ferry Ord Ferry, Chico, northwest of Parrott Landing, about 1 mile southeast of Ord Ferry, about 2 miles southeast of Ordbend, about 12 miles (air) southwest of Chico, 28 Oct 1999,

Ahart, L. 8319 (JEPS); Butte County, South edge of the North Fork of the Feather River, north side of Highway 70, about 1/8 mile west of the Poe Dam, about 3/4 mile northeast of Pulga, about 21 miles (air) northeast of Oroville, 11 Sep 2006, *Ahart, L. 13296* (JEPS); Butte County, On the east side of the North Fork of the Feather River, on the west side of Highway 70, about 50 yards southwest of the Shady Rest Area south of the Arch Rock Tunnel, 28 Sep 2006, *Ahart, L. 13377* (JEPS); Butte County, Below the high water line of Lake Oroville, about 1/2 mile east of the Power House below Kunckle Reservoir, about 3/4 mile north of the Lime Saddle Campground, about 5 1/4 miles (air) southeast of Paradise, 11 Mar 2007, *Ahart, L. 14573* (JEPS); Mendocino County, On the south side of the Russian River, below the high water line of Lake Mendocino, about 200 yards south of Highway 20, 12 Nov 2009, *Ahart, L. 16540* (JEPS); Tehama County, Along Toomes Creek, Toomes Creek at Tehama-Vina Road crossing, 1.2 miles (air) east of the Sacramento River, 3.4 miles (air) southeast of Los Molinos (High School), 3.0 miles (air) northwest of Vina, 4 Oct 2010, *Ahart, L. & Dittes, J. 17296* (JEPS); Butte County, South of CARD community, Lower Bidwell Park, Chico, 14 Jun 2012, *Ahart, L. 18185* (JEPS); Butte County, On the north side of Big Chico Creek, south of Table 34, west of Cedar Grove, Lower Bidwell Park, Chico, 20 Oct 2012, *Ahart, L. 18514* (JEPS); Colusa County, Strand of east Park Reservoir, about 2.5 air miles Se of Stonyford, about 2.5 miles NW of Lodoga, about 10 air miles NW of Sites, 1 Oct 2013, *Ahart, L. 19082* (JEPS); Butte County, Below the high water line of Lake Oroville, along the sides of Sucker Creek, about 30 yards north of the South Fork of the Feather River, about 1/4 mile northwest of the Ponderosa Dam, 13 Nov 2013, *Ahart, L. 19248* (JEPS); Butte County, Just below the high water line of Lake Oroville, about 50 yards west of the enterprise Boat Launch Area, about 11 miles (air) northeast of Oroville, 17 Nov 2014, *Ahart, L. 19911* (JEPS); Butte County, About half way of and east of One Mile Pond, about 6 miles (air) east of Biggs, 20 Oct 2015, *Ahart, L. 20562* (JEPS); Butte County, About a...>, mile southwest of the parking area at the end of Old Highway Alternate 40, strand of Lake Oroville, about 4 miles (air) south of Highway 70, about 8 miles (air) southeast of Paradise, 7 Nov 2015, *Ahart, L. 20631* (JEPS); Sonoma County, Sonoma Mountain Road, just off Warm Springs Road, 30 Sep 1985, *Anderson, B. 2832* (MEXU); Fresno County, 0.3 mi e of King Slough (at Whites Bridge, on state hwy 180, at Southern Pacific Railway crossing), Whites Bridge, 9 Nov 1962, *Bacigalupi, R. & Heckard, L. 8779* (JEPS); Tulare County, creek near Cold Spring entrance (Sequoia National Park, Mineral King Road), 10 Jul 1948, *Bailey, H. & Bailey, V. 2060* (UC); Santa Clara County, Stanford University, 15 Dec 1901, *Baker, C.F. 231* (E, ECON, F, GH, H, K, LE, MO, UBC, UC, UT); Los Angeles County, Waste ground between Beach Boulevard (Hwy 39) and Coyote Creek, N of Rosecrans Avenue, La Mirada, 18 Jan 1989, *Barbe, G.D. 4313* (UC); San Francisco County, San Francisco, Nov 1837, *Barclay, G.W. 1605* (BM); San Francisco County, San Francisco, *Barclay, G.W. 1607* (BM); San Bernardino County, Mohave Desert, Deggett, Van Dyke Ranch Deggett, Mohave Desert, Van Dyke Ranch, 25 Oct 1933, *Beal, M. s.n.* (JEPS); Tuolumne County, 1 mi e of Jacksonville, 1 Dec 1935, *Belshaw, C.M. 205* (UC); Santa Cruz County, Ridge w of Larkin Valley, 21 Feb 1936, *Belshaw, C.M. 1489* (UC); Orange County, Bolsas Marsh, 28 Jun 1932, *Booth, L.M. 1213* (JEPS, UC); Contra Costa County, Mount Diablo, Sheep Camp Spring, by west road Sheep Camp Spring; Mount Diablo, Sheep Camp Spring, 29 Aug 1931, *Bowerman, M.L. 1029* (UC); San Diego County, Southern Santa Ana Mountains, San Mateo Canyon Wilderness Area, SE of Indian Potrero at base of Easternmost segment of Clark Trail, 29 Apr 1992, *Boyd, S. & Ross, T. 7135* (MO, NY); Los Angeles County, Transverse Ranges, Liebre Mountains region, southwestern shore of Quail Lake, extreme western end of Mojave Desert at north base of Liebre Mountains, 11 Aug 2006, *Boyd, S. et al. 11702* (UC); Amador County, vicinity of Ione, Aug 1904, *Braunton, E. 1202* (MO, NY, UC); Mendocino County, Mendocino, Aug 1898, *Brown, H.E. 932* (F, GH); San Diego County, S of San Diego River, between Fashion Valley Mall Road and I-263, behind Town and Country Resort and Convetion Center at 500 Hotel Circle North, 9 Feb 1999, *Bryson, C.T. 16966* (NY); Siskiyou County, near Yreka, 18 Aug 1909, *Butler, G.D. 1010* (UC); Alameda County, Tunnel Rd, 17 Aug 1935, *Carter, A.M. & Lee, E. 943* (JEPS, UC); San Mateo County, w of Colma, 15 Mar 1901, *Chandler, H.P. 814* (UC); San Diego County, La Jolla, Howard Canyon, 23 Apr 1914, *Clements, F.E. & Clements, E.S. 87* (F, GH, UC, WIS); Tulare County, Visalia, 31 Oct 1881, *Congdon, J.W. s.n.* (RB); Humboldt County, Bull Creek Road to mouth of (and up) Bull Creek (Bull Creek Region, South Fork eel River); Bull Creek Region, South Fork eel River, 2 Jun 1934, *Constance, L. 786* (JEPS); Marin County, Bodega Lagoon, 21 May 1938, *Cook, S.A. s.n.* (GH); Tulare County, near Visalia, 20 Jul 1891, *Coville, F.V. & Funston, F. 256* (US); Tulare County, Near

Visalia, 20 Jul 1891, *Coville, F.V. & Funston, F. 1256* (K); San Francisco County, San Francisco, Aug 1925, *Dempster, L.T. 26* (UBC); Marin County, Inverness, 24 Jun 1915, *Eastwood, A. 4783* (GH); Humboldt County, Fernbridge, 0.5 miles S of Route 101, along W bank of Eel River, about 0.5 miles N of bridge on wide gravel bar, 26 Aug 1987, *Elias, T.S. & Elias, B.A. 10628* (MEXU); Alameda County, Berkeley, 1859 Catalina Ave, 6 Jul 1996, *Ertter, B. G-124* (MO); Contra Costa County, between Orinda and el Sobrante (s end of San Pablo Reservoir), San Pablo Reservoir, 28 Sep 1991, *Ertter, B.J. & Olson, B. 10845* (UC); Contra Costa County, just w of Antioch Marina (Dow Wetlands Preserve, levee at n-center of preserve, at end of road); Dow Wetlands Preserve, 20 Oct 1991, *Ertter, B.J. et al. 10879* (UC); Alameda County, Radio Point Beach at east end of Bay Bridge, dunes and marsh north of Toll Plaza, San Francisco Bay, west Oakland, 23 Sep 1994, *Ertter, B.J. et al. 13908* (UC); Contra Costa County, Northwest base of Mount Diablo, access to Foothills Regional Park from North Gate Road along Little Pine Creek, 5 Jul 1996, *Ertter, B.J. et al. 14924* (UC); Contra Costa County, West side of Mount Diablo, North Gate Road near ranches, 25 Oct 1996, *Ertter, B.J. & Thayer, C. 15384* (UC); Contra Costa County, North base of Mount Diablo, downstream from Mitchell Canyon parking lot, 1 Nov 1996, *Ertter, B.J. & Bowerman, M. 15395* (UC); Ventura County, W end of Los Turas Lake, Santa Monica Mountains, 11 Oct 1931, *Ewan, J.A. 5250* (UC); Alameda County, 4 mi s of Hayward (Zanja Creek, along Foothill Blvd.); Zanja Creek, 1 Oct 1933, *Ewan, J.A. 8157* (UC); San Mateo County, on state highway 5, above Sharp Park near junction of State Highways 5 and 1, 25 Sep 1945, *Ferris, R.S. 11104* (GH); Ventura County, San Nicolas Island, U.S. Naval Radiological Defense Laboratory, at the end of Thousand Springs Road San Nicolas Island, U.S. Naval Radiological Defense Laborato, 7 Apr 1966, *Foreman, R.E. & Smith, D. 194* (UC); road to San Jose water reservoir, 28 Jul 1965, *Frank, M.E.W. 137* (UBC); Tulare County, Middle Fork Kaweah River, Hospital Rock Middle Fork Kaweah River; Sequoia National Park, Middle Fork Kaweah River, 10 Oct 1914, *Fry, W. 339* (JEPS); Los Angeles County, near Pasadena, 15 Sep 1906, *Grinnell, J. & Grinnell, H.W. s.n.* (UC); Amador County, Jul 1985, *Hansen, G. 1175* (BM, E, K); Amador and Calaveras County, Sequoia gigantea Region, Mokelumme River and immediate tributaries. Crow point, Jul 1895, *Hansen, G. 1388* (BM, E); Siskiyou County, Oak Bottom near Somes Bar, 31 Aug 1938, *Harris, S.K. & Leland, R. 5401* (GH); El Dorado County, SE Sacramento Valley, 2 miles W of Latrobe on S Shingle Road, 12 Nov 2011, *Helmkamp, G.K. & Helmkamp, E.A. 18451* (MO); Inyo County, Darwin Falls, 28 Apr 1940, *Hitchcock, C.L. 6224* (UC); San Francisco County, above Lake Merced, 20 Mar 1927, *Hodges, M. 96* (MEXU); San Francisco County, above Lake Merced, 20 Mar 1927, *Hodges, M. 97* (MEXU); Stanislaus County, Modesto, 20 Oct 1934, *Hoover, R.F. 52* (UC); Kern County, Kernville, 14 Sep 1965, *Howell, J.T. 41381* (GH); Napa County, Napa River Basin, St. Helina St. Helina; Napa River Basin, St. Helina, 5 Nov 1894, *Jepson, W.L. s.n.* (UC); Alameda County, Berkeley, Oct 1894, *Jepson, W.L. s.n.* (GH); Alameda County, Berkeley, 21 Dec 1917, *Jepson, W.L. 7448* (JEPS); San Joaquin County, lower San Joaquin River, Union Island San Joaquin River; Union Island, San Joaquin River, 22 Oct 1923, *Jepson, W.L. 10271* (JEPS); Santa Cruz County, Ben Lomond; Santa Cruz Mountains, Ben Lomond, 27 Oct 1934, *Jepson, W.L. 16911* (JEPS); Humboldt County, South Fork eel River; Philipsville, South Fork eel River, 12 Aug 1936, *Jepson, W.L. 17889* (JEPS); Sacramento County, Andrus Island, Lower Sacramento River Andrus Island; Lower Sacramento River, 5 Oct 1893, *Jepson, W.L. 21251* (JEPS); Solano County, near Vacaville (Alamo Creek); Alamo Creek, Sep 1891, *Jepson, W.L. 21253* (JEPS); Alameda County, Berkeley, Aug 1894, *Jepson, W.L. 21254* (JEPS); Alameda County, Berkeley, Aug 1891, *Jepson, W.L. 21255* (JEPS); Los Angeles County, [el Monte]? [el Monte], 29 Jul 1918, *Johnston, I.M. 2102* (UC); Santa Cruz County, c. 3 mi NEN of Mt Hermon, along Zyance Ck Rd, Santa Cruz Mountains, 3 Nov 1962, *Kaune, S.M. 616* (K); Contra Costa County, Crockett -edwards Canyon, 2 May 2004, *Kelch, C.G. 4 0.163* (JEPS); Merced County, Merced Lake, 22 Aug 1868, *Kellogg, A. & Harford, W.G.W. 716 b* (BM); Orinda, Canyon Road, 9 Sep 1956, *Kuijt, J. s.n.* (UBC); Sonoma County, Bodega Bay, 26 Dec 1992, *Landrum, L.R. 7807* (NY); San Francisco County, San Francisco Sand Dunes, Merced Lake Merced Lake; San Francisco Sand Dunes, Merced Lake, 19 Oct 1924, *Mason, H.L. 1129* (UC); Santa Barbara County, Carpenteria, 27 Nov 1929, *Mason, H.L. 4150* (UC); Contra Costa County, 1 mi e of junction with road to Antioch (Bordon Highway); Bordon Highway, 21 Sep 1929, *Mason, H.L. 5445* (UC); Solano County, Cordelia Slough, Suisun Marsh Cordelia Slough; Suisun Marsh, 26 Jun 1947, *Mason, H.L. & Gran, V. 13259* (UC); San Diego County, Mission Hills, 23 Nov 1919, *Millspaugh, C.F. 4436* (F); San Francisco County, Lake Merced, W shore, 13 Sep

1936, *Morrison, J.L. 1882* (JEPS); Alameda County, Lower Wildcat Canyon; Berkeley Hills, Lower Wildcat Canyon, 20 Mar 1931, *Mossman, E.L. 68* (JEPS); Alameda County, Berkeley, on campus of the University of California at Berkeley, 25 Apr 1986, *Nee, M. 32519* (MO, NY); Merced County, 1.5 mi n of Los Banos (Los Banos Wildlife Refuge); Los Banos Wildlife Refuge, 8 Jul 1948, *Nobs, M.A. & Smith, G. 50* (UC); Yolo County, n of Winters (Sacramento Valley, San Joaquin Valley); Sacramento Valley, San Joaquin Valley, 18 Jul 1949, *Nobs, M.A. & Smith, G. 1003* (UC); N. Cal, *Nuttall, T. s.n.* (BM); Alameda County, Univ. of Calif campus [=UC Berkeley], 18 Sep 1919, *Ottley, A.M. 7* (NY); Mendocino County, Hickey Grove, a few mi W of highway junction Hickey Grove, 13 Jul 1947, *Paddock, E.F. 164* (UC); San Luis Obispo County, about 2 mi W of Shandon (beneath bridge, about 100 yards w of site where numbers 405 and 406 were collected (JULY 16)), 4 Aug 1951, *Paddock, E.F. 420* (UC); San Joaquin County, near Tracy, 22 Aug 1892, *Palmer, E. 2689* (UC); San Bernardino County, Santa Ana River near San Bernardino; Santa Ana River, 20 Jun 1914, *Parish, S.B. 9405* (UC); Inyo County, Darwin Falls; Argus Mountains, Darwin Falls, 7 Apr 1936, *Peirson, F.W. 11812* (JEPS); Marin County, Marin Island, 26 May 1989, *Powell, J. 1568* (UC); Fresno County, margin of swampy woods NE of Centerville, May 1930, *Quibell, C.F. 1729* (NY); Marin County, n of Bolinas (Alamere Creek, 1/2 mi from mouth); Alamere Creek, 13 Jun 1930, *Randall, M. s.n.* (UC); San Francisco County, at northern end of Lake Merced Lake Merced, 12 Apr 1954, *Raven, P.H. 6572* (JEPS); Los Angeles County, E end of Malibu [Malibou] Lake, Santa Monica Mountains, 14 Oct 1959, *Raven, P.H. & Thompson, H.J. 14635* (GH); Alameda County, UC Campus, Strawberry Canyon, w of the Botanical Garden Strawberry Canyon; Berkeley, UC Campus, Strawberry Canyon, 10 Jul 1943, *Rodin, B. 247* (UC); San Francisco County, gully E of Lake Merced, 22 Sep 1966, *Rose, L.S. 66088* (GH, H, W); Napa County, SW of Buchli Sation near countyline, 13 Jul 1986, *Ruygt, J. 1880* (JEPS); Riverside County, Mystic Lake, San Jacinto Valley, Mystic (San Jacinto) Lake, 1.4 mile SE of Eden Hot Spring, 26 Sep 1999, *Sanders, A.C. & Provance, M. 23119* (MEXU); San Joaquin County, Stockton, 1890, *Sanford, J.A. 57* (UC); San Joaquin County, Stockton, 1890, *Sanford, J.A. 386* (UC); San Francisco County, Coyote Gulch, at north end of Baker Beach, San Francisco Presidio, 28 Mar 2014, *Schneider, A.C. & Fawcett, S. 513* (JEPS); Marin County, Avalis Beach, 12 Apr 1936, *Schreiber, B. 2189* (UC); Tulare County, Tule River Canyon, along California highway 190 west of the powerhouse at the Wishon Fork Tule River, 24 Aug 1984, *Shevock, J.R. 11009* (MO); Kern County, Kern River Canyon, along California highway 178 about 9 miles above mouth of canyon, 22 Sep 1984, *Shevock, J.R. 11030* (MO); Orange County, Santa Ana River, 21 Oct 1935, *Shields, L. s.n.* (UT); Alameda County, Oakland, Jun 1886, *Shockley, W.H. s.n.* (JEPS); Kern County, Laurel Beach, 24 Jun 1937, *Simontacchi, A. 330* (UC); Yolo County, University of California campus, Davis, Yolo County, California, 5 Jun 1948, *Skoss, J.D. 17* (UC); Santa Barbara County, W of Goleta (Isla Vista Tract, in yard of Jose Torres on Sueno Road), 1 Aug 1949, *Smith, C.F. 2443* (UC); Humboldt County, C. 4 miles SE of Arcata, along Jacoby Creek, 20 Nov 1968, *Smith, D.K. 79* (H); San Francisco County, Fort Funston, 28 Jul 1940, *Stebbins, G.L. & Paddock, E.F. 87* (GH); Alameda County, Berkeley, Fish Ranch Road, 25 Aug 1940, *Stebbins, G.L. & Paddock, E.F. 92* (UBC); Santa Clara County, Hecker Pass, (nearby #104) Hecker Pass, 6 Oct 1940, *Stebbins, G.L. & Paddock, E.F. 105* (UC); San Mateo County, San Bruno Mountain, northern slope of southeast ridge of the mountain, near the ridgecrest, right-of-way of eastern-most set of PGande high-voltage powerlines crossing the mountain, 30 Mar 1988, *Taylor, D.W. 9474* (JEPS); San Mateo County, Montara Mountain, saddle between North Peak and Montara Knob, 1 Aug 1989, *Taylor, D.W. 10409* (JEPS); Butte County, Butte County line at Hwy 70, between boundry and Arch Rock tunnel, 28 Sep 1979, *Taylor, M.S. 2195* (MO); Alameda County, Berkeley, U.C. Campus Berkeley, San Francisco Bay Region, U.C. Campus, 17 Oct 1903, *Tracy, J.P. 1983* (UC); Humboldt County, Loleta, Northern Coast Ranges, Loleta, 20 Oct 1912, *Tracy, J.P. 3992* (UC); Humboldt County, near Willow Creek (Trinity River Valley); northern Coast Ranges, Trinity River Valley, 13 Sep 1919, *Tracy, J.P. 5202* (UC); Humboldt County, Shively, 4 Nov 1934, *Tracy, J.P. 13698* (UC); Humboldt County, Trinidad, 5 Oct 1940, *Tracy, J.P. 16746* (GH); Kern County, 11 mi s of Blackwell's Corner (Western San Joaquin Valley, Santos Canyon), Temblor Range, Western San Joaquin Valley, Santos Canyon, 11 Nov 1953, *Twisselmann, E.C. 822* (JEPS); Kings County, Kings River, W of Stratford, 22 Nov 1963, *Twisselmann, E.C. 9094* (GH); San Luis Obispo County, Palo Prieta Creek below Ortega Spring (western San Joaquin Valley); Temblor Range, Palo Prieta Creek, 9 Sep 1967, *Twisselmann, E.C. 13683* (JEPS); Kern County, Weldon, Rancheria Rancheria; Weldon, Rancheria, 12 Jul 1932,

Voegelin, E.W. 12 (UC); Alameda County, vicinity of Berkeley, May 1906, *Walker, H.A. 178* (BM, H, UC); Los Angeles County, Pasadena, Mar Vista and Colorado Blvd, 15 Sep 1975, *Wheeler, L.C. s.n.* (F); Los Angeles County, San Gabriel Mtns, Mountain Brook Ranch, 8 Sep 1968, *Wheeler, L.C. s.n.* (MO); Los Angeles County, 8 mi N of Long Beach (Atlantic Avenue); Atlantic Avenue, 29 Oct 1932, *Wheeler, L.C. 1439* (UC); Riverside County, Santa Ana River at Chino Creek Santa Ana River, Santa Ana River. Chino Creek, 27 Jul 1933, *Wheeler, L.C. 2041* (UC); Ventura County, First ravine east of Oak Caynon, West Anacapa Island, 21 Jul 1962, *Wheeler, L.C. 8165* (F, MO); San Bernardino County, Lone Pine Canyon, San Gabriel Mountains, 16 Sep 1967, *Wheeler, L.C. 10798* (GH); Los Angeles County, Se TeHACHAPI MTNS.: Gorman area, sag ponds and adj. slopes just N of Gorman Post Rd, 6 Jul 2006, *White, S.D. & Warniment, A. 11602* (UC); Orange County, Bryant Ranch, Bixby Avenue, 100 ft W of Hansen Road, 100 ft W of SE corner of NE40 of NE60 of Sect. 34, T4S R11W, 7 Jul 1932, *Wolf, C.E. 3837* (US); Santa Clara County, Lindsay [?], 14 Jun 1921, *Wolley-Dod, A.H. 336* (BM, K); Fresno County, along edges of small creek crossing Kings R. trail in Sierra Nat'l Forest, Kings R. Basin, Sierra Nat'l Forest, 7 Oct 1995, *York, D. & Shevock, J. 158* (JEPS). **Florida**: Lee County, Flint Pen Swamp wetlands, site 3, 1 mi S of Corkscrew Rd, Ca. 4 mi E of I-75, T46S, R26E, NW1/4, SE 1/4, sec. 28, 24 Jun 1997, *Anderson, L.C. 17746* (MO); DeSoto County, along US 17, 2 mi. N of Arcadia, 15 Jun 1968, *Beckner, J. 2256* (F); Franklin County, Apalachicola, 20 Jun 1880, *Biltmore Herbarium*, *912 c* (GH); Volusia County, Apalachicola, Jul, *Biltmore Herbarium*, *912 d2* (GH); Monroe County, Key West, *Blodgett, J.L. s.n.* (GH, NY); Gilchrist County, ca. 2 miles W of Bell, ca. junction of routes 236 & 313, *Bohs, L. 3652* (BM); Lee County, Sanibel Island, central Sanibel, 15 Feb 1967, *Brumbach, W.C. 5712* (GH); Lee County, Middle Captiva Island, 21 Dec 1974, *Brumbach, W.C. 8720* (GH, NY); Alachua County, ca. 1 m S of Gainesville on FLA441, 29 May 1965, *Burch, D. 677* (MO); Brevard County, Merritt Island, Merritts Island, Feb 1889, *Canby, W.M. s.n.* (NY); Hillsborough County, W. Tampa, 20 Mar 1923, *Churchill, J.R. s.n.* (GH); Lee County, Sanibel Island, 2 Mar 1954, *Cooley, G.R. 2545* (GH, NY); Hernando County, Indian Hill, N of Chinsegut, 24 Apr 1959, *Cooley, G.R. & Eaton, R.J. 6505* (GH); Alachua County, Vicinity of Lake Alice, Gainesville, 16 Mar 1974, *Croat, T.B. 25067* (MO); Monroe County, Key West, 8 May 1896, *Curtiss, A.H. 5595* (GH); Monroe County, ca 1 mi E of Port Largo, Key Largo, 21 Oct 1967, *D'Arcy, W.G. 2244* (MO); Alachua County, FLA236 ca. 1 mile West of Alachua County line, 26 Oct 1967, *D'Arcy, W.G. 2278* (MO); Bradford County, Bradford and Suwannee Counties, Oct 1967, *D'Arcy, W.G. 2291* (MO); Alachua County, U of Florida Campus, Gainesville, 17 Mar 1968, *D'Arcy, W.G. 2366* (MO); Alachua County, at bottom of the Devil's Millhopper, ca. 5 mi. NW of Gainesville, 18 Mar 1968, *D'Arcy, W.G. 2392* (MO); Taylor County, behind gas station, US27 ca 1 mi S of jucntion US98, W side of Perry, 3 Jun 1968, *D'Arcy, W.G. 2483* (MO); Okaloosa County, Hollywood Avenue, Fort Walton Beach, 3 Jun 1968, *D'Arcy, W.G. 2493* (MO); Santa Rosa County, junction US 90 & Fla 87, ca. 4 miles E of Milton, 4 Jun 1968, *D'Arcy, W.G. 2500* (MO); Escambia County, US98 at bridge over Pensacola Bay, Pensacola, 4 Jun 1968, *D'Arcy, W.G. 2506* (MO); Jackson County, at Grant ridge, US90, 7 Jun 1968, *D'Arcy, W.G. 2570* (MO); Indian River County, N limit of Indian River Shores, 4 Aug 1968, *D'Arcy, W.G. 2852* (MO); Indian River County, just s of Wabasso Island, in Indian River, 7 Aug 1968, *D'Arcy, W.G. 3002* (MO); Palm Beach County, Palm Beach, 24 Feb 1904, *Deam, C.C. 1834* (GH); Broward County, NE 19th St. just west of State Rd. 7, 19 May 1971, *Dolan, J. 19* (MO); De Soto County, sin. loc, 3 Dec 1903, *Fredholm, A. 6224* (GH); Dade County, Key Biscayne, Cape Florida State Park, near lighthouse, 1 Dec 1968, *Gillis, W.T. 7233* (A); Dade County, Key Biscayne, Cape Florida State Park, 15 Mar 1969, *Gillis, W.T. 7677* (MO); Levy County, Cedar Key, 25 Jan 1955, *Godfrey, R.K. & Redfearn, P. 52830* (GH, NY); Leon County, 2 miles E of Tallahassee, 2 May 1955, *Godfrey, R.K. 53220* (GH); Hernando County, 4.5 miles S of Brooksville, 12 Jul 1958, *Godfrey, R.K. 57199* (GH); Dade County, Miami, 22 Mar 1917, *Harris, J.A. C-17450* (GH); Glades County, around W shore of Lake Okeechobee, ca. 3.5 miles S of Lakeport, 3 Jan 1975, *Haynes, R.R. 4496* (GH); Osceola County, in the Good Samaritan Village, in Kissimmee, 25 Sep 1996, *Henderson, N.C. 96-739* (MO); Lee County, Myers, Jul 1900, *Hitchcock, A.S. 239* (GH, NY); Sarasota County, Palmer Point, extreme south end of Siesta Key, 19 Mar 1997, *Holst, B.K. et al. 6027* (NY); Volusia County, Seebreeze, 3 Mar 1912, *Hunnewell, F.W. s.n.* (GH); Monroe County, Big Pine Key, hammock along old road northeast of Inn, 10 Apr 1951, *Killip, E.P. 41162* (K, US); Monroe County, Big Pine Key, opposite former S.R.D. camp, 17 Dec 1951, *Killip, E.P. 41582* (NY); Lee County, Sanibel Island, Gulf of Mexico, near W coast of Florida, Feb 1907, *Kitching, L. s.n.* (K);

Martin County, Sewall's point, E of Stuart cr Indian River, 22 Mar 1982, *Kral, R. 68065* (MO, NY); Lee County, Pine Island, south of Jct. #767 and #78, 7 Jan 1967, *Lakela, O.K. et al. 30601* (BM); Dade County, Brickell Hammock, vicinity of US#1 and Rickenbacker Causeway, Miami, 16 Apr 1969, *Long, R.W. & Andorfer, H. 2828* (MO); Seminole County, Sanford, at E end of Oakway, 28 Apr 2008, *Longbottom, W.D. & Williams, D.H. 11015* (NY); Monroe County, Big Pine Key, 30 Jan 1940, *Martin, R.F. 1321* (NY, US); Dade County, Miami area, 5 mi N of Cutler, 28 Dec 1963, *Meriläinen, J. & Roe, K. 92* (H); Monroe County, Big Coppitt Key, 29 Dec 1963, *Meriläinen, J. & Roe, K. 102* (H); Orange County, S shore of Lake Apopka, Winter Garden, 2 Jan 1964, *Meriläinen, J. & Roe, K. 1103* (H); in Florida, *Michaux, F.A. s.n.* (G-DC); Dade County, Buena Vista, 17 Jan 1930, *Modenke, H.N. 442* (MO); Dade County, Buena Vista, in old rock pit, 17 Jan 1930, *Moldenke, H.N. 442* (K, NY); Monroe County, Pine Crest, 1 Apr 1930, *Moldenke, H.N. 867 a* (NY); Monroe County, Key West, *Mr Bennett*, *s.n.* (NY); Hillsborough County, private campground 3.3 miles E of Hwy 579 on Hwy 92, 31 Mar 1972, *Nee, M. 4929* (MEXU); Leon County, Tallahassee, around parking lot by Motel 6, 23 Jul 2013, *Nee, M. & McClelland, D. 60246* (MO, NY); Taylor County, Perry, Hwy 19/98 at Hwy 221, 23 Jul 2013, *Nee, M. & McClelland, D. 60247* (MO, NY); Alachua County, Gainesville, University of Florida campus, along Museum road, 24 Jul 2013, *Nee, M. & McClelland, D. 60248* (MO, NY); Palm Beach County, Pahokee, Triangle Park, 101 Conners Highway, Canal Point. UTM Zone 17, 536814mE, 2971190mN. (NAD83/WGS84), 10 Feb 1996, *Nelson, B. et al. 150* (ILLS); Sarasota County, Indianola Property, north of Osprey, west of Highway 41, between South Holiday Drive and Springfield Dr, 16 Apr 1997, *Nolan, M.E. 19* (MO); Lake County, 3 miles S of Clermont, 6 Apr 1970, *Porter, C.L. & Porter, M.W. 10792* (NY, UC); Volusia County, ca. 9 miles S of New Smyrna Beach at Turtle Mound (ancient shell midden), 27 Apr 1981, *Ray, J.D. et al. 10775* (GH); Dade County, Long Rue Key, 23 Apr 1952, *Robertson, W.B. 202* (GH); Key West, Feb 1846, *Rugel, F. s.n.* (BM); Key West, Feb 1846, *Rugel, F. 44* (BM, GH); Miami-Dade County, between Cocoanut Grove and Cutler, 31 Oct 1903, *Small, J.K. & Carter, J.J. s.n.* (K); Dade County, near the unfinished railroad grade between Cocoanut Grove and Cutler, 31 Oct 1903, *Small, J.K. & Carter, J.J. 607* (NY); Palm Beach County, Boca Ratone Lake, below Delray, 19 Nov 1903, *Small, J.K. & Carter, J.J. 952* (NY); Monroe County, lower part of Key Largo, 10 Jan 1909, *Small, J.K. & Carter, J.J. 3029* (NY); Monroe County, Big Pine Key, 27 Feb 1911, *Small, J.K. et al. 3564* (NY); Monroe County, Key West, 15 Nov 1912, *Small, J.K. 3724* (NY); Dade County, hammocks between Miami and Cocoanut Grove, 26 Nov 1913, *Small, J.K. & Small, G.K. 4696* (NY); Dade County, hammocks between Miami and Cocoanut Grove, 26 Nov 1913, *Small, J.K. & Small, G.K. 4703* (NY, UC); Dade County, Sykes Hammock, Everglade Keys, 23 Feb 1915, *Small, J.K. & Mosier, C.A. 5488* (NY); Dade County, Humbugus Prairie, 28 Feb 1915, *Small, J.K. & Mosier, C.A. 5588* (NY, US); Dade County, Humbugus Prairie, 28 Feb 1915, *Small, J.K. & Mosier, C.A. 5592* (NY); Monroe County, Rachel Key, 6 Feb 1916, *Small, J.K. 7465* (NY); Martin County, eastern shore of Lake Okeechobee, 9 May 1917, *Small, J.K. 8254* (GH, NY); Brevard County, Cocoa, Indian River "Coco", 9 May 1918, *Small, J.K. 8712* (NY); Brevard County, coastal dunes opposite Melbourne, 21 Dec 1921, *Small, J.K. et al. 10335* (NY); Brevard County, Merritt Island, kitchen midden opposite tip of Merritt's Island, 8 Jan 1923, *Small, J.K. et al. 10786* (NY); Lee County, vicinity of Fort Myers, 1916, *Standley, J.P. 72* (GH, MO, NY); Monroe County, Big Pine Key, near road to lobstery, 27 Mar 1958, *Stern, W.L. & Chambers, K.L. 317* (GH); Monroe County, Key West, Bladgett[i], *Torrey, J. s.n.* (K); Okaloosa County, in alley between basket shop and Tony's Pizza, just off Main St. Ft. Walton Beach, 11 May 1967, *Ward, D.B. 6404* (MO); Marion County, Ocala, E 14th Street, south side, 19 Dec 1982, *Wible, M. 703* (A); Gadsden County, River Junction, 6 Aug 1927, *Wiegand, K.M. & Manning, W.E. 2821* (GH); Leon County, Edge of parking lot beside Mecca Cafeteria, across from Fla State Univ. entrance gate, Tallahassee, 13 Jul 1967, *Windler, D.R. & Windler, B.K. 2731 b* (MO); Lee County, 3 mi S of Bonita Springs, 8 Dec 1974, *Wunderlin, R.P. et al. 5386* (MO). **Georgia**: Bulloch County, Banks Road, N of Westside Dr. 4 mi. W of Statesboro, 7 May 2005, *Averett, J.E. 1253* (MO); Oglethorpe County, Echol's Mille SE of Point Peter, 4 Aug 1941, *Duncan, W.H. 3835* (MO); Camden County, along Hwy 40, W of I-95 at Exit 3, 25 Jul 2013, *Nee, M. & McClelland, D. 60249* (MO, NY); Bryan County, Richmond Hill, around intersection of I-95 Exit 87 and Hwy 17 around Motel 6, 25 Jul 2013, *Nee, M. & McClelland, D. 60259* (MO, NY); Chatham County, Savannah, historic district between the old train station/tourist information and museum building, now much of the old docks and warehouse region converted to tourist bars and restaurants, 26 Jul 2013, *Nee, M. & McClelland, D. 60270* (GH,

MO, NY); Chatham County, Savannah, along Victory Drive, just W of Harry S Truman Ave, 27 Jul 2013, *Nee, M. & McClelland, D. 60276* (MO, NY). **Idaho**: Canyon County, Nampa, [as Oregon on label], 30 Jul 1894, *Henderson, L.F. 4592* (GH). **Illinois**: Cook County, Chicago. Illinois, 10 Sep 1980, *Burger, W.C. s.n.* (CR); Chicago, Cook Co. 4200 N. Hazel St, 18 Sep 1982, *Nee, M. s.n.* (BM). **Louisiana**: Jefferson Davis Parish, Lake Arthur, 8 Jul 1918, *Dickinson, D. s.n.* (LSU); Livingston Parish, Collected at Tickfaw State Park, about 7 mi SW of Springfield, along Canoe launch Road (part of Pine Hardwood Forest Trail) SSW of parking lot.; Tickfaw State Park, 20 Apr 2006, *Hazel, A. & Urbatsch, L.E. s.n.* (LSU); Plaquemines Parish, Tidewater, growing along an unpaved shell road near a baseball field, 15 Jan 1987, *Lievens, A.W. & Gregory, B.M. 2056* (LSU); East Baton Rouge Parish, Along bike path on east shore of University Lake, Baton Rouge, 4 Dec 1976, *Shirley, M.G. 213* (LSU); Lafourche Parish, LUMCON field station.; LUMCON Field Station, 6 Nov 1982, *Sullivan, G.A. et al. 1193* (LSU); Ouachita Parish, along railroad tracks near Royal Feed and Seed at Desiard and Sixth in Monroe, 28 Nov 1979, *Thomas, D. 69805* (MO); St. Bernard Parish, North Chandeleur Island near Chandeleur Light House in Chandeleur Sound and the Gulf of Mexico NE of Venice, 11 Jul 1984, *Thomas, R.D. et al. 89754* (H); Ouachita Parish, junction of Lapine Road and Cooney Barnette Road, 6.6 miles southwest of La. 34 and La. 3033 southwest of West Monroe, Sec. 19, T16N 2RE, 15 Nov 1985, *Thomas, R.D. 95065* (BM); Orleans Parish, N of New Orleans Convention Center from Howard Street W to elevated highway, 10 Nov 1991, *Thomas, R.D. 126797* (NY); Plaquemines Parish, Port Eads, 22 Aug 1900, *Tracy, S.M. & Lloyd, ? 3 2a* (BM). **Maryland**: Glenn Dale Introduction Garden, 14 Sep 1938, *Cowgill, W.H. 1072* (ECON). **Mississippi**: Harrison County, Gulfport, N of railroad depot, W of 26th Aven, just E of N-S railroad, 20 Jul 2013, *Nee, M. & McClelland, D. 60201* (MO, NY); Harrison County, Gulfport, N of railroad depot, W of 26th Ave, just E of NS railroad, 20 Jul 2013, *Nee, M. & McClelland, D. 60203* (MO, NY); Harrison County, Gulfport, between 27th and 30th Avenues, just N of Hwy. 90, 20 Jul 2013, *Nee, M. & McClelland, D. 60214* (MO, NY); Harrison County, Biloxi, Canaan, Bayview Ave. fishing dock area at edge of inlet of Back Bay, 20 Jul 2013, *Nee, M. & McClelland, D. 60220* (MO, NY); Harrison County, Biloxi, historical site of Old Brick House of Mayor John Henley, Bayview Ave. on Back Bay, 20 Jul 2013, *Nee, M. & McClelland, D. 60223* (MO, NY); Jackson County, Pascagoula, edge of Gulf of Mexico at small park and boat landing at inlet to Lake Yazoo, 21 Jul 2013, *Nee, M. & McClelland, D. 60226* (MO, NY); Jackson County, Pascagoula, edge of Gulf of Mexico at small park and boat landing at inlet to Lake Yazoo, 21 Jul 2013, *Nee, M. & McClelland, D. 60228* (MO, NY); Jackson County, Pascagoula, along cyclone fence at Dupont Avenue, and Front St. near edge of inlet and boat docks, 21 Jul 2013, *Nee, M. & McClelland, D. 60235* (MO, NY); Hinds County, MISSISSIPPI. Hinds Co. JACKSON QUAD, TR006N001E, Sec. 36; on roadside between river channel and Eubanks Creek, just north of riverbend at Lowhead Dam N32¡ 1927, W 090¡ 0911, 19 Aug 1996, *Wieland, R.G. 7527* (DSC). **Missouri**: Saint Louis City, Saint Louis, Baden freight yards of MO, KS, TX railroads, road of Hall Street, 16 Oct 1969, *D'Arcy, W.G. 3899* (NY); Saint Louis City, Saint Louis, Baden freight yards of MO, KS, TX railroads, road of Hall Street, 16 Oct 1969, *D'Arcy, W.G. 3900* (NY). **New Mexico**: Mogollon Mountains, [Grant or Catron County], Aug 1881, *Rusby, H.H. s.n.* (NY). **New York**: 4 miles SE of Rhinebeck, Amy Goldman's heirloom and research garden, 21 Aug 2006, *Nee, M. & Goldman, A. 54632* (BM). **North Carolina**: Cumberland County, N of Rockfish Creek near E end of Claude Lee Road, just E of I-95, E of Hope Mills, 17 Sep 2000, *Sorrie, B.A. 10627* (GH); **Oregon**: Willamette County, Salem, 1871, *Hall, E. s.n.* (F); Benton County, Corvallis; Marys River near its confluence with the Willamette River, 18 Sep 2001, *Halse, R.R. 6115* (BM); Lane County, Off of State Hwy. 126 at its junction with Hill Road ca. 2.5 miles east of Florence, 26 Nov 2012, *Halse, R.R. 8733* (GH, MO, NY, UC); Lane County, Willamette National Forest; along State Hwy 58 ca. 8.7 miles southeast of Dextar, Hardesty Mountain Trailhead turnout; Lookout Point Reservoir, 27 Oct 2014, *Halse, R.R. 9306* (ASU); Clackamas County, Oregon City, Jun 1890, *Hicks, G.H. 256* (GH); Wasco County, John Day Ferry, 6 Sep 1894, *Leiberg, J.B. 877* (GH, NY, UC); Washington County, Forest Grove, 10 Sep 1893, *Lloyd, F.E. s.n.* (NY); Washington County, Forest Grove, Sep 1894, *Lloyd, F.E. s.n.* (NY); "Oregon Boundary Commission -near the 40th parallel of Lat.", 1858, *Lyall, D. s.n.* (K); Willamette County, Salem, 26 Jul 1917, *Nelson, J.C. 1765* (GH); Douglas County, Reedsport, 11 Jul 1967, *Paddock, E.F. 161* (UC); Curry County, 2 miles S of Port Orford, 30 Jun 1919, *Peck, M.E. 8605* (GH, MO); Benton County, Clear Lake, SE of Corvallis, 17 Oct 1953, *Steward, A.N. 6597* (GH); Columbia County, near Clatskanie, 2 Sep 1927, *Thompson, J.W. 3729* (K);

Multnomah County, Hayden Island, 4 Sep 1927, *Thompson, J.W. 3766 a* (MO); Multnomah County, Sauvies Island, 9 Sep 1927, *Thompson, J.W. 3802* (K). **South Carolina**: Lexington County, off Hwy 378, 13 Oct 1994, *Chappell, C. 32* (UBC); Anderson County, Anderson, Brunis Place, 28 Jun 1920, *Davis, J. s.n.* (MO); Richland County, Maxy Gregg Park off Blossom Street, 1 Oct 1994, *Drakeford, W.T. 13* (UBC); Richland County, Columbia, *Herb. Richardson*, *121* (BM); Florence County, Florence, 5 Sep 1957, *Irvine, F.R. 27* (BM); Chesterfield County, Pee Dee River 4 miles SE of Cheraw, 11 Aug 1956, *Radford, A.E. 15688* (UC). **Texas**: Starr County, Terrace of Rio Grande, ca. 4.1 air miles W of jct. FM 650 and US Rt. 83, WNW of Roma. Lower Rio Grande Valley NWR, Fronton Tract. Roma-Los Saenz West Quadrangle. Elev. 205 -210 ft, 19 Apr 1994, *Carr, W.R. & Elliott, L. 13594* (TEX); Bastrop County, N edge of parkign lot for golf course at Bastrop State Park, ca. 1.4 airmiles ENE of jct St Rt 21 and St Rt 95 in Bastrop, area not burned during Bastrop Complex Wildfire of Setember 2011, 13 Jun 2012, *Carr, W.R. 30854* (NY); Cameron County, Brownsville, 10 Dec 1933, *Clover, E.U. 1516* (NY); Aransas County, Goose Island State Park, 6 Jun 1958, *Correll, D.S. & Correll, H.B. 18969* (GH); Kleberg County, 4.7 miles inside city limits of Kingsville from the North, US highway 77, 2 Mar 1987, *Croat, T.B. & Hannon, D.P. 66041* (MO); Cameron County, northwest of Brownsville, along railroad, 12 Mar 1942, *Lundell, C.L. & Lundell, A.A. 10697* (LL, TEX, US); Galveston County, About 0.5km NW of te bay side entrance to the park (management unit 5) along the caracara trail, 12 Jul 2011, *Rosen, D.J. & Winzer, S. 5304* (MO, TEX); Brazos County, Bee Creek Park, 3 May 1994, *Smith, C. 33* (MO); Hidalgo County, Santa Ana National Wildlife Refuge, 14 Apr 1977, *Solomon, J.C. 2697* (MO); Jefferson County, Beaumont City Limits, off 11th St. in back of Red Lobster, 14 Apr 2004, *Stone, J. et al. 4167* (MO). **Utah**: Washington County, Zion National Park, Grotto Campground, 10 Jul 1949, *Bailey, H. & Bailey, V. 3516* (UC); Washington County, Zion National Park, the Narrows Trail, Zion Canyon, 16 Jul 1949, *Bailey, H. & Bailey, V. 3772* (UC); Washington County, Beaver Dam mountains, Lytle Ranch; LB DNA #1015, 29 Sep 2002, *Bohs, L. 3095* (BM, UT); Washington County, N of Kolob Canyon in a small box canyon, 20 Aug 1970, *Fisher, J.C. 1325* (NY); Washington County, Beaver Dam Wash, 12 Sep 1925, *Harrison, B.F. & Woodbury, R. 1221* (UT); Washington County, St. George, 17 Jun 1934, *Harrison, B.F. 6248* (MO); Washington County, Beaverdam Mountains, at Lytles Ranch on the Beaverdam Wash, 9 Jul 1966, *Higgins, L.C. 757* (NY); Washington County, St. George, [locality inferred from Palmer's colelcting itinerary as documented in McVaugh R 1956 (Edward Palmer: Plant explorer of the American West, Univ. Oklahoma Press, Norman, see pp. 60-73), 1877, *Palmer, E. 362 [a]* (NY); Washington County, Zion National Park, 24 Sep 1928, *Thackery, F.A. 524* (DUKE); Washington County, Danish Ranch, Pine Valley Mountains, R14W, T41S, S4, 6 Oct 1985, *Warrick, R.B. 431* (NY). **Washington**: Mud Lake, 14 Nov 1908, *Bailey, W. s.n.* (NY); Grays Harbour County, Basaltic District, in Melbourne [Washington State?, Canada BC?], Aug 1868, *Edwards, H. s.n.* (UC); King County, Seattle, 26 Feb 1889, *Piper, C.V. s.n.* (NY); Columbia [=Columbia River, border or Washington and Oregon], *Scouler, J. 121* (NY); King County, Seattle, 26 Jun 1889, *Smith, E.C. s.n.* (MO); Klickitat County, Bingen, Jul 1909, *Suksdorf, W. s.n.* (NY); Klickitat County, sandy banks of the Columbia River, 8 Sep 1914, *Suksdorf, W. 2318* (GH, NY, UC).

VIRGIN ISLANDS (US). **St. Croix**: Bassin, 17 Mar 1897, *Ricksecker, J.J. 251* (E, F, MO). **St. John**: Dirt road to Bordeaux Mountain, about 1.5 km from Center Line Road, 5 Jan 1990, *Acevedo Rodríguez, P. 3140* (MO); Concordia, Coral Bay Quarter, near fresh water pond, 19 Feb 1991, *Acevedo Rodríguez, P. et al. 4254* (MO). **St. Thomas**: St. Peters (dedit. A. Toeffler), Jan 1881, *Eggers, H.F.A. 286* (K, PAL, W).

2. *Solanum chenopodioides* Lam.
UNITED STATES OF AMERICA. **California**: San Diego County, Mission Hills, 23 Nov 1919, *Millspaugh, C.F. 4432* (F). **Florida**: Alachua County, 2 miles South of FLA 340, 1 mile West of Alachua County line, ca 8 miles SW of High Springs, 12 May 1967, *D'Arcy, W.G. 2467* (MO); Levy County, sin. loc, Jun 1898, *Hitchcock, A.S. s.n.* (F); Lake County, Eustis, Jun 1894, *Hitchcock, A.S. s.n.* (F, MO); Dade County, Miami, Jul 1898, *Hitchcock, A.S. s.n.* (F); Polk County, sin. loc, 26 Apr 1894, *Ohlinger, L.B. 1396* (F); Escambia County, Pensacola, 11 Apr 1893, *Ralfs, P.H. 718* (MO); Escambia County, Pensacola, 11 Apr 1893, *Rolfs, P.H. 718* (F); Seminole County, 17 Home, 25 Oct 1965, *Schallert, P.O. 3765* (W); Palm Beach County, about Boca Ratone Lake, below Delray, 19 Nov

1903, *Small, J.K. & Carter, J.J. s.n.* (F); Sarasota County, Hidden Oaks, entrance number 3, Bahia Vista Road between McIntosh and Cattlemans, 29 Jul 1979, *Watson, J. 12* (F). **Georgia**: Chatham County, Fort Pulaski, Apr 1864, *Grosvenor, J.W. s.n.* (F). **Louisiana**: Cameron Parish, Marsh beside La. 82 at Sabine Pass and the Texas State Line west of Johnsons Bayou, Sec. 12, T15S, R16W, 16 Jul 1988, *Thomas, R.D. & Aquatic Plants Class 105879* (MO). **Maryland**: Baltimore County, Canton, 27 Sep 1953, *Reed, C.F. 32789* (MO). **Missouri**: Saint Louis County, Missouri Botanical Garden, 14 Aug 1989, *Crosby, M.T. 7* (MO). **North Carolina**: New Hanover County, Wilmington, 11 Aug 1970, *Leonard, S.W. & Hackney, J.I. 3470* (MEXU, MO). **South Carolina**: Richland County, alogn entrance road to boat ramp on N side of Congaree River, US 601 bridge, 23 Oct 2009, *Nelson, J.B. 28059* (GH). **Wisconsin**: Richland County, Originally from France, Jardin des Plantes, Montpellier; grown in garden, 3 miles SE of Richland Centre. (T10 N; R 1 E; NE1/4NE1/4 Sec. 35), 7 Oct 1977, *Nee, M. 14678* (BM, K, MEXU); Richland County, 3 miles SE of Richland Center, 24 Sep 1978, *Nee, M. 16093* (CORD, RB); Richland County, 3 miles SE of Richland Center, 28 Sep 1979, *Nee, M. 18112* (MO).

3. *Solanum corymbosum* Jacq.
MEXICO. sin. loc, *Ruiz, H. & Pavón, J.A. s.n.* (BM); sin. loc, *Schmitz, A. s.n.* (LE); "Solanum de Mexico", *Sessé, M. & Mociño, J.M. s.n.* (BM); sin. loc, *Sessé, M. & Mociño, J.M. 1414 bis* (MA). **Chiapas**: Tapachula, terreno plano a la orilla de camino, 24 Sep 1984, *Ventura V, E. et al. 345* (MO). **Distrito Federal**: Texcoco, San Vicente Chicoloapan, municipio de Texcoco, 16 Sep 1982, *Rzedowski, J. 54* (CORD); Texcoco, San Vicente Chicoloapan, municipio Texcoco, 16 Sep 1982, *Ventura A, A. 4105* (CORD, MO). **Guanajuato**: Cerro del Santuario de Guadalupe, Guanajuato, 23 Oct 1977, *Arellano, M. 12* (CORD); Rancho La Misión, 8km al Noreste de San Luis de la Paz; matorral arbustivo, terreno plano, 1988, *Ventura V, E. & López, E. 6116* (MO). **Hidalgo**: Huichapan, alrededores del pueblo; chaparrel espinoso y cultivadas; em un cellajón, 17 Mar 1979, *Argüelles, E. 1211* (MO); 2 km al E de Pachuaca [Pachuca], 6 Feb 1966, *Cruz-Cisneros, R. 472* (US); Zimapán, Zimapán, 10km al NW de Zimapan, 27 Oct 1965, *Gonzales Q, L. 3240* (CORD); Cardonal, 22 kms al Este de Ixmiquilpan; hacia Tolangongo, 3 Oct 1979, *Hernández Magaña, R. 3752* (MO); Cardonal, Cardonal, 21 km al E de Ixmiquilpan, 11 Sep 1981, *Hernández Magaña, R. & Hernández M, I. 6436* (CAS, MO); Pachuca, 2km al ESE de Pachuca, municipio de Pachuca, 22 Oct 1977, *Medina C, M. 2095* (CORD); 4 km al ESE de Pachuca, sobre la carretera a Tulancingo, 14 Aug 1966, *Rzedowski, J. 22943* (CORD, MO); Zempoala, Zempoala, municipio Zempoala, 28 Sep 1975, *Ventura A, A. 345* (CORD). **México**: Sierra de Guadalupe, 21 Jul 1938, *Balls, E.K. B 5084* (BM, E); entre Lerma et Montepoyo [prob. valley of Mexico], *Schaffner, J.G. s.n.* (G); Valle de Mejico, *Schmitz, A. 1655* (BM); Texcoco, San Vicente Chicoloapan, 16 Sep 1982, *Ventura V, E. 54* (MO); Texcoco, Texcoco. Estación del ferrocarril, 15 Nov 1984, *Williams, D.E. 592* (MO). **Puebla**: Puebla, Petite mare devant le Cimetiere de la Piedad, 29 Sep 1906, *Arsène, G. 262* (MO); Puebla, Prés du Cimentiese, 27 Jun 1907, *Arsène, G. 2166* (MO); Puebla, Rancho Pasadas, 15 Jul 1909, *Nicolas, F. s.n.* (LE); Puebla, Rancho Pasadas, 14 Feb 1909, *Nicolas, F. s.n.* (LE). **Zacatecas**: cultivated garden in city. Low annual about 1 meter across, 10 Aug 1948, *Dressler, R.L. 118* (MO).

4. *Solanum douglasii* Dunal
CLIPPERTON ISLAND (Île de Clipperton, minor overseas territory of France, geographically off the Pacific coast of Mexico). Clipperton Island, NE side of island, near landing place, 2 May 1958, *Klawe, W.L. 1465* (US); Clipperton Island, NE side of island, near landing place, 2 May 1958, *Klawe, W.L. 1466* (US); Clipperton Island, flat ground at base of E face of Clipperton Rock, 10 Aug 1958, *Sachet M.H. 309* (US); Clipperton Island, NE side of island, near landing place, 26 Aug 1958, *Sachet, M.H. 358* (US); Clipperton Island, 21 Jul 1938, *Schmitt, W.L. 105* (F, NY, US).

EL SALVADOR. **San Salvador**: Cráter del Boquerón, Volcán de San Salvador. Crece en las laderas externas del fondo del volcán, 1995, *Renderos, M.A. & Renderos, H. de 10* (MO). **San Vicente**:

Volcán San Vicente, 7 Mar 1922, *Standley, P.C. 21476* (NY); Volcán San Vicente, 7 Mar 1922, *Standley, P.C. 21477* (NY). **Santa Ana**: Near top of Cerro Verde. [originally reported as part of dept. Sonsonate], 30 Jul 1977, *Croat, T.B. 42188* (MO); Chalchuapa, Volcán Chingo, falda del sitio, 10 Feb 2009, *Rodríguez, D. et al. 1417* (BM, MO); Cerro Verde National Park, 23 Jan 1998, *Sidwell, K.J. et al. 449* (BM, MO); Slope of Ayeco, northwest flank of Volcan de Santa Ana, 13 Apr 1942, *Tucker, J.M. 1223* (CORD, F, K).

GUATEMALA. Sin. loc., *Fraser, L. s.n.* (BM). **Chimaltenango**: 8 miles W of Patzicía on highway from Patzún, 14 Jul 1977, *Croat, T.B. 41082* (MO); Chimaltenango, Escuela Nacional de Agricultura, 28 Nov 1940, *Cutler, H.C. 4341* (F). **El Quiché**: Nebaj, Arroyo de la Presa, about 500 m E, 7 Jul 1964, *Contreras, E. 5290* (F). **Quetzaltenango**: 8 km S of Quetzaltenango on highway 9s, near hydroelectric plant 0.5 km N of Zunil, 31 Jul 1965, *Roe, K.E. et al. 700* (G). **Sacatepéquez**: 2.3 miles SW of Alotenango on road from Antigua to Escuintla, slopes of Volcán del Fuego, 26 Jul 1977, *Croat, T.B. 41994* (MO). **Sololá**: 15 km S of Panajachel, S of Lake Atitlán, 8 May 1972, *Burch, D. 5991* (F).

HONDURAS. **Francisco Morazán**: Distrito Central, 20 km de Tegucigalpa, Montaña la Tigra, 14 May 1977, *García, M. 53* (MO); Lepaterique, Cerro Lepaterique, cerca del Río Lepaterique, 6 Oct 1970, *Hernández R, J. & Hernández M, M. 5262* (MO); Distrito Central, 20 km de Tegucigalpa, Montaña La Tigra, 5 Jun 1977, *Rubio G, A. 68* (MO); Lepaterique, Cacerio La Brea cerca de Lepaterique, 21 May 1977, *Yong, G. 303* (MO). **La Paz**: Guajiquiro, 24 May 1993, *Liesner, R.L. 26560* (MEXU, MO).

MEXICO. sin. loc., 7 May 1897, *Maltby, F.S. 72* (NY, US); sin. loc., 1833, *Andrieux, G. s.n.* (G); 15 Apr 1847, *Gregg, J. 459* (MO); sin. loc., *Grisebach, A.H.R. s.n.* (K); sin. loc. [prob. also Orizaba], 1846, *Heller, C. 151* (W); sin. loc., Jan 1889, *Palmer, E. 647* (F, NY); "Valley of Mexico", 1905, *Rose, J.N. & Painter, J.H. 9255* (US); Valle de Mejico, 1855, *Schmitz, A. 162* (BM, F, W). **Baja California**: Tijuana, "Tia Juana" [label also suggests this is from San Diego County just over the border in the USA], 14 May 1903, *Abrams, L. 3488* (MO); Rosarito, 1936, *Bravo H, H. s.n.* (MEXU); Ensenada, Santa Catarina, 63 miles SW of Ensenada, Priscilla Flores' milpa, 24 Aug 1960, *Broder, R.E. 313* (US); Ensenada, Santa Catarina, in wash above upper dam, 64 miles S of Ensenada, 19 Aug 1961, *Broder, R.E. 562* (US); Sandy wash along road between Hwy 3 and Rancho Mike in the Sierra de San Pedro Mártir, 7.8 mi SW of Hwy 3, 20 May 1981, *Daniel, T.F. 1407* (ASU); Descanso Bay, Sep, *Fosberg, F.R. 55780* (F); Ensenada, Isla Guadelupe, 1893, *Franceschi, F. s.n.* (US); Ensenada, Isla Guadelupe, "Guadelupe", Dec 1892, *Franceschi, F. 17* (IND, K, LE, MO); Ensenada, Cañón de Doña Petra, atrás de El Popular, 20 Nov 1998, *González R, A. 323* (MEXU); Jamul Valley, Lower California, 2 Jul 1894, *Mearns, E.A. 3845* (NY, US); Ensenada, Isla Guadelupe, South Twin Canyon, 22 Apr 1958, *Moran, R. 6602* (MEXU, NY, UCR, US); Isla Guadalupe, South Twin Canyon, 22 Apr 1958, *Moran, R.C. 6602* (BM, K); Ensenada, 9 mi E of Mision San Borja in the La Libertad Mountains, 19 Dec 1977, *Nixon, K.C. & Sauls, M.L. 974* (MEXU); Near Encenada de Todos Santos Bay, Lower California, 12 Apr 1882, *Orcutt, C.R. s.n.* (MO); Socorro, Northern Lower California, 7 May 1904, *Orcutt, C.R. s.n.* (MO); sin. loc. (at Muleje and Guaymas), 1897, *Palmer, E. 9* (US); Isla Guadalupe, 1875, *Palmer, E. 60 [a]* (MO, UPS); Isla Guadalupe, 1875, *Palmer, E. 61* (BM, MO, NY); wash area bewteen San Francisquito and Colmalli, 28.3 miles SW of San Francisquito, 24 May 1996, *Rebman, J.P. et al. 3175* (ASU); Tijuana, "Lower California, Tia Juana", 1912, *Smith, H.H. 5198* (US); Ca. 7 road miles SW of Ejido San Matiás along road to Mike's Sky Rancho, S of Hwy. 3 between H. de la Independencia and Crucero La Trinidad. In foothills of Sierra San Pedro Mártir, 12 Aug 1995, *Snow, N.W. & Prinzie, T. 6436* (MO); Rancho Loma Linda, Las Animas, 15 Mar 1987, *Thorne, R.F. et al. 62266* (MEXU); junction of Hwy 3 and Laguna Hanson roads, near junction, 27 May 1987, *Thorne, R.F. et al. 62489* (F, RSA); ca. 5 km NW of Punta Blanco, 29 Mar 1987, *Webster, G.L. 26047* (MEXU); Baja California. 2 miles north of Rosarito Beach, 7 Sep 1929, *Wiggins, I.L. & Gillespie, J.W. 3875* (F, MO, NY, SBBG, US); small canyon 25 miles S of Santo Tomás, 26 Feb 1930, *Wiggins, I.L. 4266* (NY, US); bank of Río Santo Domingo, about 4 miles above the Mission, 10 Sep 1930, *Wiggins, I.L. & Demaree, D. 4779* (F, US); Arroyo l'Agua Marga, southern Sierra San Pedro Martir, 13 May 1941, *Wiggins, I.L. 9946* (US); 1.5 miles NW of the village of Valle Trinidad, 3

Apr 1960, *Wiggins, I.L. & Wiggins, D.B. 16062* (LE). **Baja California Sur**: long interior valley (La Laguna) south of Pico La Aguja on hte Sierra La Laguna, 22 Oct 1977, *Breedlove, D.E. & Axelrod, D.I. 43298* (MEXU); Arroyo Hondo, near base of cliffs on N side of Cerro de la Giganta, 27 Nov 1947, *Carter, A. et al. 2093* (US); Llano de Magdalena, 11.5km south of San Domingo, 7 Dec 1947, *Carter, A. et al. 2149* (K, US); Campamento Palo Extraño, Sierra de la Laguna, 9 Sep 1986, *Domínguez Adena, R. 290* (MEXU); La Paz, campamento palo extrano, Sierra de La Laguna, B.C.S, 16 Sep 1980, *León de la Luz, J.L. 4008* (NY); El Chorro, cañon mouth, Cape Region, 30 Apr 1959, *Moran, R. 7299* (MEXU, US); Cerro Azufre, north slope, 14 Apr 1973, *Moran, R. 20503* (MEXU); Occasional on low north slope of Volcán las Tres Vírgenes, 11 Apr 1973, *Moran, R.V. 20412* (MO); Santa Anita, Jan 1906, *Nelson, E.W. & Goldman, E.A. 7549* (NY, US); La Paz, Sierra las Cacachilas; N of Hwy. 286 between La Paz & San Juan de los Planes; W of El Sargento; on top of Cerro Morror to the north of El Picacho, 18 Oct 2014, *Rebman, J.P. et al. 29073* (ARIZ). **Chiapas**: San Cristóbal de las Casas, San Felipe Ecatepec, 9 Jul 1988, *Alemán S, T. 92* (MO); Villaflores, a 0.5 km del poblado Nuevo Independencia, 9 Jul 2004, *Álvarez, D. 9939* (MEXU); Villa Corzo, Predio Particular Santa Martha, 11 Nov 1988, *Bachem C, U. et al. 184* (MEXU); San Cristóbal d Las Casas, N end of San Cristobal de las Casas, 6 Jul 1964, *Breedlove, D.E. 6050* (F, IND); ixtapa, along Mexican Highway 190 in the Zinacatán paraje of Muctajoc, 17 Aug 1965, *Breedlove, D.E. 11819* (F, MEXU, US); San Cristóbal de las Casas, Cerro San Cristóbal, 18 Aug 1965, *Breedlove, D.E. 11911* (F, US); Tenejapa, paraje of Pahal Ton, 1 Oct 1965, *Breedlove, D.E. 12600* (F, US); Motozintla, Steep canyon, SW side of Cerro Mozotal, 11 km NW of the junction of the road to Motozintla along the road to El Porvenir and Siltepec, 27 Jun 1972, *Breedlove, D.E. 25885* (MEXU, MO); Ocosingo, Slope 16 km NE of Oxchuc along road to Ocosingo, 23 Sep 1972, *Breedlove, D.E. 27792* (MO); Motozintla, Steep slope. On the N and W slope of Cerro Mozotal below the microwave tower along the road from Huixtla to El Porvenir and Siltepec, 19 Sep 1976, *Breedlove, D.E. 40279* (MO); Zinacantán, Slope along road from Zinacantan center to Ixtapa near Paraje Vo Bits, 12 Oct 1976, *Breedlove, D.E. 40721* (MO); Motozintla, southwest side of Cerro Motozal, 11 km northwest of hte junction of the road to Motozintla along the road to El Porvenir and Siltepec, 21 Nov 1976, *Breedlove, D.E. 41616* (MEXU); Teopisca, A 5 km al N de la Carr. San Cristobal de las Casas-Comitan con rumbo a Ocosingo, 19 May 1982, *Cabrera, E. & Cabrera, H. de 2817* (MO); a 5 km a; N de la carretera San Cristobal de las Casas-Comitan, con rumbo a Ococingo, 19 Jun 1982, *Cabrera, E. & Cabrera, H. de 2817* (MEXU); San Cristóbal de las Casas, 11 miles W of San Cristóbal de Las Casas, along Highway 190, 7 Jul 1977, *Croat, T.B. 40481* (MEXU, MO); Cacahoatán, Alpujarras, 27 Jul 2005, *Farrera S, O. 3917* (MEXU); Amatenango del Valle, Yax Ja', 6 km al este de Amatenango del Valle, 17 Apr 1988, *Gómez-López, M. 426* (MEXU); along route 190, about 10 miles E of Teopisca, 24 Jun 1960, *King, R.M. 3033* (US); San Cristóbal de las Casas, Barrio Custutal in San Cristobal de las Casas, 21 Jul 1966, *Laughlin, R.M. 1363* (MEXU, US); Tuxtla Gutiérrez, Tuxtla Gutiérrez, Puesto 202 y 203, Mercado Gustavo Diaz Ordaz, 16 May 1991, *Linares, E. & Herrera, E. 1110* (MEXU); Salul Ja', 5km al este de la cabecera de Amatenango;bosque, 9 Mar 1988, *López P, J. 326* (MO); Region north of Bochil, road 195; gravel pit by the side of mountain road, 11 Jul 1965, *Maxwell, R.H. 231* (MO); Yajalón, Rancho San Luis. N. L. "Hioen" (Tzelt, 5 Nov 1982, *Méndez G, A. 4981* (MO); Arroyo Las Piedrecitas en el lado Oriente de San Cristóbal de las Casas, 15 Nov 1985, *Méndez G, A. 8600* (MO); San Cristóbal de las Casas, near San Cristobal, 18 Sep 1895, *Nelson, E.W. 3130* (US); San Cristóbal de las Casas, near San Cristobal, 18 Sep 1895, *Nelson, E.W. 3179* (US); Along eastern ridge of Chiapas Highlands, 15 km N of Pueblo Nuevo on highway 195, 13 Aug 1965, *Roe, K.E. 1263* (NY); Tenejapa, paraje of Matsab, 5 Oct 1966, *Shilom Ton, A. 1280* (IND, MEXU, NY); Pueblo Nuevo Solistahuacán, along ridge above Pueblo Nuevo Solistahuacán, 15 Aug 1967, *Shilom Ton, A. 2766* (NY); Santa Catarina Pantelho, near Rancho Buena Vista near Pantelho, 13 Feb 1968, *Shilom Ton, A. 3670* (MEXU); San Cristóbal de las Casas, Arroyo de las Piedrecitas, 5 Jun 1984, *Shilom Ton, A. 7625* (MEXU); San Cristóbal de las Casas, Cerro Huitepec, al W de San Cristóbal, 10 Jun 1985, *Shilom Ton, A. 8261* (MEXU, NY); San Cristóbal de las Casas, Arroyo del Rancho Pellizzi, al E de San Cristóbal, 20 Jun 1986, *Shilom Ton, A. 9165* (MEXU, NY); San Cristóbal de las Casas, about 14 miles E of Comitan towards San Cristóbal de las Casas, 26 Jul 1984, *Wilbur, R.L. 35744* (DUKE). **Chihuahua**: Madera, proximidades al campo 1, ejido El Largo, 26 Aug 1990, *Benítez, A. 1979* (MEXU); Campsite on road between Babicora and Yepomera. Pine Oak forest sparsely scattered. All volcanic bosaltic soils, *Bennett, C.A. et al. 811* (MO); Madera, La Tinaja, ejido El LArgo, 29 Aug

1990, *Bravo-Bolaños, O. 1333* (MEXU); Madera, Arroyo La Quinta, ejido El Largo, 30 Aug 1990, *Bravo-Bolaños, O. 1380* (MEXU); Madera, Arroyo de las Varitas, ejido El Largo, 1 Sep 1990, *Bravo-Bolaños, O. 1446* (MEXU); Batopilas, Barranca de Batopilas. Vicinity of Arroyo San Fernando and Slope to W, Overlooking Rio Batopilas, W of La Bufa, in short tree forest. tarahumara and Mexican name is Chichiquelite, 23 Apr 1973, *Bye, R.A. 3583* (DES, MO); Cusarare, S of Creel: near the walls of the church, 3 Sep 1973, *Bye, R.A. 4867* (MO); S of Creel, walls of church, Sierra Madre Occidental, 3 Sep 1973, *Bye, R.A. 4867* (MEXU); Batopilas, E of La Bufa, on S side of Barranca de Batopilas in lower Oak zone and Upper short-tree forest, 27 Oct 1973, *Bye, R.A. 5678* (MO); Batopilas, E of La Bufa, on S side of Barranca de Batopilas, 27 Oct 1973, *Bye, R.A. 5678* (MEXU); Bridge over Rio Urique, road from Creel to La Bufa, 7 Oct 1975, *Bye, R.A. 6981* (MO); Guachochi, Ejido Cusarare, E of Casarare. In vicinity of Bajichi, by cultivated land and dwellings (Tara-Humara), Pine-Oak Forest, UTm Zone 13 305 10Km N, 25 10Km E. Weed along margin of Maize field at house of Frederico Gonzales at Puerto Wipinapuchi Tarah, 8 Oct 1979, *Bye, R.A. 9518* (MO); Guerrero, ca. 15 km S de San Pedro, S de Arroyo Seco, ca. km 15 camino San Pedro-San Juanito CHIH-127, 8 Sep 1987, *Bye, R.A. & Ramamoorthy, T.P. 15669* (MEXU); Guachochic, Ejido Cusárare, W de pueblo de Cusárare, 13 Sep 1987, *Bye, R.A. et al. 15800* (MEXU); From Field N of Church, 4 Jun 1984, *Davis, T. 1142* (BM, MEXU, MO); From field N of church. Lots of plants in all fields around small pueblo. Norógachi, 4 Jun 1984, *Davis, T. 1143* (MO); Mojarachic, 4 Aug 1938, *Knobloch, I.W. 5498* (F); Nabogame, Chihuahua, 28 Jul 1987, *Laferrière, J.E. 561* (MO); Nabogame, 10 Sep 1987, *Laferriere, J.E. 1089* (MO); Nabogame, Chihuahua, 21 Sep 1987, *Laferrière, J.E. 1226* (MEXU, MO); Chihuahua, Nabogame, Sep 1988, *Laferrière, J.E. 1958* (MO); Bachimba, 19 Oct 1935, *LeSueur, H. 441* (F, K, MO, US); Organos Mountains, 8 Sep 1937, *LeSueur, H. 1447* (F); SW Chihuahua, Aug 1885, *Palmer, E. 204* (BM, K, NY, US); Yepáchic, 5 Sep 1971, *Pennington, C.W. 42* (MEXU); Ocampo, Parque Nacional de Cascada Basaseachic, on nearly barren rock at overlook ca. 1 km airline S of Cascada, 3 Oct 1986, *Spellenberg, R.W. et al. 8650* (MO); Rancho de Encinillas, below ranch house, 6 Jul 1941, *Stewart, R.M. 729* (BM, GH); El Caracol 30Km, al S de Colonia Juárez. Mpio Casas Grandes. Veg. Encinar. Suelo amarillo forestal, 23 Sep 1982, *Tenorio L, P. & Romero de T, C. 1677* (MO); Ocampo, Cascada de Basaseachic, 4 Oct 1982, *Tenorio L, P. & Romero de T, C. 1955* (MEXU, MO); Batopilas, Pasihuare, 25 km, Brecha Creel-Guachochi. Bosque de pino encino. Suelo amarillo rocoso, 8 Oct 1982, *Tenorio L, P. & Romero de T, C. 1988* (MO); Guachochi, Humirá, 58 km Brecha Creel-Guachochi, 9 Oct 1982, *Tenorio L, P. & Romero de T, C. 2033* (MO); Cascada de Basasechic, 3 km. al SE de Basaseachic. Bosque mixto pinus, Quercus y Cupressus. Suelo rocoso, 26 Sep 1983, *Torres R, R. & Tenorio L, P. 3805* (MO); Sta. Eulalia plains, 21 Jul 1883, *Wilkinson, J. s.n.* (US); Parque Nacional Cascada de Ba saseachic, Mpio de Ocampo, bosque de pino-encino, 1994, *Yen, L. & Estrada, E. 2868* (MO); mountains of southern Chihuahua, 1931, *Zingg, R.M. A-46* (F). **Coahuila**: Villa Acuña, Hacienda Pedra Blanca, Cañón de Sentenela, Sierra del Carmen, 1936, *Bravo H, H. s.n.* (MEXU); ca. 54 air miles SE of Big Bend National Park Basin in S end of Sierra Maderas del Carmen in the cañon de la Fronteriza, 2 miles NE of Rancho San Isidro, at end of Cañon del Alamo, 6 Aug 1976, *Henrickson, J. & Prigge, B. 10539* (MEXU); Cuatrociénegas, Ca. 35 (air) miles W of Cuatro Ciénegas in mid-Canyon de la Hacienda of Sierra de la Madera, 6 Aug 1973, *Henrickson, J. & Wendt, T. 11978* (ARIZ, ASU); Del Carmen Mountains, 9 Aug 1936, *Marsh, E.G. 651* (F, MEXU); Jardín del Sur, 3 Sep 1936, *Marsh, E.G. 770* (MEXU); E edge of campus at Buenavista, 15 Jul 1975, *Seigler, D. & Holstein, G. DS 9303* (MO); Cañon del Invierno (C. El Infiernillo) (N-graining): lower part of canyon, about 1.4 mi. N of woodcutters, 29 Aug 1974, *Wendt, T. & Lott, E.J. 651* (MEXU, MO); Muzquiz, Rincón de María, 24 Aug 1975, *Wendt, T. et al. 1321* (MEXU); Acuña, Municipio de Villa Acuña. Sierra del Carmen; Canyon de Sentenela on Hacienda Piedra Blanca, 7 Jul 1936, *Wynd, F.L. & Mueller, C.H. 567* (NY, US). **Colima**: Cuchilla, northeast of Volcano Colima, 22 Jul 1995, *Goldsmith, P. 64* (F, MO, NY, US). **Distrito Federal**: circa Tolucam, Apr 1834, *Andriewe, G. s.n.* (G-DC); Del. Coyacan, UNAM, Jardín Botánico Exterior, W of Arboretum I, 6 Sep 1999, *Bye, R.A. et al. 26949* (MEXU); Base of road and Cerro Ajusco, 15 Sep 1978, *D'Arcy, W.G. 11916* (MO); Tepozteco, Teposteco, near Mexico City, along new road on geologically recent aa lava flow, 6 Sep 1959, *Degener, O. & Degener, I. 26274* (US); Barrio Los Reyes, San Gregoria Atlapulco, Area Natural Protegida, Ejidos de Xochimilco, Del. Xochimilco, 26 Jul 2005, *Espinosa H, A. et al. 95* (MEXU); Pedregal de San Angel, 18 Jul 1924, *Fisher, G.L. s.n.* (F); San Angel, 18 Jul 1924, *Fisher, G.L. 255* (US); Pedregal de San Angel, al O de la Cuidad

Universitaria, 1 Sep 1954, *Gold, D.B. 629* (MEXU); Pedregal, Aug 1927, *Lyonnet, E. 213* (BM, MEXU, MO, NY, US); Desierto de los Leones, 18 Aug 1935, *MacDaniels, L.H. 93* (F); Sierra de Guadalupe, Cerro Grande, 5 km al NNW de Cuautepec, deleg. G.A. Madero, 1973, *Moreno G, S. 273* (MEXU, MO); San Luis Tlaxialtemalco-Cerranía, 17 Nov 1992, *Nava Rodríguez, V. 85* (MEXU); Pedregal de San Angel, 1 km S of UNAM, 23 Jul 1967, *Nee, M. 255* (MEXU); Pedregal de San Angel, Ciudad Universitaria. Camellón cerca del Instituto de Biología, en el Circuito Escolar CU2 (o Circuito Deportivo), 16 Oct 2015, *Ochoterena, H. et al. 932* (BM, MEXU); Pedregal San Angel, al sur de C.U, 20 Jun 1981, *Proyecto Pedregal de San Angel*, *68* (MEXU); Volcán Malacatepec, a 4.25 km en linea recta del poblado El Capulin, Area Natural Protegida Agua Grande, Del. Tlalpán, 16 Dec 2003, *Rivera H, J. & Espinosa, A. 3413* (MEXU); Tlalpán, 22 Aug 1930, *Russell, P.G. & Souviron, M.J. 46* (US); El Desierto, [georef to ruins of Convento Desierto de los Leones], 25 Aug 1930, *Russell, P.G. & Souviron, M.J. 48* (US); El Corazon, 1 Sep 1930, *Russell, P.G. & Souviron, M.J. 102* (US); Peñón del Marquéz, 27 Apr 1951, *Rzedowski, J. 237* (US); 6 km al SW de Contreras, 8 May 1977, *Rzedowski, J. 34725* (MEXU); 1 km SW de Santa Cruz Acalpixca, ladera W de Cerrp Tecualleli, Deleg. Xochimilco, 9 Sep 1985, *Serrano, J.G. 21* (MEXU); Río de la Magdalena, between Contreras and the 2nd dynamo, 23 Jul 1944, *Sharp, A.J. 4410* (US); El Desierto de los Leones, Aug 1918, *Standley, P.C. 93* (BH); Milpa Alta, San Lorenzo. [San Lorenzo Tlacoyucan], 7 Mar 1976, *Ventura A, A. 1117* (CORD, MO); Milpa Alta, San Lorenzo, 4 Apr 1976, *Ventura A, A. 1265* (CORD, MEXU, MO); Milpa Alta, Cuaute, El Mirador. [Cuauhtec, Santa Ana Tlacotenco], 1 May 1976, *Ventura A, A. 1380* (CORD, MO); Tlalpan, Cerro del Ajusco, 24 Apr 1977, *Ventura A, A. 2713* (CORD, MO); Coyoacán, Jardín Botánico de la Universidad Nacional Autónoma de México, cerca de la entrada del Jardín Botánico pero afuera, 12 Oct 2000, *Vibrans, H. 6813* (MEXU). **Durango**: Suchil, Potrero Los Anegados, a 3 Km. de Alemán. Cerca de arroyo en bosque de encino, 1986, *Acevedo, F. 253* (MEXU, MO); Tepehuanes, Mesa Alta de Canales, 6 Sep 1989, *Bravo-Bolaños, O. 235* (MEXU); Santiago Papasquiaro, km 12.5 carretera Santiago Papasquiaro-Altares, 29 Jul 1990, *Bravo-Bolaños, O. 1019* (MEXU); El Salto, 8 km E, 9 Jul 1961, *Detling, L.E. 8442* (US); forestry farm near Cd. Durango, 17 Nov 1959, *Gentry, H.S. & Arguelles, J. 18218* (US); Two miles east of El Espinazo, 20.5 miles east of El Palmito, Sierra Madre Occidental, between Mazatlán and Durango, 8 Jun 1962, *Hutchison, P.C. 2515* (F, K, MEXU, MO, US); 19 km SW of Buenos Aires, 25 Jun 1964, *Mick, G. & Roe, K.E. 147* (MEXU); 78.8 miles E of Villa Union, El Espinazo del Diablo, 3 Sep 1967, *Oliver, R.L. et al. 790 a* (MO); La Bajada, Tamazula, 1921, *Ortega, J.G. 4259* (US); San Ramón, 21 Apr 1906, *Palmer, E. 167 [a]* (F, NY, US); Puente de los Minmres 43 Km. al NW de Durango. Carr. Durango-Mazaltan, Mpio. Durango. Veg. Bosque de pino -encino. En la orilla de la carretera. Suelo negro forestal, 2 Jul 1982, *Tenorio L, P. & Romero de T, C. 788* (MEXU, MO); 16 Km. al N de Herreras. Mpio. de Santiago Papasquiaro. Veg. Matorral espinoso. Suelo amarillo derivado de conglomerados, 19 Jul 1982, *Tenorio L, P. & Romero de T, C. 1096* (MEXU, MO); 18 km, al E de La Posta de Jihuites. Mpio. Santa Maria del Oro. encinar. Suelo amarillo pedregoso, 12 Oct 1982, *Tenorio L, P. & Romero de T, C. 2075* (MO); Mezquital, 33 km al SW de Mesquital, 6 Mar 1985, *Tenorio L, P. & Romero de T, C. 8097* (F, MEXU); 52.7 km al W del Tarahuamar camino Tepehuanes-Tabahueto, 28 Aug 1983, *Torres C, R. et al. 3493* (MEXU); 16 km al W de Las Cruces, camino Tepehuanes-Tabahueto, 28 Aug 1983, *Torres C, R. et al. 3527* (MEXU); Tabahueto (el catorce) 196 Km. al W de Tepehuanes. Selva alta caducifolia alterada, 31 Aug 1983, *Torres C, R. et al. 3548* (MEXU, MO); Tepehuanes, Las Cruces, 52.7 Km. Al W del Tarahumar, Cam, 28 Aug 1983, *Torres R, R. et al. 3493* (MO); Tepehuanes, 16 Km. al W de Las Cruces, camino Tepehuanes-Tabahueto.Veg. Econtonia de bosque pino-encino con matorral de Suelo pedregoso, 28 Aug 1983, *Torres R, R. et al. 3527* (MO). **Guanajuato**: San José Iturbide, El Zorillo, 4 Jul 2003, *Monroy, V.E. et al. 18* (MEXU); La Loma, cerca de Tócuaro, 4 km al S de Acámbaro, 11 Jul 1986, *Santos Martínez, J. 1510* (MEXU). **Guerrero**: Tlacoapa, Filo de Caballos, 5.24 km S (Mun. General Heliodoro Castillo), 7 Oct 1999, *Calónico-Soto, J. 11103* (MEXU); Coyuca de Catalán, Cerro del Chivo, 25 km al N de Las Palancas, 8 Feb 1974, *González Medrano, F. et al. 6643* (MEXU); Mina, Zihuaqueo-Filo Mayor-1700. Mina Gro, 20 Aug 1936, *Hinton, G.B. 9288* (K, NY, US); Juan Escudero, Tierra Colorada, 3 Mar 1968, *Kruse, H. 1656* (MEXU); Taxco de Alarcón, Agua Escondida, 10 m al O, rumbo a Puerto Obscuro, 20 Aug 1997, *Martínez Gordillo, M. 1657* (MEXU); 4 km al NE del Jilguero, camino Filo de Caballo-Puerto del Gallo, 4 Jun 1983, *Soto N, J.C. et al. 5179* (MO, NY); Chichihualco, a 5 km al SO de Filo de Caballo, 21 Apr 1985, *Soto N, J.C. & Aureoles Conejo, S. 8316* (MEXU); A 26 Km al

NE de Vallecito de Zaragoza, municipio de Azueta. Veg. Selva mediana subcaducifolia, 24 Jul 1985, *Soto N, J.C. et al. 9708* (MEXU, MO); Azueta, El Bálsamo, 32 km al NE de Vallecito de Zaragoza (Mun. Zihuatanejo -alt. name), 10 Dec 1985, *Soto N, J.C. et al. 11538* (MEXU); Loc. 13 Km, al NW de Pinal de Amoles. Carretera Pinal de Amoles-Jalpan. Veg. selva media alterada. Suelo negro forestal, 22 Oct 1982, *Tenorio L, P. & Romero de T, C. 2322* (MEXU, MO); 20.1 km al E de Petlaltina o 68.1 km al E d Chilapa, camino a Tlapa, 14 Nov 1982, *Torres C, R. et al. 1893* (MEXU); Ixcateopan de Cuauhtémoc, 4.5 km al N, camino Taxco-Ixcateopan, 23 Sep 1997, *Valencia Avalos, S. 1629* (MEXU). **Hidalgo**: Tepeapulco, NE Cerro Jihuingo, ca. 12.5 km road from Tepepulco to Coatepec-Tulancingo, 30 May 2001, *Bye, R.A. et al. 28196* (MEXU); Ajacuba, Planicie de Tulancalco, zona de cultivos del ejido Emiliano Zapata, al S del poblado, 6 Jun 1989, *Díaz Vilchis, I. 544* (NY); Ajacuba, Jagüey 'El Palo Seco', plancie de Tulancalco, aprox. 4 km sobre el camino de terraceria que se inicia al S de Emiliano Zapata, ejido San Nicolas Tecomatlán, 15 Jul 1989, *Díaz Vilchis, I. 559* (MEXU, NY); Ajacuba, planicie de Tulancalco (zona de cultivos) ejido Emiliano Zapata, aprox. 3 km al S del poblado E. Zapata, 21 Jul 1990, *Díaz Vilchis, I. 905* (MEXU); Ajacuba, planicie de Tulancalco (zona de cultivos) ejido Emiliano Zapata, aprox. 6 km al S del poblado E. Zapata, 17 Dec 1989, *Díaz Vilchis, I. et al. 1377* (MEXU, NY); Near Pass. Hillside along turnpike from Mexico City to Queretaro, 12 Aug 1972, *Dziekanowski, C.T. et al. 1861* (MEXU, MO); tutotepec; en carcavas junto con zarzamora, 6 May 1972, *Gimate L, J. 616* (CORD, F, MO); Acatlán, 3 kms al norte de Catlán, 4 Nov 1980, *Hernández Magaña, R. 5281* (MEXU); 3 ó 5 Km. al sur de Apam, mpio. de Apam. Vegetación semiárida de cactaceae, copressaceae etc. Suelo calizo, somero, pedregoso, 23 Mar 1981, *Hernández Magaña, R. & Rodríguez B, D. 5559* (MEXU, MO); Tepeji del Río, 6 kms. al sur de Habitat: vegeación baja de matorral de leguminosas y pastizales. Suelo casi blanco, arcilloso, somero, 17 Nov 1981, *Hernández Magaña, R. 6593* (MEXU, MO); 2 km al NW de Tepeapulco, 10 Oct 1971, *Jiménez S, F. 207* (MEXU, MO); Tula de Allende, Cañón de las Adjuntas, Santa Maria Macuá, 15 Jun 2003, *Romero, L. & Rivera H, J. 81* (MEXU); near Tula, 3 Jul 1905, *Rose, J.N. et al. 8337* (US); Tula de Allende, Tula, Jul 1905, *Rose, J.N. et al. 8339* (NY); Pachuca, Jul 1905, *Rose, J.N. et al. 8796* (NY, US); Ixmilquipan, 1905, *Rose, J.N. et al. 9078* (US); Zempoala, Hab. pastizal a la orilla de un camino, 18 Aug 1975, *Ventura A, A. 126* (CORD, F, MEXU, MO); Acaxochitlán, San Francisco, 1 Aug 1984, *Villa Kamel, A. 53* (MEXU); Cerro de Santa Monica, N. of Santa Monica, 40 km. NW of APAM, on Pachuca highway. NW slopes, 11 Jul 1966, *West, R.C. P-25* (NY). **Jalisco**: Bolaños, 16 km al NW de Bolaños, camino a Tuxpan de Bolaños, Arroyo El Nalgaso, 6 Jul 1996, *Calónico-Soto, J. & Flores-Franco, G. 2527* (MEXU); Reserva BiÃ³sfera Sierra de ManantlÃ¡n. Whittaker 1000 mÂ² quadrat. Upper W edge of Cerro Grande, 18-50 km NW of Colima, 5.1 km by rd. (4 km airline distance) NNW of El Terrero, 37 km due NW of Colima. Sampling site 2, 9 Jun 1991, *Cochrane, T.S. 12568* (NY); Bolaños, Arroyo del Stafiate, 3 km al SW de la Berenjenas, 8 km al W del Crucero Tuxpan de Bolaños-Los Amoles 'Vanderitas', 3 Jul 1996, *Flores-Franco, G. & Calónico-Soto, J. 4643* (MEXU); Cerro Viejo, south face, above Zapotitan Hidalgo, a village about 1 mi. N of highway Mex. 15. About 25 mi. due south, or 45 road miles from Guadalajara. Foot of mt, 27 Jun 1956, *Gregory, D.P. & Eiten, G. 220* (MEXU, MO, NY); Ayutla, Paraje Las Iglesias, campamento de Silvicultura de Occidente, 13 Aug 1979, *Guízar N, E. 514* (MEXU); Ayutla, Parajae Las Iglesias, campamento de Silvicultura de Occidente SA, 13 Aug 1979, *Guízar N, E. 517* (MEXU); Ixtlahuacán del Río, 10 kms al N de Ixtlahuacán del Río, 8 Aug 1984, *Hernández Magaña, R. et al. 9489* (MEXU); Zapopan, Bosques de la Primavera, 9 Aug 1984, *Hernández Magaña, R. et al. 9497* (MEXU); Cerro de Talcozagua (on Sierra de Tapalpa) 2-3 km E of Tapalpa, 5 Aug 1960, *Iltis, H.H. et al. 721* (MEXU); Zapotlán el Grande, Nevado de Colima, 20 km de Fresnito por la brecha a la torre de televisión y La Joya (Mun. Cd. Guzmán on label), 12 Nov 1989, *Koch, S.D. et al. 89-113* (F, US); Zapotitlán, Parque del Volcán de Colima, 11 km sobre la desviación al Navado de Colima, 14 Jun 1988, *Martínez B, A. et al. 16* (MEXU); Tolimán, 1 km al E de El Terrero, rumbo a Tapeixte, 18 Apr 1991, *Niz, D. de, et al. 201* (MEXU); Venustiana Carranza, a 300 m de la brecha El Floripondio a la estación de microondas Las Víboras, 16 Sep 1991, *Ramírez, R. & Vargas, O. s.n.* (MEXU); Tapalpa, camino que conduce de Juanacatlán a Tepec, 26 Aug 1987, *Rodríguez C, A. & Suárez J, J.J. 969* (MEXU); Venustiana Carranza, Rancho El Izote, Carretera Cd. Guzmán-Autlán, 3 Dec 1991, *Rodríguez C, A. & Dean, E. 2128* (MEXU). **México**: San Juan Totolapan, municipio de Tepetlaoxtoc. terreno plano; orilla de camino, 1982, *Ascencio V, M.A. 111* (MO); Tezcoco, Area Experimental Forestal Mario Avila Hernández, 2 km al oriente de San Luis Huexotla, 13 Sep 1985, *Asteinza, G. &*

Rey, J. s.n. (MEXU); Mt. Popo-Ixta [Popocatépetl to Iztaccihuatl] road to peak, 3 Apr 1980, *Balogh, P. 999* (US); Ocuilón, Parque Nacional Lagunas de Zempoala, carretera Zempoala-Huitzilac km 4-11, 28 Jul 1987, *Castañeda Robles, J. 228* (MEXU); Ocuilán, San Juan Atzingo, Parque Nacional Laguans de Zempoala, 15 Oct 1987, *Castañeda Robles, J. 711* (MEXU); Ocuilón, Parque Nacional Lagunas de Zempoala, San Juan Atzingo, 15 Oct 1987, *Castañeda Robles, J. 718* (MEXU); Donato Guerra, camino a Llano redondo, ejido de San Juan Xoconuxco, 26 Dec 2005, *Cornejo Tenorio, G. et al. 1706* (MEXU); Along Hwy 57 ca. 70 miles NW of Mexico city, ca. 4miles S of Encinillas, 25 Aug 1977, *Croat, T.B. 44051* (BM, CORD, MO); Polotitlán, 0-2 miles W of Encinillas, along Highway 57, ca. 70 miles NW of Mexico City, 26 Aug 1977, *Croat, T.B. 44159* (MO); Temascaltepec, ca. 2 mi. from hwy. to Temascaltepec along road toward Nevado de Toluca, 17 Aug 1972, *Dziekanowski, C.T. et al. 1952* (MO); Mpio Tezcoco, 1 Km. al E de Huexolta, por camino a la mina de arena. Veg. de matorral scundario con varios cultivos, lugares mut perturbados. Suelo arcilloso, 31 Jul 1982, *García P, J. 1643* (MO); Teotihuacán, Teotihuacan ruins, 31 Jul 1965, *Hawkes, J.G. 2504* (K); Volcán Toluca, Cocastepec, *Heller, C. 395* (W); Temascaltepec, El Crucero, 19 Jul 1932, *Hinton, G.B. 1100* (BM, K); Atlautla de Victoria, Tepecoculco, borde de camino, 13 Sep 1987, *Jiménez Flores, J. 577* (MEXU); San Marcos Coajomulco, 8 Jun 2010, *la Cruz Bolaños, A. de*, *Adec-12* (MEXU); Cerro de San Gregorio, Valle de Mexico, 1963, *Matuda, E. 21319* (MEXU); Polotitlán, 14 Sep 1952, *Matuda, E. 26529* (MEXU); Chalco, Cerro Xico, 15 Jun 1981, *Popper, V.S. 122* (MEXU); El Oro, At border between Mexico and Michoacan states. On route 15 wetween Toluca and Morelia, 14 Aug 1984, *Randolph, D. 316* (MO); NW slopes of Nevado de Toluca, 10 km (by road) SW of jct of roads to Sultepec and Temascaltepec on Hwy 130 to Temascaltepec or 27 km (by road) SW of Toluca, 16 Jul 1965, *Roe, K.E. 290* (NY); W slopes of Nevado de Toluca, 35 km (road) SW of Toluca on highway 130, 29 Aug 1965, *Roe, K.E. 1494* (NY); Hacienda La Encarnación, 7 Jul 1905, *Rose, J.N. et al. 8435* (US); Amecameca, Cerro Venacho, ladera W, 7 Jan 1979, *Rzedowski, J. 36035* (MEXU, MO); Cerro de la Cruz, 6 km al N de Tepotzotlán, 12 Oct 1980, *Rzedowski, J. 37059* (MEXU); Cerro de Jaltepec, municipio de Ajapusco. Hab. Cañada, 1975, *Ventura A, A. 525* (MEXU, MO); Chalco, Tlaxchayote, 13 Jan 1976, *Ventura A, A. 831* (MO); Atenco, Santa Isabel Ixtapán, 24 Apr 1983, *Ventura V, E. 766* (CORD, F); Valle de Bravo, carretera de Toluca a Valle de Bravo por Santa María del Monte, cerca de Santa María Pipioltepec, 4 Aug 1995, *Vibrans, H. 5363* (MEXU); Amecameca, 1 km al NE de San Antonio, 28 Sep 1980, *Zerón B, F. ME-34* (MEXU). **Michoacán**: W of Loma Zapote, vicinity of Morelia, 28 Aug 1909, *Arsène, G. s.n.* (US); Morelia, Punguato, 16 Jul 1909, *Arsène, G. 2892* (MEXU, MO, US); Vicinity of Morella, 20 Jun 1912, *Arsène, G. 8302* (BM, CM, F, K, MO, NY, US); Tzintzuntlan [Tzintuntzan] Ruins, near Patzcuaro, 5 Apr 1980, *Balogh, P. 1025* (US); Angangueo, Sierra Chincua, Reserva de la Biósfera Mariposa Monarca, alrededores del vivero forestal, 2 Jun 2000, *Cornejo Tenorio, G. et al. 3* (MEXU); Contepec, Llano Las Golondrinas, Cerro Altamirano, 21 Sep 2001, *Cornejo Tenorio, G. & Ibarra Manríquez, G. 357* (MEXU, NY); Contepec, Cerro Altamirano, camino al llano, 22 Aug 2004, *Cornejo Tenorio, G. et al. 726* (MEXU); Angangueo, Sierra Chincua, 25 Jun 2005, *Cornejo Tenorio, G. et al. 1105* (MEXU); Contepec, Cerro Altamirano, 2 km de Contepec, 15 Sep 2005, *Cornejo Tenorio, G. & Salinas Melgoza, M.A. 1588* (MEXU); Morelia, Cerro del Aguila, subiendo por Tacicuaro, 30 Aug 2007, *Cornejo Tenorio, G. et al. 2195* (MEXU); Zinapécuaro, Low valley, Zinapécauro, 2 May 1849, *Gregg, J. 755* (MO); Zitácuaro, Zitacuaro -San Felipe, 6 Jan 1938, *Hinton, G.B. 11898* (BM, F, GH, K, MEXU, NY, US); Uruapan, Tancitaro, 11 Jun 1940, *Hinton, G.B. 15638* (F, NY, US, W); Above Rancho Las Cerquillas at km. 11 on Mex. Hwy. 120, half-way between Uruetaro and Alvaro Obregón, ca. 22 km. NE of Morelia, 3 Dec 1971, *Iltis, H.H. 244* (NY); along highway #15, 3 km ESE of Comaja (16 km ESE of Zacapu), 26 Jul 1960, *Iltis, H.H. et al. 446* (MEXU); rancho Las Cascadas, arriba de Tacámbaro, vía de Patzcuaro km 35, 3 Mar 1978, *Kishler, J. 213* (MEXU); arriba de Tacámbaro, 4 km del Rancho La Cascada, 26 May 1979, *Kishler, J. 632* (MEXU); Coeneo, 3 km al E de Comanja, 17 Aug 1983, *Labat, J.N. 415* (P); Los Reyes, Ojo de Agua, Atapán, 1 Jul 1984, *Labat, J.N. 894* (MEXU); Angamacutiro, Cerro Chongo, 5 Sep 1984, *Labat, J.N. 1138* (P); Tancítaro, Michoacán de Ocampo. Tancítaro. 6 mi. north of Tancítaro, 25 Jul 1940, *Leavenworth, W.C. 352* (NY); Tancítaro, Pedregal lava flow 2 mi. S of Tancitaro, 16 Aug 1940, *Leavenworth, W.C. 592* (F); Tancítaro, Tancitaro region, 5 Jul 1941, *Leavenworth, W.C. & Hoogstraal, H. 4020* (F, NY); Zitácuaro, San Miguel Chichimequillas, La Mesa, 12 Jul 2007, *Ledesma-Corral, C. & Torres-Diaz, A. 295* (MEXU); Zitácuaro, San Miguel Chichimequillas, Volcán El Molcajete, 13 Jul 2007, *Ledesma-Corral, C. &*

Torres-Diaz, A. 363 (MEXU); Uruapan, Huerta de Aguacate, C.E.E. Barranca de Cupatitzio, 4 Oct 1978, *Madrigal Sánchez, X. 3039* (MEXU); Tingambato, San Francisco Pichataro, Lago de Patzcuaro, 10 Nov 1978, *Mapes, C. 41 -C* (MEXU); Contepec, Cerro Altamirano, Santa Maria de los Angeles, camino a El Llano, 25 Sep 2004, *Martínez Cruz, J. 1123* (MEXU); camino entre Lamatácuaro y San Benito, 23 May 1981, *Motte, M.E. 66* (MEXU); carretera entre San Isidro y Latamban, 29 Oct 1981, *Motte, M.E. 169 a* (MEXU); Cerro del Aguila, subiendo por San Nicolás Obispo, municipio de Morelia, 12 Jul 1977, *Sánchez G, E. et al. 104* (K); Morelia, Cerro del Aguila, subiendo por San Nicolás Obispo, 12 Jul 2007, *Sánchez Garcia, E. et al. 104* (MEXU); southeast shore of Lake Patzcuaro, 15 Jul 1941, *Schery, R.W. 137* (MO); A 5 km al E de San José de Los Alzati, por la desv. a Angangueo. bosque de pino-encino, 9 Sep 1979, *Soto N, J.C. & Silva R, G. 1476* (MEXU, MO); cerca de Laguna Verde, 7 Jul 1985, *Soto N, J.C. et al. 9072* (MEXU); Aguililla, El Manguito, 10 km al NO de Aguililla, camino a Dos Aguas, 12 Jul 1985, *Soto N, J.C. et al. 9303* (MEXU); Aguililla, La Alberca, 22 km al NO de Aguililla, 13 Jul 1985, *Soto N, J.C. et al. 9368* (MEXU); Ocampo, San Juan, 3 km al SO de Ocampo, 8 Sep 1985, *Soto N, J.C. & Silva Ramírez, G. 10346* (MEXU); Uruapan, a 2.5 km al SO de Angahuan, por el camino al volcán Paricutín, 14 Aug 1990, *Soto N, J.C. et al. 13806* (MEXU); Morelia, Found along hwy. Mex.15, ca. 20 mi W, *Watkins, J. et al. 633* (MO); Pátzcuaro, Los Nogales, 28 Apr 1996, *Zamudio R, S. 9775* (MEXU). **Morelos**: Cuautla, W de Cuautlixco (NW de Cuautla), 20 Mar 1987, *Bye, R.A. et al. 15312* (MEXU); Huitzilac, Parque Nacional Lagunas de Zempoala, Laguna de Compila, 29 Oct 1986, *Cardoso, V.L. 1467* (MEXU); auropista México-Cuernavaca, km 53, 7 Sep 1958, *Espinosa, J. 75* (MEXU); environs de Tres Marias route de Mexico a Cuernavaca, 1 Jul 1958, *Humbert, H. & Miranda, F. 31295* (US); Zempoala, Dec 1932, *Lyonnet, E. 1237* (US); Lagunas de Zempoala, 17 Sep 1938, *Lyonnet, E. 2474* (US); carretera nueva Mex.-Cuernavaca km 56, en pedregal, 4 Feb 1964, *Matuda, E. 37364* (MEXU); Parque Nacional Lagunas de Zempoalas, 26 Jul 1949, *Quiram, H.G. 11* (MEXU); Parque Nacional Lagunas de Zempoala, 28 Jul 1949, *Smith, W.T. 13* (MEXU); Parque Nacional Lagunas de Zempoala, 28 Jul 1949, *Smith, W.T. 26* (MEXU); Jojutla, carretera Jojutla-Alta Vista, km 7, Río Los Muros, 8 Apr 1983, *Zambrano C, O. 992* (MEXU). **Nayarit**: Tepic, alrdedores del Venustiano Carranza, aprox. 11 km al O de Tepic, carretear 66, 9 Dec 1989, *Aguilar, S. 498* (MEXU); Tepic, colorad de la Mora, Arroyo de los Negros, 500 al NW del pobaldo, 4 Mar 1992, *Bravo-Bolaños, O. 2147* (MEXU); San Blas, Along Route 28; 1.8 miles WSW [west] of Jalcocotán [Jalcacatán], between Tepic and Santa Cruz, 8 Jan 1979, *Croat, T.B. 45319* (MO); San Blas, Along Route 28; 1.8 miles WSW [west] of Jalcocotán [Jalcacatán], between Tepic and Santa Cruz, 8 Jan 1979, *Croat, T.B. 45330* (MO); Xalisco, 4 km al NW del entronque a Confradia de Chocolon-Xalisco, 17 May 1994, *Flores-Franco, G. et al. 3482* (MEXU); Xalisco, 15 km al SW de Xalisco en el camino de terraceria a el Carrizal, 17 Oct 1994, *Flores-Franco, G. et al. 4128* (MEXU); Tepic, 4 km al W del pobaldo La Yerba, 18 Oct 1994, *Flores-Franco, G. et al. 4169* (MEXU); Xalisco, 17 km al SW de Xalisco, en el camino de terraceria al Carrizal, 17 Oct 1994, *Flores-Franco, G. et al. 4179* (MEXU); Tepic, 4 km al W del poblado La Yerba (en canñada). bosque de Quercus, 1994, *Flores-Franco, G. et al. 4196* (MO); Tepic, 4 km al W del poblado La Yerba, 18 Oct 1994, *Flores-Franco, G. et al. 4196* (MEXU); Tepic, 6 km al W del pobaldo La Yerba, 18 Oct 1994, *Flores-Franco, G. et al. 4310* (MEXU, MO); San Blas, Isla María Madre, Tres Marias Island group, 15 May 1897, *Maltby, F.S. 118* (US); San Blas, Isla María Madre, Tres Marias Islands, 3 May 1897, *Nelson, E.W. 4200* (F, US); Jalisco, In deep ravine about 3 miles north of El Refilon on road to Tepic, 13 Jul 1970, *Norris, D.H. & Taranto, D.J. 12637* (MEXU, MO); Tepic, Tepic, 5 Jan 1892, *Palmer, E. 2067* (US); Tepic, Tepic, 10 Sep 1972, *Poff, J.M. & Hanson, W.J. 103* (F); Mun. Nayar a 28 Km del entronque a San Juan Peyotán, sobre la carr. Jesús María-Huejuquilla. bosque de Pinus-Quercus, 3 Sep 1991, *Ramírez R, R. & Flores-Franco, G. 926* (MO); Nayar, a 28 km de entronque a San Juan Peyotán, sobre la carretera Jesus Maria-Huejuquilla, 3 Sep 1991, *Ramírez, R. & Flores-Franco, G. 926* (MEXU); Tepic, between Santa Gertrudis and Santa Teresa, 8 Aug 1897, *Rose, J.N. 3381* (US); Sierra Madre, near Santa Teresa, Territorio de Tepec, 10 Aug 1897, *Rose, J.N. 3390* (K, US); Nayar, Cerro Cangrejo, cañada al NE del poblado Villa de Guadalupe. Ecotonia de encinar y selva baja caducifolia, 18 Sep 1989, *Tenorio L, P. & Flores-Franco, G. 16155* (MO). **Oaxaca**: Tehuantepec, Aug, *Andrieux, G. 189 [b]* (K); along hwy 175 between Pochutla and Oaxaca, 0.5km by road n of San José del Pacifico, 34km, 24 Jun 1986, *Diggs, G.M. 3952* (MO, NY); sin. loc, 1840, *Galeotti, H.G. 1229 [1]* (W); Zimatlán, Paraje El Campanario, comunidad de San Pedro El Alto, 18 Sep 1998, *Guízar N, E. et al. 4260* (MEXU); Miahuatlan, Mpio.

San Juan Mixtepec. Primary school, 6 Aug 1996, *Hunn, E. OAX-50* (MO); San Juan Mixtepec, Dist. Miahuatlán, 13 Jul 1997, *Hunn, E. OAX-1457* (MEXU); Santiago Textitlán, Paraje El Berro, Dist. Sola de Vega, 27 Jun 2006, *Jacob Salinas, M.E. 75* (MEXU); Ixtlán de Juárez, Loc. 6 Km NE de la Gumbrea Mpio. Teococuilco Dto. Ixtlán de Juárez. Region Sierra Norte, 24 Jun 1985, *López, ?, 286* (MO); Ixtlan, Santiago Comaltepec; S. Comaltepec bosque de coniferas y de encino, 1 Sep 1989, *López, L. 377* (MO); Santiago Comaltepec, Distrito de Ixtlan: S. Comaltepec, 4 Aug 1988, *López, L.M. 166* (NY); Santiago Comaltepec, Distrito de Ixtlan: S. Comaltepec, 1 Sep 1989, *López, L.M. 377* (NY); Salul Ja', 5 km al este del al cabecera de Amatenango, 9 Mar 1988, *López-Pérez, J. 326* (MEXU); Ixtlan, 4km east of Ixtlan de Juarez on the road to Capulalpan; in the clay soil of Rancho Teja; full sun, 4 Sep 1983, *Martin, G.J. 152* (MO); Ixtlan, Distrito de Ixtlan: Municipio de Yolox: on streambank along the road between Yolox and Comaltepec. In moist rocky clayloam. Ful sun, 21 Mar 1981, *Martin, G.J. 448* (MO); vicinity of Cerro San Felipe, 1894, *Nelson, E.W. 1130* (US); Trinidad Zimatlán, 21 May 1978, *Solano, C. 171* (MO); Putla, Cerro de Yutunda, a 6 km en LR (NW) de Santa Cruz Itundujia, 19 Jul 2007, *Velasco G, K. et al. 2070* (MO); Santiago Textitlán, Paraje la Yerba Buena, rumbo a Lachixao, Dist. Sola de Vega, 31 Jul 2006, *Zarate-Marcos, A. 183* (MEXU). **Puebla**: Xochiapulco, Rosa Chica, sitio llamado La Cruz, tomando la carretera a Xochiapulco, a 15 minutos del pueblo, 29 Apr 2015, *Amith, J.D. et al. 30687* (BM); Puebla, 13 Jun 1907, *Arsène, G. 1483* (US); barranca d l'Alseseca, Hacienda Guadelupe, vicinity of Puebla, 13 Jun 1907, *Arsène, G. 1484* (US); Cholula, Campo Zafiro, Santiago Momoxpa, 30 Apr 1988, *Castillo, R. & Cálix, H. 88-32* (MEXU); Cholula, S.A. Calpán, 13 May 2005, *Guarneros, Y. & Carvajal, D. 412* (MEXU); Tlatlauquitepec, Predio Xicalhutamaría, 2 km al NW de Huexotitlán, 27 May 2001, *Guízar Nolazco, E. & Miranda M, A.G. 5587* (MEXU); Rancho-Pasadas/Puebla, 20 Jun 1909, *Nicolas, F. 994* (K); Chalchicomula, above Serdán [Ciudad Serdán], Cabecero, 25 Sep 1944, *Sharp, A.J. 441005* (US); Barranca al SW de San Simón. Mpio. Caltepec. Veg. Ecotonia Matorral espinoso Matorral calcícola mixto con: Vernonia uniflora: Brahea dulcis: B. nitida: juniperus flaccida. Suelo amarillo arcilloso, 9 Dec 1983, *Tenorio L, P. & Romero de T, C. 5051* (MO); Cholula, camino al Cerro Testón por el lado oriente de San Pedro, 3 Aug 1987, *Tlapa, M. & Ubierna, G. 1* (MEXU); Cholula, camino por el lado sur de Santiago Xalitzintla, 12 Aug 1987, *Tlapa, M. & Ubierna, G. 105* (MEXU); Cholula, Tlamililelpa, por el lado Peniente de San Pedro Yancuictlalpan, 26 Sep 1987, *Tlapa, M. & Ubierna, G. 618* (MEXU). **Querétaro**: carretera Querétero-Amealco, 5 kms psando entronque para Huimilpan, 26 May 1985, *Argüelles, E. 2295* (MEXU); 8km al ne de Amealco; bosque de Quercus, cañada, 1991, *Carranza, E. 3281* (MEXU, MO); Landa, Enbocadero, 2.5 km al S de el Puerto de El Sabino, 26 Aug 1988, *Rubio, H. 29* (MEXU). **San Luis Potosí**: 24 miles East of Ojuelos. Plants in protection of cactus, 11 Sep 1978, *D'Arcy, W.G. 11882* (MO). **Sinaloa**: Las Mesas, Sierra Surotato, 15 Sep 1941, *Gentry, H.S. 6611* (MO, NY); Sierra de Surutato, Sierra Surotato, Canyon Mansana, 12 Dec 1959, *Gentry, H.S. & Arguelles, J. 18393* (US); Culiacán, a 51 km al N de Culiacán, camino a El Barco, 30 Jul 1983, *Martínez S, E.M. et al. 4131* (MEXU); San Ignacio, Cerro de las Cruces, 23 Nov 1917, *Narváez Montes, M. & Salazar, A.E. 102* (US); San Blas, along Río Fuerte, 24 Mar 1910, *Rose, J.N. et al. 13383* (NY, US); El Rosario, vicinity of [El] Rosario, 14 Apr 1910, *Rose, J.N. et al. 14596* (US); Agua Caliente de Zevada, en la huerta de Lorenzo Perez por el arroyo, 9 Apr 1975, *Shapiro, G. 49* (MEXU). **Sonora**: Alamos, Rio Mayo watershed, San Bernardo and vicinity, Curahui, 25 Aug 1959, *Argüelles, J. 152* (US); Yécora, Cañón Tepoca, km 177 a los lados de la carretera Federal 16, 7 Sep 1996, *Búrquez, A. et al. 96-961* (MEXU); W of Villa de Seria, on banks of Río Sonora, 11 Nov 1939, *Drouet, F.E. & Richards, D. 3559* (F); on west side of Río Magdalena, 29 Nov 1939, *Drouet, F.E. & Richards, D. 3800* (F); 4 mi W of Alamos on Alamos-Navajoa road, 1 Mar 1973, *Fish, J. 10* (MEXU); Sierra Charuco, Río Mayo, 10 Sep 1935, *Gentry, H.S. 1712* (MEXU); Alamos, Agua Marina, Sierra de los Alamos, 15 Sep 1959, *Gentry, H.S. 17816* (US); Fronteras, 1.09 km (air) west northwest of El Aserradero along trail to Puerto Apache, 24.0 km (by air) WSW of Fronteras, Sierra Buenos Aires, Ajos-Bavispe Reserva Forestal Nacional y Refugio de Fauna Silvestre, 16 Aug 2016, *Hale, S. & Carnahan, S. 5348* (ARIZ); Fronteras, 0.75 km (air) west northwest of El Aserradero along Arroyo San Vicente, descending northeast slope of range, 24.0 km (by air) WSW of Fronteras, Sierra Buenos Aires, Ajos-Bavispe Reserva Forestal Nacional y Refugio de Fauna Silvestre, 16 Aug 2016, *Hale, S. & Carnahan, S. 5353* (ARIZ); Nacozari de García, Rancho la Zulema, Sierra Juriquipa, 13.2 mi east (by road) HWY 17, 15.9 km (by air) SE of Nacozari de García, 13 Aug 2017, *Hale, S. et al. 5506* (ARIZ); Puenta La Pila, drainage above El Palmar, ca. 20 km E of Onavas, 3 Apr 1991, *Joyal,*

E. 1558 (MEXU); Hermosillo, 10 Jun 1897, *Maltby, F.S. 223* (US); Hermosillo, 10 Jun 1897, *Maltby, F.S. 226* (US); Santa Cruz, 22 Oct 1893, *Mearns, E.A. 2630* (US); Cerro Saguarivo, E of San Bernardo, Pacific slope, 7 Aug 1938, *Pennell, F.W. 19649* (US); Onavas, Rancho La Mula, 5 km west of Agua Amarilla, 28.2 km southeast of Río Yaqui on MEX 16 (Km 195 east of Hermosillo), 9 Jan 2001, *Reina G, A.L. & Van Devender, T.R. 2001-51* (MO); Yécora, Tepoca, 21 Mar 1998, *Reina G, A.L. 98-400* (NY); Yécora, Mesa del Campanero, W of Yecora, 26 May 1996, *Reina, A.L. et al. 96-258* (MEXU); vicinity of Hermosillo, 8 Mar 1910, *Rose, J.N. et al. 12541* (US); Tórim, Rio Yaqui, Torim, 17 Mar 1914, *Studhalter, R.A. 1412* (US); Tórim, Rio Yaqui, Torim, 17 Mar 1914, *Studhalter, R.A. 1413* (US); Fronteras, Jun 1877, *Thurber, G. 364* (GH); Rancho La Junta, 1 Km upstream from Mesa Colorado, 3.0 Km (by air) north-northeast of Burapaco: junction of arroyo Guajaray and Rio Mayo, 17 Mar 1993, *Van Devender, T.R. et al. 93-379* (MO); El Rancheria crossing of Rio Cuchujaqui, ca. 22.5 Km)13.5 miles) south of Almos on road to El Chinal, 10 Oct 1992, *Van Devender, T.R. et al. 92-1167 A* (MO); Cañón de Huépari, north or Aribabi, 2 Sep 1939, *White, S.S. 2634* (MEXU); canyon 35 miles NE of Cajeme on road to Tesopaco, 3 Mar 1933, *Wiggins, I.L. 6411* (US); along Rí de los Alisos, 31 miles S of Nogales, 6 Sep 1934, *Wiggins, I.L. 7020* (US). **Tamaulipas**: San Carlos, cerro del diente e la sierra de san carlos; bosque de quercus; suelo somero calcareo, 20 Aug 1985, *Jiménez, J. 245* (MEXU, MO); mountains along route 70, ca. 8 miles S of Ciudad Victoria, 7 Apr 1961, *King, R.M. 452* (US); Tamaulipas. Mountains along route 70, ca. 8 miles south of Ciudad Victoria, 7 Apr 1961, *King, R.M. 4521* (F, NY); San Carlos, Cerro del Diente en La Sierra de San Carlos. Bosque de Quercus, 2 Jun 1986, *Martínez, M. 1136* (MO); Jaumave, Sierra near San Lucas, 1932, *Rozynski, H.W. von*, *515* (F). **Tlaxcala**: Ixtenco, a 6 km oeste del pobaldo de Ixtenco, cabanas de CREA, 1 Jul 1999, *Sánchez T, V. 325* (MEXU); Domingo Arenas Muñoz, Muñoz, ruina de una casa entre los caompos de cultivo (maíz) en los alrededores del pueblo; alrededor de la casa caída, 8 Sep 1983, *Williams, D.E. 158* (MEXU). **Veracruz**: Perote, Los Molinos, 2 Jun 1938, *Balls, E.K. B 4671* (BM, E, K, US); Orizaba, 1846, *Heller, C. 152* (W); Cordoba, Aug 1936, *Matuda, E. 1034* (MEXU); Huayacocotla, Las Blancas, 29 Sep 1994, *Pérez G, I. 994* (MEXU).

NICARAGUA. **Estelí**: Llano La Almaciguera, 4 km al S del puente del Río Estanzuela; bosque de pinos, 29 Sep 1980, *Guzmán, M. et al. 1262* (MO); Cerro Quiabú, al NO de Estelí, 24 Jul 1980, *Moreno, P.P. 1312* (MO); Cerro Quiabú; bosque enano, 14 Jan 1981, *Moreno, P.P. 6011* (MO); La Guayaba, 10 km de la Carretera Pan-americana carretera a San Nicolás, 26 May 1981, *Moreno, P.P. & Henrich, J. 8653* (MO); 5 km al N de San Nicolás, 23 Sep 1981, *Moreno, P.P. 11374* (MO); 8 km sobre la carretera a San Nicolas, San José de la Laguna; bosque de pino, 6 Nov 1981, *Moreno, P.P. 12656* (MO); Municipio de Esteli. Reserva Natural Tisey. Comunidad La Almaciguera, 16 Mar 2000, *Rueda, R.M. et al. 13190* (MO). **Granada**: Volcán Mombacho, en las útimas antenas, 23 Feb 1981, *Moreno, P.P. & López, A. 7130* (MO); Mombacho, 27 Jan 1970, *Narváez S, E. 3927* (BM, MEXU, MO); Volcán Mombacho, Plan de Flores to W rim, 14 Nov 1978, *Stevens, W.D. 10859* (MO). **León**: along new road from Hwy. 1 (at ca. Km 135.5 and ca. 10.6 km W of bridge at La Trinidad) to San Nicolás, ca. 9.5 km from Hwy. 1, 21 Sep 1980, *Stevens, W.D. & Montiel, O.M. 17956* (MO). **Managua**: Las Nubes, hacienda El Yemen, creciendo sobre un farallón, 23 Jul 1980, *Guzmán, M. et al. 470* (MO); Carretera entre Las Conchitas y Masachapa, Km 27-30, 8 Aug 1981, *Guzmán, M. & Castro, D. 1945* (MO); Municipio San Rafael del Sur, en los márgenes del Río Los Gutiérrez, 8 Aug 1981, *Guzmán, M. & Castro, D. 1978* (MO); Llanos de Pacaya along the road between Las Conchitas and San Rafael Del Sur, 1 km SW of Las Conchitas, 26 Aug 1983, *Miller, J.S. 1317* (MO); El Crucero, hacienda El Abandono, 20 Jun 1981, *Reyes V, L. 13* (MO); Along Hwy. 8 ca. 2.4 km SW of intersection with Hwy. 2, Km 28, 18 Sep 1977, *Stevens, W.D. 3969* (MEXU, MO); ca. 0.6 km from Hwy. 2 (Carretera Sur) on road along ridge of Sierra de Managua from Hwy. 2 to Hwy. 12 (Carretera vieja a León), 19 Oct 1977, *Stevens, W.D. 4740* (MO). **Masaya**: Miravalle, 4 km de Las Nubes, 11 Dec 1980, *Moreno, P.P. 5242* (MO).

UNITED STATES OF AMERICA. Sin. loc., 8 Jul 1900, *Howard, O. s.n.* (UT). **Arizona**: Yavapai County, 6 miles SW of Prescott along Hwy 89, Prescott National Forest, 5 Sep 1991, *Atwood, N.D. 17037* (MO); Santa Cruz County, 1 mile E of Santa Rita Lodge, Madera Canyon, 22 Aug 1968, *Averett, J.E. & Watson, T.J. 408* (MO); Greenlee County, About 12 miles W of Mule Creek, 29 Aug 1968, *Averett, J.E. & Watson, T.J. 435* (MO); Maricopa County, Desert Botanical Garden, Phoenix, 4

Mar 1951, *Blakley, E.R. 65* (DES); Santa Cruz County, Sycamore Canyon, 18 Aug 1950, *Blakley, E.R. 589* (DES); Cochise County, Cardwell's Cabin, Wood CAnyon; may be introduced, 14 Nov 1906, *Blumer, J.C. 1512* (ARIZ, E, F, K, L, MO, NY, RM, W); Cochise County, Paradise, Chiricahua Mountains, 17 Oct 1907, *Blumer, J.C. 1797* (ARIZ, F, K, L, MIN, MO, NY, U, UC); Cochise County, Paradise, Chiricahua Mountains, 21 Oct 1907, *Blumer, J.C. 2263* (F); Pima County, Spud Ranch, Rincon Mountains, 28 Sep 1909, *Blumer, J.C. 3426* (ARIZ, F, MO); Yavapai County, Montezuma Castle National Monument. Montezuma Castle, 20 Nov 1939, *Bowen, W.L. s.n.* (DES); Yavapai County, Montezuma Castle, Montezuma Castle Nat Mon, 20 Nov 1939, *Bowen, W.L. s.n.* (ARIZ); Pima County, Alamo Canyon, ca. 1 mi upcanyon from Alamo Canyon campground, Ajo Mountains, Organ Pipe Cactus Nat Mon, 3 Dec 1977, *Bowers, J.E. 968* (ARIZ); Graham County, The Santa Teresa Mountains. BLM Wilderness. Preacher Canyon. E of Dryden's ranch. S of Black Rock, 19 May 1998, *Buegge, J.J. 161* (NY); Graham County, The Santa Teresa Mountains. BLM Wilderness. Near Black Rock. Near jeep trail off road 292, 15 Dec 1998, *Buegge, J.J. 632* (NY); Santa Cruz County, Salero Ranch, Unit 5: upper Ash Canyon near FSR 143; 6mi WNW of Patagonia, 4 Nov 2014, *Carnahan, S. SC-892* (ARIZ); Santa Cruz County, Salero Ranch: Viceroy Mine Canyon, 5mi NW of Patagonia, 15 Jul 2015, *Carnahan, S. & Slaughter, S. 1224* (ARIZ); Gila County, Boyce Thompson Arboretum; along Queen Creek, *Charnofsky, S. s.n.* (ASU); Coconino County, Canyon of the Colorado, Havasupai Canyon, 7 Sep 1943, *Clover, E.U. 7177* (ARIZ); Yavapai County, Bottom of Sullivan's Canyon ca. 1.9 mi SE of Paulden, 18 Sep 2012, *Coburn, F.S. 1349 b* (ASU); Pima County, Alamo canyon, Organ Pipe Cactus Nat'l Mon. Pima County, 16 Apr 1952, *Cottam, W.P. 12893* (UT); Fort Whipple, Hassayampa Creek, south of Prescott, 10 Aug 1865, *Coues, E. & Palmer, E. 162* (MO); Superstition Mt. Pinal Co. Ca. 5 1/2 mi. E. Apache Junction, West side of mountain range, 29 Mar 1960, *Crosswhite, F.S. et al. 544* (K); Pinal County, Pinal Mountains, near Miami, 24 Sep 1935, *Cutak, L. & Christ, A. 8* (MO); Maricopa County, Tonto National Forest, T2N R9E sect. 9. Superstition Mountains Off state Hwy 88 (Apache Trail Road), south side of Canyon Lake. At the bottom of a north-facing slope, 2 curves away from the bridge, 18 Oct 2002, *Damrel, D.Z. et al. 1764 B* (DES); Pinal County, ca. 1 mile from route 80/89 junction on route 77, 14 Sep 1976, *Davis, T. 664* (BM, MO); Pima County, along dirt road to Rosemont, Ca. ? miles from route 83, 14 Sep 1976, *Davis, T. 667* (MO); Cochise County, Ft. Huachuca. camp Greenlee, picnic area at north end of Huachuca canyon. Overstory to 40 feet, 14 Sep 1976, *Davis, T. 671* (BM, MEXU, MO); Santa Cruz County, Patagonia, 12 Oct 1979, *Davis, T. 1001* (BM, MO); Maricopa County, Tonto National Forest, Seven Springs Recreational Area. Along Seven Springs Rd. at Seven springs Campground, 3 Aug 2001, *Doan, S. et al. 891* (DES); Maricopa County, Tonto National Forest, Seven Springs: Along Seven Springs Rd. ca. 0.1 mi S of FS road 2047, 14 Aug 2001, *Doan, S. 914* (DES); Maricopa County, Found 1 mile S. of Canyon Lake on roadside of Apache Trail, Maricopa Co, 29 Apr 1960, *Dullas, W. 123* (K); Maricopa County, 2 mi W of Humbolt Lookout, 21 Dec 1974, *Engard, R.G. & Squires, D. s.n.* (DES); Santa Cruz County, Sonoita Creek below Patagonia (Nature Conservancy Preserve), 12 May 1972, *Engard, R.G. s.n.* (DES); Maricopa County, Rte 88, foot of Fish Creek Hill, 10 Nov 1973, *Engard, R.G. 158* (DES); Gila County, Needle Canyon Trail, Superstition Mountains, 23 Mar 1974, *Engard, R.G. & Getz, M.L. 182* (DES); Pinal County, Wide bench in Hidden Canyon, Mineral Hills, 8 May 1976, *Engard, R.G. & Haughey, R.A. 930* (DES); Graham County, Salazar Ranch, Aravaipa Canyon, 24 Oct 1976, *Engard, R.G. 988* (DES); Maricopa County, Rte 88, 3 mi north of Tortilla Flats, 10 Nov 1973, *Engard, R.G. & Lehr, J.H. 1150* (DES); Pima County, Organ Pipe Cactus National Monument; Bull Pasture trail in Estes Canyon, Ajo Mountains, 9 Apr 2005, *Felger, R.S. & Enderson, E.F. 05-170* (ARIZ); Pima County, Organ Pipe Cactus National Monument, Alamo Canyon, 29 Mar 2003, *Felger, R.S. et al. 03-406* (ARIZ); Pima County, Organ Pipe Cactus National Monument, N-facing slopes of small side canyon immediately below on west sdie of the arch in arch Canyon, Ajo Mountains, 2 Dec 1990, *Felger, R.S. & Baker, M.A. 90-506* (ARIZ, MEXU); Cochise County, Chiricahua Mountains, entrance to Chiricahua National Monument, 8 Jul 1940, *Ferris, R.S. 9998* (UC); Maricopa County, Superstition Mountains: Mesquite Wash (Tonto National Forest), west of Hwy 87 ca 20 miles N of Apache Junction. T5N R8E S34 SE4, 8 Oct 2014, *Fertig, W. et al. 30076* (IND); Maricopa County, Arizona Uplands: SW Superstition Mountains, Crosscut Trail, ca 0.5 miles NE of Lost Dutchman State Park, 4.5 miles NE of US 60/89 at Apache Junction. T2N R9E S31 E2 SW4, 1 Apr 2015, *Fertig, W. 30376* (ASU); Pima County, Honey Bee Canyon, Valley between Santa Catalina and Tortolita Mountains, about 7kmn north of Oro Valley, 29

Mar 1995, *Fishbein, M. et al. 2216* (ARIZ); Pima County, Cienega Creek Natural Preserve, Empirita Ranch Headquarters tract, in floodplain of Cienega Creek, 15 Aug 2015, *Fonseca, J. & Bertelsen, D. 2015-545* (ASU); Pima County, Cienega Creek Natural Preserve, Empirita Ranch Headquarters tract, in floodplain of Cienega Creek, 15 Aug 2015, *Fonseca, J. & Bertelsen, D. 2015-545* (ARIZ); Pinal County, Box Canyon, 2 miles east of Florence junction and 4 miles south, 11 Oct 1968, *Foote, J. s.n.* (DES); Pima County, Ajo, Organ Pipe Cactus National Monument, Alamo Canyon, 28 Jun 1947, *Fouts, C.L. 188* (US); Yavapai County, upper Oak Creek, 18 Jun 1934, *Fulton, H.J. 9668* (US); Yavapai County, Prescott Mountains, Aug 1937, *Gentry, H.S. 3083* (MICH); Maricopa County, Tonto National Forest where FS Road 1530 meets Lime Creek, 18 Sep 2006, *Goldman, D. & Ward, J. DG-568* (DES); Pima County, Alamo Canyon, Ajo Mountains, 9 Mar 1946, *Goodding, L.M. 11 -46* (ARIZ, NY); Pima County, Canyon Bottom. Moristo Canyon, Baboquivari Mountains, 31 Oct 1945, *Goodding, L.M. 406 -45* (MICH, UT); Pima County, Alamo canyon, Ajo Mountains, 10 Dec 1945, *Goodding, L.M. 482 -45* (ARIZ, MICH, MIN, NY, UT); Cochise County, E of Douglas, 28 May 1907, *Goodding, L.M. 2285* (MO, RM); Greenlee County, East Eagle Creek, 24 miles from Coronado Trail road junction, 6 Sep 1948, *Gould, F.W. & Robinson, M.E. 5210* (ARIZ, NY); Pinal County, North side at mouth of Box Canyon; ca. 3 miles northeast of Price, 25 Mar 1966, *Haase, E.F. et al. 715* (UT); Pinal County, near San Fernando, 21 Aug 1932, *Harrison, G.J. & Kearney, T.H. 8949* (F); Maricopa County, Brushy Basin, 8 Nov 1977, *Haughey, R.A. s.n.* (DES); Pinal County, Box Canyon, 1 Apr 1977, *Haughey, R.A. & Krummen, S. 157* (DES); Yuma County, Palm Canyon, Kofa Mountains, *Hodgson, W.C. & Mittleman, M. H-434* (DES); Pima County, Molino Basin Campground, Santa Catalina Mountains, 16 Aug 1980, *Hodgson, W.C. H-954* (DES, H); Pinal County, Superstition Mountains, Hierglyphic Spring, 4 Mar 1981, *Hodgson, W.C. H-1190* (DES, MEXU); Cochise County, Across from Stewart Campground, 4 mi SW of Portal, Chiricahua Mountains, 20 Aug 1981, *Hodgson, W.C. & Buchanan, D. H-1513* (DES); Gila County, Ca. 0.5 mi S of state fish hatchery, Tonto National Forest, 16 Sep 1981, *Hodgson, W.C. H-1628* (DES); Maricopa County, Off Rte 88, at Canyon Lake, between first and second bridges, 10 Mar 1983, *Hodgson, W.C. & Woodruff, M. H-1990* (DES); Pinal County, Picnic area, Peralta Canyon, Superstition Mountains, 18 Mar 1980, *Hodgson, W.C. & Lehr, J.H. 2152* (DES); Pinal County, Peralta Trail between picnic area and Fremont Saddle, Superstition Mountains, 18 Mar 1980, *Hodgson, W.C. & Lehr, J.H. 2161* (H); Pinal County, Peralta Trail between picnic area and Fremont Saddle, Superstition Mountains, 18 Mar 1980, *Hodgson, W.C. & Lehr, J.H. 2161* (DES); Pinal County, S of Superior; Walnut Canyon ca. 1 mi S of junction with White Canyon, 11 Dec 1983, *Hodgson, W.C. & Buchanan, D. H-2619* (DES); Gila County, Tonto National Forest, Salt River Wilderness Area, approximately across from where Canyon Creek enters Salt River, 23 Feb 1989, *Hodgson, W.C. & Harris, S. 5403* (DES); Santa Cruz County, Tumacacori Mountains, SW of Rock Corral and FSR 4175, in Rock Corral Canyon, 12 May 1992, *Hodgson, W.C. 6671* (DES); Mohave County, Grand Canyon, Hualapai Indian Reservation, Spencer Canyon within 1/2 mile south of Colorado River at Colorado River Mile 246, 12 Sep 1994, *Hodgson, W.C. et al. 8537* (DES); Mohave County, Grand Canyon National Park, Tuck Up Canyon, 15 Mar 1996, *Hodgson, W.C. & Brian, N. 9677* (DES); Pinal County, Tonto National Forest, along Arizona Trail, ca 1/2 mile north of Telegraph Canyon Road (FR 4), ca 9 miles southwest of highway 177, in vicinity of south end of Alamo Canyon, 1 Mar 2001, *Hodgson, W.C. & Slauson, L. 13270* (DES); Coconino County, Grand Canyon National Park, Phantom Canyon, ca. 1/2 to 3/4 mile above (upstream from) junction with Haunted Canyon, 13 Sep 2003, *Hodgson, W.C. et al. 17433* (DES); Pima County, Santa Catalina Mountains, Molino Basin, Arizona National Scenic Trail, east of highway and campground, 27 Apr 2005, *Hodgson, W.C. et al. 20128* (DES); Mohave County, Grand Canyon National Park, Matkatamiba Canyon, river left, 350109 E, 4022976 N, NAD 83, 10 May 2006, *Hodgson, W.C. et al. 21014* (DES); Mohave County, Grand Canyon, Hualapai Indian Reservation, National Canyon, Transect T1A, 330114 E, 4014056 N, NAD 83, 11 May 2006, *Hodgson, W.C. et al. 21033* (DES); Mohave County, Grand Canyon, Hualapai Nation, Mohawk Canyon, 13 May 2006, *Hodgson, W.C. et al. 21105* (DES); Mohave County, Grand Canyon, Hualapai Indian Reservation, Mohawk Canyon, 12S 323073 E, 4010136 N, NAD 83, 13 May 2006, *Hodgson, W.C. et al. 21105* (DES); Coconino County, Grand Canyon National Park, Dutton Spring, north of (above) where Deer Creek Trail crosses Deer Creek, 18 May 2013, *Hodgson, W.C. et al. 28481* (DES); Pima County, Baboquivari Mountains, just above Baboquivari Camp, 18 airline miles east-southeast of Sells, 12 Apr 1973, *Holmgren, N.H. 6766* (NY); Pima County, Madera Canyon Recreation Area, 19 Aug 1968, *Hsiao,*

T.H. 102 (F); Maricopa County, Spur Cross Ranch Conservation Area. Cave Creek, just south of border with Tonto National Forest, 28 Jun 2008, *Hunkins, S. & Smith, K. 524* (DES); Maricopa County, Mormon Flat, 13 Mar 1920, *Irish, F.M. s.n.* (ASU); Pima County, Santa Rita Mts, 22 Aug 1903, *Jones, M.E. s.n.* (BM); Maricopa County, McDowell Sonoran Preserve, Tom's Thumb Trail below Glass Dome, along trail, 2011, *Jones, S. 1502* (ASU); Maricopa County, Tonto National Forest, Camp Creek, 0.55 mile south of Bartlett Dam Road on FS trail 413, 21 May 2015, *Jones, S. 2031* (DES); Maricopa County, 0.8 miles south of Canyon Lake Lookout on Apache Trail, 8 Apr 1967, *Keil, D.J. 1152* (ASU); Gila County, headquarters, Sierra Ancha Experimental Forest, 22 Aug 1968, *Keil, D.J. 3513* (F); Maricopa County, Apache Lake, 18 Apr 1942, *Kimball, T. s.n.* (DES); Pima County, Charco #1, Papago Indian Reservation, 4 Sep 1968, *Kobetich, G.C. 68-161* (NY); Maricopa County, upland Sonoran Desert; six miles E of Tortilla Flat on hwy 88, 1 Oct 1986, *Landrum, L.R. & Landrum, S.S. 5296* (ASU, MO); Pinal County, Tonto Nat. Forest. Peralta Canyon Trail Head along path to Fremont Saddle. Between trailhead and ca. Â¼ mi along trail, 28 Feb 1995, *Landrum, L.R. 8506* (NY); Pima County, Molina Basin Campground, Mt. Lemmon Highway, 9 Aug 1972, *Lehr, J.H. 1652* (MEXU); Coconino County, Cove Canyon in bowl below falls of tamisk removal site, 11 Jun 2004, *Makarick, L.J. & Watters, K. 37* (DES); Cochise County, San Pedro Riparian National Conservation Area, Upper San Pedro River floodplain, approx 2 miles south of Palominas Rd, ~50 m east of San Pedro, 17 Aug 2001, *Makings, E. 546* (NY); Maricopa County, Tonto National Forest; unnamed wash that intersect Cline Cabin Road toward Four Peaks near target shooting area, 29 Sep 2015, *Makings, L. et al. 4750* (ASU); Cochise County, San Bernardino Ranch; 17 miles east of Douglas, AZ, 25 May 1981, *Marrs, G.E. 535* (ASU); Maricopa County, Mormon Flat Dam, 11 Apr 1971, *McGill, L. s.n.* (DES); Pima County, Tanque Verde Road to Redington Pass through Coronado National Forest, 11 Apr 1992, *Merello, M. et al. 162* (MO); Mohave County, Gold Roads, 1 Apr 1959, *Michaels, C.C. 2094* (ARIZ); Maricopa County, ca. 7 miles E of Tortilla Flat on Arizona route 88, at Fish Creek, 30 Jun 1992, *Miller, J.S. et al. 7731* (MO); Pinal County, Devil's Canyon, Pinal Mountains between Superior and Miami (Rte 60), 16 Apr 1980, *Mittleman, M. & Lehr, J.H. 2209* (DES); Maricopa County, Canyon Lake, Apache Trail, 2 May 1935, *Nelson, A. & Nelson, R.A. 1725* (K, MO, NY, RM, WIS); Gila County, near Globe, 3 May 1925, *Nelson, A. 10326* (NY, RM); Maricopa County, on the lake shore, Canyon Lake, 19 Mar 1930, *Nelson, A. 11220 a* (MO, NY, RM); Pima County, Organ Pipe Cactus National Monument; Pitahaya Canyon, Ajo Mts., 23 Feb 1939, *Nichol, A.A. s.n.* (ARIZ); Yuma County, Gila Mountains, upper terrace of reno Creek drainage, reno Creek, just W of highway 188 on FS 524, ca. 3.2 miles W on FS 524, then turn directly S to enter the general drainage area, 30 Aug 2006, *Price, T. 470* (MEXU); Yuma County, Gila Mountains, Reno Creek ca. 3 miles from Hwy 188, jsut south of Reno Creek Road, 23 Sep 2006, *Price, T. & Porter, B. 554 b* (MEXU); Pinal County, Tonto National Forest. Superstition Wilderness Area. Peralta Trailhead, 7 mi. N of U.S. Hwy. 60, on Forest Rd. 77. Trail 102, 26 Mar 1991, *Rice, K.C. 1146* (DES); Pinal County, Tonto National Forest. Superstition Wilderness Area. Heiroglyphic Spring, NE of U.S. Hwy. 60 ca. 3 mi on turnoff to Gold Canyon Ranch, *Rice, K.C. et al. 1316* (DES); Pinal County, Tonto National Forest, Superstition Wilderness Area, Heirglyphic Spring, 2 miles N of US Why 60 at Gold Canyon ranch turnoff, 12 Oct 1991, *Rice, K.C. et al. 1587* (ARIZ, ASC, MEXU); Cochise County, Pat Hills, ca. 30 km (by air) SE of Willcox, Sulphur Springs Valley, 10 Sep 2015, *Roll, C.M. 954* (ARIZ); Pima County, Camp Grant, Jul 1874, *Rothrock, J.T. 388* (GH); Pima County, Fort Lowell, "Camp Lowell", Sep 1874, *Rothrock, J.T. 707* (F, NY, US); Gila County, Camp McDonald, [Fort (William) McDonald, near Payson], 19 Sep 1956, *Smart*, *33* (GH); Yavapai County, Grapevine Canyon approx 4 miles N.W. of Mayer. 1.18 miles up canyon from trail 4 trailhead on FR87A, *Smith, S. et al. 110* (DES); Maricopa County, 1 mile West of Tortilla Flats on State Route 88, 26 Dec 1972, *Solomon, J.C. 419* (MO); Maricopa County, Superstition Mountains; 2.5 miles from Tortilla Flat; along highway 88 near mile post 216, 6 Feb 2016, *Solves, J.-.P. et al. 25* (ASU); Maricopa County, Tortilla creek, *Stepan, R. s.n.* (ASU); Coconino County, Grand Canyon National Park; Colorado River mile 148. Matkatamiba Canyon, 18 Apr 1992, *Stevens, L.E. s.n.* (ARIZ); Pima County, Canyon Diablo, Ajo Mountains, Organ Pipe Cactus Nat Mon, 15 Apr 1951, *Supernaugh, W.R. 443* (ARIZ); Pima County, Organ Pipe Cactus National Monument, Boulder Canyon, first steep side drainage on the south side of Boulder Canyon, after entering the canyon proper, 11 Sep 2004, *Tewksbury, J.J. & Carlo, T. s.n.* (ARIZ); Pima County, Fort Lowell, 31 Jul 1883, *Thornber, J.J. 249* (ASC, MO, NY); Yavapai County, 3 miles SW of Cottonwood at the Quail Springs Ranch, 5 Sep

2013, *Thornburg, D.C. 1153* (NY); Cochise County, Chiricahua Mtns, 26 Jul 1894, *Toumey, J.W. s.n.* (NY); Pima County, Tucson, 20 Jul 1895, *Toumey, J.W. s.n.* (NY); Yavapai County, Copper Basin, 25 Jun 1892, *Toumey, J.W. 397* (UC, US); Pima County, S fork of Alamo Canyon, Ajo Mountains, Organ Pipe Cactus Nat Mon, 31 Aug 1978, *Van Devender, T.R. s.n.* (ARIZ); Pima County, Saguaro National Park, Rincon Mountain District; Rincon Creek, 24 May 2001, *West, P.A. 710* (ARIZ); Coconino County, Havasu Canyon, Oct 1940, *Whiting, A.F. 1047-4587* (ARIZ); Pinal County, 14 miles south of Kalvin, 5 May 1961, *Wicklund, C.P. 34* (BM); Ft. Huachuca, May 1892, *Wilcox, T.E. s.n.* (NY); Yavapai County, along Wet Beaver Creek above its confluence with Long Canyon ca. 2.06 km SSE of Casner Canyon Tank; Coconini N.F. Casner Butte Quad (7 1/2 min.); T15N, R6E, Sec. 24; UTM -3836975 m.N by 439775 m.E (Zone 12), 4 Sep 1993, *Windham, M.D. 93-233* (MO, UT); Yavapai County, Sycamore Canyon Wilderness, Sycamore Creek and Summer Spring ; N 38 60 300, E 4 02 550; T17N R3E NW1/4 SE1/4 s5, 26 Jun 1992, *Wright, T. & Baker, M. 92-172* (DES); Yavapai County, Sycamore Canyon Wilderness, Sycamore Creek at Summer Spring, 26 Jun 1992, *Wright, T. & Baker, M. 92-172* (ASU). **California**: Ventura County, Sulphur Mountain Spring, Sulphur Mountains, 1 Jun 1908, *Abrams, L. & McGregor, E.A. 23* (GH); San Bernardino County, Lone Pine Canyon, slopes of San Gabriel Mountains, 5 Jul 1908, *Abrams, L. & McGregor, E.A. 690* (GH); Los Angeles County, Inglewood, 31 May 1902, *Abrams, L. 2488* (BM, E, F, GH, K, MO, NY); Santa Barbara County, vicinity of Pelican Bay (Santa Cruz Island); Santa Cruz Island, 26 Apr 1930, *Abrams, L.R. & Wiggins, I.L. 75* (GH, MIN, UC); Santa Barbara County, vicinity of Smuggler's Cove (Santa Cruz Island); Santa Cruz Island, 29 Jun 1931, *Abrams, L.R. & Wiggins, I.L. 195* (UC); Los Angeles County, vicinity of Mosquito Harbor (San Clemente Island); San Clemente Island, 6 Jul 1931, *Abrams, L.R. & Wiggins, I.L. 346* (MICH, UC); San Mateo County, Ano Nuevo Point, 3 Jun 1939, *Abrams, L.R. 425* (MICH, UC); San Diego County, Witch Creek, May 1894, *Alderson, R.D. s.n.* (UC); San Diego County, Aug 1897, *Allen, T.F. s.n.* (NY); San Diego County, Del Mar, 23 Apr 1895, *Angier, B.S. 93* (MO); Santa Barbara County, St. Ynez [Santa Ynez], 4 Feb 1855, *Antisell, T. s.n.* (NY); Orange County, Rancho Santa Ana in the Santa Ana Canyon, 8 Feb 1952, *Balls, E.K. 8185* (BM, E); Santa Barbara County, East end of Prisoner's Harbour. Santa Cruz Island, 13 Oct 1958, *Balls, E.K. 11839* (BM, E); Santa Barbara County, e end of Prisoner's Harbor, Santa Cruz Island Prisoner's Harbor, Santa Cruz Island, Prisoner's Harbor, 13 Oct 1958, *Balls, E.K. & Blakley, E.R. 23678* (UC); San Diego County, Agua Tibia Mountains, NW Paolmar Mountains, Wilderness Gardens Preserve, ca. 3.4 miles E of Paia south of Hwy 76 along the San Luis Rey River, areas along the main trail network from teh parking area alogn the primary driveway within the river bottom to areas of native grasslands on the higher slopes, 8 Apr 1997, *Banks, D.L. 1714* (F); Los Angeles County, Santa Monica experiment Station, 16 Mar 1897, *Barber, J.H. 65* (UC); Los Angeles County, W side of Rustic Conyon ca. 1 km N of Pacific Ocean, 24 Dec 1983, *Bartholomew, B. 1521* (GH); Santa Cruz County, Ridge w of Larkin Valley, 21 Feb 1936, *Belshaw, C.M. 1488* (UC); San Luis Obispo County, 1.4 mi ssw of Hollister Peak, 23 Mar 1936, *Belshaw, C.M. 1714* (UC); Los Angeles County, Huntington Palisades, 29 Apr 1929, *Bettys, J.A. s.n.* (JEPS); Santa Barbara County, old sand dunes on cliff above n.w. anchorage Beechers Bay, Channel Isl.'s, Santa Rosa Isl, 5 Apr 1960, *Blakley, E.R. 3124* (JEPS, NY, OBI, SBBG); Santa Barbara County, Santa Cruz Island, on stream terrace at the bottom of Portezuelo Grade, 2 Mar 1961, *Blakley, E.R. 3894* (K, RSA); Santa Barbara County, base of cliffs at Cuyler Harbor (San Miguel Isl.), Channel Isl.'s, Cuyler Harbor, 28 Apr 1961, *Blakley, E.R. 4186* (JEPS, RSA, SBBG); Santa Barbara County, at foot of e. side Green Mt. (at airplane wreck, San Miguel Isl.), Channel Isl.'s, San Miguel Isl. Green Mt, 3 Apr 1962, *Blakley, E.R. 5063* (JEPS, RSA, SBBG); Orange County, Orange, Uplands, 10 Mar 1929, *Blankinship, J.W. s.n.* (MO); San Luis Obispo County, Cambria, Santa Rosa Creek, on the farm of C. O. Blodgett Santa Rosa Creek; Cambria, Santa Rosa Creek, 4 Aug 1951, *Blodgett, C.O. & Paddock, E.F. 415* (UC); San Luis Obispo County, 2 mi se of mouth of Osos Creek, 14 Jan 1936, *Bolt, B. 529* (UC); Orange County, Newport Bay, 19 May 1932, *Booth, L.M. 1075* (UC); Riverside County, Vail Lake Area, Temecula Creek Canyon, below the dam Temecula Creek Canyon, Vail Lake Area, Temecula Creek Canyon, 10 Jun 1989, *Boyd, S. et al. 3665* (RSA, UC); Los Angeles County, San Clemente Island. Lower half of China Cyn, S end of island, W side, 7 Apr 1990, *Boyd, S. et al. 4259* (BM, F, MO, SBBG); Los Angeles County, San Clemente Island, 25 Aug 1894, *Brandegee, T.S. s.n.* (UC); Santa Barbara County, near Santa Barbara (Santa Inez Mountains); Santa Inez Mountains, *Brandegee, T.S. s.n.* (UC); Los Angeles County, sin. loc, 1860, *Brewer, W.H. 33* (UC); Ventura County, Oxnard,

1901, *Burtt Davy, J. 7796* (UC); San Luis Obispo County, 1.1 mi nnw of Corbett, 21 Mar 1936, *Carlson, N.K. 200* (UC); Los Angeles County, Claremont, 21 Jun 1897, *Chandler, H.P. s.n.* (UC); Santa Barbara County, San Marcos Creek, 30 Jan 1930, *Clar, C.R. 55* (UC); San Benito County, San Diego, *Cleveland, D. s.n.* (GH); Santa Barbara County, Santa Cruz Island; Santa Barbara Islands, Pelican Bay, Santa Cruz Island, 2 May 1931, *Clokey, I.W. 5201* (BUT, CM, F, GH, NY, RENO, RSA, UC, WIS); San Luis Obispo County, San Luis Obispo, Mar 1905, *Condit, I.J. s.n.* (UC); Ventura County, Oxnard, 10 Jul 1908, *Condit, I.J. s.n.* (UC); Los Angeles County, Los Angeles, 1860, *Cooper, J.G. 33* (GH); sin. loc, *Coulter, T. s.n.* (K); sin. loc. [one sheet has Monterey in pencil], *Coulter, T. 591* (GH); sin. loc. [mounted on sheet with S. triquetrum -surely not hte same colelction number! SKnapp 12/2014], *Coulter, T. 592* (K); Los Angeles County, Mystic Canyon, Angeles National Forest, San Gabrielle Mountains. 2 mi NE of Glendora, California, 8 May 1971, *Cromwell, G.D. & Wheat, D.W. 634* (UT); San Bernardino County, Ca 3/4 mi SE of San Antonio Dam bottom of S-facing slope. Ontario Quad 15'. SE corner of Sec 24, T1N, R8W, 5 Apr 1977, *Danna, J.A. 2* (MO); [Palomino]? Canyon, 30 Jun 1915, *Davis, A.R. s.n.* (MO, NY); Los Angeles County, stream bank s side of Tuna Canyon ca. 15 m s of Tuna Canyon Road and ca. 1.0 km n of Pacific Coast Highway, ca. 6.0 air km w of Pacific Palisades; T1S, R16W, Sec. 30 extreme se corner, 11 Apr 1982, *Dawson, A.W. 4* (MO); San Mateo County, 2 miles S of Pigeon Point, 27 Jul 1933, *Demaree, D. 10443* (MO); San Francisco County, San Francisco Bay region, *Dempster, L.T. 1097* (UBC); Nova California, 1833, *Douglas, D. s.n.* (BM, G-DC, K); California, 1833, *Douglas, D. s.n.* (K); Santa Cruz County, on Bonny Doon road a few miles inland from the coast (south of Davenport), 10 May 1951, *Dress, W.J. 3024* (BM, LE); San Luis Obispo County, Cambria, 3 Apr 1902, *Dudley, W.R. s.n.* (NY); Monterrey [pt. perros]?, 30 May 1912, *Eastwood, A. 187* (GH, MO, NY); Monterey County, near Asilomar 28 Jan 1935, *Eastwood, A. & Howell, J.T. 1924* (F, MICH, MO, NY, POM, RSA, UC); Monterey County, Pacific Grove, Monterey, 9 Nov 1912, *Eastwood, A. 2407* (GH); San Luis Obispo County, Parfuma Canyon, 21 Apr 1941, *Eastwood, A. 18966* (UC); Los Angeles County, Echo Mountain, Apr 1901, *Eby, A.F. s.n.* (MO); Santa Barbara County, Santa Cruz Island, along road to and near Prisoner's Harbor, 3-4.2 miles from the field station, 2 Aug 1987, *Elias, T.S. & Wisura, W. 10580* (MEXU); Santa Barbara County, Santa Barbara, May 1902, *Elmer, A.D.E. 3945* (E, GH, K, MIN, MO, NY, POM, UC); Santa Barbara County, 3 mi n of Summerland, Santa Barbara National Forest, 8 Jan 1935, *Embree, F.W. 264* (UC); Santa Barbara County, just e of mouth of Toro Canyon, 25 Feb 1935, *Embree, F.W. 315* (UC); Los Angeles County, Mandeville Canyon, Santa Monica Mountains, 8 Mar 1930, *Epling, C. s.n.* (LE); Los Angeles County, Mandeville Canyon, Santa Monica Mountains, 8 Mar 1930, *Epling, C. s.n.* (MO); Los Angeles County, Mandeville Canyon, southern California, 8 Mar 1930, *Epling, C. s.n.* (K); Los Angeles County, Mandeville Canyon, southern California, 8 Mar 1930, *Epling, C. s.n.* (K); Los Angeles County, Santa Monica Mountains, Mandeville Canyon Mandeville Canyon; Santa Monica Mountains, Mandeville Canyon, 8 Mar 1930, *Epling, C. s.n.* (UC); Los Angeles County, Mandeville Canyon, 8 Mar 1930, *Epling, C.C. s.n.* (F); Los Angeles County, Santa Catalina Island, 29 Mar 1908, *Evermann, B.W. s.n.* (GH); Los Angeles County, Verdugo Range, Southard Peak Trail, 8 Mar 1930, *Ewan, J.A. 3715* (UC, UCR); Santa Barbara County, Los Alamos, 1 Aug 1937, *Ewan, J.A. 10775* (GH); California, uplands; Millenium Seed Bank Project, serial number 0071299, *Feltwell, J. 19* (K); San Diego County, Mesa Grande, along old stage road Mesa Grande, 20 May 1917, *Ferguson, E. 69* (JEPS); San Mateo County, State Highway 5, above Sharp Park, near junction of State Highway 5 and 1, 25 Sep 1945, *Ferris, R.S. 11105* (GH); San Mateo County, State Highway 1 n of Pedro Point, 25 Sep 1945, *Ferris, R.S. 11106* (GH, UC); San Diego County, near Rainbow, 17 May 1946, *Fiker, C.B. 3469* (UC); Los Angeles County, San Antonio Canyon, San Gabriel Mountains, 20 Oct 1952, *Finch, A.J. 208* (UBC); San Mateo County, 2.6 mi n of Pigeon Point (beside the main highway), 18 Feb 1934, *Follett, W.I. 10* (JEPS); Los Angeles County, Santa Catalina Island, Hamilton Canyon, 20 Mar 1931, *Fosberg, F.R. S 4312* (F, MO, NY, RSA, UC, WIS); 1845, *Frémont, J.C. s.n.* (K); 1845, *Frémont, J.C. s.n.* (K); 1845, *Frémont, J.C. s.n.* (K); sin. loc, 1846, *Frémont, J.C. s.n.* (NY); sin. loc, 1845, *Frémont, J.C. s.n.* (GH); Ventura County, Sycamore Canyon, 10 May 1934, *French, N. 342* (UC); San Luis Obispo County, Santa Barbara National Forest, Cuesta Pass Cuesta Pass, Santa Barbara National Forest, Cuesta Pass, 13 Jan 1936, *French, N. 664* (UC); Orange, 31 May 1889, *Fritch, R. s.n.* (MO); Los Angeles County, Orange, 4 Mar 1889, *Fritchey, J.Q.A. s.n.* (MO); Riverside County, 12 mi s of Corona, 17 Apr 1943, *Gould, F.W. 2115* (UC); Los Angeles County, near corner of Skyline and Ardsheal Drives, Whittier Hills Whittier Hills, 16 Apr

1944, *Gould, F.W. 2298* (RSA, UC); Los Angeles County, Pasadena, 10 May 1904, *Grant, G.B. 1219* (F, GH); Monterey County, Carmel, "all California", 1885, *Gray, A. s.n.* (GH); Solano County, South Vallejo, 1874, *Greene, E.L. s.n.* (LE); Santa Barbara County, Santa Cruz Island, Aug 1886, *Greene, E.L. s.n.* (F); Los Angeles County, near Pasadena, 26 Aug 1906, *Grinnell, J. & Grinnell, H.W. 361* (UC); Ventura County, Los Padres National Forest on Hwy 23, Forest Rd 6N16 in the Sespe Condor Sanctuary, 18 Mar 2005, *Gust, G. & Nye, L. 603* (MO); Riverside County, vicinity of Riverside, Aug 1901, *Hall, H.M. s.n.* (UC); San Luis Obispo County, head of Old Creek, Cayucos Road, 10 Mar 1958, *Hardham, C.B. 2882* (JEPS, RSA); Los Angeles County, Los Angeles, May 1889, *Hasse, H.E. s.n.* (F); Los Angeles County, Laguna Beach, 17 Nov 1938, *Hastings, G.T. s.n.* (NY); Santa Barbara County, Surf, just n of railway station Surf, 16 Jun 1970, *Heckard, L.R. & Chuang, T.I. 2455* (JEPS); Monterey County, on R bank of Carmel River 3 miles above the Mission, 16 Apr 1903, *Heller, A.A. s.n.* (GH); San Luis Obispo County, Central Coast, junction of Martigale and Buckskin in Los Osos, 23 Nov 1995, *Helmkamp, E.A. 110* (UBC); *Herb. Hooker*, *s.n.* (K); Orange County, Santa Ana Canyon, 8 miles E of Yorba Linda, 17 Mar 1964, *Hitchcock, C.L. & Muhlick, C.V. 23063* (F, LE, NY, UC, UT); Monterey County, Monterey Peninsula, Moss Beach Moss Beach; Monterey Peninsula, Moss Beach, 25 May 1941, *Hoover, R.F. 5235* (NY, UC); Los Angeles County, below C. C. C. Camp (Dalton Canyon, Dalton Watershed), Dalton Canyon, 22 May 1935, *Horton, J.S. 143* (UC); Orange County, Santa Ana, 26 Oct 1894, *Hughes, E.L. 32* (GH); Los Angeles County, Garvanza, 27 Dec 1894, *JBP*, *s.n.* (GH); San Luis Obispo County, Mouth of San Carpoforo Creek San Carpoforo Creek, 6 Jul 1930, *Jensen, H.A. 37* (UC); Kern County, Tejon Ranch. Tejon Hills, Comanche Point. In and immediately around Comanche Spring, 7 Sep 2014, *Jensen, N. & Kramer, N. 2470* (UC); Monterey County, between Malpaso Creek and Carmel (Monterey Coast); Monterey Coast, 17 Sep 1906, *Jepson, W.L. 2618* (JEPS); San Bernardino County, Waterman Canyon, 15 May 1913, *Jepson, W.L. 5559* (JEPS); Santa Barbara County, Santa Inez Mountains, Gaviota Canyon, 8 Apr 1920, *Jepson, W.L. 8452* (JEPS); San Diego County, w of Borrego Valley (Palm Canyon of Mount San Ysidro), Mount San Ysidro, Palm Canyon, 28 Apr 1920, *Jepson, W.L. 8806* (JEPS); Los Angeles County, Mission San Fernando, 3 May 1920, *Jepson, W.L. 8919* (JEPS); Santa Barbara County, Santa Inez Mountains, 13 Apr 1921, *Jepson, W.L. 9119* (JEPS); Santa Barbara County, Canada del Capitan, 24 May 1927, *Jepson, W.L. 11911* (JEPS); San Luis Obispo County, Pismo, 14 Apr 1939, *Jepson, W.L. 19089* (JEPS); San Luis Obispo County, Pismo, 14 Apr 1939, *Jepson, W.L. 19090* (JEPS); Ventura County, 2 1/2 mi N of Ventura (Los Sauces Creek), 4 Oct 1940, *Jepson, W.L. 20160* (JEPS); Riverside County, Riverside, "from Riverside to Santa Rosa Peak and Palomar" Riverside, 17 May 1901, *Jepson, W.L. 21256* (JEPS); Ventura County, 2 1/4 mi w of Santa Susana (Tapo Canyon), Tapo Canyon, 29 Jan 1934, *Johannsen, P.L. 254* (UC); Ventura County, e of Fillmore, 21 Mar 1934, *Johannsen, P.L. 331* (UC); Los Angeles County, W of Pomona, 16 Mar 1926, *Jones, M.E. s.n.* (GH); Ventura County, onshore bluffs just w. of mouth of Cherry Canyon, West Anacapa Isl, 3 Oct 1978, *Junak, S.A. et al. WA-22* (JEPS); San Mateo County, Ano Nuevo Point, at Ano Nuevo Creek Ano Nuevo Point; Ano Nuevo Creek, 7 Jul 1934, *Keck, D.D. 2970* (POM, UC); Los Angeles County, Los Angeles, end of sidewalk along Eagle Rock View Drive, south face of the Eagle Rock, 19 Jan 2001, *Keefe, J.M. 30944* (MEXU, RSA); San Luis Obispo County, Camp San Luis Obispo. Chorro Creep drainage. Chorro Creek Bog Thistle exclosure. Vicinity of 35.3442i?1/2N, 120.6800i?1/2W, 15 Jun 2001, *Keil, D.J. et al. 29577* (UC); Mereed Lake, 22 Aug 1868, *Kellogg, A. & Harford, W.G.W. 716 a* (BM, NY); Monterey County, Rancho Encinal, 15 Apr 1903, *Kellogg, W. s.n.* (GH); Monterey County, Rancho encinal, Apr 1904, *Kellogg, W. 3* (UC); Orange County, Santa Ana, 1 Mar 1908, *King, A. s.n.* (JEPS); Santa Catalina Island, *Knopf, E.C. 65 236* (BM, K); Los Angeles County, Pebble Beach Canyon, 26 Mar 1921, *Knopf, E.C. 65* (F); Los Angeles County, Santa Catalina Island, from Avalon Canyon to right of road near Chicken John's, 15 Sep 1921, *Knopf, E.C. 180* (F); Santa Catalina Island, *Knopf, E.C. 188 -236* (BM, K); Los Angeles County, Santa Catalina Island, near summit of Quartz Mountain, W of Catalina Harbor, at the Isthmus, 9 Oct 1921, *Knopf, E.C. 188* (F); Los Angeles County, Santa Catalina Island, Howlands, 8 Oct 1921, *Knopf, E.C. 198* (BM, F, K); Los Angeles County, Santa Catalina Island, Eagle's Nest, *Knopf, E.C. 220* (F); Los Angeles County, Santa Catalina Island, Middle Ranch Canyon, between Middle Ranch and the mouth of the canyon, 21 Nov 1921, *Knopf, E.C. 236* (BM, F, K); Los Angeles County, Santa Catalina Island, Camp Banning Canyon, 6 Nov 1921, *Knopf, E.C. 239* (BM, F, K, UC); Los Angeles County, Santa Catalina Island, Avalon Canyon, just beyond Chicken John's, 24 Jan 1922, *Knopf, E.C. 278* (F, UC); Riverside

County, Woodcrest area: 1/2 mi NE of intersection of Van Buren Blvd. & Mockingbird Canyon Rd, 7 Apr 1989, *LaRue, E. s.n.* (DES); Monterey County, on ne side of Whaler's Knoll (Point Lobos State Park); Point Lobos State Park, 9 Feb 1935, *Lee, E. & Mason, H.L. 9196* (UC); Monterey County, ne of Whaler's Knoll (Point Lobos State Park); Point Lobos State Park, 9 Feb 1935, *Lee, E. & Mason, H.L. 9197* (UC); Santa Barbara County, 4 1/2 mi s of Carreaga, 25 Apr 1935, *Lee, H.C. 305* (UC); San Luis Obispo County, 2 mi se of Pichacho (ne of Los Berros), 24 Feb 1936, *Lee, H.C. 417* (UC); Orange County, Santa Ana Canyon, Mar 1934, *Lewis, A. 337* (UC); Riverside County, Temescal Canyon, Ste. Monica mte, Temescal Cañon, 25 Mar 1933, *Lloyd, E. s.n.* (MO); Los Angeles County, Santa Monica Mountains, Temescal Canyon Temescal Canyon; Santa Monica Mountains, Temescal Canyon, 25 Mar 1933, *Lloyd, E. s.n.* (UC); Los Angeles County, Verdugo Canyon near Los Angeles, 25 Jun 1915, *Macbride, J.F. & Payson, E. 772* (GH); Riverside County, Tahquitz Canyon, SW of Palm Springs, 15 Oct 1941, *Macbride, J.F. & Drouet, F.E. 4477* (ECON); Los Angeles County, Tuna Canyon, Verduga Hills, 18 Mar 1931, *MacFadden, F.A. 6 E* (GH, MO, UC); San Luis Obispo County, 11 mi w of San Luis Obispo (in Pecho Hills); Pecho Hills, 6 Mar 1936, *Mason, H.L. s.n.* (UC); Ventura County, near Ojai, 19 Jun 1923, *Mason, H.L. 520* (UC); Santa Cruz County, mouth of Waddell Creek, Ano Nuevo Point Waddell Creek; Ano Nuevo Point, Waddell Creek, 4 Oct 1927, *Mason, H.L. 4124* (UC); Santa Barbara County, 1 mi s of Carpinteria (near sea), 27 Nov 1927, *Mason, H.L. 4153* (UC); Mendocino County, South Kibesillah Gulch angling access area on Highway 1, 16 Apr 1971, *Maze, J. 1060* (UBC); Harbison Canyon, 20 Apr 1950, *McCalla, W.C. 10758* (UBC); San Luis Obispo County, Hazard Canyon, 5 Mar 1936, *McMinn, H.E. 4358* (UC); San Diego County, Escondido, 1 Jul 1927, *Meyer, C.V. 252* (JEPS, UC); Santa Barbara County, Santa Barbara Ranger District. At pull off on Painted Cave Rd. about 0.5 mi. from CA Hwy 154, 26 Mar 2007, *Meyer, K.M. et al. 31* (MO); San Luis Obispo County, sin. loc, 1886, *Miles, M.M. s.n.* (GH); San Diego County, Mission Hills, 23 Nov 1919, *Millspaugh, C.F. 4433* (F); Los Angeles County, Santa Catalina Island, Avalon, 4 Dec 1919, *Millspaugh, C.F. 4476* (F); Los Angeles County, Santa Catalina Island, Avalon, beyond Golf Links, 2 Jan 1920, *Millspaugh, C.F. 4514* (F); Los Angeles County, Santa Catalina Island, Middle Ranch, 19 Jan 1920, *Millspaugh, C.F. 4606* (F); Los Angeles County, Santa Catalina Island, Rock Spring Canyon, 13 Feb 1920, *Millspaugh, C.F. 4707* (F); Los Angeles County, Avalon Canyon, 18 Feb 1920, *Millspaugh, C.F. 4719* (F); Los Angeles County, Whites Landing, Santa Catalina Island, 8 Mar 1941, *Moran, R. 688* (MO, SBBG); Los Angeles County, Whittier Hills, 20 Mar 1939, *Muenscher, W.C. & Muenscher, M.W. 14712* (A); Los Angeles County, San Clemente Island, 8 Apr 1923, *Munz, P.A. 6605* (GH, UC); Orange County, Costa Mesa, 29 Mar 1932, *Munz, P.A. 12190* (MO, UC); Monterey County, Carmel, May 1921, *Newlon, L.M. 100* (JEPS); Los Angeles County, just W of Malibu, 8 Sep 1948, *Nobs, M.A. & Smith, G. 647* (UC); Orange County, Baker Canyon, 6 Mar 1931, *Norton, H.E. 35* (UC); Los Angeles County, Santa Catalina Island, 1920, *Nuttall, L.W. 35* (BM, F, K); Los Angeles County, Santa Catalina Island, Avalon Valley, near Chicken Johnny's, 7 May 1920, *Nuttall, L.W. 139* (F); Los Angeles County, Santa Catalina Island, 13 May 1920, *Nuttall, L.W. 203* (BM, F, K); Los Angeles County, Santa Catalina Island, 10 Jun 1920, *Nuttall, L.W. 494* (BM, F, K); Los Angeles County, Santa Catalina Island, Hamilton Canyon, 17 May 1920, *Nuttall, L.W. 703* (F); Los Angeles County, Rancho Santa Ana Botanic Garden, also seen growing in wash SE of the Garden, 27 Oct 1968, *Oettinger, F.W. 831* (GH); Santa Clara County, Hecker Pass, 6 Oct 1940, *Paddock, E.F. 104* (UC); Ventura County, Point Mugu, U.S. Highway #101 Point Mugu, 17 Sep 1941, *Paddock, E.F. 141* (UC); Mendocino County, Rockport, Harding Creek Harding Creek; Rockport, Harding Creek, 13 Jul 1947, *Paddock, E.F. 165* (UC); Mendocino County, Mendocino-Sonoma County line, 14 Jul 1947, *Paddock, E.F. 169* (UC); San Luis Obispo County, Cambria, 20 Jul 1947, *Paddock, E.F. 170* (UC); San Luis Obispo County, San Luis Obispo Creek, where U.S. Highway 101 bridge crosses the creek at S border of city, 12 Jul 1951, *Paddock, E.F. 410* (SBBG, UC); Orange County, near San Diego County line (San Clemente State Beach, near picnic tables, near railroad tracks); San Clemente State Beach, 22 Jul 1951, *Paddock, E.F. 411* (UC); Santa Barbara County, Point 7 mi N of Buellton (7 mi SE of Los Alamos, and about 1 mi N of Los Olivos turnoff, on US 101), 3 Aug 1951, *Paddock, E.F. 413* (SBBG, UC); San Luis Obispo County, 1/2 mi N of Pismo Beach community (edge of US Hwy 101); Pismo Beach, 3 Aug 1951, *Paddock, E.F. 414* (UC); San Luis Obispo County, Cayucos, Moro [Morro] Rock, Moro Strand Beach State Park, 5 Aug 1951, *Paddock, E.F. 421* (SBBG, UC); Santa Barbara County, 0.1 mi n of Gaviota Post Office (0.3 mi s of Pass Historical marker, U.S. Highway 101,), 3 Aug 1951, *Paddock, E.F. 422* (UC); San Diego

County, southern part of San Diego Co, 1875, *Palmer, E. 261* (BM, MO, NY); Los Angeles County, Los Angeles, 12 Aug 1908, *Palmer, E.D. s.n.* (JEPS); Los Angeles County, Los Angeles, *Palmer, E.D. s.n.* (UC); San Diego County, Alnus forest along roadside on Rte S-6, 2.5 miles south off S-7, Palomar Mountain, 20 Jun 1984, *Parfitt, B.D. 3309* (DES); San Bernardino County, Sep 1887, *Parish, S.B. s.n.* (MO); San Bernardino County, vicinity of San Bernardino, Jul 1895, *Parish, S.B. s.n.* (JEPS); San Bernardino County, San Bernardino, May 1886, *Parish, S.B. & Parish, W.F. 321* (JEPS, MO, RB, UC); San Bernardino County, San Bernardino, 4 Oct 1903, *Parish, S.B. 5295* (F); 1886, *Pearce, W.A. s.n.* (BM); Los Angeles County, Rubio Canyon, San Gabriel Mountains, Rubio Canyon, 15 Oct 1918, *Peirson, F.W. 282* (JEPS); Santa Barbara County, ca. 1/2 mi. s.e. of summit Green Mt. (San Miguel Isl.), Channel Isl.'s, San Miguel Isl, 25 Jun 1964, *Philbrick, R.N. B64-267 b*-(JEPS); Monterey County, Gorda, Willow Creek, Santa Lucia Mtns, 5 Mar 1898, *Plackett, R.A. 40* (GH); Monterey County, Santa Lucia Mountains, Mar 1898, *Plaskett, R.A. 40* (UC); Ventura County, Foster Park, n face of Red Mountain Foster Park, Red Mountain, Foster Park, 6 May 1971, *Pollard, H.M. s.n.* (JEPS, RSA); Ventura County, Ojai, North Signal Street, 20 Oct 1944, *Pollard, H.M. s.n.* (GH); Monterey County, Big Creek Reserve, 7 Jul 1986, *Powell, J. 1494* (UC); San Mateo County, 2 miles S of Pigeon Point, 27 Jul 1933, *Purer, E.A. 5389* (GH); Los Angeles County, Point Dume, just E of Zuma Beach, 4 Apr 1959, *Raven, P.H. 13961* (GH); Los Angeles County, about 1.5 mi N of Thirst (W side of San Clemente Island), San Clemente Island, 10 Apr 1962, *Raven, P.H. 17248* (RSA, UC); San Bernardino County, near Fontana power Plant north of Rialto, 17 Sep 1961, *Rawen, P.H. 16674* (BM); Los Angeles County, San Rafael Hills, Angeles National Forest, San Rafael Hills, 3 Mar 1930, *Raymond, F.H. 52* (UC); San Luis Obispo County, Mouth of Coon Creek Coon Creek, 25 Sep 1930, *Raymond, F.H. 121* (UC); Riverside County, San Jacinto Valley, 1 Jun 1897, *Reinhardt, G.F. s.n.* (UC); Santa Barbara County, at Pt. Sal, 25 Jun 1972, *Rodin, R.J. 8737* (K); Monterey County, Monterey, 28 Jan 1935, *Rose, L.S. 35009* (LE, MO); Riverside County, Palm Springs, 26 Dec 1945, *Rose, L.S. 45326* (GH); Monterey County, Asilomar, 8 Jun 1963, *Rose, L.S. 63155* (NY); Los Angeles County, Whittier Hills (Puente Hills): Upper Turnbull Canyon ca 1/5 air-mile WNW of Turnbull Canyon Rd x Skyline Drive; T.2 S. R.11 W. SE 1/4 NW 1/4 sect 14, 23 Feb 1991, *Ross, T. 4110* (MO); Los Angeles County, Whittier Hills (Puente Hills): Upper Turnbull Canyon on slope dropping wnw from Turnbull Canyon Road x Skyline Drive Whittier Hills, Upper Turnbull Canyon, 23 Feb 1991, *Ross, T.S. 4090* (UC); Los Angeles County, elephant Hill, west Pomona elephant Hill, west Pomona, 3 Jun 1991, *Ross, T.S. & Ross, A. 5518* (UC); Los Angeles County, BROWN'S GULCH: (draining to San Gabriel Canyon at the base of San Gabriel Dam). T2N R9W, SW/4 NW/4 SW/4 section 31, 24 May 1992, *Ross, T.S. 6423* (BM, F, MO); Santa Barbara County, sin. loc, Jun 1874, *Rothrock, J.T. 83* (GH); San Diego County, West end of Otay Mesa, E of San Ysidro, Moody Canyon, *Sanders, A.C. et al. 7053* (DES); Monterey County, Big Sur Park, 29 Aug 1945, *Schallert, P.O. s.n.* (MO); Los Angeles County, Monrovia Canyon, 27 Apr 1944, *Schallert, P.O. s.n.* (MO); Los Angeles County, Monrovia Canyon, 27 Apr 1944, *Schallert, P.O. s.n.* (UC); Los Angeles County, Monrovia, 19 Apr 1943, *Schallert, P.O. s.n.* (NY); Marin County, West side of CA Stat Hwy 1, north of Muir Beach, south of border of Golden Gate National Recreation Area, 28 Mar 2014, *Schneider, A.C. & Fawcett, S. 445* (JEPS); Los Angeles County, elysian Park, 13 Mar 1901, *Setchell, W.A. s.n.* (UC); Los Angeles County, Claremont, Los Angles Co. Calif, 16 Mar 1936, *Shields, L. s.n.* (UT); Los Angeles County, Claremont, Los Angles Co. Calif, 30 Sep 1935, *Shields, L. s.n.* (UT); Kern County, Buena Vista Lake, 7 Jun 1934, *Short, L.R. & Johnson, I.H. S 281* (UC); Ventura County, .8 mi se of Devils Gateway (on Agua Blanca Creek), Santa Barbara National Forest, Agua Blanca Creek, 4 Jun 1935, *Simontacchi, A. 71* (UC); Monterey County, Pacific Grove, S.P. Railroad, above the depot, 25 Jun 1905, *Smith, C.P. 979* (MICH); Los Angeles County, Oak Knoll, 7 Mar 1912, *Smith, H.H. 4382* (F); Los Angeles County, eagle Rock Valley, 1 May 1912, *Smith, H.H. 4885* (UC); Los Angeles County, Santa Catalina Island, Avalon Canyon Avalon Canyon, Santa Catalina Island, Avalon Canyon, 26 May 1912, *Smith, H.H. 5005* (UC); San Diego County, San Diego, 31 May 1897, *Snyder, M.S. s.n.* (F); Ventura County, Head Canada de San Joaquin Canada de San Joaquin, 24 Feb 1934, *Sowder, J.E. 185* (UC); Riverside County, Saboba Hot Springs, San Jacinto, Riverside Co, 16 Jul 1921, *Spencer, M.F. 1724* (GH, K, MICH); Monterey County, Little Sur, 30 May 1926, *Stason, M. s.n.* (UC); Santa Barbara County, Santa Barbara, 1 Jan 1939, *Stebbins, G.L. 41* (UC); San Mateo County, San Gregorio, 17 Aug 1939, *Stebbins, G.L. & Paddock, E.F. 63* (GH, UC); Los Angeles County, Whittier, 23 Dec 1939, *Stebbins, G.L. & Paddock, E.F. 80* (UBC); Santa Clara County,

Hecker Pass, 6 Oct 1940, *Stebbins, G.L. & Paddock, E. 106* (GH); Monterey County, Pacific Grove, 12 Oct 1940, *Stebbins, G.L. & Paddock, E. 107* (GH); Santa Barbara County, Buellton, 16 Jun 1941, *Stebbins, G.L. & Paddock, E.F. 133* (UC); San Diego County, Camp Pendleton Marine Corps Base, off Stuart Mesa Road on floodplain of San Luis river, 7 Apr 2000, *Stone, J. & Bodine, S. 2891* (MO); San Luis Obispo County, S.L.O. County, Sep 1886, *Summers, R.W. s.n.* (K); San Luis Obispo County, sin. loc, 1 Sep 1886, *Summers, R.W. s.n.* (UC); San Luis Obispo County, *Summers, R.W. 607* (K); Los Angeles County, San Gabriel Mountains: Arcadia Wilderness Park, north area of park, 2240 Highland Oaks Dr. lower Santa Anita Cyn. (Mt. Wilson 7.5' Q.) (T1N R11W S15), 15 Mar 2011, *Swinney, R.G. 14082* (ARIZ); Los Angeles County, San Gabriel Mountains: Islip Canyon, tributary of San Gabriel Cyn. from Hwy 39 to 0.4 mile upstream, NE of Morris Reservoir (Glendora 7.5' Q.) (T1N R9W S6), 26 Apr 2011, *Swinney, R.G. 14484* (ARIZ); Santa Cruz County, coastal bluff 0.2 miles N of Greyhound Rock (near Highway 1 and N intersection with Swanton Road), 30 Mar 1989, *Taylor, D.W. 10194* (JEPS); Santa Cruz County, on steep bank s Soquel Drive (e of Soquel Creek), Santa Cruz Mountains, Aptos, 11 Dec 1994, *Taylor, D.W. 14733* (JEPS); Monterey County, ca. 10-50 yds S of Carmel River, ca. 1-2 ft E of Hwy 1, Carmel, 30 May 1975, *Taylor, M.S. 627* (MO); San Benito County, On S side of Hwy 129, ca. 5 mi E of Watsonville, 27 Mar 1977, *Taylor, M.S. & Pierce, E. 1304* (MO); San Mateo County, White House Road area, 30 Aug 1978, *Taylor, M.S. 1860* (MO); Ventura County, Ojai Valley, 30 Aug 1915, *Thacher, O. 45* (JEPS); Los Angeles County, near mouth of Cobal Canyon, foothills of San Gabriel Mts. ca. 4 miles N of Claremonth, 5 May 1963, *Thorne, R.F. & Henrickson, J. 32284* (BM); Los Angeles County, Santa Catalina Island, Hay Press area Santa Catalina Island, Hay Press area, 28 Apr 1966, *Thorne, R.F. 36247* (GH, UC); Los Angeles County, San Clemente island, middle portion of Norton Canyon, 11 Apr 1973, *Thorne, R.F. 42847* (MO); sin. loc, 1852, *Thurber, G. 577* (F, GH, NY); Los Angeles County, W side of Colima Ave, just N of Punta del Este Road, between Whittier and West Covina, 2 Apr 1969, *Tilforth, C.W. 180* (A); Santa Barbara County, sin. loc, 1865, *Torrey, J. 353* (GH); Los Angeles County, San Clemente island, May 1903, *Trask, B. 238* (GH); Riverside County, Box Springs, 1 Apr 1935, *True, G.H. 195* (UC); San Mateo County, N of Sharpe Park, 23 Jul 1937, *True, G.H. 806* (UC); Santa Cruz County, Santa Cruz Mountain Unit, 30 Aug 1928, *Tyrell, T. & Clar, C.R. 2* (UC); Colton, Feb 1881, *Vasey, G.R. 383* (BM, K); San Bernardino County, San Bernardino, May 1880, *Vasey, G.R. 442* (NY); Los Angeles County, Rancho Santa Ana Botanic Garden, Claremont; San Gabriel Mt Range, Pacific slope drainage area, 5 Mar 1961, *Venrick, B. 480* (MO); San Diego County, San Diego, 1908, *Westerlind, S. s.n.* (H); Los Angeles County, San Gabriel Mountains: Morgan Canyon at Ferguson Motorway; T1N R9W NW1/4 sec. 27, 17 Mar 1968, *Wheeler, L.C. s.n.* (MO); Los Angeles County, foothills of San Gabriel Mts. West Debris Basin above Brown School for Girls, 21 Mar 1968, *Wheeler, L.C. s.n.* (F); Los Angeles County, San Gabriel Mts. Morgan Canyon at Ferguson Motorway, 17 Mar 1968, *Wheeler, L.C. s.n.* (F); San Bernardino County, San Gabriel Mts. Lytle Creek Village, 4 Nov 1967, *Wheeler, L.C. s.n.* (F); Los Angeles County, Thompson Creek Wash, 30 Nov 1929, *Wheeler, W.H. s.n.* (K); San Diego County, 1.25 mi w of Campo, 30 Dec 1922, *Wiggins, I.L. 1058* (UC); San Diego County, 6 mi below Alpine (on highway); Cuyamaca Mountains Region, 20 Mar 1926, *Wiggins, I.L. 2053* (UC); San Diego County, 3 mi below Dulzura (above bridge across Dulzura Creek); Dulzura Creek, 10 Apr 1927, *Wiggins, I.L. 2218* (UC); San Diego County, 7 mi above Lakeside (on Mussey Grade); Mussey Grade, 19 May 1927, *Wiggins, I.L. 2527* (UC); San Diego County, 7 mi above Lakeside (on Mussey Grade); Mussey Grade, 19 May 1927, *Wiggins, I.L. 2527* (UC); San Diego County, 4 mi e of Ramona (on Highway to Santa Ysabel, along Hatfield Creek); Hatfield Creek, 29 Aug 1927, *Wiggins, I.L. 2606* (UC); San Diego County, San Mateo River, near the ocean San Mateo River, 12 Jun 1928, *Wiggins, I.L. 2969* (UC); San Diego County, San Mateo River, near ocean San Mateo River, 12 Jun 1928, *Wiggins, I.L. 3002* (POM, UC); San Luis Obispo County, Morro Bay, 27 Jun 1933, *Winblad, Y.W. s.n.* (UC); 1867, *Without Collector s.n.* (K); Los Angeles County, "Hollywood Cal.", *Without Collector s.n.* (UT); Santa Barbara County, entrance to Romero Canyon, 20 Feb 1997, *Without Collector s.n.* (F); Santa Barbara County, Santa Barbara, *Without Collector s.n.* (UC); San Diego County, between National City and Chula Vista, 26 May 1931, *Wolf, C.B. 2087* (RSA, UC); Monterey County, Monterey Peninsula, Asilomar Dunes, 13 Jul 1946, *Wood, R.D. s.n.* (F); Ventura County, beside bridge over Santa Clara River, at S edge of Santa Paula, 15 Jul 1951, *Wootton, D.M. 412* (UC); California australis, 1841, *Wosnessensky, ?, 58 bis* (LE); San Mateo County, 1/2 mi se of Pigeon Point, 3 Feb 1935, *Yates, H.S. 5007* (UC); sin. loc, Jun 1931, *Yuncker, T.G. & Welch, W.H.*

3918 (NY); Los Angeles County, Azusa, 21 Jun 1931, *Yuncker, T.G. & Welch, W.H. 3998* (NY). **New Mexico**: Chaves County, 43 mi SE of Cloudcroft, 1966, *Sikes, S. 71* (MO); Hidalgo County, In the extreme SW corner of the county, in Guadalupe Canyon and tributaries, on and in vicinity of Hadley Ranch; in the narrow, steep-walled Guadalupe Canyon, 14 Aug 1979, *Spellenberg, R.W. 5276* (NY); Dona Ana County, Mesilla valley, Jul 1906, *Standley, P.C. s.n.* (MO); Mimbre Mts, 26 Jun 1936, *Stewart, W.M. s.n.* (MO). **Texas**: Jeff Davis County, Limpia Canyon, 10 miles NE of Fort Davis, 20 Aug 1946, *Correll, D.S. 14052* (LL); Jeff Davis County, On upper slopes and on summit of Sawtooth Mt. Davis Mts, 11 Sep 1967, *Correll, D.S. 34986* (LL); Brewster County, Window Trail, Chisos Mountains, 7 Jul 1944, *Cory, V.L. 44760* (GH); Jeff Davis County, Fort Davis, 19 Sep 1920, *Eggleston, W.W. 17396* (NY); Jeff Davis County, Mt. Livermore, 22 Sep 1934, *Hinckley, L.C. 51* (TEX); Jeff Davis County, Mount Livermore, Davis Mtns. TransPecos Texas, 21 Aug 1935, *Hinckley, L.C. 168* (F, NY); Jeff Davis County, Mt. Livermore, 6 Oct 1934, *Hinckley, L.C. 991* (TEX); Jeff Davis County, Northeastern Davis Mtns. 11 miles W of Hwy. 17, FM 1832, Nations Ranch, dirt roadside, N-facing slope of Pig Pen Canyon. Elev. 5200 ft, 18 May 1985, *Larke, J. et al. 131* (TEX); Brewster County, Oak Canyon, 6 Jul 1937, *Marsh, E.G. 85* (F, GH); Brewster County, Green Gulch, 18 Jul 1932, *Mueller, C.H. s.n.* (TEX); Jeff Davis County, Davis Mountains, upper Limpia Creek, 11 Jun 1926, *Palmer, E.J. 30751* (MO, TEX); Jeff Davis County, Timber Mt. W of Balmorhea, 23 Jun 1977, *Powell, A.M. 3173* (TEX); Jeff Davis County, Near Mt. Livermore peak, ridges SE of the peak, 17 Sep 1977, *Powell, A.M. et al. 3196* (LL); Jeff Davis County, Ca. 1 mile E of Boy Scout Ranch, FM 1832 in small draw alongside road. Corollas white, 1 Jul 1967, *Sikes, S.W. 345* (TEX); Brewster County, Sunny Glenn near Alpine, 29 Sep 1935, *Warnock, B.H. s.n.* (TEX); Brewster County, South slopes of Casa Grande, Chisos Mts., 15 Jun 1937, *Warnock, B.H. 848* (TEX); Jeff Davis County, Limpia Canyon, 1 mile above Ft. Davis., 28 Jul 1947, *Warnock, B.H. 6584* (TEX); Jeff Davis County, Madera Canyon, Mt. Livermore, Davis Mountains., 13 Sep 1947, *Warnock, B.H. 7498* (TEX); Jeff Davis County, At head of HO Canyon near Sawtooth on Scenic Loop, Davis Mountains. Elev. 5100 ft, 26 Jul 1952, *Warnock, B.H. 10890* (LL); Brewster County, Along trail from Boot Spring & Basin, Chisos Mts. North side of Emory Peak, Big Bend National Park. Elev. 6500 ft, 15 Jul 1955, *Warnock, B.H. 12727* (LL); Jeff Davis County, Along drive from entrance to lodge at Davis Mountains State Park at an. Elev. 5000 ft, 15 Jul 1967, *Warnock, B.H. 22370* (TEX); Jeff Davis County, Davis Mts, 21 Aug 1914, *Young, M.S. s.n.* (TEX); Jeff Davis County, Davis Mts, 9 Sep 1918, *Young, M.S. s.n.* (TEX).

5. *Solanum emulans* Raf.
CANADA. **British Columbia**: Huntingdon, 22 Sep 1957, *Cowan, I.M. 3* (UBC); Vancouver, Stanley Park, 30 m W of Lost Lagoon footbridge, Ceperley Meadow, 10 Sep 2007, *Lomer, F. 6418* (UBC); Vancouver, Claredon Street, W side, S of 33rd Avenue, 11 Aug 2014, *Lomer, F. 8870* (UBC); Ccentral Bowen Island, 16 Aug 1978, *Small, M. s.n.* (UBC). **Manitoba**: Otterburne, aux abords de la pepinière de la Maison Saint-Joseph, 13 Aug 1954, *Bernard, J.-.P. 54-506* (MT); The Pas, 6 Sep 1959, *Krivda, W. 2185* (MT). **Ontario**: 3.8 miles NW of Harrow, Lot 3, Conc. 3, Colchester S. Twp. Essex County, farm of Kimball, 28 Jul 1961, *Alex, J.F. 725* (MT); Bruce Co. Bruce Twp, 26 Jul 1965, *Ball, M.F. s.n.* (MT); Simcoe County, Conc. Xiv lot 3, Tap tp, 29 Jul 1972, *Bobbette, R.S.W. 1973* (MICH); Simcoe County, Conc. Ix lot 25, Flos tp, 15 Jul 1972, *Bobbette, R.S.W. 1989* (MICH); Simcoe County, 26 Jul 1972, *Bobbette, R.S.W. 2089* (MICH); Casselman, canton de Cambridge, comté de Russel, Chemin d'Embrun, 30 Aug 1981, *Brisson, S. 81342* (MT); Toronto, Ashbridge's Bay, 9 Sep 1930, *Brown, H.H. 1253* (UBC); Pottageville, York County, 21 Jul 1934, *Brown, H.H. 4207* (UBC); Toronto, S end of Spadina Avenue, car park weed in city, 14 Oct 1996, *Brummit, R.K. 19533* (K); St. Andrew's, [locality inferred], Sep, *Campbell, R. s.n.* (UC); Lefauré [Lefaivre], près crique Poliquin, 7 Jul 1961, *Charlebois, J.E. 464* (MT); Kars, 5 Aug 1932, *Charles*, *1932* (MT); Hawkesbury, comté de Prescott, 20 Sep 1926, *Cléonique-Joseph*, *12618* (MT); Leeds County, Oliver's Ferry, 17 Aug 1898, *Edmondson, T.W. s.n.* (NY); Plevna, 8 Aug 1902, *Fowler, J. s.n.* (GH); S of Otter Lake, Kingston, 29 Jul 1965, *Garwood, A.E. 1321* (MT); shore of northern Lake Huron, Cockburn Island, 12 Aug 1932, *Grassl, C.O. 5612* (MICH); Duck Islands, Main Duck Island, 1 Aug 1964, *Hainault, R. 3501* (MT); York County, Metropolitan Toronto, gardens in front of Trinity College, York Co. Toronto, 3 Jul 1974, *Hoy, D. & Gad, L.M. s.n.* (MICH); 3 miles S of St Thomas, Elgin County, 3 Sep 1952, *James, L.E. 1253* (MT); Elgin County, 5 miles S of St. Thomas, 17 Jul

1952, *James, L.E. 1929* (MT, W); Elgin County, 3 miles S of St. Thomas, 3 Sep 1952, *James, L.E. 1930* (MT, W); Stokes Bay, Bruce Peninsula, 27 Jul 1934, *Krotkov, P. 9376* (NY, US); Tobermory, Bruce County, Bruce Peninsula, 25 Jul 1933, *Krotkov, P.V. 7759* (GH); Cedar Island, 7 Jun 1965, *Leach, E. 350* (UC); Avonmore, 19 Aug 1895, *Lemon, J.H. 1608* (UBC); Fort Malden, Canada West [Amherstburg], 12 Sep 1848, *Maclagan, R. s.n.* (E); Ottawa, 15 Aug 1894, *Macoun, J. s.n.* (US); Ottawa, Oct 1883, *Macoun, J. 1608* (BM); Lake Region and Ontario, 16 Aug 1877, *Macoun, M.A. 1181* (K); Prescott, Alfred, 7 Apr 1935, *Major-Barnabé*, *3942* (MT); German Mills, Cressman's Woods, Waterloo County, 10 Sep 1939, *Montgomery, F.H. 337* (GH); Papoose Island, off Killarney, Georgian Bay, 12 Oct 1975, *Morton, J.K. & Venn, J.M. NA-8684* (MT); Norfolk Tp. Long Point, Hasting's Drive; Haldimand-Norfolk Regional Municipality, 18 Sep 1987, *Oldham, M.J. 7928* (MICH); Rainy River County, Atwood Township, ca. 2 km upriver from mouth of Rainy River, near Oak Grove camp, ca. 14 km NW of Rainy River Post Office, 8 Aug 1995, *Oldham, M.J. & Bakowsky, W. 17976* (MICH); disturbed area W of QEW highway, on Hamilton Harbour, Hamilton-Wentworth Regional Municipality, 1 Oct 1995, *Oldham, M.J. 18341* (MICH); Peterborough County, Belmont Township, Havelock Sewage lagoon, 1.3 km SE of Hevelock Post Office, 3 Sep 1998, *Oldham, M.J. 21325* (MICH); Vineland, Niagara Region, Horticultural Research Institute, 15 Jul 1977, *Pérusse, Y. 77400* (MT); Simcoe County, 30 Aug 1969, *Reznicek, A. 1141* (MICH); Norfolk County, Long Point, just E of Cedar Creek, about 12.5 miles from base of point, 2 Aug 1980, *Reznicek, A.A. et al. 6030* (MICH); vallee de l'Ottawa, 20 Aug 1921, *Rolland-Germain*, *236* (MT); Toronto County, Toronto downtown waterfront, portlands; W side of Cherry Street, 2 Oct 2005, *Rothfels, C.J. & Johnson, M.T. 2046* (MICH); Lake Huron, *Todd, C.C. s.n.* (K); Galt, Waterloo Regional Municipality [Galt now dissolved and amalgamated with others to become Cambridge], 16 Aug 1899, *Umbach, L.M. 10656* (WIS); St. Williams For[est] Sta[tion], Norfolk County, 19 Sep 1970, *Wellwood, A.A. 1993* (MT); Lake Rosseau, rocky ground above the lake, 1 Aug 1904, *Wright, W.F. 129* (US). **Quebec**: St-Bonaventure, 25 Sep 1997, *[SG]*, *25* (MT); Mont la Mennais, Oka, Deux Montagnes, 4 Sep 1951, *Arsène-Marie*, *s.n.* (MT); Île Perrot, 29 Aug 1966, *Bernard, G. 66661* (MT); Montréal, co. Ahuntsic, blvd Gouin, cote nord, 21 Sep 1973, *Bernard, G. 73111* (MT); St. Antoine-Abbé, co. Huntingdon, 20 Aug 1977, *Bernard, G. 77068* (MT); Longueuil; parc régional de Longueuil, 2 Aug 1996, *Bernard, J.-.P. B96-302* (MT); Montréal, Arrondissement Ville-Marie, Quartier des Spectacles, rue Clark à ca. 5 m du coin avec Ontario, le long du mur du 51 Ontario ouest près de son extrémité nord, 1 Nov 2015, *Brouillet, L. 2015-16* (MT); Beauharnois, Bois Robert, sur le sentier principal au sud de la halte "3", 19 Aug 1998, *Charest, R. & Drouin, J.N. 603* (MT); St. Gregoire, Co. Iberville, autour de la cabane a sucre Gouaneau, 13 Sep 1957, *Cinq-Mars, L. s.n.* (MT); St. Gregoire, Co. Iberville, autour de la cabane a sucre Gouaneau, 13 Sep 1957, *Cinq-Mars, L. s.n.* (MT); Riv Rich Riv Oueste L 7, 30 Jul 1974, *Clément, A.-.M. 741438* (MT); Mont Royal, 19 Aug 1932, *Dansereau, A. 954* (MT); Rigaud ?? M.R.C. Vaudreuil-Soulanges, 7 Aug 1940, *Gaboriault, V. s.n.* (MT); Montebello, 21 Sep 1958, *Hainault, R. & Charlebois, J.E. 770* (MT); Île des Soeurs, rivage de la grande baie, 3 Sep 1967, *Hébert, L.-.P. s.n.* (MT); Rivière des Prairies, tronçon 14, île en aval de la précédente, 8 Oct 1975, *Lagacé, M. & Brouillet, L. 751008-0757* (MT); sin. loc, 24 Jul 1942, *Lamarre, G. s.n.* (UBC); Île Perrot, Pincourt, entre la pointe aux Roches et le pont (sta. 60), 31 Aug 1965, *Lévesque, L. & Pageau, G. 650831-2569* (MT); Longueuil, 16 Sep 1933, *Lorenzo*, *s.n.* (MT); St. Placide, MRC Deux-Montagnes, au 1411, rt. 344, 8 Aug 1952, *Louis-Alphonse*, *1175* (MT); Baie Missisquoi, 15 Aug 1952, *Louis-Alphonse*, *1429* (MT); Baie Missisquoi, près de la frontiere, 9 Aug 1953, *Louis-Alphonse*, *3560* (MT); Baie Missisquoi, 16 Aug 1953, *Louis-Alphonse*, *3631* (MT); environs de La Trappe, Comté des Deus-Montagnes, 24 Jul 1942, *Louis-Marie, P. s.n.* (UC); Oka, P.A, Aug 1912, *Marie-Victorin*, *3895* (MT); Longueuil, *Marie-Victorin*, *et al. 3942* (MT); Ironside, vallee de rivière Gatineau, 20 Aug 1921, *Marie-Victorin*, *& Rolland-Germain 15677* (MT); Aylmer, près de Hull sur la rivière Ottawa, 25 Aug 1921, *Marie-Victorin*, *& Rolland-Germain 15678* (MT); Saint-Bruno, comté de Chambly, 16 Sep 1933, *Marie-Victorin*, *& Rolland-Germain 45753* (MT); Saint-Bruno, comté de Chambly, 10 Aug 1938, *Morin-Gauthier, C. s.n.* (MT); Ste-Anne des Monts, près du couvent, 25 Jul 1962, *Racine, M. s.n.* (MT); Longueuil (Chemin du lac), 14 Sep 1935, *Racine, M. s.n.* (MT); Île au Diable, iles des rapides de Lachine, comté de Verdun, 30 Aug 1977, *Ranger, J.L. 907* (MT); St-Armand, comté de Missisquoi, 1 Sep 1952, *Raymond, M. & Cinq-Mars, L. s.n.* (MT); St-Armand, comté de Missisquoi, 1939, *Raymond, M. 667* (MT); Saint-Armand, comté de Missisquoi, 17 Sep 1942, *Raymond, M. 7030* (MT); Rigaud, Co. Yaudreuil, 17 Aug 1934, *Robert, A. 1353* (MT);

Chambly, Aug 1914, *Rolland-Germain*, *558* (MT); Montréal, terrain de l'Université de Montréal entre le métro Université et la résidence des filles à 9 m d'un carré de béton ancré à 6m du sentier rouge, 22 Aug 1999, *Roy, C. 3* (MT); Vaudreuil-Soulanges County, Rigaud, 24 Jul 1935, *Roy, E. 3841* (MICH); Ironsides, 8 Jul 1891, *Scott, W. s.n.* (BM, NY); ile de Monl [Montréal], Jul 1890, *Soeur Marie de Sainte-Amélie*, *s.n.* (MT); ile de Montréal, Jul 1887, *Soeurs de Sainte-Croix*, *s.n.* (MT); Ironside, vallee de rivière Gatineau, 7 Oct 1946, *Verret, L. s.n.* (MT); Reserve Indienne de Caughnawaga [Kahnawake], section au nord de la voie maritime du Saint-Laurent, comté de Laprairie, 21 Aug 1978, *Vincent, G. 78-852* (MT). **Saskatchewan**: Saskatoon, 519 Walmer Road, 29 Jul 1941, *Child, E.M. s.n.* (SASK).

UNITED STATES OF AMERICA. Sin. loc., 25 Sep 1898, *Bicknell, E.P. 7711* (NY); Fort Smith to the Rio Grande [prob. Texas Oklahoma border along 35th parallel, 3 Aug 1853, *Bigelow, J.M. s.n.* (NY, US); *Drummond, T. s.n.* (K); Nov. Ebor, 1839, *Gray, A. s.n.* (K); "M.A.C.", 22 Sep 1904, *Loen, F.A. s.n.* (UC); 'Amer. bor.', *Rafinesque, C.S. s.n.* (W); "Fl. Bor. Am." -sin. loc, *Without Collector s.n.* (NY); "ex herbario Collegi Pharmaciae Neo-Eboracensis", *Without Collector s.n.* (NY); "herbario Collegi Pharmaciae Neo-Eboracensis", *Without Collector s.n.* (NY); "herbario Collegi Pharmaciae Neo-Eboracensis", *Without Collector s.n.* (NY); "herbario Collegi Pharmaciae Neo-Eboracensis", *Without Collector s.n.* (NY); sin. loc, *Without Collector s.n.* (NY). **Alabama**: St. Clair County, Chandler Mountain, above and to SE of Camp Sumtanga, at overlook at around Creel Chapel, 31 Aug 1999, *Bryson, C.T. et al. 17674* (NY); Henry County, AL 10 @ W side of Chattahoochee River bank, 24 Jul 2008, *Dyess, K.K. & Diamond, A. 23* (UNA); Lee County, Auburn, 15 Aug 1897, *Earle, F.S. & Baker, C.F. 958* (NY); Escambia County, Solon Dixon Forestry Education Center, 1.8 miles N from center offices on Center Rd at recent tornado blow down areas; Auburn University, 14 Aug 2008, *Hansen, C.J. & Goertzen, L.R. 3432* (W); Tuscaloosa County, 0.5 mi SSE of Holt. Along rail bed just SE of Holt school, 8 Aug 2002, *Keener, B.R. 2347* (UNA); Lowndes County, by Ala 41, 19 mi. SSW Sardis, 7 Oct 1971, *Kral, R. 44963* (MO); Baldwin County, N side of Gulf Shores, just N of Intercoastal Waterway, 14 Aug 1973, *Kral, R. 51310* (MO); Autauga County, Prattville, Jul 1874, *Mohr, C. s.n.* (US); sin. loc, *Winchell, A. 368* (US). **Arkansas**: Craighead County, Lake City, 27 Jun 1927, *Demaree, D. 3427* (MO); Pulaski County, Natural Steps, 15 Oct 1931, *Demaree, D. 8571* (BUT, NY, US); Pulaski County, Natural Steps, 21 Oct 1931, *Demaree, D. 8738* (BUT, NY); Johnson County, Shade in Ark[ansas] river bottoms at Pine Bluff, *Demaree, D. 8789* (US); Chicot County, Lake Village, Greenville Ferry, banks of Saline River, 31 Oct 1936, *Demaree, D. 14077* (NY, US); Logan County, Jacks Creek Camp, P.O.Strictland, 25 Aug 1937, *Demaree, D. 16032* (MO, NY); Hot Springs County, Magnet Cove, 10 Oct 1937, *Demaree, D. 16455* (NY); Yell County, Ola, 30 Aug 1938, *Demaree, D. 18217* (NY); Yell County, Dardanelle, bench side of Mount Nebo, 16 Aug 1939, *Demaree, D. 20087* (MIN, NY); Clay County, Corning, 25 Aug 1939, *Demaree, D. 20323* (NY); Clay County, Corning, 25 Aug 1939, *Demaree, D. 20327* (F, NY); Drew County, Monticello, 9 Oct 1945, *Demaree, D. 25558* (GH); Garland County, above Hot Water Supply Reservoir at side of paved road on Hot Springs Mt. in Hot Springs National Park, *Gregg, H.R. 217* (US); Saline County, N shore of Lake Norrell, ca 12 mi NNW of Benton; 34.725733 -92.648336, 14 Sep 2001, *Sundell, E. et al. 15* (MISSA); Union County, along dirt road 2.5 miles north of Aurelle, sec. 14, T19S, R13W, 10 May 1990, *Thomas, R.D. 116082* (MO); Chicot County, Along a hunting club road between levee and Mississippi River two miles south of end of Ark, 142 South of US82, and easte of Lake Village, Sec. 21, 30 Sep 1994, *Thomas, R.D. et al. 142721* (MO, NY); Desha County, on the S bank of the Arkansas River beside Ark 212 at Pendelton Recreation Area about 1 mile E of Ark 1 N of Back Gate (Sec. 27), 8 Aug 1995, *Thomas, R.D. et al. 145394* (NY); Howard County, railroad tracks and Ark 27 at US 371 and Mine Creek in Nashville, 26 May 1999, *Thomas, R.D. & Amason, C. 160424* (NY); Nevada County, Ark 32 about 0.5 miles east of the Hempstead County Line W of Bodcaw, Sec. 34, T13S, R23W, 22 May 2001, *Thomas, R.D. & Amason, C. 169908* (MO, NY); Saline County, norhern tip of eastern prong of Lake Norrell SE of Paron and NNW of Benton (Sec. 7), 14 Sep 2001, *Thomas, R.D. et al. 171536* (MO, NY); Sebastian County, Arkansas River floodplain, Sec 26, T8N, R31W, 4 Sep 1989, *Thompson, R.A. C 1060* (MO); Faulkner County, Conway, 5 Jul 1934, *Welch, W.H. 1266* (NY). **Colorado**: Fremont County, Grape Creek, 2 miles SE of Cañon City, 2 Sep 1924, *Bacigalupi, R. 1003* (UC); Fremont County, Canon City, Rocky Mts, 1871, *Brandegee, T.S. 5873* (MO); Las Animas County, S slope of Tecolote Mesa, near Troy, region of Mesa de Maya, 1 Jul 1947, *Rogers,*

C.M. 4485 (MICH, US); Fremont County, Cañon City, 8 Aug 1896, *Shear, C.L. 3777* (NY). **Connecticut**: Hartford County, Southington, Sep, *Andrews, L. s.n.* (NEBC); New London County, Ledyard, 22 Jul 1923, *Avery, A.G. s.n.* (CONN); Hartford County, Wethersfield, Folly Brook Natural Area, 7 Sep 1977, *Barringer, K. 630* (CONN); New Haven County, New Haven, 19 Aug 1906, *Bartlett, A.I. s.n.* (NCBS); New Haven County, New Haven, Aug, *Bartlett, A.J. s.n.* (NEBC); New Haven County, Guilford, 14 Aug 1903, *Bartlett, G.H. 316* (NCBS); Hartford County, East Windsor, Jul, *Bissell, C.H. 1* (NEBC); Hartford County, East Windsor, Jul, *Bissell, C.H. 883* (GH); Hartford County, East Windsor, bank of Connecticut River, 28 Jul 1899, *Bissell, C.H. 5153* (NCBS); Hartford County, Southington, Liberty Street, 29 Jul 1891, *Bissell, C.H. 5154* (NCBS); Hartford County, Hartford, 6 Sep 1903, *Bissell, C.H. 8911* (NCBS); New Haven County, Waterbury, 5 Oct 1909, *Blewitt, A.E. 2799* (NCBS); Harford County, Berlin, *Brandegee, M. s.n.* (UC); New Haven County, New Haven, yard of the Yale Analytical Lab, *Brewer, W.H. s.n.* (YU); Middlesex County, Chester, 25 Sep 1932, *Burton, R.H. s.n.* (YU); Hartford County, Hartford, South Meadow, 4 Oct 1908, *Clark, H.S. s.n.* (YU); Fairfield County, Wilton, roadside off Oak Ledge Road, Aug 1970, *Conservation Committee*, *s.n.* (WGCH); Hartford County, South Manchester, *Cook, A.B. s.n.* (CONN); Fairfield County, Stratford, Aug 1872, *Day, W.F. s.n.* (NY); New Haven County, New Haven, 1914, *Denslow, W.W. s.n.* (NY); Fairfield County, Bridgeport, Aug, *Eames, E.H. s.n.* (GH); Fairfield County, Bridgeport, Aug, *Eames, E.H. s.n.* (NEBC); New Haven County, Milford, 5 Sep 1940, *Eames, E.H. s.n.* (CONN); Fairfield County, Bridgeport, 13 Aug 1893, *Eames, E.H. 3123* (CONN); Litchfield County, Castle Rock, Woodbury, 29 Sep 1905, *Eames, E.H. & Godfrey, C.C. 5399* (CONN, YU); Litchfield County, Washington, 7 Sep 1921, *Evans, A.W. s.n.* (YU); New Haven County, New Haven, Wasteground -Boulevard, 1 Aug 1920, *Evans, A.W. s.n.* (YU); New Haven County, New Haven, Wasteground -Boulevard, 1 Aug 1920, *Evans, A.W. s.n.* (YU); New Haven County, East Haven, 15 Sep 1888, *Evans, A.W. s.n.* (YU); New London County, New London, Connecticut arboretum, vernal pools, 23 Sep 1987, *Evans, T.A. 162-87/ 11* (CCNL); Windham County, Woodstock Valley, 6 Sep 1997, *Flanagan, M. 8* (CONN); Middlesex County, East Haddam, Burnham Brook Preserve of The Nature Conservancy, Dolbie Hill Farm, 12 Aug 1984, *Goodwin, R.H. 84-342* (CCNL); Hartford County, East Hartford, 12 Jul 1905, *Hanmer, C.C. s.n.* (CONN); Hartford County, South Windsor, 16 Sep 1906, *Hanmer, C.C. s.n.* (CONN); New Haven County, Oxford, 19 Sep 1894, *Harger, E.B. s.n.* (NCBS); New Haven County, Oxford, 20 Aug 1879, *Harger, E.B. WES-467* (US); New Haven County, Oxford, Oct, *Harger, E.B. 6903* (NEBC); New Haven County, New Haven, 26 Jul 1873, *Hawes, G.W. s.n.* (YU); Hartford County, Windsor Locks, 6 Aug 1877, *Hayden, N.W. 105* (VT); New London County, Stonington, Lyddy's Island off Latimer's Point, 4 Sep 1972, *Hill, S.R. 945* (ECON, NY, VT); New London County, old cemetery just W of W town line of Mystic, Groton Rte 1, 16 Aug 1986, *Hill, S.R. 17081* (GH, MO); Hartford County, Southington, 7 Oct 1973, *Hurie, E.T. 60* (CONN); New London County, New London, Connecticut College, 9 Jul 1933, *Jansson, K.P. s.n.* (CONN); New London County, Groton, 10 Sep 1930, *Jansson, K.P. s.n.* (CONN); Tolland County, Mansfield, Storrs, University of Connecticut, 21 Sep 1989, *Kusmin, J.D. 9* (CONN); Tolland County, Tolland, 6 Aug 1990, *Kusmin, J.D. 98* (CONN); New Haven County, Southbury, Platt Park, 21 Aug 1995, *McCauley, K.M. 616* (NHA); Tolland County, Mansfield, Spring Manor Farm, next to barn, 31 Oct 1973, *Mehrhoff, L.J. 912* (NHA); Fairfield County, Norwalk, Chimon Island, Stewart B. McKinney National Wildlife Refuge, 14 Jul 1981, *Mehrhoff, L.J. 4298* (CONN); Fairfield County, Danbury, WCSU Westside Campus, 17 Jul 1995, *Moran, M.A. 142* (WCSU); Fairfield County, Danbury, WCSU Westside Campus, 17 Jul 1995, *Moran, M.A. 143* (WCSU); Fairfield County, Stamford, Bartlett Arboretum Forest, 11 Jul 2007, *Morgan, E.C. s.n.* (BART); New Haven County, Southbury, Housatonic River, 6 Sep 1941, *Neale, J.J. 3878* (CONN); Litchfield County, Woodbury, Parker Farm, Old Flanders Road, 14 Aug 1977, *Parker, R. & Parker, S. 77-64* (CONN); Litchfield County, Woodbury, Parker Farm, Old Flanders Road, 5 Jul 1969, *Parker, R. & Parker, S. 69-180* (CONN); Litchfield County, Woodbury, Parker Farm, Old Flanders Road, 6 Aug 1969, *Parker, R. & Parker, S. 69-253* (CONN); Litchfield County, Salisbury, 25 Jul 1904, *Phelps, O.A. s.n.* (CONN); Tolland County, Mansfield, Storrs, Botanic Garden, University of Connecticut, 20 Jul 1912, *Schulze, A.F. s.n.* (CONN); Fairfield County, Vaughn's Neck, Candlewood Lake, New Fairfield, 20 Jul 1962, *Seymour, F.C. 20068* (MO); Middlesex County, East Haddam, 29 Jul 1978, *Smith, E.J. s.n.* (CONN); Hartford County, Suffield, Manitook Mountain, along RR N of Manituck Quarry (Quarry Road), 13 Aug 1953, *Smith, J.F. s.n.* (CONN); Hartford County, Suffield, canal bank, near quarry, *Smith, J.F.*

s.n. (NCBS); New Haven County, North Haven, 20 Jul 1993, *Snyder, J. 27* (CONN); Middlesex County, Portland, Stonycroft, 18 Aug 1896, *Starmer, F.W. s.n.* (NY); New London County, Lyme, 30 Jul 1937, *Stutz, S.S. s.n.* (NCBS); New London County, Lyme, 8 Aug 1936, *Stutz, S.S. s.n.* (NCBS); Middlesex County, East Haddam, 21 Aug 1895, *Thompson, E.J. s.n.* (CONN); Hartford County, East Hartford, Jul, *Weatherby, C.A. s.n.* (NEBC); Hartford County, Glastonbury, Aug 1889, *Wilson Starmer, M.F. s.n.* (NCBS); Hartford County, Glastonbury, 5 Sep 1890, *Wilson Starmer, M.F. s.n.* (NCBS); New Haven County, East Haven, South End, 1 Sep 1886, *Winton, A.L. s.n.* (YU); New Haven County, New Haven, Jul 1856, *Without Collector s.n.* (YU); Fairfield County, Bridgeport, hillside, 1887, *Without Collector s.n.* (NCBS); Hartford County, East Hartford, Connecticut River, 23 Sep 1909, *Woodward, R.W. s.n.* (NCBS); Hartford County, East Hartford, Connecticut River, 23 Sep 1909, *Woodward, R.W. s.n.* (NCBS); New Haven County, New Haven, Oct 1903, *Woodward, R.W. s.n.* (NCBS); New London County, Franklin, 13 Oct 1920, *Woodward, R.W. s.n.* (NCBS); Hartford County, Windsor, Rainbow, Northwest Park, 19 Aug 1998, *Zebryk, T.M. 5509* (CONN). **Delaware**: New Castle County, Wilmington, *Canby, W.M. s.n.* (NY); Sussex County, Gravel Hill, Steiner Road (Road 320) just S of US Rt 9-DE Rt 404, Lewes Georgetown Highway (Road 18) south of railroad tracks, 30 Jun 2011, *Longbottom, W.D. 15354* (MARY, NY); Kent County, 2 mi S of Smyrna, 6 Nov 1977, *Reed, C.F. 103008* (MO). **District of Columbia**: opposite Georgetown, 10 May 1891, *Blanchard, F. s.n.* (NY); Washington, D.C, *Camby, W.J. s.n.* (US); Washington DC area, 30 Sep 1873, *Chickering, J.W. s.n.* (US); Rock Creek Park, W of Rock Creek, S of Broad Branch Road and Beach Drive Rock Creek Park and vicinity, 17 Aug 1988, *Fleming, P. 897* (US); Rock Creek Park, W of Rock Creek, S of Broad Branch Road and Beach Drive Rock Creek Park and vicinity, 7 Sep 1988, *Fleming, P. 929* (US); Fort Circle Parks. Fort Slocum Park facing Kansas Ave. NW, 2 Aug 1989, *Fleming, P. 1197* (US); Columbia Island, S bank of Potomac River, N of Navy Marine Memorial, 11 Aug 1989, *Fleming, P. 1243* (US); Kenilworth Park, adjacent to White House Greenhouses, 28 Aug 1989, *Fleming, P. 2367* (US); Anacostia Park, next to Anacostia Dr, S of John Philip Sousa Bridge SE Section D, 11 Sep 1989, *Fleming, P. 2418* (US); North slope of Mount Hamilton, 11 Sep 1932, *Leonard, E.C. 10350* (US); Rock Creek near west gate of Zoo Rock Creek Park and vicinity, 15 Sep 1895, *Pollard, C.L. 650* (US); Washington DC, along Constitution Avenue, 1 Dec 1980, *Reed, C.F. 113699* (MO); Washington DC, Along Polomac River, Theo. Roosevel Blvd, 11 Sep 1974, *Reed, C.F. 143179* (MO); Near Washington D.C, *Seaman, W.H. s.n.* (US); Potomac Flats C. and O. Canal, 13 Sep 1911, *Tidestrom, I.F. 5342* (US); *Vasey, G. s.n.* (US). **Florida**: Alachua County, Talus slope, Cabin Ridge, 12 Aug 1937, *Anderson, E.S. s.n.* (MO). **Georgia**: Gwinnet County, Thompsons Mills and vicinity, 14 May 1908, *Allard, H.A. 208* (US); Bulloch County, ca. 1 mile S of Statesboro, campus of Ga. Southern College, 9 Jul 1961, *Boole, J.A. 1151* (GH); Elbert County, end of county road #86, toward Broad River, 15 Jul 1978, *Coile, N.C. & Bates, N. 1910* (BM); Cherokee County, S side of Etowah R about 1 mi. E of Little R and 6 mi. S 55W of Canton, 22 Aug 1948, *Duncan, W.H. 8783* (GH, MO, NY, US); Berrien County, near old barn 1 mile NNE of Nashville, 19 Aug 1950, *Duncan, W.H. 11840* (GH); Grady County, Arbor Farm, 6 mi S of Whigham, 7 Sep 1964, *Faircloth, W.R. 1553* (MO); Jefferson County, Louisville, 15 Jul 1897, *Hopkins, M.H. s.n.* (NY); Camden County, Kingsland, Hospitality Avenue, off GA rt 40, East King Avenue, east of I-95, 27 Feb 2013, *Longbottom, W.D. 18483* (NY); Houston County, Oaky Woods WMA, 20 Aug 2009, *Lynch, P.S. & Zomlefer, W.B. 631* (GA); Houston County, USGS 7.5 minute Hayneville Quadrangle; Oaky Woods WMA; North of Site 1A, 20 Aug 2009, *Lynch, P.S. & Zomlefer, W.B. 631* (ARIZ); Houston County, Oaky Woods WMA, 10 Jul 2010, *Lynch, P.S. & Darby, K. 1224* (GA); Jasper County, Monticello Glades (Chattahoochee-Oconee NF parcel 045 050); 4.7 km south of Monticello; bordered by Feldspar Road to south, State Route 11 to east. Sunny, disturbed creek bank in Piedmont gabbro upland depression forest, 7 Jul 2012, *Sewell, S. 563* (GA); Jasper County, Monticello, Gladesville (part of Chattahoochee-Oconee NF parcel 049 008); bordered by State Route 83 to NW, Whatley Road to SW, 22 Sep 2012, *Sewell, S. 929* (GA); Clarke County, Athens, State Botanical Garden of Georgia, Elaine Nash Prairie, 1 Oct 2015, *Sewell, S. & Zomlefer, W.B. 1652* (GA); Catoosa County, At the southern tip of Sand Mountain in the highest elevations of the karst woods. Site name: 247 Waypoint #: 247, 31 May 2012, *Shaw, J. & Estes, D. s.n.* (APSC); McIntosh County, Darien Junction, 25 Jun 1895, *Small, J.K. s.n.* (NY); Grady County, 22 Sep 2005, *Taylor, M.D. s.n.* (CLEMS); Macon County, along brick wall of E border of SE section of the Andersonville National Cemetery, 30 Sep 2014, *Zomlefer, W.B. 3896* (GA). **Illinois**: Winnebago County, 2 mi E of Harrison, 10 Jul 1935,

Anthony, R.B. s.n. (WIS); Cook County, Chicago, Lincoln Park, 2 Sep 1957, *Bennett, H.R. s.n.* (UC); St. Clair County, Cahokia Mounds, State Park, Monk's Mound, 16 Oct 1965, *Bennett, J.P. 35* (MO); Kane County, Aurora, City Dump and RR tracks [also partly in DuPage County], 29 Aug 1972, *Bergling, F. 2401* (F); Kankakee County, Iroquois River Watershed, Iroquois Woods Nature Preserve, Otto Township, 4 miles south of Kankakee. Quadrangle: Kankakee, Iroquois River Watershed. (22), 13 Aug 2004, *Busemeyer, D.T. et al. 2030* (MOR); Kankakee County, Iroquois Woods Nature Preserve, Otto Township, 4 miles south of Kankakee, IL, 13 Aug 2004, *Busemeyer, D.T. et al. 2030* (ILLS); McLean County, Funk's Grove 7.5; Funk's Grove Nature Preserve is an 18 acre tract of land within a larger parcel known as Funk's Grove, one of the largest remnants of virgin forest in Illinois. The grove contains mesic upland forest of the Grand Prairie Natural Divisio, 18 Jul 2005, *Busemeyer, D.T. et al. 2414* (ILLS); Grundy County, Coal City, Short Pioneer Cemetery Prairie Nature Preserve. 7 mile east and 2.5 mile south of Morris, Illinois, 21 Sep 2005, *Busemeyer, D.T. et al. 2520* (ILLS); Winnebago County, Sugar Creek Forest Preserve, 8 Oct 1949, *Chase, V.H. 10941* (NY); Tazewell County, East Peoria, 3 Sep 1956, *Chase, V.H. 14309* (MEXU, W); Macon County, sin. loc, Aug 1896, *Clokey, I.W. 233* (UC); Cook County, Chicago, 9 Sep 1882, *Deane, W. s.n.* (GH); Galva, 11 Sep 1891, *Dowell, P. 8652* (GH); Knox County, Maquon, Galesburg Section of the Western Forest Prairie Natural Division. Private property about 1.5 mile west of Douglas, Illinois. Site west of County Road 1800 East and north of railroad tracks, 8 Aug 2004, *Ellis, J. 100* (ILLS); Jackson County, Mississippi river shore, Station #6, 26 Oct 1968, *Evans, D.K. 544* (MO); Cook County, Chicago, Ravenswood, 27 Jul 1905, *Gates, F.C. 64* (F); Cook County, Chicago, Jefferson St, N Congress St, 24 Jul 1968, *Glassman, S.F. 8609* (F); Mason County, Havana, 15 Aug 1904, *Gleason, H.A. s.n.* (GH); Champaign County, Urbana, 28 Sep 1898, *Gleason, H.A. 121* (GH); Jackson County, Grand Tower, 20 Aug 1900, *Gleason, H.A. 1806* (GH); Champaign County, Champaign, 26 Jul 1900, *Gleason, H.A. 1938* (GH); Cook County, Willow Springs, 27 Jul 1876, *Grassly, C.W. s.n.* (F); Cook County, Chicago, 17 Sep 1876, *Grassly, C.W. s.n.* (F); Cook County, Berwyn, 1 Sep 1934, *Haynie, N.V. & Schantz, O.M. 2943* (F); Du Page County, Valley View of Glen Ellyn, 3 S 270 Shagbark, 15 Aug 2000, *Hess, W. 9290* (NY); Champaign County, Urbana, 1 Jul 1940, *Jones, G.N. 12297* (GH); Champaign County, Sangamon River 15 miles W of Urbana, 25 Aug 1940, *Jones, G.N. 12926* (GH); Vermilion County, along Vermilion River between Oakwood and Collison, 14 Sep 1940, *Jones, G.N. 13016* (GH); Champaign County, Near Urbana, 25 Aug 1945, *Jones, G.N. 17353* (WCSU); Fulton County, Canton Lake, near Canton, 9 Aug 1958, *Jones, N. 22375* (K); Will County, Plainfield, 23744 W Main Street, 20 Jun 2010, *Landrum, L.R. 11394 a* (NY); Cook County, Thornton, 14 Jul 1902, *Lansing, O.E. 1378* (F); Cook County, Chicago, Ravenswood, 2 Jul 1887, *Lloyd, R.N. s.n.* (F); Kankakee County, Leesville, Sweetfern Sand Savanna Land and Water Reserve. Approximately half a mile north of Pembroke School, 15 Oct 2002, *Marcum, P.B. & Phillippe, L.R. 1766* (ILLS); McLean County, Funks Grove Illinois Natural Areas Inventory (INAI) site. Funks Grove Nature Preserve. WGS 84 map datum; +/-23.0 ft, 11 Oct 2005, *Marcum, P.B. et al. 3797* (ILLS); Woodford County, El Paso, ParkLands Foundation's Chinquapin Bluffs Preserve; located approximately 5 miles north of Carlock, Illinois. Map Datum: WGS84/NAD83, 18 Sep 2009, *Marcum, P.B. 5911* (ILLS); La Salle County, Starved Rock, 7 miles southwest of Ottawa. 0.2 miles west of E. 15th Road, 0.2 mile north of N. 2450th Road, just inside forest, 21 Sep 2013, *Mathis, A.T. 2* (ILLS); Madison County, Watershed Nature Center, 1591 Tower Avenue, Edwardsville, 16 Jul 2001, *Moe, J.A. 271* (MO); Mason County, About 2 mile northeast of Bath, Illinois. Matanzas Prairie Nature Preserve. Illinois River Section of the Illinois and Mississippi River Sand Area Natural Division. Kilbourne Quadrangle, 21 Sep 1990, *Morris, M. et al. 676* (ILLS); Cook County, Chicago, *Munroe, H.F. s.n.* (F); Iroquois County, Bonnieäó»s Prairie Nature Preserve, 26 Sep 2005, *Murphy, M.J.C. & Phillippe, L.R. 800* (ILLS); Woodford County, Spring Bay Fen Nature Preserve. NAD83/WGS84, 28 Jul 2008, *Murphy, M.J.C. et al. 3091* (ILLS); Marshall County, Hopewell, IL. Approximately 5 miles north of Chillicothe, IL. 0.4 mile due west of IL Route 29. Lots 184, 185, & 186, 3 Jul 2012, *Murphy, M.J.C. et al. 5072* (ILLS); Jo Daviess County, vicinity of cemetery on SW side of Hanover, T26N, R2E, SE 1/4, SW 1/4, Sec. 9, 26 Sep 1981, *Nee, M. 22013 b* (MO, NY, WIS); Cook County, Chicago, 4200N Haxel St, 28 Aug 1982, *Nee, M. 25542* (CORD, MO, NY); Champaign County, along hwy. I-57, 5 miles SW of Champaign. T 18 N; R 8 E; NE1/4NW1/4 sec 16, 25 Sep 1982, *Nee, M. & Taylor, K. 25715* (BM, GH, MEXU, MO, US, WIS); Champaign County, Champaign, 11 Sep 1909, *Pease, A.S. 12474* (GH); Marion County, Omega, Stephen A. Forbes State Park. Mount Vernon Hill Country Section of the

Southern Till Plain Natural Division. Coordinate Datum: WGS84/NAD83. Northwest of Forbes Lake where stream un-named stream joins Lost Fork at the north end of Forbes Lake. On the west, 13 Aug 2001, *Phillippe, L.R. et al. 33576* (ILLS); Mason County, Sand Ridge State Forest. Illinois River Section of the Illiinois River and Mississipi River Sand Arreas Nataural Division. Duck Island Quadrngle. Elevation about 500 feet. Burns Prairie. (WGS84/NAD83), 1 Jun 2004, *Phillippe, L.R. & Busemeyer, D.T. 36787* (ILLS); McLean County, Funk's Grove in Bottomland Woods along Timber Creek. University of Illinois Property. About 2.3 mile northeast of McLean, Illinois. In the Grand Prairie Section of the Grand Prairie Natural Division, 8 Jul 2005, *Phillippe, L.R. & Marcum, P.B. 37712* (ILLS); McLean County, Funk's Grove in Bottomland Woods along Timber Creek. University of Illinois Property. About 2.3 mile northeast of McLean, Illinois. In the Grand Prairie Section of the Grand Prairie Natural Division, 12 Aug 2005, *Phillippe, L.R. & Marcum, P.B. 37883* (ILLS); Will County, In the Grand Prairie Section of the Grand Prairie Natural Division. Midewin National Tallgrass Prairie South Patrol Road Restoration Area. About 3 mile north by northwest of Wilmington and east of Prairie Creek. GPS Coordinates from WGS84/NAD 83, 29 Sep 2005, *Phillippe, L.R. et al. 38342* (ILLS); Will County, Channahon, In the Grand Prairie Section of the Grand Prairie Natural Division. Blodgett Road Dolomite Prairie on the Des Plaines Wildlife Conservation Area. About 6 mile northwest of Wilmington and along west side of Interstate 55. GPS coordinates from WGS84/NAD83, 30 Sep 2005, *Phillippe, L.R. & Molano-Flores, B. 38387* (ILLS); Will County, Grant Creek Nature Preserve in the Midewin National Tallgrass Prairie Property. Along the east side of Interstate 55, Exit 241 near Wilmington, go to the east frontage road of Interstate 55, then turn north and go 0.8 mile. Grand Prairie Section of the G, 10 Aug 2006, *Phillippe, L.R. et al. 39060* (ILLS); Will County, Wilmington Shrub Prairie Nature Preserve. 3 miles east of Braidwood (junction of Route 53 & 113) along Route 113, 1.5 mile north on Zilm Road, and then 0.5 mile west. In the Kankakee Sand Area Section of the Grand Prairie Natural Division. Coordinate dat, 15 Aug 2007, *Phillippe, L.R. et al. 40095* (ILLS); Ogle County, Grand Detour, White Pine State Park on the west side of Pine Creek along Spring Creek. About 13 mile north of Dixon and 3.75 mile east of Buffalo, Illinois. In the Freeport Section of the Rock River Hill Country Natural Division. Coordinate Datum: WGS84/NAD83, 17 Sep 2008, *Phillippe, L.R. & Ebinger, J.E. 41277* (ILLS); Cook County, Chicago, along Lake Michigan between the Adler Planetarium and Shedd Aquarium, 19 Jul 1971, *Reed, P. & Reed, L. s.n.* (MO); Grundy County, Dresden Lakes area, 31 Jul 1967, *Smith, E.M. 325* (F); Champaign County, Urbana, near Busey Pasture, 28 Aug 1946, *Snyder, D.P. 89* (MICH); Knox County, Cemetery, NE1/4, Sec. 17, Cedar Twp, 23 Jul 1974, *Solomon, J.C. 1092* (MO); Madison County, Illinois Terminal Railroad yard, 2 miles N of Interstate 55 ILL 203, R9W T3N sect. 31, 18 Jun 1978, *Solomon, J.C. 3853* (MO); DuPage County, Naperville, 4 Aug 1897, *Umbach, L.M. s.n.* (F, WIS); Du Page County, Naperville, 4 Aug 1897, *Umbach, L.M. s.n.* (US); Cook County, Chicago, West Pullman, Jul 1899, *Wessen, L.J.R. 3211* (F); Knox County, Galesburg, *Without Collector s.n.* (WIS). **Indiana**: Putnam County, Greencastle, 9 May 1911, *Banker, H.J. 1490* (NY); Jefferson County, Clifty Park, 25 Jul 1933, *Banta, E. s.n.* (IND); Montgomery County, Crawfordsville, [Wabash] College campus, 5 Sep 1927, *Bechtel, A.R. 11886* (NY); Lawrence County, 1 mile north, 1/3 mile east of Pleasant Run Church, 9 Sep 1972, *Burton, D.L. 616* (IND); Monroe County, Off Rifle Range Road, Bloomington, 10 Oct 1972, *Burton, D.L. 617* (IND); Jefferson County, Hanover College, 1877, *Coulter, J.M. s.n.* (NY); Madison County, south side of White River about 2 miles north of Anderon, 11 Aug 1907, *Deam, C.C. 2415* (IND); Ripley County, laugherty Creek near Versailles, 23 Jul 1913, *Deam, C.C. 13763* (IND); Grant County, 5 miles northeast of Fairmount, 4 Sep 1914, *Deam, C.C. 15278* (IND); Spencer County, 2 miles west of Grandview, 30 Jun 1915, *Deam, C.C. 16648* (IND); Knox County, Bordering the Claypole Pond, 8 Jul 1915, *Deam, C.C. 17016* (IND); Harrison County, 4 mi. southeast of Corydon, 5 Sep 1915, *Deam, C.C. 18631* (IND); Perry County, bordering the Ohio River 6 mi. east of Cannelton, 2 Oct 1920, *Deam, C.C. 33355* (IND); Gibson County, 1 mi. east of Skelton, 27 Sep 1921, *Deam, C.C. 35125* (IND); Posey County, Along the Wabash River about 2 mi. below New Harmony, 28 Sep 1921, *Deam, C.C. 35151* (IND); Jennings County, 1 mi. west of Vernon, 23 Jul 1922, *Deam, C.C. 37075* (IND); Warrick County, 1.5 miles northeast of Dayville, 14 Aug 1922, *Deam, C.C. 37606* (IND); Monroe County, along Clear Creek about 2 mi. northwest of Harrodsburg, 17 Sep 1922, *Deam, C.C. 37998* (IND); Crawford County, Ohio River about 1 mi. below Leavenworth, 15 Jul 1925, *Deam, C.C. 41569* (IND); Fountain County, along Big Shawnee Creek about 4 mi S of Attica, 18 Sep 1932, *Deam, C.C. 53116* (IND); Henry

County, just west of the marl bog about .5 miles east of Mt. Summit, 30 Sep 1936, *Deam, C.C. 57458* (IND); Franklin County, 4 mi. west of Metamora, 9 Aug 1937, *Deam, C.C. 58077* (IND); Ohio County, Along the Ohio River in Rising Sun, 10 Aug 1937, *Deam, C.C. 58159* (IND); Washington County, about a mile north of Livonia, 21 Aug 1945, *Deam, C.C. 63478* (IND); Howard County, alley West Mulberry Street [Kokomo], 13 Aug 1940, *Ek, C.M. s.n.* (NY); Howard Conty, 12 miles W of Kokomo, 8 Aug 1941, *Ek, C.M. s.n.* (NY); Brown County, Youngman Farm on Clay Lick Road, 0.5 miles NE of Nashville, 8 Aug 1943, *Friesner, R.F. 17874* (NY); Putnam County, hillsides S of Russellville, 18 Sep 1910, *Grimes, E.J. 367* (NY); Monroe County, J.S. Brown's Ellettsville, 30 Jun 1924, *Guillon, M.A. 4234* (IND); Posey County, Posey Co, Ind, 1954, *Heiser, C.B. s.n.* (IND); Millers, 8 Aug 1915, *Johnson, F.W. 2044* (NY); Daviess County, 4 miles west of Washington, Daviess Co. Indiana on old Hwy. 50, 26 Jul 1956, *Langford, F.L. 89* (IND); Porter County, dunes at Oak Hill edge, 21 Aug 1929, *Lyon, M.W. s. n.* (MICH); Vigo County, Terre Haute, active sand pit site, between Conrail Railway and CSX Railway rights-of-way, 800 m west of N 25 Street, east of the eastern end of North Avenue and N 19 Street (Sample Unit 15), 11 Oct 2005, *Macdonald, I.D. a 14* (IND); Putnam County, sin. loc, 4 Oct 1889, *MacDougal, D.T. s.n.* (NY); Putnam County, sin. loc, 10 Jul 1888, *MacDougal, D.T. s.n.* (NY); Floyd County, IN. Floyd County, Brock-Sampson Nature Preserve, 3 Sep 1987, *Maxwell, R.H. 2736* (IND); Vermillion County, Sect. 15, R9W, T17N, 7 Oct 1977, *McClain, W. 210 D* (IND); Vermillion County, Sect. 15, R9W, T17N, 7 Oct 1977, *McClain, W. 2122* (IND); Gibson County, Gibson Power Station Property, NE 1/4, Sect. 33, R12W, T1S, 13 Oct 1977, *McClain, W. 2193* (IND); Jennings County, sin. loc, 6 Aug 1932, *McCoy, S. 551* (NY); Hamilton County, along White River 3 miles S of Noblesville, 23 Sep 1934, *McCoy, S. 2172* (NY); Marion County, John Holliday Park Botanical Garden, 25 Jul 1942, *McCoy, S. 5929* (NY); Newton County, Goodland, 30 Aug 1922, *McKee, M. 3* (NY); Owen County, Upper field at 3251 E Romona Road, 3.5 miles WSW of Gosport, 19 Aug 2017, *Mow, D.M. 282* (IND); Owen County, Romona Road near railroad track farm crossing, 1.5 miles west of Gosport, 13 Sep 2017, *Mow, D.M. 317* (IND); Putnam County, Greencastle, 17 Sep 1987, *Nee, M. 35905* (CORD, MO, NY); Marion County, NW part of Indianapolis, SE side side of junction of I-65 and Crawfordsville Road, 1 Aug 2013, *Nee, M. 60311* (NY); St. Joseph County, Shedelbakesi Woods, South Bend, NW Indiana, 16 Oct 1913, *Nieuwland, J.A. 11598* (MO); Porter County, Mineral Springs, 4 Sep 1920, *Peattie, D.C. s.n.* (GH); Greene County, Greene County, off Highway 67, 1 mile S of Switz City, 3 Aug 1983, *Purepong, W. 122* (IND); Jefferson County, Hanover, *Raben, C. s.n.* (NY); Wayne County, [Richmond?], *Ried, A.S. 163* (NY); Grant County, west of Reade Center, campus, Taylor Univ, 5 Oct 1984, *Rothrock, P.E. 1823* (IND); Marshall County, Lake Maxinkuckee, W shore of Lake, 30 Oct 1900, *Scovell, I.T. & Walton Clark, H. 1514* (NY); Marshall County, Near Lake Maxinkuckee, 1 Aug 1900, *Scovell, J.T. & Clark, H.W. 1333* (US); St. Joseph County, Mishawaka, Aug 1891, *Uline, E.B. s.n.* (F); Lake County, Miller [Gary], 25 Aug 1898, *Umbach, L.M. s.n.* (F); Lake County, Millers, 27 Jul 1909, *Umbach, L.M. 4121* (UC); Clarke, [Clark County?], 18 Sep 1909, *Umbach, L.M. 5097* (UC); Lake County, Millers, 26 Aug 1914, *Umbach, L.M. 6747* (UC); Morgan County, W. of Martinsville, 11 Jul 1913, *Weatherwax, P. 1045* (IND); Jasper County, Carpenter Township, 16 Jul 1923, *Welch, W.H. 122* (NY); Monroe County, First ravine west of 1st Huckleberry Hill, 27 Sep 1927, *Welch, W.H. & Price, ? 3887* (IND); Hamilton County, Mattsville, 5 Sep 1892, *Wilson, G. s.n.* (NY); Putnam County, Greencastle, 20 Aug 1925, *Without Collector s.n.* (NY); Putnam County, field near Greencastle, 22 Jun 1922, *Yuncker, T.G. 1453* (NY); Putnam County, Greencastle, 13 Jul 1922, *Yuncker, T.G. 1800* (NY); Vanderburgh County, North of river slough -Evansville, In, 21 Jun 1941, *Zeiner, H.M. s.n.* (IND); Vanderburgh County, Angel Mounds, Evansville, 4 Jun 1941, *Zeiner, H.M. s.n.* (IND); **Iowa**: Marion County, Union Twp. adjacent to Red Rock Cliff N1/2 Sec. 3, 18 Jun 1957, *Bruggen, T. van, 2134* (UC); Mitchell County, Iowa 218 ca 3 mi N of Osage, 26 Aug 1972, *D'Arcy, W.G. 6891* (MO); Story County, Ames, Brookside Park, 29 Aug 1969, *Davidse, G. 1967* (MO); Van Buren County, Southeastern Iowa. Barnyard fence-row, O. C. Newbold farm, app. 1 mi. W of Hillsboro, SW ¼ Sec. 25, T-70N, R-8W, *Davidson, R.A. 3354* (US); Cedar County, Sect. 16, Linn Twp, 31 Aug 1950, *Fay, M.J. 1738* (UC); Fayette County, Fayette, Jun 1894, *Fink, B. s.n.* (GH); Fayette County, sin. loc, 1 Aug 1894, *Fink, B. 365* (US); Cherokee County, Rock Township, 20 Aug 1908, *Heddle, J. 1151* (WIS); Lee County, Indian Trail County Park, floodplain along SW side of Skunk River, just upstream from the Hwy. 61 bridge, 16 Aug 2013, *Nee, M. 60371* (BM, NY); Story County, Ames, Sep 1897, *Pammel, L.H. 586* (MO); Jasper County, Ames, Sep 1897, *Pammel, L.H. 586* (GH, US);

Dickinson County, Okoboji Lake, S. of Lakeside Lab, 4 Aug 1910, *Shimek, B. s.n.* (WIS); Johnson County, Iowa City, 28 Aug 1909, *Somes, M.P. 3765* (US); Allamakee County, Hanover twp, Upper Iowa valley, 7 Jul 1933, *Tolstead, W.L. s.n.* (MO); Allamakee County, near west county line in Upper Iowa Valley, 16 Jul 1933, *Tolstead, W.L. s.n.* (UC); Marshall County, Albion, Jul 1878, *Without Collector s.n.* (NY); sin. loc, 2 Aug 1882, *Without Collector 249* (UT). **Kansas**: Clark County, sin. loc, 28 Jul 1938, *Cook, W. s.n.* (UTC); Dickinson County, 5 miles S of Abilene, Oct 1964, *Jacobson, J. 5* (MO); Cowley County, 4 mi NW of Arkansas City, 9 Aug 1966, *Koch, R.G. 1736* (WIS); Cowley County, Arkansas River valley, 5 mi W, 4 mi N of Arkansas City, 9 Aug 1966, *Koch, R.G. 1781* (WIS); Cowley County, along Arkansas River, 7 mi W of Winfield, 11 Aug 1966, *Koch, R.G. 1830* (WIS); Cowley County, 5 mi N, 1 mi E of Cambridge, 10 Sep 1966, *Koch, R.G. 2255* (WIS); Cowley County, Cowley County State Lake; 13 mi E, 1 mi N of Arkansas City, 13 Sep 1966, *Koch, R.G. 2297* (WIS); Cowley County, 4 mi NW of Arkansas City, 5 Oct 1966, *Koch, R.G. 2479* (WIS); Cowley County, 0.5 mi S of Arkansas City, 28 Jun 1967, *Koch, R.G. 3959* (WIS); Republic County, at W edge of grazed prairie pasture in partial shde just W of pond in NW1/4 sec. 23 T4S, R2W, 15 Aug 1961, *Morley, G.E. 1296* (NY); Crawford, Pittsburg, 6 miles N of town, 21 Jun 1929, *Rydberg, P.A. & Imler, R. 150* (NY); Cowley County, Arkansas City, S of Arkansas River, 2 Jul 1929, *Rydberg, P.A. & Imler, R. 467* (NY); Logan County, Bilby's Lakes, 30 miles S of Oakley, 17 Jul 1929, *Rydberg, P.A. & Imler, R. 1021* (NY); Osborne County, within 5 mile radius of Osborne City, S Frok Solomon River, 20 Jul 1894, *Shear, C.L. 177* (GH); Hamilton County, Syracuse, 12 Jul 1893, *Thompson, C.H. 114* (MO, NY, US); Riley County, Manhattan, Jul 1889, *Varney, M. s.n.* (MICH); Lyon County, Ross National History Reservation, 4 mi W of Americus, 9 Aug 1960, *Weber, A.V. s.n.* (WIS); Mitchell County, NE shor of Lake Waconda, W of Glen Elder, 28 Jul 1982, *Wingate, J. 1831* (KHD). **Kentucky**: Ballard County, Ballard Waterfowl Management Area, 24 Aug 1973, *Athey, R. 2515* (MO); Cumberland County, on small tributary to Crocus Creek; along Crocus Creek, 1.3 miles s. of Adair-Cumberland Co. line on Ky. hwy. 704, 7 Sep 1997, *Beck, J.B. et al. 104* (EKY); Kenton County, Devou Park, 19 Sep 1942, *Braun, E.L. 4641* (US); Henry County, old road leading from Ky R from dirt road off Ky 389, W of Ky R near natural lake, this point 2.4 miles E of Ky 389, 19 Jul 1962, *Browne, E.M. & Browne, E.T. 5963* (NY); Campbell County, along RR tracks ca. 8 miles SE of Silver Grove on Rt. 8, 11 Sep 1983, *Buddell, G.F. 1433* (NY); Lyon County, Kuttawa, Tennessee-Cumberland River region, 27 Sep 1909, *Eggleston, W.W. 5321* (NY); Henry County, Ky 561, 1.2 miles SW of Gest, 0.5 mile from highway, S end of Hardin Bottom, 10 Aug 1962, *Gentry, J.L. 671* (NY); Elliott County, at Tree Farm Road, 1 Sep 2012, *Greene, M. s.n.* (EKY); Madison County, Berea College Forest Area, above Cowbell Lake, along lumber road, 13 Jul 1965, *Grossman, J. 560* (NY); Union County, E bank of hte Ohio River, boat launch ramp and parking area, Blackburn, 0.6 miles N of the Rte. 13/56 bridge, 22 Sep 1994, *Hill, S.R. 26155* (NY); sin. loc, 1916, *King, A. 146* (US); Edmonson County, Mammoth Cave National Park. MCNP-Cedar Sink, 1 Oct 1949, *Lix, H.W. 657* (US); Hickman County, near Fulton, 26 Sep 1973, *McCoy, T. 73-2033* (MO); Barren County, Cave City, (Route 31 W not found on map), 17 Jun 1969, *Reed, C.F. 81849* (MO); Scott County, Field, farm C. C. Singer, Stamping Ground, 14 Jul 1930, *Singer, J.W. 351* (US); Wayne County, Beaver Creek, SW of Monticello, 12 Jul 1937, *Smith, L.B. & Hodgdon, A.R. 3903* (GH); Marion County, 2.5 miles SW of New Market, 10 Aug 1939, *Wharton, M.E. 5143* (MICH); Powell County, 3 miles N of Waltersville, 3 Oct 1939, *Wharton, M.E. 5210* (MICH). **Louisiana**: Orleans Parish, just W of lake in the new section of Brechtel Park near the Lennox St entrance in Algiers, New Orleans, 29 Nov 1981, *Pruski, J. 2237* (NY); Madison Parish, S of US 80, 0.8 miles E of Bayou Macon (Sec. 17, T17N, R10E), 11 Jun 1978, *Rich, N. & Rich, J. 879* (NY); De Soto Parish, proposed Cajun Electric's lignite power plant site SE of Evelyn and La 177 (Sec. 31, T12N, R10W), 28 Sep 1978, *Thomas, R.D. & Dixon, D. 61226* (NY); Ouachita Parish, Monroe, along railroad tracks near Royal Feed and Seed at DeSiard and Sixth, 28 Nov 1979, *Thomas, R.D. 69805* (GH, NY); Lincoln Parish, Roadbank of I-20, 2.6 miles east of Bienville Parish Line west of Simsboro, Sec. 13, T18N, R5W, 11 Oct 1986, *Thomas, R.D. 98597* (MO, NY); West Carroll Parish, South of La. 134 two miles east of Richland Parish line and about 6.2 miles west of Epps, Sec. 21, T19N, R9E, 27 May 1987, *Thomas, R.D. 99955* (MO, NY); Ouachita Parish, South and East of La. 557 west of Luna at Cypress Turnoff, sec. 2, T15N, R2E, 12 Oct 1988, *Thomas, R.D. & Dendrology Class 108232* (MO, MOR, NY); Natchitoches Parish, Under and beside elevated La. 6 Bridge on north bank of Red River noth of Natchitoches and grand Ecore, Sec. 46, T10N, R7W, 24 Nov 1989, *Thomas, R.D. 114863* (MO, NY); Ouachita Parish,

Ouachita River Levee, along Levee and La 553 at corner S of Sterling and N of Keystone Road (Sec. 31 T20N, R4E), 7 Oct 1992, *Thomas, R.D. & Dendrology Class 132327* (NY); Winn Parish, beside LA 156 at Saline Bayou east of Goldonna, 31 Oct 1992, *Thomas, R.D. et al. 132980* (MO); St. Bernard Parish, along both sides of La. 47 at Quality Inn Motel in Chalmette, Sec. 46, T12S, R12E, 11 Mar 1994, *Thomas, R.D. 138877* (MO); East Baton Rouge Parish, median of US 61 N of La 964 about 5 miles N of I-110, Baton Rouge (Sec. 44, T5S, R1W), 5 Dec 1994, *Thomas, R.D. & Thomas, B.G. 143348* (NY); Bienville Parish, on N side of Gentry Hill at roads to firetower and to Driskill Mountain beside la 507 (Sec. 4, T16N and Sec. 33, T17N, R5W), 21 Nov 1995, *Thomas, R.D. 148045* (NY); Claiborne Parish, Kisatchie N.F. Caney Dist. Comp. 16, Stands 4&6, along CN16C south of USFS911, along stream south of the end of CN16C, N of La. 9 & Antioch, Sec. 15, T22N, R6W, 23 Aug 1996, *Thomas, R.D. 151462* (MO); Beauregard Parish, Beside N-to-S railroad tracks south of US190 in downtown DeRidder, Sec. 32, T2S, R9W, 7 Dec 1996, *Thomas, R.D. 153473* (MO, NY). **Maine**: Hancock County, Cranberry Isles, Bear Island, 12 Jul 1963, *Armstrong, J.C. s.n.* (NHA); Oxford County, West Paris, Berry Ledge, Aug, *Bean, R.C. s.n.* (NEBC); Sagadahoc County, Phippsburg, Small Point, Jul, *Bean, R.C. s.n.* (NEBC); Franklin County, Strong, Sep, *Chamberlain, E.B. & Knowlton, C.H. s.n.* (NEBC); Lincoln County, Bristol, Pemaquid Point, Aug, *Chamberlain, E.B. 127* (NEBC); Cumberland County, Cumberland, Jul, *Chamberlain, E.B. 420* (NEBC); Lincoln County, Southport, Sep, *Cole, A.E. 3717/ 3211* (NEBC); Somerset County, Pittsfield, 27 Aug 1893, *Drake, O. s.n.* (VT); Lincoln County, Boothbay, Damariscove Island, Jul, *Fassett, N.C. 437* (NEBC); Sagadahoc County, Phippsburg, Small Point, Aug, *Fassett, N.C. 10447* (NEBC); Washington County, Eastport, Carlow Island, Passamaquoddy Bay, Aug, *Fernald, M.L. 2090* (NEBC); Hancock County, Bar Harbor, Sep, *Furbish, K. s.n.* (NEBC); Hancock County, W. Sister's Co. Penob[scot] B[ay] [georef to Penobscot], Aug 1896, *Harvey, F.L. & Harvey, L.H. 701* (NHA, US); Knox County, Isle au Haut, Kiimballs Island, Jul, *Hill, A.F. 1657* (NEBC); York County, Kennebunkport, Aug, *Kennedy, G.G. s.n.* (GH); Franklin County, Chesterville, Aug, *Knowlton, C.H. s.n.* (NEBC); Franklin County, Chesterville, Aug, *Knowlton, C.H. s.n.* (NEBC); Franklin County, Chesterville, Aug, *Knowlton, C.H. 737* (GH); Hancock County, Bear Island, Penobscot Bay, on low ground near shoreline at NW corner of island, 7 Aug 1971, *McVaugh, M.R. 134* (MICH); Knox County, Owl's Head, 20 Aug 1902, *Moore, A.H. 331* (UC); Cumberland County, Scarborough, 11 Oct 1910, *Norton, A.H. s.n.* (NHA); Sagadahoc County, Bowdoinham, 28 Sep 1919, *Norton, A.H. s.n.* (NHA); Sagadahoc County, Georgetown, Aug, *Noyes, H.M. s.n.* (NEBC); Oxford County, Woodstock, Mt Perham, Aug, *Parlin, J.C. s.n.* (NEBC); Oxford County, Woodstock, Pesham Int, 1887, *Parlin, J.C. s.n.* (GH); Hancock County, Mount Desert, Flying Mt, Aug, *Rand, E.L. s.n.* (NEBC); Hancock County, Mount Desert, Great Cranberry Island, Aug, *Rand, E.L. s.n.* (NEBC); Hancock County, Little Cranberry Island, 17 Jul 1900, *Rand, E.L. s.n.* (YU); Hancock County, Mount Desert, S shore of L. Cranberry Isl, Aug, *Redfield, J.H. s.n.* (NEBC); York County, Old Orchard Beach (Ocean Park), 7 Sep 1970, *Wells, T.W. s.n.* (NEBC). **Maryland**: Howard County, Ellicott City, 7 Jul 1916, *Arsène, G. 594* (UC); Frederick County, Rocky woods near Foxville Tower, Catoctin Range, 28 Aug 1955, *Baltars, E. 669* (US); Montgomery County, west end tip by Islands, 11 Aug 1982, *Beyersdorfer, M. 181* (US); Calvert County, Camp Roosevelt, 28 Aug 1943, *Deignan, H.G. 199* (US); Montgomery County, small un-named island in Potomac River near Dot Island and SW of Plummers Island, 11 Oct 1993, *Dorr, L.J. 7960* (NY, US); Forest Glen, 4 Sep 1904, *Dowell, P. 8642* (GH); Talbot County, 1/2 mile SW x S of Easton, 1 Oct 1944, *Earle, E.C. 4001* (GH); Caroline County, Idlewild Wildlife Management Area. Along Marshyhope Creek. Bloomery Road entrance, 27 Sep 2015, *Hall, J.D. & Norman, C.M. JH-24* (MARY); Montgomery County, Plummer's Island, 15 Jul 1914, *Hitchcock, A.S. 13016* (US); Montgomery County, Plummer's Island, 9 Sep 1917, *Hitchcock, A.S. 15746* (US); Prince Georges County, Patuxent Wildlife Refuge, 29 Sep 1937, *Hotchkiss, N. 5719* (US); Prince Georges County, Ammendale, 1918, *Hyacinth, F. 1575* (US); Montgomery County, Plummer's Island; mainland, edge of Stewart Farm, 9 Sep 1928, *Killip, E.P. 13313* (US); Montgomery County, Plummer's Island; east sandbar, 13 Jul 1952, *Killip, E.P. 42225* (US); Montgomery County, Chesapeake and Ohio Canal National Historical Park, Plummers Island, mid island, near north shore, 11 Jun 2004, *Kress, W.J. & Butts, M.B. 04-7514* (US); Ciyt of Baltimore, Baltimore, 1866, *Le Roy, P.V. s.n.* (NY); Montgomery County, Plummer's Island, west tip, 27 Sep 1960, *Leonard, E.C. & Hotchkiss, N. 3340* (US); Montgomery County, Plummer's Island; Boat Landing, 27 Sep 1960, *Leonard, E.C. & Hotchkiss, N. 3341* (US); Dorchester County, N of East New Market and NW of Hurlock, along MD Rte 16-331 at

Beulah Road and Gravel Run, 13 Sep 2008, *Longbottom, W.D. 12187* (NY); Anne Arundel County, Pasadena, Beachwood Park, Beachwood Park Road and Magothy Bridge Road, MD rte 607 at Magothy River, 29 Aug 2010, *Longbottom, W.D. 14228* (NY, US); Caroline County, Preston, along Railroad Avenue near Back Landing Road, 16 Jun 2011, *Longbottom, W.D. 15221* (MO, NY); Caroline County, between the towns of Preston, Harmony and Bethlehem, along Harris Road between Grove Road and Newton Road, 10 Jul 2011, *Longbottom, W.D. 15495* (MO, NY); Caroline County, Preston, along Railroad Avenue near Back Landing Road, along hedgerow by the baseball field, 6 Sep 2011, *Longbottom, W.D. 16013* (MARY, NY); Caroline County, Town of Preston, at the W end of Railroad Avenue near Lednum Avenue, 30 Oct 2011, *Longbottom, W.D. 16649* (MARY); Harford County, Darlington, MD Rt. 623, Flintville Road, near Paddrick Road, edge of woods along road; 38.694239 -76.240833; 38.694239 -76.240833, 14 Sep 2014, *Longbottom, W.D. & Brighton, J.D. 21660* (MARY); Worcester County, Snow Hill, 5 Aug 1924, *Moldenke, H.N. 2136* (NY); Baltimore County, Port Covington, foot of Light Street, 12 Jul 1979, *Reed, C.F. 9008* (MO); Baltimore County, Chesapeake Bay near New Bay Shore, 13 Aug 1950, *Reed, C.F. 21692* (MO); Host County, Fields near Glen Echo, S of Dublin, Rt#1, 2 Oct 1990, *Reed, C.F. 134766* (MO); Baltimore County, Baltimore, Slopes, Lake Poland nr. Dam, Rophat E Lee Mem. Park, Mt. Washington Jones Falls, 14 Sep 1981, *Reed, C.F. 146306* (MO); Montgomery County, High Island, Washington, D.C, 6 Jun 1878, *Ward, L.F. s.n.* (US); Baltimore County, area of Cromwell Bridge Road and Glen Arm Road - North of Towson, 24 Aug 1970, *Windler*, *3251* (H). **Massachusetts**: Hampden County, Springfield, slope to Connecticut River, 8 Oct 1966, *Ahles, H.E. 64847* (NY); Hampden County, Westfield, 21 Jul 1973, *Ahles, H.E. 77911* (WIS); Hampshire County, Northampton, Ryan Road, Jul, *Ahles, H.E. 85747* (NEBC); Hampshire County, Northampton between Daymon Road and Connecticut River, 18 Aug 1978, *Ahles, H.E. 86317* (BM, H); Franklin County, Shelburne, 1 Aug 1873, *Anderson, S.E. s. n.* (MICH); Hampden County, Springfield, banks of Mill River, Aug, *Andrews, L. s.n.* (NEBC); Essex County, Swampscott, Aug, *Batchelder, C.F. 1691* (NEBC); Middlesex County, Wakefield, Aug, *Bean, R.C. s.n.* (NEBC); Franklin County, Leyden, south of East Glen Road and east of East Glen Brook, 4 Sep 2014, *Bertin, R.I. & Searcy, K.B. 6800* (MASS); Nantucket County, Nantucket Island, 12 Aug 1906, *Bicknell, E.P. s.n.* (NY); Dukes County, Edgartown, Martha's Vineyard, 8 Oct 1917, *Bicknell, E.P. s.n.* (NEBC); Dukes County, Edgartown, near to shore of Trapps Pond, Martha's Vineyard, 16 Sep 1917, *Bicknell, E.P. s.n.* (NEBC); Nantucket County, Nantucket, shore of Lachacha, 16 Sep 1917, *Bicknell, E.P. s.n.* (NEBC); Dukes County, Martha's Vineyard, Edgartown, 16 Sep 1917, *Bicknell, E.P. 7717* (NY); Suffolk County, Near Boston, 1816, *Boot, ?*, *1323* (US); Franklin County, Sunderland, Sep, *Brooks, S.C. s.n.* (UC); Plymouth County, Scituate, Jul, *Brooks, W.P. s.n.* (NEBC); Berkshire County, Sheffield, Oct, *Churchill, J.R. s.n.* (NEBC); Franklin County, Montague, Conn[ecticut] R[iver], 3 Aug 1887, *Churchill, J.R. s.n.* (GH); Norfolk County, Hillside St. Milton, 3 Sep 1899, *Churchill, J.R. 713* (MO); Middlesex County, Malden, Goulding's Mills, Aug, *Collins, F.S. s.n.* (NEBC); Barnstable County, Provincetown, Jul, *Collins, F.S. s.n.* (NEBC); Barnstable County, Eastham, Jul, *Collins, F.S. 896* (NEBC); Middlesex County, Cambridge, shore of Charles River between Mass. Ave. and Anderson Bridge, Sep, *Collins, F.S. 3837* (NEBC); Dukes County, Chilmark, Muemsha Pond, Aug, *Cushman, J.A. 7492* (NEBC); Barnstable County, Barnstable Town, Hyannisport, Aug, *Deane, W. s.n.* (NEBC); Middlesex County, Concord, G.F. Root's Farm, Sandy Pond Rd, Oct, *Eaton, R.J. s.n.* (NEBC); Middlesex County, Lincoln, R.J.E.'s garden, Sep, *Eaton, R.J. s.n.* (NEBC); Barnstable County, Falmouth, Woods Hole, Aug, *Farlow, W.G. s.n.* (NEBC); Middlesex County, Cambridge, *Faxon, C.E. s.n.* (GH); Barnstable County, Provincetown, about old hosue site, Aug, *Fernald, M.L. & Long, B. 19068* (NEBC); Barnstable County, West Yarmouth, Oct, *Fernald, M.L. et al. 153682* (GH); Middlesex County, Westford, *Fletcher, E.F. s.n.* (GH); Middlesex County, Westford, *Fletcher, E.F. s.n.* (NEBC); Dukes County, Elizabeth Islands, Penikeck, 24 Jul 1923, *Fogg, J.M. 1462* (MO, NEBC); Dukes County, Gosnold, Nonamesset, S shore near E end, Aug, *Fogg, J.M. 2872* (NEBC); Essex County, Ipswich, Little Neck, Aug, *Forbes, F.F. s.n.* (NEBC); Franklin County, Buckland, Aug, *Forbes, F.F. s.n.* (NEBC); Norfolk County, Dover, Aug, *Fuller, T.O. s.n.* (NEBC); Plymouth County, yPlymouth, "colld. by my sister", Aug, *Fuller, T.O. s.n.* (NEBC); Worcester County, Worcester, weed in Gates garden, Sep, *Gates, B.N. s.n.* (GH); Worcester County, Sutton, Sep, *Gates, B.N. s.n.* (NEBC); Worcester County, Millbury, small un-named pond near center, 21 Sep 1944, *Gates, B.N. et al. 7170* (CONN); Worcester County, Sutton, Oct, *Gates, B.N. 13431* (GH); Worcester County, Sutton, 8 Oct 1945, *Gates, B.N. 18499* (CONN); Worcester County, Worcester, 11

Jul 1957, *Gates, W.C. 32495* (CONN); Middlesex County, Cambridge, "Hort. Cantab.", 1845, *Gray, A. s.n.* (GH); Dukes County, Chilmark, Aug, *Harris, S. s.n.* (NEBC); Essex County, Haverhill, dump, Primrose St. H12 on city map, Sep, *Harris, S.K. 655* (NEBC); Essex County, Boxford, our yard, Main Street, Oct, *Harris, S.K. 9650* (NEBC); Essex County, near Swampscott Cemetery, Jul, *Harris, S.K. 10691* (NEBC); Essex County, Groveland, Main Street, Sep, *Harris, S.K. 11181* (NEBC); Essex County, Haverhill, dump, Groveland Rd, Bradford, Sep, *Harris, S.K. 11198* (NEBC); Essex County, Marblehead, town dump, Sep, *Harris, S.K. 11455* (NEBC); Essex County, Peabody, dump, rte. 128, Sep, *Harris, S.K. 11545* (NEBC); Essex County, Boxford, dump on Spofford Road, Sep, *Harris, S.K. 11568* (NEBC); Essex County, Danvers, dump off Rte. 114, Sep, *Harris, S.K. 11640* (NEBC); Essex County, Lawrence, dump, Oct, *Harris, S.K. 11800* (NEBC); Essex County, Lynn, city dump, Oct, *Harris, S.K. 11841* (NEBC); Essex County, Saugus, Ballard Street, Oct, *Harris, S.K. 11869* (NEBC); Essex County, Gloucester, dump, Magnolia, Nov, *Harris, S.K. 11992* (NEBC); Essex County, Salem, North Gooseberry Island, Jul, *Harris, S.K. 13276* (NEBC); Essex County, Newburyport, dump, Crow Lane, Sep, *Harris, S.K. 20746* (NEBC); Franklin County, Deerfield, shore of Connecticut River, Sep, *Harris, S.K. 27090* (NEBC); Dukes County, Oak Bluffs, 14 Aug 1916, *Heatley, M. s.n.* (NY); Bristol County, Dartmouth, Nonquitt, Sep, *Hervey, E.W. s.n.* (NEBC); Bristol County, New Bedford, Sep, *Hervey, E.W. s.n.* (NEBC); Woburn, 20 Sep 1936, *Hill, H.E. s.n.* (CONN); Berkshire County, Sheffield, Sep, *Hoffmann, R. s.n.* (NEBC); Essex County, Rowley, Pingru Island, *Horner, C.N.S. s.n.* (NEBC); Worcester County, Princeton, Aug, *Hunnewell, F.W. s.n.* (NEBC); Middlesex County, Sherborn, Aug, *Hunnewell, F.W. 4942* (NEBC); Middlesex County, Hopkinton, Jul, *Hunnewell, F.W. 17996* (NEBC); Middlesex County, Bedford, Steams' barnyard, Aug, *Jenks, C.W. s.n.* (NEBC); Middlesex County, Lowell, Aug, *Jenks, C.W. s.n.* (NEBC); Worcester County, Hardwick, Sep, *Johnson, R.E. 32755* (NEBC); Norfolk County, Quincy, path end, Blue Hills Reservation, Sep, *Kennedy, G.G. s.n.* (GH); Middlesex County, Medford, Middlesex Falls Reservation, Oct, *Kidder, N.T. s.n.* (NEBC); Norfolk County, West Quincy, Willand Rd, Aug, *Kidder, N.T. s.n.* (NEBC); Norfolk County, Milton, my place, Sep, *Kidder, N.T. s.n.* (NEBC); Middlesex County, Cambridge, Botanic Garden, 13 Oct 1898, *Leavitt, R.A. s.n.* (ECON); Nantucket County, Nantucket Island, Siasconset, 14 Aug 1960, *MacKeever, F.C. N-433* (NEBC, NY); Middlesex County, Reading, Jul, *Manning, W.H. s.n.* (NEBC); Essex County, Plum Island, Hellcat Swamp, 28 Jul 1977, *McDonnell, M.J. 769* (NEBC, NHA); Plymouth County, Marshfield, Branch's Island, Jul, *Morss, C.H. s.n.* (NEBC); Plymouth County, Marshfield, Branch's Island, Aug, *Morss, C.H. s.n.* (NEBC); Worcester County, Petersham, gravel pit, 2 Sep 2005, *Motzkin, G. & Jenkins, J. HF2005-645* (HF); sin. loc, *Nuttall, T. s.n.* (BM); Middlesex County, North Chelmsford, Sep, *Pease, A.S. 1168* (NEBC); Franklin County, New Salem, *Peirce, G. 9-5* (NEBC); Suffolk County, South Boston, Sep, *Perkins, C.E. s.n.* (NEBC); Suffold County, Roxbury, Jul, *Plowman, T. 3341* (ECON); Middlesex County, Concord, Sep, *Potter, D. 1580* (GH); Middlesex County, Concord, Sep, *Potter, D. 7163* (GH); Worcester County, Auburn, 8 Aug 1958, *Potter, D. 32721* (CONN); Middlesex County, Fletcher Farm, Westford, 17 Sep 1979, *Reed, C.F. 105503* (MO); Suffolk County, Chelsea, Aug, *Rich, W.P. s.n.* (NEBC); Middlesex County, Wakefield, Happy Hollow, Sep, *Ripley Jr, W.S. 15921* (NEBC); Middlesex County, Cambridge, Sep, *Robinson, B.L. s.n.* (GH); Dukes County, Oak Bluffs, M.V, Aug, *Sanford, S.N.F. s.n.* (NEBC); Essex County, Ipswich beach, Sep, *Sears, J.H. s.n.* (GH); Dukes County, West Tisbury, Seth's Pond, near Red Lodge, Martha's Vineyard, Sep, *Seymour, F.C. 2006* (GH); Worcester County, Clinton, Clamshell Pond, near ice house, 4 Sep 1944, *Seymour, F.C. 7111* (CONN); Norfolk County, Needham, 12 Longfellow Road, Jun, *Standley, L.A. s.n.* (NEBC); Norfolk County, Needham, 12 Longfellow Road, 7 Jul 2001, *Standley, L.A. s.n.* (NEBC); Essex County, Gloucester, 23 Jul 1881, *Swan, C.W. s.n.* (YU); Middlesex County, Winchester, 28 Sep 1912, *Torrey, G.S. 108* (CONN); Hampshire County, Amherst, Aug, *Torrey, R.E. s.n.* (GH); Plymouth County, Marion, Sep 1888, *Vail, A.M. s.n.* (NY); Berkshire County, North Adams, Hoosic River bank, 8 Jul 1986, *Weatherbee, P.B. 563* (MASS); Berkshire County, Lanesborough, Constitution Hill; "Lanesboro", 9 Aug 1989, *Weatherbee, P.B. 2487* (MASS); Berkshire County, Williamstown, 11 Aug 1990, *Weatherbee, P.B. 3211* (NHA); Essex County, Newburyport, dump land by Merrimac River, Aug, *White, D. 406* (NEBC); Norfolk County, Needham, Charles River Village, 10 Aug 1912, *Wiegand, K.M. s.n.* (NY); Barnstable County, Woods Hole, Nanchan Island, 28 Jul 1912, *Wiegand, K.M. s.n.* (NY); Middlesex County, Waltham, N of Prospect Hill, Oct, *Williams, E.F. s.n.* (GH); Middlesex County, Concord, Oct, *Williams, E.F. s.n.* (GH); Middlesex County, Cambridge,

Fayerweather Street, Sep, *Williams, E.F. s.n.* (NEBC); Middlesex County, Winchester, 4 Sep 1941, *Williams, F.R. 2294* (A); Middlesex County, Cambridge, near Parker Street, Oct, *Collector*, *s.n.* (GH); Middlesex County, Cambridge, near Parker Street, Oct, *Without Collector s.n.* (GH); Middlesex County, Cambridge, Sep 1881, *Without Collector s.n.* (BSN); Suffolk County, Revere, Aug, *Young, H.A. s.n.* (NEBC); Suffolk County, Revere, Oak Island, Jul, *Young, H.A. s.n.* (NEBC). **Michigan**: Washtenaw County, Ann Arbor, *Almendinger, E.C. s.n.* (MICH); Antrim County, sec.30, T29N R7W, west side of Gilda's Rd. just south of Leonard Rd, *Appel, H. & Gendlin, J. 719* (MICH); Kent County, Gd. Rap. [=Grand Rapids], *Bailey, H.M. s.n.* (MICH); Ottawa County, along beach at Macatawa Park, Macatawa Lake, 7 Apr 1944, *Bazuin, C.W. 6084* (F); Washtenaw County, S.E. edge of 3rd Sister Lake in disturbed clearing, Saginaw Forest, Ann Arbor, *Benninghoff, W.S. 10880* (MICH); Berrien County, Collected at Warren Woods, *Billington, C. s.n.* (MICH); Berrien County, Collected at Warren Woods, *Billington, C. s.n.* (MICH); Livington County, ner Island Lake, 4 Aug 1929, *Brown, C.G. 2685* (NY); Jackson County, *Camp, S.H. & Camp, D.R. s.n.* (MICH); Mason County, Ludington, Hamlin Lake, 1910, *Chaney, R.W. 191* (NY); Kent County, Vergennes, *Cole, E.J. s.n.* (MICH); Allegan County, Pratt Lake, 15 Aug 1956, *Comte, F. 112* (MO); Ingham County, Lansing, Jul 1905, *Craig, M. 12552* (UC); Delta County, Escanaba -Wells, *Dachnowski, A. s.n.* (MICH); Gratiot County, Alma, *Davis, C.A. s.n.* (MICH); Grand Traverse County, near fork of Boardman River (Sect. 3 & 4, T 26 N, R 9 W), *Dieterle, J.V.A. 1211* (MICH); Mason County, Ludington State Park, *Dister, D.C. 264* (MICH); St. Clair County, [Port Huron], *Dodge, C.K. s.n.* (MICH); Huron County, Near Grindstone City, *Dodge, C.K. s.n.* (MICH); Huron County, near Grindstone City, *Dodge, C.K. s.n.* (MICH); Marquette County, Near lumber year at Big Bay, *Dodge, C.K. s.n.* (MICH); St. Clair County, Near Port Huron, *Dodge, C.K. s.n.* (MICH); St. Clair County, City of Port Huron, *Dodge, C.K. s.n.* (MICH); St. Clair County, Fort Gratiot, *Dr Pitcher*, *s.n.* (NY); St. Clair County, Fort Gratiot, *Dr Pitcher*, *s.n.* (NY); Monroe County, Section 4, Ida Twp. Jackman Road south of Ida West Road, *Easterly, N.W. 12015* (MICH); Emmet County, near Pellston, *Ehlers, J.H. 612* (MICH); Cheboygan County, Monroe Lake, *Ehlers, J.H. 1122* (MICH); Cheboygan County, Grapevine Point, Douglas Lake, *Ehlers, J.H. 1593* (MICH); Washtenaw County, Nichols Arboretum Ann Arbor, *Erlanson, C.O. 848* (MICH); Kent County, Grand Rapids, *Fallass, M.B. s.n.* (MICH); Wayne County, Detroit, *Foote, L.E. s.n.* (MICH); Hillsdale County, T5S, R1W, section 7; North side of U.S. 12. About 20 km NE of Hillsdale, *Fritsch, P.W. 807* (MICH); Montmorency County, Hillman Twp. T31N, R4E, Sec.24 NE1/4 1/2 mile east village of Hillman on Progress St, *Garlitz, D. 694* (MICH); Manistee County, Filer Twp; at corner of Pine and Water Streets on the S. side of Manistee River near the mouth; T21N, R17W, Sec.11 NE1/4, *Garlitz, D. 927* (MICH); Presque Isle County, Rogers Twp. T35N, R5E, Sec.21 SE1/4 at S.W. corner of M-68 and US-23 on west side of Rogers City, *Garlitz, D. 1006* (MICH); Gladwin County, Buckeye Twp. T18N, R1W, Sec.6 SW1/4 in front of grain elevators on south side of M-61 in the village of Gladwin, *Garlitz, D. 1114* (MICH); Saginaw County, James Twp. T11N, R4E, Sec. 5 SW1/4 on N. side of Swam Creek Rd. ca. 2 mi. S.W. of Saginaw city limits, *Garlitz, D. 1140* (MICH); Tuscola County, Vassar Twp. T11N, R8E, Sec. 7 SW1/4 at the Lions Park on the west side of village of Vassar, *Garlitz, D. 1227* (MICH); Isabella County, in the City of Mt. Pleasant Island Park. Union Twp. T14N, R4W, Sec. 15 NW1/4, *Garlitz, D. 1505* (MICH); Mecosta County, on the S. side of M-20 ca. 1/2 mi. E. of Maple St. ca. 2 1/2 mi. S.E. of Big Rapids. Big Rapids Twp. T15N, R9W, Sec. 18 SW1/4, *Garlitz, D. 1521* (MICH); Alpena County, Alpena Twp. T31N, R8E, Sec.21, SW1/4 on N. side of M-32 near Bagley St. on W. side of Alpena, *Garlitz, R. 2145* (MICH); Ontonagon County, Between old Hwy. M-28 and the north shore of Lake Gogebic, about 3 miles west of Bergland; T48N, R 43W, NW1/4 NE1/4 Section 12, *Garske, S.C. 0898-21* (MICH); St. Clair County, Fort Gratiot, 7 Aug 1872, *Gillman, H. s.n.* (NY); Menominee County, City of Menominee, *Grassl, C.O. 2500* (MICH); *Gray, A. s.n.* (K); Dickinson County, SW2, T42N, R29W 8 mi east of Sagola, *Guidinger, J.H. 2353* (MICH); Bay County, along Saginaw Bay, *Hamilton, C. & Krager, K. 4195* (MICH, MO); St. Joseph County, Mendon Twp, *Hanes, C.R. s.n.* (MICH); Van Buren County, Huzzy Lake, *Hanes, C.R. s.n.* (MICH); Washtenaw County, Ann Arbor, *Harrington, M.W. s.n.* (MICH); Benzie County, north of M-22 about 1/4 mile east of Deadstream Rd, *Hazlett, B.T. 2991* (MICH); Leelanau County, at former house site off Co. 669 near School Lake, *Hazlett, B.T. 3585* (MICH); Leelanau County, Near the foundation of the Sleeping Bear Inn, Glen Haven, *Hazlett, B.T. 3680* (MICH); Leelanau County, South Fox Island sec. 30, *Hazlett, B.T. 3779* (MICH); Leelanau County, South Manitou Island near new maintenance area, *Hazlett, B.T. 4333* (MICH); Schoolcraft

County, E1/2 Sec 32 T41N-R16W, *Henson, D. 990* (MICH); Delta County, S 1/2 Sec. 25 T40N-R23W ca. 3 1/2 mi. WSW of Gladstone, *Henson, D. 3200* (MICH); Dickinson County, Sec. 14 T39N-R30W ca. 1/2 mi. E Niagara, WI, *Henson, D. 3283* (MICH); Washtenaw County, Ann Arbor; Horti Botanici Universitatis Michiganensis, *Hermann, F.J. 1273* (MICH); St. Joseph County, Sandy northeast shore of Long Lake, near intersection of Lucas and Day roads, *Hyypio, P.A. & Schuyler, A.E. 3495* (MICH); Branch County, Quincy, *Kanouse, B.B. s.n.* (MICH); Washtenaw County, Cascade Glen Ann Arbor, *LaRue, C.D. s.n.* (MICH); Luce County, Lakefield Twp. 14 miles SW of Newberry, 1.5 miles W of Helmer, S side of CR 98 (Ten Curves Road AKA H-44), *MacKinnon, W.A. 2171* (MICH); Monroe County, Pte. Mouill?e State Game Area, *McDonald, M.E. 5436* (MICH); Lorain County, halfway between Lorain and Vermilion, along railway tracks parallel to Lake Michigan, 8 Sep 2000, *Nee, M. & Atha, D. 50966* (CORD, NY, WIS); Alger County, Pictured Rocks National Lakeshore road from Melstrand to Chapel Lake. sec. 17 T47N R17W, *Read, R.H. 312* (MICH); Ontonagon County, Keweenaw Peninsula, 1863, *Robbins, J.W. 74* (GH); Mecosta County, Chippewa Twp. Sec. 17 CE 1/4, T16N, R8W 21 Mi Rd at 105th Ave north to Long Lake, *Ross, S. 670* (MICH); Antrim County, Torch Lake, Hayo-Went-Ha Camp & vicinity, *Sargent, H.E. 3828* (MICH); Allegan County, ca. 8 miles W of Allegan, in the Allegan State Game Area. ca. 800 yards E of 48th Street and 135 yards S of 116th Avenue, *Schipper, R.G. s.n.* (MICH); Lenawee County, n. of Wolf Cr. e. of Bent Oak Hwy. w. of railroad, city of Adrian, *Smith, R.W. 819* (MICH); Lenawee County, Adrian; se. side of Island Park, *Smith, R.W. 1388* (MICH); Lenawee County, n. of Bent Oak Ct. Adrian (Adrian Twp.), *Smith, R.W. 1939* (MICH); Ottawa County, Ottawa Beach, *Sones, G.D. s.n.* (MICH); Washtenaw County, A.A. [=Ann Arbor], *Sudworth, G.B. 252* (MICH); Crawford County, At SW corner of junction of Three Sister Road and road leading to Range 13 (6 miles SW of Grayling). T26N R04W, Sec. 33 SW1/4 NE1/4, *Trana, T.D. 17937* (MICH); Kalamazoo County, Area 7: along and just S of Territorial Road, 1/3 mile W of Longman Road. T2S R9W, Sec. 11 SW 1/4 SE 1/4, *Trana, T.D. 18620* (MICH); Van Buren County, Magician Lake (or Cass County), *Umbach, L.M. 7210* (MICH); Emmet County, 0.5 mi. W of Bliss, *Voss, E.G. 1794* (MICH); Hillsdale County, NW1/4 sec.16, Moscow Tp. ca. 2.5 mi. WSW of Moscow, *Voss, E.G. 7977* (MICH); Emmet County, SW corner Jamet & DuJaunay Sts. Mackinaw City, *Voss, E.G. 12375* (MICH); Mackinac County, south of lighthouse, St. Helena Island, *Voss, E.G. 16808* (MICH); Genesee County, W of Fenton, NE side of Marl Lake, S of Owen Road between Lake Forest Drive and the lake; T5N R6E section 33 NW1/4 SW1/4, *Wade, C.A. et al. 1495* (MICH); Washtenaw County, 721 Mount Pleasant Avenue, ANN ARBOR, *Wagner, W.H. s.n.* (MICH); Washtenaw County, Cemetery Hill -Ypsilanti, *Walpole, B.A. s.n.* (MICH); Jackson County, Jackson, *Wright, J. & Bull, G.H. s.n.* (MICH). **Minnesota**: Nicollet County, Minneapolis, Swan Lake, Jul 1892, *Ballard, C.A. s.n.* (BM, E, GH, UC, US); Pine County, Sturgeon Lake, roadside by lake. 30 Jul 1976, *D'Arcy, W.G. 10522* (MO); Washaba County, Weaver, 28 Aug 1926, *Fassett, N.C. & Hotchkiss, N. 3394* (NY); Houston County, Jul 1912, *Freiberg, G.W. s.n.* (MO); Cottonwood County, 2-3 miles N of Mountain Lake, ca. 15 miles NE of Windom, ca. 25 miles W of St. James, 20 Aug 1978, *Harms, V.L. 24800* (GH); Winona County, sin. loc, Jul 1886, *Holzinger, J.M. s.n.* (US); Saint Louis County, Duluth, at the bus terminal Sec. 12, 9 Jul 1936, *Lakela, O.K. 1499* (NY, US); Hennepin County, Ft. Snelling, 3 Aug 1890, *Mearns, E.A. 492* (US); Dakota County, South St. Paul, on Fifth Avenue, 1/2 block S of Southview Boulevard, 4 Jul 1938, *Moore, J.W. & Moore, M.F. 10235* (UC); Lincoln County, Growing in abandoned field on island at north east end of Lake Benton, 22 Sep 1954, *Moore, J.W. & Ownbey, G.B. 22273* (US); Chippewa County, Montevideo, Aug 1892, *Moyer, L.R. s.n.* (NY); Goodhue County, Tower View Farm, near Red Wing, 9 Sep 1926, *Rydberg, P.A. 9626* (NY); Becker County, Lake Abbey, 1923, *Shunk, I.V. & Hylan, D.R. s.n.* (US); Cass County, Leech Lake, Squaw Point, 15 Aug 1958, *Stevens, O.A. 1994* (NY, UC); Houston County, Site 28 (Mississippi River navigation Pool 8, Mile 691-702.6), 9 Sep 1975, *Swanson, S.D. 2251* (MO, WIS); Otter Tail County, S side of Lake Orwell, *Williams, R.P. 2399* (MSC); Houston County, Island 14, Mississippi river mile #691.6, T103N R4W Sec 13, small site west side of main channel, 30 Jul 1975, *Ziegler, S.R. & Leykom, M.F. 2147* (MO); Houston County, Mississippi River; mi 694.6; W side of Island 19; large island W side of main channel, 26 Sep 1975, *Ziegler, S.R. & Leykom, M.F. 2941* (WIS). **Mississippi**: Clay County, D. Barr tract centered at lat/long; 33.786111 -88.849444; 33.786111 -88.849444, 25 Sep 2011, *Campbell, J. & Seymour, R. 1797-1-1572* (MISS); Lafayette County, Burney's Hill, 2 miles NE Oxford, 27 Jun 1947, *Hocking, G.M. 13* (NY); Monroe County, roadside of Hwy. 45 Alt. at ca 1 mi S of Chickasaw Co.

line, 7 Jul 1996, *MacDonald, J.R. & Warren, R. 9736* (USMS); Sunflower County, ca. 2 miles ENE of Moorehead [Moorhead], near Woods, 5 May 1976, *McDaniel, S. 20634* (MO); Jasper County, Ca 1 mi S of Newton County line, near MS15, clearcut, Jackson Prairie Region, T4N R11E S3, 13 Jun 1988, *McDaniel, S. 29971* (MO, NY); Jones County, 1.75 miles ESE of Sandersville, SE4, sect. 5, 29 Jun 1978, *Morgan, D. 862* (MO); Pike County, "the Old Cooper Place" ca. 3 miels W of Holmseville and ca. 7 miles E of McComb, 23 Oct 1984, *Pruski, J. & Pruski, R. 3023* (NY); Lowdnes County, 10 miles S of State College, 31 Jul 1956, *Ray, J.D. 7315* (GH); Wilkinson County, alogn new gas line right of way in gorges N of MS 24 E of Lessley and about 10 miles W of Woodville (T3N, R3W), 17 Jun 1993, *Thomas, R.D. et al. 140073* (NY). **Missouri**: Saint Louis County, Grain elevators and railroad along the Mississippi River at the E end of E. Prairie Ave, 20 Sep 1975, *Boufford, D.E. & Muehlenbach, V. 18017* (MO); Cass County, Bottoms, 20 Jun 1865, *Broadhead, G.C. s.n.* (MO); Cass County, Aug 1865, *Broadhead, G.C. s.n.* (MO); Barry County, Eagle Rock, 18 Sep 1896, *Bush, B.F. 143* (MO); Taney County, Swan, 24 Sep 1899, *Bush, B.F. 332* (MO); Jackson County, Sheffield, 16 Jun 1896, *Bush, B.F. 689* (MO); Jackson County, Courtney, 22 Sep 1903, *Bush, B.F. 1882* (MO); Jackson County, Sheffield, 23 Jun 1915, *Bush, B.F. 7673* (MO); Jackson County, Courtney, 25 Aug 1917, *Bush, B.F. 8098* (MO); Saint Louis City, Saint Louis, 4236 Arsenal St, St. Louis MO 63116; backyard, 20 Oct 2009, *Bussmann, R.W. 15965* (MO); Linn County, Pershing State Park; area around Camp Bluebird bluff along Locust Creek, 25 Jul 1985, *Castaner, D. & Priesendorf, T.A. 8646* (MO); Vernon County, Osage Prairie; Sec 4, T34N, R31W, 4 Sep 1989, *Chang, Y. 613* (MO); Jefferson County, Seckman, 19 Sep 1972, *Christ, A. s.n.* (MO, NY); Dade County, Bona Glade Natural Area, 1 mi W of Bona on Highway 215; Bona 7.5 minutes, 14 Sep 1985, *Collett, R.D. & Collett, A.J. 61* (MO); Saint Louis County, Glendale, 22 Aug 1956, *Comte, F. 772 a* (MO); Saint Louis County, Weed in garden, Glendale, 17 Apr 1963, *Comte, F. 4920* (MO); Franklin County, Mo. Bot. Gard. Arboretum, 10 Sep 1967, *Croat, T.B. 3965* (MO); Lincoln County, Lincoln County. North of Foley 2-3 miles along road to Lincoln Quarry, 6 Sep 1982, *Crosby, M.R. & Morin, N.R. 14571* (MO); Iron County, Crane Pond Shut-ins, 1 Oct 1969, *D'Arcy, W.G. 3875* (MO); Pemiscot County, Island Number 14, 9.86 airline miles NE of Hayti, west bank of the Mississippi River, 9 Oct 2011, *Davidse, G. 38552* (MO); Perry County, 6 airline miles east of McBride, at the northern end of Perry County Road 220, near the base of the levee, 9 Oct 2011, *Davidse, G. 38586* (MO); Saint Louis City, 6573 Scanlan Street, 17 Aug 2003, *Davidse, G. 39023* (MO); Grundy County, Crowder State Park, Tall Oaks Trail, 20 Sep 2013, *Davidse, G. 41078* (MO); Marion County, Hannibal, 7 Nov 1912, *Davis, J. 96* (MO); Marion County, Hannibal, 7 Jul 1913, *Davis, J. 371* (MO); Marion County, Hannibal city limits, 7 Jul 1913, *Davis, J. 3714* (MO); Lafayette County, banks of Missouri River, 2 Aug 1983, *DeLozier, P.E. 1356* (MO); Butler County, Poplar Bluff, *Dewart, F.W. s.n.* (MO); Butler County, Poplar Bluff, 7 Aug 1892, *Dewart, F.W. 53* (MO); St. Louis, Saint Louis, 1832, *Drummond, T. s.n.* (K); St. Louis, Saint Louis, *Drummond, T. s.n.* (K); St. Louis County, St. Louis, 1831, *Drummond, T. s.n.* (E); Saint Louis County, Opposite Pacific, *Eggert, H.K.D. s.n.* (MO); Saint Louis County, Sandy banks of the Mississippi, Oct 1845, *Engelmann, G. s.n.* (MO); Saint Louis County, Ladue, Epstein residence, 1017 Winwood Drive, 23 Sep 1983, *Epstein, D.B. s.n.* (MO); Pike County, Near Clarksville, 1948, *Etter, A.G. 246* (MO); Pike County, Near Clarksville, 1948, *Etter, A.G. 286* (MO); St. Louis County, Ranken, 8 Oct 1933, *Gallagher, J. s.n.* (MO); Ste. Genevieve County, Wolf Hollow, T29N R7E S15, Dandy MO & Bloomsdale MO-IL, 3 Sep 2001, *George, S. s.n.* (MO); Callaway County, T47N, R7W, SW 1/4, Sec. 3; W side of gravel road along sandstone bed of tributary to Prairie Branch (ca. ?? mi S of Williamsburg), 28 Aug 1988, *Gereau, R.E. & Brant, A.E. 2421* (MO); Wie es um St. Louis verkommt, mehrmals erwähut fand es Oktober 1838 um Spirit Lake im N. Westen. Wahrscheinlich auch für einheimisch immer schlanker und mehr niederliegend als in Deutschland. [Google translate -As it is about St. Louis deceased, it was mentioned several times in October 1838 around Spirit Lake in the N. West. Probably also for native always slimmer and more inferior than in Germany.], Oct 1838, *Geyer, C.A. s.n.* (W); Saint Louis County, 1889, *Glatfelter, N.M. s.n.* (MO); Saint Louis County, 1895, *Glatfelter, N.M. s.n.* (MO); Saint Louis County, Kirkwood, Sep 1913, *Greenman, J.M. & Greenman, M.T. 3734* (MO); Cass County, Powell Gardens, about 4 mi SE of Peculiar weed in the Iris Garden, 1 Jul 1991, *Henderson, N.C. 91-54* (MO); De Kalb County, Along U.S. Highway 36, about 4 mi W of Stewarsville, 4 Oct 1994, *Henderson, N.C. 94-1183* (MO); Franklin County, Riverfront Trail, 1.5 miles E of W trailhead, between trail and Missouri River, Washington. (T44N, R1W, SEC23, SE1/4, NW1/4), 7 Nov 2003, *Holmberg, N.J. 227* (MO);

Franklin County, Whiskey Creek Sheep Fram at 530 W. Whiskey Creek Road, 3.5 mi SW of Krakow MO, N side of barn, 7 Sep 2006, *Holmberg, N.J. 1950* (MO); Warren County, Robert Martin property, 0.55 mi W of house, E side of Tuque Creek, 5 mi NE of Marthasville, 24 Oct 2006, *Holmberg, N.J. 2038* (MO); Perry County, Seventy Six Conservation Area, 5 mi NE of Brazeau, 30 m W of Mississippi River, 30 m S of parking lot and N of Clines Branch, 29 Oct 2006, *Holmberg, N.J. 2085* (MO); Franklin County, Shaw Nature Reserve, Gray Summit; Dana Brown Woods, 0.5 mi SW of Dana Brown Overnight Center, 1 Jun 2006, *Holmberg, N.J. 2121 A* (MO); Saint Louis City, Saint Louis, 4007 Arsenal Road, across from Tower Grove Park, in back yard, 23 May 2007, *Holmberg, N.J. & Rigel, R. 2248* (MO); Crawford County, Blue Springs Creek Conservation Area, 5.3 miles SE of Bourbon MO, alogn Blue Springs Creek, 0.17 mi W of Meramac River, 16 Jul 2007, *Holmberg, N.J. & Baughman, K. 2317* (MO); Camden County, Toronto Springs Conservation Area, 1 mi. E of Toronto, S side of Wet Glaize Creek, 50 m N on parth from parking lot, 16 Sep 2007, *Holmberg, N.J. 2359* (MO); Knox County, 6 mi SE of Edina MO, S side of S[outh] Fabius River, along County Road 282, 19 Sep 2007, *Holmberg, N.J. & Baer, J. 2371* (MO); Franklin County, Shaw Nature Reserve, Gray Summit; Dana Brown Woods, 0.23 mi SW of Dana Brown Overnight Center, 20 m N of trail, 28 May 2007, *Holmberg, N.J. 2462* (MO); Franklin County, Shaw Nature Reserve, Gray Summit; Dana Brown Woods, 0.4 mi SW of Dana Brown Overnight Center on S slope of spur, 7 Jun 2007, *Holmberg, N.J. 2497* (MO); Washington County, Anthonies Mill, Sherri DeRousse and Andy property in a resort development, 6 Jul 2008, *Holmberg, N.J. 2696* (MO); Warren County, Razor Hollow Natural Area, in the Daniel Boone Conservation Area, 6 miles S of Jonesburg, 650 m SE of Tower Road at trail head for multi-use, 8 Aug 2008, *Holmberg, N.J. 2722* (MO); Washington County, Kingston Access on the Mineral Fork, 600 m N of Washington State Park, 15 Oct 2012, *Holmberg, N.J. 4389* (MO); Chariton County, Swan Lake National Wildlife Area, along W side of Teal Road, W slope of berm, 13 Jun 2014, *Holmberg, N.J. et al. 5058* (MO); Butler County, Godwin's Bluff, 22 Sep 1992, *Hudson, H.S. s.n.* (MO); Butler County, S of Military Crossing Cemetery near railroad, 12 Sep 1992, *Hudson, H.S. s.n.* (MO); Butler County, Shady Lane, Poplar Bluff ditch beside street, 5 Oct 1992, *Hudson, H.S. s.n.* (MO); Wayne County, Cedar Bluff on Wappapello Lake, W of Highway D and S of Highway 60 on top of bluff toward the back side, 4 Aug 1993, *Hudson, H.S. 116* (MO); Butler County, Countyy Road 428, just before the bridge on Ten Mile Creek growing on a small gravel bar of a spring-fed tributary to Ten Mile Creek, 15 Oct 1993, *Hudson, H.S. 354* (MO); Phelps County, Jerome, 7 Oct 1914, *Kellogg, J.H. s.n.* (MO, NY); Saint Louis County, Allenton, 21 Jul 1922, *Kellogg, J.H. s.n.* (MO); Dunklin County, Kennett, 1 Sep 1930, *Kellogg, J.H. s.n.* (MO); Phelps County, Jerome, 2 Sep 1928, *Kellogg, J.H. 490* (MO); Philpr. County, Jerome, 2 Sep 1928, *Kellogg, J.H. 1990* (MO); Franklin County, Pacific rocky banks, 14 Jun 1932, *Kellogg, J.H. 25993* (MO); Cape Girardeau, E side of Apple Creek Conservation Area, ca. 5 mi E of New Wells on N side of County Highway CC, along Apple Creek, 30 May 2000, *Laatsch, J.R. s.n.* (MO); Saint Louis County, Allenton along Meramec River bank, 13 Aug 1933, *Lodewycks, M.C. 32* (MO); Greene County, Fossnight Park, Springfield, 10 Jan 1938, *McMillon, W. s.n.* (F); Saint Louis City, 3457 Shenandoah Ave, 11 Aug 1998, *Miller, J.S. 9783* (MO); Sainte Genevieve County, Hickory Canyon Natural Area, L-A-D Foundation Property, ca. 2 mi N of Sprott, via dirt road off County Road EE, 27 Jul 1989, *Mohlenbrock, R.H. 9240* (MO); Saint Louis City, South of the right-of-way of the Frisco Railroad, on a large open area between Kingshighway and the Frigidaire Warehouse, 5 Sep 1954, *Muehlenbach, V. 392* (MO); Saint Louis City, Between the eastern shed of Evans and Howard Sewer Pipe Company and the Frisco RR, W of Kingshighway, 9 Sep 1956, *Muehlenbach, V. 1079* (MO); Saint Louis City, Right-of-way of the Manufacturers Railway; classification tracks, north of the round house, Arsenal Street, 10 Jul 1965, *Muehlenbach, V. 2493 B* (MO); Saint Louis City, In the Public River-Rail-Truck Terminal of the City of St. Louis, served by the Terminal Railroad Association, south of the most northern warehouse, 2 Jul 1971, *Muehlenbach, V. 3594* (MO); Saint Louis City, Right-of-way of the Terminal Railroad Association, southern embankment, opposite the water tower of the Chevrolet plant, 3 Nov 1971, *Muehlenbach, V. 3721* (MO); Saint Louis City, Right-of-way of the Missouri-Pacific Railroad, along the siding beside the foot of Alaska Avenue east of Tesson Street, opposite Monsanto's Carondelet plant, 8201 Idaho Avenue, 6 Nov 1971, *Muehlenbach, V. 3728* (MO); Saint Louis City, North St. Louis freight yard of the Burlington Northern Railroad, south of the car repair shop, 10 Sep 1972, *Muehlenbach, V. 3793* (MO); Saint Louis City, St. Louis Terminal Corporation (served by the Terminal Railroad Assoc.) N of the office building few specimens, 1 Aug 1980,

Muehlenbach, V. 4395 (MO); Franklin County, RR south of Gainesville, 22 Jun 1937, *Murrill, W.A. s.n.* (MO); Saint Louis County, Ladue: Litzsinger Road Ecology Center, Litzsinger Road, 20 Aug 1992, *Ochs, C. 238* (MO); Jasper County, Common in waste places. Webb City, 17 Jun 1902, *Palmer, E.J. 257* (MO); Jasper County, Webb City, 4 Sep 1910, *Palmer, E.J. 3109* (MO); Jasper County, 3 miles NW of Sarcoxie, 18 Sep 1910, *Palmer, E.J. 3208* (MO); Jasper County, Rocky woods, Neck City, 25 Sep 1910, *Palmer, E.J. 3220* (MO); Jasper County, Webb City, 2 Oct 1910, *Palmer, E.J. 3237* (MO); Saint Charles County, Spur dike access Road State Route 67 by New Lock and Dam 26 on Mississippi River, Aug 1987, *Raechal, L.J. 29* (MO); Jefferson County, 6.5 mi (10.5 W of DeSoto). T39N, R3E, Sec. 11. Woods along dirt road just above and W of Parker Creek, ca. 0.3 mi (500 m) NNE of its confluence with Big River. Elev. 580 ft (175 m), 27 Jul 1986, *Raven, P.H. 27250* (MO); Jefferson County, 6.5 mi (10.5 Km) W of Desoto. Township 39 N. Range 3 E. Section 11. Along Big River c. 50 m below (W of) mouth of Parker Creek, 17 Sep 1988, *Raven, P.H. & Raven, T.E. 27702* (BM, GH, MO, UC); Clark County, Battle of Athens State Historic Site, along bank of Des Moines River, 21 Aug 2012, *Schuette, B. 3088* (MO); Cole County, Jefferson City, parking lot on south side of E Dunklin St. at intersection with Cherry St, 24 Oct 2012, *Skornia, E. & Hershey, D. 60* (MO); Spesutie Island. [interpreted, illegible -but Spesutie Island is a place in Harford County MD], *Smith, J.D. s.n.* (US); Ozark County, Ca. 2.0 mi ESE of Crossroads, steep, E-facing bluff above W bank of North Fork River; NW of North Fork Springs, 20 Sep 1989, *Smith, T.E. 2997* (MO); Maries County, Ca/ 5.0 mi WSW pf Argyle, Argyle Quad. On property of Tim Barnhart, a dentist in Westphallia, 22 Jun 2007, *Smith, T.E. 4415* (MO); Greene County, Woods near Hayo Station. Vicinity of Springfield, 31 Aug 1911, *Standley, P.C. 8517* (US); Greene County, Vicinity of Willard, limestone cliffe, 30 Aug 1912, *Standley, P.C. 9649* (US); Jefferson County, In shaded low ground, below line of St. Peter sandstone bluffs, near Meramec river, about 5 miles southeast of Pacific, 4 Sep 1927, *Steyermark, J.A. 841* (MO); Scott County, ca. 3 miles E of Morely and 0.6 mile S of Highway C on County Road 405, 3 Sep 1998, *Summers, B. et al. 8869* (MO); Howell County, Peggie L. Skinner farm on COunty Road 6070, 0.5 mi SW of West Plains city limits, 11 Oct 2012, *Summers, B. 10625* (MO); Howell County, Ca. 4 1/2 mi W of Willow Springs on County Road NW 571, down stream from Lowe's Ford on Indian Creek, 11 Sep 1993, *Summers, J.W. 6442* (MO); Oregon County, Greer Spring Special Management Area, Mark Twain National Forest, bluff top on Hurricane Creek, about 1/4 mi N of the Eleven Point River base, 28 Sep 1994, *Summers, J.W. 7192* (MO); Saint Louis County, 8613 Neir Lane in Affton, N of Gravois Road, 31 Jul 1998, *Summers, J.W. 8829* (MO); Shannon County, Devil's Well, Ozark National Scenic Riverways; ca. 1 1/2 mi S of Highway KK inside basin of Devil's Well, 4 Aug 1998, *Summers, J.W. 8831* (MO); Scott County, Ca. 3 mi E of Morely and 0.6 mi S of Highway C on County Road 405, 3 Sep 1998, *Summers, J.W. 8869* (MO); Scott County, Hickory Grove Cemetery, ca. 3 mi E of Morley on S side of Highway C at its junction with County Road 405, 3 Sep 1998, *Summers, J.W. 8888* (MO); Howell County, Eldon Summers Farm; 7 1/2 mi E of West Plains on Highway ZZ, 6 Sep 1998, *Summers, J.W. 8919* (MO); Jefferson County, Missouri Department of Conservation, Pacific Palisades Conservation Area, ca. 1 mi S of Pacific on Highway F, 3 Oct 2001, *Summers, J.W. 9908* (MO); Saint Louis County, Along Meramac River, between river and Marshall Rd, opposite ball park, 1.2 mi SW of jct of Big Bend Rd with Marshall Rd, ca. 2 mi E of Valley Park, 3 Oct 1987, *Taylor, M.S. 5701* (MO); Saint Charles County, Along River Road where RR crosses from N side to S side of road, ca. 2 air miles SW of St. Charles. T46N R4E Sec. 60. Kempville quad, 10 Jul 1988, *Taylor, M.S. 5816* (MO); Dunklin County, Indian Hill, Buffalo Island, E. Bertig, 28 Sep 1897, *Trelease, W. 499* (MO); Taney County, Gravel bed of White River, 7 Aug 1897, *Trelease, W. 500* (MO); Jasper County, Turney Creek. Joplin, 7 Oct 1897, *Trelease, W. 501* (MO); Saint Francois County, Flat River, 13 Oct 1897, *Trelease, W. 539* (MO); Madison County, SW edge of Venice on IL3, Mississippi River bottoms, 3 Oct 1994, *Utech, F.H. 94-2249* (MO); Bollinger County, Weaver farm 2 at NE corner of junction Hwy 72 and County Rd 223, in old field at W edge of property, 25 Sep 2010, *Weaver, J. 146* (MO); Jefferson County, Kimmswick, 15 Jul 1885, *Wislizenus, F.A. 334* (MO); Saint Louis County, Jul 1833, *Without Collector 320* (MO); Saint Louis City, 4500 block of Shaw Avenue, between Vandeventer Road and Kingshighway, SW portion of Missouri Botanical Garden's Monsanto Research Center, near fence along railroad tracks scattered, stems hairy, 4 Aug 2003, *Yatskievych, G.A. 03-87* (MO); Saint Charles County, Riverlands Environmental Demonstration Area (REDA), [US Army Corps of Engineers, St. Louis District]. Along the Mississippi River, directly across from Alton, Illinois, adjacent to the Melvin Price Lock

and Dam (26). Teal Pond, 25 Sep 1992, *Zhu, G.H. 1403* (MO). **Montana**: McCone County, Circle, 1 Oct 1951, *Cade, L.P. s.n.* (MONT); Musselshell County, Roundup, 18 Sep 1967, *Clausen, Z. s.n.* (MONT); Wibaux County, [No locality given.], 9 Sep 1969, *Weber, G.F. s.n.* (MONT). **Nebraska**: Webster County, Red Cloud, 13 Sep 1909, *Bates, J.M. 5023* (GH); Burt County, 5 miles S of Decatur on Hwy 73 and 1.5 miles W, sec. 35 T23N R10E, 5 Oct 1975, *Churchill, S.P. 6847* (MO, NY); Holt County, Northeastern Nebraska. Paddock, 27 Jul 1893, *Clements, F. 2789* (GH, US); Otoe County, Along state hwy. 2, ca. 17.5 mi W of Nebraska City, 12 Aug 1984, *Miller, J.S. & Lowry, P.P. 1975* (MO); Holt County, 28km S of O'Neill, Hwy 95 at Hwy 281 junction, 17 Jul 2014, *Nee, M. 61306* (MO); Holt County, SE side of O'Neil, Elms Motel, 19 Jul 2014, *Nee, M. 61357* (BM, MO); Arthur County, Arapaho Prairie, in bottom of Rose Blowout (T18N R39W, Sect. 31, 32), 11 Jul 1978, *Vescio, L.S. & Mahoney, D.L. 388* (NY); Cass County, Weeping Water, Jul 1885, *Williams, T.A. s.n.* (US). **New Hampshire**: Strafford County, Rochester, Sep, *Bartow, E. s.n.* (NEBC); Cheshire County, Hinsdale, Sep, *Batchelder, C.F. s.n.* (NEBC); Rockingham County, Seabrook Dunes, 14 Sep 1982, *Bertrand, T. & Dunlop, D. CZ-615* (NHA); Sullivan County, Plainfield, valley of Conn[ecticut] River, Aug, *Cowden, M.C. et al. 5370* (NEBC); Cheshire County, Walpole, Jul, *Fernald, M.L. 206* (NEBC); Grafton County, Haverhill, Aug, *Fernald, M.L. 15566* (NEBC); Rockingham County, Rye, Isle of Shoals, Star Islands, Aug, *Gates, J. s.n.* (NEBC); Sullivan County, Cornish, Altai Hill, sugar trail, Yatsevitch Forest, Aug, *Grunden, B. et al. 12-172* (NEBC); Rockingham County, Kingston, North Danville Road, Sep, *Harris, S.K. 26089* (NEBC); Strafford County, Lee, Aug, *Hodgdon, A.R. 2409* (NEBC); Strafford County, Durham, near Colony Cove, Aug, *Hodgdon, A.R. 4226* (NEBC); Grafton County, Lebanon, Sep, *Kennedy, G.G. s.n.* (GH); Hillsborough County, Pelham, Oct, *Knowlton, C.H. s.n.* (NEBC); West Lebanon, Jul, *Pease, A.S. 1167* (NEBC); Grafton County, Bath, Aug, *Pease, A.S. 29706* (NEBC); Cheshire County, Fitzwilliam, E slope of Little Horsebrook, Sep, *Rand, E.L. & Robinson, B.L. 651* (GH); Sullivan County, Cornish, Altai Hill, sugaring area, Yatsevitch Forest, Aug, *Schori, A. & Grunden, B. 12-165* (NEBC); Cheshire County, Walpole, on the Conn[ecticutt], Jul, *Williams, E.F. s.n.* (GH); Sullivan County, Meridan, *Wood, A. s.n.* (NY). **New Jersey**: Cape May County, Vicinity of Cape May, 23 Sep 1920, *Abbott, W.L. 30* (US); Ocean County, Forked River, 18 Sep 1932, *Alexander, E.J. s.n.* (NY); Cape May County, Cape May Point, 15 Aug 1927, *Benner, W.M. s.n.* (NY); Cape May County, Wildwood, 31 May 1897, *Bicknell, E.P. s.n.* (NY); Cape May County, Cape May, Schellinger's landing, 29 Aug 1917, *Gershov, A. 600* (GH); sin. loc, *Knieskern, P.D. s.n.* (GH); Avalon, Seven Mile Beach, 10 Sep 1936, *Langenheim, J.H. 88* (GH); Bergen County, Palisades, 14 Aug 1865, *Leggett, W.H. s.n.* (NY); Cumberland County, Moores Beach, (Robinsons Beach) S of Delmont, shore of Delaware Bay, 22 Jun 1933, *Long, B. 40687* (GH); Gloucester County, Fancy Hill, SW of Gloucester City, 27 Sep 1899, *MacElwee, A. s.n.* (GH, NY); Cape May County, Five-Mile Beach, 3 Oct 1899, *MacElwee, A. 1449* (MO, NY); Camden County, Gloucester, 17 Oct 1860, *Maich, J.M. s.n.* (NY); Camden County, Camden, Aug 1879, *Martindale, I.C. s.n.* (NY); Repampo, 5 Sep 1914, *McKenzie, K.K. 6277* (NY); Warren County, Marksboro, Sep 1915, *McKenzie, K.K. 6820* (NY); Morris County, Halsey Island, Lake Hopatcong, Lake Hopatcong, Jefferson, 26 Aug 1990, *Mehrhoff, L.J. 13782* (CONN); Passiac County, Notch Mountain, near Paterson, 8 Sep 1889, *Nash, G.V. 302* (NY); Somerset County, Rocky Hill, Montgomery Avenue, garden at residence, 9 Sep 2012, *Nee, M. 59444* (NY); Bergen County, Carlstadt, 28 Jun 1883, *Niederer, A. s.n.* (NY); Bergen County, Carlstadt, 1878, *Oehler, J. s.n.* (NY); Cape May County, Marsh near Sea Isle City, 13 Jul 1957, *Reed, C.F. 39206* (MO); Camden County, Ferry Avenue, foot of Jackson St, 5 Jul 1979, *Reed, C.F. 141504* (MO); Hudson County, Weehawken, 11 Jul 1877, *Schrenk, J. s.n.* (NY); Bergen County, Roadside, Fort Lee, 15 Jul 1894, *Sickle, W.M. van, s.n.* (US); Cape May County, Cape May Point, 25 Aug 1901, *Stewart, C.C. 2175* (NY); Ventnor, near Savannah Avenue, 11 Jul 1924, *Stone, H.E. s.n.* (GH); Union County, Plainfield, Jul 1877, *Tweedy, F. s.n.* (YU); Mercer County, Princeton, 9 Aug 1876, *Without Collector s.n.* (NY); Bergen County, Carlstadt, Jul 1883, *Without Collector s.n.* (NY). **New Mexico**: Mogollan Mountains, Sep 1881, *Rusby, H.H. s.n.* (MICH, MO). **New York**: Putnam County, ca. 7 km (by air) W of New York/Connecticut border and ca. 10 km (by air) N of Putnam/Westchester county border, Ice Pond Conservation Area, E of Ice Pond Road, N of Route 312, 13 Jul 2008, *Atha, D.E. & Abramsons, D. 6419* (NY); Putnam County, ca. 7 km (by air) W of New York/Connecticut border and ca. 10 km (by air) N of Putnam/Westchester county border, Ice Pond Conservation Area, E of Ice Pond Road, N of Route 312, 27 Jul 2008, *Atha, D.E. 6514* (NY); New York City, Central Park, 79th Street traverse, between 78th and 79th Streets and between 7th and

8th Avenues, 2 Jul 2014, *Atha, D.E. & Alvarez, R. 14687* (NY); Westchester County, Sing Sing, [near Ossining], 5 Sep 1888, *Barnhart, J.H. 229* (NY); Bronx County, Van Cortlandt Park, and vicinity, 4 Oct 1896, *Bicknell, E.P. 7710* (NY); Nassau County, Long Island, Long Beach, 26 Aug 1913, *Bicknell, E.P. 7715* (NY); Bronx County, bridge across Northern Railway below van C[ortland] Park, 4 Oct 1896, *Bicknell, E.P. 7718* (NY); Bronx County, Jerome Park Reservoir, 30 Sep 1901, *Burnham, S.H. 642* (GH, NY); sin. loc. [see Brittonia 34(4) 1982 -Grimes & Keller], 17 Aug 1887, *Child, C.M. s.n.* (NY); Chautauqua County, Lake Chautauqua, Bemus Point, 3 Aug 1896, *Churchill, J.R. s.n.* (GH); Tompkins County, Ithaca, 25 Jul 1885, *Coville, F.V. s.n.* (US); Westchester County, Bryn Mawr Park, [Yonkers], 29 Aug 1903, *Curtis, C.C. s.n.* (NY); Westchester County, Port Chester, 6 Aug 1905, *Curtis, C.C. s.n.* (NY); Orange County, Goshen, 11 Sep 1912, *Denslow, H.M. s.n.* (NY); Orange County, Craigville, 21 Aug 1922, *Denslow, H.M. s.n.* (NY); Staten Island, Richmond Hill, 26 Sep 1903, *Dowell, P. 8653* (GH); Tompkins County, Ithaca, behind Soils Building, Cornell University Campus, 16 Jul 1915, *Eames, A.J. & Thomas, C.C. 4925* (GH); Cayuga County, Ledyard, lower Paine's Creek Ravine, 3 Aug 1919, *Eames, A.J. et al. 12866* (GH); Westchester County, New Rochelle, Jun 1878, *EGK, s.n.* (NY); Suffolk County, Long Island, Easthampton, 16 Oct 1919, *Ferguson, W.C. s.n.* (NY); Suffolk County, Long Island, Montauk, 10 Aug 1924, *Ferguson, W.C. 3186* (NY); Suffolk County, Long Island, Point of Woods, 20 Aug 1924, *Ferguson, W.C. 3240* (NY); Bronx County, New York Botanical Garden, Bronx Park, 11 Jul 1939, *Gilly, C. 87* (NY); Madison County, Lenox, border Oneida Lake, 20 Sep 1903, *Haberer, J.V. 638* (GH); Monroe County, town of Sweden, Village of Brockport, 14 Chappell St, 29 Jul 1975, *Hammond, H.D. 7812* (NY); Tompkins County, Ludlowville, "Ludloville", 5 Aug 1897, *Hastings, G.T. s.n.* (NY); Westchester County, Greenburgh, Bronx River, just N of Cemetery Road bridge, 25 Sep 1980, *Holmgren, N.H. 9756* (NY); Westchester County, White Plains, Bronx River, along the footpath just NE fof the PArkway overpass, 26 Oct 2003, *Holmgren, N.H. 15073* (NY); New York City, Coney Island, Sep 1861, *Leggett, W.H. s.n.* (NY); Rockland County, Suffern, dirt road east of Wanamaker Road, Ramapo Township, 5 Sep 1961, *Lehr, J.H. 648* (NY); Suffolk County, Ocean Beach, 15 Jul 1914, *Lighthipe, L.H. s.n.* (NY); Chemung County, [sheet has two stems, one from Elmira and the other from "Big Island Chen. R. Fr. Sept 14 1888"] Upper Waters of the Susquehanna, 7 Jul 1896, *Lucy, T.F. 7843* (NY); Bronx County, New York Botanical Garden, along W side of Bronx River, N side of Hester Bridge, 27 Sep 2006, *McClelland, D. 357* (NY); Bronx County, New York Botanical Garden, Twin Lakes parking lot, 16 Sep 2012, *Meyer, R.S. 403* (NY); Bronx County, Bronx, 5 Sep 1946, *Moldenke, H.N. 18367* (NY); Suspension Bridge, 29 Aug 1875, *Morong, T. s.n.* (NY); Bronx County, Academy of Mount St. Ursula, W of intersection of Bedford Park Boulevard and Marion Avenue, 15 Jun 2011, *Naczi, R.F.C. 13727* (NY); Dutchess County, Millbrook, Cary Arboretum, 8 Sep 1984, *Nee, M. 30111* (BM, COL, CORD, G, GH, K, MEXU, MO, NY, US, WIS); Bronx County, Bronx, at the New York Botanical Garden, 16 Jul 1993, *Nee, M. 43613* (CORD, MEXU, MO, NY); Bronx County, Bronx, Mosholu Parkway and Kazimiroff Blvd, between the Metro North railroad lines and the New York Botanical Garden, 2 Aug 2005, *Nee, M. & McClelland, D. 53366* (BM, MO, NY); Dutchess County, 4 miles SE of Rhinebeck; the Amy Goldman research vegetable garden, 9 Sep 2005, *Nee, M. & Goldman, A. 53508* (NY); Bronx County, Bronx, New York Botanical Garden, near the service entrance to the Enid Haupt Conservatory and demonstration vegetable garden, 10 Aug 2010, *Nee, M. & Knapp, S. 57199* (BM, MO, NY); Suffolk County, Long Island Horticultural Research Laboratory, 3059 Sound Avenue, 6.75 km NW of center of Riverhead, 24 Aug 2012, *Nee, M. 59426* (NY); Bronx County, New York Botanical Garden, coniferous forest, 17 Sep 1978, *Petrino, M. 7* (NY); Lawrence County, Canton, 25 Jul 1914, *Phelps, O.P. 843* (GH); St. Lawrence County, De Kalb, 4 Aug 1914, *Phelps, O.P. 844* (GH, NY); Lawrence County, Canton, 27 Jul 1914, *Phelps, O.P. 845* (GH); Yates County, Penn Yan, *Sartwell, H.P. s.n.* (GH, K, NY); Battle Island County, Oswego, 23 Aug 1883, *Sheldon, C.S. 5976* (UC); Suffolk County, Fishers Island, 10 Apr 1920, *St John, H. 2902* (GH); Tompkins County, Ithaca, Cornell University campus, Aug 1917, *West, F.G. s.n.* (WIS); Cortland County, Cortland, N of Chicago Bog, 12 Sep 1917, *Wiegand, K.M. 8719* (GH); New York City, Staten Island, Crooke's Point, 8 Sep 1915, *Wilson, P. s.n.* (NY); Rockland County, Pailsades, 26 Sep 1915, *Wilson, P. s.n.* (NY); Westchester County, Black Pond, sin. loc, *Without Collector s.n.* (NY); Bronx County, New York Botanical Garden, Native Plant Garden, east wall, 10 Sep 1967, *Yarrow, E. s.n.* (NY). **North Carolina**: Anson County, Jones Creek, 3.5 miles SW of Pee Dee on S.C. 85, 21 Sep 1956, *Ahles, H.E. & Leisner, R.S. 19310* (GH); Biltmore, 17 Jul 1897, *Biltmore Herbarium*, *912 b*

(GH, NY, W); New Hanover County, Wrightsville Beach, on Summer Rest Road, 20 May 1973, *Boufford, D.E. & Ahles, H.E. 9039* (A); Granville County, Hester, 24 Jul 1937, *Godfrey, R.K. 2001* (GH); Carteret County, Beaufort, 20 Jul 1938, *Gray, R. s.n.* (NY); Haywood County, in moist soil shaded by big rock near Pigeon River, 21 Jun 1935, *Hendrix, O.R. B-55* (NY); Hyde County, Pig pen along US 264 about one half mile west of jct. of NC 94 at Mattamuskeet, 30 Jul 1970, *Leonard, S.W. & Radford, A.E. 3306* (BM, E, GH, H, NY, UBC, UC); New Hanover County, Bald Head Island, "Federal Road" N of Lifesaving Station, 5 Nov 1988, *Pyne, M. & Wentworth, T. 88-225* (GH); Wilkes County, Field, North Wilkesboro near Yadkin River, 5 Aug 1956, *Radford, A.E. 15136* (E); Carteret County, Radio isl. M. Marehead city, 20 May 1979, *Reed, C.F. 140691* (MO); Lee County, 1 mile south of Juniper Spring Church, 7 Jun 1958, *Stewart, S. 459* (BM); Chatham County, White Pines Natural Area, Newton's driveway, 16 Sep 1988, *Swab, E.C. 607* (NY); Gaston County, Crowders Mountain State Park, 16 Aug 2011, *Tompkins, R. s.n.* (UNCC). **North Dakota**: Benson County, Leeds, 26 Aug 1907, *Lunell, J. s.n.* (NY); Benson County, Lake Ibsen, 3 Aug 1910, *Lunell, J. s.n.* (NY); Benson County, Peninsula of Lake Ibsen, 3 Aug 1910, *Lunell, J. s.n.* (US); McLean County, Dogden Buttes, 12 Sep 1956, *Stevens, O.A. & Moir, D.R. 1668* (UC); Cass County, Chaffee, Maple River, 21 Sep 1958, *Stevens, O.A. 2032* (UC). **Ohio**: Licking County, Fredonia, 19 Jul 1888, *Barrick, E. s.n.* (NY); Ross County, sin. loc, 16 Aug 1937, *Crowl, G.S. s.n.* (NY); Hamilton County, sin. loc, 9 Aug 1889, *Dickerson, L. 43* (NY); Franklin County, Columbus, *Drake, L.S. s.n.* (CCNL); Cuyahoga County, Cleveland, Wade Park, 22 Sep 1911, *Dreisbach, R. 3211* (MICH); Wood County, Henry, 2 mi NE of Hoytville on Oil Center Rd, *Fuess, F.W. 23* (MSC); Lorain County, Oberlin, 27 Sep 1890, *Kofoid, C.A. s.n.* (GH); Clark County, Garden of the Russel Leonard Farm about 2 mi. n. w. of Lawrenceville, 18 Sep 1930, *Leonard, G.M. & Leonard, E.C. 15895* (US); Hamilton County, near Cincinnati, 25 Jun 1890, *Lloyd, C.G. s.n.* (MO); Hamilton County, near Cincinnati, 31 Jul 1882, *Lloyd, C.G. 2196* (MICH); Franklin County, Prairie Township, E bank of Big Darby Creek just off Rte. 40, 10 Sep 1991, *Lowden, R.M. 4816* (NY); Hamilton County, sin. loc, *Matthes, B. 305* (W); Erie County, Berlin Heights, 31 Jul 1914, *McDaniels, L.H. 140* (NY); Lorain County, Elyria, Ohio, 30 Sep 1888, *Metcalf, M.M. s.n.* (MO); sin. loc, *O'Neal, C.E. 41-141 -1* (NY); Butler County, Along the edge of Jess McQuigg's garden, Springfield Rd. between Indian Creek Rd. and Weaver Rd. west of Reily. Collected at 11:30 pm with the aid of headlamps and two glasses of wine. Collected specimen was less than 1 ft in height. small weed (12 cm i, 13 Oct 2016, *Reece, E. & McQuigg, J. 22* (MU); Butler County, Middletown, Hawthorne Hills, 15 Sep 2007, *Sollmann, A. 12* (W); Brown County, Georgetown, 27 Sep 1930, *Stephenson, B.C. s.n.* (BM); Darke County, Drew Woods Sate Nature Preserve, Brown Twp. sect. 9, Zumbrum Road, 14 Sep 1985, *Vincent, M.A. 1493* (MEXU); Lucas County, Swanton Twp. Whiteshouse Quad, 0.34 mile east of Berkey Southern Road (SR 295) and just north of Airport Road (SR2), SE1/4 of SW1/4 sec 3, 21 Sep 2007, *Walters, T.L. 11985* (MO); Portage County, Garrettsville, 26 Aug 1899, *Webb, R.J. 393* (GH). **Oklahoma**: Bryan County, Vicinity of Durant, 1931, *Blain, W.L. 171* (US); Bryant County, vicinity of Durant, 1931, *Blain, W.L. 173* (US); Le Flore County, near Page, 11 Jul 1914, *Blakely, O.W. 1508* (GH); Lefore County, near Page, 11 Jul 1914, *Blakley, O.W. 1508* (K); Creek County, Sapulpa, 22 Jul 1894, *Bush, B.F. 390* (MO); Comanche County, Fort Sill, 6 Jun 1916, *Clemens, J. 11762* (GH); Payne County, A. & M. Campus, Stillwater, 10 Jul 1939, *Graumann, H. 80* (MO); Payne County, on OSU [Oklahoma State University] farm property on W. Virginia Road, 17 Sep 2015, *Harris, A.J. & Post, A. 2015-05* (US); McCurtain County, flood plain of Little River, 7 miles N of Idabel, 20 May 1944, *Hopkins, M. et al. 408* (GH); Johnston County, near Tishomingo, Apr 1916, *Houghton, H.W. 3526* (GH); McCurtain County, Near Idabel, 25 May 1916, *Houghton, H.W. 3774* (GH, MO); McCurtain County, near Shawneetown [near Idabel], 26 May 1916, *Houghton, H.W. 3852 1/2* (GH); Comanche County, Wichita Mountains Wildlife Refuge, Rocky soil, W. end of Big Four Mountain, 19 Jun 1939, *McMurry, F.B. 702* (US); Murray County, Platt National Park, 14 Jun 1935, *Merrill, G.M. 651* (NY); Murray County, Platt National Park, Sulphur, 23 Jul 1935, *Merrill, G.M. 969* (NY); McIntosh County, Fountainhead State Park along Eupaula Lake, 12 Jun 1982, *Nee, M. 24369* (MO, NY); Payne County, Stillwater, A[gricultural] & M[echanical College] Hort. Garden, 3 Aug 1939, *Rose, L.E. s.n.* (UC); Swanson County, near Mountain Park, 23 Jun 1913, *Stevens, G.W. 1298* (GH, NY); Kay County, near Tonkawa, 4 Aug 1913, *Stevens, G.W. 1831* (GH); Ottawa County, by Spring River near Ottawa, 29 Aug 1913, *Stevens, G.W. 2529* (GH); Comanche County, Medicine Creek at 4-Mile crossing, Fort Sill Reservation, 16 Aug 1989, *Thompson, R.A. et al. S-0735* (MO); Oklahoma County, Oklahoma City,

25 Aug 1939, *Waterfall, U.T. 1679* (GH); Woodward County, Hal and Fern Cooper Wildlife Management Area; central portion of North Pasture; northeast of stock tank, 4 Jun 2007, *Winter, S. & Richards, J. 1916* (ASU). **Pennsylvania**: Philadelphia County, Manaynuck, 23 Sep 1888, *Brinton, J.B. s.n.* (UC); Centre County, Apt. 12M Graduate Circle, Penn State Graduate Housing, 11 Aug 1996, *Cook, L. 527* (GH); Lehigh County, Allentown, 8 Sep 1898, *Dowell, P. 8651* (GH); Pittsburgh County, Hays, Penna, 29 Aug 1963, *Farnsworth, N.R. SW-397* (F); Lancaster County, At Lancaster, End of W. Walnut St, 24 Sep 1900, *Heller, A.A. s.n.* (US); Chester County, West Chester, 1836, *Jeffries, W.W. s.n.* (US); Chester County, West Chester, 1836, *Jeffries, W.W. s.n.* (US); Lancaster County, Penn Township, Dead End Road, state game land 156, 29 Jul 2012, *Longbottom, W.D. & Velsir, G. van 17840* (MO, NY); Northampton County, Easton, *Porter, T.C. s.n.* (US); Philadelphia County, south Philadelphia dump at foot of Pattison Street at Front Street, 5 Jul 1979, *Reed, C.F. 5407* (MO); Northampton County, 1/4 mile N of Glendon, 11 Sep 1946, *Schaeffer Jr, R.L. 25304* (W); Delaware County, sin. loc, *Without Collector s.n.* (US); Berks County, "found at Reading" [{Penn?], *Wolle, J. s.n.* (GH). **Rhode Island**: Washington County, Block Island, 28 Sep 1975, *Allen, B.A. 46* (CONN); Newport County, Newport, 31 Aug 1908, *Bailey, W.W. s.n.* (BRU); Providence County, Providence, 1871, *Bailey, W.W. s.n.* (BRU); Washington County, New Shoreham, Block Island, 24 Aug 1898, *Bailey, W.W. s.n.* (BRU); Washington County, Westerly, between Babcock's Pond and the ocean, Aug, *Bissell, C.H. et al. s.n.* (NEBC); Washington County, South Kingstown, Kingston, 31 Aug 1891, *Burlingame, G.W. s.n.* (KIRI); Providence County, Providence, Aug, *Collins, J.F. s.n.* (NEBC); Washington County, Block Island, Sep, *Collins, J.F. & Spaulding, P. s.n.* (NEBC); Providence County, Old Cone Basin, Providence [georef to Providence centroid], 10 Jul 1892, *Collins, J.F. s.n.* (US); Kent County, Greene Island, Warwick, 13 Aug 1921, *Collins, J.F. s.n.* (CONN); Newport County, Little Compton, Sakonnet, Sakonnet, Little Compton, 7 Oct 1928, *Collins, J.F. s.n.* (CONN); Providence County, Lincoln Woods State Park, Lincoln, 2 Oct 1920, *Collins, J.F. s.n.* (CONN); Providence County, Cranston, Silver Hook, 14 Sep 1932, *Collins, J.F. s.n.* (CONN); Providence County, Providence, 2 Oct 1916, *Collins, J.F. s.n.* (CONN); Washington County, Block Island, near Old Harbour, Sep, *Fernald, M.L. et al. 10338* (GH); Washington County, Block Island, Aug, *Gravatt, G.F. s.n.* (NEBC); Washington County, South Kingstown, Campus of the Univ. of R.I, 31 Aug 1976, *Halvorson, W.L. 76-24* (KIRI); Providence County, Cranston, 129 Wentworth Avenue, Edgewood, Jun, *Hope, T. s.n.* (NEBC); Providence County, Providence, Roger Williams Park, Jul, *Hope, T. s.n.* (NEBC); Bristol County, Bristol Ferry, Sep, *Leland, G.H. s.n.* (NEBC); Washington County, Point Judith, 5 Sep 1937, *Lombard, R.H. s.n.* (GH); Providence County, Providence, Aug 1887, *Thornton, E.C. s.n.* (BRU); Washington County, Westerly, Avondale, Aug, *Ware, R.A. & Fernald, M.L. s.n.* (NEBC); Washington County, Westerly, west of Watch Hill Pond, Aug, *Weatherby, C.A. & Collins, J.F. s.n.* (NEBC); Providence County, East Providence, woodland bank, 27 Aug 1880, *Without Collector s.n.* (BRU); Washington County, New Shoreham, Block Island, corn field, Aug 1870, *Without Collector s.n.* (BRU). **South Carolina**: Richland County, Clemson Sandhill Experimental Station, 3 Oct 1980, *Brant, A. 196* (GH); Aiken County, Aiken, 14 Jul 2007, *Damrel, D.Z. 4118* (CLEMS); Anderson County, Anderson, 5 Jul 1919, *Davis, J. s.n.* (MO); Pickens County, 136 Cedar Creek Circle, Central, 21 Aug 1988, *Hill, S.R. 19867* (GH); Pickens County, 156 Cedar Creek Circle, Central, 18 Aug 1991, *Hill, S.R. 22538* (GH, NY); Richland County, Columbia, S side of Coker Life Sciences Bldg on Devine St, 3 Jun 1996, *Nelson, J.B. 17318* (NY); Sumter County, Poinsett Weapons Range, about midway bewneen SC 261 and main HQ on S side of entrance road to base, 7 Aug 1996, *Nelson, J.B. & Ryan, D. 17646* (NY); McCormick County, Sumter National Forest, mid-channel of Long Cane Creek on W edge of bottomland hardwood with the place name "Big Bottom" (on topo map), immediately W of "Laurel Hill" bluff system, approx, 1 mile NE of Long Cane Church on Sec Hwy 36, 21 Jul 2000, *Nelson, J.B. et al. 21366* (NY); Georgetown County, Brookgreen Gardens, just NE of education center dearn deer pen enclosure, betwee Litchfield and Murrells Inlet (UTM approx. 17 677829E, 37 11037N), 29 Sep 2000, *Nelson, J.B. 21602* (NY); Richland County, 3801 Duncan Street, Columbia, 20 Jul 2008, *Nelson, J.B. 27255* (F, GH, H, US); Lexington County, Congaree River, West Columbia, 27 May 1957, *Radford, A.E. 23416* (H); Lee County, Bishopsville, 6 Jun 1957, *Radford, A.E. 24169* (UBC); Pickens County, sin. loc, 7 Jul 1897, *Without Collector 1326* (NY). **South Dakota**: Brule County, Thicket near Chamberlain, 2 Jul 1914, *Over, W.H. 2834* (US); Clay County, Edge of Vermillion River, 7 Sep 1914, *Over, W.H. 5153* (US). **Tennessee**: Decatur County, sin. loc, 29 May 1855, *Ames, G.L. s.n.* (MICH); Perry County, flood

plain of Buffalo River, 0.9 air miles ENE of Sugar Hill Bridge, 15 Sep 1967, *Browne, E.M. et al. T-40* (MEXU); Blount County, along Hwy from Maryville toward Greenback on Little Baker Creek, 8 Oct 1961, *Drumke, J. et al. 29642* (GH); Obion County, Reelfoot Lake, stump in Upper Blue Basin, 30 Jun 1942, *Eyles, D.E. & Eyles, M.S. 8408* (GH); Lauderdale County, S of Hwy 87A about a mile west of its split with Hwy 87, at the large bridge over an unamaed creek from the Hatchie River, 5 Jul 1972, *Keiran, P. 438* (MEXU); Lewis County, Natchez Trace Parkway: Meriwether Lewis National Monument [Hohenwald, TN], 28 Aug 1946, *King, C.B. 401* (US); Lewis County, road shoulder of Tenn 99, also Natchez Trace Parkway ca. 1.5mi NE Park Headquarters, 24 Sep 1971, *Kral, R. 44479* (MO); Maury County, Big Bigby Creek, S of Mount Pleasant by US43, 14 Sep 1972, *Kral, R. 48421* (MO); Wayne County, Perennial grass community. Natchez Trace Parkway, 22 Sep 1947, *McDougall, W.B. 1502* (US); Marshall County, junction of Rts. 272 and 31A, 8 Jun 1998, *McNeilus, V.E. 98-322* (GH); Macon County, along Long Creek on Long Creek Road, about 8 miles N of Lafayette, 12 Sep 1999, *McNeilus, V.E. 99-889* (MEXU, NY); Davidson County, Little Marrowbone Creek Region, On fertile Creek bank, 24 Jun 1939, *Moffitt, G. 8* (US); Grundy County, Four miles west of Altamont. State highway No. 108, 3 Sep 1939, *Moffitt, G. 9* (US); Lake County, at Long Point, Reelfoot Lake, Jun 1932, *Moore, C.E. B 28* (MO); Franklin County, TUS Domain, Charlotte Gailor's garden on North Carolina Avenue, 15 Aug 2002, *Priestley, M.P. s.n.* (UOS); Knox County, Knoxville, 10 Aug 1909, *Ruth, A. 149* (GH); Knox County, Knoxville, waste places, 20 Jul 1898, *Ruth, A. 687* (E); Knox County, Knoxville, Sep 1897, *Ruth, A. 3418* (NY); Davidson County, Nashville, 24 Aug 1922, *Svenson, H.K. 256* (GH); Obion County, Reelfoot Lake Wildlife Refuge, Walnut Log division, Blue Basin, 18 Sep 1982, *Utech, F. et al. 82-479* (GH). **Texas**: *Ball, J. s.n.* (E); Lee County, Cole Springs Ranch, private land, alogn CR 419, approx 500 ft E of junction with CR 320, 2 May 2004, *Bergman, C. 711* (NY); Lee County, Patschke property (private) -Patschke Bog and adjacent land; bog created by Owens Branch of Middle Yegua Creek, private residence off CR 322, 0.7 miles W of junction with FM 1624, 10 Oct 2004, *Bergman, C. 1037* (NY); Lee County, along Road C, just N of CR 309, 1.4 miles S of junction with FM 696, 11 May 2005, *Bergman, C. 1180* (NY); Cooke County, Just E and downhill from main barn on LO Unit of Dixon Ranch, ca. 0.7 airmiles S of Co. Rd. 398 bridge over Clear Creek, ca. 1.4 airmiles NNW of jct. Cooke, Denton and Wise counties. Elev. 800-820ft, 30 Sep 2009, *Carr, W.R. 28625* (TEX); Bastrop County, E bank of Alum Creek ca. 200-500 ft. downstream from (SW of) crossing of Hoppy Spring Tract road, ca. 1.4 airmiles SE to SSE of jct. St. Rt. 21 and F. M. 1441 NE of Bastrop. Bastrop State Park. Smithville NW Quadrangle. Elev. 400-410 ft, 18 Apr 2012, *Carr, W.R. 30427* (TEX); Bastrop County, ESE to SE of Hoppy Spring, ca. 1.1 airmiles S to SSE of jct. St. Rt. 21 and F. M. 1441 NE of Bastrop. Bastrop State Park. Smithville NW Quadrangle. Elev. 460 ft, 2 May 2012, *Carr, W.R. & Pullman, E. 30565* (TEX); Reeves County, Balmorhea, Toyah Creek, 9 May 1946, *Cory, V.L. 52234* (NY); Brazos County, W of Wellborn, 29 May 1971, *Fryxell, P.A. 1767* (NY); Bowie County, Texarkana, 14 Sep 1898, *Heller, A.A. & Heller, E.G. s.n.* (NY, US); Childress County, ca. 8 miles E of Memphis along hwy 256, near Jonah and Salt Creek, 29 Aug 1973, *Higgins, L.C. 8309* (NY); Gillespie County, Crabapple Creek, *Jermy, G. 199* (MO, US); "College Lank" [Texas A&M ?], 26 May 1890, *Lake, T.W. s.n.* (NY); Smith County, Moist woods by meadow below hospital, Camp Fannin, 8 mi. NE of Tyler, 15 Oct 1943, *Moore, H.E. 563* (US); McLennan County, Waco, 1904, *Pace, L. s.n.* (MO); Lubbock County, Western Texas. In garden patch. Lubbock, *Reed, E.L. 3250* (US); Navarro County, Chambers Creek, near Corsicana, Oct, *Reverchon, J. s.n.* (MO); Dallas County, Dallas, Aug 1876, *Reverchon, J. 482* (NY); Dallas County, Dallas, *Reverchon, J. 670* (MO); Hemphill County, dune area adjacent to Canadian River, ca. 9 miles E of Canadian, 25 May 1965, *Rowell, C.M. 10839* (GH); Tarrant County, near Polytechnic, 20 Jun 1914, *Ruth, A. 511* (NY); Bosque County, 1.8 miles W of Iredell, bottoms of Bosque River, 10 Jul 1953, *Shinners, L.H. 15279* (UC); Jefferson County, Beaumont City Limits. Off 11th St. in back of Red Lobster, 14 Apr 2004, *Stone, J.R. & Gust, G. 4167* (MO); Wichita County, Red River above Burkburnett, 21 Jul 1921, *Tharp, B.C. 562* (NY, TEX); Dallas County, NW of Wycliff Avenue and Stouffer Rennaisance Dallas Hotel near I-35E, Dallas, 22 Sep 1995, *Thomas, R.D. 145732* (MO, NY); Hartley County, along US route 54, 16 miles SW of Middle Water, 16 Aug 2001, *Vincent, M.A. & Lammers, T.G. 10056* (W); Ochletree County, banks of Wolf Creek, 12 miles SE of Perryton on US 83 and 5 miles E, 20 Sep 1958, *Wallis, C.S. 7929* (MICH, TEX); Brazos County, College Station, front yard 3300 Bluestem Circle, near front door, 11 Sep 2001, *Williams, S. 7* (GH). **Vermont**: Chittenden County, Burlington, Jul 1898, *[WAO], & [JEH] s.n.*

(VT); Chittenden County, N end of North Road, near jct. of Winooski River and Lake Champlain, 27 Sep 1975, *Ahles, H.E. 81628* (H); Chittenden County, Burlington, *Ayer, K.A. & Sullivan, J.R. 639* (VT); Windham County, Brattleboro, 3 Aug 1897, *Bates, J.M. s.n.* (VT); Windsor County, Weathersfield, Sep, *Bean, R.C. s.n.* (NEBC); Grand Isle County, North Hero, Knight's Is, Aug, *Brainerd, E. s.n.* (GH); Grand Isle County, North Hero, Knight's Island, 22 Aug 1883, *Brainerd, E. s.n.* (VT); Norwich, Sep 1890, *Burbank, L.W. s.n.* (VT); Rutland County, Middletown Springs, Sep 1904, *Carpenter, D.S. s.n.* (VT); Chittenden County, southwestern part of town, shores of Lake Champlain, approximately 4 miles from mouth of Winooski River, on the E side of the abandoned railroad bed, in the area to the N of the State's Colchester Point Fishing Access Area, Aug, *Charette, L.A. 3073* (NEBC, VT); Chittenden County, Colchester, shore of Lake Champlain near Barney Point, Sep, *Countryman, W.D. 1428* (NEBC); Windham County, Vernon, 21 Sep 1969, *Countryman, W.D. 2167* (CONN, VT); Chittenden County, Winooski, 4 Aug 1938, *Dole, E.J. s.n.* (VT); Rutland County, Brandon, 23 Jul 1921, *Dutton, D.L. s.n.* (MO); Rutland County, Brandon, 25 Sep 1922, *Dutton, D.L. s.n.* (VT); Rutland County, Brandon, At edge of woods, 23 Jul 1921, *Dutton, D.L. s.n.* (BRU); Windsor County, Rochester, 4 Aug 1906, *Dutton, D.L. 398* (VT); Chittenden County, Winooski, Juxtaviam, 4 Aug 1938, *EJD*, *555* (VT); Chittenden County, Milton, 27 Sep 1990, *Fisher, M. 60* (VT); Chittenden County, Burlington, Athletics Park, 26 Sep 1903, *Flynn, N.F. s.n.* (VT); Chittenden County, Burlington, Starr Farm, 20 Sep 1903, *Flynn, N.F. s.n.* (VT); Chittenden County, Milton, 28 Jul 1893, *Grout, A.J. s.n.* (VT); Windsor County, Norwich, 6 Oct 1883, *Hazen, A. s.n.* (VT); Chittenden County, Burlington, 14 Aug 1900, *Heur, C.D. s.n.* (VT); Rutland County, Pittsford, 1 Aug 1878, *Hitchcock, M. 2196* (NY); Windham County, Brattleboro, 31 Jul 1895, *Jones, L.R. s.n.* (VT); Rutland County, Wallingford, Jul, *Kennedy, G.G. s.n.* (GH); Windsor County, Windsor, Aug, *Leland, G.H. s.n.* (NEBC); Windsor County, Norwich, 4 Oct 1888, *Loveland, M.A. s.n.* (VT); Addison County, Ferrisburgh, 29 Aug 1883, *Pringle, C.G. s.n.* (VT); Addison County, Middlebury, Morgan Barn, 16 Sep 1966, *Rand, F.V. s.n.* (VT); Orange County, Bradford, bank of Connecticut River, 17 Aug 1959, *Seymour, F.C. 18192* (WIS); Windham County, Brattleboro, fork of elm tree near R.C. church, Aug, *Wheeler, L.A. 289801* (NEBC); Windham County, Townshend, West River valley and tributaries, Sep, *Wheeler, L.A. 289902* (NEBC); Windham County, Newfane, Sep, *Wheeler, L.A. 290104* (NEBC); Chittenden County, Shelburne, 226 Pierson Dr. at back of house, 30 Jul 1998, *White, H.S. 78* (VT); Chittenden County, Burlington, *Without Collector s.n.* (K); Rutland County, Wallingford, 6 Sep 1881, *Without Collector s.n.* (VT); Chittenden County, vicinity of Saint Louis and Manhatten Drive, Burlington, 9 Jul 1980, *Zika, P.F. 1668* (CR, H, NEBC). **Virginia**: Fauquier County, High Point near Broad Run Station, western slope of Bull Run Mountains Bull Run Mts, 22 Sep 1935, *Allard, H.A. 950* (GH, US); Fauquier County, Ridges between Broad Run and Hopewell Gap, western slope of Bull Run Mountains Bull Run Mts, 19 Sep 1936, *Allard, H.A. 2269* (US); Loudoun County, Conglomerate on Potomac River near Conrads Ferry, off Route 15 C. and O. Canal, 3 Aug 1952, *Allard, H.A. 20780* (US); Loudoun County, Near bridge over Goose Creek and Route 7, 26 Aug 1952, *Allard, H.A. 20980* (US); Arlington County, Fort Runyon Station, along Potomac River, 25 Oct 1893, *Bailtcher, F.L.J. 495* (MICH); Arlington County, Fort Runyon Station, along Potomac River near Washington D.C, 25 Oct 1893, *Boettekin, F.L.J. 495* (GH, UC); Bedford County, sin. loc, 30 Sep 1872, *Curtiss, A.H. s.n.* (GH); City of Falls Church, Falls Church City, 610 Oak Haven Drive, near the marker fo rhte westernmost point; original boundary of the District of Columbia, 12 Sep 1993, *Dorr, L.J. 7941* (NY, US); Northampton County, S of Kendall Grove, 13 Oct 1935, *Fernald, M.L. et al. 5439* (GH); Southampton County, Assamoosick Swamp, S of Sebrell, 20 Jul 1939, *Fernald, M.L. & Long, B. 10808* (GH, NY); Henrico County, Richmond, freight yard of Altantic Coast Line, 19 Aug 1940, *Fernald, M.L. & Long, B. 12793* (GH); James City County, Grove Landing, bluff by James River, southeast of Grove, 30 Jul 1941, *Fernald, M.L. & Long, B. 13439* (GH, NY, US); Fairfax County, Sleepy Hollow, along Tripps Run, 1mi S of Falls Church, 7 Sep 1941, *Fosberg, F.R. 18545* (MO); Princess Anne County, Cape Henry, Seashore State Park, near western tip of, 22 Sep 1945, *Fosberg, F.R. 24120* (US); Accomack County, Assateague Island, south end of island, end of road at restuarant, (SW of Chincoteague, a barrier reef on the Atlantic Ocean), 27 Aug 1965, *Iltis, H.H. & Iltis, M.G. 24094* (UC); Princess Anne County, Cape Henry, 26 Jul 1898, *Kearney, T.H. 1818* (US); Henrico County, sin. loc, Oct 1932, *Langley, R. 2519* (GH); Bedford County, Appalachian Trail, 0.75 mi N of Petites Gap, 16 Aug 1976, *Ramsey, G.W. et al. 22577* (WIS); Princess Anne County, Pungo, bordering West Neck Creek, Jun 1929, *Randolph, L.F. & Randolph, F.R. 509* (GH,

UC); Henrico County, Richmond, U of R campus, stream on Junior Trail, 26 Sep 1934, *Ryland, A. 3819* (GH); Smyth County, St. Clair's Bottom, S. fork Holton River, 30 Jul 1892, *Small, J.K. s.n.* (F). **West Virginia**: Wirt County, on bank of run about 1 mile up Little Kanahwa River from Palestine, 29 Aug 1940, *Bartholomew, E.A. 859* (UC); Pendleton County, Seneca Rocks, "Seneca Rock", 22 Jul 1931, *Core, E.L. 3219* (GH); Tucker County, Hendricks, along Blackwater River, 10 Sep 1904, *Greenman, J.M. 281* (GH); Tucker County, Parsons, 8 Sep 1904, *Moore, A.H. 1998* (GH); Jefferson County, Shenandoah River, Shannondale, 4 Sep 1899, *Palmer, W. 141* (US); Ritchie County, Berea, 21 Aug 1922, *Randolph, L.F. & Randolph, F.R. 1360* (GH); Raleigh County, Beckley, Beaver Avenue, 2 Sep 1942, *Tosh, J.P. 504* (UC). **Wisconsin**: Grant County, "Jack Oak Island". Mississippi River bottoms. [SE of Lynxville] [02N, R04W -original in error], 21 Sep 1963, *Ackerman, G. s.n.* (WIS); Columbia County, 2mi. SE of Poynette, 29 Sep 1945, *Alberts, M. s.n.* (WIS); Iron County, sin. loc, 14 Sep 1957, *Allen, J. s.n.* (WIS); Walworth County, East Troy, 3 Aug 1926, *Almon, L. s.n.* (WIS); Green County, 3mi. SW of Brodhead, 22 Oct 1934, *Anthony, R.B. s.n.* (WIS); Winnebago County, Oshkosh, Menominee park, 18 Sep 1966, *Antonie, P. 15* (WIS); Green County, Monticello, 21 Sep 1952, *Arsdel, E. van*, *s.n.* (WIS); Green County, Monticello, 21 Sep 1952, *Arsdel, E. van*, *s.n.* (WIS); Fond du Lac County, along path in big field to E of cabin on Bluebird Hill, 15 Aug 1994, *Aussem, D. 265* (WIS); Waushara County, N/A, 30 Sep 1962, *Barker, C. s.n.* (WIS); Columbia County, Near Lodi disposal plant, 11 Aug 1966, *Barnes, W. 306* (WIS); Lafayette County, Ipswich, 1956, *Bausman, D. s.n.* (WIS); Manitowoc County, 6mi. N of Two Rivers, on Hwy 147 0.25mi. E. [orig. T25N-err], 27 Sep 1964, *Belonger, B. s.n.* (WIS); Monroe County, [N of Clifton], 15 Aug 1962, *Bennett, S. s.n.* (WIS); Monroe County, Diezlman lawn [E of Tomah], 24 Sep 1961, *Bennett, S. s.n.* (WIS); Iowa County, [E of Arena], 19 Sep 1959, *Berlin, D. s.n.* (WIS); Columbia County, Arlington Research Station, 15 Oct 1978, *Bhowmik, P. 30* (WIS); Marquette County, 5mi. E of Oxford, 29 Sep 1934, *Botany 162 Class*, *s.n.* (WIS); Jefferson County, Farmers Island, 27 Sep 1941, *Botany 162 Class*, *s.n.* (WIS); Brown County, At 1488 Servais St. Green Bay, 21 Sep 1968, *Brouette, T. s.n.* (WIS); Dane County, Pine Bluff, 17 Jul 1948, *Burton, R. 144* (WIS); Brown County, Suamico Twp, 7 Sep 1952, *Byle, N. de*, *s.n.* (WIS); Grant County, 1 mi. NW of Potosi on Hwy. 133, 1973, *Carns, P. s.n.* (WIS); Green County, [S of New Glarus], 20 Sep 1959, *Chambers, E. 25* (WIS); Dodge County, Beaver Dam, 12 Sep 1895, *Chandler, H.P. 429* (UC); Dane County, Madison, 2 Oct 1890, *Cheney, L. s.n.* (WIS); Bayfield County, Lake Superior Region, near Drummond, 30 Jun 1896, *Cheney, L. 4392* (WIS); Walworth County, lakefront lot on Deerwood Rd. off Kettle Moraine Dr. on Whitewater Lake, 4 Oct 1970, *Christensen, D. 87* (WIS); Dane County, Rocky Dell Road, ca. 5mi. W of Middleton, 5 Aug 1966, *Clarke, H. 21* (WIS); Sauk County, N shore of Wisconsin River, S of Sauk City, 24 Aug 1970, *Cochrane, T.S. & Warnes, B. 3005* (WIS); Sauk County, sin. loc, 16 Jul 1972, *Cochrane, T.S. & Cochrane, B.A. 5142* (WIS); Polk County, Interstate Park [SW of St. Croix Falls], 14 Aug 1972, *Cochrane, T.S. & Cochrane, B.A. 5351* (WIS); Lafayette County, [NW of Calamine], 5 Aug 1961, *Coogan, A.H. et al. 268* (WIS); Grant County, Bagley, 4 Oct 1964, *Cook, C. s.n.* (WIS); Waukesha County, Waukesha, 30 Nov 1847, *Cornwall, G. s.n.* (WIS); Dane County, Stewart's Woods [3mi. S of Verona], 18 Jul 1946, *Cottam, G. 160* (WIS); Iowa County, [W of Arena], 4 Aug 1959, *Cross, R.C. 237* (WIS); Burnett County, S shore of Spirit Lake -6mi. due NW of Frederic, 13 Aug 1987, *Culbertson, C. 100* (WIS); Waukesha County, Between Hartland and Pewaukee, 14 Sep 1946, *Cull, I. 748* (WIS); Waukesha County, Between Hartland and Pewaukee, 14 Sep 1946, *Cull, I. 748 a* (WIS); Crawford County, Hwy 35, 0.25mi. S of Lansing bridge, 15 Sep 1972, *Dagnon, C. 18* (WIS); Columbia County, 0.25 mi. W of Columbus on Hwy 60, 25 Sep 1965, *Daniel, T.C. 28* (WIS); Richland County, Lone Rock, 29 Sep 1936, *Davis, J. s.n.* (WIS); Richland County, Lone Rock, 26 Sep 1936, *Davis, J. s.n.* (WIS); Richland County, Lone Rock, 24 Sep 1936, *Davis, J. s.n.* (WIS); Fond du Lac County, Fond du Lac, 14 Sep 1900, *Denniston, R. s.n.* (WIS); St. Croix County, 0.25mi. off river [Mississippi River] [NW of Somerset], 19 Sep 1964, *Deshpande, B. s.n.* (WIS); Marinette County, At end of Michaelis St, 3 Sep 1972, *Djupstrom, B. s.n.* (WIS); Chippewa County, Cather Lake [NE of New Auburn], 15 Aug 1960, *Ebert, T. s.n.* (WIS); Chippewa County, School House Lake [NE of New Auburn], 5 Jul 1960, *Ebert, T. s.n.* (WIS); Pepin County, Pepin, 27 Aug 1927, *Fassett, N.C. 5602* (WIS); Pierce County, Prescott, 30 Aug 1927, *Fassett, N.C. 5627* (WIS); Burnett County, Shore of Long Lake, Hertel, 21 Sep 1928, *Fassett, N.C. 8158* (WIS); Green Lake County, Marquette, 18 Sep 1929, *Fassett, N.C. 8793* (WIS); Trempealeau County, Trempealeau, 23 Aug 1927, *Fassett, N.C. 9778* (WIS); Grant County, North Andover, 6 Sep 1930, *Fassett, N.C. 13364* (WIS); Grant

County, Dickeysville, along Platte River, 2 Sep 1930, *Fassett, N.C. 13445* (WIS); Sauk County, Along US 12 & 16, W of Kilbourn [Columbia Co.] [Wisconsin Dells], 22 Sep 1932, *Fassett, N.C. & Wilson, L.R. 14672* (GH, WIS); Polk County, Saint Croix Falls, Interstate park, 2 Sep 1927, *Fassett, N.C. 15553* (WIS); Door County, Sister Bay, 25 Sep 1932, *Fassett, N.C. & Wilson, L.R. 16218* (WIS); Kewaunee County, Kewaunee, 25 Sep 1932, *Fassett, N.C. & Wilson, L.R. 16220* (GH, WIS); Dane County, Pine Bluff, 25 Oct 1937, *Fassett, N.C. 19169* (WIS); Green County, SW of Juda on CTH KS, 29 Jul 1958, *Fell, E. 58-533* (WIS); Rock County, Nelson Rd, 24 Jul 1957, *Fell, E. 57-860* (WIS); Green County, Hoosier Rd. [SE of Monroe], 19 Oct 1957, *Fell, E. 57-1454* (WIS); Green County, 4mi. NE of Albany, 11 Aug 1929, *Fernholz, D. s.n.* (WIS); Taylor County, Gravel pit. Chequamegon National Forest, 28 Sep 1993, *Fields, D. 818* (WIS); Taylor County, Chequamegon National Forest, 25 Aug 1994, *Fields, D. 1992* (WIS); Grant County, County Rd Z; just 0.3 km S of Louisburg Rd, opposite Transland Dairy 21 Jul 2018, *Funk, V. & Knapp, S. 14109* (US); Grant County, County Rd Z; 0.8 km S of Louisburg Rd; behind the Visitor Center at the Aldo Leopold Foundation Visitor Center [Levee Rd, Baraboo, WI 53913] 21 Jul 2018, *Funk, V. & Knapp, S. 14111* (US); Kenosha County, Kenosha, 30 Aug 1909, *Gates, F.C. 3252* (MICH); Dane County, [ENE of Cross Plains], 31 Aug 1961, *Glaeser, O. s.n.* (WIS); Sheboygan County, Sheboygan, 30 Jun 1918, *Goessl, C. s.n.* (WIS); Richland County, farm at end of Moss Hollow Road, 2mi. N of CTH JJ at Gotham, 22 Sep 1979, *Goytowski, A. & Newenhouse, A. 52* (WIS); Trempealeau County, [S of Dodge], 2 Oct 1965, *Graham, J. s.n.* (WIS); Dane County, Verona, Stewart's woods, 31 Jul 1949, *Greeley, F. s.n.* (UC); Bayfield County, Bluff overlooking Lake Superior. [NNE of Bayfield], 23 Sep 1966, *Greidanus, T. 609* (WIS); Columbia County, UW Hort. Farm [SE of Arlington], 10 Sep 1960, *Gressel, J. s.n.* (WIS); Green County, CTH [country highway] N, 3.4mi. S of CTH H. [SSW of New Glarus], 17 Sep 1966, *Gruber, P. s.n.* (WIS); Iowa County, Cox Hollow [N of Dodgeville], 21 Oct 1956, *Habeck, J. s.n.* (WIS); Waushara County, Poy Sippi, 30 Sep 1956, *Haggene, T. s.n.* (WIS); Lafayette County, 2.5mi. W of Gratiot, 28 Sep 1956, *Haggene, T. et al. s.n.* (WIS); Dane County, Madison, *Hale, T. s.n.* (WIS); Wood County, 5mi. E of Wisconsin Rapids on CTH W, 20 Sep 1964, *Hanneman, R.E. 22* (WIS); Barron County, 1.5mi. S of Dallas, 25 Aug 1972, *Hansen, B. & Hansen, J. 1317* (WIS); Pierce County, Across main channel of Mississippi River; 0.25mi. N of Red Wing, Minn. [S of Hager City], 14 Sep 1976, *Hansen, B. et al. 3981* (WIS); Pierce County, Across main channel of Mississippi River; 0.25mi. N of Red Wing, Minn. [S of Hager City], 14 Sep 1976, *Hansen, B. et al. 4009* (WIS); Pierce County, CTH E, 1.75mi. NW of Hager City, 14 Sep 1976, *Hansen, B. et al. 4058* (WIS); Trempealeau County, In downtown Pleasantville, 22 Sep 1976, *Hansen, B. et al. 4235* (WIS); Buffalo County, Old RR terminus along Buffalo River in south Mondovi, 23 Sep 1976, *Hansen, B. et al. 4685* (WIS); Vernon County, Viroqua, 7 Aug 1929, *Hansen, H. 222* (WIS); Manitowoc County, 1301 Cherry Rd. Manitowoc, 24 Sep 1977, *Hansen, J.J. 18* (WIS); Jackson County, Black River bottomlands, W of CTH V. [SE of North Bend], 15 Aug 1957, *Hansen, T. s.n.* (WIS); Winnebago County, Bay View Road just S of the end of Cecil Street, Neenah. 100yds inland from shore of Lake Winnebago, 27 Aug 1965, *Harker, M. s.n.* (WIS); Winnebago County, Neenah, 3 Oct 1964, *Harker, M. 609* (WIS); Jefferson County, Lake Mills, Wollein's Maple Woods, 16 Aug 1962, *Harper, K.T. 631* (UT); La Crosse County, Behind Riverside Park, City of La Crosse, 26 Jun 1956, *Hartley, T. 855* (WIS); La Crosse County, Yard weed in LaCrosse, 7 Jul 1956, *Hartley, T.B. 1234* (US); sin. loc, *Hasse, H.E. s.n.* (NY); Racine County, Racine, 21 Aug 1906, *Heddle, J. 273* (WIS); Dunn County, Menomonie, 23 Jul 1928, *Hill*, *& Rossler s.n.* (WIS); Grant County, Lancaster, 932 Western Ave, 1998, *Hollingsworth, T. s.n.* (WIS); Jefferson County, [S of Sullivan], 30 Sep 1961, *Hooper, J. 37* (WIS); Iowa County, Upper N slope of Blue Mound [ENE of Barneveld], 27 Jul 1961, *Hoover-Green*, *129* (WIS); Oneida County, 2mi. N of CTH K on Horsehead Rd, 3 Aug 1992, *Hughes, C. 179* (WIS); Marquette County, Observatory Hill, 5mi. S of Montello, 21 Jul 1955, *Iltis, H.H. et al. 5617* (WIS); Marquette County, N shore of Ennis Lake [S of Glen Oak], 14 Jul 1956, *Iltis, H.H. et al. 6219* (WIS); Sauk County, Owl's Head, an isolated sandstone peak (alt. 1060 ft.) 1mi N of Wisconsin River and 2mi E of Merrimac, 11 Aug 1956, *Iltis, H.H. & Botany 130 Class 6845* (WIS); Pierce County, Overlooking Rush River and US #10. [N of Maiden Rock], 24 Aug 1956, *Iltis, H.H. & Noamesi, G.K. 8008* (WIS); Columbia County, 1mi. SW of Columbus, N of US 151, 10 Sep 1956, *Iltis, H.H. & Koeppen, R. 8183* (WIS); Sheboygan County, Along Lake Michigan. 2mi. E of Cedar Grove, 10 Sep 1956, *Iltis, H.H. & Koeppen, R. 8205* (WIS); Ozaukee County, Strand of Lake Michigan. Shore of Lake Michigan, 4mi. NNE of Port Washington, 10 Sep 1956, *Iltis, H.H. & Koeppen, R. 8294* (WIS); Rock County, SE

shores of Lake Koshkonong [W of Koshkonong, Jefferson Co.], 7 Oct 1956, *Iltis, H.H. & Janecek, J.J. 8465* (WIS); Buffalo County, RR track at Alma, 26 Jun 1957, *Iltis, H.H. & Neess, J. 9330* (WIS); Richland County, 2mi N of Ithaca on Wisc. #58. Valley of Willow Creek, 28 Aug 1957, *Iltis, H.H. & Salamun, P.J. 9913* (WIS); Grant County, Tufa Falls, 3mi. SSW of Platteville, 17 Sep 1957, *Iltis, H.H. et al. 10443* (WIS); Grant County, Horse Thief Hollow, 0.5mi. E of McCartney, along Mississippi River, 25 Jul 1959, *Iltis, H.H. et al. 13797* (WIS); Iowa County, On CTH K, 3mi. NE of Barneveld, 2 Aug 1959, *Iltis, H.H. & Rudolph, E. 13951* (WIS); Columbia County, Between RR and CTH P, just E of Pardeeville, 7 Aug 1959, *Iltis, H.H. 14131* (WIS); Adams County, Witches' Gulch [N of Wisconsin Dells] Above Wisconsin River, 9 Oct 1960, *Iltis, H.H. 17030* (WIS); Marathon County, Dells of the Eau Claire River County Park. 3mi SW of Hogarty, 5 Sep 1962, *Iltis, H.H. 20815* (WIS); Marathon County, Dells of the Eau Claire River County Park. 3mi SW of Hogarty, 5 Sep 1962, *Iltis, H.H. 20855* (WIS); Dane County, Madison, University of Wisconsin campus, just S of Warf Building parking lot, 30 Sep 1989, *Iltis, H.H. 30225* (NY, WIS); Sauk County, Pine Hollow in Baraboo Hills, 8 Oct 1966, *Irwin, H. s.n.* (WIS); Kewaunee County, 3mi. S of Algoma. Lake Michigan shore, 6 Sep 1956, *Iwen, M.M. 181* (WIS); Winnebago County, Oshkosh, 9 Jul 1894, *James, M. s.n.* (WIS); Polk County, Near Diamond Lake. [N of Frederic], 25 Aug 1960, *Johnson, M. s.n.* (WIS); Oconto County, Nauke Rd. [NE of Breed], 9 Aug 1981, *Judziewicz, E. 2449* (WIS); Bayfield County, Raspberry Island, at light; Apostle Islands, Apostle Islands National Lakeshore, 24 Aug 1992, *Judziewicz, E. 9928* (WIS); Oconto County, At ""Butternut Pines"", 16 Aug 1997, *Judziewicz, E. 12458* (WIS); Door County, NW coast and ""prong"" of island. Shore of Lake Michigan, 16 Sep 1998, *Judziewicz, E. 13890* (WIS); Marinette County, Green Island, 80 acre island in Green Bay, 9 Aug 1999, *Judziewicz, E. et al. 14101* (WIS); Crawford County, 0.125mi. W of Town Road, 0.125mi. N of CTH B. [N of Towerville], 23 Sep 1962, *Kilen, T. s.n.* (WIS); Lafayette County, Middie Mine, abandoned in 1928. Ca. 1mi. SW of Benton, *Kimmerer, R. s.n.* (WIS); Lafayette County, Thomson Mine, abandoned in 1945. 1 mi. N of White Oak, ca. 5 mi. SW of Shullsburg, 31 May 1981, *Kimmerer, R. s.n.* (WIS); Vernon County, [W of Valley], 13 Jul 1973, *Kline, V. s.n.* (WIS); Dane County, West Hill Farm, 12 Oct 1958, *Klisiewicz, J. s.n.* (WIS); Dodge County, CTH G, 1mi. S of Beaver Dam, 9 Oct 1958, *Klisiewicz, J. s.n.* (WIS); Dane County, 1.5mi. S of Middleton on Hwy 12-13, 13 Sep 1955, *Koeppen, R. 97* (WIS); Jefferson County, Hope Lake, 4mi. SW of Lake Mills, 24mi. E of Madison, 13 Oct 1954, *Kuhlman, F. s.n.* (WIS); Eau Claire County, Fall Creek, 24 Aug 1928, *Kunz, H. 36* (WIS); Eau Claire County, Fall Creek, 6 Sep 1928, *Kunz, H. 241* (WIS); Ashland County, Ironwood Island, [SE side of Island], 6 Jul 1955, *Lane, F. 2379* (WIS); Milwaukee County, Milwaukee, *Lapham, I. s.n.* (WIS); Adams County, [E of Big Flats], 18 Sep 1965, *LeClair, R. s.n.* (WIS); Waukesha County, Genesee (Genesee Oak Opening State Natural Area), 17 Jul 1989, *Leitner, L. 2867* (WIS); Shawano County, [SSE of Bonduel], 6 Sep 1964, *Liesner, R. 107* (WIS); Columbia County, 1mi. N of Okee. Adjacent to Wisconsin River, 4 Oct 1965, *Lynch, G. s.n.* (WIS); Door County, Portage County Park. Along Lake Michigan shore, 1 Sep 1999, *Mahlberg, P. 618* (WIS); Portage County, 400 block Center Street, Stevens Point, 24 Sep 1960, *Malick, L. s.n.* (WIS); Polk County, [SW of Osceola], 1 Sep 1964, *Marcks, B. 32* (WIS); Monroe County, 1.5mi. N of Norwalk along sideroad 0.25mi. N of CTH T, 16 Sep 1972, *Marcks, B. & Marcks, C. 1382* (WIS); Sauk County, [SSE of Hill Point], 18 Sep 1976, *Matlosz, D. 13* (WIS); Shawano County, 1.5mi. N, 0.5mi. E of 5 Corners. [N of Bowler], 4 Aug 1964, *Mauritz, J. 988* (WIS); Rock County, 10mi. W of Janesville, 19 Sep 1965, *McCarten, M. s.n.* (WIS); Green Lake County, [NE of Green Lake], 29 Sep 1962, *McConnell, C. s.n.* (WIS); Columbia County, Harmony Grove, 18 Sep 1965, *McCown, B. 35* (WIS); Shawano County, [NE of Cecil], 13 Sep 1958, *Melchert, T. s.n.* (WIS); Waukesha County, Delafield Fish Hatchery grounds, 24 Jul 1956, *Melville, R. 130* (WIS); Sauk County, 2mi. NW of Black Hawk. On Ranch Road, 26 Sep 1964, *Meslow, E. s.n.* (WIS); Dunn County, S of Elk Mound, 3 Aug 1953, *Meyer, D. 203* (WIS); Columbia County, Columbia Energy Center property, 3 mi S of Portage, 0.5 mi W of Hwy 51; in bed of Duck Creek, 22 Sep 1975, *Middleton, B. 5* (WIS); Dane County, Near Fish Lake. [N of Roxbury], 20 Aug 1967, *Miller, R.B. 385* (WIS); Dane County, [E of Madison], 3 Oct 1959, *Monroe, R. s.n.* (WIS); Washington County, 1.5mi. SE of West Bend, 8 Oct 1966, *Muth, C. 19* (WIS); Richland County, [SW of Ithaca], 3 Nov 1970, *Nee, M. 3462* (UC, WIS); Sawyer County, CTH B at bridge over West Fork Chipeewa River. [N of New Post], 31 Aug 1971, *Nee, M. 4705* (WIS); Sawyer County, Chicago Bay, Chippewa Flowage. [NW of New Post], 1 Sep 1971, *Nee, M. 4710* (WIS); Richland County, 3 miles SE of Richland Center, T10N, R1E, NE1/4,

NE1/4 Sec 35, 10 Sep 1977, *Nee, M. 16020* (CORD, MEXU, MO, NY, WIS); Jefferson County, Fort Atkinson, S of the Rock River, 9 Sep 1979, *Nee, M. 18011* (WIS); Jefferson County, At wayside along Hwy 26, 6mi. SW of Fort Atkinson, 15 Aug 1981, *Nee, M. 21772* (WIS); Jefferson County, 0.5 mile S of Fort Atkinson, 5 Sep 1981, *Nee, M. 21874* (F, WIS); Jefferson County, along C and NW railroad tracks and Hwy. 26-89, 1.5miles NE of Fort Atkinson, 6 Sep 1981, *Nee, M. 21933* (F, WIS); Richland County, 1.5 miles N of Lone Rock, 4 Sep 1982, *Nee, M. 25602* (CORD, NY, WIS); Grant County, Bertom Lake, landing along the Mississippi River, 24 Sep 2000, *Nee, M. & Atha, D.E. 51036* (CORD, NY, WIS); Richland County, Rudy Nigl property, along Marshall Road, 2.2 miles (by air) WNW of Gillingham, 21 Jul 2007, *Nee, M. 55498* (NY); Crawford County, 5.5 km (by air) NNE of Wauzeka, Husher Wayside Park, along Hwy 131, 2 Sep 2013, *Nee, M. 60433* (NY); Grant County, Fennimore, eastern outskirts, N of Hwy Q, near top of Military Ridge, 27 Aug 2014, *Nee, M. 61510* (MO, NY); Wood County, Babcock, 30 Jul 1937, *Oehmcke, A. 95* (WIS); Door County, Along access road near Group Camping Area. [W of Ephraim], 22 Oct 1968, *Olsen, E. 16* (WIS); Lafayette County, sin. loc, 28 Sep 1969, *Palzkill, D. 3* (WIS); Bayfield County, sin. loc, 25 Sep 1964, *Partosoedarso, M. s.n.* (WIS); Pierce County, [SW of River Falls], 28 Jun 1959, *Patman, J. et al. s.n.* (WIS); Dane County, 1mi. E on Hwy 19 from jct. with Hwy 13 and 0.75mi. N, 20 Sep 1959, *Paulson, W. s.n.* (WIS); Dane County, Rt. K [SE of Marxville], 23 Aug 1958, *Peters, R. 154* (WIS); La Crosse County, Third Ward, Onalaska, 30 Jun 1957, *Peterson, A. 256* (WIS); Waupaca County, Along Wolf River, 5mi. SW of New London, 19 Oct 1975, *Petit, A. 34* (WIS); Outagamie County, [N of New London], 29 Jul 1978, *Petit, A. 235* (WIS); Dane County, sin. loc, 2 Oct 1958, *Pienkowski, R. s.n.* (WIS); Door County, along edge of country road X [N of Sturgeon Bay], 26 Oct 1958, *Pirone, T. s.n.* (WIS); Dodge County, Hubbard Twp. 1.5 mi. W of Hustisford on Hwy 60, N on St. Helena Rd. E on Arrowhead Tr to the end (lot No. 10), 1971, *Pisek, S. s.n.* (WIS); Waushara County, Triple Ridge Farm [SE of Coloma] Route 1 [georef to Coloma], 27 Sep 1958, *Pochmann, R. s.n.* (WIS); Waushara County, Triple Ridge Farm [SE of Coloma] Route 1 [georef to Coloma], 28 Sep 1957, *Pochmann, R. 26* (WIS); Waushara County, Triple Ridge Farm [SE of Coloma] Route 1 [georef to Coloma], 23 Aug 1957, *Pochmann, R. 45* (WIS); Polk County, Interstate State Park [NW of Dresser], 2 Sep 1936, *Pohl, R. 604* (WIS); Crawford County, 1 mi. NW of Wauzeka on CTH N and N 6 mi. on Plum Creek Rd, 1977, *Pomerening, B. s.n.* (WIS); Jefferson County, Corner of CTH XX and IWPP Road, 2mi. N of Route 18, 8mi. S of Watertown, 21 Sep 1969, *Pope, R. 38* (WIS); Washington County, [SE of Kewaskum], 20 Sep 1966, *Rabideau, J. s.n.* (WIS); St. Croix County, Just N of Glennwood City, CTH X runs N & S through center. [orig. R14W-err], 28 Sep 1964, *Radcliffe, L. 30* (WIS); St. Croix County, W shore of Pine Lake from park area N to end of lake. [N of Baldwin], 4 Oct 1964, *Radcliffe, L. 203* (WIS); Dane County, 2.5mi. E of Blue Mounds, Hwy 151, 8 Oct 1959, *Reinert, R. s.n.* (WIS); Marinette County, At edge of Green Bay, 93 W. Bay Shore St. Marinette, 2 Sep 1974, *Reinke, M. s.n.* (WIS); Dodge County, Reeseville, 1 Aug 1931, *Rhodes, J. 272* (WIS); Rock County, CMSPP RR right-of-way [SW of Clinton], 6 Jul 1969, *Rice, W. 646* (WIS); Rock County, CNW RR right-of-way, SE of Clinton. From W boundary 0.25mi. E to stream, 27 Jul 1969, *Rice, W. 867* (WIS); Green County, Right-of-way of CTH KS and CMSPP RR. [SW of Juda], 23 Jul 1972, *Rice, W. & Rice, M. 1614* (WIS); Rock County, SW edge of Murphy Woods at Murphy Woods Road, 17 Aug 1972, *Rice, W. 1748* (WIS); Green County, CMStPP RR right-of-way, 15 Jul 1973, *Rice, W. & Rice, M. 1948* (WIS); Wood County, In Griffith State Nursery [E of Port Edwards], 6 Aug 1960, *Riffle, N. s.n.* (WIS); Iowa County, [W of Arena], 20 Sep 1959, *Riffle, N. s.n.* (WIS); Waupaca County, Clintonville, 16 Aug 1959, *Rill, K. s.n.* (WIS); Chippewa County, [N of Holcombe], 31 Aug 1965, *Robarge, M. s.n.* (WIS); Columbia County, Poynette, 20 Jun 1886, *Russell, H. s.n.* (WIS); Kenosha County, 2mi. S of Kenosha in Pleasant Prairie Twp, 27 Sep 1964, *Ryzicki, G. s.n.* (WIS); Door County, Shoreline of Lake Michigan; Schauer's Park, near Cave Point, 11mi. NE of Sturgeon Bay, 26 Sep 1976, *Sanford, J. 16* (WIS); Lincoln County, S of CTH A. [N of Tomahawk], 18 Sep 1956, *Schlising, R.A. 586* (WIS); Oneida County, Along Pier Lake [SW of Hazelhurst], 9 Sep 1957, *Schlising, R.A. et al. 747* (WIS); Brown County, sin. loc, 27 Jul 1900, *Schuette, J. s.n.* (WIS); Brown County, sin. loc, 27 Jul 1900, *Schuette, J. s.n.* (WIS); Milwaukee County, N. 59 St, 8 Mar 1966, *Scott, C. 542* (H); Outagamie County, Appleton, 28 Jul 1949, *Seymour, F. 10816* (WIS); Lincoln County, Route FF, 14 Aug 1950, *Seymour, F. 12074* (WIS); Lincoln County, Route E, 19 Jul 1954, *Seymour, F. 15890* (WIS); Lincoln County, Pine River Twp, 20 Jul 1954, *Seymour, F. 15946* (WIS); La Crosse County, Mississippi River, navigation pool #8, Site 5, plateau 2 m above normal water level;

Mississippi River mile 691-702.5, 29 Jul 1975, *Shannon, S.D. 1816* (NY); Jackson County, City Point, 24 Sep 1967, *Sharp, B. s.n.* (WIS); Waukesha County, [N of Monterey], 7 Sep 1957, *Sheaffer, W. s.n.* (WIS); Green Lake County, Green Lake [Marquette Co. -original in error], 31 Jul 1938, *Shinners, L. s.n.* (WIS); Trempealeau County, 1mi. S of Independence on Hwy 93 then 0.5mi. W on CTH X, 28 Jul 1990, *Skroch, P. 50* (WIS); Wood County, Central Cranberry Co. [NW of Nekoosa], 23 Aug 1961, *Skroch, W. Cen-238* (WIS); Walworth County, Geneva Twp, 12 Sep 1884, *Smith, H.H. s.n.* (WIS); Sauk County, Baraboo Bluffs, 9 Aug 1922, *Smith, H.H. 8189* (WIS); Kenosha County, Benedict Prairie, 28 Aug 1988, *Smith, J.F. & Kuchenreuther, M.A. 1034* (WIS); Green Lake County, 1mi. SE of Princeton, 21 Sep 1968, *Soberalske, R. s.n.* (WIS); La Crosse County, E oertel farm, end of Smith Valley Road about 3 miles from County Road B, 10 Sep 1968, *Sohmer, S.H. 5055* (UT); Adams County, sin. loc, 13 Jul 1962, *Sorensen, P. 2366* (WIS); Marquette County, sin. loc, 28 Jul 1962, *Sorensen, P. 3228* (WIS); Dane County, Madison, 20 Jul 1926, *Sprague, R. 1204* (WIS); Washington County, Formerly city of Hartford dump, 9 Jul 1993, *Spuhler, D. 93-161* (WIS); Adams County, [N of Monroe Ctr.], 19 Aug 1973, *Stahmann, C. 452* (WIS); Adams County, Petenwell Lake, 25 Aug 1973, *Stahmann, C. 580* (WIS); Door County, Hwy 57, N of Logerquist Road, 19 Sep 2001, *Standish, M. 437* (WIS); Shawano County, Navarino Wildlife Area grouse survey, 21 Aug 1980, *Stefano, S. de*, *186* (WIS); Columbia County, Goose Pond (boundary Sec. 25 of Arlington & Sec. 19 Leeds) [SE of Arlington], 16 Sep 1961, *Stewart, M. s.n.* (WIS); Grant County, Near Dugway Rd. and CTH A. [SW of Bloomington], 25 Sep 1976, *Stueber, S. 31* (WIS); Columbia County, N/A, 22 Sep 1963, *Swann, C. s.n.* (WIS); La Crosse County, Mississippi River, navigation pool #8, site 5, mile 691-702.5, plateau 2 m above normal water level, 29 Jul 1975, *Swanson, S.D. 1816* (MO); La Crosse County, Site 20, railroad levee built on an island alogn a side channel with rip-rap laong portions of its bank; Mississippi River miles 691-702.5, navigation pool #8, 31 Jul 1975, *Swanson, S.D. 1954* (NY); La Crosse County, Site 40, old spit on the edge of a young hardwood forest, Mississippi River mile 691-702.5, navigation pool #8, 25 Sep 1975, *Swanson, S.D. 2421* (NY); Grant County, 1 mi. N of Glen Haven, 27 Sep 1964, *Tamulevich, S. 29* (WIS); Portage County, Alban Twp, 4 Jul 1962, *Tessene, M. 210* (WIS); Buffalo County, 4.5mi WNW of Fountain City, on Mississippi River, 26 Jul 1991, *Thompson, K.L. 604* (WIS); Dane County, At Cave of the Mounds, behind rest area [between Mt. Horeb and Blue Mounds], 1 Oct 1966, *Torin, M. s.n.* (WIS); Richland County, 3mi. W of Gotham, Hwy 60, 25 Jul 1963, *Torrealba, P. s.n.* (WIS); Sauk County, Baraboo, 6 Jul 1891, *True, R. s.n.* (WIS); Dane County, On UW campus, 20 Sep 1959, *Tseng, P. s.n.* (WIS); Sawyer County, N end Connors Lake, Flambeau State Forest. [S of Oxbo], 1 Sep 1959, *Ugent, D. et al. s.n.* (WIS); Door County, E side of Lake Mackaysee, 27 Aug 1961, *Ugent, D. 1111 [a]* (WIS); Walworth County, Williams Bay, Conference Point, 2 Sep 1909, *Umbach, L.M. 4843* (WIS); Sauk County, [N of Spring Green], 25 Sep 1964, *Utech, F. s.n.* (WIS); Manitowoc County, Wallen's, 122 So. 15th, Manitowoc, 2 Oct 1964, *Wallen, R. 46* (WIS); Dane County, Hwy 78 [E of Mazomanie], 28 Sep 1958, *Weber, P. s.n.* (WIS); Pierce County, River Falls, 4 Sep 1892, *Weinzirl, J. s.n.* (WIS); Columbia County, 1mi. W of Hwy 51, 22, and 60 intersection. [SE of Arlington], 22 Sep 1964, *Weis, G. 5* (WIS); Taylor County, Aurora Twp, 11 Jul 1949, *White, K.L. 107* (WIS); Rock County, E of Janesville, 31 Jul 1947, *Wickham, J. 13* (WIS); Monroe County, Training Area C3; Military Coordinates X, Y: 690150, 4879850, 27 Aug 2000, *Williams, M. 892* (WIS); Richland County, Loyd, 25 Sep 1957, *Wills, D. s.n.* (WIS); Green Lake County, Camp Tichora [S of Green Lake], 19 Aug 1957, *Wills, D. s.n.* (WIS); Rock County, Off Rt. 138 bordering Dane & Rock Counties, 22 Sep 1964, *Wilson, M. s.n.* (WIS); Fond du Lac County, [SW of Fond du Lac], 21 Sep 1960, *Wollitz, A. s.n.* (WIS); La Crosse County, Island 21; Mississippi Rivermile #695, E side of main channel, 1975, *Ziegler, S.R. & Leykom, M.F. s.n.* (WIS); Vernon County, Island 3; Mississippi Rivermile #685.1. Navigation Pool 8. Small island W side main channel, 8 Jul 1975, *Ziegler, S.R. & Leykom, M.F. 1657* (NY, WIS); La Crosse County, Island 18, Mississippi River mile 692.2, Island on E side of main channel, 24 Jul 1975, *Ziegler, S.R. & Leykom, M.F. 1982* (H); La Crosse County, Island 20, Mississippi River mile 695, small island E sdie of main channel, central area of island [T15N, R7W, sec. 17], 25 Jul 1975, *Ziegler, S.R. & Leykom, M.F. 2038* (MEXU); Vernon County, Island 8 Mississippi River mile #688.4, T14N R7W Sec 18; large island E side of main channel, 3 Sep 1975, *Ziegler, S.R. & Leykom, M.F. 2430* (NY); Vernon County, Island 3; Mississippi Rivermile #685.1. Navigation Pool 8. Small island W side main channel, 12 Sep 1975, *Ziegler, S.R. & Leykom, M.F. 2609* (WIS). **Wyoming**: Goshen County, 3 miles NE of Torrington, (T25N, R60W, Sec 31 S 1/2, S 1/4), 15 Aug 1993, *Dorn, R. 5469* (NY).

6. *Solanum furcatum* Dunal

UNITED STATES OF AMERICA. **California**: San Mateo County, along upper slopes of the edgemar Road (northwestern San Mateo County), 3 Aug 1954, *Bacigalupi, R. et al. 4734* (JEPS); San Mateo County, San Pedro Point, 8 Mar 1908, *Brandegee, K. s.n.* (UC); Marin County, Spender Battery, near Golden Gate Bridge, 17 Apr 2011, *Christenhusz, M.J.M. 6230* (H); Contra Costa County, Brooks island loading dock, North side of island, 20 Mar 2012, *Doran, A.S. 672* (UC); San Mateo County, San Bruno Mountains, near Radio Towers on top San Bruno Mountains, 28 Mar 1966, *Doyen, J.T. 34* (UC); Santa Barbara County, near Santa Maria, Jun 1906, *Eastwood, A. 375* (GH); Monterey County, Beach near Point Pinos, 14 Apr 1903, *Heller, A.A. 6572* (E, F, GH, IND, LE, MO, NY, POM, UC); San Francisco County, n slope Russian Hill along Hyde Street, San Francisco Russian Hill; San Francisco, Russian Hill along Hyde Street, 20 Jul 1969, *Howell, J.T. 45872* (JEPS); San Mateo County, hills w of colma (vicinity of San Francisco Bay), 15 Oct 1923, *Jepson, W.L. 10256* (JEPS); Humboldt County, Trinidad, near boat landing Trinidad, 13 Jul 1947, *Paddock, E.F. 163* (UC); Mendocino County, Wesport, 14 Jul 1947, *Paddock, E.F. 167* (UC); Mendocino County, Westport, 14 Jul 1947, *Paddock, E.F. 168* (UC); San Francisco County, East side of Lake Merced, just S of pedestrian crossway, 11 Nov 1967, *Porter, D.M. & Porter, S.H. 1577* (MO); San Mateo County, On the outstreets of Colma, 16 Apr 1950, *Raven, P.H. 1773* (MO, UC); San Francisco County, Turk Street at Stanyan Street Turk Street, 19 Sep 1954, *Raven, P.H. 8175* (JEPS); San Francisco County, Vicinity of Fort Point, Presidio, just below Battery, 21 Jul 1990, *Rubtzoff, P. & Smith, S. 10202* (MO); San Mateo County, Mussell Rock, 24 Oct 1938, *Stebbins, G.L. & Duffield, J.W. 19* (UC); San Mateo County, Tobin, 28 Jul 1940, *Stebbins, G.L. & Paddock, E.F. 89* (GH); San Mateo County, at junction of three upper forks of San Pedro Creek (1 mi from seashore, San Pedro Valley), San Pedro Valley, 7 Sep 1940, *Stebbins, G.L. & Paddock, E.F. 101* (UC); San Mateo County, Calera Valley, 7 Sep 1940, *Stebbins, G.L. & Paddock, E.F. 103* (UBC); Humboldt County, Trinidad, 26 May 1941, *Stebbins, G.L. 130* (UC); Humboldt County, Trinidad, near boat landing Trinidad, 5 Oct 1940, *Tracy, J.P. 16749* (JEPS, UC). **Oregon**: Curry County, The Heads, Port Oxford, 20 Jun 1919, *Peck, M.E. 9059* (GH, MO); Linton near Portland, 2 Sep 1911, *Suksdorf, W.N. 1720* (GH).

7. *Solanum interius* Rydb.

UNITED STATES OF AMERICA. **Colorado**: Fremont County, near Cañon City, 1871, *Brandegee, T.S. 320* (NY); Fremont County, Canon City, Rocky Mts, 1871, *Brandegee, T.S. 5873* (MO); Larimer County, Fort Collins, 18 Oct 1897, *Cowan, J.H. 4197* (NY); Yuma County, 10km E of Yuma, 1km NE of Hwy 34, nr RR tracks, just W of jct of Rd R & Rd 375, 14 Aug 1996, *Hazlett, D.L. & Hazlett, E. 9523* (MO, NY); Weld County, Fort Lupton, 12 Sep 1900, *Johnston, E.L. 672* (US); Weld County, Fort Lupton, 11 Sep 1916, *Johnston, E.L. 760 B* (MO, US); Weld County, Fort Lupton, 12 Sep 1916, *Johnston, E.L. 760* (MO); Denver County, along the Platte River, Denver, 13 Aug 1878, *Jones, M.E. 605* (BM, NY); Larimer County, Temnath, vicinity of New Windsor, 21 Jul 1899, *Osterhout, G.E. s.n.* (F, NY); Las Animas County, slope of Mesa de Maya (Black Mesa) 3 miles N of Kenton, 10 Jul 1947, *Rogers, C.M. 4770* (US); plains near Denver, 19 Sep 1888, *Smith, H.G. s.n.* (US); Boulder County, plains and foothills near Boulder, Jul 1902, *Tweedy, F. 5236* (NY); Baca County, in tributary to East Carrizo Creek 5.5 miles SW of Kirkwell, 29 Aug 1949, *Weber, W.A. & Anderson, J.M. 5086* (UC); El Paso County, Manitou, *Without Collector s.n.* (NY). **Idaho**: Kootenai County, Spirit Lake, "Nicollet's North-Western Expedition", 20 Sep 1838, *Geyers, C.A. 251* (US). **Iowa**: Black Hawk County, 4 Jul 1929, *Burk, M. 829* (MO); Lee County, near western limits of city of Ft. Madison, immediate W of cemetery located on Ia. Hwy 2, 22 Jul 1954, *Davidson, R.A. 2845* (US). **Kansas**: Meade County, Meade County State Park, edge of pond #5 in N group of fish ponds, 23 Jul 1965, *Bare, J. 79* (GH); McPherson County, Lindsborg, Jul 1887, *Bodin, J.E. s.n.* (MIN); Ellis County, Hays, 1 Aug 1969, *Dutt, O.D. 192* (MO); Geary County, along Lyon Creek at Wreford N of US 77 in Lyon tp, 19 Oct 1935, *Gates, F.C. 18861* (MO); McPherson, Jul 1892, *Hitchcock, A.S. s.n.* (MO); Reno County, 25 Aug 1897, *Hitchcock, A.S. 362 a* (GH, MIN, MO, NY, US); Meade County, Meade County Park, edge of hatchery, 14 Jun 1951, *Horr, W.H. & McGregor, ? 3898* (GH, US); Riley County, Manhattan,

10 Jun 1887, *Kellerman, W.A. s.n.* (MO); Wallace County, at Smokey Hill River, 2.8 mi E of Sharon Springs on US 40, 5 Aug 1984, *Lowry, P.P. & Miller, J.S. 11* (MO); Reno County, 3 mi S, 2 mi E of Sylvia, Peace Creek Landand Cattle property, Packetbush parcel, 21 Jun 2006, *Morse, C.A. 13308* (NY); Riley County, Fields, 26 Jun 1895, *Norton, J.B. 362* (GH, MO, NY, US); Ellis County, vicinity of Hays, along Big Creek, west side of College campus, 20 Jul 1929, *Rydberg, P.A. & Imler, R. 1218* (NY); Sedgewick County, along Arkansas River near I 235 bridge in Wichita, 29 Jun 1984, *Walter, D. & Sisco, A.J. 9609* (MO). **Louisiana**: Sin. loc., Apr 1903, *Riddell, J.L. 1229* (K). **Montana**: Phillips County, Bowdoin National Wildlife Refuge, near headquarters, 26 Sep 1940, *Hazeltine, B.M. 1* (US); Sheridan County, Westby, 26 Aug 1927, *Larsen, E.L. 183* (MO). **Nebraska**: Webster County, Red Cloud, 12 Jul 1910, *Bates, J.M. 5200* (NY); Cass County, along Missouri river, 2 Aug 1898, *Fitzpatrick, T.J.; Fitzpatrick, M.F.L. 77* (MO, NY); Cedar County, Randolph, SE side of town along Sholes Road, 17 Jul 2014, *Nee, M. 61297* (MO, NY); Hooker County, 1.9 km N of junction with Hwy. 2 in Mullen, NW side of Hwy. 97 birdge over Middle Loup River (more or less topotype 'on Middle Loup River near Mullen among bushes' -this is the nearest point on the Middle Loup to Mullen), 17 Jul 2014, *Nee, M. 61337* (BM, MO, NY); Hooker County, southern outskirts of Mullen along Hwy 97, 18 Jul 2014, *Nee, M. 61349* (MO, NY); Hooker County, residential area of Mullen, along Hwy 2 near Blaine Ave, 18 Jul 2014, *Nee, M. 61350* (MO); Thomas County, 2.5km WSW of Halsey, near ranger station of Nebraska National Forest, 18 Jul 2014, *Nee, M. 61355* (MEXU, MO); flora of the Sandhills and Western Nebraska, 25 Jun 1912, *Pool, R.J. s.n.* (MO); Hooker County, On Middle Loup River, near Mullen 20 Jul 1893, *Rydberg, P.A. 1385* (GH, H, NEB, NY, US); Garden County, Blue Lake, [part of Crescent Lake National Wildlife Refuge], 27 Sep 1915, *Thomson, R. 320* (US). **Nevada**: Pershing County, Unionville Valley, Oct 1867, *Watson, S. 939* (US). **New Mexico**: **New Mexico**: Grant County, Mangas Canon, 16 miles west-northwest of Silver City, 24 Sep 1944, *Barkley, F.A. 14585 C* (IND, K); Santa Fe County, Santa Fe College Campus, 1 Nov 1935, *Arsène, G. 21830* (US); Bernalillo County, Rio Grande Valley State Park, alogn the E side of the Rio Grande, S of Bridge St in Albuquerque, 3 Aug 2005, *Bay, R.F. MS 28 -6* (DBG); from the Raton Mts to Santa Fe, Aug 1867, *Bell, W.M. 50* (BM); Grant County, Copper Mines [prob = Santa Rita del Cobre, NM; inferred from report of Mexican Boundary Survey collection under direction of Major W.H. Emory], *Bigelow, J.M. s.n.* (NY); Grant County, Fort Bayard Watershed, Forest Nursery, 20 Aug 1905, *Blumer, J.C. 16* (GH, NY); Grant County, Fort Bayard Watershed, Forest Nursery, 27 Aug 1905, *Blumer, J.C. 34* (GH, NY); San Miguel County, vicinity of Las Vegas, Aug 1923, *Bro Anect*, *131* (MO); Sierra County, Tierra Blanca Canyon, bottom of canyon, 24 Aug 1915, *Chapline, W.R. 301* (NY, RM); Sierra County, 4 mi. E of Emory Pass, 9 Jun 1965, *Crutchfield, J.R. 173* (NY); Santa Fe County, Cañada de los Alamos, 25 Aug 1959, *Curtin, L.H. s.n.* (F); along route 90 from Silver City, ca. ? miles S form Junction with Interstate 85, 15 Sep 1976, *Davis, T. 678* (MO); Sierra County, above Hillsboro, along Percha Creek, 7 Oct 1919, *Eggleston, W.W. 16310* (F); Grant County, Silver City, 16 Oct 1919, *Eggleston, W.W. 16436* (F); Grant County, Shelly Ranch, Gila river bottoms above Cliff, 22 Jul 1920, *Eggleston, W.W. 16766* (MO); Capulin Cañon, Sandia Mountains [probably Bandelier National Monument], 30 Jul 1914, *Ellis, C.C. 381* (MO, NY, NY, US); San Miguel County, Conchas Canyon at Cuevas Canyon below Crystal Pasture, 8.5 mi due W of Trujillo, 22 Aug 1982, *Hill, S.R. & Levandoski, P.A. 12182* (GH, MO, NY); San Miguel County, Cuervito Canyon Pasture, T.M. O'Connor Ranch, near windwmil #TM14; 18 mi E of Las Vegas, ca. 5.5 mi S of Rte. 104, 28 Jul 1984, *Hill, S.R. 14851* (GH, NY); Grant County, Dog Spring, 28 May 1892, *Mearns, E.A. 121* (US); Socorro County, Mogollon Mountains, middle fork of the Gila River, 9 Aug 1903, *Metcalfe, O.B. 429* (BM, E, K, MO, NY, US); Sierra County, collected in and around the south end of the Black Range. Kingston, 25 Jun 1904, *Metcalfe, O.B. 1018* (BM, E, GH, MO, NY); sin. loc, *Palmer, E. s.n.* (GH); Dona Ana County, Mesilla Valley, Jul 1906, *Standley, P.C. 482* (US); Grant County, Mangas Springs, 18 miles NW of Silver City, 16 Jul 1928, *Wolf, C.B. 2756* (GH); Dona Ana County, at Mesilla, 22 Jun 1898, *Wooton, E.O. s.n.* (MO); Dona Ana County, Organ Mountains, 20 Sep 1908, *Wooton, E.O. & Standley, P.C. s.n.* (US); Gila bottoms, 12 Jul 1900, *Wooton, E.O. s.n.* (US); Dona Ana County, Mesilla, 22 Jun 1989, *Wooton, E.O. 50* (E, K, NY); Dona Ana County, Mesilla, 22 Jun 1897, *Wooton, E.O. 56* (US); Sierra County, Black Range, approx. 2.5 mi W of Kingston up Percha Creek Canyon, 13 Jul 1985, *Worthington, R.D. 13353* (NY); Hidalgo County, Peloncillo Mts. Coronado Natl. Forest, Clanton Draw 2.3 rd. mi. W of the E side of the natl. forest by Forest Rd. 63, 29 Aug 1986, *Worthington, R.D. 14990* (NY). **North Dakota**: Stutsman County, Jamestown, 24 Sep 1911,

Bergman, H.F. 1355 (UC); Benson County, Leeds, 24 Aug 1898, *Lunell, J. s.n.* (GH); Sheridan County, McClusky, vacant lot in town, 24 Aug 1956, *Moir, D.R. 1645* (US); Cass County, Fargo, 17 Sep 1944, *Stevens, O.A. 802* (UC, US); Baranes County, Valley City, Jul 1898, *Westergaard, C. s.n.* (NY); Kidder County, 10 mi N. 3.5 mi E of Robinson, 8 Nov 1972, *Williams, R.P. 1350* (MO). **Oklahoma**: Cleveland County, Norman, 3 Oct 1914, *Emig, W.H. 373* (MO); Roger Mills County, E1/2, NW 1/4 Cheyenne, S side of town, Cheyenne Memorial Park, 4 Oct 2001, *Freeman, C.C. & Morse, C.A. 18469* (MO); Comanche County, Wichita Wild Life Reservation, 21 Oct 1944, *Hopkins, M. et al. 821* (GH, MO, UC); Woods County, Near Alva, 28 May 1913, *Stevens, G.W. 676* (GH, MO); Blaine County, near Canton, 10 Jun 1913, *Stevens, G.W. 838* (GH, K, MO, NY, US); Kingfisher County, near Hennessy, 1 May 1914, *Stevens, G.W. & Young, V. 1566* (GH); Woods County, near Alva, 11 Jul 1934, *Stevens, G.W. 1679* (GH); Ellis County, near Shattuck, 11 Oct 1913, *Stevens, G.W. 2910 3/4* (GH); sin. loc, *Waugh, F.A. 240* (US); Garfield County, 7 Jul 1899, *White, M. 242* (MO). **South Dakota**: Ewans Siding, S miles SO of Hot Springs [Fall River County], 19 Aug 1926, *Hayward, H.E. 551* (F, NY); Spink County, vicinity of Redfield, 18 Aug 1903, *Ricksecker, A.E. 127* (UC); Fall River County, Hot Springs, Black Hills, 18 Jun 1892, *Rydberg, P.A. 906* (US); Bennett County, LaCreek [National Wildlife Refuge], 8 Dec 1911, *Visher, S.S. 2250* (F). **Texas**: Randall County, Palo Duro Canyon, sandy flood plain of Red River, 2 Sep 1907, *Ball, C.R. 1256* (NY, US); Hartley County, along Punta de Agua Creek, between Romero and Middle Water, 9 Oct 1964, *Correll, D.S. 30328* (LL); Red River Valley, 12 Aug 1900, *Eggert, H. s.n.* (F); Brewster County, Ridge Springs, 9 mi south of Marathon, 15 Jul 1921, *Ferris, R.S. & Duncan, C.D. 2837* (MO, NY); Randall County, Palo Duro Canyon, about 2 miles NE of canyon off Interstate 87, 2 Sep 1977, *Higgins, L.C. 11392* (NY); Tarrant County, sin. loc, 1926, *Killias, O.L. 6922* (US); El Paso County, Belen, 19 Jun 1893, *Mearns, E.A. 1510* (NY, US); Castro County, Dimmitt, Tierra Blanca Creek, 21 Jun 1932, *Reed, E.L. 3555* (US); Hemphill County, 5 miles E of Canadian, 23 Jun 1955, *Rowell Jr, C.M. 4176* (TEX); Brewster County, Green Gulch, Chiosos Mountains, 14 Sep 1941, *Sperry, O.E. 1926* (US); Uvalde County, N part of [Uvalde] County, 15 Jun 1957, *Sperry, O.E. 3099* (UC); Wheeler County, Sand hills, 15 Sep 1950, *Tharp, B.C. 51-341* (MEXU, TEX); Anderson County, Palestine, 7 Jun 1920, *Tharp, B.C. 724* (US); Taylor County, 1 mile N of Abilene, 24 Jun 1943, *Tolstead, W.L. 42507* (GH); sin. loc, *Without Collector s.n.* (W); Bell County, near Little River, *Wolff, S.E. 1305* (US). **Utah**: Washington County, Indian farm, 13 Jun 1899, *Abers*, *s.n.* (UT, W); Salt Lake County, Salt Lake Valley, Jul 1884, *Without Collector s.n.* (UT). **Washington**: Yakima County, ["Squaw Creek Petrified Forest"?], 26 Aug 1902, *Cotton, J.S. 871* (US). **Wyoming**: Goshen County, 2 mi. E of Torrington (T24N R61W Sec 12 N 1/2 N 1/2 N1/2), 30 Jun 1994, *Dorn, R. 5753* (MO).

8. *Solanum macrotonum* Bitter
COSTA RICA. vicinity of El Alto R.R. station on road to Cartago [San José?], 1 Dec 1937, *Allen, P.H. 662* (MO). **Alajuela**: N of Sarchi Norte, c. 8km beyond La Luisa, 3 Feb 1984, *Khan, R. et al. 785* (BM, CR, MEXU); Poás, Poás, cultivated al Indiana University, 26 Mar 1957, *Soria, J. 109* (CR); Naranjo, Environs de San Ramon, 10 May 1913, *Tonduz, A. 17757* (BM, CR). **Cartago**: El Guarco, Distr. San Isidro, Rio Macho. Est. Ojo de Agua. Camino a Torre 46, 16 Apr 1999, *Alfaro, E. & Alfaro, M. 2232* (CR); Oreamuno, Volcán Irazú, pasture along CR route 40 from Cartago to the crater, 21 Jun 1983, *Barringer, K. et al. 3262* (DUKE, MEXU, MO); About 10 km. south of Tapantí along the new road on the east slope above the Rio Grande de Orosi, 10 Jun 1968, *Burger, W.C. & Stolze, R.G. 5717* (CR, DUKE, US); Paraíso, Dist. Orosi, Dense elfin forest of small (5 m) trees and steep slopes with dense growth of shrubs adn bamboo 1...2 m, tall just below the highest point of interamerican Hwy, NW of La Asunción, 5 Feb 1982, *Burger, W.C. & Barringer, K.A. 11508* (CR); Cartago: Oreamuno. Alrededores de la entrada al Parque Prusia, 29 Sep 1995, *Cascante, A. 755* (K); Volcán Irazu, ca. 3km below crater along road to Cartago, 21 Jan 1983, *Davidse, G. et al. 23151* (BM, CR, MO); El Guarco, R.F. Río Macho. Cuenca del Savegre. Estación Ojo de Agua. Sendero el Mascarilla, 12 Jan 1996, *Gamboa, B. & Picado, A. 942* (BM, MO); Oreamuno, Dist. Potrero Cerrado, Slopes SW of Volcán Irazú, 21 Jan 1983, *Garwood, N. et al. 338* (CR); Slope SW of Volcán Irazú, 21 Jan 1983, *Garwood, N.C. et al. 338* (BM, MEXU, MO); Paraíso, Distr. Orori, Cerros de la Muerte, 12 Jan 2012, *González B, F.A. & Morales Q, J.F. 3308* (CR); Paraíso, Distr. Orori, Cerros de la Muerte, 12 Jan 2012, *González B, F.A. & Morales Q, J.F. 3309* (CR); Paraíso, Distr. Orori, Cerros de la Muerte, 12 Jan 2012, *González B, F.A. & Morales Q, J.F. 3310* (CR); El Guarco, 53 km north of San

Isidro del General, 5 Jul 1971, *Harmon, W.E. & Fuentes, J.A. 6138* (MO); Irazú, wooded ravine about 1/2 mile below Finca Robert, 4 Oct 1953, *Heiser, C.B. 3597* (F, IND); Border of Cartago & San José Provinces. Paramo; Cerro de la Muerte, 24 Oct 1971, *Heithaus, E.R. 420* (MO); Volcán Irazú, South slope of Volcan Irazu, on highway about 5 km. NE of Finca Robert, 26 Jun 1949, *Holm, R.W. & Iltis, H.H. 164* (BM, CR, G); Paraíso, Distr. Orori, ca. 4 km oeste de Villa Mills, carretera Interamericana, 16 Aug 2002, *Kriebel, R. 965* (CR); Cartago, Dist. Quebradilla, Cercas de Coris, 24 Oct 1940, *León, J. 207* (CR); Oreamuno, on Highway 8 towards Volcán Irazú, 28-30 km north of San José, 10 Jun 1970, *Luteyn, J.L. 675* (MO); Defrichements de Roble (Massif del Irazu), Jul 1891, *Pittier, H.F. & Durand, T. 4237* (BM); Turrialba, Dist. Santa Cruz, P.N. Volcán Turrialba, 29 Mar 2004, *Quesada H, A. et al. 1293* (CR); Ca. 3.6 km de San Juan de Chicuá, ruta al P.N. Irazú, 13 Mar 2014, *Rodríguez G, A. & Quesada, J.F. 14051* (field observation only); Oreamuno, Distr. Santa Rosa, Cartago, San Gerardo de Robert, 21 Jun 2008, *Santamaría, D. & Solano, D. 7554* (CR); Volcán Irazú, southern slopes, 27 Mar 1967, *Wilbur, R.L. & Stone, D.E. 8733* (DUKE). **Heredia**: Cerro Chompipe (falda norte) San Rafael, 16 Dec 1993, *Cascante, A. et al. 117* (K); Barva, Parque Nacional Braulio Carrillo, From La Montura to los Chorritos, 28 Jan 1984, *Gómez, L.D. et al. 20912* (CORD, K, MEXU); Braulio Carrillo National Park, S edge, above Sacramento on the SW slopes of Volcán Barba, 24 Jul 1980, *MacDougal, J.M. 777* (DUKE); Heredia, Distr. Varablanca, P.N. Braulio Carrillo. Volcán Barva. Sendero Laguna Copey, 20 Jan 2005, *Solano, D. & González, L. 1748* (CR). **Limón**: P.N. Chirripó. Valle de los Leones, 15 Feb 1983, *Garwood, N. et al. 1235* (CR); Chirripó National Park, Valle de los Leones, 15 Feb 1983, *Garwood, N.C. et al. 1235* (BM, MO); Parque Nacional Chirripó. Del Puesto Los Crestones, 2.5 Km al Sur, camino a "La Sabana de los Leones". Bosque de Quercus, afectado por el incendio de 197, 29 Mar 1988, *Robles, R. 1790* (MO). **Puntarenas**: Pérez Zeledón, P.I. La Amistad. Cordillera de Talamanca. Entre las Nacientes de las quebradas Barranca y Río Blanco. Finca San Carlos, 5 Apr 1995, *Aguilar, R. & Garrote, O. 3860* (BM, CR, MO); Monteverde village, Near but not in the Tropical Science Centre Monteverde Cloud Forest Reserve. The pacific side of the continental devide. Disturbed primary/secondary forest or pasture, Aug 1981, *Hepper, D.N. 40* (BM). **San José**: Pérez Zeledón, Cuenca Térraba-Sierpe. Fila Cementerio de la Máquina, 2 May 1997, *Aguilar, R. 5046* (CR, MO); Pérez Zeledón, Parque Nacional Chirripó, Cordillera de Talamanca. Sendero hacia Las Sabanas Chirripó, 26 Jan 1996, *Alfaro, E. & Curso Int Flora Páramo de Chirripó 425* (BM, CR, MO); Pérez Zeledón, Parque Nacional Chirripó, Cuenca Térraba-Sierpe. Camino a Chirripó (por todo el Sendero), 6 Dec 1996, *Alfaro, E. et al. 951* (CR, MO); Dota, R.F. Los Santos. Cuenca del Savegre. Camino a Providencia, colectado a orilla del camino, 20 May 1998, *Alfaro, E. 1657* (CR, MO); Pérez Zeledón, Dist. Páramo, San José/Cartago, 25 km S of El Empalme on the Interamerican Highway roadside, 4 Aug 1994, *Alverson, W.S. & Potgieter, K. 3331* (CR); Pérez Zeledón, near Division, Cerro del la Muerte, 6 Oct 1978, *Antonio, T.M. 651* (CR, MO); Pérez Zeledón, Dist. Rivas, El Sitio II transect. El Sitio Biological Station, below CATIE property, Atlantic slope, Alto de Jaular, Cordillera de Talamanca. Upper montane wet forest. Dominated by oaks, 22 Jun 2002, *Boyle, B. 6284* (CR); Pérez Zeledón, Summit of the Interamerican Highway near La Asunción, provinces of San José and Cartago, 21 Nov 1969, *Burger, W.C. & Liesner, R.L. 6305* (CR, MO); Pérez Zeledón, Summit of the Interamerican Highway near La Asunción, provinces of San José and Cartago, 21 Nov 1969, *Burger, W.C. & Liesner, R.L. 6328* (CR, MO); Pérez Zeledón, Dist. Rivas, P.N. Chirripó. High montane oak forest with trees 8-20 m tall on slopes and ridges along the trail from Canaán to Chirripó vía Los Ángeles above (north of) the río Talari at 3100-3200 m, 19 Jan 1970, *Burger, W.C. & Liesner, R.L. 7492* (CR); Along highway between Cartago and San Isidro, Cerro de la Muerte, vicinity of San Gerardo, 9 Sep 1996, *Croat, T.B. & Hannon, D. 79083* (BM); along Interamerican Highway Rt. 2, km 100, at base of small water fall, 24 Nov 1984, *Crow, G.E. 6258* (MO); Pérez Zeledón, Pacific slope, Cerro Chirripó, 5 Apr 1969, *Davidse, G. & Pohl, R.W. 1624* (MO); Pérez Zeledón, San José-Cartago, Cordillera de Talamanca, upper slopes, western ridge of Cerros Cuericí, 15 Sep 1983, *Davidse, G. 24665* (CR, MO); Pérez Zeledón, San José-Cartago, Cordillera de Talamanca, upper slopes, western ridge of Cerros Cuericí, trail along ridge through oak forest with bamboo (Chusquea) understory, 15 Sep 1983, *Davidse, G. 24685* (MO); Pérez Zeledón, San José-Cartago, Cordillera de Talamanca, upper slopes, western ridge of Cerros Cuericí, 15 Sep 1983, *Davidse, G. 24717* (CR, MO); Cantón de Pérez Zeledón. P.N. Chirripó. Cuenca Térraba-Sierpe, sendero a Cerro Chirripó, Llano Bonito, bosque primario, 4 May 1997, *Gamboa, B. & Alfaro, E. 1371* (MO); Cerro de la Muerte, 4 Apr 1973, *Gentry, J.L. & Burger, W.C. s.n.* (CR); Dota, Pedregoso (in

valley of Río Pedregoso, ca. 2.5 km southeast of Copey), 27 Feb 1990, *Grayum, M.H. et al. 9714* (CR, MEXU, MO); Pérez Zeledón, Dist. Rivas, 6 km East of Siberia near Cerro de la Muerte on Carretera Interamericana, next to Catie Reserve, finca Cuericí, montane rain forest on Pacific slope, 31 Jul 1992, *Haber, W.A. & Zuchowski, W. 11339* (CR, MO); Dota, Distr. Copey, Dota, Camino a Providencia, 2.5Km de Carretera Interamericana Sur, 4 Feb 2005, *Hammel, B. & Pérez, I. 23601* (CR); Pérez Zeledón, Dist. Páramo, Villa Mills. Cerro de la Muerte, 3 Jan 1979, *Hudson, W.D. 306* (CR); Cerro de la Muerte, just off Pan American highway, Atlantic slope, Talamancas, 14 Feb 1981, *Knapp, S. 824* (CR); Pérez Zeledón, Northern slopes of Cerro Buenavista, to south of Interamerican Highway crossing Cerro de la Muerte, 11 Jul 1994, *Kress, W.J. & Flores, T.C. 944285* (MO); Pérez Zeledón, Dist. Páramo, Lado sur de la carretera Interamericana N2 Vía San José, 11 Jun 2005, *Michelangeli, F.A. & Pardo, A. 933* (CR); Dota, Distr. Copey, Providencia Dota. Fila Quebrada Seca, Falda NW, Rio Brujo, 2 km antes Providencia, 6 Mar 2008, *Morales Q, J.F. 16084* (CR); Z. P. Cerros de Escazú. Cedral. Bosque primario y secundario en la cuenca del río Cedral, 1 Sep 1991, *Morales, J.F. 150* (MO); Provincia: San Jose, Canton: Dota. Junction of road to savegre abd Panamerican Highway at bus stop, 28 Jun 2006, *Moran, R. & Stern, S. 7781* (BM); Pérez Zeledón, Parque Nacional Chirripó, Cordillera de Talamanca. Sendero al Mirador, 18 Aug 1995, *Picado, A. & Gamboa, B. 257* (BM, CR, MO); Cerro las Vueltas, 29 Dec 1925, *Standley, P.C. & Valerio, J. 43516* (US); Along Interamerican Hwy. ca. 25.0 km SW of road to La Cima and 4.1 km NW of Cerro Asunción, E end of abandoned section of road, 11 Sep 1979, *Stevens, W.D. 14315* (MO); Pérez Zeledón, North of San Isidro del General, 12 Aug 1971, *Vaughan, J. et al. 641* (MO); P.N. Chirripó, Cerros Los Quemados, camino al Chirripó, 7 Mar 1957, *Weber, H. & Jiménez, A. 1889* (CR); slopes of Cerro Sakira, Buenavista massif, 14 Aug 1969, *Weston, A.S. 5824* (CR, MO).

DOMINICAN REPUBLIC. **La Vega**: A few plants on at base of shale landslide, ca. 7 km above Nuez, 2 Jul 1968, *D'Arcy, W.G. & Hoffman Jr, C. 2653* (MO).

EL SALVADOR. **Chalatenango**: San Ignacio, Depto. Chalatenango. San Ignacio, cerro El Pital, alrededores de la entrada, 22 Jul 2008, *Rodríguez, D. & Monterrosa, J. 1315* (B, BM, INB, LAGU, MEXU, MHES, MO). **Santa Ana**: North slope of Volcán de Santa Ana, 8 Jun 1970, *Davidse, G. & Pohl, R.W. 2054* (CORD, MO).

GUATEMALA. sin. loc, Mar 1879, *Türckheim, H. von*, *384* (BM); La Soledad, Volcán de Acatenango, 8 Aug 1992, *Véliz, M. 92-2226* (MEXU); **Quetzaltenango**: Canyon above Baños Georginas (hot sulfur springs), 8 km above Zunil, 9 Apr 1986, *Gereau, R.E. & Martin, G.J. 1863* (MO). **Sacatepéquez**: Santa Maria de Jesus, Volcán de Agua, Mal Paso, 13 Apr 2000, *Véliz, M. et al. 2M 8445* (MO). **Totonicapán**: Sololá Border, Hwy. CA-1 between Huehuetenango and Chimaltenango, between junction in road to Quezaltenango and Nahualá, ca. 10 mi SSE of junction to Quezaltenango, 23 Jan 1987, *Croat, T.B. & Hannon, D.P. 63499* (MEXU, MO).

JAMAICA. vicinity of Portland Gap, 16 Mar 1920, *Maxon, W.R. & Killip, E.P. 1133* (F); Blue Mountains, coffee plantations, 25 May 1850, *Prior, A. 619* (K); Mountains of Jamaica, 12 Dec 1890, *Rothrock, J.T. 46* (F). **Portland**: Greenhills, 27 May 1962, *Adams, C.D. 11219* (BM); Blue Mountains. Parish: Portland. Cart Falls, growing on large boulder at foot of falls, 29 Dec 1973, *Morley, B.D. & Whitefoord, C. 870* (BM). **Saint Thomas**: Above Portland Gap, 18 Feb 1962, *Adams, C.D. 10620* (BM); Above Portland Gap, 18 Feb 1962, *Adams, C.D. 10653* (BM)Along forestry road North of Union Hill. Roadside bank, 19 Oct 1977, *Proctor, G.R. 37365* (MO); trail from Whitfield Hall to Blue Mountain, 21 Jul 1967, *Weaver, R.E. & Weaver, S. 1132* (DUKE).

NICARAGUA. **Estelí**: Cerro Quiabú, 8 km al noroeste de Estelí; bosque enano, nublado, 19 Oct 1979, *Grijalva, A. & Araquistain, M. 634* (MO).

PANAMA. **Bocas del Toro**: Cordillera de Talamanca, 2-5 airline km NW of the peak of Cerro Echandi on the Costa Rican-Panamanian international border, 1 Mar 1984, *Davidse, G. et al. 25141* (CR, MO); Cordillera de Talamanca, headwaters of the Río Culubre, 6 airline km NW of the peak of

Cerro Echandi on the Costa Rican-Panamanian international border, 2 Mar 1984, *Davidse, G. et al. 25168* (CR, MO); Parque Nacional La Amistad, Cerro Fabrega, 14 Mar 2003, *Klitgaard, B.B. et al. 783* (BM, MO); Parque Nacional La Amistad, Cerro Fabrega, 16 Mar 2003, *Klitgaard, B.B. et al. 807* (BM, MO). **Chiriquí**: Boquete, Cerro Pata de Macho, ca. 5 mi NE of Boquete along trail to Continental Divide which leads to Finca Francisco Serrano (Francisco Serrano: Pacific slope), 22 Nov 1979, *Antonio, T.M. 2651* (MO); Bugaba, Vicinity of Las Nubes, 2.7 miles NW of Río Chiriquí Viejo, west of Cerro Punta, 27 Feb 1973, *Croat, T.B. 22394* (MO); Boquete, 12 miles above Boquete on road to Volcán Barú, 18 May 1976, *Croat, T.B. 34893* (MO); Bajo Grande. Slope of Cerro Respinga above Cerro Punta, 8 Aug 1972, *D'Arcy, W.G. & D'Arcy, J.J. 6569* (MO); La Popa, above Boquete, 20 Mar 1977, *D'Arcy, W.G. 10892* (CORD, F); La Nivera, below summit of El Barú, 14 Mar 1979, *D'Arcy, W.G. & Hammel, B. 12479* (MO); Bugaba, Between Guadalupe and Paseo [Paso] Respingo, 2 Apr 1979, *D'Arcy, W.G. et al. 12823* (MO); Bugaba, Ridgetop above Alto Boquete, 9 Apr 1979, *D'Arcy, W.G. et al. 13127* (MO); Bugaba, Beside Las Cumbres, approximately 1 mile from road near Cerro Punta dairy, 9 Apr 1979, *D'Arcy, W.G. et al. 13160* (MO); Bugaba, Beside Las Cumbres, approximately 1 mile from road near Cerro Punta dairy, 9 Apr 1979, *D'Arcy, W.G. et al. 13177* (MO); Llanos SW side of Barú, 14 Apr 1979, *D'Arcy, W.G. et al. 13268* (MO); Boquete, 11 km directly WNW of Boquete, western slope of Volcán de Chiriquí (Barú) the ridges looking down on Potrero Muleto, 20 Nov 1975, *Davidse, G. & D'Arcy, W.G. 10248* (MO); Road from Boquete to top of Baru; all collections within 7 km top; just past house, 23 Oct 1977, *Folsom, J.P. & Page, R. 6074* (MO); East slope of Volcán Barú; along slopes of draw head waters of Río Calga WSW of Yen Finca, 19 Mar 1979, *Hammel, B. et al. 6555* (MO); E of Cerro Punta roadside W of Paso Respingo below ridgetop, 2 Apr 1979, *Hammel, B. et al. 6637* (MO); Alto Pineda, end of road, right turn just before cooperativa entrance to Cerro Punta, 11 Apr 1979, *Hammel, B. et al. 6997* (MO); Alto Pineda, end of road, right turn just before cooperativa entrance to Cerro Punta, 11 Apr 1979, *Hammel, B. et al. 7003* (MO); Bajo Grande, 1-3 km E of town of Cerro Punta. Roadsides, pastures and remnants of lower montane wet forest, 24 Feb 1974, *Nee, M. 10016* (MO); Chiriquí Volcano, Chiriqui Volcano, El Potrero Camp, 10 Mar 1911, *Pittier, H.F. 3104* (GH, GH, NY, US, US); 3.7 km along road through Bajo Grande from bridge NE of Cerro Punta, 9 Nov 1980, *Sytsma, K. & Stevens, W.D. 2135* (MO).

9. *Solanum nigrescens* M.Martens & Galeotti

BAHAMAS. Coppice, near Fresh Creek, Northern Section, 28 Jan 1910, *Small, J.K. & Carter, J.J. 8805* (F, K, NY, P, US). **Acklin's Island**: Spring Point, 21 Dec 1905, *Brace, L.J.K. 4346* (F). **Berry Islands**: Whale Cay, 29 Jan 1905, *Britton, N.L. & Millspaugh, C.F. 2181* (F); Frozen Cay, 30 Jan 1905, *Britton, N.L. & Millspaugh, C.F. 2210* (F). **Cat Island**: Bennett's Harbor, found in along a road that runs east of the main road, down from Minerva Rolle's house, 29 Oct 1999, *Richey, L.R. 99-712* (ASU). **Grand Bahama**: Eight Mile Rocks, 5 Feb 1905, *Britton, N.L. & Millspaugh, C.F. 2416* (F). **Great Exuma**: Rolletown, 22 Feb 1905, *Britton, N.L. & Millspaugh, C.F. 3088* (F). **Long Cay**: Clarence Town, and vicinity, 16 Mar 1907, *Britton, N.L. & Millspaugh, C.F. 6332* (F). **New Providence**: Nassau, Nov 1890, *Hitchcock, A.S. s.n.* (F); South Shore Beach, 7 Apr 1904, *Millspaugh, C.F. 2270* (F). **Rum Cay**: Rum Cay, east of Port Nelson about 1 mile, edge of old salt lake, 7 Mar 1976, *Gillis, W.T. 13025* (BM).

BELIZE. sin. loc, 1905, *Peck, M.E. 189* (K). **Belize**: Airstrip near St. Johns College, Belize City, 21 Feb 1970, *Dieckman, L. 172* (MO); Belize City; at 35 Gabourel Lane, 8 Sep 1970, *Ugent, D. 85* (MO); Belize City, vacant lots, roadsides, disturbed sites about town. Sci. Coll. Permit CD/60/3/92 (123), 9 Aug 1992, *Worthington, R.D. 21411* (MO). **Cayo**: Chila road, 16 Feb 1993, *Arvigo, R. et al. 730* (MO); Vicinity of old lumber camp at Grano de Oro, 2 Jun 1973, *Croat, T.B. 23380* (MO); Along Sibun River, just W of Hummingbird Highway, 21 Jun 1973, *Croat, T.B. 24847* (MO); Boundary of Cayo and Stann Creek Districts. Hummingbird Highway between Mile 25-34, 13 Aug 1975, *Dwyer, J.D. & Dieckman, L. 13061* (MO); Along Sibun River near crossing of Hummingbird Highway, 21 Jun 1973, *Gentry, A.H. 8445* (MO); Maya Mountains, 6 miles W of Cockscomb range, Jan 1991, *Nash, R. R-4* (K); Xunantunich (Maya ruins), 2 Dec 1968, *Proctor, G.R. 29611* (BM); Ceibo Chico track, 1 Mar 2000, *Short, M.J. et al. 248* (BM, MEXU, MO). **Orange Walk**: Indian Church, 15 May

1977, *Arnason, T. & Lambert, J. 17542* (MO). **Stann Creek District**: Stann Creek, Apr 1916, *Robertson, J. 212* (BM); Stann Creek, 1 Dec 1931, *Schipp, W.A. 842* (BM, K, MO). **Toledo**: Maya Mountains, canyon along Bladen Branch from Richardson Creek to Quebrada de Oro, 12 Mar 1987, *Davidse, G. & Brant, A.E. 32397* (MO); Southern Maya Mountains: Bladen Nature Reserve, area around 'AC Camp' helicopter landing site, along the upper part of the Bladen Branch, 9 May 1996, *Davidse, G. et al. 35727* (BM, MEXU, MO); Collected at first and second bridge near Columbia Forest Station, 26 Jun 1972, *Dwyer, J.D. 9997* (MO); Las Sierritas, 20km west of Big Creek Settlement. Hill 1.2km west of Cerrito in the Las Sierritas hills, 8 Dec 1997, *Hawkins, T. 1763* (BM, MEXU).

CAYMAN ISLANDS. **Cayman Brac**: Vicinity of Tibbett's Turn, 9 Aug 1968, *Proctor, G.R. 29079* (BM).

COSTA RICA. Carretera Panamericana, 26 Aug 1982, *Córdoba, J.J. 2181* (CR); sin. loc., 1877, *Endrès, A.R. 183* (BM, K, W); Aguacaliente, 2 May 1890, *Pittier, H.F. 2438* (G); Río del Convento, vers le Paiso [Puntarenas/San José], 30 Jan 1891, *Pittier, H.F. 3884* (US); sin. loc., Jun 1875, *Polakowski, H. 36* (BM, W); "San José et Cartago", Nov 1853, *Raimann, R. s.n.* (W). **Alajuela**: Reserva Biológica Monteverde Río Peñas Blancas, Quebrada Celeste, 5 Aug 1988, *Bello, E. 249* (CR, MO); San Ramón, Distr. Peñas Blancas, La Torre. Camino y sendero hacia Montewood, 27 Feb 1992, *Bello, E. 4437* (CR); San Ramon, San Rafael de San Ramón, 11 Mar 1983, *Carvajal, A. 528* (MO); San Rafael de San Ramón, 11 Mar 1983, *Carvajal, A. 528* (MEXU); Cordillera Central, 7 mi north of Carrizal, between Volcán Poáz and Volcán Barba, 25 May 1976, *Croat, T.B. 35488* (MO); south of Zarcero, ca. 11 km on route 15;altitude ca. 4700 feet, 18 Jun 1976, *Davis, T. 620* (MO); southeast slope of Volcan Poás, near Poasito, 16 Apr 1973, *Gentry, J.L. & Burger, W.C. 2962* (MO); San Ramón, Dist. Peñas Blancas, R.B. Monteverde. Evergreen cloud forest and wet wind gap formations (Lower montane and premontane rain forest life zone) on and near the continental divide about 2 to 5 km east and southeast of Monteverde, 25 Feb 1977, *Gentry, J.L. 3794* (CR); Monteverde Reserve, Atlantic slope, Río Peñas Blancas valley, on trail above quebrada Leona, 19 Nov 1984, *Haber, W.A. 1032* (MEXU, MO); Peñas Blancas, Collected near Río Penas Blancas, Caribbean side of the Continental Divide, Aug 1981, *Hepper, D.N. 88* (BM); Grecia, Distr. Bolívar, San Miguel Arriba de Grecia, 26 Jan 2003, *Kriebel, R. & Larraguivel, J. 2403* (CR); Beside Quebrada Guillermina, north side of Volcán Arenal, 21 Apr 1973, *Lent, R.W. et al. 3394* (MO); near Quebrada Guillermina, Arenal Volcano, 21 Apr 1973, *Lent, R.W. et al. 3404* (CR, MO); Los Chiles, R.N.V.S. Caño Negro. Llanura de Guatuso. Caño Negro, San Antonio, Punta la Chompipera, Finca la Flor, 26 Aug 1993, *Martínez, K. & Canales, G. 198* (BM, CR, MO); Fraijanes, 20 Jul 1982, *Ocampo, R.Á. 3719* (CR); Poás, Distr. San Juan, Volcan Poas Nat. Pk, 7 May 2003, *Rodríguez G, A. et al. 7925* (CR); Alfaro Ruiz, Distr. Zarcero, Finca de Rogelio Rojas, 20 Apr 2006, *Rodríguez G, A. et al. 10105* (CR); San Juan de Grecia, Río Sarchí, 48 km al NW de San José, 8 Feb 1981, *Téllez V, O. et al. 4282* (MEXU); Valverde Vega, Dist. San Pedro, Rìo Sarchì en San Juan de Grecia, a 48 km al NW de San Josè, 8 Feb 1981, *Téllez, O. et al. 4282* (CR); Rancho de l'Achiote (Poas), Nov 1896, *Tonduz, A. 10810* (US); San Ramón, Environs de San Ramon, 10 May 1913, *Without Collector 17757* (K). **Cartago**: Cartago, Parque Nacional Tapantí, Macizo de La Muerte, Cuenca del Reventazón, colectado a orilla de sendero Alto El Roble, 21 Jan 2000, *Alfaro V, E. & Alfaro, M. 2748* (MO); Cartago, Distr. San Francisco, Alto el Roble, 21 Jan 2000, *Alfaro, E. & Alfaro, M. 2748* (CR); Oreamuno, Distr. Santa Rosa, En las afueras del Parque, 29 Apr 2003, *Alfaro, E. & Quirós, K. 4217* (CR); Oreamuno, Volcán Irazú ca. 15 km from the crater, 6 Jan 1974, *Almeda, F. & Flowers, M. 2386* (MO); El Guarco, Distr. San Isidro, Km 30 Interamericana Sur. Ruta 2, 5 Aug 1994, *Alverson, W. & Prinzie, T.P. 2640* (CR); Cartago, 15 km south of Cartago on Inter-American Highway, 5 Aug 1994, *Alverson, W.S. & Prinzie, T.P. 2640* (CR, MO); El Guarco, Dist. San Isidro, Cerros de Talamanca, UCR. reserve along the carretera Interamericana, Sphagnetum and oak forest, 10 Jun 1983, *Barringer, K.A. 3108* (CR); Oreamuno, Dist. Potrero Cerrado, P.N. Volcán Irazú. pasture along C.R. route 40 from Cartago to the crater, 21 Jun 1983, *Barringer, K.A. et al. 3236* (CR); Cartago, Rancho Redonda, Llano Grande, 24 Oct 1946, *Barrus, M.F. 308* (MO); Paraíso, Dist. Orosi, Dense elfin forest of small (5 m) trees and steep slopes with dense growth of shrubs and bamboo 1-2 m, tall just below the highest point on the Interamerican Highway, NW of la Asunción. San José and Cartago Provinces, 27 Oct 1975, *Burger, W.C. & Baker,*

R.A. 9531 (CR); Cartago, Dist. Llano Grande, Alrededores de la entrada al Parque Prusia, 29 Sep 1995, *Cascante M, A. 755* (CR); Paraiso, Tapantí, Orosi, 29 Jan 1983, *Chacón, I.A. 265* (MO); Cartago, Oct 1887, *Cooper, J.J. 5863* (US); Turrialba, Instituto Interamericano de Ciencia Agrícolas, Turrialba, 24 Feb 1958, *Córdoba, J. 379* (MO); Paraiso, Southeast slope of Cerro de la Muerte, Cordillera de Talamanca, along Interamerican Highway, 23 May 1976, *Croat, T.B. 35405* (CR, MEXU, MO); Parque Nacional Tapanti, along road from main gate to hydroelectric plant, 8-13.5 km from main gate. On rocks near stream, 7 Sep 1996, *Croat, T.B. 79059* (BM, MO); Volcán Irazú, declivitate SW, circa Guayabillos [Finca Guayabillos], 29 May 1930, *Cufodontis, G. 410* (W); Volcán Irazú, declivitate SW, circa Guayabillos [Finca Guayabillos], 22 May 1930, *Cufodontis, G. 438* (W); along trail and summit of Arazlu, 24 Nov 1947, *DeWolf, G.P. 149* (BH, K); Cartago, Valle del Reventazón, El Carmen, Finca de A. Fernández, colindante con el Residencial El Carmen, 8 Feb 1993, *Fernández, A. & Fernández, J.A. 588* (MO); Paraiso, Tapantí, ICE reservation, 21 Jun 1971, *Gentry, A.H. 982* (MO); Turrialba, Sendero al Volcán Turrialba, a unos 2900 m, 3 Aug 1980, *Gómez-Laurito, J. 5674* (CR); Cartago, Dist. Quebradilla, Bermejo, entre Coris y Tobosi, 1 Oct 1980, *Gómez-Laurito, J. 5928* (CR); Hacienda Retes. Volcán Irazú, 7 Mar 1987, *Gómez-Laurito, J. 11378* (CR); El Guarco, Distr. San Isidro, El Guarco, San Isidro, Casa Mata, 1 Nov 2010, *González B, F.A. et al. 1403* (CR); El Guarco, Distr. San Isidro, El Guarco, San Isidro, Casa Mata, 1 Nov 2010, *González B, F.A. et al. 1404* (CR); El Guarco, Distr. San Isidro, El Guarco, San Isidro, Casa Mata, 1 Nov 2010, *González B, F.A. et al. 1405* (CR); El Guarco, Distr. San Isidro, El Guarco, San Isidro, Casa Mata, 1 Nov 2010, *González B, F.A. et al. 1406* (CR); El Guarco, Distr. San Isidro, Carretera Interamericana Cañon del Guarco, cerca de La Escuela, 16 Nov 2010, *González B, F.A. et al. 2461* (CR); El Guarco, Distr. San Isidro, Carretera Interamericana Cañon del Guarco, cerca de La Escuela, 16 Nov 2010, *González B, F.A. et al. 2462* (CR); Paraíso, Distr. Orori, Cerros de la Muerte, 12 Jan 2012, *González B, F.A. & Morales Q, J.F. 3311* (CR); Paraíso, Distr. Orori, Las Torres T.V. 2 Km O. Villa Mills, 18 Mar 1997, *González, J. & Hammel, B. 1833* (CR); Turrialba, Catie. Florencia Norte. Suelo: Serie Colorado, 1 Oct 1981, *Hazlett, D. 3108* (CR); Turrialba, Grounds Instituto Interamericano de Ciencias Agrícolas, 12 Aug 1953, *Heiser, C. 3417* (CR); Turrialba, Interamerican Institute of Agricultural Sciences, 13 Oct 1953, *Heiser, C. 3627* (CR); Cartago, Dist. San Nicolás, Alto de Ochomogo. En terreno humedo alterado, junto a acequia de desagüe, 10 Apr 1987, *Hernández, R. 870410 -7* (CR); Cartago, Rancho Redondo, near Llana Grande, 24 Oct 1946, *Imle, E.P. 309* (MO); 19 Jan 1984, *Khan, R. et al. 352* (BM, CR); La Unión, Dist. San Ramón, San Ramón de Tres Ríos: Westl. Talhang des Río Tiribí, untere Nebelwaldregion, unter Gebüsch am Wege, auf Lehm, 3 Mar 1971, *Kuhbier, H. 179* (CR); Paraiso, near future dam site, 17 km southeast of Tapantí, 27 Apr 1969, *Lent, R.W. 1640* (CR, MO); Cartago, Dist. Quebradilla, Z.P. Cerros de La Carpintera. Forest on Cerros de La Carpintera, 14 Oct 1973, *Lent, R.W. 3661* (CR); Turrialba, Turrialba, Mar 1949, *León, J. 470* (MO); Turrialba, Faldas del Volcán Turrialba, 28 Jul 1955, *León, J. 4631* (CR); El Guarco, Distr. Patio de Agua, Fila Alto Indias. Bosques residuales y arboles en potreros cerca del cruce a Casamata, 7 Apr 2003, *Morales Q, J.F. 9200* (CR); Cerro de la Muerte, 95.5 km from San José on the Pan American highway, 19 Jun 1966, *Mori, S.A. & Anderson, R. 91* (F); Paraiso, near new concrete bridge on electric company's private road, along Río Grande de Orosi, 15 km south of Tapantí, 26 Dec 1974, *Nee, M. et al. 14020* (DUKE, MO); Cartago, Dist. Tierra Blanca, 28 Jul 1976, *Ocampo, R.Á. 1612* (CR); Oreamuno, Dist. Cot, 2 Sep 1982, *Ocampo, R.Á. 3728* (CR); Birris, 30 Mar 1888, *Pittier, H.F. & Durand, T. 148* (G); Cultures a San Rafael de Cartago, 28 Aug 1892, *Pittier, H.F. 6981* (US); Birris, au flanc S de l'Irazu [volcano], 30 Mar 1888, *Pittier, H.F. & Durand, T. 149* (G); Volcan Irazu, 4 Aug 1920, *Rowlee, W.W. & Stork, H.E. 897* (US); Cartago, Dist. San Nicolás, Z.P. La Carpintera. Potrero alrededores de los tanques de agua, 22 Jun 2007, *Sánchez G, J. et al. 1927* (CR); Cerro de La Carpintera, Feb 1924, *Standley, P.C. 34240* (US); Southern slope of Volcan de Turrialba, near the Finca del Volcán de Turrialba, 22 Feb 1924, *Standley, P.C. 34951* (US); Cartago, Cartago, 14 Apr 1928, *Stork, H.E. 1483* (MO); Turrialba, Lower Río Turrialba, 9 Jun 1928, *Stork, H.E. 2459* (MO); Turrialba, Mar 1949, *Toro, R.A. 470* (MO); Turrialba, Distr. Santa Cruz, Mirador caribe. Madriguera de coyotes, 10 Jan 2008, *Vargas, L.D. & Castillo, D. 2936* (CR). **Guanacaste**: Liberia, P.N. Guanacaste. Cordillera de Guanacaste. Estación Cacao, Sendero Arenal, 10 Feb 1995, *Alfaro, E. 74* (BM, CR, MO); Abangares, Dist. Sierra, R.B. Monteverde. Cañitas, Santa Elena, Cabeceras del Río Lagarto, Zona Monteverde, 23 Jun 1988, *Bello, E. 12* (CR); along Río Higuerón near agricultural experimentation area near Taboga, 29 Jun 1977, *Liesner, R.L. et al. 2784* (CR, MO). **Heredia**: Barva,

Distr. San José de la Montaña, Estación Volcán Barva, 24 Jan 2006, *Acosta, L. et al. 3926* (CR); 1.4km E of Peurto Viejo near Río Sarapiqui, 14 Jun 1966, *Anderson, R.C. & Mori, S.A. 3* (BM); Heredia, Parque Nacional Braulio Carrillo, upper Volcán Barva, Atlantic slope, ca. 0.5 km below (to north of) main trail to Laguna Barva, descending vía old "Sendero del Transecto" then traversing 250 m directly east, Treeplot 2750, 28 Apr 1992, *Boyle, B. 813* (CR, MO); Barva, on the southwest slope of Volcán Barba near Sacramento, 26 Jul 1971, *Burger, W.C. & Burger, M. 7699* (CR, MO); Barva, on the southwestern slopes of Volcán Barva, above Sacramento at 2200 to 2300 m altitude and along the road to the National Park at ca. 2500 m, 3 Feb 1982, *Burger, W.C. et al. 11447* (COL, MEXU, MO); Heredia, Dist. Varablanca, Cerro Chompipe (falda norte) San Rafael, 16 Dec 1993, *Cascante M, A. et al. 117* (CR); Barva, Distr. San José de la Montaña, Parcela # 1. Carril # 5, 15 Mar 1995, *Fernández, A. 1526* (CR); Heredia, Río Vueltas (upper Río Patria) on the eastern slope of Volcán Barba, on the Caribbean side of the Continental Divide, 1 Apr 1973, *Gentry, J.L. et al. 2874* (MO); Sarapiquí, Catarata del río La Paz, camino a Sarapiquí, 13 Nov 1980, *Gómez-Laurito, J. 6080* (CR); Barva, Dist. San José de la Montaña, Camino a la Laguna del Volcán Barva, 3 Feb 1982, *Gómez-Laurito, J. 7557* (CR); Belén, Distr. San Antonio, Camino que conduce a Los Playones. Puente Mulas, Planta Electrica Electriona, 4 Oct 2002, *González, J. & Aragón, C. 2196* (CR); Headwaters of Río Santo Domingo, ca. 3 km E of San Rafael de Vara Blanca, N slope of Volcán Barva, 15 Apr 1986, *Grayum, M.H. et al. 7128* (CR, MO); Slopes and ridges above Laguna del Barva and summit of Volcán Barva. 10º08'N 84º06.5'W 2840-2900 m, 27 Apr 1986, *Grayum, M.H. & Quesada, F. 7434* (MO); Southwest, west and north slopes of Volcán Barva, along road from Sacramento to Colonia Jesús María, 29 Apr 1986, *Grayum, M.H. 7523* (CR, MEXU, MO); La Selva Biological Station, 24 May 1980, *Hammel, B. 8783* (MO); Barva, Distr. San José de la Montaña, Entre Porrosati y Sacramento; faldas de V. Barva, 9 Apr 2008, *Hammel, B. et al. 24586* (CR); Barva, Dist. San José de la Montaña, Cuenca del Tárcoles. Entre Porosantí y Sacramento, 9 Apr 2008, *Hammel, B.E. et al. 24586* (CR); La Cinchona, 19 Nov 1953, *Heiser, C.B. 3754* (US); Calle 1 just north of junction with Avenida 9, 10 Feb 1984, *Khan, R. et al. 899* (BM, CR, MO); Santo Domingo, Open lot between Rio Virilla, 6 Jan 1974, *Lent, R.W. 3757* (CR, MO); Roadside, 3km by road N of Vara Blanca, on road towards Puerto Viejo, 2km before Salto La Paz, 25 Dec 1974, *Nee, M. 14004* (BM, DUKE, MO); Rancho Flores, [slopes of Volcán Barva], 22 Feb 1890, *Pittier, H.F. & Durand, T. 2095* (G); Barva, Distr. San José de la Montaña, Montana La Isla, 3 Km al Norte de Porrosati, 21 Apr 1990, *Rivera, G. 214* (CR); San Rafael, Distr. Angeles, Cerro Dantas, 17 Sep 2005, *Santamaría, D. et al. 3035* (CR); Vicinity of Vara Blanca, north slope of Central Cordillera; between Poás and Barba volcanoes, Mar 1938, *Skutch, A.F. 3712* (K, MO, US); Barva, entre Porosatí y Sacramento, cuenca del Tárcoles, 27 Mar 2006, *Solano, D. et al. 2971* (CR, G, INB); Heredia, Variblanca [Vara Blanca], 15 Mar 1976, *Solomon, J.C. & Fallen, M. 2659* (MO); Heredia, Finca Murillo, R.F. Cordillera Volcánica Central, Cuenca del Sarapiquí, 5 Apr 2005, *Soto, A. & González, F. 586* (CR, G, INB); Heredia, Dist. Varablanca, R. F. Cordillera Volcánica Central. Cuenca del Sarapiquí. Finca Murillo, ALAS-1500, 5 Apr 2005, *Soto, A.D. & González, F. 586* (CR); Yerba Buena, northeast of San Isidro, 22 Feb 1926, *Standley, P.C. & Valerio, J. 49646* (US); Yerba Buena, northeast of San Isidro, 22 Feb 1926, *Standley, P.C. & Valerio, J. 49767* (US); Yerba Buena, northeast of San Isidro, 22 Feb 1926, *Standley, P.C. & Valerio, J. 49932* (US); Yerba Buena, northeast of San Isidro, 22 Feb 1926, *Standley, P.C. & Valerio, J. 50022* (US); Cerros de Zurqui, northeast of San Isidro, 3 Mar 1926, *Standley, P.C. & Valerio, J. 50342* (US); Forêts de Rancho Flores [S slope Vocán Barva], 22 Feb 1890, *Tonduz, A. 2095* (US); Barva, Dist. San José de la Montaña, Near the summit of Volcán Barva, aprox 1 km south of Laguna Barva. Pastured slopes and roadbanks, 1 May 1975, *Utley, J.F. & Burt-Utley, K. 2312* (CR); Heredia, Distr. Varablanca, Volcan Barva. Alrededor del mirador de la Laguna, 15 Jun 2006, *Vargas, L.D. & Villalobos, G. 1394* (CR); Barva, Distr. San José de la Montaña, Sector Volcan Barva. Alrededor de la estacion Vieja y area de camping, 8 Feb 2010, *Vargas, L.D. & Villalobos, G. 3949* (CR). **Limón**: Cordillera de Talamanca, head-waters of the unnamed western branch of the Río Teribe, between the Río Sini and te Continental devide at Cerro Bekom, 21 Mar 1984, *Davidse, G. et al. 26150* (BM, CR, MEXU, MO); Cordillera de Talamanca, Atlantic slope, canyon of the Río Siní, 15 Sep 1984, *Davidse, G. & Herrera, G. 29109* (CR, MEXU, MO); Cantón de Talamanca. P.N. Cordillera de Talamanca Cordillera de Talamanca. Frente unión Queb. Kirigú con Río Coén. Entre Ujarrás y San José Cabécar, 28 Mar 1993, *Herrera, G. 6063* (CR, MO); Talamanca, Distr. Bratsi, Bratsi, Parque Internacional La Amistad, sendero Transtalamanca. Entre Ujarrás y San

José Cabécar. Alrededores del Río Lori, 25 Feb 2007, *Rodríguez G, A. et al. 10821* (CR); Talamanca, Parque Nacional La Amistad, Cuelca del Sixaola, Río Coen, 23 Feb 2007, *Santamaría, D. 5838* (BM, CR); Talamanca, Distr. Bratsi, Transecto Transtalamanca, Ujarrás-San José Cabécar, 25 Feb 2007, *Solano, D. 4041* (CR); Talamanca, Parque Nacional La Amistad, Cuelca del Sixaola, transecto 2 y alrededores, 4 Mar 2007, *Solano, D. 4180* (BM, CR); Talamanca, Talamanca, P.N. La Amistad. Cuenca del sixaota. Bratsl. Cercantas de Punto 1-15 Ruta a Laguna Dabagri, 20 Jul 2007, *Solano, D. et al. 4286* (BM, CR); Talamanca, Distr. Bratsi, Valle del Silencio, alrededores del albergue, 17 Nov 2004, *Soto, A. & González, L. 253* (CR); Talamanca, Dist. Telire, P. N. La Amistad. Tararia. Alrededores del albergue-estación, 17 Nov 2004, *Soto, A.D. & González, L. 253* (CR). **Puntarenas**: Coto Brus, Z.P. Las Tablas. Cuenca Térraba-Sierpe. Las Tablas, Sendero Echandi, 10 Aug 1997, *Alfaro, E. et al. 1240* (CR, MO); Buenos Aires, R.I. Boruca-Térraba. Cuenca Térraba-Sierpe. Orilla de la carretera, después de Rey Curré, 5 May 2000, *Alfaro, E. 3067* (CR, MO); Buenos Aires, P.N. La Amistad. Cuenca Térraba-Sierpe. Sendero de Bekon a Tres Colinas, 18 Apr 2002, *Alfaro, E. et al. 3987* (CR, MO); Monteverde, Reserve, Pacific slope, 15 Nov 1985, *Bello, E. 3361* (MEXU, MO); Monteverde, San Luis, Pacific slope, 15 Nov 1985, *Bello, E. 3438* (MO); Cordillera de Talamanca, upper slopes of Cerro Echandi, 23 Aug 1983, *Davidse, G. et al. 23993* (CR, MO); Coto Brus, Dist. Sabalito, Foothills of the Cordillera de Talamanca, lower montane forest in the area of Sitio Cotón (Cotonsito), along the road to Sitio Coto Brus. Forest edge, 3 Sep 1983, *Davidse, G. 24608* (CR); Valle de Coto Colorado, Río Coto Colorado; altitude less than 100 m, 13 Jun 1976, *Davis, T. 609* (MO); R.B. Monteverde. Provincias de Alajuela, Puntarenas y Guanacaste. Cordillera de Tilarán, comunidad, 15 Aug 1976, *Dryer, V.J. & Bello, E. 568* (CR); Along west side of Río Grande de Tárcoles, ca. 0.5 km S of mouth of Río Turrubares. Elev. ca. 15 m. 9º50'N, 84º34'W, 24 Apr 1985, *Grayum, M.H. et al. 5220* (CR, MEXU, MO); R.B. Monteverde, 27 Feb 1974, *Haber, W.A. s.n.* (CR); Puntarenas, Monteverde, Cerro Plano, 3 Oct 1984, *Haber, W.A. 606* (MO); Puntarenas, Monteverde, lower community, 13 Oct 1984, *Haber, W.A. 635* (MO); Puntarenas, Monteverde, upper community on Pacific slope, 14 Aug 1990, *Haber, W.A. & Zuchowski, W. 10051* (CR, F); Puntarenas, Cordillera de Tilarán. Monteverde, cliff edge on Pacific slope, Bajo Tigre trail, 20 Oct 1990, *Haber, W.A. & Zuchowski, W. 10094* (CR, MO); Reserva Biológica Monteverde Road to divide from field station and near La Ventana, Pacific slope, wet and rain forest, 14 Sep 1991, *Haber, W.A. & Zuchowski, W. 10875* (BM, CR, MEXU, MO); Near road leading from Monteverde Biological Reserve, 2 Aug 1994, *Kress, W.J. & Sawyer, N.W. 94-4999* (US); Osa, Rincon de Osa. Airfield, 16 Feb 1974, *Liesner, R.L. 2198* (MO); near San Vito and the Las Cruces Forest, 8 Mar 2001, *Mayfield, M.H. s.n.* (MO); Zona Protectora Las Tablas, San Vito, Coto Brus-Sabalito, Finca Neblinas, 24 Sep 1990, *Mora, G. 105* (CR, MO); Provincia: Puntarenas, Canton: Coto Brus. Near own of San Vito. In secondary forest patch on grounds of Wilson Botanical Garden, 18 Jun 2006, *Moran, R. & Stern, S. 7668* (BM); Puntarenas, Monteverde Cloud Forest Reserve (Reserva Bosque Nuboso de Montevere), Lower Montane Rain Forest (Holdridge Classification), side of road paralleling the Sendero Numboso included in the area bounded by the "triangle tra, 2 Jul 1981, *Murray, K.G. & Feinsinger, P. 811021* (MO); Puntarenas, Monteverde Cloud Forest Reserve, Cordillera de Tilarán, Pacific slope of Continental Divide, 14 Dec 1983, *Pounds, W.Z. 136* (MO); Coto Brus, Distr. Sabalito, Quebrada Pizote, Finca Cafrosa.San Vito, 22 Sep 1990, *Ramirez, M. 94* (CR); Cantón de Coto Brus. Z.P. Las Tablas. Cordillera de Talamanca. Quebrada Pizote, Finca Cafrosa. San Vito, 22 Aug 1990, *Ramírez, M. 94* (MO); Osa, cuenca Terraba-Sierpe, Puerto Cortés, Quebrada Rey (acompaña Joaquin Moreno), 17 Aug 2005, *Santamaría, D. & Moraga, M. 2854* (BM, CR, INB); Buenos Aires, Distr. Potrero Grande, Valle del silencio, camino a Casa Coca, 20 Feb 2006, *Santamaría, D. 3913* (CR); Monteverde, 18 Apr 1907, *Weston, A.S. s.n.* (MO). **San José**: Pérez Zeledón, Cordillera de Talamanca. San Isidro de El General, Repunta, Finca de Miguel Quesada, 1 Aug 1993, *Aguilar, R. & Quesada, F. 2073* (BM, CR, MO); Braulio Carrillo Nacional parck, between Zurqui tunnel and tollbooth on Braulio Carrillo highway, 3 Aug 1994, *Alverson, W.S. 2989* (COL); Vázquez de Coronado, Dist. Cascajal, Pastures and forest remnants along the río Cascajal near the waterfalls at 1600 m, 5 Nov 1978, *Antonio, T.M. 785* (CR); San José, Dist. Zapote, Along calle 13, and above the zoological gardens, 22 Nov 1978, *Antonio, T.M. 852* (CR); Puriscal, San José road from Parrita to Santiago de Puriscal, 1 Mar 1982, *Barringer, K. 1831* (MO); Dota, Provinces of San José and Cartago, montane forest formation with open landslides and road cuts and small swamps, about 22 km southeast of Empalme, along the Interamerican Highway, 9 Aug 1971, *Burger, W.C. 7986* (MO);

Pérez Zeledón, along the trail from Canaán to Chirripó via Los Angeles, above (north of) the Río Talari, 24 Aug 1971, *Burger, W.C. 8338* (CR, MO); Moravia, Dist. San Jerónimo, Pasture land and wooded areas the escarpment with frequent wind and rain from the Caribean, La Palma area, northeast of San Jeronimo, above the La Hondura Valley, 15 Sep 1978, *Burger, W.C. & Antonio, T.M. 11082* (CR); Pérez Zeledón, Dist. Páramo, Western slopes of the General Valley along the Interamerican hwy with evergreen premontane wet forest formations San Jose, 7 Feb 1982, *Burger, W.C. & Barringer, K.A. 11562* (CR); San Jose, University of Costa Rica, San José, 6 Jul 1965, *Croat, T.B. 965* (MO); Lourdes de Montes de Oca, 200 yards NW from railroad crossing, 29 Aug 1960, *Cruz, M. 101* (F); Agua Blanca, Strassenrand zwischen San Ignacio und Colón, sudwestlich San José, 27 May 1989, *Döbbeler, P. 1309* (MEXU); Paraiso, Mar 1896, *Donnell Smith, J. 6667* (US); Parque Bolívar, 1 May 1946, *Echeverría, J.A. 391* (CR); Pérez Zeledón, Distr. Rivas, P.N. Chirripó. Llano Bonito, 4 May 1997, *Gamboa R, B.; Billen*, *& Alfaro, E. 1371* (CR); San Pedro, Campus of University of Costa Rica, San Pedro, 20 Jun 1971, *Gentry, A.H. 949* (MO); Dota, Dist. Copey, R.F. Los Santos. Steep slopes with forest remnants, open areas with bog-like vegetation, and scondary growth along the interamerican Hwy near El Trinidad and km 72. 15 Mar 1973, *Gentry, J.L. & Burger, W.C. 2672* (CR); Dota, along the Interamerican Highway near El Trinidad and Km 72 (about 20 km southeast from Empalme) San José and Cartago Provinces, 15 Mar 1973, *Gentry, J.L. & Burger, W.C. 2675* (MO); Vazquez de Coronado, La Palma, 5 Apr 1973, *Gentry, J.L. & Burger, W.C. 2884* (MO); Interamerican Hwy. above San Isidro, 21 Feb 1977, *Gentry, J.L. 3778* (MEXU, MO); San Jose, Moist open banks of Río Torres, San José, 31 Jan 1965, *Godfrey, R.K. 66028* (MO); San Jose, Along Río Torres, San José, 31 Jan 1965, *Godfrey, R.K. 66048* (MO); Vazquez de Coronado, Parque Nacional Braulio Carrillo, from La Montura to Los Chorritos, 28 Jan 1984, *Gómez P, L.D. et al. 20912* (CR, MO); Costado Este, Lago Dabagri, Talamanca, 11 Sep 1984, *Gómez, L.D. et al. 23321* (MO); A la orilla de la carretera, en las cercanías de Villa Mills. Cordillera de Talamanca, 12 Oct 1980, *Gómez-Laurito, J. 5964* (CR); Dota, Dist. Copey, R.F. Los Santos. Tres de Junio, carretera Interamericana Sur, 28 Jan 1981, *Gómez-Laurito, J. 6309* (CR); Pérez Zeledón, Distr. Rivas, Sendero a Base Crestones, P. N. Chirripó, 29 Jan 2002, *González, L. & Alfaro, E. 1519* (CR); Dota, Along Quebrada Cima, upstream from junction with Quebrada Palmital, ca. 1.5 km (crow-wise) northeast of Copey, 27 Feb 1990, *Grayum, M.H. et al. 9696* (CR, MO); Montes de Oca, San Pedro, east of San José, 25 Jan 1922, *Greenman, J.M. & Greenman, M.T. 5308* (MO); Goicoechea, Distr. Rancho Redondo, Rancho Redondo, Coronado. Ganadera Guayabillos, 12 Oct 2012, *Hammel, B. & Pérez, I. 26442* (CR); Rancho Redondo, near Lleua Grande, 24 Oct 1946, *Imle, E.P. 307* (BH); Rancho Redondo, near Lleua Grande, 24 Oct 1946, *Imle, E.P. 308* (BH); Dota, Dist. Santa María, Bosque Municipal Chalchuapa, Santa María de Dota, 2 Dec 2011, *Jiménez V, J.E. & Chaves F, J.M. 568* (CR); Escazú, Distr. San Antonio, C. Pico Blanco, Escazu, 18 Jul 2002, *Kriebel, R. 503* (CR); Pérez Zeledón, Distr. Rivas, Estacion Cuerici. Colectado a orilla de potrero, 15 Jun 2012, *Kriebel, R. 5785* (CR); High above Río Hondura, 10 Mar 1973, *Lent, R.W. 3228* (MO); near Río Parrita Chiquita, 5 Km N. of Santa Maria de Dota, 10 Oct 1976, *Lent, R.W. 3927* (CR, MO); Sidewalk and vacant lot near Pensión Canada. 1200 m alt. 9º56'N, 84º05'W, 11 Apr 1983, *Liesner, R.L. 14183* (CR, MEXU, MO, QCA); Lomas Ayarco, 15 Jun 1982, *Lumer, C. 1314* (CR); Paraiso, 1896, *Mart*, *6667* (K); San Jose, By a small stream in San José, 13 Dec 1966, *Meyer, F.G. 10044* (MO); Aserrí, Distr. Tarbaca (Praga), Cedral. Bosque primario y secundario en la cuenca del rio Cedral, 1 Sep 1991, *Morales Q, J.F. 150* (CR); Acosta, Distr. Cangrejal, Acosta, Fila El Triunfo, Camino a Fila Aguabuena, 1 Jul 2004, *Morales Q, J.F. et al. 10888* (CR); Dota, Dist. Copey, Pueblo San Gerardo. Savegre Lodge. On road to lodge off of the Pan American Highway Montane Cloud Forest. Disturbed habitat, 17 Jun 2004, *Moran, R.C. & Green, A. 6988* (CR); Dota, Dist. Copey, Providencia de Dota, Reserva Forestal Los Santos, Cordillera de Talamanca. Camino de Providencia a Ojo de Agua, 6 Apr 1996, *Oosterhoorn, M. & Kappelle, M. 230* (CR); Bord des Chemins à San José, Oct 1890, *Pittier, H.F. & Durand, T. 3041* (BM, G); Aserrí, Dist. Tarbaca, Z.P. Cerros de Escazú. Cedral, 11 Dec 2001, *Quesada H, A. et al. 886* (CR); Pérez Zeledón, Distr. Rivas, P.N. Chirripó. Llano Bonito, 29 Aug 2000, *Rodríguez G, A. 6354* (CR); Pérez Zeledón, Reserva Forestal Los Santos, R.F. Los Santos, cuenca del Savegre, Cerro de la Muerte, 22 May 2005, *Santamaría, D. & Torke, B. 2100* (BM, INB); La Palma, 15 Feb 1970, *Schnell, C.E. 1116* (MEXU, MO); La Palma, 17 Mar 1924, *Standley, P.C. 38164* (US); Las Nubes, 20 Mar 1924, *Standley, P.C. 38543* (US); Las Nubes, 20 Mar 1924, *Standley, P.C. 38689* (US); Vicinity of Santa Maria de Dota, 14 Dec 1925, *Standley, P.C. 42524* (US); Near

Finca La Cima, above Los Lotes, North of El Copey, 21 Dec 1925, *Standley, P.C. 42701* (US); Near Finca La Cima, above Los Lotes, North of El Copey, 21 Dec 1925, *Standley, P.C. 42744* (F, US); along Interamerican Highway ca. 8.5 km E of road to La Cima, 30 Jul 1979, *Stevens, W.D. 13390* (CR, MEXU); Ca. 0.7 km N of Tarbaca on road to Aserrí, 26 Aug 1979, *Stevens, W.D. 13669* (CR, MO); Rio Blanca. Above El Copey, 24 Jun 1932, *Stork, H.E. 2997* (MO); Dota, Dist. Copey, Off the Pan Am highway near Cerro de la Muerte, 26 Dec 1977, *Todzia, C.A. 101* (CR); Pâturages de Santa Rosa du Copey, Apr 1898, *Tonduz, A. 12258* (US); Aserrí, Dist. Aserrí, Barrio Las Mercedes. Alrededores del Barrio, 28 Apr 1998, *Valverde, O. 852* (CR); Dota, Distr. Copey, R. B. Cerro Vueltas, 16 Nov 2006, *Vargas, L.D. et al. 1857* (CR); Vázquez de Coronado, Distr. Cascajal, Bajo La Hondura, por la entrada, 8 Mar 2007, *Vargas, L.D. & Castillo, D. 2249* (CR); Vazquez de Coronado, Parque Nacional Braulio Carrillo, P.N. Braulio Carrillo, cuenca del Sarapiquí, La Ventana sector, 6 Mar 2008, *Vargas, L.D. & Castillo, D. 3083* (BM, CR, INB); Dota, Dist. Copey, R.F. Los Santos. San Gerardo, 1 May 1989, *Velzen, H. van, & Genze 73* (CR); Aserrí, Dist. Tarbaca, Ca. 6 km south of Aserrí, 17 Jan 1967, *Weston, A.S. et al. 4148* (CR); Goicoechea, Dist. Rancho Redondo, Pastures. Finca La Cabana, 3 km west of Rancho Redondo. Highway 6, 20 May 1967, *Weston, A.S. et al. 4863* (CR).

CUBA. **Isla de la Juventud**: Pedernales Point, "Isle of Pines" [=Isla de la Juventud], 16 Feb 1899, *Millspaugh, C.F. 1444* (F). **La Habana**: Habana, 27 Nov 1905, *Baber, ?, 3377* (F); Santiago de las Vegas, 1905, *Hermann, H.A. van, 527* (BM, K, MO); Santiago de las Vegas, 19 Mar 1906, *Hitchcock, A.S. s.n.* (F). **Pinar del Río**: San Juan De Zayas to San Gabriel. Thicket, 17 Jan 1912, *Shafer, J.A. 11830* (MO). **Villa Clara**: Mun. Caibarién, Cayo Santa María (parte oriental), sendero ecológico El Bagá, 20 Feb 2005, *Greuter, W. et al. 26375* (PAL).

DOMINICA. South Chiltern Estate between Pointe Michel and Soufrière Bay, 13 May 1964, *Ernst, W.R. 1320* (MO).

DOMINICAN REPUBLIC. **Azua**: Loma Lucí, Hispaniola, Civ. Santo Domingo, Sierra de Vera, prov. de Azua, San Jose de Vera, Loma Lucia, 22 Mar 1929, *Ekman, E.L. 12012* (K); El Número, Km. 98 Carretera Sánchez, Santo Domingo-Azua, 5 Mar 1980, *Mejía, M. 124* (MO). **La Vega**: arroyo just below Las Aguas Blanco, 8 Dec 1986, *Carter, R. 5138* (MO); 37 km NW of Ocoa on road to Constanza (at Nuez), 1 Jul 1968, *D'Arcy, W.G. & Hoffman, C. 2637* (MO); A few plants on recently exposed roadside ca 7 km above Nuez, 2 Jul 1968, *D'Arcy, W.G. & Hoffman Jr, C. 2654* (BH, MO); 10.7 km. W from Autopista Duarte on road to Arroyo Prieto and El Rio; on NE side of road, 8 Apr 1980, *Mejía, M. & Zanoni, T.A. 4984* (MO); 49 km S of Constanza (via El Convento) on road to San José de Ocoa humid rich basin on E side of road in pine forest, 24 Jul 1980, *Mejía, M. & Zanoni, T.A. 7572* (MO). **Elías Peña (La Estrelleta)**: Cordillera Central, Loma Nalga de Maco: entre Pinar Claro y la cima de la loma, 21 May 1992, *Santana, B. et al. 948* (MO).

EL SALVADOR. **Ahuachapán**: San Benito, al W del lote EL Marañón, San Alfonso, 17 Nov 1992, *Sandoval, E. & Sandoval, M. 833* (MO); Laguna Verde, 31 Jan 1999, *Sidwell, K.J. et al. 807* (BM); **Chalatenango**: East slope of Los Esesmiles, 13 Mar 1942, *Tucker, J.M. 1040* (MO). **Cuscatlán**: El Salvador. Cuscatlán. Ecocentro Cojutepeque (CESTA), 11 Mar 1999, *Dar, S. s.n.* (NY); Cojutepeque, 11 Mar 1999, *Dar, S. 3688* (MO, NY). **La Libertad**: San Diego, El Amatal, 12 Nov 1993, *Campos, A. & Ibarra, R. s.n.* (MO); Jardín Botánico La Laguna, Antiguo Cuscutlán, San Salvador, 21 Apr 2008, *Knapp, S. 10145* (BM); Antiguo Cuscutlán, Jardin Botánico La Laguna, zona 32, Parque Ecológico, 29 Apr 2010, *Rodríguez, D. 1888* (BM); Nueva San Salvador, Santa Tecla, 21 Sep 1946, *Williams, L.O. & Molina R, A. 9076* (F, MO). **La Unión**: Cantón Sitio del Niño. La Hacienda. San Juan Opico. La Liberta, 25 Jun 2007, *Gálan, P. 21* (CR); A.N.P. Volcán de Conchagua, alrededores del area administrativa, 24 May 2011, *Rodríguez, D. et al. 2324* (BM). **Morazán**: eastern edge, finca of General J.T. Calderón, Montes de Cacaguatique, 4 Jan 1942, *Tucker, J.M. 714* (CORD, K); Montes de Cacaguatique, about 4 km E of finca General J.T. Calderón, 12 Jan 1941, *Tucker, J.M. 776* (CORD, K). **San Salvador**: Finca Montecristo, 4 Mar 1988, *Berendsohn, W. et al. 1040* (MO); Lake Ilopango, 3 Feb 1998, *Sidwell, K.J. et al. 546* (BM, MO); Lake Illopango, 3 Feb 1998, *Sidwell, K.J. et al. 595* (BM). **Santa Ana**: Cerro Monte Cristo ca. 14 miles NE of Metapán. Disturbed area above Los

Planes, 31 Jul 1977, *Croat, T.B. 42376* (MO); Cerro del Aguila, camino entre La Cumbre y El Olimpo, faldas del Cerro Aguila, 23 May 1993, *Linares, J.L. 374* (MEXU); P.N. Los Volcanes, sector Los Andes, sendero al cráter, 26 May 2005, *Monterrosa, J. et al. 910* (MO); Depto Santa Ana P.N. Los Volcanes, sector Los Andes, el paramo, 21 Jul 2005, *Rodríguez, D. et al. 41* (BM, MO); P.N. Montecristo, Km. 8.5 del parque, 27 Nov 2007, *Rodríguez, D. et al. 1044* (BM, MO); Metapán, Parque Nacional San Diego-La Barra, sector 12, Bosque La Barra, 17 Jan 2011, *Rodríguez, D. et al. 2242* (BM).

GRENADA. Victoria, Wayside, 5 Oct 1945, *Beard, P. 1271* (K, MO).

GUADELOUPE. Sin. loc., *Without Collector s.n.* (MPU). **Basse-Terre**: on road to Soufriere above Sainte Claude, 8 Jul 1964, *Hespenheide, H.A. & Wiseman, D.R. 529* (F).

GUATEMALA. in loc. [Guatemala City?], 16 Oct 1947, *Barrientos, R. 4013* (MEXU); San Rafael près Mixco, 28 Nov 1929, *Rodríguez, L. 1249* (P); sin. loc, *Skinner, F.E. s.n.* (W); Guatemala, *Without Collector s.n.* (MO). **Alta Verapaz**: Along highway CA-14, 5 miles N of Cobán, 17 Jul 1977, *Croat, T.B. 41378* (MO); 1 mi N of Cobán on gravel road that pases new Deportiva, along mud path that leads off road, 11 Dec 1977, *Ramos, C.H. et al. 555* (MEXU); Cubilquitz, [Cubilhuitz on Google Earth], Oct 1903, *Türckheim, H. von*, *8554* (K). **Baja Verapaz**: Along road (National Hwy. 5) between Guatemala City and Rabinal, between Granados and Rabinal; southern slopes of Sierra de Chuacús, 2.8 mi N of El Chol; . 15º00'N, 90º29'W, 25 Jan 1987, *Croat, T.B. & Hannon, D.P. 63596* (MO); Salama, Down slope from Salamá at waterfall along CA-14. (3 miles N of Km. 126), 2 Jan 1978, *Jansen, R.K. & Harriman, N.A. 531* (MO); Unión Barrios, west of km 154, on the Cobán Road, 18 Aug 1975, *Lundell, C.L. & Contreras, E. 19683* (F, MO). **Chimaltenango**: Tecpán, along road to Iximche ruins, 12 Jan 1966, *Molina R, A. et al. 16121* (F); Volcán Acatenango, 22 May 1993, *Sajtoj, I. & Véliz, M. 93-3084* (MEXU). **Chiquimula**: Jocotán, Jocotán, Botanical Garden, 22 Sep 2000, *Kufer, J. 83* (BM, W). **El Progreso**: San Augustín Acasaguastlán, Aldea Tulumaje, Río Tulumajillo, between Tulumaje and Tulumajillo, 10 Mar 2009, *Christenhusz, M.J.M. et al. 5649* (BM, H); San Agustín Acasaguastlán, aldea La Hierba Buena, 31 Jan 1996, *Orellana, R. 17* (MEXU); km 89 camino a Cobán (transecto Pasasagua), 7 Mar 2003, *Ramirez, F. & García, M. 397* (MEXU). **Guatemala**: Guatemala City, 26 Feb 1970, *Harmon, W.E. 2018* (MO); Guatemala, Jardín Botánico, T #88, 10 Oct 1990, *Leysan, M.E. 1382* (MO); Palencia, 26 Apr 1992, *Ramírez, C. 6* (MEXU); El Hippodromo près Guatemala, 28 Apr 1930, *Rodríguez, L. 2316* (P); at the trailer park of Las Hamacas, ca. 20 mi south of Guatemala City, Hwy. CA-2 to Escuintla, 7 Aug 1975, *Torke, K.J. & Dunn, D.B. 484* (MO). **Huehuetenango**: Todos Santos Cuchumatán, Aldea Max, Crucero a Chinhuitz, 26 Feb 2009, *Christenhusz, M.J.M. et al. 5349* (BM); Along the road from Barillas to Malpais, 28 Feb 2009, *Christenhusz, M.J.M. et al. 5385* (BM, H, MO, NY); San Juan Ixcoy, San Juan Ixcoy, orillas del camino, 18 Sep 2006, *Santamaria A, D. et al. 4834* (MO); Aldea Canibal, orillas del Río Cuilco, 23 Nov 2006, *Velásquez, P. et al. 512* (MEXU); Santa Eulalia, carretera a Petén, 19 Oct 1999, *Véliz, M. & Morales, R. 99-7529* (MEXU, MO); La Libertad, Peña Blanca, 19 Apr 2000, *Véliz, M. et al. 2M-9766* (MEXU, MO). **Izabal**: 4 km N of Río Dulce; along road to Modesto Méndez, 26 Jun 1970, *Harmon, W.E. & Dwyer, J.D. 2660* (MO). **Jutiapa**: 12 miles west of Jutiapa on Inter-American highway, 9 Sep 1964, *Elias, T.S. et al. 1671* (MO). **Petén**: [Lake Petén Itza], Santa Elena, Km 1 [on La Cueva de Jobitzinaj Road], 9 Sep 1966, *Contreras, E. 6052* (MEXU, MO); "El Paso, Peten" [Paso Caballos], 19 Apr 1932, *Lundell, C.L. 1512* (MO, US); La Libertad, sitio arqueológico La Joynac, 22 Jun 2001, *Véliz, M. & Luarca, R. 11362* (MEXU); San Luis, 19 Mar 1976, *Ventur, P. 27* (F). **Quetzaltenango**: Quetzaltenango, May 1878, *Bernoulli, K.G. & Cario, R. 2332* (GOET, K); Volcán Zunil, carretera Altotonga "Tlacopacoyan", 7 Aug 1934, *Skutch, A.F. 963* (F); Volcán Pecul, 21 May 2000, *Véliz, M. & Paiz, Y. 2M-9232* (MEXU); Cumbre de Alaska, 6 Jun 2000, *Véliz, M. et al. MV 2M 9254* (BM); Cumbre de Alaska, 6 Jun 2000, *Véliz, M. et al. 2M-9254* (MEXU). **Sacatepéquez**: along road from Palín to Antigua, overlooking a corn field, 18 Jul 1976, *Davis, T. 641* (BM, MO); Santa Teresa of Santa Lucia de Milpas Altas, 14 Nov 1971, *Molina R, A. & Molina, A.R. 27097* (F); Volcán de Agua, Santa María de Jesus, Mal Paso, 13 Apr 2000, *Véliz, M. et al. 2M-8445* (MEXU); San Miguel Dueñas, 5 Jan 2003, *Véliz, M. 12694* (MEXU); San Miguel Dueñas, 17 Aug 2005, *Véliz, M. 16975* (MEXU). **San Marcos**: Tacana, Cunlaj, 14 Nov 2006, *Avila, R. & Quilo, A.*

3517 (MO); Finca Armenia, San Rafael Pie de Cuesta, 7 Aug 1980, *Dwyer, J. 15211* (MEXU); Finca Armenia, San Rafael pie de la Cuesta, 7 Aug 1980, *Dwyer, J.D. 15211* (MO); Finca Armenia, San Rafael pie de la Cuesta to hydroelectric plant, 8 Aug 1980, *Dwyer, J.D. 15238* (MO). **Santa Rosa**: Chiapas, 1892, *Maj, M. 3439* (K). **Sololá**: Aldea El Tablón, Sololá petrol station, 7 Mar 2009, *Christenhusz, M.J.M. et al. 5636* (BM, H, MO, NY); Santa Lucia Utatlán, aldea Chuchexic, 20 Dec 1973, *Cosminsky, S. 26* (F); 3 miles above Panajachel on road to Patzicía around Lake Atitlán, 14 Jul 1977, *Croat, T.B. 41051* (MO); forest of Maria Tecum, vicinity of Los Encuentros, 19 Sep 1971, *Molina R, A. & Molina, A.R. 26647* (F, MEXU); above Lake Atitlán, about 3-5 km west of Panajechel, 6 Dec 1963, *Williams, L.O. et al. 25303* (F). **Suchitepéquez**: Volcán Santa Clara, between Finca El Naranjo and upper slopes, 23 May 1942, *Steyermark, J.A. 46635* (F). **Totonicapán**: San Cristobal Totonicapan, Chuicotón. Caserio, 8 Aug 2007, *Castillo, F. 271* (MO).

HAITI. Massif de la Selle, Pétionville, Morne Tranchant, 1 Jun 1928, *Ekman, E.L. 10010* (G).

HONDURAS. **Atlántida**: El Porvenir, Orillas del Río Bonito. Sobre el nivel del mar, 13 Mar 1977, *Alduvín, C. 129* (MO); La Ceiba, CURLA [Centro Universitario Regional del Litoral Atlántico] y alrededores, 23 May 1977, *Alduvín, C. 189* (MO); Tela, Along Tela river, between Peñas Gordas and Tela; secondary forest and thickets, 10 Apr 1970, *Molina R, A. & Molina, A.R. 25682* (MO). **Copán**: Copán Ruins airport, 2 Jan 1983, *Molina R, A. et al. 32195* (MEXU). **Cortés**: Santa Cruz de Yojoa, Finca Fé, near Peña Blanca, 18 Oct 1980, *Dey, D. & Siegerist, E.S. 107 A* (MO); Cascada Río Cusuco, Sendero Quetzal trail to the Río Cusuco waterfall; Cusuco National Park, 21 Mar 1993, *Hawkins, T. & Allen, B. 690* (MO); San Francisco de Yojoa, Pulhapanzak waterfall. Río Lindo, 12 Dec 1982, *Molina R, A. et al. 31969* (MO); Puerto Cortés, Aldea La Pita; 5 km SO de Puerto Cortés. Clima lluvioso tropical, 26 Nov 1975, *Nelson, C. et al. 3166* (MO). **El Paraíso**: El Paraíso, Río Arriba, El Paraíso, 8 km NO de la ciudad. Bosque mixto húmedo subtropical, 10 Jan 1982, *Escobar N, J.A. 236* (MO). **Francisco Morazán**: Villa Nueva, alrededores, 15 Sep 1978, *Bustilla, S. 142* (MEXU, MO); Distrito Central, Colonia Río Grande, 8 km S de Comayagüela. Bosque seco subtropical, 7 May 1982, *Cámbar, I. 126* (MEXU, MO); Distrito Central, Tegucigalpa, 21 Aug 1972, *D'Arcy, W.G. & D'Arcy, J.J. 6841* (MO); Mpio. Tegucigalpa, 11 km NE of Tegucigalpa. La Tigra National Park, summit, 26 May 1992, *D'Arcy, W.G. 18012* (MO); Distrito Central, Colonia Suyapa, Tegucigalpa, campo abierto. Bosque seco subtropical, 2 May 1982, *Espinal O, F. 237* (MO); Distrito Central, Campo abierto de Ciudad Universitaria, Tegucigalpa, 10 Oct 1978, *Espinal, M. 179* (MO); Distrito Central, Quebrada la Orejona, detras de la Unah, bosque seco subtropical, 23 Apr 1983, *Lorena O, V. 139* (MO); Distrito Central, La Montañita, Tegucigalpa, 24 May 1981, *Martínez C, H.A. 178* (MO); Distrito Central, Tegucigalpa, Cerro Juan A. Laínez [Cerro Juana Laínez], campo libre, 21 Oct 1981, *Mejía, C. 64* (MO); Distrito Central, El Hatillo, 15 km NE de Tegucigalpa, 7 Mar 1982, *Midence, S. 164* (MO); San Antonio de Oriente, San Antonio de Oriente cementery, 12 Mar 1980, *Molina R, A. 31802* (MEXU, MO); Ojojona, Alrededores de Ojojona, 9 Jul 1978, *Tróchez, L. 57* (MO); Zamorano, 25 Oct 1946, *Williams, L.O. & Molina R, A. 10733* (MEXU, US); Distrito Central, Col. 15 de Sept. Tegucigalpa, 12 Aug 1978, *Zelaya, L. 77* (MEXU, MO). **Gracias a Dios**: La Mosquitia, Palacios, embankment of old railway track, 6 Oct 1982, *Ashe, C. 75* (BM); proposed Reserva Biosféra Tawahka Asangi, middle drainage of Río Patuca, 1994, *House, P. s.n.* (BM); proposed Reserva Biosfera Tawahka Asangni, middle drainage of Río Patuca, 1994, *House, P. 1630* (BM); proposed reserva de la Biosfera Tawahka Asangni, middle drainage of Río Patuca, 1994, *House, P.R. 1502* (MEXU); proposed Reserva de la Biosféra Tawahka Asangni, middle drainage of Río Patuca, 1994, *House, P.R. 1511* (BM). **Intibucá**: La Esperanza, La Esperanza. Clima frío, 8 Sep 1979, *Lezama, D.I. 58* (MO). **Lempira**: Gracias, Montaña de Celaque: "Camp Don Thomas" and trail below, 10 km SW of Graci, 10 May 1992, *D'Arcy, W.G. 17877* (MO). **Ocotepeque**: Aldea El Portín, Agua Caliente (Guatemalan border)-Santa Rosa de Copán, 18.1 mi E of Santa Fe; 26.8 mi SW of bridge over Río Higuito (or Río Grande) near village of Cucuyagua Copán, 28 Jan 1987, *Croat, T.B. & Hanlon, D.P. 63776* (COL, MEXU, MO). **Olancho**: Campamento, Campmento. Montaña de Chifiringo, 6 Oct 1979, *Calderón C, R. 95* (MO); Along Río Olancho, on road between San Francisco de la Paz and Gualaco, 13.6 mi SW of Gualaco, in disturbed virgin forest on steep slope ca 1/2 mi E of main road, along gravel road into private property, 6 Feb 1987, *Croat, T.B. & Hannon, D.P. 64242* (MEXU, MO); Catacamas, Márgenes del Río Talgua (Cuevas del Talgua), 8.5 km NE de Catacamas, bosque muy húmedo sub-

tropical, 10 May 1987, *Ortega U, A. 324* (MO); alrededores de Catacamas, 7 Apr 1971, *Rendón, G. & Hernández M, M. 5473* (MEXU). **Santa Bárbara**: Lago Yojoa, Punta Gorda, 2 Nov 1988, *MacDougal, J.M. et al. 3102* (MEXU); alrededores de Aldea Las Vegas, 22 Jul 1976, *Yong, G. et al. 49* (MO). **Yoro**: Santa Rita, Guanchías. Camino a Las Guanchías, 21 Apr 1971, *Mancías, J. & Hernández, J. 1008* (MEXU, MO); Victoria, Victoria. Orilla del Río Sulaco; bosque de vega tropical, 21 Jan 1981, *Nelson, C. 7052* (MEXU, MO).

JAMAICA. **Clarendon**: Mason River Savanna, 2.75 miles due northwest of KELLITS, 28 Mar 1965, *Proctor, G.R. 26316* (BM, MO); **Portland**: Blue Mt. Peak, 13 Dec 1890, *Hitchcock, A.S. s.n.* (MO); Hardwar Gap, 17 Jul 1903, *Nichols, G.E. 89* (F, GH, MO, NY, US, YU); Summit of Blue Mountain Peak...Moist glade of mossy montane forest, 24 Feb 1968, *Proctor, G.R. 28555* (F, MO); 'Muriel's Rock', along road between Section and Hardwar Gap, 24 Nov 1971, *Proctor, G.R. 32736* (BM). **Saint Andrew**: Newcastle, 27 May 1962, *Adams, C.D. 11223* (BM); Grand Ridge of the Blue Mountains, between Harrison's Gap and Sir John Peak, 28 Apr 1990, *Bellingham, P.J. 1199* (BM); Along track between Bellevue and Mt. Rosanna, Port Royal Mts, 26 May 1963, *Proctor, G.R. 23593* (BM); near Upper Alberga House above Newcastle, at roadside, 4 Feb 1956, *Stearn, W.T. 192* (BM, MO). **Saint Thomas**: Upper west slope of Blue Mountain Peak, 18 Sep 1963, *Proctor, G.R. 24026* (BM); St. Thomas (West) parish, between Whitfield Hall and Mossmans Peak, 10 Jan 1956, *Stearn, W.T. 122* (BM, MO). **Trelawny**: Pours, Jan 1982, *Lloyd, R.N. 1136* (MO); sin. loc., *Without Collector s.n.* (G).

MEXICO. Cerros Bravos, 20 Sep 1848, *Gregg, J. 474* (MO); Mirador ni Sabanen, 1844, *Helbrxx, C. s.n.* (W); sin. loc, 1841, *Karwinski, W.H. 577* (LE); sin. loc., Aug 1927, *Lyonnet, E. 231* (K); Col de Valle, Jan 1929, *Lyonnet, E. 362* (K, MEXU); Taviera, *Sallé, C. s.n.* (BM); Valle de Mejico, 1855, *Schmitz, A. 161* (BM, W); 4 May 1985, *Ventura, E. & López, E. 1652* (MEXU); 16 Dec 1995, *Vibrans, H. & Vibrans, G. 5682* (MEXU); 30 Jan 1985, *Villa Kamel, A. 103* (MEXU); sin. loc., 1855, *Berlandier, J.L. s.n.* (BM); sin. loc., *Without Collector 2322* (MO). **Baja California Sur**: Mulegé, Northern Sierra La Giganta. Clear stream at base of Mesa San Felipe, 1.7 mile S of road to San Isidro, 11 miles W of Hwy 1, 3 Apr 1991, *Boyd, S. & Ross, T.S. 5892* (MEXU, MO). **Campeche**: Hopelchén, a 1.4 km del pobaldo Chun-ek, 5 Jan 2004, *Álvarez, D. & Jiménez, I. 7800* (MEXU); Hopelchén, a 2.34 km al E del poblado Chun-ek (laguna), 31 Mar 2004, *Álvarez, D. 8594* (MEXU); Hopelchén, a 4.61 km al ENE de Chan-chen, 5 Aug 2004, *Álvarez, D. et al. 10323* (MEXU); Hopelchén, 4.16 km al N de Xcancha, 2 Apr 2005, *Álvarez, D. 10806* (MEXU); 7 km al NO del entronque a Palizada, carretear Villahermosa-Escárcega, 7 Apr 1985, *Cabrera, E. & Cabrera, H. de 8177* (COL, MEXU); Mun. Ciudad del carmen En el margan del Río Palizada y Carrizales. Dentro del Sistema Pluvio-Lagunar del Río Palizada. Vegetación riparia. 24 Nov 1987, *Cabrera, E. & Cabrera, H. de 14903* (MO); Ciudad del Carmen, en el margen del Río Palizada y Carrizales, dentro del sistema Pluvio-Lagunar del Río Palizada, 24 Nov 1987, *Cabrera, E. & Cabrera, H. de 14903* (MEXU); Ciudad del Carmen, 6 km al NE de Sabancuy, sobre la carretera a Escárcega y Villahermosa, 29 Nov 1987, *Cabrera, E. & Cabrera, H. de 15138* (MEXU); Cuidad de Carmen, 1 km al oeste de Centenario, sobre la carretera Escárcega-Chetumal, 27 Mar 1988, *Cabrera, E. & Cabrera, H. de 15966* (MEXU); Escárcega, 5 km al sur de Ejido Matamoros, 29 Jan 1985, *Chan, C. 4463* (CICY, MEXU); Carmen, 5 kms antes de llegar al limite del Estado de Campeche con Tabasco, 18 Feb 1986, *Chan, C. 6138* (CICY, MEXU); Campo Experimental Forestal Tropical El Tormento, km 5 carretera Escárcega a Candelaria, 24 Dec 1965, *Chavelas P, J. & Quero, H. ES-119* (MEXU); Calkiní, Tankuché, 9 Jun 1988, *Chávez, M. & Simá, P. 148* (CICY, MEXU); Campeche, Pich, cacrretera a Qetal Edzná, 9 Aug 1992, *Faust, ?, et al. s.n.* (MEXU); Escárcega, Candelaria, aprox. 67 km al SSW de Escárcega, 30 May 1988, *Flores Castoreno, A. & Ramirez, R. 718* (MEXU); Champotón, Ejido Revolución, enter Escársega y Champotón, 1 May 2001, *Gutiérrez Baez, C. 7130* (MEXU); Escárcega, Ejido Nuevo Chontalpa, parte norte del ejido, 5 Nov 1999, *Martin, C. 883* (MEXU); Calakmul, Mina de yeso Coconal, a 23 km al W de Xpujil, camino a Escárcega, 12 Aug 1997, *Martínez S, E.M. et al. 28426* (MEXU); Calakmul, a 16 km al N de Xpujil, camino a Dzibalchén, 12 Oct 1997, *Martínez S, E.M. et al. 28823* (MEXU); Calakmul, a 2 km al SW de Dos Naciones, camino a El Civalito, 23 Oct 1997, *Martínez S, E.M. et al. 29272* (MEXU); Calakmul, a 3 km al W de Eugenio Echeverria Castellot I, sobre la carretera Escárcega-Chetumal, 28 Oct 1997, *Martínez S, E.M. et al. 29589* (MEXU); Calakmul, a 300 m al W de 16 de Septiembre, 6 Dec 1998,

Martínez S, E.M. et al. 31546 (MEXU); Calakmul, Pioneros del Río Xno-ha, 6 Jan 1999, *Martínez S, E.M. et al. 31871* (MEXU); Calakmul, Laguna La Valeriana, 5.4 km al E de Bel-há, 27 Aug 2005, *Martínez S, E.M. 38233* (MEXU); San Benito Ixil, camino de Progreso (25 km) a Ozilam de Bravo, 8 Feb 1983, *Téllez V, O. et al. 6430* (MEXU); Campeche, Unidad de Evaluaciuón y Monitoreo de Hampolol, 24 Apr 1996, *Zamora C, P. & Méndez D, D. 4987* (MEXU). **Chiapas**: Ocosingo, Rancho El Edén, 29 Apr 2002, *Águilar M, G. et al. 562* (MEXU); Ocosingo, al E de Crucero de San Javier, 26 Aug 2002, *Águilar M, G. et al. 2294* (MEXU); Las Rosas, road to Las Rosas, from San Cristobal de Las Casas, about 16 km after Teopisca, 6 km before Las Rosas, 2 Dec 2012, *Bohs, L. et al. 3938* (BHCB, MEXU, UT); Tenejapa, paraje of Koltol Te', 10 Jul 1964, *Breedlove, D.E. 6137* (F, IND); Tenejapa, Pokolum, paraje of Sibanil Ha', 22 Nov 1964, *Breedlove, D.E. 7451* (F); San Cristóbal de las Casas, southwest of Mexican Hwy 190 near Rancho Nuevo, about 9 miles southeast of San Cristóbal de las Casas, 5 Mar 1965, *Breedlove, D.E. 9229* (F); Comitán, 4 miles S of Comitán along Mexican highway 190, 23 Jun 1965, *Breedlove, D.E. 10458* (US); Tenejapa, Kulak'tik, 14 Jul 1965, *Breedlove, D.E. 10991* (F, US); Venustiano Carranza, Aguacatenango, 22 Jul 1965, *Breedlove, D.E. 11231* (F); San Cristóbal de las Casas, Steep NE slope of Zontehuitz near summit, with Quercus, 20 Sep 1965, *Breedlove, D.E. 12341* (BM, US, USF); Tuxtla Gutiérrez, Tuxtla Gutiérrez, sold in market, 16 Oct 1965, *Breedlove, D.E. & Raven, P.H. 13400* (US); Ocozocoautla de Espinosa, 32km N of Ocozocoautla along road to Mal Paso, 19 Oct 1965, *Breedlove, D.E. & Raven, P.H. 13588* (BM, MEXU, US); southwest of Mexican Highway 190 near Roncho Nuevo about 9 miles southeast of San Cristobal las Casas. Municipio of san Cristobal las Casas, 7 Nov 1965, *Breedlove, D.E. 14155* (BM, US, USF); Tenejapa, Kulak'tik, 18 Jul 1966, *Breedlove, D.E. 14584* (MEXU, NY, US); Berriozábal, Limestone fissured ridge, 13 km N of Berriozabal near Pozo Turipache and Finca El Suspiro, 24 Jul 1972, *Breedlove, D.E. 26339* (MEXU, MO); Ocozocoautla de Espinosa, 32 km N of Ocozocoautla on road to Mal Paso, 6 Oct 1974, *Breedlove, D.E. 38222* (MEXU); San Juan Cancuc, De San Juan Cancuc. Mo'em 3900 La Gloria 4 km. al, 6 Jul 1990, *Brett, J. 69* (MO); San Juan Cancuc, San Juan Cancuc, Cancuc abajo, potero, 21 Aug 1991, *Brett, J. 350* (MO); San Juan Cancuc, Plantas Medicinales De San Juan Cancuc. Onte'el. milpa, 19 Sep 1991, *Brett, J. 496* (CR, MO); San Juan Cancuc, Yaxa'. San Juan Cancuc, 4 Feb 1991, *Brett, J. 746* (MO); San Juan Cancuc, De San Juan Cancuc. Boj toj 4700 campo, 17 Feb 1992, *Brett, J. 845* (MO); El Chichón, Northside of San Pablo tributary valley, on reworked pyroclastic flow, 18 Feb 1984, *Burnham, R. & Spicer, R. 117* (BM, MO); 3.5 km upstream of Xochimilco along Río Magdalena, 18 Feb 1984, *Burnham, R.J. & Spicer, R.A. 117* (MEXU); a 10 km al S de las Laguans de Monte Bello, cerca de las ruinas de Chincultic, 29 Jun 1982, *Cabrera, E. & Cabrera, H. de 3078* (MEXU); San Cristóbal de las Casas, En el Cerro del Huitepec, al oeste de San Cristobal de las Casas, 4 Dec 1983, *Cabrera, E. & Cabrera, H. de 6021* (MEXU, MO); Ocosingo, A 5 km al N de Ococingo, sobre la carretera a Palenque, 7 Oct 1984, *Cabrera, E. & Huft, M.J. 7587* (MO); Ocosingo, 5 km al N de Ocosingo, sobre la carretera a Palenque, 7 Oct 1984, *Cabrera, E. & Huft, M. 7587* (MEXU); Tenejapa, 12 km al SE de Tenejapa, sobre el camino a San Cristóbal de las Casas, 30 Mar 1985, *Cabrera, E. & Cabrera, H. de 7943* (MEXU); 9 km al S de Palenque, sobre la carretera Palenque-Ocosingo, 6 Apr 1985, *Cabrera, E. & Cabrera, H. de 8162* (MEXU); Villa Flores, Reserva de la Biósfera La Sepultura, 1.37 km al SE de Josefa Ortiz de Dominguez, sobre un arroyo, 22 Apr 2002, *Calónico-Soto, J. et al. 22930* (MEXU); Jiquipilas, a 7.83 km al NO de Tiltepec, 25 Apr 2002, *Calónico-Soto, J. et al. 22937* (BM); Jiquipilas, Reserva de la Biósfera La Sepultura, 1 7.83 km al NO de Tiltepec, 25 Apr 2002, *Calónico-Soto, J. et al. 22937* (MEXU); Ocosingo, Crucero de San Javier, a 2 km al NO de Corozal, 10 Oct 2002, *Calónico-Soto, J. 24111* (MEXU); Ocosingo, a 3.5 km al E de Lancanjá Chansayab, 20 Oct 2002, *Calónico-Soto, J. 24818* (MEXU); Villa Flores, 6 km al O de Los Angeles, 11 Jul 2004, *Calónico-Soto, J. & Álvarez, D. 25517* (MEXU); Zinacantán, Tierra Blanca, 8 Feb 1995, *Chamé, A. & Luna, A. 1206* (MEXU); Ixtacomitán, Solosuchiapa, Jun 1965, *Chavelas P, J. et al. 389* (MEXU); Palenque, Along gravel road between Palenque and Bonampak, 88-90 miles SE of Palenque, 5 Jul 1977, *Croat, T.B. 40212* (MEXU, MO); Escuintla, Along trail between Finca California (at base of S slope of Monte Ovando and ca. 4 km N of of Ovando Turquia) and summit of Cerro Ovando, 14 Feb 1979, *Croat, T.B. 47546* (MEXU, MO); Escuintla, Along trail between Finca California (at base of S slope of Monte Ovando and ca. 4 km N of of Ovando Turquia) and summit of Cerro Ovando, 14 Feb 1979, *Croat, T.B. 47563* (MO); Cintalapa, Along dirt road from Rizo de Oro to Colonia Rodulfo Figeroa (ca. 11 mi N of Rizo de Oro); 5.5 to 9 mi N of Rizo de Oro, 15 Feb 1979, *Croat, T.B. 47582* (MO);

ixtapa, Along Highway 195 from Ixtapa to Pichucalco, 1.5 mi N of Ixtapa, 16 Feb 1969, *Croat, T.B. 47672* (MO); Mapastepec, Sierra de Soconusco, new unfinished road to Tuxtla Gutierrez from Hwy. 200 (5.5 mi NW of turnoff to Mapastepec), 6.5-8.5 mi up the road, 20 Jan 1987, *Croat, T.B. & Hanlon, D.P. 63334* (COL, MEXU); La Trinitaria, Along Highway 190, between Guatemalan border at Cuauhtémoc (El Ocotal) and San Cristóbal de las Casas; 21.4 mi N of border, 22.3 mi S of Trinitaria, small ranch W of Highway, 14 Feb 1987, *Croat, T.B. & Hannon, D.P. 64807* (MEXU, MO); San Cristóbal de las Casas, Along Highway 190, between San Cristóbal de las Casas and Tuxtla Gutierrez, 10.4 mi W of San Cristóbal de las Casas, 15 Feb 1987, *Croat, T.B. & Hannon, D.P. 64864* (MEXU, MO); Ocozocoautla de Espinosa, Along dirt road between San Fernando and Maravillas (near Lago Malpaso), 4-66 mi NW of San Fernando, 15 Feb 1987, *Croat, T.B. & Hannon, D.P. 64980* (MO); Rayón, Along Hwy. 195 between Chiapa de Corzo and Pichucalco, ca. 6 mi NW of Pueblo Nuevo Solistahuacán, along mirador over looking Caribbean slope; Selva Negra lookout point at trail to Col. Pinabeto, vicinity km. 99, 17 Feb 1987, *Croat, T.B. & Hannon, D.P. 65199* (MEXU, MO); Ocosingo, Along road between Palenque and San Cristóbal de las Casas, vicinity of Ocosingo, 3.4 mi W of Ocosingo, 7 Jun 1987, *Croat, T.B. 66137 A* (MO); Acacoyagua, Ejida Las Golondrinas, lower slopes of Cerro Ovando, along road between Golandrinas and Los Cacaos, 22 Aug 1996, *Croat, T.B. 78525* (MO); Acacoyagua, Ejido Las Golandrinas, Cerro Ovando, along road between Los Cacaos and Las Golandrinas, 5km SW of Las Golandrinas, 23 Aug 1996, *Croat, T.B. 78564* (BM); Arriaga, Reserva de la Biósfera La Sepultura, Cerro Monte Bonito, 1 km al SE de Col. Alianza, 29 Nov 2002, *Cruz D, R. & Reyes García, A. 5672* (MEXU); Between San Cristobal de las Casas and Cd Cuauhtemoc, 20 Sep 1978, *D'Arcy, W.G. 12097* (MO); Motozintla, Finca Santa Fé, 24 Jan 1994, *Damon, A. 43* (MEXU); Motozintla, Finca Lorena, *Damon, A. 163* (MEXU); Ocosingo, Lacanjá-Chansayab, 9 Nov 1990, *Domínguez Vázquez, G. 9* (MEXU); Ocosingo, comunidad Lacandona de Nahá, 27 km al SE de Palenque, por la carretera fronteriza hasta el crucero Chancalá, despues 55.6 km por el camino de terracería hacia Monte Libano, 13 Jun 1997, *Durán F, A. et al. 624* (MEXU); Ocosingo, comunidad Lacandona de Nahá, 27 km al SE de Palenque, por la carretera fronteriza hasta el crucero Chancalá, despues 55.6 km por el camino de terracería hacia Monte Libano, 27 Jun 1997, *Durán F, A. et al. 637* (MEXU); Juárez, 1 km al S de Estación Juárez Casa Blanca, 18 Feb 1985, *Espejo, A. & Hernández, S. 1403* (MEXU, MO); La Concordia, Reserva El Triunfo, Cerro Venado, *Farrera S, O. 491* (MEXU); Cintalapa, Reserva de la Biósfera La Sepultura, Carretera México 190, km 29 al N de mirador Iris, al S de Rizo de Oro, 14 Oct 2003, *Flores-Franco, G. et al. 5222* (MEXU); hwy 195, 63.4 miles from the junction of highways 190 and 195; between San Cristobal de las Casas and Tuxtla Gutierrez, below Mirador El Caminero, 27 Nov 1977, *Funk, V.A. & Ramos A, C.H. 2582* (F); Amatenango del Valle, De Amatenango del Valle. Lomol Ja'mal, 26 Oct 1987, *Gómez López, M. 156* (MO); Amatenango del Valle, Barrio Amarillo, 3 km al este de Amatenango del Valle, carretera Teopisca, 15 Feb 1988, *Gómez López, M. 309* (MEXU); Amatenango del Valle, Yax Ak', 4 km al sur del la cabecera municipal, 14 May 1988, *Gómez López, M. 468* (MEXU); Oxchuc, Bajk'en (Bajchen), 8 km al norte de Oxchuc, 12 May 1988, *Gómez S, F. 168* (MO); Oxchuc, Pak'bil NaJ, 3 km al sur de Oxchuc, 20 Mar 1988, *Gómez Santiz, F. 21* (MEXU); Oxchuc, Bajk'en, 8 km al N de Oxchuc, 12 May 1988, *Gómez Santíz, F. 168* (MEXU); chenpil, 4km al poiente de oxchuc carretera internacional, bosque, tierra fría, 15 Jul 1988, *Gómez Santiz, F. 275* (MO); calvario, 1km del centro de Oxchuc al norte bosque, 5 Aug 1988, *Gómez Santiz, F. 310* (MO); Oxchuc, Calvario, 1 km del centro de Oxchuc al norte, 5 Aug 1988, *Gómez Santiz, G. 310* (MEXU); Larráinzar, De San Andrés Larráinzar. Yolte' 1 km oeste monte alto tierra fria, 26 Nov 1987, *González G, L. 239* (MO); Catazajá, crucero a Ignacio Zaragoza, 7 Dec 1998, *Gutiérrez B, C. 6077* (MEXU); Coapilla, Llano Grande, 22 Jul 2003, *Gutiérrez, M.J. & Acero T, ? 484* (MEXU); Jaltenango, El Triunfo Reserve, Trail NNW from El Triunfo camp to Palo Gordo camp, 1km from El Triunfo camp, 20 Feb 1990, *Hampshire, R.J. et al. 488* (BM, MEXU, MO); Pueblo Nuevo Solistahuacán, Clinica La Yerbabuena, 23 Oct 1989, *Heath, M. & Long, A. AM 67* (BM, MEXU); Pueblo Nuevo Solistahuacán, Clinica La Yerbabuena, 23 Oct 1989, *Heath, M. & Long, A. AM 94* (BM); Jaltenango, Reserva El Triunfo, Poligono 1, campamento HQ, Nov 1989, *Heath, M. & Long, A. 540* (MEXU); San Pablo Chalchihuitán, Chakte'al, al NO de la escuela Cantcal, 7 Jun 1988, *Hernández Gutiérrez, E. 64* (MEXU); Villa Corzo, Buenos Aires, 16 Nov 2002, *Hernández Najarro, F. et al. 1250* (MEXU); 22 km from San Cristóbal de las Casas on road to Tenejapa, then right 3 km on the road to Matzam, 29 Sep 1984, *Huft, M.J. et al. 2192* (MEXU); Ocozocoautla de Espinosa, Altos de Ocuilapa, 5 Mar 2009, *Isidro V, M.A. 764* (MEXU);

San Cristóbal de las Casas, along route 190, about 19 miles E of San Cristobal de las Casas, 11 Jun 1960, *King, R.M. 2825* (US); Acala, 6 km SE of Acala along the road to Venustiano Carranza, 26 Feb 1966, *Laughlin, R.M. 321* (MEXU, US); Ocosingo, comunidad Lacandona de Lacanha-Chansayab, a 130 km al sureste de Palenque, por la carretera fronteriza hasta el crucero San Javier, despues 8 km hacia el oeste, 9 Nov 2004, *Lévy T, S. & Durán F, A. 253* (MEXU); Amatenango del Valle, Salul Ja', 5 km al este de la cabecera de Amatenango, 9 Mar 1988, *López Pérez, J. 332* (MEXU); Zinacantán, 22 Sep 1988, *López Santos, B.Y. & Martínez, F. 170* (MEXU); 10 km de recorrido camino a Matzam, Tenejapa, 14 Dec 1988, *López Santos, B.Y. & Martínez, F. 770* (MEXU); San Juan Chamula, Los Ranchos, 20 Jul 1989, *López Santos, B.Y. & Martínez, F. 859* (MEXU); Palenque, La Cascada, 12 Oct 1993, *Lukasser, B. 125* (MEXU); Ocozocoautla de Espinosa, La Roblada, 8 Apr 1971, *MacDougall, T. s.n.* (F); Ishuatán, a 9 km al N de Tapilula, 11 Feb 1983, *Martínez S, E. et al. 3217* (MEXU); Francisco León, en las ruinas del pobaldo El Naranjo, en la zona que afectó al explosión del volcán Chichonal en 1982, 22 Jan 1984, *Martínez S, E.M. 6004* (MEXU); Ocosingo, Crucero Corozal, 170 km al SE de Palenque, camino a Boca Lacantum, sobre la carretera fronteriza del Sur, 23 Feb 1985, *Martínez S, E.M. 11166* (MEXU, MO); Ocosingo, Crucero Corozal, sobre el camino Palenque-Boca Lacantum, 18 Apr 1985, *Martínez S, E.M. 12111* (MEXU); Ocosingo, Arroyo Agua Azul, a 3 km al W de Frontera Corozal, camino a Palenque, 1 Nov 1986, *Martínez S, E.M. 14557* (MEXU); En Arroyo Agua Azul de 3 m. al W de Frontera Corozal, Mpio. Ococingo. Veg. Selva alta perennifolia inundable con Bambu, 15 Jan 1986, *Martínez S, E.M. 16236* (MEXU, MO); Ocosingo, a 12 km al E de Pico de Oro, camino a Benemérito de las Américas, 19 Apr 1986, *Martínez S, E.M. 18413* (MEXU); Unión Juárez, En el volcán Tacaná por el camino de Talquián a la cima del volcán, por la linea divisoria con Giatemala, 6 Feb 1987, *Martínez S, E.M. et al. 19701* (MEXU, MO); Unión Juárez, Volcán Tacaná, a 500 m al E de Talquián, 7 Feb 1987, *Martínez S, E.M. et al. 19728* (MEXU); entre Reserva El Triunfo y Finca Prusia, 2 Jun 1987, *Martínez S, E.M. et al. 21582* (MEXU); Ocosingo, Ejido Chajul, 26 Jan 1993, *Martínez S, E.M. et al. 26163* (MEXU); Ocosingo, Estación Chajul, sobre el Río Lacantún, 23 Apr 1993, *Martínez S, E.M. et al. 26492 A* (MEXU); Villa Flores, a 1.2 km al NO de California, 7 Jul 2004, *Martínez S, E.M. & Águilar M, G. 36650* (MEXU); Acacoyagua, Acacoyagua, 5 Apr 1937, *Matuda, E. 257* (MEXU, US); El Porvenir, Mt. Male, Porvenir, 6 Jul 1941, *Matuda, E. 4618* (MEXU, MO); Villa Corzo, La Pozuma, el Refugio, 27 Dec 2003, *Meléndez López, E. 2256* (MEXU); San Cristóbal de las Casas, Santa cruz en San Felipe, 15 Nov 1986, *Méndez G, A. & López, M.C. de 9480* (MO); Tenejapa, Kulaktik, 10 Sep 1982, *Mendez Ton, A. 4546* (MEXU); Yajalón, Rancho San Luis, 5 Nov 1982, *Mendez Ton, A. 4981* (MEXU); Tenejapa, Kulzak'tik, 30 Jul 1983, *Mendez Ton, A. 6419* (MEXU); San Cristóbal d Las Casas, Santa Cruz en San Filipe, 15 Nov 1986, *Mendez Ton, A. & Martínez de Lopez, M.C. 9480* (MEXU); Ocosingo, 2 km al de Lacanha Cantzayab, 14 Jan 1984, *Narave F, H. et al. 1184* (MEXU); Ocosingo, entre Plan de Ayutla y Velasco Suarez, 21 Jan 1984, *Narave F, H. et al. 1275* (MEXU); Ruinas de Chinkultic, 32 km E of La Trinitaria, 15 Apr 1993, *Neff, J.L. 93-4-15 -1* (MEXU); Ocozocoautla de Espinosa, table land about Ocuilapa, 21 Aug 1895, *Nelson, E.W. 3065 a* (US); Tumbalá, Tumbalá, 20 Oct 1895, *Nelson, E.W. 3319* (US); Las Margaritas, Ejido José Castillo T. 14 Mar 1986, *Ochoa, M. 85* (MEXU); Ocozocuautla, reserva ecológica. Selva alta, 15 Feb 1986, *Ortiz, J.J. 979* (MEXU, MO); Jitotol, Francisco I. Madero, 11 May 1999, *Osorio Hernández, C. 41* (MEXU); La Concordia, desvio hacia Finca Santa Cruz, 5 Nov 1988, *Palacios Espinosa, E. & Breedlove, D.E. 1061* (MEXU); San Pablo Chalchihuitán, Yut Uk'um, Pom, 16 Nov 1987, *Pérez Gómez, M. 76* (MEXU); San Pablo Chalchihuitán, Pom, 4 Jan 1988, *Pérez Gómez, M. 159* (MEXU); San Juán Chamula, Chik'omtantik, 15 Jun 1988, *Pérez López, J. 908* (MEXU); Altamirano, Puebla Nueva, 11 Nov 1987, *Pérez M, A. 13-* (MEXU); Puebla Nueva, Municipio of Altamirano, 17 Oct 1987, *Pérez M, A. 67* (MEXU, MO); Puebla Nueva. Municipio of Altamirano, 21 Oct 1987, *Pérez M, A. 83* (MEXU, MO); Puebla Nueva. Municipio of Altamirano, 11 Nov 1987, *Pérez M, A. 130* (MO); Comalapa, 15 km sobre la carretera Amatenago-Motozintla, 24 Apr 1987, *Reyes García, A. & Martínez S, E.M. 42* (MEXU, MO); Mun. comitán En Lahuna Coilá, 2 Km al E de Uninajab y 500 m al S de Santa Rita (Trapichito). Veg. secundaria a orillas de la laguna, 7 Jul 1990, *Reyes García, A. et al. 1851* (MO); La Concordia, 6 km al N de Finca Custepec, sobre el camino a Finca El Porvenir, 13 Jul 1990, *Reyes García, A. et al. 1867* (MEXU); Mpio, Ocosingo, en las orillas de la Colonia Benito Juárez Miramar, 20 Aug 1993, *Reyes García, A. & Sousa S, M. 2064* (BM, MEXU); Villa Flores, 11 km al W de Agronomos Mexicanos, sobre la terraceria al ejido Nueva Esperanza, 2 Mar 2002, *Reyes García, A. et al. 4234*

(BM, MEXU); Arriaga, Reserva de la Biósfera La Sepultura, cerca de Rancho Monte Bonito, sobre la carretera Mexico 190, 23 Apr 2002, *Reyes García, A. et al. 4483* (MEXU); Tonalá, Reserva de la Biosfera La Sepultura, en el poblado Agua Prieta, cerca a un rio, 27 Apr 2002, *Reyes García, A. & Gómez C, D. 4518* (BM, MEXU); Arriaga, Reserva de la Biósfera La Sepultura, en Pozo Galana, 12 Aug 2002, *Reyes García, A. et al. 5367* (MEXU); Arriaga, Reserva de la Biósfera La Sepultura, al S del ejido Tierra y Libertad, 11 Oct 2003, *Reyes García, A. et al. 5935* (MEXU); Cintalapa, Reserva de la Biósfera La Sepultura, Rancho La Cabaña, 8 Jul 2004, *Reyes García, A. et al. 6904* (MEXU); Villa Flores, Reserva de la Biósfera La Sepultura, Rancho Salto Chiquito, 17 Jul 2004, *Reyes García, A. et al. 7431* (MEXU); Arriaga, Reserva de la Biosfera La Sepultura. Al S del ejido Tierra y Libertad, 11 Oct 2003, *Reyes-García, A. et al. 5935* (MO); km 33 by road S of Sureste on road to Mal Paso (near Tabasco border), 23 Aug 1965, *Roe, K.E. et al. 1378* (MEXU); San Juan Cancuc, Kruzil ja', 3 km de la comunidad de Cancu, 13 Aug 1987, *Santíz Cruz, E. 44* (MEXU, MO); San Juan Cancuc, Muxuk Balumilal, esta en el campo a 3 km al noreste de Cancuc, 9 Mar 1988, *Santíz Cruz, E. 492* (MEXU, MO); San Juan Cancuc, Tz'utoj Te'el, 12 km al noreste del poblado, 22 Mar 1988, *Santíz Cruz, E. 523* (MEXU); San Juan Cancuc, Te' MAa 6 km al sureste del poblado, 12 Jun 1988, *Santíz Cruz, E. 630* (MEXU, MO); San Juán Chamula, Yok Milbil Tulan, 30 Aug 1987, *Santíz Ruiz, C. 74* (MEXU); Tenejapa, Colonia Ach'lum, 27 Dec 1965, *Shilom Ton, A. 439* (F, MEXU, NY); Tenejapa, Paraje Matsab, 5 Jan 1966, *Shilom Ton, A. 468* (MEXU, NY); Tenejapa, Paraje Balum K'anal, 13 Apr 1966, *Shilom Ton, A. 807* (MEXU, NY); Tenejapa, paraje of 'Oshewits, 22 Nov 1966, *Shilom Ton, A. 1578* (F, MEXU); Tenejapa, Yash'anal, 21 Feb 1967, *Shilom Ton, A. 2119* (MEXU); Ocozocoautla de Espinosa, Presa de Malpaso, southwest side, 5 Dec 1967, *Shilom Ton, A. 3314* (MEXU); Pueblo Nuevo Solistahuacán, above Pueblo Nuevo Solistahuacán, 3 Apr 1968, *Shilom Ton, A. 3968* (MEXU); Ocozocoautla de Espinosa, en el río de la cueva Yash Zanal, 9 Apr 1982, *Shilom Ton, A. 4184* (MEXU); Tenejapa, Kulaktik, mpio Tenejapa, 10 Sep 1982, *Shilom Ton, A. 4546* (MO); Ocosingo, Estación Chajul, camino a Miranda 2 km, Selva Lacandona, *Sinaca Colín, S. 1139 bis* (MEXU); Jiquipilas, A 7.83 km al NO de Tiltepec, 25 Apr 2002, *Soto, J.C. et al. 22937* (MO); Motozintla, Track from Ejido Boqueron to Cerro Boqueron, 9 Feb 1990, *Stafford, P.J. et al. 379* (BM, MA, MEXU, MO); San Cristóbal de las Casas, jsut N of San Cristóbal along the road to Tenejapa about 15 miles N of its junction with the pereferico, 30 Jul 1983, *Taylor, C.M. 2634* (DUKE); La Trinitaria, A 3 km al E de Trinitaria, camino a Las Lagunas de Montebello. Veg. Ruderal, secundaria, 17 Nov 1984, *Téllez V, O. et al. 7991* (MEXU, MO); Acapetahua, Montaña de Zacualpita, a 12 km al sureste del pobaldo de Escuintla, 23 Aug 1988, *Trujillo Eslava, R. et al. 56* (MEXU); Ocosingo, Yaxchilán, campamento arqueologico del INAH, Río Usumacinta, 9 Apr 1998, *Valle Doménech, A. 185* (MEXU); Ocosingo, Yaxchilán, campamento arqueologico de INAH, 4 Feb 1999, *Valle Doménech, A. 272* (MEXU); Ocosingo, 3 km al W de Plan de Ayutla, 16 Jan 1984, *Vázquez B, F. & Avendaño Reyes, S. 1590* (MEXU); Villa Corzo, Arroyo La Vainilla, 6 Aug 2003, *Vázquez Hernández, J.P. 51* (MEXU); Villa Corzo, La Nueva Dos, 18 Jul 2003, *Vázquez Hernández, J.P. 340* (MEXU, MO); Mazatán, Ejido 4 Caminos, 10 Dec 1984, *Ventura, E. & López, E. 868* (MEXU); Unión Juárez, Unión Juárez, 8 Feb 1985, *Ventura, E. & López, E. 1091* (MEXU); Cacahoatán, La Palma, 28 May 1985, *Ventura, E. & López, E. 1780* (MEXU); Cacahoatán, Cuatimoc, 19 Jul 1985, *Ventura, E. & López, E. 2121* (MEXU); Cacahoatán, El Progreso, 17 Sep 1985, *Ventura, E. & López, E. 2423* (MEXU); Unión Juárez, Ejido 11 de Abril, 8 Feb 1986, *Ventura, E. & López, E. 3208* (MEXU); Cacahoatán, Unión Roja, 11 Mar 1986, *Ventura, E. & López, E. 3349* (MEXU); Unión Juárez, 5 km al NE de 11 de Abril, 20 Jul 1988, *Ventura, E. & López, E. 5365* (MEXU). **Chihuahua**: Near Creel east fork of Río Otures; elevation 7200 feet, 29 Jun 1977, *Davis, T. 703* (MO); bank of canyon Barranca de Cobre, 1 Jul 1977, *Davis, T. 712* (MO); Along arroyo bank among rocks toward Hueymayo, 1 Jun 1984, *Davis, T. 1132* (MO); Along arroyo bank among rocks toward Hueymayo, 1 Jun 1984, *Davis, T. 1133* (MO); Along arroyo bank among rocks toward Hueymayo, 1 Jun 1984, *Davis, T. 1134* (MO); SW of Nuevo Casas Grandes in Sierra Madre Occidental. Ca. 3 miles E of fork in road leading to Colonia Garcia and Colonia Pacheco en route from Colonia Juarez, 4 Sep 1979, *Keil, D.J. et al. 13323* (ASU); Chihuahua, 1 Sep 1886, *Pringle, C.G. s.n.* (MEXU); Fern Canyon, side canyon of Santa Elena Canyon of the Río Grande, on S (Chi.) side of river, ca. 3 mi W of Sta. Elena picnic area of Big Bend National Park, Texas, near mouth of canyon along river, 24 Nov 1973, *Wendt, T. & Lot, E.J. 90* (MEXU); Aquiles Serdán, Sta. Eulalia plain, Sep 1885, *Wilkinson, E. s.n.* (US). **Coahuila**: along hwy 57 ca .20 mi s of Saltillo, *Dunn, D.B. et al. 22489* (MO); along stream tributary to Río Babia, between Nueva Rosita

and Palau, 18 Jun 1976, *Fryxell, P.A. 2669* (IND, MEXU); Saltillo, Saltillo, 5 Dec 1946, *Hinton, G.B. 16619* (GH, US); Muzquiz, Sabinas River, 11 Jul 1936, *Marsh, E. 394* (GH, MEXU); Muzquiz, Múzquiz, Sabinas River, 11 Jul 1936, *Marsh, E.G. 394* (F); Monclova, Hermanas, 20 Apr 1939, *Marsh, E.G. 1589* (F, GH); Monclova, Monclova, 7 May 1939, *Marsh, E.G. 1719* (F, GH); Saltillo and vicinity, 1898, *Palmer, E. 119* (BM, F, K, MO, NY, US); 3 kilo. southwest of Fraile, 11 Jul 1941, *Stanford, L.R. et al. 345* (MO); vicninity of La Noria a broad valley in the calcareous Sierra del Pino, western Coahuila, 27 Aug 1941, *Stewart, R.M. 1215* (F); 25 miles SW of Monterrey, 1 Dec 1945, *Warnock, B.H. & Barkley, F.A. 14889* (NY). **Colima**: Comala, Rancho El Jabalí, NE side of Lago El Jabalí, 22 km (airline) NNW of Colima in the SW foothills of Volcán de Colima, Colima/Jalisco line passes through the ranch, 19 Jun 1991, *Vázquez V, L. & Phillips, B.L. 611* (MEXU, NY); Comala, Rancho El Jabal, 22 km (airline) NNW of Colima in the SW foothills of hte Volcán de Colima, Colima/Jalisco line passes through ranch; on road from headquarters to Lago Epazote, 14 Jul 1991, *Vázquez V, L. & Phillips, B.L. 877* (MEXU, NY); Comala, Rancho El Jabali, 20 km (airline) N of Colima in the SW foothills of the Volcan de Colima. Near Lago Epazote, in a cleared area near the chicken ranch, 4 Aug 1991, *Vázquez V, L. 1017* (NY). **Distrito Federal**: Sierra de Guadalupe, 21 Jul 1938, *Balls, E.K. B 5067* (BM, US); Gustavo A. Madero, Cerro del Chiquihuite, 4 Jan 1976, *Cervantes G, C. 169* (MEXU); Base of road and Cerro Ajusco, 15 Sep 1978, *D'Arcy, W.G. 11909* (MO); Base of road and Cerro Ajusco, 15 Sep 1978, *D'Arcy, W.G. 11909* (MO); Pedregal de San Angel, 18 Oct 1965, *Diego P, N. 220* (MO); Ciénagas de Lerma, 12 Nov 1978, *Fuchs, F.M. 115* (MEXU); Area Silvestre de la Escuela Nacional de Antropología e Historia, 20 Jan 1993, *Hernández del Olmo, J. 356* (MEXU); Cerro de León, Valle de Mexico, 19 Mar 1950, *Matuda, E. 19121* (F); Tlalpan, Parque Ecológico de la Cd. de Mexico, por las vias del ferrocarril, 15 May 2002, *Mora G, E. et al. 196* (MEXU); La Magdalena Contreras, Cañada de Contreras, cerca del primer dinamo, Area Natural Protegida Bosques de la Magdalena Atlitic, 7 May 2004, *Rivera H, J. et al. 3661* (MEXU); near Eslava, 19 Jul 1910, *Rusby, H.H. 145* (US); near Eslava, 19 Jul 1910, *Rusby, H.H. 334* (US); 1 km al norte de Tlaltenco, delegación de Tláhuac, 13 Dec 1980, *Rzedowski, J. 37140* (CORD); Tlalpan, La Cumbre, al W del Pueblo de Parras, 6 Oct 1994, *Tenorio L, P. 18947* (MEXU); Chapultepec, 27 Jul 1872, *USDA, s.n.* (US); Pedregal de San Angel, al sur de C.U. junto al ducto al principio de la gran hondonada, 27 Nov 1981, *Valiente Banuet, A. & Alumnos 65* (MEXU); Tlalpan, Centro de Educación Ambiental Ecoguardas, 12 Sep 1996, *Velázquez, N. et al. 68* (MEXU); Xochimilco, Santa Cecilia Tepetlapa, 11 Aug 1976, *Ventura A, A. 2032* (CORD, MO). **Durango**: Sáchil, potrero El Olvido, rancho El Temascal, Reserva de la Biosfera, 27 Sep 1985, *Alvarado, J. 106* (MEXU); Mesquital, 11 Aug 1982, *González Elizondo, M.S. 458* (MEXU); Tamazuda, La Bajada, *González Ortega, J. 585* (MEXU); Mezquital, ca. 3 km al W de Santa Maria Ocotán, 15 Jul 1984, *González, M. 1410* (MEXU); Tepehuanes, 30-35 km al W de Tepehuanes, hacia Tapia, 22 Jul 1982, *Hernández Magaña, R. 8153* (MEXU); Vicente Guerrero, camino al Rancho Los Ojitos, 3 Oct 1984, *Jiménez, R. & Acevedo, S. 153* (MEXU); torre de microondas Fco. I, mader Pánuco de Coronado, Aug 1992, *Martínez Marín, J.L. 402* (MEXU); El Taray, Reserva de la Biosfera "La Michilia" a 70 km al SE de la cuidad de Durango, 28 Jul 1981, *Morales Garcia, M.A. 43* (MEXU); Hombre de Dios 45 m S of Durango, Apr 1896, *Palmer, E. 101* (BM, F, K, MO, NY, US); San Ramón, 29 Aug 1908, *Palmer, E. 167 [b]* (K); El Salto, 2 km del entronque de la brecha a Chavarria con la carretera Durango-Mazatlán, 10 Jul 1982, *Tenorio L, P. & Romero de T, C. 968* (MEXU); Tayoltita, El Pino, 20 km del entronque a Spaioris, con la Brecha Coyotes-San Miguel de Cruces, 8 Jul 1984, *Tenorio L, P. et al. 6317* (MEXU). **Guanajuato**: Alrededores de Sierra Verde, carr. León-San Felipe, municipio de san Felipe. fondo de cañada, 1998, *Galván, R. & Galván, J.D. 3266* (MO); San José Iturbide, 2 km al NW de El Capulin, km 9 carretera San José Iturbide-Tierra Blanca, 21 Sep 2002, *Gutiérrez G, J. & Castillejos, C. 558* (MEXU); Guanajuato, Cañada de La Virgen, antigua carretera Guanajuato-Dolores Hidalgo, 7 Sep 1993, *Pérez C, E. & Diaz B, H. 2795* (MEXU); Victoria, San Agustín, en los alrededores, 11 Aug 1991, *Rzedowski, J. 50841* (MEXU); Uriangato, 4 km al N de Uriangato, 16 Oct 1992, *Rzedowski, J. 51817* (MEXU). **Guerrero**: Tlacoachixtlahuaca, Jicayán de Tovar, orilla S del pueblo, 2 Apr 1986, *Avila, A. de, 52* (MEXU); Chilpancingo, en la cuidad, cerca del COnjuunto Jacarandas, 6 Jun 1978, *Blanco, M. & Toledo, O. 279* (MEXU); El Paraiso, 7.27 km al N, 19 Jul 2005, *Calónico-Soto, J. et al. 27103* (MEXU); Iguala, Cañon de La Mano, entre Los Amates y El Naranjo, 10 km al N de Iguala por el ferrocarril, cerca de tunel no. 2 (border Mpio. Buenavista), 21 Sep 1986, *Catalán H, C. 204* (MEXU); Iguala, Cañon de La Mano, entre Los Amates y El Naranjo, 10 km al N

de Iguala por el ferrocarril, cerca de tunel no. 2 (border Mpio. Buenavista), 27 Sep 1986, *Catalán H, C. 234* (MEXU); Iguala, Cañon de La Mano, entre Los Amates y El Naranjo, 10 km al N de Iguala por el ferrocarril (border Mpio. Buenavista), 24 Jan 1987, *Catalán H, C. 633* (MEXU); Taxco, 5 Sep 1959, *Degener, O. & Degener, I. 26198* (NY); José Azueta, ca. 100 m al S del caserio La Vainilla, camino al mirador, 23 Mar 1990, *Gallardo, C. et al. 393* (MEXU); Cuautepec, San Agustin Cuilutla, Costa Chica, 10 Jul 1985, *Herrera Castro, N. 89* (MEXU); Acapulco, Puente de la Estancia, 24 Jul 1982, *Iizuka, M. & Yokoi, M. 41* (MEXU); En la estacíon demicroondas El Fresno, a 6 Km. al E de El Ocotito, carr. Acapulco-Chilpancingo, Municpio Mochitlán. Bosque de pinus, 25 Mar 1982, *Martínez S, E.M. & Téllez V, O. 193* (MEXU, MO); Atoyac de Álvarez, a 10 km al SW de Puerto del Gallo, camino a Atoyac, 20 Oct 1983, *Martínez S, E.M. et al. 5093* (MEXU); Zihuatanejo, 37 km al NE de Zihuatanejo camino a Cd. Altamirano, 20 Nov 1983, *Martínez S, E.M. & Carrie, F. 5414* (MEXU); along the dirt road between Atoyac and Filo de Caballo near Nueva Deli, 1 Feb 1983, *Miller, J.S. & Tenorio L, P. 601* (MEXU, MO); Acapulco, plants of Mexico collected in acapulco and vicinity, Oct 1894, *Palmer, E. 554* (NY, US); 21 km al SO de Teloloapan, carretera Iguala-Ciudad Altamirano, 25 Oct 1984, *Soto N, J.C. 6709* (F, MEXU); Chilpancingo, 6 km al NW de Omiltemi, brecha Chilpancingo-Omiltemi-Las Joyas, 11 Nov 1982, *Tenorio L, P. et al. 2633* (MEXU); Alcozauca, Amapilca, Sep, *Viveros, J.L. & Casas, A. 95* (MEXU); Alcozauca, San José Lagunas, 9 Sep 1983, *Viveros, J.L. & Casas, R. 168* (MEXU); Malinaltepec, Malinaltepec, 17 Jul 1989, *Wagenbreth, I. 68* (MEXU); Malinaltepec, Malinaltepec, 15 Aug 1989, *Wagenbreth, I. 120* (MEXU). **Hidalgo**: Acaxochitlán, 4 km por el camino Acaxochitlán-Chimapala, 27 May 2008, *Contreras J, J.L. 9574* (MEXU); Along Highway 105 between Pachuca and Tampico, 18-23 mi S of Huejutla. Roadside vegetation. Vegetation type: selva alta perennifolia, 28 Feb 1987, *Croat, T.B. & Hannon, D.P. 66001* (MO); Ajacuba, Cerro El Crestón, ca. 2 km antes de llegar a la desviación al centro de Emiliano Zapata, rumbo a Ajacuba sobre la carretera pavimentada, ejido Tecomatlán, 24 Aug 1988, *Díaz Vilchis, I. & Valverde G, A. 120* (MEXU); Tlachinol, 2.5 km al E de Tlanchinol, 21 May 1976, *Flores Martinez, ?, 255* (MEXU); between pachuca and Real del Monte, 27 Aug 1944, *Goodman, G.J. 3413* (F); Pachuca, 15 kms. al Este de, 5 May 1980, *Hernández Magaña, R. & Hernandez V, R. 4365* (MO); Zempoala, Xochihuacan, 13 km al norte de Zempoala, 3 Nov 1980, *Hernández Magaña, R. 5257* (MEXU, MO); Tulancingo, Jaltepec, a 5 km. al Norte de Tulancingo. Entre cultivos de maíz y alfalfa. Suelo casi negro, profundo, 4 Nov 1980, *Hernández Magaña, R. 5267 [b]* (MO); Tenango de Doria, Santa María, 22 kms al este de Tenango de Doria, 20 Feb 1981, *Hernández Magaña, R. & Rodríguez B, D. 5524* (MEXU, MO); Metztitlán, Zoquizoquiapan, 5 kms. al norte de Alumbres. Bosque perturbado de encinos y pinos principalmente. Suelo rojizo, profundo, 26 Apr 1981, *Hernández Magaña, R. et al. 5826* (MO); Tianguistengo, Alrededores de Tianguistengo. Vegetación secundaria de ailes. Suelo casi negro, pedregoso, 26 Apr 1981, *Hernández Magaña, R. et al. 5875* (MO); 4km al E de Tlaxiaca, 24 Jun 1981, *Hernández Magaña, R. 6143* (MO); Tlaxiaca, 4 km al E de Tlaxiaca, 24 Jun 1981, *Hernández Magaña, R. 6143* (MEXU); Zacualtipán, Zoquizoquipan, 5 kms al norte de Zacualtipan, 21 Dec 1981, *Hernández Magaña, R. 6922* (MEXU); Pisaflores, 6 km al S de Pisaflores, 12 Feb 1982, *Hernández Magaña, R. & Tenorio L, P. 6971* (MEXU); Huehuetla, Acuautla, 7 km al W, 19 May 1982, *Hernández Magaña, R. 7280* (MEXU); Pachuca, 15 km al W de Pachuca, 5 May 1980, *Hernández, M.R. & Hernández V, R. 4365* (MEXU); La Misión, 2.1 miles S of El Chamal on Rte 85 (S of Sna Luis Potosi border), 25 Nov 1977, *La Duke, J. et al. 584* (F); Epazoyucan, 3 km al SE de Tezuantla, 15 Jun 1975, *Medina C, M. 422 a* (MEXU); Pachuca, 3 km al NW de Pachuca, 28 Jun 1975, *Medina C, M. 448* (MEXU); Real del Monte, 2 km WSW de Real del Monte, 4 Aug 1975, *Medina C, M. 594* (CORD, MEXU, MO); Epazoyucan, 3 km al SE de Tezuantla, 16 Sep 1976, *Medina C, M. 1610* (MEXU); Epazoyucan, 3 km al SE de Tezuantla, 16 Sep 1976, *Medina C, M. 1620 b* (MEXU); Pachuca, Barrio Las Peñitas, extremo NW de la cuidad de Pachuca, 20 Nov 1976, *Medina C, M. 1799* (MEXU); Epazoyucan, 1.5 km al NNW de Nopalillo, 14 May 1976, *Medina C, M. 1950* (MEXU); Actopan, El Arenal, Cerro de las Canteras, west of Pan American highway at Puerto San Pedro, km 104, 9 Oct 1946, *Moore, H.E. 1392* (BH); Parque Nacional El Chico, 19 Apr 1980, *Ortiz Calderón, A. 12* (MEXU); Shaded, west facing roadside slope Northeast of Jacala, ca 301 km from Mexico City, 21 Dec 1961, *Robinson, ?, s.n.* (MO); Cerro Grande, 2 km al S de Epazoyucan, 21 Mar 1972, *Rzedowski, J. 28958* (CORD, F, MEXU); Parada Jacuba [Ajacuba?], *Schmitz, A. 696* (W); Acaxochitlán, San Francisco Atotonilco, 30 Jul 1984, *Villa, A. 48* (MEXU). **Jalisco**: Ojuelos, Potrero San Carlos, 29 Sep 1983, *Alcocer R, M. 141* (MEXU); 5-10 km SW of Talpa de Allende on

road toward Tomatlán, 23 Sep 1983, *Anderson, W.R. 12770* (MEXU, MO); Playa Careyitos, ca. 7 km al SW de la Estación de Bioligía Chamela, km 53 de la carretera Barra de Navidad-Puerto Vallarta, 10 Sep 1985, *Ayala, M.G. 221* (MEXU); carretera Barra de Navidad-Puerto Vallarta, 21 Feb 1986, *Ayala, M.G. 652* (MEXU); Cuautitlán, Rancho La Pintada, ca. 3 km al NW de Cuzalapá, 12 May 1990, *Cárdenas, A.L. et al. 900* (MEXU); Perisferico 39.8 km from route 15, junction to Aeropuerto and Chapala. 0.4 km NE of Iteso (to 5) turn off. Elevation 5050 feet, 22 Jul 1977, *Davis, T. 760* (MO); Zapopan, Cerro El Colli, 1.5 km al SE de Ciudad Granja, 22 Aug 1993, *González, G. 19* (MEXU); Terrero, woods near football field, Reserva Biósfera Sierra de Manantlán, 15 Mar 1987, *Iltis, H.H. et al. 29602 a* (MEXU); Atoyac, Dec 1882, *Kerber, E. 177* (BM, CORD, G, PAL); Cuautitlán, 6.5-7.5 km al NW de Cuautitlán, 2 km al SE de Cuzalapa, 26 Jul 1989, *Niz, D. de*, *et al. 58* (MEXU); Ciudad Guzmán, km 9.5 hacia el Nevado de Colima, 15 Jun 1994, *Reynoso, J.J. 2203* (MEXU); Tuxcacuesco, 600 m al SE de Zenzontla, 12 Sep 1988, *Robles, L. 667* (MEXU); Zapopan, Las Agujas, Vivero de la Facultad de Agricultura, Nextipac, 25 Jul 1991, *Rodríguez C, A. & Vargas, O. 1095* (MEXU); Zapotlanejo, Puente Calderón, 30 Nov 2004, *Rodríguez C, A. & Castro-Castro, A. 4236* (MEXU); Tuxpan, 8 miles W of Atenquique, 1 Jun 1975, *Stevart, B.G. 30* (DUKE); Tlajomulco de Zúñiga, 10 km E of La Calera on Hwy 80, 10 Sep 1986, *Swagel, K. 122* (F); Lagos de Moreno, Lagos de Mareno, 17 miles NW of Lagos, 15 Aug 1957, *Waterfall, U.T. & Wallis, C.S. 13825* (F). **México**: Texcoco, Area Experimental Forestal Mario Avila Hernández, 2 km al oriente de San Luis Huexotla, 13 Sep 1985, *Asteinza, G. & Rey, J. s.n.* (MEXU); 20 miles E of Toluca, 10 Aug 1947, *Barkley, F.A. et al. 618* (F, MEXU); Texcoco, jardines de los nuevos dormitorios del Campus Universitaria de la Universidad Autonoma de Chapingo, 24 Jun 1991, *Bonilla Beas, R. & Monsalvo C, E. 99* (MEXU); Amecameca, 12 km al E de Amecameca, 26 Nov 1978, *Bonilla R, C. 16 2* (CORD, MO, US); Coacalco, S of Coacalco, east branch Arroyo Las Canosas, 1 May 2001, *Bye, R.A. 28166* (MEXU); Coatepec Harinas, cuenca del Río Tlapala, carretera San Luis, 28 Aug 2010, *Dorantes-Hernandez, F.D. et al. 257* (MEXU); Ixtapáluca, Estación Forestal Experimental Zoquiapán, Universidad Nacional Autonoma de Chapingo, 26 Aug 1994, *Estrada, E. s.n.* (MEXU); 1 km del entronque de el polvonin, a 13 km de Temascaltepec, carretera Temascaltepec-Toluca, 9 May 1988, *Flores-Franco, G. & Terpán A. L. 816* (MEXU); ca. 10 miles NE of Temascaltepec along road to Toluca, 6 Dec 1961, *Gentry, H.S. et al. 19601* (US); Temascaltepec, Los Hornos, 17 Mar 1932, *Hinton, G.B. 399* (BM); Temascaltepec, Ocotepec, 12 Oct 1932, *Hinton, G.B. 2917* (BM, F, K, NY, US); Temascaltepec, Comunidad, 5 Oct 1933, *Hinton, G.B. 3855* (BM, F, K, MEXU, NY, US); Temascaltepec, Mexico. México. Temascaltepec. Pungarancho, 28 Mar 1935, *Hinton, G.B. 7566* (NY, US); above Lake Texcoco and ca. 2 km E of tis edge, on road from Chalco to Amecameca, ca. 3 km W of Tlamanalco, 30 Sep 1980, *Iltis, H.H. et al. 3005* (MEXU); Atlautla de Victoria, Tepecoculco, 29 May 1987, *Jiménez Flores, J. 210* (MEXU); Valle de Bravo, Rancho Cerro Gordo. Avándaro, Cerro Gordo. Vereda de la Barranca Fresca (tramo entre la cascada y el camino principal), 20 Mar 2011, *Ledesma Corral, J.C. 1578* (MO); Villa de Allende, San Cayetano (estacón experimental) al N de Agua escondida y a 28 km NE de Valle de Bravo, 6 Jul 1974, *Maury Hernandez, M.E. et al. s.n.* (MEXU); [Volcán] Ixtaccíhuatl, Jan 1906, *Purpus, C.A. 1736* (F, MO, NY); Lerma, Jun 1952, *Ramírez, D. & Herrera, T. s.n.* (MEXU); Metepec, canal adyacente en al carretera Metepec-San Mateo Atenco, a 100 m de la desviación de a la Autopista Mexico-Toluca, 1 Aug 1994, *Ramos, V.L.J. 489* (MEXU); Coatepec Harinas, El Salto, San José del Progreso, 13 Nov 2010, *Rodríguez-Barquet, E. et al. 115* (MEXU); Huehuetoca, Cerro Sinoque, ladera E, 1 Nov 1980, *Romero-Rojas, ?, 778* (MEXU); 7 Km al W de Huixquilucan, 1971, *Rzedowski, J. 28345* (CORD, MEXU, MO, NY); Cerro Calavario, vertiente NE, 4 km al SE de Chalco, 2 Jan 1972, *Rzedowski, J. 28768* (F); Amecameca, San Antonio, 1 km al NE, 2 Oct 1977, *Rzedowski, J. 35329* (F, MEXU); Mun. Amecameca, Cerro Venacho, vertiente W, 1982, *Rzedowski, J. 38040* (MO); 1 Km al N de Citaltepec [Zitaltepec], municipio de Zumpango, 1980, *Sánchez S, E. 516* (MO); San Felipe del Progreso, Santa Ana Nichi, 23 Jan 1977, *Schwabe, W. s.n.* (MEXU); a 15 km al SO de Villa Victoria, sobre la carretera a El Oro, 7 Sep 1985, *Soto N, J.C. & Silva Ramírez, G. 10321* (MEXU); Ixtapaluca, Ixtapaluca. Estación Experimental de Investigación y Enseñanza de Zoquiapan. 8 km. al S. de Río Frio, 3 Jul 1978, *Vega A, R. 244* (MEXU, MO, NY); Cerro del Tipayo Jaltepec, municipio de Ajapusco [Axapusco], 1975, *Ventura A, A. 672* (CORD, MO); Texcoco, Santa Maria Tecuanulco, 23 Nov 1982, *Ventura V, E. 319* (CORD, MO); Texcoco, Santa Catarina, 17 Feb 1983, *Ventura V, E. 537* (CORD, F); Amecameca, Volcán Popocatepetl, circuito Tlamaca rumbo a Paso de Cortés, 24 Nov 1981, *Zúñiga B, G. 113* (MEXU).

Michoacán: près Morelia, 4 May 1909, *Arsène, G. s.n.* (K); Paricutin Volcano, 4 Jul 1950, *Eggler, W.A. 4* (MO); Morelia, Jacuaro, 23 Sep 1992, *Escobedo, J.M. 2466* (MEXU); Huetamo, Mich, 28 Feb 1934, *Hinton, G.B. 5709* (BM, K, MO, US); Coalcomán, Coalcomán, 14 Feb 1939, *Hinton, G.B. & Hinton, J.C. 12976* (MO, US); Zitácuaro, Cerro El Cacique, El Agua de la Difunta, 14 Oct 1979, *Ibarra C, G. 861* (F); Mil Cumbres, next to overlook, 15 Sep 1977, *Iltis, H.H. & Doebley, J.F. 55* (F, MEXU); Zinapécuaro, lado SE de la presa La Gapuchina, 18 Jan 1990, *Jasso, M.J. 1765* (MEXU); Angangueo, Chincua, zona nucleo, 28 Sep 2004, *Martínez Cruz, J. et al. 1368* (MEXU); Jungapeo, Río Tuxpan, abajo de Agua Blanca, 24 Apr 1982, *Martínez S, E.M. 345* (MEXU); Pátzcuaro, Muelle de Janitzio, 31 Mar 2011, *Morales Lopez, B. & Chavez Carbajal, M.A. 38* (MEXU); A 10 km al SE de Villa Madero, camino a Nocupétaro. En la orilla de un bosque de pino-encino, 30 Sep 1982, *Soto N, J.C. 4745* (MEXU, MO); a 4km al ne de Angangueo, camino a villa Victoria, 12 Aug 1984, *Soto N, J.C. et al. 6546* (MO); Coahuayana, a 17 al E de Palos Marías, 23 Dec 1984, *Soto N, J.C. et al. 7214* (F, MEXU); Villa Escalante, cerro a ca. 2 km al E de Rancho Cruz Gorda, 26 Nov 1970, *Takaki, F. S-190* (MEXU); Zitácuaro, Macho de Agua, 7 km, al E de Zitacuaro, Carr. Zitacuaro-Toluca. bosque de pino, alterado. Suelo amarillo forestal, 27 Aug 1982, *Tenorio L, P. et al. 1581* (MO); Morelia, al suroeste de la ciudad de Morelia, campus de la UNAM, *Toledo, M. & Flores Tolentino, M. 18* (MEXU). **Morelos**: Huitzilac, Parque Nacional Laguans de Zempoala, 11 Aug 1986, *Cardoso, V.L. 1233* (MEXU); Huitzilac, Rancho San Lorenzo, km 53.5 de la carretera federal Mexico-Acapulco (95), al SW del pobaldo Tres Marías, 7 Sep 1989, *Díaz V, I. & Noriega T, R. 1107* (MEXU); autopista Mexio-Cuernavaca km 53, 19 Jul 1989, *Espinosa, J. 164* (MEXU); autopista Mexico-Cuernavaca km 55, Nov 1959, *Espinosa, J. 248* (MEXU); Tepoztlán, 2-3 km al N de Tepoxtlán, sobre la cañada de la zona arqueologica del Tepozteco, 5 Oct 1986, *Flores-Franco, G. & Cabrera, E. 159* (MEXU); Jonacatepec, 2 km al S de Tlayca, 18 Oct 1986, *Flores-Franco, G. & Cabrera, E. 291* (MEXU); Cuernavaca, Cuernavava, Avenida Morelos, centro de la ciudad de Cuernavaca, 26 Aug 1987, *Fuchs Q, F.M. & Quezada R, M. 1745* (MEXU); 15 miles NE of Cuernavaca along toll road to Mexico, 4 Dec 1961, *Gentry, H.S. et al. 19596* (US); Tlalnepantla, limoite con estado Mexico, 15.5 km al NE desviación a Tlalnepantla, carretera Oaxtepec-Xochimilco, 400 m al al izquierda por una brecha, 21 Feb 1981, *González de L, D. 8* (MEXU, MO); km 56-57 on road from D.F. to Cuernavaca, 13 Oct 1940, *Moore, H.E. 127* (MEXU); Puente de Ixtla, Xoxocotla, 5 Oct 1985, *Ortiz, S.A. 135* (MEXU); Puente de Ixtla, Xoxocotla, terrenos de cultivo del Sr. Ricardo Alberto, 5 Oct 1985, *Ortiz, S.A. & Romero, M.M. 939* (MEXU); Jiutepec, Sur de Progreso, 22 Sep 1984, *Quezada, M. 451* (MEXU); Tepoztlán, Tepoztlán, 1926, *Redfield, R. 11* (US); Coatlán del Río, Coatlán del Río, 6 Oct 1975, *Torres, B. 97* (MEXU); Zempoala, 8 Oct 1972, *Vázquez, J. 3910* (MEXU); Old Rt. 95, 30 Km. S of Mexico City, 28 Jun 1966, *Windler, D.R. & Snider, J.A. 1025* (MO); Cuautla, Cuautla, al sur de vivero, 24 Jun 1985, *Without Collector 568* (MEXU). **Nayarit**: Santa María de Oro, Arroyo de Santa Fe, cerca de Paso de Golodrinas, Río Santiago, 4 Dec 1991, *Acevedo R, R. & Sosa L, J. 1105* (MEXU); San Pedro Lagunillas 5 Km al S del Poblado de San Pedro Lagunillas, en las faldas del Cerro Grande de San Pedro. Bosque de Pinus y Quercus, 25 Aug 1993, *Calzada, J.I. et al. 18759* (MEXU, MO); Along Route 28 between tepic and Jalcacatlan (Route 66 on some maps), between Km 14 and Km 15, 7 Jan 1979, *Croat, T.B. 45216* (MO); Tepic, 1 km al SW de El Cuarenteño camino a El Cora, o 4 km al N del entronque del camino El Cora-Palapitas, 16 May 1994, *Flores, G. et al. 3456* (MO); Tepic, 1 km al SW de El Cuarenteño camino a El Cora, o 4 km al N del entronque del camino El Cora-Palapitas, 16 May 1994, *Flores-Franco, G. et al. 3456* (MEXU); Tepic, 5 km al W del poblado La Yerba, en cañada, 18 Oct 1994, *Flores-Franco, G. et al. 4270* (MEXU); Volcán Ceboruco, 1-4km from microwave station, 21 Jun 1987, *Miller, J.S. & Téllez V, O. 3218* (BM, MO); Tepic, between Colomo and Arroyo Juan Sanchez, 6 Apr 1897, *Nelson, E.W. 4168* (F, US); Nayar, 28km del entronque a San Juan Peyotán, sobre la carr. Jesúa María-Huejuquilla; bosque de Pinus-Quercus, 3 Sep 1991, *Ramírez R, R. & Flores-Franco, G. 926* (MO); Tepic, Acaponeta, 25 Jun 1897, *Rose, J.N. 1461* (US); 6. km al NW de Xalisco en el camino a la Estación de Microondas Cerro Boludo. Riparia, secundaria, en arroyo seco, 30 Mar 1987, *Téllez V, O. 10114* (MEXU, MO). **Nuevo León**: Rio Sta Catarina, Jul 1911, *Arsène, G. 6149* (MO, US); island in Río Santa Catarina [Monterrey], 28 Oct 1944, *Barkley, F.A. et al. 44M 805* (US); Monterrey, Monterrey, Apr 1891, *Dodge, C.K. 24* (US); Vic. of horsetail Falls, El Cercado, 12 Jun 1961, *Duke, J.A. 3979* (MO); 21.2 mi north of Montemorelos, out of Margarita. Hwy 85 between Monterrey and Montemorelos, 9 Aug 1972, *Dziekanowski, C.T. et al. 1716* (MO); Iturbide, below San Pedro Iturbide,

I.R.F. Itrubide, 25 Sep 1979, *Hinton, G.B. 17607* (MEXU); Iturbide, near San Pedro Iturbide, 25 Sep 1979, *Hinton, G.B. 17697* (MEXU); Cañon Huajuco, east of Villa Santiago, in the valley of Río San Juan, 26 Feb 1946, *Johnson, J.C. & Barkley, F.A. 16 /38M* (F); Cerro Potosi, 9 Jul 1963, *McGregor, R.L. et al. 399* (US); Diente Canyon, mountains near Monterrey, Jul 1933, *Mueller, C.H. & Mueller, M.T. 133* (F); Monterrey, Monterry, Feb 1880, *Palmer, E. 934* (K, NY, US); Monterrey, near Las Mitras [Cerro de las Mitras], 4 miles from Monterrey, Aug 1946, *Roybal, J.J. 621* (US); near Monterrey, 4 mi SE of La Pastora, 19 Jun 1946, *Roybal, J.J. 1055* (MEXU); Galeana, Hacienda Pablillo, 10 Aug 1936, *Taylor, M. 126* (F, K, W); Montemorelos, Ojo de Agua, 28 Mar 1988, *Tirado, M. 162* (MEXU); Montemorelos, Ojo de Agua, 5 Apr 1988, *Tirado, N. 42* (MEXU); Montemorelos, Ojo de Agua, 15 Apr 1988, *Tirado, N. 107* (MEXU); Montemorelos, Ojo de Agua, 27 Mar 1988, *Tirado, N. 138* (MEXU). **Oaxaca**: San Juan Juquila Vijanos, alrededores de la Reforma, Dto. Villa Alta; Sierra Norte, 12 Jun 1996, *Aguilar Santelises, R. 650* (MEXU); Cerro de la Minas [label illegible], 20 Aug 1903, *Altamirano, F. 1593* (MEXU); Lázaro Cárdenas, 1 km después de la desviación hacia Yucunifoco viniendo de Putla, Dist. Juxtlahuaca, 27 Sep 2008, *Ayala-Hernández, ?, 576* (MEXU); San Lucas Ojitlán, poblado de Vista Hermosa, 21 Jan 1989, *Calzada, J.I. 14221* (MEXU); Santa María Jacatepec, a 4 km del poblado La Joya, carretera a Macederia, parcela de Ramon Garcia, 18 Jun 1990, *Calzada, J.I. 15461* (MEXU); Santa María Jacatepec, a 4 km del pobaldo La Joya, carretera a Macedonia, parcela de Ramón Garcia, 18 Jun 1990, *Calzada, J.I. 15461* (MEXU); San Juan La Lana, al NE de Santiago Jalahui, parcela de Vicente Palomaque, 2 Dec 1990, *Calzada, J.I. et al. 16748* (MEXU); Santa María Ecatepec, Yautepec, 100 m al S de Santa María Ecatepec, 18 Feb 1993, *Camacho, J.P. 20* (MEXU); along the Oaxaca-Tuxtepec road, highway 175 between km 130 and 140 beyond Cerro Pelon, 3 Jan 1974, *Carlson, M. 4134* (F); San Pedro Pochutla, Finca La Concordia, 15 km al N de Chacalapa, 26 Apr 1983, *Cedillo T, R. & Torres R, R. 2291* (MEXU); Roadside at Km 218 of Hwy. 175 at La Cumbre ca. 30 air km NE of Oaxaca, 25 Aug 1974, *Conrad, J. & Conrad, R. 3100* (F, MO); Roadside at Km 218 of Hwy. 175 at La Cumbre ca 30 air km N.E, 25 Aug 1974, *Conrad, J. & Conrad, R. 3113* (F, MO); Quercus-Pinus forest on 40-70 degree S-facing slope, 5 km S. of El Punto on Hwy. 175, ca. 30 air km N.E. of Oaxaca, 26 Aug 1974, *Conrad, J. & Conrad, R. 3133* (MO); Km 160 near Ixtlan on Hwy. 175 ca. 40 air km NE of Oaxaca, 28 Aug 1974, *Conrad, J. & Conrad, R. 3179* (MO); San Pedro Pochutla, [San Rafael Toltepec], 24 Apr 1891, *Conzatti, C. 3178* (MEXU); road between Natividad and Talea, 17 km from Natividad, 30 Jun 1983, *Costich, D.E. & Baldwin, D. 1507* (F); Putla, Along Highway 125 between Pinotepa and Tlaxiaco, 4.4 km S of Putla de Guerrero, 17 Jan 1979, *Croat, T.B. 45845* (MEXU, MO); [Mun. San Miguel Suchixtepec]. Along Highway 175 between Oaxaca and Pochutla, 35.8 mi S of Miahuatlán, 2.5 mi S of Suchixtepec, 19 Jan 1979, *Croat, T.B. 46019* (MEXU, MO); Along road from Valle National to Arriba Tortuga and Armadillo via Rancho Grande and Loma San Rafael 5-6 km NW of Valle Nacional Center (Hotel de la Valle), 2 Mar 2008, *Croat, T.B. 100097* (MO); San Andrés Teotilalpam, Santa Cruz Teotilalpam, Paraje Agua Tuza a 1 km al SW de Santa Cruz Teotililpam, 24 Nov 2004, *Cruz Espinosa, C.A. 1864* (MEXU); 22.3 km on road past Diaz Ordaz from junction route 190, 31 Jul 1977, *Davis, T. 800* (MEXU, MO); sin. loc, 28 Jun 1900, *Dean, C.C. 14* (US); entre Llano de las Flores y Tuxtepec, 25 Dec 1965, *Delgadillo, C. 96* (MEXU); Santiago Astata, Barra de la Cruz, 1 km SE por río, Dist. Tehuantepec, 20 Mar 1998, *Elorsa C, M. 27* (MEXU); Santiago Astata, Barra de la Cruz, 1 km SE por río, Dist. Tehuantepec, 31 Jul 1998, *Elorsa C, M. 497* (MEXU); Santiago Astata, Barra de la Cruz, 1 km al NE, al pie del cerro, Dist. Tehuantepec, 31 Oct 1998, *Elorsa C, M. 1015* (MEXU); Santiago Astata, Barra de la Cruz, 1 km SSE por el río, 18 Dec 1998, *Elorsa C, M. 1322* (MEXU); Santiago Astata, Barra de la Cruz, 1 km SSE por el río, 11 Feb 1999, *Elorsa C, M. & Scheidegger, D. 1729* (MEXU); Santiago Astata, Barra de la Cruz, 1 km SSE por el río, Dist. Tehuantepec, 11 Feb 1999, *Elorsa C, M. & Scheidegger, D. 1732* (MEXU); Tehuantepec, Mpio. Santiago Astata.Barra de la Cruz, 0.5 km N por el río, 16 Mar 1999, *Elorsa C, M. 1908* (MEXU, MO); Tehuantepec, Mpio. Santiago Astata. Barra de la Cruz, 0.5 km SE, 6 Apr 1999, *Elorsa C, M. 1995* (MEXU, MO); Santiago Astata, Barra de la Cruz, 1.5 km SE, laguna de cerrito, Dist. Tehuantepec, 28 May 1999, *Elorsa C, M. 2141* (MEXU); Santiago Astata, El Zapotal, 1 km E de Barra de la Cruz, Dist. Tehuantepec, 3 Feb 2000, *Elorsa C, M. 2685* (MEXU); Santiago Astata, El Zapotal, 2 km al SE de Barra de la Cruz, Dist. Tehuantepec, 7 Apr 2000, *Elorsa C, M. 2922* (MEXU); Santiago Astata, Barra de la Cruz, 1 km al S, Dist. Tehuantepec, 12 Jun 2000, *Elorsa C, M. 3093* (MEXU); Santiago Astata, Barra de la Cruz, 1 km al S, Dist. Tehuantepec, 24 Jul 2000, *Elorsa C, M.*

3307 (MEXU); Santiago Astata, 1 km al sureste de Barra de la Cruz, Dist. Tehuantepec, 29 Dec 2000, *Elorsa C, M. 4110* (MEXU); Santiago Astata, Barra de la Cruz, 1 km al SE, Dist. Tehuantepec, 5 Jan 2001, *Elorsa C, M. 4209* (MEXU); Santiago Astata, El Zapotal, 2 km al SE de Barra de la Cruz, Dist. Tehuantepec, 5 Mar 2001, *Elorsa C, M. 4343* (MEXU); Santiago Astata, Barra de la Cruz, 1 km al SE colinando con el Zapotal, Dist. Tehuantepec, 8 May 2001, *Elorsa C, M. 4549* (MEXU); San Carlos Yautepec, 500 m al S de San Miguel Chonga, Dist. Yautepec, 21 Jun 2001, *Elorsa C, M. 4724* (MEXU); Santiago Astata, 1 km al sureste de Barra de la Cruz, sobre el camino al campamento tortuguero, Dist. Tehuantepec, 14 Jul 2001, *Elorsa C, M. 4828* (MEXU); San Miguel del Puerto, Cascadas de Copalitilla, 9 km al oeste de Sta. Maria Xadani, Dist. Pochutla, 24 Jul 2001, *Elorsa C, M. 4921* (MEXU); Santiago Astata, 1 km al sureste de Barra de la Cruz, Dist. Tehuantepec, 14 Sep 2001, *Elorsa C, M. 5171* (MEXU); Santiago Astata, 1 km al N de Barra de la Cruz, Dist. Tehuantepec, 29 Apr 2002, *Elorsa C, M. 6023* (MEXU); Ixtlán de Juárez, Camino Real de Tiltepec a Tierra Caliente, 28 Nov 1999, *Flores V, J.C. et al. 51* (MEXU); Santo Domingo Petapa, Santo Domingo Petapa, 18 Sep 1992, *Frei, B. & Heinrich, M. 103* (MEXU); sin. loc, 1840, *Galeotti, H.G. 1229 [2]* (W); "Ruipiceau a Terr.", 1840, *Galeotti, H.G. 1229 [c]* (P); Aux bords des ruisseuaux de la cordillera de Yavezia, a 7, 000 pieds, Apr 1840, *Galeotti, H.G. 1238* (BR, P, W); Distrito de Ixtlan: Municipio de Santiago Comaltepec: Puerto Eligio bosque mesofilo de montaña, 19 Nov 1987, *García, ?, 51* (MO); Ixtlan, Santiago Comaltepec, 3 Nov 1984, *Gómez N, C. 112* (MEXU); Miahuatlan, Mpio. San Juan Mixtepec; in town, 22 Dec 1996, *Hunn, E. OAX-668* (MEXU, MO); San Juan Mixtepec, San Juan Mixtepec, Dist. Miahuatlán, 8 Feb 1997, *Hunn, E. OAX-838* (MEXU); San Juan Mixtepec, San Juan Mixtepec, near town, Dist. Miahuatlán, 20 Apr 1997, *Hunn, E. OAX-1056* (MEXU); San Juan Mixtepec, San Juan Mixtepec, 2 km SSW near C. Crux family milpa. Dist. Miahuatlán, 8 Jul 1997, *Hunn, E. OAX-1383* (MEXU); San Juan Mixtepec, Dist. Miahuatlán, 10 Aug 1998, *Hunn, E. 1849* (MEXU); along the Pan-American highway (routes 185 & 190) one-half kilometer east of the city of Tehuantepec, 30 Jun 1958, *King, R.M. 330* (MEXU); Río Soyolape gorge, Hwy. 175, 4 Aug 1975, *Lawton, R.O. 794* (F); km 127.9, Hwy 175, 22 Aug 1975, *Lawton, R.O. 830* (F); Tuxtepec, on hwy. 175 from 43.6 mi from 190 & 175, *LeDoux, D.G. et al. 2271* (MO); Comaltepec, Puerto Eligio, Dist. Ixtlán, 19 Nov 1987, *López García, E. & Martin, G.J. 51* (MEXU); Ixtlan, Municipio de Comaltepec: S. Comaltepec. Bosque de coniferas y de encino, 4 Aug 1988, *López L, L. & Martin, G.J. 166* (MO); Santiago Comaltepec, Comaltepec, Dist. Ixtlán, 4 Aug 1988, *López López, L. 166* (MEXU); Comaltepec, Comaltepec, Dist. Ixtlán, 1 Sep 1989, *López López, L. 377* (MEXU); Santiago Comaltepec, Comaltepec, Dist. Ixtlán, 28 Jun 1990, *López López, L. 500* (MEXU); Ixtlan, santiago comaltepec; La Esperanza bosque mesófilo de montala, 27 Sep 1987, *López Luna, R. 31* (MEXU, MO); Mixe, santiago tepitongo.bosque mesofila de montaña, 8 Apr 1987, *López, ?, 24* (MO); Mixe, Totontepec: Santiago Tepitongo, bosque mesófilo de montaña, 5 Feb 1988, *López, ?, 190* (MO); San Miguel del Puerto, Panteón, Dist. Pochutla, 16 May 2000, *López, F. 160* (MEXU); Ixtlan, Llano de las Flores, ruta 175 Tuxtepec a Oaxaca, Sierra de Juárez, 5 Aug 1981, *Lorence, D.H. et al. 3658* (F, MO); San Miguel Amatlán, La Nopalera, Dist Ixtlán, *Luna Castellanos, F. 151* (MEXU); Pochutla, Concordia, 26 Feb 1937, *Makrinius, E. 773* (US); Santiago Laxopa, Santiago Laxopa, 6 Dec 1986, *Maldonado Vasquez, N. 95* (MEXU); mercado de Tlahuitoltepec, *Martin, G.J. 28* (MO); Miahuatlan, San Andres Paxtlán, 15 Jun 1987, *Martin, G.J. M-51* (MEXU, MO); San José Chiltepec, Chiltepec, 2 Nov 1941, *Martínez Calderón, G. 778* (MEXU); Tehuantepec, Hierba Santa, 12 km al O de Tehuantepec, 22 Apr 1987, *Martínez R, C. 906* (MEXU); Ixtepeji, Cañada del Estudiante, Dist. Ixtlán, Jul 2001, *Martínez, S. 260* (MEXU); San José Chiltepec, district of Tuxtepec, Chiltepec and vicinty, Jul 1940, *Martínez-Calderón, G. 172* (US); San Miguel Chimalapa, Río Escondido, (Arroyo Baul) 0.8 km al O de la union con el Río Portamonedas y de la Congregación Benito Juárez, ca. 38 km en linea recta al N de San Pedro Tapanatepec, 17 Aug 1984, *Maya J, S. 453* (MEXU); Sn. Miguel Chimalapa: Congregación Benito Juárez, valle del Río Portamonedas, ca. 38 km. en línea recta al N. de San Pedro Tapanatepec. Lugares perturbados cerca del pueblo, 24 Aug 1984, *Maya J, S. 496* (MEXU, MO); San Miguel Chimalapa, Congrecación Benito Juárez, valle del Río Portamonedas, ca. 38 km en linea recta al N de San Pedro Tapanatepec, 8 Oct 1984, *Maya J, S. 672* (MEXU, MO); Ayutla, Distrito Mixe, Ayutla, 17 Dec 1985, *Nee, M. 32196* (BM, MO); Choapam, west slope of Mount Zempoaltepec, 5 Jul 1894, *Nelson, E.W. 578* (US); Totontepec, vicinity of Totontepec, 15 Jul 1894, *Nelson, E.W. 725* (US); Cuicatlán, Cuicatlán, vicinity of Cuicatlan, 8 Oct 1894, *Nelson, E.W. 1679* (US); Santa Marían Huatulco, Arroyo Arena, Dist. Pochutla, 20 Feb 2001, *Ortega Lavariega, R. 443* (MEXU); Asunción

Ixtaltepec, a 1.4 km linea recta al SO (192°) de Nizanda, 5 Feb 1996, *Pérez-Garcia, E. & Reyes Dias, B. 991* (MEXU); Sierra de San Felipe, 6 Oct 1894, *Pringle, C.G. 4948* (BM, BR, E, GOET, KFTA, MO, NY, PH, US, W, Z); Río Magdalena, 20 Apr 1917, *Reko, B.B. 3173* (US); San Juan Mixtepec, Caba Yáa, (Peña de Aguilas), Dist. Juxtlahuaca, 3 Sep 1989, *Reyes S, J. 1924 a* (MEXU); Municipio de Totontepec. ca. Bosque mesofilo de montana, 22 Feb 1988, *Reyes, J.R. & Martin, G.J. 956* (MO); Santa María Chimalapa, San Antonio Nuevo Pariaso, a 3 km al W, Plan de la Ceiba, Dist. Juchitán, 21 Sep 1997, *Rivera H, J. et al. 492* (MEXU); Santa María Chimalapa, San Antonio Nuevo Paraiso, en los alrededores, 11 May 1998, *Rivera H, J. et al. 661* (MEXU); San Miguel del Puerto, Finca El Faro, 1 km al S por la brecha hacia Fina Montecario, Dist. Pochutla, 19 May 2000, *Rivera H, J. 2412* (MEXU); totontepec; bosque mesofilo, 16 Apr 1986, *Rivera Reyes, J. 219* (MO); Totontepec, Totontepec, Dist. Mixe, 22 Feb 1988, *Rivera Reyes, J. & Martin. G.J. 956* (MEXU); Mixe, Municipio de Totontepec: Totontepec. Bosque mesofilo de montana, 7 Aug 1988, *Rivera Reyes, J. & Martin, G.J. 1114* (MO); Totontepec, bosque mesofilo, 2 Dec 1989, *Rivera Reyes, J. 1298* (MEXU, MO); Cerro Pelón, 47 km al N de Ixtlán de Juárez, sobre la carretera a Tuxtepec, 23 Oct 1977, *Rzedowski, J. 35441* (F); San Miguel del Puerto, La Hamaca, Copalita, 1 km al W sobre la terraceria a Santa Maria Huatulco, Dist. Pochutla, 27 Jul 2000, *Salas M, S. 5044* (MEXU); Santiago Textitlán, Paraje Cruz de Hielo, 17 May 2006, *Salas M, S. et al. 5678* (MEXU); Pluma Hidalgo, Santa María Magdalena, 0.5 km brecha a un lado de cemeterio, Dist. Pochutla, 23 Feb 1999, *Salas M, S.H. et al. 1943* (MEXU); Pochutla, Mun: San Miguel del Puerto. La Hamaca, Copalita, 1 km al W sobre la terraceria a Sta. María Huatulco. Selva mediana subperennifolia. Secundaria, 27 Jul 2000, *Salas M, S.H. 3044* (MO); San Pedro Huamelula, Puente Zimatán, 500 m al E, sobre la carretera Huatulco-Salina Cruz, Dist. Tehuantepec, 2 Nov 2000, *Salas M, S.H. et al. 3574* (MEXU); San Carlos Yautepec, 6.6 kmm al norte de la desviación a Guadelupe Victoria, Dist. Yautepec, 25 Aug 2001, *Salas M, S.H. et al. 3970* (MEXU); Santiago Textitlán, Paraje El Berro, Dist. Sola de Vega, 27 Jun 2006, *Salinas, M.E.J. 74* (MEXU); Santiago Textitlán, Cara de León, rumbo a Recibimiento, Dist. Sola de Vega, 23 Aug 2006, *Salinas, M.E.J. 331* (MEXU); Santiago Textitlán, Paraje Toma de Agua, rumbo a pueblo viejo, Dist. Sola de Vega, 31 Aug 2006, *Salinas, M.E.J. 502* (MEXU); Santiago Textitlán, cerca de Piedra Azul, Dist. Sola de Vaga, 22 Jan 2007, *Salinas, M.E.J. 1466* (MEXU); Santiago Textitlán, La Yerba Santa, dist. Sola de Vega, 19 Feb 2007, *Salinas, M.E.J. 1598* (MEXU); Santiago Textitlán, arriba de Barranca Nube, Dist. Sola de Vega, 28 Feb 2007, *Salinas, M.E.J. 1787* (MEXU); Santiago Textitlán, abajo de Río Platanar, Dist. Sola de Vega, 15 Mar 2007, *Salinas, M.E.J. 1865* (MEXU); Juchitán de Zaragoza, La Ventosa, Istmo de Tehuantepec, 7 Feb 2014, *Sánchez L, F. & Morales C, G. 529* (US); San Miguel del Puerto, Finca El Faro, lado norte, 22 Aug 2001, *Saynes V, A. et al. 2503* (MEXU); San Juán Bautista Valle Nacion, Valle Nacional [San Juán Bautista Valle Nacional] por la carretera, 24 Feb 1976, *Shapiro, G. 389* (MEXU); Sierra de Clavellinas, 18 Oct 1894, *Smith, C.L. 697 [b]* (F); San Pedro Pochutla, Finca Cafétalera Dolores, entrada 5 km de Chacalapa, carretera Pochutla-Oaxaca, Dist. Pochutla, 18 Mar 1983, *Tenorio L, P. & Torres C, R. 3562* (MEXU); Santa María Chimalapa, Chihuahua, 16.5 km al NE de Santiago Ixtaltepec, brecha a Santa Maria Chimalapa, 17 Jan 1984, *Tenorio L, P. & Torres C, R. 5205* (MEXU); Matias Romero, El Jobo, 11 km aW de Palomares carretera a Playa Vicente, 18 Jan 1984, *Tenorio L, P. & Torres C, R. 5341* (MEXU); Teotitlán de Flores, Magón, 3 km al SW del Puerto de la Soledad, brecha a Capultitla, Dist. Teotitlán de Flores, 21 Dec 2000, *Tenorio L, P. 19869* (MEXU); Villa Díaz Ordáz, a 18.4 km al N de Díaz Ordáz, por la desviación a Cuajimoloyas, 14 May 1983, *Torres C, R. et al. 2849* (MEXU); Juchitan, 16.5 km al NE de Santiago Ixtaltepec, hacia Sta. Maria Chimalapa. Veg. Secundaria de ecotonia Encinar-selva alta perennifolia. Suelo arcilloso, 17 Jan 1984, *Torres C, R. & Tenorio L, P. 4442* (MEXU, MO); Matías Romero Avendaño, El Jobo, 11 km W de Palomares carretera a Playa Vicente, 18 Jan 1984, *Torres C, R. & Tenorio L, P. 4477* (MEXU); 7 km al N de la desv. A San andres Yaa, la cuál esta a 4 km al NE de San Miguel Metepec. Bosque mesófilo con Quercus, Litsea, Clethra, Saurauia, Magnolia y Carpinus, 22 May 1984, *Torres C, R. & Villaseñor, J.L. 5111* (MO); 4 Km al N del campamento Río Molino, entre sn. José del Pacífico y Suchixtepec. bosque de pino-encino, 13 Jun 1985, *Torres C, R. & García Mendoza, A.J. 6816* (MEXU, MO); Tehuantepec, Arroyo Las Minas, entrando por El Limón, la entrada es por el camino Hierba Santa-Buenos Aires, Dist. Tehuantepec, 22 May 1986, *Torres C, R. & Martínez, C. 8329* (MEXU); Soyaltepec, Cerro Verde, al SO de Temascal, subiendo por La Torre, a La Antigua Caseta de Radio, camino al Vertedor, 20 Oct 1987, *Torres C, R. & Cortes, L. 10132* (MEXU); Soyaltepec, Vertedor de la Presa Miguel Alemán, al SO de Temascal, Dist.

Tuxtepec, 22 Oct 1987, *Torres C, R. & Cortes, L. 10159* (MEXU); Mixe, 14 Km al N de Yacochi, camino a Totontepec, Mpio Totontepec. Bosque mesófilo, 26 Oct 1987, *Torres C, R. & Cortés A, L. 10358* (MO); Santa María Jacatepec, bajada del predio El Aguila a San Agustín, entrando por La Reforma, 28 km al SO de Tuxtepec, carretera a Matías Romero, Dist. Tuxtepec, 19 Jan 1988, *Torres C, R. & Martínez S, E.M. 11051* (MEXU, MO); Talcolula, 13.8 km al N de Diaz Ordaz, camino a Cuajimoloyas, Dist. Talcolula, 16 Sep 1988, *Torres C, R. & Martinez R, C. 12398* (F); Tlacolula, a 18.4 km al N de Díaz Ordáz, por la desviación a Cuajimoloyas. Bosque de pino-encino, 14 May 1983, *Torres R, R. et al. 2849* (MO); Talcolula Dto. 13.8 km al N de Diaz Ordáz camino al Cuajimoloyas. Bosque de pino-encino, 16 Sep 1988, *Torres R, R. & Martínez R, C. 12398* (MO); Pochutla, 15 km al N de Chacalapa, en Finca la Concordia, 26 Apr 1983, *Trigos, R.C. & Torres C, R. 2291* (MO); Santiago Textitlán, Paraje Llano Chicle, 23 Aug 2006, *Trujilio Olazo, I. 298* (MEXU); Santiago Textitlán, Pueblo Viejo, Dist. Sola de Vega, 28 Jul 2006, *Trujillo Olazo, I. 166* (MEXU); Santiago Textitlán, paraje desviación de Recebimiento y Lachixao, Dist. Sola la Vega, 5 Feb 2007, *Trujillo Olazo, I. 1518* (MEXU); Santiago Textitlán, Paraje El Naranjo, Dist. Sola de Vega, 21 Feb 2007, *Trujillo Olazo, I. 1681* (MEXU); Santiago Textitlán, Río Platanar, 29 Mar 2007, *Trujillo Olazo, I. 1929* (MEXU); Santiago Textitlán, Barranco del Llano Lumbre, Dist. Sola de Vega, 15 Sep 2006, *Trujillo Vásquez, R. 491* (MEXU); Santiago Textitlán, abajo de Temascal, Dist. Sola de Vega, 10 Sep 2006, *Trujillo Vazquez, R. 422* (MEXU); Totontepec, Villa de Morelos, Chinantequilla, Dist. Mixe, 12 Dec 1989, *Vargas Ruiz, E. 502* (MEXU); Ixtlan, Municipio de Santiago Laxopa; S. Laxopa, 6 Dec 1986, *Vasquez M, N. 95* (MO); Totontepec, Villa de Morelos, Tepitongo, Dist. Mixe, 16 Feb 1990, *Velasco López, E. ES 0385* (MEXU); Totontepec, Tepitongo, Dist. Mixe, 16 Feb 1990, *Velasco López, E. & Martin, G.J. 386* (MEXU); San Miguel Chimalapa, Cerro Baúl, filo S. un poco al S. de la cima, ca. 23 Km. en Linea recta al N de SanPedro tapanatepec; bosque mesófilo (quemado hace 1 año) con Cupressus. Oreopanax. etc. 10-12 m. de alto. con muchas epifitas, filo casi plano muy pedregoso, 18 Jul 1985, *Wendt, T. et al. 4953* (MO, NY); Sola de Vega, Municipio: Santiago Textitlán. Arriba de las Juntas, 29 Sep 2006, *Zarate M, A. 589* (MO); Santiago Textitlán, Rancho Alegre, Dist. Sola de Vega, 26 Aug 2006, *Zarate Marcos, A. 318* (MEXU); Santiago Textitlán, Río Aguacate, Dist. Sola de Vega, 22 Sep 2006, *Zarate Marcos, A. 546* (MEXU); Santiago Textitlán, abajo de Llano Lumbre, Dist. Sola de Vega, 15 Sep 2006, *Zarate Marcos, A. 630* (MEXU). **Puebla**: Zongozotla, Zongozotla, Los Tanques, 2.5 km al S del Municipio, 20 Oct 2015, *Amith, J.D. et al. 21364* (US); Zautla, Rosa del Castillo, lugar llamado Chechelopan, se toma la carretera rumbo a Zayolapan, la entrada está a 3 km del centro del pueblo, 26 Jan 2015, *Amith, J.D. et al. 30504* (BM); Xochiapulco, Rosa Chica, sitio llamada La Loma, 300 m abajo de la clinica, 23 Mar 2015, *Amith, J.D. et al. 30588* (BM); Rancho Posada, vicinity of Puebla, 14 Feb 1909, *Arsène, G. 10* (US); Puebla, source au-dessous Finca Guadelupe, 20 Nov 1906, *Arsène, G. 71* (US); Hacienda Alamos, vicinity of Puebla, 6 Dec 1906, *Arsène, G. 126* (US); Jardin, vicinity of Puebla, 30 Aug 1906, *Arsène, G. 434* (US); Puebla, Barranca Alsesaca près Hda. Sta. Barbara. [Santiago Alseseca], 18 Jul 1907, *Arsène, G. 1031* (MO, US); Vicinity of Puebla, State of Puebla. Cerro and Fort Guadalupe, 3 Oct 1907, *Arsène, G. 1190* (MO, US); Puebla, pres du cimetiere, 23 Jun 1907, *Arsène, G. 2235* (US); Puebla, en cimitiere, 23 Jun 1907, *Arsène, G. 2238* (MEXU); vicinity of Puebla, Jardin, 30 Aug 1906, *Arsène, G. 10127* (US); camino al bosque Ajuigilre [illegible, could be Aguillila?], May 1959, *Bravo H, H. 236* (MEXU); Amozoc, 30 Km. de la carretera 150. Suelo arenoso, 12 Aug 1979, *Bretting, P.K. M 3* (MO); Huauchinango, Mercado. Procedencia: Local, 1 Sep 2012, *Bye, R.A. et al. 176* (MO); Pahuatlán, Pahuatlán, S del pueblo en cafetales, 23 Jul 1988, *Bye, R.A. & Linares, E. 16387* (MEXU); Piedras Negras, El Salto, 9 km al NE de La Ceiba, 25 Feb 1987, *Campos V, A. et al. 58* (MEXU); Zacatlán, Mex Hwy 119 (N side at km 76) 4.3 km by road NW of Zacatlán on road to Ahuazaotepec, 3 Jul 1978, *Cochrane, T.S. et al. 8481* (F, MEXU); Aljojuca, Mina de Arena, al E de Aljojuca, 2 Dec 2004, *Contreras J, J.L. 8242* (MEXU); Aljojuca, cerro al NO de San Miguel Tecuitlapa, 16 Jun 2005, *Contreras J, J.L. 8324* (MEXU); Juan Galindo, al E de la Hidroeletrica Tepexi, siguiendo el curso del río, 31 Aug 2007, *Contreras J, J.L. 8915* (MEXU); Hueytamalco, Campo Experimental "Las Margaritas", Instituto Nacional de Invstigaciones Forestales, Agricolas y Pecuarias (INIFAP), 17 Apr 2008, *Cornejo Tenorio, G. & Ibarra Manríquez, G. 2678* (MEXU, MO); Izúcar de Matamoros, 50 km NW of Puebla along route 190, 18 Feb 1976, *Croat, T.B. 32726* (MO); Xochitlán de Vicente Suarez, Texcalapa, 26 Mar 2002, *Cruz Rivas, A. 130 ac* (MEXU); Oriental, 16 Oct 1945, *Hernández X, E. X-30* (MEXU); Zautla, Rosa de Castilla, en el lugar llamado Checheloapan, camino a Jilotepec, 26 Sep

2014, *Jiménez Chimil, M. JDA-30238* (BM); Zautla, Rosa de Castilla, en un lugar llamado Takes, por la carretera rumbo a Chilapa, en la parte suroeste del pueblo, a 1 km de la iglesia, 14 Dec 2014, *Jiménez Chimil, M. & Gorostiza Salazar, M. JDA-30419* (BM); Zacapoaxtla, San Juan Tahitic, Calle Ramonco, a 250 m de la base de micros, en la localidad de Atawohteno, 20 Sep 2014, *Ledesma, C. & Sotero Hernández, A. JDA-20153* (BM); Chinantla, Chinantla, May 1841, *Liebmann, F.M. s.n.* (US); outsdie the CIMMYT compound opposite hte estern cistern, alogn an unpaved road off of Mexico Highway 130, 10 Apr 1987, *Lievens, A.W. & Gregory, B.M. 2255* (MEXU); Puebla, caminoa Matacaba por lado este de San Miguel Canoh, 16 Jul 1987, *Macias Acevedo*, *13* (MEXU); Huauchinango, El Paraiso, 8 km al N de Huauchinango, 1 Apr 1973, *Moreno G, S. 86* (F); Chalchicomula, near Chalchicomule [Chalchicomula], 15 Mar 1894, *Nelson, E.W. 249* (US); Río Cazones, 18 Nov 1950, *Ramírez, D. 236* (MEXU); Villa Juárez, "El Paraiso", 12 Apr 1962, *Sarukhan, J. et al. s.n.* (MEXU); Villa Juárez, km 8 de la cerrera que baja a la Mina "El Paraiso", 22 Apr 1962, *Sarukhan, J. et al. 1183* (MEXU); San Martín Texmelucan, San Cristobal Tepatlaxco, 5 km al NE de San Martin Texmelúcan, 30 Oct 1988, *Solís M, A. 4904* (MEXU); Texmelucán, San Cristóbal Tepatlaxco, camino San Martin Tex.-El Verde, 21 Aug 1988, *Solís, A. 4874* (MEXU); Barranca de los Membrilloa. Mpio. Caltepec. veg. Matorral Subtropical: Lysiloma divaricata: L. acapulcense: Bursera copallifera: Ipomoea murucoides; Pistacia mexicana: Prosopis sp.; Erythrina sp. Suelo pedregoso rojizo, 21 Oct 1983, *Tenorio L, P. & Romero de T, C. 4806* (MO); Nicolas Bravo, Rancho Cabras, 4 km al E [W] de San Bernardino Lagunas [E on label clearly incorrect], 13 Jan 1984, *Tenorio L, P. et al. 5120* (US); Honey, Arroyo Grande, 7 km al SE de Chila, 23 Feb 1987, *Tenorio L, P. et al. 12506* (MEXU); Piedras Negras, El Salto, 9 km al NE de La Ceiba, 25 Feb 1987, *Tenorio L, P. et al. 12638* (MEXU); Cholula, camino a Santiago Xalitzintla entrada por el lado oriente, 10 Aug 1987, *Tlapa A, M. & Ubierna, G. 80* (MEXU); Cholula, Cañada Grande, por el lado noreste de San Pedro Yancuictlalpan, 17 Aug 1987, *Tlapa A, M. & Ubierna, G. 162* (MEXU); Cholula, camino a San Nicolas de los Ranchos, por el lado oriente, 21 Aug 1987, *Tlapa A, M. & Ubierna, G. 232* (MEXU); 5 km al E de Río Frio, sobre la super-carretera a Puebla, 5 Jul 1970, *Weber, R. 450* (F); Cuetzalan, carretera Cuetzalan-San Andresito, 4 Mar 1976, *Zola B, M.G. 280* (MEXU). **Querétaro**: Fracc. Los Claustros, 26 Apr 1975, *Argüelles, E. 22* (MEXU); camino entre Huimilpan y Galindo, atrevasando la montaña, 15 Jun 1986, *Argüelles, E. 2520* (MEXU); sin. loc, 1911, *Arsène, G. & Agniel (Bro) 10339* (US); Arroyo Seco, Río Atarjea, 4-6 km al SW de El Limón, 23 Jun 1991, *Carranza, E. 3209* (MEXU); Landa, San Onofre, ca. 2 km al W, 11 Feb 1992, *Carranza, E. 3880* (F); Colón, 1.5 km del Ejido Trigos, parte baja del Cerro Zamorano en su ladera sur, 14 Aug 1998, *Gómez, M. et al. 598* (MEXU); Landa de Matamoros, Vuelta, al este de Landa de Matamoros, 25 Dec 1982, *Hernández Magaña, R. 9307* (MEXU); Arroyo Seco, al sur de Conca, 10 Feb 1988, *Herrera, A. 26* (MEXU); Landa de Matamoros, ca. 3-4 km al NW de Landa de Matamoros, 28 Jan 1989, *Lugo L, E. 55* (MEXU); Jalpan de Sierra, entronque ca. 10.5 km carretera Jalpan-Río Verde, ca. 1 km camino El Rayo via La Gachupina, 23 Apr 2010, *Ramosa Ventura, L.J. et al. 1143* (K); Hacienda Ciervo, 20 Aug 1905, *Rose, J.N. et al. 9670* (GH, NY, US); Landa de Matamoros, 1 km al oriente de El Purto de El Sabino, 15 Feb 1992, *Rubio, H. 2222* (MEXU); Landa de Matamoros, 10 km al SE de Agua Zarca, sobre el camino a Pisaflores, 16 May 1987, *Rzedowski, J. 43280* (MEXU); El Picacho, 22 Sep 1990, *Serrano, V. 10* (MEXU). **Quintana Roo**: José María Morelos, a 4.5 km al E del poblado Plan de la Noria, 16 Mar 2004, *Álvarez, D. & Ramírez, A. 8210* (MEXU); José María Morelos, a 6.19 km al NO de San Isidro Poniente camino a Sabana San Francisco (aguada seca), 17 Mar 2004, *Álvarez, D. & Ramírez, A. 8340* (MEXU); José María Morelos, a 2.78 km al N del poblado Zafarrancho, 19 Mar 2004, *Álvarez, D. & Ramírez, A. 8475* (MEXU); Othón P. Blanco, a 0.2 km al SSE de Dos Aguadas, 18 Jun 2004, *Álvarez, D. et al. 9380* (MEXU); En la entrada de la Brecha a Vallarta, a 2 km al S de Puerto Morelos, 7 Nov 1980, *Cabrera, E. & Cortés, L. 129* (BM, MEXU); brecha de Xaret 5 km al S de Playa del Carmen, 4 Dec 1980, *Cabrera, E. & Durán, G. 502* (MEXU); Puerto Morelos, 2 km al sur, en la brecha a Vallarte, 11 Jan 1981, *Cabrera, E. & Torres R, R. 875* (BM, MEXU); Akumal, 1 km al sur, carretera Cancún-Tulum, 14 Feb 1981, *Cabrera, E. & Ibarra Manríquez, G. 1151* (BM, MEXU); en los alrededores del al Laguna Muyil, a 20 km al S de Tulum, 7 Aug 1982, *Cabrera, E. & Cabrera, H. de 3373* (MEXU); Cozumel, Por el camino al Cedral, ó a 19 km al sur de la zona urbana de Cozumel, Isla de Cozumel, acahual de selva mediana, 4 Jun 1986, *Cabrera, E. & Cabrera, H. de 11424* (MO); 500m al N de Playa Lancheros, sobre el camino al Restaurant Hacienda Gomar, Isla Mujeres, *Cabrera, E. & Cabrera, H. de 15401* (BM, MEXU); Isla Mujeres, Isla Mujeres, 500 m al

norte de Playa Lancheros, sobre el camino al Restaurant Hacienda Gomar, ecotono entre manglar-selva baja-duna costera, 6 Jan 1988, *Cabrera, E. & Cabrera, H. de 15401* (MO); Isla Mujeres, Isla Mujeres, "Mugeres Island, Bay of Honduras", *Gaumer, G.F. s.n.* (K); Cozumel, Cozumel Island, 25 Apr 1885, *Gaumer, G.F. 61* (K); Felipe Carrillo Puerto, X-Hazil, 14 Oct 1987, *Mata P, S. 87-82* (MEXU); 6 km al S de Puerto Morelos, sobre la carretera Cancún-Tulum, 27 Jun 1976, *Moreno, P.P. 837* (MEXU); Benito Juárez, carretera Cancun-Puerto Juárez, 1 Jul 1980, *Puch, A. 24* (CICY, MEXU); Othón P. Blanco, camino al cenote Cocodrilo Dorado, La Unión, 2 km al E de poblado de La Unión, 6 Dec 2002, *Tapia, J.L. et al. 1370* (BM); A 14 km al este-noreste de Cobá, 3 Feb 1980, *Téllez V, O. & Cabrera, E. 1414* (MO); Felipe Carrillo Puerto, km 4 carretera Vigia Chico-Felipe Carrillo Puerto, 16 Dec 1985, *Villanueva, R. 555* (MEXU). **San Luis Potosí**: Ciudad Santos, Tsak Anam, 11 Dec 1978, *Alcorn, J.B. 2220* (MEXU); E of San Luis Postosi, Hwy. 86, 25 miles from Juarez Circle; beyond Xoconostle, 5 Jul 1971, *Andreasen, M. et al. 541* (MO); Mexquitic, 1.5 km al N de Tepona, 18 Aug 1976, *Bustos, M. et al. s.n.* (MEXU); 12 km N of El Cubo, 43 km W of Charcas on road to Cateras, 3 Jul 1972, *Chiang, F. et al. 8232 A* (MEXU); 35 miles E of San Luis Potosi, 10 miles W of sta Catarina, 11 Sep 1978, *D'Arcy, W.G. 11869* (MO); Media Luna, cerca del Río Verde, 13 Dec 1983, *Fernández y Acosta*, *2067* (F, MEXU); Villa de Reyes, 20 Km. porla carretera S.L.P.-Querétaro. 3.9 Km. por la carretera que va a Villa de Reyes. Matorral crassicaule con Opuntia y Myritillocactus geometrizans. Suelo franco-a-renoso, 8 Oct 1976, *García P, J. et al. 1255* (MO); on road from Tamasopo to Agua Buena, 14 Jun 1981, *Hahn, W. 531* (MEXU); Tamasopo, along road from hwy. 70 to city limits, 13 Jun 1981, *Hahn, W.J. 528* (MO); 7 Km. SW of Pozuelos and 22 Km. SW of San Luis Potosi on the highway to Guadalajara; then E. into sierra of igneous rocks. 22o 03' N 101o 101o 10'W, 21 Aug 1973, *Johnston, M.C. et al. 12266* (MEXU, MO, NY); El Naranjo, El Salto, 20 Feb 1961, *King, R.M. 3895* (US); El Naranjo, 2-3 miles W of Naranjo, 27 Feb 1961, *King, R.M. 3980* (US); along gravel road 6 miles generally NE of Xilitla, 24 Mar 1961, *King, R.M. 4259* (US); Charcas, Charcas, Jul 1934, *Lundell, C.L. 5163* (US); 22o N. Lat, 1878, *Parry, C.C. & Palmer, E. 634* (MO, NY, US); between Matehuala and Dr. Arroyo, 20 Aug 1973, *Powell, A.M. & Tomb, A.S. 2576* (US); km 30 carretera San Luis Potosí-Ríoverde, 20 Aug 1955, *Rzedowski, J. 6238* (MEXU); Santa María del Río, Ojocaliente, 7 Apr 1957, *Rzedowski, J. 8766* (US); San Luis Potosí, in convalli, 1877, *Schaffner, J.G. 692 [b]* (MEXU); Charcas, Charcas, Jul 1934, *Whiting, A.F. 913 -EB* (NY). **Sinaloa**: Mazatlan, On Isla Piedra, 31 Dec 1894, *Lamb, F.H. 362* (MO, US); Villa Union, Jan 1893, *Lamb, F.H. 408* (MO, US); **Sonora**: Cuesta Alta, Río Yaqui, 2.5 km S of Potam, 19 Nov 1985, *Felger, R.S. et al. 85-1361* (MEXU); in the vicinity of Alamos, Sonora, 6 Mar 1910, *Rose, J.N. et al. 12951* (F, NY, US); Navajoa, in the vicinity of Navojoa, Sonora, 21 May 1910, *Rose, J.N. et al. 13152* (NY, US); Rio Cuchujaqui near junction with Arroyo el Mentidero south of El Chinal road, 11.3 Km south of Alamos; tropical deciduos forest; 26, 5 Oct 1992, *Van Devender, T.R. et al. 92-955* (MO, UCR). **Tabasco**: Agua Blanca, Macuspana, 28 Mar 1987, *7to Semestre B*, *15* (MEXU); La Estrella, 22 Oct 1982, *Brigada Dunas*, *1103* (MEXU); Ciudad del Carmen, 1 km E de San Pedro, sobre la carretera Frontera-Zacatal, 28 Nov 1987, *Cabrera, E. & Cabrera, H. de 15044* (MEXU); Huimanguillo, 3.31 km al SO de Malpasito, rumbo a la Candelaria, 1 Feb 2002, *Calónico-Soto, J. et al. 21384* (MEXU); Centro, terreno de Pemex junto al Río Carrizal, 15 May 1997, *Castillo A, O. & Ascencio R, J.M. 63* (MEXU); Las Gaviotas, centro de la colonia, 20 Jan 1987, *Colorado L, J.R. s.n.* (MEXU); Huimanguillo, Ocuapán, rumbo a Mecatepec, km 9 al sure de Huimanguillo, km 2 rumbo a Fco. Rueda, 16 Jan 1979, *Cowan, C. 1809* (MEXU); Nacajuca, Nasateupa, al N de Nacajuca, Río Cuca, 17 Jan 1979, *Cowan, C. 1868* (MEXU); Nacajuca, Canellones chontales de tucta, cerca de Nacajuca, 17 Jul 1979, *Cowan, C. 2367* (MEXU); Centro, Ejido Corregidora Ortiz del Mezcalapa, 19 Jun 1982, *Escolastico, R. 65* (MEXU); a 13 km de Villahermosa por la carretera Internacional y a 800 m por el camino a El Zapote, 18 Jan 1966, *González L, L.A. & Pérez J, L.A. P-553* (MEXU); Villa Hermosa, a 13 kms de Villahermosa, por la carretera a Escárcega (Campeche) y a unos 800 m al SE, 10 Dec 1965, *González, L.A. & Pérez, L.A. 4152* (MEXU); Centla, camino al Ejido El Faisan, a orillas de camino, 20 Jan 1999, *Guadarrama O, M.A. et al. 6607* (MEXU); Jonuta, Rancho El Pastal, aproximadamente a 3 km hacia Cd. Pemex de la desviación a Monte Grande, 20 Jan 1999, *Guadarrama O, M.A. et al. 6842* (MEXU); Jonuta, a 5 km de El Palmar, orillas del Río San Pedro y San Pablo, 17 Sep 1988, *Guadarrama O, M.A. et al. 6959 .5* (MEXU); Macajuca, letrian de Pemex al las orillas de la Laguna Julivá, 14 Jun 1997, *Hanan A, M.A. et al. 996* (MEXU); Nacajuca, Letrina de Pemex a orillas de la Laguna Julíva. Con desechos de Pemex, 14 Jun 1997, *Hanan-Alipi, A.M. et al.*

996 (MO); just east of the Puente Frontera on Mexico 180, turn southward onto unpaved road to Jonuta and travel approx. 8.2 miles; near a concrete lower line pole marked "CFE 11700 1982 CRESET" and a surveyor's mark of station "13+870", 19 Apr 1987, *Lievens, A.W. & Gregory, B.M. 2377* (MEXU); Jonuta, en el km 7 de Colomo hacia la Pitaya, 13 Nov 1985, *Magaña, M.A. 1337* (MEXU); Jonuta, en km 10 de colomo hacia la Pitaya, 13 Nov 1985, *Magaña, M.A. 1339* (MEXU); Comalcalco, Ranchería Alrena, a 4 km de la Villa de Aldama, 10 Mar 1986, *Magaña, M.A. 1428* (MEXU); *Ortiz, P.S. s.n.* (MO); Cárdenas, C 9, 29 Sep 1971, *Puig, H. 199* (MEXU). **Tamaulipas**: Tampico, *Berlandier, J.L. 118* (BM, G-DC); Tampico, *Berlandier, J.L. 503* (BM, G-DC); Matamoros [circa Matamoros urbem], May 1831, *Berlandier, J.L. 2322* (BM, G-DC, GH, K, NY, W, W); San José, Sierra San Carlos, 19 Jul 1985, *Briones V, O.L. 1989* (MEXU); MEx 101, 12 miles E of Jaumave, 8 Sep 1978, *D'Arcy, W.G. 11843* (MO); Aldama, 5 km NW del Ej. El Nacimiento, 24 Mar 1985, *Díaz, R. et al. 334* (MEXU); Tampico, 3 km al W del pueblo de Tancol, 6 Feb 1968, *García Saucedo, D. s.n.* (F); Casas, 20 kms al NE de Casas, 29 Dec 1968, *González Medrano, F. 1874* (MEXU); Ciudad Victoria, 1 km al sur el Rancho El Novillo, a 20 km al W de Ciudad Victoria, 20 Nov 1991, *González Medrano, F. et al. 3215* (MEXU); Puerto de Arrazola, Sierra de Guadelupe, 18 Aug 1985, *González Medrano, F. 14713* (MEXU); Camino de Gomez Farias, Bosque Tropical Bosque Tropical Subcaducifolio, 13 Feb 1986, *Hernández Magaña, R. 1637* (MO); Guerrero, San Ignacio, 3 km al NE de la desv. del km 40 carretera Nuevo Laredo-Guerrero, 19 Oct 1984, *Hernández, L. 1228* (MEXU); 2 miles NE of Altamira, 3 Mar 1961, *King, R.M. 4087* (US); San Nicolás, Tamps. a 2 km del inicio de la brecha a González, 28 Nov 1998, *Martínez, M. 5345* (MEXU); Vicinity of Victoria, 1 Feb 1907, *Palmer, E. 27* (F, K, MO, US); Tampico, 1 Jan 1910, *Palmer, E. 40* (BM, K, US); Mesa del Diente, [part of Sierra de San Carlos], 20 Dec 1948, *Roybal, J.J. 2051* (US); in hills 19 km SE of Miquihuana on road to Palmillas, 11 Aug 1941, *Stanford, L.H. et al. 840* (MEXU); 6 kilo. W. of Miquihuana, 6 Aug 1941, *Stanford, L.R. et al. 724* (MO); Miquihuana, near reservoir at base of hills, 10 Jul 1949, *Stanford, L.R. et al. 2386* (US); 3 mi N of Miquihuana, 14 Jul 1949, *Stanford, L.R. et al. 2445* (US); Jaumave, Jaumave, Jul 1930, *Viereck, H.W. 772* (US); Jaumave, Jaumave, Dec 1930, *Viereck, H.W. 885* (US). **Tlaxcala**: Españita, 4 km al E de Españinta, ca. de Las Pilas, 26 Aug 1989, *Ángeles, E. 6* (MEXU); Tecarona, Mt Malinche, 21 Jun 1938, *Balls, E.K. B 4863* (BM, K, US); Los Pilares, Mt. Malinche, Tlax, 15 Oct 1938, *Balls, E.K. & Gourlay, W.B. B 5645* (K, US); Los Pilares, Mt Malinche, 15 Oct 1938, *Balls, E.K. B 5646* (BM, E, US); Españita, 4 km al E de Españinta, ca. de Las Pilas, 30 Jun 1990, *Ramírez, R. 237* (MEXU). **Veracruz**: Veracruz, San Juan Ulua, en la entrada, 20 Apr 2004, *Acosta R, I. & Palestina, R. 1948* (MEXU); La Antigua, J. Ingenieros, 22 Apr 2004, *Acosta R, I. & Palestina, R. 2104* (MEXU); Tuxpam, Central Termoeléctrica TUXPAN V, en la barrera de casuarinas de enfrente, 5 May 2004, *Acosta R, I. & Palestina, R. 2143* (MEXU); Xalapa, Rancho Guadalupe, 15 Feb 1987, *Acosta, L.E. 17* (MEXU); San Miguel, carretera Mexico-Veracruz km 445, 28 Mar 1982, *Alzate, H. 22* (MEXU); Huatusco, camino a Tepampa a 4 km de la cerretera Huatusco-Coscomatepec, 10 Aug 1979, *Avendaño Reyes, S. & Calzada, J.I. 416* (F); Huatusco, km 5 camino Huatusco-Elatepec, 15 May 1993, *Avendaño Reyes, S. & Duran E, C. 3118* (MEXU); Loma-Grande, 17t Orujaha [hand writing unclear], 28 Apr 1938, *Balls, E.K. & Gourlay, W.B. B 4381* (E, K, US); Mun. Perote, below San Vigas, 4 Jun 1938, *Balls, E.K. & Gourlay, W.B. B 4747* (K); San Andrés Tuxtla, Volcán San Martin Tuxtla, dentro del crater, 29 Jun 1972, *Beaman, J.H. & Álvarez de C, C. 6320* (F); Orizaba [Veracruz/Puebla], *Botteri, M. s.n.* (F); Orizaba, Aug 1852, *Botteri, M. 846* (BM, K); region de Orizaba, 30 Jun 1866, *Bourgeau, E. 2555* (K); Coscomatepec, 5 km al W de Tetelcingo, 12 Aug 1979, *Bretting, P.K. M 4* (MEXU); 5 km al N de Jalapa, cerca de la orilla de la carretera 140, 20 Oct 1979, *Bretting, P.K. 121* (MEXU); Hidalgotitlán, alrededores del Campamento Hermanos Cedillo, 9 Mar 1974, *Brigada Dorantes*, *2471* (F, MEXU); Hidalgotitlán, kms 2-3 del camino de Paln de Arroyos-Alvaro Obregón, 12 Apr 1974, *Brigada Dorantes*, *2746* (MEXU); Hidalgotitlán, alrededores del campamento Hnos. Cedillo, 5 Nov 1974, *Brigada Dorantes*, *3739* (MEXU); Hidalgotitlán, brecha Hnos. Cedillo-Agustin Melgar, 28 Apr 1974, *Brigada Vásquez*, *463* (MEXU); Mariano Escobedo, road to La Perla N of Orizava, near Rancho del Mirador, 10 Jul 1977, *Bye, R.A. 7558* (MEXU); Huatusco, camino a Tecampa a 4 km de la carretera Huatusco-cuscomatepec, 1 Aug 1979, *Calzada, J.I. 416* (MEXU); Catemaco, Las Cabañas, 5 km al E de la desviación de la carretera que va al Jicacal, 22 Oct 1974, *Calzada, J.I. 1607* (MEXU); Jalapa, Rancho La Laguna a 1 km del Rio Sordo Carretera Nueva de Jalapa Coatepec, Jalapa, Veracruz, Mexico, 6 Aug 1975, *Calzada, J.I. 1848* (K, MO); San Andrés Tuxtla, camino al Cerro De Vigia, al N

de San Andrés Tuxtla, 6 Apr 1985, *Calzada, J.I. 11951* (MEXU); Oluta, Oluta, 3 km de Acayucán, 11 Sep 1986, *Calzada, J.I. 12687* (MEXU); Oluta, Oluta, 3 km de Acayucán, 11 Sep 1986, *Calzada, J.I. 12705* (MEXU); Xalapa, Rancho Guadelupe, Jardín Botánico a 3 km de Xalapa por la carretera vieja a Coatepec, 26 Jul 1976, *Castillo C, G. 3* (F); Actopán, El Moro de la Mancha, Playa Paraíso, 6 Sep 1977, *Castillo C, G. 188* (MEXU); Alto Lucero de Gutiérrez Barri, La Piedra Cuata, entre Plan de las Hayas y Tierra Blanca, 7 Apr 1981, *Castillo C, G. & Vázquez, F. 1373* (F); Veracruz, Playa Norte de Veracruz, al N de la planta de tratamiento, 15 May 1996, *Castillo Campos, G. 14751* (MEXU); Zongolica, Reserva del Bicentenario, zona de amortiguimiento Instituto Tecnologico Suprior de Zongolica, km 4 de la Carretera a la Compañia, 3 Feb 2012, *Castillo-Hernández, L.A. 370* (MEXU); Perote, malpais 5 km al O de Frijol Colorado hacia Guadelupe Zarabia, 23 Dec 1980, *Cházaro B, M. et al. 6089* (MEXU); Papantla, Cazuelas, brcha Papantla-Pte. Remolino, 12 Nov 1981, *Cortés, M.E. 68* (MEXU); Coatzintla, Region Centro-Norte del Edo. De Veracruz. Palmar de Zapata, 26 Jan 1982, *Cortés, M.E. 113* (MEXU, MO); Papantla, Cerro del Carbón, 3 Feb 1982, *Cortés, M.E. 156* (MEXU); Coatzintla, palmar de Zapata, Mpio. de Coatzintla, 8 Oct 1982, *Cortés, M.E. 463* (MO); Coatzintla, Palmar de Zapata, 8 Oct 1982, *Cortés, M.E. 463* (MEXU); Xalapa, *Coulter, T. 1232* (K); Córdoba, Hillside above San José de Gracia, 1 mile S of highway between Córdoba and Veracruz, 28 Jun 1977, *Croat, T.B. 39585* (MO); San Andrés Tuxtla, Along road between Sontecomapan and Estación de Biología Tropical Los Tuxtlas, N of San Andrés Tuxtla, S of Montepio, 5 mi and 2 km E of Estación Biología Tropica Los Tuxtlas, 16 Jan 1987, *Croat, T.B. & Hannon, D.P. 63122* (MEXU, MO); Paved road from Orizaba to La Perla off route 180. At rancho on it hand side. Elevation 4600 fe[et], 11 Jul 1977, *Davis, T. 735* (MEXU, MO); San Andrés Tuxtla, Balzapote, 20 May 1995, *Díaz Rico, A. 95* (MEXU); alrededores del campamento hnos. cedillo. hidalgotitlan, 1974, *Dorantes, B. 2471* (MO); Hidalgotitlán, Kms. 2-3 del camino de plan de arroyos-alvaro obregon, selva alta perennifolia, secundaria, suelo cafe obscuro arcilloso, con mucha roca, 12 Apr 1974, *Dorantes, B. 2746* (MO); Emiliano Zapata, 1 km N of Lencero, 15 Nov 1972, *Dorantes, J. et al. 1865* (MEXU); Emiliano Zapata, Tiradores, 20 Apr 1973, *Dorantes, J. & Acosta, M. 1983* (MEXU); Coxquihui, Ejido Sabanas de Xalostoc, 27 Jul 1980, *Evangelista, M. & Mendoza, M. 45* (MEXU); Vigas de Ramirez, R. Ramirez, 23 Jun 1973, *Gandara, J.M. & Dorantes, J. 84* (F, MEXU); Córdoba, 25 Jan 1906, *Greenman, J.M. 167* (F); Poza Rica de Hidalgo, 1 km. N, 23 Jun 1980, *Hansen, B.F. & Nee, M. 7419* (F, MO); S of Tampico along Hwy 180, 3.8 mi N of Ozuluama, 3 Jan 1984, *Hardison, L. et al. 52* (MEXU); Emiliano Zapata, Barranca de San Antonio, 8 Apr 1976, *Hernández A, C. 90* (K, MEXU); Huayacocotla, camino a Arroyo Hondo, 22 Oct 1970, *Hernández, R. & Cedillo Trigos, R. 835* (MEXU); Xalapa, Lago del Castillo, alrededores, 15 Jan 1987, *Herrera C, M. 83* (MEXU); 8 miles west of Jalapa on Hwy 140, 3 Sep 1980, *Hsiao, T.H. 104* (F); Huayacocotla, Agua de la Calabaza, camino de la cascada 17 km NE de Huayacocotla, 22 Apr 1981, *Juárez G, L.G. & Vasquez B, F. 44* (F); Sierra Santa Marta, 8 Jul 1999, *Leonti, M. & MEXU 86* (MEXU); ca 1km E Puebla line Rte 150D, 23 Mar 1970, *Long, R.W. & Burch, D. 3083* (F, MO); 2 km de La Joya, camino a Perote, 12 May 1969, *Lot, A. 272* (F); San Francisco, a 7 km de Tempoal rumbo a Tantoyuca, 12 Aug 1969, *Lot, A. 422* (MEXU); Isla Lobos, 14 Apr 1971, *Lot, A. 1314* (MEXU); Dunas de Barrillas, recodo de Laguna Ostion, 17 Dec 1971, *Lot, A. 1586* (MEXU); Veracruz, 1700 m de km 6 de la carretera Veracruz-Cardel, 6 Dec 1987, *Luna M, V.E. 114* (MEXU); Xico, Isleta Chica, 27 Nov 1993, *Luna M, V.E. 760* (MEXU); Jesús Carranza, 2 km al W del campamento Hnos. Cedillo, 18 Oct 1974, *Márquez Ramírez, W. 285* (MEXU); Atzalán, La Calavera, carretera Altotonga "Tlapacoyan", 17 May 1973, *Márquez, W. & Dorantes, J. 137* (F, MEXU, MO); San Lorenzo Tenochtitlan, 29 Nov 1967, *Martínez A, M.A. 535* (MEXU); Otatitlán, Playa de Vaca, *Martínez C, G. 1342* (MEXU); San Andrés Tuxtla, Estación Biológica de Los Tuxtlas, 16 Jan 1969, *Martínez C, G. 1830* (MEXU, MO); Calcahualco, Xamaticpaz, camino a Calcahualco, barranca Jamapa, ladera N, 24 May 1985, *Martínez, J.L. & Vazquez, F. 251* (MEXU); Calcahualco, Escola, camino a Xamaticpac, barranca del Río Jamapa, 29 Jul 1985, *Martínez, J.L. & Vázquez, F. 488* (MEXU); Calcahualco, a 2 km de la desviación a Xamaticpac, camino a Xacaxomulco, 28 Aug 1985, *Martínez, J.L. & Vázquez B, F. 643* (MEXU); Maltrata, May 1937, *Matuda, E. 1296* (MO, US); Ixhuatlancillo and vicinity, ca. 10 km north of Orizaba, 3 Jun 1987, *Miller, J.S. & Torres C, R. 2980* (MO); Orizaba, Jul 1887, *Mohr, C. s.n.* (US); Tampico Alto, Isla Lobos, Sep 1994, *Moreno C, P. 1325 B* (MEXU); Coatepec, Tuzamapán, 25 Feb 1975, *Murrieta, Y. 56* (F); Catemaco, cultivated in Illinois, from Playa Escondida on Gulf of Mexico, 10km N of Sontecomapan, voucher Schatz G & Nee M 210 Jan 14 1981 18 Sep 1982, *Nee, M. s.n.*

(GH, MEXU, MO); Tecolutla, 1.5 km SE oF Guadelupe along Mex. Hwy 180, 24 Jun 1980, *Nee, M. & Hansen, B.F. 18510* (F, MEXU); Atzalán, Puente de Rieles, 4 km NE of Altotonga (^.5 km by road) on road to Tlapacoyan, 28 Jun 1980, *Nee, M. & Hansen, B.F. 18718* (F); Tantoyuca, 10km SE of Tantoyuca, along hwy. Mex. 105, 4 Jan 1981, *Nee, M. et al. 19535* (BM, F, MEXU); Calcahualco, 5.3 km W of Escola on road to Jacal, 18.5 km by road NW of Coscomatepec, 12 Jan 1981, *Nee, M. & Schatz, G. 19727* (F); Chalma, 7.5 km N of Heujutla, on aroad to Platón Sánchez and Tempoal, 24 Oct 1981, *Nee, M. 22356* (F); Perote, 1.5 km N of Tenextepec and 5.5 km SSE of Guadalupe Victoria (+-= Libertad), Mun. Perote, 7 Nov 1981, *Nee, M. 22817* (F); Chocamán, 8.5 km by road Wof Chocamán, at about the highest point on gravel road to Xocotla, 18 Nov 1981, *Nee, M. 23310* (F); Teocelo, Gorge of Río Teocelo, 2km NW of Teocelo, 7km (by air) SSW of Coatepec, along Teocelo-Coatepec highway, 2 Dec 1981, *Nee, M. 23556* (BM, CORD, F, MEXU); San Andrés Tuxtla, 5.5 km by road S of Sihuapan and junction with hwy Mex 180, alogn dirt road to Salto de Eyipantla, 6 km (by air) S of San Andrés Tuxtla, 4 Dec 1981, *Nee, M. 23696* (F); Villa Azueta, South edge of Tesechoacán, W side of Río Tesechoacán (= Río Playa Vicente), 6 Dec 1981, *Nee, M. 23821* (BM, F); Chocamán, 1 km N of Chocomán, gorge of river upstream from Chocomán-Coscomatepec highway, 7 Dec 1981, *Nee, M. 23899* (F); Soteapan, Volcán Santa Marta, along trails to base of volcano, 0-3 km E of village of Santa Marta, 29 Jun 1982, *Nee, M. et al. 24663* (F); Huayacocotla, 3 km SW of Huayacocotla on road to Palo Bendito, 22 Jul 1982, *Nee, M. & Diggs, G. 25198* (F); Hidalgotitlán, Near Campamento La Laguna, 5 Mar 1984, *Nee, M. 29987* (BM, MEXU, MO, US); Perote, 4km by air NW of Frijol Colorado, 24 Aug 1986, *Nee, M. 32945* (BM, F, LE, MO, US); Omealca, near Omealca, 6 Mar 1894, *Nelson, E.W. 169* (US); Lagunetas de Nistamalapa, 1-2 km cerca del Hotel Playa Azul, 14 Apr 1969, *Nevling, L. & Gomez-Pompa, A. 851* (MEXU); 5 km del limite de los estados Puebla y Veracruz, autopista, 8 Jul 1970, *Nevling, L.I. & Gomez-Pompa, A. 1288* (F); carretera Mexico-Orizaba, cerca del limite con Puebla, 26 Jul 1971, *Nevling, L.I. & Gomez Pompa, A. 2004* (MEXU); Sanborn, 18 Apr 1910, *Orcutt, C.R. 6786* (MEXU); Siteapán, Volcán Santa Martha, en las faldas, 21 Dec 1978, *Ortega B, R. 1143* (MEXU); Soteapan, Volcán Santa Marta, en las faldas, 21 Dec 1978, *Ortega O, R. 1143* (F); Jilotepec, Vista Hermosa, 20 Oct 1977, *Ortega Ortíz, R. 724* (F, MEXU); Xalapa, carretera vieja a Coatepec, 13 Jun 1981, *Ortega, J.F. et al. s.n.* (MEXU); Jilotepec, Jilotepec, 3 km al oeste, 10 May 1976, *Ortega, R. 236* (F); Banderilla, Vivero Banderilla (S.A.R.H.), 17 Apr 1980, *Palma G, J. 139* (F); Catemaco, Isla de Agaltepec, lado NW, 26 Apr 1974, *Ponce C, F. & Albarez, C. 328* (MEXU); Alchichica, 28 Oct 1967, *Ramos A, C.H. 119* (MEXU); al sur de El Limón, 25 May 1968, *Ramos A, C.H. 217* (MEXU); Texistepec, meseta de San Lorenzo Tenoochtitlan (potrero), 20 Nov 1995, *Ramos, C.H. & Martínez S, E.M. 1176* (MEXU); Coatzacoalcos, laguneta en al Congrecación de Mundo Nuevo, 15 Jun 2003, *Ramos, C.H. 2618* (MEXU); Tezonapa, 3 km aproximadamente al souroeste de Marzorango, cerca de la finca de Cedonio Hernández, 28 Feb 1986, *Robles G, R. 423* (MEXU); Tezonapa, Sierra Cruz Tetela, a 3 km aprox. al sureste de Motzorongo, rumbo a la parcela de Olivares, 11 Apr 1986, *Robles G, R. 593* (MEXU); Axocuapán, Monte Rey, Ejido Coatzalan, 4 Mar 1983, *Robles H, L. 23* (MEXU); orizaba 51-4-B, 17 Sep 1966, *Rosas R, M. 52* (K, MEXU); Tocuila al N de Orizaba, D-1-51, 19 Nov 1967, *Rosas R, M. 770* (BM); Cuapichapa Km 2 al S del Puente de San Miguel. E-3-51, 7 Jan 1968, *Rosas R, M. 952* (BM, MEXU); Rincón Barrientos-Cuauhtlapan, D-3-51, 25 Jan 1968, *Rosas R, M. 1082* (BM, MEXU); al n de frijol colorado, camino a Mastalogon; matorral de Nolina-Yucca con Junipereus, suelo arenoso, 1982, *Sandoval, ?*, *CS-115* (MO); Catemaco, Playa Escondida, 10 km N of Sontecomapan, 14 Jan 1981, *Schatz, G.E. & Nee, M. 208* (BM, F, MEXU); Catemaco, Playa Escondida, 10km N of Sontecomapan, 14 Jan 1981, *Schatz, G.E. & Nee, M. 210* (BM, F); Jalapa, 1829, *Schiede, C.J.W. & Deppe, F. s.n.* (BM); Jalapa, Mexico, *Schiede, C.J.W. 133* (MO); Mt. Orizaba [Veracruz/Puebla], 4 Aug 1891, *Seaton, H.E. 153* (F); San Andrés Tuxtla, Laguna Escondida, 3 km al NO de la Estación Biológica Las Tuxtlas, 23 Jan 1986, *Sinaca Colín, S. 404* (MEXU); San Andrés Tuxtla, Estación Biológica Las Tuxtlas, 18 Mar 1992, *Sinaca Colín, S. 1708* (MEXU); Tuxtepec, Cerro Brujo, 12 Sep 1961, *Sousa, M. 761* (MEXU); Luz del Barrio, 10 Jun 1993, *Sousa-Peña, M. 510* (MEXU); Perote, W of Jalapa along Hwy 140 to Peubla about 30 miles W of Jalapa, about 2 miles E of Perote, 17 Jul 1983, *Taylor, C.M. 2261* (DUKE); Perote, road to Los altos, 5 miles S of Hwy 140 at Perote, 17 Jul 1983, *Tucker, G.C. 2126* (DUKE); Tepetzintla, Sierra de Otontepec, al NE de Tepetzintla, 26 Aug 1981, *Vázquez B, F. 196* (F); Emiliano Zapata, Obscuro, por la carretera Chevarrillo-El Palmar, 27 Jul 1983, *Vázquez B, F. 355* (MEXU); Teocelo, Barranca de Teocelo, 12

Dec 1975, *Vazquez T, V. 73* (F); Fortín, Fortín, 21 Apr 1976, *Velázquez L, C. 182* (MEXU, MO); Jalacingo, Ocotepec, 27 Jul 1970, *Ventura A, F. s.n.* (CORD); Jalacingo, Ocotepec, 27 Jul 1972, *Ventura A, F. 5818* (F, MEXU); Jilotepec, Rincón del Muerto, 6 Apr 1974, *Ventura A, F. 9850* (F); Huatusco, Coscomatepec, Ejido Chavaxtla, 12 Mar 1984, *Yacotú, J.L. 11* (MEXU); Xalapa, Tiro de Haya, ejido del Sumidero, 7 Dec 1989, *Zamora C, P. 1586* (MEXU); Tlalnehuayocan, entre los Carolinas y Rancho Viejo, 24 Nov 1990, *Zamora C, P. 2801* (MEXU); Jilotepec, Jilotepec, 3 km al oeste, 10 May 1976, *Zola B, M.G. 410* (F); Jilotepec, El Esquilón, El Esquilon, Jiltopec, veracruz; bosque de pino-encino, secundaria suelo roca volcanica, 11 Jun 1976, *Zola B, M.G. 428* (K, MEXU, MO); Veracruz, Rancheria Neveria, carretera antigua nacional Xalapa-Veracruz, 14 May 1981, *Zola B, M.G. et al. 1018* (MEXU); Paso de Ovejas, La Vibora, 3 Apr 1986, *Zola B, M.G. & Zamoa, P. 1113* (MEXU); Veracruz, km 4 carretera Veracruz-[José] Cardel [georeference incorrect on sheet, says 19°12'N, 19°12'W], 10 Mar 1988, *Zola B, M.G. 2399* (MEXU). **Yucatán**: Tekax, a 2.2 km al S de Sudzal Chico (primera aguada), 19 Aug 2005, *Álvarez, D. et al. 11564* (MEXU); Mérida, Midway between Progreso and Dzibilchaltún, 11 Nov 1979, *Bradburn, A.S. 1430* (MEXU, MO); 4-6 km al O de Las Coloradas, sobre camino al crucero San Felipe-Río Lagartos, 22 Mar 1988, *Cabrera, E. 15749* (MEXU); Progreso, Colonia Costa Blanca, por la carretera para Chicxulub, 5 Nov 1980, *Calzada, J.I. et al. 6543* (CICY, MEXU); Panabá, camino de San Diego a Santa Rosa (loc. dudosa), 14 Mar 1985, *Chan, C. 4883* (MEXU); Maxcanú, Chunchucmil, 3 Mar 1988, *Chávez, M. 94* (MEXU); Mérida-Progreso, Jan 1956, *Enríquez, O.G. 315* (MEXU); *Gaumer, G.F. 479* (BM, E, K, K, W); Cacalchén, Kancabdzonot, Jan 1917, *Gaumer, G.F. & Sons 23546* (MO, US); Mérida, Jardin Botánico CICY, 7 Oct 1982, *Góngora, E. 137* (MEXU); Yukalpeten, Hwy 261, 23 Nov 1980, *Hsiao, T.H. 120* (F); Mérida; selva baja caducifolia, Huerta, secundaria, suelo tzekel, 30 Nov 1990, *May-Pat, F. 668* (CICY, MO); Yaxcabá, Tixcacaltuyub, Mar 1988, *Rico-Gray, V. 710* (MEXU); Yaxcabá, Tixcacaltuyub, Mar 1988, *Rico-Gray, V. 711* (MEXU); Yaxcabá, de Tixcacaltuyub 13 km rumbo a Peto, crucero Santa Maria, 2 Jan 1988, *Simá, P. 422* (MEXU); Mérida. Jardín Botánico Regional CICY. Yucatán. Selva baja caducifolia, suelo tzekel (litosol), 28 Mar 1995, *Simá, P. et al. 1776* (CR); Dzitás, Dzitás, 13 Sep 1984, *Ucán, E. 3523* (MEXU). **Zacatecas**: La Encantada, 11 Aug 1948, *Dressler, R.L. 160* (MO); Moyahua, Cañón de Juchipila, entre el cerro La Manga y Los Pochotes, al sur de la comunidad de Santa Rosa, por el Río Juchipila, 5 Mar 1998, *Enríquez E, E.D. 1763* (MEXU); Moyahua, Cañón de Juchipila, entre el cerro La Manga y Los Pochotes, al sur de la comunidad de Santa Rosa, por el Río Juchipila, 8 Jul 1998, *Enríquez E, E.D. & Balleza C, J.J. 1773* (MEXU); Concepción del Oro, Sierra Madre Oriental [Aranzazú del Cobre], 19 Jul 1934, *Pennell, F.W. 17441* (US); 23.5 mi NW of jct. of Rte. 45 and 49 on Rte. 45, 16 Aug 1979, *Wagner, W.L. & Solomon, J.C. 4249* (MO).

NICARAGUA. "Guatemala, San Juan de Nicaragua", 1841, *Friedrichsthal, E. von*, *545* (W). **Atlántico Norte**: Reserva Bosawas, Mpio. Musawas, zona Musawas, Waspuk, 1 Mar 2001, *Aker, C. 422* (MO); Municipio de Bonanza, Reserva de Bosawas, Comunidad de Musawas, suroeste de Musawas, 22 Sep 2003, *Coronado, I. & Gurdián, C. 341* (MEXU, MO); Carretera nueva a Waní, El Hormiguero, 1 Apr 1983, *Ortiz, F. 1205* (MO); Cerro Waylawás, W slope of central range, 12 Mar 1979, *Pipoly, J.J. 4530* (MO); Finca below (S of) La Pimienta, 19 Mar 1980, *Pipoly, J.J. 6279* (MO). **Atlántico Sur**: Nueva Guinea; roadsides and airport [Seymour series], 30 Mar 1971, *Atwood, J.T. 5367* (MO); El Bluff, near Bluefields [Seymour series], 14 Dec 1968, *Hamblett, R.B. 622* (MO); Logging camp near Quebrada La Talolinga, 19 Aug 1983, *Miller, J.S. & Sandino, J.C. 1168* (MO); Along Río Rama immediately above Rapido Machuca, 16 May 1978, *Stevens, W.D. 8896* (MO). **Boaco**: Cerro Mombachito, 8.5 km NO de Camoapa, 24 Jan 1980, *Araquistain, M. & Moreno, P.P. 1030* (MEXU, QCA); just west of Boaco, route 9; in mountains [Seymour series], 30 Dec 1968, *Moore, A.D. 1461* (MO); Cerro Mombachito, al SE de la cuidad de Boaco, 10 May 1980, *Moreno, P.P. 318* (MEXU); NE de Mombachito, 11 May 1982, *Sandino, J.C. 2860* (MEXU, MO). **Chinandega**: Volcán Casita, Montañas El Uval, Hacienda Bellavista, ca. 30 km al N de Posoltega, 16 Oct 1982, *Grijalva, A. & Grijalva, M.V. de 1441* (MEXU, MO); Volcán Casita, Montañas El Uval, Hacienda Bellavista, ca. 30 km al N de Posoltega, 16 Oct 1982, *Grijalva, A. & Grijalva, M.V. de 1508* (MEXU, MO); Río El Gallo, a 6 km de Cinco Pinos, carretera a San Francisco del Norte, 28 Sep 1981, *Moreno, P.P. 11744* (MO); Potosí, Sitio Santa Julia; bosque seco tropical, 26 Oct 1983, *Robleto, W. 72* (MO); Km 146.5 carretera a El Guasaule, Volcán Chonco, 18 Nov 1982, *Sandino, J.C.*

3830 (MEXU, MO). **Chontales**: Santo Domingo, 13 Jan 1970, *Narváez, E. 3371* (BM, MO); Along road from Juigalpa NE toward La Libertad, ca. 17.4 km NE of Río Mayales, at ford of Río El Bizcocho; pastures, gallery forest and steep cliffs S of river, 23 Sep 1977, *Stevens, W.D. 4104* (MO). **Estelí**: Cerro Tisey, faldas del lado S, 10 Jul 1981, *Moreno, P.P. 9688* (MO); 1.5 km al N del valle San José de la Laguna, camino a San Nicolas, 23 Sep 1981, *Moreno, P.P. 11348* (MO); Km 163 a 164, Llano de Santa Adelaida, 3 Jan 1982, *Moreno, P.P. 14082* (MO); Rodeo Grande, al noroeste de Estelí, 14 Jul 1982, *Moreno, P.P. 16902* (MO); Hacienda Varela, 17 km de Estelí, camino al Sauce, 17 Sep 1982, *Moreno, P.P. 17433* (MO); Quetzalcayán, al E del Cerro Tisey, 15 Oct 1982, *Moreno, P.P. 17865* (MO); Salto de Estanzuela [Seymour series], 13 Aug 1976, *Nelson, E.B. 7700* (CR, MO); Municipio de Esteli, ásentamiento Puertas Azules, 17 Apr 1999, *Rueda, R. & Velásquez, W. 10753* (MO); Municipio de Esteli, ásentamiento Puertas Azules, 17 Apr 1999, *Rueda, R. & Velásquez, W. 10873* (MO); 7 km NW of San Nicholas, near Cerro Santa Rosa, N side of peak, 12 Aug 1976, *Seymour, F.C. 7647* (MO); Llano Almaciguera, ca 8.4 km S of Hwy. 1 (just S of Estelí) on road through Estanzuela, ca 2.8 km S of Río Estanzuela bridge, 13 Nov 1978, *Stevens, W.D. 10727* (MO); N slope of Cerro Tomabú, 16 Oct 1979, *Stevens, W.D. & Araquistain, M. 14920* (MEXU, MO, QCA); 4.9-7.6 km NE of Hwy. 1 at Estelí along road to Yalí, 14 Nov 1979, *Stevens, W.D. & Grijalva, A. 15537* (MEXU, MO, QCA); 3.0 km E of Hwy 1 along road to Laguna Miraflores from near agricultural school N of Estelí, 17 Jun 2008, *Stevens, W.D. et al. 27304* (MO). **Granada**: Volcán Mombacho, Plan de las Flores, alrededor de la torre de TELCOR; nebliselva tropical, 17 Jan 1980, *Araquistain, M. & Moreno, P.P. 642* (MO); Volcán Mombacho; nebliselva, 31 Jan 1980, *Araquistain, M. & Moreno, P.P. 1110* (MEXU, MO); Volcán Mombacho; nebliselva, 31 Jan 1980, *Araquistain, M. & Moreno, P.P. 1202* (MEXU, MO); Granada, 2 Feb 1903, *Baker, C.F. 146* (MO); Volcán Mombacho, 10 km al NE de Granada sobre el camino a Cutirre, 28 Jul 1980, *Moreno, P.P. 1455* (MO); Los Charcos, 15.5 km de Granada, carretera a Panaloya, 28 Jan 1981, *Moreno, P.P. & Sandino, J.C. 6236* (MEXU, MO); 3 km de Granada, camino a Panaloya, 28 Jan 1981, *Moreno, P.P. 6251* (MO); Kuahyeri, 8 km de Granada en la carretera a Panaloya, 25 Feb 1981, *Moreno, P.P. 7189* (MO); Volcán Mombacho, 1 km al SE de Cutirre, 11 May 1981, *Moreno, P.P. & Henrich, J. 8400* (MEXU); Volcán Mombacho, 1 km al sureste de Cutirre, 11 May 1981, *Moreno, P.P. & Henrich, J.E. 8440* (MO); Lado noroeste de Volcán Mombacho, Finca San Joaquin, 15 May 1981, *Moreno, P.P. & Henrich, J. 8484* (MEXU, MO); 9 km al S de Granada, Carretera No. 6, 18 Jul 1981, *Moreno, P.P. 9949 B* (MO); Volcán Mombacho, 7.5 miles SW of Granada; part way up mountain in wet forest [Seymour series], 9 Jan 1969, *Nichols, C.E. 2013* (MEXU, MO); Volcán Mombacho, below summit [Seymour series], 1 Aug 1972, *Robbins, S.B. 6263* (MO); 7 km al norte de INTECNA, 24 Jun 1981, *Sandino, J.C. 700* (MEXU, MO); 1 km al norte de INTECNA, puente en camino hacia el Paso de Panaloya, 24 Jun 1981, *Sandino, J.C. 730* (MEXU, MO); W slope of Volcán Mombacho, road and trail above Finca Santa Ana, from reservoir to somewhat above Plan del Flores, 1 Oct 1977, *Stevens, W.D. 4369* (MO); Volcán Mombacho, camino a Las Antenas, 22 Nov 1981, *Téllez V, O. et al. 5144* (MEXU). **Jinotega**: Municipio de Wiwilí, Reserva de Bosawas, comunidad de San Andrés, Caño Pilawas, 8 Apr 2005, *Coronado G, I. et al. 1433* (MO); Municipio de Wiwilí, Reserva de Bosawas, desembocadura del Río Bocay, 9 Feb 2005, *Coronado, I. et al. 1142* (MO); Cuá Bocay, Reserva de Bosawás, Municipio de Wiwilí, Reserva de Bosawas, cominidad de San Andrés, Caño Pilawas, 11 Apr 2005, *Coronado, I. et al. 1542* (BM, MO); San Ramón, lado este de las faldas de Cerro Kilambé, 24 Mar 1981, *Moreno, P.P. & Sandino, J.C. 7402* (MO); Reserva Natural Kilambé. Muncipio de Bocay, Comunidad San Miguel de Kilambé, 6 Jan 2001, *Rueda, R. et al. 15236* (MO); macizos de Peñas Blancas, along trail between finca of Manuel Estrada (El Cielo) and finca of Socorro Mejia, 14 Jan 1979, *Stevens, W.D. 11419* (MEXU, MO); macizos de Peñas Blancas, vicinity of finca of Manuel Estrada (El Cielo), above Río El Gusaneras, 13 Jan 1979, *Stevens, W.D. 11632* (MEXU, MO); Ca. 20.3 km NE of Hwy. 1 at Estelí on road to Yalí, 16 Nov 1979, *Stevens, W.D. & Grijalva, A. 15784* (MO). **León**: León, 21 Dec 1975, *D'Arcy, W.G. 10428* (MO); Along new road from Hwy. 1 (at ca. Km 135.5 and ca. 10.6 km W of bridge at La Trinidad) to San Nicolás, ca. 9.5 km from Hwy. 1, 31 Aug 1978, *Stevens, W.D. 10255* (MEXU, MO); Along new road from Hwy. 1 (at ca. Km 135.5 and ca. 10.6 km W of bridge at La Trinidad) to San Nicolás, ca. 9.5 km from Hwy. 1, 31 Aug 1978, *Stevens, W.D. 10274* (MO); Between El Portillo and Quebrada de Agua, 21 Sep 1980, *Stevens, W.D. & Montiel, O.M. 17912* (MO); 17.1 km W of highway at Estelí along road to Achuapa, 10 Feb 2008, *Stevens, W.D. et al. 27002* (MO). **Madriz**: Falda W del cerro Volcán de Somoto (Tepesomoto), 15

Apr 1980, *Araquistain, M. & Moreno, P.P. 2080* (BM, MEXU, MO); Cerro Quisuca, summit and upper slopes, 22 Nov 1979, *Stevens, W.D. & Grijalva, A. 16051* (MEXU, MO, QCA); Lower W slope of Cerro Volcán de Somoto, 13 Dec 1979, *Stevens, W.D. & Grijalva, A. 16335* (MO). **Managua**: Finca Armenia, ca 3 km al SE de Las Conchitas (sobre carretera # 8); bosque húmedo tropical en cafetales, 2 Oct 1982, *Grijalva, A. & Grijalva, M.V. de 1322* (MO); Camino a Salamina, 1 km al O de Samaria, en el cruce del Río El Carmen, 14 Jun 1980, *Guzmán, M. & Castro, D. 130* (MEXU, MO, QCA); Península de Chiltepe, sector NE de la Laguna de Jiloá; bosque tropical seco, 3 Jul 1980, *Guzmán, M. et al. 289* (MEXU, MO); Comarca San Isidro de la Cruz Verde. 2 km S del Colegio Centro America, sobre el camino a San Isidro de la Cruz Verde, 9 May 1981, *Guzmán, M. 1805* (MEXU); Cuatro Esquinas de Ticuantepe, camino entre Santo Domingo y Las Nubes, 7 Nov 1980, *Moreno, P.P. 4397* (MEXU, MO); Finca El Convento, Km 20 Carretera Panamericana Sur, 20 Jan 1981, *Sandino, J.C. 317* (MO); Finca La UCA, Km 22.5 Carretera a León, 8 Jul 1981, *Sandino, J.C. 875* (MEXU, MO); Ca. 2.3km from Hwy 12 on road along ridge of Sierra de Managua from Hwy 12 (Carretera vieja a León) at Km17 to Hwy 2 (carretera Sur), 20 Aug 1977, *Stevens, W.D. 3430* (BM, MEXU, MO); Near intersection of Hwy 28 (Carretera nueva a León) and road up N shore of Peninsula de Chiltepe, 27 Aug 1977, *Stevens, W.D. 3571* (BM, MEXU, MO, QCA); km 11 on Hwy 2 (Carretera Sur), 2km SSW of Hwy 12 (Carretera vieja a Leon) intersection, 23 Oct 1977, *Stevens, W.D. 4773* (BM, MO, QCA); Campus of Universidad Centroamericana, Managua; weeds and cultivated plants, 30 Jun 1979, *Stevens, W.D. 13305* (MEXU, MO); alogn Hwy. 8 ca. 2.4 km SW of intersection with Hwy 2, km 28, 18 Sep 1977, *Without Collector 3969* (MEXU). **Masaya**: Quebrada Noreste de Masatepe, 17 Jul 1982, *Lara, M. 161* (MO); Laguna de Apoyo, 20 Sep 1981, *Moreno, P.P. 11139* (MO); Parque nacional Volcán Masaya, small peninsula on W shore of Laguna de Masaya, ca. 3.9km S of Hwy 4, 29 Sep 1977, *Stevens, W.D. 4300* (BM, MO, QCA). **Matagalpa**: Santa María de Ostuma, 19 Jan 1980, *Araquistain, M. & Moreno, P.P. 686* (MEXU, QCA); carretera al Tuma, Hcda. La Bonanza, 25 km NE de la ciudad de Matagalpa, 8 Sep 1980, *Guzmán, M. et al. 756* (MEXU); Carretera Matagalpa-Jinotega, en el camino hacia San Simón de Palcila, al NW de Matagalpa, 10 Sep 1980, *Guzmán, M. et al. 957* (MO); 7 km al S de la cuidad, 9 Jan 1981, *Guzmán, M. & Castro, D. 1617* (MO); camino a Aranjuez, a menos de 1 km de la carretera Matagalpa-Jinotega, lado sur, 2 Jul 1980, *Moreno, P.P. 1040* (MO); camino a Aranjuez, entre El Arenal y Santa Elena, 2 Jul 1980, *Moreno, P.P. 1096* (MO); Carretera Panamericana, de la Cuesta del Venado, 4 km al E "Las Delicia, 1 Nov 1982, *Moreno, P.P. 18337* (MO). **Nueva Segovia**: Mpio. El Jícara. Valle de Casas Viejas, 7 km al NE de El Jícaro, 2 Aug 1980, *Moreno, P.P. 1683* (MO); El Jícaro, Cerro El Pelón, al SW de Casas Viejas, 23 Dec 1981, *Moreno, P.P. 13499* (MO); El Jícaro, Río Grande, carretera a Murra, 25 Dec 1981, *Moreno, P.P. 13696* (MO); Municipio de Jalapa, Montaña Cerro de Jesús, 18 Jul 2006, *Paguaga, L.D. & Toval, N. 98* (MO); Plan Grande and Las Manos on border with Honduras, said to be on route 15 (route 1?) N of Ocotal [Seymour series], 22 Mar 1971, *Seymour, F.C. 5146* (MO); Jalapa; hillside and roadside, 29 Jun 1972, *Seymour, F.C. 5524* (BM, MEXU, MO, MO); 12 km SE of Santa María along road to Macuelizo, 18 Dec 2005, *Stevens, W.D. & Montiel, O.M. 26707* (MO); Below (S of) Cerro Mogotón, ridges around (low, 2011) camp, 16 Feb 2012, *Stevens, W.D. et al. 32871* (MO). **Río San Juan**: Municipio El Castillo, comunidad Boca de Escalera, 4 km al norte de la Las Maravillas, 28 Feb 2005, *Guzmán, R. 3064* (MO); Río San Juan, a lo largo del Río Sábalos partiendo del pueblo de Boca de Sábalos, 15 Jul 1994, *Rueda, R. et al. 2030* (MO). **Rivas**: Isla de Ometepe, 14 Sep 1983, *Moreno, P.P. 22011* (MEXU, QCA); Isla Ometepe, Volcán Concepción, cafetales La Flor, 10 Aug 1984, *Robleto, W. 1013* (MEXU); Isla Ometepe, Volcán Concepción, Cerro El Mogote de las Pilas; vegetación creciendo en la lava del volcán, 27 Oct 1984, *Robleto, W. 1412* (MO); Isla Ometepe, Volcán Concepción, Sarrem, 28 Oct 1984, *Robleto, W. 1448* (MEXU, MO); Isla de Ometepe, pantanos entre playa de Finca Santa Cruz y el Istmo de Istia|n, 17 Jul 1981, *Sandino, J.C. 1005* (MEXU, MO). **Zelaya**: 0.5 km E of San Antonio on trail to and nearly reaching San Miguel, 7 Sep 1983, *Nee, M. & Vega, S. 27876* (MO); vicinity of Waní, including Río Ulí, 22 Apr 1978, *Stevens, W.D. 7968* (BM, MEXU, MO).

PANAMA. Sin. loc., 1966, *Bristan, N. 188* (MO); sin. loc., *Duke, J.A. 8722* (MO); sin. loc., *Seemann, B.C. 1606* (BM, K). **Bocas del Toro**: Forest on hill above RR station at Milla, 7.5, 27 Jul 1971, *Croat, T.B. & Porter, D.M. 16359* (MO); Vicinity of Changuinola, 3 Aug 1976, *Croat, T.B. 38084* (MO); Station Milla 7.5 on Changuinola-Almirante Railroad, along a slender ridge to World War II

communications facility, ca. 2 km NW of ruins of U.S. Army Military barracks, 3 Aug 1976, *Croat, T.B. 38107* (MO); S slopes of Cerro Itamut, ridge above small quebrada running between Itamut and El Pyramide, 12 Mar 2006, *Knapp, S. & Monro, A.K. 9997* (BM, MO); Isla Colón, vicinity of Chiriquí Lagoon, 13 Oct 1941, *Wedel, H. von*, *2840* (MO). **Canal Area**: Road S-8, S of Fort Sherman, 5 Oct 1970, *Croat, T.B. 12513* (MO); Fort Amador, on causeway and islands, 31 Oct 1965, *Dwyer, J.D. 2027* (MO); Farfan Beach, 1 Dec 1966, *McDaniel, S. 8148* (MO); Edge of lake near Madden Dam, 18 Sep 1974, *Mori, S.A. & Kallunki, J.A. 1998* (MO); Curundu, near Tropic Survival School, 29 Nov 1965, *Tyson, E.L. & Blum, K.E. 2532* (MO); Fort San Lorenzo, 5 Mar 1966, *Tyson, E.L. & Blum, K.E. 3671* (MO); On Macapalé Island in Madden Lake, 8 Jun 1969, *Tyson, E.L. 5509* (MO). **Chiriquí**: Cerro Pate de Macho, ca. 5 mi NE of Boquete, trail leading to continental divde leading to Finca Serrano (Francisco Serrano, Pacific slope), 22 Nov 1979, *Antonio, T. s.n.* (MEXU); Vicinity of Boquete, from Boquete to 3 miles N, 12 Dec 1966, *Burch, D.G. 388* (MO); Camino entre Bambito y La Amenaza, región de Cerro Punta, 21 Apr 1969, *Correa A, M.D. 1308* (MO); Boquete, At "Monte Rey", above Boqu, 21 Jul 1971, *Croat, T.B. 15761* (MO); Bugaba, Vicinity of Las Nubes, 2.7 miles NW of Río Chiriquí Viejo, west of Cerro Punta, 27 Feb 1973, *Croat, T.B. 22408* (MO); Boquete, 10 miles above Boquete on road to Volcán Barú, 18 May 1976, *Croat, T.B. 34833* (MO); Pasture between Nueva California and Río Chiriquí Viejo, 16 Apr 1970, *D'Arcy, W.G. 4245 A* (MO); Nueva California, 16 Apr 1970, *D'Arcy, W.G. 4251* (MO); Nueva California, 16 Apr 1970, *D'Arcy, W.G. 4253 B* (MO); Between Concepión and El Hato de Volcán, 16 Apr 1970, *D'Arcy, W.G. 4261* (MO); Los Naranjos, 17 Apr 1970, *D'Arcy, W.G. 4267* (MO); Los Naranjos, 17 Apr 1970, *D'Arcy, W.G. 4271* (MO); Pasture weed near Río San Juan, 19 Apr 1970, *D'Arcy, W.G. 4290* (MO); Above Nueva Suisa, Volcán District, 8 May 1971, *D'Arcy, W.G. 5328* (MO); In quarry. 6 mi. south of Hato de Volcan, 11 May 1971, *D'Arcy, W.G. 5438* (MO); Above Boquete, 12 May 1971, *D'Arcy, W.G. 5448* (MO); Above Boquete, 12 May 1971, *D'Arcy, W.G. 5461* (MO); 5 Aug 1972, *D'Arcy, W.G. & D'Arcy, J.J. 6367* (CORD, G, K, P); Along fence bordering cafetal ¼mile W of El Hato de Volcán, 9 Aug 1972, *D'Arcy, W.G. & D'Arcy, J.J. 6585* (BM, CORD, F, G, K, P); Cafetales just above El Cantinero (Boquete), 21 Nov 1975, *D'Arcy, W.G. 9818* (MO); Boquete, Potrero Muleto to Summit of El Barú. Roadside, 27 Nov 1975, *D'Arcy, W.G. 10146* (CORD, MO); Volcán Barú, just below summit, El Baru, 20 Mar 1977, *D'Arcy, W.G. 11035* (CORD); Between Palo Alto and top of ridge (divide) near Cerro Pate Macho, above Río Pao Alto, NE of Boquete, 18 Mar 1979, *D'Arcy, W.G. et al. 12631* (MO); Alto Bambito, 8 Apr 1979, *D'Arcy, W.G. et al. 13116* (MO); Falda del Baru above Alto Pineda, road behind town of Cerro Punta, 9 Apr 1979, *D'Arcy, W.G. 13201* (NY); Boquete, Bajo Chorro, 22 Jan 1938, *Davidson, M.E. 183* (F, MO); Boquete, Llanos Francia, 4 miles from Boquete toward Dolega. Llanos bordering creek, 6 Aug 1967, *Dwyer, J.D. & Hayden, M.V. 7596* (MO); Cerro Punta, above town, 16 Mar 1977, *Folsom, J.P. et al. 2061* (MEXU); Road from Volcán to Río Sereno; 16.0 km from Río Sereno, 29 Jun 1977, *Folsom, J.P. 4046* (MEXU, MO); Cerro Colorado, area surrounding the stream that flows by Escopeta, 17 Aug 1977, *Folsom, J.P. 4908* (MO); Volcán Barú (E slope) deep draw west of Finca Yen, 17 Mar 1979, *Hammel, B. et al. 6465* (MO); In forest remnant and old fields, Alto Pineda, end of road, right turn just before cooperativa entrance to Cerro Punta, 11 Apr 1979, *Hammel, B. et al. 6981* (MO); Ca. 0.5 km E of Cerro Pate Macho, headwaters of Río Palo Alto, 12 Nov 1981, *Knapp, S. et al. 2124* (MO); Cerro Colorado, 50 km N of San Félix on the continental divide, 17 Aug 1975, *Mori, S.A. & Dressler, R.L. 7793* (MO); Pumice flats 4 km NE of El Hato del Volcán along road to Cerro Punta, 4 Jan 1975, *Nee, M. 14132* (MO); Camino de acceso al Parque Nacional Volcán Barú (vertiente oriental), 15 Nov 1991, *Vega, M. & Vargas, L. 217* (MO); Puerto Armuelles, 2 Nov 1973, *Viquez, M. 46* (MO); Boquete, Potrero Muleto to summit, Volcán de Chiriquí, 13 Jul 1940, *Woodson, R.E. & Schery, R.W. 464* (MO); Vicinity of Bajo Chorro, 20 Jul 1940, *Woodson, R.E. & Schery, R.W. 635* (MO). **Coclé**: La Mesa above El Valle; along road which ends in pasture, 21 Jul 1974, *Croat, T.B. 25294* (MO); Near summit of El Petroso, ca. 7 km north of El Copé, 9 Apr 1977, *D'Arcy, W.G. 11266* (CORD, MO); 12 miles NE of Penonomé. Granite river gorge, 24 May 1967, *Dwyer, J.D. 1510* (MO); Roadside and fields near Mata Ahogado (behind Hotel Campestre, El Valle), 27 Sep 1978, *Hammel, B. 4802* (MO); 12 miles NE of Penonomé, 24 May 1967, *Lewis, W.H. et al. 1510* (MO); Foot of Cerro Pilón, above El Valle de Antón, 28 Mar 1969, *Porter, D.M. et al. 4611* (MO). **Colón**: Portobelo, 2 miles south of Portobelo, along river, 17 Jul 1970, *Croat, T.B. 11415* (MO); Santa Rita lumber road, 15 km E of Colón. Forest, 5 Oct 1969, *Dressler, R.L. & Lewis, W.H. 3735* (MO); Mouth of Río Piedras, Beach and adjacent area, 11 Dec 1967, *Lewis,*

W.H. et al. 3204 (MO); Alrededores de Gatuncillo, 10 Mar 1971, *Rosas, ?, 26* (MO). **Darién**: 111 miles from Bayano Dam Bridge. Vicinity of Canglon, 14 May 1980, *Antonio, T.M. 4530* (MO); Vicinity of airstrip at Cana gold mine, 29 Jul 1976, *Croat, T.B. 37983* (MO); Weeds of Choco village, Piji Vasal. In and around village; sea level, 24 Jul 1977, *Folsom, J.P. 4581* (CORD, MO); Path from Canazas to Tortí, border Darién and Panamá, 26 Aug 1977, *Folsom, J.P. 4966* (MEXU, MO); between Río Jesus and Sabado, 4 Feb 1978, *Hammel, B. 1355* (MEXU, MO); Quebrada "Camachimuricate" cerca casa de Barto, 15 Mar 1973, *Kennedy, H.A. 2839* (MO); Manene to the mouth of the Río Cuasí, 28 Apr 1968, *Kirkbride Jr, J.H. & Bristan, N. 1398* (MO); Trail from Canglón-Yaviza road to Río Chucaraque, 7.7 miles E of Canglón. [Coord on original label: 08.20N 077.50W], 6 Mar 1982, *Knapp, S. & Mallet, J. 3951* (MEXU, MO); Upper Río Tuquesa, Jan 1973, *Le Clezio, ?, 217* (MO); gold mine at Cana, 26 Jul 1976, *Sullivan, G. 621* (MEXU); Gold mine at Cana, 26 Jul 1976, *Sullivan, G.A. 621* (MO); 1 mi N of Río Sabana, 16 Jul 1966, *Tyson, E.L. et al. 4776* (MO). **Guna Yala (San Blas)**: Puerto Obaldia, Trail from Puerto Obaldía to La Bonga, tributary of the Río Armila, ca. 2 hours walk from Puerto Obaldía [Coordinates on original label: 08.39.59N 077.24.59W], 17 Apr 1982, *Knapp, S. & Mallet, J. 4655* (MO); Mainland opposite Achituppu [Achutupo], from beach to 1.5 miles inland, 7 Dec 1966, *Lewis, W.H. et al. 93* (MO); Cangandí: [Coordinates on original label: 9º24'N, 79º24'W. Hills near village. Assoc: Attalea allenii, Capparis, 27 Mar 1986, *Nevers, G.C. de*, *et al. 7484* (MEXU, MO). **Herrera**: Las Minas, Road to 9 mi SW of Las Minas, 4 Nov 1979, *D'Arcy, W.G. & Antonio, T.M. 13486* (MO); Las Minas, 18 km W of Las Minas, trail to top of Alto Higo. N slope of Cerro Alto Higo (known locally as el Montoso [Montuoso]), 5 Aug 1978, *Hammel, B. 4242* (MO); Las Minas, 18 km W of Las Minas, N slope of Alto Higo. [originally reported from Veraguas province], 8 Aug 1978, *Hammel, B. 4340* (MO); Santa Maria, Comunidad de el Rincón. Orilla de la Ciénaga La Macana, 21 Jul 1996, *Montenegro, E. 1439* (MO). **Los Santos**: Tonosi, Along Río Guaniquito above El Cortezo (8.2 miles from Highway 50 turnoff), 25 Oct 1978, *Hammel, B. 5303* (MO); Tonosi, Vicinity of headwaters of Río Pedregal, 25 miles SW of Tonosí. Cloud forest and native clearing, 7 Dec 1967, *Lewis, W.H. et al. 2955* (MO); Tonosi, Vicinity of headwaters of Río Pedregal, 25 miles SW of Tonosí, 7 Dec 1967, *Lewis, W.H. et al. 2978* (MO). **Panamá**: Chepo, Chepo; habita en lugares húmedos en conjunto con una gran cantidad de arbustos, 2 Nov 1973, *Chávez, M. 10* (MO); Panama, Region of Cerro Jefé, 3 Oct 1969, *Correa A, M.D. et al. 1614* (MO); Panama, Cerro Azul, 23 Jun 1972, *Croat, T.B. 17324* (MO); La Chorrera, La Chorrera, 5 May 1971, *D'Arcy, W.G. 5257* (MO); Arraijan, Near Arraiján, 12 Sep 1976, *D'Arcy, W.G. 10663* (MO); Panama, Weeds of Río Pacora (flooded Nov. 4, 1966) just below confluence with Río Corso, 9 Jun 1967, *Duke, J.A. 12022* (MO); new dirt road N of El Llano, 25 Jul 1972, *Edmonds, J.M. C 101* (K); Chepo, Bayano. Guipo forest; 9.5 kM E of Bayano Bridge, 7 Jun 1977, *Folsom, J.P. 3514* (MEXU, MO); Chepo, 4-5 hours walk upriver from Torti Arriba, 6 Dec 1977, *Folsom, J.P. et al. 6850* (MEXU, MO); Arraijan, Cerro Cabra, small cutover hill just W of Canal Zone on the coast-Veracruz, 30 May 1978, *Hammel, B. 3285* (MO); Panama, Monte detrás del auto cine Olímpico [actualmente donde se encuentra la Plaza Carolina], 7 Oct 1974, *Him, C. 32* (MO). **Veraguas**: Montijo, Isla de Coiba. Along road to the hot spring from main camp, 20 Oct 1979, *Antonio, T.M. 2209* (MO); Santa Fe, 5 miles W of Santa Fé on road past Escuela Agricola Alto Piedra on Pacific side of divide, 18 Mar 1973, *Croat, T.B. 23035* (MO); Santa Fe, Above Santa Fe beyond Escuela Agricola Interamericana, 1.8 miles beyond fork in road on Pacific slope, 5 Apr 1976, *Croat, T.B. & Folsom, J.P. 34188* (MO); Santiago, Weedy roadside just W of Santiago, 6 May 1971, *D'Arcy, W.G. 5272* (MO); Santa Fe, SW side of Cerro Tute, "La Cuchilla", 10 Sep 1982, *D'Arcy, W.G. 15025* (MO); El Cuchillo, near Cerro Tute, up from Santa Fe, 8 Sep 1982, *Hamilton, C. et al. 1231* (MO); Santa Fe, along road to agriculture school, 3-4 km by road W of Santa Fé, 13 Apr 1974, *Nee, M. 11323* (MO).

SAINT KITTS AND NEVIS. **Saint Kitts**: Old Road river valley, 28 Jul 1937, *Sandwith, N.Y. 970* (K).

SAINT VINCENT AND THE GRENADINES. **Saint Vincent**: Gourmand. Cumberland Valley. Rain forest clearings, 29 Nov 1945, *Beard, P. 1383* (F, K, MO).

TRINIDAD AND TOBAGO. **Tobago**: Roxborough-Bloody Bay Road, 12 Aug 1958, *Purseglove, J.W. P-6309* (K); Charlotteville, Flagstaff Hill picnic area above southeast of Charlotteville, 26 Dec 2008, *Stern, S. & Tepe, E.J. 264* (BM, MO, UT). **Trinidad**: Moruga, 19 Mar 1921, *Britton, N.L. & Broadway, W.E. 2446* (US); Woodbrook, Nov 1925, *Broadway, W.E. 6307* (K); Cedros, 5 Feb 1902, *Crueger, H. s.n.* (K); Maracas Bay, 31 Jul 1949, *Simmonds, N.W. 14404* (K); B. de Tchach [Schach? -= collector?], 1824, *Without Collector s.n.* (K).

TURKS AND CAICOS ISLANDS. Grand Turk Island, the Ponds and vicinity, 20 Feb 1911, *Millspaugh, C.F. & Millspaugh, C.M. 9381* (F). **East Caicos**: Scrub around airport runway, 23 Apr 1971, *Burch, D. 4309* (F, MO).

UNITED STATES OF AMERICA. **Alabama**: Baldwin County, N of Spanish Fort by Ala 225, 5.2 mi N US 31, 22 Sep 1969, *Kral, R. 37434* (MO); Mobile County, Mobile, 4 Jul 1880, *Mohr, C. s.n.* (US); Mobile County, Mobile, 1 Oct 1896, *Mohr, C. s.n.* (US); Mobile County, Pinto Island, Mobile River, *Mohr, C. s.n.* (US). **Florida**: Volusia County, New Smyrna, East Coast, 29 Mar 1904, *Burgess, A.B. 585* (F); southern Florida, *Chapman, A.W. s.n.* (MO, NY); sin. loc, *Chapman, A.W. s.n.* (NY); Alachua County, behind McCarty Hall, University of Florida, Gainesville, 5 Jan 1967, *D'Arcy, W.G. s.n.* (MO); Alachua County, at bottom of Devil's Millhopper, 11 May 1967, *D'Arcy, W.G. 1641* (MO, NY); Gadsden County, US90, ca. 4 mi W of Quincy, 7 Jun 1968, *D'Arcy, W.G. 2574* (MO); Leon County, 2 miles W of Tallahassee, 10 Jul 1955, *Godfrey, R.K. 53610* (GH, NY); sin. loc, *Herb Ag Dept*, *s.n.* (NY); Hillsborough County, Tampa, Northgate, 22 Mar 1968, *Lakela, O.K. & Almeda, F. 31345* (GH, MEXU); sin. loc, *Le Conte, J.E. s.n.* (NY); Hillsborough County, Egmont Key, 8 Apr 1969, *Long, R.W. et al. 2883* (MO); Volusia County, Port Orange, Willow Run Drive just SE of Ana Maria Circle, near I-95, 24 Dec 1999, *Nelson, J.B. & Wnek, R.H. 20927* (NY); Lake County, sin. loc, *Piekel, F.U. 719* (F); Liberty County, Torreya State Park, between Rock Bluff and Apalachicola River, along river, 23 Dec 1983, *Plowman, T.C. 13322* (F); Monroe County, Key Largo, Newport, 26 Mar 1898, *Pollard, C.L. et al. 190* (BM, NY, W); Columbia County, Lake City, 15 Jul 1915, *Quaintance, A.L. 713* (F); Seminole County, Sanford, 28 Feb 1927, *Uhler, F.M. s.n.* (US); Palm Beach County, Lake Worth, 27 Dec 1895, *Webber, H.J. 256* (F); *Without Collector s.n.* (MO). **Louisiana**: Washington Parish, Along Bogalusa-Bush Hwy about 2 miles south of Bogalusa, 11 Oct 1970, *Allen, C.M. 75* (LSU); Allen Parish, Between railroad and US 165 ca. 1.6 miles south of Kinder, 24 Oct 1981, *Allen, C.M. & Thomas, R.D. 11535* (LSU); Terrebonne Parish, Cultivated field near Dulac, 20 Jul 1937, *Arceneaux, G. 170 a* (LSU); East Baton Rouge Parish, On Lee Drive, about 1 mile south of the intersection with Perkins Road, Baton Rouge, 6 Nov 1971, *Azimi bin Haji Hamzah*, *4* (LSU); East Baton Rouge Parish, On Highland Road, 0.5 miles north of LSU campus.; Louisiana State University campus, 6 Nov 1971, *Azimi bin Haji Hamzah*, *5* (LSU); Gretna, opposite New Orleans, 1 May 1899, *Ball, C.R. 325* (F, GH, MO, NY, US); Iberville Parish, Indigo Island 1 mile south of Bayou Pigeon, growing between road and bayou, Indigo Island, 8 Apr 1979, *Barkley, L.J. 29* (LSU); East Baton Rouge Parish, 1 mile south of Ben Hur Road junction with levee road, between Mississippi River and levee, Baton Rouge, 28 Oct 1975, *Beck, L.T. 510* (LSU); East Baton Rouge Parish, East Baton Rouge, 16 Oct 1927, *Brown, C.A. 1366* (MICH); East Baton Rouge Parish, Ben Hur Woods, 0.9 miles west of LSU Ben Hur Research Farm. 2 miles SSW of LSU campus. Baton Rouge West Quad.; Louisiana State University Agricultural Center, Central Research Station, 19 Nov 1992, *Burckhalter, R. & Tucker, S.C. 2149* (LSU); St. Mary Parish, Franklin, 6 Mar 1933, *Bynum, ?*, *et al. 91* (LSU); Lafayette Parish, sin. loc, 15 Jun 1942, *Claycomb, G.B. s.n.* (LSU); East Baton Rouge Parish, LSU campus, southwest behind "judging pavillion", Louisiana State University campus, 27 Sep 1995, *Corkern, C.B. 26* (LSU); St. Bernard Parish, Stake Islands, in South Chandeleur Islands in Chandeleur Sound and Gulf of Mexico NE of Venice, 11 Jul 1984, *Dale Thomas, R. et al. 89695* (UT); sin. loc, *Dr Hale*, *s.n.* (NY); Orleans Parish, New Orleans, batture behind Audubon Park, 5 Dec 1949, *Ewan, J. 18277* (GH, UC); Plaquemines Parish, 2 miles N of Magnolia, 15 Apr 1960, *Ewan, J. 19917* (GH, UC); Plaquemines Parish, upriver from Buras, 23 Dec 1948, *Ewan, J.A. 18001* (GH); Orleans Parish, New Orleans, on batture behind Audubon Park, 23 Oct 1949, *Ewan, J.A. 18260* (GH); Saint James Parish, site of Valcouer Aime plantation near Vacherie, 4 Oct 1959, *Ewan, J.A. 19853* (GH); East Baton Rouge Parish, Baton Rouge, *Featherman, A. s.n.* (LSU); East Baton Rouge Parish, Mississippi River levee near the LSU Vet School, intersection of

River Road and Skip Bertman Drive, Louisiana State University Veterinary School, 25 Oct 2001, *Ferguson, D.M. 474* (LSU); Assumption Parish, Junction of LA 662 and US 90, 31 Oct 1981, *Givens, F.M. & Thomas, R.D. 2294* (LSU); Terrebonne Parish, Behind low dune area at east end of Timbalier Island, 26 Oct 1988, *Givens, F.M. & Hester, M.W. 5537* (LSU); St. Bernard Parish, Fencerow along western boundary of Unit, including the mowed picnic area, adjacent fencerow, and Visitor Center. Jean Lafitte National Park and Preserve, Chalmette Battlefield Unit; Jean Lafitte National Park and Preserve, Chalmette Battlefield Unit, 8 Apr 2005, *Gunn, S.M. & Ferguson, D.M. CH-45* (LSU); Ascension Parish, Along the Amite River, next to Fred's Bar and Grill off Hwy 42, Amite River, 9 Nov 2002, *Howard, J. 52* (LSU); Metairie Swamp, 2 Apr 1889, *Joor, J.F. s.n.* (MO); New Orleans, Dec, *Joor, J.F. s.n.* (MO); Livingston Parish, Near Holden Wood Products Co. on north side of US 190, across railroad tracks 2 miles east of Holden, 29 Sep 1984, *Joye, G.F. & Rebertus, A.J. 219* (LSU); St. Martinville, 30 May 1893, *Langlois, A.B. s.n.* (MO); St. Martin Parish, St. Martinsville, 17 Oct 1832, *Langlois, A.B. s.n.* (US); sin. loc, *Langlois, A.B. 239* (NY); St. Bernard Parish, Shell Beach, 2 Nov 1958, *Lemaire, R.J. 850* (US); Ascension Parish, Along a railroad track near the junction of LA 70 and LA 3089, near Donaldsonville, 23 Aug 1987, *Lievens, A.W. 2818* (LSU); East Baton Rouge Parish, Near a ditch on the grounds of Magnolia Mound Plantation House (2161 Nicholson Drive) in Baton Rouge, 1 Oct 1990, *Lievens, A.W. 4521* (LSU); Lafourche Parish, off Highway 77, 12 miles S of Raceland, 2 Apr 1955, *Lowe, E. 24* (GH); East Baton Rouge Parish, Iberville Terrace Pond, 6.5 miles SE of gates of LSU on LA 42 and 1 mi N, Baton Rouge, 28 Sep 1975, *Luckner, C. 562* (LSU); Assumption Parish, Paincourtville, Jul 1917, *Marionneaux, E. s.n.* (LSU); Iberville Parish, 0.5 miles N of White Castle on Hwy 1, 16 Oct 1973, *Miller, T.C. 57 A* (LSU, MO); St. Charles Parish, La Branch, Indian campsite east of Bayou La Branche, shore of lake, 21 Sep 1971, *Montz, G.N. 2085* (LSU); St. Charles Parish, Bonnet Carre Spillway, center of floodway under Airline Hwy Bridge, 2 Nov 1973, *Montz, G.N. 2947* (LSU); St. Charles Parish, Bonnet Carre Spillway. Near cattle pens, near River Road and east guide levee.; Bonnet Carre Spillway, 10 Nov 1973, *Montz, G.N. 2961* (LSU); St. Charles Parish, Along fence around baseball field, Murrayhill Dr. Hill Heights Country Club, Destrehan, 20 Sep 1994, *Montz, G.N. 6993* (LSU); Tangipahoa Parish, Southeastern Louisiana University campus, at North Campus Recreational Complex, Hammond.; Southeastern/SLU campus, 25 Oct 1996, *Montz, G.N. & Montz, J. 8410* (LSU); Tangipahoa Parish, Southeastern Louisiana University campus, north of baseball field, Hammond, Southeastern/SLU campus, 8 Nov 1996, *Montz, G.N. 8429* (LSU); St. Charles Parish, Along fenced pool at Hill Heights Country Club, Murrayhill Dr. Destrehan, 17 Nov 1997, *Montz, G.N. 8884* (LSU); Lincoln Parish, Ruston, Louisiana Tech Farm, 29 Jun 1949, *Moore, J.A. s.n.* (UC); Lafourche Parish, Along NE side of Bayou Cut Off, 6 miles SE of THIBODAUX, 19 Oct 1981, *Nee, M. & Lasseigne, A. 22165* (BM, F); Jefferson Davis Parish, Welsh, 17 May 1915, *Palmer, E.J. 7658* (K, MO, US); Natchitoches, 2 Oct 1915, *Palmer, E.J. 8776* (MO); St. Tammany Parish, Oak Manor Subdivision off of Hwy 190 W. ca. 1.5 miles west of Airport Rd. in Slidell. Growing in yard of 109 Pin Oak Drive, Slidell, 18 Apr 1992, *Parnell, C. 45* (LSU); East Baton Rouge Parish, Baton Rouge, Apr 1904, *Phillips, W.D. s.n.* (LSU); East Baton Rouge Parish, LSU campus, along the Corporation Canal, Louisiana State University campus, 14 Apr 1988, *Qiu, H.-L. 50* (LSU); Vermillion Parish, Redfish Point, USl Field Station, W side of Vermillion Bay, 22 Oct 1960, *Reese, W.D. 3917* (GH); Plaquemines Parish, Pass A Loutre WMA, main campground on E side of South Pass ca 3 mi below Head of Passes, Pass a Loutre Refuge, 6 Sep 2006, *Reid, C. & Baker, T. 5884* (LSU); Plaquemines Parish, Pass A Loutre WMA, main campground on E side of South Pass ca 3 mi below Head of Passes, Pass a Loutre Refuge, 6 Sep 2006, *Reid, C. & Baker, T. 5884* (LSU); St. Charles Parish, Along the road of US 61, 2 miles N of Good Hope, 17 Nov 1973, *Salcedo, J. s.n.* (LSU); Orleans Parish, New Orleans, 23 Jun 1844, *Sallé, C. s.n.* (BM, W); Saint Charles Parish, sin. loc, 3 Apr 1913, *Sargent, C.S. s.n.* (A); Lafourche Parish, Field located across from the address of 306 Melanie Lane across a creek and into the field, Houma, 22 Nov 1990, *Savoie, T. 31* (LSU); St. Martin Parish, Cypress Island Preserve. In Cable Corral tract between the south gate and the restoration site, 28 Sep 1997, *Scarce, S. 281* (LSU); Lafourche Parish, Rt 1 below Leesville, 13 Oct 1978, *Svenson, J.E. 217* (NY); Lafourche Parish, Rt. 1 below Leesville, 13 Oct 1978, *Swenson, J.E. s.n.* (BM); Lafourche Parish, Less than 1 mile west of US 90 on LA 3199, 4 Aug 1991, *Taylor, P. PT 105 P* (LSU); Acadia Parish, bside La. 13 at bayou Quere de la Tortue at Vermilion Parish line south of Crowley [sec 21, T11S, R1E], 1 Nov 1981, *Thomas, J.D. 80103* (MEXU); Ouachita Parish, beside Ben Wink Road southeast of Lapine Road and about 1.5 miles northeast of Jackson Parish [Sec

30, T16N, R2E], 12 Nov 1990, *Thomas, J.D. 122716* (MEXU); Vermillion Parish, roadbank of La 82 in marsh 4.6 miles west of Pecan Island School, Sec. 31, T15S, R1W, 2 Nov 1990, *Thomas, R.D. 122411* (NY); Beauregard Parish, Beauregard parish; beside N-to-S railroad tracks south of U.S. 190 in downtown DeRidder; Secd. 32, T2S, R9W, 7 Dec 1996, *Thomas, R.D. 153474* (MO, NY); St. Tammany, Northeast shore of Lake Ponchartrain at Goose Point west of Bayou Lacombe southwest of Lacombe; Secs. 10 & 5, T9S, R12E, 16 Oct 1999, *Thomas, R.D. & Moreland, D. 164070* (MO, NY); Cameron Parish, Rockefeller Wildlife Refuge, 10 Sep 1985, *Thompson, S.A. & Nishida, J.H. 3079* (NY); Plaquemines Parish, Port Eads, 22 Aug 1900, *Tracy, S.M. & Lloyd, ? 52 a* (E, F, GH, US, W); Orleans Parish, New Orleans, *Tucker, H. s.n.* (NY); Terrebonne Parish, Carrion Crow Bayou hunting area, Louisiana Land and Exploration Co. property, 10 May 1976, *Urbatsch, L.E. & Carney, D.F. 2480* (LSU); Tangipahoa Parish, North of SLC campus along ditch, Hammond.; Southeastern/SLU campus, 13 Jun 1957, *Wilson, H.R. 265* (LSU); Terrebonne Parish, [Probably Terrebonne Parish], 19 May 1912, *Wurzlow, E.C. s.n.* (LSU). **Mississippi**: Harrison Parish, Gulf Islands National Seashore, East Ship Island: Extensive marsh system bordered on the south by a ridge of higher elevation dunes, East Ship Island, Gulf Islands National Seashore, 6 Jun 2006, *Gunn, S.M. & Ferguson, D.M. ESI-71* (LSU); Harrison Parish, Gulf Islands National Seashore, West Ship Island. Typha marsh east of boardwalk and southeast of Ft. Massachusetts greatly damaged from over wash from Hurricane Katrina, West Ship Island, Gulf Islands National Seashore, 27 Apr 2006, *Gunn, S.M. & Ferguson, D.M. WSI-74* (LSU); Jackson County, Gulf Islands National Seashore, Davis Bayou. Bayhead swamp just east of picnic area along edges of Stark Bayou.; Davis Bayou, Gulf Islands National Seashore, 24 May 2006, *Gunn-Zumo, S.M. & Ferguson, D.M. DB-415* (LSU); Pearl River County, Picayune, Crosby property to E of US11, SE4 S22, near Friendship Park, 15 May 1981, *McDaniel, S. 25060* (MO); Forrest County, Hattiesburg, from Shemper Steel to RR depot, 8 Aug 1974, *Rogers, K.E. 6884 A* (GH); Forrest County, ca. 1/2 mile S of Maxie on old Highway 49, 17 Oct 1971, *Rogers, K.E. 7088* (GH, NY); Harrison County, Biloxi, 22 May 1898, *Tracy, S.M. 5153* (F, IND, MO, NY, US). **North Carolina**: New Hanover County, along RR, Nutt St. Port of Wilmington, 17 Oct 1979, *Reed, C.F. 104090* (MO); New Hanover County, RR, Nutt St. Port of Wilmington, 17 Oct 1979, *Reed, C.F. 104092* (MO). **Texas**: Newton County, Caney Creek Park south of US 190 in Newton; Newton, 17 Oct 2008, *Allen, C.M. & Allen, S. 21032* (LSU); Newton County, Off CR 4076 ca. 1 mile south of Bon Wier, 16 Jul 2011, *Allen, C.M. et al. 22347* (TEX); sin. loc, *Bach, W.J. s.n.* (US); Jackson County, in Menehee Flats South of Vanderbilt, 26 Nov 1943, *Barkley, F.A. 13A 162* (K, MO, UC); Galveston County, Galveston, 19 May 1903, *Bebb, R. 1172* (F); *Berlandier, J.L. 524* (MO, NY); "Herbarium Texano-Mexicanum" [could also be Mexico], *Berlandier, J.L. 892* (NY); Bexar County, Circa Bejar, *Berlandier, J.L. 1904* (G-DC, MO, NY, P, W); Travis County, Austin, 29 Aug 1903, *Biltmore Herbarium*, *912 a* (US); Harris County, Houston, 28 May 1903, *Biltmore Herbarium*, *912 g* (US); Gonzales County, Ottine, 27 Mar 1927, *Bogusch, E.R. s.n.* (TEX); Travis County, Austin, Colo R, Deep Eddy, 30 Apr 1937, *Botany 23*, *s.n.* (UC); Travis County, Austin, Colo. R, Deep Eddy [Pool], 30 Apr 1936, *Botany-23*, *256* (MO, NY); Medina County, Ney Bat Cave, in NE part of county, 14 miles S of Bandera, 15 Jun 1950, *Bryant, P.T. & Bryant, G. 32* (UC); Travis County, Austin, 1883, *Buckley, S.B. s.n.* (NY); Pecos County, sin. loc, 1895, *Buckley, S.B. s.n.* (NY); Brazoria County, Columbia, Plants of Brazos River, 14 Apr 1899, *Bush, B.F. 156* (MO); Bexar County, San Antonio, Jevas, 2 Oct 1900, *Bush, B.F. 1213* (MO); Brazoria County, Columbia, 5 Oct 1900, *Bush, B.F. 1326* (MO); Starr County, US Highway 83, 5 miles N of Rio Grande City, 19 Mar 1963, *Cabrera, P. 83* (TEX); Kleberg County, E of San Fernando Creek, in crossing of gas pipeline near end of jeep trail leading S from gate near Vinatero Well, Pinto Pasture, King Ranch Training Area (Texas National Guard). Ricardo Quad. Elev. 0 -1 ft, 12 Apr 1993, *Carr, W.R. 12605* (TEX); San Patricio County, S edge of northern perimeter road, ca. 50 ft. S of FM 1069 ca. 0.4-0.45 miles W of FM 2725, SE of Ingleside. Naval Station Ingleside. Port Ingleside Quadrangle. Elev. ca. 20 ft, 24 May 1996, *Carr, W.R. & Brown, D.A. 15415* (TEX); Bexar County, Ca. 2200 ft. SSE of Military Dr. bridge. San Antonio Missions NHP; E end of Old Espada Dam, on old channel of San Antonio River in Espada Park, W side of new channel of San Antonio River. [Flora of San Antonio Missions NHP.]. Elev. 530 ft, 7 Aug 2002, *Carr, W.R. 21196* (TEX); San Patricio County, Along S side of US 77 at NW corner of McGregor Ranch, ca. 3.8 airmiles SW to SSW of Jct. US 77 and FM 234 at Odem. Odem Quadrangle. Elev. 10 ft, 18 Feb 2003, *Carr, W.R. 21673* (TEX); Willacy County, La Sal Vieja, ca. 5.9 airmiles SE of jct. of Willacy, Hidalgo and Kenedy counties, or 5.3-5.4 arimiles NE to NNE of jct. St.

Rt. 186 and St. Rt. 88, on Corbett Ranch. La Sal Vieja Quadrangle. Elev. 30 -35 ft, 2 Apr 2003, *Carr, W.R. et al. 21912* (TEX); Goliad County, On S side of San Antonio River, on W side of gully formed by minor tributary, ca. 1.6 airmiles W to WNW of jct. US Rt. 183 and F. M. 2441 S of Goliad, at N28o38'27.6", W097o24'30.3". Goliad Quadrangle. Elev. 130 ft, 26 Mar 2004, *Carr, W.R. & Flowers, D. 22880* (TEX); Jim Wells County, E side of N-S runway at Naval Auxiliary Landing Field Orange Grove, N of El Caro Creek, ca. 2.7-2.8 airmiles E of jct. US Rt. 281 and Co. Rd. 220, at N27o53'17.9", W098o02'39.5". San Diego NE Quadrangle. Elev. 210 ft, 5 May 2006, *Carr, W.R. & Benesh, D. 24478* (TEX); Bexar County, tropical life zone, 1 Jun 1911, *Clemens, J. 265* (MO); Hidalgo County, Alamo, along the Rio Grande, 15 Apr 1933, *Clover, E.U. 917* (NY); Presidio County, At Scheele's Goat Ranch on W side of Chinati Mountains, 10 Sep 1961, *Correll, D.S. & Johnston, M.C. 24399* (LL); Marion County, Jims Bayou, about 5 miles N of Smithland, 18 Oct 1962, *Correll, D.S. 26403* (GH); Jefferson County, 6.5 miles N of Sabine Pass, marsh area, 10 May 1965, *Correll, D.S. & Crutchfield, J. 30999* (LL, MO, NY); Cameron County, Cardin settlement, about 3 miles from Brownsville, along Rte. 4 to Boca Chica, 9 Feb 1969, *Correll, D.S. 36773* (GH, UC); Edwards County, 22 miles S of Rocksprings, W.H. East Ranch, 6 Jun 1930, *Cory, V.L. 3452* (GH); Zavalla County, 11.5 miles S of Uvalde, Nueces River, 24 Oct 1934, *Cory, V.L. 11955* (GH); Kerr County, 5.8 miles S of Mountain Home, 26 Aug 1936, *Cory, V.L. 19358* (GH); Cameron County, S side of Resaca del Rancho Viejo, 6 airline miles north east of Brownsville, 30 Nov 1945, *Cory, V.L. 51403* (GH, NY, TEX); Jefferson County, Beaumont, 28 Dec 1942, *Crockett, R.L. 1289* (LL); Cameron County, Southmost Cameron County, 1941, *Davis, A.M. s.n.* (GH, TEX); Victoria County, N Victoria, 7 Apr 1900, *Eggert, H. s.n.* (MO); Victoria County, N Victoria, 7 Apr 1900, *Eggert, H. s.n.* (MO); Comal County, Landa Park, 25 Feb 1934, *Ferguson, M.C. & Ottley, A.M. 4123* (UC); Comal County, New Braunfels, Landa Park, 25 Feb 1934, *Ferguson, M.C. et al. 4123 b* (UC); Hidalgo County, Pharr, 3 Mar 1934, *Ferguson, M.C. & Ottley, A.M. 4189* (UC); Cameron County, vicinity of Brownsville, 1 Aug 1921, *Ferris, R.S. & Duncan, C.D. 3122* (NY); Cameron County, vicinity of Brownsville, 1 Aug 1921, *Ferris, R.S. & Duncan, C.D. 3132* (MO); Bexar County, Leon Creek bank, Somerset Road, 9 mi. S of San Anton, 13 Nov 1942, *Freeborn, R. & Freeborn, B. 267* (NY); Polk County, Indian res, 21 Nov 1940, *Girvin, W. s.n.* (GH); near San Antonio, 3 Sep 1848, *Gregg, J. 425* (MO); Brazos River near Horse, 5 Dec 1918, *Hanson, H.C. s.n.* (NY); Cameron County, Brownsville, 10 miles SE, 17 May 1919, *Hanson, H.C. 469* (US); Cameron County, Brownsville, along Rio Grande, 17 May 1919, *Hanson, H.C. 488* (NY); Presidio County, Presidio, [somewhat illegible], 9 Jun 1881, *Havard, V. s.n.* (US); Hays County, San Marcos, 24 Apr 1909, *Heald, F.D. & Wolf, F.A. s.n.* (TEX); Presidio County, Sierra Tierra Vieja, Lower Musgrave Canyon, 9 Jul 1941, *Hinckley, L.C. 1923* (NY, US); Presidio County, Sierra Tierra Vieja, Knox Canyon about 1/2 mile up, 20 Jul 1941, *Hinckley, L.C. 2041* (NY); Presidio County, Sierra Tierra Vieja, Musgrave Canyon, 18 Oct 1941, *Hinckley, L.C. 2208* (NY); Presidio County, Cibolo Creek near Cieneguita 10 miles NW of Shafter, 10 Nov 1946, *Hinckley, L.C. & Warnock, B.H. 46819* (GH, UC); Presidio County, Cibolo Creek near Cienguita, 10 miles N of Shafter, 10 Nov 1948, *Hinckley, L.C. & Warnock, B.H. 48819* (K, MA, MO); Bexar County, *Jermy, G. s.n.* (MO); Bexar County, *Jermy, G. s.n.* (MO); Bexar County, *Jermy, G. s.n.* (MO); Bexar County, sin. loc, 1904, *Jermy, G. s.n.* (NY); Bexar County, San Antonio, *Jermy, G. s.n.* (NY); Bexar County, San Antonio, *Jermy, G. 79* (MO, US); Nueces County, In Agua Dulce Creek bottom near hwy 44. Elev. 86.5 ft, 29 Nov 1954, *Johnston, M.C. 542316* (TEX); Live Oak County, 2 mi NE of George West, along Nueces River, 30 Mar 1967, *Koch, R.G. 2714* (UT); sin. loc, *Le Roy, P.V. s.n.* (NY); Kleberg County, Santa Gertrudis Ranch, 3 Apr 1953, *Lehman, R.L. et al. s.n.* (TEX); Bexar County, "New Braunfels", Nov 1850, *Lindheimer, F. s.n.* (GH); Bastrop County, Bastrop State Park. NW quadrant of Park, near N property line. Elev. 530 ft, 11 Aug 2003, *Lott, E.J. & Moore, S.M. 4508* (TEX); Cameron County, Palm Grove, S of Brownsville, 6 Apr 1941, *Lundell, C.L. & Lundell, A.A. 10005* (LL, MICH, US, US); Kenedy County, Kings Ranch, near Norias, 15 Mar 1942, *Lundell, C.L. & Lundell, A.A. 10729* (US); Brewster County, Chisos Mountains, upper slopes of Green Gulch, 27 Aug 1944, *Lundell, C.L. 13255* (LL); Brewster County, Chisos Mts, in the basin, near cabins, 27 Aug 1944, *Lundell, C.L. 13274* (LL, MO); Brewster County, Big Bend National Park, Boquillos, 20 Jul 1937, *Marsh, E. 137* (GH, UC); Webb County, Casa Blanca Lake, 6 mi E of Laredo, 10 Nov 1961, *Martinez, R. & Trevino, M. 76* (TEX); Bexar County, Elmendorf Lake, 7 Aug 1931, *Metz, M.C. 481* (NY); Brewster County, Green Gulch, 18 Jul 1932, *Mueller, C.H. s.n.* (MO); Brewster County, Chisos Mts, 9 Jul 1931, *Mueller, C.H. 8155*

(MICH, MO, NY, TEX, US); Cameron County, Brazos Santiago, Texas, 1889, *Nealley, G.C. 339 (101)* (F, US); Hidalgo County, Along side road to Laguna Seca Historical Marker, just west of Hwy 281, 1.5 miles SW of Red Gate, 19 Jan 1981, *Nee, M. 20069* (F); Zapata County, at campground area of Falcon State Park, a peninsula into the Falcon Reservoir lake on the Rio Grande, 1 mile N of Falcon Dam, 19 Dec 1981, *Nee, M. 24122* (BM, CORD, F, TEX); Bandera County, Medina Lake, 14 Jun 1917, *Palmer, E.J. 12261* (MO, TEX, UC); Hidalgo County, Donna, "chiefly in the valley of the Rio Grande below Doñana", *Parry, C.C. 1010 a* (US); Jeff Davis County, Lawrence E. Woods Park off Hwy 118, 23.5 mi NW of Fort Davis, 22 Aug 2015, *Powell, A.M. & Powell, S.A. 7366* (SRSC); Jefferson County, Beaumont, along R.R, 23 Apr 1900, *Reverchon, J. 3916* (MO); Jefferson County, Beaumont, 23 Apr 1905, *Reverchon, J. 3917* (MO); De Witt County, western De Witt County, 26 Jul 1944, *Riedel, M. s.n.* (GH); Cameron County, Brownsville, 1923, *Runyon, R. 338* (TEX); Harris County, [El Jardin del Mar], 10 Jul 1923, *Runyon, R. 450* (US); Cameron County, Brownsville. Elev. 30 ft, 15 Oct 1936, *Runyon, R. 3210* (TEX); Cameron County, El Jardin tract, near Brownsville, 20 Jul 1923, *Runyon, R. 4841* (TEX); Cameron County, El Jardin, near Brownsville, 23 Apr 1936, *Runyon, R. 4843* (TEX); Bexar County, Mitchell Lake, margin of Mitchell's Lake, 17 Apr 1920, *Schulz, E.D. 126* (US); Wilson County, Walsh Ranch, 17 Mi SE of San Antonio, 1 May 1921, *Schulz, E.D. 438* (US); Cameron County, vicinity of Brownsville, 1941, *Shiller, I. 313* (US); Cameron County, 2 miles northwest of Combes, 1 Feb 1954, *Shinners, L.H. 17749* (MEXU); Presidio County, Sierra Vieja, Capote Canyon, below falls, 25 Jun 1973, *Sikes, S. & Smith, J. 672* (NY); Hidalgo County, Santa Ana National Wildlife Refuge, 14 Apr 1977, *Solomon, J.C. 2711* (MO); Brewster County, Alpine, May 1932, *Steiger, T.L. 1319* (NY); Travis County, Austin, 18 Sep 1919, *Tharp, B.C. s.n.* (W); Aransas County, St. Joseph's Bay [San Jose Island], 5 Apr 1932, *Tharp, B.C. s.n.* (TEX); Pecos County, Pecos, 14 Jun 1931, *Tharp, B.C. s.n.* (TEX); Waller County, Pattison, 22 Jul 1929, *Tharp, B.C. s.n.* (TEX); Jackson County, sin. loc, 27 Nov 1939, *Tharp, B.C. s.n.* (GH, UC); Jackson County, Menehee Flats, S of Vanderbilt, 26 Nov 1943, *Tharp, B.C. & Barkley, F.A. 13A-162* (NY); San Patricio County, Ingleside, 22 Dec 1948, *Tharp, B.C. & Brown, ? 48-195* (W); Bexar County, San Antonio, Apr 1863, *Thurber, G. s.n.* (GH, NY); San Patricio County, Lake Corpus Christi State Park camping ground area, 22 Mar 1969, *Tomb, A.S. & Tomb, B. 359* (MO, TEX); Matagorda County, Bay City, 5.5 miles S along Texas Hwy 60, 19 Jul 1956, *Traverse, A. 196* (F, GH); Harris County, Houston, Southside Palce, 3737 Bellaire Blvd, Shell Lab, 3 Nov 1958, *Traverse, A. 910* (GH); Cameron County, Laguna Atascosa National Wildlife Refuge, 2 mi. S of entrance to Unit 1, on primitive road to S from main road, 23 Apr 1959, *Traverse, A. 1075* (F, GH, MO, TEX, US); Cameron County, Southmost, at edge of palm grove, ca. 1.4mi. S from end of highway 1419 i.e. from brick gates, on dirt road, 26 Sep 1959, *Traverse, A. 1110* (F, GH, MO, US); Galveston County, Galveston Island, San Luis Pass, ca. 300 m from the water, along E-W road, 29 Apr 1960, *Traverse, A. 1460* (F, GH, LL); Aransas County, Aransas Refuge, Aransas [Wildlife] Refuge, Salada Well, 14 Aug 1939, *Uzzell, P.B. 181* (US); "Rio Grande Valley", 9 Feb 1942, *Walker, E.J. s.n.* (GH); Presidio County, Presidio, near mouth of Cibolo Creek, 18 Oct 1937, *Warnock, B.H. 155* (US); Brewster County, Near the window; Basin of Chisos Mts, 15 Aug 1937, *Warnock, B.H. 683* (TEX); Jeff Davis County, Wild Rose Pass, Kokernot Ranch, Davis Mountains, 14 miles N of Ft. Davis. Elev. 4900 ft, 4 Jul 1948, *Warnock, B.H. 7932* (LL); Presidio County, Aong Rio Grande between Redford and Presidio. Elev. 3200 ft, 12 Jul 1952, *Warnock, B.H. 10605* (LL); Jeff Davis County, along Musquiz Canyon stream, 17 miles NW of Alpine, 18 Oct 1946, *Warnock, B.H. 46548 B* (TEX, W); Bexar County, San Antonio, 1900, *Wilkinson, E.H. s.n.* (MO); Bexar County, San Antonio, 1897, *Wilkinson, E.H. 115* (MO); Bexar County, San Antonio, *Wilkinson, E.H. 154* (MO); Starr County, Roma Park, 24 Apr 1966, *Wood, A.D. 739* (TEX); Travis County, Bartum Creek, 30 Nov 1913, *Young, M.S. s.n.* (MO); Cameron County, Along Rio Grande, 27 Aug 1915, *Young, M.S. s.n.* (TEX). **Utah**: Salt Lake County, Salt Lake City, Plant grown from seed in the greenhouse at University of Utah, originally from Costa Rica, Heredia, La Selva Biological Station; seed #92-4, LB DNA #70, 8 Oct 2001, *Bohs, L. 3072* (BM, UT).

10. ***Solanum nigrum*** **L.**

CANADA. **British Columbia**: Vancouver 8: Crown/ 23 Avenue West, 19 Aug 1954, *Bird, W. 85* (BM); Vancouver, Stevenston, 26 Jul 1956, *Bird, W. 2395* (BM); Wellington, Vancouver Island, 28 Aug 1917, *Carter, W.R. s.n.* (GH); Vancouver, 51st Ave, one block W of Fraser St, 27 Sep 1978,

Cross, C. s.n. (UBC); Saturna Island, 24 Aug 1951, *Edgar, R. s.n.* (UBC); Vancouver, ex hort UBC, 28 Sep 1949, *Elvidge, D. 89* (UBC); Vancouver, at bottom on East Mall on University of British Columbia campus, 9 Nov 2018, *Knapp, S. IM-10788* (N/A); Port Coquitlam, 8 Nov 1990, *Lomer, F. s.n.* (UBC); Vancouver, 60 m S of Terminal Ave, 100 m E of Staion St, 4 Aug 2003, *Lomer, F. 4981* (UBC); Greater Vancouver, Burnaby, Byrnepark Drive, 220 m N of Southridge Drive, 3 Nov 2004, *Lomer, F. 5563* (UBC); Greater Vancouver, Burnaby, Southridge Drie, E of Brynepark Drive, 10 Nov 2004, *Lomer, F. 5568* (UBC); Greater Vancouver, Burnaby, Gaglardi Way and Hwy 1 westbound entrance, W of waste ground, 20 Aug 2005, *Lomer, F. 5710* (UBC); Fraser Valley, 5 km E of Agassiz, Herrling Island, N end, 14 Oct 2006, *Lomer, F. 6193* (UBC); Fraser Valley, Hope, Kawkawa Lake Indian Reserve 16, E end Ogilview Dr near Kawkawa Lake, 22 Aug 2007, *Lomer, F. 6374* (UBC); Fraser Valley, Popkum, 53275 Yale Rd East, excavation pit 600 m NE of Minter Gardens, 22 Aug 2007, *Lomer, F. 6380* (UBC); Greater Vancouver, Richmond, 1.3 km E of No 6 Rd, 800 m NE of E end of Williams Rd, N of Fraser River, 29 Aug 2007, *Lomer, F. 6395* (UBC); Greater Vancouver, Burnaby, cranberry farm complex S of Marine Way, between Byrne Rd and South Fraser Way, 29 Sep 2008, *Lomer, F. 6861* (UBC); Texada Island, Mount Bay, Mouat Islands, S-most islands, 8 Jul 2012, *Lomer, F. 8019* (UBC); New Westminster, Carnavon St & 4th Ave, NW corner, aprtment building concrete planter box, 13 Sep 2003, *Lomer, F. 8585* (UBC); Metro Vancouver, Burnaby, Cafferky Park 100m NW of Edmonds St and New Vista Place, 2 Oct 2014, *Lomer, F. 9077* (UBC); Vancouver, Marine Way, S side, 300 m E of Kinross Street, 29 Jul 2015, *Lomer, F. 9627* (UBC); Sumas Mountain, lower eastern slopes, 25 Sep 1936, *McCabe, T.T. 3789* (UC); New Westminster, Jul 1910, *Shimuzu, K. s.n.* (UBC); Vancouver, in Asian Garden, UBC Botanical Garden, 10 Aug 1987, *Straley, G.B. 4338* (UBC); South Pender Island, 2 Sep 1955, *Szczawinski, A.F. s.n.* (UBC); British Columbia [sin. loc.], 12 Aug 1917, *Warren, E.M. s.n.* (K); Okanagan River basin, Armstrong Agricultural Hall, 10 Sep 1906, *Wilson, E. 864* (UBC). **Manitoba**: Réserve Forestière Whiteshell, Lac Falcon, 25 Sep 1960, *Boivin, B. & Champagne, A. 14192* (MT); The Pas, 10 Sep 1951, *Krivda, W. s.n.* (BM); The Pas, 15 Aug 1956, *Krivda, W. 1843* (H, UBC); The Pas, Churchill District, 5 Sep 1959, *Krivda, W. 2185 [b]* (GH, UBC). **New Brunswick**: Saint John West, Carleton, 1 Aug 1887, *Hay, G.U. s.n.* (MT). **Nova Scotia**: Pond Cove, Brier Island, 31 Aug 1974, *Bowers, T. 2192* (MT); Bon Portage Island, Shelburne County, 2 Oct 1977, *Crouch, P.A. s.n.* (MT); Markland, Cape Forchu, Yarmouth County, 13 Jul 1921, *Fernald, M.L. et al. 24437* (GH); Dartmouth, Halifax County, 28 Jul 1921, *Fernald, M.L. et al. 24438* (GH); Bay of Fundy, Sandy Cove, Digby Co, 25 Jul 1927, *Roscoe, M.V. & Graustein, J.E. 11109* (MT); Brier Island, barrier beach near Big Pond, 29 Aug 1948, *Smith, E.C. et al. 107* (MT); Cap Forchu, Yarmouth County, 11 Sep 1953, *Smith, E.C. et al. 7120* (MT); Sable Island, main station, 14 Aug 1913, *St. John, H. 1314* (GH, K, NY). **Ontario**: "Mt Ont", *Dearness, W. s.n.* (MT); Toronto County, 2 Oct 2005, *Rothfels, C.J. & Johnson, M.T. 2047* (MICH); Toronto County, Toronto downtown waterfront/portlands; S side Lakeshore Blvd E of Cherry Street, by Redpath Sugar, 2 Oct 2005, *Rothfels, C.J. & Johnson, M.T. 2051* (MICH). **Quebec**: Chambly, comté de Chambly, 15 Sep 1934, *Cléonique-Joseph*, *8071* (MT); Laprairie, 19 Aug 1891, *Euphrosin-Joseph*, *4642* (MT); St. Placide, MRC Deux-Montagnes, au 1411, rt. 344, 16 Aug 1991, *Guertin, P. 4981* (MT).

UNITED STATES OF AMERICA. Hampstead, [USA or Europe?], 15 Aug 1838, *Without Collector s.n.* (PH); Alliance, in front of Ellis residence [also as illegible part -"Alliance Mbr", or maybe Mor, or Nbr -there is an Alliance Nebraska where a family Ellis owned the newspaper!, but this specimen looks like it was collected by E.P. Bicknell [label handwriting match], therefore probably from the NYC area], 22 Aug 1910, *Without Collector s.n.* (NY); sin. loc., 14 Aug 1905, *Without Collector 104/04* (MO). **Alaska**: City of Juneau, Juneau, 25 Jul 1917, *Anderson, J.P. 605* (NY). **California**: Alameda County, Berkeley, Mouth of Strawberry Canyon, near first bridge on path to Strawberry Pool Strawberry Canyon, Berkeley, Strawberry Canyon, 14 Sep 1934, *Belshaw, C.M. 165* (UC); Marin County, Bolinas, 1 Jan 1936, *Belshaw, C.M. 1447* (UC); San Diego County, Sweetwater March National Wildlife Refuge, San Diego City limits, 8 Mar 2005, *Gust, G. & Nye, L. 518* (MO); Colusa County, Sacramento Valley, along CA-20, 0.2 mile east of the Sutter County line, 14 Oct 2008, *Helmkamp, G.K. & Helmkamp, E.A. 14256* (MEXU); Santa Cruz County, Boulder Creek, Hesse place Boulder Creek, 14 Jun 1963, *Hesse, V.F. 3190* (UC); Yolo County, Campus of University of California, Davis, 14 Aug 1988, *Nee, M. 36780* (MO, UC); Sonoma County,

Pepperwood Ranch Natural Reserve, 3450 Franz Valley Road, T9N, R8W, sec. 35 and surrounding sections, 9 Aug 1992, *Nevers, G.C. de*, *10240* (MO); San Benito County, Union District, Hollister, 30 May 1950, *Raven, P.H. 2178 A* (K); Los Angeles County, San Gabriel Mountains, Horse Canyon drainage San Gabriel Mountains, Horse Canyon drainage, 26 Oct 1990, *Ross, T.S. & Mistretta, O. 3317* (UC); Humboldt County, 2 -5 mi e of the South Fork (along eel River, Camp Grant); eel River, Camp Grant, 17 Oct 1940, *Tracy, J.P. 16767* (UC); Kern County, Lumreau Creek, ca. 0.5 miles above Cedar Creek, 1 Oct 1965, *Twisselmann, E.C. 11634* (NY); Alameda County, Bot. Gar, 31 Aug 1909, *Walker, H.A. 1842* (UC); Los Angeles County, Pasadena, Mar Vista and Colorado Blvd, 15 Sep 1975, *Wheeler, L.C. s.n.* (BM); Los Angeles County, Pasadena, Mar Vista and Colorado Blvd, 15 Sep 1975, *Wheeler, L.C. s.n.* (MO); Los Angeles County, Altadena, 202 E. Sacramento Street, 12 Sep 1975, *Wheeler, L.C. s.n.* (UC); Los Angeles County, Los Angeles Basin: City of Pasadena. Arroyo Seco at La Loma Road bridge crossing, 23 Apr 2004, *White, S.D. & Green, J. 10228* (UC). **Connecticut**: New Haven County, New Haven, Aug 1851, *Dana, J.D. s.n.* (YU); Hartford County, Connecticut River, East Hartford, N of R.R, 26 Aug 1898, *Driggs, A.W. s.n.* (CONN); New Haven County, Housatonic River, Orange, Coxy's Place, *Driggs, A.W. s.n.* (CONN); New London County, Groton, near Bogue's, Mumford's T4, 10 Aug 1901, *Graves, C.B. s.n.* (CCNL). **District of Columbia**: Washington DC and vicinity, 28 Sep 1896, *Steele, E.S. s.n.* (MO); Washington DC, USDA; 'cultivated from Australian seed', 1879, *Vasey, G. s.n.* (US). **Florida**: Monroe County, Big Pine Key, 4 Feb 1940, *Seibert, R.J. 1297* (MO). **Georgia**: Glynn County, Brunswick, 15 Aug 1902, *Harper, R.M. 1519* (BM, MO, NY, US). **Idaho**: Canyon County, New Plymouth, 10 Sep 1910, *Macbride, J.F. 734* (GH, MO); Canyon County, New Plymouth, 10 Sep 1910, *Macbride, J.F. 738* (GH, MO, US). **Iowa**: Allamakee County, Seattle, 20 Aug 1933, *Eyerdam, W.J. s.n.* (MO). **Maine**: Knox County, Matinicus Island, Shore road to Sandy Beach, 20 Aug 1957, *Bicknell, E.C. s.n.* (MAINE); Lincoln County, Monhegan Island, Lobster Cove, 5 Sep 1921, *Churchill, J.R. s.n.* (MO); Lincoln County, Monhegan Island, Lobster Cove, 7 Aug 1921, *Churchill, J.R. s.n.* (MO); Lincoln County, Monhegan Plantation, Beach Lobster Cove, 5 Sep 1921, *Churchill, J.R. s.n.* (NHA); Lincoln County, Monhegan Plantation, beach, Lobster Cove, 7 Aug 1921, *Churchill, J.R. s.n.* (NEBC, NHA); York County, Kittery, Appledore Island, Isles of Shoals, 26 Aug 1976, *Crow, G.E. 2329* (NHA); Lincoln County, Pemaquid [georef to lighthouse] [old name for town of Bristol], *Furbish, K. s.n.* (NEBC); Lincoln County, Monhegan, along shore at Lobster Cove, 8 Sep 1967, *Hodgdon, A.R. & Hodgdon, A. 16160* (NHA); Knox County, Criehaven Township, Matinicus Rock, 2 Sep 1967, *Hodgdon, A.R. & Pike, R.B. 18273* (NHA); Knox County, Isle au Haut, beach where Seal Trap and Moore's co, 9 Sep 1927, *Kidder, N.T. s.n.* (NHA); York County, Kennebunk, 6 Aug 1879, *Merrifield, H. s.n.* (NEBC); York County, Kittery, Smuttynose Island, Isles of Shoals, 14 Aug 2006, *Nichols, W.F. & Nichols, V.C. 60* (NHA); Knox County, Matinicus Island, Matinicus Isle Plantation, 22 Aug 1905, *Norton, A.H. s.n.* (NHA); Sagadahoc County, Phippsburg, Bald Head, 12 Sep 1913, *Norton, A.H. s.n.* (NHA); Hancock County, S side of Little Cranberry Island, 3 Sep 1891, *Redfield, J.H. s.n.* (NEBC); Knox County, Matinicus Isle, South West portion of Matinicus Island, 20 Aug 1948, *Rossbach, G.B. 1370* (MAINE); Knox County, Saint George, On small, low island, Shag Ledges, 1+ mile north of Burnt Island off Port Clyde, 19 Jul 1956, *Rossbach, G.B. 3787* (MAINE); Knox County, Saint George, On small ledge island, The Brother's, 2 1/2 mile SW of Martinsville, 14 Aug 1956, *Rossbach, G.B. 3870* (MAINE); Knox County, Matinicus Isle, East shore of Metinic Island, 17 Aug 1956, *Rossbach, G.B. 3924* (MAINE); Knox County, Green Island, In niches near shore, on open island much grazed by sheep, 18 Aug 1957, *Rossbach, G.B. 4210* (MAINE); Knox County, Vinalhaven, Brimstone Island. On open summit ledges, 116ft, 22 Aug 1988, *Rossbach, G.B. 10524* (MAINE); Lincoln County, Boothbay Harbor, Fish Hawk Island, 19 Aug 1955, *Steele, F.L. & Hodgdon, A.R. 1949* (NHA); Cumberland County, Cape Elizabeth, 2 Aug 1851, *Without Collector s.n.* (NHA). **Maryland**: Howard County, Julton, 1977, *Duke, J.H. s.n.* (MO). **Massachusetts**: Nantucket County, Nantucket street, 12 Sep 1899, *Bicknell, E.P. 7719* (NY); Nantucket County, Nantucket, 20 Sep 1907, *Bicknell, E.P. 7720* (NEBC); Nantucket County, Wauwinet, Hyannis, 28 Aug 1888, *Churchill, J.R. 713* (MO); Barnstable County, Eastham, 18 Sep 1885, *Collins, F.S. s.n.* (NEBC); Barnstable County, Yarmouth, 8 Oct 1916, *Fernald, M.L. et al. 15382* (NEBC); Nantucket County, Eastern Massachusetts, Nantucket Island, near Quaise Point, 24 Aug 1928, *Fort, I. s.n.* (MO); Suffolk County, Boston, 1 Nov 1972, *Maskel, S. s.n.* (NEBC); Plymouth County, Little Brewster Island, 27 Sep 1936, *Potter, D. 7372* (GH); Middlesex County, Lowell, 4 Aug 1882, *Swan, C.W. s.n.* (YU). **Missouri**: St. Louis County, St.

Louis, Missouri Botanical Garden, 1 Sep 2010, *Nee, M. 57207* (BM, MO, NY); St. Louis County, St. Louis, Shaw Blvd. between Kingshighway and Vandeventer Ave, 18 Jul 2013, *Nee, M. & McClelland, D. 60196* (GH, MO, NY); Saint Louis County, Missouri Botanical Garden parking area NW of Shaw Blvd and Vandeventer intersection, median flower beds, 23 Jul 2015, *Turner, S.R. & Yatskievych, G. 15-100* (MO). **Montana**: Madison County, Ennis, 20 Aug 1900, *Blankinship, J.W. s.n.* (MONT); Liberty County, Vicinity of Chester, 11 Aug 2000, *Bubnash, J. s.n.* (MONT); Lewis and Clark County, Helena, 1885, *Kelsey, F.D. s.n.* (NY); Flathead County, Columbia Falls, 17 Sep 1894, *Williams, R.S. s.n.* (MONT); Liberty County, From the farm of Elmer Rude Chester, 31 Aug 1967, *Wilson, R. s.n.* (MONT); Custer County, Miles City, *Winkley, D. s.n.* (MONT). **Nevada**: Washoe County, Truckee Meadows, Reno, planters at SE corner of Arlington & 1st in downtown Reno, T19N, R19E, S15, 3 Oct 2012, *Tiehm, A. 16510* (MO, NY). **New Hampshire**: Rockingham County, Rye, Star Island, 30 Aug 2006, *Nichols, W.F. & Nichols, V.C. 62* (NHA); Rockingham County, Rye, Star Island, 30 Aug 2006, *Nichols, W.F. & Nichols, V.C. 65* (NHA). **New Jersey**: Bergen County, Carlstadt Borough, Carlstadt, Eight Day Swamp. Starke Road, NW of Meadowlands and Parkway intersection, 7 Aug 2003, *Barringer, K. 10399* (CONN); Hudson County, Communipaw Ferry, Sep 1880, *Brown, A. s.n.* (NY); sin. loc, *Gray, A. s.n.* (YU); Camden County, Camden, Sep 1879, *Martindale, I.C. s.n.* (F); Camden County, Camden, Oct 1879, *Martindale, I.C. s.n.* (NY); Camden County, Camden, Sep 1879, *Martindale, I.C. s.n.* (NY); Camden County, Camden, Sep 1979, *Martindale, I.C. s.n.* (NY); Camden County, Camden, Aug 1876, *Martindale, I.C. s.n.* (NY); Camden County, Camden, Aug 1878, *Martindale, I.C. s.n.* (US); Camden County, Camden, Sep 1879, *Martindale, I.C. s.n.* (GH); Passaic County, Pasaic County, South Paterson, at George St. and Main St, 20 Aug 2007, *Nee, M. 55538* (MO, NY, UT); Hudson County, Hoboken, RR Yards, 29 Aug 1979, *Reed, C.F. 105844* (MO). **New York**: Bronx County, E of the Grand Concourse, S of E 153rd Streetat Cardinal Hays High School, 14 Nov 2008, *Atha, D.E. & McClelland, D. 6869* (NY); Bronx County, New York Botanical Garden, grounds of garden, E of Bronx River and S of Waring Avenue, 3 Aug 2009, *Atha, D.E. 7932* (NY); New York City, Central Park, Hallett Nature Sanctuary, between 61sta and 62nd Streets and between 5th and 6th Avenues, 15 Sep 2013, *Atha, D.E. & Alvarez, R. 14060* (NY); Monroe County, near Rochester, Jul 1925, *Baxter, M.S. s.n.* (UC); Westchester County, about the Yonkers wool mill, 8 Jul 1894, *Bicknell, E.P. s.n.* (NY); Bronx County, Oak Point, 8 Sep 1908, *Curtis, C.C. s.n.* (NY); New York, seed from Mike Nee, 9 Nov 1990, *D'Arcy, W.G. 17766* (MO, NY); Bronx County, steps just N of Hester Bridge, E side of Bronx River, N edge of path, ca. 25 m uphill from river, New York Botanical Garden, 18 Oct 2010, *Furlaud, J.M. 79* (NY); Rensselaer County, Troy, 1828, *Hall, J. s.n.* (F); Camden, *Martindale, I.C. s.n.* (MO); Camden, N.Y. Camden np Ballast, Sep 1879, *Martindale, I.C. s.n.* (F); Bronx County, Bronx, New York Botanical Garden, along the path at the tip end of the Twin Lakes parking lot, 25 Oct 2006, *McClelland, D. 365* (MO, NY); Bronx County, New York Botanical Garden, Twin Lakes parking lot, 16 Sep 2012, *Meyer, R.S. 285* (NY); Bronx County, around Jerome Park Reservoir, 4 Sep 1984, *Nee, M. 30110* (BM, GH, K, MEXU, MO, NY, US); New York City, Manhattan, 125th St. and West Side Drive, 7 Sep 2000, *Nee, M. & Atha, D. 50960* (MO, NY); Bronx County, Bronx, Mosholu Parkway and Kazimiroff Blvd, between the Metro North railroad lines and the New York Botanical Garden, 2 Aug 2005, *Nee, M. 53365* (BM, MO, NY); Bronz County, Bronx, The New York Botanical Garden, 7 Nov 2006, *Nee, M. 54714* (MO, NY); Bronx County, Bronx, New York Botanical Garden, in front of Pfizer Lab, 10 Aug 2010, *Nee, M. & Knapp, S. 57198* (BM, MO, NY); Tompkins County, Ithaca, Cornell Univ, 30 Sep 1939, *Palmatier, E.A. s.n.* (KIRI); sin. loc. [NYC?], *Without Collector s.n.* (NY); Bronx County, Bronx, Norwood neighborhood, E Moshulu parkway North, at NW corner of Bainbridge Avenue (N of Moshulu Parkway), 3 Sep 2001, *Zanoni, T.A. 47320* (NY). **North Carolina**: New Hanover County, Wilmington, 28 Jun 1890, *Coville, F.V. 185* (US); Anderson County, Anderson, 5 Jul 1919, *Davis, J. s.n.* (MO); vastis "in oriente Carolina Septentrionalis", Aug 1885, *McCarthy, G. s, n,* (US); New Hanover County, Wilmington, edge of Dock St at jct with Forest St, W side of town at river front, jsut down stream from USS North Carolina battleship memorial, 4 Jun 1994, *Nelson, J.B. et al. 15508* (UBC); Buscombe County, Biltmore, 17 Jul 1897, *Without Collector 912 b* (MO). **Oklahoma**: Payne County, 22 miles SE of Stillwater, 11 Jul 1936, *Pote, L. 114* (MO). **Oregon**: Polk County, Willamette Pollen, ca. 2.5 miles N of Monmouth along State Hwy 99W, 4 Aug 1983, *Halse, R.R. 2725* (NY, UBC); Multnomah County, North of forestry building, Portland, 31 Aug 1927, *Thompson, J.W. 3663* (MO); Benton County, Corvallis, Highway 99W N of Crystal Lake Drive, near new highway

overpass, 5 Sep 1992, *Wilson, B. 5399* (MICH). **Pennsylvania**: York County, Eozoic, at McCall's Ferry, 15 Oct 1892, *Heller, A.A. & Halbach, E.G. 632* (MO). **Texas**: Jackson County, Menehee Flats South of Vanderbilt, 26 Nov 1943, *Barkly, F.O. 13A 162* (MO). **Utah**: Washington County, St. George, T42S, R15W, S30, 20 Jun 1998, *Higgins, L.C. 19750* (NY); Utah County, 8 miles W of Springville, 18 Sep 1931, *Maguire, B. 308* (UC). **Virginia**: Alexandria, [label also says Dist. Line, 2 Sept 1896], 28 Sep 1897, *Steele, E.S. s.n.* (US). **Washington**: King County, Seattle, NE 40 & Brooklyn Avenue, 22 Aug 1969, *D'Arcy, W.G. 3644* (MO); Franklin County, near Eltopia, 6 Sep 1969, *Dunn, D.B. 16384* (MO, NY); Franklin County, Fish Hook, Ferry Landing, 18 Sep 1894, *Leiberg, J.B. s.n.* (US); Franklin County, Fish Hook Ferry, [Eastern Oregon on label, but Franklin County is in the state of Washington], 18 Sep 1894, *Leiberg, J.B. 929* (GH, NY); Snohomish County, Forest Crest Playfield, 5006 236th Street Southwest, Mountlake Terrace, 10 Oct 2015, *Olmstead, R.J. 2015-55* (WTU). **Wisconsin**: Richland County, Grown in garden, 3 miles SE of Richland Center (T 10 N; R 1 E; NE1/4NE1/4 Sec. 35), 9 Sep 1978, *Nee, M. 16069* (BM, MEXU); Richland County, Grown in garden, 3 miles SE of Richland Center (T 10 N; R 1 E; NE1/4NE1/4 Sec. 35), 31 Aug 1978, *Nee, M. 16073* (BM, MEXU).

11. ***Solanum nitidibaccatum*** **Bitter**

CANADA. **Alberta**: MacLeod District, 13 Sep 1940, *Dixon, R.H. 1644* (UC). **British Columbia**: Salt Spring Island, Don Fraser's garden, Beaver Point, 24 Jul 1956, *Ashlee, T.R. s.n.* (UBC); Vancouver, 2400 West Mall, UBC, 24 Sep 1978, *Bowen, P.A. s.n.* (UBC); E side Okanegan Lake, 8 mi. N Naramata, Yale co, 27 Aug 1950, *Bowman, R.L. s.n.* (UC); Tranquillo, [Tranquille River?], 6 Oct 1962, *Brink, V.C. s.n.* (UBC); along Canadian Pacific RR, 10 Aug 1897, *Canby, W.M. et al. 210* (GH); along Can. Pac. R.R. [Canadian Pacific Railroad], Fraser River, 17 Aug 1904, *Condit, I.J. s.n.* (UC); Hedley, Similkameen District, Sep 1913, *Cormisn, E.C. s.n.* (UBC); near Spence's Bridge, 16 Jul 1913, *Davidson, J. s.n.* (UBC); Sechelt, 8 Jul 1973, *Dawe, H. s.n.* (UBC); Naksup, garden of Leland Hotel, 12 Jul 1940, *Eastham, J.W. s.n.* (UBC); Vancouver, ex hort UBC, N beds in Bot Garden, 28 Sep 1949, *Elvidge, D. 88* (UBC); Pitt Meadows, 1958, *Evans, R. s.n.* (UBC); Creston, 26 Sep 1964, *Fodor, F. 242* (UBC); Raft River Canyon, 4 km E of Clearwater Village, 20 Sep 1980, *Goward, T. & Schofield, W.B. 81-915* (UBC); Burnaby, 29 Oct 1926, *Laurence, A. s.n.* (UBC); Cache Creek, 2 km NW of Hwy 97, Horsting's Farm, 21 Sep 2004, *Lomer, F. 5479* (UBC); Metro Vancouver, Burnaby, 11 Avenue, E side, 50 m S of 15th Street, 28 Sep 2014, *Lomer, F. 9067* (UBC); Metro Vancouver, Burnaby, Edmonds Street and New Vita Place, NE corner, 2 Oct 2014, *Lomer, F. 9073* (UBC); Kamloops, 29 Jun 1889, *Macoun, J. s.n.* (BM, NY); Vancouver Island, vicinity of Victoria, 20 Aug 1893, *Macoun, J. 698* (GH); Vancouver Island, vicinity of Nanaimo, Biological Station, 4 Aug 1908, *Macoun, J. 85823* (NY); southest of Penticton, White Lake area, near observatory, 26 Jul 2011, *McIntosh, T. & Hanna, D. 2011-214* (UBC); Vancouver Island, off Cedar Hill Cross Road, Victoria, 23 Jul 1964, *Melburn, M.C. s.n.* (DUKE); Vancouver Island, Victoria, 23 Jul 1964, *Melburn, M.C. s.n.* (DUKE); Salt Spring Island, 7 Sep 1955, *Molyneux, W.E. 245* (UBC); E of Oliver, 26 Aug 1953, *Ogilvie, R.T. s.n.* (UBC); Vancouver, in front of Biological Sciences Building, UBC, 7 Oct 1977, *Pinder-Moss, J. 1374* (UBC); Vancouver, at side of huts behind Education building, UBC, 29 Aug 1979, *Pinder-Moss, J. 1395* (UBC); Peace River Lodge, 5 Sep 1943, *Raup, H.M. & Correll, D.S. 11641* (A); Vancouver, ex hort UBC, N beds in Bot Garden, 12 Jul 1948, *Rogers, C.B.W. 276* (UBC); Vancouver, UBC Botanical Garden, 4 Aug 1987, *Straley, G.B. 4326* (UBC); Vancouver, Trimble Street near 13th Avenue, 24 Sep 1992, *Taylor, T. s.n.* (UBC); New Westminster, Aug 1909, *Without Collector s.n.* (UBC); Vernon, 23 Jul 1977, *Wright, G. s.n.* (UBC). **Manitoba**: Brandon, 29 Aug 1960, *Stevenson, G.A. 1934* (MT). **New Brunswick**: South Bay, 15 Sep 1965, *Hayes, F. s.n.* (MT); St. George, 26 Jul 1965, *Kimball, D. s.n.* (MT). **Ontario**: Garden at 539 Hodder Ave. Thunder Bay City, Thunder Bay District, 6 Nov 1971, *Garton, C.E. 14758* (H, MT); Tamsalu family home, Snoddon Road, Georgina Township, York Region, 10 Oct 2005, *Rothfels, C.J. 2062* (MICH). **Quebec**: Rimouski, 27 Sep 1960, *Lepage, E. 14327* (NY).

MEXICO. **Baja California**: Sierra San Pedro Mártir, upper Corona Meadow, 31 Aug 1963, *Moran, R. 11291* (RSA, SD); Isla Guadalupe, 1875, *Palmer, E. 60 [c]* (BM, MO).

PANAMA. **Chiriquí**: Alto Pineda, end of road, right turn just before cooperativa entrance to Cerro Punta, 11 Apr 1979, *Hammel, B. et al. 6964* (MO).

UNITED STATES OF AMERICA. "PRR Survey" "N.C.M.G.", *Stevens, ?, s.n.* (US). **Alaska**: Juneau County, Juneau, 15 Jul 1917, *Anderson, J.P. 604* (NY); Yukon-Koyukuk Census Area, Circle Hot Springs, 3 Aug 1941, *Anderson, J.P. 7558* (GH). **Arizona**: Cochise County, Paradise, 5 Oct 1907, *Blumer, J.C. 1768* (K, L, NY, U); Navajo County, Mogollon Plateau, ca. 3 miles SE of Heber, 2 miles NNE of Overgaard, subdivision S of Hwy 277 (T12N R17E), 6 Sep 1992, *Ertter, B.J. 11320* (UC); Coconino County, 300 block of E Gila St. Bow & Arrow neighborhood, 25 Sep 1998, *Hammond, H.D. 11511* (MO); Coconino County, Flagstaff, Lake Mary Rd, at Latter Day Saints Church, 31 Jul 2002, *Hammond, H.D. 11928* (F, MO); Mohave County, Mt. Logan, GCPN [Grand Canyon National Park?], 11 Sep 2002, *Higgins, L.C. 24010* (NY, UC); Navajo County, 4 miles east of Heber, 14 Sep 1963, *Lehto, E. 3496* (ASU); Pinal County, Eloy, 3 Jul 1936, *Peebles, R.H. 13085* (NY); Coconino County, Coconino Co.: In picnic area along US Route 180 at N edge of Kendrick Park ca. 3.37 km E of the summit of Horseshoe Hill. Cocnino N.F. Kendrick Peak Quad (7 1/2 min.). T24N, R6E, Sec. 33. UTM-3920200 m.N by 431340 m.E (Zone 12), 13 Aug 1992, *Windham, M.D. 92-271* (UT); Coconino County, In picnic area along US Route 180 at N edge of Kendrick Park ca. 3.37km E of the summit of Horseshoe Hill Coconino N.F.Kendrick Peak Quad (7 1/2 min.) T24N, R6E, sec. 33 UTM -3920200 m.N by 431340 m.E (Zone12), 13 Aug 1992, *Windham, M.D. 91-271* (MO). **Arkansas**: Benton County, Chesney Prairie Natural Area, vicinity of Gentry, 23 Oct 2003, *Sundell, E. & Sundell, M. 16733* (NY). **California**: Butte County, Peter Ahart Ranch, about 1 1/2 miles north and 1 miles east of Honcut, about 12 miles southeast of Oroville, 30 Oct 2006, *Ahart, L. 13517* (NY); Yuba County, east of the dirt road, 20 yards northeast of the gate, about 20 yards south of the power tower, about 30 yards north of Fruitland Road, about 13 miles (air) northeast of Marysville, 14 May 2007, *Ahart, L. 13848* (JEPS); Lassen County, On the east side of Walters Road, 4235 feet south of Modoc County Line, about 4.3 miles (air) south-southwest of Adin, about 7.0 miles (air) northeast of Bieber, Big Valley, 31 Aug 2010, *Ahart, L. & Dittes, J. 17138* (JEPS); Amador County, vicinity of Ione, Aug 1904, *Braunton, E. 1200* (NY, UC); Alameda County, Bay Farm Island; Western Middle California, Bay Farm Island, 24 Sep 1898, *Burtt Davy, J. s.n.* (UC); Siskiyou County, near Yreka, 11 Jul 1908, *Butler, G.D. 1009* (UC); Los Angeles County, Compton, 24 Jul 1908, *Condit, I.J. s.n.* (UC); Mono County, Mono Lake, 15 Aug 1894, *Congdon, J.W. 65* (GH); Kern County, Farnsworth Ranch, 2.5 mile southwest of Glennville, 27 Jul 1964, *Farnsworth, E.L. 244* (NY); Mono County, Along Green Creek, junction of U.S. Highway 395 & Green Lake road, 5 mi. S of Bridgeport, 15 Sep 1957, *Ferris, R.S. 13116* (GH, NY); Alameda County, West Berkeley, 25 Aug 1891, *Greene, E.L. s.n.* (K); Alameda County, West Berkeley, 25 Aug 1891, *Greene, E.L. s.n.* (UC); Solano County, Vallejo, *Greene, E.L. s.n.* (GH); San Diego County, San Diego, Apr 1903, *Hall, H.M. s.n.* (UC); Los Angeles County, streets of Los Angeles, 1888, *Hasse, H.E. 5233* (NY); Siskiyou County, Metcal's ranch, NE base of Mt. Eddy, 24 Aug 1915, *Heller, A.A. 12208* (GH, MO, NY, UC); Mono County, Little Mormon Meadow, 25 Aug 1937, *Hendrix, T.M. 583* (UC); Upper Sarmiento, Oct 1877, *Hooker, J.D. & Gray, A. s.n.* (K); Stanislaus County, Modesto, 15 Jun 1935, *Hoover, R.F. 663* (NY); Napa County, Vineyard of Eugene and Cody Gillette Kirkham, Silverado Trail North, St. Helena, 15 Oct 1987, *Howell, J.T. s.n.* (NY); Mono County, canyon W of Walker River, 15 Sep 1966, *Howell, J.T. 41390* (NY); Lassen County, Near Susanville, Diamond Range, 1 Aug 1973, *Howell, J.T. & True, G.H. 50146 A* (NY); San Bernardino County, Upland, Jun 1917, *Johnston, I.M. 1812* (UC); Butte County, Chico, 31 Jul 1905, *Krautter, L. s.n.* (BM); Butte County, near Chico, Jun 1905, *Krautter, L. s.n.* (K); Monterey County, near Gibson Creek (well above road, Point Lobos State Park), Point Lobos State Park, 13 Oct 1935, *Lee, E. 1663* (JEPS); Los Angeles County, Los Angeles, 5 Sep 1896, *McClatchie, A.J. s.n.* (NY); Alameda County, West Oakland, 4 Nov 1904, *McGraw, H. s.n.* (UC); Inyo County, at Monte Negro Springs, 12 Jul 1964, *Mitchell, R.S. 2146* (NY); San Luis Obispo County, Cambria, C. O. Blodgett farm Cambria, 4 Aug 1951, *Paddock, E.F. 416* (UC); Los Angeles County, near Los Angeles, 8 Sep 1896, *Raben, C. s.n.* (NY); Ventura County, along Avalon Slough, just N of Point Mugu, 4 Oct 1959, *Raven, P.H. & Thompson, H.J. 14582* (GH); San Francisco County, San Francisco, Golden Gate Park, 1 Sep 1959, *Rose, L.S. 59161* (GH, NY); San Bernardino County, San Benardino Mountains; Riley's Farm on E side of Oak Glen Road ca. 2. miles above Wildwood Canyond Road (T2S, R1W, NW/4 sec. 1), 23 Aug 1992, *Sanders, A.C. 12462* (MO, UC); San Joaquin

County, Stockton, College of the Pacific, 5 Oct 1926, *Stanford, E.E. 12* (GH); San Mateo County, El Granada, 28 Jul 1940, *Stebbins, G.L. & Paddock, E.F. 97* (GH); Alpine County, 2 miles W Woodfords along West Fork Carson River canyon, 0.4 miles below Crystal Springs, 24 Aug 1974, *Taylor, D.W. 4863* (JEPS); Sonoma County, Burbank Farm, 23 Nov 1935, *Thomas, H.A. s.n.* (UC); Humboldt County, immediate vicinity of eureka, Sep 1907, *Tracy, J.P. 2605* (UC); Humboldt County, immediate vicinity of eureka, 10 Aug 1909, *Tracy, J.P. 3074* (UC); Humboldt County, eureka, 28 Sep 1940, *Tracy, J.P. 16738* (GH, JEPS); Humboldt County, Trinity River Valley at Willow Creek Willow Creek, Trinity River Valley, Willow Creek, 15 Oct 1940, *Tracy, J.P. 16758* (UC); Kern County, Weldon Highway, 0.7 mile SE of Kernville, 19 Jun 1971, *Twisselmann, E.C. 17685* (MEXU); Los Angeles County, 2 mi nne of Claremont, 6 Aug 1932, *Wheeler, L.C. 1125* (UC); Los Angeles County, 2 mi ne of La Verne, 11 Aug 1932, *Wheeler, L.C. 1130* (UC); Los Angeles County, Harrison & San Antonio Ave. W Claremont, 10 Sep 1932, *Wheeler, L.C. 1369* (GH, NY); Modoc County, mouth of Pine Creek, Warner Mountains, 6 Aug 1935, *Wheeler, L.C. 3798* (GH); Modoc County, 3 mi W of Alturas, 22 Sep 1935, *Wheeler, L.C. 3994* (MO, NY); San Mateo County, 1/2 mi s of Moss Beach, 24 Aug 1935, *Wiggins, I.L. 8164* (GH, UC); San Mateo County, Pedro Valley, inland from Pedro Point, 22 Sep 1945, *Wiggins, I.L. 11197* (GH, NY); San Mateo County, Moss Beach, 22 Sep 1945, *Wiggins, I.L. 11198* (GH); Los Angeles County, Los Angeles, 21 Jul 1925, *Without Collector s.n.* (JEPS). **Colorado**: Weld County, S. Platte River near Hardin, 11 Jun 1939, *Ewan, J.A. 12130* (UC); Boulder County, Superior, S of Louisville, 17 Oct 1941, *Ewan, J.A. 14108* (GH); Boulder County, Peaceful Valley, Van Vleet Arabian Ranch, N of Ward, headwaters of Middle St. Vrain, Sep 1943, *Ewan, J.A. 15498* (GH); Arapahoe County, 8.5 mi S of Interstate 70 on Arapahoe County Road 137, 18 Aug 1991, *Miller, J.S. 6882* (MO); Weld County, Between Fort Collins & Cheyenne, just south of the Wyoming border, 18 Aug 1984, *Neese, E.J. 16115* (NY); Boulder County, Boulder Cañon just east of tunnel, 17 Aug 1955, *Rees, M. 302* (BM); Boulder County, Jul 1902, *Tweedy, F. 5234* (NY); Larimer County, RidgeCrest road, Stapleton Greenhouse, Fort Collins, 10 Jun 1983, *Walter, D. 9008* (MO). **Connecticut**: Litchfield County, Gaylordsville, 5 Aug 1914, *Austin, E.H. s.n.* (YU). **Idaho**: Madison County, south Sugar City, 3 Sep 1966, *Barrus, B. 16* (H); Canyon County, Caldwell, Bratz residence, 1400 Willow St, 9 Jul 1960, *Bratz, R.D. s.n.* (NY); Ada County, W of Boise, 24 May 1934, *Christ, J.H. 2564* (NY); Ada County, 5 miles N of Boise, 14 Aug 1940, *Christ, J.H. 11730* (NY); Ada County, Boise, 17 Jun 1911, *Clark, J.A. 56* (GH, UC); Bannock County, Pocatello, 31 Aug 1935, *Davis, R.J. 297 -35* (UC); Adams County, New Meadows, 23 Jun 1940, *Davis, R.J. 2978* (NY); Elmore County, S side of Snake River across from King Hill, 25 Jul 1978, *Ertter, B. 2431* (NY); Ada County, Boise River Greenbelt in east Boise, middle of Bethine Church River Trail (=pedestrian only section paralleling River Run), near mile marker 3.5, 6 Jul 2010, *Ertter, B.J. 20019* (UC); Canyon County, New Plymouth [really Payette County], 10 Sep 1910, *Macbride, J.F. 737* (GH, NY, UC); Bannock County, Pocatello, 2 miles N of town, 19 Sep 1946, *Metlen, R. 3103* (W); Canyon County, Caldwell, Sep 1931, *Tucker, H.M. s.n.* (NY); Ada County, Boise, Jul 1881, *Wilcox, F.E. s.n.* (GH). **Kansas**: Cloud County, Aurora, 1 Sep 1942, *Fraser, S.V. 833* (GH). **Maine**: Penobscot County, Bangor to Ellsworth, Route US1, 8 Miles SE of Bangor, 31 Aug 1930, *True, R.H. s.n.* (NEBC). **Massachusetts**: Essex County, Newburyport, C.J. MacGregor greenhouse, 2 Aug 1949, *Bean, R.C. s.n.* (NEBC); Worcester County, Uxbridge, Blackstone Street, 8 Sep 2005, *Bertin, R.I. s.n.* (MASS); Worcester County, Leominster, near Mechanic Street and White Pond Road, market garden, 17 Sep 2007, *Bertin, R.I. 4226* (MASS, NEBC); Nantucket County, Nantucket, 4 Sep 1904, *Flynn, N.F. s.n.* (VT); Nantucket County, Nantucket, Milk and Main Ste, 4 Sep 1901, *Flynn, N.F. s.n.* (VT); Essex County, Boxford, dump, Spofford Rd, 27 Sep 1955, *Harris, S.K. 11569* (NEBC); Essex County, Lynn, 12 Oct 1955, *Harris, S.K. 11842* (NEBC); Essex County, Danvers, dump off Rte. 114, 9 Sep 1958, *Harris, S.K. 18574* (NEBC); Essex County, Saugus, dump off Salem Turnpike, 16 Sep 1958, *Harris, S.K. 18714* (NEBC); Essex County, Marblehead, Green Street, 11 Oct 1958, *Harris, S.K. 18924* (NEBC); Norfolk County, Milton, 28 Sep 1929, *Kidder, N.T. s.n.* (GH); Norfolk County, Milton, 28 Sep 1928, *Kidder, N.T. s.n.* (NEBC); Nantucket County, Nantucket Island, Siasconset, 14 Aug 1960, *MacKeever, F.C. N-434* (NEBC, NY); Nantucket County, Nantucket Island, Siasconset, 27 Aug 1962, *MacKeever, F.C. N-691* (NY); Barnstable County, S. Dennis, 28 Aug 1993, *Nickerson, N.H. s.n.* (MASS); Barnstable County, South Dennis, 31 Aug 1978, *Nickerson, N.H. s.n.* (MASS); Barnstable County, South Dennis, *Nickerson, N.H. s.n.* (CONN); Worcester County, Lancaster, 30 Aug 1944, *Seymont*, *1060* (MO); Worcester County, Lancaster, 25

Sep 1943, *Seymour, F.C. 6030* (NEBC); Middlesex County, Chelmsford, 20 Sep 1885, *Swan, C.W. s.n.* (YU). **Minnesota**: Ramsay County, University of Minnesota St. Paul campus, crop experimental fields just south of Larpenteur, 7 Sep 1999, *Cholewa, A. 2597* (MIN); Ramsay County, NE corner of parking lot at Long Lake swimming beach. NE 1/4 of NW 1/4 of section, Long Lake Regional Park, 22 Aug 2012, *Dzuik, P. 1022* (MIN); Clearwater County, Itasca State Park, On the Campus of the Biological Station, along the E shore of Lake Itasca, 7 Aug 1946, *Moore, J. 18989* (MIN); Anoka County, S shore of Fish Lake, Cedar Creek National History Area, 13 Oct 1958, *Moore, J. 24439* (MIN); Clearwater County, Itasca State Park, Nursery, 13 Jul 1933, *Moyle, J. 842* (GH, MIN). **Missouri**: Saint Louis City, Saint Louis City, Burlington Railroad, N of East Grand Avenue two specimens [plants], on the large free place S of the grain elevator, a few meters from the right-of-way, 20 Aug 1955, *Muehlenbach, V. 729* (MO); McDonald County, McNatt, 15 Aug 1955, *Palmer, E.J. 60872* (F). **Montana**: Flathead County, Big Fork, 2 Aug 1909, *Butler, B.T. s.n.* (NY); Flathead County, Big Fork, 3 Aug 1909, *Butler, B.T. 7014* (NY); Fallon County, In Baker, 27 Jul 1982, *Castleberry, P. s.n.* (MONT); Musselshell County, From the range on the farm of Clayton Jensen, Musselshell, 18 Sep 1967, *Kautzmann, D. s.n.* (MONT); Blaine County, Chinook vicinity (RR 71), 12 Sep 2000, *Pridgeon, M. s.n.* (MONT); Carbon County, Vicinity (South of) Fromberg, 24 Aug 2001, *Sidwell, D. s.n.* (MONT); Missoula County, Lolo (near the) Forest, 2926 Brayton (3 miles north of Missoula), 9 Sep 1961, *Stickney, P.F. 450* (MONT); Lake County, [No locality given], Aug 1924, *Swingle, D.B. s.n.* (MONT); Lake County, near Yellow Bay, about halfway between Polson and Big Fork near eastern shores of Flathead Lake, forest trail 311, 4 Jul 1965, *Thomas, J.H. 10878* (US); Hill County, Havre vicinity, 5 Aug 1998, *Widdekind, P. s.n.* (MONT); Judith Basin County, Utica, 5 Sep 1966, *Without Collector s.n.* (MONT); Valley County, Hinsdale, 21 Sep 1948, *Without Collector s.n.* (MONT); Gallatin County, Bozeman, 30 Sep 1925, *Young, P.A. s.n.* (MONT). **Nevada**: Mineral County, mouth of Cory Creek, Wassuk range, 29 Aug 1938, *Archer, W.A. 6837* (UC); Mineral County, Walker Lake, west side of lake, 16 Sep 1938, *Archer, W.A. 7134* (DUKE); Mineral County, East Walker River, 20mi. E. of Sweetwater, 29 Sep 1938, *Archer, W.A. 7289* (MO, UC); Carson City Consolidated Munci, King's Canon (on label as Ormsby County), 1 Aug 1902, *Baker, C.F. 1426* (GH, MO, NY, UC, W); Washoe County, Reno Indian Colony, 8 Sep 1937, *Breene, T.L. & Sampson, H. 66* (NY); Lander County, vicinty of Big Cr[eek] Canyon, 16 miles S of Austin, 28 Aug 1937, *Goodner, F.S. & Henning, W.H. 1274* (UC); Lander County, Vicinity of Mill Cr. Ranch, about 25 mi. S of Battle Mt. along highway 8-A, Lander Co, 4 Sep 1937, *Henning, W.H. 14* (MO, NY, UT); "Bet. Virginia et Carm", 1877, *Hooker, J.D. & Gray, A. s.n.* (GH); Carson City Consolidated Munci, Lloyds Bridge, Carson River, near Pinyon Hills, Carson City, 26 Jul 1976, *Pinzl, A. 987* (NY); Nye County, Toiyabe Range, Peaving Campground, 26 Jul 1979, *Pinzl, A. 2467* (NY); Douglas County, Carson Valley, west side of Rte 395 where it crosses the Carson River, south bank of river, 14 Oct 1988, *Pinzl, A. 8457* (NY); Nye County, Longstreet Ranch, nw. Kawich Range. Drainage Basin: E. Stone Cabin, 12 Sep 1970, *Pinzl, A. 11728* (NY); Humboldt County, Quinn River Valley, 1.7 road miles west of Orovada/US 95 on Rte 293, 17 Aug 2000, *Pinzl, A.P. 13707* (NY); Douglas County, Carson River at Cradlebaugh Bride south of Carson City, 17 Jul 1977, *Tiehm, A. 3657* (NY); Washoe County, Spanish Springs Valley, east side of the Valley, north of Sparks, T20N, R20E, sec. l, 16 Aug 1980, *Tiehm, A. 6219* (MO, NY); Lyon County, East Walker River near The Elbow area, 4 Jul 1981, *Tiehm, A. 6718* (NY); Elko County, Delano Mountains, Crittenden Reservoir along Crittenden Creek north of the Twelvemile Ranch, T42N, R69E, S17, 21 Aug 1986, *Tiehm, A. & Nachlinger, J. 10921* (NY); Lander County, Shoshone Mountains, Schoonover Creek, 1.2 road miles SE of highway 2 east of Peterson Station, 15 Aug 2001, *Tiehm, A. 13715* (NY, UC); Nye County, Peavine Creek, Smokey Valley Dam, crossing on Peavine road, 19 Sep 1939, *Train, P. 3469* (NY, UC); Washoe County, Rancho San Rafael, NW section of Reo, 20 Sep 1976, *Williams, M.J. & Tiehm, A. 76-117 -11* (NY); Washoe County, Parker Ranch on Truckee River, E of Sparks, near Lockwood, 16 Oct 1976, *Williams, M.J. 76-118 -7* (NY). **New Hampshire**: Coos County, Randolph, A.S. Pearse's garden, 10 Sep 1926, *Deane, W. s.n.* (GH); Coos County, Gorham, Cascade, 11 Aug 1967, *Harris, S.K. 31465* (NEBC); Coos County, Randolph, 18 Aug 1926, *Pease, A.S. 19871* (NEBC). **New Mexico**: San Miguel County, vicinity of Las Vegas, Sep 1919, *Anect Brother*, *16* (NY); San Miguel County, 10 miles north of Las Vegas on hwy 3, 21 Aug 1974, *Higgins, L.C. 9103* (NY); San Miguel County, M. E. O'Connor Trust Ranch. House Trap, head of Cuevas Canyon; 20 mi ESE of Las Vegas; 5 mi S of Rte. 104, 16 Aug 1982, *Hill, S.R. 11924* (NY); San Miguel County, T. M. O'Connor Ranch. Cuervito

Canyon Pasture; Cuervito Canyon, near windmill # TM 14; 18 mi E of Las Vegas, ca. 5.5 mi S of Rte. 104, 27 Jul 1984, *Hill, S.R. 14831* (GH, NY); Colfax County, Vicinity of Ute Park, 1 Sep 1916, *Standly, P.C. 14213* (NY). **New York**: Arlington, Staten Island, 20 Sep 1909, *Hollick, C.A. s.n.* (NY); Orange County, Crows Nest Brook, just above Garrard road, US Military Academy Reservation [UTM 585300 4583700], 2 Jul 1992, *Mitchell, R.S. & Tucker, G.C. 7838* (MO); Suffolk County, Long Island, Flushing, 17 Sep 1936, *Monachino, J.V. 176* (BUT, DUKE, NY); Ontario County, Seneca station of Cornell University Agricultural Experiment Station, 2 miles NW of Geneva, 4 Oct 1992, *Nee, M. 43042* (CORD, MO, NY). **North Dakota**: Barnes County, Litchville, 14 Aug 1953, *Stevens, O.A. 1465* (NY, UC). **Oregon**: Umatilla County, state hospital, Pendleton, 24 Jul 1933, *Barss, H.P. s.n.* (MO); Umatilla County, near Milton, 26 Aug 1893, *Brown, H.E. 39* (F, MO, NY, UC); Benton County, Corvallis, OSU campus, in front of Wilkinson Hall, 23 Oct 2010, *Halse, R.R. 8122* (NY); Josephine County, Grant's Pass, 3 Jul 1887, *Howell, T. 232* (MO); Josephine County, Grant's Pass, 3 Jul 1887, *Howell, T.J. s.n.* (NY); Multnomah County, sin. loc, Jul 1877, *Howell, T.J. 333* (GH); Union County, 1.6 miles NW of La Grande, 3 Aug 1969, *Lewis, W.H. 7336* (MO); Multnomah County, Linnton, 29 Jul 1916, *Nelson, J.C. 858* (GH); Multnomah County, Portland, 22 Jul 1917, *Nelson, J.C. 1729* (GH); Willamette County, Salem, 26 Jul 1917, *Nelson, J.C. 1776* (GH); Willamette County, Mintois Island, Salem [Minto Island], 10 Sep 1919, *Nelson, J.C. 2927* (GH); Deschutes County, field along Deschutes R, 5 miles below Bend, 2 Aug 1920, *Peck, M.E. 9750* (GH, MO, NY); Lincoln County, South Beach, 29 Aug 1921, *Peck, M.E. 10712* (F); Linn County, Lebanon, 22 Oct 1914, *Roberts, E.C. 11388* (DUKE); Cache Bar, between Cache & Garden Creeks on Snake River, 19 Jun 1897, *Sheldon, E.P. 8326* (NY); Union County, Fry's Point, 30 Sep 1897, *Sheldon, E.P. 9036* (GH, NY); Wasco County, W of Wamic, 12 Oct 1955, *Steward, A.N. 7017* (GH, NY, UBC); Jackson County, along Applegate Road at Yale Creek Road, S of Ruch, 11 Sep 1994, *Straley, G.B. 8079* (UBC); Multnomah County, Hayden Island, 4 Sep 1927, *Thompson, J.W. 3766 b* (MO). **Pennsylvania**: Bucks County, Doylestown, along Doyle Street, rubbish dump at western edge of boro, 20 Jul 1952, *Long, R. 75317* (PH); Delaware County, Upper Darby: 0, 2 miles SE of intersection of Marshall Road and 69th Street Boulevard, 30 Sep 1941, *Wheeler, L.C. 5554* (BM). **Texas**: Walker County, Huntsville, 24 May 1917, *Palmer, E.J. 12023* (MO, UC). **Utah**: Salt Lake County, S of Salt Lake City, just S of intersection of 7400S and 20E in an active sand dune area, 24 Sep 1967, *Arnow, L. 740* (UT); Salt Lake County, Salt Lake Co., Utah: Salt Lake City intersection of 11 W and Jordan River at about 29 S, 19 Aug 1973, *Arnow, L. 3740* (UT); Salt Lake County, Salt Lake Co. Utah: Salt Lake City, W of University of Utah Campus at 3rd S and 13th E, 10 Jul 1978, *Arnow, L. 5552* (UT); Sevier County, Ca. 1 mile south of Jct. 89/12, 7 miles S. of Panguitch, Sevier River bottom, 18 Sep 1984, *Atwood, N.D. 10672* (NY); Washington County, Beaver Dam Wash, Lytle Preserve, 12 Oct 1986, *Baird, G.I. 2494* (NY); Iron County, ca. 45 miles N of Cedar City, 11 Sep 1976, *Davis, T. 661* (MO); Salt Lake County, Salt Lake City, 3 Jun 1907, *Garrett, A.O. 1097* (NY); Cache County, Lewiston, 20 Aug 1920, *Garrett, A.O. 2967* (NY); Salt Lake County, Salt Lake City, [illegible] grounds, 8 Aug 1932, *Garrett, A.O. 6219* (UT); Salt Lake County, 21 Jun 1932, *Garrett, A.O. 6606* (UT); Salt Lake County, In terrace 17th S, west of 13 E. Salt Lake City, Salt Lake, 16 Aug 1944, *Garrett, A.O. 8736* (UT); Utah County, T1N, R1E, S22 SE1/4, USM, 1.1 mi due NW of Tridell, 30 Jul 1983, *Goodrich, S. 19277* (UT); Utah County, One mile below Vivian Park, Provo Canyon. Gravelly stream bank, 7 Sep 1944, *Harrison, B.F. 10707* (UT); Piute County, CC Fremont, Box Creek Canyon, W of Greenwich, 12 Aug 1977, *Higgins, L.C. 10693* (NY); Washington County, Lytle Ranch, T42S, R19W S6, 1 Aug 1985, *Higgins, L.C. 15979* (NY); Cache County, Logan, 20 Heritage Cove, 1 Oct 2006, *Holmgren, N.H. 15471* (NY); Salt Lake County, Salt Lake County. Salt Lake Valley, 21 Jul 1880, *Howard, O. s.n.* (UT); Salt Lake County, Salt Lake County. Salt Lake Valley, Jul 1880, *Howard, O. s.n.* (UT); Salt Lake County, Murray, 8 Aug 1917, *Jones, W.W. 445* (GH); Utah County, 1 mile below Vivian Park, Provo Canyon, 7 Sep 1944, *McKnight, K. 10707* (UC); Uintah County, Vernal, garden of Duane Nelson, just N of Vernal cemetery, 29 Aug 1983, *Neese, E.J. 15025* (NY); Salt Lake County, Salt Lake Co. Utah: Salt Lake City, University of Utah Campus, 15 Sep 1975, *Player, G. s.n.* (UT); Cache County, Cache County: Hyrum, south section of city, overlooking Hyrum Reservoir, 20 Jul 1982, *Shaw, R.J. 2994* (UT); Weber County, Taylor, 2550 S. 5100 W, 10 Aug 2000, *Spencer, J. 1556* (NY); Salt Lake County, omato patch belonging to Mr. P. K. Stubbs at 6783 So. 2300 E. Cottonwood Heights, 11 Sep 1966, *Stubbs, W.J. 48* (NY); Sevier County, Tushar Mountains, Clear Creek Canyon, 31 Jul 1988, *Taye, A. 4120* (NY); Washington County, 1/2 mile N of

Lytle's ranch, in Beaver Dam Wash, 25 Jun 1985, *Thorne, K.H. 3888* (NY); Salt Lake County, Parley's Canyon, Salt Lake County, 21 Sep 1957, *Vickery, R. 1472* (UT); Emmy County, San Raphael, 15 Apr 1931, *Williams, L. 5831* (MO); Salt Lake County, In pond, Mt. Olympus Park, Salt Lake Co. Utah, 9 Sep 1965, *Winburn, S. s.n.* (UT). **Vermont**: Chittenden County, Colchester, Mallets Bay Avenue, 30 Sep 1991, *Briggs, E.C. s.n.* (VT); Chittenden County, Colchester, near Malletts Bay Avenue, 1 Sep 2009, *Gilman, A.V. & Briggs, E.C. 9148* (NEBC); Chittenden County, Westford, 8 Oct 1969, *Meunier, A.A. s.n.* (VT); Chittenden County, Westford, 5 Oct 1969, *Meunier, A.A. s.n.* (NEBC); Windham County, West Brattleboro, Ames Hill Road, Robb Farm Vegetable Garden, 25 Sep 1981, *Pofcher, H.I. s.n.* (NEBC). **Washington**: Jefferson County, Port Townsend, vic. Pt. Townsend, 12 Aug 1899, *Barber, M.A. 158* (GH); Okanagan County, Ophir, Jul 1897, *Elmer, A.D.E. 525* (NY); Seattle, Harbor Island, 1 Oct 1941, *Eyerdam, W.J. 1601* (UC); Grays Harbour County, Montesano, 1917, *Grant, J.M. s.n.* (K); Island County, Langley, South Spit, Jul 1963, *Grant, J.M. 367* (DUKE); Snohomish County, Warm Beach Sr [Senior] Commuity, Stanwood, 20 Aug 2009, *Haberman, C. s.n.* (WTU); Waitsburg, 9 Jun 1897, *Horner, R.M. B-374* (GH); King County, Seattle, Aug 1926, *Knowlton, C.H. s.n.* (GH); Thurston County, At Mud Bay, about five miles west of Olympia, 29 Aug 1936, *Meyer, F.G. 797* (GH, MO); Whatcom County, Lummi Indian Reservation, 23 Aug 1937, *Muenscher, W.C. 8397* (UC); Jefferson County, Port Townsend, 17 Oct 1937, *Otis, I.C. 2343* (UC); Whitman County, vicinity of Pullman, 16 Oct 1938, *Sharsmith, C.W. 3982* (GH, K, MO, NY); Klickitat County, Bingen, 10 Oct 1894, *Suksdorf, W. 1480* (F, GH, NY, UC); Klickitat County, near Bingen, 10 Oct 1894, *Suksdorf, W. 2317* (GH, NY, UC); San Juan County, San Juan Islands, Cattle Point, 25 Jun 1917, *Zeller, S.M. & Zeller, E.E. 1143* (GH, MO, NY). **Wisconsin**: Dane County, just south of WARF building parking lot, Univ. of Wisconsin campus, Madison, 30 Sep 1989, *Iltis, H.H. 30226* (GH, MO, NY); Portage County, Corner of Willow Springs Dr. & Dewey Dr, 6 mi due N of Stevens Point, 2 Sep 2010, *Seidl, A.C. s.n.* (NY). **Wyoming**: Albany County, Laramie, 24 Sep 1896, *Nelson, A. 2804* (GH, MO, NY).

12. *Solanum pruinosum* Dunal
MEXICO. "de Salamanca y Nueva España", *Expedición Malaspina*, *s.n.* (MA); [almost certainly central Mexico], *Sessé, M. & Mociño, J.M. 1511* (MA); [almost certainly central Mexico], *Sessé, M. & Mociño, J.M. 5341* (MA); sin. loc, Nov, *Without Collector s.n.* (K); sin. loc, *Without Collector s.n.* (W). **Distrito Federal**: malezas de Ixtapalapa, 25 Jul 1962, *Madrigal S, X. s.n.* (MEXU); about 10km SW of Mexico City, Cerro de Estrella, DF, 28 Oct 1930, *Reddick, D. 190* (BH); Chapultepec, *Schmitz, A. 1047* (W); Santa Cecilia, delegación de Xochimilco, 12 Feb 1977, *Ventura A, A. 2588* (ASU, CORD, MEXU, MO, NY). **Guanajuato**: San Luis de la Paz, 4 km de Mesas de Jesús, camino a El Verge, 'Puerto Blanco', 30 Sep 1998, *Carranza, E. 5667* (MEXU); Salvatierra, [manantial de Salvatierra -visited in 1791], *Expedición Malaspina*, *s.n.* (MA); La Mina, municipio de Xichú, 1990, *Ventura V, E. & López, E. 7963* (MO); Xichú, Rancho Beltran, 10 km al Oeste de Xichú, 9 Dec 1988, *Ventura, E. & López, E. 6484* (MEXU); Xichú, Agua Zarca, 15 km al Este de Xichú, 21 Sep 1989, *Ventura, E. & López, E. 7388* (MEXU); Xichú, El Potrerillo, 9 May 1991, *Ventura, E. & López, E. 9194* (MEXU). **Hidalgo**: 1 km de la desviación a Sta. Mónica por la carr. Alumbres -Tianguistengo, 22 Jun 1983, *Torres C, R. & Hernández, H. 3065* (MEXU, NY). **Jalisco**: Bolaños, Arroyo El Nalgaso, 16 km al NW de Bolaños, camino a Tuxpan de Bolaños, 6 Jul 1996, *Calónico-Soto, J. & Flores-Franco, G. 2528* (MEXU). **Michoacán**: Morelia, 2.5 km al NO de Tiripetío, pie de monte del Cerro Aguila, 13 Sep 2010, *Cornejo Tenorio, G. et al. 3552* (MEXU). **Nuevo León**: Dulces Nombres, Nuevo Leon, and just east of border into Tamaulipas, 14 Jul 1948, *Meyer, F.G. & Rogers, D.J. 2781* (E, K). **Oaxaca**: huertas de Oaxaca, Nov 1935, *Conzatti, C. 4736* (BM, MEXU); San Pedro Quiatoni, Camino Lachibarra -San Pedro Quiatoni. Mpio San Pedro Quiatoni. Dto. Tlacolula. Region Valles Centrales, 15 Aug 1984, *Manzanero M, G.I. 1009* (MEXU); Villa Tejupam de la Unión, Mun. Tejupan 5 km al NE de Tejupan, carretera a Coixtlahuaca. Distrito Coixtlahuaca, 20 Nov 1991, *Tenorio L, P. 18134* (MEXU, NY). **Puebla**: Cuetzalan del Progreso, San Miguel Tzinacapan, in center, in house yard of Ernesto Vázquez, 1 Aug 2014, *Amith, J.D. JDA-2084* (BM); Zautla, Rosa de Castilla, La Loma, cerca de la calle Puebla, 27 Sep 2014, *Amith, J.D. & Jiménez Chimil, M. JDA-30248* (BM); Mpio de Tatoxcac. Km 15 de la carretera a Cuetzalán, 23 Jun 1976, *Inzunza, F.M. 128* (MO). **Querétaro**: San Juan del Río, Near San Juan del Río, 18 Aug 1905, *Rose, J.N. et al. 9602* (MEXU, NY, US); San Juan del Río, near San Juan del Río, 18 Aug 1903, *Rose, J.N. et al. 9613*

(US); Landa, Cerca de Tres Lagunas, 22 Jun 1988, *Rzedowski, J. 36671* (MEXU); San Joaquín, + 4 km al N de San Joaquín por el camino a El Durazno, municipio de San Joaquín, 28 Aug 1999, *Zamudio R, S. 11120* (MEXU). **San Luis Potosí**: 23 miles East of Ojuelos, MEX 70, 11 Sep 1978, *D'Arcy, W.G. 11877* (MO); 30 miles E of San Luis Potosí along hiway 86 to Valverde [Rioverde?], 13 Jul 1963, *McGregor, R.L. et al. 571* (US); 1879, *Schaffner, J.G. 408* (BM, GOET, NY, P, US); sin. loc, 1877, *Schaffner, J.G. 691* (GH, MEXU). **Tamaulipas**: Mina de Asbesto, a 25 km al Oeste de La Libertad y a 35 km de Ciudad Victoria, 9 Dec 1987, *González Medrano, F. 16948* (MEXU); Hidalgo, 7 km al SE de Puerto Purificación, 6 Jun 1990, *González-Medrano, F. et al. 17494* (MEXU); Tula, Ejido Ricardo Garcia o La Presita km 66 carretera Tula-Victoria, 18 Jun 1986, *Martínez, M. 1211* (MO). **Veracruz**: Huayacocotla, camino al Tine, Huayacocotla, 21 Dec 1970, *Hernández Magaña, R. & Hernandez, Y. .Y. de 958* (F, MEXU). **Zacatecas**: Llano de Zaragosa, among cerros 6 miles southeast of Carboneras, 28 Sep 1948, *Gentry, H.S. 8523* (GH, US); Monte Escobedo, 2 km al Oeste de Monte Escobedo, 28 Aug 2005, *Rodríguez, A. et al. 4461* (MEXU, MU).

13. ***Solanum pseudogracile*** **Heiser**
UNITED STATES OF AMERICA. **Alabama**: Baldwin County, causeway US98-90 over Mobile Bay between Battleship Park and Spanish Ft, 19 Sep 1975, *Kral, R. 56564* (GH, MO). **Florida**: Putnam County, Johnson, 28 Apr 1897, *Barnhart, J.H. 2062* (NY); Pinellas County, Abe Landes' ranch, Ulmerton Blvd. 2 miles E of Largo, 20 Feb 1952, *Bechtel, A.R. 17733* (NY); Alachua County, along s. shore of Lake Alice, University of Florida farm, s.w. of campus, Gainesville, 3 Oct 1969, *Becker, J. 2352* (MO); Pinellas County, extreme N tip of Coquina Key, St. Petersburg, 25 Mar 1967, *Beckner, J. 1646* (MEXU, MO); Franklin County, Apalachicola, Feb, *Biltmore Herbarium*, *912 d* (NY); Duval County, near dunes of west part of the peninsula at north side of eastern end of St. Johns estuary, 25 Dec 1965, *Blake, D. s.n.* (IND, VSC); Escambia County, Pensacola, RR right-of-way in downtown Pensacola, 31 Jul 1941, *Brinker, R. 374* (MO); Brevard County, Town Park, Melbourne Beach, 27 Nov 1974, *Burton, D.L. 630* (IND); Brevard County, west of parking lot at Town Park, Melbourne Beach, 27 Nov 1974, *Burton, D.L. 630 b* (IND); Indian River County, McLarty State Museum (near Sebastian Inlet), 27 Nov 1974, *Burton, D.L. 633* (IND); Indian River County, McLarty State Museum (near Sebastian Inlet), 27 Nov 1974, *Burton, D.L. 633 a* (IND); Indian River County, McLarty State Museum (near Sebastian Inlet), 27 Nov 1974, *Burton, D.L. 633 d* (IND); Orange County, Winter Park, 10 Feb 1889, *Canby, W.M. s.n.* (GH); sin. loc, *Chapman, A.C. s.n.* (GH); sin. loc, *Chapman, A.C. s.n.* (GH); Franklin County, Apalachicola, *Chapman, A.C. s.n.* (GH); Duval County, Jacksonville, 12 Apr 1897, *Churchill, J.R. 713 [c]* (MO); Palm Beach County, Jupiter Island, south end, 27 Jul 1956, *Cooley, G.R. et al. 4800* (GH, USF); Hernando County, one mile S of county line N of Chinsegut Hill, 24 Apr 1959, *Cooley, G.R. & Eaton, R.J. 6666* (GH, USF); Lee County, Sanibel Island, 11 Feb 1967, *Cooley, G.R. 11850* (AK, USF); river above Jacksonville, 12 Jun 1896, *Curtiss, A.H. 5594* (GH); Nassau County, beside parking lot, Amelia beach, 1 Jul 1967, *D'Arcy, W.G. 1670* (MO); Indian River County, 3 mi S of Sebastian Inlet, in hammock behind foredune on Indian River Island, 5 Aug 1968, *D'Arcy, W.G. 2930* (GA, MO); Indian River County, bordering the ocean just north of Vero Beach, 28 Dec 1940, *Deam, C.C. 60240* (IND); Highlands County, Highlands Hammock State Park about 7.5 miles southwest of Sebring, 23 Jan 1946, *Deam, C.C. 64063* (IND); Highlands County, southwest side of Highlands Hammond State Park about 7.5 miles southwest of Sebring, 30 Jan 1946, *Deam, C.C. 64100* (IND); Tampa, 8 Aug 1898, *Ferguson, A.M. s.n.* (MO); Orange County, sin. loc, 14 Jul 1902, *Fredholm, A. 5405* (GH); Brevard County, Okechobee region, 3 Aug 1903, *Fredholm, A. 5914* (GH); Hillsborough County, sin. loc, 5 Mar 1904, *Fredholm, A. 6301* (GH); Taylor County, Aucilla Wildlife management area, E of the Econfina River, 14 Mar 1972, *Godfrey, R.K. 71259* (MO); Escambia County, Gulf Islands National Seashore, Fort Pickens Unit. Eastern end of main campground area.; Fort Pickens Unit, Gulf Islands National Seashore, 28 Apr 2005, *Gunn, S.M. & Ferguson, D.M. FP-84* (LSU); Lee County, Cayo-Costa Island, 4 Jan 1977, *Herwitz, S.R. 206* (GH); Volusia County, unimproved road near Drum Ave. just W of Rte A1A, Bethune Beach, just S of New Smyrna Beach, 18 Mar 1983, *Hill, S.R. 12641* (GH); Highland County, Sebring, 4 Mar 1924, *Hunnewell, F.W. 9038* (GH); Highlands County, Hooker's Hammock near Sebring, 17 May 1927, *Hunnewell, F.W. 10490* (GH); Palm Beach County, Jupiter, mouth of Loxahatchee River, 15 Nov 1956, *Jones, P. 921* (A); Monroe County, Big Pine Key, SE Hammock, 1 Feb 1937, *Killip, E.P. 32091* (GH, US); Citrus County, bank of Withlacoochee River, ca. 1.5 mile E

of Pineola, 3 Dec 1955, *Kral, R. 1905* (GH); Martin County, between causeway to Stuart Beach and House of Refuge, 15 Aug 1972, *Kral, R. 48060* (MO, NY); Martin County, Rand Estate, ca. 2 mi s. House of Refuge and e. of Stuart, 19 Jan 1973, *Kral, R. 49252* (MO); Monroe County, 19 Sep 1973, *Kral, R. 51804* (MO); Martin County, Rand estate, c. 2mi S. House of Refuge and E of Stuart, 19 Jan 1973, *Kral, R. 59247* (MO); Putnam County, Palatka, 23 Feb 1892, *Leeds, B.F. s.n.* (F); Citrus County, along Fl. route 44, E of Gospel Island Road, 20 Apr 2012, *Longbottom, W.D. 17139* (NY); Brevard County, Indianola, 25 Jan 1907, *Mell, C.D. s.n.* (US); Monroe County, Plantation Key, 28 Dec 1963, *Meriläinen, J. & Roe, K. 97* (H, WIS); Broward County, north of Long Island, 25 Jan 1930, *Moldenke, H.N. 507* (K, MO, NY); Collier County, S of Tamiami Trail, 1 Apr 1930, *Moldenke, H.N. 874* (K, MO, NY); Lake County, vicinity of Eustis, 1 Jul 1894, *Nash, G.V. 1250* (E, GH); Lake Jovita, 20 Feb 1927, *O'Neill, H. s.n.* (MO); Polk County, 26 Apr 1894, *Ohlinger, L.B. 454* (MO); "East Florida, Indian River", *Palmer, E. s.n.* (BRU, NY); East Florida, Indian River, 1874, *Palmer, E. 369* (MO, NY); Charlotte County, off Rt 41 near Point Charlotte, 11 Jul 1976, *Reed, C.F. 102464* (MO); Monroe County, Big Pine Key, southeast hammock, 22 Feb 1952, *Robertson, W.B. 54* (GH); St. Johns County, Anastasia Island, May 1848, *Rugel, F. 69* (BM, F, GH); Manatee County, "Manatee", Jul 1845, *Rugel, F. 302* (BM, F, GH, MO, NY); Broward County, Fort Lauderdale, 19 Nov 1903, *Small, J.K. 937* (NY); St. Johns County, St. Augustine, 2 Dec 1904, *Small, J.K. 2311* (NY); Palm Beach County, Hammocks, Kreamer Island, Lake Okeechobee, 11 Nov 1913, *Small, J.K. & Small, G.K. 4193* (K, NY); Martin County, Everglades, eastern shore of Lake Okeechobee, Pelican Lake to Cypress Creek, 11 Nov 1913, *Small, J.K. & Small, G.K. 4325* (NY); Dade County, Humbugus Prairie, Everglades, 18 Feb 1915, *Small, J.K. & Mosier, C.A. 5573* (NY); De Soto County, shores of Lake Hicpochee, 7 May 1917, *Small, J.K. 8181* (GH, NY); western shore of Lake Okeechobee, fisheating creek to three-mile canal, 7 May 1917, *Small, J.K. 8216* (GH, MO); Palm Beach County, eastern shore of Lake Okeechobee, 9 May 1917, *Small, J.K. 8258* (NY); Volusia County, Daytona Beach, 8 May 1918, *Small, J.K. 8681* (NY); Highlands County, shore of Lake Nancesowee, 8 Dec 1923, *Small, J.K. et al. 11079* (MO); Manatee County, Longboat Key, 27 Apr 1900, *Tracy, S.M. 6829* (BM, E, GH, NY); Charlotte County, on Little Gasparilla Island, 28 Jan 1983, *Walker, R.L. 354* (MO); Duval County, Fort George Island, 3 miles northwest of mouth of St. Johns River; Rollins Sanctuary nature walk, east side of Ft. George Id, 16 Nov 1974, *Ward, D.B. 8787* (IND); Levy County, small island of dog-tooth limestone W of Hodges Island, SW corner of Sec 35, T16S, R 15 E, Withlacoochee Bay, 4 May 1959, *Wood, C.E. & Wilson, K.A. 9197* (GH); Flagler County, on Florida route A1A, ca. 6.3 mile N of Flagler beach, 9 May 1959, *Wood, C.E. & Wilson, K.A. 9316* (GH). **Georgia**: Camden County, SE part of county, 8 Sep 1963, *Blake, D. s.n.* (GA); Glynn County, Jekyll Island, next to road in North Picnic Area, 13 Jun 1972, *Burton, D.L. 565* (IND); Glynn County, next to road in North Picnic Area, Jekyll Island, 13 Jun 1972, *Burton, D.L. 565 b* (IND); Glynn County, Jekyll Island, 25 Nov 1974, *Burton, D.L. 627* (IND); Chatham County, between Tybee Island and Savannah, 24 Oct 1953, *Duncan, W.H. 17439* (GA); McIntosh County, SW section of Sapelo Island, 14 Oct 1956, *Duncan, W.H. 20636* (GA, VSC); Chatham County, along Grove Point Road about 1.1 miles southeast of Atlantic Coast Line Railroad, 16 Jul 1958, *Duncan, W.H. 21344* (GA, GAS); Glynn County, just NE of entrance road to St. Simon's Island, 4 Oct 1974, *Duncan, W.H. 29142* (IND); Glynn County, Jekyll Island, 19 Oct 1974, *Duncan, W.H. 29165* (GA); Glynn County, S end of Jekyll Island, 11 Sep 1982, *Duncan, W.H. 30736* (GA, TTRS); Chatham County, Fort Pulaski National Monument, along the old Savannah-Tybee Roailroad Multipurpose Trail, McQueens Island, 10 Apr 1998, *Govus, T. 997* (GA); Camden County, bank of big slough (#7), Little Cumberland Island, 15 Aug 1971, *Richardson, J.I. 27* (GA); McIntosh County, Butler Island, 27 May 1909, *Smith, H.H. 2180* (F, W); Early County, Nantz Spring, 1 mile east of Arlington, 24 Mar 1949, *Thorne, R.F. & Muenscher, W.C. 9133* (GA, GH, NY, UC, US); McIntosh County, 2 miles S of Darien, 25 Jul 1937, *Wiegand, K.M. & Manning, W.E. 2318* (GH). **Louisiana**: Lafourche Parish, On the property of Gheens Golden Ranch Plantation, at the boat landing by #2 pump canal, Gheens Golden Ranch Plantation, 4 Mar 1992, *Bossert, D.C. 9* (LSU); Jefferson Parish, Grand Isle, 28 Apr 1928, *Brown, C.A. 1986* (LSU); Lafourche Parish, 12 mi S of Raceland, 2 Apr 1955, *Cockull, M. 13* (GH); Bienville Parish, on north side of Gentry Hill at roads to firetower and to Driskill Mountain beside La 507, Sec 4, T16N & Sec. 33, T17N, R5W, 21 Nov 1995, *Dale Thomas, R. 148045* (MO); Beauregard Parish, Beside N to S railroad tracks south of US 190 in downtown DeRidder, 7 Dec 1996, *Dale Thomas, R. 153473* (MO); St. Charles Parish, Bonnet Carre Spillway. Near Airline Hwy and east guide levee, 2 Nov 1973, *Montz, G.N. 2937* (LSU); Jefferson

Parish, Grande Terre Island, Grand Terre Island, salt marsh behind levee and beach near center of island on Gulf side.; Grand Terre Island, 11 Jun 2003, *Reid, C. 4515* (LSU); Jefferson Parish, Elmer's Island, 6 Sep 1975, *Urbatsch, L.E. 1903* (LSU); Jefferson Parish, Grand Isle, Jul 1937, *West, E.M. 95* (LSU). **Mississippi**: Pearl River County, 4.3 miles SE of Poplarville, junction of I-59 and MS-53, 8 Sep 1994, *Bryson, C.T. 14391* (NY); Jackson County, Gulf Islands National Seashore, Horn Island. Slash pine forest southwest of Ranger Station and west of Ranger Station trail near south shore.; Horn Island, Gulf Islands National Seashore, 10 May 2005, *Gunn, S.M. & Ferguson, D.M. HI-5* (LSU); Jackson Parish, Gulf Islands National Seashore, Horn Island. Transition zone from southern boundaries of extensive marsh system to grassy dune system in sight of south shore, Horn Island, Gulf Islands National Seashore, 23 Aug 2005, *Gunn, S.M. & Ferguson, D.M. HI-104* (LSU); Jackson County, Gulf Islands National Seashore, Petit Bois Island. Thin strip of slash pine and dunes running east/west bordered on both sides by extensive marsh.; Petit Bois Island, Gulf Islands National Seashore, 7 May 2006, *Gunn, S.M. & Ferguson, D.M. PB-111* (LSU); Harrison County, Biloxi, Canaan, Bayview Ave. fishing dock area at edge of inlet of Back Bay, 20 Jul 2013, *Nee, M. & McClelland, D. 60216* (MO, NY); Harrison County, Biloxi, historical site of Old Brick House of Mayor John Henley, Bayview Ave. on Back Bay, 20 Jul 2013, *Nee, M. & McClelland, D. 60224* (MO, NY). **North Carolina**: Onslow County, open sands behind beach dunes, north of Surf City, along North Carolina Highway 210, 16 Jul 1960, *Bell, C.R. 17061* (IND, IND, NCU); Brunswick County, Sunset Beach, 23 Nov 1968, *Borg, P. s.n.* (H); Brunswick County, s. end of co. rd. 1104, S of Long Beach, 17 Jun 1966, *Bradley, T. & Stevenson, J.A. 3314* (BM, E, H, MISS, UBC); Brunswick County, Smith's Island, clearing at lighthouse, Baldhead, 27 Aug 1949, *Fox, W.B. & Godfrey, R.K. 3243* (GH); Pender County, 1 miles N of New Topsail Beach, 1 Jul 1950, *Fox, W.B. & Boyce, S.G. 3732* (DUKE, GH, MO); Carteret County, Bogue Banks, 5 Aug 1949, *Godfrey, R.K. 49808* (MO); Hyde County, Ocracoke Island, 13 Oct 1898, *Kearney, T.H. 2267* (US); New Hanover County, Wilmington, Gingerwood Drive, just NW of junction of Hwy. 117 and Hwy Business 17, 29 Jul 2013, *Nee, M. & McClelland, D. 60279* (MO, NY); New Hanover County, Wilmington, Gingerwood Drive, just NW of junction of Hwy. 117 and Hwy Business 17, 29 Jul 2013, *Nee, M. & McClelland, D. 60281* (MO, NY); Pender County, Topsail Island, Barwick Ave, 29 Jul 2013, *Nee, M. & McClelland, D. 60292* (GH, MO, NY); Hyde County, Ocracoke Island, 24 Jun 1938, *Wells, B.W. & Shelbourne, V. 4766* (GH); Onslow County, Hammocks Beach State Park, Bear Island, eastern third of island, 24 Jul 1970, *Wilbur, R.L. 12290* (DUKE); Carteret County, Bogue Bank, western end about 4 miles west of Emerald Isle, 25 Jul 1970, *Wilbur, R.L. 12332* (DUKE); Carteret County, Radio Island, between Beaufort (Pivers Island) and Morehead City, 1 Oct 1983, *Wilbur, R.L. 33986* (DUKE); Brunswick County, Holden Beach, in western third of the island at about the 1000 block along ocean drive, 22 Jun 1988, *Wilbur, R.L. 48332* (DUKE); Onslow County, Hammocks Beach State Park, Bear Island, near Swansboro and just W of Bogue Bank, 6 Sep 1997, *Wilbur, R.L. & Alford, M. 69099* (DUKE). **South Carolina**: Charleston County, 5.2 miles from end of SC-174, vicinity of Edisto Beach State Park, 21 Jul 1957, *Ahles, H.E. s.n.* (NCU); Berkeley County, 0.5 mile W of Mt. Holly on County 45, 5 Jul 1957, *Ahles, H.E. & Haesloop, J.G. 30705* (NY); Beaufort, 23 Apr 1917, *Batchelder, C.F. 5001* (GH); Beaufort County, north end of Harbor Island north of U.S. Hwy. 21, 27 Jun 1956, *Bell, C.R. 3788* (NCU); Beaufort County, Hunting Island State Park, 6 Sep 1956, *Bell, C.R. 4782* (GH, NCU); Beaufort County, just off US 21, 1.7 miles north of Gray's Hill (or 4.5 miles north of junction with SC 116), 24 Nov 1974, *Burton, D.L. 623* (IND); Beaufort County, 24 Nov 1974, *Burton, D.L. 623 a* (IND); Beaufort County, just off US 21, 1.7 miles north of Gray's Hill (or 4.5 miles north of junction with SC 116), 24 Nov 1974, *Burton, D.L. 623 b* (IND); Beaufort County, just off US 21, 1.7 miles north of Gray's Hill (or 4.5 miles north of junction with SC 116), 24 Nov 1974, *Burton, D.L. 623 d* (IND); Beaufort County, just off US 21, 1.7 miles north of Gray's Hill (or 4.5 miles north of junction with SC 116), 24 Nov 1974, *Burton, D.L. 623 e* (IND); Beaufort, Bank of the Bay, 13 Apr 1917, *Churchill, J.R. s.n.* (GH); Charleston County, Charleston, 1858, *Gibbes, L.R. s.n.* (NY); Charleston County, Salt marsh on leeward side of Isle of Palms, 27 Oct 1970, *Leonard, S.W. 4363* (NCU); Georgetown County, just N of NE point of Upper Pine Ridge Pond, South Island, 14 May 1990, *Nelson, J.B. 8945* (MO); Georgetown County, Roadside Pawley's Island, 13 Jun 1957, *Radford, A. s.n.* (NCU); Charleston County, Charleston, in city, 29 Apr 1912, *Robinson, B.L. 207* (GH). **Texas**: Harris County, Hog Island, 14 Apr 1934, *Cory, V.L. 8154* (GH); Galveston County, Galveston, 8 Aug 1902, *Reverchon, J. 3241* (MO); San Marcos, 11 Jun 1897, *Trelease, W. s.n.* (MO).

14. ***Solanum retroflexum*** **Dunal**
UNITED STATES OF AMERICA. **California**: Solano County, University of California-Davis, S bank of Putah Creek, ca. 50 yards E of University Avenue bridge, 23 Jul 1958, *McCaskill, J. & Onaga, K. 582* (AK); Los Angeles County, Los Angeles Basin region, Los Angeles, Watts area, 658 East 111th Place between Stanford Ave. and Avalon Blvd. (Inglewood 7.5;Q;, 16 Sep 2009, *Sanders, A.C. et al. 37173* (MO, NY). **Illinois**: Cook County, Chicago, 4200 N. Hazel St, 28 Aug 1982, *Nee, M. 18259* (BM, CORD);

15. *Solanum sarrachoides* Sendtn.
UNITED STATES OF AMERICA. **Arkansas**: Washington County, Ozark Plateau, Springfield Plateau Ecoregion, Fayetteville, Woolsey Prairie, mitigation site for City of Fayetteville Sewage Treatment Plant on Broyles Road, Fayetteville 7.5' quadrangle, 21 Oct 2007, *Witsell, T. 07-605* (MO, VSC). **Connecticut**: New London County, New London, Connecticut College Arboretum, 23 Sep 2005, *Connolly, B.A. s.n.* (CONN); Tolland County, Mansfield Center, 3 Oct 2012, *Connolly, B.A. s.n.* (CONN); Tolland County, Mansfield Center, 3 Oct 2012, *Connolly, B.A. s.n.* (CONN). **Illinois**: McDonough County, Argyle Lake State Park, 28 Oct 1987, *Henry, R.D. & Scott, A.R. 5764* (W); **Kansas**: Anderson County, 3 mi s Garnett, 3 Aug 1979, *McGregor, R.L. 31057* (MO). **Maryland**: Baltimore County, Canton, Baltimore, 20 Sep 1957, *Reed, C.F. 39318* (MO). **Missouri**: Bollinger County, T29N, R8E, W2, NW4, Sec. 5. Gipsy Quadrangle. Terraces of the Castor River, east side of river, adjacent to State Hwy. Y, 4 Oct 2000, *Brant, A.E. 4590* (MO); Saint Charles County, Augusta katy Trail, 11 Oct 1990, *Christ, A. s.n.* (MO); Saint Louis City, 6573 Scanlan Street, 18 Jun 2003, *Davidse, G. 38975* (MO); Pettis County, Along U.S. Highway 65, about 1 mi S of Sedalia, 27 Aug 1992, *Henderson, N.C. 92-336* (MO); Franklin County, Whiskey Creek Sheep Farm at 530 W Whiskey Creek Road, 3.5 mi SW of Krakow, 240 m W of barn, 2 m E of fence on W property line, 25 Jun 2007, *Holmberg, N.J. 2309* (MO); Ripley County, Mud Puppy Natural Area, off N end of Highway BB, ca. 3 mi N of Flatwoods, 11 Aug 1993, *Hudson, H.S. 148* (MO); Saint Clair County, Schwartz Prairie, ca. 5 miles south/southwest of Roscoe. SW1/4 sec. 1 T36N R27W, 25 Sep 1998, *Ladd, D. s.n.* (MO); Cedar County, Wah' Kon-Tah Prairie Preserve, ca. 1 mile northeast of Eldorado Springs. NE1/4 NE1/4 sec. 15 T36N R28W, 17 Jul 2000, *Ladd, D. 22294* (MO); Lawrence County, Rest area on eastbound lane of Interstate 44, 7.6 miles west of the Greene County line, 9 Aug 1991, *Lammers, T.G. & Vincent, M.A. 8400* (MO, NY); Cole County, United Community Cathedral Church, 5210 Cowan Dr. just south of Columbia, and ca. 2mi. SE of the inter. Of AC and Rt. 63; T47N, R12W, SE1/4 of the NW1/4 of the NE1/4 of Sec. 4; Columbia 7.5; Quad;, 2 Nov 2005, *McKenzie, P.M. s.n.* (MO); Saint Louis County, Bremen Avenue of freight yard of the Terminal Railroad association in the northeastern part of the yard, 4 Jul 1958, *Muehlenbach, V. 1436* (MO); Jasper County, Joplin, 12 Aug 1951, *Palmer, E.J. 52976* (F); Clay County, Martha Lafite Thompson Nature Sanctuary sand bar of Rush Creek, 15 Jul 1983, *Raveill, J.A. 1682* (MO); Cole County, Private property of George Syrigos, Walnut Springs trail, St. Thomas, 21 Aug 2012, *Seaman, C. 41* (MO); Greene County, Bois D'Arc Conservation Area, 4 mi. WSW of Willard and 10 mi. WNW of Springfield. Along service road at Farm Rd. 94 between Farm Rd. 53 and Farm Rd. 61, 30 Jul 1998, *Sikes, K.G. & Stone, J.R. 36* (MO); Boone County, Jefferson City; on grounds of Missouri Department of Conservation headquarters, 2901 West Truman Blvd.; landscaping bed near sign at entrance to headquarters, 9 Sep 2005, *Smith, T.E. s.n.* (MO); Saint Charles County, Near a springwater trickle at the foot of the embankment of the Missouri River (KATY) Trail, near Klondike, about 1.5 miles east of Augusta, 30 Sep 1990, *Sullivan, J.M. s.n.* (MO); Dallas County, Near the Scout Group Camp at Bennett Spring State Park, upstream from the Bennett Spring outlet, 15 Aug 1990, *Sullivan, J.M. s.n.* (MO); Jefferson County, 1.5 mi SE of Barnhart rocks near Mississippi River above railroad tracks, 29 Oct 1992, *Sullivan, J.M. 102992* (MO); Greene County, Outside Springfield waste ground on south National, 13 Jun 1976, *Summers, J.W. s.n.* (MO); Howell County, 2 mi W of West Plains on County Route CC, 1 Jul 1976, *Summers, J.W. 249* (MO); Howell County, 2 1/2 miles north of Moody, T22N R9W S 19 on Hwy E, 24 Jul 1990, *Summers, J.W. 3510* (MO); Phelps County, 2 mi. southwest of St. James railroad right-of-way, 5 Jun 1991, *Summers, J.W. 4481* (MO); Howell County, Elk Creek Cumberland Presbyterian Church and Cemetery, 1/4 mi S of Highway 160, 11 Nov 1999, *Summers, J.W. 9448* (MO); Howell County, Mark Twain National Forest, Willow Springs

Ranger District, ca. 4 1/2 mi W of Willow Springs on Indian Creek at Lowe's Ford, 30 Sep 2001, *Summers, J.W. 9897* (MO); Howell County, Peggie L. Skinner Farm on County Road 6070, 0.5 mi SW of West Plains city limits, 11 Oct 2012, *Summers, J.W. 10626* (MO); Lawrence County, Along I-44 eastbound, 3.7 mi W of Goose Creek, 7.6 mi W of Greene County line, 9 Aug 1991, *Vincent, M.A. & Lammers, T.G. 5004* (MO); Henry County, City of Clinton; Holiday Inn, ca. 1/4 mi W of State highway junction on S side of State Highway 7, 22 Sep 1996, *Yatskievych, G.A. & Summers, B. 96-88* (MO). **Montana**: Custer County, Miles City vicinity, 28 Aug 2001, *Heiule, E. s.n.* (MONT). **North Carolina**: Orange County, road embankment, Chapel Hill, 5 Aug 1959, *Ahles, H.E. 52986* (DD, UBC); Orange County, Chapel Hill, University of North Carolina Campus, 28 Aug 1961, *Ahles, H.E. 55038* (BM, CAL, DD, E, GH, H, MEXU, UBC, UC, VSC). **Oklahoma**: Seqouyah County, Tenkiller Ferry Reservoir, on Illinois River, about 3 miles NNW of dam site, 30 Oct 1954, *Iltis, H.H. 5215* (GH, WIS). **Rhode Island**: Washington County, Charlestown, Earth Care Farms, NE of intersection of Biscuit City Road and Wordens Pond Road, 2003, *Tucker, G.C. & Tucker, J.W. 13470* (NEBC). **Virginia**: Orange County, along the bank of the Rapidan River at US 522, 10 Oct 1996, *Bradley, T. & Tran, N. 27306* (GMUF); Fairfax County, Potamac River, just E of the American Legion (Cabin John) Beltway bridge, 2 miles N of McLean, 28 Sep 1991, *Fleming, G.P. 6150* (GMUF); Charlotte County, banks of the Roanoke (Staunton) River, ca. 0.8 miles SE of US 360 bridge, 2 miles WSW of Public Fork, Kerr Reservoir lands, 10 Oct 1997, *Fleming, G.P. & Wieboldt, T. 14371* (GMUF); Halifax County, Kerr Reservoir/Clover Wildlife Mangaement area, along the Roanoke (Staunton) River, just N of US 360 bridge, 2.4 miles W of Public Fork, 10 Oct 1997, *Fleming, G.P. 14383* (GMUF); Frederick County, along Abrams Creek Preserve Trail and W&W railroad, opposite White's Pond and 0.3 miles SE of Rt. 621 crossing, at western city limits of Winchester, VA, 27 Sep 2012, *Fleming, G.P. & Cooper, D. 15738* (VPI); Albemarle County, 0.6 miles SW of jct. Rtes 708 and 637, 1 Aug 2014, *Fleming, G.P. & Morgan, C. 16063* (VPI); Chesterfield County, Dutch Gap, on the James River, inland from river bank path, 21 Sep 1997, *Hayden, W.J. 3838* (URV); City of Richmond, Richmond, James River park, Belle Isle in the James River, westernmsot (upstream) end of island, 22 Nov 1997, *Hayden, W.J. 3946* (URV); Henrico County, Rocket's Landing, onthe James River, ca. 0.25 miles S of the Richmond City limit, 16 Jul 2008, *Hayden, W.J. 5351* (URV); Cumberland County, about 300 m ESE of jct of John Randolph Road (Rt 638) and Cooks Road (Rt 653), 28 Sep 2013, *Hayden, W.J. 5729* (URV); Louisa County, Along Christopher Creek at US 522 NW of Wares Crossroads, 10 Oct 1983, *Lam, G. & Bradley, T. 20045* (GMUF); Mecklenburg County, bank of Roanoke River (N shore) just below John R. Kerr dam and powerplant, 11 km SE of Boydton, 8 Oct 1997, *Wieboldt, T.F. & Fleming, G.P. 9823* (VPI); Albemarle County, A.B. Davenport residence, Junction of Dick Woods Road (Rte 637) and Rte. 708 about 4.5 km SW of Ivy (VA), 4 Oct 2003, *Wieboldt, T.F. 11353* (VPI); King and Queen County, Rte 629 at the Mattaponi River, 17 Oct 1999, *Williams, L. 244* (GMUF); City of Richmond, Old Manchester section of Richmond; end of Decatur Street at East 1st Street, on rail ballast, 100 yards S of Souther States Grain Elevator at S end of Mayo Bridge, 12 Oct 1987, *Wright, R.A.S. s.n.* (VPI). **Washington**: Snohomish County, Forest Crest Playfield, 5006 236th Street Southwest, Mountlake Terrace, 10 Oct 2015, *Olmstead, R.J. 2015-54* (WTU).

16. *Solanum scabrum* Mill.

UNITED STATES OF AMERICA. **Connecticut**: Tolland County, Mansfield, Storrs, Botanic Garden, University of Connecticut, 20 Jul 1912, *Schulze, A.F. s.n.* (CONN). **Illinois**: Cook County, Chicago, 4200 Hazel St, 28 Aug 1982, *Nee, M. 18248* (BM, CORD); Cook County, Chicago, 4200 Hazel St, 13 Aug 1983, *Nee, M. 27524* (BM). **Missouri**: Missouri Botanical Garden, St. Louis, 17 Jun 1935, *Fairburn, D. s.n.* (MO); St.Louis, 7546 Wise Avenue, Richmond Heights, 4 Aug 1940, *Milnes, R.E. s.n.* (MO). **Wisconsin**: Richland County, Grown in garden, 3 miles SE of Richland Center (T 10 N; R 1 E; NE1/4NE1/4 Sec. 35), 7 Oct 1977, *Nee, M. 14623 b* (BM); Richland County, 3 miles southeast of Richland Center, from Gurney Seed Co. Yankton, South Dakota "Garden Huckleberry 233", 7 Oct 1977, *Nee, M. 14623* (MO, RB); Richland County, Grown in garden, 3 miles SE of Richland Center (T 10 N; R 1 E; NE1/4NE1/4 Sec. 35), 7 Oct 1977, *Nee, M. 14629 b* (BM, CORD, MEXU, MO, RB); Richland County, Grown in garden, 3 miles SE of Richland Center (T 10 N; R 1 E; NE1/4NE1/4 Sec. 35), 23 Aug 1978, *Nee, M. 16063* (BM, CORD, MEXU, RB); Richland County, grown in garden 3 miles SE of Richland Center, seeds originally from "Hortus Botanicus

Universitatis, Nijmegen, Nederland", 22 Aug 1978, *Nee, M. 16081* (MEXU); Richland County, grown in garden 3 miles SE of Richland Center, seeds originally from "Hortus Botanicus Universitatis, Nijmegen, Nederland", 23 Aug 1978, *Nee, M. 16088* (CORD, K, MEXU, RB); Richland County, 3 miles SE of Richland Center, 3 Sep 1978, *Nee, M. 16099* (RB).

17. ***Solanum triflorum* Nutt.**
CANADA. Boundary Line Lat 49, 23 Aug 1874, *Burgers, ?, 1147* (K); South Plain. Assin, 27 Jul 1883, *Macoun, J. s.n.* (NY). **Alberta**: Calgary District, 1 Jul 1922, *[AHB], s.n.* (E); Graigmyle District, 1 Jul 1922, *AHB, s.n.* (K); Craigmyle District, S E 28 32 16 W 4, 25 Jun 1921, *Brinkman, A.H. 140* (NY); Craigmyle, SE 28 32 16 W 4, 3 Jul 1922, *Brinkman, A.H. 653* (F); Craigmyle District, 3 Jul 1942, *Brinkman, A.H. 5210* (UBC); University Heights, NW Calgary, 2 Sep 1969, *D'Arcy, W.G. 3717* (CM, EMC, MO, UTC, WIS); Newcastle (Drumheller), 27 Jun 1971, *D'Arcy, W.G. 5599* (MO); The Nose, Calgary, 2 Jul 1971, *D'Arcy, W.G. 5675* (MO); Transcanada, highway 2 miles east of Bassano, 7 Jul 1971, *D'Arcy, W.G. 5710* (H, MO); On gopher mound, 6 miles southeast of Tilley, 7 Jul 1971, *D'Arcy, W.G. 5711* (MO); *Dickson, H.L. & Dickson, D.L. 7156* (MICH); Calgary, 9 Jul 1982, *Dieren, W. van, & Dieren, D.M. van 687* (V); Northwest Calgary, 1969, *Hallworth, B. s.n.* (MO); Warner District, Off highway 36, south side of Chin Coulee Reservoir. 7-36-W4, 25 Jun 1992, *Macdonald, I.D. s.n.* (LEA); Calgary, 22 Jul 1897, *Macoun, J. 24020* (NY); *Malte, M.O. s.n.* (COLO); Lethbridge, 26 Jul 1911, *Malte, M.V. s.n.* (W); Calgary, North Hill, 18 Jul 1947, *McCalla, W.C. 9645* (UBC); Calgary, 31 Jul 1950, *McCalla, W.C. 11221* (UBC); Calgary, North Hill, 5 Sep 1950, *McCalla, W.C. 11335* (UBC); Vicinity of Calgary. Bow Valley, 27 Aug 1913, *Moodie, M.E. 111* (NY); vicinity of Calgary, 27 Aug 1913, *Moodie, M.E. 111* (F); Vicinity of Rosedale, Red Deer Valley, 17 Aug 1915, *Moodie, M.E. 1208* (F, MO, NY); *Scoggan, H.J. 16496* (MIN); Between Blairmore and Fort MacLeod, Highway 3, junction with RGE Road 280, 3 Aug 2004, *Spribille, T. & Wagner, V. 15164* (UBC). **British Columbia**: between Cranbrook and Marysville, Sep 1950, *Brink, V.C. 50-164* (UBC); Hanceville, Chilcotin, garden of Dan Lee, Aug 1944, *Cornwall, C.F. s.n.* (UBC); Ashcroft, 29 Jun 1907, *Cowles, H.C. 232* (F, MO); Near Tuts Office, Spences Bridge, Jun 1914, *Davidson, J. s.n.* (UBC); Spences Bridge, 15 Jul 1913, *Davidson, J. s.n.* (UBC); *Eastham, J.W. s.n.* (MIN); Cranbrook, 23 Jul 1938, *Eastham, J.W. s.n.* (F, UBC); Monte Creek, 12 Jun 1940, *Eastham, J.W. s.n.* (UBC); road to Kilpoola Lake, near Osoyoos, 28 Jul 1992, *Lomer, F. 92-117* (UBC); Greater Vancouver Regional District, Burnaby, Marine way near Byrne, 12 Sep 1988, *Lomer, F. 88-168* (UBC); East Kootenay, Wardner, 70 m SE of hte boat launch near corner of Wallace Street and Laurier Street, shore of Lake Koocanusa, 6 m from water line, 11 Jul 2015, *Lomer, F. 9610* (UBC); Penticton, Aug 1926, *Perry, F. s.n.* (UBC); Michel, 11 Sep 1914, *Robertson, W.E. 7407* (UBC, WTU); Upper Hat Creek, Marble Mountains, 14 Jul 1938, *Thompson, J.W. & Thompson, E.M. 485* (F, MO, NY, WTU). **Manitoba**: District de Brandon. Brandon. GraviÃ¨re, 12 Aug 1958, *Boivin, B. 12861* (NY); entre les deux Forts Garay [Garry], 18 Jul 1857, *Bourgeau, E. s.n.* (K); Fort Ellice [near junction of Assiniboine and Qu'Appelle Rivers], 19 Aug 1857, *Bourgeau, E. s.n.* (K); R. R. Douglas, 11 Aug 1883, *Christy, R.M. 100* (BM); 16 mi N of Portage la Prairie at village of Delta, 30 Jul 1953, *Dillon, S.T. 52* (WIS); High School Grounds, Russell, Russell Twp, 27 Aug 1972, *Garton, C.E. 15255* (H, MICH); Virden, 29 Jun 1957, *Krivda, W. V-338* (NY); Virden, 29 Jun 1957, *Krivda, W. V-343* (NY); Sep 1884, *Salmon, C.E. s.n.* (BM); *Scoggan, H.J. 10011* (MIN); Birtle, 7 Aug 1951, *Scoggan, H.J. 10224* (K). **Ontario**: Along C. P. R. [Canadian Pacific Railway]; Port Arthur, 8 Sep 1889, *Britton, N.L. s.n.* (NY). **Saskatchewan**: Rosetown-Biggar District, Beechy, 20 milles a l'ouest, 19 Sep 1960, *Boivin, B. s.n.* (LEA); District de Battlefords. Rutland, 14 Jul 1952, *Boivin, B. 9823* (NY); sin. loc, 1857, *Bourgeau, E. s.n.* (GOET); 'Palliser's Brit. N. Am. Expl. Expedition. Saskatchawan', 1857, *Bourgeau, E. s.n.* (E, NY); dans les terres remue par les Bleros ou chien de Prairie, 12 Sep 1857, *Bourgeau, E. s.n.* (K); sin. loc. (Palliser's Expedition), 1857, *Bourgeau, E. s.n.* (W); Battle Creek Ranger Station, Cypress Hills, Saskatchewan, 28 Jul 1947, *Breitung, A.J. 5135* (MO); Antler River Valley SE Saskatchewan. 9 mi. S and ca. 3.5 mi. E of Carievale, 30 Jul 1988, *Harms, V.L. 39676* (UTC); Breakmore, Collected along the line of the Great Pacific Railway, 18 Jul 1906, *Herriot, W. & Macoun, J. 78428* (BRU); Brownlee Alt. 2000 [No unit of measurement included with elevation], 7 Aug 1913, *Johnson, F.W. 1263* (NY); Swift Current, 1 Aug 1969, *Looman, J. 13176* (H); Saskatchewan plains, 12 Aug 1872, *Macoun, J. 1182* (K); Old Wives Creek. N.W.T. [probably now Saskatchewan -georef to Old Wives Lake], 28 Jul 1880, *Macoun, J. 1607* (BM); Assiniboia. Crane

Lake [The name given in this record as the province was not specified on the label but was inferred from the label's locality data.], 2 Jul 1894, *Macoun, J. 5814* (NY); Breakmore, 18 Jul 1906, *Macoun, J. & Herriot, W. 78428* (F); Antelope Plains [on label as "Northwest Terr."], 9 Aug 1883, *Macoun, J.M. s.n.* (CM); along Route 21, five mi. S of Maple Creek, 24 Aug 1959, *Swink, F.A. 3020* (F).

UNITED STATES OF AMERICA. Lat. 41°, 1862, *Hall, E. & Harbour, J.P. 467* (BM, BRU, GH, MO, NY, US, W); Yellowstone National Park [Wyoming and Montana], 11 Oct 1902, *Mearns, E.A. 4844* (US); Southern Utah, Northern Arizona, &c., 1877, *Palmer, E. 362 [b]* (NY, US). **Arizona**: Coconino County, Flagstaff, 13 Jul 1910, *Adams, J.N. s.n.* (WIS); 25 May 1896, *Blankinship, L.A. s.n.* (MO); Cochise County, Paradise, Chiricahua Mountains, 7 Sep 1907, *Blumer, J.C. s.n.* (E); Cochise County, Paradise, Chricahua Mountains, 7 Sep 1907, *Blumer, J.C. 1656* (F, L, NY); Tuba Oasis, 15 Jul 1920, *Clute, W.N. 126* (NY); Mohave County, Wolf Hole Water Encasement Area (Plants of the Arizona Strip District), 22 Jun 1978, *Coombs, R.E. & Bundy, C.E. 2456* (MEXU); Navajo County, Adamana to Navajo, 12 Sep 1903, *Griffiths, D. 5787* (US); Coconino County, Flagstaff, 26 Jul 1922, *Hanson, H.C. A 132* (F, MO, NY); Coconino County, Vicinity of Flagstaff, 7 Aug 1915, *Hitchcock, A.S. s.n.* (US); Coconino County, Flagstaff, 6 Aug 1884, *Jones, M.E. 4009* (BM, F, PAL, RM, US); Coconino County, Flagstaff, Motel Six near end of Hwy 17, 18 Sep 2004, *Landrum, L.R. 10999* (NY); South foot of Elden Mesa. [interpreted], 29 Jul 1901, *Leiberg, J.B. 5775* (US); Holbrook, 2 Jul 1896, *Luck, M. s.n.* (K); Coconino County, in the vicinity of Flagstaff, 2 Jul 1898, *MacDougal, D.T. 213* (E, F, NY, US); Coconino County, San Francisco Mountains, 23 Jul 1891, *MacDougal, D.T. 10915* (RM); Cochise County, Bowie, along Apache Pass rd. 4 miles S of jctn, with Bus I-10 in Bowie, 23 May 1995, *McGill, L.A. 6254* (MO); Coconino County, Coconino National Forest: northwest edge of Black Bill Park just E of U.S. Hwy 89 on Forest Road 776, ca 10.5 air mi NE of Flagstaff, 23 Aug 2006, *Nelson, B.E. & Fowler, J.F. 69649* (RM); Coconino County, Coconino National Forest: frontage road (old U.S. Hwy 66) along I-40 on Bellemont Flat, just W of Bellemont Exit and just N of Camp Navajo Army Depot, ca 10.5 air mi WNW of Flagstaff, 24 Aug 2006, *Nelson, B.E. & Fowler, J.F. 69749* (RM); Pima County, Bear Springs, 1869, *Palmer, E. s.n.* (US); Apache County, Hulsey Tank Escudilla Mountain, 8 miles east of Nutrioso, 28 Aug 1951, *Parker, K.F. 7640* (NY); Yavapai County, Yarnell Hill, 5 Oct 1930, *Peebles, R.H. 7430* (NY); Coconino County, Bonito Tank, 7 miles east of US 180, via USFS road 523, along USFS road 416, at the junction of USFS roads 416 and 416a, north of the San Francisco Peaks, White Horse Hills Quad, R7E T24N S6, 10 Aug 1984, *Ricketson, J. 1861* (MO); Coconino County, Flagstaff, 1 Sep 1883, *Rusby, H.H. s.n.* (F, NY); Grand Canon [Canyon], 20 Aug 1915, *Rusby, H.H. s.n.* (NY); Coconino County, Flagstaff, 1 Sep 1883, *Rusby, H.H. 247* (US); Coconino County, Flagstaff, A.T, 1 Sep 1883, *Rusby, H.H. 753* (F, MO, US); Coconino County, Flagstaff, 1 Sep 1883, *Rusby, H.H. 783* (NY); Coconino County, Flagstaff, 1 Sep 1883, *Rusby, H.H. 5981* (E, US); Coconino County, Bellemont, 10 Jul 1943, *Schallert, P.O. s.n.* (NY); Apache County, Dry hills. Navajo Indian Reservation, about the north end of the Carrizo Mountains [georef to Carrizo centre], 28 Jul 1911, *Standley, P.C. 7369* (US); [Unclear: Prescolt or Prescott?], 27 Aug 1894, *Toumey, J.W. s.n.* (NY); Grand Cañon, 12 Jul 1892, *Toumey, J.W. 402* (US); Navajo County, Holbrook, 2 Jul 1896, *Zuck, M. s.n.* (F, NY, US). **Arkansas**: Adams County, Derby, 25 Jul 1950, *Demaree, D. 29533* (GH). **California**: Nevada County, On the north side of Meadow Lake Road (Forest Road 18), north of the first set of three large culverts where the road crosses Jackson Creek, closest to the east end of Bowman Lake and west of the major campgrounds and buildings, 14 Aug 2001, *Ahart, L. 9152* (JEPS); Modoc County, On the north bank of Ash Creek, Ash Creek State Wildlife Area, 4500 feet south of County Road 87, 2.7 miles (air) west of Adin, 9.1 miles (air) northeast of Bieber, 25 Aug 2008, *Ahart, L. & Dittes, J. 15329* (JEPS); Modoc County, Southeast side of Rose Creek, 700 feet southwest of confluence of Rose Creek and Pit River, on the northwest side of unnamed road, northeast of wire gate, 2710 feet east of intersection with County Road 85a, 9.3 miles (air) north-northeast of Lookout, 26 Aug 2008, *Ahart, L. & Dittes, J. 15339* (JEPS); Modoc County, Parking Area west of the old barn, on the west side of elkins Lane, Ash Creek State Wildlife Area, 320 feet north of Lassen County Line, about 4.4 miles (air) west of Adin, about 7.2 miles (air) northeast of Bieber, Big Valley, 30 Aug 2010, *Ahart, L. & Dittes, J. 17083* (JEPS); Mono County, 8 1/2 mi sw of Bodie (Mormon Ranch), Mormon Ranch, 20 Jul 1945, *Alexander, A.M. & Kellogg, L. 4503* (UC); Los Angeles County, Claremont, Grounds of Rancho Santa Ana Botanic Garden, along Indian Hill Blvd, 12 Jul 1988, *Boyd, S. 2534* (BM, MEXU, UC); Siskiyou County, Paynes Cove,

Modoc national Forest, 6 Aug 1929, *Cronemiller, F.P. s.n.* (JEPS); Modoc County, 2 mi NW of Alturas, 10 Sep 1941, *Drouet, F.E. & Richards, D. 4131* (F); Modoc County, hills 2 mi NW of Alturas, 10 Sep 1941, *Drouet, F.E. & Richards, S. 4131* (UC); Modoc County, 2 mi above Cedarville (Cedar Camp); Cedar Camp, 20 Jul 1925, *Frost, F.H. 92* (JEPS); Modoc County, 2 mi above Cedarville (Cedar Camp); Warner Mountains, Cedar Camp, 20 Jul 1925, *Frost, F.H. 92* (UC); Nevada County, Truckee, Aug 1912, *Geis, H.D. s.n.* (UC); Nevada County, Truckee, Aug 1912, *Geis, H.D. s.n.* (US); Nevada County, Truckee, Aug 1912, *Geiss, H.D. s.n.* (NY); Modoc County, Modoc Forest (Alturas), 1 Sep 1912, *Goldsmith, B.C. 74* (MEXU); Lassen County, east end of Tule Lake Tule Lake, 1 Aug 1947, *Gran, V. & Schneider, I. 8234* (UC); Modoc County, sw end of Clear Lake Clear Lake, 27 Aug 1947, *Gran, V. & Schneider, I. 8319* (UC); Lassen County, Susanville, in city park, 10 Jul 1973, *Howell, J.T. 49797* (NY); Siskiyou County, e of Mayten (e side of Little Shasta Valley), Little Shasta Valley, 23 Jun 1940, *Jepson, W.L. 19990* (JEPS); Modoc County, 2 mi e of Alturas (Dorris Reservoir); Dorris Reservoir, 10 Jul 1947, *Mason, H.L. & Grant, V. 13341* (UC); Modoc County, Warner Mountains, Cedarville Road, 1 mi e of summit Cedarville Road; Warner Mountains, Cedarville Road, 25 Jun 1926, *Peirson, F.W. 6878* (JEPS); Siskiyou County, North of Mount. Dome, Doris [Dorris], 20 Jul 1956, *Pengelly, R. 796* (SOC); San Bernardino County, NE San Bernadino Co. Clark Mt. Range, at coral near Greens Well, ca. 6.5 miles NNW of Mt Pass, 12 Jun 1974, *Prigge, B.A. 1591* (NY); Modoc County, Corporation Ranch, Likely, Jun 1943, *Robbins, & Raynor s.n.* (WIS); Lassen County, Along Ash Valley road in Ash Valley, 1 Sep 1987, *Schoolcraft, G. 1774* (NY, UC); Kern County, 3 mi sw of the head of the eugene Grade (summit of the Greenhorn Range, along Rattlesnake Road), Greenhorn Range, Rattlesnake Road, 22 Jun 1961, *Smith, C.N. 1049* (JEPS); Los Angeles County, 2 1/2 mi nne of Claremont, 6 Aug 1932, *Wheeler, L.C. 1124* (UC); Siskiyou County, Ager, 13 Jul 1935, *Wheeler, L.C. 3655* (NY); Modoc County, Springs Valley, Pit River at Rattlesnake Butte, Warm, 22 Sep 1935, *Wheeler, L.C. 3995* (MO, NY, US); Modoc County, 3 mi above e base Warner Mountains (on the grade from Lake City-Fort Bidwell Road-to Davis Creek), 5 Sep 1931, *Wolf, C.B. 2399* (UC). **Colorado**: Gunnison County, Southern Gunnison Basin: ca 1.5 air mi due E of Fitzpatrick Reservoir; ca 4.1-4.6 road mi from U.S. Hwy 50; ca 23 air mi ESE of Montrose along County Road 864, 19 Aug 1998, *Arnett, M. 3474* (RM); Gunnison County, Southern Gunnison Basin: County Road 31, ca 1.4 air mi ESE of Big Springs, ca 3 air mi NNE of Ninemile Hill; ca 4 air mi SSE of Blaine Rock, 24 Aug 1999, *Arnett, M. 7495* (RM); Gunnison County, Southern Gunnison Basin: Curecanti National Recreation Area: Colo Hwy 149 ca 0.3 road mi S of intersection with U.S. Hwy 50, ca 8 air mi WSW of Gunnison, 24 Aug 1999, *Arnett, M. 7544* (RM); Fort Collins, 10 Jun 1896, *Baker, C.F. s.n.* (NY); La Plata County, Durango, 20 Jul 1898, *Baker, C.F. et al. 622* (BM, E, F, GH, K, MO, NY, US, W); Rio Blanco County, White River, Agency [White River Agency, now the site of Meeker], Sep 1874, *Barber, E.A. s.n.* (US); Hinsdale County, Gravelly dam of one of the fish reservoirs in Fish Creek, at some distance from the pond itself, a mile or so west of Cebolla Creek, 9 Aug 1962, *Barrell, J. & Spongberg, S.A. 561 -62* (US); Baca County, Vilas, Aug 1905, *Belknap, F. 2* (F); From the neighborhood of Pike's Peak. Colorado Springs, 10 Jul 1895, *Bessey, E.A. s.n.* (NY); South Park, 1871, *Brandegee, T.S. 298* (NY); Gunnison County, Almont, 1960, *Chaney, C.D. s.n.* (WIS); Washington County, Akron Experiment Station, 2 Jul 1935, *Christ, J.H. 692* (ID); Fremont County, East-central Colorado: Mosquito Range to Plains: Arkansas River Drainage: Twin Mountain off U.S. Hwy 50, ca 5.6 air mi NW of Canon City; ca 2 road mi from turnoff to Royal Gorge, 16 Jun 1995, *Chumley, T.W. 853* (RM); Park County, East-central Colorado: Mosquito Range to Plains: South Park: off County Road 15 or Elkhorn Road, ca 1.8 rd mi S of intersection with County Road 687, ca 5 air mi SE of Como, 13 Jul 1996, *Chumley, T.W. 4492* (RM); Colorado Springs, 19 Sep 1895, *Clarke, S.L. s.n.* (NY); Colorado Springs, 20 Sep 1895, *Clarke, S.L. 191* (NY); Jefferson County, Mountain View, 7 Aug 1901, *Clements, F.E. & Clements, E.S. 148* (E, GH, MO, NY, US); Larimer County, Fort Collins, 28 Jul 1961, *Comte, F. 4551* (MO); Colorado Territory, Buffalo Peaks, 23 Jul 1873, *Coulter, J.M. s.n.* (NY); Fort Collins, 22 Jun 1893, *Cowen, J.H. s.n.* (NY); Larimer County, Fort Collins, 22 Jun 1893, *Cowen, J.H. 371* (US); Fort Collins, Jun 1982, *Crandall, C.S. 167* (GH, WIS); Larimer County, Ft. Collins, Jun 1892, *Crandall, C.S. 678* (RM); Gunnison County, 2 mi N of Almont; Gunnison National Forest; Hwy 135 right of way, 1 Aug 1960, *Crosswhite, F.S. 1245* (WIS); Estes Park, 14 Sep 1919, *Degener, O. 1203* (NY); Denver County, along the Platte River near Denver, 13 Jul 1887, *Demetrio, C.H. s.n.* (F); Lake County, Twin Lakes, 22 Aug 1874, *Derry, C.W. s.n.* (F); Boulder County, St. Vrain Creek, 9 Jun 1906, *Dodds, G.S. 1857* (RM); Chayenner Canyon,

21 Aug 1915, *Drushel, J.A. s.n.* (MO); Denver County, North Denver, along the streets, 3 Sep 1910, *Eastwood, A. s.n.* (K); Las Animas County, Wet Mountains, Wet Mountain Valley, Sangre de Cristo Range, and Vicinity: Exit 23 on I-25, 23 Jul 1998, *Elliot, B. 4027* (RM); Costilla County, Wet Mountains, Wet Mountain Valley, Sangre de Cristo Range, and Vicinity: Sangre de Cristo Range: at Mountain Home Reservoir, ca 3 mi SE of Ft. Garland, 10 Jul 1999, *Elliot, B. & Mark, E. 8807 a* (RM); Costilla County, Wet Mountains, Wet Mountain Valley, Sangre de Cristo Range, and Vicinity: San Luis Valley: Forbes Trinchera Ranch: ca 2 mi E of Fort Garland on U.S. Hwy 160, 31 Jul 1999, *Elliot, B. & Schneider, P. 10194* (RM); Montezuma County, near Mancos, Sep 1939, *Ellis, C.C. 64* (F); Clear Creek County, on Clear Creek near Empire, Aug 1874, *Englemann, G. s.n.* (MO); Rio Grande County, Eastern San Juan Mountains: Rio Grande National Forest: Colo Hwy 149 N, ca 2.5 air mi N of South Fork, 14 Aug 2003, *Flaig, J. 2621* (RM); Archuleta County, Seven miles above Arboles, Archuleta Co. Colorado, 5 Jul 1960, *Flowers, S. 146* (UT); El Paso County, Manitou, Aug 1881, *Fritchey, J.Q.A. 120* (MO); Jefferson County, "Golden City", 25 Jul 1876, *Greene, E.L. 322* (GH); Chyenne Canyon, Jul 1890, *Gurney, J. s.n.* (MO); Pueblo County, Colorado City, 1862, *Hall, E. & Harbour, J.P. 667* (F); Gunnison County, Dry Gulch, Gunnison County, Colorado, 28 Jun 1961, *Hall, H.H. 21* (UT); Gunnison County, Lake Fork, Gunnison Co. Colorado, 16 Jul 1961, *Hall, H.H. 564* (UT); Huerfano County, Wet Mountains, Wet Mountain Valley, Sangre de Cristo Range, and Vicinity: Culebra Range: Indian Creek from Indian Creek trailhead, ca 0.8 air mi NW, 24 Jul 1998, *Hartman, R.L. & Fowler, J. 62707* (RM); Las Animas County, Wet Mountains, Wet Mountain Valley, Sangre de Cristo Range, and Vicinity: I-25 frontage road to Chicosa Canyon, SE of Hoehne exchange, 17 Aug 1998, *Hartman, R.L. 63683* (RM); Saguache County, Wet Mountains, Wet Mountain Valley, Sangre de Cristo Range, and Vicinity: Cochetopa Hills: ca 2.5 air mi NNW of Colo Hwy 114 on road to Cabin Draw, 11 Jul 1999, *Hartman, R.L. & Sun, F. 65118* (RM); Fremont County, Wet Mountains, Wet Mountain Valley, Sangre de Cristo Range, and Vicinity: Sangre de Cristo Range: U.S. Hwy 50, 2.5 air mi NW of Howard, 31 Jul 1999, *Hartman, R.L. 65436* (RM); Saguache County, Wet Mountains, Wet Mountain Valley, Sangre de Cristo Range, and Vicinity: Cochetopa Hills: County Road NN56, 5 air mi NW of Villa Grove, 3 Aug 1999, *Hartman, R.L. 65767* (RM); Saguache County, Wet Mountains, Wet Mountain Valley, Sangre de Cristo Range, and Vicinity: San Luis Valley: Rattlesnake Hill, 4 mi E of Saguache, 4 Aug 1999, *Hartman, R.L. 65943* (RM); Saguache County, Wet Mountains, Wet Mountain Valley, Sangre de Cristo Range, and Vicinity: Cochetopa Hills: County Road 46AA, 4 air mi NNW of Saguache near Cottonwood Spring, 17 Aug 1999, *Hartman, R.L. 66069* (RM); Saguache County, Wet Mountains, Wet Mountain Valley, Sangre de Cristo Range, and Vicinity: Cochetopa Hills: Dry Gulch Road, 22 Aug 1999, *Hartman, R.L. 66409* (RM); Fremont County, Wet Mountains, Wet Mountain Valley, Sangre de Cristo Range, and Vicinity: Sangre de Cristo Range: Spring Gulch, 23 Aug 1999, *Hartman, R.L. 66612* (RM); Eagle County, Central Colorado: Castle Peak Area, West Slope Continental Divide: ca 0.7-2 air mi S of State Bridge off Colo Hwy 131, 26 Jun 2000, *Hartman, R.L. 67613* (RM); Conejos County, Eastern San Juan Mountains: 2.5 road mi at Colo Hwy 17, 18 Jun 2003, *Hartman, R.L. & Flaig, J. 77409* (RM); Mesa County, Grand Junction, Aug 1901, *Hedgcock, G.G. s.n.* (MO); Denver County, Cassells, Platte Canon, 1894, *Henry, A. & Henry, M.i.s.s. s.n.* (K); Rio Blanco County, Steep rocky Amelanchier slope, White River Valley, 4 miles NE. Bufford [Buford], 15 Aug 1955, *Hermann, F.J. 12150* (US); Golden City, 8 Aug 1896, *Holm, T. s.n.* (WIS); Arapahoe County, Aurora, E of Chambers Road, 19 Aug 2008, *Holmberg, N.J. & Holmberg, J. 2730* (MO); Pitkin County, Central Colorado: Sawatch Range, West Slope Continental Divide: along Forest Road 103 just E of end of pavement, ca 4 air mi N of Aspen, 4 Aug 2001, *Holt, E.A. 7624* (RM); 1877, *Hooker, J.D. & Gray, A. s.n.* (K); Fremont County, Parkdale, 26 Aug 1898, *Horner, C.N.S. s.n.* (GH); Adams County, Brighton, 30 Sep 1908, *Johnston, E.L. 144 a* (MO); Mesa County, Grand Junction, 21 Jun 1894, *Jones, M.E. 5475* (BM, NY, US); Denver County, Denver, RR yard, 20 Jun 1937, *Knowlton, C.H. s.n.* (GH); U. St, *Kuntze, C.E.O. 3018* (K); Las Animas County, Vermejo Park Ranch: in Rita Canyon 3.2 air mi S of Sarcillo and 14.6 air mi SW of Trinidad, 15 Sep 2008, *Legler, B. 11231* (RM); Colorado Ter, Aug 1873, *Leidy, J. s.n.* (NY); Garden of Gods, 8 Jul 1877, *McCosh, A.J. s.n.* (NY); Black Hawk, Rocky Mts, Aug 1871, *Meehan, T. s.n.* (NY); Park County, Along US route 24, 1 mi W of Lake George, 15 Aug 1991, *Miller, J.S. et al. 6772* (MO); sin. loc, 1876, *Mohr, P.F. 21* (US); San Miguel County, Upper Dolores River Drainage and Adjacent Areas: ca 4 road mi NE of Egnar, County Road K8 and County Road L9, ca 13 air mi N of Dove Creek, 17 Aug 1994, *Moore, L.M. 3996* (RM);

Montezuma County, Indian Camp Road, Archaeological/Housing Subdivision, 2 Aug 1998, *Murray, S. SM-39 -98* (WIS); Weld County, 0.5 mi S of Wyoming border, along dirt road paralleling I-25, 14 Aug 1984, *Neese, E.J. 16050* (NY); Jackson County, North-central Colorado: North Park: along County Road 11, ca 1-1.5 mi S of County Road 28, ca 3-3.5 air mi NW of Buffalo Peak; ca 11.5 air mi WNW of Rand; ca 16-16.5 air mi SSW of Walden, 6 Sep 2000, *Nelson, B.E. 52148* (RM); Routt County, North-central Colorado: Elkhead Mountains: along Wolf Creek and County Road 52, ca 5 air mi SW of Slippery Sides Mountain, ca 6.5 air mi ENE of Hayden, 28 Jun 2001, *Nelson, B.E. 53418* (RM); Routt County, North-central Colorado: Park Range: along County Road 179, ca 7 air mi N of Oak Creek; ca 10.5 air mi SW of Steamboat Springs, 28 Jun 2001, *Nelson, B.E. 53573* (RM); Jackson County, North-central Colorado: North Park: along County Road 6 in the vicinity of Trappers Gulch at the south end of Battle Ridge, ca 11 air mi WNW of Cowdrey; ca 17 air mi NW of Walden, 29 Aug 2001, *Nelson, B.E. 56407* (RM); Conejos County, Eastern San Juan Mountains: in The Canyon and adjacent areas on Forest Road 255 at the forest boundary, ca 12 air mi NW of Capulin; ca 15 air mi SSE of Monte Vista, 15 Jul 2003, *Nelson, B.E. 59279* (RM); Eagle County, Red Cliff, 16 Aug 1906, *Osterhout, G.E. 3360* (GH); Clear Creek County, Clear Creek Valley, 11 Jul 1876, *Patterson, H.N. s.n.* (F); El Paso County, Manitou Springs, 15 Aug 1875, *Patterson, H.N. s.n.* (F); Boulder, 1891, *Penard, E. 373* (NY); El Paso County, Colorado Springs, 31 Jul 1878, *Popenoe, E.A. s.n.* (MO); Teller County, Near Florissant, 1 Aug 1905, *Ramaley, F. 1310* (RM); Colorado Springs, 19 Jul 1872, *Redfield, J.H. 543* (NY); El Paso County, Colorado Springs, 19 Jul 1872, *Redfields, J.H. 5883* (MO); Las Animas County, Region de Mesa de Maya. South slope of Tecolote Mesa near Troy, 1 Jul 1947, *Rogers, C.M. 4462* (US); Buffalo Creek Canon [Canyon?], 14 Sep 1909, *Rusby, H.H. s.n.* (NY); Washington County, Akron Colo, 22 Jun 1909, *Shantz, H.L. 947* (US); Washington County, Akron Colo, 1912, *Shantz, H.L. 1202* (US); Washington County, Akron Colo, 15 Sep 1913, *Shantz, H.L. 1244* (US); Ouray, 24 Jul 1897, *Shear, C.L. 4159* (NY, US); Walsenburg, 11 Jul 1896, *Shear, C.L. 4779* (NY); Denver County, Denver, 1892, *Sones, G.D. s.n.* (ID); Gunnison County, Nine-mile Hill, about 5 miles S of Iola, 27 Jul 1965, *Spongberg, S.A. 65-150* (A); Boulder County, Near Boulder, Jul 1902, *Tweedy, F. 5235* (NY); Along Uncompahgre River, near Ouray, 10 Sep 1901, *Underwood, L.M. 243* (NY); Powell's Colorado Exploring Expedition. Rocky Mountains, Lat. 40-41 degrees, 1868, *Vasey, G. 460* (BRU, NY); Cucharas Valley, near La Veta, 20 Jul 1900, *Vreeland, F.K. 638* (K, NY); Montrose County, Paradox, 25 Jul 1912, *Walker, E.P. 333* (E, GH, MO, US); Costilla County, Collected along US 160, near Forbes Ranch exist, mile marker 268-269, west side of La Veta Pass, 26 Aug 1983, *Walter, D. & Walter, V. 9264* (MO); Saguache County, County Road G. (La Garita Road) at Carnero Creek Crossing, 26 Aug 1986, *Walter, D. et al. 11528* (MO); Jefferson County, Golden, 1 Aug 1881, *Ward, L.F. s.n.* (US); Boulder County, N edge of Boulder E of Lee Hill Canyon, 26 Jun 1967, *Weber, W.A. 13135* (H); Buena Vista, Jul 1886, *Without Collector s.n.* (WIS); Eagle Mountain, Manitou, 20 Jul 1900, *Without Collector s.n.* (WIS); Boulder, 1 Sep 1975, *Without Collector s.n.* (WIS); Boulder, 1 Sep 1975, *Without Collector s.n.* (WIS); Park County, South Park, Jul 1873, *Wolf, J. 88* (US); Lake County, Twin Lakes, Aug 1873, *Wolfe, J. s.n.* (F); Park County, South Park, 1873, *Wolfe, J. 88* (F, NY); Pueblo County, Pueblo, 4 Jul 1882, *Woodward, R.W. s.n.* (GH); Col, 1874, *Woolson, G.C. s.n.* (NY). **District of Columbia**: near blacksmith shop in USDA, *Wheeler, C.F. s.n.* (US). **Idaho**: sin. loc, 1873, *Allen, J.A. s.n.* (NY); Blaine County, NNE of Ant Butte, NE of Carey; CMNM. 7 Jul 2002, *Atwood, N.D. 28806* (CRMO, NY); Owyhee County, Along Sinker Creek, Silver City Range, 27 Jul 1951, *Baker, W.H. 8226* (ID, NY, WTU); Owyhee County, Along Poison Creek, 1 mile northeast of summit, Ruby Range, 6 Aug 1951, *Baker, W.H. 8381* (ID, WTU); Cassia County, About one mile east of Almo, 8 Jul 1952, *Baker, W.H. 9147* (ID, WTU); Blaine County, 3 miles east of Bellevue, 22 Aug 1953, *Baker, W.H. 11121* (ID, WTU); Lemhi County, above Pattee Creek along Warm Springs road, 6 miles NE from Tendoy, on lower west slope of Beaverhead Mts, 27 Jul 1961, *Bennett, H.R. 7749* (F, US, W); Cassia County, Raft River Valley, 1929, *Biggers, D.D. 13348* (NY); Owyhee County, Between new reservoir & Reynolds Cr, 9 Sep 1972, *Bratz, R.D. s.n.* (BBLM); Owyhee County, Between silted in stockpond + gauge #109, 7 Sep 1964, *Bratz, R.D. s.n.* (CIC); Owyhee County, Near canal; roadside at Reynold's Creek, 7 Aug 1964, *Bratz, R.D. s.n.* (CIC); Bannock County, 3 mi N of Pocatello, 16 Sep 1941, *Brenckle, J.F. & Shimmers, L.H. s.n.* (WIS); Lincoln County, Seven miles north of Shoshone, 25 Aug 1950, *Chichester, B. 514* (ID); Bannock County, Indian Rocks State Park, West side, 21 Aug 1996, *Chipman, S.J. 432* (IDS, NY, SRP); Idaho County, Whitebird, Salmon River, 21 Jul 1932, *Christ, J.H. 1741* (ID, NY); Teton County, High

Altitude Substation, Tetonia, 14 Jul 1934, *Christ, J.H. 5456* (NY); Adams County, 1 mile north of Starkey, 24 Aug 1941, *Christ, J.H. 12949* (ID, NY); Twin Falls County, 21 miles west of Rogerson, Idaho; 3 miles west of Cedar Creek Reservoir, House Creek drainage, 4 Jul 1949, *Christ, J.H. & Christ, C.B. 18326* (NY); Owyhee County, Triangle Road about 0.2 mi NE Gilmore Creek crossing, about 1.3 mi SE Toy Pass, 26 Jun 2013, *Corbin, B. & Barnes, K. 1552* (BBLM); Owyhee County, McBride Creek, just north of Hwy 95, 13 Jul 2011, *Corbin, B.L. 1475* (BBLM-OWY); Owyhee County, Triangle Road about 0.2 mi NE of Gilmore Creek crossing, about 1.3 mi SW of Toy Pass, 26 Jun 2013, *Corbin, B.L. 1552* (NY); Madison County, Rexburg, 18 Aug 1930, *Davis, R.J. s.n.* (NY); Elmore County, Pine, 19 Jul 1940, *Davis, R.J. 2829* (NY); Adams County, Starkey, 24 Apr 1940, *Davis, R.J. 2996* (ID); Owyhee County, Along CCC Road approx. 16 km SE of Bruneau, ID, 12 Jul 1993, *DeBolt, A. 1745* (SRP); Owyhee County, Along CCC Rd. 16 km SE of Bruneau, 12 Jul 1993, *DeBolt, A. 1745* (BBLM); Minidoka County, 10 mi. NE of Burley, 7 Jul 1937, *Detling, L.E. 2224* (OSC); Camas County, along St. Rd. 68, 8.4 mi W of Hill City, 13 Aug 1976, *Dziekanowski, C.T. et al. 2554* (MO, NY, WIS); Madison County, Rexburg, 31 Aug 1895, *Elrod, M.J. s.n.* (F); Mal County, Mud Springs in Leslie Gulch, 22 Sep 1974, *Ertter, B.J. 163 /4* (CIC); Owyhee County, Bruneau Plateau S of Winter Camp Butte, 19 Jul 1978, *Ertter, B.J. 2373* (CIC, NY); Ada County, lower Hulls Gulch in northeast foothills of Boise, north end of The Grove trailhead parking area, 25 Sep 2010, *Ertter, B.J. 20649* (SRP); Owyhee County, just W of Bruneau Dunes State Park; Approximately 100 km SE of Boise, Idaho, 23 Jun 1992, *Frogley, W. 1663* (BBLM, SRP); Ada County, [No locality given on label.], 19 Jul 1974, *Frohlich, D. DF-4 -P* (CIC); Power County, Fort Hall Indian Reservation, Michaud Creek and Taghee Canal, 28 Jul 1987, *Glennon, J.M. 1500* (NY, SRP); Owyhee County, At Bruneau Sand Dunes State Park, 21 Oct 1978, *Grimes, J. et al. 1304* (CIC); Elmore County, Hammett, 2 Jun 1920, *Haegele, R.W. s.n.* (ID); Twin Falls County, Idaho, 19 Jul 1926, *Haegele, R.W. s.n.* (BOIS); Twin Falls County, Twin Falls, 19 Jul 1926, *Haegele, R.W. A-11* (ID); Twin Falls County, Twin Falls, Jul 1929, *Hills, O.A. 5* (OSC); Caribou County, Portneuf Valley, along the Portneuf River Road; 24 km (15 mi) air distance north-northwest of Bancroft, 3 Sep 2012, *Holmgren, N.H. 16430* (NY); Caribou County, 5 miles N. of Soda Springs, 11 Oct 1971, *Holte, K.E. 33* (WS); Lemhi County, Lemhi Valley: Lemhi Hole recreation site, 0.9 air mi N of Salmon, 20 Jul 2012, *Irwin, J.J. 8764* (RM); Cassia County, The Albion Mountains. Disturbed area along roadside south of junction between Sisters and Emery Canyon Roads, 2 Aug 1992, *John, T. 945* (IDS); Pocatees. [interpreted], 23 Aug 1905, *Johnson, F.W. s.n.* (US); Cassia County, 4 miles south of Burley, 18 Sep 1937, *Maguire, B. s.n.* (NY); Owyhee County, Along Piute Creek, north of road crossing in northern part of Piute Basin and south of E. Fork Owyhee River, in northern YP Desert, 12 Aug 2015, *Mansfield, D. & Stevens, M. 15-297* (CIC); Valley County, Frank Church-River of No Return Wilderness, Payette National Forest. Big Creek trail from Taylor Wilderness Research Station to Soldier Bar, just past Goat Creek, 20 Jun 2011, *Martling, S. et al. 76* (ID); Caribou Forest. Roadside 1 mi. above Hall's ranch-Graw Creek, 20 Jul 1927, *McDonald, C.H. & Bruce, J. 640* (IDS); Cassia County, 0.5 miles W of Declo, 21 Jul 1951, *Morton, H. & Zappettini, G. s.n.* (ID); Valley County, ca.1 1/2 miles downstream of Taylor Wilderness Research Station; along Big Creek trail, just past hidden pictographs, 7 Jun 2011, *O'Conner, C. & Martling, S. 29* (ID); Canyon County, Dautrich Memorial Preserve on the north side of the dust dune, Oct 1973, *Packard, P.L. 73-287* (CIC); Snake Plains; Blackfoot, 9 Aug 1893, *Palmer, E. 466* (US, WIS); Twin Falls County, 6 mi. SW of Buhl, Idaho; Twin Falls Co, 25 Aug 1966, *Peterson, E. s.n.* (CIC); Twin Falls County, Twin Falls, Idaho, 4 Sep 1930, *Piemeisel, R.L. 30-543* (BOIS); Owyhee County, Deep Creek rd, N fork of Owyhee crossing, 7 Jul 1977, *Richards, S.J. 77-483* (BBLM); Owyhee County, [No locality given on label.], Sep 1981, *Rosentreter, R. 2459* (CIC); Fremont County, Spencer, 9 Jul 1916, *Rust, H.J. 426* (ID); Teton County, [no locality given on label], 14 Jul 1934, *Rust, H.J. 5455* (ID); Lincoln County, Shoshone, 5 Aug 1893, *Rutter, C. s.n.* (F); Owyhee County, Mud Flat road, Poison Creek Recreation site, 9 Sep 2012, *Smith, J.F. 10716* (CIC, NY, SRP); Bannock County, Garden Creek, 18 Sep 1941, *Taylor, H. s.n.* (IDS); Canyon County, Caldwell, 15 Jul 1932, *Tucker, H.M. s.n.* (CIC, NY); Butte County, Near group campsite rd. intersection, n. toe of Sunset Crater, 22 Aug 2003, *Wolken, P. 1156* (CRMO); Blaine County, Common in empty lots at Hailey, 15 Aug 1909, *Woods, C.N. & Tidestrom, I.F. 2765* (US, WIS). **Illinois**: Athens From [illegible: E?ds], 1863, *Hall, E. s.n.* (NY); Will County, Joliet, 6 Sep 1904, *Skeels, H.C. 520* (F); Cook County, Chicago, E side of Calumet Lake, between Torrence Avenue at about 11900 South and Nickel Plate railroad, between Ford plant and Solway

Coke plant, 4 Aug 1949, *Steyermark, J.A. 68846* (F). **Iowa**: Fayette County, Fayette, along the Volga River, 6 Aug 1894, *Fink, B. s.n.* (GH). **Kansas**: Rooks County, Rockport, 10 Aug 1889, *Bartholomew, E. s.n.* (MO); Gove County, Plains, 24 Jul 1895, *Hitchcock, A.S. 361* (GH, MO, NY, US); Hodgeman County, 10 Mi. W. 1Â½ Mi. S. Jetmore, 28 Aug 1951, *McGregor, R.L. 5176* (NY); Greely County, Trebune [Tribune], 5 Aug 1892, *Reed, M. s.n.* (US); Turkey Creek, east of McCallaster, 18 Jul 1929, *Rydberg, P.A. 1147* (NY); sin. loc, *Seaman, W.H. s.n.* (US); Osborne County, Collected within a radius of five miles of Osborne City, 24 Jun 1894, *Shear, C.L. 134* (NY); Osborne County, within 5 miles radius of Osborne City, 23 Jul 1894, *Shear, C.L. 197* (GH). **Massachusetts**: Gosnold, 11 Aug 1926, *Fogg, J.M. 1806* (NEBC). **Michigan**: Washtenaw County, Ann Arbor, 890 Wickfield Court, 13 Sep 1984, *Reznicek, A.A. 7454* (MICH). **Minnesota**: Clay County, Morehead, [Moorhead], 4 Jun 1901, *Ballard, C. 2828* (MIN); Clay County, Muskoda, [historic RR station near Hawley], 14 Aug 1901, *Ballard, C. 3160* (MIN); Hennepin County, Near Minneapolis;;N;;, Jul 1892, *Burglehaus, F. 3212* (MIN); Hubbard, Straight River, SW of Park Rapids, 4 Aug 1950, *Gale, M. 6* (WIS); Clay County, Sabin;;N;;, Jun 1891, *Piper, I. s.n.* (MIN); Buffalo River State Park, 23 Jul 1964, *Stevens, O.A. 2731* (BRU, MIN, US). **Missouri**: Jackson County, Sheffield, 13 Jun 1906, *Bush, B.F. 3007* (GH, MO, NY); Jackson County, Sheffield, 23 May 1906, *Bush, B.F. 3882* (GH, MO, NY); Jackson County, Sheffield, 13 Jun 1906, *Bush, B.F. 3962* (GH, MO, US); Jackson County, Courtney, 21 Jul 1910, *Bush, B.F. 6007* (MO); Jackson County, Sheffield, 20 Aug 1913, *Bush, B.F. 7096 A* (GH, MO, NY, US); Jackson County, Sheffield [The name given in this record as the county was not specified on the label but was inferred from the label's locality data.], 20 Aug 1913, *Bush, B.F. 7096 B* (NY); Jackson County, Sheffield, 22 Jun 1914, *Bush, B.F. 7142* (GH, NY); Jackson County, Sheffield, 17 Jun 1915, *Bush, B.F. 7662* (GH, NY, US); Courtney, 10 Jul 1930, *Bush, B.F. 11818* (NY); Jackson County, Courtney waste ground, 26 Jun 1931, *Bush, B.F. 12167* (MO); Jackson County, Sheffield, 20 Aug 1913, *Bush, B.F. 70968* * (US); 'Coteau de Missouri Dawson 6/77', Jun 1877, *Dawson, G.M. s.n.* (E); sin. loc, *Geyer, C.A. s.n.* (W); Jackson County, Courtney. Along RR, 18 Jul 1932, *Kellogg, J.H. s.n.* (MO); Laclede County, Hwy 66, 5 mi E, 1 Jul 1937, *Moore, G. s.n.* (F). **Montana**: Madison County, Between Madison and Jefferson Rivers, 7 Sep 1871, *Adams, R. s.n.* (US); Missoula County, Missoula, along proposed Southwest Higgins Avenue, 23 Jun 1956, *Addor, E.E. 172* (MEXU, MONTU); Cascade County, Great Falls, 15 Oct 1886, *Anderson, F.W. s.n.* (F); Sand Coulee, 10 Jul 1887, *Anderson, F.W. 5981* (NY); Ex territorio Montana juxta, Aug 1884, *Ball, J. s.n.* (K); Gallatin County, Bozeman, 13 Jul 1898, *Blankinship, J.W. s. n.* (MONTU, RM, WTU); Gallatin County, Bozeman, 16 Aug 1905, *Blankinship, J.W. s.n.* (SRP); Gallatin County, Bozeman, 21 Aug 1898, *Blankinship, J.W. s.n.* (MONT); Gallatin County, Bozeman, 13 Jul 1898, *Blankinship, J.W. s. n.* (RM); Gallatin County, Bozeman, 16 Aug 1905, *Blankinship, J.W. 408* (F, MO, MONTU, US); Wibaux County, Yates, 4 Sep 1937, *Bochmeier, J. 452* (MONTU); Wibaux County, Yates, 12 Jul 1937, *Bochmeier, J. 978* (MONT, MONTU); Carter County, Alzada, 1/2 mile south, 19 Jun 1948, *Booth, W.E. s.n.* (WTU); Big Horn County, Wyola. 1 mile North, 22 Aug 1954, *Booth, W.E. s.n.* (MONT); Carbon County, Fromberg, 3 miles north, 2 Aug 1941, *Booth, W.E. s.n.* (MONT); Carter County, Alzada, 0.5 mile south, 19 Jun 1948, *Booth, W.E. s.n.* (MONT); Carbon County, From about 1 to 15 miles north of the Wyoming line north of Lovell, Wyoming, 18 Jul 1954, *Booth, W.E. 54547* (ID, MONT, WTU); Big Horn County, Southwestern Bighorn County. East of Bighorn River about 2 miles above the Wyoming border, 18 Jul 1954, *Booth, W.E. 54602* (MONT); Sheridan County, Raymond. Just south of town, 17 Jul 1957, *Booth, W.E. 57445* (MONT); Powder River County, Broadus, 4 Aug 1961, *Booth, W.E. 61226* (MONT); Petroleum County, Alberion, about 3 miles west, 1 Aug 1957, *Booth, W.E. 571041* (MONT); Big Horn County, CX Coal Site; Decker Quadrangle; S. of the Decker Post Office, 20 Jul 1977, *Brink, D.E. et al. 1423* (MONTU, RM); Whitehall, 15 Jul 1909, *Butler, B.T. 4145* (NY); McCone County, Circle, 1 Oct 1951, *Cade, L.P. s.n.* (MONT); Phillips County, On Border Road, in the vicinity of a natural gas pipeline compression station, ca 1/4 air mi W of Whitewater Reservoir, ca 6 air mi NNW of Whitewater; ca 28 air mi NNE of Malta, 30 Jun 2010, *Charboneau, J.L.M. 782* (RM); Phillips County, N of Sunnyslope Road, ca 7 air mi WSW of the Morgan Port of Entry, ca 12 air mi NNW of Loring; ca 42 air mi NNW of Malta, 22 Jul 2010, *Charboneau, J.L.M. 2048* (RM); Valley County, E of Glentana South Road, ca 8.5 air mi NW of Johnnys Hill, ca 3.5 air mi S of Glentana; ca 9 air mi ESE of Opheim, 1 Aug 2010, *Charboneau, J.L.M. 2698* (RM); Blaine County, Black Coulee National Wildlife Refuge: Black Coulee, ca 10.5 air mi S of Turner, ca 21 air mi NE of Harlem; ca 34.5 air mi NW of Malta, 30 Jul

2011, *Charboneau, J.L.M. 8339* (RM); Phillips County, Spring Coulee, S of Nylander Road, ca 6.5 air mi NNE of Dodson, ca 8 air mi N of the Milk River, ca 17 air mi WNW of Malta, 9 Aug 2011, *Charboneau, J.L.M. 9265* (RM); Phillips County, PR-22 Reservoir, N of Whitewater Creek, ca 4.5 air mi SW of 3 Way No. 2 Reservoir, ca 10 air mi NW of Whitewater; ca 37.5 air mi NNE of Malta, 11 Aug 2011, *Charboneau, J.L.M. 9450* (RM); Hill County, Havre, 19 Aug 1963, *Connor, R.J. s.n.* (MONT); Gallatin County, Bozeman, 23 Jul 1904, *Cooke, A.M. s.n.* (MONT); Park County, Gallatin Range: Gallatin National Forest: Yankee Jim Canyon, on west side of Yellowstone River, ca 12 mi NW of Gardiner, 8 Jul 1991, *Evert, E.F. 21480* (RM); Yellowstone County, Billings, *Hawkins, P.H. s.n.* (MONT); Cascade County, 15 miles south of Great Falls, 28 Aug 1957, *Heikes, E. s.n.* (MONT); Gallatin County, Bozeman, 1897, *Henshall, J.A. 197* (F); Wheatland County, Harlowton; ca. 12 mi SE, schoolhouse, District 35, Aug 1934, *Hitchcock, C.L. 2449* (MONTU); Missoula County, Missoula, Jul 1916, *Hughes, J.A. s.n.* (MONTU); Silver Bow County, Butte vicinity (W. Granite), 24 Sep 1998, *Johns, H. s.n.* (MONT); Helena, 1890, *Kelsey, F.D. s.n.* (SRP); Cascade County, Blackfoot Valley; Northern Rocky Mountains, 11 Aug 1917, *Kirkwood, J.E. s.n.* (MONTU); sin. loc, 1888, *Knowlton, F.H. s.n.* (US); Flathead County, Hog Heaven Mining District, 21 Jul 1977, *Lackschewitz, K.H. 7665* (MONTU); Phillips County, Slippery Anne Ranger Station; East of R.S, 13 Jun 1978, *Lackschewitz, K.H. 8150* (MONT, MONTU); McCone County, S. Fork Rock Creek Road, 29 Jun 1978, *Lackschewitz, K.H. 8264* (MONT, MONTU); Beaverhead County, Medicine Lodge Creek; Tendoy Mtns, 27 Jul 1993, *Lesica, P.C. 6126* (MONTU, NY); Yellowstone County, Sevenmile Flat; on the north side of the Yellowstone River, 9 Sep 1998, *Lesica, P.C. & Miles, S. 7803* (MONTU); Cascade County, Cascade; along the roadside of hwy 91 ca 9 mi NE of town, 16 Jun 1936, *Marsh, V.L. 125* (MONTU); Park County, Yellowstone National Park. Gardiner River, 27 Jul 1902, *Mearns, E.A. 2341* (US); Gallatin County, Campus. Bozeman, 29 Jul 1917, *Morris, E.R. & Streets, R.B. 581* (MONT); Valley County, Hinsdale Wildlife Management Area: along the Milk River above and below the Bear Creek confluence, ca 1.5-2 air mi N of Hinsdale, 14 Sep 2010, *Nelson, B.E. 81168* (RM); Phillips County, At the corner of Grabofsky and North Sunnyslope roads, 1 mi S of Saskatchewan, ca 45 air mi NNW of Malta, 28 Jun 2011, *Nelson, B.E. 82530* (RM); Rosebud County, 1 km southeast of Colstrip, on native range adjacent to MSU-ERDA Plant Succession Study Site # 1924-3, 21 Jul 1976, *Plantenberg, P.L. 4* (MONT); Toole County, Toole County, 21 Jul 1936, *Rishoff, M. 437* (MONTU); Custer County, Miles City, 15 May 1936, *Roberts, E.L. 519* (MONT, MONTU); Fergus County, Lewistown, 23 Jul 1960, *Ross, J.K. s.n.* (MONT); Park County, Yellowstone River, near Fridley, 22 Aug 1897, *Rydberg, P.A. & Bessey, E.A. 4905* (K, NY, US); Missoula County, Near Bonner, 30 Aug 1892, *Sandberg, J.H. et al. 985* (BM, GH, K, NY, US); Custer County, Miles City, 3 Sep 1884, *Seymour, A.B. s.n.* (GH, MO); Gallatin County, Cottonwood Creek. Bozeman, 31 Aug 1892, *Shaw, W.J. s.n.* (MONT); Garrison, 10 Jul 1895, *Shear, C.L. 5249* (NY, US); Gallatin County, Bozeman, Campus, 29 Jul 1917, *Streets, R.B. & Morris, E.R. 581* (MONT); Park County, Suksdorf's Gulch, 9 miles northwest of Wilsall, 30 Aug 1921, *Suksdorf, W.N. 902* (GH, ID, NY, OSC, RM, UBC, US, WTU); Carter County, Powderville Road badlands; ca. 17 air miles WSW of Ekalaka, 15 Jul 1997, *Vanderhorst, J. 5741* (MONT); Madison County, Gallatin National Forest: Jourdain Creek, 25 Sep 1932, *Whitham, J.C. 1328* (USFS); Gallatin County, Bozeman, 15 Aug 1898, *Wilcox, E.V. s.n.* (US); Dawson County, Glendive. Belle Prairie Route, 5 Jul 1956, *Williams, O. s.n.* (MONT); Great Falls, 11 Sep 1891, *Williams, R.S. 406* (MONT, NY, US); Roosevelt County, Bracton, Jul 1924, *Wisner*, *s.n.* (MONT); Beaverhead County, Red Rock Pass; SE Beaverhead County, 23 Aug 1952, *Without Collector s.n.* (MONT). **Nebraska**: Ord. [unsure placement], 16 Jun 1899, *Armstrong, A. s.n.* (US); Alliance, 9 Feb 1911, *Churchill, C.H. s.n.* (NY); Brown County, Northeastern Nebraska. Plum Creek, Brown Co, 26 Aug 1893, *Clements, F. 2915* (US); Brown County, Collected in a prairie dog town in Salzman Pasture, ca. 12 mi. N of Johnstown, 16 Jul 1982, *Freeman, C.C. 1510* (NY); Kearney County, Minden, 3 Jun 1940, *Hapeman, H. s.n.* (ID); Bon Homme County, Running Water, 18 Aug 1854, *Hayden Expedition*, *s.n.* (MO); Fort Union, 1853, *Hayden, F.V. s.n.* (NY); [From protologue: "Near White River, Nebraska, Ter.; Dr. F. V. Hayden." White River is in NW corner of state], 1858, *Hayden, F.V. s.n.* (NY); Buffalo County, Kearney, Aug 1889, *Holms, J.H. s.n.* (US); Franklin County, Republican Valley, Franklin, 17 Jul 1893, *Leybourn, W.A. 75* (MO); Prairie Dog Town, clay hills, 20 Jul 1933, *Palmatier, E.A. 378* (KIRI); Banner County, Prairie-dog Town, Lawrence Fork, 8 Jul 1891, *Rydberg, P.A. 265* (NY); Thomas County, On Middle Loup River, near Thedford, 2 Jun 1893, *Rydberg, P.A. 1393* (NY, US);

Thomas County, on Middle Loup River, near Thedford, 2 Jun 1893, *Rydberg, P.A. 1395* (GH); Dodge County, Jamestown, 1 Aug 1889, *Sheldon, C.S. s.n.* (MO); Hooker County, 2 miles W of Mullen, 12 Jul 1970, *Stephens, S. & Brooks, R. 41414* (GH); Arthur County, Arapaho Prairie, 24 Jun 1983, *Sutherland, D.M. 5659* (NY); Knox County, Niobrara Game Reserve, 10 Aug 1936, *Tolstead, W.L. 699* (GH); Arthur County, Arapaho Prairie, 14 Jul 1977, *Vescio, L.S. 120* (NY); Culbertson, 11 Aug 1911, *Wagner, G.F. s.n.* (WIS); Sioux County, Agate Fossil Beds National Monument, 22 Aug 1983, *Weedon, R.R. & Phillips Hardy, J. 8214* (CIC); Exeter, 15 Sep 1879, *Wibbe, J.H. s.n.* (WIS); Lancaster County, Lincoln, May 1887, *Williams, T.A. s.n.* (US). **Nevada**: Churchill County, Fallon, 6 Jul 1937, *Allen, R.A. 285* (F); Mineral County, Mouth of Cory Creek, Wassuk Range Mineral Co, 29 Aug 1938, *Archer, W.A. 6836* (GH, UT); Lander County, Ten miles south of Brown's Station on road to Maestretti Ranch, 21 Jul 1940, *Beach, K.H. 969* (CORD); Nye County, Drainage Basin: Stone Cabin. Stream below Longstreet Ranch, nw. Kawich Range, 12 Sep 1970, *Beatley, J.C. 11729* (NY); Nye County, Drainage Basin: NW Reveille. Reveille Valley Rd 1-2 mi so. of Reveille Mill, below e. slope of no. Kawich Range, 4 Sep 1971, *Beatley, J.C. 13209* (NY); Clark County, Wilson's ranch. Larrea belt, 1 Aug 1939, *Clokey, I.W. 8485* (BM, CR, F, GH, H, K, MEXU, MO, NY, US, UT, W, WIS); 35 mi NW of Pioche, 19 Jan 1930, *Davis, E.W. s.n.* (WIS); Lander County, vicnity of Big Creek Canyon, 16 miles S of Austin, 28 Aug 1937, *Goodner, F.S. & Henning, W.H. 1273* (GH); Churchill County, Fallon. U.S. Expt. Farm, *Harter, L.L. 1* (US); Elko County, East Humboldt or Ruby Mountains, Tony's Ranch, west side of Harrison Pass (5700 feet), 17 Aug 1908, *Heller, A.A. 9458* (E, MO, NY, US); Ely, 13 Aug 1913, *Hitchcock, A.E. 1343* (NY); Elko, 2 Aug 1913, *Hitchcock, A.S. 958* (GH); Eureka County, Grass Valley, Nevada Route 21, 10 km (6.2 mi) north of Lander Co. line, 26 Jun 1991, *Holmgren, N.H. 11570* (NY); Churchill County, Carson Sink Region, Oct 1907, *Kennedy, P.B. 1720* (E, F, GH, K, MO, NY, US); Lincoln County, 12 mi. E of Caliente, 6 Aug 1937, *La Rivers, I. 767* (F); Lincoln County, 12 mi E of Caliente on Divide of Highland Range, 6 Aug 1937, *LaRivers, I. & Hancock, N.F. 767* (MO); Till County, near Idavada, 23 Jul 1930, *Palmer, E.J. 38000* (GH, MO); White Pine County, Lake Valley, US 93, about 1.1 road miles north of Lincoln County line, by waterway emptying into Travis Reservoir, 9 Sep 1986, *Pinzl, A.P. 7748* (NY); Elko County, Adobe Range, about 1 road mile west of Rte 225 on road about 1.2 road miles north of Adobe Ranch, 5 Sep 1990, *Pinzl, A.P. 9245* (NY); Lincoln County, East of Panaca, north of Dow Mtn. Rte 319 at Crestline turnoff, 23 Sep 1991, *Pinzl, A.P. 9766* (NY); Washoe County, Spanish Springs Valley, east side of the Valley, north of Sparks, T20N, R20E, sec. l, 16 Aug 1980, *Tiehm, A. 6218* (MO, NY); White Pine County, Steptoe Valley, 1.5 road miles east of Cave Valley road on road to Ward Charcoal Oven, T14N, R64E, sec. 20, 27 Aug 1980, *Tiehm, A. 6255* (MO, NY); Lyon County, Pine Nut Mts, 3 air miles SE of Como, near a reservoir, T15N, R23E, sec. 20, 24 Jul 1982, *Tiehm, A. & Williams, M. 7412* (MO, NY); Lincoln, Clover Mountains, Lincoln Co. Clover Mts. Pine Canyon Reservoir, 1.5 road miles SE of am, E of the reservoir flats, T5S R69E, S32, 5 Sep 2005, *Tiehm, A. & Nachlinger, J. 15075* (UT); Ormsby County, Fuji Park, Clear Creek, 0.4 road miles west of highway 395 on Clear Creek Road, 22 Aug 2007, *Tiehm, A. 15484* (NY); Eureka County, Diamond Mountains, 2.4 road miles west of Newark Summit on the road to Eureka, 31 Aug 2009, *Tiehm, A. 15960* (NY); Washoe County, Mosquito Mountains, near end of the pipline, west of the power line on the south side of 12 Mile Creek, 11 Aug 2016, *Tiehm, A. 17643* (NY); Clark County, Apex, station on U.P.Railroad, along hgwy. 91 from Las Vegas to Moapa, 25 May 1938, *Train, P. 1838* (F, MO, NY); Lincoln County, Panaca, 10 Sep 1938, *Train, P. 2501* (NY); Nye County, Peavine Creek, Smokey Valley Dam, crossing on Peavine road, 19 Sep 1939, *Train, P. 3470* (NY); Churchill County, W of Eastgate, near Hwy 50, 7 Feb 1975, *Williams, M.J. 75-66 -7* (NY); Storey County, Upper Long Valley, 23 Aug 1977, *Williams, M.J. 77-84 -10* (NY). **New Mexico**: San Miguel County, vicinity of Las Vegas, 8 m SE, 2 Sep 1926, *Arsène, G. 17348* (MO); Rio Arriba County, Chama, 10 Sep 1899, *Baker, C.F. 577* (BM, E, F, GH, K, MO, NY, RM, US); Santa Fe County, Santa Fe, 28 Jul 1916, *Bertaud Brother*, *68* (NY); Quay County, "San Jon site", 26 Aug 1942, *Bryan, K. s.n.* (GH); San Miguel County, Gallinas Canyon, Trout Spring, *Cockerell, T.D.A. & Cockerell, W.P. s.n.* (RM); Rio Arriba County, Carson Nat. Forest, Hwy. 17, 30 miles west of Dulce; Gobernador River, 25 Aug 1970, *Correll, D.S. 39597* (NY); Lincoln County, Gray, [=Capitan], Jul 1900, *Earle, F.S. & Earle, E.S. 152* (BM, E, K, NY, W); Lincoln County, Gray [= Capitan], Jul 1900, *Earle, F.S. & Earle, E.S. 152* (US); Lincoln County, Gray [=Capitan], Jul 1900, *Earle, F.S. & Earle, E.S. 192* (MO); Socorro County, Datil, 29 Sep 1919, *Eggleston, W.W. 16189* (F); Lincoln County, Ruidoso, Lincoln Forest, 1 Aug 1923, *Eggleston, W.W.*

18924 (US); Catron County, Near Luna, Datil Forest, 17 Jul 1924, *Eggleston, W.W. 20256* (US); Bernalillo County, Sandia Mountains, Balsam Park, Jul 1914, *Ellis, C.C. 251* (MO, NY); sin. loc, 1847, *Fendler, A. 671* (BM, GH, K, LE, MO); San Antonio, Apache Refuge, 5 Jul 1950, *Fleetwood, R.J. s.n.* (NY); Socorro County, San Antonio, Bosque del Apache National Wildlife Refuge, *Fleetwood, R.J. 759* (US); Colfax County, Philmont Scout Ranch, near Cimarron: Rimrock Trail, 2 mi S of Old Abreu Lodge, 17 Jul 1968, *Hartman, R.L. 2409* (RM); Sandoval County, Valles Caldera National Preserve: Jemez Mountains: ca 1 air mi SW of La Jara Creek, 21 Jul 2001, *Hartman, R.L. 72358* (RM); Sandoval County, Valles Caldera National Preserve: Jemez Mountains: Redondo Creek, 2.2-1.1 air mi SE of west entrance, 21 Jul 2001, *Hartman, R.L. 72430* (RM); Sandoval County, Valles Caldera National Preserve: Jemez Mountains: El Cajate Canyon from NM Hwy 4 at East Fork Jemez River E ca 1.5 trail mi, 23 Jul 2001, *Hartman, R.L. 72684* (RM); Sandoval County, Valles Caldera National Preserve: Jemez Mountains: Banco Bonito, 1.5 air mi NW of Jemez Falls Campground entrance, 30 Jul 2001, *Hartman, R.L. 73292* (RM); Sandoval County, Santa Fe National Forest and Vicinity: Jemez Mountains: NM Hwy 126, 1.2 road mi from Forest Road 20, Telephone Canyon, 21 Jul 2002, *Hartman, R.L. 76208* (RM); Taos County, Carson National Forest and Vicinity: Arroyo Aquaje de la Petaca, 15 Aug 2006, *Hartman, R.L. 83392* (RM); Taos County, Carson National Forest and Vicinity: Cerrito Dormilon, 18 Aug 2006, *Hartman, R.L. 83604* (RM); Colfax County, 4 miles west of Ute Park along hwy 64 along Cimmaron Creek, 21 Jul 1972, *Higgins, L.C. 5798* (NY); Mora County, 10 miles south of Black Lake near Guadalupita on hwy 38, 21 Aug 1974, *Higgins, L.C. 9116* (NY); San Miguel County, M.E. O'Connor Trust Ranch, Cuevas Canyon, roadcut below Cuevas Trap, 5.5 mi S of Rte 104; 10 mi WSW of Trujillo, 8 Jun 1982, *Hill, S.R. & Cress, A.D. 11711* (GH, MO, NY); San Miguel County, M. E. O'Connor Trust Ranch. Crystal Pasture, N fenceline; at head of Hermosa Canyon; 2.8 mi. S of Rte. 104, 18 mi. E of Las Vegas, 21 Aug 1982, *Hill, S.R. 12131* (NY); San Miguel County, T. M. O'Connor Ranch. Spring Pasture, E of foreman's house near NE corner of East Trap; 5 mi S of Rte. 104, 15 mi E of Las Vegas, 20 Jul 1984, *Hill, S.R. 14561* (GH, NY); Taos County, Between Amalia and Ute Springs, 5.5 airline miles east of Costilla, 14 Aug 1973, *Holmgren, N.H. 7163* (NY); Taos County, Cerros de Taos, 0.2 miles south of U.S. Highway 64, about 18 (16.5 airline) miles northwest of Taos, 20 Aug 1973, *Holmgren, N.H. 7304* (NY); Grant County, S.W. New Mexico. In Grant County in the vicinity of Silver City, Fort Bayard, Santa Rita, Fierro, the Mimbres Valley and East Canyon tributary to it, and on the G.O.S. Ranch, in canyons within 10 miles of the ranch house. Santa Rita [georef to Santa Rita], 1 Aug 1911, *Holzinger, J.M. s.n.* (US); Colfax County, Eagle Nest Lake, 5 mi S of Eagle Nest on US 64 (ca. 16 mi ENE of Taos), 9 Jul 1953, *Iltis, H.H. 4313* (WIS); Santa Fe County, 16 Bishop's Trail, subdivision across hwy from entrance to Bishop's Lodge, 31 Jul 2015, *Knapp, S. IM-10782* (image only); Colfax County, Vermejo Park Ranch: 0.4 air mi SE of junction of Lookout Canyon and Cerrososo Canyon; 18 air mi NNW of Cimarron, 23 Jul 2007, *Legler, B. 6420* (RM); Colfax County, Vermejo Park Ranch: along south side of NM Hwy 555 at Crow Canyon access gate; 15.8 air mi W of Raton, 15 Jul 2008, *Legler, B. 9744* (RM); Taos County, Peñasco, 18 Aug 1939, *Marcelline, S.M. 2865* (F); Cloverdale, west of The Animas Valley, 15 Jul 1892, *Mearns, E.A. 450* (NY, US); Hidalgo County, Base of San Luis Mts, 5 Sep 1893, *Mearns, E.A. 2156* (US); Grant County, at Mangas Springs, 18 miles NW of Silver City, Jul 1901, *Metcalfe, O.B. s.n.* (MO); Grant County, Collected in the Gila River bottom, 13 Jun 1903, *Metcalfe, O.B. 138* (BM, E, GH, K, MO, NY, US); Sierra County, Kingston, Collected in and around the south end of the Black Range, 17 Jun 1904, *Metcalfe, O.B. 1005* (BM, E, F, GH, NY, US); Grant County, Fort Bayard, 25 Jul 1895, *Mulford, I. 460* (MO); San Juan County, 6 2/3 mi. N of Pueblo Pintado by road, 16 Jun 1976, *Reitzel, J. 4184* (NY); Dona Ana County, Mesilla Valley. Old field, 25 Jun 1906, *Standley, P.C. 420* (US); San Miguel County, near Pecos, 15 Aug 1908, *Standley, P.C. 4923* (GH, NY, RM, US); San Miguel County, near Pecos, 15 Aug 1908, *Standley, P.C. 4928* (MO); Union County, On and near the Sierra Grande, 19 Jun 1911, *Standley, P.C. 6114* (US); Rio Arriba County, Along the river. Vicinity of Chama, 8 Jul 1911, *Standley, P.C. 6604* (US); San Juan County, Waste ground. Vicinity of Farmington, 17 Jul 1911, *Standley, P.C. 6911* (US); San Juan County, Sandy field. Navajo Indian Reservation, in the Tunitcha Mountains, 8 Aug 1911, *Standley, P.C. 7737* (US); Colfax County, Vicinity of Ute Park, 5 Sep 1916, *Standley, P.C. 14377* (NY); sin. loc, 1902, *Stevenson, M.C. 141* (US); sin. loc, 1881, *Vasey, G.R. s.n.* (W); 1880, *Vasey, G.R. s.n.* (WIS); Socorro County, Socorro, May 1881, *Vasey, G.R. s.n.* (US); Socorro County, Socorro, May 1881, *Vasey, G.R. 352* (BM, NY); Sandoval County, along US Rt. 350, 6.2 miles South of its junction (in Cuba) with St. Rt. 197; in

Cuba, 14 Aug 2001, *Vincent, M.A. & Lammers, T.G. 9965* (SRP); Union County, Near Mount Dora, 26 Jul 1941, *Wiltbank, W.E. 307* (RM); Colfax County, Castle Rock, 28 Aug 1913, *Wooton, E.O. s.n.* (NY); Grant County, Head + Wilson Ranch [Head and Wilson ranch, N of Carlisle [mine]], 13 Jul 1900, *Wooton, E.O. s.n.* (US); Socorro County, Manguitas Spring [western Socorro County fide Wooton & Standley 1915], 23 Jun 1892, *Wooton, E.O. s.n.* (US); Socorro County, Manguitas Spring [western Socorro County fide Wooton & Standley 1915], 23 Jun 1892, *Wooton, E.O. 49* (US); Lincoln County, White Mountains, 26 Jul 1897, *Wooton, E.O. 214* (E, GH, K, MO, NY, RM, US); San Juan County, Farmington, 8 Aug 1904, *Wooton, E.O. 2684* (US); McKinley County, Ojo Caliente [georef to Zuni reservation site, another one in Sierra County, from Wooton & Standley 1915 could be either], 18 Jul 1904, *Wooton, E.O. 2686* (US). **North Dakota**: Rolette County, vicinity of Pipestone on US Rt. 281, 11 Jul 1971, *Ahles, H.E. 72523* (H); Pembina County, Walhalla, 8 Jul 1912, *Bergman, H.F. 2057* (MO); Pierce County, Rugby, 30 Jul 1912, *Bergman, H.F. 2568* (MO); Willow City, *Brannon, M.A. s.n.* (WIS); sin. loc, 20 Aug 1911, *Brenkle, J.F. s.n.* (W); Eddy County, New Rockford, 1885, *Butler, E. s.n.* (F); Ransom County, Ft. Ransom, 14 Oct 1898, *Fieldstad, F.L. s.n.* (NY); Burke County, In gravel in road, Des Lacs National Wildlife Refuge, 2 miles north-northeast of US Highway 52 bridge, 5 Aug 1942, *Hotchkiss, N. 6821* (US); Billings County, near Medora, 16 Aug 1927, *Larsen, E.L. 176* (MO); Benson County, Leeds, 12 Sep 1906, *Lunell, J. s.n.* (NY); Benson County, Leeds, 22 Sep 1907, *Lunell, J. s.n.* (NY); Benson County, Leeds, 16 Sep 1909, *Lunell, J. s.n.* (NY); Benson County, Leeds, 13 Aug 1915, *Lunell, J. s.n.* (US); Benson County, Leeds, 11 Aug 1900, *Lunell, J. s.n.* (GH); Bottineau County, Willow City, 21 Jul 1896, *Rider, S.L. 344* (F); Bismarck, 30 Aug 1884, *Seymour, A.B. s.n.* (GH, WIS); Burke County, 20 miles SW Bowbells, 4 Jul 1967, *Stephens, S. & Brooks, R. 13113* (GH); Ransom County, near Leonard, 3 Sep 1936, *Stevens, C.A. 226* (F); Ransom County, Leonard, 3 Sep 1936, *Stevens, O.A. 226* (WIS); Mercer County, Bare field below Great Hidatsa Village site mouth of Knife River -see Rhodora 48:102 [unsure placement], 16 Aug 1941, *Stevens, O.A. 913* (US); Stark County, Dickinson, 7 Aug 1951, *Stevens, O.A. 1287* (ID, US); Thedford [possibly Nebraska], 14 Jul 1889, *Webber, H.J. s.n.* (MO); Kidder County, on Mike Krous Farm, 5 mi E, 6.5 mi N of Tappen, 11 Jul 1972, *Williams, R.P. 1139* (MO); Kidder County, 10 mi N. 3.5 mi E of Robinson, 11 Aug 1972, *Williams, R.P. 1351* (MO). **Ohio**: Belmont County, Barnesville, L. Miller's pigeon yard, 16 Jun 1918, *Laughlin, E.E. s.n.* (OS); Belmont County, Barnesville, L. Miller's pigeon yard, 16 Jun 1918, *Laughlin, E.E. s.n.* (OS); Warren, Lebanon, Aug 1890, *Nelson, N.L.T. s.n.* (MIN); Warren, Lebanon, Aug 1890, *Nelson, N.L.T. s.n.* (MIN). **Oklahoma**: Kingfisher County, sin. loc, 25 May 1896, *Blankinship, L.A. s.n.* (GH, US); Neutral Strip [=Oklahoma Panhandle, incl. Cimarron, Beaver and Texas counties], Aug 1891, *Carleton, M.A. 382* (US); Kingfisher County, Holbrook, 2 Jul 1896, *Luck, M. s.n.* (MO); Blaine County, near Canton, 10 Jun 1913, *Stevens, G.W. 852* (GH, K, MO, NY); Harmon County, near Hollis, 23 Jun 1913, *Stevens, G.W. 1096* (GH); Cimarron, Signature Rock Area, 7 mi W and 5.5 mi N of Boise City, 11 Aug 1977, *Taylor, J. & Tayler, C. 25237* (WIS); Cimarron County, 4 miles north of Kenton, 10 Aug 1949, *Waterfall, U.T. 9248* (GH, NY); Woods County, Woods County, 27 Jun 1900, *White, P.J. s.n.* (WIS); **Oregon**: Deschutes County, Biak Training Center; southeast of Redmond; on top of the North Unit Main Canal levee, 1.2 miles, 216Â° southwest of the southern Bailey Bridge Site, 10 Aug 1999, *Alexander, J.A. 1045* (OSC); Wasco County, Tygh Ridge road, aprx. 6 miles east of Hwy 197 near cemetery, Jul 1985, *Carolyn Wright*, *& Gross, D. 1775* (OSC); Harney County, Pueblo Mtns. along REaux Creek road to Domingo Pass, Jul 1985, *Carolyn Wright*, *& Gross, D. 1779* (OSC); Crook County, Hwy 26, 1.5 mi. northeast of jct. with Ochoco Creek Rd. ca. 16 mi. east-northeast of Prinville, Jul 2013, *Carr, G.D. 1600* (OSC); Malheur County, J.I. Brady farm, 4 miles NW of Nyssa, 3 Sep 1953, *Clarkson, Q.C. 277* (OSC); Lake County, sandy roadside south of Paisley, Jul 1928, *Constance, L. 9939* (OSC); Malheur County, Sandhills, 1 mile W of Malheur Butte, between Vale and Ontario, Twp. 18S, R. 46 E, about S8, 13 Jun 1955, *Cronquist, A.J. 7827* (NY, W); Malheur County, 1 mile west of Malheur Butte, between Vale and Ontario, 13 Jun 1955, *Cronquist, A.J. 7827* (GH, NY, WS, WTU); Lake County, Sand Dunes 1/2 mile west of Mound Springs (north-east of Christmas Valley), 28 Aug 1980, *Crosby, V. 2595* (OSC, WTU); eastern Oregon, 10 Sep 1908, *Cusick, W.C. 3326 a* (OSC, SRP, WS, WTU); Deschutes County, near Cline Buttes, Aug 1942, *Detling, L.E. 5438* (OSC); Harney County, along highway 8 miles north of Burns, 6 Aug 1937, *Dillon, L.A. 915* (GH, MO, NY, WS, WTU); Deschutes County, Redmond, Jun 1934, *Dykstra, T.P. s.n.* (OSC); Malheur County, Mud Springs in Leslie Gulch, ca 24 airmi NNE of Jordan Valley, 24 Jul 2014, *Ertter, B.J. &*

Fields, P. 22113 (CIC); Multnomah County, East end of Hayden Island near the "Interstate Bridge", Sep 1915, *Flinn, M.A. s.n.* (OSC); Multnomah County, East end of Hayden Island, Columbia River, 17 Oct 1915, *Flinn, M.A. 1524* (HPSU); Multnomah County, East end of Hayden Island, Columbia River, 7 Oct 1915, *Flinn, M.A. 1525* (HPSU); Multnomah County, East of Hayden Island, Columbia River, 7 Oct 1915, *Flinn, M.A. 1526* (HPSU); Morrow County, Irrigon, Jul 1937, *Freese, C.R. s.n.* (WS); Gilliam County, Near vegetation transect group BT5 at Pebble Springs Study Area, 28 Sep 1977, *Glad, J.B. 77-74* (CIC); Gilliam County, sunny and grassy slopes near Condon, Sep 1925, *Gorman, M.W. s.n.* (OSC); Sand spits to sandy banks at head of Hayden Ild. Portland, 23 Sep 1915, *Gorman, M.W. 3657* (WS); Harney County, vicinity of Diamond Craters, Bureau of Land Managment, just East of Malheur Natl. Wildlife Refuge, 12 Sep 1979, *Gruber, E.H. 232* (OSC); Harney County, between Lawen and Crane, 8 Jul 1952, *Hansen, C.G. s.n.* (OSC); Harney County, Steens Mts. Region, 4 1/2 mi. east and 1 1/4 mi. due north Frenchglen, Sep 1954, *Hansen, C.G. 1055* (OSC); Grant County, John Day River, These, Kinberlay, 23 Jun 1925, *Henderson, L.F. 5510* (GH, MO, OSC); Baker County, Roadside ditch 5 miles south of North Powder, 15 Jul 1952, *Hitchcock, C.L. 19660* (WS, WTU); Harney County, Catlow Rim (Frenchglen-Fields) Road, at crossing of Home Creek, 20.3 km (12.6 mi) south of Steens Mountain Loop Road turnoff, 29 km (18mi) air distance west-northwest (292 degrees) of Andrews, Aug 1985, *Holmgren, N.H. & Holmgren, P.K. 11049* (NY, OSC); Jefferson County, Priday Ranch; road to Horse Heaven Mine 17 miles North of Madras then 5-6 miles East of the highway, Jul 1940, *Ireland, O.L. 1552* (OSC); Malheur County, Three Fingers Gulch, 8 Sep 1989, *Kaye, T.N. 1152* (OSC); Morrow County, Balm Fors, Aug 1979, *Kerr, H. s.n.* (OSC); Baker County, none, *Ketels, J.F. s.n.* (OSC); Gilliam County, Clem, Aug 1956, *Kirsch, E.J. s.n.* (OSC); Malheur County, near Cow Lakes, 29 Sep 1974, *Kotter, M. 25 MK* (CIC); Wheeler County, About 9 miles up the county road towards Rancheria from Twickingham Bridge. Blue Mountains foothills, 29 Jul 1987, *Ladd, M. s.n.* (WCW); Harney County, hill region near Burns, Aug 1913, *Lawrence, W.E. s.n.* (OSC); Sherman County, northeast of Moro, 7 Jul 1917, *Lawrence, W.E. 416* (F, OSC); Klamath County, 10 miles South of Klamath Falls, 6 Aug 1919, *Lawrence, W.E. 2588* (OSC); Union County, near Telocaset, Aug 1922, *Lawrence, W.E. 3592* (OSC); Harney County, 25.5 miles south of Crane, 3 Jul 1927, *Lawrence, W.E. 4087* (OSC); Harney County, South of Burns, 13 Jul 1927, *Lawrence, W.E. 4368* (MO, OSC); Sherman County, Clodfetter Ranch, Jul 1928, *Lawrence, W.E. 4576* (OSC); Lake County, meadow 3 mi. west of Lakeview, Jun 1927, *Leach, L. 448* (OSC); Malheur County, Owyhee Uplands, Dry Creek basin, Dry Creek at crossing with Crowley Road, 42 air miles southwest of Vale, Jul 2004, *Lytjen, D. & Otting, N. 250* (CIC, OSC); Malheur County, Jordan Craters North Quad. Growing in disturbed dirt roadside by the Birch Creek take-out campground about 2 miles down river from the Birch Ck. Ranch, 12 Sep 2002, *Mansfield, D. 02-1021* (BLMVL); Malheur County, Jordan Craters North Quad. By the Birch Creek take-out campground about 2 miles down river from the Birch Ck. Ranch, 12 Sep 2002, *Mansfield, D.H. 21021* (CIC, NY); Jefferson County, North Grizzly Pasture, 9 Aug 1956, *Mohan, J. 1024* (ID); Multnomah County, Hayden Island, opposite Vancouver, 11 Oct 1919, *Nelson, J.C. 2942* (GH); Multnomah County, sandy shores of Columbia R. on Hayden I. opp. Vancouver, Wash.; western Oregon, Jun 1922, *Nelson, J.C. 4779* (OSC); Malheur County, road from Juntura to Riverside, Jul 1977, *Packard, P.L. 77-112* (CIC, OSC); "Oregon" [sin. loc.], 3 Aug 1936, *Parker, C.S. s.n.* (MO); Deschutes County, Harper, Aug 1937, *Parsell, C.C. s.n.* (OSC); Union County, Kamela, Jul 1915, *Peck, M.E. 6920* (OSC); Malheur County, Brogan, Jun 1910, *Peck, M.E. 6923* (OSC); Union County, La Grande, Jul 1910, *Peck, M.E. 6924* (OSC); Wasco County, Bank of John Day R. Clarno, 3 Jul 1921, *Peck, M.E. 10000* (NY, OSC); Lake County, Goose Lake Valley near Lakeview, Jun 1927, *Peck, M.E. 15307* (OSC); Klamath County, Fremont Forest, south end Sawmill field, 7 Aug 1936, *Richardson, 73648* (WTU); Deschutes County, east of Sisters 10 miles on Highway 126, Jul 1971, *Rodin, R.J. 8555* (OSC); Malheur County, in an onion field by Ontario Experiment Station, 5 Jun 1956, *Ross, J. s.n.* (OSC); Harney County, Virginia Valley, S. Burns, 3 Jul 1927, *Scullen, H.A. 396* (OSC); Deschutes County, Bend, Rawhide Drive, 3 Oct 2004, *Smith, R.M. s.n.* (W); Deschutes County, Forum Dr. Bend, 11 Aug 2004, *Smith, R.M. s.n.* (OSC, WS); Lake County, 30 miles e. of Hart Mt, Jul 1992, *Spencer, S. s.n.* (HPSU); Baker County, Richland, Oregon, 8 Sep 1928, *Sprague, R. & Gilkey s.n.* (SRP); Harney County, 2 miles north of fields, on Cattow Valley Road, Aug 1954, *Steward, A.N. & Hansen, C. 6831* (OSC); Wasco County, 1 mi. S.W. of Wamic, 12 Oct 1955, *Steward, A.N. & Steward, C.B. 7018* (OSC); Harney County, near Narrows, 10 Jul 1935, *Thompson, J.W. 11984* (MO, WTU); Douglas County, N. Fork

Umpqua River at Panther Leap, 3 Apr 1977, *Wagner, D. 1159* (OSC); Grant County, John Day Valley near Spray, 3 Sep 1936, *Without Collector s.n.* (WCW). **South Dakota**: Hand County, Ree Heights, *Butler, E. s.n.* (F); Little Missouri River, Aug 1882, *Canby, W.M. s.n.* (F); Butte County, Horse Creek, near Newell, *Carr, W.P. 162* (US); Meade County, Black Hills, near Ft. Meade, 16 Aug 1887, *Forwood, W.H. 276* (US); Stanley County, about Fort Pierre, 1839, *Geyer, C.A. s.n.* (NY); Stanley County, Fort Pierre, 6 Jun 1839, *Geyer, C.A. 248* (US); R.R.Rit o way, Faith, 10 Aug 1912, *Moyer, L.R. 230* (NY); Custer, 16 Jul 1892, *Rydberg, P.A. 905* (NY, US); Iroquois, "Iroquoix", 7 Aug 1894, *Thornber, J.J. s.n.* (GH); McPherson County, 9 mi W of Leola off SD 10; Samuel H. Ordway Jr. Memorial Prairie, 10 Jul 1988, *Umbanhowar, C.E. 45* (WIS); Harding County, Boxelder Creek, 15 Jul 1910, *Visher, S.S. 300* (F); Harding County, Extreme northwest corner of county, 15 Jul 1910, *Visher, S.S. 304* (RM); Washington County, Cheyenne Valley, 23 Jul 1911, *Visher, S.S. 2147* (F); Fall River County, Oelrichs, 4 Aug 1902, *Visher, S.S. 2722* (F); Walworth County, Swan Wells, 31 Jul 1911, *Visher, S.S. 3326* (MO); White River, 16 Jul 1896, *Wallace, E.J. s.n.* (NY). **Texas**: El Paso County, Canutillo, Rio Grande Valley, 7 Jul 1911, *Barlow, B. s.n.* (F); Fort Union, Jul 1854, *Hayden, F.V. s.n.* (MO); Randall County, Canyon, State Normal School Campus, 3 Jun 1918, *Palmer, E.J. 13882* (H, MO, US, WIS); Deaf Smith County, Hereford, *Reed, E.L. 3546 a* (US); Hall County, Vermon, Estelline, 8 Jul 1903, *Reverchon, J. 3919* (GH, MO); Hemphill County, 5 miles E of Canadian, 29 Jun 1955, *Rowell Jr, C.M. 4218* (); Hemphill County, 5 mi. E of Canadian, 29 Jun 1955, *Rowell, C.M. 4218* (TEX); Culberson County, Ploughman Tank, West Dog Canyon, Guadelupe Mountains, 3 Sep 1954, *Warnock, B.H. 11990* (LL); Bexar County, San Antonio River, 19 Apr 1931, *Whitehouse, E. s.n.* (); Bexar County, San Antonio River, 19 Apr 1931, *Whitehouse, E. s.n.* (TEX). **Utah**: Garden, Campus, Cortez Quad UTM 71048E, 413652N, 18 Sep 1990, *Adams, K. 114* (NY); San Juan County, San Juan Co. Utah: Ca. 10 miles west of Hwy 163 on Hwy 95. Disturbed dry wash area in Pinyon-juniper, 7 Jun 1976, *Albee, B. & Arnow, L. 3177* (UT); Salt Lake County, Salt Lake Co. Utah: Salt Lake City, University of Utah Campus, 15 Aug 1967, *Arnow, L. 544 a* (UT); Salt Lake County, Salt Lake Co. Utah: 10 mi. SW of Salt Lake City, Utah in dry sitch along road S of County Dump at 13 S and 5400 W, 11 Jul 1971, *Arnow, L. 3189* (UT); Salt Lake County, Salt Lake Co. Utah: SW of Salt Lake City at Jordan River crossing at 7200 S between towns of W. Jordan and Midvale. Along ditch ca. 100 yds. W of the river, 26 Aug 1975, *Arnow, L. 4692* (UT); Wasatch County, Wasatch Co.: Ca. 3 mi SW of town of Midway, ca. 2 mi W of head of Deer Creek Reservoir, in an area 100-200m W of Cascade Springs Rd, 23 Jun 1991, *Arnow, L. 6779* (UT); Salt Lake County, University of Utah Campus, U of U Campus, 15 Aug 1974, *Arnow, L. 7175* (UT); Washington County, 2.5 miles south of Hebron along Enterprize. Reservoir road, 26 Jul 1991, *Atwood, D. 16093* (ID); Washington County, Foothills south of Pinto on the north slope of the Pine Valley Mt, 18 Aug 1973, *Atwood, N.D. 5893* (NY); Millard County, Ca 4 mi SW of Minersville, T29S, R11W, S 12, 7 Sep 1988, *Atwood, N.D. & Thompson, B. 13495* (MO); Washington County, 2.5 miles south of Hebron along Enterprize. Resevoir road, 26 Jul 1991, *Atwood, N.D. 16093* (NY); Grand County, Cottonwood Canyon; s. Book Cliffs. Also T19S, R22E, S6, 3 Oct 2003, *Atwood, N.D. 30058* (NY); Millard County, Devils Kitchen, ca. 8 mi. E of Spring Lake on Ut Hwy 101, 3 Sep 1984, *Baird, G.I. 1498* (NY); Washington County, Beaver Dam Wash, Lytle Ranch, along stream from reservoir to house, 7 Jul 1985, *Baird, G.I. 1720* (NY); Washington County, Beaver Dam Wash, Lytle Ranch, along stream ca. 3/4 mile north of ranch house, 28 Aug 1985, *Baird, G.I. 1820* (NY); Salt Lake County, Salt Lake City, weed in garden bed at 1437 E Gilmer Dr.; LB DNA #861, 9 Aug 2001, *Bohs, L. 3062* (BM, UT); Salt Lake County, Salt Lake City. Cultivated in University of Utah greenhouse, 7 Sep 2001, *Bohs, L. 3068* (NY); Washington County, Washington Co. Utah. In bottom of Santa Clara River canyon ca. 1 mile below the U.S. Highway 91 bridge; NW1/4 Sec. 35 T41S R17W, 22 Jun 1963, *Christian, R.W. 1031* (UT); Summit County, Kamas, Summit County, 17 Aug 1928, *Cottam, W.P. 3877* (UT); Iron County, ca. 45 miles N of Cedar City, 11 Sep 1976, *Davis, T. 662* (MO); Iron County, between Fort Hamilton and Kanarrville, 4 Sep 1918, *Eggleston, W.W. 14742* (GH); Uintah County, Bench west of Little Brush creek, 13 Sep 1977, *England, J.L. 1062* (NY); Daggett County, Lucerne Valley above Lenwood on bench, Daggett Co. Utah, 22 Jul 1959, *Flowers, S. et al. 94* (UT); 24 Sep 1927, *Flowers, S. 853* (UT); Garfield County, SC Paunsaugunt-Sevier, 2 mi. N of Mud Spring Creek on Rt 12, 24 Aug 1977, *Foster, R. 5470* (NY); Utah, 29 Sep 1906, *Garrett, A.O. 2087* (UT); Salt Lake County, R.R. track towards Parley's Canyon. Salt Lake Co, 28 Aug 1907, *Garrett, A.O. 2184* (NY, UT); Near [Bathurst?], 21 Jun 1929, *Garrett, A.O. 5219* (UT); Summit County, Summit Co. Utah, 2 Aug 1937,

Garrett, A.O. 7480 (UT); Along Flood Plain, East Canyon, Morgan Co, 19 Sep 1943, *Garrett, A.O. 8671* (UT); Ephriain Plateau, 13 Aug 1927, *Harris, J.A. 27756* (MO); Uintah County, Vernal, 14 Jul 1933, *Hermann, F.J. 4912* (GH, MO); Wayne County, CC Fremont, 8 Mi SSW of Fish Lake on a dirt road off Hwy 24, 11 Aug 1977, *Higgins, L.C. 10569* (NY); Iron County, Modena Draw, 9 km (5.6 mi) north of State Route 56 (from Modena Junction) [The elevation also appears on the label as 5990 ft.], 19 Aug 2001, *Holmgren, N.H. 14516* (NY); Iron County, Markagunt Plateau, Parowan Canyon, State Route 143, south of Parowan, 13 Aug 2009, *Holmgren, N.H. 16155* (NY); *Howard, O. s.n.* (UT); Duchesne County, Chokecherry Canyon, Antelope Drainage, 12 Aug 1999, *Huber, A. 4193* (NY); Garfield County, Bryce Junction, 24 Aug 1928, *Hunnewell, F.W. 10821* (GH); Alta, Wahsatch Mountains, Aug 1879, *Jones, M.E. s.n.* (NY); Utah County, American Fork Canyon, 29 Jul 1880, *Jones, M.E. 1485* (BM, F); Emery County, Central and Southern Utah. Huntington, 19 Jun 1894, *Jones, M.E. 5464 h* (US); Salt Lake County, Murray, 23 Aug 1916, *Jones, W.W. 234* (GH); Emery County, San Raphael, 15 Apr 1930, *Larsen, E.L. 5830* (MO); Utah County, Provo Bench, Provo, Oct 1933, *Larsen, E.L. 7208* (MO); Salt Lake County, Salt Lake City, 13 Oct 1883, *Leonard, F.E. s.n.* (GH); Garfield County, Along the East Fork of the Sevier River Road ca. 1 mi. S of Hwy. 12 near where the East Fork of the Sevier River crosses the road, 14 Jul 2000, *Madsen, M. 1122* (NY); Garfield County, Mountain Springs Fork of Deer Creek on the E. slope of the Sevier Plateau SW. of Antimony, 22 Aug 2005, *Madsen, M. 2637* (NY); Washington County, Pine Valley Ranger District/Dixie National Forest 3 air mi. n.w. of Irontown. Pine Valley, 16 Sep 2008, *Madsen, M. 4338* (SRP); Rich County, So. side Bear Lake, 14 Aug 1931, *Maguire, B. 311* (NY); San Juan County, Cottonwood Canyon, 17 miles west of Blanding, 24 Jun 1932, *Maguire, B. & Redd, J.D. 2066* (MO); Grand County, Book Cliff Div, E. Tavaputs Plateau, between Moon Ridge & Cedar Camp Ridge, 7 Sep 1983, *Neese, E.J. 15285* (NY); Washington County, St. George, 1875, *Palmer, E. s.n.* (E); Washington County, St. George [The name given in this record as the county was not specified as such on the label but was inferred from the label's locality data.], 1875, *Palmer, E. s.n.* (NY); Washington County, St. George [The name given in this record as the county was not specified as such on the label but was inferred from the label's locality data.], 1875, *Palmer, E. s.n.* (NY); southern Utah, 1875, *Palmer, E.J. 5884* (MO); Morgan County, Weber River, Peterson, plants of Wahsatch Mountains, 18 Jul 1902, *Pammel, L.H. & Blackwood, R.E. 3897* (GH, MO); Iron County, Cedar City, 1885, *Rémy, J. s.n.* (GOET); Daggett County, 7 mi S of Manila; Sheep Creek Canyon, 4 Jun 1966, *Russell, N.H. 66-49* (WIS); Piute County, Along Bullion Creek, above Marysvale, 21 Jul 1905, *Rydberg, P.A. & Carlton, E.C. 7010* (NY, US); Thompson's Spring, 29 Jun 1911, *Rydberg, P.A. 8339* (NY); Western slope of La Sal Mountains, near Little Springs, 5 Jul 1911, *Rydberg, P.A. 8532* (NY); Head of Dry Wash, Abajo Mountains, 11 Aug 1911, *Rydberg, P.A. 9613* (NY); Sevier County, Forest: Fishlake National Forest.Â¶Exact locality: Lost Cr. Div, 29 Aug 1939, *Sargent, W.B. 46 S* (SRP); Salt Lake County, Travers Mountain Range. Camp W. G. Williams Training Area UTM: 12 422600 4475840, 6 Aug 1993, *Shultz, L.M. 570* (NY); Beaver County, 1.5 mi E of Beaver Municipal Airport, 6 Jul 1985, *Taye, A. 3391* (NY); Rich County, Desert Land & Livestock Co, Saleratus Reservoir, 17 Sep 1983, *Thorne, K.H. 3106* (NY); Iron County, Above Bone Hollow Spring, ca. 4.2 mi E of Jct with I15, 24 Sep 1992, *Thorne, K.H. 15167* (NY); Sanpete County, In alfalfa [interpreted] fields, near Ephraim. Wasatch Mountains, 10 Sep 1907, *Tidestrom, I.F. 550* (US); Sanpete County, Gunnison, 2 Sep 1875, *Ward, L.F. 717* (GH, US); Garfield County, "Garfield Co. Utah?", 10 Jun 1935, *Weight, K.E. kew 907 Z* (UT); Washington County, Zion National Park, Zion Nat'l Park, Wash Co, Utah. East Entrance, 17 Jun 1935, *Weight, K.E. 935 Z* (UT); Beaver County, Frisco, 13 Sep 1963, *Welsh, S.L. 2669* (NY); Wasatch County, 23 Jul 1893, *Without Collector s.n.* (UT). **Washington**: Klickitat County, Dot Road, circa 10.8 miles south of Cleveland, 11 Oct 2007, *Brainerd, R.E. 1004* (WTU); San Juan County, American Camp, San Juan Island National Historical Park, 15 Jun 2016, *Combs, J. 170* (WTU); Benton County, White Bluff Prairie, 10 Sep 1926, *Cook, N.M. s.n.* (WIS); Whitman County, La Crosse, 30 Jul 1916, *Domer, B.F. 684* (WS); LaCrosse, Jul, *Dunkle, M.B. s.n.* (ID); Franklin County, Near Eltopia, 9 Jun 1969, *Dunn, D.B. 16383* (NY); Benton County, Vacant lot on the corner of Ash and Arizona, 300 Area West, 31 May 1996, *Fortner, G. & Zufelt, R. s.n.* (PNNL); Franklin County, near Eltopia, 6 Sep 1969, *Franklin, ?, & Dunn, D.B. 16383* (MO); sin. loc, Jun, *Gibb, W.E. s.n.* (WWB); Walla Walla County, 1-2 Mi above Luckenbill bridge on Touchet River, 13 Sep 1974, *Grable, A.E. 6000* (WS); Klickitat County, Along the Dallesport Road .2 miles west of its junction with U.S. Hwy. 197 near the N end of The Dalles Bridge over the Columbia River. T:2N

R:13E S:35, 26 Aug 1993, *Halse, R.R. 4702* (BM, K, NY, OSC); Whitman County, Pine City, 30 Jul 1933, *Harthill, M.P. s.n.* (PSM); Franklin County, Columbia River, near Pasco, 12 Jun 1892, *Henderson, L.F. 2495* (WTU); Franklin County, Pasco, 15 Jun 1896, *Hindshaw, H.H. s.n.* (WTU); Yakima County, (YIN) Satus Creek at 4th US Hwy 97 bridge, 25 miles southwest of Toppenish, 3 Oct 1977, *Hunn, E. 765* (WTU); Prosser, Aug 1926, *Jones, G.N. 323* (WS); Kittitas County, Abandoned field along Swauk Creek about 1 mile from Yakima River, 15 Oct 2008, *Knoke, D. 1738* (WTU); Walla Walla County, Burbank, 29 May 1922, *Lechiner, H.J. 1* (WS); Walla Walla County, Burbank, 29 May 1922, *Lechiner, H.J. 9* (WS); Whitman County, Along the fence at the bottom of the north-facing slope, Botany Dept. Prairie Strip, College Farm, Pullman, 8 Jul 1947, *Lingenfelter, R.L. 452* (SRP, WS); Walla Walla County, Luckenbill Bridge, 14 Aug 1974, *McCluskey, L. 271* (WS); Walla Walla County, Luckenbill Bridge, 14 Aug 1974, *McCluskey, L. 272* (WS); Benton County, Hanford Reservation, Upper Snively Canyon, 10 Sep 1972, *O'Farrell, J.T. & O'Farrell, T.P. 72-253* (WS, WTU); King County, Corea, 20 Sep 1918, *Otis, I.C. 873* (WS); Connell, May 1903, *Palmer, A.D.E. s.n.* (WIS); Whitman County, Pullman, along O.W.R. & N.RR. tracks, 13 Sep 1915, *Pickett, F.L. 350* (WS, WTU); Whitman County, Pullman, 29 Jun 1906, *Piper, C.V. s.n.* (WS); Yakima County, North Yakima, 31 Aug 1894, *Piper, C.V. 1806* (F, US, WCW, WS); Benton County, IAREC, Prosser, Jun 1972, *Rincker, P. 59* (EWU); Benton County, 109 Riverwood, Richland, 29 Jun 1992, *Sackschewsky, M.R. 269* (PNNL); Wind Erosion Plot near Beetle Plot #3, 13 Jun 1974, *Sauer, R.H. & Hinds, W.T. s.n.* (PNNL); Benton County, Weed everywhere in fields, Prosser, 9 Jul 1929, *Smith, L. 235* (WSTC); Grant County, Grab Creek, of Moses Lake, 25 Jun 1921, *St. John, H. et al. 4938* (MO, WS, WTU); Whitman County, Near Rock Lake, 18 Oct 1924, *St. John, H. & Warren, F.A. 6772* (WS); Klickitat County, prope Gilmer, 24 May 1908, *Suksdorf, W. s.n.* (E); Klickitat County, Bingen, 3 Aug 1926, *Suksdorf, W. 12045* (BM, GH, K, MO); Klickitat County, Bingen, 5 Sep 1895, *Suksdorf, W.N. s.n.* (GH, WS, WTU); Klickitat County, Bingen, 13 Oct 1894, *Suksdorf, W.N. s.n.* (WS); Spokane County, Road south of Philleo Lake, 24 Jul 1916, *Suksdorf, W.N. 8934* (WS); Klickitat County, Bingen, 3 Aug 1926, *Suksdorf, W.N. 12045* (NY, US, WS, WTU); Kittitas County, Along the Columbia River near Vantage, 21 Jun 1933, *Thompson, J.W. 9069* (WTU); Grant County, In Grand Coulee near Soap Lake, 27 Jun 1935, *Thompson, J.W. 11807* (NY, WTU); Grant County, in Grand Coulee near Soap Lake, 27 Jun 1935, *Thompson, J.W. 11907* (MO); Kittitas County, Ellensburg, Jul 1897, *Whited, K. s.n.* (WS); Kittitas County, Ellensburg, 1898, *Whited, K. 600* (OSC); Whitman County, Weed on C.A. Stratton place, Pullman, 16 Jul 1928, *Wilson, C.B. s.n.* (WS). **Wisconsin**: Sheboygan County, Sheboygan, 30 Jun 1918, *Goessl, C. s.n.* (WIS). **Wyoming**: Park County, Yellowstone National Park. Mammoth Hot Springs, 28 Jul 1902, *[ECS]*, *104* (US); Campbell County, Ca 70 air km SSE of Gillette, 14 air km E of Wyo Hwy 59, 11 Aug 1977, *Allen, E.B. 35* (RM); Albany County, Near Country Road 61, 9 mi from Rock River, 20 Jul 1972, *Asplund, R.O. 72-29* (RM); Carbon County, East end of Freezeout Mountains, Miller Ranch on TB Creek, ca 12.5 air mi N of Medicine Bow, 7 Aug 1985, *Blomquist, F. 8* (RM); Laramie, *Buffum, B.C. s.n.* (WIS); Albany County, University Campus, 29 Jul 1891, *Buffum, B.C. s.n.* (RM); Weston County, Newcastle, 19 Jul 1942, *Degener, O. & Reiler, L. 16190* (F, MO, NY, US); Laramie County, 3 mi W of Cheyenne, 8 Jul 1979, *Dorn, R.D. 3323* (RM); Uinta County, Ca 15 mi S of Evanston, 27 Aug 1987, *Dorn, R.D. 4823* (RM); Goshen County, 28 Aug 1994, *Dorn, R.D. 5816* (RM); Natrona County, Ca 8 mi NW of Arminto, 14 Sep 1997, *Dorn, R.D. 7548* (RM); Natrona County, Ca 8 mi NW of Arminto, 14 Sep 1997, *Dorn, R.D. 7548* (RM); Goshen County, 28 Aug 2003, *Dorn, R.D. 9716* (RM); Johnson County, Powder River Basin: ca 5 air mi NNE of Buffalo, 2 Aug 1979, *Dueholm, K.H. 8620* (RM); Johnson County, Powder River Basin: Jack Moore Reservoir, along Boxelder Creek, ca 1.5 air mi E of south end of Lake De Smet, 3 Aug 1979, *Dueholm, K.H. 8653* (RM); Converse County, Thunder Basin National Grassland and Vicinity: above and N of South Fork Reservoir on County Road 23, ca 20 air mi N of Glenrock; ca 36 air mi NW of Douglas, 20 Jul 2002, *Ebertowski, P.J. 1958* (RM); Campbell County, Thunder Basin National Grassland and Vicinity: sagebrush grassland ca 1.3 mi NW of Soda Well, on Forest Road 1032, ca 31 air mi NNE of Gillette; ca 35 air mi NNW of Moorcroft, 3 Jul 2003, *Ebertowski, P.J. 6025* (RM); Weston County, Thunder Basin National Grassland and Vicinity: Wildcat Creek and hills to SW, on County Road 54, ca 27 air mi SW of Newcastle, ca 36 air mi S of Upton, 9 Jul 2003, *Ebertowski, P.J. 6349* (RM); Converse County, Thunder Basin National Grassland and Vicinity: scoria hills SE of Logan Draw, on Forest Road 1416, ca 23 air mi SE of Wright; ca 49 air mi N of Douglas, 21 Aug 2003, *Ebertowski, P.J. 8468* (RM);

Campbell County, Thunder Basin National Grassland and Vicinity: hills ca 1 mi E of Dry Fork Spring Creek, on two-track road off York or Forest Road 900, ca 29 air mi NNE of Gillette; ca 31 air mi NW of Moorcroft, 25 Aug 2003, *Ebertowski, P.J. 8663* (RM); Converse County, Thunder Basin National Grassland and Vicinity: Red Hills, ca 1 mi S of Antelope Creek, on four-wheel drive road, ca 23 air mi SE of Wright; ca 58 air mi WSW of Newcastle, 27 Aug 2003, *Ebertowski, P.J. 8693* (RM); Laramie County, Campus of Laramie County Community College, SE of Cheyenne, 1 Jul 1983, *Edwards, W.C. 97* (RM); Laramie County, Laramie Mountains: ca 22.5 air mi W of Cheyenne; ca 3.5 air mi NE of Buford, 5 Jul 1979, *Edwards, W.C. 144* (RM); Park County, Along U.S. Hwy 14, ca 2 mi W of Cody, just S of rodeo grounds, 29 Jul 1982, *Evert, E.F. 4523* (RM); Park County, Absaroka Mountains: North Fork Shoshone River Drainage: along U.S. Hwy 14, 16, and 20, ca 7 mi W of Cody, 29 Jul 1982, *Evert, E.F. 4538* (RM); Park County, Absaroka Mountains: North Fork Shoshone River Drainage: at Bill Cody's Ranch Inn, ca 25 mi W of Cody, 18 Sep 1982, *Evert, E.F. 5058* (RM); Park County, North Fork Shoshone River Drainage: Absaroka Mountains: along the north side of the North Fork Shoshone River at Game and Fish public access, ca 1/8 mi E of Wapiti, 26 Jun 1989, *Evert, E.F. 17172* (RM); Hot Springs, Bighorn Basin: Legend Rocks State Historical Site just N of Cottonwood Creek, ca 25 mi NW of Thermopolis, 21 Jun 1997, *Evert, E.F. 33103* (RM); Hot Springs, Bighorn Basin: ridge ca 4 mi W of Gebo; 14 mi NW of Thermopolis, 11 Jun 1998, *Evert, E.F. 34999* (RM); Fremont County, Wind River Range: Torrey Creek Trailhead at end of road, 8 mi SSE of Dubois, 12 Aug 1998, *Evert, E.F. 36428* (RM); Albany County, Laramie: open field directly behind and to the W of [old] Wal-Mart on Grand Avenue, 31 Aug 1991, *Fertig, W. 12368* (RM); Carbon County, North Platte River Valley: southwest end of the Freezeout Mountains, along County Road 121; ca 0.6 mi N of the Medicine Bow River; ca 2 mi SE of the south end of Beer Mug Mountain, 20 Aug 1997, *Fertig, W. 18026* (RM); Belle Fourche Valley, 17 Aug 1897, *Griffiths, D. s.n.* (NY); Fremont County, Riverton, 323 Velma Jo Drive, 10 Jul 1982, *Haines, J. 1987* (RM); Fremont County, East side of Boysen Reservoir, southern edge of bay, 22 Jun 1985, *Haines, J. 4464* (RM); Carbon County, Granite Mountain Uplift: Willow Creek, ca 5 air mi NW of Muddy Gap Junction (Three Forks), 30 Jun 1985, *Haines, J. 4489* (RM); Fremont County, Big Camp Creek Area, ca 6 air mi WSW of Muddy Gap Junction (Three Forks), 4 Jul 1985, *Haines, J. & Haines, G. 4746* (RM); Fremont County, Southeast edge of Trail Lake at inlet of Torrey Creek, ca 8.2 air mi SSE of Dubois; ca 6.9 mi S on Trail Lake Road, 27 Jul 1985, *Haines, J. & Haines, G. 5060* (RM); Fremont County, Granite Mountain Uplift: beside road between Gas Hills and Jeffrey City where it drops down over Beaver Rim, ca 14 mi NE of Jeffrey City, 17 Aug 1985, *Haines, J. 5606* (RM); Carbon County, 0.8 mi from Elk Mountain/I-80 turnoff along Rattlesnake Road, 4 Sep 1977, *Hammel, B. & Hartman, R.L. 655* (RM); Carbon, Carbon CO. Wyoming: Near China Butte Stand 31. Road edge, 10 Jul 1976, *Harper, K.T. 578* (UT); Albany County, Johnson Creek on Boswell Creek Road (Forest Rt. 526), ca. 1.5 mi W of Wyo Rt. 10, 27 Aug 1977, *Hartman, R.L. 4928* (NY, RM); Carbon County, Upper North Platte and Laramie River Drainages: Ferris Mountains: Sand Creek, ca 2 air mi SW of summit of Bear Mountain, 7 Jul 1984, *Hartman, R.L. 17435* (RM); Park County, Southwestern Absarokas: Ishawooa Hills and Creek, 22 Jun 1987, *Hartman, R.L. & Snow, N. 22991* (RM); Sweetwater County, Green River Basin: Eden Reservoir, south side, ca 8.5 air mi NE of Farson, 29 Jul 1994, *Hartman, R.L. 48636* (RM); Park County, Northern Absaroka Area: road on south side of the North Fork Shoshone River, ca 5 air mi E of Wapiti, 13 Aug 1997, *Hartman, R.L. 59600* (RM); Park County, Northern Absaroka Area: Buffalo Bill Reservoir, ca 10.5 air mi E of Wapiti, 13 Aug 1997, *Hartman, R.L. 59688* (RM); Park County, Northern Absaroka Area: Rock Creek and South Fork Road, ca 17 air mi SW of Cedar Mountain summit, 15 Aug 1997, *Hartman, R.L. 59861* (RM); Big Horn County, Bighorn Canyon National Recreation Area: on Yellowtail Reservoir shore ca 3/4 mi N of the inlet of John Blue Canyon, 15 Jul 2001, *Heidel, B. 2083* (RM); Niobrara County, Western part of the Great Plains Prairie: Jireh, 27 Jun 1915, *Hess, W.L. & Edwards, W.C. 109* (RM); Teton County, Hoback River, 5 km (3 mi) air distance east of Hoback Junction, 17 Aug 1997, *Holmgren, N.H. 12923* (NY); Near Ft. Bridger, Aug 1872, *Leidy, J. s.n.* (NY); Natrona County, Roughlock Hills, 16 Jul 1980, *Lichvar, R.W. 3165* (NY); Laramie County, Ca 6 mi W of Midway, ca 2 mi N of U.S. Hwy 85 near silo PA-4, 14 Aug 1984, *Long, D. & Loh, J. von s.n.* (RM); Crook County, Devils Tower National Monument: southwest dog town, 25 Jun 1981, *Marriott, H. 735* (RM); Crook County, Black Hills, Devils Tower trading post and post office, ca .8 air mi E of Devils Tower, 4 Aug 1983, *Marriott, H. 5599* (RM); Crook County, Black Hills, Belle Fourche River Valley, ca. 6 air mi S of Colony, ca. 17

air mi WNW of Belle Fourche, SD, 21 Aug 1984, *Marriott, H. 9319* (NY, RM); Albany County, Laramie, 8 Aug 1900, *Merrill, E.D. 465 [/1]* (US); Laramie, 15 Sep 1894, *Nelson, A. 1141* (GH, NY, RM); Albany County, Laramie, 15 Aug 1900, *Nelson, A. 8076* (GH, K, MO, NY, RM, US); Albany County, Laramie, N 7th between Lewis and Bradley, 1 Oct 1975, *Nelson, B.E. & Nelson, L. 1439* (NY, RM); Hot Springs County, Bighorn Basin: ca 2.5 air mi W of Kirby; ca 1 mi N of Gebo; ca 11 air mi N of Thermopolis, above and along Sand Draw, 20 Jul 1983, *Nelson, B.E. 10240* (RM); Goshen County, Southern Powder River Basin/Southeastern Plains: Southeastern Plains: "Five Buttes", ca 5.3 air mi of Jay Em; ca 27.5 air mi N of Lingle, just E of U.S. Hwy 85, 10 Sep 1984, *Nelson, B.E. 11574* (RM); Crook County, Black Hills: along Boundary Gulch, ca 5 air mi SSE of Beulah, ca 16 air mi ENE of Sundance, 16 Sep 1984, *Nelson, B.E. 11955* (RM); Park County, Northern Absarokas: along County Road 7G0, Sunlight Road, ca 4.5-5.5 air mi ENE of Sunlight Ranger Station, ca 25 air mi NW of Cody, 24 Aug 1985, *Nelson, B.E. 13222* (RM); Goshen County, Southern Powder River Basin/Southeastern Plains: Goshen Hole: along Wyo Hwy 159, ca 5.5 air mi N of Torrington; ca 7.5 air mi E of Lingle, 3 Aug 1993, *Nelson, B.E. 28250* (RM); Converse County, Southern Powder River Basin/Southeastern Plains: Southern Powder River Basin: along Cow Creek and the north end of Cow Creek Buttes, ca 15 air mi ENE of Bill; ca 43 air mi NE of Douglas, 21 Jun 1994, *Nelson, B.E. 31854* (RM); Converse County, Southern Powder River Basin/Southeastern Plains: Southern Powder River Basin: along Lake Creek and the buttes to the SE ca 6 air mi SW of Dull Center; ca 45.5 air mi NNE of Douglas, 21 Jun 1994, *Nelson, B.E. 31962* (RM); Niobrara County, Southern Powder River Basin/Southeastern Plains: Hartville Uplift: along a triburtary of Silver Springs Creek on Silver Springs or County Road 54, ca 8.5 air mi SSW of Lusk, 11 Jul 1994, *Nelson, B.E. 32696* (RM); Converse County, Southern Powder River Basin/Southeastern Plains: Southern Powder River Basin: above the head of Shonsy Draw off Pine Ridge, ca 28 air mi NNW of Glenrock; ca 47.5 air mi NW of Douglas, 13 Jul 1994, *Nelson, B.E. 32805* (RM); Goshen County, Southern Powder River Basin/Southeastern Plains: Goshen Hole: Sinnard Reservoir and vicinity, ca 4.5 air mi WNW of Hawk Springs; ca 19 air mi SSW of Torrington, 4 Aug 1994, *Nelson, B.E. et al. 33441* (RM); Laramie County, Southern Powder River Basin/Southeastern Plains: Southeastern Plains: along Chivington Draw W of Egbert or County Road 154 ca 10 air mi N of Egbert; ca 13.3 air mi NW of Pine Bluffs, 18 Aug 1994, *Nelson, B.E. 33994* (RM); Converse County, Southern Powder River Basin/Southeastern Plains: Southern Powder River Basin: along Ross or County Road 31 between Dry Fork Cheyenne River and Cheyenne River Draw and along Cheyenne River Draw, ca 36 air mi NNW of Douglas, 21 Aug 1994, *Nelson, B.E. 34132* (RM); Converse County, Southern Powder River Basin/Southeastern Plains: Southern Powder River Basin: along Antelope Creek and Ross or County Road 31, ca 31 air mi WNW of Bill; ca 50.5 air mi NNW of Douglas; ca 3.5 air mi SE of Ross, 21 Aug 1994, *Nelson, B.E. 34233* (RM); Converse County, Southern Powder River Basin/Southeastern Plains: Southern Powder River Basin: along Bull Gulch and Ross or County Road 31, ca 31 air mi WNW of Bill; ca 50.5 air mi NNW of Douglas; ca 3.5 air mi SE of Ross, 21 Aug 1994, *Nelson, B.E. 34233* (RM); Carbon County, South-central Wyoming: Sierra Madre: along a tributary of Canary Grove Draw, ca 3/4-1 mi SW of Muddy Creek, ca 28.5 air mi SSW of Rawlins; ca 25.5 air mi NE of Baggs, 21 Jul 1996, *Nelson, B.E. 39204* (RM); Carbon County, South-central Wyoming: Sierra Madre: on the divide between Wild Cow and Little Savery drainages, ca 2.5-3 air mi N of Ketchum Buttes, ca 32 air mi SSW of Rawlins; ca 22.5 air mi NE of Baggs, 21 Jul 1996, *Nelson, B.E. 39235* (RM); Sweetwater County, South-central Wyoming: Washakie Basin: along Patrick Draw or County Road 24, ca 1.5 air mi SW of Bitter Creek siding, ca 32.5 air mi E of Rock Springs; ca 31.5 air mi WSW of Wamsutter, 11 Aug 1996, *Nelson, B.E. 39658* (RM); Carbon County, South-central Wyoming: along Muddy Creek and the Overland Trail, ca 6 air mi SW of Bridger Pass, ca 25 air mi SW of Rawlins; ca 31.5 air mi NNE of Baggs, 19 Aug 1996, *Nelson, B.E. 39998* (RM); Carbon County, South-central Wyoming: along Cottonwood Creek and McCarty Canyon or County Road 503, ca 5 air mi NNE of Dixon; ca 50.5 air mi SSW of Rawlins, 20 Aug 1996, *Nelson, B.E. 40098* (RM); Carbon County, South-central Wyoming: along Cottonwood Creek and McCarty Canyon or County Road 503, ca 5 air mi NNE of Dixon; ca 50.5 air mi SSW of Rawlins, 20 Aug 1996, *Nelson, B.E. 40105* (RM); Campbell County, Thunder Basin National Grassland and Vicinity: along a draw ca 3/4-1 mi S of Porcupine Creek, ca 0.5 mi W of Teckla; ca 14.5 air mi SE of Wright, 15 Aug 2002, *Nelson, B.E. 58088* (RM); Converse County, Thunder Basin National Grassland and Vicinity: Robertson Flat on Steckley or Forest Road 942, ca 13 air mi NW of Bill; ca 50 air mi N of Douglas,

16 Aug 2002, *Nelson, B.E. 58106* (RM); Albany County, Laramie, 14 Sep 1916, *Olson, T. 11* (MO, NY, RM, WIS); Carbon County, Carbon, 18 Jul 1899, *Pammel, L.H. 2* (MO); Platte County, Between Fish Creek and Fish Creek Road, 18 road mi NW of Wheatland, 3 Sep 1989, *Pollet, J. 752* (RM); Fort Bridger, Wyoming Territory [The name given in this record as the state was not specified as such on the label but was inferred from the label's locality data.], 6 Aug 1873, *Porter, T.C. s.n.* (NY); Sweetwater County, Basins and Mountains of Southwest Wyoming: Flaming Gorge National Recreation Area: ca 16 air mi SW of Green River; Blacks Fork crossing on east side of Wyo Hwy 530 on south side of the river, 27 Jul 1994, *Refsdal, C.H. 2165* (RM); Campbell County, Black Thunder Strip Mining Area: North Prong Thunder Creek and Little Thunder Creek, 17 Aug 1973, *Ries, R.E. & Sabinske, D.W. 165* (RM); Carbon County, Upper North Platte and Laramie River Drainages: Pathfinder Reservoir: Tye Draw leading into reservoir, ca 8 air mi N of Seminoe Dam; ca 38 air mi NE of Sinclair, 12 Jul 1997, *Roderick, A.J. 2084* (RM); Carbon County, Upper North Platte and Laramie River Drainages: Fort Steele State Recreation Area, ca 6.6 air mi NW of Walcott Junction; ca 8.4 air mi E of Sinclair, 6 Aug 1997, *Roderick, A.J. 3293* (RM); Natrona County, Upper North Platte and Laramie River Drainages: along Poison Spider Road, ca 19.4 air mi SE of Waltman, 15 Aug 1998, *Roderick, A.J. 8296* (RM); Carbon County, Hayden National Forest: Encampment, 9 Sep 1923, *Rose, F.H. 202* (RM); Carbon County, Hayden National Forest: Encampment, 9 Sep 1923, *Rose, F.H. 202* (RM); Park County, Northwestern Wyoming. Near Marquette [now under Buffalo Bill Reservoir, confluence of N and S fork of Shoshone River], 4 Aug 1893, *Rose, J.N. 116* (US); Weston County, 20-24 air mi S of Newcastle, 16 Jul 1976, *Schreibeis, R.G. E-617* (RM); Fremont County, Weed in Lee Corey's yard, 31 Jul 1984, *Scott, R.W. 3916* (RM); Sheridan County, Sheridan, 8 Aug 1912, *Sharp, S.S. 241* (RM); Sweetwater County, Green River Mountains, 1934, *Sheppard, W.B. 76* (MO); Carbon County, Ferris Road, ca 10 air mi WSW of Ferris and ca 28 air mi NNW of Rawlins, 26 Aug 1981, *Storto, J. & Wood, T. 301* (RM); Between Sheridan and Buffalo, 15 Jun 1900, *Tweedy, F. 3531* (NY, RM); Carbon County, Encampment, 10 Jul 1901, *Tweedy, F. 4619* (NY, US); Carbon County, Ca 27 air mi N of Rawlins, 15 Aug 1981, *Warren, A. 137* (RM); Sweetwater County, State of Wyoming. Sweetwater Co. WYO: 35 miles E of Rock Springs along Interstate 80, 9 Aug 1984, *Wiens, D. s.n.* (UT); Carbon County, Sierra Madre: ca 1.5 air mi S of Encampment, 14 Aug 1984, *Williams, R.L. 32* (RM); Carbon County, Sierra Madre: North Fork Encampment River, ca 2 air mi SW of Encampment, 6 Aug 1988, *Williams, R.L. 734* (RM); Laramie County, Cheyenne River at Mouth of Ludian Creek, 24 Aug 1891, *Williams, T.A. s.n.* (MO); Hot Springs County, Wind River, *Williams, T.A. s.n.* (GH).

18. ***Solanum villosum* Mill.**
CANADA. **British Columbia**: Vancouver, Marine Way, S side, 300 m E of Kinross Street; base of construction fence, 29 Jul 2015, *Lomer, F. 9626* (UBC).

UNITED STATES OF AMERICA. **California**: Alameda County, UCGarden, Berkeley, 23 Oct 1930, *McKay, J. s.n.* (K, MO). **Florida**: Escambia County, Pensacola, 25 Jul 1899, *Curtiss, A.H. 6493* (E, K, MO, MO, UC). **Idaho**: Ada County, Boise, 1881, *Dr Philcox*, *s.n.* (NY). **Illinois**: Cook County, Chicago, 4200 Hazel Street, garden, 18 Sep 1982, *Nee, M. 18239* (CORD); **Maryland**: Baltimore County, Chrome ore piles, Canton, Md. Baltimore, 27 Sep 1953, *Reed, C.F. 32667* (MO); Baltimore County, Chrome ore piles, Canton, Md. Baltimore, 27 Sep 1953, *Reed, C.F. 32796* (MO); Baltimore County, Chrome ore piles, Canton, Md. Baltimore, 12 Oct 1953, *Reed, C.F. 32880* (MO); Baltimore County, Canton, 12 Oct 1953, *Reed, C.F. 32891* (MO). **Massachusetts**: Middlesex County, Cambridge, 28 Aug 1913, *Fernald, M.L. & Long, B. 10339* (GH, NEBC). **Missouri**: St. Louis, Cult. MBG [Missouri Botanical Garden] 102/98, 28 Jun 1838, *Norton, J.B.S. s.n.* (MO). **New Jersey**: Camden County, Camden, Ballast, NY or NJ, Sep 1879, *Martindale, I.C. s.n.* (MO); Newark County, Along RR, Newark, 29 Aug 1979, *Reed, C.F. 104036* (MO). **Pennsylvania**: Philadelphia County, Philadelphia, foot of Wolf Street and Delaware River, Piers 82 & 84, 9 Oct 1920, *Meredith, H.B. s.n.* (GH); Philadelphia, "Ballast Ground" below the Navy Yrd [Yard], 1 Sep 1866, *Smith, A.H. s.n.* (PH). **Washington**: Walla Walla County, Walla Walla region, May 1883, *Tweedy, F. 831* (NY).
