# Supplementary material for: A revision of the Morelloid Clade of Solanum L. (Solanaceae) in North and Central America and the Caribbean
Source: PhytoKeys. 2019 May 30;123:1–144. doi: 10.3897/phytokeys.123.31738 (PMC6554266; doi:10.3897/phytokeys.123.31738)
Supplement: Supplementary material 2 [file phytokeys-123-001-s002.pdf]

Appendix 2. Index to numbered collections

Here we record the collector and number for all numbered collections across the entire of range of the species (incl. the Old World and South America) included in this revision. Only first collectors in collections made by two or more collectors are listed here; pairs of collectors are listed in the Specimens examined sections of species treatments, while groups of three or more are listed as *et al*. Collections by anonymous collectors without date or other identifying features are not listed here. Full collector strings can be found on Solanaceae Source (http://www.solanaceaesource.org) or on the NHM Data Portal (https://doi.org/10.5519/0079673).

7to Semestre B 15 (*nigrescens*).

Abbott, J.R. 16181 (*americanum*).

Abbott, W.L. 30 (*emulans*).

Abd El Ghani, M. 3184, 6139 (*villosum*).

Abdallah, C.S. 1630 (*villosum*).

Abeid, Y.S. 150, 533 (*villosum*); 2848 (*scabrum*).

Aberdeen University Amanus Expedition C1170, D1234 (*villosum*).

Abrams, L. 23, 690, 2488 (*douglasii*); 2520, 2570 (*americanum*); 3488 (*douglasii*).

Abrams, L.R. 75, 195, 346, 425 (*douglasii*).

Abrão, H.R.S. 5 (*americanum*).

Abruzzi, M.L. 1104 (*americanum*).

Abu-Laila, K. 70-4 (*nigrum*); 70-2 (*villosum*).

Acevedo R, R. 1105 (*nigrescens*).

Acevedo Rodríguez, P. 3140, 4254 (*americanum*).

Acevedo, F. 253 (*douglasii*).

Acocks, J.P.H. 21049 (*triflorum*).

Acosta R, I. 1948, 2104, 2143 (*nigrescens*).

Acosta, L. 3926 (*nigrescens*).

Acosta, L.E. 17 (*nigrescens*).

Acosta-Solís, M. 5351, 6055 (*americanum*); 7314, 7940, 11201 (*macrotonum*).

Acra, L.A. 137 (*americanum*).

Adam, J.G. 18728 (*scabrum*); 20529 (*americanum*); 23788 (*scabrum*).

Adames, P. 577 (*scabrum*).

Adams, C.D. 6145 (*americanum*); 10620, 10653, 11219 (*macrotonum*); 11223 (*nigrescens*); 11774 (*americanum*).

Adams, K. 114 (*triflorum*).

Adams, L.G. 537 (*triflorum*).

Adamson, R.S. 3225 (*retroflexum*); 3317 (*scabrum*).

Addor, E.E. 172 (*triflorum*).

Adelowokan, M.A. 5 (*nigrum*).

Adema, F. 499 (*triflorum*).

Adsersen, A. 174, 506 (*americanum*).

Aedo, C. 10790 (*americanum*); 13543 (*nigrum*).

Aellen, P. 3 (*villosum*); 8, 16, 18, 63975 (*nigrum*).

Agacino, M. 164934 (*villosum*).

Agha, A.S. NHI-5370 (*villosum*).

Agnew, A.D.Q. 1227, 1228, 5598 (*villosum*).

Agostini, G. 00024 (*nigrescens*); 1031 (*americanum*).

Agra, M.F. 96, 786, 790, 792, 1108, 1110, 1114, 1116, 1245, 1292, 2111, 3138, 3892, 5784 (*americanum*).

Agudelo, C.A. 1566, 1703 (*macrotonum*).

Águilar M, G. 562, 2294 (*nigrescens*); 8184 (*americanum*).

Aguilar Santelises, R. 650 (*nigrescens*).

Aguilar, R. 1744 (*americanum*); 2073 (*nigrescens*); 3860, 5046 (*macrotonum*).

Aguilar, S. 498 (*douglasii*).

Aguirre G, L.E. 192 (*macrotonum*).

Aguon, C. 134 (*americanum*).

Ahart, L. 4830, 5440, 8200, 8261, 8261, 8319 (*americanum*); 9152 (*triflorum*); 13296, 13377 (*americanum*); 13517, 13848 (*nitidibaccatum*); 14573 (*americanum*); 15329, 15339 (*triflorum*); 16540 (*americanum*); 17083 (*triflorum*); 17138 (*nitidibaccatum*); 17296, 18185, 18514, 19082, 19248, 19911, 20562, 20631 (*americanum*).

Ahles, H.E. 19310 (*emulans*); 30705 (*pseudogracile*); 52986, 55038 (*sarrachoides*); 64847 (*emulans*); 72523 (*triflorum*); 77911, 81628, 85747, 86317 (*emulans*).

Ahmad, H. NHI-9409 (*nigrum*).

Ahti, T. 16434, 16467, 16511, 16512 (*villosum*); 16513, 16514 (*scabrum*); 16515 (*villosum*); 16786 (*scabrum*); 16843 (*villosum*); 64094, 71572, 71678, 75149 (*nigrum*).

Ahumada, O. 350 (*chenopodioides*); 1085 (*americanum*).

Aitchison, J.E.T. 314 (*nigrum*); 611 (*villosum*); 727 (*nigrum*).

Aker, C. 422 (*nigrescens*).

Akeroyd, J.R. 239, 3583 (*villosum*).

Akhani, H. 11571 (*nigrum*); 12246 (*villosum*); 12247, 12252 (*nigrum*).

Akpabla, G. 2117 (*scabrum*).

Al Dabbagh NHI-45862 (*villosum*).

Al Khaisi NHI-42715 (*villosum*); NHI-45296 (*nigrum*); NHI-46088, NHI-50505 (*villosum*); NHI-50616 (*nigrum*); NHI-51173 (*villosum*).

Al Khayat NHI-46318 (*villosum*).

al Rawi, A. NHI-5951, NHI-32404 (*nigrum*).

al Zebar, A.M. 9272 (*villosum*).

Alanko, P. 113841, 120700, 135920, 137354 (*villosum*).

Alba, A. 19 (*nigrescens*).

Albee, B. 3177 (*triflorum*).

Albers, C.C. 62349 (*villosum*).

Albert, R. 286, 721, 973 (*villosum*).

Albert, V. 96/10, 96/12 (*triflorum*).

Albrecht, D.E. 384, 483 (*furcatum*); 738 (*americanum*); 2499 (*nitidibaccatum*).

Albuquerque, J.M. de INPA143090, INPA143091 (*americanum*).

Alcocer R, M. 141 (*nigrescens*).

Alcock, C.R. 1268, 2099 (*retroflexum*); 3939 (*triflorum*); 4454 (*americanum*); 6808 (*triflorum*); 8588, 8594, 8665, 8741 (*americanum*); 11053 (*nitidibaccatum*); 11266, 11267, 11273 (*americanum*); 11425 (*nigrum*).

Alcorn, J.B. 2220 (*nigrescens*); 2347 (*americanum*).

Aldridge, A.E. 962 (*nigrum*).

Alduvín, C. 129, 189 (*nigrescens*).

Alemán S, T. 92 (*douglasii*).

Alex, J.F. 725 (*emulans*).

Alexande, E. 41, 43, 52 (*americanum*).

Alexander, A.M. 4503 (*triflorum*).

Alexander, J.A. 1045 (*triflorum*).

Alexander, J.C.M. 126 (*villosum*).

Alexiades, M. 850052 (*americanum*).

Alfaro V, E. 2748 (*nigrescens*).

Alfaro, E. 74 (*nigrescens*); 425, 951 (*macrotonum*); 1240 (*nigrescens*); 1657, 2232 (*macrotonum*); 2748, 3067 (*nigrescens*); 3517 (*americanum*); 3987, 4217 (*nigrescens*).

Allard, H.A. 208, 950, 2269, 20780, 20980 (*emulans*); 22042, 22459 (*americanum*).

Allart, A. 313 (*macrotonum*).

Allen, B.A. 46 (*emulans*).

Allen, B.M. 6505 (*villosum*).

Allen, C.M. 75, 11535, 21032, 22347 (*nigrescens*).

Allen, E.B. 35 (*triflorum*).

Allen, G.G. 101 (*triflorum*).

Allen, P.H. 662 (*macrotonum*); 876 (*americanum*).

Allen, R.A. 285 (*triflorum*).

Allorge, L. 2632 (*americanum*).

Almeda, F. 2386 (*nigrescens*).

Almeida de Jesus, J. 1495 (*americanum*).

Almeida, J. 451, 1969 (*americanum*).

Almeida, S.S. de 665 (*americanum*).

Alonso G, P. 19 (*americanum*).

Alston, A.H.G. 11212 (*villosum*); 1289, 11214, 11218, 11268, 17405 (*nigrum*).

Altamirano, F. 1593 (*nigrescens*).

Alvarado, J. 106 (*nigrescens*).

Álvarez M, D. 303 (*americanum*).

Álvarez, D. 4038 (*americanum*); 7800, 8210, 8340, 8475, 8594 (*nigrescens*); 8726 (*americanum*); 9380 (*nigrescens*); 9939 (*douglasii*); 10323, 10806 (*nigrescens*); 11053 (*americanum*); 11564 (*nigrescens*).

Álvarez, R. 343 (*americanum*).

Alvear-P, M. 615 (*nigrescens*).

Alverson, W. 2640 (*nigrescens*).

Alverson, W.S. 2640, 2989 (*nigrescens*); 3331 (*macrotonum*); 3856 (*americanum*).

Alves, E.M. 493 (*americanum*).

Alves, M. EU-26 (*villosum*).

Alzate, H. 22 (*nigrescens*).

Am Sharif NHI-47305 (*nigrum*).

Amaral, A. 418 (*americanum*).

Amaral, I.L. 808 (*americanum*).

Ambrish, K. NC-120913 (*americanum*).

Ambrosetti, J.A. 1474 (*triflorum*); 1478 (*nitidibaccatum*).

American Colony Jerusalem 102 (*villosum*).

Amith, J.D. JDA-2084 (*pruinosum*); 21364 (*nigrescens*); JDA-30248 (*pruinosum*); 30504, 30588 (*nigrescens*); 30687 (*douglasii*).

Amorim, A.M. 21 (*scabrum*); 8020 (*americanum*).

Amparo M, L. 83 (*nigrescens*).

Ana Virginia, L. 15 (*macrotonum*).

Ancuash Atsut, E. 447 (*americanum*).

Anders 1587 (*nigrum*).

Anders, O. 3705, 4826, 5138, 5351, 10364, 10426, 10586, 10801 (*villosum*).

Andersen, J.S. 247 (*nigrum*).

Anderson, [?] 157 (*villosum*).

Anderson, B. 1920, 2832 (*americanum*).

Anderson, D. 128b (*americanum*).

Anderson, D.L. 2464, 3265 (*nitidibaccatum*); 3271 (*triflorum*); 3300 (*sarrachoides*); 3427 (*nitidibaccatum*); 3428 (*triflorum*); 3594 (*nitidibaccatum*); 3609 (*triflorum*).

Anderson, E.A. 3773 (*nigrum*).

Anderson, E.F. 3836 (*nigrum*).

Anderson, F.W. 5981 (*triflorum*).

Anderson, J. 10, 140 (*furcatum*).

Anderson, J.P. 604 (*nitidibaccatum*); 605 (*nigrum*); 7558 (*nitidibaccatum*).

Anderson, J.R. 254 (*nitidibaccatum*).

Anderson, L.C. 17746 (*americanum*).

Anderson, R.C. 3 (*nigrescens*).

Anderson, T. 1018, 1027 (*nigrum*).

Anderson, W.R. 9935 (*americanum*); 12770 (*nigrescens*); 36930 (*chenopodioides*).

Andrade, B.O. 147, 302 (*americanum*).

André, E. 571 (*macrotonum*); 638 (*nigrescens*); K640, 715, 1588 (*macrotonum*).

Andreasen, M. 541 (*nigrescens*).

Andreasen, M.L. 623 (*americanum*).

Andreata, R. 180 (*americanum*).

Andrews, C. 658 (*nigrum*).

Andrews, F.W. 3590 (*villosum*).

Andrieux, G. 189[b] (*douglasii*).

Anect Brother 16 (*nitidibaccatum*).

Ángeles, E. 6 (*nigrescens*).

Angier, B.S. 93 (*douglasii*).

Angus, A. 1528 (*scabrum*).

Annadale, N. EB-323 (*nigrum*).

Annels, A.R. 4680 (*americanum*).

Antío, J.A. 128 (*americanum*).

Antonie, P. 15 (*emulans*).

Antonio, T.M. 651 (*macrotonum*); 785, 852, 2209 (*nigrescens*); 2651 (*macrotonum*); 4530 (*nigrescens*).

Aparecida da Silva, M. 2753 (*americanum*).

Aparecida, M. 2753 (*americanum*).

Apolo, D. 62493 (*nitidibaccatum*).

Aponte, H. 256 (*americanum*).

Appel, H. 719 (*emulans*).

Arakaki, M. 70 (*corymbosum*); 85 (*americanum*); 184 (*corymbosum*).

Araque M, J. 882 (*sarrachoides*).

Araquistain, M. 174 (*americanum*); 642, 686, 1030, 1110, 1202, 2080 (*nigrescens*); 2716 (*americanum*).

Araújo, F. 333AE (*americanum*).

Araújo-M, A. 1977, 3107 (*americanum*).

Arbo, M.M. 1019, 7920 (*americanum*).

Arceneaux, G. 170a (*nigrescens*).

Archbold, M.E. 937 (*americanum*); 2545 (*villosum*).

Archer, W.A. 44 (*americanum*); 1284 (*nigrescens*); 4197 (*americanum*); 6836 (*triflorum*); 6837, 7134, 7289 (*nitidibaccatum*).

Archibald, E. 5905 (*chenopodioides*).

Archibald, J. 2661, 2778 (*villosum*).

Archley, J.C. 161 (*villosum*).

Arellano, M. 12 (*corymbosum*).

Arenas, J.M. 494 (*nigrescens*).

Argent, G.C.G. C87-179 (*scabrum*); 1305, 6362 (*americanum*); PPI-9752 (*nigrum*); 92248 (*americanum*).

Argüelles, E. 22 (*nigrescens*); 667, 996 (*americanum*); 1211 (*corymbosum*); 2295 (*douglasii*); 2520 (*nigrescens*); 2876, 3441 (*americanum*).

Argüelles, J. 122 (*americanum*); 152 (*douglasii*).

Ariste-Joseph [Frère] B11 (*nigrescens*).

Ariza E, L. 3621 (*chenopodioides*).

Armstrong, J.A. 98 (*nigrum*).

Arnason, J.T. 17626 (*americanum*).

Arnason, T. 17542 (*nigrescens*).

Arnay, E. d' 272 (*triflorum*).

Arnett, M. 3474, 7495, 7544 (*triflorum*).

Arnold, H. 10206 (*retroflexum*).

Arnoldo, M. 1719 (*americanum*).

Arnow, L. 544a (*triflorum*); 740 (*nitidibaccatum*); 3189 (*triflorum*); 3740 (*nitidibaccatum*); 4692 (*triflorum*); 5552 (*nitidibaccatum*); 6779, 7175 (*triflorum*).

Aroha, C.M. NC-38461, NC-53202 (*villosum*).

Arora, C.M. 1437, 6073 (*villosum*).

Arsène, G. [Frère] 10, 71, 126 (*nigrescens*); 262 (*corymbosum*); 434 (*nigrescens*); 594 (*emulans*); 1031, 1190 (*nigrescens*); 1483, 1484 (*douglasii*); 2166 (*corymbosum*); 2235, 2238 (*nigrescens*); 2892 (*douglasii*); 3334 (*americanum*); 6149 (*nigrescens*); 8302 (*douglasii*); 10127, 10339 (*nigrescens*); 17348 (*triflorum*); 21830 (*interius*).

Arsène, L. 517/3 (*villosum*).

Artavia, M. 7261 (*americanum*).

Articó, L. 240, 249 (*triflorum*).

Arvidsson, I. 191 (*villosum*).

Arvigo, R. 730 (*nigrescens*).

Ascencio V, M.A. 111 (*douglasii*).

Ashe, C. 75 (*nigrescens*).

Asplund, E. 1372 (*nigrum*); 5283 (*americanum*); 6552 (*macrotonum*); 8941, 15390, 16600 (*americanum*); 16911 (*macrotonum*); 18053 (*americanum*); 18633 (*macrotonum*); 19851 (*americanum*); 20534 (*macrotonum*).

Asplund, R.O. 72-29 (*triflorum*).

Assadi, M. 1690, 1976 (*villosum*).

Aston, H.I. 2013 (*americanum*).

Astridge, S. AEE-4106 (*americanum*).

Aswal, B.S. NC-55377 (*villosum*).

Atchison, G. 1 (*nitidibaccatum*); 2 (*chenopodioides*); 3, 4, 5, 6, 7, IM-8, IM-9 (*nigrum*).

Atha, D.E. 5196 (*villosum*); 5835 (*nigrum*); 6419, 6514 (*emulans*); 6869, 7932, 14060 (*nigrum*); 14687 (*emulans*).

Atherton, G.E. 301, 387, 669, 861 (*villosum*).

Athey, R. 2515 (*emulans*).

Atkinson, C.L.W. 35 (*americanum*).

Atwood, D. 16093 (*triflorum*).

Atwood, J.T. 5367 (*nigrescens*).

Atwood, N.D. 5893 (*triflorum*); 10672 (*nitidibaccatum*); 13495, 16093 (*triflorum*); 17037 (*douglasii*); 26560 (*americanum*); 28806 (*triflorum*); 29082 (*americanum*); 30058 (*triflorum*).

Aubert, G. d' 205 (*chenopodioides*).

Aucher-Eloy, P.M.R. 2482 (*villosum*); 5030 (*nigrum*); 5031 (*villosum*).

Auld, B. 120043 (*chenopodioides*).

Aussem, D. 265 (*emulans*).

Avendaño Reyes, S. 126 (*americanum*); 416, 3118 (*nigrescens*).

Averett, J.E. 408, 435 (*douglasii*); 1253 (*americanum*).

Avila, A. de 52 (*nigrescens*).

Avila, R. 3517 (*nigrescens*).

Avilán, L. 19 (*nigrescens*).

Ayala, M.G. 221 (*nigrescens*); 449 (*americanum*); 652 (*nigrescens*).

Ayala-Hernández, [?] 576 (*nigrescens*).

Ayanzi, F.B. Edin.5887 (*nigrum*).

Aye, T.T. 20623 (*americanum*).

Ayer, K.A. 639 (*emulans*).

Aylmer, G. 171, 517 (*villosum*).

Aymard, G. 4942 (*nigrescens*).

Azimi bin Haji Hamzah 4, 5 (*nigrescens*).

Baber, [?] 3377 (*nigrescens*).

Babu, C.R. NC-34679a (*nigrum*); NC-34679 (*americanum*).

Bachem C, U. 184 (*douglasii*).

Bacigalupi, R. 1003 (*emulans*); 4734 (*furcatum*); 8779 (*americanum*).

Bacon, E. 93 (*nigrum*).

Baez, J.R. 31 (*chenopodioides*).

Baez, S. 32B (*macrotonum*).

Bailey, H. 2060, 3516, 3772 (*americanum*).

Bailey, L.H. 564 (*nigrescens*).

Bailtcher, F.L.J. 495 (*emulans*).

Baird, G.I. 1498, 1720, 1820 (*triflorum*); 2494 (*nitidibaccatum*).

Bajhang 2009 Expedition 20913010 (*nigrum*).

Bakari, D.N.G. 144 (*americanum*).

Baker, C.F. 146 (*nigrescens*); 231 (*americanum*); 577, 622 (*triflorum*); 677 (*americanum*); 1426 (*nitidibaccatum*).

Baker, J.G. 50 (*americanum*).

Baker, M. 981, 1010, 1019 (*furcatum*).

Baker, M.L. 1707 (*triflorum*); 2203 (*nitidibaccatum*).

Baker, W.H. 8226, 8381, 9147, 11121 (*triflorum*).

Bakhuizen van den Brink, R.C. 6552 (*nigrum*).

Bakia, A. 603 (*americanum*).

Balaka, J.L. 2025 (*scabrum*).

Balakrishnan, N. 996 (*americanum*).

Balakrishnan, N.P. 1177 (*villosum*); 1177[b] (*nigrum*).

Balapure, K.M. 537 (*nigrum*).

Balkwill, K. 6291 (*retroflexum*); 7163, 9130 (*chenopodioides*).

Ball, C.R. 325 (*nigrescens*); 1256 (*interius*).

Ballantyne, G.H. 418 (*nigrum*).

Ballard, C. 2828, 3160 (*triflorum*).

Balls, E.K. B4381 (*nigrescens*); B4671 (*douglasii*); B4747, B4863, B5067 (*nigrescens*); B5084 (*corymbosum*); B5645, B5646 (*nigrescens*); B7516, B7528 (*macrotonum*); 8185, 11839, 23678 (*douglasii*).

Bally, P.R.O. B1022, B11056 (*villosum*); 12482 (*nigrum*).

Balodi, P. NC-75653 (*americanum*).

Balogh, P. 999, 1025 (*douglasii*).

Balsinhas, A. 647 (*americanum*); 2901 (*retroflexum*); 3290 (*scabrum*).

Balslev, H. 2070 (*macrotonum*).

Baltars, E. 669 (*emulans*).

Balvanera L, P. 204, 259 (*americanum*).

Banerjee, D. 30, 33 (*villosum*).

Banerjee, R.N. 16180 (*americanum*).

Banerju, R.N. CNH-16284, CNH-17468 (*nigrum*).

Bang, M. 539, 1462 (*americanum*).

Bangerter, E.B. 5024 (*nigrum*); 5165 (*americanum*); 5508 (*nigrum*).

Banker, H.J. 1490 (*emulans*).

Banks, D.L. 1714 (*douglasii*).

Banlugan, G. 72719 (*nigrum*).

Banze, A.C. 277 (*americanum*).

Barabas, N. 585 (*nigrum*).

Barbe, G.D. 4313 (*americanum*).

Barber, J.H. 65 (*douglasii*).

Barber, M.A. 158 (*nitidibaccatum*).

Barbon, E.B. PPI1565, PPI-1832 (*americanum*); PPI-9142 (*nigrum*).

Barboza, G.E. 69, 73 (*triflorum*); 190, 197 (*nitidibaccatum*); 566 (*chenopodioides*); 1574, 1575 (*macrotonum*); 1579, 2091 (*americanum*); 2128, 2292, 2316, 2317 (*chenopodioides*); 2345 (*triflorum*); 2399 (*furcatum*); 2429 (*triflorum*); 3058 (*americanum*); 3682, 3687 (*triflorum*); 4211 (*nitidibaccatum*).

Bârca, C. 267 (*nigrum*).

Barclay, A.S. 3235 (*macrotonum*).

Barclay, C. 2342 (*nigrum*).

Barclay, G.W. 1605, 1607 (*americanum*).

Bare, J. 79 (*interius*).

Bark, P. 109 (*americanum*).

Barker, W.R. 51 (*nigrum*).

Barkley W, F.A. 1623, 1775 (*nigrescens*).

Barkley, F.A. 13A162, 618, 44M805, 1775 (*nigrescens*); 3709 (*villosum*); 3716 (*nigrum*); 33Ir4015, 9042 (*villosum*); 14585C (*nigrescens*).

Barkley, L.J. 29 (*nigrescens*).

Barkly, F.O. 13A162 (*nigrum*).

Barnard, C. 12 (*chenopodioides*).

Barnes, F.T. 955 (*nigrum*).

Barnes, W. 306 (*emulans*).

Barnhart, J.H. 229 (*emulans*); 2062 (*pseudogracile*).

Baron, R. 860, 1744, 1744 (*scabrum*).

Barragán-Fonseca, K. 9 (*macrotonum*).

Barrell, J. 561-62 (*triflorum*).

Barrientos, R. 4013 (*nigrescens*).

Barringer, K. 630 (*emulans*); 1831 (*nigrescens*); 3262 (*macrotonum*); 10399 (*nigrum*).

Barringer, K.A. 3108 (*nigrescens*); 3215 (*americanum*); 3236 (*nigrescens*).

Barros, A.A.M. de 2267, 3504, 5160 (*americanum*).

Barroso, R.M. 131, 135 (*americanum*).

Barrus, B. 16 (*nitidibaccatum*); 308 (*nigrescens*).

Barry, R. 1 (*chenopodioides*); 9 (*americanum*).

Barta, T. 2006-20 (*nigrum*); 2005-34, 53, 121, 2004-138, 175 (*villosum*); 2003-372 (*nigrum*); 1151, 1153 (*villosum*); 1219 (*nitidibaccatum*); 2181, 2205 (*villosum*); 2223, 2606, 3064, 3139 (*nitidibaccatum*); 3155 (*villosum*); 3198, 3270, 4122 (*nitidibaccatum*).

Barter, C. 1054 (*scabrum*).

Barthelat, F. 210, 885 (*scabrum*).

Bartholomew, B. 1521 (*douglasii*); 6200 (*nigrum*).

Bartholomew, E.A. 859 (*emulans*).

Bartlett, G.H. 316 (*emulans*).

Bartlett, H.H. 479 (*americanum*); 8569, 14825 (*nigrum*).

Barunjee, R.N. 18760 (*americanum*).

Basak, R.K. 101, 109 (*villosum*).

Bassam, M.J. 8202 (*americanum*).

Bassignan, F. 1904 (*triflorum*).

Bastos, B.C. 134 (*americanum*).

Basu, P. BSHC-8481 (*nigrum*).

Batanouy, K.H. 2281 (*villosum*).

Batchelder, C.F. 1691 (*emulans*); 5001 (*pseudogracile*).

Bates, J.M. 5023 (*emulans*); 5200 (*interius*).

Bates, R.J. 953 (*triflorum*); 16832 (*americanum*); 17501 (*nitidibaccatum*); 41122 (*chenopodioides*); 51012, 64001 (*triflorum*).

Bateson, G. 121 (*nigrum*).

Batianoff, G.N. 92-0727, 98-1061 (*americanum*); 11110, 2001-11125, 2001-11141, 2001-11342, 2001-11356, 2001-12169 (*chenopodioides*); 205108, 210495, 210654, 210686, 410011, 630272, 2010485, 2112477 (*americanum*).

Battiscombe, E. K453 (*villosum*).

Batty, M. 274 (*villosum*); 1100 (*americanum*).

Bauen, I. 37 (*americanum*).

Bauer, K. 1108 (*nigrum*).

Baur, R. 102 (*retroflexum*).

Baxter, P. 4142 (*villosum*).

Baxter, P.R. 24 (*furcatum*).

Bay, R.F. MS28-6 (*interius*).

Baylis, G.T.S. 556c (*douglasii*); 10134 (*nigrum*).

Bayliss, R.D.A. BRI-B-761, 2994, 3171 (*retroflexum*); 4594 (*scabrum*); 5292 (*chenopodioides*); 5476 (*scabrum*); 6785 (*chenopodioides*); 7182, 8679 (*retroflexum*).

Bayly, I. 94 (*americanum*).

Bayma, I.A. 285 (*americanum*).

Baytop, A. 13628 (*villosum*).

Bazuin, C.W. 6084 (*emulans*).

Beach, K.H. 46 (*nigrum*); 969 (*triflorum*); 5410 (*villosum*).

Beaman, J.H. 6320 (*nigrescens*); 6908 (*americanum*); 8216 (*nigrum*); 9048, 9079, 10798 (*americanum*).

Bean, A.R. 18613 (*chenopodioides*); 20752 (*americanum*); 29340, 31405, 32579, 32684 (*chenopodioides*).

Bean, P.A. 1366 (*retroflexum*).

Beard, P. 1271, 1383 (*nigrescens*).

Beatley, J.C. 11729, 13209 (*triflorum*).

Beauglehole, A.C. 4332, 12438, 21847 (*americanum*); 33117, 33186, 33238, 35771, 36566 (*triflorum*); 37084, 37084, 37804, 41744, 41744, 41769, 41769 (*nitidibaccatum*); 50427, 58411, 62800 (*americanum*); 67535, 67651A, 67673, 68030 (*triflorum*); 68690 (*nitidibaccatum*); 70231, 70633, 71312, 71472, 71844, 79123 (*americanum*); 79124 (*nitidibaccatum*); 79189, 79190, 79646, 83205 (*americanum*).

Bebb, R. 1172 (*nigrescens*).

Bechtel, A.R. 11886 (*emulans*); 17733 (*pseudogracile*).

Beck, J.B. 104 (*emulans*).

Beck, L.T. 510 (*nigrescens*).

Becker, J. 2352 (*pseudogracile*).

Beckley, V.A. 670 (*villosum*).

Beckner, J. 1646 (*pseudogracile*); 2256 (*americanum*).

Bedi, R. 667 (*villosum*).

Beever, R.E. 80-095 (*nigrum*).

Béguin, D. 108 (*scabrum*).

Béguinot, A. 2515, 2516, 2518 (*nigrum*); 2520, 2521, 23618 (*villosum*); 23622, 23623 (*nigrum*); 23624, 23625, 23626 (*villosum*).

Belibasis, L. de 58 (*americanum*).

Belknap, F. 2 (*triflorum*).

Bell, A.S. Mrs 36 (*villosum*).

Bell, C.R. 3788, 4782, 17061 (*pseudogracile*).

Bell, W.M. 50 (*interius*).

Bellingham, P.J. 0142 (*nigrum*); 637 (*chenopodioides*); 1199 (*nigrescens*).

Bellini, A. 464 (*villosum*).

Bello, E. 12, 249 (*nigrescens*); 2986 (*americanum*); 3361, 3438, 4437 (*nigrescens*); 4945 (*americanum*).

Bello, M.A. 498, 613 (*nigrescens*).

Belshaw, C.M. 165 (*nigrum*); 205 (*americanum*); 1447 (*nigrum*); 1488 (*douglasii*); 1489 (*americanum*); 1714 (*douglasii*).

Beltrán, G. 176 (*macrotonum*); 189, 195 (*americanum*).

Beltrán, G.D. 21 (*americanum*); 65 (*nigrescens*); 71, 72 (*macrotonum*); 84, 94 (*nigrescens*).

Benedetto, P. 49 (*villosum*).

Benedictis, A. de 389 (*villosum*).

Benítez de Rojas, C.E. 2[a], 104, 232, 309, 409, 430, 453, 558, 700, 721, 1561 (*nigrescens*); 2506 (*macrotonum*); 2513 (*nigrescens*); 2981 (*americanum*); 4190 (*nigrescens*); 4441 (*macrotonum*).

Benítez, A. 1979 (*douglasii*).

Bennet, A. 874b (*villosum*).

Bennet, S.S.R. 75, 267 (*villosum*).

Bennett, C. 8 (*nigrum*).

Bennett, C.A. 811 (*douglasii*).

Bennett, H.R. 7749 (*triflorum*).

Bennett, J.P. 35 (*emulans*).

Bennett, M. 1088 (*americanum*).

Benninghoff, W.S. 10880 (*emulans*).

Benoist, R. 62 (*americanum*); 147 (*scabrum*).

Benol, D.M. 3 (*americanum*).

Bensman, R. 177 (*americanum*).

Benson, J.S. 2791 (*chenopodioides*).

Bentley, P.S. 55, 169, 306 (*americanum*).

Bento, M.S. 54 (*americanum*).

Benusan, J. 51 (*villosum*).

Berendsohn, W. 1040 (*nigrescens*).

Berg, C.C. P-19836 (*americanum*).

Bergling, F. 2401 (*emulans*).

Bergman, C. 711, 1037, 1180 (*emulans*).

Bergman, H.F. 1355 (*interius*); 2057, 2568 (*triflorum*).

Berlandier, J.L. 46 (*americanum*); 118, 503 (*nigrescens*); 504 (*americanum*); 524 (*nigrescens*); 586 (*americanum*); 751 (*pruinosum*); 892, 1904, 2322 (*nigrescens*).

Berlin, B. 1682, 1965, 3671 (*americanum*).

Bernacci, L.C. 25856, 25857 (*americanum*).

Bernal G, C.A. 300, 525, 551 (*macrotonum*).

Bernal, R. 1718 (*nigrescens*); 1732 (*macrotonum*); 2670 (*americanum*).

Bernard, G. 66661, 73111, 77068 (*emulans*).

Bernard, J.-.P. [Frère] B96-302, 54-506 (*emulans*).

Bernardi, L. 6112 (*macrotonum*).

Bernoulli, K.G. 2332 (*nigrescens*); 2346, 2379, 2386, 2406 (*americanum*).

Berro, M.B. 1458, 7206 (*sarrachoides*).

Bertaud Brother 68 (*triflorum*).

Bertero, C.L.G. 633 (*furcatum*); 636, 637 (*nitidibaccatum*); 638, 1324 (*furcatum*).

Bertin, R.I. 4226 (*nitidibaccatum*); 6800 (*emulans*).

Bertoncini, A.P. 1091 (*americanum*).

Bertrand, T. CZ-615 (*emulans*).

Best, E.B. 499 (*retroflexum*).

Betancourt A, C. 28 (*americanum*).

Betancur, J.C. 1405 (*americanum*); 5768 (*nigrescens*); 9533 (*macrotonum*).

Betche, E. 59 (*americanum*).

Beusekom, C.F. van 1030, 1030 (*americanum*).

Beuther, A. 58 (*nigrescens*).

Beyersdorfer, M. 181 (*emulans*).

Bhargava, N. ANC-4371 (*americanum*).

Bhattacharyya, U.C. NC-12853, NC-12986, NC-13026 (*villosum*); NC-13891 (*americanum*); NC-17976 (*villosum*); NC-18435 (*americanum*); NC-21043 (*nigrum*); NC-37783 (*americanum*); 37783 (*villosum*); NC-40707 (*americanum*); NC-44895 (*villosum*); NC-45161 (*nigrum*); NC-45932 (*villosum*).

Bhowmik, P. 30 (*emulans*).

Bianor 957, 958 (*villosum*).

Bicknell, E.P. 7710, 7711, 7715, 7717, 7718 (*emulans*); 7719, 7720 (*nigrum*).

Biegel, H.M. 1413 (*retroflexum*); 1767 (*scabrum*).

Biel, B. IM-16054 (*chenopodioides*).

Biganzoli, F. 130 (*americanum*).

Biggers, D.D. 13348 (*triflorum*).

Billiet, F. 5526 (*furcatum*).

Billot, C. 22 (*nigrum*); 25 (*villosum*).

Biltmore Herbarium 912b (*emulans*); 912d (*pseudogracile*); 912a, 912g (*nigrescens*); 912c, 912d2 (*americanum*).

Bingham, M.G. 1544 (*scabrum*).

Binot, A. 34 (*americanum*).

Bird, W. 85, 2395 (*nigrum*).

Bis Ram 347 (*nigrum*).

Bisby, R. 4036 (*chenopodioides*).

Bishop, W. 178 (*chenopodioides*).

Bissell, C.H. 1, 883, 5153, 5154, 8911 (*emulans*).

Bisset, J. 272, 733, 734, 3559 (*nigrum*).

Biswas, K. 3049 (*villosum*); 6754 (*americanum*).

Biswas, M.C. 244 (*americanum*).

Biurrun, F. 1711, 2160, 3211 (*nitidibaccatum*); 4261 (*triflorum*); 4281, 6086, 6155 (*nitidibaccatum*).

Blain, W.L. 171, 173 (*emulans*).

Blake, A.L. 175 (*triflorum*); 186 (*nitidibaccatum*).

Blake, M.E. 116 (*nitidibaccatum*).

Blake, S.T. 19542, 21507 (*americanum*).

Blakely, O.W. 1508 (*emulans*).

Blakley, E.R. 65, 589, 3124, 3894, 4186, 5063 (*douglasii*).

Blakley, O.W. 1508 (*emulans*).

Blanchet, J.S. 183, 869 (*americanum*).

Blanco, M. 279 (*nigrescens*).

Blanco, N. 26 (*nigrescens*).

Blankinship, J.W. 408 (*triflorum*).

Blatter, E. 383 (*americanum*).

Blaylock, B.J. 2357 (*chenopodioides*).

Blewitt, A.E. 2799 (*emulans*).

Blodgett, C.O. 415 (*douglasii*).

Bloembergen, S. 3995 (*americanum*).

Blom, C.M. 36 (*villosum*); 1373 (*nigrum*); 1376 (*sarrachoides*).

Blom, M. van 176 (*villosum*).

Blomquist, F. 8 (*triflorum*).

Blum, K.E. 477 (*americanum*).

Blumer, J.C. 16, 34 (*interius*); 1512 (*douglasii*); 1656 (*triflorum*); 1768 (*nitidibaccatum*); 1797, 2263, 3426 (*douglasii*).

Bobbette, R.S.W. 1973, 1989, 2089 (*emulans*).

Böcher, T.W. 200 (*americanum*).

Bochmeier, J. 452, 978 (*triflorum*).

Bocquet, G. 15520, 16910 (*nigrum*).

Bodner, C.C. 150 (*nigrum*).

Boelcke, O. 56a (*chenopodioides*); 4201 (*triflorum*); 6464 (*furcatum*); 16088, 16250 (*triflorum*).

Boettekin, F.L.J. 495 (*emulans*).

Bohs, L. 2099 (*sarrachoides*); 2400 (*americanum*); 2534, 2698 (*nigrum*); 3062, 3068 (*triflorum*); 3072 (*nigrescens*); 3095 (*americanum*); 3109 (*corymbosum*); 3189a, 3309 (*americanum*); 3531, 3531 (*nigrum*); 3617 (*macrotonum*); 3652 (*americanum*); 3938 (*nigrescens*).

Boiteau, P.L. 371 (*americanum*).

Boivin, B. 9823, 12861 (*triflorum*); 14192 (*nigrum*).

Boldrini, I. 277 (*americanum*).

Bolt, B. 529 (*douglasii*).

Bolus, F. 55 (*retroflexum*).

Bolus, H. 50 (*retroflexum*).

Bonilha, C. 313 (*americanum*).

Bonilla Beas, R. 99 (*nigrescens*).

Bonilla R, C. 162 (*nigrescens*).

Boole, J.A. 1151 (*emulans*).

Boone, W. 199, 510 (*americanum*).

Boot, [?] 1323 (*emulans*).

Booth, E.S. 77 (*nigrum*).

Booth, L.M. 1075 (*douglasii*); 1213 (*americanum*).

Booth, R. 2100, 2162 (*americanum*).

Booth, W.E. 54547, 54602, 57445, 61226, 571041 (*triflorum*).

Borden, T.E. 2070 (*americanum*).

Bornmüller, A. 565 (*americanum*).

Bornmüller, J. 496, 636 (*nigrum*); 1798 (*villosum*).

Boron, B. NC-75048 (*nigrum*).

Boron, D. NC-76339 (*villosum*).

Bos, J.J. 3803 (*americanum*).

Bosser, J.M. 926, 8658, 14707 (*americanum*).

Bossert, D.C. 9 (*pseudogracile*).

Botany staff National Herb. Iraq NHI-43788, NHI-43803 (*villosum*).

Botany students 1458 (*villosum*).

Botany-23 256 (*nigrescens*).

Boto, M.M.P. 70 (*americanum*).

Botta, P.E. [30D] (*villosum*).

Botteri, M. 846 (*nigrescens*).

Bouchon, A. 6703 (*chenopodioides*).

Boufford, D.E. 9039, 18017 (*emulans*); 24134, 29001 (*nigrum*); 31001 (*villosum*); 37533 (*nigrum*).

Boughey, A.S. 109 (*scabrum*).

Boulos, L. 7563, 11195, 14102, 14223, 14225 (*villosum*); 15722 (*nigrum*); 15839, 16400 (*villosum*); 16730 (*nigrum*); 17155, 17234, 17419 (*villosum*); 19046 (*nigrum*); 19200, 19384, 19457 (*villosum*); 20191 (*nigrum*).

Bourgeau, E. 557, 934 (*villosum*); 2525 (*americanum*); 2555 (*nigrescens*).

Bourne, E.T. 359 (*americanum*); 405, 472, 3673 (*villosum*).

Bovey, M. 61 (*nigrum*).

Bovini, M.G. 1056, 1827, 2207, 2884, 3646 (*americanum*).

Bowden, L.F. 250, 367, 685, 871 (*nigrum*).

Bowen, H. 294 (*nigrum*).

Bowerman, M.L. 1029 (*americanum*).

Bowers, J.E. 968 (*douglasii*).

Bowers, T. 2192 (*nigrum*).

Bowman, E.M. 104 (*americanum*).

Box, H.E. 649, 1107 (*americanum*).

Boyd, S. 2534 (*triflorum*); 3665, 4259 (*douglasii*); 5892 (*nigrescens*); 7135, 11702 (*americanum*).

Boyle, B. 813 (*nigrescens*); 6284 (*macrotonum*).

Brace, L.J.K. 518, 1580 (*americanum*); 4346 (*nigrescens*).

Brack, P. 1658, 1789 (*americanum*).

Bradburn, A.S. 1430 (*nigrescens*).

Bradley, T. 3314 (*pseudogracile*); 27306 (*sarrachoides*).

Braga, J.M.A. 11, 293, 636, 3056 (*americanum*).

Bragg, K. 133 (*nigrum*).

Brain, C.K. 10574 (*retroflexum*).

Brainerd, R.E. 1004 (*triflorum*).

Brandbyge, J.S. 42414 (*nitidibaccatum*).

Brandegee, T.S. 298 (*triflorum*); 320 (*interius*); 5873 (*emulans*); 5873 (*interius*).

Brant, A. 196 (*emulans*); 1044 (*americanum*).

Brant, A.E. 4590 (*sarrachoides*).

Brasil, I. 105 (*americanum*).

Braun, E.L. 4641 (*emulans*).

Braunton, E. 1200 (*nitidibaccatum*); 1202 (*americanum*).

Bravo H, H. 236 (*nigrescens*).

Bravo-Bolaños, O. 235, 1019, 1333, 1380, 1446, 2147 (*douglasii*).

Breckwoldt, A. 1 (*chenopodioides*).

Breedlove, D.E. 6050 (*douglasii*); 6137, 7451, 9229, 10458, 10991, 11231 (*nigrescens*); 11819, 11911 (*douglasii*); 12341 (*nigrescens*); 12600 (*douglasii*); 13400, 13588, 14155, 14584 (*nigrescens*); 25885 (*douglasii*); 26339 (*nigrescens*); 27792 (*douglasii*); 38222 (*nigrescens*); 39222 (*americanum*); 40279, 40721, 41616, 43298 (*douglasii*); 47646 (*americanum*).

Breene, T.L. 66 (*nitidibaccatum*).

Breidy, J. LEB-31 (*nigrum*); LEB-34 (*villosum*).

Breitung, A.J. 5135 (*triflorum*).

Brenan, J.P.M. 1449, 12271 (*chenopodioides*).

Breteler, F.J. 11599 (*scabrum*).

Brett, J. 69, 350, 496, 746, 845 (*nigrescens*).

Bretting, P.K. M3, M4, 121 (*nigrescens*); 151 (*americanum*).

Brewer, W.H. 33 (*douglasii*).

Breyne, H. 2227 (*scabrum*).

Bridges, T. 400 (*nitidibaccatum*).

Brierley, E.M. 127, 142 (*retroflexum*).

Brigada Dorantes 2471, 2746, 3739 (*nigrescens*).

Brigada Dunas 1103 (*nigrescens*).

Brigada Vásquez 392 (*americanum*); 463 (*nigrescens*).

Briggs, B.G. 4046 (*triflorum*); 6964 (*chenopodioides*).

Brink, D.E. 1423 (*triflorum*).

Brink, V.C. 50-164 (*triflorum*).

Brinker, R. 374 (*pseudogracile*).

Brinkman, A.H. 140, 653, 5210 (*triflorum*).

Brinton-Lee, D. 1002 (*villosum*).

Briones V, O.L. 1989 (*nigrescens*).

Brisson, S. 81342 (*emulans*).

Bristan, N. 188 (*nigrescens*).

Bristow, S.C. 17 (*nigrum*).

Britez, R.M. 2046 (*americanum*).

Britton, N.L. 2181, 2210, 2416, 2446, 3088, 6332 (*nigrescens*).

Brizuela, A. 543 (*nitidibaccatum*); 1042 (*sarrachoides*); 1077 (*nitidibaccatum*); 1621 (*chenopodioides*).

Bro Anect 131 (*interius*).

Broadway, W.E. 258, 791 (*nigrescens*); 2817, 3126, 3620 (*americanum*); 6307 (*nigrescens*).

Broder, R.E. 313, 562 (*douglasii*).

Brodhurst-Hill, E. 328 (*villosum*).

Brodie, C.J. 192 (*nigrum*); 1171, 1183 (*chenopodioides*); 1247, 1346, 1653, 2448, 2520, 2546, 2601, 2669, 2859, 2888, 3040, 3111 (*nigrum*); 5435, 5435 (*chenopodioides*).

Brooke, W.M.A. 122 (*villosum*); 11376 (*chenopodioides*).

Brotherus, A.H. 767 (*nigrum*).

Brouillet, L. 2015-16 (*emulans*).

Brown, [?] 330 (*retroflexum*).

Brown, C.A. 1366 (*nigrescens*); 1986 (*pseudogracile*).

Brown, C.G. 2685 (*emulans*).

Brown, H.E. 39 (*nitidibaccatum*); 932 (*americanum*).

Brown, H.H. 1253, 4207 (*emulans*).

Brown, K.L. 158 (*americanum*).

Brown, R.C. 6019 (*scabrum*).

Brown, S. 198 (*americanum*).

Browne, E.M. T-40, 5963 (*emulans*).

Bruce, E.M. 524 (*scabrum*).

Bruegmann, M.M. 143 (*americanum*).

Bruggen, T. van 2134 (*emulans*).

Brumbach, W.C. 5712, 8720 (*americanum*).

Brummit, R.K. 19533 (*emulans*); 20476 (*chenopodioides*).

Brummitt, R.K. 298[a] (*nigrum*); 10323 (*retroflexum*); 21567 (*chenopodioides*).

Brunner, D.R. 859, 1272, 1298 (*americanum*); 1639 (*sarrachoides*).

Brunner, S. 108 (*scabrum*).

Brunt, M. 2056 (*americanum*); 2490 (*villosum*).

Bryant, E.G. 1002 (*scabrum*).

Bryant, P.T. 32 (*nigrescens*).

Bryson, C.T. 14391 (*pseudogracile*); 16966 (*americanum*); 17674 (*emulans*).

Buchanan, A.M. 13453 (*americanum*); 15695 (*triflorum*); 15827 (*nitidibaccatum*).

Buchanan, R.E. 470 (*americanum*).

Buchner, P. 83-14-8 (*villosum*).

Buchtien, O. 1443, 3247, 3890, 4027[b], 4384, 5548, 5550 (*americanum*).

Buddell, G.F. 1433 (*emulans*).

Buegge, J.J. 161, 632 (*douglasii*).

Buendía S, M.C. 23 (*americanum*).

Bueno, O. 356, 896, 976, 1685, 2726, 4284 (*americanum*).

Bufrem, A. 318 (*americanum*).

Búggenhout, G. van 10607 (*nigrum*).

Buller, R.H. REP-19191 (*villosum*).

Bullock, J.R. 323 (*nigrum*).

Bunting, G.S. 5572 (*americanum*); 11364 (*nigrescens*).

Burch, D. 677, 1033, 2336, 2379, 2401, 2535, 4307 (*americanum*); 4309 (*nigrescens*); 5991 (*douglasii*).

Burch, D.G. 388 (*nigrescens*); 1033 (*americanum*).

Burchell, W.J. 78 (*villosum*); 856 (*retroflexum*).

Burckhalter, R. 2149 (*nigrescens*).

Burg, W.J. van der 1177 (*scabrum*).

Burger, W.C. 374, 378 (*villosum*); 5717, 6305, 6328, 7492 (*macrotonum*); 7699, 7986 (*nigrescens*); 7992 (*americanum*); 8338, 9531, 11082, 11447 (*nigrescens*); 11508 (*macrotonum*); 11562 (*nigrescens*).

Burgers, [?] 1147 (*triflorum*).

Burgess, A.B. 585 (*nigrescens*).

Burglehaus, F. 3212 (*triflorum*).

Burk, M. 829 (*interius*).

Burkart, A. 6303 (*nitidibaccatum*); 7693 (*chenopodioides*); 7695 (*nitidibaccatum*); 8276, 8912 (*sarrachoides*); 12571, 19874 (*nitidibaccatum*).

Burkhill, H.M. 852, 2876, SF-13939 (*americanum*).

Burkill, I.H. 190 (*nigrum*); 31067 (*villosum*).

Burkill, J.H. 119 (*americanum*).

Burman, A.E. 33 (*nigrum*).

Burne, E. 86 (*villosum*).

Burnham, R. 117 (*nigrescens*).

Burnham, R.J. 117 (*nigrescens*).

Burnham, S.H. 642 (*emulans*).

Burns, T.E. 433, 611, 736 (*nigrum*).

Búrquez, A. 92-287 (*americanum*); 96-961 (*douglasii*).

Burton, D.L. 565, 565b (*pseudogracile*); 616, 617 (*emulans*); 623, 623a, 623b, 623d, 623e, 627, 630, 630b, 633, 633a, 633d (*pseudogracile*).

Burton, R. 144 (*emulans*).

Burton, R.M. AE88.133 (*nigrum*).

Burtt Davy, J. 7796 (*douglasii*); 17658 (*retroflexum*).

Busemeyer, D.T. 2030, 2030, 2414, 2520 (*emulans*).

Bush, B.F. 143 (*emulans*); 156 (*nigrescens*); 332, 390, 689 (*emulans*); 1213, 1326 (*nigrescens*); 1882 (*emulans*); 3007, 3882, 3962, 6007, 7096A, 7096B, 7142, 7662 (*triflorum*); 7673, 8098 (*emulans*); 11818, 12167, 70968* (*triflorum*).

Bussmann, R.W. 15965 (*emulans*).

Bustilla, S. 142 (*nigrescens*).

Butler, B.T. 4145 (*triflorum*); 7014 (*nitidibaccatum*).

Butler, G.D. 1009 (*nitidibaccatum*); 1010 (*americanum*).

Bye, R.A. 176 (*nigrescens*); 3583, 4867, 4867, 5678, 5678 (*douglasii*); 6067 (*americanum*); 6981 (*douglasii*); 7330, 7334, 7346 (*americanum*); 7558 (*nigrescens*); 9518, 15312, 15669, 15800 (*douglasii*); 16387 (*nigrescens*); 16922 (*chenopodioides*); 18175, 18188, 18421, 18436, 19414 (*americanum*); 26929 (*nitidibaccatum*); 26949 (*douglasii*); 28166 (*nigrescens*); 28196 (*douglasii*).

Bynum, [?] 91 (*nigrescens*).

Caballero, L.M. 101 (*macrotonum*).

Cabezas, F. 1095 (*scabrum*); 1191 (*americanum*).

Cabrera R, I. 3694 (*americanum*); 7893 (*nigrescens*).

Cabrera, A.L. 5024 (*furcatum*); 5267 (*chenopodioides*); 33163, 33217 (*triflorum*); 34181 (*nitidibaccatum*).

Cabrera, E. 129, 502, 875, 1151 (*nigrescens*); 2817, 2817 (*douglasii*); 3078, 3373, 6021, 7587, 7587, 7943, 8162, 8177, 11424 (*nigrescens*); 11424 (*americanum*); 14903, 14903, 15044, 15138, 15401, 15401, 15749 (*nigrescens*); 15867 (*americanum*); 15966 (*nigrescens*); 16124 (*americanum*).

Cabrera, P. 83 (*nigrescens*).

Cadena G, A.L. 153 (*nigrescens*).

Calatayud, G. 4350 (*americanum*).

Calder, C.C. 153 (*americanum*).

Calderón C, R. 95 (*nigrescens*).

Callejas, R. 3144, 7859, 11325 (*macrotonum*).

Calónico-Soto, J. 2527 (*douglasii*); 2528 (*pruinosum*); 11103 (*douglasii*); 21384, 22930, 22937, 22937, 24111, 24818, 25517, 27103 (*nigrescens*).

Calvert, J. KEW-84 (*triflorum*).

Calvo, J. 2457 (*nigrum*); 2501 (*triflorum*); 3846 (*villosum*).

Calzada, J.I. 416, 1607, 1848 (*nigrescens*); 4914 (*americanum*); 6543 (*nigrescens*); 7765, 9745 (*americanum*); 11951, 12687, 12705, 14221, 15461, 15461, 16748, 18759 (*nigrescens*); JIC-24498 (*americanum*).

Camacho, J.P. 20 (*nigrescens*).

Camargo, C. de 122 (*americanum*).

Camargo, O.R. 65, 2184, 3274 (*americanum*).

Cámbar, I. 126 (*nigrescens*).

Cambridge University Expedition H.91 (*nigrum*).

Cameron, D.G. 6123, 8123 (*americanum*).

Cameron, E.K. A-49a, A-118, 2110, 3398, 6217, 6767, 9896 (*americanum*); 10948a, 11642 (*nigrum*); 12506, 12543, 12995, 13237 (*americanum*); 13822 (*nigrum*); 14001, 14394 (*americanum*); 14430 (*nigrum*); 15205 (*americanum*); 15371, 15560, 15642, 15792 (*nigrum*); 16127, 16879, 26836 (*americanum*).

Camp, W.H. E2158 (*macrotonum*).

Campbell, D.G. P22040 (*americanum*).

Campbell, J. 1797-1-1572 (*emulans*).

Campos V, A. 58 (*nigrescens*).

Campos, A.L. 20 (*americanum*).

Campos, J. 2097, 2755, 6474, 6925 (*americanum*).

Canby, W.M. 210 (*nitidibaccatum*).

Canning, E.M. 4232, 4284, 5736, 6209 (*americanum*); 6421, 6549 (*triflorum*).

Cannon, J.F.M. 3154, 3349, 3526, 4428, 5038 (*nigrum*).

Cano, A. 2190, 2265, 4735, 5776, 8688 (*americanum*); 8862, 11345 (*corymbosum*); 12631 (*americanum*).

Cano, E. 365, 1724, 2832, 4019, 4019, 4060, 4061, 4708 (*triflorum*).

Cantino, P. 729, 746 (*nitidibaccatum*).

Canton Christian College 373 (*americanum*); 373 (*nigrum*); 3383 (*americanum*); 51926 (*nigrum*).

Cao Zi-yu 16 (*nigrum*).

Capparelli, P.N. 257 (*americanum*).

Carauta, J.P.P. 3227 (*americanum*).

Carauta, P. 310, 1236 (*americanum*).

Cárdenas, A.L. 900 (*nigrescens*).

Cárdenas, M. 2053, 4356 (*americanum*).

Cárdenas, V. 12275 (*corymbosum*).

Cardona Naranjo, F.A. 238 (*macrotonum*).

Cardoso, J. 134, 194 (*americanum*).

Cardoso, L.J.T. 79 (*americanum*).

Cardoso, V.L. 1233 (*nigrescens*); 1467 (*douglasii*).

Cardozo, A. 974 (*macrotonum*).

Carenzo, V. 3636 (*chenopodioides*).

Carleton, M.A. 382 (*triflorum*).

Carlson, M. 4134 (*nigrescens*).

Carlson, N.K. 200 (*douglasii*).

Carnahan, S. SC-892, 1224 (*douglasii*).

Carne, W.M. 53 (*americanum*).

Carolina, M. 17 (*americanum*).

Carolyn Wright 1775, 1779 (*triflorum*).

Carr, C.J. 534 (*villosum*).

Carr, G.D. 1600 (*triflorum*).

Carr, W.P. 162 (*triflorum*).

Carr, W.R. 12605 (*nigrescens*); 13594 (*americanum*); 15415, 21196, 21673, 21912, 22880, 24478 (*nigrescens*); 28625, 30427, 30565 (*emulans*); 30854 (*americanum*).

Carranza Batista, H. (1)3 (*americanum*).

Carranza, E. 3209 (*nigrescens*); 3281 (*douglasii*); 3880 (*nigrescens*); 5667 (*pruinosum*).

Carrasquilla, L. 3024 (*americanum*).

Carrick, J. 2169 (*nigrum*).

Carter, A. 2093, 2149 (*douglasii*).

Carter, A.M. 943 (*americanum*).

Carter, H.E. 569 (*villosum*).

Carter, J.E. 717 (*villosum*).

Carter, R. 5138 (*nigrescens*).

Carter, S. 381, 1815 (*villosum*).

Carvajal, A. 528 (*nigrescens*).

Carvalho, B. 16, 26 (*americanum*).

Carvalho, D.M.G. 5 (*americanum*).

Carvalho, L. ASE8047 (*americanum*).

Carvalho, M.F. do 3708, 4305 (*scabrum*); 5522 (*americanum*).

Carvalho-Silva, M. 1487, 1562 (*americanum*).

Carvalo, G.P. de 80 (*americanum*).

Cascante M, A. 117, 755 (*nigrescens*).

Cascante, A. 117, 755 (*macrotonum*).

Casey, E.C. 24, 1283 (*villosum*).

Casey, G.B. 442 (*americanum*).

Castañeda Robles, J. 228, 711, 718 (*douglasii*).

Castaner, D. 8646 (*emulans*).

Castaño A, N. 1660 (*americanum*).

Castellanos, A. 17860 (*chenopodioides*).

Castillo A, O. 63 (*nigrescens*).

Castillo C, G. 3, 188, 1373 (*nigrescens*).

Castillo Campos, G. 14751 (*nigrescens*).

Castillo, F. 271 (*nigrescens*).

Castillo, J.J. 1653 (*americanum*).

Castillo, R. 88-32 (*douglasii*).

Castillo-Hernández, L.A. 370 (*nigrescens*).

Castro, R. 17690 (*americanum*).

Castroviejo, S. 18239 (*villosum*).

Catalán H, C. 204, 234, 633 (*nigrescens*).

Catat, L.D.M. 1208 (*scabrum*).

Cavalcanti, A.C.S. 136, 163 (*americanum*).

Cavalcanti, F. JPB18718 (*americanum*).

Cavalerie, P.J. 3815 (*nigrum*); 7581 (*americanum*).

Cazzaniga, M.G. 3400 (*chenopodioides*).

Cedillo T, R. 2291 (*nigrescens*).

Cemé, J. 75 (*americanum*).

Cerón M, C.E. 15905, 18038 (*macrotonum*); 18909 (*americanum*).

Cerón, C.E. 15001 (*nigrescens*); 15513 (*americanum*); 15905 (*nigrescens*); 17617 (*macrotonum*); 20545, 29283 (*americanum*).

Cerrate, E. 259 (*corymbosum*); 5064 (*americanum*).

Cervantes G, C. 169 (*nigrescens*).

Chacón, I.A. 265 (*nigrescens*).

Chakraborty, P. BSHC-925, BSHC-1176, BSHC-1934 (*americanum*); BSHC-2159 (*nigrum*); ANC-4650 (*americanum*).

Chakravady, H.L. NHI-30765 (*nigrum*).

Chamberlain, E.B. 127, 420 (*emulans*).

Chambers, E. 25 (*emulans*).

Chamé, A. 1206 (*nigrescens*).

Chan, C. 177, 352 (*americanum*); 4463, 4883 (*nigrescens*); 4924 (*americanum*); 6138 (*nigrescens*).

Chandler, H.P. 429 (*emulans*); 814 (*americanum*).

Chandra, S. 1304 (*americanum*).

Chandrabose, M. 28410 (*americanum*).

Chaney, R.W. 191 (*emulans*).

Chang, Y. 613 (*emulans*).

Chapline, W.R. 301 (*interius*).

Chapman, J.D. 2914 (*scabrum*).

Chappell, C. 32 (*americanum*).

Charanpreet 9456 (*americanum*).

Charboneau, J.L.M. 782, 2048, 2698, 8339, 9265, 9450 (*triflorum*).

Chardou, C.Z. 87 (*americanum*).

Charest, R. 603 (*emulans*).

Charette, L.A. 1509 (*nigrum*); 1866 (*americanum*); 3073 (*emulans*).

Charlebois, J.E. 464 (*emulans*).

Charles 1932 (*emulans*).

Charpin, A. 158 (*nigrum*); 21516 (*sarrachoides*).

Charrabanty, P. 3338 (*villosum*).

Chase, N.C. 2857 (*scabrum*).

Chase, V.H. 10941, 14309 (*emulans*).

Chavarría, U. 2080 (*americanum*).

Chavelas P, J. ES-119, 389 (*nigrescens*).

Chávez A, E. 46 (*americanum*).

Chávez, A. 15 (*americanum*).

Chávez, E. 46 (*americanum*).

Chávez, M. 10, 94, 148 (*nigrescens*).

Chawdhury, H.I. NC-78102 (*villosum*).

Cházaro B, M. 6089 (*nigrescens*).

Chemin-Bassler, H. 90 (*americanum*).

Chen Chih-Hsiung 04625 (*americanum*).

Chen, L. 110 (*nigrum*).

Cheney, L. 4392 (*emulans*).

Cheo, T.Y. 60 (*nigrum*).

Chesterfield, E.A. 101 (*americanum*).

Chevalier, A. 5518 (*scabrum*).

Chevallier, L. 73bis (*villosum*).

Chiang, F. 408 (*americanum*); 8232A (*nigrescens*).

Chiao, C.Y. 18517 (*nigrum*).

Chiapella, J. 1806 (*nitidibaccatum*); 1809 (*triflorum*); 1840 (*nitidibaccatum*); 1888 (*triflorum*).

Chiar, P. 105 (*chenopodioides*).

Chiarini, F. 650, 795, 805, 820 (*nitidibaccatum*).

Chichester, B. 514 (*triflorum*).

Chien, S.S. 198, 395 (*nigrum*).

Chilton, L. 41, 122 (*nigrum*).

Chin, S.C. 3713 (*americanum*).

Chinchilla A, I.F. 90, 217 (*americanum*).

Ching-I Peng 4506 (*americanum*).

Chiovenda, E. 1204 (*villosum*).

Chipman, S.J. 432 (*triflorum*).

Chipunga, L. 168 (*retroflexum*).

Cholewa, A. 2597 (*nitidibaccatum*).

Chorley, M. 21 (*nigrum*).

Chow, K.S. 80-300 (*nigrum*); 79057 (*americanum*).

Christ, J.H. 692, 1741 (*triflorum*); 2564 (*nitidibaccatum*); 5456 (*triflorum*); 11730 (*nitidibaccatum*); 12949, 18326 (*triflorum*).

Christenhusz, M.J.M. 894, 895 (*nigrum*); 5349, 5385, 5636, 5649 (*nigrescens*); 5827 (*villosum*); 6230 (*furcatum*).

Christensen, D. 87 (*emulans*).

Christerpherson, E. 976, 2254 (*americanum*).

Christian, R.W. 1031 (*triflorum*).

Christian, T. 6, 31 (*furcatum*).

Christophersen, E. 2302, 2324 (*nigrum*); 2759 (*americanum*).

Christy, R.M. 100 (*triflorum*).

Chu, K.L. 3819 (*nigrum*).

Chuang, T.I. 3896, 4324 (*americanum*).

Chumley, T.W. 853, 4492 (*triflorum*).

Chun, N.K. 40185 (*americanum*).

Chung, H.H. 782 (*americanum*); 1647 (*nigrum*); 1729 (*americanum*); 4892 (*nigrum*); 5621 (*villosum*); 5974 (*nigrum*).

Churchill, J.R. 713 (*nigrum*); 713 (*emulans*); 713[c] (*pseudogracile*).

Churchill, S.P. 6847 (*emulans*).

Clar, C.R. 55 (*douglasii*).

Clark, J.A. 56 (*nitidibaccatum*).

Clarke, C.B. 1827 (*villosum*); 6653 (*americanum*); 9011 (*nigrum*); 22378 (*villosum*); 26311, 26835 (*americanum*); 27399, 36740 (*nigrum*).

Clarke, H. 21 (*emulans*).

Clarke, I.C. 1479, 1544 (*chenopodioides*); 1682, 1682, 1682, 1772, 1772 (*nitidibaccatum*); 2714, 2890 (*chenopodioides*); 2891 (*americanum*); 4181 (*chenopodioides*).

Clarke, S.L. 191 (*triflorum*).

Clarkson, J.R. 5498, 9357 (*americanum*).

Clarkson, Q.C. 277 (*triflorum*).

Claude-Joseph [Frère] 595 (*furcatum*).

Claussen, P. 180, 16846 (*americanum*).

Cleef, A.M. 3927 (*macrotonum*).

Cleland, J.B. 28 (*americanum*).

Clemens, J. 265 (*nigrescens*); 3020 (*nigrum*); 29744[a] (*americanum*); 29774[b] (*nigrum*).

Clemens, Mrs J. 11762 (*emulans*).

Clément, A.-.M. 741438 (*emulans*).

Clement, E.J. TB93/723 (*americanum*).

Clements, F. 2789 (*emulans*); 2915 (*triflorum*).

Clements, F.E. 87 (*americanum*); 148 (*triflorum*).

Cléonique-Joseph 8071 (*nigrum*); 12618 (*emulans*).

Clevidence, J.P. 7526 (*americanum*).

Clokey, I.W. 233 (*emulans*); 5201 (*douglasii*); 8485 (*triflorum*).

Clos, C.E. 169, 238, 439, 2871, 2878, 4152 (*chenopodioides*); 4170 (*triflorum*); 5336 (*chenopodioides*).

Clos, E.C. 1139, 1843, 3357, 3840 (*chenopodioides*).

Close, B. 1094-22 (*americanum*).

Clover, E.U. 917 (*nigrescens*); 1516 (*americanum*); 7177 (*douglasii*).

Clute, W.N. 126 (*triflorum*).

Coburn, F.S. 1263 (*americanum*); 1349b (*douglasii*).

Cochrane, A.C. 563 (*chenopodioides*).

Cochrane, T.S. 3005, 5142, 5351 (*emulans*); 8481 (*nigrescens*); 12568 (*douglasii*).

Cockull, M. 13 (*pseudogracile*).

Cocucci, A.A. 399 (*triflorum*); 2589 (*nitidibaccatum*).

Codd, L.E. 7243 (*scabrum*); 7443 (*villosum*); 10152 (*retroflexum*).

Codrington, K. 41 (*nigrum*).

Coeli, R. 116 (*americanum*).

Cogollo P, Á. 601 (*americanum*).

Cogollo, A. 451 (*americanum*).

Coile, N.C. 1910 (*emulans*).

Cole, A.E. 3717/3211 (*emulans*).

Cole, D.T. 923 (*scabrum*).

Cole, M.M. 6019 (*scabrum*).

Coleridge, A. 55 (*villosum*).

Collenette, C.L. 239 (*villosum*).

Collenette, I.S. 72-146, 1542, 1592, 1730, 2675, 2696, 3133, 3309, 3313, 3332, 4349 (*villosum*); 6212 (*nigrum*).

Collett, H. 111, 416 (*villosum*).

Collett, R.D. 61 (*emulans*).

Collins, D.J. 1579 (*nigrum*).

Collins, F.S. 281 (*americanum*); 896, 3837 (*emulans*).

Combita, S. 114, 114 (*macrotonum*).

Combs, J. 170 (*triflorum*).

Commerson, P. 45, 46 (*chenopodioides*).

Commis, R.B. 283 (*americanum*).

Compton, R.H. 659, 781 (*americanum*); 25011 (*retroflexum*).

Comte, F. 112, 772a (*emulans*); 4551 (*triflorum*); 4920 (*emulans*).

Conceição, S.F. 310 (*americanum*).

Congdon, J.W. 65 (*nitidibaccatum*).

Conklin, H.C. 79580 (*americanum*).

Conn, B.J. 88, 323, 490 (*americanum*).

Connock, J. 262 (*americanum*).

Conrad, J. 1998, 2120, 2551 (*americanum*); 2653 (*chenopodioides*); 2852 (*americanum*); 3100, 3113, 3133, 3179 (*nigrescens*).

Constable, E.F. 5630 (*chenopodioides*); 5633 (*americanum*); 5966, 6161, 6164, 6897 (*chenopodioides*); 11099 (*triflorum*).

Constable, H.L.W. 1192 (*americanum*).

Constance, L. 786 (*americanum*); 9939 (*triflorum*).

Contreras J, J.L. 8242, 8324, 8915, 9574 (*nigrescens*).

Contreras, E. 5290 (*douglasii*); 6052 (*nigrescens*).

Conzatti, C. 3178 (*nigrescens*); 4736 (*pruinosum*).

Coode, M.J.E. 1141 (*villosum*); 4582 (*scabrum*).

Coogan, A.H. 268 (*emulans*).

Cook, A.C. 809 (*americanum*).

Cook, L. 527 (*emulans*).

Cook, O.F. 460, 533 (*americanum*).

Cooke, L.A. 46 (*villosum*).

Cooley, G.R. 2545 (*americanum*); 4800 (*pseudogracile*); 6505 (*americanum*); 6666, 11850 (*pseudogracile*).

Coombs, R.E. 2456 (*triflorum*).

Cooper, J.G. 33 (*douglasii*).

Cooper, J.J. 5863 (*nigrescens*).

Cooper, R. 6019 (*nigrum*).

Cooper, R.E. 3355 (*americanum*).

Cooper, T. 187 (*retroflexum*).

Cooray, R.G. 68-100301R (*americanum*).

Copley, B. 1838 (*retroflexum*).

Coppejans, E. 3 (*nigrum*).

Corbin, B. 1552 (*triflorum*).

Corbin, B.L. 1475, 1552 (*triflorum*).

Corcoran, M.R. MARC-42 (*americanum*).

Cordeiro, J. 1938 (*americanum*).

Córdoba, J. 379 (*nigrescens*).

Córdoba, J.J. 2181 (*nigrescens*).

Córdoba, W.A. 422 (*americanum*).

Core, E.L. 1011 (*macrotonum*); 3219 (*emulans*).

Corkern, C.B. 26 (*nigrescens*).

Corley, R.H.V. 68 (*nigrum*).

Cornejo Tenorio, G. 3, 357, 726, 1105, 1588, 1706, 2195 (*douglasii*); 2678 (*nigrescens*); 3552 (*pruinosum*); 3628 (*americanum*).

Cornell Tropical Botany Field Trip 61, 71 (*macrotonum*).

Cornes, M. 263 (*villosum*).

Coronado G, I. 1433 (*nigrescens*).

Coronado, I. 341, 1142, 1542 (*nigrescens*).

Correa A, M.D. 1308, 1614 (*nigrescens*).

Correa Luna, H. 9147 (*furcatum*).

Correa, A. 2991 (*americanum*).

Correa, M.N. 5041, 5046, 5125 (*americanum*); 5138 (*chenopodioides*); 8467 (*americanum*).

Correia, M.F. 1136, 1741 (*villosum*).

Correll, D.S. 14052 (*douglasii*); 18969 (*americanum*); 24399, 26403 (*nigrescens*); 30328 (*interius*); 30999 (*nigrescens*); 34986 (*douglasii*); 36773 (*nigrescens*); 39597 (*triflorum*).

Corrick, M.G. 7567 (*americanum*).

Cortés R, C. 55, 137 (*americanum*).

Cortés, M.E. 13 (*americanum*); 68, 113, 156, 463, 463 (*nigrescens*).

Cory, V.L. 3452 (*nigrescens*); 8154 (*pseudogracile*); 11955, 19358 (*nigrescens*); 44760 (*douglasii*); 51403 (*nigrescens*); 52234 (*emulans*).

Cosminsky, S. 26 (*nigrescens*).

Costa, D. 242 (*americanum*).

Costa-Lima, J.L. 1605, 1837 (*americanum*).

Costich, D.E. 841 (*americanum*); 1507 (*nigrescens*).

Cottam, G. 160 (*emulans*).

Cottam, W.P. 3877 (*triflorum*); 12893 (*douglasii*).

Cotton Experimental Station 10 (*americanum*).

Cotton, J.S. 871 (*interius*).

Coues, E. 162 (*douglasii*).

Coulter, T. 591, 592 (*douglasii*); 1231 (*americanum*); 1232 (*nigrescens*).

Countryman, W.D. 1428, 2167 (*emulans*).

Couto, D.R. 677 (*americanum*).

Coveny, R.G. 742 (*chenopodioides*); 825 (*triflorum*); 4036 (*chenopodioides*); 5425 (*triflorum*); 6087 (*nigrum*); 6088, 6088, 6159 (*chenopodioides*); 6397 (*nigrum*); 6398, 6398, 6424, 6486, 6486, 6487 (*americanum*); 6535 (*chenopodioides*); 6536, 6537, 7465 (*americanum*); 7466, 7466 (*chenopodioides*); 9020, 9998 (*americanum*); 11827, 11827 (*chenopodioides*); 13589 (*nigrum*).

Coville, F.V. 185 (*nigrum*); 256, 1256 (*americanum*).

Cowan, C. 1809, 1868, 2367 (*nigrescens*).

Cowan, I.M. 3 (*emulans*).

Cowan, J.H. 4197 (*interius*).

Cowan, R.S. 38176 (*americanum*).

Cowden, M.C. 5370 (*emulans*).

Cowen, J.H. 371 (*triflorum*).

Cowgill, W.H. 1072 (*americanum*); 2076 (*nigrum*).

Cowie, I. 4476 (*americanum*).

Cowles, H.C. 232 (*triflorum*).

Craib, W.G. 579 (*nigrum*).

Craig, M. 12552 (*emulans*).

Crandall, C.S. 167, 678 (*triflorum*).

Craven, L. 9442 (*americanum*).

Craven, L.A. 2580 (*chenopodioides*).

Crawford, I. 418 (*americanum*); 4024, 5827 (*chenopodioides*); 6196 (*triflorum*); 6312 (*chenopodioides*).

Creed, K.E. 280 (*americanum*).

Cremers, G. 8084 (*americanum*).

Creswell, A.M. 31 (*villosum*).

Cribb, P. 10246, 10345 (*americanum*).

Crisp, M.D. 1984, 2141 (*americanum*); 2494 (*chenopodioides*); 4470 (*americanum*).

Crisp, P. 123 (*nigrum*).

Croat, T.B. 965 (*nigrescens*); 3965 (*emulans*); 7618, 11415, 12513, 15761, 16359, 17324 (*nigrescens*); 19668, 20836 (*americanum*); 21488 (*macrotonum*); 21544 (*nigrescens*); 21777

(*americanum*); 21810 (*macrotonum*); 21810 (*nigrescens*); 22394 (*macrotonum*); 22408, 23035, 23380 (*nigrescens*); 24287 (*americanum*); 24847 (*nigrescens*); 25067 (*americanum*); 25294 (*nigrescens*); 28440, 28489, 28522, 28684 (*americanum*); 29606, 29698 (*scabrum*); 32726, 34188 (*nigrescens*); 34391, 34504 (*americanum*); 34833 (*nigrescens*); 34893 (*macrotonum*); 35405, 35488, 37983, 38084, 38107 (*nigrescens*); 38415 (*americanum*); 38473 (*nigrescens*); 38843 (*macrotonum*); 39585, 40212 (*nigrescens*); 40481 (*douglasii*); 41051 (*nigrescens*); 41082 (*douglasii*); 41378 (*nigrescens*); 41994, 42188 (*douglasii*); 42376 (*nigrescens*); 44051, 44159 (*douglasii*); 45216 (*nigrescens*); 45319, 45330 (*douglasii*); 45845, 46019, 47546, 47563, 47582, 47672 (*nigrescens*); 51125, 51537 (*americanum*); 54460 (*nigrescens*); 54826 (*macrotonum*); 55237 (*nigrescens*); 55251 (*macrotonum*); 57786, 58383 (*americanum*); 58409 (*corymbosum*); 60534 (*macrotonum*); 62947 (*americanum*); 63122, 63334 (*nigrescens*); 63499 (*macrotonum*); 63596, 63776, 64242 (*nigrescens*); 64402 (*americanum*); 64807, 64864, 64980, 65199 (*nigrescens*); 65478 (*americanum*); 66001 (*nigrescens*); 66041 (*americanum*); 66137A (*nigrescens*); 70552 (*americanum*); 72219 (*macrotonum*); 74460, 74531 (*americanum*); 77396 (*nigrum*); 78525, 78564, 79059 (*nigrescens*); 79083 (*macrotonum*); 81916, 85407 (*americanum*); 86293, 86468, 89211, 89882, 89999, 90777 (*macrotonum*); 92283 (*americanum*); 93223, 93228 (*macrotonum*); 97419 (*nigrescens*); 97618 (*americanum*); 98274 (*nigrescens*); 99171, 99281, 99634 (*macrotonum*); 100097 (*nigrescens*); 100194 (*americanum*).

Crockett, R.L. 1289 (*nigrescens*).

Crofts, F. 7 (*americanum*).

Cromwell, G.D. 634 (*douglasii*).

Cronk, Q.C.B. 335 (*nigrum*); 346 (*villosum*); 409, 410, 447, 460 (*nigrum*); 488 (*villosum*).

Crookshank, H. 98 (*villosum*).

Crosby, C.S. 117 (*americanum*).

Crosby, M.R. 1717, 2905 (*americanum*); 14571 (*emulans*).

Crosby, M.T. 7 (*chenopodioides*).

Crosby, V. 2595 (*triflorum*).

Cross, R.C. 237 (*emulans*).

Crosswhite, F.S. 544 (*douglasii*); 1245 (*triflorum*).

Crouch, V.E. 1029 (*americanum*).

Crovetto, M. 4954 (*americanum*).

Crow, G.E. 2329 (*nigrum*); 6258 (*macrotonum*).

Cruden, R.W. 1100 (*americanum*).

Crutchfield, J.R. 173 (*interius*); 3199 (*americanum*).

Cruz A, S.P. 612 (*nigrescens*).

Cruz D, R. 5672 (*nigrescens*).

Cruz Espinosa, C.A. 1864 (*nigrescens*).

Cruz Rivas, A. 130ac (*nigrescens*); 244ac, 268ac (*americanum*).

Cruz, J.S. de la 1664, 4237, 4442, 4602 (*americanum*).

Cruz, M. 101 (*nigrescens*).

Cruz-Cisneros, R. 472 (*corymbosum*).

Cuatrecasas, J. 278 (*macrotonum*); 1703 (*nigrescens*); 5374, 7897, 11810, 12017, 12379, 12491, 14750, 18456, 18884, 20235 (*macrotonum*); 22491 (*nigrescens*); 22978 (*americanum*); 27658 (*macrotonum*).

Cuello, N. 1376 (*macrotonum*).

Cueva M, M. 612 (*americanum*).

Cufodontis, G. 410, 438 (*nigrescens*).

Culbertson, C. 100 (*emulans*).

Culhati, S.C. 13 (*nigrum*).

Cull, I. 748, 748a (*emulans*).

Cuming, H. 266[a], 266[b] (*furcatum*); 649 (*americanum*); 783[b] (*nitidibaccatum*); 999 (*americanum*); 1033 (*nigrum*); 21263 (*nitidibaccatum*).

Cumming, R.J. 22821 (*americanum*).

Cunha, L.S. 72 (*americanum*).

Cunnell, G.J. 167 (*villosum*).

Cuong, N.M. 551 (*americanum*).

Curle, C. 27 (*villosum*).

Curran, H.M. 64 (*americanum*); 188 (*chenopodioides*).

Curtis, A.G. 310 (*villosum*).

Curtiss, A.H. 5594 (*pseudogracile*); 5595 (*americanum*); 6493 (*villosum*); 6863 (*nigrum*).

Cushman, J.A. 7492 (*emulans*).

Cusick, W.C. 3326a (*triflorum*).

Cutak, L. 8 (*douglasii*).

Cutler, H.C. 4341 (*douglasii*).

D'Arcy, W.G. 543 (*americanum*); 1641 (*nigrescens*); 1670 (*pseudogracile*); 2244, 2278, 2291, 2366, 2392 (*americanum*); 2467 (*chenopodioides*); 2483, 2493, 2500, 2506, 2570 (*americanum*); 2574 (*nigrescens*); 2597, 2619A, 2624, 2625 (*americanum*); 2637 (*nigrescens*); 2638 (*americanum*); 2653 (*macrotonum*); 2654 (*nigrescens*); 2852 (*americanum*); 2930 (*pseudogracile*); 3002 (*americanum*); 3644 (*nigrum*); 3692 (*americanum*); 3717 (*triflorum*); 3875 (*emulans*); 3899, 3900, 3966 (*americanum*); 4245A, 4251, 4253B, 4261, 4267, 4271, 4290 (*nigrescens*); 5122 (*americanum*); 5257, 5272, 5328, 5438, 5448, 5461 (*nigrescens*); 5533 (*americanum*); 5599, 5675, 5710, 5711 (*triflorum*); 6035, 6052, 6171 (*americanum*); 6367 (*nigrescens*); 6569 (*macrotonum*); 6585, 6841 (*nigrescens*); 6891 (*emulans*); 9818, 10146, 10428 (*nigrescens*); 10474 (*americanum*); 10522 (*emulans*); 10663 (*nigrescens*); 10892 (*macrotonum*); 11035, 11266 (*nigrescens*); 11676B (*americanum*); 11843, 11869 (*nigrescens*); 11877 (*pruinosum*); 11882 (*douglasii*); 11892 (*americanum*); 11909, 11909 (*nigrescens*); 11916 (*douglasii*); 11986 (*americanum*); 12097 (*nigrescens*); 12125 (*americanum*); 12479 (*macrotonum*); 12631 (*nigrescens*); 12823 (*macrotonum*); 13116 (*nigrescens*); 13127, 13160, 13177 (*macrotonum*); 13201 (*nigrescens*); 13268 (*macrotonum*); 13386 (*americanum*); 13486 (*nigrescens*); 14041 (*americanum*); 14834 (*macrotonum*); 15025 (*nigrescens*); 15201, 15212 (*americanum*); 15215 (*scabrum*); 15282, 15304, 15305, 15307, 15348 (*americanum*); 15607 (*nigrescens*); 15623 (*macrotonum*); 15657 (*nigrescens*); 15667, 16468, 16498, 16501A (*macrotonum*); 17521, 17535 (*americanum*); 17571 (*scabrum*); 17602 (*americanum*); 17617 (*scabrum*); 17681 (*americanum*); 17682 (*scabrum*); 17684, 17697

(*americanum*); 17698 (*scabrum*); 17698 (*americanum*); 17765 (*scabrum*); 17766 (*nigrum*); 17877, 18012 (*nigrescens*); 18243 (*macrotonum*); 19269 (*americanum*).

Dagnon, C. 18 (*emulans*).

Dahl, A. 905 (*nigrum*).

Dahl, H.S. D.39b., D.45a. (*nigrum*).

Dahlstrand, K.A. 2919 (*retroflexum*).

Dainelli, G. 90, 94, 98 (*villosum*).

Dalby, J.M. 88/05 (*nigrum*).

Dale Thomas, R. 89695 (*nigrescens*); 148045, 153473 (*pseudogracile*).

Dalton, M.G. 8 (*villosum*).

Daly, D. 6634 (*americanum*).

Daly, D.C. 9161 (*americanum*).

Dalziel, J.M. 1188 (*americanum*); 1188a, 1188b (*scabrum*).

Damon, A. 43, 163 (*nigrescens*).

Damrel, D.Z. 1764B (*douglasii*); 4118 (*emulans*).

Dandy, J.E. 135 (*villosum*); 624 (*scabrum*).

Dangol, D.R. 12336 (*nigrum*).

Daniel, P. NC-67369 (*americanum*).

Daniel, P.M. 117 (*scabrum*).

Daniel, T.C. 28 (*emulans*).

Daniel, T.F. 1407 (*douglasii*).

Dann, M.P. 60 (*nigrum*).

Danna, J.A. 2 (*douglasii*).

Dansereau, A. 954 (*emulans*).

Dantas, M. 18 (*americanum*).

Dantas, M.M. 18 (*americanum*).

Dar, S. 3688 (*nigrescens*).

Daramola, B.D. 154 (*scabrum*).

Daramola, B.O. FHI-72483 (*scabrum*).

Darko, K.O. 1032, 5115 (*scabrum*).

Das, C.R. 112 (*nigrum*).

Das, D. 105 (*villosum*); 209 (*americanum*).

Das, R.K. 33921 (*nigrum*).

Dash, S.S. BSHC-21171 (*americanum*).

Daveau, J. 2446 (*villosum*).

Davidse, G. 1624 (*macrotonum*); 1967 (*emulans*); 2054 (*macrotonum*); 5089 (*nigrescens*); 5472 (*americanum*); 6704, 6812 (*retroflexum*); 7371 (*americanum*); 10248 (*macrotonum*); 10549, 11202, 11819 (*americanum*); 13450 (*nigrescens*); 14511 (*americanum*); 18475, 20926A (*nigrescens*); 23151 (*macrotonum*); 23993, 24608 (*nigrescens*); 24665, 24685, 24717, 25141, 25168 (*macrotonum*); 26150, 28073, 28147, 29109, 32397, 35727 (*nigrescens*); 36560 (*americanum*); 38552, 38586 (*emulans*); 38975 (*sarrachoides*); 39023, 41078 (*emulans*).

Davidson, C. 10577 (*americanum*).

Davidson, M.E. 183 (*nigrescens*).

Davidson, R.A. 2845 (*interius*); 3354 (*emulans*).

Davies, J.N. 222 (*americanum*).

Davies, R.M. 1514 (*scabrum*).

Dávila, M. 19 (*nigrescens*).

Davis, D. 62812 (*villosum*).

Davis, J. 96, 371, 3714 (*emulans*).

Davis, P.H. 26067, 1514K, 2080, 6070B, 26067, D38512, 40176, D47039, D48539, 50060, 53890, 55296, 67986, D68139 (*villosum*); 70402 (*chenopodioides*).

Davis, R.J. 297-35 (*nitidibaccatum*); 2829 (*triflorum*); 2978 (*nitidibaccatum*); 2996 (*triflorum*).

Davis, R.M. 1190 (*retroflexum*).

Davis, T. 609, 620, 641 (*nigrescens*); 661 (*nitidibaccatum*); 662 (*triflorum*); 664, 667, 671 (*douglasii*); 678 (*interius*); 703, 712 (*nigrescens*); 714 (*americanum*); 735, 760, 800 (*nigrescens*); 1001 (*douglasii*); 1132, 1133, 1134 (*nigrescens*); 1142, 1143 (*douglasii*).

Dawe, M.T. 289 (*nigrescens*).

Dawson, A.W. 4 (*douglasii*).

Dawson, G. 434 (*americanum*); 2457, 2716 (*nitidibaccatum*).

DBL 373 (*villosum*).

DCI (Darwin Chilean Initiative 2002-2005) 1815 (*furcatum*).

de Lange, P.J. CH-625, K-790 (*americanum*); K-814, K-847 (*nigrum*); K-863, K-1269 (*americanum*); K-1270, K-1294, K-1317, 2913, 3632 (*nigrum*); 6292 (*nitidibaccatum*).

de Mera, G. 1510 (*nitidibaccatum*).

Deam, C.C. 1834 (*americanum*); 2415, 13763, 15278, 16648, 17016, 18631, 33355, 35125, 35151, 37075, 37606, 37998, 41569, 53116, 57458, 58077, 58159 (*emulans*); 60240 (*pseudogracile*); 63478 (*emulans*); 64063, 64100 (*pseudogracile*).

Dean, C.C. 14 (*nigrescens*).

Dean, C.H. 8987 (*villosum*).

Deb, D.B. BG-3534 (*villosum*); 27496 (*americanum*).

DeBolt, A. 1745, 1745 (*triflorum*).

Decary, R. 755[a], 755[b], 2600, 6179, 6656, 19370 (*americanum*).

Decker, B.G. 1280, 2185 (*americanum*).

Degen, R. 831, 2203 (*americanum*); 3132 (*sarrachoides*).

Degener, O. 1203, 16190 (*triflorum*); 26198 (*nigrescens*); 26274 (*douglasii*); 33618, 33660, 34134, 35908, 36120 (*americanum*).

Deginani, N. 1281, 1321 (*americanum*).

Deguchi, K. 5408 (*nigrum*).

Deighton, F.C. 474, 1033 (*americanum*); 4571 (*scabrum*); 5660 (*americanum*).

Deignan, H.G. 199 (*emulans*).

Del Aguila, M. 162 (*americanum*).

Del Vitto, L.A. 851 (*triflorum*); 3411, 4071 (*nitidibaccatum*).

Delgadillo, C. 96 (*nigrescens*).

Delgado B, A.A. 11 (*americanum*).

Delgado, L.B.S. 2773 (*americanum*).

Delimitation Commission under Colonel Wahab 60 (*villosum*).

Delnatte, C. 1544 (*nigrescens*); 2622 (*nigrum*); 2930, 3039, 3484 (*americanum*).

DeLozier, P.E. 1356 (*emulans*).

Demaio, P. 294 (*chenopodioides*).

Demaree, D. 3427, 8571, 8738, 8789 (*emulans*); 10443 (*douglasii*); 14077, 16032, 16455, 18217, 20087, 20323, 20327, 25558 (*emulans*); 29533 (*triflorum*).

Dempster, L.T. 26 (*americanum*); 1097 (*douglasii*).

Denham, S.S. 352 (*chenopodioides*).

Descoings, B. 775 (*americanum*).

Déséglise, A. 457 (*villosum*); 460 (*nigrum*).

Desert Locust Survey P.T.49 (*villosum*).

Despréaux, J.M. Webb-44 (*villosum*).

Detling, L.E. 2224, 5438 (*triflorum*); 8442 (*douglasii*).

Dewart, F.W. 53 (*emulans*).

DeWolf, G.P. 149 (*nigrescens*).

Dey, D. 107A (*nigrescens*).

Dhoromick, D.C. 5291 (*americanum*).

Dhwoj, L. 51 (*nigrum*).

Diabate, M. 1419 (*scabrum*).

Diamini, M.D. 612 (*retroflexum*).

Dias, R.S. 02 (*americanum*).

Díaz Gonzáles, J. 791 (*americanum*).

Díaz M, D. 1010 (*nigrescens*); 1057 (*americanum*).

Díaz P, S. 502 (*macrotonum*).

Díaz Rico, A. 95 (*nigrescens*).

Díaz S, C. 2354 (*americanum*).

Díaz V, I. 1107 (*nigrescens*).

Díaz Vilchis, I. 120 (*nigrescens*); 381 (*americanum*); 544, 559, 905, 1377 (*douglasii*).

Díaz Z, A.L. 193 (*americanum*).

Díaz, C. 3539, 9478, 9503 (*americanum*).

Díaz, R. 334 (*nigrescens*).

Diáz, W. 5295 (*nigrescens*).

Dickerson, L. 43 (*emulans*).

Dickson, H.L. 7156 (*triflorum*).

Dickson, H.V. 387 (*villosum*).

Dickson, J.D. 103 (*nigrum*).

Dieckman, L. 172 (*nigrescens*); 177 (*americanum*).

Diego P, N. 220 (*nigrescens*).

Dieren, W. van 687 (*triflorum*).

Dieterle, J.V.A. 1211 (*emulans*).

Dieterlen, A. 157 (*retroflexum*).

Dietrich, A. 1523 (*americanum*).

Diggs, G.M. 3952 (*douglasii*).

Dik, A. 1310 (*americanum*).

Dillewaard, H.A. 210 (*americanum*).

Dillon, L.A. 915 (*triflorum*).

Dillon, M.O. 3641 (*americanum*).

Dillon, S.T. 52 (*triflorum*).

Dinklage, M. 2116 (*americanum*).

Dinsmore, J.E. 907, 1151, 2810, 5171, 8810, 9151, 10810, 11151, 12102, 12810 (*villosum*).

Dister, D.C. 264 (*emulans*).

Dittrich, V.A.O. 159 (*chenopodioides*).

Dixon, R.H. 1644 (*nitidibaccatum*).

Dixon, S. 1/99 (*nigrum*); 148/99 (*villosum*).

DNEP1 303 (*nigrum*).

Doan, S. 891, 914 (*douglasii*).

Döbbeler, P. 1072 (*americanum*); 1309 (*nigrescens*).

Dobereiner 756 (*americanum*).

Dobson, A.T. 283, 300, 337 (*nigrum*).

Dodds, G.S. 1857 (*triflorum*).

Dodge, C.K. 24 (*nigrescens*).

Dodson, C.H. 1817A (*macrotonum*); 11523 (*americanum*); 12067 (*macrotonum*).

Dolan, J. 19 (*americanum*).

Dolman, J. 331 (*nigrum*).

Dombey, J. 63 (*corymbosum*).

Dombrowski, L.T. 237, 310, 477, 3564, 3689, 4153, 4798, 7776, 9767 (*americanum*).

Domer, B.F. 684 (*triflorum*).

Domingo, M.A. 57 (*nitidibaccatum*).

Domínguez Adena, R. 290 (*douglasii*).

Domínguez Cadena, R. 2936 (*americanum*).

Domínguez Vázquez, G. 9 (*nigrescens*).

Donaldson Smith, A. 218, 221 (*villosum*).

Donat, A. 55, 55a (*triflorum*); 415 (*nitidibaccatum*).

Dönmez, A.A. 13562, 16221 (*nigrum*).

Donnell Smith, J. 6667 (*nigrescens*).

Donner, N.N. 10365 (*nigrum*).

Dony, J.G. 4165 (*sarrachoides*).

Doran, A.S. 672 (*furcatum*).

Dorantes, B. 2471, 2746 (*nigrescens*).

Dorantes, J. 1865, 1983 (*nigrescens*); 4009 (*americanum*).

Dorantes-Hernandez, F.D. 257 (*nigrescens*).

Dorn, R. 5469 (*emulans*); 5753 (*interius*).

Dorn, R.D. 3323, 4823, 5816, 7548, 7548, 9716 (*triflorum*).

Dorr, L.J. 2729 (*americanum*); 5400 (*nigrescens*); 7941, 7960 (*emulans*); 8414, 8971 (*macrotonum*).

Doug, J.G. 1401 (*nitidibaccatum*).

Douglas, D. 23 (*americanum*).

Doutrelepont, H. 1203 (*scabrum*).

Dowell, P. 8642, 8651, 8652, 8653 (*emulans*).

Dowson, W.J. 299 (*villosum*).

Doyen, J.T. 34 (*furcatum*).

Drakeford, W.T. 13 (*americanum*).

Drège, J.F. 7864a, 7864b (*retroflexum*).

Dreisbach, R. 3211 (*emulans*).

Dress, W.J. 3024 (*douglasii*).

Dressler, R.L. 118 (*corymbosum*); 160, 3735 (*nigrescens*).

Drewe, P.B. 174 (*retroflexum*).

Drouet, F.E. 2412 (*americanum*); 3559, 3800 (*douglasii*); 4131 (*triflorum*).

Drumke, J. 29642 (*emulans*).

Drummond, J.R. 1872, 2293, 20551, 25857 (*nigrum*); 25867 (*villosum*); 25868 (*nigrum*).

Dryander, E. 2769 (*nigrescens*).

Dryer, V.J. 568 (*nigrescens*); 1312 (*americanum*).

Du Parquet, R. 253 (*villosum*).

Duan, L.D. 3648, 20020641 (*nigrum*).

Duaneh, J. 319, 320, 444 (*americanum*).

Dubugnon, N. 187 (*chenopodioides*).

Dueholm, K.H. 8620, 8653 (*triflorum*).

Duffey, E.A.G. 200 (*americanum*).

Duffour, C. 5538 (*nitidibaccatum*).

Dugand G, A. 1103, 4537, 5024 (*americanum*); 7080 (*nigrescens*).

Dugand, A. 4537 (*nigrescens*); 5024 (*americanum*).

Dujhlón, F.C. FCD1811 (*scabrum*).

Duke, J.A. 3979 (*nigrescens*); 4044, 5199, 7336 (*americanum*); 8722, 12022 (*nigrescens*); 15437 (*americanum*).

Dullas, W. 123 (*douglasii*).

Dulong Jiang Investigation Team 46 (*americanum*); 241, 287, 1022 (*nigrum*).

Dulta, A.K. 32, 235 (*villosum*).

Dunaski, A. 89 (*americanum*).

Duncan, W.H. 3835 (*americanum*); 8783, 11840 (*emulans*); 17439, 20636, 21344, 29142, 29165, 30736 (*pseudogracile*).

Dunn, D.B. 16383 (*triflorum*); 16384 (*nigrum*); 22489 (*nigrescens*).

Duno de Stefano, R. 1203 (*macrotonum*).

Duque Jaramillo, J.M. 2476 (*americanum*); 3649 (*nigrescens*); 3665, 4047A (*americanum*); 4100 (*nigrescens*).

Durán F, A. 624, 637 (*nigrescens*).

Durán, R. 1425 (*americanum*).

Duré, R. 208 (*americanum*).

Dusén, P. 386 (*furcatum*); 5465 (*triflorum*); 9512, 10201 (*americanum*).

Duthie, J.F. 7535 (*villosum*).

Dutt, A.K. 647 (*villosum*); 651 (*nigrum*).

Dutt, O.D. 192 (*interius*).

Dutta, R.M. 50 (*nigrum*); 261, 507 (*americanum*).

Dutton, D.L. 398 (*emulans*).

Duvdevani, S. 284 (*villosum*).

Dwyer, J. 13193 (*villosum*); 15211 (*nigrescens*).

Dwyer, J.D. 1510 (*nigrescens*); 1855 (*americanum*); 2027 (*nigrescens*); 6787, 7325 (*americanum*); 7596, 9997 (*nigrescens*); 11145 (*americanum*); 13061 (*nigrescens*); 13147, 13811 (*villosum*); 15211, 15238 (*nigrescens*).

Dybowska, A. 853 (*nigrum*).

Dyess, K.K. 23 (*emulans*).

Dziekanowski, C.T. 1716 (*nigrescens*); 1861, 1952 (*douglasii*); 2554 (*triflorum*).

Dzuik, P. 1022 (*nitidibaccatum*).

Eames, A.J. 4925, 12866 (*emulans*).

Eames, E.H. 3123, 5399 (*emulans*).

Earle, E.C. 4001 (*emulans*).

Earle, F.S. 152, 152, 192 (*triflorum*); 958 (*emulans*).

Earthy, E.D. 8 (*scabrum*).

East India Company 2618 (*villosum*).

Easterly, N.W. 12015 (*emulans*).

Eastwood, A. 187 (*douglasii*); 375 (*furcatum*); 1924, 2407 (*douglasii*); 4783 (*americanum*); 18966 (*douglasii*).

Ebertowski, P.J. 1958, 6025, 6349, 8468, 8663, 8693 (*triflorum*).

Echeverría, J.A. 391 (*nigrescens*); 1015 (*americanum*).

Economic Botanist 25214 (*nigrum*).

Economides, S. ARI-980 (*villosum*).

Edaño, G.E. 24897 (*americanum*); 37715 (*nigrum*).

Edgecombe, M. 94 (*americanum*).

Edgeworth 7009, 7045 (*villosum*).

Edmonds, J.M. C73, C74 (*retroflexum*); C101 (*nigrescens*).

Edmondson, J.R. 3311, E3419 (*villosum*).

Edwards, D.C. 3104 (*villosum*).

Edwards, S. 3674 (*villosum*).

Edwards, W.C. 97, 144 (*triflorum*).

Egea, J. de 807 (*americanum*).

Eggers, H.F.A. 286 (*americanum*).

Eggler, W.A. 4 (*nigrescens*).

Eggleston, W.W. 5321 (*emulans*); 14742, 16189 (*triflorum*); 16310, 16436 (*interius*); 16766 (*nigrescens*); 17396 (*douglasii*); 18924, 20256 (*triflorum*).

Ehlers, J.H. 612, 1122, 1593 (*emulans*).

Eichler, H. 21649 (*chenopodioides*).

Eichler, J. 43 (*triflorum*).

Eickwort, G. 7 (*americanum*).

Einarsson, S. 234 (*nigrum*).

Eisinger, S. 37, 38, 40 (*americanum*).

Eiten, G. 1596, 1895, 4753, 6239, 7211, 7876, 10152 (*americanum*).

EJD 555 (*emulans*).

Ekman, E.L. 10010, 12012 (*nigrescens*); H-12038 (*americanum*).

El Assi, A. 523 (*villosum*).

El Ghani, M.A. 1195, 1453, 1653, 3633, 4339, 4517 (*nigrum*); 4552 (*villosum*); 5151, 6982, 7106 (*nigrum*).

Elbert, J. 4547 (*americanum*).

Elias 219 (*americanum*); 853 (*nigrescens*).

Elías, H. 2954 (*villosum*).

Elias, S.I. 178 (*americanum*).

Elias, T.S. 1671 (*nigrescens*); 10580 (*douglasii*); 10628 (*americanum*).

Elliot, B. 4027, 8807a, 10194 (*triflorum*).

Ellis, C.C. 64, 251 (*triflorum*); 381 (*interius*).

Ellis, J. 100 (*emulans*).

Ellis, R. 557 (*villosum*).

Ellman, E. 68, 338 (*nigrum*).

Elmer, A.D.E. 525 (*nitidibaccatum*); 3945 (*douglasii*); 8105 (*americanum*); 8788, 15697 (*nigrum*); 17677 (*americanum*).

Elorsa C, M. 27 (*nigrescens*); 101 (*americanum*); 497, 1015, 1322 (*nigrescens*); 1444 (*americanum*); 1729, 1732, 1908, 1995, 2141, 2685, 2922, 3093, 3307, 4110, 4209, 4343, 4549, 4724, 4828, 4921, 5171, 6023 (*nigrescens*).

Elvidge, D. 87 (*americanum*); 88 (*nitidibaccatum*); 89 (*nigrum*).

Embree, F.W. 264, 315 (*douglasii*).

Emig, W.H. 373 (*interius*).

Emson, H.E. 296 (*americanum*).

Emwiogbon, J.A. FHI-43533 (*scabrum*).

Encarnación B, R. 71 (*americanum*).

Endlich, R. 596 (*villosum*).

Endrès, A.R. 183 (*nigrescens*).

Engard, R.G. 158, 182, 930, 988, 1150 (*douglasii*).

England, J.L. 1062 (*triflorum*).

Enríquez E, E.D. 1763, 1773 (*nigrescens*).

Enríquez, O.G. 315 (*nigrescens*).

Enti, A.A. 1723 (*scabrum*).

Erlanson, C.O. 848 (*emulans*).

Erlanson, E.W. 5070 (*nigrum*).

Ern, H. 883 (*scabrum*).

Ernst, W.R. 1320 (*nigrescens*).

Ertter, B. G-124 (*americanum*); 2431 (*nitidibaccatum*).

Ertter, B.J. 163/4, 2373 (*triflorum*); 10845, 10879 (*americanum*); 11320 (*nitidibaccatum*); 13908, 14924, 15384, 15395 (*americanum*); 20019 (*nitidibaccatum*); 20649, 22113 (*triflorum*).

Ervi, L.O. 783302 (*nigrum*).

Escobar N, J.A. 236 (*nigrescens*).

Escobar, L.A. 6459 (*macrotonum*).

Escobedo, J.M. 2466 (*nigrescens*).

Escolastico, R. 65 (*nigrescens*).

Espejo, A. 1403 (*nigrescens*).

Espinal O, F. 237 (*nigrescens*).

Espinal, M. 179 (*nigrescens*).

Espínola, M.C. JPB666 (*americanum*).

Espinosa Garcia, F.J. 197, 243 (*americanum*).

Espinosa H, A. 47 (*americanum*); 95 (*douglasii*).

Espinosa, J. 75 (*douglasii*); 164, 248 (*nigrescens*).

Espinoza, R. 85 (*americanum*).

Espinoza, Y. 33 (*americanum*).

Espírito Santo, J. 218 (*scabrum*).

Esplen, J.C. 103 (*villosum*).

Essi, L. 303 (*americanum*).

Estelrich 7 (*triflorum*).

Estève, L. 111 (*scabrum*).

Estrada, J. 149 (*nigrescens*).

Etkin, N.L. 63A (*villosum*); 63[b] (*scabrum*).

Etter, A.G. 246, 286 (*emulans*).

Etuge, M. 1556 (*scabrum*).

Eugenio, J. 1089 (*americanum*).

Euphrosin-Joseph [Frère] 4642 (*nigrum*).

Eupunino, E. 489 (*americanum*).

Evangelista, M. 45 (*nigrescens*).

Evans, D.K. 544 (*emulans*).

Evans, I.M. 57 (*villosum*).

Evans, O.D. 3 (*chenopodioides*).

Evans, T.A. 162-87/11 (*emulans*).

Everist, S.L. 2642, 5606 (*americanum*); 7291 (*nigrum*); 10054 (*americanum*).

Evert, E.F. 4523, 4538, 5058, 17172, 21480, 33103, 34999, 36428 (*triflorum*).

Evrard, C. 10603 (*furcatum*).

Ewan, J. 18277, 19917 (*nigrescens*).

Ewan, J.A. 3715 (*douglasii*); 5250, 8157 (*americanum*); 10775 (*douglasii*); 12130, 14108, 15498 (*nitidibaccatum*); 15691 (*americanum*); 18001, 18260, 19853 (*nigrescens*).

Exell, A.W. 89, 396 (*scabrum*).

Expedition Purdy 109 (*villosum*).

Eyden, V. van den 463, 701, 980 (*americanum*).

Eyenhuisen, G. van 7, 25 (*scabrum*).

Eyerdam, W.J. 1601 (*nitidibaccatum*); 23162 (*chenopodioides*); 23555 (*triflorum*); 23682 (*chenopodioides*); 23905, 23954, 24449 (*triflorum*).

Eyles, D.E. 8408 (*emulans*).

Faber, E. 63, 618 (*nigrum*).

Fabris, H.A. 3175 (*nitidibaccatum*).

Faden, R.B. 67/198, 74/676 (*villosum*).

Fagbemi, A. 438 (*scabrum*).

Fagerlind, F. 517, 607, 696 (*americanum*); 770, 1436 (*macrotonum*).

Fagerström, K. 62 (*villosum*).

Faircloth, W.R. 1553 (*emulans*).

Falcão, M. 133 (*americanum*).

Fan Hsioh Niao 9120 (*nigrum*).

Fan, C.S. 179 (*nigrum*).

Fang, W.P. 12359, 12462, 19724 (*nigrum*).

Fang, W.Y. 8001 (*nigrum*).

Fanshawe, D.B. F1085 (*americanum*); F-5353, JMM-8310 (*retroflexum*).

Farfán, J. 1196, 1721 (*americanum*).

Farnsworth, E.L. 244 (*nitidibaccatum*).

Farnsworth, N.R. SW-397 (*emulans*).

Farrera S, O. 491 (*nigrescens*); 3917 (*douglasii*).

Farruggia, F. 2637, 2646, 2713, 2737, 2781 (*americanum*).

Fassett, N.C. 437, 3394, 5602, 5627, 8158, 8793, 9778, 10447, 13364, 13445, 14672, 15553, 16218, 16220, 19169 (*emulans*).

Faulkner, H.G. 1780, 3120 (*americanum*); 3746 (*villosum*); 4036, 4065 (*americanum*); 4858 (*villosum*).

Faurie, U. 512 (*nigrum*); 636, 637 (*americanum*); 690, 777 (*nigrum*); 863, 880 (*americanum*); 1172, 1915, 3109, 5979, 6720 (*nigrum*).

Fay, J.M. 1012 (*villosum*).

Fay, M.J. 1738 (*emulans*).

Fayed, A.A. 1295 (*villosum*).

Fealy, C. 31 (*chenopodioides*).

Felger, R.S. 90-73, 89-170 (*americanum*); 05-170, 03-406, 90-506 (*douglasii*); 88-614, 85-667 (*americanum*); 85-1361 (*nigrescens*).

Felisbino, [?] 25 (*americanum*).

Félix, A.L. 55 (*americanum*).

Fell, E. 58-533, 57-860, 57-1454 (*emulans*).

Feltwell, J. 19 (*douglasii*).

Fendler, A. 249, 606 (*americanum*); 671 (*triflorum*); 1021 (*nigrescens*).

Feng, H.T. 31 (*nigrum*).

Feng, K.M. 2350, 3391 (*nigrum*).

Fensham, R. 1290 (*americanum*).

Feres, F. 0796 (*americanum*).

Ferguson, D.M. 474 (*nigrescens*).

Ferguson, E. 69 (*douglasii*).

Ferguson, L.F. 2731, 2884, 2927 (*villosum*).

Ferguson, M.C. 4123, 4123b, 4189 (*nigrescens*).

Ferguson, W.C. 3186, 3240 (*emulans*).

Fernald, M.L. 206, 2090, 5439, 10338 (*emulans*); 10339 (*villosum*); 10808, 12793, 13439 (*emulans*); 15382 (*nigrum*); 15566, 19068 (*emulans*); 24437, 24438 (*nigrum*); 153682 (*emulans*).

Fernandes, A. 9373 (*nigrum*).

Fernandes, D. 235 (*chenopodioides*); 291 (*americanum*).

Fernández Alonso, J.L. 5845A (*americanum*); 6409 (*nigrescens*); 6498, 6746, 6772, 6911 (*americanum*); 6934, 6957, 7111, 13247 (*nigrescens*); 15973, 16864 (*americanum*); 19978 (*macrotonum*); 20782 (*nigrescens*); 20790, 21674 (*americanum*); 21802, 24755 (*nigrescens*); 24816 (*americanum*).

Fernández N, R. 1352 (*americanum*).

Fernández y Acosta 2067 (*nigrescens*).

Fernández, A. 588 (*nigrescens*).

Fernandez, A. 1526 (*nigrescens*).

Fernández, P. 49 (*chenopodioides*).

Fernández-Casas, J. 7290, 7389 (*americanum*); 11725, 11820 (*scabrum*).

Ferreira, A.P. 236 (*americanum*).

Ferreira, V.F. 3045 (*americanum*).

Ferreyra, E.C. de 7773 (*corymbosum*).

Ferreyra, R. 716 (*corymbosum*); 4402, 11625, 12836 (*americanum*); 13760 (*corymbosum*); 17231, 17683, 18866, 19959 (*americanum*).

Ferris, R.S. 2837 (*interius*); 3122, 3132 (*nigrescens*); 9998 (*douglasii*); 11104 (*americanum*); 11105, 11106 (*douglasii*); 13116 (*nitidibaccatum*).

Fertig, W. 12368, 18026 (*triflorum*); 30076, 30376 (*douglasii*).

Fidalgo de Carvalho, M. 3150 (*americanum*).

Field, H. 24 (*nigrum*).

Fields, D. 818, 1992 (*emulans*).

Figueiredo, E. 95 (*scabrum*).

Figueroa Rosas, J. 34 (*americanum*).

Fiker, C.B. 3469 (*douglasii*).

Filipi, I. 1167 (*villosum*).

Filipps, R. De 164 (*americanum*).

Filson, R.B. 3743 (*americanum*).

Finch, A.J. 208 (*douglasii*).

Fink, B. 365 (*emulans*).

Fiori, A. 1597, 1598, 1599, 1958 (*villosum*).

Firmin, G. 611 (*macrotonum*).

Fischer, S. 26 (*nigrum*).

Fischer, W. 100, 215 (*triflorum*).

Fish, J. 10 (*douglasii*).

Fishbein, M. 2216 (*douglasii*).

Fisher, G.L. 255 (*douglasii*).

Fisher, J.C. 1325 (*americanum*).

Fisher, M. 60 (*emulans*).

Fishlock, W.C. 8 (*americanum*).

Fitzpatrick, T.J.; Fitzpatrick, M.F.L. 77 (*interius*).

Fitzpatrick, W.M. 9 (*villosum*).

Flaig, J. 2621 (*triflorum*).

Flamigni, A. 352 (*scabrum*).

Flanagan, M. 8 (*emulans*).

Fleetwood, R.J. 759 (*triflorum*).

Fleming, G.P. 6150, 14371, 14383, 15738, 16063 (*sarrachoides*).

Fleming, P. 897, 929, 1197, 1243, 2367, 2418 (*emulans*).

Flinn, M.A. 1524, 1525, 1526 (*triflorum*).

Florence, J. 2724, 3946, 4154, 4365, 5031 (*americanum*).

Flores C, A. 718 (*americanum*).

Flores Castoreno, A. 718 (*nigrescens*).

Flores Martinez, [?] 255 (*nigrescens*).

Flores Tolentino, M. 251 (*americanum*).

Flores V, J.C. 51 (*nigrescens*).

Flores, G. 27 (*americanum*); 3456 (*nigrescens*).

Flores, S. 8, 61, 133 (*americanum*).

Flores-Franco, G. 159, 291, 816, 3456 (*nigrescens*); 3482, 4128, 4169, 4179, 4196, 4196 (*douglasii*); 4270 (*nigrescens*); 4310, 4643 (*douglasii*); 5222 (*nigrescens*).

Flowers, S. 94, 146, 853 (*triflorum*).

FLSP 759, 949, 1116, 1325, 1415 (*americanum*).

Flynn, D. 17 (*chenopodioides*).

Fodor, F. 242 (*nitidibaccatum*).

Fogaça, J. 02 (*americanum*).

Fogg, J.M. 1462 (*emulans*); 1806 (*triflorum*); 2872 (*emulans*).

Foggie, A. 21 (*villosum*).

Fohlen S-40a (*villosum*).

Follett, W.I. 10 (*douglasii*).

Folsom, J.P. 2061, 3514, 4046, 4581, 4908, 4966 (*nigrescens*); 5161 (*americanum*); 6074 (*macrotonum*); 6850 (*nigrescens*).

Fonnegra G, R. 2452, 2722 (*americanum*); 5148, 5193 (*nigrescens*); 6167 (*macrotonum*).

Fonnegra, R. 2670 (*macrotonum*).

Fonseca B, M.H. 12 (*macrotonum*).

Fonseca, J. 2015-545, 2015-545 (*douglasii*).

Font Quer, P. 560, 561 (*villosum*).

Fontana, A.P. 811, 4837, 5764 (*americanum*).

Forbes, F.B. 307 (*nigrum*).

Forbes, H.O. 2544 (*nigrum*); 3785 (*americanum*); 3881 (*nigrum*).

Forbes, S.J. 341A, 909 (*triflorum*); 937 (*americanum*).

Foreman, R.E. 194 (*americanum*).

Forero P, L.E. 426 (*nigrescens*); 428, 466, 1620 (*americanum*).

Forero, E. 15 (*macrotonum*); 1974 (*americanum*).

Forest Office 42514 (*villosum*).

Forrest, G. 4479, 7289, 8373, 12277 (*nigrum*).

Forster, P.I. 26512, 26512, 29317 (*americanum*); 37983, 42109 (*chenopodioides*).

Forstner, S. 2464 (*nigrum*).

Fortunato, R.H. 6041, 6063 (*americanum*); 7480 (*triflorum*).

Forwood, W.H. 276 (*triflorum*).

Forzza, R.C. 4309 (*americanum*).

Fosberg, F.R. S4312 (*douglasii*); 18545 (*emulans*); 20503 (*americanum*); 20559 (*macrotonum*); 24120 (*emulans*); 25149, 25348 (*americanum*); 27528 (*macrotonum*); 27930 (*americanum*); 27961[a] (*corymbosum*); 27961[b], 28236, 29282, 31177, 32631, 35310, 44774, 44785, 44788, 44823, 48351, 48896 (*americanum*); 55780 (*douglasii*); 57125, 57408, 59773, 61328 (*americanum*).

Foster, R. 5470 (*triflorum*).

Foster, R.B. 9756, 10334 (*americanum*).

Fouts, C.L. 188 (*douglasii*).

Fox, M.D. 8204069 (*americanum*).

Fox, W.B. 3243, 3732 (*pseudogracile*).

Franceschi, F. 16 (*americanum*); 17 (*douglasii*).

Francia, P. 4 (*americanum*).

Franck, C.W. 180 (*nigrum*).

Frank, M.E.W. 137 (*americanum*).

Franklin, [?] 16383 (*triflorum*).

Fraser, S.V. 833 (*nitidibaccatum*).

Fredholm, A. 5405, 5914 (*pseudogracile*); 6224 (*americanum*); 6301 (*pseudogracile*).

Freeborn, R. 267 (*nigrescens*).

Freeman, C.C. 1510 (*triflorum*); 18469 (*interius*).

Freeman, J.D. 15 (*americanum*).

Frei, B. 103 (*nigrescens*).

Freire de Carvalho, L d'A 148, 153, 570 (*americanum*).

Freitag, H. 2075, 5203 (*villosum*).

Freitas, E. 285 (*americanum*).

Freitas, L. 4, 5 (*americanum*).

French, N. 342, 664 (*douglasii*).

Friedrichsthal, E. von 545 (*nigrescens*); 558 (*americanum*); 1729 (*nigrescens*).

Friesner, R.F. 17874 (*emulans*).

Friis, I. 6612, 8350, 10411, 10524, 10525, 10536, 10699 (*villosum*).

Frisch, R.O. 133 (*americanum*).

Fritchey, J.Q.A. 120 (*triflorum*).

Fritsch, P.W. 807 (*emulans*).

Fróes, R.L. 11663 (*americanum*).

Frogley, W. 1663 (*triflorum*).

Frohlich, D. DF-4-P (*triflorum*).

Frost, F.H. 92, 92 (*triflorum*).

Fry, W. 339 (*americanum*).

Fryxell, P.A. 1767 (*emulans*) 2669 (*nigrescens*).

Fuchs Q, F.M. 211 (*americanum*); 1745 (*nigrescens*).

Fuchs, F.M. 115 (*nigrescens*).

Fuentes, R.E. PPI-38985 (*nigrum*).

Fuess, F.W. 23 (*emulans*).

Fujikawa, K. 50167, 89358, 94443 (*americanum*).

Fulton, H.J. 9668 (*douglasii*).

Funez, L.A. 129 (*americanum*).

Funk, V. 14109, 14111 (*emulans*).

Funk, V.A. 2582 (*nigrescens*).

Furlaud, J.M. 79 (*nigrum*).

Furse, P. 2821, 7778 (*nigrum*); 8230 (*villosum*); 8932 (*nigrum*).

Furtado, C.X. 25897 (*scabrum*).

Furuse, M. 1962 (*americanum*); 4837 (*nigrum*); 7793 (*americanum*); 9548, 26892 (*nigrum*); 40504 (*americanum*); 40522, 40677, 40678, 42704, 42834, 44637, 45139, 46072, 46168 (*nigrum*).

Gabrielith, R. 24, 121 (*villosum*).

Gachathi, F.N. 366 (*villosum*).

Gadelha Neto, P.C. 736 (*americanum*).

Gaerlan PPI-5373[a] (*americanum*).

Gaerlan, F. PPI-5373[b] (*nigrum*).

Gálan, P. 21 (*nigrescens*).

Galán, P. 4753 (*scabrum*).

Gale, M. 6 (*triflorum*).

Galeano, G. 2156, 2176B, 7602 (*macrotonum*).

Galeano, M.P. 239, 495, 2270 (*macrotonum*).

Galeotti, H.G. 1229[1] (*douglasii*); 1229[2], 1229[c], 1238 (*nigrescens*).

Galindo T, R. 1237 (*macrotonum*).

Gallagher, M.D. 7487/10 (*villosum*).

Gallardo, C. 393 (*nigrescens*).

Gallinal PE3710 (*chenopodioides*).

Galpin, E.E. M-747 (*retroflexum*); 14832 (*chenopodioides*).

Galván, R. 3266 (*nigrescens*).

Gamble, J.S. 3398 (*nigrum*); 4478A (*villosum*); 15153, 17435 (*americanum*).

Gamboa R.; Billen 1371 (*nigrescens*).

Gamboa, B. 942, 1371 (*macrotonum*).

Game, J.C. 69/306 (*americanum*).

Gammie, G.A. 276 (*americanum*); 18462 (*nigrum*).

Gandara, J.M. 84 (*nigrescens*).

Gandoger, M. 17, 27, 215, 282 (*nigrum*).

Gaoligong Shan Biodiversity Survey 15695, 17210, 18169, 19296 (*nigrum*); 19656 (*americanum*); 21066, 21646, 22185, 22260, 22490, 22849, 23095, 23409 (*nigrum*); 23485 (*americanum*); 25609, 25832, 27451 (*nigrum*); 27683, 28955 (*americanum*); 32463, 33280, 34191 (*nigrum*).

Gaoligong Shan Biotic Survey Expedition Autumn 19656 (*nigrum*).

Gaoligong Shan Expedition 7345 (*americanum*).

García E, J.D. 16 (*americanum*).

García P, J. 1255 (*nigrescens*); 1643 (*douglasii*).

García, [?] 51 (*nigrescens*).

García, M. 53 (*douglasii*).

García, M.A. 1417 (*villosum*).

García, P. 210 (*americanum*).

Garcia, R.J.F. 464 (*americanum*).

García-Barriga, H. 17 C751 (*macrotonum*); 6308 (*americanum*); 7784, 11700 (*macrotonum*); 12321 (*nigrescens*); 12684, 12875, 12931 (*macrotonum*); 18415 (*americanum*); 20736, 20977 (*macrotonum*).

Gardiner, J.S. 108 (*americanum*).

Gardner, G. 838, 1788, 2266 (*americanum*).

Gardner, M.F. 82, 94, 174 (*furcatum*); 1252 (*villosum*); 4034, 6738, 6963, 8322, 8356, 8462, 8478, 8649 (*furcatum*).

Gardner, R.O. 10165, 11230 (*americanum*).

Gargiullo, M.B. 902 (*americanum*).

Garlitz, D. 694, 927, 1006, 1114, 1140, 1227, 1505, 1521 (*emulans*).

Garlitz, R. 2145 (*emulans*).

Garnett, C.S. 1017 (*villosum*).

Garrett, A.O. 1097 (*nitidibaccatum*); 2087, 2184 (*triflorum*); 2967 (*nitidibaccatum*); 5219 (*triflorum*); 6219, 6606 (*nitidibaccatum*); 7480, 8671 (*triflorum*); 8736 (*nitidibaccatum*).

Garske, S.C. 0898-21 (*emulans*).

Garton, C.E. 14758 (*nitidibaccatum*); 15255 (*triflorum*).

Garwood, A.E. 1321 (*emulans*).

Garwood, N. 338, 1235 (*macrotonum*).

Garwood, N.C. 338, 1235 (*macrotonum*).

Gasper, A.L. de 3194 (*americanum*).

Gasperetti, P.R. PG-422 (*nigrum*).

Gates, B.N. 7170, 13431, 18499 (*emulans*).

Gates, F.C. 64, 3252 (*emulans*); 18861 (*interius*).

Gates, W.C. 32495 (*emulans*).

Gathorne-Hardy, R.E. 823 (*villosum*).

Gaudichaud, C. 112 (*furcatum*); 520 (*chenopodioides*); 521, 522 (*americanum*); 1847 (*furcatum*).

Gaumer, G.F. 61, 479 (*nigrescens*); 1533 (*americanum*); 23546 (*nigrescens*).

Gautier, L. 5198 (*scabrum*).

Gay, C. 2 (*furcatum*).

Gbile, E.C. FHI-20567 (*scabrum*).

Ge, X. 242 (*americanum*).

Geay, F. 8096, 8978 (*americanum*).

Geerinck-Coutrez 4368 (*nigrum*).

Geerling, C. 417 (*scabrum*); 1498 (*americanum*).

Gentle, P.H. 7799 (*americanum*).

Gentry, A.H. 949, 982 (*nigrescens*); 6621, 6758 (*americanum*); 8445, 9040 (*nigrescens*); 9040 (*macrotonum*); 9375 (*americanum*); 15129, 15129 (*macrotonum*); 16416, 16464 (*americanum*); 17066, 17066 (*macrotonum*); 17944, 25381, 27256, 27384, 28535 (*americanum*); 30379, 30379, 41286 (*macrotonum*); 47738, 54148 (*nigrescens*); 60329 (*macrotonum*); 63561, 63701 (*americanum*).

Gentry, H.S. 1269 (*americanum*); 1712, 3083 (*douglasii*); 4232 (*americanum*); 6611 (*douglasii*); 8523 (*pruinosum*); 17816, 18218, 18393 (*douglasii*); 19596, 19601 (*nigrescens*).

Gentry, J.L. 671 (*emulans*); 2672, 2675, 2874, 2884, 2962 (*nigrescens*); 3018 (*americanum*); 3778, 3794 (*nigrescens*).

Geoffray, C. 180 (*americanum*).

George, A.S. 3172 (*americanum*); 16913 (*triflorum*).

Gereau, R.E. 1548 (*americanum*); 1863 (*macrotonum*); 2421 (*emulans*).

Germishuizen, G. 7215 (*retroflexum*).

Gerrans, M.B. 1560, 1619 (*nigrum*).

Gershov, A. 600 (*emulans*).

Geyer, C.A. 248 (*triflorum*).

Geyers, C.A. 251 (*interius*).

Ghafoor, A. 4432 (*villosum*); 5204 (*nigrum*).

Ghosh, A.K. CNH-30437, 30497 (*villosum*); 37675 (*americanum*).

Ghosh, M.K. 57, 75 (*americanum*); CU-11036 (*villosum*).

Giacomin, L.L. 1646, 1971, 1974, 2009, 2013, 2021, 2103 (*americanum*).

Giardelli, M.L. 1165 (*sarrachoides*).

Gibbons, R.B. 294 (*villosum*).

Gibbs Russell, G.E. 3704 (*retroflexum*).

Gibbs, P.E. 1236.69 (*villosum*).

Gibert, E.J. 147 (*sarrachoides*).

Gibson, N. 3953 (*americanum*).

Gil, M. 21 (*americanum*).

Gilbert, M.G. 6305 (*villosum*).

Gilbert, V.C. E37, 2655 (*villosum*).

Gill, N. 645 (*villosum*).

Gillespie, J.W. 1110 (*americanum*).

Gillespie, L. 25 (*villosum*).

Gillett, J.B. 4126, 4923 (*villosum*); 15249 (*scabrum*); 16081, 19889 (*villosum*).

Gillett, J.M. 16487 (*americanum*).

Gilli, A. 30 (*nigrum*); 222 (*americanum*); 373 (*macrotonum*); 3240, 3241, 3242 (*villosum*).

Gilliat-Smith, B. 2123a (*villosum*).

Gillies, J. 3 (*sarrachoides*); 18, 35 (*triflorum*); 38 (*chenopodioides*); 43, 44 (*triflorum*); 151 (*chenopodioides*); 1434 (*triflorum*).

Gilliland, H.B. 476B, 974 (*retroflexum*).

Gillis, W.T. 7233, 7677 (*americanum*); 13025 (*nigrescens*).

Gilly, C. 87 (*emulans*).

Gilman, A.V. 9148 (*nitidibaccatum*).

Gilmartin, A.J. 581 (*americanum*).

Gilon, A. 315[a] (*scabrum*).

Gimate L, J. 616 (*douglasii*).

Gines, H. 2072 (*americanum*).

Giordano, L.C. 748 (*americanum*).

Giordano, O. 3 (*nitidibaccatum*); 8 (*triflorum*); 22 (*nitidibaccatum*).

Giraldez, X. 18394 (*nitidibaccatum*).

Giraldo, G. 979 (*americanum*).

Givens, F.M. 2294, 5537 (*nigrescens*).

Glad, J.B. 77-74 (*triflorum*).

Glanville, R.R. 412 (*scabrum*).

Glassman, S.F. 8609 (*emulans*).

Glaumann, F. 105 (*americanum*).

Gleason, H.A. 121, 1806, 1938 (*emulans*).

Glen, H.F. 1943 (*retroflexum*).

Glenfield Vet Research Station SN45-13 (*triflorum*); SN48/2638 (*chenopodioides*).

Glennon, J.M. 1500 (*triflorum*).

Glocker, E.F. von 85 (*americanum*).

Glover, P.E. 180, 494, 615, 1238, 2252 (*villosum*).

Godfrey, R.K. 2001 (*emulans*); 49808 (*pseudogracile*); 52830, 53220 (*americanum*); 53610 (*nigrescens*); 57199 (*americanum*); 66028, 66048 (*nigrescens*); 71259 (*pseudogracile*).

Godman, Dame A. 93 (*villosum*).

Godoy, S.A.P. de 2024, 2033, 2047, 2063 (*americanum*).

Goel, A.K. NC-53907 (*americanum*); NC-64200, NC-64620 (*villosum*).

Goës, C.C. 596 (*americanum*).

Góes, O.C. 97 (*americanum*).

Goetghebeur, P. 2605, 5252, 8626 (*triflorum*).

Gold, D.B. 629 (*douglasii*).

Goldblatt, P. 5553 (*retroflexum*); 6780 (*chenopodioides*); 7915, 8416 (*retroflexum*).

Goldman, D. DG-568 (*douglasii*).

Goldsmith, B.C. 74 (*triflorum*).

Goldsmith, P. 64 (*douglasii*).

Gomes, E. 279 (*americanum*).

Gomes, J.M.L. 150 (*americanum*).

Gomes, L.A. 495 (*americanum*).

Gómez Chagala, B. 306 (*americanum*).

Gómez López, M. 156, 309, 468 (*nigrescens*).

Gómez Marín, E. 157 (*scabrum*).

Gómez N, C. 112 (*nigrescens*).

Gómez P, L.D. 20912 (*nigrescens*).

Gómez S, F. 168 (*nigrescens*).

Gómez Santiz, F. 21 (*nigrescens*).

Gómez Santíz, F. 168 (*nigrescens*).

Gómez Santiz, F. 275, 310 (*nigrescens*).

Gómez Santiz, G. 310 (*nigrescens*).

Gomez, A. 5, 75 (*macrotonum*).

Gómez, J. 51 (*corymbosum*).

Gómez, J.C. 5 (*macrotonum*).

Gómez, L.D. 20912 (*macrotonum*); 23321 (*nigrescens*).

Gómez, M. 598 (*nigrescens*).

Gómez-Laurito, J. 5674, 5928, 5964, 6080, 6309, 7557, 11378 (*nigrescens*).

Gómez-López, M. 426 (*douglasii*).

Goncalves, A.C. PSACF_EX4342 (*americanum*).

Goncalves, B. 240 (*nigrum*); 374 (*chenopodioides*); 1277, 2262, 2607, 3513, 3762, 4064 (*nigrum*); 4698 (*chenopodioides*).

Gonde, H.P. 371 (*scabrum*).

Góngora, E. 137 (*nigrescens*).

Gonzales Q, L. 3240 (*corymbosum*).

Gonzales, A. 583 (*americanum*).

Gonzáles, P. 6 (*corymbosum*); 1738 (*americanum*); 1818, 2860, 2929, 2937 (*corymbosum*); 2964, 2972 (*americanum*).

Gonzales, R. 518 (*macrotonum*).

González B, F.A. 1403, 1404, 1405, 1406, 2461, 2462 (*nigrescens*); 3308, 3309, 3310 (*macrotonum*); 3311 (*nigrescens*).

González de L, D. 8 (*nigrescens*).

González E. 822 (*americanum*).

González Elizondo, M.S. 458 (*nigrescens*).

González G, L. 239 (*nigrescens*).

González L, L.A. P-553 (*nigrescens*); 4152 (*americanum*).

González Medrano, F. 1874, 3215 (*nigrescens*); 6643 (*douglasii*); 14713 (*nigrescens*); 16948 (*pruinosum*); 16964 (*americanum*).

González Ortega, J. 585 (*nigrescens*).

González R, A. 323 (*douglasii*).

González R, E. 42 (*americanum*).

González, C.E. 3466 (*nigrescens*).

González, F. 1656 (*nigrescens*).

González, G. 19 (*nigrescens*).

González, H. 17 (*macrotonum*).

González, J. 1833, 2196 (*nigrescens*).

González, L. 1519 (*nigrescens*).

González, L.A. 4152 (*nigrescens*).

González, M. 1410 (*nigrescens*).

González, S. 3112 (*americanum*).

González-Espinosa, M. 1185 (*americanum*).

González-Medrano, F. 17494 (*pruinosum*).

Goodding, L.M. 11-46, 406-45, 482-45, 2285 (*douglasii*).

Goode, P.M. G3-72 (*scabrum*).

Goodier, R. 287 (*villosum*).

Goodman, C.M. 613 (*triflorum*).

Goodman, G.J. 3413 (*nigrescens*).

Goodner, F.S. 1273 (*triflorum*); 1274 (*nitidibaccatum*).

Goodrich, S. 19277 (*nitidibaccatum*).

Goodwin, R.H. 84-342 (*emulans*).

Goodwing, H.B. 197 (*americanum*).

Goring, V. 2 (*nigrum*).

Gorman, M.W. 3657 (*triflorum*).

Gosper, D. 12 (*americanum*).

Gossweiler, J. 464 (*americanum*); 530b (*scabrum*).

Gould, B.J. 628 (*nigrum*).

Gould, F.W. 2115, 2298, 5210 (*douglasii*).

Goulding, J.H. 563, 718 (*nigrum*).

Govus, T. 997 (*pseudogracile*).

Goward, T. 81-915 (*nitidibaccatum*).

Goytowski, A. 52 (*emulans*).

Graan, J.F. van 406 (*retroflexum*).

Grable, A.E. 6000 (*triflorum*).

Grace 6 (*villosum*).

Graham, J.G. 231, 4146 (*americanum*).

Graham, R.A. 1409 (*nitidibaccatum*).

Graham, R.D. 315 (*villosum*).

Grainger, J. 67 (*villosum*).

Granados-Tochoy, J.C. 892 (*americanum*).

Grant V. 8234, 8319 (*triflorum*).

Grant, G.B. 1219 (*douglasii*).

Grant, J.M. 367 (*nitidibaccatum*).

Grant, M.L. 3623, 7433, 7439 (*americanum*); 15861 (*nigrum*).

Grassely, P.G. AR426 (*triflorum*).

Grassl, C.O. 2500, 5612 (*emulans*).

Graumann, H. 80 (*emulans*).

Gray, M. 3712, 5901 (*triflorum*); 6796 (*nitidibaccatum*).

Grayum, M.H. 5220, 7128, 7434, 7523, 9696 (*nigrescens*); 9714 (*macrotonum*).

Graywood Smyth, E. 19 (*americanum*).

Green, P.S. RSNH1265, 1571 (*americanum*).

Greenaway, J. JG-50 (*americanum*).

Greene, E.L. 322 (*triflorum*).

Greenman, J.M. 50 (*americanum*); 167 (*nigrescens*); 281, 3734 (*emulans*); 5308 (*nigrescens*).

Greenway, P.J. 6882, 6997, 10635 (*villosum*).

Gregg, H.R. 217 (*emulans*).

Gregg, J. 425 (*nigrescens*); 459 (*douglasii*); 474 (*nigrescens*); 755 (*douglasii*).

Gregory, D.P. 220 (*douglasii*).

Greidanus, T. 609 (*emulans*).

Gremmen, N.J.M. T070024 (*nigrum*).

Greuter, W. 26375 (*nigrescens*); 26375 (*americanum*).

Grierson, A.J.C. 1324 (*americanum*).

Grierson, M. 253 (*villosum*).

Grierson, R. H.955-18 (*triflorum*).

Griffith [663] (*nigrum*).

Griffith, W. 5901/1 (*nigrum*).

Griffiths, D. 5787 (*triflorum*).

Grijalva, A. 634 (*macrotonum*); 1322, 1441 (*nigrescens*); 1506 (*americanum*); 1508 (*nigrescens*); 2976, 4098 (*americanum*).

Grimes, E.J. 367 (*emulans*).

Grimes, J. 1304 (*triflorum*).

Grimshaw, J.M. 93-455 (*americanum*); 93-728 (*villosum*).

Grinnell, J. 361 (*douglasii*).

Grisales, A. 9 (*nigrescens*).

Grondona, [?] 3014, 3058 (*americanum*).

Grossheim, A. I27 (*villosum*).

Grossman, J. 560 (*emulans*).

Grove, A.T. 5, 31A (*villosum*).

Grubb, P.J. 562 (*macrotonum*).

Gruber, E.H. 232 (*triflorum*).

Grunden, B. 12-172 (*emulans*).

Guadarrama O, M.A. 6607, 6842, 6959.5 (*nigrescens*).

Guaglianone, E.R. 1443 (*nitidibaccatum*).

Gualteros, F. 3 (*nigrescens*).

Guam Experiment Station 438 (*americanum*).

Guánchez M, F.J. 2122 (*americanum*).

Guarneros, Y. 412 (*douglasii*).

Güemes, J. 1517 (*villosum*).

Güerere, I. 42 (*nigrescens*).

Guerrero, W. 336 (*americanum*).

Guertin, P. 4981 (*nigrum*).

Guest, E.R. 181, 304 (*nigrum*).

Guichard, K.M. KG/HAD/234 (*nigrum*); KG/Lib/447 (*villosum*).

Guidinger, J.H. 2353 (*emulans*).

Guillarmond, A.J. 5158 (*sarrachoides*); 5160 (*retroflexum*).

Guillon, M.A. 4234 (*emulans*).

Guimarães, J.G. 14 (*americanum*).

Guinea, E. 1111, 1348, 1637 (*scabrum*).

Guinena, A. 42 (*americanum*).

Guiol, F.G. 1564 (*nigrum*); 1601 (*villosum*); 1602 (*nigrum*).

Guízar N, E. 514, 517, 4260 (*douglasii*).

Guízar Nolazco, E. 5587 (*douglasii*).

Gunn, S.M. HI-5 (*pseudogracile*); CH-45, ESI-71, WSI-74 (*nigrescens*); FP-84, HI-104, PB-111 (*pseudogracile*).

Gunn-Zumo, S.M. DB-415 (*nigrescens*).

Gurini, L. 301 (*chenopodioides*).

Gust, G. 518 (*nigrum*); 603 (*douglasii*).

Gutiérrez B, C. 6077 (*nigrescens*).

Gutiérrez Baez, C. 7130 (*nigrescens*).

Gutiérrez G, J. 558 (*nigrescens*).

Gutiérrez V, G. StaRsa-1 (*macrotonum*); 17C669 (*americanum*); 430 (*nigrescens*); 17 C669 (*macrotonum*).

Gutiérrez, G. Sib-3 (*macrotonum*).

Gutiérrez, J. 14 (*chenopodioides*).

Gutiérrez, M.J. 484 (*nigrescens*).

Gutiérrez, R. SRB43, SRB98 (*nigrescens*).

Gutte, P. 8149, 8619 (*americanum*).

Guzmán, M. 130, 289 (*nigrescens*); 470 (*douglasii*); 756, 957 (*nigrescens*); 1262 (*douglasii*); 1397 (*americanum*); 1617, 1805 (*nigrescens*); 1945, 1978 (*douglasii*).

Guzmán, R. 3064 (*nigrescens*).

Guzmán-Teare, M. 756, 979, 1805 (*americanum*).

Haarer, A.E. 566 (*villosum*).

Haase, E.F. 715 (*douglasii*).

Haast, J.F. von 265 (*nigrum*).

Haber, W.A. 606, 635, 1032 (*nigrescens*); 3438 (*americanum*); 10051,10094 (*nigrescens*); 10149 (*americanum*); 10875 (*nigrescens*); 11198 (*americanum*); 11339 (*macrotonum*); 11714, 11891 (*americanum*).

Haberer, J.V. 638 (*emulans*).

Hackforth-Jones, J. 396 (*americanum*).

Haegele, R.W. A-11 (*triflorum*).

Haegi, L. 1602 (*chenopodioides*); 1607 (*americanum*); 1711 (*chenopodioides*).

Haegi, L.A.R. 1607 (*americanum*); 1714 (*chenopodioides*); 1715 (*americanum*); 1724 (*chenopodioides*); 1725 (*americanum*); 1730 (*chenopodioides*); 1737, 1744 (*americanum*); 1756 (*chenopodioides*).

Hage, J.L. 485, 927, 989 (*americanum*).

Hagelund, K. 132, 1024, 1469, 4548, 5110, 5452, 7361, 7649, 7690, 7790, 7820, 8237, 9494, 10565, 10749, 10885, 10894, 11737, 14520, 15291, 15996 (*americanum*).

Hahn, L. 1, 325, 325[a] (*americanum*).

Hahn, W. 531 (*nigrescens*); 766, 4818 (*americanum*); 5010 (*nigrescens*); 5021 (*macrotonum*).

Hahn, W.J. 528 (*nigrescens*); 531, 2056, 2470 (*americanum*); 5021 (*macrotonum*).

Hainault, R. 770, 3501 (*emulans*).

Haines, H.H. 1979 (*villosum*).

Haines, J. 1987, 4464, 4489, 4746, 5060, 5606 (*triflorum*).

Hajra, P.K. NC-82320 (*americanum*).

Hale, M. 44 (*villosum*).

Hale, S. 5348, 5353, 5506 (*douglasii*).

Haley, B.R. 228 (*villosum*).

Hall, E. 467, 667 (*triflorum*).

Hall, G.P. 29, 84 (*villosum*).

Hall, H.H. 21, 564 (*triflorum*).

Hall, H.N. 41 (*nigrum*).

Hall, J.B. 2411, 2416 (*americanum*); 46137 (*scabrum*); 47120 (*americanum*); 47139 (*scabrum*).

Hall, J.D. JH-24 (*emulans*).

Hallé, N. RSNH6400 (*americanum*).

Halliday, T.A. 354 (*americanum*).

Halse, R.R. 2725 (*nigrum*); 4702 (*triflorum*); 6115 (*americanum*); 8122 (*nitidibaccatum*); 8733, 9306 (*americanum*).

Halvorson, W.L. 76-24 (*emulans*).

Hamad, K. NHI-46355 (*villosum*).

Hamblett, R.B. 622 (*nigrescens*).

Hamel, C. 535 (*scabrum*).

Hamid, H. NHI-42695 (*villosum*).

Hamilton, C. 1231 (*nigrescens*); 4195 (*emulans*).

Hammel, B. 655 (*triflorum*); 1355, 3285 (*nigrescens*); 3827 (*americanum*); 4242, 4340, 4802, 5303 (*nigrescens*); 5404 (*americanum*); 5895 (*furcatum*); 6465 (*nigrescens*); 6555, 6637 (*macrotonum*); 6964 (*nitidibaccatum*); 6981 (*nigrescens*); 6997, 7003 (*macrotonum*); 8783 (*nigrescens*); 22881 (*americanum*); 23601 (*macrotonum*); 24586, 26442 (*nigrescens*).

Hammen, T. van der 916 (*nigrescens*).

Hammond, H.D. 7812 (*emulans*); 11511, 11928 (*nitidibaccatum*).

Hammond, M.M. 172 (*villosum*).

Hampshire, R.J. 80 (*nigrum*); 417 (*villosum*); 439 (*nigrum*); 488 (*nigrescens*); 730 (*americanum*).

Hams, E.H.M. 10 (*villosum*).

Hanan A, M.A. 996 (*nigrescens*).

Hanan-Alipi, A.M. 996 (*nigrescens*).

Hanbury-Tracy, J. 454 (*nigrescens*).

Hance, H.F. 861 (*nigrum*).

Handel-Mazzetti, H.F. 859 (*villosum*); 920 (*nigrum*); 2561, 2923 (*villosum*); 3133 (*nigrum*).

Hanekom, W.J. 564 (*chenopodioides*); 2499 (*retroflexum*).

Hanneman, R.E. 22 (*emulans*).

Hansen, B. 1317 (*emulans*); 1721 (*americanum*); 3981, 4009, 4058, 4235, 4685 (*emulans*).

Hansen, B.F. 7419 (*nigrescens*).

Hansen, C.G. 1055 (*triflorum*).

Hansen, C.J. 3432 (*emulans*).

Hansen, G. 1175, 1388 (*americanum*).

Hansen, H. 222 (*emulans*).

Hansen, J.J. 18 (*emulans*).

Hanson, C.G. SP-07-20 (*villosum*); BS9625, BS9730 (*nigrum*); Yugo86-12 (*villosum*); CG96*N3 (*furcatum*); 107a (*sarrachoides*); 107b (*nitidibaccatum*); 255 (*americanum*); 329 (*villosum*); 499 (*nitidibaccatum*); 2000*C (*villosum*).

Hanson, H.C. A-132 (*triflorum*); 469, 488 (*nigrescens*).

Hara, H. 1544, 63-03541, 15412 (*nigrum*).

Haradjian, M. 330 (*villosum*).

Hardham, C.B. 2882 (*douglasii*).

Hardial 528 (*americanum*).

Harding, D.P. 22 (*villosum*).

Hardison, L. 52 (*nigrescens*).

Harger, E.B. WES-467, 6903 (*emulans*).

Harker, M. 134 (*americanum*); 609 (*emulans*).

Harley, R.M. 17271, 22191, 22926, 27292 (*americanum*).

Harley, W.J. 621, 1029 (*scabrum*).

Harling, G. 209, 310, 372 (*americanum*); 5761 (*macrotonum*); 9142 (*americanum*); 13120 (*macrotonum*); 21596 (*americanum*); 22061 (*macrotonum*); 22194, 25021, 26240 (*americanum*).

Harmon, W.E. 2018, 2660 (*nigrescens*); 6138 (*macrotonum*).

Harms, V.L. 24800 (*emulans*); 39676 (*triflorum*).

Harper, K.T. 578 (*triflorum*); 631 (*emulans*).

Harper, R.M. 1519 (*nigrum*).

Harris, A.J. 2015-05 (*emulans*).

Harris, B.J. 1078 (*americanum*).

Harris, J.A. C-17450 (*americanum*); 27756 (*triflorum*).

Harris, K. 4 (*furcatum*).

Harris, S.K. 655 (*emulans*); 5401 (*americanum*); 9650, 10691, 11181, 11198, 11455, 11545, 11568 (*emulans*); 11569 (*nitidibaccatum*); 11640, 11800, 11841 (*emulans*); 11842 (*nitidibaccatum*); 11869, 11992, 13276 (*emulans*); 18574, 18714, 18924 (*nitidibaccatum*); 20746, 26089, 27090 (*emulans*); 31465 (*nitidibaccatum*).

Harris, T.M. 90 (*americanum*).

Harris, W. 8538, 11898 (*americanum*).

Harrison, B.F. 1221, 6248 (*americanum*); 10707 (*nitidibaccatum*).

Harrison, C.G. 502 (*americanum*).

Harrison, G.J. 8949 (*douglasii*).

Hart, J.A. 1283 (*americanum*).

Hart, R. 4013 (*americanum*).

Harter, L.L. 1 (*triflorum*).

Hartley, T. 855 (*emulans*).

Hartley, T.B. 1234 (*emulans*).

Hartman, R.L. 2409, 4928, 17435, 22991, 48636, 59600, 59688, 59861, 62707, 63683, 65118, 65436, 65767, 65943, 66069, 66409, 66612, 67613, 72358, 72430, 72684, 73292, 76208, 77409, 83392, 83604 (*triflorum*).

Hartweg, K.T. 202 (*americanum*); 207 (*nigrescens*).

Harvey, F.L. 701 (*emulans*).

Hashemi 12929E (*villosum*).

Hasse, H.E. 5233 (*nitidibaccatum*).

Hassler, É. 474, 622, 3104, 6400 (*americanum*); 10271 (*chenopodioides*); 12197 (*americanum*).

Hastings, G.T. 47, 337 (*furcatum*).

Hatschbach, G. 63010 (*americanum*); 71691 (*sarrachoides*); 72692 (*americanum*).

Hattum, H.J. van 4073, 4073 (*triflorum*).

Haughey, R.A. 157 (*douglasii*).

Haught, O. 138 (*americanum*); 5002, 5692 (*macrotonum*); 6590 (*americanum*).

Hausmann, F. von 126 (*nigrum*); 44, 183, 274 (*villosum*).

Hawkes, J.G. 80, 106 (*macrotonum*); 2504 (*douglasii*).

Hawkins, T. 690, 1763 (*nigrescens*).

Hay, W.R. 286, 463 (*villosum*).

Hayden, N.W. 105 (*emulans*).

Hayden, W.J. 3838, 3946, 5351, 5729 (*sarrachoides*).

Haynes, R.R. 4496 (*americanum*).

Haynie, N.V. 2943 (*emulans*).

Hayward, H.E. 551 (*interius*).

Hazel, C. 486 (*scabrum*).

Hazeltine, B.M. 1 (*interius*).

Hazlett, B.T. 2991, 3585, 3680, 3779, 4333 (*emulans*).

Hazlett, D. 3108 (*nigrescens*).

Hazlett, D.L. 9523 (*interius*).

Hazra, P. 24 (*villosum*).

Head, D. 170 (*retroflexum*).

Healy, A.J. 99/34 (*nitidibaccatum*); 68/159 (*villosum*); 59/283 (*nigrum*); 64/425 (*furcatum*).

Heap, J. SOLTR1&2 (*triflorum*).

Heath, I.B. 340A, 394 (*villosum*).

Heath, M. AM67, AM94, 540 (*nigrescens*).

Heckard, L.R. 2455 (*douglasii*).

Hedberg, O. 4965 (*villosum*).

Heddle, J. 273, 1151 (*emulans*).

Hedge, I.C. 3931, W4073, W4290 (*villosum*).

Heidel, B. 2083 (*triflorum*).

Hein, P. 7000a (*villosum*).

Heiser, C. 3417, 3627 (*nigrescens*).

Heiser, C.B. S128 (*americanum*); 3536 (*nigrescens*); 3597 (*macrotonum*); 3627 (*americanum*); 3754 (*nigrescens*); 5001 (*macrotonum*); 6098 (*americanum*).

Heithaus, E.R. 420 (*macrotonum*).

Heldreich, T. von 3206 (*nigrum*).

Heller, A.A. 632 (*nigrum*); 6572 (*furcatum*); 9458 (*triflorum*); 12208 (*nitidibaccatum*).

Heller, C. 151, 152, 395 (*douglasii*).

Heller, T.M. 289, 340 (*villosum*).

Helmkamp, E.A. 110 (*douglasii*).

Helmkamp, G.K. 14256 (*nigrum*); 18451 (*americanum*).

Hemming, C.F. 2226 (*villosum*).

Hemsley, J.H. L-79 (*nigrum*).

Henderson, G. 477 (*villosum*).

Henderson, L.F. 2495 (*triflorum*); 4592 (*americanum*); 5510 (*triflorum*).

Henderson, N.C. 91-54 (*emulans*); 92-336 (*sarrachoides*); 96-739 (*americanum*); 94-1183 (*emulans*).

Henderson, R.J.F. 123 (*retroflexum*); 128 (*chenopodioides*); 281, 288, 298, 299 (*americanum*); 301 (*chenopodioides*); 404, 405 (*nigrum*); 518, 523, 544, 559, 563, 564 (*americanum*); 1241 (*nitidibaccatum*); 1242, 1271, 1273, 1274 (*nigrum*); 1303, 1343, 1358, 1361, 1362, 1405, 1414, 1572, 2278, 2284 (*americanum*); 2314 (*nigrum*); 2607, 2791 (*americanum*); 3131 (*nigrum*).

Henderson, S. 98 (*chenopodioides*).

Hendrix, O.R. B-55 (*emulans*).

Hendrix, T.M. 583 (*nitidibaccatum*).

Heng, L. 10342 (*nigrum*).

Henning, W.H. 14 (*nitidibaccatum*).

Henrich, J.E. 450 (*americanum*).

Henrici, M. 3080 (*retroflexum*).

Henrickson, J. 10539, 11978 (*douglasii*).

Henriques, C. 53 (*villosum*).

Henry, A. 625, 1024, 3210, 8511, 9870 (*nigrum*).

Henry, A.N. 52 (*americanum*).

Henry, R.D. 5764 (*sarrachoides*).

Henshall, J.A. 197 (*triflorum*).

Henson, D. 990, 3200, 3283 (*emulans*).

Hepper, D.N. 40 (*macrotonum*); 88 (*nigrescens*).

Hepper, F.N. 1297 (*americanum*); 3109 (*villosum*); 4412, 4455, 4478, 4600, 4784 (*americanum*); 4823, 4837, 5500, 5614, 5628, 5649A, 5930 (*villosum*); 5984 (*americanum*); 6132, 6285, 6311, 6600, 6694, 7052 (*villosum*); 7453 (*americanum*); 7662, 7696 (*nigrum*); 7849 (*scabrum*); 8696, 8742 (*villosum*); 9760 (*nigrum*).

Herb. Déséglise 169 (*nigrum*).

Herb. Gadeceau 1006 (*villosum*); 5106 (*scabrum*).

Herb. Kew K225 (*nigrescens*).

Herb. Lacaita 11416, 11417 (*villosum*).

Herb. Miller 166 (*villosum*).

Herb. Richardson 121 (*americanum*).

Herb. Sampson 442A (*nigrum*).

Herb. Willdenow 4336 (*chenopodioides*).

Herbet, D. 745 (*americanum*).

Herbst, D. 6010, 6350, 6499, 6824, 6866 (*americanum*).

Heringer, E.P. 723, 4928, 15204 (*americanum*).

Hermann, F.J. 1273 (*emulans*); 4912, 12150 (*triflorum*).

Hermann, H.A. van 527 (*nigrescens*); 527, 633 (*americanum*).

Hermann, M. 417 (*chenopodioides*).

Hernández A, C. 90 (*nigrescens*).

Hernández del Olmo, J. 356 (*nigrescens*).

Hernández Gutiérrez, E. 64 (*nigrescens*).

Hernández M, M. 5473 (*americanum*).

Hernández Magaña, R. 958 (*pruinosum*); 1637 (*nigrescens*); 3752 (*corymbosum*); 4365, 5257, 5267[b] (*nigrescens*); 5281 (*douglasii*); 5524 (*nigrescens*); 5559 (*douglasii*); 5826, 5875, 6143, 6143 (*nigrescens*); 6436 (*corymbosum*); 6593 (*douglasii*); 6717 (*americanum*); 6922, 6971, 7280, 8153 (*nigrescens*); 9218 (*americanum*); 9307 (*nigrescens*); 9489, 9497 (*douglasii*).

Hernández Najarro, F. 1250 (*nigrescens*).

Hernández Ortega, R. 62 (*americanum*).

Hernández P, L.A. 186 (*nigrescens*).

Hernández R, J. 5262 (*douglasii*); 5334 (*americanum*).

Hernández S, M. 108, 707 (*macrotonum*).

Hernández X, E. X-30 (*nigrescens*).

Hernández, A. 35 (*nigrescens*).

Hernández, C. 63, 153, 240 (*americanum*).

Hernández, J. 91 (*macrotonum*).

Hernández, L. 1228 (*nigrescens*).

Hernández, M.R. 4365 (*nigrescens*).

Hernández, R. 835 (*nigrescens*); 840323-23 (*americanum*); 870410-7 (*nigrescens*); 870410-14, 870506SR.15 (*americanum*).

Hernández-Ramos, J.F. 30 (*nigrescens*).

Herrera C, M. 83 (*nigrescens*).

Herrera Castro, N. 89 (*nigrescens*).

Herrera, A. 26 (*nigrescens*); 438 (*americanum*).

Herrera, E. 864 (*macrotonum*).

Herrera, G. 6063 (*nigrescens*).

Herriot, W. 78428 (*triflorum*).

Herter, W.G. 186 (*chenopodioides*); 17039 (*sarrachoides*); 17060, 70323 (*chenopodioides*).

Herwitz, S.R. 206 (*pseudogracile*).

Hespenheide, H.A. 529 (*nigrescens*); 932 (*americanum*).

Hess, W. 9290 (*emulans*).

Hess, W.L. 109 (*triflorum*).

Hesse, V.F. 3190 (*nigrum*).

Heyligers, P.C. 88025 (*chenopodioides*); 88050 (*americanum*); 88051 (*chenopodioides*); 89016, 92010, 94009, 95003 (*americanum*); 95004 (*chenopodioides*); 95005 (*americanum*); 95006 (*chenopodioides*); 99003 (*americanum*); 99004 (*chenopodioides*); 99005, 99006 (*americanum*).

Heywood, V.H. 955 (*nigrum*).

HFC (Herbario de Flora de Cuba) 43449 (*americanum*).

HGT 754 (*villosum*).

HHV FLSP759, FLSP1723a, 03570 (*americanum*); 04175 (*corymbosum*).

Hicks, G.H. 256 (*americanum*).

Hieronymus, G. 710 (*triflorum*).

Higgins, D.M. 1016 (*nigrum*).

Higgins, L.C. 757 (*americanum*); 5798 (*triflorum*); 8309 (*emulans*); 9103 (*nitidibaccatum*); 9116, 10569 (*triflorum*); 10693 (*nitidibaccatum*); 11392 (*interius*); 15979 (*nitidibaccatum*); 19750 (*nigrum*); 24010 (*nitidibaccatum*).

Hikmat Abbas 616 (*nigrum*).

Hildebrandt, J.M. 865 (*villosum*); 1626[B], 3110, 3401a (*scabrum*); 3796 (*americanum*); 34014a (*scabrum*).

Hill, A.F. 1657 (*emulans*).

Hill, S.R. 945 (*emulans*); 11711 (*triflorum*); 11924 (*nitidibaccatum*); 12131 (*triflorum*); 12182 (*interius*); 12641 (*pseudogracile*); 14561 (*triflorum*); 14831 (*nitidibaccatum*); 14851 (*interius*); 17081, 19867, 22538, 26155 (*emulans*).

Hilliard, O.M. 15180 (*retroflexum*); 15537 (*chenopodioides*).

Hills, O.A. 5 (*triflorum*).

Him, C. 32 (*nigrescens*).

Hinchinsbrooke, [?] 69 (*villosum*).

Hinckley, L.C. 51, 168, 991 (*douglasii*); 1923, 2041, 2208, 46819, 48819 (*nigrescens*).

Hingley, M.R. 168 (*villosum*).

Hinton, G.B. 399 (*nigrescens*); 1100 (*douglasii*); 2917, 3855, 5709, 7566 (*nigrescens*); 9288, 11898 (*douglasii*); 12976 (*nigrescens*); 15638 (*douglasii*); 16619, 17607, 17697 (*nigrescens*).

Hipe, N.T. 1129 (*nigrum*).

Hislop, A. Z-155 (*retroflexum*).

Hislop, M. 2026 (*americanum*).

Hitchcock, A.E. 1343 (*triflorum*).

Hitchcock, A.S. 239 (*americanum*); 361 (*triflorum*); 362a (*interius*); 958 (*triflorum*); 13016 (*emulans*); 13679 (*americanum*); 15746 (*emulans*); 19974, 20357 (*americanum*).

Hitchcock, C.L. 2449 (*triflorum*); 6224 (*americanum*); 19660 (*triflorum*); 23063 (*douglasii*).

Hitchcock, M. 2196 (*emulans*).

Hjerting, J.P. 645 (*americanum*).

Hmama, H. 894 (*nigrum*).

Hoare, A. 16 (*americanum*); 125 (*nigrum*).

Hobbs, J.F.F. 13297 (*nitidibaccatum*).

Hocking, G.M. 13 (*emulans*).

Hodgdon, A.R. 2409, 4226 (*emulans*); 16160, 18273 (*nigrum*).

Hodge, J.B.S. D.4288 (*triflorum*).

Hodge, W.H. 799 (*americanum*).

Hodges, M. 96, 97 (*americanum*).

Hodgkin, H.T. 23, 82 (*scabrum*).

Hodgson, W.C. H-434, H-954, H-1190, H-1513, H-1628, H-1990, 2152, 2161, 2161, H-2619, 5403, 6671, 8537, 9677, 13270, 17433, 20128, 21014, 21033, 21105, 21105, 28481 (*douglasii*).

Hoehne, W. SPF-12502, JPB17122, JPB17127 (*americanum*).

Hoenicka, [?] 55 (*nigrescens*).

Hoenicka, H. 56 (*nigrescens*).

Hoff, M. 6245 (*americanum*).

Hoffmann, W.A. 245 (*americanum*).

Hofmeister, W. [42] (*villosum*).

Hohenacker, R.F. 768 (*nigrum*); 1077 (*americanum*).

Hojra, P.K. BSHC-37 (*americanum*).

Holdridge, L.R. 1139 (*americanum*).

Hollermayer, A. 42 (*furcatum*).

Holliday, L.G. 9 (*americanum*).

Holm, R.W. 164 (*macrotonum*).

Holm-Nielsen, L.B. 2027, 2048, 2735 (*americanum*); 3462, 3547, 5831, 6206, 6773, 6798, 6813 (*macrotonum*); 18830 (*americanum*); 26412, 26412 (*macrotonum*); 27946, 27985, 28899 (*americanum*).

Holmberg, N.J. 227, 1950, 2038, 2085, 2121A, 2248 (*emulans*); 2309 (*sarrachoides*); 2317, 2359, 2371, 2462, 2497, 2696, 2722 (*emulans*); 2730 (*triflorum*); 4389, 5058 (*emulans*).

Holmgren, I. 33 (*americanum*); 480 (*macrotonum*).

Holmgren, N.H. 6766 (*douglasii*); 7163, 7304 (*triflorum*); 9756 (*emulans*); 11049, 11570, 12923, 14516 (*triflorum*); 15073 (*emulans*); 15471 (*nitidibaccatum*); 16155, 16430 (*triflorum*).

Holst, B.K. 6027 (*americanum*).

Holt, E.A. 7624 (*triflorum*).

Holt, E.G. 177 (*nigrescens*).

Holte, K.E. 33 (*triflorum*).

Holway, E.W.D. 63 (*furcatum*).

Hoogland, R.D. 8720, 11308 (*americanum*).

Hooker, J.D. s62[?], 639 (*villosum*).

Hooper, D. 39413 (*americanum*).

Hooper, J. 37 (*emulans*).

Hooper, S.S. 2014 (*villosum*).

Hoover, R.F. 52 (*americanum*); 663 (*nitidibaccatum*); 5235 (*douglasii*).

Hoover, W.S. Deden89 (*scabrum*); 1583 (*macrotonum*); 30037 (*nigrum*); 30064 (*scabrum*).

Hoover-Green 129 (*emulans*).

Hopa, T.H. NC-87129 (*americanum*).

Hopkins, M. 408 (*emulans*); 821 (*interius*).

Hörandl, E. 26365 (*nigrum*).

Horiuchi, K. FOK-057483 (*nigrum*).

Hornby, H.H. 350 (*villosum*).

Horner, R.M. B-374 (*nitidibaccatum*).

Horr, W.H. 3898 (*interius*).

Horton, J.S. 143 (*douglasii*).

Horton, P. 56301 (*nigrum*).

Hosaka, E.Y. 3112 (*americanum*).

Hoshino, T. 9666069 (*villosum*).

Hosking, J.R. 888, 959, 2117, 3091, 3496 (*chenopodioides*).

Hosseus, C.C. 147 (*nitidibaccatum*).

Hostmann, F. 992 (*americanum*).

Hostmann, F.W.R. 992 (*americanum*).

Hotchkiss, N. 5719 (*emulans*); 6821 (*triflorum*).

Houghton, H.W. 3526, 3774, 38521/2 (*emulans*).

House, P. 1630 (*nigrescens*).

House, P.R. 1502, 1511 (*nigrescens*).

Howard, J. 52 (*nigrescens*).

Howard, R.A. 6558, 8506, 9640 (*americanum*); 15903 (*emulans*).

Howell, J.T. 41381 (*americanum*); 41390 (*nitidibaccatum*); 45872 (*furcatum*); 49797 (*triflorum*); 50146A (*nitidibaccatum*).

Howell, T. 232 (*nitidibaccatum*).

Howell, T.J. 333 (*nitidibaccatum*).

Hsiao, T.H. 102 (*douglasii*); 104, 120 (*nigrescens*).

Hu, [?] 972 (*nigrum*).

Hu, S.Y. 19992 (*nigrum*); 22241 (*americanum*).

Huang, T.C. 1953 (*americanum*).

Huapalla, J. 001407, 002496, 2550, 2935, 03259 (*corymbosum*).

Huashikat, V. 52, 206 (*americanum*).

Hubbard, C.E. 2508 (*americanum*); 13261, 13262, 13263 (*nitidibaccatum*).

Huber, A. 4193 (*triflorum*).

Hucks, M. 627 (*villosum*).

Hudson, H.S. 116 (*emulans*); 148 (*sarrachoides*); 354 (*emulans*).

Hudson, J. 964 (*americanum*).

Hudson, W.D. 306 (*macrotonum*).

Huertas P, G. 5918 (*macrotonum*).

Huft, M.J. 2192 (*nigrescens*).

Hügel, A. 1723 (*americanum*).

Hugh 72/28 (*villosum*).

Hughes, C. 179 (*emulans*).

Hughes, E.L. 32 (*douglasii*).

Huidobro, A.M. 1410 (*triflorum*).

Huk, A. 57 (*americanum*).

Hukui, K. 26 (*villosum*).

Hull, H. 68 (*chenopodioides*).

Humbert, H. 1244, 1375, 6671 (*scabrum*); 11308 (*americanum*); 11309, 12191 (*scabrum*); 13124 (*americanum*); 14849 (*retroflexum*); 18679 (*scabrum*); 19091, 19707, 20182bis (*americanum*); 26180 (*macrotonum*); 31295 (*douglasii*).

Humbles, J. 5014 (*villosum*).

Humphries, C.J. 206 (*villosum*); 334 (*nigrum*).

Hundt, O. 797, 798 (*scabrum*).

Hunkins, S. 524 (*douglasii*).

Hunn, E. OAX-50 (*douglasii*); OAX-205 (*americanum*); OAX-668 (*nigrescens*); 765 (*triflorum*); OAX-793 (*americanum*); OAX-838, OAX-1056, OAX-1383 (*nigrescens*); OAX-1457 (*douglasii*); 1849 (*nigrescens*).

Hunnewell, F.W. 4942 (*emulans*); 9038, 10490 (*pseudogracile*); 10821 (*triflorum*); 17996 (*emulans*).

Hunter, R. 20 (*americanum*).

Hunziker, A.T. 614 (*sarrachoides*); 1624, 3301 (*chenopodioides*); 5548 (*americanum*); 8829 (*nitidibaccatum*); 9781, 11629 (*triflorum*); 11868 (*nitidibaccatum*); 12544, 13037 (*triflorum*); 13991, 14078, 14114, 14126 (*nitidibaccatum*); 14398, 15028 (*triflorum*); 19552 (*americanum*); 20141 (*triflorum*); 23521 (*americanum*); 24228, 24785 (*sarrachoides*); 24805, 24807 (*triflorum*); 25322 (*nitidibaccatum*).

Hunziker, J.H. 1624 (*americanum*); 3816 (*chenopodioides*); 3847, 7254, 11022 (*americanum*).

Huq, A.M. 10557 (*americanum*).

Hurie, E.T. 60 (*emulans*).

Hurrell, J.A. 6037, 6439 (*chenopodioides*).

Hurworth, W.E. 35 (*nigrum*).

Hutchinson, J. 3767 (*retroflexum*).

Hutchison, P. 3603 (*corymbosum*).

Hutchison, P.C. 2515 (*douglasii*); 3458 (*americanum*); 3603 (*corymbosum*); 3853 (*americanum*).

Huttel, C. 599 (*americanum*).

Hutton, D. N13413 (*nitidibaccatum*).

Hyacinth, F. 1575 (*emulans*).

Hynniewta, T.M. BSHC-31971 (*americanum*).

Hyypio, P.A. 3495 (*emulans*).

Ibarra C, G. 861 (*nigrescens*).

Ibarra Manríquez, G. 3189 (*americanum*).

Ichikawa, K. 33 (*nigrum*).

Idjan 63 (*americanum*).

Idjan, [?] 63 (*americanum*).

Idrobo, J.M. 3230 (*macrotonum*).

Ignacio, D. 4 (*americanum*).

Iizuka, M. 41 (*nigrescens*).

Ikabanga, D. 110 (*americanum*).

Ikegami, Y. 15792 (*nigrum*).

Illin, N. 273 (*nitidibaccatum*).

Illsey G, C. 1297 (*americanum*).

Iltis, H.H. 55 (*nigrescens*); 244 (*douglasii*); 355, 388 (*americanum*); 446, 721 (*douglasii*); 744, 1554, 1593 (*americanum*); 3005 (*nigrescens*); 4313 (*triflorum*); 5215 (*sarrachoides*); 5617, 6219, 6845, 8008, 8183, 8205, 8294, 8465, 9330, 9913, 10443, 13797, 13951, 14131, 17030, 20815, 20855, 24094 (*emulans*); 29602a (*nigrescens*); 30225 (*emulans*); 30226 (*nitidibaccatum*).

Imaguire, N. 105, 317 (*americanum*).

Imle, E.P. 307, 308, 309 (*nigrescens*).

Ináyat Khan 19969 (*villosum*); 22443 (*nigrum*).

Ingall, R.G. 005 (*nigrum*).

Ingoldby, C.M. 553 (*villosum*).

Inostroza, [?] CONC-35154 (*furcatum*).

Inzunza, F.M. 128 (*pruinosum*).

Ireland, O.L. 1552 (*triflorum*).

Irvine, F.R. 27 (*americanum*); 93, 3607, 5095, 5096, 5191 (*scabrum*).

Irwin, H.S. 2069, 5865 (*americanum*); 9151 (*chenopodioides*); 9186, 10990, 13755, 17893, 18095, 18123, 19170, 19812a, 20328, 20958, 23142, 23766, 24177, 28500, 29614, 30413a, 34909, 55947 (*americanum*).

Irwin, J.J. 8764 (*triflorum*).

Isern, J. 6302 (*americanum*).

Isidro V, M.A. 764 (*nigrescens*).

Islip, S. 44 (*nigrum*).

Islomov, B. UPL-00304 (*nigrum*).

Ivens, G.W. 2503 (*villosum*).

Iwatsuki, K. t-10367 (*americanum*).

Iwen, M.M. 181 (*emulans*).

Izabella 200 (*americanum*).

Izuzquiza, A. 1082AI (*villosum*).

Jacintho, S.F. 2 (*americanum*).

Jackson, E.N.S. 5976 (*nitidibaccatum*).

Jackson, J.K. 2887, 4187 (*villosum*).

Jacob Salinas, M.E. 75 (*douglasii*).

Jacobs, M. 8533 (*retroflexum*).

Jacobs, S.W.L. 420, 767 (*americanum*); 2834 (*chenopodioides*).

Jacobson, J. 5 (*emulans*).

Jacquemont, M.M. 783, 937 (*villosum*).

Jacques-Georges, A. 26547 (*scabrum*).

Jaeschke, H. 304 (*villosum*).

Jafei, S.M.H. 1173 (*nigrum*).

Jaimes, M.S. 10 (*americanum*).

Jalas, J. 5117, 5155 (*nigrum*).

Jallu, J. 1038 (*nitidibaccatum*).

James, L.E. 1253, 1929, 1930 (*emulans*).

James, T. 29 (*americanum*).

Jameson, W. 96, 153 (*americanum*).

Janaki Ammal, E.K. 1523 (*villosum*).

Janardhanan, K.P. NC-46338, NC-46372, NC-52836, NC-77525 (*villosum*).

Jangoux, J. 166 (*americanum*).

Jansen, J.W.A. 1193 (*americanum*); 2015 (*scabrum*).

Jansen, P.C.M. 1644 (*villosum*); 8065 (*americanum*).

Jansen, R.K. 499 (*americanum*); 531 (*nigrescens*).

Jansen-Jacobs, M.J. 5503 (*americanum*).

Japp, R.N. 362 (*americanum*).

Jara, A. 483 (*americanum*).

Jara-Muñoz, A. 287 (*americanum*).

Jaramillo, J. 8183 (*macrotonum*); 8237 (*nigrescens*); 8786 (*macrotonum*); 13151, 15214, 20940 (*americanum*); 26699 (*macrotonum*); 26870 (*americanum*).

Jarquin L, E. 22 (*americanum*).

Jarrett, T. 454 (*villosum*).

Jarvis, C.E. 46 (*nigrum*).

Jasso, M.J. 1765 (*nigrescens*).

Játiva, C.D. 153 (*americanum*).

Jayakar, A.S.G. 41 (*villosum*).

Jayasuriya, A.H.M. 2228 (*americanum*).

Jayaweera, D.M.A. 1043 (*nigrum*).

Jeanes, J.A. 2662 (*furcatum*).

Jeffery, G.W. K480 (*villosum*).

Jeffrey, C. 522, 2326 (*americanum*); 2521 (*nigrescens*).

Jelski, C. de 49 (*americanum*).

Jenke, D. 17 (*americanum*).

Jenman, G.S. 4510, 4573, 5131 (*americanum*).

Jennings, E.A. 20 (*nigrum*).

Jensen, H.A. 37 (*douglasii*).

Jensen, N. 2470 (*douglasii*).

Jepson, W.L. 2618, 5559 (*douglasii*); 7448 (*americanum*); 8452, 8806, 8919, 9119 (*douglasii*); 10256 (*furcatum*); 10271 (*americanum*); 11911 (*douglasii*); 16911, 17889 (*americanum*); 19089, 19090 (*douglasii*); 19990 (*triflorum*); 20160 (*douglasii*); 21251, 21253, 21254, 21255 (*americanum*); 21256 (*douglasii*).

Jermy, A.C. 9070, 9239 (*nigrum*); 9805 (*villosum*).

Jermy, G. 79 (*nigrescens*); 199 (*emulans*).

Jermyn, S.T. 42, 338 (*nigrum*).

Jerónimo 6, 279 (*nigrum*).

Jiménez Chimil, M. JDA-30238, JDA-30419 (*nigrescens*).

Jiménez E, N.D. 315 (*americanum*).

Jiménez Flores, J. 210 (*nigrescens*); 577 (*douglasii*).

Jiménez M, Q. 1188 (*americanum*).

Jiménez S, F. 207 (*douglasii*).

Jiménez V, J.E. 568 (*nigrescens*).

Jiménez, B. 107BJ (*chenopodioides*); 1339 (*americanum*).

Jiménez, J. 245 (*douglasii*).

Jiménez, Q. 1188 (*americanum*).

Jiménez, R. 153 (*nigrescens*).

Jirbedi, G.N. Pharm-505 (*villosum*).

Jobson, P.C. 1037 (*chenopodioides*).

Johannsen, P.L. 254, 331 (*douglasii*).

John, T. 945 (*triflorum*).

Johns, T.J. 86-481 (*scabrum*).

Johnson, F.W. 1263 (*triflorum*); 2044 (*emulans*).

Johnson, J.C. 16/38M (*nigrescens*).

Johnson, L. 1272 (*americanum*).

Johnson, L.A.S. 49, 78, 1272 (*americanum*); 23530 (*triflorum*).

Johnson, R.E. 32755 (*emulans*).

Johnston, E.L. 144a (*triflorum*); 672, 760B, 760 (*interius*).

Johnston, I.M. 1812 (*nitidibaccatum*); 2102 (*americanum*).

Johnston, M.C. 12266, 542316 (*nigrescens*).

Johnstone, R. 2311 (*americanum*).

Joly, A.B. JPB17125 (*americanum*).

Jones, G.N. 323 (*triflorum*); 12297, 12926, 13016, 17353 (*emulans*).

Jones, J. 1 (*americanum*).

Jones, M.E. 605 (*interius*); 1485, 4009, 5464h, 5475 (*triflorum*); 24119, 27342 (*americanum*).

Jones, N. 22375 (*emulans*).

Jones, P. 921 (*pseudogracile*).

Jones, R.F. 48 (*villosum*).

Jones, S. 1502, 2031 (*douglasii*).

Jones, W.T. 238(h), 3317 (*americanum*).

Jones, W.W. 234 (*triflorum*); 445 (*nitidibaccatum*).

Jönsson, G. 893a (*americanum*).

Jordan, H.D. 1059 (*scabrum*).

Jörgensen, P. 91, 114 (*americanum*); 529 (*triflorum*); 3340 (*sarrachoides*).

Jorgensen, P.M. 56275 (*americanum*); 56403, 61466 (*macrotonum*).

Joseph, J. 15280, 39848, 48402 (*americanum*).

Jouvin, P.P. 179, 533 (*americanum*).

Joyal, E. 1558 (*douglasii*); 1588 (*americanum*).

Joye, G.F. 219 (*nigrescens*).

Juárez G, L.G. 44 (*nigrescens*).

Judziewicz, E. 2449, 9928, 12458, 13890, 14101 (*emulans*).

Junak, S.A. WA-22 (*douglasii*).

Juncosa, A. 751, 2220 (*americanum*).

Junge, C. 1271, 2611, 2862, 3083, 3087 (*furcatum*).

Jungmann, M.K. 22 (*americanum*).

Jungner, J.R. 1375 (*nitidibaccatum*).

Junk, W.J. 245 (*americanum*).

Junod, H.A. 575 (*scabrum*).

Jury, S.L. 12606, 12717 (*villosum*); 13040 (*nigrum*); 13276, 15047 (*villosum*); 16503, 17766 (*nigrum*); 17823, 19031, 20761 (*villosum*).

Jørgensen, P.M. 56275 (*americanum*).

Kahatt Soto, N. 156 (*americanum*).

Kahn, F. 2980 (*americanum*).

Kairo, A. 60, 10641 (*americanum*).

Kalheber, H. 78-530 (*nigrum*); 88-2907 (*nitidibaccatum*).

Kamau, W.M. 734 (*villosum*).

Kao, H.S. 4696 (*nigrum*).

Kappler, A. 1663 (*nigrescens*).

Karwinski, W.H. 577 (*nigrescens*).

Kässner, L.C.T. 434 (*americanum*).

Kaune, S.M. 616 (*americanum*).

Kaur, U. 16 (*americanum*).

Kausel, E.M.L. 3236, 3260, 4554, 4655 (*furcatum*).

Kayambo, E.J. 54 (*americanum*).

Kayap, R. 1182, 1436 (*americanum*).

Kaye, T.N. 1152 (*triflorum*).

Kayombo, C.J. 3225 (*villosum*).

Kearney, T.H. 1818 (*emulans*); 2267 (*pseudogracile*); 6249 (*americanum*).

Keck, D.D. 2970 (*douglasii*).

Keefe, J.M. 30944 (*douglasii*).

Keener, B.R. 2347 (*emulans*).

Kegler, A. 1511 (*americanum*).

Keighery, B.J. 754, 755 (*americanum*).

Keighery, G.J. 1282 (*nigrum*); 1722, 5018, 8887, 9645, 11071, 15045, 16223, 16460 (*americanum*).

Keil, D. 13323 (*nigrescens*); 29577 (*douglasii*).

Keil, D.J. 1152, 3513 (*douglasii*).

Keiran, P. 438 (*emulans*).

Keith, H.G. 352, 384, 419 (*nigrum*); 1032 (*villosum*); 1033 (*nigrum*); 1036 (*villosum*).

Kelaole, E.T. A81 (*villosum*).

Kelch, C.G. 40.163 (*americanum*).

Kellman, M. ANU-1745 (*americanum*).

Kellogg, A. 716a (*douglasii*); 716b (*americanum*).

Kellogg, J.H. 490, 1990, 25993 (*emulans*).

Kellogg, W. 3 (*douglasii*).

Kendrick, L. 64 (*villosum*).

Kenneally, K.F. 6095 (*nigrum*).

Kennedy, H.A. 2839 (*nigrescens*).

Kennedy, P.B. 1720 (*triflorum*).

Kerber, E. 177 (*nigrescens*).

Kercher, P. AL.137 (*villosum*).

Kerfoot, O. 3547 (*villosum*).

Kern, J.H. 4610 (*triflorum*); 14998 (*nitidibaccatum*).

Kerr, A.F.G. 403, 1231 (*nigrum*); 3500 (*americanum*); 4567 (*nigrum*).

Kerr, F.H.W. 2436A (*villosum*).

Kerstan, G. 2016 (*villosum*).

Kesharanandi 720 (*villosum*).

Kessler, M. 18 (*americanum*).

Kessler, P.J.A. 3103 (*americanum*).

Keytel, P.C. 1807 (*nigrum*).

Khan, R. 23 (*americanum*); 352 (*nigrescens*); 785 (*macrotonum*); 899 (*nigrescens*); 1000 (*americanum*).

Khanna, K.K. GC-39916 (*villosum*).

Kharkevich, S. 686 (*nigrum*).

Khutsishvili, M. 7, 172 (*nigrum*).

Kiesling, R. 6500, 6562 (*triflorum*).

Killias, O.L. 6922 (*interius*).

Killip, E.P. 2001 (*macrotonum*); 9744 (*nigrescens*); 13313 (*emulans*); 14060 (*americanum*); 15569, 16681, 16970 (*macrotonum*); 17011 (*nigrescens*); 18090, 18132 (*macrotonum*); 18490 (*nigrescens*); 18731, 18896, 18956, 19920, 20011, 20723 (*macrotonum*); 20795, 20911 (*nigrescens*); 22970, 23424, 24058, 25218, 28827 (*americanum*); 32091 (*pseudogracile*); 41162, 41582 (*americanum*); 42225 (*emulans*).

Kindeketa, W. 2549 (*scabrum*).

King's collector 550 (*nigrum*); 566 (*americanum*).

King, A. 146 (*emulans*).

King, C.B. 401 (*emulans*).

King, D.O. 24 (*triflorum*); 266 (*chenopodioides*); 401B (*furcatum*); 401A (*nitidibaccatum*); 535 (*chenopodioides*); 537 (*triflorum*).

King, R.A. 308 (*villosum*).

King, R.M. 330 (*nigrescens*); 452 (*douglasii*); 2825 (*nigrescens*); 3033 (*douglasii*); 3895, 3980, 4087, 4259 (*nigrescens*); 4521 (*douglasii*).

Kingdon-Ward, F. 17085, 20022 (*nigrum*); 20240 (*americanum*).

Kings, W. C.B.65 (*americanum*).

Kinoshita, L.S. 33 (*americanum*).

Kirizawa, M. 1048 (*americanum*).

Kirkbride Jr, J.H. 1398 (*nigrescens*); 1597 (*americanum*); 1853 (*macrotonum*); 2143 (*americanum*); 2314 (*macrotonum*); 2564 (*nigrescens*); 2684 (*americanum*).

Kishler, J. 213, 632 (*douglasii*).

Kissane, J.C. 709 (*chenopodioides*).

Kitaibel, P. 349 (*villosum*).

Klawe, W.L. 1465, 1466 (*douglasii*).

Klein, V.L.G. 700, 1192 (*americanum*).

Klevens, M.J. 17 C318 (*nigrescens*).

Klitgaard, B.B. 783, 807 (*macrotonum*); 1405 (*americanum*).

Knapp, S. 824 (*macrotonum*); 1181, 1808 (*americanum*); 2124, 3951, 4655 (*nigrescens*); 7626a, 7627, 7738, 7802 (*americanum*); 9052 (*macrotonum*); 9054 (*americanum*); 9838 (*nigrum*); 9997 (*nigrescens*); IM-10079 (*chenopodioides*); IM-10095 (*nigrum*); 10112 (*americanum*); 10143 (*nigrum*); 10145 (*nigrescens*); IM-10161 (*americanum*); IM-10162, IM-10163, IM-10164, IM-10165, IM-10166, IM-10167 (*nigrum*); 10205, 10206, 10210, 10294, 10302, 10360 (*americanum*); 10488, 10504, 10513 (*triflorum*); 10579 (*americanum*); 10603 (*corymbosum*); 10608 (*americanum*); 10634, 10637 (*corymbosum*); 10648 (*americanum*); 10677 (*villosum*); 10690, 10754 (*americanum*); 10780 (*villosum*); IM-10782 (*triflorum*); 10785 (*villosum*); IM-10786 (*nigrum*); IM-10787 (*americanum*); IM-10788 (*nigrum*).

Knight, D.H. 756 (*americanum*); 1062, 1145 (*macrotonum*).

Knobloch, I.W. 5498 (*douglasii*).

Knoke, D. 1738 (*triflorum*).

Knopf, E.C. 65-236, 65, 180, 188-236, 188, 198, 220, 236, 239, 278 (*douglasii*).

Knowlton, C.H. 737 (*emulans*).

Kobakhidze, L. 1009 (*nigrum*).

Kobetich, G.C. 68-161 (*douglasii*).

Koch, R.G. 1736, 1781, 1830, 2255, 2297, 2479 (*emulans*); 2714 (*nigrescens*); 3959 (*emulans*).

Koch, S.D. 89-113 (*douglasii*).

Kodela, P.G. 577 (*americanum*); 578 (*chenopodioides*).

Koelz, W. 11622 (*villosum*); 12172 (*nigrum*); 13217 (*villosum*); 13219, 13911 (*nigrum*); 14269, 14583 (*villosum*).

Koeppen, R. 97 (*emulans*).

Koga, T. 146 (*americanum*).

Kohn, E. 1469 (*nigrescens*).

Köie, M. 2897 (*villosum*); 3127 (*nigrum*).

Kolenati, F.A.R. 2057 (*nigrum*).

Komaromi, R. 48, 61 (*scabrum*).

Komarov, V. 1376 (*nigrum*).

Koning, J. de 519 (*scabrum*); 715, 1743 (*americanum*); 1747 (*scabrum*); 7088 (*americanum*).

Konta, F. 3309 (*americanum*).

Koponen, A. 16146 (*nigrum*).

Koponen, T. 15834 (*nigrum*); 16522 (*americanum*).

Korhonen, M. 1077, 1108 (*villosum*).

Koritschoner, H. 2191 (*scabrum*).

Kotschy, C.G.T. 166[c], 184[b], 184[c], 313, 355 (*villosum*).

Kotschy, K.G.T. 313, 446, 622 (*villosum*).

Kotter, M. 25MK (*triflorum*).

Koyama, H. 7669 (*nigrum*).

Koziol, E. 1635 (*nigrum*).

Krajina, V.J. 660622035 (*americanum*).

Kral, R. 1905 (*pseudogracile*); 37434 (*nigrescens*); 38204 (*americanum*); 44479, 44963 (*emulans*); 48060 (*pseudogracile*); 48421 (*emulans*); 49252 (*pseudogracile*); 51310 (*emulans*); 51349 (*americanum*); 51804 (*pseudogracile*); 53519 (*americanum*); 56564, 59247 (*pseudogracile*); 68065 (*americanum*).

Kranz, W.M. 93 (*americanum*).

Krapovickas, A. 2729 (*triflorum*); 2824, 3275 (*chenopodioides*); 4348 (*triflorum*); 6036 (*nitidibaccatum*); 20006 (*americanum*); 22308 (*triflorum*); 25161 (*americanum*); 26156, 27529, 27720 (*chenopodioides*); 44650 (*americanum*); 47749 (*sarrachoides*).

Krause, L. 139 (*americanum*).

Kress, W.J. 94-4666, 94-4995 (*americanum*); 94-4999 (*nigrescens*); 04-7514 (*emulans*); 944285 (*macrotonum*).

Kriebel, R. 195 (*americanum*); 503 (*nigrescens*); 965 (*macrotonum*); 2403, 5785 (*nigrescens*).

Krieger, L. 3311, CESJ7199, CESJ15365, 19452 (*americanum*).

Krishna, B. BSHC-851 (*nigrum*); BSHC-1526 (*americanum*).

Krishnan, E.K. 30 (*nigrum*).

Krivda, W. V-338, V-343 (*triflorum*); 1843 (*nigrum*); 2185 (*emulans*); 2185[b] (*nigrum*).

Krotkov, P. 9376 (*emulans*).

Krotkov, P.V. 7759 (*emulans*).

Krug, B. 7450 (*nigrum*).

Krüger, H. 700 (*triflorum*).

Kruse, H. 1028 (*americanum*); 1656 (*douglasii*).

Kubo, R.R. 101 (*americanum*).

Kucera, D. 171 (*nigrum*).

Kufer, J. 83 (*nigrescens*).

Kuhbier, H. 51 (*americanum*); 179 (*nigrescens*).

Kuhlmann, J.G. 2602 (*americanum*).

Kujikat, A. 457 (*americanum*).

Kukkonen, I. 6656 (*nigrum*); 7203, 7617, 7657, 7836, 8166 (*villosum*); 8525, 9677, 10731 (*nigrum*).

Kumar, K.R. 3350 (*americanum*).

Kumar, S. ANC-26937, ANC-26968, NC-81210, NC-92384 (*americanum*); NC-93367, NC-101615 (*nigrum*).

Kumar, V. 731 (*nigrum*).

Kummer, F. 5811 (*villosum*).

Kuniyoshi, Y.S. 74, 120, 327 (*americanum*).

Kunstler, H. 566 (*americanum*).

Kuntze, C.E.O. 234 (*americanum*); 3018 (*triflorum*).

Kuntze, O. 1657, 2295 (*nigrescens*).

Kunz, H. 36, 241 (*emulans*).

Kurbanov, D. 1606 (*nigrum*).

Kurosawa, T. 3149 (*nigrum*).

Kurtto, A. 3029 (*villosum*).

Kurtz, F. 4612, 4707, 5534b (*triflorum*); 6994a (*nitidibaccatum*); 14205 (*triflorum*).

Kurwari, G.R. 12959 (*americanum*).

Kurz, S. 201 (*nigrum*).

Kusmin, J.D. 9, 98 (*emulans*).

Kwatha, R.B. 210 (*scabrum*).

la Cruz Bolaños, A. de Adec-12 (*douglasii*).

La Duke, J. 584 (*nigrescens*).

La Rivers, I. 767 (*triflorum*).

la Sota, A. de 4577 (*americanum*).

La Torre, M.I. 417, 426 (*corymbosum*).

La Touche, J. de 512 (*nigrum*).

Labat, J.N. 415, 894, 1138 (*douglasii*).

Lace, J.H. 580 (*nigrum*); 3394, 4034 (*villosum*).

Lachashvili, N. 1050 (*nigrum*).

Lackschewitz, K.H. 7665, 8150, 8264 (*triflorum*).

Ladd, D. 22294 (*sarrachoides*).

Ladell, W.R.S. 263 (*nigrum*).

Laferrière, J.E. 561 (*douglasii*).

Laferriere, J.E. 1089 (*douglasii*).

Laferrière, J.E. 1226, 1958 (*douglasii*).

Lagacé, M. 751008-0757 (*emulans*).

Lahitte, R. 293 (*chenopodioides*).

Lai, M.J. 11694 (*americanum*).

Lai, P. NC-63043 (*americanum*).

Lakela, O.K. 1499 (*emulans*); 30601 (*americanum*); 31345 (*nigrescens*).

Lakshnakara, M.C. 368 (*nigrum*).

Lam, G. 20045 (*sarrachoides*).

Lamb, F.H. 362, 408 (*nigrescens*).

Lambdon, P.W. A036 (*americanum*).

Lambinon, J. 86/354, 80/Co/882, 906 (*nigrum*); 907 (*villosum*); 80/907 (*nigrum*); 1461 (*triflorum*).

Lambrecht, F.L. 90 (*scabrum*).

Lammers, T.G. 8400 (*sarrachoides*); 8490 (*americanum*).

Lamond, J. 1611, 2283 (*villosum*).

Lamoureux, C. 2225, 2298 (*americanum*).

Lamoureux, C.H. 4075 (*americanum*).

Landrum, L.R. 1506 (*furcatum*); 2594 (*americanum*); 4416 (*furcatum*); 4427 (*nitidibaccatum*); 5296 (*douglasii*); 7429 (*furcatum*); 7807 (*americanum*); 8506 (*douglasii*); 10999 (*triflorum*); 11349 (*americanum*); 11394a (*emulans*); 11508 (*furcatum*).

Lane, F. 2379 (*emulans*).

Lange, P.J. de 6292 (*nitidibaccatum*).

Langenheim, J.H. 88 (*emulans*); 3336 (*americanum*); 3546 (*macrotonum*).

Langford, F.L. 89 (*emulans*).

Langley, R. 2519 (*emulans*).

Langlois, A.B. 239 (*nigrescens*).

Lanjouw, J. 3122 (*americanum*).

Lankester, T.E. 336 (*villosum*); 1419 (*nigrum*).

Lansing, O.E. 1378 (*emulans*).

Lara, M. 161 (*nigrescens*).

LaRivers, I. 767 (*triflorum*).

Larke, J. 131 (*douglasii*).

Larsen, E.L. 176 (*triflorum*); 183 (*interius*); 5830, 7208 (*triflorum*).

Larsen, K. 67 (*nigrum*); 34265 (*americanum*).

Lasser, T. 739 (*americanum*).

Latilo, M.G. FHI-77429 (*scabrum*).

Latz, P.K. 13385 (*nigrum*).

Lau, S.K. 209 (*americanum*); 1005 (*nigrum*); 3752 (*americanum*); 4236[a], 4238, 5025 (*nigrum*); 5963, 25582 (*americanum*).

Laughlin, R.M. 321 (*nigrescens*); 1363 (*douglasii*).

Laukkonen, P. 18 (*villosum*).

Lavranos, J.J. 15827, 15875 (*villosum*).

Lawalrée, A. 15503, 21201 (*nigrum*); 22320 (*villosum*); 23602 (*nigrum*).

Lawrance, A.E. 168 (*americanum*).

Lawrence, D.W. 169 (*chenopodioides*).

Lawrence, E. 138 (*americanum*).

Lawrence, W.E. 416, 2588, 3592, 4087, 4368, 4576 (*triflorum*).

Lawton, R.O. 794, 830 (*nigrescens*).

Lazar, Y. 92 (*nigrum*).

Lazarides, M. 332 (*nigrum*).

Lazeo, F.S.M. 5 (*americanum*).

Lazor, R.L. 2136, 2795 (*americanum*).

Le Clezio 259 (*americanum*).

Le Clezio, [?] 217 (*nigrescens*).

Le Cussan, J.M. 939 (*americanum*).

Le Tram Chan C-96 (*americanum*).

Leach, E. 350 (*emulans*).

Leach, L. 448 (*triflorum*).

Léandri, J. 3092 (*americanum*).

Leavenworth, W.C. 352, 592, 4020 (*douglasii*).

LeBreton, C. 237 (*americanum*).

Lebrun, J.P. 8015 (*americanum*).

Lechiner, H.J. 1, 9 (*triflorum*).

Ledesma Corral, J.C. 1578 (*nigrescens*).

Ledesma, C. JDA-20153 (*nigrescens*).

Ledesma-Corral, C. 295, 363 (*douglasii*).

LeDoux, D.G. 2271 (*nigrescens*).

Lee, E. 1663 (*nitidibaccatum*); 9196, 9197 (*douglasii*).

Lee, H.C. 305, 417 (*douglasii*).

Leeuwenberg, A.J.M. 2130 (*americanum*); 4668 (*scabrum*).

Legler, B. 6420, 9744, 11231 (*triflorum*).

Legrand, C.D. 419 (*chenopodioides*).

Legrand, D. 1917, 1918, 1961 (*sarrachoides*).

Lehr, J.H. 648 (*emulans*); 1652 (*douglasii*).

Lehto, E. 3496 (*nitidibaccatum*).

Lei, C.I. 159 (*americanum*).

Leiberg, J.B. 877 (*americanum*); 929 (*nigrum*); 5775 (*triflorum*).

Leigh, J.H. S600 (*triflorum*).

Leishman, A.J. 115 (*americanum*).

Leistner, O.A. 2706 (*retroflexum*).

Leitão, F. 111, 133 (*americanum*).

Leite, J.E. 270 (*americanum*).

Leitner, L. 2867 (*emulans*).

Leiva G, S. 699, 707 (*corymbosum*).

Lejeune, A.L.S. 55 (*scabrum*).

Leland, B. 78 (*americanum*).

Lely, H.V. 31 (*scabrum*); P.392 (*villosum*).

Lemaire, R.J. 850 (*nigrescens*).

Lemes, F.O.A. 603 (*americanum*).

Lemon, J.H. 1608 (*emulans*).

Lent, R.W. 582 (*americanum*); 1640, 3228, 3394, 3404, 3661, 3757, 3927 (*nigrescens*).

León de la Luz, J.L. 4008 (*douglasii*).

León G, J. 48 (*americanum*).

León, H. 17 (*nigrescens*).

León, J. 207 (*macrotonum*); 470, 4631 (*nigrescens*).

Leonard, E.C. 3340, 3341, 10350 (*emulans*).

Leonard, G.M. 15895 (*emulans*).

Leonard, J. 3627 (*villosum*); 3708 (*nigrum*); 4408 (*villosum*); 4738 (*nigrum*).

Leonard, S.W. 3306 (*emulans*); 3470 (*chenopodioides*); 4363 (*pseudogracile*).

Leoni, L.S. 108, 108, 6554 (*americanum*).

Leonis, C. 137 (*villosum*).

Leonti, M. 86 (*nigrescens*).

Lepage, E. 14327 (*nitidibaccatum*).

Lepschi, B.J. 137 (*chenopodioides*); 381, 391 (*americanum*); 481, 489, 493, 539 (*chenopodioides*); 542, 555 (*triflorum*); 683, 740, 786, 897 (*chenopodioides*); 903, 904 (*americanum*); 930 (*chenopodioides*); 1153 (*americanum*); 1729 (*nitidibaccatum*); 1752, 2580, 4113 (*americanum*); 5088 (*chenopodioides*).

Lescure, J.P. 630 (*americanum*).

Lesica, P.C. 6126, 7803 (*triflorum*).

Lester, R.N. 43, 45 (*scabrum*).

LeSueur, H. 441, 1447 (*douglasii*).

Leteinturier, B. 145 (*villosum*).

Letouzey, R. 6880 (*scabrum*).

Leveque, R. 36, 200A (*americanum*).

Lévesque, L. [Frère] 650831-2569 (*emulans*).

Levine, C.O. 178 (*americanum*).

Lévy T, S. 253 (*nigrescens*).

Lewalle, J. 2978, 5327 (*villosum*); 9857 (*nigrum*); 10085, 10673 (*villosum*); 11182 (*nigrum*); 11247 (*villosum*).

Lewis, A. 337 (*douglasii*).

Lewis, C.M. 674 (*triflorum*).

Lewis, M. 88105 (*americanum*).

Lewis, W.H. 93 (*nigrescens*); 388, 554 (*americanum*); 1510, 2955, 2978, 3204 (*nigrescens*); 3299, 3398, 5183 (*americanum*); 7336 (*nitidibaccatum*); 8003, 10197 (*americanum*).

Leybourn, W.A. 75 (*triflorum*).

Leysan, M.E. 1382 (*nigrescens*).

Lezama, D.I. 58 (*nigrescens*).

Li Sheng-tang 81-1327 (*americanum*).

Li, H. 8809 (*americanum*); 10342, 12853, 13913, 15251 (*nigrum*).

Liane 3661 (*americanum*).

Libbey, R.P. H536/44 (*nitidibaccatum*).

Licata, A. 383 (*americanum*).

Lichvar, R.W. 3165 (*triflorum*).

Liebenberg, L.C.C. 6877, 7410 (*retroflexum*); 7909 (*chenopodioides*); 8240 (*retroflexum*); 8568 (*chenopodioides*); 8842 (*retroflexum*).

Liesner, R. 107 (*emulans*); 13443 (*americanum*).

Liesner, R.L. 2198, 2784 (*nigrescens*); 4397, 5388 (*americanum*); 7988 (*macrotonum*); 10147, 12623, 12908 (*nigrescens*); 12911 (*macrotonum*); 14183 (*nigrescens*); 26560 (*douglasii*).

Lievens, A.W. 2056 (*americanum*); 2255, 2377, 2818, 4521 (*nigrescens*).

Liljeblad, E. 32, 33 (*scabrum*).

Lim, B.K. 1440 (*nigrum*).

Lima, J.R. 84 (*americanum*).

Lima-Verde, L.W. 360, 921 (*americanum*).

Limbach, C.F. 145 (*americanum*).

Lin, C.H. 147, 953 (*americanum*).

Linares, E. 1110 (*douglasii*).

Linares, J.L. 374 (*nigrescens*).

Lind, E.M. 82 (*villosum*).

Lindberg, K. 756 (*villosum*).

Lindeman, J.C. 1373 (*americanum*).

Linden, J.J. 250 (*americanum*); 2204 (*nigrescens*).

Lindsay, N. 1023 (*nigrum*).

Liney, J. 2765 (*chenopodioides*).

Lingenfelter, R.L. 452 (*triflorum*).

Liogier, A.H. 9024-14 (*americanum*).

Liou, S.Z. 305 (*nigrum*).

Lippert, W. 16053 (*villosum*).

Lisakowski, [?] 26 (*americanum*).

Lithgow, G.M. 246 (*americanum*).

Liu Pi 6042, 6512 (*nigrum*).

Liu, S.L. 89-109, 890109 (*nigrum*).

Liu, Y.C. 302 (*nigrum*).

Lix, H.W. 657 (*emulans*).

Llanos, F. 1785 (*nigrescens*).

Llatas Q, S. 645 (*corymbosum*); 733 (*americanum*).

Llatas Quiroz, S. 645 (*corymbosum*).

Lleras P, E. 1943 (*americanum*).

Lloyd, C.G. 2196 (*emulans*).

Lloyd, R.N. 1136 (*nigrescens*).

Lobão, A.Q. 13, 45 (*americanum*).

Lobo Miranda, F.C. 2444 (*villosum*).

Lodewycks, M.C. 32 (*emulans*).

Loibl, J. 1954 (*villosum*).

Lojacono, M. 215 (*nigrum*).

Lomer, F. 92-117, 88-168 (*triflorum*); 4981 (*nigrum*); 5043 (*americanum*); 5479 (*nitidibaccatum*); 5549 (*americanum*); 5563, 5568, 5710 (*nigrum*); 6126, 6185 (*americanum*); 6193, 6374 (*nigrum*); 6375 (*americanum*); 6380, 6395 (*nigrum*); 6418 (*emulans*); 6425 (*americanum*); 6861 (*nigrum*); 7212 (*americanum*); 8019 (*nigrum*); 8576 (*americanum*); 8585 (*nigrum*); 8870 (*emulans*); 9067, 9073 (*nitidibaccatum*); 9077 (*nigrum*); 9610 (*triflorum*); 9626 (*villosum*); 9627 (*nigrum*).

Londoño U, R. 131 (*macrotonum*).

Long, B. 40687 (*emulans*).

Long, C. 124 (*americanum*).

Long, C.R. 1720, 2238, 2318, 2383 (*americanum*).

Long, R. 75317 (*nitidibaccatum*).

Long, R.W. 2828 (*americanum*); 2883, 3083 (*nigrescens*).

Longbottom, W.D. 11015 (*americanum*); 12187, 14228, 15221, 15354, 15495, 16013, 16649 (*emulans*); 17139 (*pseudogracile*); 17840, 18483, 21660 (*emulans*).

Looman, J. 13176 (*triflorum*).

Looser, G. 4451 (*furcatum*).

López C, R. 7017 (*nigrescens*); 7828 (*americanum*).

López Ch, L. 234 (*americanum*).

López García, E. 51 (*nigrescens*).

López L, L. 166 (*nigrescens*).

López L, L.M. 18 (*americanum*).

López López, L. 166, 377, 500 (*nigrescens*).

López Luna, R. 31 (*nigrescens*).

López P, J. 326 (*douglasii*).

López Pérez, J. 332 (*nigrescens*).

López R, C.A. 48 (*macrotonum*).

López Santos, B.Y. 170, 770, 859 (*nigrescens*).

López, [?] 24, 190 (*nigrescens*); 286 (*douglasii*).

López, A. 8 (*nigrescens*).

López, F. 160 (*nigrescens*).

Lopez, I. 32 (*americanum*).

López, L. 31 (*nigrescens*); 377 (*douglasii*).

López, L.M. 166, 377 (*douglasii*).

López, N. 73 (*nigrescens*).

López, N. de 417, 462, 463 (*nigrescens*); 924 (*americanum*).

López-Palacios, S. 2042 (*nigrescens*).

López-Pérez, J. 326 (*douglasii*).

Lorena O, V. 139 (*nigrescens*).

Lorence, D.H. M179 (*americanum*); 3658 (*nigrescens*); 5751 (*americanum*).

Lorentz, P.G. 26 (*chenopodioides*); 1132 (*triflorum*).

Lot, A. 272, 422, 1314, 1586 (*nigrescens*).

Lotan, R. Soni-161008, Soni-200150 (*nigrum*).

Lott, E.J. 3397 (*americanum*); 4508 (*nigrescens*).

Louis, A.M. 1990 (*scabrum*); 2292 (*americanum*).

Louis, J.L.P. 10507 (*scabrum*).

Louis-Alphonse 1175, 1429, 3560, 3631 (*emulans*).

Lourteig, A. 1135, 1912 (*americanum*).

Lousley, J.E. W/493 (*triflorum*); 605 (*americanum*).

Lovett, J.C. 2122 (*villosum*); 3696 (*americanum*).

Low, A. 382 (*villosum*).

Lowden, R.M. 4816 (*emulans*).

Lowe, E. 24 (*nigrescens*).

Lowe, J. 2951 (*scabrum*); 4153 (*americanum*).

Lowe, R.T. 16[A], 16[B], 37[A], 37[B], 119 (*nigrum*); 547, 722, 722[a], 722[b] (*villosum*).

Lowry, P.P. 11 (*interius*).

Lox, B. Q94-01 (*chenopodioides*).

Lozano C, G. 2899 (*nigrescens*).

Luckner, C. 562 (*nigrescens*).

Lucy, T.F. 7843 (*emulans*).

Ludlow, F. 6010 (*nigrum*).

Lugagne, R. 7256 (*nigrum*).

Lugas, L. 2760 (*americanum*).

Lugo L, E. 55 (*nigrescens*).

Lugo S, H. 6082, 6094 (*americanum*).

Lukasser, B. 125 (*nigrescens*).

Luke, Q. 12339 (*scabrum*).

Luke, W.R.Q. 14210 (*villosum*).

Lumer, C. 1314 (*nigrescens*).

Luna Castellanos, F. 151 (*nigrescens*).

Luna M, V.E. 114, 760 (*nigrescens*).

Lund, P.W. 59 (*americanum*).

Lundell, C.L. 1185 (*americanum*); 1512, 5163, 10005 (*nigrescens*); 10697 (*americanum*); 10729, 13255, 13274, 19683 (*nigrescens*).

Lurvey, E. 480 (*americanum*).

Luteyn, J.L. 675, 4816 (*macrotonum*); 12126 (*nigrescens*).

Luz, [?] 9 (*americanum*).

Lynch, P.S. 631, 631, 1224 (*emulans*).

Lyne, A.M. 1936 (*chenopodioides*).

Lynes, H. 37c, 132, 134 (*villosum*).

Lyonnet, E. 213 (*douglasii*); 231, 362 (*nigrescens*); 1237, 2474 (*douglasii*).

Lyons, M.N. 4693 (*americanum*).

Lytjen, D. 250 (*triflorum*).

Maas Geesternanus, R.A. 4314 (*nigrum*).

Mabatha, F.W. 2542 (*americanum*).

Macbride, J.F. 734 (*nigrum*); 737 (*nitidibaccatum*); 738 (*nigrum*); 772, 4477 (*douglasii*).

MacBryde, B. 1033, 1061, 1111 (*americanum*); 1269 (*macrotonum*).

MacDaniels, L.H. 93 (*douglasii*); 174 (*americanum*).

MacDonald, E.C. 117, 137 (*retroflexum*); 164, 174 (*chenopodioides*).

Macdonald, I.D. a14 (*emulans*).

MacDonald, J.R. 9736 (*emulans*).

MacDougal, D.T. 213, 10915 (*triflorum*).

MacDougal, J.M. 777 (*macrotonum*); 3102 (*nigrescens*); 3754 (*americanum*).

MacElwee, A. 1449 (*emulans*).

MacFadden, F.A. 6E (*douglasii*).

Machado, B. 1826 (*americanum*).

Machado, D.N.S. 337 (*americanum*).

Macias Acevedo 13 (*nigrescens*).

Maciel, A.A. 40 (*americanum*).

MacIntosh, D. 62 (*villosum*).

MacKee, H.S. 20631, RSNH24143, RSNH24217 (*americanum*).

MacKeever, F.C. N-433 (*emulans*); N-434, N-691 (*nitidibaccatum*).

MacKinnon, W.A. 2171 (*emulans*).

Macmillan, H.F. 219, 220 (*nigrum*).

Macmillan, H.G. 22 (*furcatum*).

Maconochie, J.R. 2990 (*villosum*).

Macoun, J. 698 (*nitidibaccatum*); 1182, 1607 (*triflorum*); 1608 (*emulans*); 5814, 24020, 78428 (*triflorum*); 85823 (*nitidibaccatum*).

Macoun, M.A. 1181 (*emulans*).

Macrae, J. 635 (*nigrum*).

Madrigal Sánchez, X. 3039 (*douglasii*).

Madrigal, B. 540 (*americanum*).

Madsen, J.E. 63299, 63358, 63457 (*americanum*).

Madsen, M. 1122, 2637, 4338 (*triflorum*).

Madulid, D.A. 910, 1565, 8801 (*americanum*).

Maesen, L.J.G. van der 962 (*americanum*); 3190, 7766 (*villosum*).

Maffey, Lady 5 (*villosum*).

Magaji, S.O. MG-725 (*scabrum*).

Magaña, M.A. 876 (*americanum*); 1337, 1339, 1428 (*nigrescens*).

Maguire, B. 308 (*nigrum*); 311, 2066 (*triflorum*).

Maheshwari, J.K. 4027 (*villosum*); 4635, 5614 (*nigrum*).

Mahlberg, P. 618 (*emulans*).

Mahu, M. 7617 (*furcatum*).

Maioli, V. 168 (*americanum*).

Maire, E.E. 78, 409 (*nigrum*); 531 (*villosum*); 813 (*americanum*).

Maitland, D. 103 (*nigrum*).

Maitland, T.D. 414, 522 (*villosum*).

Maity, D. BSHC-21633, BSHC-23245 (*americanum*); BSHC-25627, BSHC-25657 (*nigrum*).

Maj, M. 3439 (*nigrescens*).

Major-Barnabé 3942 (*emulans*).

Majumdar, S.C. NC-86359 (*villosum*).

Majunder, N.C. 8, 10, 203 (*americanum*).

Makarick, L.J. 37 (*douglasii*).

Makin, J. 168 (*nigrum*).

Makings, E. 546 (*douglasii*); 4677 (*americanum*).

Makings, L. 4750 (*douglasii*).

Makinson, R.O. 209, 842, 842 (*americanum*).

Makrinius, E. 773 (*nigrescens*).

Malcomber, S.T. 1716 (*americanum*).

Maldonado Vasquez, N. 95 (*nigrescens*).

Malhotra, B.K. NC-19716 (*americanum*).

Malhotra, C.L. NC-12018, NC-12054, NC-12464 (*americanum*); NC-13359 (*nigrum*); NC-13480 (*villosum*); NC-22832 (*nigrum*); NC-23596 (*americanum*); NC-23691 (*nigrum*); NC-26752, NC-26817 (*americanum*); NC-27338, NC-31432 (*villosum*); NC-31595, NC-50575 (*nigrum*).

Malhotra, S.K. NC-13156 (*nigrum*); NC-15364, NC-15874, NC-19165 (*americanum*); NC-19716, NC-22715 (*villosum*); NC-28784 (*americanum*); NC-31226 (*villosum*).

Malick, K.C. 45 (*villosum*).

Malik, K.I. 115 (*nigrum*).

Maliwanag, E. 132 (*americanum*).

Maltby, F.S. 72, 118, 223, 226 (*douglasii*).

Malthastra, M.C. NC-19580 (*villosum*).

Mamadou, E.M. 194 (*americanum*).

Mamaslu 2008 Expedition 20816047 (*nigrum*).

Man, H. 85132 (*americanum*).

Man, L.S. 54019, 55105, 92854 (*americanum*); 93206 (*nigrum*); 96061 (*americanum*).

Manaslu 2008 Expedition 20815026 (*nigrum*).

Mancías, J. 1008 (*nigrescens*).

Mandal, N.R. BSHC-10021 (*americanum*); BSHC-10183, BSHC-10441 (*nigrum*).

Mandaville, J.P. 308, 2499, 3414 (*nigrum*); 3538, 3986, 6286, 6358, 6406, 6673, 6980, 7414 (*villosum*).

Manikandan, R. NC-112491 (*americanum*).

Manissadjian, A. 349b, 981b (*villosum*).

Manning, S.D. 235 (*scabrum*).

Manoharan, C. 19575 (*americanum*).

Mansfield, D. 15-297, 02-1021 (*triflorum*).

Mansfield, D.H. 21021 (*triflorum*).

Mantuano, M. 64 (*americanum*).

Manua, M.K. 998, 1092 (*americanum*).

Manzanero M, G.I. 1009 (*pruinosum*).

Mapes, C. 41-C (*douglasii*).

Marcan, A. 2225 (*nigrum*).

Marcelline, S.M. 2865 (*triflorum*).

Marcelo, J. 62, 63 (*americanum*).

Marchett, F. 327 (*americanum*).

Marcks, B. 32, 1382 (*emulans*).

Marcolino, F. 163 (*americanum*).

Marcondes-Ferreira, W. 1752 (*americanum*).

Marcum, P.B. 1766, 3797, 5911 (*emulans*).

Margery, C. 4071 (*americanum*).

Marie-Victorin [Frère] 3895, 3942, 15677, 15678, 45753 (*emulans*).

Maries, C. 191, 249 (*nigrum*).

Marín, M.F. 66 (*nigrescens*).

Marodin, S.M. 368 (*americanum*).

Marquete, N. 535 (*americanum*).

Marquete, R. 4312, 4555 (*americanum*).

Márquez Ramírez, W. 285 (*nigrescens*); 677 (*americanum*).

Márquez, W. 137 (*nigrescens*).

Marriott, H. 735, 5599, 9319 (*triflorum*).

Marrs, G.E. 535 (*douglasii*).

Marsh, E. 137, 394 (*nigrescens*).

Marsh, E.G. 85 (*douglasii*); 394 (*nigrescens*); 651, 770 (*douglasii*); 1589, 1719 (*nigrescens*).

Marsh, V.L. 125 (*triflorum*).

Mart 6667 (*nigrescens*).

Marten, A.M. van 100 (*americanum*).

Martensz, P.N. 76 (*triflorum*); 188 (*americanum*).

Marticorena, C. 1077 (*furcatum*).

Martin, C. 883 (*nigrescens*).

Martin, G.J. 28, M-51 (*nigrescens*); 152 (*douglasii*); M-242 (*americanum*); 448 (*douglasii*).

Martin, J.H. 3243 (*villosum*); 3592 (*americanum*).

Martin, J.S. 17 (*americanum*).

Martin, P. 6848 (*chenopodioides*).

Martin, R.F. 1321 (*americanum*).

Martinelli, G. 3579, 3969 (*americanum*).

Martínez A, M.A. 535 (*nigrescens*).

Martínez B, A. 16 (*douglasii*).

Martínez C, G. 1342, 1830 (*nigrescens*).

Martínez C, H.A. 178 (*nigrescens*).

Martínez Calderón, G. 778 (*nigrescens*); 1342 (*americanum*).

Martínez-Crovetto, R. 1923 (*triflorum*); 1508 (*chenopodioides*); 1750 (*triflorum*); 1975 (*chenopodioides*); 2507, 2507 (*triflorum*); 4201, 4444 (*americanum*); 4762, 5068, 9278 (*chenopodioides*).

Martínez Cruz, J. 1123 (*douglasii*); 1368 (*nigrescens*).

Martínez Gordillo, M. 1657 (*douglasii*).

Martínez L, A. 124 (*americanum*).

Martínez Marín, J.L. 402 (*nigrescens*).

Martínez R, C. 906 (*nigrescens*).

Martínez S, E. 3217 (*nigrescens*); 8044, 29419, 29430 (*americanum*).

Martínez S, E.M. 193, 345 (*nigrescens*); 4131 (*douglasii*); 5093, 5414, 6004, 11166, 12111, 14557, 16236, 18413, 19701, 19728, 21582, 26163, 26492A, 28426, 28823, 29272, 29589, 31546, 31871, 36650, 38233 (*nigrescens*); 39832 (*americanum*).

Martínez, F.R. 95 (*villosum*).

Martínez, J.L. 251, 488, 643 (*nigrescens*).

Martínez, K. 198 (*nigrescens*).

Martínez, L. 1202 (*americanum*).

Martínez, M. 1136 (*douglasii*); 1211 (*pruinosum*); 5345 (*nigrescens*).

Martinez, R. 76 (*nigrescens*).

Martínez, S. 260 (*nigrescens*).

Martínez-Calderón, G. 172 (*nigrescens*); 1830 (*americanum*).

Martins, J. 310 (*nigrum*).

Martins, S.A. 28 (*americanum*).

Martius, C.F.P. 1255 (*scabrum*).

Martling, S. 76 (*triflorum*).

Mashaly, I. 20189 (*villosum*).

Mason, C.T. 3875 (*americanum*).

Mason, H.L. 520 (*douglasii*); 1129 (*americanum*); 4124 (*douglasii*); 4150 (*americanum*); 4153 (*douglasii*); 5445, 13259 (*americanum*); 13341 (*triflorum*).

Mason, R. 1363, 1609, 1724, 1726, 2170, 2200, 2298, 2476 (*nigrum*).

Mata P, S. 87-82 (*nigrescens*).

Maté, C. 14 (*scabrum*).

Matesevach, M. 10 (*nitidibaccatum*); 38 (*triflorum*).

Mathenge, S.G. 64 (*villosum*).

Mathews, A. 144H (*corymbosum*); 270 (*furcatum*); 730 (*americanum*).

Mathey, A. 164 (*americanum*).

Mathis, A.T. 2 (*emulans*).

Matlosz, D. 13 (*emulans*).

Matos, E.C.A. 7 (*americanum*).

Matthei, O. 73 (*furcatum*); 138 (*nitidibaccatum*); 510 (*americanum*).

Matthes, B. 305 (*emulans*).

Matthew, K.M. 17002, 19984 (*americanum*); 24905 (*nigrum*); 46324 (*americanum*).

Mattos, J.R. 50, 74 (*americanum*).

Matuda, E. 257 (*nigrescens*); 1034 (*douglasii*); 1296, 4618, 19121 (*nigrescens*); 21319, 26529, 37364 (*douglasii*).

Matzenbacher, N.I. 3064 (*americanum*).

Mauad, L.P. 29, 443 (*americanum*).

Mauritz, J. 988 (*emulans*).

Mautone, L. D23, 554 (*americanum*).

Maxon, W.R. 1133 (*macrotonum*); 3230 (*americanum*).

Maxwell, J.F. 92-100 (*nigrum*); 91-474 (*americanum*); 94-536 (*nigrum*); 88-845 (*americanum*); 94-1244 (*nigrum*).

Maxwell, R.H. 231 (*douglasii*); 2736 (*emulans*).

May-Pat, F. 668 (*nigrescens*).

Maya J, S. 453, 496, 672 (*nigrescens*).

Maze, J. 1060 (*douglasii*).

Mazzucconi, V.J. 1547 (*triflorum*).

Mbuthia, K.W. 406, 734 (*villosum*).

McBarron, E.J. 9 (*chenopodioides*); 3094, 3118 (*triflorum*); 4253 (*americanum*); 6803, 7960, 9998, 11054, 11545, 11725, 12357, 14881 (*chenopodioides*); 16154 (*triflorum*); 20125 (*chenopodioides*).

McCabe, T.T. 3789 (*nigrum*); 3799 (*americanum*).

McCalla, W.C. 9645 (*triflorum*); 10758 (*douglasii*); 11221, 11335 (*triflorum*).

McCallum-Webster, M. 1431, 2142, 7015, 8768 (*nitidibaccatum*).

McCaskill, J. 582 (*retroflexum*).

McCauley, K.M. 616 (*emulans*).

McClain, W. 210D, 2122, 2193 (*emulans*).

McClean, A.P.D. 378 (*retroflexum*); 869 (*chenopodioides*); 28270 (*retroflexum*).

McClelland, D. 357 (*emulans*); 365 (*nigrum*).

McCluskey, L. 271, 272 (*triflorum*).

McCook, L. 1118 (*macrotonum*).

McCornish, J.D. 46 (*americanum*).

McCown, B. 35 (*emulans*).

McCoy, S. 551, 2172, 5929 (*emulans*).

McCoy, T. 73-2033 (*emulans*).

McDaniel, S. 8148 (*nigrescens*); 8866, 13861 (*americanum*); 20634 (*emulans*); 22404 (*nigrescens*); 23804 (*americanum*); 25060 (*nigrescens*); 25378 (*americanum*); 29971 (*emulans*); 30219, 32880 (*americanum*).

McDaniels, L.H. 140 (*emulans*).

McDonald, [?] 1528 (*americanum*).

McDonald, C.H. 640 (*triflorum*).

McDonald, M.E. 5436 (*emulans*).

McDonald, T.J. 00548 (*americanum*).

McDonnell, M.J. 769 (*emulans*).

McDougall, W.B. 1502 (*emulans*).

McDowell, T. 2604, 3207 (*americanum*).

McFadden, I. 9 (*nigrum*).

McGill, L. 20785 (*americanum*).

McGill, L.A. 6254 (*triflorum*).

McGillivray, D.J. 3627 (*americanum*).

McGregor, R.L. 399 (*nigrescens*); 571 (*pruinosum*); 5176 (*triflorum*); 31057 (*sarrachoides*).

McIntosh, T. 2011-214 (*nitidibaccatum*).

McKee, H.S. 654 (*triflorum*); 3794 (*americanum*).

McKee, M. 3 (*emulans*).

McKenna, S.G. 217, 468 (*americanum*).

McKenzie, K.K. 6277, 6820 (*emulans*).

McKnight, K. 10707 (*nitidibaccatum*).

McLeish, I.M. 425, 2006, 2140, 2257, 2510, 3317, 3636 (*villosum*).

McMahon, A.H. 47/118 (*villosum*).

McMinn, H.E. 4358 (*douglasii*).

McMurry, F.B. 702 (*emulans*).

McNamara, S. 12 (*nigrum*).

McNeill, J. 225, 496 (*villosum*).

McNeilus, V.E. 98-322, 99-889 (*emulans*).

McPherson, G. 4594 (*americanum*).

McVaugh, M.R. 134 (*emulans*).

Mearns, E.A. 121 (*interius*); 450 (*triflorum*); 492 (*emulans*); 1510 (*interius*); 2156, 2341 (*triflorum*); 2630, 3845 (*douglasii*); 4844 (*triflorum*).

Medeiros 395 (*americanum*).

Mederios, E.V.S.S. 395 (*americanum*).

Medina C, M. 422a, 448, 594, 1610, 1620b, 1799, 1950 (*nigrescens*); 2095 (*corymbosum*).

Medina C, M.A. 228 (*americanum*).

Medina, A. 4 (*macrotonum*).

Medler, B. 105 (*americanum*).

Medley Wood, J. 83 (*americanum*); 811 (*retroflexum*).

Medri, C. 303 (*americanum*).

Meenakshi, B. 65 (*americanum*).

Mehrhoff, L.J. 912, 4298, 13782 (*emulans*).

Meier, W. 3769, 4865 (*macrotonum*); 6425, 8465, 8674, 9345 (*nigrescens*); 16386 (*americanum*).

Meikle, R.D. 2735, 5026 (*villosum*); 5048 (*nigrum*).

Meinertzhagen, R. 152 (*villosum*).

Mejía P, F. 278 (*nigrescens*).

Mejía, C. 64 (*nigrescens*).

Mejía, M. 124, 4984, 7572 (*nigrescens*).

Melampy, M. 904 (*macrotonum*).

Meléndez López, E. 2256 (*nigrescens*).

Melo, P.H.A. 875 (*americanum*).

Melville, F.A. 4 (*americanum*); 188 (*scabrum*).

Melville, R. 130 (*emulans*).

Memeryan 5159 (*villosum*).

Mena V, P. 123 (*macrotonum*).

Méndez C, V. 170 (*nigrescens*).

Méndez G, A. 4981, 8600 (*douglasii*); 9480 (*nigrescens*).

Mendez Ton, A. 4546, 4981, 6419, 9480 (*nigrescens*).

Méndez, R. 187 (*americanum*).

Mendonça, F.A. 1646 (*villosum*).

Meneghel, R. 34 (*americanum*).

Menendez, F. 216 (*americanum*).

Menjívar, J. 415 (*americanum*).

Mentz, L.A. 33, 51, 173, 353, 434, 438, 498 (*americanum*).

Merello, M. 162 (*douglasii*).

Meriläinen, J. 92 (*americanum*); 97 (*pseudogracile*); 102, 1103 (*americanum*).

Merrill, E.D. 361 (*americanum*); 464 (*nigrum*); 465[/1] (*triflorum*).

Merrill, G.M. 651, 969 (*emulans*).

Merton, L. ARI-8 (*villosum*).

Messer, E. 212a (*americanum*).

Metcalfe, O.B. 138 (*triflorum*); 429 (*interius*); 1005 (*triflorum*); 1018 (*interius*).

Metlen, R. 3103 (*nitidibaccatum*).

Metz, M.C. 481 (*nigrescens*).

Mexia, Y. 4336, 4529, 5854, 5930, 6298 (*americanum*).

Meyer, C.V. 252 (*douglasii*).

Meyer, D. 203 (*emulans*).

Meyer, F.G. 797 (*nitidibaccatum*); 2781 (*pruinosum*); 10044 (*nigrescens*).

Meyer, K.M. 31 (*douglasii*).

Meyer, R.S. 285 (*nigrum*); 403 (*emulans*).

Meyerhoff, E. 22 (*villosum*).

Meyers, F. B-4102 (*nigrum*); 4810 (*villosum*).

Meyers, F.S. 102, 102c, 102a (*villosum*); 401 (*nigrum*); 809, 2809, 3151 (*villosum*); 4102 (*nigrum*); 5151, 6102 (*villosum*).

Meza T, E.I. 856 (*triflorum*).

Michaels, C.C. 2094 (*douglasii*).

Michel, R. de 2769 (*sarrachoides*).

Michelangeli, F.A. 933 (*macrotonum*).

Mick, G. 147 (*douglasii*).

Middleton, B. 5 (*emulans*).

Middleton, D.J. 2476 (*americanum*).

Midence, S. 164 (*nigrescens*).

Miers, J. 276 (*furcatum*); 1235, 1412, 1427, 1428 (*chenopodioides*); 1815, 4540[b] (*americanum*).

Miéville, R. 37297 (*americanum*).

Migeod, F.W.H. 88, 358 (*scabrum*).

Mikoláš, V. 2375, 2403 (*nigrum*); 2541 (*villosum*); 2901 (*nigrum*); 3928 (*villosum*); 5956 (*nigrum*); 6304, 6359, 6369 (*villosum*); 6723, 7622, 7729 (*nigrum*); 7887, 8082 (*villosum*); 8271, 8436 (*nigrum*); 8530, 8588, 8619 (*villosum*); 8720 (*nigrum*).

Millan, B. 01 (*americanum*).

Millar, A.N. NGF-15825[a] (*americanum*).

Mille, L. 977 (*americanum*).

Miller, A. 114, 555, 592, 873 (*villosum*).

Miller, A.G. 42 (*villosum*); 114 (*nigrum*); 147, 374 (*villosum*); 592 (*nigrum*); 2395 (*villosum*); 3424 (*nigrum*); 5292, 6632, 7196 (*villosum*); 7715 (*nigrum*); M.8248 (*villosum*).

Miller, E.M. M12/226 (*americanum*).

Miller, J.S. 601, 1168 (*nigrescens*); 1317 (*douglasii*); 1975 (*emulans*); 2980, 3218 (*nigrescens*); 6349 (*americanum*); 6772 (*triflorum*); 6882 (*nitidibaccatum*); 7731 (*douglasii*); 8833 (*americanum*); 9208 (*nigrum*); 9783 (*emulans*).

Miller, O.B. 4341 (*retroflexum*).

Miller, R.B. 385 (*emulans*).

Miller, T.C. 57A (*nigrescens*).

Milliken, W. 1362 (*nigrum*).

Millspaugh, C.F. 1082 (*americanum*); 1444, 2270 (*nigrescens*); 4432 (*chenopodioides*); 4433 (*douglasii*); 4436 (*americanum*); 4476, 4514, 4606, 4707, 4719 (*douglasii*); 9381 (*nigrescens*).

Milne-Redhead, E.W. 1834 (*triflorum*); 10186 (*villosum*).

Milthorpe, P.L. 5072 (*triflorum*).

Mimoro, K. 2645 (*nigrum*).

Miranda, A.G. 562 (*americanum*).

Miranda, A.M. 2859, 3483, 3641 (*americanum*).

Misra, O.P. 9771 (*villosum*); NC-41511 (*americanum*); NC-41840, NC-44354 (*villosum*); NC-44450 (*americanum*); NC-46912 (*villosum*).

Mitchell, A.A. 5513, 5513, 6682, 6682, 6682, 7413, 7413, 8071 (*americanum*).

Mitchell, R.S. 2146, 7838 (*nitidibaccatum*).

Mittleman, M. 2209 (*douglasii*).

Miyamoto, F. 10017, 94-10017 (*nigrum*).

Miyazaki, T. 99-1120II/5 (*villosum*); 0310334 (*americanum*).

Mlangwa, J.A. 482 (*villosum*).

Mocquerys, A. 163 (*americanum*); 763, 1164 (*nigrescens*).

Modenke, H.N. 442 (*americanum*).

Moe, J.A. 271 (*emulans*).

Moffitt, G. 8, 9 (*emulans*).

Mohan, A. 13135 (*americanum*).

Mohan, J. 1024 (*triflorum*).

Mohlenbrock, R.H. 9240 (*emulans*).

Mohr, P.F. 21 (*triflorum*).

Moir, D.R. 1645 (*interius*).

Mokim, S. 1280 (*villosum*).

Molas, L. 1391 (*sarrachoides*).

Moldenke, H.N. 442 (*americanum*); 507 (*pseudogracile*); 867a (*americanum*); 874 (*pseudogracile*); 2136, 18367 (*emulans*).

Molepo, L. 18 (*retroflexum*).

Molina M, A. 7 (*nigrescens*).

Molina R, A. 16121, 25682, 26647, 27097, 31802, 31969, 32195 (*nigrescens*); 35125 (*americanum*).

Molina, A. 209 (*americanum*).

Molina, A.M. 257, 2323 (*nitidibaccatum*).

Molinari, E. 186 (*chenopodioides*).

Moll, E.J. 4138 (*scabrum*); 5119 (*americanum*).

Moller, A.F. 2444 (*nigrum*).

Molyneux, W.E. 245 (*nitidibaccatum*).

Monachino, J.V. 176 (*nitidibaccatum*).

Monod, T. 11733, 11884 (*scabrum*).

Monro, A.K. 2916, 3953, 6415 (*americanum*).

Monro, C.F.H. 1024 (*retroflexum*).

Monroy, V.E. 18 (*douglasii*).

Monteiro, O.P. 76-886 (*americanum*).

Monteiro, T. dos PSACF_EX3733 (*americanum*).

Montenegro, E. 1439 (*nigrescens*).

Montero O, G. 212, 5618 (*furcatum*).

Monterrosa, J. 910 (*nigrescens*).

Montes, J.E. 943, 2143, 2324, 16065 (*americanum*).

Montgomery, F.H. 337 (*emulans*).

Montz, G.N. 2085 (*nigrescens*); 2937 (*pseudogracile*); 2947, 2961, 6993, 8410, 8429, 8884 (*nigrescens*).

Moodie, M.E. 111, 111, 1208 (*triflorum*).

Mooney, H.F. 2236, 2473 (*americanum*); 3160, 5743, 5858, 6680 (*villosum*); 22361 (*americanum*).

Moore, A.D. 1461 (*nigrescens*).

Moore, A.H. 331, 1998 (*emulans*).

Moore, C.E. B28 (*emulans*).

Moore, C.W.E. 3055 (*triflorum*); 9158, 9391, 9450 (*americanum*).

Moore, G.C. 327 (*americanum*).

Moore, H.E. 127 (*nigrescens*); 563 (*emulans*); 1392 (*nigrescens*).

Moore, J. 18989, 24439 (*nitidibaccatum*).

Moore, J.W. 10235, 22273 (*emulans*).

Moore, L.M. 3996 (*triflorum*).

Moorthy, S. AC-341 (*nigrum*).

Mora G, E. 196 (*nigrescens*).

Mora O, L.E. 666 (*nigrescens*).

Mora, G. 105 (*nigrescens*); 124 (*americanum*).

Morales Garcia, M.A. 43 (*nigrescens*).

Morales Lopez, B. 38 (*nigrescens*).

Morales Q, J.F. 150 (*nigrescens*); 8597 (*americanum*); 9200 (*nigrescens*); 9942 (*americanum*); 10888 (*nigrescens*); 14736, 14748 (*americanum*); 16084 (*macrotonum*).

Morales, [?] 25 (*macrotonum*).

Morales, J.F. 150 (*macrotonum*).

Morales, M. 3985 (*corymbosum*).

Moran, M.A. 142, 143 (*emulans*).

Moran, R. 688 (*douglasii*); 4978 (*nigrum*); 6448 (*americanum*); 6602, 7299 (*douglasii*); 7668 (*nigrescens*); 7781 (*macrotonum*); 11291 (*nitidibaccatum*); 20118 (*americanum*); 20503 (*douglasii*).

Moran, R.C. 6602 (*douglasii*); 6988 (*nigrescens*); 7781[b] (*americanum*).

Moran, R.V. 4978, 6914 (*americanum*); 20412 (*douglasii*); 29534 (*americanum*).

Moreira, E. 384 (*americanum*).

Morel, I. 2740, 2857, 2930, 3466, 3749, 5782 (*americanum*).

Moreno B, L. .M. 343 (*nigrescens*).

Moreno C, P. 1325B (*nigrescens*).

Moreno G, S. 86 (*nigrescens*); 273 (*douglasii*).

Moreno, P. 301 (*macrotonum*).

Moreno, P.P. 318 (*nigrescens*); 347, 820 (*americanum*); 837, 1040, 1096 (*nigrescens*); 1312 (*douglasii*); 1455, 1683 (*nigrescens*); 2628, 2752, 2990 (*americanum*); 4397 (*nigrescens*); 5242, 6011 (*douglasii*); 6236, 6251 (*nigrescens*); 6691, 7070 (*americanum*); 7130 (*douglasii*); 7189, 7402 (*nigrescens*); 8142 (*americanum*); 8400, 8440, 8484 (*nigrescens*); 8653 (*douglasii*); 9688, 9949B (*nigrescens*); 9975, 10181, 10561, 10998 (*americanum*); 11139, 11348 (*nigrescens*); 11374 (*douglasii*); 11744 (*nigrescens*); 12656 (*douglasii*); 13499, 13696, 14082, 16902, 17433, 17865, 18337, 22011 (*nigrescens*).

Morero, R.E. 110 (*chenopodioides*).

Moretti, C. 1168 (*americanum*).

Morgan, D. 862 (*emulans*).

Mori, S.A. 91, 1998, 7793 (*nigrescens*); 10992, 11624 (*americanum*).

Moriarty, V.K. 1513, 1538 (*americanum*).

Moritz, J.W.K. 1643 (*macrotonum*).

Morley, B.D. 870 (*macrotonum*).

Morley, G.E. 1296 (*emulans*).

Morley, M. 680 (*americanum*).

Morong, T. 262 (*americanum*); 1104 (*furcatum*).

Morris, D.I. 86418 (*nitidibaccatum*).

Morris, E.R. 581 (*triflorum*).

Morris, M. 676 (*emulans*).

Morrison, A. 14228 (*nigrum*).

Morrison, J.L. 1882 (*americanum*).

Morrone, O. 646, 1286 (*americanum*).

Morse, C.A. 13308 (*interius*).

Morton, C.V. 2658, 4923 (*americanum*).

Morton, J.K. 48, K243 (*scabrum*); A-435, SL-892 (*americanum*); K1398 (*scabrum*); A-1796 (*americanum*); A-2383, A-3675, A-4028, A4277 (*scabrum*); 8101 (*americanum*); NA-8684 (*emulans*); GC-24359 (*scabrum*).

Moscone, A. 84, 105 (*chenopodioides*).

Mosén, C.W.H. 4483 (*americanum*).

Moss, C.E. 13307 (*chenopodioides*).

Mossman, E.L. 68 (*americanum*).

Mott, P.J. 236C (*retroflexum*).

Motta, J.T. 98, 101, 1115, 1198 (*americanum*).

Motte, M.E. 66, 169a (*douglasii*).

Motzkin, G. HF2005-645 (*emulans*).

Mouhair, F. 98-7 (*nigrum*).

Moulton, L.A. 7 (*americanum*).

Moura-Júnior, E.G. 8 (*americanum*).

Mow, D.M. 282, 317 (*emulans*).

Moyer, L.R. 230 (*triflorum*).

Moyle, J. 842 (*nitidibaccatum*).

Mr Drake 44 (*villosum*).

Mrkvicka, A.C. 1964, 4638 (*villosum*).

Mshasha, E. 144 (*scabrum*).

Muasya, J. GBK003/008/, 628, 729 (*villosum*).

Muehlenbach, V. 392 (*emulans*); 729 (*nitidibaccatum*); 1079 (*emulans*); 1436 (*sarrachoides*); 2493B, 3594, 3721, 3728, 3793, 4395 (*emulans*).

Mueller, C.H. 133, 8155 (*nigrescens*).

Muenscher, W.C. 8397 (*nitidibaccatum*); 14712 (*douglasii*).

Muir, T.B. 2789 (*triflorum*).

Mukerjee, S.K. 5016 (*americanum*).

Mukherjee, A. MN-2462 (*nigrum*).

Mulford, I. 460 (*triflorum*).

Müller, G.K. 3639 (*nitidibaccatum*).

Múller, P.J. 54 (*retroflexum*).

Muller, T. 743 (*retroflexum*).

Müller-Hohenstein, K. 544 (*villosum*).

Munn-Estrada, X. 841 (*americanum*).

Muñoz C, E. 301, 327 (*nigrescens*).

Muñoz, E. 301 (*nigrescens*).

Munton, P.N. 17 (*villosum*).

Munz, P.A. 6605, 12190 (*douglasii*).

Murata, G. T-14870 (*americanum*); 20136 (*nigrum*); 39046, 6303595 (*americanum*).

Murata, J. 25627 (*nigrum*).

Murcia, C. 475 (*nigrescens*).

Murfet, D.E. 3676 (*triflorum*).

Murillo R, F. 276 (*americanum*).

Murphy, H. 314, 475 (*americanum*).

Murphy, M.J.C. 800, 3091, 5072 (*emulans*).

Murray, K.G. 811021 (*nigrescens*).

Murray, S. SM-39-98 (*triflorum*).

Murrieta, Y. 56 (*nigrescens*).

Murti, S.K. NC-62161 (*americanum*).

Murty, V.K. 56 (*americanum*).

Musk, H. 157 (*scabrum*).

Muth, C. 19 (*emulans*).

Mutimushi, J.M. 2820 (*scabrum*).

Mutis, J.C. 3569 (*nigrescens*).

Naczi, R.F.C. 13727 (*emulans*).

Nafday, K.U.R. 30 (*americanum*).

Naganuma, R. 01 (*americanum*).

Naguib, M.I. 9 (*nigrum*).

Nair, N.C. NC-1099 (*americanum*); NC-2034 (*villosum*); ANC-6250 (*americanum*); NC-16121, NC-18954 (*nigrum*); NC-20005, NC-21756 (*villosum*); NC-22000 (*americanum*); NC-22166, NC-22215, NC-22480 (*villosum*); NC-23125 (*nigrum*); NC-24678 (*americanum*); NC-24764 (*nigrum*); NC-25266 (*villosum*); NC-26044 (*nigrum*); NC-26179 (*americanum*); NC-27506 (*nigrum*); NC-29874 (*americanum*); NC-32949 (*villosum*); NC-35793 (*americanum*); NC-36383 (*nigrum*); 61452 (*americanum*).

Nair, V.J. NC-14732 (*nigrum*); 14778 (*villosum*); NC-19915 (*americanum*); NC-19972, NC-21618 (*villosum*); NC-23226, 57128 (*americanum*).

Naithani, B.D. II-210, NC-43928, NC-53810, NC-68002 (*villosum*).

Naithani, H.B. 213 (*villosum*); 6018 (*americanum*).

Nalhoiba, C.L. NC-51540 (*villosum*).

Nantinjal, K.C. 25421 (*nigrum*).

Napier, E.R. 135 (*villosum*).

Narave F, H. 1184, 1275 (*nigrescens*).

Narayanaswami, V. 3339 (*nigrum*).

Narváez Montes, M. 102 (*douglasii*).

Narváez S, E. 3927 (*douglasii*).

Narváez, E. 3371 (*nigrescens*).

Nash, E. 68 (*villosum*).

Nash, G.V. 302 (*emulans*); 1250 (*pseudogracile*).

Nash, R. R-4 (*nigrescens*).

Nasher, A.K. 26/85, IH126 (*villosum*).

Nath, B.K. 13362 (*americanum*).

Nation, O. 49 (*retroflexum*).

Nava Rodríguez, V. 80 (*americanum*); 85 (*douglasii*).

Ndegwa, J. 177 (*villosum*).

Neale, J.J. 3878 (*emulans*).

Nealley, G.C. 339(101) (*nigrescens*).

Neck, J. 72B (*americanum*).

Necker, W.L. 16, 326 (*americanum*).

Nee, M. 255 (*douglasii*); 3397, 3407, 3435 (*americanum*); 3462 (*emulans*); 3494, 3621 (*americanum*); 3785 (*nigrescens*); 3870 (*macrotonum*); 3923 (*nigrescens*); 3966 (*americanum*); 4021 (*macrotonum*); 4043 (*americanum*); 4138 (*macrotonum*); 4176 (*americanum*); 4705, 4710 (*emulans*); 4929, 7651, 8586, 9081, 9182, 9544 (*americanum*); 10016 (*macrotonum*); 11323, 14004, 14020 (*nigrescens*); 14121 (*americanum*); 14132 (*nigrescens*); 14623b, 14623, 14629b (*scabrum*); 14678 (*chenopodioides*); 16020 (*emulans*); 16063 (*scabrum*); 16069, 16073 (*nigrum*); 16081, 16088 (*scabrum*); 16093 (*chenopodioides*); 16099 (*scabrum*); 16672, 16674 (*americanum*); 16839 (*macrotonum*); 16872, 16902 (*nigrescens*); 17003 (*macrotonum*); 18011 (*emulans*); 18112 (*chenopodioides*); 18239 (*villosum*); 18248 (*scabrum*); 18259 (*retroflexum*); 18510, 18718 (*nigrescens*); 19522 (*americanum*); 19535, 19727, 20069 (*nigrescens*); 21772, 21874, 21933, 22013b (*emulans*); 22165, 22356, 22817, 23310, 23556, 23696, 23821, 23899, 24122 (*nigrescens*); 24369 (*emulans*); 24663, 25198 (*nigrescens*); 25542, 25602, 25715 (*emulans*); 27524 (*scabrum*); 27876, 29987 (*nigrescens*); 30110 (*nigrum*); 30111 (*emulans*); 30237, 30257, 30275, 30488,

31258, 31331, 31350, 31415, 31508, 31813, 31899, 32011 (*americanum*); 32196 (*nigrescens*); 32519 (*americanum*); 32945 (*nigrescens*); 33342, 33520, 34965 (*americanum*); 35905 (*emulans*); 36780 (*nigrum*); 37163, 38763, 42350, 42564 (*americanum*); 43042 (*nitidibaccatum*); 43613 (*emulans*); 45551 (*nigrum*); 45983 (*americanum*); 47396 (*sarrachoides*); 50082 (*americanum*); 50960 (*nigrum*); 50966, 51036 (*emulans*); 51080 (*nitidibaccatum*); 52967 (*americanum*); 53365 (*nigrum*); 53366, 53508 (*emulans*); 53590, 54632 (*americanum*); 54714 (*nigrum*); 54867, 55229 (*americanum*); 55498 (*emulans*); 55538 (*nigrum*); 57020 (*villosum*); 57198 (*nigrum*); 57199 (*emulans*); 57207 (*nigrum*); 59426, 59444 (*emulans*); 60196 (*nigrum*); 60201, 60203, 60214 (*americanum*); 60216 (*pseudogracile*); 60220, 60223 (*americanum*); 60224 (*pseudogracile*); 60226, 60228, 60235, 60237, 60242, 60246, 60247, 60248, 60249, 60259, 60270, 60276 (*americanum*); 60279, 60281, 60292 (*pseudogracile*); 60311, 60371, 60433 (*emulans*); 61297 (*interius*); 61306 (*emulans*); 61337, 61349, 61350, 61355 (*interius*); 61357, 61510 (*emulans*).

Neese, E.J. 15025 (*nitidibaccatum*); 15285, 16050 (*triflorum*); 16115 (*nitidibaccatum*).

Neff, J.L. 93-4-15-1 (*nigrescens*).

Negri, G. 136, 307, 563 (*villosum*).

Negrón Bonilla, L.A. 2 (*americanum*).

Neldner, V.J. 3987 (*americanum*).

Nelson, A. 1141 (*triflorum*); 1725 (*douglasii*); 2804 (*nitidibaccatum*); 8076 (*triflorum*); 10326, 11220a (*douglasii*).

Nelson, B. 150 (*americanum*).

Nelson, B.E. 1439, 10240, 11574, 11955, 13222, 28250, 31854, 31962, 32696, 32805, 33441, 33994, 34132, 34233, 34233, 39204, 39235, 39658, 39998, 40098, 40105, 52148, 53418, 53573, 56407, 58088, 58106, 59279, 69649, 69749, 81168, 82530 (*triflorum*).

Nelson, B.W. 782 (*americanum*).

Nelson, C. 3166, 7052 (*nigrescens*).

Nelson, D.J. 523 (*nigrum*).

Nelson, E.B. 7700 (*nigrescens*).

Nelson, E.W. 169 (*nigrescens*); 191 (*americanum*); 249, 578, 725 (*nigrescens*); 1130 (*douglasii*); 1679, 3065a (*nigrescens*); 3130, 3179 (*douglasii*); 3319 (*nigrescens*); 3868 (*americanum*); 4168 (*nigrescens*); 4200 (*douglasii*); 6640, 7176 (*americanum*); 7549 (*douglasii*).

Nelson, J. 52775 (*triflorum*).

Nelson, J.B. 8945 (*pseudogracile*); 15508 (*nigrum*); 17318, 17646 (*emulans*); 20927 (*nigrescens*); 21366, 21602, 27255 (*emulans*); 28059 (*chenopodioides*).

Nelson, J.C. 858, 1729 (*nitidibaccatum*); 1765 (*americanum*); 1776, 2927 (*nitidibaccatum*); 2942, 4779 (*triflorum*).

Neto, L. 14 (*americanum*).

Neubauer, H.F. 849, 3153 (*villosum*).

Nevers, G.C. de 7484 (*nigrescens*); 10240 (*nigrum*).

Neves, R. 222, 226 (*americanum*).

Nevling, L. 851 (*nigrescens*).

Nevling, L.I. 1288, 2004 (*nigrescens*).

Newberry, P.E. 202 (*villosum*).

Newbould, J.G.B. 83 (*villosum*).

Newby, J. ZP-51 (*villosum*).

Newlon, L.M. 100 (*douglasii*).

Newman, E.I. 170 (*villosum*).

Ngomba, J.N. 3 (*scabrum*).

Ngoni, J.F. 409 (*scabrum*).

Nicholas, A. 2369 (*retroflexum*).

Nichols, C.E. 2013 (*nigrescens*).

Nichols, G.E. 89 (*nigrescens*).

Nichols, W.F. 60, 62, 65 (*nigrum*).

Nicolas, F. 994 (*douglasii*).

Niederlein, G. 149, 286 (*nigrum*).

Nielsen, I. 230 (*nigrum*).

Nieuwland, J.A. 11598 (*emulans*).

Nixon, K.C. 974 (*douglasii*).

Niz, D. de 58 (*nigrescens*); 201 (*douglasii*).

Njoroge, S.J. S4 (*villosum*).

NMK 18 (*villosum*).

Noblick, L.R. 4260 (*americanum*).

Nobs, M.A. 50 (*americanum*); 647 (*douglasii*); 1003 (*americanum*).

Nohan, A. 312, 13505 (*americanum*).

Nolan, M.E. 19 (*americanum*).

Nordström, Å. 129 (*villosum*); 572 (*nigrum*).

Norlindh, T. 4199 (*retroflexum*).

Norman, C. 119 (*americanum*).

Norman, J.K. UNK-9 (*americanum*).

Norris, D.H. 12637 (*douglasii*).

Norris-Rogers, A.J. 599 (*retroflexum*).

Northrop, J.I. 557 (*americanum*).

Norton, H.E. 35 (*douglasii*).

Norton, J.B. 362 (*interius*).

Novelo R, A. 752 (*americanum*).

Nunes, T.S. 1398 (*americanum*).

Nunes, V.F. 156-7 (*americanum*).

Núñez V, P. 6017, 6490, 8601, 8764, 9514, 12319, 20824 (*americanum*).

Nusbaumer, L. LN1674 (*americanum*).

Nuttall, L.W. 35, 139, 203, 494, 703 (*douglasii*).

Nuvunga, A. 256 (*americanum*).

Nyakundi, D. 728 (*villosum*).

O'Conner, C. 29 (*triflorum*).

O'Donell, C.A. 4490 (*chenopodioides*).

O'Donnell, C.A. 1140 (*nitidibaccatum*).

O'Farrell, J.T. 72-253 (*triflorum*).

O'Keefe, J. 37 (*americanum*).

O'Neal, C.E. 41-141-1 (*emulans*).

O'Ryan, K. 39, 39 (*chenopodioides*).

Oberneder, R. 6650 (*villosum*).

Oberti, J.C. CORD297 (*triflorum*).

Obregón B, R. 17C911 (*americanum*).

Ocampo, R.Á. 1145, 1595 (*americanum*); 1612 (*nigrescens*); 3574 (*americanum*); 3719, 3728 (*nigrescens*).

Occhioni, E.M. 526 (*americanum*).

Occhioni, P. 459 (*americanum*).

Ochoa, C.M. 2688 (*furcatum*); OCH11161, OCH11781 (*macrotonum*); OCH14625 (*corymbosum*); 15043 (*americanum*).

Ochoa, M. 85 (*nigrescens*).

Ochoterena, H. 931 (*americanum*); 932 (*douglasii*); 933 (*americanum*).

Ochs, C. 238 (*emulans*).

Octjabreva, N. 5887 (*nigrum*).

Odewo, T.K. 130, 151, 157, 159 (*scabrum*); 592 (*americanum*); 727 (*scabrum*).

Oehmcke, A. 95 (*emulans*).

Oettinger, F.W. 831 (*douglasii*).

Ogden, F.H. 11 (*villosum*).

Ogle, C.C. 5928 (*nitidibaccatum*).

Ohashi, H. 774285 (*nigrum*).

Ohba, H. 8310439, 85-30831 (*nigrum*); 8350528, 8350558 (*villosum*).

Ohlinger, L.B. 454 (*pseudogracile*); 1396 (*chenopodioides*).

Oldeman, R.A.A. 22 (*scabrum*).

Oldham, M.J. 7928, 17976, 18341, 21325 (*emulans*).

Oldham, R. 236, 336, 575, 852 (*nigrum*).

Olet, E.A. 1 (*americanum*); 40 (*villosum*); 48, 49 (*scabrum*); 66 (*americanum*); 88 (*scabrum*).

Olivas Matey, C.M. 138 (*americanum*).

Oliveira de Britto, Y.L. 242 (*americanum*).

Oliveira Filho, N.E. 74 (*americanum*).

Oliveira, D.G. 292 (*americanum*).

Oliveira, D.M. 142 (*americanum*).

Oliveira, J.S. 36 (*americanum*).

Oliver, R.L. 790a (*douglasii*); 2677 (*americanum*).

Olmstead, R.J. 2015-54 (*sarrachoides*); 2015-55 (*nigrum*).

Olorunfemi, J. FHI-57034 (*scabrum*).

Olsen, E. 16 (*emulans*).

Olson, T. 11 (*triflorum*).

Omar, S. NHI-50389 (*villosum*); NHI-50421 (*nigrum*).

Ommanney, H.T. 140 (*retroflexum*).

Onishi, E. 33 (*americanum*).

Oosterhoorn, M. 230 (*nigrescens*).

Oosterzee, P. van 15 (*americanum*).

Opler, P.A. 906, 1605 (*americanum*).

Orchard, A.E. 3351 (*americanum*); 3364 (*nigrum*); 3412 (*americanum*); 3413 (*nigrum*); 3435, 3454 (*americanum*); 3499, 3639 (*nigrum*); 3680 (*americanum*); 3917, 3948, 5024 (*nigrum*).

Orcutt, C.R. 594 (*americanum*); 6786 (*nigrescens*).

Ordóñez, M.J. 68 (*americanum*).

Ordóñez, M.T. 14 (*americanum*).

Ordoñez, O. 14 (*americanum*).

Orejuela, A. 221, 694 (*nigrescens*); 696, 701, 762 (*macrotonum*); 2699, 2707, 2754, 2819, 2835, 2896 (*americanum*).

Orellana, R. 17 (*nigrescens*).

Orlandini, P. 182 (*americanum*).

Orozco, C.I. 1833 (*macrotonum*); 2045, 3374 (*nigrescens*); 3699, 3786, 3869 (*macrotonum*); 3914 (*nigrescens*).

ORSINI, [?] 27 (*nigrescens*).

Orststroom, S.J. van 13791 (*americanum*); 13867 (*nigrum*).

Ortega B, R. 1143 (*nigrescens*).

Ortega Lavariega, R. 443 (*nigrescens*).

Ortega O, R. 1143 (*nigrescens*).

Ortega Ortíz, R. 724 (*nigrescens*).

Ortega U, A. 324 (*nigrescens*).

Ortega, J.G. 4224 (*americanum*); 4259 (*douglasii*).

Ortega, L.C. de 123, 1164, 1528, 1828, 2096 (*americanum*).

Ortega, R. 236 (*nigrescens*).

Ortiz Calderón, A. 12 (*nigrescens*).

Ortiz V, E. 140, 1215, 1239 (*americanum*).

Ortiz, E.M. 1215, 1239 (*americanum*).

Ortiz, F. 1205 (*nigrescens*).

Ortíz, F. 1798 (*americanum*).

Ortiz, J.J. 979 (*nigrescens*).

Ortiz, M. 1121 (*americanum*).

Ortiz, N. 29 (*nigrescens*).

Ortíz, R.T. 1168 (*americanum*).

Ortiz, S.A. 135, 939 (*nigrescens*).

Osborne-Day, C. 55 (*americanum*).

Osorio Hernández, C. 41 (*nigrescens*).

Osten, C. 4506, 22526 (*sarrachoides*).

Ostenfeld, C.H. 5298 (*chenopodioides*).

Osterhout, G.E. 3360 (*triflorum*).

Oswald, P.H. 22 (*villosum*).

Otero, J.I. 165 (*americanum*).

Otis, I.C. 873 (*triflorum*); 2343 (*nitidibaccatum*).

Otobed, D.O. PW-10137 (*americanum*).

Ottley, A.M. 7 (*americanum*).

Over, W.H. 2834, 5153 (*emulans*).

Pabst, G. 4251 (*americanum*).

Pachano, A. 169 (*nitidibaccatum*).

Paciornick, E.F. 414 (*americanum*).

Packard, P.L. 77-112, 73-287 (*triflorum*).

Paddock, E.F. 104, 141 (*douglasii*); 161 (*americanum*); 163 (*furcatum*); 164 (*americanum*); 165 (*douglasii*); 167, 168 (*furcatum*); 169, 170, 410, 411, 413, 414 (*douglasii*); 416 (*nitidibaccatum*); 420 (*americanum*); 421, 422 (*douglasii*).

Páez V, J.A. 13 (*americanum*).

Paget, A. 2919 (*chenopodioides*).

Paguaga, L.D. 98 (*nigrescens*).

Paixão, J.L. 1542 (*americanum*).

Palacio, M. 23 (*macrotonum*).

Palacios Espinosa, E. 1061 (*nigrescens*).

Palacios, M.A. 749 (*chenopodioides*).

Palma G, J. 139 (*nigrescens*).

Palmatier, E.A. 378 (*triflorum*).

Palmer, E. 9 (*douglasii*); 11 (*americanum*); 27, 40 (*nigrescens*); 60[a] (*douglasii*); 60[b] (*americanum*); 60[c] (*nitidibaccatum*); 61 (*douglasii*); 101, 119, 167[b] (*nigrescens*); 167[a] (*douglasii*); 196[a] (*americanum*); 204, 261 (*douglasii*); 362[a] (*americanum*); 362[b] (*triflorum*); 363 (*americanum*); 369 (*pseudogracile*); 466 (*triflorum*); 554 (*nigrescens*); 647 (*douglasii*); 860 (*americanum*); 934 (*nigrescens*); 959 (*americanum*); 2067 (*douglasii*); 2689 (*americanum*).

Palmer, E.J. 257 (*emulans*); 554 (*americanum*); 3109, 3208, 3220, 3237 (*emulans*); 5884 (*triflorum*); 7658, 8776 (*nigrescens*); 12023 (*nitidibaccatum*); 12261 (*nigrescens*); 13882 (*triflorum*); 30751 (*douglasii*); 38000 (*triflorum*); 52976 (*sarrachoides*); 60872 (*nitidibaccatum*).

Palmer, P.R. D-7 (*americanum*).

Palmer, W. 141 (*emulans*).

Palzkill, D. 3 (*emulans*).

Pammel, L.H. 2 (*triflorum*); 586 (*emulans*); 3897 (*triflorum*).

Panatkool, M. 221, 322 (*nigrum*).

Pancho, J.V. 14990 (*americanum*).

Pandey, R.P. 4186 (*villosum*).

Pani, P.C. NC-80670 (*americanum*).

Panigrahi, G. CC-2132 (*nigrum*); CC-2839 (*villosum*); 6157 (*americanum*); 11246, 11606 (*villosum*); CC-12610 (*nigrum*); 15543 (*americanum*).

Panigrahi, S. 5778 (*nigrum*).

Pannister, C.W. 983 (*chenopodioides*).

Pant, P.C. NC-43450, NC-43694 (*americanum*); NC-43796 (*villosum*).

Paoli, G. 935 (*villosum*).

Pappi, A. 2901, 3188, 3772 (*villosum*); 4318 (*scabrum*); 4387 (*villosum*).

Parada, G.A. 1004, 1635 (*americanum*).

Parada-Gutierrez, G.A. 1635 (*americanum*).

Parangbye, N.P. Rustam-38 (*nigrum*).

Paredes, S. 23 (*macrotonum*).

Parfitt, B.D. 3309 (*douglasii*).

Paris, E.G. 279 (*villosum*).

Parish, S.B. 321, 5295 (*douglasii*); 9405 (*americanum*).

Parishani, M.R. 14098 (*villosum*).

Park, B.C. 3, 519 (*villosum*).

Parker, C. O88, E558 (*villosum*).

Parker, K.F. 7640 (*triflorum*).

Parker, R. 77-64, 69-180, 69-253 (*emulans*).

Parmar, P.J. AC-8623 (*nigrum*); 9573 (*villosum*).

Parnell, C. 45 (*nigrescens*).

Paroisse, G. 10 (*americanum*).

Parra O, C. 632 (*macrotonum*).

Parris, B.S. 12071 (*nigrum*).

Parry, C.C. 634, 1010a (*nigrescens*).

Parslow, R. SV-92781115 (*nitidibaccatum*).

Pastore, G.J. 1185 (*chenopodioides*).

Pataskan, R.D. 104144 (*villosum*).

Patel, I.H. 204 (*americanum*).

Patiño, D. 4 (*americanum*).

Patoni, C. 527 (*americanum*).

Patrick, S.J. 5063 (*americanum*); 5066 (*chenopodioides*).

Patzlaff, R. 55 (*americanum*).

Paul, T.K. CNH-43603 (*villosum*); 43603, 43752 (*americanum*).

Paula, C.H.R. de 277, 621 (*americanum*).

Paula-Souza, J. 6076, 7178 (*americanum*).

Pauwels, L. 5099 (*americanum*).

Pawek, J. 8205 (*scabrum*).

PBG 184 (*nigrum*).

Pearce, R.D. 154, 155 (*chenopodioides*); 156 (*americanum*).

Pearson, H.H.W. 9834 (*retroflexum*).

Pease, A.S. 1167, 1168, 12474 (*emulans*); 19871 (*nitidibaccatum*); 29706 (*emulans*).

Peck, M.E. 189 (*nigrescens*); 6920, 6923, 6924 (*triflorum*); 8605 (*americanum*); 9059 (*furcatum*); 9750 (*nitidibaccatum*); 10000 (*triflorum*); 10712 (*nitidibaccatum*); 15307 (*triflorum*).

Peden, A. 3 (*triflorum*).

Pedersen, T.M. 385, 3079 (*americanum*); 7253 (*sarrachoides*); 8252 (*chenopodioides*); 10890 (*americanum*); 12514 (*chenopodioides*); 12893 (*sarrachoides*); 13868 (*americanum*); 15379 (*nitidibaccatum*).

Pedley, L. 2774, 5024 (*americanum*); 5403 (*chenopodioides*); 5816 (*americanum*).

Pedra do Cavalo, G. 80 (*americanum*).

Pedraza F, M. 10 (*nigrescens*).

Peebles, R.H. 7430 (*triflorum*); 13085 (*nitidibaccatum*).

Peirce, G. 9-5 (*emulans*).

Peirson, F.W. 282 (*douglasii*); 6878 (*triflorum*); 11812 (*americanum*).

Peña-Chocarro, M.C. 1489, 1529 (*chenopodioides*).

Penard, E. 373 (*triflorum*).

Pengelly, R. 796 (*triflorum*).

Pennell, F.W. 3388 (*nigrescens*); 6611, 6955 (*macrotonum*); 12663 (*furcatum*); 14460, 16799 (*americanum*); 17441 (*nigrescens*); 19649 (*douglasii*).

Pennington, C.W. 42 (*douglasii*).

Pensiero, J. 2741 (*americanum*).

Penther, A. 1846 (*retroflexum*).

Perdomo, R. 22 (*americanum*).

Perea, J. 3413 (*americanum*).

Pereira, A. 533 (*americanum*).

Pereira, E. 1810, 4098 (*americanum*).

Pereira, S.C. 1511A (*chenopodioides*).

Pérez Arbeláez, E. 6336 (*americanum*).

Pérez C, E. 2795 (*nigrescens*).

Pérez G, I. 994 (*douglasii*).

Pérez Gómez, M. 76, 159 (*nigrescens*).

Pérez López, J. 908 (*nigrescens*).

Pérez M, A. 13-, 67, 83, 130 (*nigrescens*).

Pérez, A.J. 8163, 8281 (*macrotonum*).

Pérez, B. 19, 86 (*americanum*); 207 (*sarrachoides*); 263, 675 (*americanum*).

Pérez, L. 196 (*americanum*).

Pérez-Garcia, E. 991 (*nigrescens*).

Perret, H. 1765 (*villosum*).

Perrottet, G.S. 555, 556 (*scabrum*); 903[a], 904 (*americanum*).

Perry, G. 255 (*americanum*).

Perry, W. 2 (*americanum*).

Perumal, P. 16700, 16724 (*americanum*).

Pérusse, Y. 77400 (*emulans*).

Peters, R. 154 (*emulans*).

Peterson, A. 256 (*emulans*).

Petetin, C.A. 1575 (*americanum*).

Petit, A. 34, 235 (*emulans*).

Petrak, F. 676 (*nigrum*).

Petrino, M. 7 (*emulans*).

Petruscu, C. 2465 (*triflorum*).

Petterson, B. 62-146 (*villosum*).

Pettersson, B. 62-199 (*scabrum*).

Phelps, O.P. 843, 844, 845 (*emulans*).

Phengklai, C. 3085 (*americanum*).

Philbrick, R.N. B64-267b- (*douglasii*).

Philcox, D. 1994 (*emulans*); 3986, 4647 (*americanum*).

Philippi, F. 742 (*furcatum*).

Philippi, R.A. 278 (*furcatum*).

Philipson, W.R. 10168 (*americanum*).

Phillippe, L.R. 33576, 36787, 37712, 37883, 38342, 38387, 39060, 40095, 41277 (*emulans*).

Phillips, A.M. 9372 (*americanum*).

Phillips, M.E. 88, 933, 1047, 1064 (*americanum*).

Phillipson, P.B. 1077 (*retroflexum*); 1353 (*sarrachoides*); 1354 (*retroflexum*); 3272 (*americanum*); 4757 (*retroflexum*); 4784 (*villosum*); 5256 (*sarrachoides*).

Phipps, J.B. 1368, 2653 (*scabrum*).

Picado, A. 257 (*macrotonum*).

Piccinini, B.G. 2000 (*nitidibaccatum*); 3430 (*americanum*).

Pickard, J. 1216 (*nigrum*); 1470, 2749, 2763, 2832, 2896, 2904, 2930, 3002 (*americanum*).

Pickett, F.L. 350 (*triflorum*).

Piekel, F.U. 719 (*nigrescens*).

Piemeisel, R.L. 30-543 (*triflorum*).

Piffard, F. 22 (*villosum*).

Pilz, G.E. 2108 (*scabrum*).

Pinder-Moss, J. 1374, 1395 (*nitidibaccatum*).

Pinkard, A.B. 41 (*nigrum*).

Pinto da Silva, A.R. 6754 (*chenopodioides*).

Pinto, G.C.P. 186, 196/84 (*americanum*).

Pinzl, A. 987, 2467, 8457, 11728 (*nitidibaccatum*).

Pinzl, A.P. 7748, 9245, 9766 (*triflorum*); 13707 (*nitidibaccatum*).

Piovano, G. 512 (*villosum*).

Piper, C.V. 1806 (*triflorum*).

Pipoly, J.J. 4530, 6279 (*nigrescens*); 6509 (*macrotonum*); 7319 (*americanum*).

Pire, S.M. 2054 (*chenopodioides*).

Pires, J.M. 9223 (*americanum*).

Pistarino, A. 744 (*nigrum*).

Pitard, C.J. 1755 (*villosum*).

Pittier, H.F. 148, 149 (*nigrescens*); 313 (*macrotonum*); 2095, 2438 (*nigrescens*); 2543 (*americanum*); 3041 (*nigrescens*); 3104 (*macrotonum*); 3271 (*americanum*); 3884 (*nigrescens*); 4237 (*macrotonum*); 4378, 6087 (*americanum*); 6092 (*nigrescens*); 6253 (*macrotonum*); 6333, 6981, 8281, 9948 (*nigrescens*); 10453, 10453 (*macrotonum*); 11634, 11790, 11793 (*nigrescens*); 12887 (*macrotonum*); 13544, 14681 (*nigrescens*).

Pivetta, [?] 930 (*americanum*).

Pizziolo, W. 47 (*americanum*).

Plackett, R.A. 40 (*douglasii*).

Plantenberg, P.L. 4 (*triflorum*).

Plaskett, R.A. 40 (*douglasii*).

Platt, J.W.G. 397, 512 (*nigrum*).

Platt, J.W.O. 397, 512 (*nigrum*).

Plowman, T. 3341 (*emulans*); 8013 (*macrotonum*).

Plowman, T.C. 2403 (*americanum*); 2621, 2622 (*furcatum*); 3871, 4346 (*macrotonum*); 5513, 8395, 8930 (*americanum*); 13322 (*nigrescens*).

Po, U.S. 12110 (*nigrum*).

Pochmann, R. 26, 45 (*emulans*).

Podlech, D. 8624 (*triflorum*); 11394, 11517, 12710 (*villosum*); 16842, 18676 (*nigrum*); 19449 (*villosum*); 19792, 19950, 32146 (*nigrum*); 32273 (*villosum*).

Podzorski, A.C. 1198 (*villosum*).

Poeppig, E.F. 156 (*furcatum*); 538 (*nitidibaccatum*); 1366 (*americanum*).

Poff, J.M. 103 (*douglasii*).

Pohl, J.B.E. 600, 2489, 5408 (*americanum*).

Pohl, R. 604 (*emulans*).

Poilane, E. 11791 (*americanum*); 20429 (*nigrum*).

Pojarkova, A. 6, 210 (*villosum*).

Polakowski, H. 36 (*nigrescens*).

Polgár, S. 2698 (*americanum*).

Polhill, E. 139 (*villosum*).

Polhill, R.M. 828, 974, 1207, 1686, 12188 (*villosum*).

Pollard, C.L. 190 (*nigrescens*); 650 (*emulans*).

Pollet, J. 752 (*triflorum*).

Polunin, O. 353, 1858 (*nigrum*); 4281, 13930 (*villosum*).

Ponce C, F. 328 (*nigrescens*).

Ponsonby, L. 26 (*scabrum*).

Pope, R. 38 (*emulans*).

Popov, G.B. PB17, 69131, GP7231, 18734, 18719 (*villosum*).

Popovkin, A.V. 317 (*americanum*).

Popper, V.S. 122 (*douglasii*).

Porter, C.L. 10792 (*americanum*).

Porter, D.M. 1577 (*furcatum*); 4067 (*americanum*); 4611 (*nigrescens*).

Porto, M.L. 2871 (*americanum*).

Post, G.E. 281 (*villosum*).

Pote, L. 114 (*nigrum*).

Potter, D. 1580, 7163 (*emulans*); 7372 (*nigrum*); 32721 (*emulans*).

Pottier, R. 36c, 171 (*americanum*).

Pounds, W.Z. 136 (*nigrescens*).

Poveda Á, L.J. 3097 (*americanum*).

Powell, A.M. 2576 (*nigrescens*); 3173, 3196 (*douglasii*); 7366 (*nigrescens*).

Powell, D.A. 410 (*nigrum*).

Powell, J. 1494 (*douglasii*); 1568 (*americanum*).

Powell, J.M. 804 (*americanum*).

Pozner, R. 584 (*triflorum*).

Pradhan, P. 126, 201 (*villosum*).

Prado, M. 34 (*americanum*).

Prain's collector 745 (*nigrum*).

Prance, G.T. 6261, 6797, 10131, 58736 (*americanum*).

Prasad, M. 39377 (*villosum*).

Prata, A.P. 2388, 2593 (*americanum*).

Press, J.R. 26 (*nigrum*); 169 (*villosum*); 515, 607, 623 (*nigrum*); 1166 (*villosum*); 1209 (*nigrum*); 1285 (*villosum*).

Preston, N.C. 37 (*villosum*).

Prévost, M.F. 3911 (*americanum*).

Price, T. 470, 554b (*douglasii*).

Prigge, B.A. 1591 (*triflorum*).

Pringle, C.G. 4948 (*nigrescens*).

Prior, A. 619 (*macrotonum*).

Proctor, G.R. 11443, 15277, 16962, 17630, 17826, 18369, 19020, 19115, 21179 (*americanum*); 23593 (*nigrescens*); 23681 (*americanum*); 24026 (*nigrescens*); 24759 (*americanum*); 26316, 28555, 29079, 29611, 32736 (*nigrescens*); 37365 (*macrotonum*).

Prokes, F. 5 (*villosum*).

Proyecto Pedregal de San Angel 68 (*douglasii*).

Prudhomme, E. 28 (*scabrum*).

Pruski, J. 2237, 3023 (*emulans*).

Puch, A. 24 (*nigrescens*).

Puig, H. 199 (*nigrescens*).

Pujupet, J. 1047 (*americanum*).

Puleston de 7631 (*americanum*).

Pullen, R. 43, 2600 (*triflorum*); 3964 (*chenopodioides*); 8005 (*americanum*).

Pulley, J. 1234 (*americanum*).

Purdie, R.W. 2001 (*triflorum*); 6559 (*americanum*); 6711 (*triflorum*); 7081, 8316 (*chenopodioides*); 8386, 9350 (*triflorum*).

Purepong, W. 122 (*emulans*).

Purer, E.A. 5389 (*douglasii*).

Purpus, C.A. 1736 (*nigrescens*).

Purseglove, J.W. P2712 (*scabrum*); P-6309 (*nigrescens*).

Pusalkar, P.K. NC-104639 (*americanum*).

Put, N. 3548, 4188 (*nigrum*).

Pyne, M. 88-225 (*emulans*).

Qaiser 154 (*villosum*).

Qisheng Ma 88-0804 (*nigrum*).

Qiu, H.-.L. 50 (*nigrescens*).

Quaintance, A.L. 713 (*nigrescens*).

Quarín, C. 357, 1148, 2369 (*americanum*).

Quarré, P. 3297, 3509 (*scabrum*).

Queiroz, E.P. 2161 (*americanum*).

Quesada H, A. 886 (*nigrescens*); 1293 (*macrotonum*).

Quesada, J. 457 (*americanum*).

Quesada, J.F. 457 (*americanum*).

Quevedo G, E. 33 (*americanum*).

Quevedo, F.L. 1794 (*americanum*).

Quezada, M. 451 (*nigrescens*).

Quibell, C.F. 1729 (*americanum*).

Quinlan, M.B. 51, 182 (*americanum*).

Quintas, F.J.D. 47 (*americanum*).

Quiram, H.G. 11 (*douglasii*).

Quirico, A.-.L. 7 (*americanum*).

Quiroz, S.L. 3840 (*corymbosum*).

Radcliffe, L. 30, 203 (*emulans*).

Radcliffe-Smith, A. 3884 (*villosum*).

Radford, A.E. 15136 (*emulans*); 15688 (*americanum*); 23416, 24169 (*emulans*).

Raechal, L.J. 29 (*emulans*).

Raes, N. 59 (*americanum*).

Ragazzi, V. 53, 158 (*americanum*).

Ragonese, A.E. 8715, 8750, 8751, 8785, 8794, 9017 (*triflorum*).

Rai, S.K. BSHC-24575 (*americanum*).

Raimundo, A.F. 838 (*americanum*).

Rainha, B.V. 6177 (*chenopodioides*).

Raizada, M.B. 7426 (*villosum*).

Rajeurlar 31057 (*americanum*).

Raju, D.C.S. BSHC-4088 (*americanum*).

Rakotozafy, A. 59 (*americanum*).

Ralfs, P.H. 718 (*chenopodioides*).

Ram, B. 2184 (*nigrum*).

Ramaley, F. 1310 (*triflorum*).

Ramamoorthy, T.P. 3593 (*americanum*).

Ramamurthy, K. 16047, 50680 (*americanum*).

Rambo, B. 29020, 42637, 43100 (*americanum*); 45745 (*chenopodioides*).

Ramírez B, A. 10 (*americanum*).

Ramírez H, A. 2 (*americanum*).

Ramírez P, R.R. 13078 (*nigrescens*).

Ramírez R, R. 836 (*americanum*); 926 (*nigrescens*); 926 (*douglasii*).

Ramírez, C. 6 (*nigrescens*).

Ramírez, D. 236 (*nigrescens*).

Ramirez, F. 397 (*nigrescens*).

Ramírez, G. 20 (*macrotonum*).

Ramirez, M. 94 (*nigrescens*).

Ramírez, M. 94 (*nigrescens*).

Ramírez, N. 993 (*macrotonum*); 2095 (*nigrescens*); 2670 (*americanum*).

Ramírez, R. 237 (*nigrescens*); 926 (*douglasii*).

Ramírez-Delgadillo, R. 2668 (*americanum*).

Ramos A, C.H. 119, 217 (*nigrescens*).

Ramos, C.H. 555, 1176, 2618 (*nigrescens*).

Ramos, J. 5972, 6211 (*macrotonum*).

Ramos, J.E. 442 (*americanum*); 6602, 7304, 7471 (*macrotonum*).

Ramos, M. 27573, 40353 (*nigrum*).

Ramos, V.L.J. 489 (*nigrescens*).

Ramosa Ventura, L.J. 1143 (*nigrescens*).

Ramsbottom, J. x7c, x19 (*villosum*).

Ramsey, G.W. 22577 (*emulans*).

Rand, E.L. 651 (*emulans*).

Rand, R.F. 171 (*retroflexum*).

Randolph, D. 316 (*douglasii*).

Randolph, L.F. 509, 1360 (*emulans*).

Randriamampionona, B. 692 (*scabrum*).

Ranga, D. 245 (*villosum*).

Rangel, O. 2496, 11269 (*nigrescens*); 12351 (*macrotonum*); 12405 (*nigrescens*).

Ranger, J.L. 907 (*emulans*).

Ranjan, [?] 45320 (*villosum*).

Rankin, M. 2749 (*americanum*).

Rao, A.S. NC-63640 (*americanum*); NC-63680 (*villosum*).

Rao, A.V.N. 18242 (*americanum*).

Rao, R.S. 7454, 7857 (*nigrum*); 10291, 10316, 10857, 10879, 17489 (*americanum*).

Rao, T.A. NC-444, NC-626 (*americanum*); 626, 848 (*villosum*); NC-1061 (*americanum*); NC-4975, NC-4975, NC-6584 (*villosum*); NC-6584[b] (*americanum*); NC-7346 (*villosum*); NC-11009 (*nigrum*).

Rasingam, L. ANC-25856 (*americanum*).

Ratkowsky, D.A. 359 (*nigrum*).

Ratter, J.A. R.4262 (*americanum*).

Rattray, J.M. 560 (*villosum*).

Rau, M.A. NC-1318 (*americanum*); NC-3543, NC-8126 (*villosum*); NC-12625 (*americanum*); NC-14592 (*villosum*).

Raup, H.M. 11641 (*nitidibaccatum*).

Raveill, J.A. 1682 (*sarrachoides*).

Raven, P.H. 1773 (*furcatum*); 2178A (*nigrum*); 6572 (*americanum*); 8175 (*furcatum*); 13961 (*douglasii*); 14582 (*nitidibaccatum*); 14635 (*americanum*); 17248 (*douglasii*); 19912 (*americanum*); 27250, 27702 (*emulans*).

Rawen, P.H. 16674 (*douglasii*).

Rawi, A. NHI-10215 (*villosum*); 11679 (*nigrum*); NHI-19683, NHI-19706, NHI-19809, NHI-26567, NHI-29033 (*villosum*).

Ray, G.P. 2516 (*villosum*).

Ray, J.D. 7315 (*emulans*); 10775 (*americanum*).

Raymond, F.H. 52, 121 (*douglasii*).

Raymond, M. 667, 7030 (*emulans*).

Raynal, A. 18654 (*americanum*).

Read, R.H. 312 (*emulans*).

Reading MSc Expedition 227 (*villosum*).

Reading University/BM Expedition 160 (*villosum*).

Rebman, J.P. 3175, 29073 (*douglasii*).

Rechinger, K. 278 (*americanum*).

Rechinger, K.H. 87, 3630 (*villosum*); 5640 (*nigrum*); 5710, 8060 (*villosum*); 8117, 8418 (*nigrum*); 13017, 16293, 16549, 16957 (*villosum*); 19350 (*nigrum*); 19498 (*villosum*); 24868 (*nigrum*); 27945, 28191, 28669, 29683, 29684, 29885, 30305, 30866, 31004, 34624 (*villosum*); 37815, 38210, 39047 (*nigrum*); 45775 (*villosum*); 61377, 61404 (*nigrum*).

Reddick, D. 190 (*pruinosum*).

Redfield, J.H. 543 (*triflorum*).

Redfield, R. 11 (*nigrescens*).

Redfields, J.H. 5883 (*triflorum*).

Redgen, B. 48 (*americanum*).

Reece, E. 22 (*emulans*).

Reed, C.F. 5407, 9008, 21692 (*emulans*); 32667 (*villosum*); 32789 (*chenopodioides*); 32796, 32880, 32891 (*villosum*); 39206 (*emulans*); 39318 (*sarrachoides*); 81849 (*emulans*); 102464 (*pseudogracile*); 103008 (*emulans*); 104036 (*villosum*); 104090, 104092 (*nigrescens*); 105503 (*emulans*); 105844 (*nigrum*); 113699, 134766, 140691, 141504, 143179, 146306 (*emulans*).

Reed, E.L. 3250 (*emulans*); 3546a (*triflorum*); 3555 (*interius*).

Reekmans, M. 9831, 9922 (*villosum*).

Rees, M. 302 (*nitidibaccatum*).

Reese, W.D. 3917 (*nigrescens*).

Refsdal, C.H. 2165 (*triflorum*).

Regnell, A.F. III-970 (*americanum*).

Rego, L.N.A.A. 6 (*americanum*).

Reiche, C.F. 69 (*furcatum*).

Reid, C. 4515 (*pseudogracile*); 5884, 5884 (*nigrescens*).

Reid, J.C. 2717 (*nitidibaccatum*).

Reijenga, T.W. 995 (*americanum*).

Reina G, A.L. 2001-51, 98-400 (*douglasii*); 97-446 (*americanum*).

Reina, A.L. 96-258 (*douglasii*).

Reina, G. 406 (*macrotonum*).

Reitz, P.R. 6.006 (*corymbosum*); c72, C693, 871, 2173 (*americanum*); 6066 (*corymbosum*); 6907 (*americanum*).

Reitz, R. 464 (*americanum*).

Reitzel, J. 4184 (*triflorum*).

Rekas, K. 290 (*nigrum*).

Reko, B.B. 3173 (*nigrescens*).

Renderos, M.A. 10 (*douglasii*).

Rendle, A.B. 71, 292, 396 (*americanum*).

Rendón, G. 5473 (*nigrescens*).

Renjifo, L.M. 227 (*nigrescens*).

Rentería A, E. 714 (*nigrescens*); 1624 (*americanum*).

Renvoize, S.A. 885 (*americanum*); 2312 (*scabrum*).

Resgate, E. 196 (*americanum*).

Reverchon, J. 482, 670 (*emulans*); 3241 (*pseudogracile*); 3916, 3917 (*nigrescens*); 3919 (*triflorum*).

Revilla, J. 11 (*americanum*).

Rex Smith, G. 9 (*villosum*).

Reyes García, A. 42, 1851, 1867, 2064, 4234, 4483, 4518 (*nigrescens*); 4947 (*americanum*); 5367, 5935, 6904, 7431 (*nigrescens*).

Reyes S, J. 192 (*americanum*); 1924a (*nigrescens*).

Reyes V, L. 13 (*douglasii*).

Reyes, J.R. 956 (*nigrescens*).

Reyes, P. 14270, 14356 (*americanum*).

Reyes-García, A. 2064 (*americanum*); 5935 (*nigrescens*).

Reynoso D, J.J. 635 (*americanum*).

Reynoso, E. PPI-21644 (*americanum*).

Reynoso, J.J. 2203 (*nigrescens*).

Reznicek, A. 1141 (*emulans*).

Reznicek, A.A. 6030 (*emulans*); 7454 (*triflorum*).

Rhodes, J. 272 (*emulans*).

Ribas, O.S. 6840B (*americanum*).

Ribeiro, B.G.S. 1391 (*americanum*).

Rice, K.C. 1146, 1316, 1587 (*douglasii*).

Rice, W. 646, 867, 1614, 1748, 1948 (*emulans*).

Rich, H.H. 26 (*villosum*).

Rich, N. 879 (*emulans*).

Richards, A.F. 149 (*americanum*).

Richards, H.M. Mrs 9775B, 12233, 16686, 18304 (*villosum*).

Richards, S.J. 77-483 (*triflorum*).

Richardson 73648 (*triflorum*).

Richardson, J.I. 27 (*pseudogracile*).

Riches, C.R. 25 (*americanum*).

Richey, L.R. 99-712 (*nigrescens*).

Richter, L. von 436 (*chenopodioides*).

Ricketson, J. 1861 (*triflorum*).

Ricksecker, A.E. 127 (*interius*).

Ricksecker, J.J. 251 (*americanum*).

Rico, V.M. 352, 355 (*nigrescens*).

Rico-Gray, V. 710, 711 (*nigrescens*).

Riddell, J.L. 1229 (*interius*).

Rider, S.L. 344 (*triflorum*).

Ridley, H.N. 77, 14983 (*americanum*).

Ridoutt, C.A. 11183a, 11520 (*corymbosum*).

Ried, A.S. 163 (*emulans*).

Riedel, L. 402[b] (*americanum*).

Ries, R.E. 165 (*triflorum*).

Rimachi Y, M. 518 (*nigrescens*); 4134, 7077, 8340, 8897, 10277, 10760, 11116, 11804 (*americanum*).

Rincker, P. 59 (*triflorum*).

Rincón G, A. 1998 (*americanum*).

Ripart, J. 249 (*nigrum*).

Ripley Jr, W.S. 15921 (*emulans*).

Rishoff, M. 437 (*triflorum*).

Risler, J. 970 (*americanum*).

Ritchie, [?] 508/2 (*nigrum*).

Ritter, N. 1124 (*americanum*).

Rivera D, O. 3234 (*nigrescens*).

Rivera H, J. 492, 661 (*nigrescens*); 2164 (*americanum*); 2412 (*nigrescens*); 3413 (*douglasii*); 3661 (*nigrescens*); 3708 (*americanum*).

Rivera Reyes, J. 219 (*nigrescens*); 415 (*americanum*); 956, 1114, 1298 (*nigrescens*); 2545 (*americanum*).

Rivera, G. 214 (*nigrescens*).

Robbins, F. ACB-50381 (*chenopodioides*).

Robbins, J.W. 74 (*emulans*).

Robbins, S.B. 6263 (*nigrescens*).

Robbrecht, E. 2857 (*nigrum*).

Robert, A. [Frère] 1353 (*emulans*).

Roberts, E.C. 11388 (*nitidibaccatum*).

Roberts, E.L. 519 (*triflorum*).

Robertson, J. 212 (*nigrescens*).

Robertson, K.R. 105 (*corymbosum*).

Robertson, S.A. 643 (*villosum*); 3454 (*americanum*).

Robertson, W.B. 54 (*pseudogracile*); 202 (*americanum*).

Robertson, W.E. 7407 (*triflorum*).

Robinson, B.L. 207 (*pseudogracile*).

Robinson, H.C. 86, 175 (*americanum*).

Robles G, R. 423, 593 (*nigrescens*).

Robles H, L. 23 (*nigrescens*).

Robles, L. 667 (*nigrescens*).

Robles, R. 1790 (*macrotonum*).

Robles, S.T. 584, 1860, 1898, 2096, 2139, 2277 (*chenopodioides*).

Robleto, W. 72, 1013 (*nigrescens*); 1039 (*americanum*); 1412 (*nigrescens*); 1412 (*americanum*); 1448 (*nigrescens*).

Rocha e Silva, I. 63 (*americanum*).

Rocha, E.A. 1722 (*americanum*).

Rockley, C.T. 4 (*chenopodioides*).

Rocrlach, K.D. 290 (*nigrum*).

Rodarte, A. 2E (*americanum*).

Rodd, A.N. 455 (*triflorum*); 688, 690, 691 (*americanum*); 1640 (*chenopodioides*); 1751 (*americanum*); 121632 (*chenopodioides*).

Roderick, A.J. 2084, 3293, 8296 (*triflorum*).

Rodin, B. 247 (*americanum*).

Rodin, R.J. 8555 (*triflorum*); 8737 (*douglasii*).

Rodrigues, J.E. 189 (*americanum*).

Rodríguez B, D. 101 (*americanum*).

Rodríguez C, A. 594 (*nigrescens*); 969 (*douglasii*); 1095 (*nigrescens*); 2128 (*douglasii*); 2133 (*americanum*); 4236 (*nigrescens*).

Rodríguez E, M. 140 (*macrotonum*).

Rodríguez G, A. 3297 (*americanum*); 6354, 7925 (*nigrescens*); 8780 (*americanum*); 10105, 10821 (*nigrescens*); 14051 (*macrotonum*).

Rodríguez M, G.M. 317, 962 (*nigrescens*).

Rodríguez, A. 4461 (*pruinosum*).

Rodríguez, D. 41, 1044 (*nigrescens*); 1315 (*macrotonum*); 1417 (*douglasii*); 1888, 2242, 2324 (*nigrescens*).

Rodríguez, L. 1249, 2316 (*nigrescens*).

Rodríguez, R. 4, 185 (*americanum*).

Rodríguez-Barquet, E. 115 (*nigrescens*).

Rodschied, E.C. 31 (*americanum*).

Rodway, F.A. 121 (*nigrum*); 735, 1092, 1334, 1377 (*americanum*); 1661 (*chenopodioides*); 2258, 2694 (*nigrum*); 4766, 6483, 6486, 6488, 6489, 6490, 6496, 11333 (*americanum*); 14904, 14940 (*chenopodioides*).

Roe, K.E. 290, 700, 1263 (*douglasii*); 1378 (*nigrescens*); 1494 (*douglasii*); 2320 (*americanum*).

Roe, R. 695 (*triflorum*).

Rogers, [?] 14 (*nigrum*).

Rogers, C.B.W. 276 (*nitidibaccatum*).

Rogers, C.M. 4462 (*triflorum*); 4485 (*emulans*); 4770 (*interius*).

Rogers, F.A. 08, 103 (*villosum*); 7402, 8256 (*scabrum*).

Rogers, K.E. 6884A, 7088 (*nigrescens*).

Roivainen, H. 75, 147 (*chenopodioides*); 2479, 2646, 2773 (*triflorum*); 2789 (*furcatum*).

Rojas M, M. 10 (*americanum*).

Rojas, R. 882 (*americanum*).

Rojas, T. 2493 (*sarrachoides*); 5975 (*americanum*).

Roldan, F.J. 1293, 1293 (*americanum*); 1704 (*nigrescens*); 2227 (*macrotonum*); 3276 (*nigrescens*).

Rolfs, P.H. 718 (*chenopodioides*).

Roll, C.M. 954 (*douglasii*).

Rolla, S.R. 88606 (*villosum*).

Rolland-Germain [Frère] 236, 558 (*emulans*).

Román, M.L. 978, 1267 (*americanum*).

Rombouts, H.E. 718 (*americanum*).

Romero, E. 70 (*americanum*).

Romero, L. 81 (*douglasii*).

Romero-Castañeda, R. 743 (*americanum*); 891, 2474 (*macrotonum*); 6386 (*americanum*); 6386, 6389, 6579, 6930 (*nigrescens*); 7446 (*macrotonum*); 8937 (*nigrescens*); 8961 (*macrotonum*); 9172, 10669, 11066, 11304 (*nigrescens*).

Romero-Rojas, [?] 778 (*nigrescens*).

Romo Díza, G. 1263 (*americanum*).

Ronchi, P.S. 461, 710 (*americanum*).

Rondeau, R. 134 (*americanum*).

Roque, J. 22, 87, 208 (*americanum*); 296 (*corymbosum*); 396, 693 (*americanum*).

Ros, [?] da 132 (*nigrescens*).

Rosa, N.A. 434, 2189, 3548 (*americanum*).

Rosário, C.S. 1878 (*americanum*).

Rosas R, M. 52, 770, 952, 1082 (*nigrescens*); 1167 (*americanum*).

ROSAS, [?] 26 (*nigrescens*).

Rosas, A. 30, 310 (*americanum*).

Roscoe, M.V. 11109 (*nigrum*).

Rose, F.H. 202, 202 (*triflorum*).

Rose, J.N. 116 (*triflorum*); 1461 (*nigrescens*); 3381, 3390, 8337, 8339, 8435, 8796, 9078, 9255 (*douglasii*); 9602, 9613 (*pruinosum*); 9670 (*nigrescens*); 12422 (*americanum*); 12541 (*douglasii*); 12951, 13152 (*nigrescens*); 13383 (*douglasii*); 13811, 14134, 14363 (*americanum*); 14596 (*douglasii*); 14697, 14877, 22532 (*americanum*).

Rose, L.S. 35009, 45326 (*douglasii*); 59161 (*nitidibaccatum*); 63155 (*douglasii*); 66088 (*americanum*).

Rosen, D.J. 5304 (*americanum*).

Rosengurtt, B. 775 (*sarrachoides*); 5783 (*chenopodioides*).

Rosentreter, R. 2459 (*triflorum*).

Ross, R. SAN128, SAN585 (*americanum*).

Ross, S. 670 (*emulans*).

Ross, T. 4110 (*douglasii*).

Ross, T.S. 3317 (*nigrum*); 4090, 5518, 6423 (*douglasii*).

Rossato, M. 3339, 3480 (*americanum*).

Rossbach, G.B. 1370, 3787, 3870, 3924, 4210, 10524 (*nigrum*).

Rossoni, M.G. 133, 633 (*americanum*).

Rossow, R. 5721 (*chenopodioides*).

Rothfels, C.J. 2046 (*emulans*); 2047, 2051 (*nigrum*); 2062 (*nitidibaccatum*).

Rothrock, J.T. 46 (*macrotonum*); 83, 388, 707 (*douglasii*).

Rothrock, P.E. 1823 (*emulans*).

Rousselle, J. 13526 (*nigrum*).

Roux, J.P. 2080 (*nigrum*).

Rowan, D. 299 (*triflorum*).

Rowell Jr, C.M. 4176 (*interius*); 4218 (*triflorum*).

Rowell, C.M. 4218 (*triflorum*); 10839 (*emulans*).

Rowlee, W.W. 897 (*nigrescens*).

Roy, C. 3 (*emulans*).

Roy, E. 3841 (*emulans*).

Roy, G.P. AC-3863 (*nigrum*); 5060 (*villosum*); CC-34072 (*nigrum*).

Roybal, J.J. 621, 1055, 2051 (*nigrescens*).

Royce, R.D. 5846, 8408 (*americanum*).

Royl, [?] 6826 (*villosum*).

Rozynski, H.W. von 515 (*douglasii*).

Rubio G, A. 68 (*douglasii*).

Rubio, H. 29 (*douglasii*); 2222 (*nigrescens*).

Rubtzoff, P. 10202 (*furcatum*).

Rudatis, H. 1805 (*retroflexum*).

Ruddle, K. 15 (*nigrescens*).

Rueda, R. 2030, 10753, 10873, 15236 (*nigrescens*).

Rueda, R.M. 13190 (*douglasii*).

Rugel, F. 44 (*americanum*); 69, 302 (*pseudogracile*).

Ruiru Pest Control Staff 354 (*villosum*).

Ruiz Huidobro, A.M. 1410 (*triflorum*); 1449 (*chenopodioides*).

Ruiz, E. 1007 (*furcatum*).

Ruiz-Terán, L.E. 511, 1557 (*macrotonum*); 3545 (*nigrescens*); 7329 (*macrotonum*); 8545 (*nigrescens*); 11959 (*americanum*); 12390, 14155, 15502 (*macrotonum*).

Runyon, R. 338, 450, 3210, 4841, 4843 (*nigrescens*).

Rusby, H.H. 145 (*nigrescens*); 247 (*triflorum*); 334 (*nigrescens*); 753, 783, 5981 (*triflorum*).

Russell, N.H. 66-49 (*triflorum*).

Russell, P.G. 46, 48, 102 (*douglasii*).

Russell-Smith, J. 5421 (*americanum*).

Rust, H.J. 426, 5455 (*triflorum*).

Ruth, A. 149, 511, 687, 3418 (*emulans*).

Rutile, A. 36 (*chenopodioides*); 66 (*americanum*); 108, 111, 137, 218 (*chenopodioides*).

Ruygt, J. 1880 (*americanum*).

Rwaburindore, P.K. 701 (*scabrum*); 1705 (*americanum*); 2992 (*scabrum*); 3936, 4199 (*americanum*).

Ryan, P. 289 (*scabrum*).

Rydberg, P.A. 150 (*emulans*); 265 (*triflorum*); 467 (*emulans*); 905 (*triflorum*); 906 (*interius*); 1021 (*emulans*); 1147 (*triflorum*); 1218, 1385 (*interius*); 1393, 1395, 4905, 7010, 8339, 8532, 9613 (*triflorum*); 9626 (*emulans*).

Ryding, P.O. 1128, 1536 (*villosum*).

Ryland, A. 3819 (*emulans*).

Rzedowski, J. 54 (*corymbosum*); 237 (*douglasii*); 6238, 8766 (*nigrescens*); 22943 (*corymbosum*); 28345, 28768, 28958 (*nigrescens*); 34725 (*douglasii*); 35329, 35441 (*nigrescens*); 36035 (*douglasii*); 36671 (*pruinosum*); 37059 (*douglasii*); 37140, 38040 (*nigrescens*); 39828, 40130

(*americanum*); 43280 (*nigrescens*); 46293, 49107 (*americanum*); 50841 (*nigrescens*); 51543 (*americanum*); 51817 (*nigrescens*).

Saab, S.A.G.P. 184 (*americanum*).

Sabaya, W.D. 34 (*villosum*).

Sabeti 1018 (*villosum*).

Sabnis, S.D. 568, 582 (*villosum*).

Saccardo, D. 35 (*villosum*).

Sachet, M.H. 309, 358 (*douglasii*); 2254 (*americanum*).

Sachse, B. 5 (*retroflexum*).

Sackschewsky, M.R. 269 (*triflorum*).

Safford, W.E. 1396 (*americanum*).

Safrui, B. 20 (*americanum*).

Sagástegui, A. 7828, 7854, 12289 (*corymbosum*); 16128 (*americanum*); 17235 (*corymbosum*).

Sagot, P.A. 453, 493 (*americanum*).

Sahebi 2047 (*villosum*).

Sahmi, K.C. 25025 (*americanum*).

Sainty, G.R. 329 (*triflorum*).

Sajtoj, I. 93-3084 (*nigrescens*).

Salama, A.M. 68 (*nigrescens*); 343 (*macrotonum*).

Salas M, S. 5044, 5678 (*nigrescens*).

Salas M, S.H. 1943, 3044, 3574, 3970 (*nigrescens*).

Salasoo, H. 937, 1815 (*chenopodioides*); 3333 (*americanum*).

Salavakan 3035 (*villosum*).

Saldanha, C.J. 13567 (*villosum*); 16110 (*americanum*).

Salgado, C. 14 (*macrotonum*).

Salinas, M.E.J. 74, 331, 502, 1466, 1598, 1787, 1865 (*nigrescens*).

Salm, H. 1 (*americanum*).

Salter, T.M. 9711 (*retroflexum*).

Salubeni, A.J. 4242 (*scabrum*); 4841 (*villosum*).

Salzmann, P. 391, 3855 (*americanum*).

Sampaio, A.F. 8955 (*americanum*).

Samuelsson, G. 1086 (*villosum*).

Sánchez G, E. 104 (*douglasii*).

Sánchez G, J. 1927 (*nigrescens*); 2784 (*americanum*).

Sánchez Garcia, E. 104 (*douglasii*).

Sánchez L, F. 529 (*nigrescens*).

Sánchez S, E. 516 (*nigrescens*).

Sánchez T, V. 325 (*douglasii*).

Sánchez Vega, I. 4600 (*corymbosum*).

Sánchez, G. 73 (*macrotonum*).

Sandberg, J.H. 985 (*triflorum*).

Sandeman, C.A.W. 5361 (*corymbosum*).

Sanders, A.C. 7053 (*douglasii*); 12462 (*nitidibaccatum*); 23119 (*americanum*); 37173 (*retroflexum*).

Sandino, J.C. 317 (*nigrescens*); 677 (*americanum*); 700, 730 (*nigrescens*); 777 (*americanum*); 875, 1005 (*nigrescens*); 2731 (*americanum*); 2860, 3830 (*nigrescens*).

Sandoval, [?] CS-115 (*nigrescens*).

Sandoval, E. 833 (*nigrescens*).

Sandoya-Sanchez, C.V. 563 (*macrotonum*).

Sands, M.J.S. 333 (*americanum*).

Sandwith, C.I. 3169a (*villosum*).

Sandwith, N.Y. 970 (*nigrescens*); 972, 1703 (*americanum*); 2395, 3413 (*villosum*).

Sanford, J. 16 (*emulans*).

Sanford, J.A. 57, 386 (*americanum*).

Sankey, H.J. 200 (*retroflexum*).

Sanou, L.F. BUR-596 (*scabrum*).

Santamaria A, D. 4834 (*nigrescens*).

Santamaría, D. 1844 (*americanum*); 2100, 2854, 3035, 3913 (*nigrescens*); 4273, 5783 (*americanum*); 5838 (*nigrescens*); 7554 (*macrotonum*).

Santana, B. 948 (*nigrescens*).

Santapau, H. 204 (*americanum*).

Sántiz C, E. 523 (*americanum*).

Santíz Cruz, E. 44, 492, 523, 630 (*nigrescens*).

Santíz Ruiz, C. 74 (*nigrescens*).

Santo, E. 218 (*scabrum*).

Santos Martínez, J. 1510 (*douglasii*); 1510[a] (*americanum*).

Santos, J.K. 32073 (*nigrum*).

Santos, L.A.S. 310 (*americanum*).

Santos, T.S. dos 3379 (*americanum*).

Sanyal, M.N. 649 (*villosum*).

Saran, R. 2429 (*villosum*).

Saravia T, C. 257, 908 (*nigrescens*); 1249 (*macrotonum*); 1435, 1883 (*nigrescens*); 2398, 3428 (*americanum*); 3692 (*nigrescens*).

Saravia, C. 3692 (*nigrescens*).

Sargent, H.E. 3828 (*emulans*).

Sargent, W.B. 46 S (*triflorum*).

Sarin, Y.K. NC-1501, NC-1509, NC-5159 (*americanum*); NC-8573 (*villosum*).

Särkinen, T. 4015, 4017, 4018, 4023, 4027, 4033, 4041 (*americanum*); 4066 (*corymbosum*); 4072 (*americanum*); 4075 (*corymbosum*); 4076 (*nitidibaccatum*); 4078 (*corymbosum*); 4080 (*americanum*); 4085 (*nitidibaccatum*); 4089 (*corymbosum*); 4113, 4116 (*americanum*); 4117 (*chenopodioides*); 4505 (*americanum*); 4509 (*corymbosum*); 4511, 4514, 4520, 4528, 4534, 4543, 4555, 4574, 4579 (*americanum*); 4604B (*corymbosum*); 4619, 4622, 4632, 4649 (*americanum*); 4687, 4792, 4793, 4795, 4802 (*corymbosum*); 4818, 4823, 4862, 4867 (*americanum*).

Sartori, J. 86 (*villosum*).

Sartori, M. 97 (*americanum*).

Sarukhan, J. 1183 (*nigrescens*).

Sasaki, S. 275 (*nigrum*).

Sastre, C. 2708 (*americanum*).

Sastry, A.R.K. 40638 (*nigrum*).

Saueressig, D. 1909 (*americanum*).

Saulleres, A. 63 (*americanum*).

Savatier, L. 875 (*nigrum*).

Savoie, T. 31 (*nigrescens*).

Saxena, H.O. 525, 582, 2159 (*americanum*).

Sayago, M. 796 (*nitidibaccatum*).

Saynes V, A. 2245 (*americanum*); 2503 (*nigrescens*).

Sayre, M. 20 (*corymbosum*).

Scarce, S. 281 (*nigrescens*).

Scarda, F.M. 27 (*americanum*).

Scarlett, N.H. 87-38 (*americanum*); 87-39 (*nigrum*).

Scatigna, A.V. 863 (*americanum*).

Schaeffer Jr, R.L. 25304 (*emulans*).

Schäfer, H. 5569, 9370 (*nigrum*).

Schafer, J.A. 3 (*americanum*).

Schäfer, P.A. 5306, 7053 (*americanum*).

Schäffler, Y. 55 (*americanum*).

Schaffner, J.G. 408, 691 (*pruinosum*); 692[a] (*americanum*); 692[b] (*nigrescens*).

Schallert, P.O. 3765 (*chenopodioides*).

Schantz, H.L. 646 (*scabrum*).

Schatz, G.E. 208, 210 (*nigrescens*); 1444 (*americanum*).

Scheepers, J.C. 54 (*retroflexum*); 281 (*americanum*).

Schery, R.W. 137 (*douglasii*).

Schiavone, M.M. 11681C (*sarrachoides*).

Schiede, C.J.W. 46 (*americanum*); 133 (*nigrescens*).

Schimper, G.H.W. 255, 2043 (*villosum*).

Schimpff, H.J.F. 218 (*americanum*).

Schindler, A.K. 1409 (*nigrum*).

Schinini, A. 4746 (*chenopodioides*); 5174, 5427, 6806, 6845, 11102, 11276 (*americanum*); 16377 (*sarrachoides*); 17370 (*americanum*).

Schinz, H. 806 (*retroflexum*).

Schipp, W.A. 842 (*nigrescens*).

Schlagintweit, H.A.R. von 4368 (*nigrum*).

Schlechter, F.R.R. 2165 (*scabrum*).

Schlieben, H.J. 3550 (*villosum*); 7009 (*retroflexum*); 11670 (*scabrum*).

Schlim, L.J. 194 (*americanum*).

Schlising, R.A. 586, 747 (*emulans*).

Schmidt, A.A. 592 (*scabrum*).

Schmitt, G. 163 (*americanum*).

Schmitt, W.L. 105 (*douglasii*).

Schmitz, A. 61 (*americanum*); 161 (*nigrescens*); 162 (*douglasii*); 696 (*nigrescens*); 1047 (*pruinosum*); 1655 (*corymbosum*).

Schneider, A.C. 445 (*douglasii*); 513 (*americanum*).

Schneider, C.K. 705 (*nigrum*).

Schnell, C.E. 1116 (*nigrescens*).

Schnetter, M.L. 162 (*nigrescens*).

Schoch, O. 67 (*nigrum*).

Schoenwetter, J. JSOX-58 (*americanum*).

Scholten, G. 38 (*nigrum*).

Schoolcraft, G. 1774 (*triflorum*).

Schori, A. 12-165 (*emulans*).

Schott, A.C.V. 811 (*nigrescens*); 5410 (*americanum*).

Schreibeis, R.G. E-617 (*triflorum*).

Schreiber, B. 2189 (*americanum*).

Schreiter, R. 7410 (*chenopodioides*).

Schroeber, C. 59 (*americanum*).

Schuette, B. 3088 (*emulans*).

Schultes, R.E. 27 (*macrotonum*); 5139 (*americanum*); 5700, 7265, 7266, 7566, 7582, 7767 (*macrotonum*).

Schultz, A.R. 212, 1071, 4019, 7802 (*americanum*).

Schultz, F.G. 705 (*villosum*).

Schultz, N. 166 (*chenopodioides*).

Schulz, E.D. 126, 438 (*nigrescens*).

Schunke V, J. 1448, 4958, 6077, 6992 (*americanum*).

Schwabe, H. 388 (*nitidibaccatum*).

Schwarz, G.J. 1138, 1212, 4632, 4697, 8145 (*americanum*); 8838 (*chenopodioides*); 8877 (*americanum*).

Schweinfurth, G.A. 1259, 1402 (*villosum*); 1404 (*nigrum*); 1406, 1410 (*villosum*); 1411 (*nigrum*).

Scoggan, H.J. 10011, 10224, 16496 (*triflorum*).

Scott, C. 542 (*emulans*).

Scott, H. 240 (*nigrum*); 269 (*villosum*); 438 (*nigrum*); 491 (*villosum*).

Scott, R.W. 3916 (*triflorum*).

Scott-Elliot, G.F. 173, 416 (*furcatum*); 3354 (*nigrum*).

Scouler, J. 121 (*americanum*).

Scovell, I.T. 1514 (*emulans*).

Scovell, J.T. 1333 (*emulans*).

Scullen, H.A. 396 (*triflorum*).

Seaman, C. 41 (*sarrachoides*).

Sears, R.R. 141 (*americanum*).

Seaton, H.E. 153 (*nigrescens*).

Sebastine, K.M. 1311, 2173, 2790, 4008, 6782, 8488 (*americanum*); 8954, 13928 (*villosum*); 15356, 16691, 24589 (*americanum*).

Seegeler, C.J.P. 2038, 2472 (*villosum*).

Seemann, B.C. 81 (*americanum*); 1606 (*nigrescens*).

Sehnem, A. 2070 (*americanum*).

Seibert, R.J. 601 (*nigrescens*); 1297 (*nigrum*).

Seidel, R. 2717 (*americanum*).

Seidenschwarz, F.G. 111 (*americanum*).

Seigler, D. DS9303 (*douglasii*).

Seijo, G. 1869 (*chenopodioides*); 2315 (*triflorum*).

Sell, P.D. 671814 (*nigrum*).

Sellow, F. 225, 280 (*chenopodioides*).

Semajunde NC-73194 (*americanum*).

Senderayi, E. 226 (*retroflexum*).

Sendulsky, T. 472 (*americanum*).

Sengupta, G. 122, 1221, 1348 (*americanum*); CC-14548 (*nigrum*).

Sennen, Frere 125 (*nigrum*).

Senni, L. 310 (*americanum*); 877 (*villosum*).

Sequeira, M. 2790 (*villosum*).

Serrano, J.G. 21 (*douglasii*).

Serrano, V. 10 (*nigrescens*).

Sérsic, A.N. 5040 (*triflorum*).

Sessé, M. 1414bis (*corymbosum*); 1511, 5341 (*pruinosum*).

Setchell, W.A. 137 (*americanum*).

Seto, K. 31840 (*nigrum*).

Sewell, S. 563, 929, 1652 (*emulans*).

Seydel, R. 1162 (*scabrum*); 3783 (*retroflexum*).

Seymont 1060 (*nitidibaccatum*).

Seymour, F. 10816, 12074, 15890, 15946 (*emulans*).

Seymour, F.C. 2006 (*emulans*); 5146, 5524 (*nigrescens*); 6030 (*nitidibaccatum*); 7111 (*emulans*); 7647 (*nigrescens*); 18192, 20068 (*emulans*).

Seyrig, A. 163 (*americanum*); 217 (*scabrum*).

Shabetai, J.R. 190 (*nigrum*).

Shafer, J.A. 11830 (*nigrescens*).

Shah, M. 170 (*americanum*).

Shankarmani 164 (*nigrum*).

Shannon, S.D. 1816 (*emulans*).

Shantz, H.L. 947, 1202, 1244 (*triflorum*).

Shapiro, G. 49 (*douglasii*); 389 (*nigrescens*).

Sharifi, M.R. 531 (*nigrum*); 540 (*villosum*).

Sharma, J.B. NC-67127 (*americanum*).

Sharma, J.P. NC-70364 (*americanum*); NC-77942 (*villosum*).

Sharma, S. S.S.359 (*villosum*).

Sharma, V.S. 583 (*villosum*).

Sharp, A.J. 4410, 441005 (*douglasii*).

Sharp, S.S. 241 (*triflorum*).

Sharsmith, C.W. 3982 (*nitidibaccatum*).

Shaw, I. 112 (*chenopodioides*).

Shaw, R.J. 2994 (*nitidibaccatum*).

Shear, C.L. 134 (*triflorum*); 177 (*emulans*); 197 (*triflorum*); 3777 (*emulans*); 4159, 4779, 5249 (*triflorum*).

Sheik, A.H. 13176, 13329 (*villosum*).

Sheldon, C.S. 5976 (*emulans*).

Sheldon, E.P. 8326, 9036 (*nitidibaccatum*).

Shepherd, C.J. 215 (*nitidibaccatum*); 718 (*chenopodioides*).

Sheppard, W.B. 76 (*triflorum*).

Shetty, B.V. 42 (*villosum*); 384, AC-566, AC-2214 (*nigrum*); 4107, 8134 (*villosum*).

Shevock, J.R. 11009, 11030 (*americanum*).

Shiki, D. 143 (*americanum*).

Shiller, I. 313 (*nigrescens*).

Shillingford, C.A. 209 (*americanum*).

Shilom Ton, A. 439, 468, 807 (*nigrescens*); 1280 (*douglasii*); 1578, 2119 (*nigrescens*); 2766 (*douglasii*); 3314 (*nigrescens*); 3670 (*douglasii*); 3968, 4184, 4546 (*nigrescens*); 7625, 8261, 9165 (*douglasii*).

Shinners, L.H. 15279 (*emulans*); 17749 (*nigrescens*).

Shiota, K. 107, 3815, 3999, 4663, 9095 (*nigrum*).

Shipchinskii, N. 286 (*villosum*).

Shirley, M.G. 213 (*americanum*).

Shiu Ying Hu 5432, 6781, 7009 (*americanum*).

Shivas, R. 811 (*americanum*).

Short, L.R. S281 (*douglasii*).

Short, M.J. 46 (*nigrum*); 248 (*nigrescens*).

Shukla, A.N. NC-123920 (*americanum*).

Shukla, B.K. BSHC-22169 (*nigrum*).

Shukla, R.K. BSHC-18673 (*americanum*).

Shultz, L.M. 570 (*triflorum*).

Si Boeea, R. 5991, 7308, 8026, 8301, 8611, 9227, 10755 (*americanum*); 10775 (*scabrum*).

Si Toroes, R. 407 (*scabrum*).

Sibil, J. 48, 171, 252, 317 (*americanum*).

Sidey, J.L. 1624 (*retroflexum*).

Sidwell, K.J. 449 (*douglasii*); 546, 595, 807 (*nigrescens*).

Siehe, W. 298 (*villosum*).

Sih-Chiu, M. 442 (*nigrum*).

Sikdar, J.K. 126, 167 (*americanum*); CNH-236, CNH-4183 (*villosum*); 4303, 4338 (*americanum*).

Sikes, K.G. 36 (*sarrachoides*).

Sikes, S. 71 (*douglasii*); 672 (*nigrescens*).

Sikes, S.W. 345 (*douglasii*).

Silva Filho, P.J.S. 716 (*americanum*).

Silva Neto, S.J. 981, 1021 (*americanum*).

Silva, [?] 60 (*americanum*).

Silva, A.C.C. 134 (*americanum*).

Silva, C.A.S. 18 (*americanum*).

Silva, E.M.G.B. 20 (*americanum*).

Silva, G.P. da 2259 (*americanum*).

Silva, J.M. 147 (*americanum*).

Silva, J.S. 32 (*americanum*).

Silva, L.A.M. 3509 (*americanum*).

Silva, M. Da 1685 (*chenopodioides*).

Silva, N.C.B. 47 (*americanum*).

Silva, S.J. 981 (*americanum*).

Silva, S.M. 1646 (*americanum*).

Silveira, G.H. 493 (*americanum*).

Silveira, M. 4176 (*americanum*).

Silveira, N. 8387 (*americanum*).

Silverstone Sopkin, F.A. 756 (*nigrescens*); 2041, 3239 (*americanum*).

Silverstone-Sopkin, P. 756 (*nigrescens*).

Sim, J. JS10 (*americanum*).

Simá, P. 422, 1776 (*nigrescens*).

Simaga, J.M. 713 (*americanum*).

Simmonds, N.W. 14404 (*nigrescens*).

Simon, P.M. 165 (*triflorum*).

Simonds 32 (*villosum*).

Simonis, J.E. 125 (*americanum*).

Simontacchi, A. 71 (*douglasii*); 330 (*americanum*).

Simpson, D.R. 439 (*americanum*).

Simpson, N.D. 16, 112, 1390, 1429, 2995, 3021, 3062, 3787i, 3787ii, 3852 (*villosum*); 4460 (*nigrum*); 4494, 4795 (*villosum*); 5354 (*nigrum*); 5456 (*villosum*); 5663, 5781 (*nigrum*); 6077, 6334 (*villosum*); 8691, 8865, 9394, 9728, 28096, 28118, 34461, 34479 (*nigrum*); 53043 (*villosum*); 68010 (*chenopodioides*).

Sinaca Colín, S. 404, 1139bis, 1708 (*nigrescens*).

Sinclair, J. SF-40388 (*americanum*).

Singer, A. Soni-40708, Soni-80507 (*nigrum*).

Singer, J.W. 351 (*emulans*).

Singh, A.N. 3264, 3282, 6232 (*villosum*).

Singh, D.K. NC-88573 (*nigrum*).

Singh, G. ANU18340 (*chenopodioides*).

Singh, H. 528 (*americanum*).

Singh, K.N. 109 (*nigrum*).

Singh, M.P. NC-19653 (*americanum*).

Singh, N.P. NC-19452 (*villosum*); NC-23085, NC-31620, NC-31632, NC-31930 (*americanum*).

Singh, N.T. NC-25543 (*villosum*).

Singh, P. BSHC-15537, BSHC-16449 (*americanum*).

Singh, R. 421 (*villosum*).

Singh, S. 34010, NC-89850 (*villosum*); NC-90276, NC-90499 (*americanum*).

Singh, U. 12, 42 (*americanum*).

Singh, V. 2988 (*nigrum*); 5397, 5618 (*villosum*).

Sinha, A.K. 43249 (*americanum*).

Sinha, G.P. BSHC-15201, BSHC-15236 (*americanum*); BSHC-16704 (*nigrum*).

Sino-American Expedition 429 (*nigrum*).

Sintenis, P. 251 (*americanum*); 636 (*nigrum*); 675, 1126 (*villosum*); 3513, 3573 (*americanum*); 7469 (*nigrum*).

Skeels, H.C. 520 (*triflorum*).

Skornia, E. 60 (*emulans*).

Skoss, J.D. 17 (*americanum*).

Skottsberg, C. 198, 260, 363 (*furcatum*).

Skroch, P. 50 (*emulans*).

Skroch, W. Cen-238 (*emulans*).

Skutch, A.F. 963, 3712 (*nigrescens*); 5331 (*americanum*).

Slater, C. 45 (*americanum*).

Slee, A.V. 4662 (*triflorum*).

Sleumer, H. 2091 (*americanum*).

Small, J.K. 607 (*americanum*); 937 (*pseudogracile*); 952 (*americanum*); 2311 (*pseudogracile*); 3029, 3564, 3724 (*americanum*); 4193, 4325 (*pseudogracile*); 4696, 4703, 5488 (*americanum*); 5573 (*pseudogracile*); 5588, 5592, 7465 (*americanum*); 8181, 8216 (*pseudogracile*); 8254 (*americanum*); 8258, 8681 (*pseudogracile*); 8712 (*americanum*); 8805 (*nigrescens*); 10335, 10786 (*americanum*); 11079 (*pseudogracile*).

Smart, Dr. 33 (*douglasii*).

Smith, [?] 69 (*americanum*).

Smith, A.C. 9450 (*americanum*).

Smith, C. 33 (*americanum*).

Smith, C.E. 4912 (*corymbosum*).

Smith, C.F. 2443 (*americanum*).

Smith, C.L. 697[b] (*nigrescens*).

Smith, C.N. 1049 (*triflorum*).

Smith, C.P. 979 (*douglasii*).

Smith, D.K. 79 (*americanum*).

Smith, D.N. 6691 (*americanum*).

Smith, E.C. 107, 7120 (*nigrum*).

Smith, E.E. S&S85 (*furcatum*).

Smith, E.M. 325 (*emulans*).

Smith, H. 4552, 6586, 6842 (*nigrum*).

Smith, H.H. 450, 639 (*americanum*); 1165 (*nigrescens*); 1167 (*macrotonum*); 1168, 1170 (*nigrescens*); 2180 (*pseudogracile*); 4382, 4885, 5005, 5198 (*douglasii*); 8189 (*emulans*).

Smith, J.F. 182 (*americanum*); 1034 (*emulans*); 10716 (*triflorum*).

Smith, J.M.B. ANU-15352 (*nigrum*); 15825 (*triflorum*).

Smith, L. 235 (*triflorum*).

Smith, L.B. 3903 (*emulans*); 9725, 12573 (*americanum*); 13848 (*chenopodioides*).

Smith, L.S. 3071 (*nigrum*).

Smith, N. B-19, 4382 (*americanum*).

Smith, P.A. 1080 (*scabrum*); 1494 (*retroflexum*).

Smith, R.V. 64/118, 64/118 (*triflorum*).

Smith, R.W. 819, 1388, 1939 (*emulans*).

Smith, S. 110 (*douglasii*).

Smith, S.F. 743 (*americanum*).

Smith, S.G. 1141 (*americanum*).

Smith, T.E. 2997, 4415 (*emulans*).

Smith, W.T. 13, 26 (*douglasii*).

Smith, W.W. 721 (*americanum*).

Smittenberg-Visser, L. 61 (*scabrum*).

Sneddon, B. 43 (*nigrum*).

Sneidern, K. von 298 (*nigrescens*); 4833 (*americanum*); 5641 (*nigrescens*).

Sniderman, K. 8 (*chenopodioides*); 10 (*nitidibaccatum*).

Snow, N.W. 6436 (*douglasii*).

Snyder, D.P. 89 (*emulans*).

Snyder, J. 27 (*emulans*).

Soakai, E. 941 (*americanum*).

Sobral, M. 1780, 3334 (*americanum*); 3941 (*chenopodioides*).

Sobrinho, F.A. 62 (*americanum*).

Socalski, N.D. 493 (*villosum*).

Sodiro, A. 11 (*macrotonum*).

Sodombekov, I. KPL_00292 (*villosum*).

Soest, L. van 54869 (*nigrum*).

Sohmer, S.H. 5055 (*emulans*).

Soibeh, D. 51 (*americanum*).

Solano, C. 171 (*douglasii*); 506 (*americanum*).

Solano, D. 1748 (*macrotonum*); 1780 (*americanum*); 2971, 4041, 4180, 4286 (*nigrescens*).

Solano, F. 15 (*macrotonum*).

Solís M, A. 4904 (*nigrescens*).

Solís, A. 4874 (*nigrescens*).

Solís, F. 33 (*americanum*).

Solling, K.L. 117 (*americanum*).

Sollmann, A. 12 (*emulans*).

Solomon, J.C. 419 (*douglasii*); 692 (*americanum*); 1092 (*emulans*); 2659 (*nigrescens*); 2697 (*americanum*); 2711 (*nigrescens*); 3853 (*emulans*); 4099 (*nitidibaccatum*); 4120 (*chenopodioides*); 4665 (*triflorum*).

Solves, J.-.P. 25 (*douglasii*).

Somes, M.P. 3765 (*emulans*).

Sorensen, P. 2366, 3228 (*emulans*).

Sorger, F. 80-41-4, 82-23-50, 84-72-100, 85-41-59 (*villosum*).

Soria, J. 109 (*macrotonum*).

Soria, N. 1514, 2671 (*americanum*).

Soriano, A. 832 (*nitidibaccatum*); 2606 (*triflorum*).

Sorrie, B.A. 10627 (*americanum*).

Soto N, J.C. 1476 (*douglasii*); 4745 (*nigrescens*); 5179 (*douglasii*); 5275, 5802 (*americanum*); 6546, 6709, 7214 (*nigrescens*); 7252, 7253, 7319, 7346, 7998 (*americanum*); 8316, 9072, 9303, 9368, 9708 (*douglasii*); 10321 (*nigrescens*); 10346, 11538 (*douglasii*); 12894 (*americanum*); 13806 (*douglasii*).

Soto, A. 158 (*americanum*); 253, 586 (*nigrescens*); 1362 (*americanum*).

Soto, A.D. 158 (*americanum*); 253, 586 (*nigrescens*).

Soto, J.C. 22937 (*nigrescens*).

Soukup, J. 2550 (*americanum*); 4589 (*corymbosum*); 4616 (*americanum*).

Sousa, M. 761 (*nigrescens*).

Sousa-Peña, M. 510 (*nigrescens*).

Southcott, A.M. B1070 (*nigrum*).

Souza, G.R. 1686 (*americanum*).

Souza, J.P. 1011 (*americanum*).

Souza, LOFde 102 (*americanum*).

Souza, M.A.D. de 848 (*americanum*).

Souza, R.S. 470 (*americanum*).

Souza, V.C. 3289, 4305, 12295, 32004 (*americanum*).

Sowder, J.E. 185 (*douglasii*).

Sowerby, B. 204 (*nigrum*).

Sparre, B. 13011, 13014, 13212, 13218 (*americanum*); 14930, 14934 (*macrotonum*); 15262, 15423, 15561, 15562 (*americanum*); 15947, 16900, 16902, 16980, 16986, 17375, 17695, 17697 (*macrotonum*); 17945, 17994, 18107, 18112 (*americanum*); 18949 (*macrotonum*); 19055, 19454, 19816, 19833 (*americanum*).

Spegazzini, C.L. 141 (*chenopodioides*).

Spegazzini, R.A. 56646 (*nitidibaccatum*).

Spellenberg, R.W. 5276 (*douglasii*); 7174 (*nigrum*); 7258, 7328 (*villosum*); 8650 (*douglasii*).

Spellman, D.L. 1861 (*americanum*).

Spence, D.H.N. S233 (*villosum*).

Spencer, J. 1556 (*nitidibaccatum*).

Spencer, M.F. 1724 (*douglasii*).

Sperling, C.R. 5653 (*americanum*).

Sperry, O.E. 1926, 3099 (*interius*).

Spire, C.J. 1060 (*americanum*).

Spitzenberger, F. 180 (*villosum*).

Spongberg, S.A. 65-150 (*triflorum*).

Spooner, A.G. 3341 (*nitidibaccatum*); 5777, 5777 (*triflorum*); 6898 (*nitidibaccatum*); 14125 (*triflorum*).

Sprague, R. 1204 (*emulans*).

Spribille, T. 15164 (*triflorum*).

Spruce, R. 3983, 3984 (*americanum*).

Spuhler, D. 93-161 (*emulans*).

Srinivasan, S.R. 63653 (*americanum*).

Srivastava, N. NC-105544 (*americanum*).

Srivastava, R.C. BSHC-10371 (*americanum*); BSHC-10371[b[, BSHC-13167 (*nigrum*).

Srivastava, S.K. NC-73736 (*nigrum*); NC-96030 (*villosum*); NC-96090, NC-96751 (*americanum*).

St Clair-Thompson, G.W. 204 (*scabrum*).

St John, H. 2902 (*emulans*).

St. John, H. 1314 (*nigrum*); 4938, 6772 (*triflorum*); 19374, 22763 (*americanum*).

Staff NC-857 (*americanum*).

Stafford, P.J. 379 (*nigrescens*).

Stahmann, C. 452, 580 (*emulans*).

Stainton, J.D.A. 53, 150 (*nigrum*); 3142 (*villosum*); 3384, 4959 (*nigrum*).

Stajsic, V. 4292, 4292, 4292, 4292 (*furcatum*).

Standish, M. 437 (*emulans*).

Standley, J.P. 72 (*americanum*).

Standley, P.C. 93 (*douglasii*); 420 (*triflorum*); 482 (*interius*); 4923, 4928, 6114, 6604, 6911, 7369, 7737 (*triflorum*); 8517, 9649 (*emulans*); 14377 (*triflorum*); 21476, 21477 (*douglasii*); 25290, 32374, 32644 (*americanum*); 34240, 34951, 38164, 38543, 38689 (*nigrescens*); 39022 (*americanum*); 42524, 42701, 42744 (*nigrescens*); 43516 (*macrotonum*); 46920 (*americanum*); 49646, 49767, 49932, 50022, 50342 (*nigrescens*).

Standly, P.C. 14213 (*nitidibaccatum*).

Stanes, E. 28148 (*villosum*).

Stanford, E.E. 12 (*nitidibaccatum*).

Stanford, L.H. 840 (*nigrescens*).

Stanford, L.R. 345, 724, 2386, 2445 (*nigrescens*).

Stapf, O. 2731 (*villosum*).

Staple, A.J. 17 (*nigrum*).

Starmuehlner, F. 270 (*nigrum*).

Stearn, W.T. 113 (*americanum*); 122 (*nigrescens*); 181 (*americanum*); 192 (*nigrescens*); 271, 330, 365, 432, 483, 542, 584, 1000 (*americanum*); 1145, 1174 (*villosum*).

Stebbins, G.L. 19 (*furcatum*); 41, 63, 80 (*douglasii*); 87 (*americanum*); 89 (*furcatum*); 92 (*americanum*); 97 (*nitidibaccatum*); 101, 103 (*furcatum*); 105 (*americanum*); 106, 107 (*douglasii*); 130 (*furcatum*); 133 (*douglasii*).

Steele, F.L. 1949 (*nigrum*).

Steenis, C.G.G.J. van 24079 (*retroflexum*).

Stefano, S. de 186 (*emulans*).

Stehmann, J.R. 180, 192, 270, 1553, 1616, 6162, 6340 (*americanum*).

Steiger, T.L. 1319 (*nigrescens*).

Steiner, E. 22 (*nigrum*).

Steinmann, V.W. 4052 (*americanum*).

Stellfeld, C. 1065, 1202 (*americanum*).

Stephens, S. 13113, 41414 (*triflorum*).

Stergios, B. 3723 (*nigrescens*); 20559 (*macrotonum*).

Stern, S. 88 (*americanum*); 98 (*corymbosum*); 264 (*nigrescens*); 301 (*americanum*).

Stern, W.L. 317 (*americanum*).

Steudner, H. 734, 734 (*villosum*).

Stevart, B.G. 30 (*nigrescens*).

Stevens, C.A. 226 (*triflorum*).

Stevens, G.W. 676, 838 (*interius*); 852, 1096 (*triflorum*); 1298 (*emulans*); 1566, 1679 (*interius*); 1831, 2529 (*emulans*); 29103/4 (*interius*).

Stevens, O.A. 226 (*triflorum*); 802 (*interius*); 913, 1287 (*triflorum*); 1465 (*nitidibaccatum*); 1668, 1994, 2032 (*emulans*); 2731 (*triflorum*).

Stevens, W.D. 2931 (*americanum*); 3430, 3571 (*nigrescens*); 3969 (*douglasii*); 4104, 4300, 4369 (*nigrescens*); 4740 (*douglasii*); 4773 (*nigrescens*); 5009, 7310 (*americanum*); 7968, 8896 (*nigrescens*); 9524, 10088, 10163 (*americanum*); 10255, 10274, 10727 (*nigrescens*); 10859 (*douglasii*); 11419, 11632, 13305, 13390, 13669 (*nigrescens*); 14315 (*macrotonum*); 14522 (*americanum*); 14920, 15537 (*nigrescens*); 15590 (*americanum*); 15784 (*nigrescens*); 15848 (*americanum*); 16051, 16335 (*nigrescens*); 16638 (*americanum*); 17912 (*nigrescens*); 17956 (*douglasii*); 20895, 21299, 21300, 21894 (*americanum*); 26707, 27002, 27304, 32871 (*nigrescens*).

Stevenson, G.A. 1934 (*nitidibaccatum*).

Stevenson, M.C. 141 (*triflorum*).

Stevenson, P. 1940 (*americanum*).

Steward, A.N. 3022, 6597 (*americanum*); 6831 (*triflorum*); 7017 (*nitidibaccatum*); 7018 (*triflorum*); 9691 (*nigrum*).

Stewart, A. 3406, 3407, 3409 (*americanum*).

Stewart, C.C. 2175 (*emulans*).

Stewart, G. 497 (*chenopodioides*).

Stewart, K. sol1 (*triflorum*).

Stewart, R.M. 729 (*douglasii*); 1215 (*nigrescens*).

Stewart, R.R. 19404A (*nigrum*).

Stewart, S. 459 (*emulans*).

Steyermark, J.A. 841 (*emulans*); 13206, 35953 (*macrotonum*); 46635 (*nigrescens*); 55529 (*macrotonum*); 68846 (*triflorum*); 88968 (*americanum*); 96948, 96970, 104828 (*macrotonum*); 110480 (*americanum*); 118311, 118569 (*nigrescens*); 121839 (*macrotonum*); 122055, 124737 (*nigrescens*); 125438, 127922 (*macrotonum*); 130943 (*americanum*).

Stickney, P.F. 450 (*nitidibaccatum*).

Stoddart, D.R. 2198, 2249, 7126, 8103 (*americanum*).

Stone, B.C. 4701[b], 14055 (*americanum*).

Stone, J. 2891 (*douglasii*); 4167 (*americanum*).

Stone, J.R. 4167 (*emulans*).

Stork, H.E. 1483, 2459, 2997 (*nigrescens*).

Storto, J. 301 (*triflorum*).

Strachey, R. 1 (*nigrum*); 1[b] (*villosum*).

Straka, G. 9 (*americanum*).

Straley, G.B. 4326 (*nitidibaccatum*); 4338 (*nigrum*); 8079 (*nitidibaccatum*).

Straube, F. 66 (*americanum*).

Street, J.K. 222, 249, 258, 306 (*villosum*).

Streets, R.B. 581 (*triflorum*).

Streimann, H. 8078 (*americanum*).

Strey, R.G. 4311, 4573 (*americanum*); 9409 (*retroflexum*); 11303 (*americanum*).

Strid, A. 2882 (*scabrum*).

Stubbs, W.J. 48 (*nitidibaccatum*).

Stuckert, T. 5022 (*triflorum*); 17713a (*nitidibaccatum*).

Studhalter, R.A. 1412, 1413 (*douglasii*).

Stueber, S. 31 (*emulans*).

Suárez, M.C. 174 (*nigrescens*).

Subba Rao, G.V. 66 (*americanum*); 19565 (*nigrum*).

Subils, R. 35 (*triflorum*); 2333, 3941 (*nitidibaccatum*); 4059, 4076, 4108, 4113 (*triflorum*); 4168, 4269 (*americanum*); 4668 (*chenopodioides*).

Subramaniam, K. 142, 6473 (*americanum*); 7199, 64511 (*villosum*).

Suclli, E. 1140 (*americanum*).

Sucre, D. 1976, 4952, 6464, 7842, 9644, 9680 (*americanum*).

Sudworth, G.B. 252 (*emulans*).

Sugden, A. 170 (*americanum*).

Sugiyama, M. 293 (*chenopodioides*).

Suksdorf, W. 1480, 2317 (*nitidibaccatum*); 2318 (*americanum*); 12045 (*triflorum*).

Suksdorf, W.N. 902 (*triflorum*); 1720 (*furcatum*); 8934, 12045 (*triflorum*).

Sulit, M.D. 16987 (*americanum*).

Sullivan, G. 621 (*nigrescens*).

Sullivan, G.A. 621 (*nigrescens*); 1193 (*americanum*).

Sullivan, J.M. 102992 (*sarrachoides*).

Sumithraarachchi, D.B. 512 (*americanum*).

Summers, B. 8869, 10625 (*emulans*).

Summers, J.W. 249, 3510, 4481 (*sarrachoides*); 6442, 7192, 8829, 8831, 8869, 8888, 8919 (*emulans*); 9448, 9897 (*sarrachoides*); 9908 (*emulans*); 10626 (*sarrachoides*).

Summers, R.W. 607 (*douglasii*).

Sun, S.C. 634 (*nigrum*).

Sundell, E. 15 (*emulans*); 16733 (*nitidibaccatum*).

Suominen, J. 2010, 2042 (*nigrum*).

Supernaugh, W.R. 443 (*douglasii*).

Surgeon Major Hamilton 17900 (*villosum*).

Sutherland, D.M. 5659 (*triflorum*).

Sutherland, J. 281 (*villosum*).

Suzuki, M. 88-11167 (*villosum*); 88-20090, 88-20210, 92-40045 (*nigrum*).

Svendsen, J. 400 (*nigrum*).

Svendson, J. 400 (*nigrum*).

Svenson, H.K. 256 (*emulans*).

Svenson, J.E. 217 (*nigrescens*).

Swab, E.C. 607 (*emulans*).

Swagel, K. 122 (*nigrescens*).

Swan, J.M. 128 (*americanum*).

Swann, E.L. 1931, K3258, K3259 (*nitidibaccatum*).

Swanson, S.D. 1816, 1954, 2251, 2421 (*emulans*).

Swarbrick, J.T. 2572 (*scabrum*); 7451 (*nitidibaccatum*); 10855 (*chenopodioides*); 13131 (*americanum*).

Swink, F.A. 3020 (*triflorum*).

Swinney, R.G. 14082, 14484 (*douglasii*).

Swynnerton, C.F.M. 1794 (*retroflexum*).

Sykes, W.R. 36-08, 87-T, 514/K (*americanum*); 521/93 (*nigrum*); 897/K (*americanum*); 902/K, 937/K, 1055/K, 1057/K (*nigrum*); 1059/K, 1102/K, 1379/K, 1466/K, 175961 (*americanum*).

Symon, D.E. SS-83, SS-85 (*nigrum*); SS-86 (*nitidibaccatum*); SS-89 (*nigrum*); SS-91 (*nitidibaccatum*); SS-92 (*nigrum*); SS-94, SS-95 (*americanum*); SS-99, SS-108, SS-109, 110, 111, 112, 113, SS-115, 117, 118, 119, 120, SS-125 (*nigrum*); SS-127 (*americanum*); SS-137,

SS-138, SS-141 (*nigrum*); SS-142, SS-143 (*americanum*); 222, SS-265 (*nigrum*); SS-267, 268, SS-268, SS-270 (*americanum*); SS-276, SS-293, SS-294, SS-313, SS-314 (*nigrum*); SS-315 (*americanum*); SS-316, SS-318, 879, S-1142, 1165, 1181, NPYE-1604, 1944, 2089, 2109, 2134, 2918, 2921, 3430, 4065, 4531 (*nigrum*); 4742, 4742 (*triflorum*); 4779, 4879, 4880 (*americanum*); 4954, 5449, 5462, 5486, 5599, 5945 (*nigrum*); 6090 (*americanum*); 6608, 6635, 6690, 7263, 7618 (*nigrum*); 7626 (*nitidibaccatum*); 7627, 7631, 7633, 8012, 8164, 8845 (*nigrum*); 9807 (*triflorum*); 9922, 9929, 9931, 9952, 10310 (*nigrum*); 10665 (*americanum*); 11275 (*nigrum*); 11515 (*chenopodioides*); 11521, 11525 (*americanum*); 11587, 11777, 13742, 14144, 14261, 14613, 14618, 14855 (*nigrum*); 14882 (*americanum*); 15064, 15165, 15263 (*nigrum*); 15462 (*chenopodioides*); 15941, 16610, 16858, 16910, 17075, 18149 (*nigrum*); 35881, 35883 (*scabrum*).

Syngrassides, A. 244 (*villosum*).

Synnott, T.J. 737 (*scabrum*); 1404 (*villosum*).

Sytsma, K. 2135 (*macrotonum*).

Sørensen, T. 1756 (*nigrum*).

Taam Ying-Wah 1144 (*americanum*).

Taborda, I. 4 (*americanum*).

Tadesse Ebba 627 (*villosum*).

Tadesse, E. 627 (*villosum*).

Tai, H.H. 81 (*nigrum*).

Tai, L.Y. 11835 (*nigrum*).

Takaki, F. S-190 (*nigrescens*).

Takamatsu, M. 772 (*americanum*).

Takayama, H. 92-39004 (*nigrum*).

Tamashiro, J.Y. 143, 1009 (*americanum*).

Tamulevich, S. 29 (*emulans*).

Tamura, M. 26714 (*americanum*).

Tanaka, T. 1760 (*nigrum*).

Tang Siu Ging 6978, 7322, 13156, 13358, 13526, 13981 (*nigrum*).

Tanner, R.E.S. 829, 924, 3020, 4532 (*villosum*).

Tap, N. HLF3387 (*americanum*).

Tapia, J.L. 1370 (*nigrescens*).

Taquet, E.J. 298, 1145 (*nigrum*).

Tate, R. 258(219) (*americanum*).

Tavares, S. 167 (*americanum*).

Taws, N.M. 592 (*americanum*).

Taye, A. 3391 (*triflorum*); 4120 (*nitidibaccatum*).

Taylor J. 25237 (*triflorum*).

Taylor, C.M. 2045 (*americanum*); 2261, 2634 (*nigrescens*); 6684, 6706, 7161 (*americanum*); 10187 (*furcatum*); 11096, 11804, 12967 (*americanum*).

Taylor, D.W. 4863 (*nitidibaccatum*); 9474 (*americanum*); 10194 (*douglasii*); 10409 (*americanum*); 14733 (*douglasii*).

Taylor, H.C. 3205 (*retroflexum*).

Taylor, J. 15637 (*americanum*).

Taylor, L.E. 362 (*retroflexum*).

Taylor, M. 126 (*nigrescens*).

Taylor, M.J. 267 (*triflorum*).

Taylor, M.S. 627, 1304, 1860 (*douglasii*); 2195 (*americanum*); 5701, 5816 (*emulans*).

Taylor, P. PT105P (*nigrescens*).

Taylor, R.J. 15637 (*americanum*).

Taylor, R.S. BS87-182, 291a, 1530, 1530 (*triflorum*).

Taylor, W.E. 100 (*americanum*).

Teague, A.J. 74 (*retroflexum*).

Teixeira, L.O.A. 724 (*americanum*).

Teixeira, W.A. 25084 (*americanum*).

Telford, I.R. 7215 (*americanum*); 7352 (*triflorum*).

Téllez V, O. 1414 (*americanum*); 1414, 4282, 5144, 6430, 7991, 10114 (*nigrescens*).

Téllez, O. 4282 (*nigrescens*).

Ten, S. 12 (*nigrum*).

Teng, S.W. 259, 91053 (*nigrum*).

Tengwall, T.A. 40 (*villosum*).

Tenore, M. 152 (*villosum*).

Tenorio L, P. 788 (*douglasii*); 968 (*nigrescens*); 1096 (*douglasii*); 1581 (*nigrescens*); 1677, 1955, 1988, 2033, 2075, 2322 (*douglasii*); 2633, 3562, 4806 (*nigrescens*); 5051 (*douglasii*); 5120, 5205, 5341, 6317 (*nigrescens*); 8097 (*douglasii*); 12506, 12638 (*nigrescens*); 16155 (*douglasii*); 18134 (*pruinosum*); 18947, 19869 (*nigrescens*).

Tepe, E.J. 2272 (*americanum*); 2579, 2929, 3053, 3067, 3350 (*macrotonum*).

Terracciano, A. 187 [2206] (*scabrum*); 807, 984 [2207], 1600, 1716, 2105, 2109, 2116, 2188 (*villosum*); 2536 (*scabrum*).

Terry, P.J. 3027 (*americanum*).

Tessene, M. 210 (*emulans*).

Tessmann, G. 1217, 2759 (*americanum*).

Thacher, O. 45 (*douglasii*).

Thackery, F.A. 524 (*americanum*).

Thamer NHI-46781 (*nigrum*); NHI-47404 (*villosum*).

Tharp, B.C. 13A-162, 48-195 (*nigrescens*); 51-341 (*interius*); 562 (*emulans*); 724 (*interius*).

Thode, V. 91 (*americanum*).

Thomas, A.S. Th4070 (*scabrum*).

Thomas, D. 1005 (*villosum*); 69805 (*americanum*).

Thomas, D.W. 1239, 2997 (*scabrum*).

Thomas, J.D. 80103, 122716 (*nigrescens*).

Thomas, J.H. 10878 (*nitidibaccatum*).

Thomas, M.B. MT-488, MT551, MT569, MT-595 (*americanum*).

Thomas, N.W. 2042, 3703 (*scabrum*); 4396 (*americanum*); 4909, 6408, 9638 (*scabrum*).

Thomas, R. 661 (*americanum*).

Thomas, R.D. 61226, 69805 (*emulans*); 89754, 95065 (*americanum*); 98597, 99955 (*emulans*); 105879 (*chenopodioides*); 108232, 114863, 116082 (*emulans*); 122411 (*nigrescens*); 126797 (*americanum*); 132327, 132980, 138877, 140073, 142721, 143348, 145394, 145732, 148045, 151462, 153473 (*emulans*); 153474 (*nigrescens*); 160424 (*emulans*); 164070 (*nigrescens*); 169908, 171536 (*emulans*).

Thompson, C.H. 114 (*emulans*).

Thompson, J.W. 485 (*triflorum*); 3663 (*nigrum*); 3729, 3766a (*americanum*); 3766b (*nitidibaccatum*); 3802 (*americanum*); 9069, 11807, 11907, 11984 (*triflorum*).

Thompson, K.L. 604 (*emulans*).

Thompson, M.F. 134 (*retroflexum*).

Thompson, O. 159 (*chenopodioides*).

Thompson, R.A. S-0735, C1060 (*emulans*).

Thompson, S.A. 1753 (*scabrum*); 3079 (*nigrescens*).

Thomson, P.J. 481 (*scabrum*).

Thomson, R. 320 (*interius*).

Thomson, T. 62 (*nigrum*); 62[b] (*americanum*); 62[c] (*villosum*); 62[d] (*nigrum*); 62[e] (*villosum*).

Thornber, J.J. 249 (*douglasii*).

Thornburg, D.C. 1153 (*douglasii*).

Thorne, K.H. 3106 (*triflorum*); 3888 (*nitidibaccatum*); 15167 (*triflorum*).

Thorne, R.F. 9133 (*pseudogracile*); 32284, 36247, 42847, 62266, 62489 (*douglasii*).

Thothathri, K. 9960 (*nigrum*); 10461 (*americanum*).

Threlfall, W. 10 (*villosum*).

Thurber, G. 364, 577 (*douglasii*).

Thuret, G.A. 12 (*villosum*); 334 (*nigrum*).

Thurston, H.R. 3195 (*americanum*).

Tidestrom, I.F. 550 (*triflorum*); 5342 (*emulans*).

Tiehm, A. 3657 (*nitidibaccatum*); 6218 (*triflorum*); 6219 (*nitidibaccatum*); 6255 (*triflorum*); 6718 (*nitidibaccatum*); 7412 (*triflorum*); 10921, 13715 (*nitidibaccatum*); 15075, 15484, 15960 (*triflorum*); 16510 (*nigrum*); 17643 (*triflorum*).

Tilden, J.E. 78 (*americanum*); 832 (*nigrum*).

Tilforth, C.W. 180 (*douglasii*).

Till, W. 71 (*nigrum*).

Timaná, M. 2008, 2203, 2214, 2226, 2678, 3251 (*americanum*).

Timberlake, J.R. 1635 (*villosum*); 4521 (*retroflexum*).

Tink, E. 266 (*americanum*).

Tipaz, G. 1585 (*macrotonum*).

Tirado, M. 162 (*nigrescens*).

Tirado, N. 18 (*americanum*); 42 (*nigrescens*); 76 (*americanum*); 107, 138 (*nigrescens*).

Titov, V.S. 2220 (*nigrum*).

Tiwari, G.C. 35A (*americanum*).

Tiwari, G.L. AC-836 (*nigrum*).

Tlapa A, M. 80, 162, 232 (*nigrescens*); 438 (*americanum*).

Tlapa, M. 1, 105, 618 (*douglasii*).

To Hara 79/41, 115/35 (*nigrum*).

Tobey, C. 817 (*nigrum*); 2244 (*villosum*).

Todaro, A. 874 (*villosum*); 875 (*nigrum*).

Todzia, C.A. 101 (*nigrescens*); 1988 (*americanum*).

Toledo, M. 18 (*nigrescens*).

Tolstead, W.L. 699 (*triflorum*); 42507 (*interius*).

Tomb, A.S. 359 (*nigrescens*).

Tonduz, A. 148b, 1537 (*americanum*); 2095 (*nigrescens*); 3041, 4237, 6605, 8358, 8711 (*americanum*); 10810, 12258 (*nigrescens*); 17757 (*macrotonum*).

Topa, E. 2684 (*villosum*).

Toppin, S.M. 82 (*nigrum*).

Toriz A, G. 716 (*americanum*).

Torke, K.J. 484 (*nigrescens*).

Toro, R.A. 470 (*nigrescens*).

Torre, A.R. 2214 (*villosum*); 4957 (*retroflexum*).

Torrecillas, E. 200 (*americanum*).

Torreggiani, [?] 13 (*chenopodioides*).

Torres C, R. 1893 (*douglasii*); 2336 (*americanum*); 2849 (*nigrescens*); 3065 (*pruinosum*); 3493, 3527, 3548 (*douglasii*); 4442, 4477, 5111, 6816, 8329, 10132, 10159, 10358, 11051, 12398 (*nigrescens*); 15288 (*americanum*).

Torres R, J.H. 19, 61 (*macrotonum*).

Torres R, R. 2849 (*nigrescens*); 3493, 3527, 3805 (*douglasii*); 12398 (*nigrescens*).

Torres, B. 97 (*nigrescens*).

Torres, R.B. 344A (*americanum*).

Torrey, G.S. 108 (*emulans*).

Torrey, J. 353 (*douglasii*).

Toscani, H.L. 50 (*chenopodioides*).

Tosh, J.P. 504 (*emulans*).

Toth, J. 3 (*triflorum*); 9 (*americanum*).

Toumey, J.W. 397 (*douglasii*); 402 (*triflorum*).

Tovar, J.D. 4 (*americanum*).

Townsend, C.C. 85/4 (*villosum*); 591003/01 (*emulans*).

Tracy, J.P. 1983 (*americanum*); 2605, 3074 (*nitidibaccatum*); 3992, 5202, 13698 (*americanum*); 16738 (*nitidibaccatum*); 16746 (*americanum*); 16749 (*furcatum*); 16758 (*nitidibaccatum*); 16767 (*nigrum*).

Tracy, S.M. 32a (*americanum*); 52a, 5153 (*nigrescens*); 6829 (*pseudogracile*).

Train, P. 1838, 2501 (*triflorum*); 3469 (*nitidibaccatum*); 3470 (*triflorum*).

Trana, T.D. 17937, 18620 (*emulans*).

Trapnell, C.G. 1453 (*scabrum*).

Trask, B. 238 (*douglasii*).

Travers, W.A. 116 (*nigrum*).

Traverse, A. 196, 910, 1075, 1110, 1460 (*nigrescens*).

Tregrubov, V. 168 (*nigrum*).

Trelease, W. 499, 500, 501, 539 (*emulans*).

Tressens, S.G. 6401 (*americanum*).

Trethewy, A.W. 83, 183, 193, 262 (*villosum*).

Triana M, L.A. 266 (*americanum*).

Triana, J.J. 3855 (*americanum*).

Tribedi, G.N. 1, 201 (*americanum*).

Trigos, R.C. 2291 (*nigrescens*).

Tróchez, L. 57 (*nigrescens*).

Troiani, H.O. 3431, 3648A (*triflorum*).

Troncoso, N.S. 2288 (*americanum*).

Trott, A.C. 90 (*villosum*); 982A (*nigrum*); 1283 (*villosum*).

True, G.H. 195, 806 (*douglasii*).

Trujilio Olazo, I. 298 (*nigrescens*).

Trujillo Eslava, R. 56 (*nigrescens*).

Trujillo Olazo, I. 166, 1518, 1681, 1929 (*nigrescens*).

Trujillo Vasquez, R. 43 (*americanum*).

Trujillo Vásquez, R. 491 (*nigrescens*).

Trujillo Vazquez, R. 422 (*nigrescens*).

Trujillo, B. 18996 (*macrotonum*).

Trusty, J. 456 (*americanum*).

Tsai, H.T. 52294, 52833, 53616, 54308 (*nigrum*); 54780, 54844 (*americanum*); 55155 (*villosum*); 56071, 56549, 57961, 58490, 58839, 59074, 60501, 62021, 63005 (*nigrum*).

Tsang, W.T. 231, 331, 412 (*americanum*); 16639, 20890 (*nigrum*).

Tschopp, E. 77 (*americanum*).

Tsi Zhanhuo 92-162, 91-390 (*americanum*).

Tsiang Ying 133 (*nigrum*); 1968 (*americanum*); 10817 (*nigrum*).

Tsiang, Y. 16371 (*americanum*).

Tsugaru, S. B-12, B-2383, 3378 (*americanum*); 7604 (*chenopodioides*); 7717 (*nigrum*).

Tucker, G.C. 2126 (*nigrescens*); 13470 (*sarrachoides*).

Tucker, J.M. 714, 776, 1040 (*nigrescens*); 1223 (*douglasii*).

Tullgren, A. 95 (*villosum*).

Tun Ortíz, R. 34, 1168, 1490 (*americanum*).

Tuomisto, H. 62 (*americanum*).

Türckheim [Tuerckheim], H. von 384 (*macrotonum*); 8554 (*nigrescens*).

Turner, D. DT113/77(2) (*villosum*).

Turner, S.R. 15-100 (*nigrum*).

Tweedie, J. 38, 39 (*chenopodioides*); 432 (*sarrachoides*).

Tweedy, F. 831 (*villosum*); 3531, 4619 (*triflorum*); 5234 (*nitidibaccatum*); 5235 (*triflorum*); 5236 (*interius*).

Twisselmann, E.C. 822, 9094 (*americanum*); 11634 (*nigrum*); 13683 (*americanum*); 17685 (*nitidibaccatum*).

Tyrell, T. 2 (*douglasii*).

Tyrer, P.J. 110 (*villosum*).

Tyrrel, A. 24 (*americanum*).

Tyson, E.L. 1732, 2136 (*americanum*); 2532, 3671, 4776 (*nigrescens*); 5103 (*americanum*); 5509 (*nigrescens*); 6303 (*americanum*).

Ucán Ek, E. 2270, 3247 (*americanum*); 3523, 4390 (*nigrescens*).

Udulutsch, R.G. 196 (*americanum*).

Ugent, D. 85 (*nigrescens*); 687 (*americanum*); 1111[a] (*emulans*); 2717 (*americanum*); 5304 (*corymbosum*).

Ule, E. 4310 (*chenopodioides*).

Ulibarri, E.A. 457 (*nitidibaccatum*).

Umaña, G. 624 (*americanum*).

Umbach, L.M. 4121, 4843, 5097, 6747, 7210, 10656 (*emulans*).

Umbanhowar, C.E. 45 (*triflorum*).

Underwood, L.M. 243 (*triflorum*).

Ungaretti, I. 545 (*americanum*).

United Fruit Company 228 (*americanum*).

Universidade dos Açores-NHM NHM37, 46 (*nigrum*).

Uniyal, B.P. NC-61013 (*americanum*); NC-80282 (*villosum*); NC-91885, NC-92625 (*americanum*).

Uong Sing Po 12110 (*nigrum*).

Uotila, P.J. 10926, 10930, 15112 (*villosum*); 17654, 17713 (*nigrum*); 18172, 18387, 18448, 18822, 19014 (*villosum*); 19021, 19033 (*nigrum*); 19052, 19080, 19207, 19251, 19462, 19669, 19873, 19926, 20156 (*villosum*); 20278 (*nigrum*); 20307, 20503, 20709b (*villosum*); 20709a (*nigrum*); 20780, 20782 (*villosum*); 20942 (*nigrum*); 37632 (*villosum*).

Urbatsch, L.E. 1903 (*pseudogracile*); 2480 (*nigrescens*).

Urrea, G. 37 (*macrotonum*).

USA Typhus Commission 71 (*americanum*).

Utech, F. 82-479 (*emulans*).

Utech, F.H. 94-2249 (*emulans*).

Utitiay, A. 0 (*americanum*).

Utley, J.F. 2312 (*nigrescens*).

Uzzell, P.B. 181 (*nigrescens*).

Vainio-Mattila, K. 95-9 (*americanum*).

Vajravelu, E. 22435 (*americanum*); 60641 (*villosum*).

Valdemarin, K.S. 686 (*americanum*).

Vale, G.D. 101 (*americanum*).

Valencia Avalos, S. 1629 (*douglasii*).

Valenzuela, L. 468, 5148, 6650, 6785, 12648 (*americanum*).

Valeur, E.J. 138, 1021 (*americanum*).

Valiente Banuet, A. 65 (*nigrescens*).

Válka, R.J. 1477 (*americanum*).

Valkenburg, J.L.C.H. van 362 (*americanum*).

Valle Doménech, A. 185, 272 (*nigrescens*).

Valverde, O. 852 (*nigrescens*).

van der Werff, H. 7685 (*macrotonum*); 14657 (*corymbosum*); 15742 (*americanum*).

Van Devender, T.R. 93-300, 93-378 (*americanum*); 93-379 (*douglasii*); 92-955 (*nigrescens*); 93-1012 (*americanum*); 92-1167A (*douglasii*).

Vanderhorst, J. 5741 (*triflorum*).

Vanegas, M. 1 (*nigrescens*).

Vanni, R. 2304 (*sarrachoides*).

Vanni, R.O. 679, 804 (*americanum*).

Vanoverbergh, M. 3542 (*nigrum*).

Vänskä, H. 5166 (*villosum*).

Väre, H. 10268, 11270, 12568, 20026 (*villosum*).

Varela, F. 687 (*chenopodioides*).

Vargas C, I.G. 2663 (*americanum*).

Vargas R, C. 214 (*americanum*).

Vargas Ruiz, E. 502 (*nigrescens*).

Vargas, [?] 63 (*nigrescens*).

Vargas, C.A. 2486 (*macrotonum*).

Vargas, H. 4821 (*macrotonum*).

Vargas, I.G. 6185 (*americanum*).

Vargas, J.M. 63 (*nigrescens*).

Vargas, L.D. 1056 (*americanum*); 1394, 1857, 2249, 2936, 3083, 3949 (*nigrescens*).

Vargas, P. 304 (*villosum*).

Vasey, G. 460 (*triflorum*).

Vasey, G.R. 352 (*triflorum*); 383, 442 (*douglasii*).

Vasquez M, N. 95 (*nigrescens*).

Vásquez, E. 192 (*americanum*).

Vásquez, R. 13379, 20795, 25177 (*americanum*).

Vasudeva Rao, M.K. ANC-7429, ANC-13066 (*americanum*).

Vatova, A. 101, 1854 (*villosum*).

Vaughan, J. 641 (*macrotonum*).

Vaughan, J.H. 1205 (*americanum*).

Vauthier, M. 537 (*americanum*).

Vázquez B, F. 196, 355, 1590 (*nigrescens*).

Vázquez Hernández, J.P. 51, 340 (*nigrescens*); 351 (*americanum*).

Vazquez T, V. 73 (*nigrescens*).

Vázquez V, L. 611, 877, 1017 (*nigrescens*).

Vázquez, J. 3910 (*nigrescens*).

Vega A, R. 244 (*nigrescens*); 4278 (*americanum*).

Vega, C. 870 (*chenopodioides*).

Vega, M. 217 (*nigrescens*).

Vegetti, [?] 746 (*americanum*).

Veillon, J.M. 3402 (*americanum*).

Veken, P. van der 12319 (*triflorum*).

Velarde Nuñez, O. 874 (*corymbosum*).

Velasco G, K. 2070 (*douglasii*).

Velasco López, E. ES0385, 386 (*nigrescens*).

Velasco, V. 177 (*nigrescens*).

Velásquez, P. 512 (*nigrescens*).

Velayos, M. 9450 (*nigrum*); 9695 (*villosum*); 10893 (*nigrum*); 10916 (*villosum*); 11623 (*americanum*).

Velázquez L, C. 182 (*nigrescens*).

Velázquez, N. 68 (*nigrescens*).

Vélez N, M.C. 743 (*americanum*).

Vélez, C. 6333 (*americanum*).

Véliz, I. 1357 (*americanum*).

Véliz, M. 92-2183 (*americanum*); 92-2226 (*macrotonum*); 99-7529 (*nigrescens*); 2M8445 (*macrotonum*); 2M-8445, 2M-9232, MV 2M9254, 2M-9254, 2M-9766, 10326, 11362, 12694, 16975 (*nigrescens*).

Velzen, H. van 73 (*nigrescens*).

Vendruscolo, G.S. 474, 487 (*americanum*); 721 (*sarrachoides*).

Venrick, B. 480 (*douglasii*).

Ventania, P. 870 (*chenopodioides*).

Ventur, P. 27 (*nigrescens*).

Ventura A, A. 126 (*douglasii*); 345 (*corymbosum*); 525 (*douglasii*); 672 (*nigrescens*); 831, 1117, 1265, 1380 (*douglasii*); 2032 (*nigrescens*); 2588 (*pruinosum*); 2713 (*douglasii*); 4105 (*corymbosum*).

Ventura A, F. 5818, 9850 (*nigrescens*).

Ventura V, E. 54 (*corymbosum*); 319 (*nigrescens*); 345 (*corymbosum*); 537 (*nigrescens*); 766 (*douglasii*); 6116 (*corymbosum*); 7963 (*pruinosum*).

Ventura, E. 868, 1091, 1652, 1780, 2121 (*nigrescens*); 2273 (*americanum*); 2423, 3208, 3349 (*nigrescens*); 3468 (*americanum*); 5365 (*nigrescens*); 6484, 7388, 9194 (*pruinosum*).

Venturi, S. 64, 5005 (*chenopodioides*).

Verdcourt, B. 564 (*villosum*).

Vergara, L.K. 84 (*nigrescens*).

Verma, A.K. BSHC-3568 (*nigrum*); BSHC-4226, BSHC-6728 (*americanum*); CC-30761 (*nigrum*).

Verma, D.M. CC-1816, 6728 (*villosum*).

Vervoorst, F. 3479 (*sarrachoides*); 5723 (*triflorum*).

Vescio, L.S. 120 (*triflorum*); 388 (*emulans*).

Vesey-Fitzgerald, L.D.E.F. 7540, 17068/3 (*villosum*).

Viana, J.J. JPB18720 (*americanum*).

Vianna, E.C. 11, 18, 146 (*americanum*).

Vibrans, H. 4449, 4544, 4968 (*americanum*); 5363 (*douglasii*); 5682 (*nigrescens*); 6260 (*americanum*); 6813 (*douglasii*); 6933, 7315 (*americanum*).

Vicherek, J. 1554 (*nigrum*).

Vickery, R. 1472 (*nitidibaccatum*).

Vidal López, M. 67 (*villosum*).

Vidal, M.R.R. 354 (*americanum*).

Vidyamayi, [?] 1134 (*villosum*).

Vieira, R.F. 430, 1676 (*americanum*).

Viereck, H.W. 772, 885 (*nigrescens*).

Vietnam Highland VH-5351 (*americanum*).

Vignoli-Silva, M. 239 (*americanum*).

Vilcapoma, G. 56, 157 (*corymbosum*).

Villa Kamel, A. 53 (*douglasii*); 103 (*nigrescens*).

Villa, A. 48 (*nigrescens*).

Villamil, E. 113 (*nigrescens*).

Villanueva, R. 555 (*nigrescens*).

Villegas, [?] 71 (*americanum*).

Vincent, G. 78-852 (*emulans*).

Vincent, M.A. 1493 (*emulans*); 5004 (*sarrachoides*); 9965 (*triflorum*); 10056 (*emulans*).

Viquez, M. 46 (*nigrescens*).

Virgo, K.J. 130 (*nigrum*).

Visanatham, M.V. NC-55656 (*americanum*).

Visher, S.S. 300, 304, 2147 (*triflorum*); 2250 (*interius*); 2722, 3326 (*triflorum*).

Viswanathan, M.V. 55656 (*villosum*).

Viswe, A.K. BSHC-4765 (*americanum*).

Vitek, E. 02-18 (*nigrum*); 97-25 (*villosum*); 96-136 (*nigrum*); 99-269 (*villosum*); 96-381 (*nigrum*); 98-1622 (*villosum*); 03-1710 (*nigrum*).

Viveros, J.L. 95, 168 (*nigrescens*); 215 (*americanum*).

Voegelin, E.W. 12 (*americanum*).

Vogel, J.R.T. 8 (*americanum*).

Vogelmann, H.W. 2006 (*macrotonum*).

Vohra, J.N. NC-6035A, NC-9900, NC-11244, NC-58005 (*americanum*); NC-78290 (*villosum*).

Von Platen, L. 149 (*triflorum*).

Vorontsova, M.S. 162 (*americanum*); 184, 185 (*scabrum*); 200 (*villosum*).

Voss, E.G. 1794, 7977, 12375, 16808 (*emulans*).

Votava, F. 36 (*americanum*).

Vreeland, F.K. 638 (*triflorum*).

Vueeden, D.V. 97 (*retroflexum*).

Wade, C.A. 1495 (*emulans*).

Wadhwa, B.M. 216, 786 (*villosum*); CC-5211, CC-7495 (*nigrum*); NC-52902, NC-52914 (*americanum*); NC-57375, NC-60184 (*villosum*); NC-60187, NC-62972, NC-63483, NC-66840, NC-85432 (*americanum*); NC-83715 (*nigrum*).

Wagenbreth, I. 68, 120 (*nigrescens*).

Wagenknecht, R. 18532 (*furcatum*).

Wagner, D. 1159 (*triflorum*).

Wagner, W.L. 4249 (*nigrescens*); 5708 (*americanum*).

Walker, E.H. 5442 (*nigrum*).

Walker, E.P. 333 (*triflorum*).

Walker, H.A. 178 (*americanum*); 1842 (*nigrum*).

Walker, R.L. 354 (*pseudogracile*).

Wallace, K.L. 346 (*americanum*).

Wallen, R. 46 (*emulans*).

Wallich, N. 197, 2615E (*americanum*); 2615 (*nigrum*); 2615[Y] (*americanum*).

Wallis, C.S. 7929 (*emulans*).

Wallnöfer, B. 4213 (*villosum*); 4343 (*nigrum*).

Walsh, N.G. 2255 (*americanum*); 3644 (*chenopodioides*); 7011 (*americanum*).

Walter, B.M.T. 4143 (*americanum*).

Walter, D. 9008 (*nitidibaccatum*); 9264 (*triflorum*); 9609 (*interius*); 11528 (*triflorum*).

Walter, J. 01/0245A (*villosum*); 4135, 4136 (*nigrum*); 4189 (*nitidibaccatum*); 4674 (*nigrum*); 5583, 5595 (*villosum*); 7085, 7086 (*nitidibaccatum*); 7509b, 7509a (*nigrum*); 8946 (*villosum*); 9163a (*nigrum*).

Walters, T.L. 11985 (*emulans*).

Walton, D. 186 (*villosum*).

Wang Zhong-tao 87-0016 (*nigrum*).

Wang, [?] 399 (*americanum*).

Wang, B.M. 050 (*nigrum*).

Wang, C. 33561, 35331 (*americanum*); 43427 (*nigrum*); 44166 (*villosum*).

Wang, C.M. 2550 (*nigrum*).

Wang, C.W. 62905, 66363, 67054, 69255, 73077, 74462 (*nigrum*).

Wang, C.Y. 7567 (*nigrum*).

Wapstra, M. 1115 (*triflorum*).

Ward, D.B. 6404 (*americanum*); 8787 (*pseudogracile*).

Ward, L.F. 717 (*triflorum*).

Warnock, B.H. 155, 683 (*nigrescens*); 848, 6584, 7498 (*douglasii*); 7932, 10605 (*nigrescens*); 10890 (*douglasii*); 11990 (*triflorum*); 12727 (*douglasii*); 14889 (*nigrescens*); 22370 (*douglasii*); 46548B (*nigrescens*).

Warren, A. 137 (*triflorum*).

Warren, R.C. 18 (*villosum*).

Warrick, R.B. 431 (*americanum*).

Wasum, R. 1084 (*chenopodioides*); 1941 (*americanum*); 4079, 7398 (*chenopodioides*).

Wasum, R.A. 3157, 3993 (*americanum*); 4298 (*sarrachoides*).

Waterfall, U.T. 1679 (*emulans*); 9248 (*triflorum*); 13825 (*nigrescens*); 15748 (*americanum*).

Waterhouse, B.M. 1273, 5401, 5466, 5519, 5956 (*americanum*).

Waterlot, E.G. 407 (*scabrum*).

Watermeyer, A.M. 123 (*villosum*).

Watkins, J. 633 (*douglasii*).

Watson, H.C. 146, 146b (*nigrum*).

Watson, J. 12 (*chenopodioides*).

Watson, J.M. 744 (*villosum*).

Watson, S. 939 (*interius*).

Watt, G. 7053 (*scabrum*).

Waugh, F.A. 240 (*interius*).

Wawra, H. 719, 2599 (*americanum*).

Weatherbee, P.B. 563, 2487, 3211 (*emulans*).

Weatherwax, P. 1045 (*emulans*).

Weaver, J. 146 (*emulans*).

Weaver, R.E. 1132 (*macrotonum*).

Webb, P.B. 44 (*villosum*).

Webb, R.J. 393 (*emulans*).

Webber, H.J. 256 (*nigrescens*).

Weber, H. 1889 (*macrotonum*).

Weber, R. 450 (*nigrescens*).

Weber, W.A. 5086 (*interius*); 13135 (*triflorum*).

Webster, G.L. 15941 (*americanum*); 26047 (*douglasii*).

Wedel, H. von 2840 (*nigrescens*).

Weedon, R.R. 8214 (*triflorum*).

Weight, K.E. kew907Z, 935Z (*triflorum*).

Weinberg, B. 636 (*americanum*).

Weis, G. 5 (*emulans*).

Welch, B. 5298 (*sarrachoides*).

Welch, W.H. 122, 1266, 3887 (*emulans*).

Wells, B.W. 4766 (*pseudogracile*).

Wellwood, A.A. 1993 (*emulans*).

Welman, M. 369 (*retroflexum*).

Welsh, S.L. 2669 (*triflorum*).

Welwitsch, F.M.J. 226 (*nigrum*); 6033, 6053, 6099, 6100, 6101, 6102, 6103[a], 6103[b], 6108 (*scabrum*).

Wendelbo, P. 1475, 14504 (*nigrum*).

Wendt, T. 90 (*nigrescens*); 651, 1321 (*douglasii*); 4953 (*nigrescens*).

Wenzel, C.A. 242 (*nigrum*).

Werdermann, E. 359 (*furcatum*).

Werger, M.J.A. 236 (*retroflexum*).

Werling, L. 236 (*macrotonum*).

Wessels Boer, J.G. 1740 (*macrotonum*).

Wessen, L.J.R. 3211 (*emulans*).

West, E.M. 95 (*pseudogracile*).

West, P.A. 710 (*douglasii*).

West, R.C. P-25 (*douglasii*).

Westaway, J.O. 3656, 4018 (*americanum*).

Western, R. 135, 197 (*villosum*).

Weston, A.S. 4148, 4863 (*nigrescens*); 5824 (*macrotonum*).

Weston, S.C. 67 (*triflorum*).

Westphal, E. 1483, 1674, 1749, 1924, 2548, 2616, 2740, 2908, 3496, 3963 (*villosum*); 8733, 8739, 8758, 8765, 8766, 8803, 9429, 9660, 9675, 9700, 10014 (*scabrum*).

Westphal-Stevels, J.M.C. 1989 (*scabrum*).

Westra, L.Y.T. 179 (*villosum*).

Westwater, M. 192954 (*scabrum*).

Weyland, M.C. 447, 2040 (*americanum*).

Whalen, M.D. 853, 895 (*americanum*).

Wharton, M.E. 5143, 5210 (*emulans*).

Wheeler Haines, R. 12, W-1359 (*villosum*).

Wheeler, L.A. 289801, 289902, 290104 (*emulans*).

Wheeler, L.C. 1124 (*triflorum*); 1125, 1130, 1369 (*nitidibaccatum*); 1439, 2041 (*americanum*); 3655 (*triflorum*); 3798, 3994 (*nitidibaccatum*); 3995 (*triflorum*); 5554 (*nitidibaccatum*); 8165, 10798 (*americanum*).

Whissen, P.S. 4742 (*triflorum*).

Whistler, A. 1929, 2589, 4675, 6746, 8436, 8858, 10810, 12899 (*americanum*).

Whitcombe, R. 183, 464, 815, 873 (*villosum*).

White, D. 406 (*emulans*).

White, H.S. 78 (*emulans*).

White, J.C. 100 (*americanum*).

White, K.L. 107 (*emulans*).

White, M. 242 (*interius*).

White, O.E. 1091 (*americanum*).

White, S. 731 (*americanum*).

White, S.D. 10228 (*nigrum*); 11602 (*americanum*).

White, S.S. 2634 (*douglasii*).

Whited, K. 600 (*triflorum*).

Whitefoord, C. 3678 (*americanum*).

Whitehead, B. 7 (*americanum*).

Whitham, J.C. 1328 (*triflorum*).

Whiting, A.F. 913-EB (*nigrescens*); 1047-4587 (*douglasii*).

Whiting, M.M. 35-60 (*villosum*); 100 (*americanum*).

Whitmee, S.J. 22 (*americanum*).

Whitson, M.K. 2 (*americanum*).

Whittaker, J. 87, 601 (*nigrum*).

Wiadrowski, R. 1 (*triflorum*).

Wible, M. 703 (*americanum*).

Wickens, G.E. 1191, 1711, 2391 (*villosum*).

Wickham, J. 13 (*emulans*).

Wicklund, C.P. 34 (*douglasii*).

Widjaja, E.A. 1360 (*americanum*).

Wieboldt, T.F. 9823, 11353 (*sarrachoides*).

Wiegand, K.M. 2318 (*pseudogracile*); 2821 (*americanum*); 8719 (*emulans*).

Wieland, R.G. 7527 (*americanum*).

Wieringa, J.J. 6785 (*triflorum*).

Wierzbicki, P. 393, 2375 (*nigrum*).

Wiesbaur, J. 2239 (*villosum*).

Wiggins, I.L. 126, 547 (*americanum*); 1058, 2053, 2218, 2527, 2527, 2606, 2969, 3002, 3875, 4266 (*douglasii*); 4731 (*americanum*); 4779 (*douglasii*); 5678 (*americanum*); 6411, 7020 (*douglasii*); 8164 (*nitidibaccatum*); 9946 (*douglasii*); 11197, 11198 (*nitidibaccatum*); 16062 (*douglasii*); 18265 (*americanum*).

Wight, A.E. 111 (*americanum*).

Wight, R. 2010 (*nigrum*); 2012[c] (*villosum*); 2326 (*americanum*).

Wilbur, R.L. 8733 (*macrotonum*); 12290, 12332, 33986 (*pseudogracile*); 35744 (*douglasii*); 36451, 36689 (*americanum*); 48332 (*pseudogracile*); 63114 (*americanum*); 69099 (*pseudogracile*).

Wild, H. 539, 3961 (*retroflexum*).

Wilde, J.J.F.E. de 12, 24 (*scabrum*); 1839 (*villosum*).

Wilde, W.J.J.O. de 5377 (*villosum*); 18171 (*americanum*).

Wilford, C. 68 (*americanum*).

Wilkinson, E.H. 115, 154 (*nigrescens*).

Williams, D.E. 158 (*douglasii*); 592 (*corymbosum*); 959 (*americanum*).

Williams, F.R. 2294 (*emulans*).

Williams, I. 2744 (*retroflexum*).

Williams, L. 244 (*sarrachoides*); 5831 (*nitidibaccatum*); 12825 (*americanum*).

Williams, L.D. 379, 4112 (*triflorum*).

Williams, L.H.J. 8336 (*nigrum*).

Williams, L.O. 9076, 10733, 25303 (*nigrescens*).

Williams, M. 892 (*emulans*).

Williams, M.J. 75-66-7, 77-84-10 (*triflorum*); 76-117-11, 76-118-7 (*nitidibaccatum*).

Williams, R.L. 32, 734 (*triflorum*).

Williams, R.P. 1139 (*triflorum*); 1350 (*interius*); 1351 (*triflorum*); 2399 (*emulans*).

Williams, R.S. 406 (*triflorum*); 1079 (*americanum*).

Williams, S. 7 (*emulans*).

Williams, S.L. 287 (*scabrum*); 1079 (*retroflexum*).

Williamson, B. 502, 533 (*villosum*).

Williamson, C. 48 (*retroflexum*).

Williamson, J. 53 (*villosum*); 440 (*triflorum*).

Willian, J.G. 258[a] (*americanum*).

Willkomm, H.M. 475 (*nigrum*); 637 (*villosum*).

Wilman, M. 3140, 3192 (*retroflexum*).

Wilmott, A.J. D207 (*nigrum*).

Wilms, F. 1022a, b1022, 3458 (*retroflexum*).

Wilson, B. 5399 (*nigrum*).

Wilson, C.T. 77 (*scabrum*).

Wilson, E. 864 (*nigrum*).

Wilson, E.H. 2660 (*nigrum*).

Wilson, H.R. 265 (*nigrescens*).

Wilson, J. 19 (*americanum*).

Wilson, J.G. 370, 633 (*villosum*).

Wilson, K.L. 1254 (*americanum*); 1276, 2410 (*chenopodioides*); 3842 (*americanum*).

Wilson, P. 7832, 8213 (*americanum*).

Wilson, P.G. 18 (*chenopodioides*); 3597 (*nigrum*); 6069, 6249 (*americanum*).

Wiltbank, W.E. 307 (*triflorum*).

Winchell, A. 368 (*emulans*).

Windham, M.D. 92-271 (*nitidibaccatum*); 93-233 (*douglasii*); 91-271 (*nitidibaccatum*).

Windler 3251 (*emulans*).

Windler, D.R. 979 (*americanum*); 1025 (*nigrescens*); 2731b (*americanum*).

Wingate, J. 1831 (*emulans*).

Winter, B. de 5550 (*scabrum*).

Winter, S. 1916 (*emulans*).

Wiriadianata, H. 2032 (*americanum*).

Wislizenus, F.A. 334 (*emulans*).

Wissman, H. von 3230 (*nigrum*).

Wit, [?] de A-2921 (*scabrum*).

Without Collector 1 (*triflorum*); 19741 (*nitidibaccatum*); 3 (*scabrum*); 104/04 (*nigrum*); 197142 (*nitidibaccatum*); 6 (*chenopodioides*); 11 (*furcatum*); 55, 93 (*nigrum*); 128 (*villosum*); SN45/151 (*triflorum*); 170 (*americanum*); 249 (*emulans*); 259 (*villosum*); 269 (*nitidibaccatum*); 303 (*chenopodioides*); 303/138 (*americanum*); 304 (*nigrum*); 320 (*emulans*); 330 (*americanum*); 348 (*chenopodioides*); 449 (*nigrum*); DM.483 (*furcatum*); 502 (*triflorum*); 558 (*sarrachoides*); 568 (*nigrescens*); 650 (*chenopodioides*); 688 (*nigrum*); 690 (*nigrescens*); 693 (*emulans*); 848, 848 (*corymbosum*); 912b (*nigrum*); 1010 (*villosum*); 1229 (*americanum*); 1248 (*chenopodioides*); 1326 (*emulans*); 1473 (*villosum*); 1571, 2093

(*nigrum*); 2322 (*nigrescens*); 2416 (*nigrum*); 2605 (*triflorum*); 2817 (*americanum*); 3969 (*nigrescens*); 4119 (*chenopodioides*); 4269, 11048 (*americanum*); CBL-12261, ECO-14156 (*villosum*); CNH-14266 (*nigrum*); 14983 (*americanum*); 17757 (*nigrescens*).

Witsell, T. 07-605 (*sarrachoides*).

Witte, G.F. de 2757 (*americanum*).

Wolf, C.B. 2087 (*douglasii*); 2399 (*triflorum*); 2756 (*interius*).

Wolf, C.E. 3837 (*americanum*).

Wolf, J. 88 (*triflorum*).

Wolff, S.E. 1305 (*interius*).

Wolken, P. 1156 (*triflorum*).

Wolley-Dod, A.H. 56A (*nigrum*); 56 (*villosum*); 336 (*americanum*); 856 (*retroflexum*); 1542, 1595 (*villosum*); 1606 (*nigrum*); 3180 (*scabrum*); 3318 (*retroflexum*).

Wolston, A. 856 (*americanum*).

Wood, A.D. 739 (*nigrescens*).

Wood, C.E. 9197, 9316 (*pseudogracile*).

Wood, D. 1445 (*americanum*).

Wood, J.R.I. 71-27, Y/74-73, S/72-84, Y/74-265, Y/75-742, Y/75-757, 2157, 2193, 2206, 2293 (*villosum*); 13577, 17194, 21698 (*americanum*).

Woodruff, M. H-1990 (*americanum*).

Woods, C.N. 2765 (*triflorum*).

Woodson, R.E. 464, 635 (*nigrescens*); 942 (*americanum*).

Wooton, E.O. 49 (*triflorum*); 50, 56 (*interius*); 214, 2684, 2686 (*triflorum*).

Wootton, D.M. 412 (*douglasii*).

Worth, C.R. 16487 (*furcatum*).

Worthington, R.D. 12432 (*americanum*); 13353, 14990 (*interius*); 21411 (*nigrescens*).

Wosnessensky, [?] 58bis (*douglasii*).

Woytkowski, F. 6758 (*corymbosum*); 8152 (*americanum*).

Wright, A.E. 629 (*americanum*); 853 (*nigrum*); 1204, 1405 (*americanum*); 1896, 2484, 2532, 2920 (*nigrum*); 3438 (*americanum*); 4848 (*nigrum*); 4953 (*americanum*); 5237 (*nigrum*); 5311 (*americanum*); 5465 (*nigrum*); 5828, 6233, 6343, 6518 (*americanum*); 7054 (*nigrum*); 7285, 7755, 8842 (*americanum*); 8876 (*nigrum*); 11422 (*americanum*); 12532 (*chenopodioides*); 12984, 12991 (*nigrum*).

Wright, C. 47 (*villosum*); 196, 347, 383 (*americanum*); 1077 (*nigrum*).

Wright, T. 92-172, 92-172 (*douglasii*).

Wright, W.F. 129 (*emulans*).

Wright, W.G. 1256 (*americanum*).

Wrigley, T.C. 188, 426, 493 (*scabrum*).

Wuang, S.T. 399 (*americanum*).

Wullschlägel, H.R. 374 (*americanum*).

Wunderlin, R.P. 5386, 8418 (*americanum*).

Wyld, J.W.G. 347, 842 (*scabrum*).

Wylie, J. 3420 (*chenopodioides*).

Wynd, F.L. 567 (*douglasii*).

Xavier, F.C. 78 (*americanum*).

Xavier, L.P. JPB887, JPB2117, JPB3611 (*americanum*).

Xiao Bai-Zhong 4195, 4230 (*nigrum*).

Xu Ren-xin 40 (*nigrum*).

Yacotú, J.L. 11 (*nigrescens*).

Yao, K. 8384, 8509, 8611 (*nigrum*).

Yates, C.C. 56 (*scabrum*).

Yates, H.S. 2622 (*nigrum*); 5007 (*douglasii*).

Yatskievych, G.A. 03-87 (*emulans*); 96-88 (*sarrachoides*).

Yen, L. 2868 (*douglasii*).

Yepes A, S. 789 (*macrotonum*); 1118 (*americanum*).

Yonekura, K. 3620, 97453 (*americanum*).

Yong, G. 49 (*nigrescens*); 303 (*douglasii*).

Yorimitsu, T. FOS-5704 (*nigrum*).

York, D. 158 (*americanum*).

Young, A.L. 152 (*americanum*).

Young, T.P. 1032 (*villosum*).

Youthed, G. 790 (*chenopodioides*).

Yshmull, [?] 60 (*americanum*).

Yu, T.T. 564, 8530, 13333, 17345 (*nigrum*).

Yugovic, J.Z. 218 (*furcatum*).

Yuncker, T.G. 1453, 1800 (*emulans*); 3918, 3998 (*douglasii*); 5670, 9632, 15272, 18015 (*americanum*).

Záchia, R.A. 176, 3097 (*americanum*).

Zak, V. 1.419, 661, 859, 1009, 1140, 1375, 1377, 1378, 1379, 1394, 1400, 1417, 1466, 1550, 1553, 1556, 1559, 1562, 1579, 1581, 1588, 1619, 1638, 1736, 1740, 1765, 1913, 1914 (*macrotonum*); 1980 (*americanum*); 2077, 2097, 2474, 2483, 2490, 2499, 2501, 2742, 3038 (*macrotonum*).

Zambrano C, O. 992 (*douglasii*).

Zambrano, G. 66 (*nigrescens*).

Zamora C, P. 1586, 2801, 4987 (*nigrescens*).

Zamudio R, S. 9775 (*douglasii*); 11120 (*pruinosum*).

Zanoni, T.A. 11466, 30178 (*americanum*); 47320 (*nigrum*).

Zarate M, A. 589 (*nigrescens*).

Zarate Marcos, A. 318, 546, 630 (*nigrescens*).

Zarate-Marcos, A. 183 (*douglasii*).

Zardini, E. 5701 (*americanum*).

Zardini, E.M. 2712, 3385, 3426, 4974, 5335, 5753, 5756, 5792, 6642, 6650, 6983, 11390, 13465, 13605, 14630, 15600, 16042, 20517, 21222, 22107, 22282, 22465, 22745, 28234, 28350, 32112, 32116, 32856 (*americanum*); 40428 (*sarrachoides*); 54796, 55288 (*americanum*).

Zárgani 14236-E (*villosum*).

Zarucchi, J.L. 2623, 4006 (*americanum*); 5807 (*nigrescens*); 6886, 6949 (*macrotonum*).

Zavala-Gallo, L. 180 (*triflorum*).

Zebryk, T.M. 5509 (*emulans*).

Zehutner 169 (*americanum*).

Zeigler, S.R. 1657 (*emulans*).

Zelaya, L. 77 (*nigrescens*).

Zeller, S.M. 1143 (*nitidibaccatum*).

Zerón B, F. ME-34 (*douglasii*).

Zeyher, C.L.P. 3473 (*retroflexum*).

Zhu, G.H. 1403 (*emulans*); 2228, 2897 (*nigrum*).

Ziegler, S.R. 1657, 1982, 2038, 2147, 2430, 2609, 2941 (*emulans*).

Zika, P.F. 1668 (*emulans*).

Zingg, R.M. A-46 (*douglasii*).

Zohrab, J. 57 (*nigrum*).

Zola B, M.G. 280, 410, 428, 1018, 1113, 2399 (*nigrescens*).

Zoller, C. 1991.184 (*americanum*).

Zollinger, H. 678 (*nigrum*); 1279 (*americanum*); 2177bis (*nigrum*).

Zöllner, O. 8950, 14543 (*furcatum*).

Zomlefer, W.B. 3896 (*emulans*).

Zuccarini, J.G. 186 (*villosum*).

Zuloaga, F.O. 4258, 4282 (*macrotonum*); 5577, 6653 (*americanum*).

Zunguze, D. 113, 597 (*americanum*).

Zúñiga B, G. 113 (*nigrescens*).

Zwickey, A.L. 148 (*nigrum*).
